# RNA-Seq Analysis Reveals Candidate Genes for Ontogenic Resistance in *Malus-Venturia* Pathosystem

**DOI:** 10.1371/journal.pone.0078457

**Published:** 2013-11-04

**Authors:** Michele Gusberti, Cesare Gessler, Giovanni A. L. Broggini

**Affiliations:** Institute of Integrative Biology Zürich, Plant Pathology Group, Swiss Federal Institute of Technology, Zürich, Switzerland; China Agricultural University, China

## Abstract

Ontogenic scab resistance in apple leaves and fruits is a horizontal resistance against the plant pathogen *Venturia inaequalis* and is expressed as a decrease in disease symptoms and incidence with the ageing of the leaves. Several studies at the biochemical level tried to unveil the nature of this resistance; however, no conclusive results were reported. We decided therefore to investigate the genetic origin of this phenomenon by performing a full quantitative transcriptome sequencing and comparison of young (susceptible) and old (ontogenic resistant) leaves, infected or not with the pathogen. Two time points at 72 and 96 hours post-inoculation were chosen for RNA sampling and sequencing. Comparison between the different conditions (young and old leaves, inoculated or not) should allow the identification of differentially expressed genes which may represent different induced plant defence reactions leading to ontogenic resistance or may be the cause of a constitutive (uninoculated with the pathogen) shift toward resistance in old leaves. Differentially expressed genes were then characterised for their function by homology to *A. thaliana* and other plant genes, particularly looking for genes involved in pathways already suspected of appertaining to ontogenic resistance in apple or other hosts, or to plant defence mechanisms in general.

In this work, five candidate genes putatively involved in the ontogenic resistance of apple were identified: a gene encoding an “enhanced disease susceptibility 1 protein” was found to be down-regulated in both uninoculated and inoculated old leaves at 96 hpi, while the other four genes encoding proteins (metallothionein3-like protein, lipoxygenase, lipid transfer protein, and a peroxidase 3) were found to be constitutively up-regulated in inoculated and uninoculated old leaves. The modulation of the five candidate genes has been validated using the real-time quantitative PCR. Thus, ontogenic resistance may be the result of the corresponding up- and down-regulation of these genes.

## Introduction

Apple (*Malus x domestica* Borkh.) is one of the most cultivated fruit crops in temperate climates. The major constraint of apple cultivation is the apple scab, a fungal disease caused by *Venturia inaequalis*, which can lead to important crop losses if not properly controlled [1].

In apple, at least 17 major resistance genes (*Rvi1* to *Rvi17*) against *V. inaequalis* have been found [2]. However, only *Rvi6* (previously *Vf* from *Malus floribunda* 821) has been extensively used for resistance breeding to date [3]. Since the breakdown of the *Riv6* gene in the early nineties [4] new breeding programmes have started to investigate other resistance genes for future resistance breeding [5]–[9]. An increase in resistance with increasing apple leaf age (ontogenic resistance) has been observed in all apple genotypes and is known to act against all known *Venturia inaequalis* strains. To date, no report of the breakdown of this type of resistance has been found in the literature; thus, ontogenic resistance is considered durable [10].

Goethe [11] and Aderhold [12] are believed to have been the first researchers noticing age-related resistance in the *Malus*-*Venturia* pathosystem. The authors observed a decrease of leaf susceptibility with increasing tissue age. Nearly three decades later, Keitt and Jones [13] showed an increase in incubation period and a decrease of disease severity by increasing leaf age. Following these observations, many researches have been carried out on *Malus*-*Venturia* interaction during leaf infection. Gessler and Stumm [14], Li and Xu [15], and Gusberti et al. [16] showed that the fungus grew faster in young leaves compared to old ones. The first unfurled and expanding leaf is considered susceptible to the apple scab disease, while the fifth leaf (starting from the top of the shoot) is considered fully resistant [14]–[17].

Disease resistance mechanisms during tissue ontogenesis have been studied in different plant pathogen systems and some factors have been suggested to be correlated to the observed age-related resistance. Among them, the most important appears to be chemical compounds such as salicylic acid [18], [19] and pathogenesis-related proteins [19], [20], physiological barriers like the cuticle [21]–[23], lenticels [24], restricted phloem movement [25] or a limiting nutritional substrate for fungal infection [26]. However, since a different mechanism for age-related resistance is described in each crop plant, much work remains to unveil the mechanism underlying this type of resistance in other plants.

In apple, several aspects have been investigated in order to unveil the nature of ontogenic resistance. Physiological barriers like the cuticle and papillae [27], were not correlated to the age-related resistance. Research focusing on chemical barriers like melanoproteins, phenols, flavonols, polygalacturonases-inhibition proteins and the activity of different enzymes (e.g. phenylalanine ammonia lyase, polyphenoloxidase, β-glucosidase, chitinase, and fungal polygalacturonases) has been performed [17], [29]. However, despite nearly half a century of research, no clear patterns for ontogenic resistance of apple were found.

Thus, since physical and biochemical barriers have been exhaustively studied in this pathosystem without any clear pattern for the observed ontogenic resistance, other aspects to be considered are genes that are differentially expressed between young and old leaves. Furthermore, analyses to unveil the constitutive or pathogen-induced nature of the ontogenic resistance are needed. Today, the rapidly evolving sequencing techniques based on total RNA sequencing (RNA-seq), have decreased costs of analysis and increased the precision of results, allowing the researcher to maximise data outputs minimising their laboratory work and manipulation bias [30], [31]. RNA-seq uses next generation sequencing (NGS) technology (Illumina's Genome Analyser, SOLID from Applied Biosystems, or the 454 Genome Sequencer) to sequence and quantify transcripts [31]. With the technical progress of this rapidly evolving technology, some studies have focused the research at the transcriptome level to find fungal effectors [32],[33] and mechanisms involved in plant defences against microorganisms like chemical defences [34] and structural defences [35]. Thus, today, NGS appears to be the most promising methodology to study plant pathogen interactions in non-model species [31] like *Malus x domestica*. Moreover, the genome sequence of *Malus x domestica* ‘Golden Delicious’ has been recently published [36].

The aim of this work was to identify and characterise genes that are significantly differentially expressed during the shift from the susceptibility of young leaves to the resistance of old fully expanded leaves (ontogenic resistance) of the apple plant. Moreover, the constitutive or induced mechanism behind ontogenic resistance were studied by comparing inoculated and uninoculated leaves in the early phase of fungal colonisation at 72 and 96 hours post-inoculation.

The data presented in this work will add more knowledge to the *Venturia-Malus* pathosystem and provide new insight for future researches on ontogenic resistance in apple.

## Materials and Methods

### Plant material

Plant material comprised young *Malus x domestica* (Borkh.) ‘Golden Delicious’ saplings grafted on M9T337 rootstock. Plants were kept in active growth with a 16h/8h (day/night) photoperiod and a minimum of 65.5 µmol m^-2^ s^-1^ light intensity provided with white fluorescent lamps (Philips Master TL-D 36W/830); they were fertilised once with Obstdünger 12∶8∶16∶2∶0.2 (N∶P∶K∶Mg∶B; OBA-Lanze, Hauert AG, Switzerland) one week prior to the start of the experiment. Plants were grown under greenhouse conditions at 20±2°C and 70% RH until 10 to 15 leaves stadium. Rape-seed oil (Maag AG, Switzerland) mixed with Alaxon 50 (Maag AG, Switzerland) and two weeks later Vertimec (Maag AG, Switzerland) were used as chemical treatments before starting the experiment to keep the plants free of pest-insects. Before inoculation, leaves were numbered and marked from the top of the shoot toward the base, with leaf 1 being the youngest unfurled and expanding leaf. Previous work has shown that leaf 1 is completely susceptible [14]–[17] and that leaf 5 (starting from the top of the shoot) is already expressing ontogenic resistance [16]. In this experiment, we selected leaf 7 (*i.e*. 4 to 5 days older than leaf 5 [28], [37]) in order to ensure clear differences between the leaf age classes.

### Inoculum, inoculation procedure and sampling

For inoculation, conidia of *Venturia inaequalis* (Cooke) single spore isolate no. 1639 [7], [38] were first multiplied on potato dextrose agar medium (Difco, USA) over 90-mm filter-papers (Whatmann International Ltd., USA); conidia were then suspended in sterile distilled water and stored in the refrigerator at -20°C until use. The second step of conidia multiplication was performed *in planta* on susceptible cultivar ‘Golden Delicious’ until enough conidia were produced. Sporulating leaves were then dried at room temperature in paper boxes and stored in plastic bags in the refrigerator at -20°C until inoculation. Inoculation, with a spore suspension of 5×10^5^ spores ml^-1^, was performed at 17±2°C and >98% RH, as previously described [16]. Plants were incubated under these conditions for 48 h following inoculation allowing the fungus to penetrate the cuticle and establish the primary sub-cuticular stroma. Half of the plants were challenged with the apple scab pathogen and half were mock inoculated. After the treatment (scab or mock inoculation), plants were grown at 17±2°C and 70% RH until sampling. The first sets of samples were collected 72 hours post-inoculation (hpi). This enabled the plants to acclimatise at the lower relative humidity (70%) for one day after the incubation period. The second sets of samples have been collected at 96 hpi, which is the moment when the pathogen is first recognised by the plant [39]. Samples have been collected in biological triplicate from leaf 1 (L1) and leaf 7 (L7) by removing the leaf tip (<100 mg) of each leaf from inoculated and uninoculated shoots, with 12 samples for each time point (72 and 96 hpi), making 24 in total. Moreover, to enable the validation of RNA-seq data using the real-time quantitative reverse-transcription PCR (qRT-PCR), independent samples of mock inoculated *M. x domestica* ‘Golden Delicious’ leaf 1 and leaf 7 were collected at 72 and 96 hpi.

### Total RNA isolation

Fresh leaf tissue (<100 mg) was sampled from inoculated and uninoculated leaf samples at 72 and 96 hpi and collected in 2-ml Eppendorf tubes (Eppendorf, Germany), previously prepared with 5 to 10 2-mm sterile glass beads, immersed in liquid nitrogen immediately after sampling and stored at −80°C until processing. Tissues were ground twice with the FP 120 Fast-Prep machine (Bio 101 Savant Instruments Inc., Qbiogene, France) for 30 s at a speed of 5.5 m s^-1^ with an intermediate immersion in liquid nitrogen between the two grinding steps. RNA was extracted with the SV Total RNA Isolation System (Promega Corporation, USA) and column purified following the manufacturer's instructions. After the addition of RNA Lysis Buffer, samples were homogenised with the FP 120 Fast-Prep machine again for 30 s at a speed of 5.5 m s^-1^. RNA integrity and quality was tested with the Agilent 2100 Bioanalyzer RNA 6000 NANO assay (Agilent Technologies, Palo Alto, CA, USA). After RNA isolation and quality assessment, samples were stored at -80°C until cDNA library construction and transcriptomic assay.

### Libraries preparation for Illumina HiSeq 2000

Complementary DNA (cDNA) libraries were constructed, starting from 1 µg of total RNA, at the Functional Genomic Center Zurich (FGCZ) following the TruSeq RNA Sample preparation protocol v.2 instructions (Low Throughput protocol, Illumina, Inc.). The quality of the isolated RNA was further determined with a Qubit® (1.0) Fluorometer (Life Technologies, CA, USA) and a Bioanalyzer 2100 (Agilent Technologies). Only those samples with a 260 nm/280 nm ratio between 1.8–2.1 and a 28S/18S ratio within 1.5–2 were further processed. The TruSeq RNA Sample Prep Kit v2 (Illumina, Inc., CA, USA) was used in the succeeding steps. Briefly, total RNA samples (1 µg) were polyA-enriched and then reverse-transcribed into double-stranded cDNA. TruSeq adapters were ligated to double-stranded cDNA. Fragments containing TruSeq adapters on both ends were selectively enriched with PCR. The quality and quantity of the enriched libraries were validated using Qubit® (1.0) Fluorometer and the Caliper GX LabChip® GX (Caliper Life Sciences, Inc., USA). The products resulted in a smear with an average fragment size of approximately 260 bp. The libraries were normalised to 10 nM in Tris-HCl 10 mM, pH 8.5 with 0.1% Tween 20.

### Sequencing and processing of RNA-Seq data

Bar-coded libraries were spread over 4 Illumina HiSeq 2000 lanes, avoiding biological replicates in the same lane to assure the same instrument variation for the entire experiment. The TruSeq PE Cluster Kit v3-cBot-HS (Illumina, Inc., California, USA) was used for cluster generation using 2 pM of pooled normalized libraries on the cBOT. Sequencing was performed on the Illumina HiSeq 2000 paired-end at 2×101 bp using the TruSeq SBS Kit v3-HS (Illumina, Inc.). RNA-seq reads were quality-checked with fastqc which computes various quality metrics for the raw reads. Reads were aligned to the genome and transcriptome with Tophat v 1.3.3. Before mapping, the low quality ends of the reads were clipped (3 bases from the read start and 10 bases from the read-end). Tophat was run with default options. The fragment length parameter was set to 100 bases with a standard deviation of 100 bases. Based on these alignments the distribution of the reads across genomic features was assessed. Isoform expression was quantified with the RSEM algorithm (http://www.biomedcentral.com/1471-2105/12/323) with the option for estimation of the read-start position distribution turned on. All raw data were deposited in the European Nucleotide Archive (ENA: http://www.ebi.ac.uk/ena/data/view/ERP003589) and experimental meta-data are available in the ArrayExpress database (www.ebi.ac.uk/arrayexpress) under accession number E-MTAB-1726.

The analysis of Tophat files was performed on the CLC Genomics Workbench v. 5.5.1 (CLC bio, Aarhus N, Denmark), following the manufacturer's instructions. Sequences were then analysed with the RNA-seq analysis program of the CLC platform and mapped against unannotated *M. x domestica* 63541 genes set reference v.1.0 (http://genomics.research.iasma.it/). The insert size for paired-end reads was set between 150 and 250 bp and normalisation of expression values was performed using RPKM values [40]. All other parameters were kept at default levels. The CLC Genomic Workbench was further used to perform a principal component analysis with all differentially expressed genes (DEGs) found in each cDNA library.

Identification of DEGs was based on normalised gene expression calculated as RPKM, analysed using the Baggerley's test [41] and filtered with the False Discovery Rate (FDR) *P*-value correction of 0.0001 (one false discovery in 10000 discoveries). The resulting DEGs were then loaded on Blast2Go v. 2.5.1 (http://www.blast2go.com/b2glaunch; [42]) for Blastx and gene ontology analysis, separated using the Gene Ontology (GO) vocabulary (http://www.geneontology.org/). Ontology annotations were then refined using InterPro Scan, ANNEX, GoSlim and KEGG (Kyoto Encyclopaedia of Genes and Genomes; http://www.genome.jp/kegg) functions of the Blast2Go platform. The 20 most abundant transcripts for each cDNA library were filtered using the RPKM normalisation procedure on the CLC Genomic Workbench. Sequences were then loaded on Blast2Go v.2.5.1 for gene ontology functional annotation using level 2 GO vocabulary for biological process terms, molecular function terms, and cellular component terms.

Moreover, a fasta file with all DEGs was generated and sent through the Mercator webtool (http://mapman.gabipd.org/web/guest/app/mercator) for Bincode mapping, and through webtool MapMan v.3.5.1 (http://mapman.gabipd.org/web/guest/mapman, [43]) for pathway analysis. Default parameters were used and JGI Chlamy Augustus models, TIGR5 rice proteins, InterProScan, and a Blast cut-off of 50 were selected.

Pathway analysis was performed using the KEGG function of the Blast2Go webtool. KEGG pathway maps were then enriched by inserting the most significant DEGs found with the MapMan webtool.

The analysis focused on signalling and hormone pathways, genes encoding chemical defences like pathogenesis-related proteins, genes encoding physical barriers like cuticle, waxes and callose, genes acting on the biosynthesis or transport of substances connected to fungal nutrition and genes regulating the acidity of leaf tissues like proton transporters and cation/anion co-transporters.

### Real-Time quantitative PCR validation of RNA-seq data

Specific primers for the five candidate genes were designed (File S1) using the Primer-BLAST webtool (http://www.ncbi.nlm.nih.gov/tools/primer-blast) of the National Center for Biotechnology Information (NCBI: http://www.ncbi.nlm.nih.gov) and verified against *Malus x domestica* genome v. 1.0 using the BLAST function of the Genome Database for Rosaceae (GDR: www.rosaceae.org). Independent RNA samples (*M. x domestica* ‘Golden Delicious’ uninoculated L1 and L7 leaves collected at 72 and 96 hpi) were reverse-transcribed in triplicate using the RevertAid First Strand cDNA Synthesis Kit (Thermo Scientific, Fermentas, Hilden, Germany), following the manufacturer's instructions.

The concentration of cDNA samples was quantified using a NanoDrop ND-8000 spectrophotometer (Thermo Scientific, Wilmington, USA).

Preliminary specificity tests were performed using the end-point PCR performed with each of the five primer pairs (MT3, LOX, LTP, PX and EDS, File S1) in a 20 µl reaction. The Mastermix comprised 1× PCR Buffer (Fermentas, Hilden, Germany), 0.1 mM dNTPs, 0.05 µM primer pairs, 0.07 U µl^-1^ DreamTaq DNA Polymerase (Fermentas) and 5 µl (20.1±3.9 ng µl^-1^) cDNA. PCR thermo-cycler conditions were: 3 min at 94°C, followed by 35 cycles of 94°C for 30 s, 60°C for 30 s, and 72°C for 30 s. The PCR products were then loaded on a 1% (w/v) agarose gel in 0.5× Tris-Borate-EDTA (TBE) buffer at 125 Volts for 1.5 h.

Real-time quantitative PCR analyses were then performed on the ABI 7500 Fast Real-Time Sequences Detection System (Applied Biosystems, Foster City, CA, USA). Amplification conditions were 15 min at 95°C, followed by 45 cycles of 30 s at 95°C and 1 min at 60°C. Reaction mix (10 µL reaction) comprised 1× Hot FirePol EvaGreen qRT-PCR Mix Plus (ROX) buffer (Solis BioDyne, Tartu, Estonia), 10 µM forward and reverse primer pairs and 3 µl (201.3±39.3 ng µl^-1^) cDNA. Melting curve analysis was performed to confirm the specificity of the amplification product. Threshold line was set manually at 0.2 in every analysis, performed using the Sequence Detection Software v. 2.0.6 (Applied Biosystems). Each 96-well plate was loaded with No Template Controls, No RT control and positive controls in triplicate. Ubiquitin conjugating enzyme (UBC: MDP0000223660) was chosen as a housekeeping internal standard gene during qRT-PCR, as published in a previous work [44], and the relative expression of the five candidate genes has been calculated using the 2^−ΔΔCT^ method as previously described [45]. Before comparing the fold- change between qRT-PCR and RNA-seq data, all L1 values were normalised to L1_average_ = 1. After the normalisation procedure, the differential gene expression between qRT-PCR and RNA-seq data was assessed using the one-tailed T-test (*p*<0.05) using JMP v. 10.0.2 (SAS Institute Inc., Cary, US) on Windows 7.

## Results and Discussion

**Figure 1:**
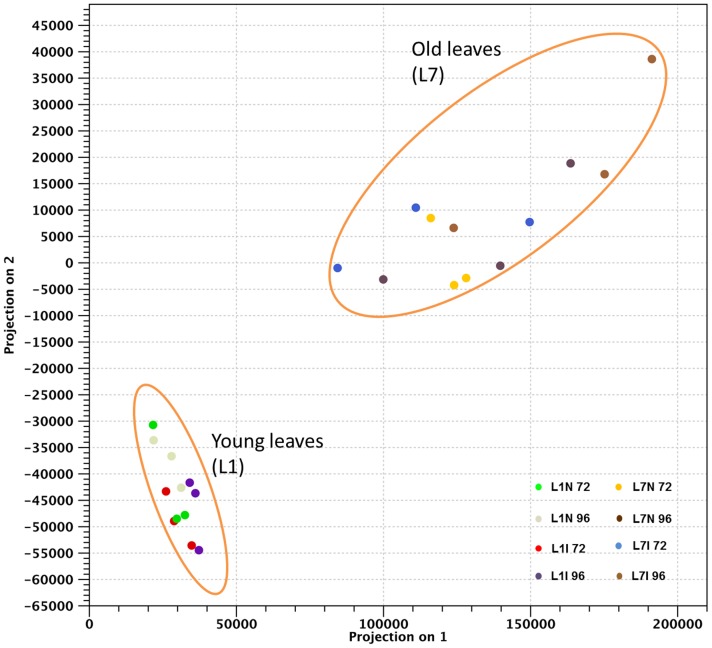
Principal component analysis performed with the CLC Genomics Workbench v. 5.5.1. Genes differentially expressed for leaves of different age (L1 and L7), for inoculated and uninoculated leaves (I and N), and for the two time point analysed (72 and 96 hpi).

Approximately 30–55 million paired-end reads for each cDNA library were obtained (Table 1). The principal component analysis (PCA) of DEGs showed two main clusters between old leaves (L7) and young ones (L1) for both inoculated and uninoculated leaves (Figure 1).

**Table 1:** Reads and genes mapped in each generated cDNA library.

Hours post inoculation	Condition a	Biological replicate	Reads mapped in pairs b(Mio.)	Reads mapped in broken pairs (Mio.)	Reads not mapped (Mio.)	Total reads (Mio.)	Mean reads per library (Mio.)	SD	Genes mapped by unique reads (%) c
72	L1 I	1	4.9	11.8	19.5	36.2			20917 (33)
		2	4.5	11.6	29	45.1	39.4	4.95	21626 (34)
		3	5.5	10.5	20.9	36.9			22501 (35)
	L1 N	1	3.7	10.1	21.9	35.7			21401 (34)
		2	2.3	6.7	21.7	30.7	34.0	2.89	19534 (31)
		3	6.5	11.2	18	35.7			24080 (38)
	L7 I	1	4.5	12.2	33.6	50.3			20359 (32)
		2	2.7	7.9	26.8	37.4	42.1	7.10	17211 (27)
		3	6.2	12	20.5	38.7			21652 (34)
	L7 N	1	4.6	13.1	26.3	44			20023 (32)
		2	5	11.7	25.3	42	46.3	5.86	20816 (33)
		3	7.9	17.5	27.6	53			22210 (35)
96	L1 I	1	10.7	9	17.2	36.9			25046 (39)
		2	10.8	9.6	22	42.4	41.8	4.63	24708 (39)
		3	9.9	10.5	25.7	46.1			24849 (39)
	L1 N	1	9	7.8	29.1	45.9			24714 (39)
		2	20.6	15.7	19.2	55.5	52.0	5.30	27606 (43)
		3	18.1	14.7	21.8	54.6			27172 (43)
	L7 I	1	13.8	12.4	17.4	43.6			22604 (36)
		2	16.8	14.3	23.8	54.9	45.1	9.10	24473 (39)
		3	7.1	7	22.8	36.9			22088 (35)
	L7 N	1	9.9	9.9	22.3	42.1			22196 (35)
		2	12.3	11.3	22	45.6	44.7	2.32	22765 (36)
		3	14.1	14.1	18.3	46.5			23635 (37)

a Conditions: L1 and L7 correspond to Leaf 1 and Leaf 7 (enumeration starting from the top of the shoot toward the base) for *V. inaequalis* inoculated (I) and uninoculated (N) leaves at 72 and 96 hours post inoculation;

b Reads mapped, not mapped and total reads obtained with the CLC Genomics Workbench v. 5.5.1;

c Genes mapped by at least one unique read and proportion of genes mapped by at least one unique read out 63541 apple genes. Values obtained with the CLC Genomics Workbench v. 5.5.1.

Differential expression analysis of *M. x domestica* with an FDR *P*-value correction of 0.0001 resulted in 6 and 16 DEGs between uninoculated (L1N) and inoculated (L1I) young leaves at 72 and 96 hpi, respectively, while between uninoculated (L7N) and inoculated old leaves (L7I), 56 and 6 DEGs were found at 72 and 96 hpi, respectively. The analysis performed on leaves of different ages (L1 vs. L7) resulted in 3119 and 1784 DEGs at 72 hpi, for uninoculated and inoculated leaves, respectively. At 72 hpi, 1027 DEGs were present in both uninoculated (33%) and inoculated (57%) old leaves. At 96 hpi, DEGs between leaves of different ages for uninoculated and inoculated plants were 3750 and 2490, respectively. At this time point, 1877 DEGs were found in both uninoculated (50%) and inoculated (75%) old leaves. Young leaves analysed at 72 and 96 hpi showed 52 DEGs, while old leaves showed 99 DEGs. Nine common DEGs were found at both time points, corresponding to 17% and 9% of the DEGs found for young and old leaves, respectively. In total, we obtained 5823 DEGs among the ten conditions tested (File S2).

Bin annotation and mapping of the 5823 DEGs resulted in 23.93% unannotated genes with a blast cut-off value of 50 (Files S3, S4).

Results of 72 and 96 hpi were discordant for inoculated old leaves. In the last sampling point (96 hpi), relatively fewer DEGs were found compared to the same situation at 72 hpi. Some of the DEGs were found for inoculated and uninoculated old leaves at 72 hpi but not at 96 hpi with an FDP *P*-value correction of 0.0001. These differences were connected to the highly stringent FDP *P*-value correction used during the analyses, which may have hidden the effect of some DEGs at 96 hpi.

### Differentially expressed genes putatively involved in ontogenic resistance

In this part of the work, we examined DEGs during leaf ontogenesis using an RNA-seq approach to find a possible explanation to the observed ontogenic resistance in apple against *Venturia inaequalis*. The analysis was performed with three biological replicates for young and old leaves, either challenged or not with the pathogen at two time points (72 and 96 hpi).

The biological variability in preceding transcriptomic experiments has been found to be low [46], [47]. However, our experiment displays a biological variability of single gene' RPKM values between 1% and 98% of the average (File S5), showing the importance of taking at least three biological replicates in this type of experiments.

Moreover, the accumulation of metabolites is possible when the corresponding biosynthetic pathway genes are highly expressed or not modulated in young tissues compared to old ones (e.g. callose, lignin, wax, flavonoids, phenols, salicylic acid, and tocopherol). This is highly dependent on the rapport between the rapidity of production and degradation of the compounds; with this type of experiment, the production, accumulation or degradation steps could not be investigated. Thus, quantification of compounds in young and old leaves, inoculated and uninoculated, by means of proteomic or metabolomics approaches, may be more informative than RNA-seq experiments.

**Figure 2:**
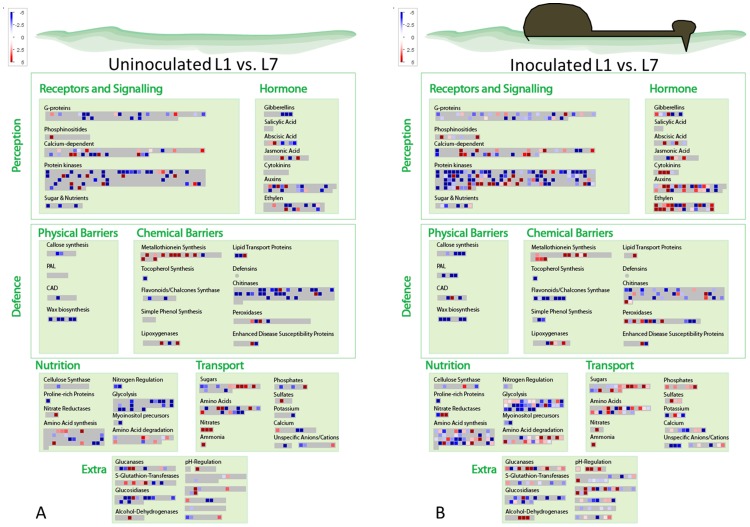
MapMan ontogenic resistance overview map at 72-inoculation (hpi). Differentially expressed genes found between young and old leaves (L1 vs. L7) for both uninoculated (A) and inoculated (B) leaves. Red: up-regulation and Blue: down-regulation of genes.

**Figure 3:**
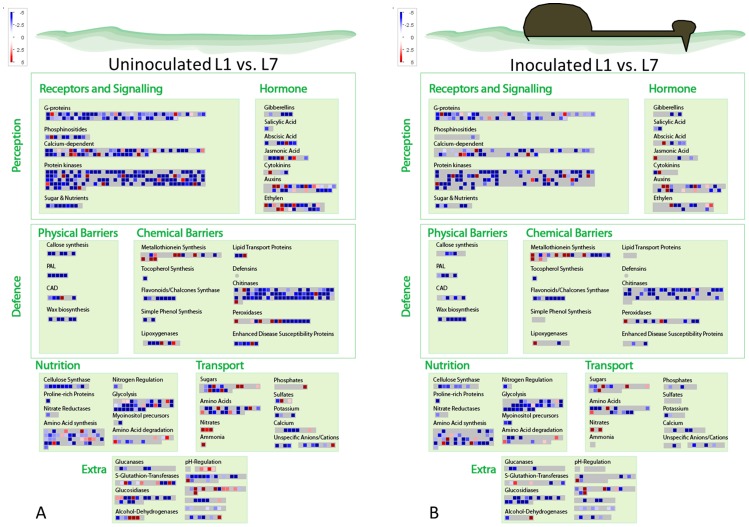
MapMan ontogenic resistance overview map at 96-inoculation (hpi). Differentially expressed genes found between young and old leaves (L1 vs. L7) for both uninoculated (A) and inoculated (B) leaves. Red: up-regulation and Blue: down-regulation of genes.

Differential gene expression analysis was performed between young (L1) and old (L7) leaves for inoculated (I) and uninoculated (N) shoots at both time points (72 and 96 hpi) on the 5823 DEGs found with a FDR *P*-value correction of 0.0001 (File S2). Results of DEGs in the different conditions tested have been summarized in the figures 2 and 3.

KEGG analysis resulted in 127 pathways (File S6). The most abundant sequences were found in the starch and sucrose metabolism (147 sequences; 6%), followed by purine metabolism (101 sequences; 4%), glycolysis and glucogenesis (75 sequences; 3%), carbon fixation in photosynthetic organisms (66 sequences; 3%) and pentose and glucuronate interconversions (65 sequences; 3%).

#### Signalling

**Figure 4:**
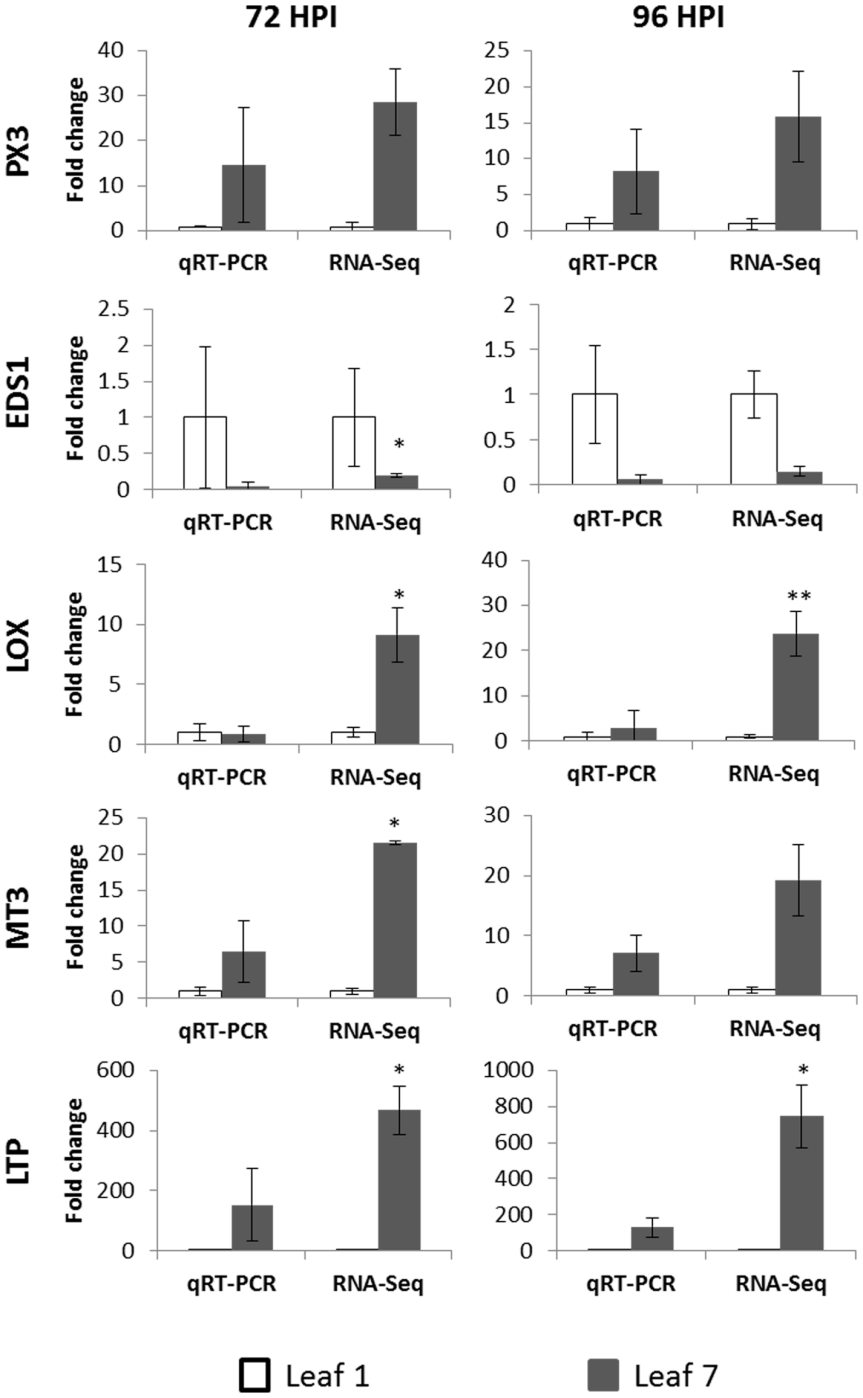
Quantitative reverse-transcription real-time PCR (qRT-PCR) validation of the candidate genes. Genes differentially expressed between young (L1: white bars) and old (L7: gray bars) apple leaves. PX3: Peroxidase 3; EDS1: Enhanced Disease Susceptibility 1; LOX: Lipoxygenase; MT3: Metallothionein 3-like; LTP: Lipid Transfer Protein. Significant differences between qRT-PCR and RNA-seq data are indicated with * (*p*<0.05) and ** (*p*<0.01).

Hormones and signalling mechanisms were investigated in *Arabidopsis thaliana* [18]; the authors showed that the accumulation of salicylic acid (SA) was the only factor which correlated with the observed age-related resistance. In our experiment, we found that genes involved in the biosynthesis of hormone precursors were, in general, up-regulated in old leaves if uninoculated and down-regulated upon inoculation (e.g. files S7, S8). For both the inoculated and uninoculated old leaves, a down-regulation of transmembrane amino acid transporters encoding genes was observed. This may be correlated to the cessation of cell enlargement and cell division hormone response (auxin, cytokinine, brassinosteroid) in the old leaves. Moreover, we could identify an enhanced disease susceptibility 1 (EDS1: MDP0000253215) protein, first observed in a mutant of *A. thaliana* mutant that was susceptible to *P. parasitica* [48]. EDS-silencing increased disease resistance in *Arabidopsi*s [48], [49]. The EDS gene has been found to be necessary for the functionality and signal transduction of other resistance genes in *Arabidopsis* plants [48]. In our experiment, the EDS1 gene encoding protein was down-regulated in both inoculated and uninoculated old leaves only at 96 hpi (Table 2), which was confirmed by qRT-PCR analysis. However, the high variability between the three biological replicates does not enable us to detect significant differences between young and old uninoculated leaves at 72 hpi (Figure 4). The EDS1 gene encoding protein has been classified under biotic stress signalling molecule during the MapMan analysis; however, the mechanism behind EDS in apple plants remains to be unveiled.

**Table 2:** Summary of the candidate genes for the ontogenic resistance in Apple found at 72 and 96(hpi).

					Fold-change at 72 hpi b		Fold-change at 96 hpi	
Sequence Name	Sequence Description	Length	E-Value	Similarity a	L1N vs. L7N	L1I vs. L7I	L1N vs. L7N	L1I vs. L7I
MDP0000149327	peroxidase 3	978	9.4E-167	0.78	28.5	23.36	15.87	NDE
MDP0000253215	enhanced disease susceptibility 1	1947	0	0.64	NDE c	NDE	−6.82	−2.72
MDP0000312397	lipoxygenase	3363	0	0.86	9.07	23.67	23.81	NDE
MDP0000466190	metallothionein-like protein	201	8.4E-33	0.79	21.53	16.20	19.22	13.55
MDP0000940078	lipid transfer protein	342	5.5E-34	0.66	466.32	206.56	743.78	NDE

a Mean protein similarity;

b Fold-change of the genes differentially expressed between Leaf 1 (L1) and Leaf 7 (L7) for inoculated (I) and uninoculated (N) leaves at 72 and 96 hours post inoculation. Gene expressions of L1 have been normalised to L1_average_ = 1 before calculating the fold-change for L7;

c NDE: Not Differentially Expressed genes with the FDR *P*-value correction of 0.0001 procedure.

#### Structural defences

Genes involved in the synthesis of cell wall precursors, wax precursors (Eceriferums) and lignin precursors (Phenylalanine ammonia lyase: PAL and cinnamyl-alcohol-NADPH dehydrogenase: CAD) were down-regulated in old leaves at both time points. Thus, a reduced production of phenylpropanoid alcohols (coumaryl-, caffeyl-, coniferyl-, hydroxyconiferyl- and sinepsyl-alcohols) could be expected (e.g. files S9, S10).

The studies of Stadler [27] and Valsangiacomo and Gessler [28] showed that physical barriers, such as cuticle and papillae, were not linked to ontogenic resistance in apple. Our analysis confirmed this result since callose synthase genes, wax biosynthesis genes and lignin biosynthesis genes were found to be down-regulated in old leaves of both inoculated and uninoculated leaves compared to young ones.

#### Chemical defences

Chemical barriers, such pathogenesis related (PR-) proteins, were linked to age-related resistance in some pathosystems [19], [20]. In apple leaves, PR-proteins (β-glucanase, chitinase, endochitinase, thaumatine-like, defensin, oxalate oxidase, and protease inhibitor) encoding genes were found to be down-regulated in old leaves (L1 vs. L7) while other genes involved in metabolite production (peroxidase, lipid transfer proteins, and lipoxygenase) were up-regulated in general.

At 72 hpi (Figure 2, Table 2), a PR-protein gene (LTP: lipid transfer protein; MDP0000940078) showed increased gene expression in old leaves of both uninoculated and inoculated old leaves. At 96 hpi (Figure 3, Table 2), LTP was up-regulated only in uninoculated old leaves (L1N vs. L7N), and not in the inoculated (L1I vs. L7I) old ones with an FDR *P*-value correction of 0.0001. However, without considering the FDR *P*-value correction, we also found a statistically significant (*p* = 0.022) up-regulation of LTP also for the inoculated old leaves. The results of the qRT-PCR confirmed the differential LTP expression between young and old uninoculated apple leaves at both time points (Figure 4). LTPs have been suggested to be linked to antifungal activity through different possible paths upon pathogen attack [50]. A recent study [51] on LTPs showed the potential inhibition of germination and fungal growth *in vitro*. Moreover, the authors produced a transgenic tobacco plant overexpressing the LTP gene, resulting in inhibition of pathogen growth in this plant [51]. In *Malus-Venturia* pathosystem, the effect of LTPs may be connected to fungal growth inhibition rather than to inhibition of fungal germination, as no difference in conidia germination on leaves of different age has been observed [14].

Peroxidases (PXs) were linked to avirulent-microbe defence [52]. The biochemical functions of PXs were connected to the lignin and suberin biosynthesis [53] and to the regulation of reactive oxygen species (ROS) [54]. Silencing plant PX resulted in an increase in plant susceptibility [52] and its overexpression enhanced plant resistance [52], [55]. In *Capsicum annuum*, PX expression increased upon pathogen attack [52]. However in apple, PX3 (MDP0000149327) increased gene expression in old leaves has been observed in both inoculated and uninoculated leaves at 72 hpi. At 96 hpi, we could observe a significant up-regulation of PX3 for the uninoculated old leaves (Table 2) with the stringent FDR *P*-value correction procedure. However, without the FDR *P*-value correction procedure, also the marginally significant (*p* = 0.047) up-regulation for the inoculated old leaves could be observed. The analysis performed with the qRT-PCR confirmed the differential PX3 expression between young and old uninoculated apple leaves at both time points (Figure 4). The effect of PXs on *V. inaequalis* growth may be linked to strengthening of the cell wall and consequently reducing the nutrient availability necessary for the fungal growth [56].

Lipoxygenases (LOX) have been reported in numerous plant species [57] and are involved in the first step of jasmonate biosynthesis pathway [58]. Transformation of plants by LOX-silencing resulted in enhanced susceptibility to some microbial pathogens [59], [60]. In our experiment, LOX (MDP0000312397) encoding protein showed an increased expression in both uninoculated and inoculated old leaves at 72 hpi. At 96 hpi, we could observe a statistically significant up-regulation of LOX for the uninoculated old leaves but not for the old inoculated ones (Table 2) and as observed with other genes encoding proteins, the up-regulation of LOX in the inoculated old leaves was still significant (*p* = 0.024), but was discharged due to the stringent FDR *P*-value correction procedure. However, qRT-PCR results were in discordance with RNA-seq data (Figure 4). The increased expression of the LOX encoding protein may inhibit fungal growth by the production of fungal inhibitor oxylipin substances (e.g. hexanal and colnelenic acid) or by its own antimicrobial activity, as previously described [57], [61].

Metallothioneins (MT) are proteins connected to heavy metal detoxification in stressed plants [62]. In apple plants, MT protein encoding genes have been found [63], [64]. In our work, we observed an up-regulation of MT3-like protein encoding gene (MDP0000466190) in old leaves at both time points (Table 2). The results of the qRT-PCR confirmed the differential MT3 expression between young and old uninoculated leaves at both time points (Figure 4). The role of MTs in response to biotic stress is not fully understood; however, some suggestions were made: the up-regulation of MT3 may inhibit fungal growth through metal ion sequestration, leading to an unsuitable habitat for fungal growth, or by decreasing the fungal enzymatic activity [65]; thus, in both situations, an inhibition of fungal growth may be expected. However, since ontogenic resistance in old senescing leaves is no longer functional [66], further studies on MTs at the senescence stadium must be performed.

Tocopherol, part of the vitamin E group, has been postulated to have antioxidant qualities to maintain the chemical and physical properties of the epicuticular waxes [67]. This substance was found to improve fruit quality by decreasing disease incidence [68], [69]. In the work of Bringe et al. [70], an increase of tocopherol between leaf one and leaf seven has been observed. In our work, we found a constant down-regulation of genes involved in the biosynthesis of tocopherol in old leaves at both time points. This result does not contrast with the findings of Bringe et al. [70] if tocopherol, as suggested previously [71], accumulates in old leaves. However, at the onset of autumn, leaves lose their ontogenic resistance [66]; therefore, it is unlikely that tocopherol plays an important function in this resistance mechanism.

Phenols and flavonoids have been extensively studied in the past five decades in apple tissues [29], [72]–[76] apparently without any conclusive answer to the observed age-related resistance. In the present work, flavonoids and phenols precursor genes were down-regulated or not differentially expressed in old leaves of the conditions tested.

#### Old leaves as suitable substrate for fungal growth

After analysing structural and chemical defences in apple leaves, we focused on the suitability of old leaves for fungal growth in the early phase of tissue colonisation.

Fungal growth in artificial media has been investigated in the past. Leben and Keitt [77] showed that the best carbon sources for *V. inaequalis* were sugars and alcohols and the most suitable nitrogen sources were amino acids (arginine, glutamic acid, histidine and proline), urea and ammonia compounds (-sulphate and –phosphate). Thiamine was the only vitamin essential for fungal growth.

In the present study, we observed a general up-regulation of sugar biosynthesis genes and sugar transporter genes in old leaves at 72 hpi (Figure 2), while at 96 hpi (Figure 3), sugars transporter genes were down-regulated in uninoculated old leaves and up-regulated in the inoculated ones. Thus, it does not seem probable that sugar amount in old leaves plays an important role in apple ontogenic resistance.

Thiamine synthesis precursor genes did not show any differential regulation between young and old leaves at both time points, indicating that thiamine is not the limiting factor leading to ontogenic resistance.

Nitrate, ammonium, sulphate and phosphate transporter genes were up-regulated in both uninoculated and inoculated old leaves in most of the tested conditions. Genes involved in amino acid biosynthesis and cell wall precursor synthesis (cellulose and proline-rich proteins) appeared in general to be down-regulated in old leaves compared to young ones at 72 hpi, while at 96 hpi they displayed up-regulation in uninoculated old leaves (L1N vs. L7N) and down-regulation in the inoculated (L1I vs. L7I) old leaves. The transmembrane amino acid transporter encoding genes showed a down-regulation in both inoculated and uninoculated old leaves. However, the action of peroxidases and the consequent cell wall lignification may limit the diffusion of these nutrient compounds between the cell and the sub-cuticular space, limiting therefore the fungal growth [56].

Fothergill and Ashcroft [78] showed that *V. inaequalis* growth was stimulated at pH values above 5.8. Later works [74], [75] suggested a different pH between young (pH = 6) and old leaves (pH = 5). With these works it may be suggested that the fungal growth, as a result of sub-optimal growth conditions in old leaves, may be inhibited. In our work, we observed an up-regulation of proton transporter precursor genes in both uninoculated and inoculated old leaves at 72 hpi, while at 96 hpi they were down-regulated in both uninoculated and inoculated old leaves. Magnesium ion transmembrane transporter genes did not show any differential expression at 72 hpi and were down-regulated in both uninoculated and inoculated old leaves at 96 hpi. Potassium, sodium, chlorine, and calcium ion transporter genes showed a down-regulation in old leaves compared to young ones at both time points. Other unspecified anion transporter genes were down-regulated. However, in the work of Raa [74] and Raa and Overeem [75], the difference in pH between leaves of different ages was determined with leaf homogenates, which make the assumption of a different pH between young and old leaves difficult to prove with RNA-seq experiments and to connect to *V. inaequalis* growth. In fact, this pathogen invaded only the sub-cuticular space of the leaf, thus the acidity of the sub-cuticular space would be a better factor to analyse in future researches.

### Functional annotation of the most abundant transcripts

**Figure 5:**
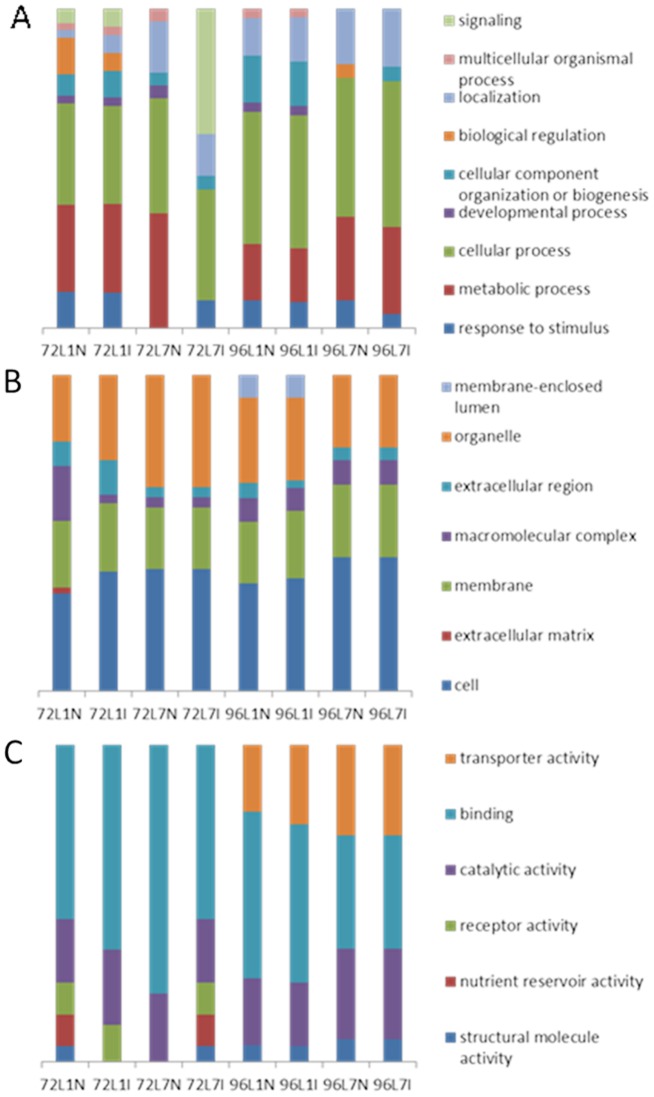
Gene ontology (GO) functional annotation of the 20 most abundant transcripts of each cDNA library. L1 and L7 correspond to Leaf 1 and Leaf 7 for *V. inaequalis* inoculated (I) and uninoculated (N) leaves at 72 and 96 hours post inoculation. The analysis was performed with Blast2Go v. 2.5.1 using the level 2 GO vocabulary: (A) Biological process terms, (B) Cellular component terms and (C) Molecular function terms.

Analysis with the level 2 GO vocabulary of young (L1) and old (L7), inoculated (I) and uninoculated (N) leaves at both time points (72 and 96 hpi) was performed for the 20 most abundant transcripts found in each tested condition (File S11), using the three GO classes, *i.e*. biological process, molecular functions, and cellular components (Figure 5). At 72 hpi, 50% of the 20 most abundant transcripts were present in both uninoculated and inoculated young leaves (L1), while 80% of the transcripts were present in both inoculated and uninoculated old leaves (L7). At this time point, only one gene encoding protein (5%) was present in all tested conditions (File S11). At 96 hpi, 95% of the 20 most abundant transcripts were common in both inoculated and uninoculated young (L1) leaves. The same proportion could also be observed in the old leaves (L7). At this time point, eleven (55%) gene encoding proteins could be found in all tested conditions (File S11).

#### Biological process

At 72 hpi, inoculation of young leaves (L1N vs. L1I) resulted in a decrease of biological regulation terms (GO:0065007), cellular process (GO:0009987), metabolic process (GO:0008152), and response to stimulus (GO:0050896), while localisation terms (GO:0051179) increased. Inoculation of old leaves (L7N vs. L7I) resulted in an increased response to stimulus (GO:0050896), and signalling (GO:0023052), while metabolic process (GO:0008152), cellular process (GO:0009987), developmental process (GO:0032502), localisation components (GO:0051179), and multicellular organismal process (GO:0032501) decreased (Figure 5A). Inoculation of old leaves (L7N vs. L7I) led to an increased response to stimulus and signalling of biological components, while metabolic processes showed the most significant decrease. Signalling components indicate the transmission of information within a biological system and ends up with a cellular response. In our work, signalling may be connected to the pathogen perception and induction of resistance response. The response to stimulus component was connected to high expression of two PR-10 protein genes (both coding for Mal d 1.0105) found in old inoculated leaves at 72 hpi. The biological function of PR-10 proteins is not entirely known, but some work suggests that the PR-10 protein possesses a ribonuclease activity [79], [80], which may prevent fungal growth [81] in host plants. The underrepresentation of metabolic process terms in inoculated old leaves was connected to reduced metabolism, which was probably also influenced by the decreased photosynthesis detected upon inoculation, as observed in other crop plants [82].

At 96 hpi, we did not observe any major changes between inoculated and uninoculated young leaves: a slight decrease of cellular process (GO:0009987) and localisation terms (GO:0051179) was observed. Old inoculated leaves showed a decrease in response to stimulus terms (GO:0050896) and biological regulation terms (GO:0065007) (Figure 5A).

#### Cellular components

Inoculation of young leaves (L1N vs. L1I) resulted in a decrease of cellular components (GO:0005623), extracellular matrix components (GO:0031012), organelle components (GO:0043226), membrane components (GO:0016020), and macromolecular complex components (GO:0032991) at 72 and 96 hpi (Figure 5B). The “macromolecular complex” components showed a decrease of terms from 9 to 1 between uninoculated and inoculated young leaves. The macromolecular complex term implies a stable assembly of two or more macromolecules in which both constituents function together. In our experiment, this function is highly underrepresented in inoculated young leaves. In the context of leaf inoculation, this was shown by decreased photosynthesis due to a decrease of photosystem I reaction centre subunit XI, chlorophyll a-b binding protein 4 precursor, photosystem II reaction centre w chloroplastic, and light-harvesting complex II protein lhcb3. The inhibition of photosynthesis upon pathogen attack has already been reported [83].

Inoculation of old leaves (L7N vs. L7I) did not show any major difference at any time point (72 and 96 hpi). Between young and old leaves, differences were found principally between the cell components, which decreased from young to old leaves at both time points (Figure 5B).

#### Molecular functions

At 72 hpi, inoculation of young leaves (L1N vs. L1I) showed only minor changes in structural molecule activity terms (GO:0005198) and nutrient reservoir activity terms (GO:0045735), while at 96 hpi, differences were found only for transporter activity terms (GO:0005215). Inoculation of old leaves (L7N vs. L7I) showed an increase of structural molecule activity terms (GO:0005198), of nutrient reservoir activity terms (GO:0045735), of receptor activity terms (GO:0004872), and catalytic activity terms (GO:0003824) at 72 hpi, while no differences between inoculated and uninoculated leaves were found at 96 hpi (Figure 5C).

## Conclusions

The RNA-seq technology is becoming an important research tool to investigate plant-pathogen interaction at the transcriptomic level. In this work, we described for the first time genes that are differentially expressed during the shift from susceptibility of young leaves to the resistance of old leaves of *Malus x domestica*, challenged or not with *Venturia inaequalis*, using a transciptomic approach.

In this RNA-seq experiment the importance of using at least three biological replicates was shown: the standard deviation of the averaged RPKM values ranged from 1% to 98%. The high biological variability was also observed during the validation of the five candidate gene encoding proteins by means of the real-time quantitative reverse-transcription PCR.

Nevertheless, we could propose five candidate gene encoding proteins linked to ontogenic resistance of apple: a metallothionein 3-like, a lipoxygenase, a lipid transfer protein, an enhanced disease susceptibility 1 protein and a peroxidase 3.

The results presented in this study suggest that the ontogenic resistance in apple is the consequence of the fungal growth inhibition, either due to metal ion sequestration and inhibition of pathogens' enzymatic activity (MT3), low mineral diffusion between the cell and the sub-cuticular space (PX3), secondary substances produced (LOX), or by the direct action of specific enzymes (LTP and LOX). Moreover, the transduction signal effect due to EDS1 needs further studies to determine the influence of this gene on the ontogenic resistance in apple.

Further works, to test the relative expression of the candidate genes in several unrelated apple genotypes is needed. Additionally, the correlation between gene expression, protein and metabolite levels by means of proteomic and metabolomics approaches is desired to further determine the contribution of these genes to ontogenic resistance in apple.

## Supporting Information

File S1 **List of primer pairs used for qRT-PCR validation of RNA-seq data.**
(DOC)

File S2 **Complete list of the genes differentially expressed in all tested conditions.** Data were generated with the CLC Genomics Workbench v. 5.5.1 with a FDR *P*-value correction of 0.0001.
(XLSX)

File S3 **Mercator's Bins mapping used for the functional annotation and pathway analysis of the 5823 differentially expressed genes obtained with the CLC Genomics Workbench v. 5.5.1 with a FDR** ***P*** **-value correction of 0.0001.**
(TIF)

File S4 **Mercator's Bins mapping file of the 5823 differentially expressed genes of apple.**
(DOCX)

File S5 **Biological variation of the transcription level observed for the five candidate genes involved in the ontogenic resistance of apple.**
(XLS)

File S6 **Overview of the 127 pathways assigned with the KEGG pathway analysis of the 5823 differentially expressed genes obtained with the CLC Genomics Workbench v. 5.5.1 with a FDR** ***P*** **-value correction of 0.0001.**
(XLSX)

File S7 **KEGG map for plant hormone signal transduction enriched with the genes differentially expressed found with MapMan v. 3.5.1 at 72 hpi between uninoculated leaf 1 and leaf 7.**
(JPG)

File S8 **KEGG map for plant hormone signal transduction enriched with the genes differentially expressed found with MapMan v. 3.5.1 at 72 hpi between inoculated leaf 1 and leaf 7.**
(JPG)

File S9 **KEGG map for cell wall and lignin precursors enriched with the genes differentially expressed found with MapMan v. 3.5.1 at 72 hpi between uninoculated leaf 1 and leaf 7.**
(JPG)

File S10 **KEGG map for cell wall and lignin precursors enriched with the genes differentially expressed found with MapMan v. 3.5.1 at 72 hpi between inoculated leaf 1 and leaf 7.**
(JPG)

File S11 **List of the 20 most abundant transcripts found in each cDNA library.**
(XLSX)
