# Supplementary material for: RNA-Seq Analysis Reveals Candidate Genes for Ontogenic Resistance in Malus-Venturia Pathosystem
Source: PLoS One. 2013 Nov 4;8(11):e78457. doi: 10.1371/journal.pone.0078457 (PMC3817206; doi:10.1371/journal.pone.0078457)
Supplement: File S1 — List of primer pairs used for qRT-PCR validation of RNA-seq data. (DOC) [file pone.0078457.s001.doc]

File S1

| **Accession Number ^a^** |  | **Name ^b^** | **Sequence (5’- 3’)** | **Start** | **Stop** | **Tm ^c^** | **GC(%)** | **Product size** |
| --- | --- | --- | --- | --- | --- | --- | --- | --- |
| MDP0000253215 | *Enhanced Disease Susceptibility 1* | EDS1_F_  EDS1_R_ | GGAGTTGCACGCACTTTCTG  TTGCTGAGAACCCCATCCAC | 1623  1733 | 1642  1714 | 60.04  59.96 | 55  55 | 111 |
| MDP0000322397 | *Lipoxygenase* | LOX_F_  LOX_R_ | CTTACGCCGGATACCTTCCC  CGTGTCTCGCTTACCCAAGT | 2942  3135 | 2961  3134 | 59.97  60.04 | 60  55 | 212 |
| MDP0000940078 | *Lipid Transfer Protein* | LTP_F_  LTP_R_ | GGAGTGTTTGGAGGCACAGT  GGAGAGCCACAGACATCACC | 121  299 | 140  280 | 60.18  60.11 | 55  60 | 179 |
| MDP0000466190 | *Metallothionein 3* | MT3_F_  MT3_R_ | GGCTACGACTTGGTGATCGT  ACTGACCACAGGTGCAGTTC | 64  199 | 83  180 | 59.83  60.18 | 55  55 | 136 |
| MDP0000149327 | *Peroxidase 3* | PX3_F_  PX3_R_ | CCTGGAAGCGTCAGGACTTT  GCAGTGAACCTTTGAGCAGC | 730  868 | 749  849 | 59.96  60.04 | 55  55 | 139 |

*^a^ Accession number: Genome Database for Rosaceae (GDR:* [*http://www.rosaceae.org*](http://www.rosaceae.org/) *)*

*^b^ F = Forward Primer; R = Reverse Primer*

*^c^ Tm: melting temperature*
