# Supplementary figures and images for: RNA-Seq Analysis Reveals Candidate Genes for Ontogenic Resistance in Malus-Venturia Pathosystem
Source: PLoS One. 2013 Nov 4;8(11):e78457. doi: 10.1371/journal.pone.0078457 (PMC3817206; doi:10.1371/journal.pone.0078457)

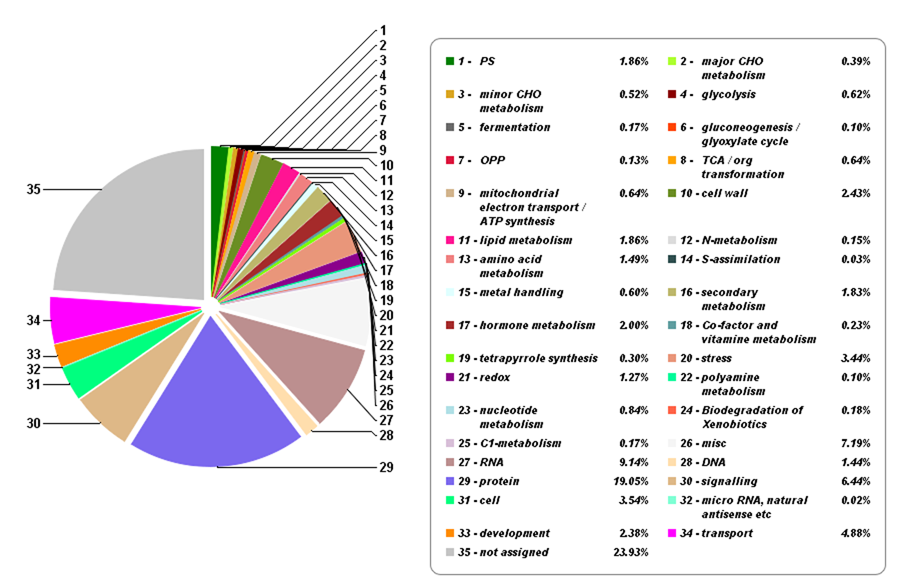

Supplement: File S3 — Mercator's Bins mapping used for the functional annotation and pathway analysis of the 5823 differentially expressed genes obtained with the CLC Genomics Workbench v. 5.5.1 with a FDR P -value correction of 0.0001. (TIF) [file pone.0078457.s003.tif]

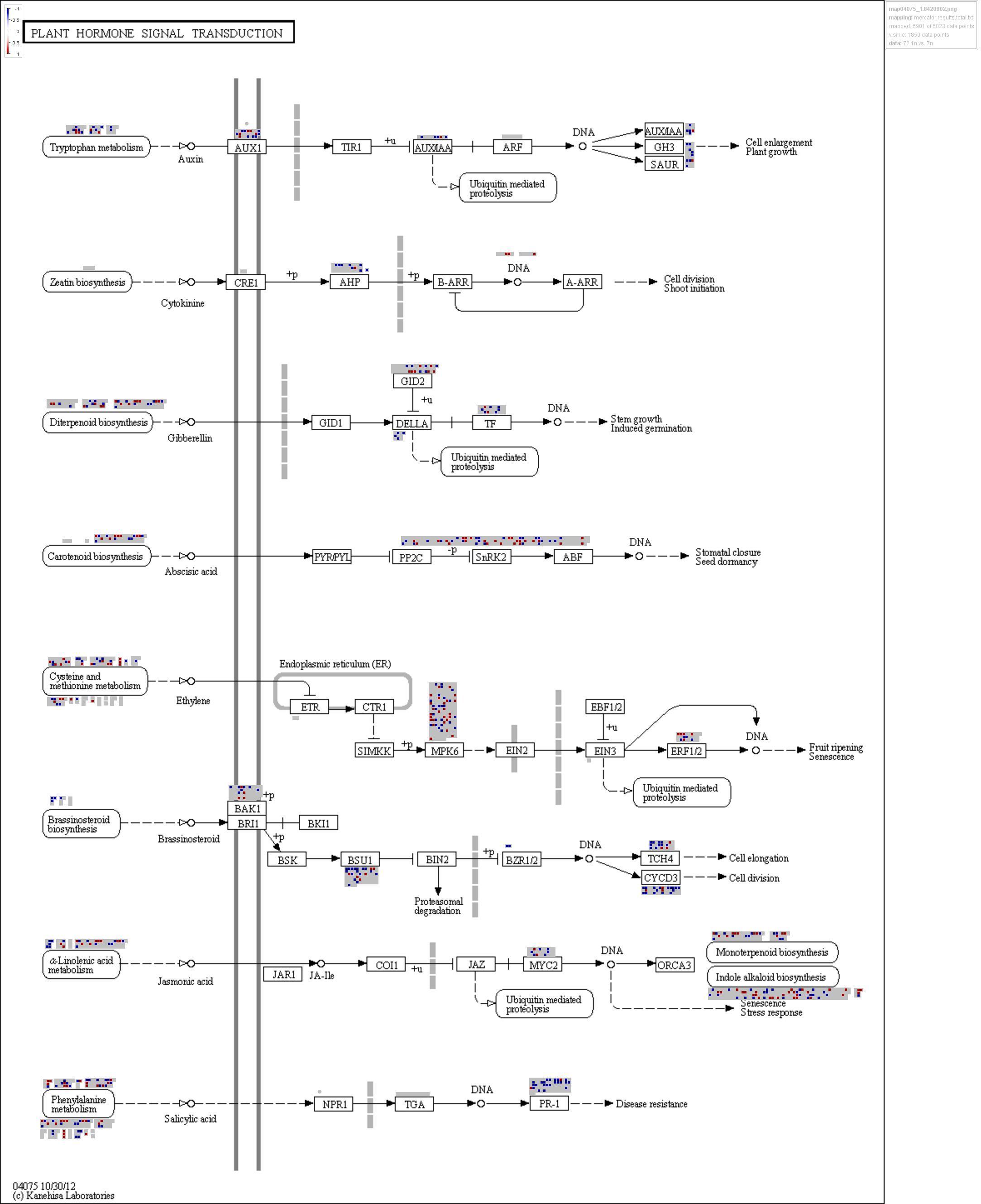

Supplement: File S7 — KEGG map for plant hormone signal transduction enriched with the genes differentially expressed found with MapMan v. 3.5.1 at 72 hpi between uninoculated leaf 1 and leaf 7. (JPG) [file pone.0078457.s007.jpg]

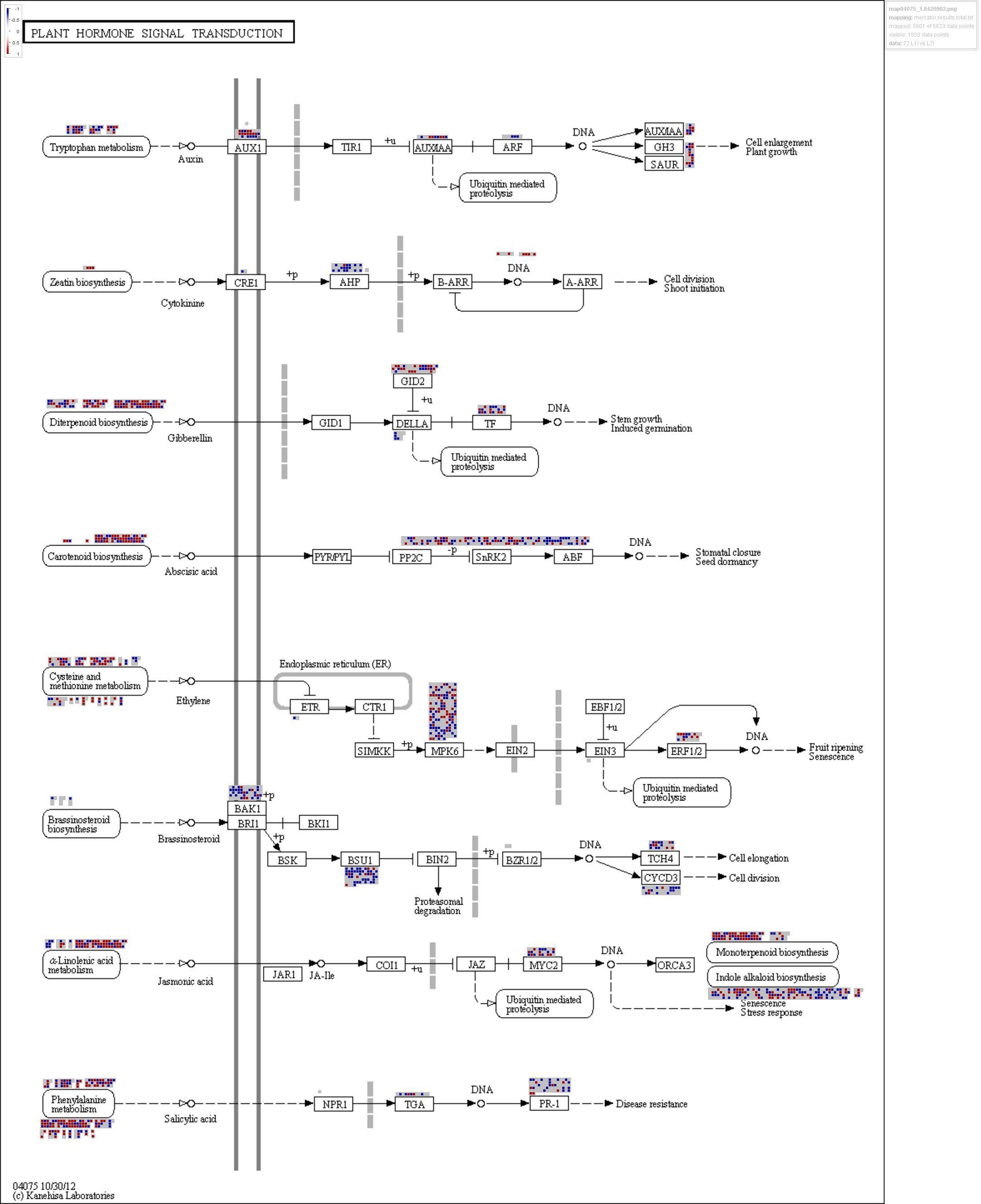

Supplement: File S8 — KEGG map for plant hormone signal transduction enriched with the genes differentially expressed found with MapMan v. 3.5.1 at 72 hpi between inoculated leaf 1 and leaf 7. (JPG) [file pone.0078457.s008.jpg]

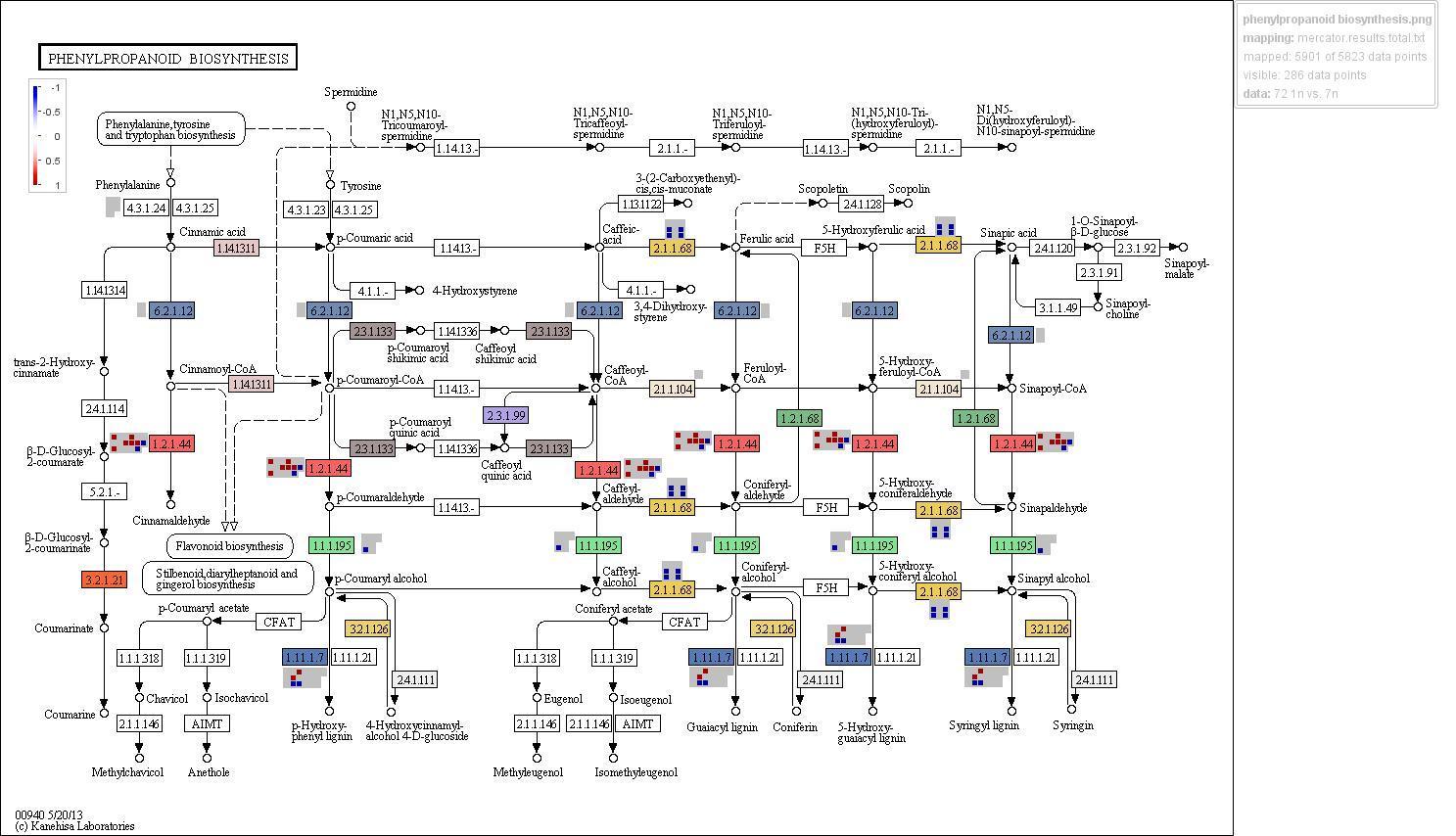

Supplement: File S9 — KEGG map for cell wall and lignin precursors enriched with the genes differentially expressed found with MapMan v. 3.5.1 at 72 hpi between uninoculated leaf 1 and leaf 7. (JPG) [file pone.0078457.s009.jpg]

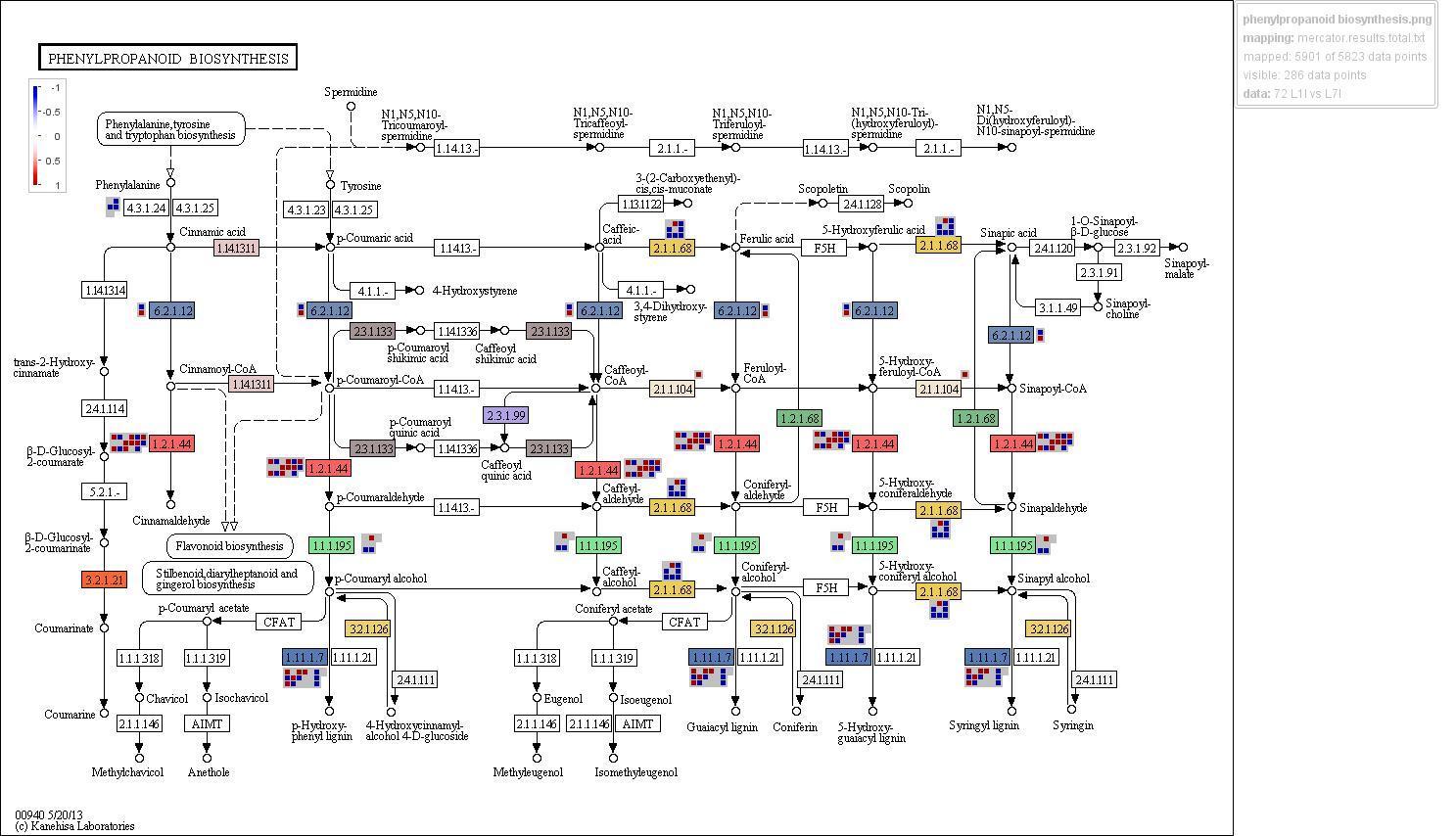

Supplement: File S10 — KEGG map for cell wall and lignin precursors enriched with the genes differentially expressed found with MapMan v. 3.5.1 at 72 hpi between inoculated leaf 1 and leaf 7. (JPG) [file pone.0078457.s010.jpg]
