# Supplementary material for: RNA-Seq Analysis Reveals Candidate Genes for Ontogenic Resistance in Malus-Venturia Pathosystem
Source: PLoS One. 2013 Nov 4;8(11):e78457. doi: 10.1371/journal.pone.0078457 (PMC3817206; doi:10.1371/journal.pone.0078457)
Supplement: File S4 — Mercator's Bins mapping file of the 5823 differentially expressed genes of apple. (DOCX) [file pone.0078457.s004.docx]

BINCODE NAME IDENTIFIER DESCRIPTION TYPE

-1 root

0 control genes

1 PS

1.1 PS.lightreaction

1.1.1 PS.lightreaction.photosystem II

1.1.1.1 PS.lightreaction.photosystem II.LHC-II

1.1.1.2 PS.lightreaction.photosystem II.PSII polypeptide subunits

1.1.1.3 PS.lightreaction.photosystem II.biogenesis

1.1.1.4 PS.lightreaction.photosystem II.LHC biogenesis

1.1.1001 PS.lightreaction

1.1.1002 PS.lightreaction.binding proteins.Chlorophyll a

1.1.1003 PS.lightreaction.binding proteins.Chlorophyll b

1.1.1004 PS.lightreaction.PS II.PQ

1.1.2 PS.lightreaction.photosystem I

1.1.2.1 PS.lightreaction.photosystem I.LHC-I

1.1.2.2 PS.lightreaction.photosystem I.PSI polypeptide subunits

1.1.2.3 PS.lightreaction.photosystem I.biogenesis

1.1.3 PS.lightreaction.cytochrome b6/f

1.1.3.1 PS.lightreaction.cytochrome b6/f.iron sulfur subunit

1.1.3.2 PS.lightreaction.cytochrome b6/f.subunit 4 (PETD)

1.1.3.3 PS.lightreaction.cytochrome b6/f.cytochrome b6 (CYB6)

1.1.3.4 PS.lightreaction.cytochrome b6/f.apocytochrome f (CYF)

1.1.3.5 PS.lightreaction.cytochrome b6/f.biogenesis

1.1.30 PS.lightreaction.state transition

1.1.4 PS.lightreaction.ATP synthase

1.1.4.1 PS.lightreaction.ATP synthase.alpha subunit

1.1.4.2 PS.lightreaction.ATP synthase.beta subunit

1.1.4.3 PS.lightreaction.ATP synthase.epsilon chain

1.1.4.4 PS.lightreaction.ATP synthase.gamma chain

1.1.4.5 PS.lightreaction.ATP synthase.subunit C

1.1.4.6 PS.lightreaction.ATP synthase.chloroplastic subunit a

1.1.4.7 PS.lightreaction.ATP synthase.delta chain

1.1.4.8 PS.lightreaction.ATP synthase.subunit B (ATPF)

1.1.4.9 PS.lightreaction.ATP synthase.subunit B_ (ATPX)

1.1.40 PS.lightreaction.cyclic electron flow-chlororespiration

1.1.5 PS.lightreaction.other electron carrier (ox/red)

1.1.5.1 PS.lightreaction.other electron carrier (ox/red).plastocyanin

1.1.5.2 PS.lightreaction.other electron carrier (ox/red).ferredoxin

1.1.5.3 PS.lightreaction.other electron carrier (ox/red).ferredoxin reductase

1.1.5.4 PS.lightreaction.other electron carrier (ox/red).ferredoxin oxireductase

1.1.50 PS.lightreaction.NPQ

1.1.6 PS.lightreaction.NADH DH

1.1.60 PS.lightreaction.state transition

1.1.70 PS.lightreaction.hydrogenase

1.1.70.1 PS.lightreaction.hydrogenase.FeFe-hydrogenase

1.1.70.2 PS.lightreaction.hydrogenase.NiFe-hydrogenase

1.1.99 PS.lightreaction.unspecified

1.1.99.1 PS.lightreaction.unspecified.TEF

1.2 PS.photorespiration

1.2.1 PS.photorespiration.phosphoglycolate phosphatase

1.2.1001 PS.photorespiration.glycerate

1.2.1002 PS.photorepiration.glycolate

1.2.1003 PS.photorepiration.glycine

1.2.1004 PS.photorepiration.serine

1.2.1005 PS.photorepiration.glyoxylate

1.2.1006 PS.photorepiration.hydroxypyruvate

1.2.2 PS.photorespiration.glycolate oxydase

1.2.3 PS.photorespiration.aminotransferases peroxisomal

1.2.4 PS.photorespiration.glycine cleavage

1.2.4.1 PS.photorespiration.glycine cleavage.P subunit

1.2.4.2 PS.photorespiration.glycine cleavage.T subunit

1.2.4.3 PS.photorespiration.glycine cleavage.L subunit

1.2.4.4 PS.photorespiration.glycine cleavage.H protein

1.2.5 PS.photorespiration.serine hydroxymethyltransferase

1.2.6 PS.photorespiration.hydroxypyruvate reductase

1.2.7 PS.photorespiration.glycerate kinase

1.3 PS.calvin cycle

1.3.1 PS.calvin cycle.rubisco large subunit

1.3.10 PS.calvin cycle.Rib5P Isomerase

1.3.1001 PS.calvin cycle.ribulose-1,5-bisP

1.3.1002 PS.calvin cycle

1.3.1003 PS.calvin cycle

1.3.1004 PS.calvin cycle

1.3.1005 PS.calvin cycle

1.3.1006 PS.calvin cycle

1.3.1007 PS.calvin cycle

1.3.11 PS.calvin cycle.RPE

1.3.12 PS.calvin cycle.PRK

1.3.13 PS.calvin cycle.rubisco interacting

1.3.2 PS.calvin cycle.rubisco small subunit

1.3.3 PS.calvin cycle.phosphoglycerate kinase

1.3.4 PS.calvin cycle.GAP

1.3.5 PS.calvin cycle.TPI

1.3.6 PS.calvin cycle.aldolase

1.3.7 PS.calvin cycle.FBPase

1.3.8 PS.calvin cycle.transketolase

1.3.9 PS.calvin cycle.seduheptulose bisphosphatase

1.4 PS.reductive PP

1.5 PS.carbon concentrating mechanism

1.5.1 PS.carbon concentrating mechanism.C4

1.5.2 PS.carbon concentrating mechanism.CAM

1.5.3 PS.carbon concentrating mechanism.algal

10 cell wall

10.1 cell wall.precursor synthesis

10.1.1 cell wall.precursor synthesis.NDP sugar pyrophosphorylase

10.1.1.1 cell wall.precursor synthesis.NDP sugar pyrophosphorylase.GDP mannose

10.1.1.2 cell wall.precursor synthesis.NDP sugar pyrophosphorylase.GDP fucose and fucokinase

10.1.1.20 cell wall.precursor synthesis.NDP sugar pyrophosphorylase.multiple NDP-Sugars

10.1.10 cell wall.precursor synthesis.UDP-glucose 4,6-dehydratase

10.1.11 cell wall.precursor synthesis.UER

10.1.12 cell wall.precursor synthesis.KDO pathway

10.1.12.1 cell wall.precursor synthesis.KDO pathway.arabinose-5-phosphate isomerase

10.1.12.2 cell wall.precursor synthesis.KDO pathway.KDO-8-Phosphate Synthase

10.1.12.3 cell wall.precursor synthesis.KDO pathway.KDO-8-Phosphate Phosphatase

10.1.12.4 cell wall.precursor synthesis.KDO pathway.CMP-KDO Synthetase

10.1.2 cell wall.precursor synthesis.UGE

10.1.20 cell wall.precursor synthesis.phosphomannose isomerase

10.1.21 cell wall.precursor synthesis.phosphomannomutase

10.1.3 cell wall.precursor synthesis.AXS

10.1.30 cell wall.precursor synthesis.sugar kinases

10.1.30.1 cell wall.precursor synthesis.sugar kinases.galacturonic acid kinase

10.1.30.2 ell wall.precursor synthesis.sugar kinases.arabinose-1-kinase

10.1.30.3 cell wall.precursor synthesis.sugar kinases.glucuronic acid kinase

10.1.4 cell wall.precursor synthesis.UGD

10.1.5 cell wall.precursor synthesis.UXS

10.1.6 cell wall.precursor synthesis.GAE

10.1.7 cell wall.precursor synthesis.GMD

10.1.8 cell wall.precursor synthesis.GER

10.1.9 cell wall.precursor synthesis.MUR4

10.2 cell wall.cellulose synthesis

10.2.1 cell wall.cellulose synthesis.cellulose synthase

10.2.1001 cell wall degradation

10.2.1002 cell wall degradation

10.2.1003 cell wall degradation

10.2.1004 cell wall degradation

10.2.1005 cell wall degradation

10.2.1006 cell wall degradation

10.2.1007 cell wall degradation

10.2.1008 cell wall degradation

10.2.1009 cell wall degradation

10.2.1010 cell wall degradation

10.2.1011 cell wall degradation

10.2.1012 cell wall degradation

10.2.1013 cell wall degradation

10.2.2 cell wall.cellulose synthesis.COBRA

10.3 cell wall.hemicellulose synthesis

10.3.1 cell wall.hemicellulose synthesis.xyloglucan

10.3.1.1 cell wall.hemicellulose synthesis.xyloglucan.XXXG galactose Transferase

10.3.2 cell wall.hemicellulose synthesis.glucuronoxylan

10.3.3 cell wall.hemicellulose synthesis.arabinoxylan

10.3.4 cell wall.hemicellulose synthesis.glucomannan

10.3.5 cell wall.hemicellulose synthesis.galactomannan

10.4 cell wall.pectin synthesis

10.4.1 cell wall.pectin synthesis.homogalacturonan

10.4.1.1 cell wall.pectin synthesis.homogalacturonan.Galacturonic Acid Trnasferase

10.4.2 cell wall.pectin synthesis.rhamnogalacturonan I

10.4.3 cell wall.pectin synthesis.rhamnogalacturonan II

10.4.3.6 cell wall.pectin synthesis.rhamnogalacturonan II.Xylose Transferase with Fucose Acceptor

10.4.4 cell wall.pectin synthesis.Xylogalacturonan

10.4.4.1 cell wall.pectin synthesis.Xylogalacturonan.Xylose Transferase

10.5 cell wall.cell wall proteins

10.5.1 cell wall.cell wall proteins.AGPs

10.5.1.1 cell wall.cell wall proteins.AGPs.AGP

10.5.1.2 cell wall.cell wall proteins.AGPs.AGP Fucosyltransferase

10.5.2 cell wall.cell wall proteins.proline rich proteins

10.5.3 cell wall.cell wall proteins.LRR

10.5.4 cell wall.cell wall proteins.HRGP

10.5.5 cell wall.cell wall proteins.RGP

10.6 cell wall.degradation

10.6.1 cell wall.degradation.cellulases and beta -1,4-glucanases

10.6.1001 cell wall.degradation

10.6.2 cell wall.degradation.mannan-xylose-arabinose-fucose

10.6.3 cell wall.degradation.pectate lyases and polygalacturonases

10.7 cell wall.modification

10.8 cell wall.pectin*esterases

10.8.1 cell wall.pectin*esterases.PME

10.8.2 cell wall.pectin*esterases.acetyl esterase

10.8.99 cell wall.pectin*esterases.misc

11 lipid metabolism

11.1 lipid metabolism.FA synthesis and FA elongation

11.1.01 lipid metabolism.FA synthesis and FA elongation.Acetyl CoA Carboxylation

11.1.1 lipid metabolism.FA synthesis and FA elongation.Acetyl CoA Carboxylation

11.1.1.1 lipid metabolism.FA synthesis and FA elongation.Acetyl CoA Carboxylation.homomeric Enzyme

11.1.1.2 lipid metabolism.FA synthesis and FA elongation.Acetyl CoA Carboxylation.heteromeric Complex

11.1.1.2.1 lipid metabolism.FA synthesis and FA elongation.Acetyl CoA Carboxylation.heteromeric Complex.alpha Carboxyltransferase

11.1.1.2.2 lipid metabolism.FA synthesis and FA elongation.Acetyl CoA Carboxylation.heteromeric Complex.beta Carboxyltransferase

11.1.1.2.3 lipid metabolism.FA synthesis and FA elongation.Acetyl CoA Carboxylation.heteromeric Complex.Biotin Carboxyl Carrier Protein

11.1.1.2.4 lipid metabolism.FA synthesis and FA elongation.Acetyl CoA Carboxylation.heteromeric Complex.Biotin Carboxylase

11.1.10 lipid metabolism.FA synthesis and FA elongation.beta ketoacyl CoA synthase

11.1.1001 lipid metabolism.FA synthesis and FA elongation

11.1.1002 lipid metabolism.FA synthesis and FA elongation

11.1.1003 lipid metabolism.FA synthesis and FA elongation

11.1.11 lipid metabolism.FA synthesis and FA elongation.fatty acid elongase

11.1.12 lipid metabolism.FA synthesis and FA elongation.ACP protein

11.1.13 lipid metabolism.FA synthesis and FA elongation.acyl-CoA binding protein

11.1.15 lipid metabolism.FA synthesis and FA elongation.ACP desaturase

11.1.2 lipid metabolism.FA synthesis and FA elongation.Acetyl CoA Transacylase

11.1.20 lipid metabolism.FA synthesis and FA elongation.MCD

11.1.3 lipid metabolism.FA synthesis and FA elongation.ketoacyl ACP synthase

11.1.30 lipid metabolism.FA synthesis and FA elongation.pyruvate kinase

11.1.31 lipid metabolism.FA synthesis and FA elongation.pyruvate DH

11.1.4 lipid metabolism.FA synthesis and FA elongation.ACP oxoacyl reductase

11.1.5 lipid metabolism.FA synthesis and FA elongation.beta hydroxyacyl ACP dehydratase

11.1.6 lipid metabolism.FA synthesis and FA elongation.enoyl ACP reductase

11.1.7 lipid metabolism.FA synthesis and FA elongation.ACP thioesterase

11.1.8 lipid metabolism.FA synthesis and FA elongation.acyl coa ligase

11.1.9 lipid metabolism.FA synthesis and FA elongation.long chain fatty acid CoA ligase

11.10 lipid metabolism.glycolipid synthesis

11.10.1 lipid metabolism.glycolipid synthesis.MGDG synthase

11.10.1001 lipid metabolism.galactolipid synthesis

11.10.1002 lipid metabolism.galactolipid synthesis

11.10.2 lipid metabolism.glycolipid synthesis.DGDG synthase

11.10.3 lipid metabolism.glycolipid synthesis.UDP-sulfoquinovose synthase

11.10.4 lipid metabolism.glycolipid synthesis.sulfolipid synthase

11.1001 lipid metabolism

11.1002 lipid metabolism

11.1003 lipid metabolism

11.1004 lipid metabolism

11.1005 lipid metabolism

11.1006 lipid metabolism

11.1007 lipid metabolism

11.1008 lipid metabolism

11.1009 lipid metabolism

11.1010 lipid metabolism

11.1011 lipid metabolism

11.1012 lipid metabolism

11.1013 lipid metabolism

11.1014 lipid metabolism

11.1015 lipid metabolism

11.1016 lipid metabolism

11.1017 lipid metabolism

11.1018 lipid metabolism

11.1019 lipid metabolism

11.1020 lipid metabolism

11.1021 lipid metabolism

11.1022 lipid metabolism

11.1023 lipid metabolism

11.1024 lipid metabolism

11.1025 lipid metabolism

11.1026 lipid metabolism

11.1027 lipid metabolism

11.1028 lipid metabolism

11.1029 lipid metabolism

11.1030 lipid metabolism

11.1031 lipid metabolism

11.1032 lipid metabolism

11.1033 lipid metabolism

11.1034 lipid metabolism

11.1035 lipid metabolism

11.1036 lipid metabolism

11.1037 lipid metabolism

11.1038 lipid metabolism

11.1039 lipid metabolism

11.1040 lipid metabolism

11.1041 lipid metabolism

11.2 lipid metabolism.FA desaturation

11.2.1 lipid metabolism.FA desaturation.desaturase

11.2.1001 lipid metabolism.FA desaturation

11.2.2 lipid metabolism.FA desaturation.a hydroxylase

11.2.3 lipid metabolism.FA desaturation.omega 3 desaturase

11.2.4 lipid metabolism.FA desaturation.omega 6 desaturase

11.3 lipid metabolism.Phospholipid synthesis

11.3.1 lipid metabolism.Phospholipid synthesis.1-acylglycerol-3-phosphate O-acyltransferase

11.3.10 lipid metabolism.Phospholipid synthesis.(S)-coclaurine-N-methyltransferase

11.3.2 lipid metabolism.Phospholipid synthesis.choline kinase

11.3.3 lipid metabolism.Phospholipid synthesis.phosphatidate cytidylyltransferase

11.3.4 lipid metabolism.Phospholipid synthesis.CDP-diacylglycerol-inositol 3-phosphatidyltransferase

11.3.5 lipid metabolism.Phospholipid synthesis.diacylglycerol kinase

11.3.6 lipid metabolism.Phospholipid synthesis.choline-phosphate cytidylyltransferase

11.3.7 lipid metabolism.Phospholipid synthesis.cyclopropane-fatty-acyl-phospholipid synthase

11.3.8 lipid metabolism.Phospholipid synthesis.phosphatidylserine decarboxylase

11.3.9 lipid metabolism.Phospholipid synthesis.choline monooxygenase

11.4 lipid metabolism.TAG synthesis

11.5 lipid metabolism.glyceral metabolism

11.5.1 lipid metabolism.glyceral metabolism.glycerol kinase

11.5.1001 lipid metabolism.glyceral metabolism

11.5.1002 lipid metabolism.glyceral metabolism

11.5.2 lipid metabolism.glyceral metabolism.Glycerol-3-phosphate dehydrogenase (NAD+)

11.5.3 lipid metabolism.glyceral metabolism.FAD-dependent glycerol-3-phosphate dehydrogenase

11.6 lipid metabolism.lipid transfer proteins etc

11.7 lipid metabolism.unassigned

11.8 lipid metabolism.'exotics'(steroids, squalene etc)

11.8.1 lipid metabolism.'exotics' (steroids, squalene etc).sphingolipids

11.8.1.1 lipid metabolism.'exotics' (steroids, squalene etc).sphingolipids.ceramidase

11.8.1.2 lipid metabolism.'exotics' (steroids, squalene etc).sphingolipids.serine C-palmitoyltransferase

11.8.1.3 lipid metabolism.''exotics'' (steroids, squalene etc).sphingolipids.ceramide glucosyltransferase

11.8.10 lipid metabolism.'exotics' (steroids, squalene etc).phosphatidylcholinesterol O-acyltransferase

11.8.1001 lipid metabolism.'exotics' (steroids, squalene etc)

11.8.1002 lipid metabolism.'exotics' (steroids, squalene etc)

11.8.1003 lipid metabolism.'exotics' (steroids, squalene etc)

11.8.1004 lipid metabolism.'exotics' (steroids, squalene etc)

11.8.1005 lipid metabolism.'exotics' (steroids, squalene etc)

11.8.1006 lipid metabolism.'exotics' (steroids, squalene etc)

11.8.1007 lipid metabolism.'exotics' (steroids, squalene etc)

11.8.1008 lipid metabolism.'exotics' (steroids, squalene etc)

11.8.1009 lipid metabolism.'exotics' (steroids, squalene etc)

11.8.1010 lipid metabolism.'exotics' (steroids, squalene etc)

11.8.1011 lipid metabolism.'exotics' (steroids, squalene etc)

11.8.1012 lipid metabolism.'exotics' (steroids, squalene etc)

11.8.1013 lipid metabolism.'exotics' (steroids, squalene etc)

11.8.2 lipid metabolism.'exotics' (steroids, squalene etc).methylsterol monooxygenase

11.8.3 lipid metabolism.'exotics' (steroids, squalene etc).UDP-glucose:sterol glucosyltransferase

11.8.4 lipid metabolism.'exotics' (steroids, squalene etc).3-beta hydroxysteroid dehydrogenase/isomerase

11.8.5 lipid metabolism.'exotics' (steroids, squalene etc).squalene monooxygenase

11.8.6 lipid metabolism.'exotics' (steroids, squalene etc).cycloartenol synthase

11.8.7 lipid metabolism.'exotics' (steroids, squalene etc).trans-2-enoyl-CoA reductase (NADPH)

11.8.8 lipid metabolism.'exotics' (steroids, squalene etc).squalene synthase

11.9 lipid metabolism.lipid degradation

11.9.1 lipid metabolism.lipid degradation.palmitoyl[protein] hydrolase

11.9.2 lipid metabolism.lipid degradation.lipases

11.9.2.1 lipid metabolism.lipid degradation.lipases.triacylglycerol lipase

11.9.2.2 lipid metabolism.lipid degradation.lipases.acylglycerol lipase

11.9.3 lipid metabolism.lipid degradation.lysophospholipases

11.9.3.1 lipid metabolism.lipid degradation.lysophospholipases.phospholipase D

11.9.3.2 lipid metabolism.lipid degradation.lysophospholipases.carboxylesterase

11.9.3.3 lipid metabolism.lipid degradation.lysophospholipases.glycerophosphodiester phosphodiesterase

11.9.3.4 lipid metabolism.lipid degradation.lysophospholipases.phospholipase A2

11.9.3.5 lipid metabolism.lipid degradation.lysophospholipases.phosphoinositide phospholipase C

11.9.4 lipid metabolism.lipid degradation.beta-oxidation

11.9.4.13 lipid metabolism.lipid degradation.beta-oxidation.acyl CoA reductase

11.9.4.14 lipid metabolism.lipid degradation.beta-oxidation.enoyl isomerase

11.9.4.2 lipid metabolism.lipid degradation.beta-oxidation.acyl CoA DH

11.9.4.3 lipid metabolism.lipid degradation.beta-oxidation.enoyl CoA hydratase

11.9.4.4 lipid metabolism.lipid degradation.beta-oxidation.hydroxybutyryl CoA DH

11.9.4.5 lipid metabolism.lipid degradation.beta-oxidation.acyl-CoA thioesterase

11.9.4.9 lipid metabolism.lipid degradation.beta-oxidation.multifunctional

11.9.4.99 lipid metabolism.lipid degradation.beta-oxidation.misc

12 N-metabolism

12.1 N-metabolism.nitrate metabolism

12.1.1 N-metabolism.nitrate metabolism.NR

12.1.2 N-metabolism.nitrate metabolism.nitrite reductase

12.2 N-metabolism.ammonia metabolism

12.2.1 N-metabolism.ammonia metabolism.glutamate synthase

12.2.1.1 N-metabolism.ammonia metabolism.glutamate synthase.ferredoxin dependent

12.2.1.2 N-metabolism.ammonia metabolism.glutamate synthase.NADH dependent

12.2.1001 N-metabolism.ammonia metabolism

12.2.1002 N-metabolism.ammonia metabolism

12.2.1003 N-metabolism.ammonia metabolism

12.2.2 N-metabolism.ammonia metabolism.glutamine synthetase

12.2.99 N-metabolism.ammonia metabolism.unspecified

12.3 N-metabolism.N-degradation

12.3.1 N-metabolism.N-degradation.glutamate dehydrogenase

12.4 N-metabolism.misc

13 amino acid metabolism

13.1 amino acid metabolism.synthesis

13.1.1 amino acid metabolism.synthesis.central amino acid metabolism

13.1.1.1 amino acid metabolism.synthesis.central amino acid metabolism.GABA

13.1.1.1.1 amino acid metabolism.synthesis.central amino acid metabolism.GABA.Glutamate decarboxylase

13.1.1.1.2 amino acid metabolism.synthesis.central amino acid metabolism.GABA.GABA transaminase

13.1.1.1.3 amino acid metabolism.synthesis.central amino acid metabolism.GABA.SSADH

13.1.1.1.4 amino acid metabolism.synthesis.central amino acid metabolism.GABA.gamma-hydroxybutyrate DH

13.1.1.2 amino acid metabolism.synthesis.central amino acid metabolism.aspartate

13.1.1.2.1 amino acid metabolism.synthesis.central amino acid metabolism.aspartate.aspartate aminotransferase

13.1.1.2.1001 amino acid metabolism.synthesis.central amino acid metabolism.aspartate

13.1.1.3 amino acid metabolism.synthesis.central amino acid metabolism.alanine

13.1.1.3.1 amino acid metabolism.synthesis.central amino acid metabolism.alanine.alanine aminotransferase

13.1.1.3.10001 amino acid synthesis.alanine

13.1.1.3.11 amino acid metabolism.synthesis.central amino acid metabolism.alanine.alanine-glyoxylate aminotransferase

13.1.2 amino acid metabolism.synthesis.glutamate family

13.1.2.1 amino acid metabolism.synthesis.glutamate family.glutamine

13.1.2.2 amino acid metabolism.synthesis.glutamate family.proline

13.1.2.2.1 amino acid metabolism.synthesis.glutamate family.proline.delta 1-pyrroline-5-carboxylate synthetase

13.1.2.2.10 amino acid metabolism.synthesis.glutamate family.proline.ornithine aminotransferase

13.1.2.2.1001 amino acid metabolism.synthesis.glutamate family.proline

13.1.2.2.2 amino acid metabolism.synthesis.glutamate family.proline.pyrroline-5-carboxylate reductase

13.1.2.2.3 amino acid metabolism.synthesis.glutamate family.proline.pyrroline-2-carboxylate reductase

13.1.2.2.4 amino acid metabolism.synthesis.glutamate family.proline.ornithin-cyclodeaminase

13.1.2.3 amino acid metabolism.synthesis.glutamate family.arginine

13.1.2.3.1 amino acid metabolism.synthesis.glutamate family.arginine.N-acetyl-L-glutamate 5-phosphotransferase

13.1.2.3.1001 amino acid metabolism.synthesis.glutamate family.arginine

13.1.2.3.1002 amino acid metabolism.synthesis.glutamate family.arginine

13.1.2.3.1003 amino acid metabolism.synthesis.glutamate family.arginine

13.1.2.3.1004 amino acid metabolism.synthesis.glutamate family.arginine

13.1.2.3.11 amino acid metabolism.synthesis.glutamate family.arginine.carbamoyl-phosphate synthase

13.1.2.3.2 amino acid metabolism.synthesis.glutamate family.arginine.acetylglutamate kinase

13.1.2.3.21 amino acid metabolism.synthesis.glutamate family.arginine.ornithine carbamoyltransferase

13.1.2.3.22 amino acid metabolism.synthesis.glutamate family.arginine.arginosuccinate synthase

13.1.2.3.23 amino acid metabolism.synthesis.glutamate family.arginine.argininosuccinate lyase

13.1.2.3.3 amino acid metabolism.synthesis.glutamate family.arginine.N-Acetylglutamate-5-P reductase

13.1.2.3.31 amino acid metabolism.synthesis.glutamate family.arginine.aspartate racemase

13.1.2.3.32 amino acid metabolism.synthesis.glutamate family.arginine.aspartate 4-decarboxylase

13.1.2.3.4 amino acid metabolism.synthesis.glutamate family.arginine.acetylornithine aminotransferase

13.1.2.3.5 amino acid metabolism.synthesis.glutamate family.arginine.N2-Acetylornithine deacetylase

13.1.3 amino acid metabolism.synthesis.aspartate family

13.1.3.1 amino acid metabolism.synthesis.aspartate family.asparagine

13.1.3.1.1 amino acid metabolism.synthesis.aspartate family.asparagine.asparagine synthetase

13.1.3.1.1001 amino acid metabolism.synthesis.aspartate family.asparagine

13.1.3.2 amino acid metabolism.synthesis.aspartate family.threonine

13.1.3.2.1 amino acid metabolism.synthesis.aspartate family.threonine.threonine synthase

13.1.3.2.1001 amino acid metabolism.synthesis.aspartate family.threonine

13.1.3.4 amino acid metabolism.synthesis.aspartate family.methionine

13.1.3.4.1 amino acid metabolism.synthesis.aspartate family.methionine.cystathionine gamma-synthase

13.1.3.4.1001 amino acid metabolism.synthesis.aspartate family.methionine

13.1.3.4.1002 amino acid metabolism.synthesis.aspartate family.methionine

13.1.3.4.1003 amino acid metabolism.synthesis.aspartate family.methionine

13.1.3.4.11 amino acid metabolism.synthesis.aspartate family.methionine.S-adenosylmethionine synthetase

13.1.3.4.12 amino acid metabolism.synthesis.aspartate family.methionine.homocysteine S-methyltransferase

13.1.3.4.13 amino acid metabolism.synthesis.aspartate family.methionine.methionine S-methyltransferase

13.1.3.4.2 amino acid metabolism.synthesis.aspartate family.methionine.cystathionine beta-lyase

13.1.3.4.3 amino acid metabolism.synthesis.aspartate family.methionine.methionine synthase

13.1.3.5 amino acid metabolism.synthesis.aspartate family.lysine

13.1.3.5.1 amino acid metabolism.synthesis.aspartate family.lysine.dihydrodipicolinate synthase

13.1.3.5.10 amino acid metabolism.synthesis.aspartate family.lysine.diaminopimelate dehydrogenase

13.1.3.5.1001 amino acid metabolism.synthesis.aspartate family.lysine

13.1.3.5.2 amino acid metabolism.synthesis.aspartate family.lysine.dihydrodipicolinate reductase

13.1.3.5.3 amino acid metabolism.synthesis.aspartate family.lysine.LL-diaminopimelic acid aminotransferase

13.1.3.5.4 amino acid metabolism.synthesis.aspartate family.lysine.diaminopimelate epimerase

13.1.3.5.5 amino acid metabolism.synthesis.aspartate family.lysine.diaminopimelate decarboxylase

13.1.3.5.6 amino acid metabolism.synthesis.aspartate family.lysine.2,3,4,5-tetrahydropyridine-2,6-dicarboxylate N-succinyltransferase

13.1.3.5.7 amino acid metabolism.synthesis.aspartate family.lysine.tetrahydrodipicolinate N-acetyltransferase

13.1.3.5.8 amino acid metabolism.synthesis.aspartate family.lysine.N-acyl-l-2-amino-6-oxopimelate aminotransferase

13.1.3.5.9 amino acid metabolism.synthesis.aspartate family.lysine.N-acyl-ll-2,6-diaminopimelate deacylase

13.1.3.6 amino acid metabolism.synthesis.aspartate family.misc

13.1.3.6.1 amino acid metabolism.synthesis.aspartate family.misc.homoserine

13.1.3.6.1.1 amino acid metabolism.synthesis.aspartate family.misc.homoserine.aspartate kinase

13.1.3.6.1.10 amino acid metabolism.synthesis.aspartate family.misc.homoserine.bifunctional aspartate kinase/homoserine dehydrogenase

13.1.3.6.1.1001 amino acid metabolism.synthesis.aspartate family.misc.homoserine

13.1.3.6.1.2 amino acid metabolism.synthesis.aspartate family.misc.homoserine.aspartate semialdehyde dehydrogenase

13.1.3.6.1.3 amino acid metabolism.synthesis.aspartate family.misc.homoserine.homoserine dehydrogenase

13.1.3.6.1.4 amino acid metabolism.synthesis.aspartate family.misc.homoserine.homoserine kinase

13.1.3.6.2 amino acid metabolism.synthesis.aspartate family.misc.homocysteine

13.1.3.6.2.1001 amino acid metabolism.synthesis.aspartate family.misc.homocysteine

13.1.4 amino acid metabolism.synthesis.branched chain group

13.1.4.1 amino acid metabolism.synthesis.branched chain group.common

13.1.4.1.1 amino acid metabolism.synthesis.branched chain group.common.acetolactate synthase

13.1.4.1.2 amino acid metabolism.synthesis.branched chain group.common.ketol-acid reductoisomerase

13.1.4.1.4 amino acid metabolism.synthesis.branched chain group.common.branched-chain amino acid aminotransferase

13.1.4.3 amino acid metabolism.synthesis.branched chain group.valine specific

13.1.4.3.1001 amino acid metabolism.synthesis.branched chain group.valine specific

13.1.4.4 amino acid metabolism.synthesis.branched chain group.leucine specific

13.1.4.4.1 amino acid metabolism.synthesis.branched chain group.leucine specific.2-isopropylmalate synthase

13.1.4.4.1001 amino acid metabolism.synthesis.branched chain group.leucine specific

13.1.4.4.2 amino acid metabolism.synthesis.branched chain group.leucine specific.isopropylmalate isomerase

13.1.4.4.3 amino acid metabolism.synthesis.branched chain group.leucine specific.3-isopropylmalate dehydrogenase

13.1.4.4.4 amino acid metabolism.synthesis.branched chain group.leucine specific.aminotransferase

13.1.4.5 amino acid metabolism.synthesis.branched chain group.isoleucine specific

13.1.4.5.1 amino acid metabolism.synthesis.branched chain group.isoleucine specific.threonine ammonia-lyase

13.1.4.5.1001 amino acid metabolism.synthesis.branched chain group.isoleucine specific

13.1.5 amino acid metabolism.synthesis.serine-glycine-cysteine group

13.1.5.1 amino acid metabolism.synthesis.serine-glycine-cysteine group.serine

13.1.5.1.1 amino acid metabolism.synthesis.serine-glycine-cysteine group.serine.phosphoglycerate dehydrogenase

13.1.5.1.1001 amino acid metabolism.synthesis.serine-glycine-cysteine group.serine

13.1.5.1.2 amino acid metabolism.synthesis.serine-glycine-cysteine group.serine.phosphoserine aminotransferase

13.1.5.1.3 amino acid metabolism.synthesis.serine-glycine-cysteine group.serine.phosphoserine phosphatase

13.1.5.2 amino acid metabolism.synthesis.serine-glycine-cysteine group.glycine

13.1.5.2.1 amino acid metabolism.synthesis.serine-glycine-cysteine group.glycine.glycine hydroxymethyltransferase

13.1.5.2.1001 amino acid metabolism.synthesis.serine-glycine-cysteine group.glycine

13.1.5.2.11 amino acid metabolism.synthesis.serine-glycine-cysteine group.glycine.threonine aldolase

13.1.5.2.2 amino acid metabolism.synthesis.serine-glycine-cysteine group.glycine.glycine transaminase

13.1.5.2.3 amino acid metabolism.synthesis.serine-glycine-cysteine group.glycine.serine glyoxylate aminotransferase

13.1.5.2.31 amino acid metabolism.synthesis.serine-glycine-cysteine group.glycine.2-amino-3-ketobutyrate coenzym A ligase

13.1.5.2.41 amino acid metabolism.synthesis.serine-glycine-cysteine group.glycine.sarcosine oxidase

13.1.5.3 amino acid metabolism.synthesis.serine-glycine-cysteine group.cysteine

13.1.5.3.1 amino acid metabolism.synthesis.serine-glycine-cysteine group.cysteine.OASTL

13.1.5.3.1001 amino acid metabolism.synthesis.serine-glycine-cysteine group.cysteine

13.1.5.3.1002 amino acid metabolism.synthesis.serine-glycine-cysteine group.cysteine

13.1.5.3.1003 amino acid metabolism.synthesis.serine-glycine-cysteine group.cysteine

13.1.5.3.1004 amino acid metabolism.synthesis.serine-glycine-cysteine group.cysteine

13.1.5.3.2 amino acid metabolism.synthesis.serine-glycine-cysteine group.cysteine.SAT

13.1.6 amino acid metabolism.synthesis.aromatic aa

13.1.6.1 amino acid metabolism.synthesis.aromatic aa.chorismate

13.1.6.1.1 amino acid metabolism.synthesis.aromatic aa.chorismate.3-deoxy-D-arabino-heptulosonate 7-phosphate synthase

13.1.6.1.10 amino acid metabolism.synthesis.aromatic aa.chorismate.dehydroquinate/shikimate dehydrogenase

13.1.6.1.1001 amino acid metabolism.synthesis.serine-glycine-cysteine group.cysteine

13.1.6.1.1002 amino acid metabolism.synthesis.aromatic aa.chorismate

13.1.6.1.2 amino acid metabolism.synthesis.aromatic aa.chorismate.3-dehydroquinate synthase

13.1.6.1.3 amino acid metabolism.synthesis.aromatic aa.chorismate.3-dehydroquinate dehydratase

13.1.6.1.4 amino acid metabolism.synthesis.aromatic aa.chorismate.shikimate dehydrogenase

13.1.6.1.5 amino acid metabolism.synthesis.aromatic aa.chorismate.shikimate kinase

13.1.6.1.6 amino acid metabolism.synthesis.aromatic aa.chorismate.5-enolpyruvylshikimate-3-phosphate synthase

13.1.6.1.7 amino acid metabolism.synthesis.aromatic aa.chorismate.chorismate synthase

13.1.6.2 amino acid metabolism.synthesis.aromatic aa.phenylalanine and tyrosine

13.1.6.2.1 amino acid metabolism.synthesis.aromatic aa.phenylalanine and tyrosine.chorismate mutase

13.1.6.2.2 amino acid metabolism.synthesis.aromatic aa.phenylalanine and tyrosine.glutamate—prephenate aminotransferase

13.1.6.3 amino acid metabolism.synthesis.aromatic aa.phenylalanine

13.1.6.3.1 amino acid metabolism.synthesis.aromatic aa.phenylalanine.arogenate dehydratase / prephenate dehydratase

13.1.6.3.1001 amino acid metabolism.synthesis.aromatic aa.phenylalanine

13.1.6.3.1002 amino acid metabolism.synthesis.aromatic aa.phenylalanine

13.1.6.3.1003 amino acid metabolism.synthesis.aromatic aa.phenylalanine

13.1.6.4 amino acid metabolism.synthesis.aromatic aa.tyrosine

13.1.6.4.1 amino acid metabolism.synthesis.aromatic aa.tyrosine.arogenate dehydrogenase \& prephenate dehydrogenase

13.1.6.4.1001 amino acid metabolism.synthesis.aromatic aa.tyrosine

13.1.6.4.2 amino acid metabolism.synthesis.aromatic aa.tyrosine.prephenate dehydrogenase

13.1.6.4.3 amino acid metabolism.synthesis.aromatic aa.tyrosine.tyrosine transaminase

13.1.6.5 amino acid metabolism.synthesis.aromatic aa.tryptophan

13.1.6.5.1 amino acid metabolism.synthesis.aromatic aa.tryptophan.anthranilate synthase

13.1.6.5.1001 amino acid metabolism.synthesis.aromatic aa.tryptophan

13.1.6.5.2 amino acid metabolism.synthesis.aromatic aa.tryptophan.anthranilate phosphoribosyltransferase

13.1.6.5.3 amino acid metabolism.synthesis.aromatic aa.tryptophan.phosphoribosyanthranilate isomerase

13.1.6.5.4 amino acid metabolism.synthesis.aromatic aa.tryptophan.indole-3-glycerol phosphate synthase

13.1.6.5.5 amino acid metabolism.synthesis.aromatic aa.tryptophan.tryptophan synthase

13.1.6.99 amino acid metabolism.synthesis.aromatic aa.misc

13.1.7 amino acid metabolism.synthesis.histidine

13.1.7.1 amino acid metabolism.synthesis.histidine.ATP phosphoribosyl transferase

13.1.7.10 amino acid metabolism.synthesis.histidine.imidazole glycerol phosphate synthase

13.1.7.1001 amino acid metabolism.synthesis.histidine

13.1.7.1002 amino acid metabolism.synthesis.histidine

13.1.7.11 amino acid metabolism.synthesis.histidine.ribose-phosphate diphosphokinase

13.1.7.2 amino acid metabolism.synthesis.histidine.bifunctional phosphoribosyl-ATP diphosphatase and phosphoribosyl-AMP cyclohydrolase

13.1.7.3 amino acid metabolism.synthesis.histidine.phosphoribosyl-AMP cyclohydrolase

13.1.7.4 amino acid metabolism.synthesis.histidine.N'-5'-phosphoribosyl-formimino-5-aminoimidazole-4-carboxamide ribonucleotide isomerase

13.1.7.5 amino acid metabolism.synthesis.histidine.Imidazole glycerol phosphate synthase

13.1.7.6 amino acid metabolism.synthesis.histidine.imidazoleglycerol-phosphate dehydratase

13.1.7.7 amino acid metabolism.synthesis.histidine.histidinol-phosphate aminotransferase

13.1.7.8 amino acid metabolism.synthesis.histidine.histidinol-phosphate synthase

13.1.7.9 amino acid metabolism.synthesis.histidine.histidinol dehydrogenase

13.1001 amino acid metabolism.methionine S-oxide

13.2 amino acid metabolism.degradation

13.2.1 amino acid metabolism.degradation.central amino acid metabolism

13.2.1.1 amino acid metabolism.degradation.central amino acid metabolism.GABA

13.2.1.2 amino acid metabolism.degradation.central amino acid metabolism.aspartate

13.2.2 amino acid metabolism.degradation.glutamate family

13.2.2.1 amino acid metabolism.degradation.glutamate family.glutamine

13.2.2.2 amino acid metabolism.degradation.glutamate family.proline

13.2.2.3 amino acid metabolism.degradation.glutamate family.arginine

13.2.2.3.1 amino acid metabolism.degradation.glutamate family.arginine.arginase

13.2.2.3.1001 amino acid metabolism.degradation.glutamate family.arginine

13.2.2.3.2 amino acid metabolism.degradation.glutamate family.arginine.agmatinase

13.2.2.3.3 amino acid metabolism.degradation.glutamate family.arginine.urease

13.2.3 amino acid metabolism.degradation.aspartate family

13.2.3.1 amino acid metabolism.degradation.aspartate family.asparagine

13.2.3.1.1 amino acid metabolism.degradation.aspartate family.asparagine.L-asparaginase

13.2.3.2 amino acid metabolism.degradation.aspartate family.threonine

13.2.3.2.1001 amino acid metabolism.degradation.aspartate family.threonine

13.2.3.4 amino acid metabolism.degradation.aspartate family.methionine

13.2.3.4.1 amino acid metabolism.degradation.aspartate family.methionine.methionine gamma-lyase

13.2.3.5 amino acid metabolism.degradation.aspartate family.lysine

13.2.3.5.1 mino acid metabolism.degradation.aspartate family.lysine.lysine decarboxylase

13.2.3.5.1001 amino acid metabolism.degradation.aspartate family.lysine

13.2.3.5.1002 amino acid metabolism.degradation.aspartate family.lysine

13.2.4 amino acid metabolism.degradation.branched chain group

13.2.4.1 amino acid metabolism.degradation.branched chain group.shared

13.2.4.1.7 amino acid metabolism.degradation.branched chain group.shared.methylglutaconyl-CoA hydratase

13.2.4.2 amino acid metabolism.degradation.branched chain group.val ile

13.2.4.3 amino acid metabolism.degradation.branched chain group.valine

13.2.4.4 amino acid metabolism.degradation.branched chain group.leucine

13.2.4.4.6 amino acid metabolism.degradation.branched chain group.leucine.methylcrotonoyl-CoA carboxylase

13.2.4.4.8 amino acid metabolism.degradation.branched chain group.leucine.hydroxymethylglutaryl-CoA lyase

13.2.4.5 amino acid metabolism.degradation.branched chain group.isoleucine

13.2.4.99 amino acid metabolism.degradation.branched chain group.unspecified

13.2.5 amino acid metabolism.degradation.serine-glycine-cysteine group

13.2.5.1 amino acid metabolism.degradation.serine-glycine-cysteine group.serine

13.2.5.1.1001 amino acid metabolism.degradation.serine-glycine-cysteine group.serine

13.2.5.2 amino acid metabolism.degradation.serine-glycine-cysteine group.glycine

13.2.5.2.11 amino acid metabolism.degradation.serine-glycine-cysteine group.glycine.threonine aldolase

13.2.5.3 amino acid metabolism.degradation.serine-glycine-cysteine group.cysteine

13.2.5.4 amino acid metabolism.degradation.serine-glycine-cysteine group.misc

13.2.6 amino acid metabolism.degradation.aromatic aa

13.2.6.1 amino acid metabolism.degradation.aromatic aa.phenylalanine

13.2.6.2 amino acid metabolism.degradation.aromatic aa.tyrosine

13.2.6.2.6 amino acid metabolism.degradation.aromatic aa.tyrosine.fumarylacetoacetase

13.2.6.3 amino acid metabolism.degradation.aromatic aa.tryptophan

13.2.6.4 amino acid metabolism.degradation.aromatic aa.chorismate

13.2.6.5 amino acid metabolism.degradation.aromatic aa.misc

13.2.7 amino acid metabolism.degradation.histidine

13.99 amino acid metabolism.misc

13.99.1 amino acid metabolism.misc.central amino acid metabolism

13.99.2 amino acid metabolism.misc.glutamate family

13.99.2.4 amino acid metabolism.misc.glutamate family.hydroxyproline

14 S-assimilation

14.1 S-assimilation.ATPS

14.10 S-assimilation.S-scavenging

14.1001 Mineral Nutrition

14.15 S-assimilation.AKN

14.2 S-assimilation.APR

14.3 S-assimilation.sulfite redox

14.5 S-assimilation.sulfite oxidase

15 metal handling

15.1 metal handling.acquisition

15.2 metal handling.binding, chelation and storage

15.3 metal handling.regulation

16 secondary metabolism

16.1 secondary metabolism.isoprenoids

16.1.1 secondary metabolism.isoprenoids.non-mevalonate pathway

16.1.1.1 secondary metabolism.isoprenoids.non-mevalonate pathway.DXS

16.1.1.10 secondary metabolism.isoprenoids.non-mevalonate pathway.geranylgeranyl pyrophosphate synthase

16.1.1.1010 secondary metabolism.isoprenoids.mevalonate pathway

16.1.1.1011 secondary metabolism.isoprenoids.non-mevalonate pathway

16.1.1.1012 secondary metabolism.isoprenoids.non-mevalonate pathway

16.1.1.1013 secondary metabolism.isoprenoids.non-mevalonate pathway

16.1.1.1014 secondary metabolism.isoprenoids.non-mevalonate pathway

16.1.1.2 secondary metabolism.isoprenoids.non-mevalonate pathway.DXR

16.1.1.3 secondary metabolism.isoprenoids.non-mevalonate pathway.CMS

16.1.1.4 secondary metabolism.isoprenoids.non-mevalonate pathway.CMK

16.1.1.5 secondary metabolism.isoprenoids.non-mevalonate pathway.MCS

16.1.1.6 secondary metabolism.isoprenoids.non-mevalonate pathway.HDS

16.1.1.7 secondary metabolism.isoprenoids.non-mevalonate pathway.HDR

16.1.2 secondary metabolism.isoprenoids.mevalonate pathway

16.1.2.1 secondary metabolism.isoprenoids.mevalonate pathway.acetyl-CoA C-acyltransferase

16.1.2.1001 secondary metabolism.isoprenoids.mevalonate pathway

16.1.2.1002 secondary metabolism.isoprenoids.mevalonate pathway

16.1.2.1003 secondary metabolism.isoprenoids.mevalonate pathway

16.1.2.1004 secondary metabolism.isoprenoids.mevalonate pathway

16.1.2.1005 secondary metabolism.isoprenoids.mevalonate pathway

16.1.2.1006 secondary metabolism.isoprenoids.mevalonate pathway

16.1.2.1007 secondary metabolism.isoprenoids.mevalonate pathway

16.1.2.1008 secondary metabolism.isoprenoids.mevalonate pathway

16.1.2.1009 secondary metabolism.isoprenoids.mevalonate pathway

16.1.2.1010 secondary metabolism.isoprenoids.mevalonate pathway

16.1.2.1011 secondary metabolism.isoprenoids.mevalonate pathway

16.1.2.1012 secondary metabolism.isoprenoids.mevalonate pathway

16.1.2.1013 secondary metabolism.isoprenoids.mevalonate pathway

16.1.2.1014 secondary metabolism.isoprenoids.mevalonate pathway

16.1.2.2 secondary metabolism.isoprenoids.mevalonate pathway.HMG-CoA synthase

16.1.2.3 secondary metabolism.isoprenoids.mevalonate pathway.HMG-CoA reductase

16.1.2.4 secondary metabolism.isoprenoids.mevalonate pathway.mevalonate kinase

16.1.2.5 secondary metabolism.isoprenoids.mevalonate pathway.phosphomevalonate kinase

16.1.2.6 secondary metabolism.isoprenoids.mevalonate pathway.mevalonate diphosphate decarboxylase

16.1.2.7 secondary metabolism.isoprenoids.mevalonate pathway.isopentenyl pyrophosphate:dimethyllallyl pyrophosphate isomerase

16.1.2.8 secondary metabolism.isoprenoids.mevalonate pathway.geranyl diphosphate synthase

16.1.2.9 secondary metabolism.isoprenoids.mevalonate pathway.farnesyl pyrophosphate synthetase

16.1.3 secondary metabolism.isoprenoids.tocopherol biosynthesis

16.1.3.1 secondary metabolism.isoprenoids.tocopherol biosynthesis.hydroxyphenylpyruvate dioxygenase

16.1.3.1001 secondary metabolism.isoprenoids.tocopherol biosynthesis

16.1.3.1002 secondary metabolism.isoprenoids.tocopherol biosynthesis

16.1.3.1003 secondary metabolism.isoprenoids.tocopherol biosynthesis

16.1.3.1004 secondary metabolism.isoprenoids.tocopherol biosynthesis

16.1.3.1005 secondary metabolism.isoprenoids.tocopherol biosynthesis

16.1.3.2 secondary metabolism.isoprenoids.tocopherol biosynthesis.homogentisate phytyltransferase

16.1.3.3 secondary metabolism.isoprenoids.tocopherol biosynthesis.MPBQ/MSBQ methyltransferase

16.1.3.4 secondary metabolism.isoprenoids.tocopherol biosynthesis.tocopherol cyclase

16.1.3.5 secondary metabolism.isoprenoids.tocopherol biosynthesis.tocopherol methyltransferase

16.1.4 secondary metabolism.isoprenoids.carotenoids

16.1.4.1 secondary metabolism.isoprenoids.carotenoids.phytoene synthase

16.1.4.10 secondary metabolism.isoprenoids.carotenoids.carotenoid cleavage dioxygenase

16.1.4.1001 secondary metabolism.isoprenoids.carotenoids.phytoene

16.1.4.1002 secondary metabolism.isoprenoids.carotenoids.zeta carotene

16.1.4.1003 secondary metabolism.isoprenoids.carotenoids.lycopene

16.1.4.1004 secondary metabolism.isoprenoids.carotenoids.alpha carotene

16.1.4.1005 secondary metabolism.isoprenoids.carotenoids.beta carotene

16.1.4.1006 secondary metabolism.isoprenoids.carotenoids.delta carotene

16.1.4.2 secondary metabolism.isoprenoids.carotenoids.phytoene dehydrogenase

16.1.4.21 secondary metabolism.isoprenoids.carotenoids.violaxanthin de-epoxidase

16.1.4.3 secondary metabolism.isoprenoids.carotenoids.zeta-carotene desaturase

16.1.4.4 secondary metabolism.isoprenoids.carotenoids.lycopene epsilon cyclase

16.1.4.5 secondary metabolism.isoprenoids.carotenoids.lycopene beta cyclase

16.1.4.6 secondary metabolism.isoprenoids.carotenoids.carotenoid beta ring hydroxylase

16.1.4.7 secondary metabolism.isoprenoids.carotenoids.carotenoid epsilon ring hydroxylase

16.1.4.8 secondary metabolism.isoprenoids.carotenoids.carotenoid isomerase

16.1.5 secondary metabolism.isoprenoids.terpenoids

16.1.5.1001 secondary metabolism.isoprenoids.terpenoids

16.1.5.1002 secondary metabolism.isoprenoids.terpenoids

16.1.5.1003 secondary metabolism.isoprenoids.terpenoids

16.10 secondary metabolism.simple phenols

16.2 secondary metabolism.phenylpropanoids

16.2.1 secondary metabolism.phenylpropanoids.lignin biosynthesis

16.2.1.1 secondary metabolism.phenylpropanoids.lignin biosynthesis.PAL

16.2.1.10 secondary metabolism.phenylpropanoids.lignin biosynthesis.CAD

16.2.1.1001 secondary metabolism.phenylpropanoids.lignin biosynthesis

16.2.1.1002 secondary metabolism.phenylpropanoids.lignin biosynthesis

16.2.1.1003 secondary metabolism.phenylpropanoids.lignin biosynthesis

16.2.1.1004 secondary metabolism.phenylpropanoids.lignin biosynthesis

16.2.1.1007 secondary metabolism.phenylpropanoids.lignin biosynthesis

16.2.1.1008 secondary metabolism.phenylpropanoids.lignin biosynthesis

16.2.1.1010 secondary metabolism.phenylpropanoids.lignin biosynthesis

16.2.1.1011 secondary metabolism.phenylpropanoids.lignin biosynthesis

16.2.1.2 secondary metabolism.phenylpropanoids.lignin biosynthesis.C4H

16.2.1.3 secondary metabolism.phenylpropanoids.lignin biosynthesis.4CL

16.2.1.4 secondary metabolism.phenylpropanoids.lignin biosynthesis.HCT

16.2.1.5 secondary metabolism.phenylpropanoids.lignin biosynthesis.C3H

16.2.1.6 secondary metabolism.phenylpropanoids.lignin biosynthesis.CCoAOMT

16.2.1.7 secondary metabolism.phenylpropanoids.lignin biosynthesis.CCR1

16.2.1.8 secondary metabolism.phenylpropanoids.lignin biosynthesis.F5H

16.2.1.9 secondary metabolism.phenylpropanoids.lignin biosynthesis.COMT

16.2.99 secondary metabolism.phenylpropanoids.unspecified

16.2.99.1001 secondary metabolism.phenylpropanoids.unspecified

16.2.99.1002 secondary metabolism.phenylpropanoids.unspecified

16.2.99.1003 secondary metabolism.phenylpropanoids.unspecified

16.2.99.1005 secondary metabolism.phenylpropanoids.unspecified

16.2.99.1006 secondary metabolism.phenylpropanoids.unspecified

16.2.99.1007 secondary metabolism.phenylpropanoids.unspecified

16.2.99.1008 secondary metabolism.phenylpropanoids.unspecified

16.2.99.1009 secondary metabolism.phenylpropanoids.unspecified

16.2.99.1011 secondary metabolism.phenylpropanoids.unspecified

16.2.99.1012 secondary metabolism.phenylpropanoids.unspecified

16.2.99.1013 secondary metabolism.phenylpropanoids.unspecified

16.2.99.1014 secondary metabolism.phenylpropanoids.unspecified

16.2.99.1015 secondary metabolism.phenylpropanoids.unspecified

16.2.99.1016 secondary metabolism.phenylpropanoids.unspecified

16.2.99.1017 secondary metabolism.phenylpropanoids.unspecified

16.2.99.1018 secondary metabolism.phenylpropanoids.unspecified

16.2.99.1019 secondary metabolism.phenylpropanoids.unspecified

16.2.99.1020 secondary metabolism.phenylpropanoids.unspecified

16.2.99.1021 secondary metabolism.phenylpropanoids.unspecified

16.2.99.1022 secondary metabolism.phenylpropanoids.unspecified

16.2.99.1023 secondary metabolism.phenylpropanoids.unspecified

16.2.99.1024 secondary metabolism.phenylpropanoids.unspecified

16.2.99.1025 secondary metabolism.phenylpropanoids.unspecified

16.2.99.1026 secondary metabolism.phenylpropanoids.unspecified

16.2.99.1027 secondary metabolism.phenylpropanoids.unspecified

16.2.99.1028 secondary metabolism.phenylpropanoids.unspecified

16.2.99.1029 secondary metabolism.phenylpropanoids.unspecified

16.2.99.1030 secondary metabolism.phenylpropanoids.unspecified

16.2.99.1031 secondary metabolism.phenylpropanoids.unspecified

16.2.99.1032 secondary metabolism.phenylpropanoids.unspecified

16.2.99.1033 secondary metabolism.phenylpropanoids.unspecified

16.2.99.1034 secondary metabolism.phenylpropanoids.unspecified

16.2.99.1035 secondary metabolism.phenylpropanoids.unspecified

16.2.99.1036 secondary metabolism.phenylpropanoids.unspecified

16.2.99.1037 secondary metabolism.phenylpropanoids.unspecified

16.2.99.1038 secondary metabolism.phenylpropanoids.unspecified

16.2.99.1039 secondary metabolism.phenylpropanoids.unspecified

16.2.99.1040 secondary metabolism.phenylpropanoids.unspecified

16.2.99.1041 secondary metabolism.phenylpropanoids.unspecified

16.2.99.1042 secondary metabolism.phenylpropanoids.unspecified

16.2.99.1043 secondary metabolism.phenylpropanoids.unspecified

16.2.99.1044 secondary metabolism.phenylpropanoids.unspecified

16.2.99.1045 secondary metabolism.phenylpropanoids.unspecified

16.2.99.1046 secondary metabolism.phenylpropanoids.unspecified

16.2.99.1047 secondary metabolism.phenylpropanoids.unspecified

16.2.99.1048 secondary metabolism.phenylpropanoids.unspecified

16.2.99.1051 secondary metabolism.phenylpropanoids.unspecified

16.20 secondary metabolism.amino acid derivatives

16.20.1001 secondary metabolism.amino acid derivatives

16.20.1002 secondary metabolism.amino acid derivatives

16.20.1003 secondary metabolism.amino acid derivatives

16.20.1004 secondary metabolism.amino acid derivatives

16.20.1005 secondary metabolism.amino acid derivatives

16.20.1006 secondary metabolism.amino acid derivatives

16.3 secondary metabolism.shikimate pathway

16.4 secondary metabolism.N misc

16.4.1 secondary metabolism.N misc.alkaloid-like

16.4.1.1001 secondary metabolism.N misc.alkaloid-like

16.4.1.1002 secondary metabolism.N misc.alkaloid-like

16.4.1.1003 secondary metabolism.N misc.alkaloid-like

16.4.1.1004 secondary metabolism.N misc.alkaloid-like

16.4.1.1005 secondary metabolism.N misc.alkaloid-like

16.4.1.1006 secondary metabolism.N misc.alkaloid-like

16.4.1.1007 secondary metabolism.N misc.alkaloid-like

16.4.2 secondary metabolism.N misc.betaine

16.4.2.1 secondary metabolism.N misc.betaine.betaine-aldehyde dehydrogenase

16.4.3 secondary metabolism.N misc.cyanogenic glycosides

16.4.3.1 secondary metabolism.N misc.cyanogenic glycosides.cyanase

16.5 secondary metabolism.sulfur-containing

16.5.1 secondary metabolism.sulfur-containing.glucosinolates

16.5.1.1 secondary metabolism.sulfur-containing.glucosinolates.synthesis

16.5.1.1.1 secondary metabolism.sulfur-containing.glucosinolates.synthesis.aliphatic

16.5.1.1.1.1 secondary metabolism.sulfur-containing.glucosinolates.synthesis.aliphatic.branched-chain amino acid aminotransferase (BCAT/MAAT)

16.5.1.1.1.10 secondary metabolism.sulfur-containing.glucosinolates.synthesis.aliphatic.flavin-containing monooxygenase

16.5.1.1.1.11 secondary metabolism.sulfur-containing.glucosinolates.synthesis.aliphatic.glucosinolate 2-oxoglutarate-dependent dioxygenase (AOP)

16.5.1.1.1.12 secondary metabolism.sulfur-containing.glucosinolates.synthesis.aliphatic.benzoate-CoA ligase

16.5.1.1.1.13 secondary metabolism.sulfur-containing.glucosinolates.synthesis.aliphatic.2-oxoglutarate-dependent dioxygenase

16.5.1.1.1.2 secondary metabolism.sulfur-containing.glucosinolates.synthesis.aliphatic.methylthioalkylmalate synthase (MAM)

16.5.1.1.1.3 secondary metabolism.sulfur-containing.glucosinolates.synthesis.aliphatic.methylthioalkylmalate isomerase large subunit (MAM-IL)

16.5.1.1.1.4 secondary metabolism.sulfur-containing.glucosinolates.synthesis.aliphatic.methylthioalkylmalate isomerase small subunit (MAM-IS)

16.5.1.1.1.5 secondary metabolism.sulfur-containing.glucosinolates.synthesis.aliphatic.methylthioalkylmalate dehydrogenase (MAM-D)

16.5.1.1.1.6 secondary metabolism.sulfur-containing.glucosinolates.synthesis.aliphatic.CYP79F1/F2 monooxygenase

16.5.1.1.1.7 secondary metabolism.sulfur-containing.glucosinolates.synthesis.aliphatic.CYP83A1 phenylacetaldoxime monooxygenase

16.5.1.1.1.8 secondary metabolism.sulfur-containing.glucosinolates.synthesis.aliphatic.UDP-glycosyltransferase

16.5.1.1.1.9 secondary metabolism.sulfur-containing.glucosinolates.synthesis.aliphatic.sulfotransferase

16.5.1.1.2 secondary metabolism.sulfur-containing.glucosinolates.synthesis.aromatic

16.5.1.1.2.1 secondary metabolism.sulfur-containing.glucosinolates.synthesis.aromatic.CYP79A2 monooxygenase

16.5.1.1.3 secondary metabolism.sulfur-containing.glucosinolates.synthesis.indole

16.5.1.1.3.1 secondary metabolism.sulfur-containing.glucosinolates.synthesis.indole.CYP79B2 monooxygenase

16.5.1.1.3.2 secondary metabolism.sulfur-containing.glucosinolates.synthesis.indole.CYP79B3 monooxygenase

16.5.1.1.3.3 secondary metabolism.sulfur-containing.glucosinolates.synthesis.indole.indole-3-methyl-desulfoglucosinolate sulfotransferase

16.5.1.1.3.4 secondary metabolism.sulfur-containing.glucosinolates.synthesis.indole.cytochrome P450 monooxygenase

16.5.1.1.4 secondary metabolism.sulfur-containing.glucosinolates.synthesis.shared

16.5.1.1.4.1 secondary metabolism.sulfur-containing.glucosinolates.synthesis.shared.CYP83B1 phenylacetaldoxime monooxygenase

16.5.1.1.4.2 secondary metabolism.sulfur-containing.glucosinolates.synthesis.shared.alkylthiohydroximate C-S lyase

16.5.1.1.4.3 secondary metabolism.sulfur-containing.glucosinolates.synthesis.shared.UDP-glycosyltransferase

16.5.1.2 secondary metabolism.sulfur-containing.glucosinolates.regulation

16.5.1.2.1 secondary metabolism.sulfur-containing.glucosinolates.regulation.aliphatic

16.5.1.2.2 secondary metabolism.sulfur-containing.glucosinolates.regulation.aromatic

16.5.1.2.3 secondary metabolism.sulfur-containing.glucosinolates.regulation.indole

16.5.1.2.4 secondary metabolism.sulfur-containing.glucosinolates.regulation.shared

16.5.1.3 secondary metabolism.sulfur-containing.glucosinolates.degradation

16.5.1.3.1 secondary metabolism.sulfur-containing.glucosinolates.degradation.myrosinase

16.5.1.3.1.1 secondary metabolism.sulfur-containing.glucosinolates.degradation.myrosinase.TGG

16.5.1.3.2 secondary metabolism.sulfur-containing.glucosinolates.degradation.nitrilespecifier protein

16.5.1.3.2.1 secondary metabolism.sulfur-containing.glucosinolates.degradation.nitrilespecifier protein.epithiospecifier protein

16.5.1.3.3 secondary metabolism.sulfur-containing.glucosinolates.degradation.nitrilase

16.5.1.4 secondary metabolism.sulfur-containing.glucosinolates.transport

16.5.1.4.1 secondary metabolism.sulfur-containing.glucosinolates.transport.aliphatic

16.5.99 secondary metabolism.sulfur-containing.misc

16.5.99.1 secondary metabolism.sulfur-containing.misc.alliinase

16.7 secondary metabolism.wax

16.7.1001 secondary metabolism.wax

16.8 secondary metabolism.flavonoids

16.8.1 secondary metabolism.flavonoids.anthocyanins

16.8.1.1 secondary metabolism.flavonoids.anthocyanins.leucocyanidin dioxygenase

16.8.1.10 secondary metabolism.flavonoids.anthocyanins.anthocyanidin 3-O-methyltransferase

16.8.1.1001 secondary metabolism.flavonoids.anthocyanins

16.8.1.12 secondary metabolism.flavonoids.anthocyanins.anthocyanidin 3-O-glucosyltransferase

16.8.1.2 secondary metabolism.flavonoids.anthocyanins.anthocyanidin reductase

16.8.1.21 secondary metabolism.flavonoids.anthocyanins.anthocyanin 5-aromatic acyltransferase

16.8.2 secondary metabolism.flavonoids.chalcones

16.8.2.1 secondary metabolism.flavonoids.chalcones.naringenin-chalcone synthase

16.8.2.2 secondary metabolism.flavonoids.chalcones.chalcone isomerase

16.8.3 secondary metabolism.flavonoids.dihydroflavonols

16.8.3.1 secondary metabolism.flavonoids.dihydroflavonols.dihydroflavonol 4-reductase

16.8.3.2 secondary metabolism.flavonoids.dihydroflavonols.flavanone 3-hydroxylase

16.8.3.3 secondary metabolism.flavonoids.dihydroflavonols.flavonoid 3''-monooxygenase

16.8.3.4 secondary metabolism.flavonoids.dihydroflavonols.flavonoid 3',5'-hydroxylase

16.8.4 secondary metabolism.flavonoids.flavonols

16.8.4.1 secondary metabolism.flavonoids.flavonols.flavonol synthase (FLS)

16.8.4.2 secondary metabolism.flavonoids.flavonols.flavonol 3-O-glycosyltransferase

16.8.4.3 secondary metabolism.flavonoids.flavonols.flavonol-3-O-rhamnosyltransferase

16.8.5 secondary metabolism.flavonoids.isoflavones

16.8.5.1 secondary metabolism.flavonoids.isoflavones.isoflavone reductase

16.8.5.2 secondary metabolism.flavonoids.isoflavones.chalcone reductase

16.8.5.3 secondary metabolism.flavonoids.isoflavones.isoflavone synthase

16.8.5.4 secondary metabolism.flavonoids.isoflavones.hydroxyisoflavone dehydrogenase

16.8.6 secondary metabolism.flavonoids.flavonones

16.8.6.1 secondary metabolism.flavonoids.flavonones.flavone synthase (FNS)

16.8.6.2 secondary metabolism.flavonoids.flavonones.flavone 7-O-glycosyltransferase

16.8.6.3 secondary metabolism.flavonoids.flavonones.flavone apiosyltransferase

16.8.7 secondary metabolism.flavonoids.flavan 3-ol

16.8.7.1 secondary metabolism.flavonoids.flavan 3-ol.leucoanthocyanidin 4-reductase (LAR)

16.8.8 secondary metabolism.flavonoids.flavonoid glycosylations

16.8.8.1 secondary metabolism.flavonoids.flavonoid glycosylations.anthocyanidin-3-O-glycoside-6'-rhamnosyltransferase

16.8.8.2 secondary metabolism.flavonoids.flavonoid glycosylations.flavonol-3-O-glycoside-rhamnosyltransferase

16.99 secondary metabolism.unspecified

16.99.1001 secondary metabolism.unspecified

16.99.1003 secondary metabolism.unspecified

16.99.1004 secondary metabolism.unspecified

16.99.1005 secondary metabolism.unspecified

16.99.1006 secondary metabolism.unspecified

16.99.1007 secondary metabolism.unspecified

16.99.1008 secondary metabolism.unspecified

16.99.1009 secondary metabolism.unspecified

16.99.1010 secondary metabolism.unspecified

16.99.1011 secondary metabolism.unspecified

16.99.1012 secondary metabolism.unspecified

16.99.1013 secondary metabolism.unspecified

16.99.1014 secondary metabolism.unspecified

16.99.1016 secondary metabolism.unspecified

16.99.1017 secondary metabolism.unspecified

16.99.1018 secondary metabolism.unspecified

17 hormone metabolism

17.1 hormone metabolism.abscisic acid

17.1.1 hormone metabolism.abscisic acid.synthesis-degradation

17.1.1.1 hormone metabolism.abscisic acid.synthesis-degradation.synthesis

17.1.1.1.1 hormone metabolism.abscisic acid.synthesis-degradation.synthesis.zeaxanthin epoxidase

17.1.1.1.10 hormone metabolism.abscisic acid.synthesis-degradation.synthesis.9-cis-epoxycarotenoid dioxygenase

17.1.1.1.11 hormone metabolism.abscisic acid.synthesis-degradation.synthesis.short chain alcohol dehydrogenmase (ABA2)

17.1.1.1.12 hormone metabolism.abscisic acid.synthesis-degradation.synthesis.abscisic aldehyde oxidase

17.1.1.1001 hormone metabolism.abscisic acid.synthesis-degradation

17.1.1.1002 hormone metabolism.abscisic acid.synthesis-degradation

17.1.1.2 hormone metabolism.abscisic acid.synthesis-degradation.degradation

17.1.1.2.1 hormone metabolism.abscisic acid.synthesis-degradation.degradation.8-hydroxylase

17.1.2 hormone metabolism.abscisic acid.signal transduction

17.1.3 hormone metabolism.abscisic acid.induced-regulated-responsive-activated

17.2 hormone metabolism.auxin

17.2.1 hormone metabolism.auxin.synthesis-degradation

17.2.1.1001 hormone metabolism.auxin.synthesis-degradation

17.2.1.1002 hormone metabolism.auxin

17.2.1.1003 hormone metabolism.auxin

17.2.1.1004 hormone metabolism.auxin

17.2.1.1005 hormone metabolism.auxin

17.2.1.1006 hormone metabolism.auxin

17.2.1.1007 hormone metabolism.auxin

17.2.2 hormone metabolism.auxin.signal transduction

17.2.3 hormone metabolism.auxin.induced-regulated-responsive-activated

17.3 hormone metabolism.brassinosteroid

17.3.1 hormone metabolism.brassinosteroid.synthesis-degradation

17.3.1.1 hormone metabolism.brassinosteroid.synthesis-degradation.BRs

17.3.1.1.1 hormone metabolism.brassinosteroid.synthesis-degradation.BRs.DET2

17.3.1.1.2 hormone metabolism.brassinosteroid.synthesis-degradation.BRs.DWF4

17.3.1.1.3 hormone metabolism.brassinosteroid.synthesis-degradation.BRs.CPD

17.3.1.1.4 hormone metabolism.brassinosteroid.synthesis-degradation.BRs.BR6OX

17.3.1.1.5 hormone metabolism.brassinosteroid.synthesis-degradation.BRs.metabolic regulation

17.3.1.1.99 hormone metabolism.brassinosteroid.synthesis-degradation.BRs.other

17.3.1.1001 hormone metabolism.brassinosteroid.synthesis-degradation

17.3.1.1002 hormone metabolism.brassinosteroid.synthesis-degradation

17.3.1.1003 hormone metabolism.brassinosteroid.synthesis-degradation

17.3.1.1004 hormone metabolism.brassinosteroid.synthesis-degradation

17.3.1.1005 hormone metabolism.brassinosteroid.synthesis-degradation

17.3.1.1006 hormone metabolism.brassinosteroid.synthesis-degradation

17.3.1.2 hormone metabolism.brassinosteroid.synthesis-degradation.sterols

17.3.1.2.1 hormone metabolism.brassinosteroid.synthesis-degradation.sterols.SMT1

17.3.1.2.2 hormone metabolism.brassinosteroid.synthesis-degradation.sterols.SMT2

17.3.1.2.3 hormone metabolism.brassinosteroid.synthesis-degradation.sterols.CYP51

17.3.1.2.4 hormone metabolism.brassinosteroid.synthesis-degradation.sterols.FACKEL

17.3.1.2.5 hormone metabolism.brassinosteroid.synthesis-degradation.sterols.HYD1

17.3.1.2.6 hormone metabolism.brassinosteroid.synthesis-degradation.sterols.DWF7

17.3.1.2.7 hormone metabolism.brassinosteroid.synthesis-degradation.sterols.DWF5

17.3.1.2.8 hormone metabolism.brassinosteroid.synthesis-degradation.sterols.DWF1

17.3.1.2.99 hormone metabolism.brassinosteroid.synthesis-degradation.sterols.other

17.3.2 hormone metabolism.brassinosteroid.signal transduction

17.3.2.1 hormone metabolism.brassinosteroid.signal transduction.BRI

17.3.2.2 hormone metabolism.brassinosteroid.signal transduction.BZR

17.3.2.99 hormone metabolism.brassinosteroid.signal transduction.other

17.3.3 hormone metabolism.brassinosteroid.induced-regulated-responsive-activated

17.4 hormone metabolism.cytokinin

17.4.1 hormone metabolism.cytokinin.synthesis-degradation

17.4.1.1001 hormone metabolism.cytokinin.synthesis-degradation

17.4.1.1002 hormone metabolism.cytokinin.synthesis-degradation

17.4.1.1003 hormone metabolism.cytokinin.synthesis-degradation

17.4.2 hormone metabolism.cytokinin.signal transduction

17.4.3 hormone metabolism.cytokinin.induced-regulated-responsive-activated

17.5 hormone metabolism.ethylene

17.5.1 hormone metabolism.ethylene.synthesis-degradation

17.5.1.1 hormone metabolism.ethylene.synthesis-degradation.1-aminocyclopropane-1-carboxylate synthase

17.5.1.1001 hormone metabolism.ethylene.synthesis-degradation

17.5.1.1002 hormone metabolism.ethylene.synthesis-degradation

17.5.1.2 hormone metabolism.ethylene.synthesis-degradation.1-aminocyclopropane-1-carboxylate oxidase

17.5.2 hormone metabolism.ethylene.signal transduction

17.5.3 hormone metabolism.ethylene.induced-regulated-responsive-activated

17.6 hormone metabolism.gibberelin

17.6.1 hormone metabolism.gibberelin.synthesis-degradation

17.6.1.1 hormone metabolism.gibberelin.synthesis-degradation.copalyl diphosphate synthase

17.6.1.1001 hormone metabolism.gibberelin.synthesis-degradation

17.6.1.11 hormone metabolism.gibberelin.synthesis-degradation.GA20 oxidase

17.6.1.12 hormone metabolism.gibberelin.synthesis-degradation.GA3 oxidase

17.6.1.13 hormone metabolism.gibberelin.synthesis-degradation.GA2 oxidase

17.6.1.2 hormone metabolism.gibberelin.synthesis-degradation.ent-kaurene synthase

17.6.1.3 hormone metabolism.gibberelin.synthesis-degradation.ent-kaurene oxidase

17.6.1.4 hormone metabolism.gibberelin.synthesis-degradation.ent-kaurenoic acid hydroxylase/oxygenase

17.6.2 hormone metabolism.gibberelin.signal transduction

17.6.3 hormone metabolism.gibberelin.induced-regulated-responsive-activated

17.7 hormone metabolism.jasmonate

17.7.1 hormone metabolism.jasmonate.synthesis-degradation

17.7.1.1 hormone metabolism.jasmonate.synthesis-degradation.lipases

17.7.1.10 hormone metabolism.jasmonate.synthesis-degradation.jasmonate-O-methyltransferase

17.7.1.1001 hormone metabolism.jasmonate.synthesis-degradation

17.7.1.1002 hormone metabolism.jasmonate.synthesis-degradation

17.7.1.2 hormone metabolism.jasmonate.synthesis-degradation.lipoxygenase

17.7.1.3 hormone metabolism.jasmonate.synthesis-degradation.allene oxidase synthase

17.7.1.4 hormone metabolism.jasmonate.synthesis-degradation.allene oxidase cyclase

17.7.1.5 hormone metabolism.jasmonate.synthesis-degradation.12-Oxo-PDA-reductase

17.7.2 hormone metabolism.jasmonate.signal transduction

17.7.3 hormone metabolism.jasmonate.induced-regulated-responsive-activated

17.8 hormone metabolism.salicylic acid

17.8.1 hormone metabolism.salicylic acid.synthesis-degradation

17.8.1.1 hormone metabolism.salicylic acid.synthesis-degradation.synthesis

17.8.1.1.2 hormone metabolism.salicylic acid.synthesis-degradation.synthesis.isochorismate pyruvate lyase

17.8.1.1.3 hormone metabolism.salicylic acid.synthesis-degradation.synthesis.benzoic acid-2-hydroxylase

17.8.1.1.4 hormone metabolism.salicylic acid.synthesis-degradation.synthesis.SA glucosyltransferase ether bond making SAG

17.8.1.1.5 hormone metabolism.salicylic acid.synthesis-degradation.synthesis.SA glucosyltransferase ester and ether bond making SGE, SAG

17.8.1.1.6 hormone metabolism.salicylic acid.synthesis-degradation.synthesis.SA methyltransferase

17.8.1.1.7 hormone metabolism.salicylic acid.synthesis-degradation.synthesis.methyl-SA methylesterase

17.8.1001 hormone metabolism.salicylic acid.synthesis-degradation

17.8.1002 hormone metabolism.salicylic acid.synthesis-degradation.Methylsalicylate

17.8.2 hormone metabolism.salicylic acid.signal transduction

17.8.3 hormone metabolism.salicylic acid.induced-regulated-responsive-activated

18 Co-factor and vitamine metabolism

18.1 Co-factor and vitamine metabolism.molybdenum cofactor

18.1.1 Co-factor and vitamine metabolism.molybdenum cofactor.gephyrin

18.10 Co-factor and vitamine metabolism.coenzyme M

18.10.3 Co-factor and vitamine metabolism.coenzyme M.2-hydroxyacid dehydrogenase

18.1001 Co-factor and vitamine metabolism

18.1002 Co-factor and vitamine metabolism

18.1003 Co-factor and vitamine metabolism

18.1004 Co-factor and vitamine metabolism

18.1005 Co-factor and vitamine metabolism

18.1006 Co-factor and vitamine metabolism

18.1007 Co-factor and vitamine metabolism

18.1008 Co-factor and vitamine metabolism

18.1009 Co-factor and vitamine metabolism.lumichrome

18.11 Co-factor and vitamine metabolism.lipoic acid

18.12 Co-factor and vitamine metabolism.NADH kinase

18.13 Co-factor and vitamine metabolism.isochorismatase

18.2 Co-factor and vitamine metabolism.thiamine

18.2.1 Co-factor and vitamine metabolism.thiamine.thiamine diphosphokinase

18.2.2 Co-factor and vitamine metabolism.thiamine.hydroxymethylpyrimidine kinase

18.3 Co-factor and vitamine metabolism.riboflavin

18.3.1 Co-factor and vitamine metabolism.riboflavin.GTP cyclohydrolase II

18.3.2 Co-factor and vitamine metabolism.riboflavin.riboflavin synthase

18.4 Co-factor and vitamine metabolism.pantothenate

18.4.1 Co-factor and vitamine metabolism.pantothenate.branched-chain amino acid aminotransferase

18.4.2 Co-factor and vitamine metabolism.pantothenate.3-methyl-2-oxobutanoate hydroxymethyltransferase (KPHMT,PANB)

18.4.3 Co-factor and vitamine metabolism.pantothenate.2-dehydropantoate 2-reductase

18.4.4 Co-factor and vitamine metabolism.pantothenate.pantoate beta-alanine ligase (PANC, pantothenate synthetase)

18.4.5 Co-factor and vitamine metabolism.pantothenate.pantothenate kinase (PANK)

18.4.6 Co-factor and vitamine metabolism.pantothenate.phosphopantothenoylcysteine synthetase (PPCS)

18.4.7 Co-factor and vitamine metabolism.pantothenate.phosphopantothenoylcysteine decarboxylase (PPCDC)

18.4.8 Co-factor and vitamine metabolism.pantothenate.pantetheine-phosphate adenylyltransferase (PPAT)

18.4.9 Co-factor and vitamine metabolism.pantothenate.dephospho-CoA kinase (DPCK)

18.5 Co-factor and vitamine metabolism.folate & vitamine K

18.5.1 Co-factor and vitamine metabolism.folate & vitamine K.folate

18.5.1.1 Co-factor and vitamine metabolism.folate & vitamine K.folate.methylenetetrahydrofolate dehydrogenase (NADP+ dependent)

18.5.2 Co-factor and vitamine metabolism.folate & vitamine K.vitamine K

18.5.2.1 Co-factor and vitamine metabolism.folate & vitamine K.vitamine K.isochorismate synthase

18.5.2.4 Co-factor and vitamine metabolism.folate & vitamine K.vitamine K.o-Succinyl-BenzoylCoa Synthetase

18.5.2.5 Co-factor and vitamine metabolism.folate & vitamine K.vitamine K.naphthoate synthase

18.5.2.7 Co-factor and vitamine metabolism.folate & vitamine K.vitamine K.DHNA Phytyltransferase

18.5.2.8 Co-factor and vitamine metabolism.folate & vitamine K.vitamine K.2-phytyl-1,4-naphthoquinone methyltransferase

18.5.2.8.1 Co-factor and vitamine metabolism.folate & vitamine K.vitamine K.2-phytyl-1,4-naphthoquinone methyltransferase.PHYLLO

18.6 Co-factor and vitamine metabolism.biotin

18.6.1 Co-factor and vitamine metabolism.biotin.biotin synthase

18.7 Co-factor and vitamine metabolism.iron-sulphur clusters

18.8 Co-factor and vitamine metabolism.ubiquinone

18.8.1 Co-factor and vitamine metabolism.ubiquinone.hexaprenyldihydroxybenzoate methyltransferase

19 tetrapyrrole synthesis

19.1 tetrapyrrole synthesis.glu-tRNA synthetase

19.10 tetrapyrrole synthesis.magnesium chelatase

19.11 tetrapyrrole synthesis.magnesium protoporphyrin IX methyltransferase

19.12 tetrapyrrole synthesis.magnesium-protoporphyrin IX monomethyl ester (oxidative) cyclase

19.13 tetrapyrrole synthesis.divinyl chlorophyllide-a 8-vinyl-reductase

19.14 tetrapyrrole synthesis.protochlorophyllide reductase

19.15 tetrapyrrole synthesis.chlorophyll synthase

19.16 tetrapyrrole synthesis.chlorophyll b synthase

19.2 tetrapyrrole synthesis.glu-tRNA reductase

19.20 tetrapyrrole synthesis.ferrochelatase

19.21 tetrapyrrole synthesis.heme oxygenase

19.3 tetrapyrrole synthesis.GSA

19.30 tetrapyrrole synthesis.uroporphyrin-III C-methyltransferase

19.32 tetrapyrrole synthesis.sirohydrochlorin ferrochelatase

19.33 tetrapyrrole synthesis.5-aminolevulinate synthase

19.4 tetrapyrrole synthesis.ALA dehydratase

19.40 tetrapyrrole synthesis.regulation

19.5 tetrapyrrole synthesis.porphobilinogen deaminase

19.6 tetrapyrrole synthesis.uroporphyrinogen III synthase

19.7 tetrapyrrole synthesis.uroporphyrinogen decarboxylase

19.8 tetrapyrrole synthesis.coproporphyrinogen III oxidase

19.9 tetrapyrrole synthesis.protoporphyrin IX oxidase

19.99 tetrapyrrole synthesis.unspecified

2 major CHO metabolism

2.1 major CHO metabolism.synthesis

2.1.1 major CHO metabolism.synthesis.sucrose

2.1.1.1 major CHO metabolism.synthesis.sucrose.SPS

2.1.1.1001 major CHO metabolism.synthesis.sucrose

2.1.1.1002 major CHO metabolism.synthesis.sucrose

2.1.1.1003 major CHO metabolism.synthesis.sucrose

2.1.1.1004 major CHO metabolism.synthesis.sucrose

2.1.1.1005 major CHO metabolism.synthesis.sucrose

2.1.1.1006 major CHO metabolism.synthesis.sucrose

2.1.1.1007 major CHO metabolism.synthesis.sucrose

2.1.1.1008 major CHO metabolism.synthesis.sucrose

2.1.1.2 major CHO metabolism.synthesis.sucrose.SPP

2.1.1.3 major CHO metabolism.synthesis.sucrose.FBPase

2.1.2 major CHO metabolism.synthesis.starch

2.1.2.1 major CHO metabolism.synthesis.starch.AGPase

2.1.2.1001 major CHO metabolism.synthesis.starch

2.1.2.1002 major CHO metabolism.synthesis.starch

2.1.2.1003 major CHO metabolism.synthesis.starch

2.1.2.1004 major CHO metabolism.synthesis.starch

2.1.2.2 major CHO metabolism.synthesis.starch.starch synthase

2.1.2.3 major CHO metabolism.synthesis.starch.starch branching

2.1.2.4 major CHO metabolism.synthesis.starch.debranching

2.1.2.5 major CHO metabolism.synthesis.starch.transporter

2.1.2.60 major CHO metabolism.synthesis.starch.ADP Glucose Phosphorylase

2.2 major CHO metabolism.degradation

2.2.1 major CHO metabolism.degradation.sucrose

2.2.1.1 major CHO metabolism.degradation.sucrose.fructokinase

2.2.1.1001 major CHO metabolism.degradation.sucrose

2.2.1.1002 major CHO metabolism.degradation.sucrose

2.2.1.3 major CHO metabolism.degradation.sucrose.invertases

2.2.1.3.1 major CHO metabolism.degradation.sucrose.invertases.neutral

2.2.1.3.2 major CHO metabolism.degradation.sucrose.invertases.cell wall

2.2.1.3.3 major CHO metabolism.degradation.sucrose.invertases.vacuolar

2.2.1.3.30 major CHO metabolism.degradation.sucrose.invertases.inhibitors

2.2.1.4 major CHO metabolism.degradation.sucrose.hexokinase

2.2.1.5 major CHO metabolism.degradation.sucrose.Susy

2.2.1.99 major CHO metabolism.degradation.sucrose.misc

2.2.10 major CHO metabolism.degradation.acetate metabolism

2.2.10.1 major CHO metabolism.degradation.acetate metabolism.acetate kinase

2.2.10.2 major CHO metabolism.degradation.acetate metabolism.phosphate acetyltransferase

2.2.10.3 major CHO metabolism.degradation.acetate metabolism.acetyl-CoA synthetase

2.2.2 major CHO metabolism.degradation.starch

2.2.2.1 major CHO metabolism.degradation.starch.starch cleavage

2.2.2.1.1 major CHO metabolism.degradation.starch.starch cleavage.alpha amylase

2.2.2.1.2 major CHO metabolism.degradation.starch.starch cleavage.beta amylase

2.2.2.10 major CHO metabolism.degradation.starch.laforin like phosphoglucan phosphatase (SEX4)

2.2.2.1001 major CHO metabolism.degradation.starch

2.2.2.1002 major CHO metabolism.degradation.starch

2.2.2.1003 major CHO metabolism.degradation.starch

2.2.2.1004 major CHO metabolism.degradation.starch

2.2.2.2 major CHO metabolism.degradation.starch.starch phosphorylase

2.2.2.3 major CHO metabolism.degradation.starch.glucan water dikinase

2.2.2.4 major CHO metabolism.degradation.starch.D enzyme

2.2.2.6 major CHO metabolism.degradation.starch.transporter

2.2.2.8 major CHO metabolism.degradation.starch.ISA3

2.2.2.9 major CHO metabolism.degradation.starch.limit dextrinase/ pullulanase

20 stress

20.1 stress.biotic

20.1.1 stress.biotic.respiratory burst

20.1.1001 stress.biotic

20.1.2 stress.biotic.receptors

20.1.3 stress.biotic.signalling

20.1.3.1 stress.biotic.signalling.MLO-like

20.1.4 stress.biotic.kinases

20.1.5 stress.biotic.regulation of transcription

20.1.7 stress.biotic.PR-proteins

20.1.7.12 stress.biotic.PR-proteins.plant defensins

20.1.7.6 stress.biotic.PR-proteins.proteinase inhibitors

20.1.7.6.1 stress.biotic.PR-proteins.proteinase inhibitors.trypsin inhibitor

20.1001 stress

20.1002 stress

20.1003 stress

20.2 stress.abiotic

20.2.1 stress.abiotic.heat

20.2.1001 stress.abiotic

20.2.2 stress.abiotic.cold

20.2.3 stress.abiotic.drought/salt

20.2.4 stress.abiotic.touch/wounding

20.2.5 stress.abiotic.light

20.2.99 stress.abiotic.unspecified

21 redox

21.1 redox.thioredoxin

21.1.1 redox.thioredoxin.PDIL

21.1.2 redox.thioredoxin.QSOX

21.1001 redox

21.1002 redox

21.1003 redox

21.1004 redox

21.1005 redox

21.1006 redox

21.1007 redox

21.1008 redox

21.1009 redox

21.1010 redox

21.1011 redox

21.2 redox.ascorbate and glutathione

21.2.1 redox.ascorbate and glutathione.ascorbate

21.2.1.1 redox.ascorbate and glutathione.ascorbate.GME

21.2.1.2 redox.ascorbate and glutathione.ascorbate.GDP-L-galactose-hexose-1-phosphate guanyltransferase

21.2.1.3 redox.ascorbate and glutathione.ascorbate.L-galactose-1-phosphate phosphatase

21.2.1.4 redox.ascorbate and glutathione.ascorbate.L-galactose dehydrogenase

21.2.1.5 redox.ascorbate and glutathione.ascorbate.L-Galactono-1,4-lactone dehydrogenase

21.2.2 redox.ascorbate and glutathione.glutathione

21.3 redox.heme

21.4 redox.glutaredoxins

21.5 redox.peroxiredoxin

21.5.1 redox.peroxiredoxin.BAS1

21.5.2 redox.peroxiredoxin.PER1

21.6 redox.dismutases and catalases

21.99 redox.misc

22 polyamine metabolism

22.1 polyamine metabolism.synthesis

22.1.1 polyamine metabolism.synthesis.ornithine decarboxylase

22.1.2 polyamine metabolism.synthesis.SAM decarboxylase

22.1.3 polyamine metabolism.synthesis.arginine decarboxylase

22.1.4 polyamine metabolism.synthesis.agmatine deiminase

22.1.5 polyamine metabolism.synthesis.N-carbamoylputrescine amidohydrolase

22.1.6 polyamine metabolism.synthesis.spermidine synthase

22.1.7 polyamine metabolism.synthesis.spermine synthase

22.1001 polyamine metabolism

22.1002 polyamine metabolism

22.1003 polyamine metabolism

22.1004 polyamine metabolism

22.1005 polyamine metabolism

22.2 polyamine metabolism.degradation

22.2.1 polyamine metabolism.degradation.polyamin oxidase

23 nucleotide metabolism

23.1 nucleotide metabolism.synthesis

23.1.1 nucleotide metabolism.synthesis.pyrimidine

23.1.1.1 nucleotide metabolism.synthesis.pyrimidine.carbamoyl phosphate synthetase

23.1.1.10 nucleotide metabolism.synthesis.pyrimidine.CTP synthetase

23.1.1.1001 nucleotide metabolism.synthesis.pyrimidines

23.1.1.1002 nucleotide metabolism.synthesis.pyrimidines

23.1.1.1003 nucleotide metabolism.synthesis.pyrimidines

23.1.1.1004 nucleotide metabolism.synthesis.pyrimidines

23.1.1.1005 nucleotide metabolism.synthesis.pyrimidines

23.1.1.1006 nucleotide metabolism.synthesis.pyrimidines

23.1.1.1007 nucleotide metabolism.synthesis.pyrimidines

23.1.1.1008 nucleotide metabolism.synthesis.pyrimidines

23.1.1.1009 nucleotide metabolism.synthesis.pyrimidines

23.1.1.1010 nucleotide metabolism.synthesis.pyrimidines

23.1.1.1011 nucleotide metabolism.synthesis.pyrimidines

23.1.1.2 nucleotide metabolism.synthesis.pyrimidine.aspartate transcarbamoylase

23.1.1.3 nucleotide metabolism.synthesis.pyrimidine.dihydroorotase

23.1.1.4 nucleotide metabolism.synthesis.pyrimidine.dihydroorotate dehydrogenase

23.1.1.5 nucleotide metabolism.synthesis.pyrimidine.orotate phosphoribosyltransferase

23.1.1.6 nucleotide metabolism.synthesis.pyrimidine.orotidine-5'-phosphate decarboxylase

23.1.2 nucleotide metabolism.synthesis.purine

23.1.2.1 nucleotide metabolism.synthesis.purine.amidophosphoribosyltransferase

23.1.2.10 nucleotide metabolism.synthesis.purine.IMP synthase

23.1.2.1001 nucleotide metabolism.synthesis.purines

23.1.2.1002 nucleotide metabolism.synthesis.purines

23.1.2.1003 nucleotide metabolism.synthesis.purines

23.1.2.1004 nucleotide metabolism.synthesis.purines

23.1.2.1005 nucleotide metabolism.synthesis.purines

23.1.2.1006 nucleotide metabolism.synthesis.purines

23.1.2.1007 nucleotide metabolism.synthesis.purines

23.1.2.1008 nucleotide metabolism.synthesis.purines

23.1.2.1009 nucleotide metabolism.synthesis.purines

23.1.2.1010 nucleotide metabolism.synthesis.purines

23.1.2.1011 nucleotide metabolism.synthesis.purines

23.1.2.1012 nucleotide metabolism.synthesis.purines

23.1.2.2 nucleotide metabolism.synthesis.purine.GAR Synthetase

23.1.2.20 nucleotide metabolism.synthesis.purine.adenylosuccinate synthase

23.1.2.3 nucleotide metabolism.synthesis.purine.GAR transformylase

23.1.2.30 nucleotide metabolism.synthesis.purine.IMP dehydrogenase

23.1.2.31 nucleotide metabolism.synthesis.purine.GMP synthetase

23.1.2.4 nucleotide metabolism.synthesis.purine.FGAR amidotransferase

23.1.2.5 nucleotide metabolism.synthesis.purine.AIR synthase

23.1.2.6 nucleotide metabolism.synthesis.purine.AIR carboxylase

23.1.2.7 nucleotide metabolism.synthesis.purine.SAICAR synthetase

23.1.2.8 nucleotide metabolism.synthesis.purine.SAICAR lyase

23.1.2.9 nucleotide metabolism.synthesis.purine.AICAR transformylase

23.1.3 nucleotide metabolism.synthesis.PRS-PP

23.1.99 nucleotide metabolism.synthesis.unspecified

23.2 nucleotide metabolism.degradation

23.2.1 nucleotide metabolism.degradation.pyrimidine

23.2.1.1 nucleotide metabolism.degradation.pyrimidine.5'-nucleotidase

23.2.1.2 nucleotide metabolism.degradation.pyrimidine.uridine nucleosidase

23.2.1.3 nucleotide metabolism.degradation.pyrimidine.dihydrouracil dehydrogenase

23.2.1.4 nucleotide metabolism.degradation.pyrimidine.dihydropyrimidinase

23.2.1.5 nucleotide metabolism.degradation.pyrimidine.beta-ureidopropionase

23.2.1.6 nucleotide metabolism.degradation.pyrimidine.cytidine deaminase

23.2.2 nucleotide metabolism.degradation.purine

23.2.2.1 nucleotide metabolism.degradation.purine.AMP deaminase

23.2.2.10 nucleotide metabolism.degradation.purine.guanine deaminase

23.2.2.1001 nucleotide metabolism.degradation.purine

23.2.2.1002 nucleotide metabolism.degradation.purine

23.2.2.1003 nucleotide metabolism.degradation.purine

23.2.2.1004 nucleotide metabolism.degradation.purine

23.2.2.1005 nucleotide metabolism.degradation.purine

23.2.2.1006 nucleotide metabolism.degradation.purine

23.2.2.1007 nucleotide metabolism.degradation.purine

23.2.2.11 nucleotide metabolism.degradation.purine.xanthine dehydrogenase

23.2.2.8 nucleotide metabolism.degradation.purine.guanosine deaminase

23.2.2.9 nucleotide metabolism.degradation.purine.guanosine nucleosidase

23.3 nucleotide metabolism.salvage

23.3.1 nucleotide metabolism.salvage.phosphoribosyltransferases

23.3.1.1 nucleotide metabolism.salvage.phosphoribosyltransferases.aprt

23.3.1.2 nucleotide metabolism.salvage.phosphoribosyltransferases.hgprt

23.3.1.3 nucleotide metabolism.salvage.phosphoribosyltransferases.upp

23.3.2 nucleotide metabolism.salvage.nucleoside kinases

23.3.2.1 nucleotide metabolism.salvage.nucleoside kinases.adenosine kinase

23.3.2.2 nucleotide metabolism.salvage.nucleoside kinases.uridine kinase

23.3.2.3 nucleotide metabolism.salvage.nucleoside kinases.thymidine kinase

23.3.3 nucleotide metabolism.salvage.NUDIX hydrolases

23.4 nucleotide metabolism.phosphotransfer and pyrophosphatases

23.4.1 nucleotide metabolism.phosphotransfer and pyrophosphatases.adenylate kinase

23.4.10 nucleotide metabolism.phosphotransfer and pyrophosphatases.nucleoside diphosphate kinase

23.4.2 nucleotide metabolism.phosphotransfer and pyrophosphatases.guanylate kinase

23.4.3 nucleotide metabolism.phosphotransfer and pyrophosphatases.uridylate kinase

23.4.4 nucleotide metabolism.phosphotransfer and pyrophosphatases.thymidylate kinase

23.4.99 nucleotide metabolism.phosphotransfer and pyrophosphatases.misc

23.5 nucleotide metabolism.deoxynucleotide metabolism

23.5.1 nucleotide metabolism.deoxynucleotide metabolism.dihydrofolate reductase-thymidylate synthase

23.5.2 nucleotide metabolism.deoxynucleotide metabolism.pseudouridine synthase

23.5.3 nucleotide metabolism.deoxynucleotide metabolism.cytosine deaminase

23.5.4 nucleotide metabolism.deoxynucleotide metabolism.ribonucleoside-diphosphate reductase

23.5.5 nucleotide metabolism.deoxynucleotide metabolism.dUTP diphosphatase

23.5.6 nucleotide metabolism.deoxynucleotide metabolism.uridylyl transferase

23.6 nucleotide metabolism.signalling

24 Biodegradation of Xenobiotics

24.1 Biodegradation of Xenobiotics.hydroxyacylglutathione hydrolase

24.2 Biodegradation of Xenobiotics.lactoylglutathione lyase

24.3 Biodegradation of Xenobiotics.3-hydroxybutyryl-CoA dehydrogenase

25 C1-metabolism

25.1 C1-metabolism.glycine hydroxymethyltransferase

25.10 C1-metabolism.formate dehydrogenase

25.11 C1-metabolism.S-(hydroxymethyl)glutathione dehydrogenase & S-(hydroxymethyl)glutathione synthase

25.2 C1-metabolism.formate-tetrahydrofolate ligase

25.3 C1-metabolism.dihydropteridine diphosphokinase

25.4 C1-metabolism.5-formyltetrahydrofolate cyclo-ligase

25.5 C1-metabolism.Methylenetetrahydrofolate dehydrogenase & Methenyltetrahydrofolate cyclohydrolase

25.6 C1-metabolism.methylenetetrahydrofolate reductase

25.7 C1-metabolism.GTP cyclohydrolase I

25.8 C1-metabolism.tetrahydrofolate synthase

25.9 C1-metabolism.dihydroneopterin aldolase

26 misc

26.1 misc.misc2

26.10 misc.cytochrome P450

26.11 misc.alcohol dehydrogenases

26.11.1 misc.alcohol dehydrogenases.cinnamyl alcohol dehydrogenase

26.12 misc.peroxidases

26.13 misc.acid and other phosphatases

26.14 misc.oxygenases

26.15 misc.carbonic anhydrases

26.16 misc.myrosinases-lectin-jacalin

26.17 misc.dynamin

26.18 misc.invertase/pectin methylesterase inhibitor family protein

26.19 misc.plastocyanin-like

26.2 misc.UDP glucosyl and glucoronyl transferases

26.20 misc.ferredoxin-like

26.21 misc.protease inhibitor/seed storage/lipid transfer protein (LTP) family protein

26.21.1 misc.protease inhibitor/seed storage/lipid transfer protein (LTP) family protein.protease inhibitor

26.22 misc.short chain dehydrogenase/reductase (SDR)

26.23 misc.rhodanese

26.24 misc.GCN5-related N-acetyltransferase

26.25 misc.sulfotransferase

26.26 misc.aminotransferases

26.26.1 misc.aminotransferases.aminotransferase class IV family protein

26.27 misc.calcineurin-like phosphoesterase family protein

26.28 misc.GDSL-motif lipase

26.3 misc.gluco-, galacto- and mannosidases

26.3.1 misc.gluco-, galacto- and mannosidases.alpha-galactosidase

26.3.2 misc.gluco-, galacto- and mannosidases.beta-galactosidase

26.3.3 misc.gluco-, galacto- and mannosidases.alpha-mannosidase

26.3.4 misc.gluco-, galacto- and mannosidases.endoglucanase

26.3.5 misc.gluco-, galacto- and mannosidases.glycosyl hydrolase family 5

26.30 misc. other Ferredoxins and Rieske domain

26.31 misc.zinc finger

26.4 misc.beta 1,3 glucan hydrolases

26.4.1 misc.beta 1,3 glucan hydrolases.glucan endo-1,3-beta-glucosidase

26.5 misc.acyl transferases

26.6 misc.O-methyl transferases

26.7 misc.oxidases - copper, flavone etc

26.8 misc.nitrilases, *nitrile lyases, berberine bridge enzymes, reticuline oxidases, troponine reductases

26.9 misc.glutathione S transferases

27 RNA

27.1 RNA.processing

27.1.1 RNA.processing.splicing

27.1.19 RNA.processing.ribonucleases

27.1.2 RNA.processing.RNA helicase

27.1.20 RNA.processing.degradation dicer

27.1.21 RNA.processing.siRNA methyltransferase

27.1001 RNA

27.2 RNA.transcription

27.2.1 RNA.transcription.RNA Polymerases

27.3 RNA.regulation of transcription

27.3.1 RNA.regulation of transcription.ABI3/VP1-related B3-domain-containing transcription factor family

27.3.10 RNA.regulation of transcription.C2C2(Zn) YABBY family

27.3.11 RNA.regulation of transcription.C2H2 zinc finger family

27.3.12 RNA.regulation of transcription.C3H zinc finger family

27.3.13 RNA.regulation of transcription.CCAAT box binding factor family, DR1

27.3.14 RNA.regulation of transcription.CCAAT box binding factor family, HAP2

27.3.15 RNA.regulation of transcription.CCAAT box binding factor family, HAP3

27.3.16 RNA.regulation of transcription.CCAAT box binding factor family, HAP5

27.3.17 RNA.regulation of transcription.CPP(Zn),CPP1-related transcription factor family

27.3.18 RNA.regulation of transcription.E2F/DP transcription factor family

27.3.19 RNA.regulation of transcription.EIN3-like(EIL) transcription factor family

27.3.2 RNA.regulation of transcription.Alfin-like

27.3.20 RNA.regulation of transcription.G2-like transcription factor family, GARP

27.3.21 RNA.regulation of transcription.GRAS transcription factor family

27.3.22 RNA.regulation of transcription.HB,Homeobox transcription factor family

27.3.23 RNA.regulation of transcription.HSF,Heat-shock transcription factor family

27.3.24 RNA.regulation of transcription.MADS box transcription factor family

27.3.25 RNA.regulation of transcription.MYB domain transcription factor family

27.3.26 RNA.regulation of transcription.MYB-related transcription factor family

27.3.27 RNA.regulation of transcription.NAC domain transcription factor family

27.3.28 RNA.regulation of transcription.SBP,Squamosa promoter binding protein family

27.3.29 RNA.regulation of transcription.TCP transcription factor family

27.3.3 RNA.regulation of transcription.AP2/EREBP, APETALA2/Ethylene-responsive element binding protein family

27.3.30 RNA.regulation of transcription.Trihelix, Triple-Helix transcription factor family

27.3.31 RNA.regulation of transcription.TUB transcription factor family

27.3.32 RNA.regulation of transcription.WRKY domain transcription factor family

27.3.33 RNA.regulation of transcription.TUB transcription factor family

27.3.34 RNA.regulation of transcription.Orphan family

27.3.35 RNA.regulation of transcription.bZIP transcription factor family

27.3.36 RNA.regulation of transcription.Argonaute

27.3.37 RNA.regulation of transcription.AS2,Lateral Organ Boundaries Gene Family

27.3.38 RNA.regulation of transcription.AT-rich interaction domain containing transcription factor family

27.3.39 RNA.regulation of transcription.AtSR Transcription Factor family

27.3.4 RNA.regulation of transcription.ARF, Auxin Response Factor family

27.3.40 RNA.regulation of transcription.Aux/IAA family

27.3.41 RNA.regulation of transcription.B3 transcription factor family

27.3.42 RNA.regulation of transcription.Bromodomain proteins

27.3.44 RNA.regulation of transcription.Chromatin Remodeling Factors

27.3.46 RNA.regulation of transcription.DNA methyltransferases

27.3.47 RNA.regulation of transcription.ELF3

27.3.48 RNA.regulation of transcription.FHA transcription factor

27.3.49 RNA.regulation of transcription.GeBP like

27.3.5 RNA.regulation of transcription.ARR

27.3.50 RNA.regulation of transcription.General Transcription

27.3.51 RNA.regulation of transcription.General Transcription, TBP-binding protein

27.3.52 RNA.regulation of transcription.Global transcription factor group

27.3.53 RNA.regulation of transcription.High mobility group (HMG) family

27.3.54 RNA.regulation of transcription.Histone acetyltransferases

27.3.55 RNA.regulation of transcription.HDA

27.3.57 RNA.regulation of transcription.JUMONJI family

27.3.58 RNA.regulation of transcription.LUG

27.3.59 RNA.regulation of transcription.Methyl binding domain proteins

27.3.6 RNA.regulation of transcription.bHLH,Basic Helix-Loop-Helix family

27.3.60 RNA.regulation of transcription.NIN-like bZIP-related family

27.3.61 RNA.regulation of transcription.NPR1/NIM1

27.3.62 RNA.regulation of transcription.Nucleosome/chromatin assembly factor group

27.3.63 RNA.regulation of transcription.PHD finger transcription factor

27.3.64 RNA.regulation of transcription.PHOR1

27.3.65 RNA.regulation of transcription.Polycomb Group (PcG)

27.3.66 RNA.regulation of transcription.Psudo ARR transcription factor family

27.3.67 RNA.regulation of transcription.putative transcription regulator

27.3.68 RNA.regulation of transcription.PWWP domain protein

27.3.69 RNA.regulation of transcription.SET-domain transcriptional regulator family

27.3.7 RNA.regulation of transcription.C2C2(Zn) CO-like, Constans-like zinc finger family

27.3.70 RNA.regulation of transcription.Silencing Group

27.3.71 RNA.regulation of transcription.SNF7

27.3.72 RNA.regulation of transcription.Transcriptional Adaptor Zinc Bundle (TAZ) domain family

27.3.73 RNA.regulation of transcription.Zn-finger(CCHC)

27.3.75 RNA.regulation of transcription.GRP

27.3.8 RNA.regulation of transcription.C2C2(Zn) DOF zinc finger family

27.3.80 RNA.regulation of transcription.zf-HD

27.3.81 RNA.regulation of transcription.S1FA

27.3.82 RNA.regulation of transcription.plant TF (pbf2)

27.3.83 RNA.regulation of transcription.GRF zinc finger family

27.3.9 RNA.regulation of transcription.C2C2(Zn) GATA transcription factor family

27.3.99 RNA.regulation of transcription.unclassified

27.4 RNA.RNA binding

28 DNA

28.1 DNA.synthesis/chromatin structure

28.1.1 DNA.synthesis/chromatin structure.retrotransposon/transposase

28.1.1.1 DNA.synthesis/chromatin structure.retrotransposon/transposase.gypsy-like retrotransposon

28.1.1.2 DNA.synthesis/chromatin structure.retrotransposon/transposase.non-LTR retrotransposon

28.1.1.3 DNA.synthesis/chromatin structure.retrotransposon/transposase.copia-like retrotransposon

28.1.1.4 DNA.synthesis/chromatin structure.retrotransposon/transposase.hat-like transposase

28.1.1.5 DNA.synthesis/chromatin structure.retrotransposon/transposase.CACTA-like transposase

28.1.1.6 DNA.synthesis/chromatin structure.retrotransposon/transposase.Mariner-like transposase

28.1.1.7 DNA.synthesis/chromatin structure.retrotransposon/transposase.mutator-like transposase

28.1.1.8 DNA.synthesis/chromatin structure.retrotransposon/transposase.ac-like transposase

28.1.3 DNA.synthesis/chromatin structure.histone

28.1.3.1 DNA.synthesis/chromatin structure.histone.H1

28.1.3.2 DNA.synthesis/chromatin structure.histone.core

28.1.3.2.1 DNA.synthesis/chromatin structure.histone.core.H2A

28.1.3.2.2 DNA.synthesis/chromatin structure.histone.core.H2B

28.1.3.2.3 DNA.synthesis/chromatin structure.histone.core.H3

28.1.3.2.4 DNA.synthesis/chromatin structure.histone.core.H4

28.2 DNA.repair

28.99 DNA.unspecified

29 protein

29.1 protein.aa activation

29.1.1 protein.aa activation.tyrosine-tRNA ligase

29.1.10 protein.aa activation.methionine-tRNA ligase

29.1.11 protein.aa activation.serine-tRNA ligase

29.1.12 protein.aa activation.aspartate-tRNA ligase

29.1.13 protein.aa activation.tryptophan-tRNA ligase

29.1.14 protein.aa activation.glycine-tRNA ligase

29.1.15 protein.aa activation.proline-tRNA ligase

29.1.16 protein.aa activation.cysteine-tRNA ligase

29.1.17 protein.aa activation.glutamate-tRNA ligase

29.1.18 protein.aa activation.glutamine-tRNA ligase

29.1.19 protein.aa activation.arginine-tRNA ligase

29.1.20 protein.aa activation.phenylalanine-tRNA ligase

29.1.21 protein.aa activation.histidine-tRNA ligase

29.1.22 protein.aa activation.asparagine-tRNA ligase

29.1.3 protein.aa activation.threonine-tRNA ligase

29.1.30 protein.aa activation.pseudouridylate synthase

29.1.4 protein.aa activation.leucine-tRNA ligase

29.1.40 protein.aa activation.bifunctional aminoacyl-tRNA synthetase

29.1.5 protein.aa activation.isoleucine-tRNA ligase

29.1.6 protein.aa activation.lysine-tRNA ligase

29.1.7 protein.aa activation.alanine-tRNA ligase

29.1.9 protein.aa activation.valine-tRNA ligase

29.2 protein.synthesis

29.2.1 protein.synthesis.ribosomal protein

29.2.1.1 protein.synthesis.ribosomal protein.prokaryotic

29.2.1.1.1 protein.synthesis.ribosomal protein.prokaryotic.chloroplast

29.2.1.1.1.1 protein.synthesis.ribosomal protein.prokaryotic.chloroplast.30S subunit

29.2.1.1.1.1.1 protein.synthesis.ribosomal protein.prokaryotic.chloroplast.30S subunit.S1

29.2.1.1.1.1.10 protein.synthesis.ribosomal protein.prokaryotic.chloroplast.30S subunit.S10

29.2.1.1.1.1.11 protein.synthesis.ribosomal protein.prokaryotic.chloroplast.30S subunit.S11

29.2.1.1.1.1.12 protein.synthesis.ribosomal protein.prokaryotic.chloroplast.30S subunit.S12

29.2.1.1.1.1.13 protein.synthesis.ribosomal protein.prokaryotic.chloroplast.30S subunit.S13

29.2.1.1.1.1.14 protein.synthesis.ribosomal protein.prokaryotic.chloroplast.30S subunit.S14

29.2.1.1.1.1.15 protein.synthesis.ribosomal protein.prokaryotic.chloroplast.30S subunit.S15

29.2.1.1.1.1.16 protein.synthesis.ribosomal protein.prokaryotic.chloroplast.30S subunit.S16

29.2.1.1.1.1.17 protein.synthesis.ribosomal protein.prokaryotic.chloroplast.30S subunit.S17

29.2.1.1.1.1.18 protein.synthesis.ribosomal protein.prokaryotic.chloroplast.30S subunit.S18

29.2.1.1.1.1.19 protein.synthesis.ribosomal protein.prokaryotic.chloroplast.30S subunit.S19

29.2.1.1.1.1.2 protein.synthesis.ribosomal protein.prokaryotic.chloroplast.30S subunit.S2

29.2.1.1.1.1.20 protein.synthesis.ribosomal protein.prokaryotic.chloroplast.30S subunit.S20

29.2.1.1.1.1.21 protein.synthesis.ribosomal protein.prokaryotic.chloroplast.30S subunit.S21

29.2.1.1.1.1.3 protein.synthesis.ribosomal protein.prokaryotic.chloroplast.30S subunit.S3

29.2.1.1.1.1.31 protein.synthesis.ribosomal protein.prokaryotic.chloroplast.30S subunit.S31

29.2.1.1.1.1.4 protein.synthesis.ribosomal protein.prokaryotic.chloroplast.30S subunit.S4

29.2.1.1.1.1.5 protein.synthesis.ribosomal protein.prokaryotic.chloroplast.30S subunit.S5

29.2.1.1.1.1.530 protein.synthesis.ribosomal protein.prokaryotic.chloroplast.30S subunit.S30A

29.2.1.1.1.1.6 protein.synthesis.ribosomal protein.prokaryotic.chloroplast.30S subunit.S6

29.2.1.1.1.1.7 protein.synthesis.ribosomal protein.prokaryotic.chloroplast.30S subunit.S7

29.2.1.1.1.1.8 protein.synthesis.ribosomal protein.prokaryotic.chloroplast.30S subunit.S8

29.2.1.1.1.1.83 protein.synthesis.ribosomal protein.prokaryotic.chloroplast.30S subunit.PSRP3

29.2.1.1.1.1.9 protein.synthesis.ribosomal protein.prokaryotic.chloroplast.30S subunit.S9

29.2.1.1.1.2 protein.synthesis.ribosomal protein.prokaryotic.chloroplast.50S subunit

29.2.1.1.1.2.1 protein.synthesis.ribosomal protein.prokaryotic.chloroplast.50S subunit.L1

29.2.1.1.1.2.10 protein.synthesis.ribosomal protein.prokaryotic.chloroplast.50S subunit.L10

29.2.1.1.1.2.11 protein.synthesis.ribosomal protein.prokaryotic.chloroplast.50S subunit.L11

29.2.1.1.1.2.12 protein.synthesis.ribosomal protein.prokaryotic.chloroplast.50S subunit.L12

29.2.1.1.1.2.13 protein.synthesis.ribosomal protein.prokaryotic.chloroplast.50S subunit.L13

29.2.1.1.1.2.14 protein.synthesis.ribosomal protein.prokaryotic.chloroplast.50S subunit.L14

29.2.1.1.1.2.15 protein.synthesis.ribosomal protein.prokaryotic.chloroplast.50S subunit.L15

29.2.1.1.1.2.16 protein.synthesis.ribosomal protein.prokaryotic.chloroplast.50S subunit.L16

29.2.1.1.1.2.17 protein.synthesis.ribosomal protein.prokaryotic.chloroplast.50S subunit.L17

29.2.1.1.1.2.18 protein.synthesis.ribosomal protein.prokaryotic.chloroplast.50S subunit.L18

29.2.1.1.1.2.19 protein.synthesis.ribosomal protein.prokaryotic.chloroplast.50S subunit.L19

29.2.1.1.1.2.2 protein.synthesis.ribosomal protein.prokaryotic.chloroplast.50S subunit.L2

29.2.1.1.1.2.20 protein.synthesis.ribosomal protein.prokaryotic.chloroplast.50S subunit.L20

29.2.1.1.1.2.21 protein.synthesis.ribosomal protein.prokaryotic.chloroplast.50S subunit.L21

29.2.1.1.1.2.22 protein.synthesis.ribosomal protein.prokaryotic.chloroplast.50S subunit.L22

29.2.1.1.1.2.23 protein.synthesis.ribosomal protein.prokaryotic.chloroplast.50S subunit.L23

29.2.1.1.1.2.24 protein.synthesis.ribosomal protein.prokaryotic.chloroplast.50S subunit.L24

29.2.1.1.1.2.27 protein.synthesis.ribosomal protein.prokaryotic.chloroplast.50S subunit.L27

29.2.1.1.1.2.28 protein.synthesis.ribosomal protein.prokaryotic.chloroplast.50S subunit.L28

29.2.1.1.1.2.29 protein.synthesis.ribosomal protein.prokaryotic.chloroplast.50S subunit.L29

29.2.1.1.1.2.3 protein.synthesis.ribosomal protein.prokaryotic.chloroplast.50S subunit.L3

29.2.1.1.1.2.31 protein.synthesis.ribosomal protein.prokaryotic.chloroplast.50S subunit.L31

29.2.1.1.1.2.32 protein.synthesis.ribosomal protein.prokaryotic.chloroplast.50S subunit.L32

29.2.1.1.1.2.33 protein.synthesis.ribosomal protein.prokaryotic.chloroplast.50S subunit.L33

29.2.1.1.1.2.34 protein.synthesis.ribosomal protein.prokaryotic.chloroplast.50S subunit.L34

29.2.1.1.1.2.36 protein.synthesis.ribosomal protein.prokaryotic.chloroplast.50S subunit.L36

29.2.1.1.1.2.4 protein.synthesis.ribosomal protein.prokaryotic.chloroplast.50S subunit.L4

29.2.1.1.1.2.40 protein.synthesis.ribosomal protein.prokaryotic.chloroplast.50S subunit.L40

29.2.1.1.1.2.5 protein.synthesis.ribosomal protein.prokaryotic.chloroplast.50S subunit.L5

29.2.1.1.1.2.6 protein.synthesis.ribosomal protein.prokaryotic.chloroplast.50S subunit.L6

29.2.1.1.1.2.85 protein.synthesis.ribosomal protein.prokaryotic.chloroplast.50S subunit.PSRP5

29.2.1.1.1.2.86 protein.synthesis.ribosomal protein.prokaryotic.chloroplast.50S subunit.PSRP6

29.2.1.1.1.2.9 protein.synthesis.ribosomal protein.prokaryotic.chloroplast.50S subunit.L9

29.2.1.1.2 protein.synthesis.ribosomal protein.prokaryotic.mitochondrion

29.2.1.1.2.1 protein.synthesis.ribosomal protein.prokaryotic.mitochondrion.30S subunit

29.2.1.1.2.1.10 protein.synthesis.ribosomal protein.prokaryotic.mitochondrion.30S subunit.S10

29.2.1.1.2.1.11 protein.synthesis.ribosomal protein.prokaryotic.mitochondrion.30S subunit.S11

29.2.1.1.2.1.12 protein.synthesis.ribosomal protein.prokaryotic.mitochondrion.30S subunit.S12

29.2.1.1.2.1.13 protein.synthesis.ribosomal protein.prokaryotic.mitochondrion.30S subunit.S13

29.2.1.1.2.1.14 protein.synthesis.ribosomal protein.prokaryotic.mitochondrion.30S subunit.S14

29.2.1.1.2.1.16 protein.synthesis.ribosomal protein.prokaryotic.mitochondrion.30S subunit.S16

29.2.1.1.2.1.19 protein.synthesis.ribosomal protein.prokaryotic.mitochondrion.30S subunit.S19

29.2.1.1.2.1.2 protein.synthesis.ribosomal protein.prokaryotic.mitochondrion.30S subunit.S2

29.2.1.1.2.1.27 protein.synthesis.ribosomal protein.prokaryotic.mitochondrion.30S subunit.S27

29.2.1.1.2.1.29 protein.synthesis.ribosomal protein.prokaryotic.mitochondrion.30S subunit.S29

29.2.1.1.2.1.3 protein.synthesis.ribosomal protein.prokaryotic.mitochondrion.30S subunit.S3

29.2.1.1.2.1.31 protein.synthesis.ribosomal protein.prokaryotic.mitochondrion.30S subunit.S31

29.2.1.1.2.1.4 protein.synthesis.ribosomal protein.prokaryotic.mitochondrion.30S subunit.S4

29.2.1.1.2.1.7 protein.synthesis.ribosomal protein.prokaryotic.mitochondrion.30S subunit.S7

29.2.1.1.2.1.8 protein.synthesis.ribosomal protein.prokaryotic.mitochondrion.30S subunit.S8

29.2.1.1.2.2 protein.synthesis.ribosomal protein.prokaryotic.mitochondrion.50S subunit

29.2.1.1.2.2.11 protein.synthesis.ribosomal protein.prokaryotic.mitochondrion.50S subunit.L11

29.2.1.1.2.2.16 protein.synthesis.ribosomal protein.prokaryotic.mitochondrion.50S subunit.L16

29.2.1.1.2.2.2 protein.synthesis.ribosomal protein.prokaryotic.mitochondrion.50S subunit.L2

29.2.1.1.2.2.21 protein.synthesis.ribosomal protein.prokaryotic.mitochondrion.50S subunit.L21

29.2.1.1.2.2.29 protein.synthesis.ribosomal protein.prokaryotic.mitochondrion.50S subunit.L29

29.2.1.1.2.2.37 protein.synthesis.ribosomal protein.prokaryotic.mitochondrion.50S subunit.L37

29.2.1.1.2.2.5 protein.synthesis.ribosomal protein.prokaryotic.mitochondrion.50S subunit.L5

29.2.1.1.2.2.6 protein.synthesis.ribosomal protein.prokaryotic.mitochondrion.50S subunit.L6

29.2.1.1.2.51 protein.synthesis.ribosomal protein.prokaryotic.mitochondrion.L51/S25/CI-B8

29.2.1.1.3 protein.synthesis.ribosomal protein.prokaryotic.unknown organellar

29.2.1.1.3.1 protein.synthesis.ribosomal protein.prokaryotic.unknown organellar.30S subunit

29.2.1.1.3.1.1 protein.synthesis.ribosomal protein.prokaryotic.unknown organellar.30S subunit.S1

29.2.1.1.3.1.10 protein.synthesis.ribosomal protein.prokaryotic.unknown organellar.30S subunit.S10

29.2.1.1.3.1.11 protein.synthesis.ribosomal protein.prokaryotic.unknown organellar.30S subunit.S11

29.2.1.1.3.1.12 protein.synthesis.ribosomal protein.prokaryotic.unknown organellar.30S subunit.S12

29.2.1.1.3.1.15 protein.synthesis.ribosomal protein.prokaryotic.unknown organellar.30S subunit.S15

29.2.1.1.3.1.16 protein.synthesis.ribosomal protein.prokaryotic.unknown organellar.30S subunit.S16

29.2.1.1.3.1.17 protein.synthesis.ribosomal protein.prokaryotic.unknown organellar.30S subunit.S17

29.2.1.1.3.1.18 protein.synthesis.ribosomal protein.prokaryotic.unknown organellar.30S subunit.S18

29.2.1.1.3.1.19 protein.synthesis.ribosomal protein.prokaryotic.unknown organellar.30S subunit.S19

29.2.1.1.3.1.21 protein.synthesis.ribosomal protein.prokaryotic.unknown organellar.30S subunit.S21

29.2.1.1.3.1.4 protein.synthesis.ribosomal protein.prokaryotic.unknown organellar.30S subunit.S4

29.2.1.1.3.1.5 protein.synthesis.ribosomal protein.prokaryotic.unknown organellar.30S subunit.S5

29.2.1.1.3.1.6 protein.synthesis.ribosomal protein.prokaryotic.unknown organellar.30S subunit.S6

29.2.1.1.3.1.7 protein.synthesis.ribosomal protein.prokaryotic.unknown organellar.30S subunit.S7

29.2.1.1.3.1.9 protein.synthesis.ribosomal protein.prokaryotic.unknown organellar.30S subunit.S9

29.2.1.1.3.2 protein.synthesis.ribosomal protein.prokaryotic.unknown organellar.50S subunit

29.2.1.1.3.2.1 protein.synthesis.ribosomal protein.prokaryotic.unknown organellar.50S subunit.L1

29.2.1.1.3.2.10 protein.synthesis.ribosomal protein.prokaryotic.unknown organellar.50S subunit.L10

29.2.1.1.3.2.1015 protein.synthesis.ribosomal protein.prokaryotic.unknown organellar.50S subunit.L10/15

29.2.1.1.3.2.11 protein.synthesis.ribosomal protein.prokaryotic.unknown organellar.50S subunit.L11

29.2.1.1.3.2.1185 protein.synthesis.ribosomal protein.prokaryotic.unknown organellar.50S subunit.L18/L5

29.2.1.1.3.2.12 protein.synthesis.ribosomal protein.prokaryotic.unknown organellar.50S subunit.L12

29.2.1.1.3.2.13 protein.synthesis.ribosomal protein.prokaryotic.unknown organellar.50S subunit.L13

29.2.1.1.3.2.14 protein.synthesis.ribosomal protein.prokaryotic.unknown organellar.50S subunit.L14

29.2.1.1.3.2.15 protein.synthesis.ribosomal protein.prokaryotic.unknown organellar.50S subunit.L15

29.2.1.1.3.2.17 protein.synthesis.ribosomal protein.prokaryotic.unknown organellar.50S subunit.L17

29.2.1.1.3.2.1712 protein.synthesis.ribosomal protein.prokaryotic.unknown organellar.50S subunit.L7/L12

29.2.1.1.3.2.18 protein.synthesis.ribosomal protein.prokaryotic.unknown organellar.50S subunit.L18

29.2.1.1.3.2.19 protein.synthesis.ribosomal protein.prokaryotic.unknown organellar.50S subunit.L19

29.2.1.1.3.2.2 protein.synthesis.ribosomal protein.prokaryotic.unknown organellar.50S subunit.L2

29.2.1.1.3.2.20 protein.synthesis.ribosomal protein.prokaryotic.unknown organellar.50S subunit.L20

29.2.1.1.3.2.22 protein.synthesis.ribosomal protein.prokaryotic.unknown organellar.50S subunit.L22

29.2.1.1.3.2.25 protein.synthesis.ribosomal protein.prokaryotic.unknown organellar.50S subunit.L25

29.2.1.1.3.2.28 protein.synthesis.ribosomal protein.prokaryotic.unknown organellar.50S subunit.L28

29.2.1.1.3.2.3 protein.synthesis.ribosomal protein.prokaryotic.unknown organellar.50S subunit.L3

29.2.1.1.3.2.30 protein.synthesis.ribosomal protein.prokaryotic.unknown organellar.50S subunit.L30

29.2.1.1.3.2.32 protein.synthesis.ribosomal protein.prokaryotic.unknown organellar.50S subunit.L32

29.2.1.1.3.2.33 protein.synthesis.ribosomal protein.prokaryotic.unknown organellar.50S subunit.L33

29.2.1.1.3.2.34 protein.synthesis.ribosomal protein.prokaryotic.unknown organellar.50S subunit.L34

29.2.1.1.3.2.35 protein.synthesis.ribosomal protein.prokaryotic.unknown organellar.50S subunit.L35

29.2.1.1.3.2.36 protein.synthesis.ribosomal protein.prokaryotic.unknown organellar.50S subunit.L36

29.2.1.1.3.2.4 protein.synthesis.ribosomal protein.prokaryotic.unknown organellar.50S subunit.L4

29.2.1.1.3.2.510 protein.synthesis.ribosomal protein.prokaryotic.unknown organellar.50S subunit.L10A

29.2.1.1.3.2.57 protein.synthesis.ribosomal protein.prokaryotic.unknown organellar.50S subunit.L5/L7

29.2.1.1.3.2.9 protein.synthesis.ribosomal protein.prokaryotic.unknown organellar.50S subunit.L9

29.2.1.1.3.2.99 protein.synthesis.ribosomal protein.prokaryotic.unknown organellar.50S subunit.unknown

29.2.1.1.3.99 protein.synthesis.ribosomal protein.prokaryotic.unknown organellar.unknown

29.2.1.1.4 protein.synthesis.ribosomal protein.prokaryotic.non-organellar

29.2.1.1.4.1 protein.synthesis.ribosomal protein.prokaryotic.non-organellar.30S subunit

29.2.1.1.4.2 protein.synthesis.ribosomal protein.prokaryotic.non-organellar.50S subunit

29.2.1.2 protein.synthesis.ribosomal protein.eukaryotic

29.2.1.2.1 protein.synthesis.ribosomal protein.eukaryotic.40S subunit

29.2.1.2.1.10 protein.synthesis.ribosomal protein.eukaryotic.40S subunit.S10

29.2.1.2.1.11 protein.synthesis.ribosomal protein.eukaryotic.40S subunit.S11

29.2.1.2.1.12 protein.synthesis.ribosomal protein.eukaryotic.40S subunit.S12

29.2.1.2.1.13 protein.synthesis.ribosomal protein.eukaryotic.40S subunit.S13

29.2.1.2.1.14 protein.synthesis.ribosomal protein.eukaryotic.40S subunit.S14

29.2.1.2.1.15 protein.synthesis.ribosomal protein.eukaryotic.40S subunit.S15

29.2.1.2.1.16 protein.synthesis.ribosomal protein.eukaryotic.40S subunit.S16

29.2.1.2.1.17 protein.synthesis.ribosomal protein.eukaryotic.40S subunit.S17

29.2.1.2.1.18 protein.synthesis.ribosomal protein.eukaryotic.40S subunit.S18

29.2.1.2.1.19 protein.synthesis.ribosomal protein.eukaryotic.40S subunit.S19

29.2.1.2.1.2 protein.synthesis.ribosomal protein.eukaryotic.40S subunit.S2

29.2.1.2.1.20 protein.synthesis.ribosomal protein.eukaryotic.40S subunit.S20

29.2.1.2.1.21 protein.synthesis.ribosomal protein.eukaryotic.40S subunit.S21

29.2.1.2.1.22 protein.synthesis.ribosomal protein.eukaryotic.40S subunit.S22

29.2.1.2.1.23 protein.synthesis.ribosomal protein.eukaryotic.40S subunit.S23

29.2.1.2.1.24 protein.synthesis.ribosomal protein.eukaryotic.40S subunit.S24

29.2.1.2.1.25 protein.synthesis.ribosomal protein.eukaryotic.40S subunit.S25

29.2.1.2.1.26 protein.synthesis.ribosomal protein.eukaryotic.40S subunit.S26

29.2.1.2.1.27 protein.synthesis.ribosomal protein.eukaryotic.40S subunit.S27

29.2.1.2.1.28 protein.synthesis.ribosomal protein.eukaryotic.40S subunit.S28

29.2.1.2.1.29 protein.synthesis.ribosomal protein.eukaryotic.40S subunit.S29

29.2.1.2.1.3 protein.synthesis.ribosomal protein.eukaryotic.40S subunit.S3

29.2.1.2.1.30 protein.synthesis.ribosomal protein.eukaryotic.40S subunit.S30

29.2.1.2.1.31 protein.synthesis.ribosomal protein.eukaryotic.40S subunit.SA

29.2.1.2.1.4 protein.synthesis.ribosomal protein.eukaryotic.40S subunit.S4

29.2.1.2.1.5 protein.synthesis.ribosomal protein.eukaryotic.40S subunit.S5

29.2.1.2.1.515 protein.synthesis.ribosomal protein.eukaryotic.40S subunit.S15A

29.2.1.2.1.53 protein.synthesis.ribosomal protein.eukaryotic.40S subunit.S3A

29.2.1.2.1.6 protein.synthesis.ribosomal protein.eukaryotic.40S subunit.S6

29.2.1.2.1.7 protein.synthesis.ribosomal protein.eukaryotic.40S subunit.S7

29.2.1.2.1.8 protein.synthesis.ribosomal protein.eukaryotic.40S subunit.S8

29.2.1.2.1.9 protein.synthesis.ribosomal protein.eukaryotic.40S subunit.S9

29.2.1.2.2 protein.synthesis.ribosomal protein.eukaryotic.60S subunit

29.2.1.2.2.1 protein.synthesis.ribosomal protein.eukaryotic.60S subunit.L1

29.2.1.2.2.10 protein.synthesis.ribosomal protein.eukaryotic.60S subunit.L10

29.2.1.2.2.11 protein.synthesis.ribosomal protein.eukaryotic.60S subunit.L11

29.2.1.2.2.12 protein.synthesis.ribosomal protein.eukaryotic.60S subunit.L12

29.2.1.2.2.13 protein.synthesis.ribosomal protein.eukaryotic.60S subunit.L13

29.2.1.2.2.14 protein.synthesis.ribosomal protein.eukaryotic.60S subunit.L14

29.2.1.2.2.141 protein.synthesis.ribosomal protein.eukaryotic.60S subunit.L4/L1

29.2.1.2.2.15 protein.synthesis.ribosomal protein.eukaryotic.60S subunit.L15

29.2.1.2.2.16 protein.synthesis.ribosomal protein.eukaryotic.60S subunit.L16

29.2.1.2.2.17 protein.synthesis.ribosomal protein.eukaryotic.60S subunit.L17

29.2.1.2.2.1730 protein.synthesis.ribosomal protein.eukaryotic.60S subunit.L7/L30/S12

29.2.1.2.2.18 protein.synthesis.ribosomal protein.eukaryotic.60S subunit.L18

29.2.1.2.2.19 protein.synthesis.ribosomal protein.eukaryotic.60S subunit.L19

29.2.1.2.2.2 protein.synthesis.ribosomal protein.eukaryotic.60S subunit.L2

29.2.1.2.2.21 protein.synthesis.ribosomal protein.eukaryotic.60S subunit.L21

29.2.1.2.2.22 protein.synthesis.ribosomal protein.eukaryotic.60S subunit.L22

29.2.1.2.2.23 protein.synthesis.ribosomal protein.eukaryotic.60S subunit.L23

29.2.1.2.2.24 protein.synthesis.ribosomal protein.eukaryotic.60S subunit.L24

29.2.1.2.2.25 protein.synthesis.ribosomal protein.eukaryotic.60S subunit.L25

29.2.1.2.2.26 protein.synthesis.ribosomal protein.eukaryotic.60S subunit.L26

29.2.1.2.2.27 protein.synthesis.ribosomal protein.eukaryotic.60S subunit.L27

29.2.1.2.2.28 protein.synthesis.ribosomal protein.eukaryotic.60S subunit.L28

29.2.1.2.2.29 protein.synthesis.ribosomal protein.eukaryotic.60S subunit.L29

29.2.1.2.2.3 protein.synthesis.ribosomal protein.eukaryotic.60S subunit.L3

29.2.1.2.2.30 protein.synthesis.ribosomal protein.eukaryotic.60S subunit.L30

29.2.1.2.2.31 protein.synthesis.ribosomal protein.eukaryotic.60S subunit.L31

29.2.1.2.2.32 protein.synthesis.ribosomal protein.eukaryotic.60S subunit.L32

29.2.1.2.2.34 protein.synthesis.ribosomal protein.eukaryotic.60S subunit.L34

29.2.1.2.2.35 protein.synthesis.ribosomal protein.eukaryotic.60S subunit.L35

29.2.1.2.2.36 protein.synthesis.ribosomal protein.eukaryotic.60S subunit.L36

29.2.1.2.2.37 protein.synthesis.ribosomal protein.eukaryotic.60S subunit.L37

29.2.1.2.2.38 protein.synthesis.ribosomal protein.eukaryotic.60S subunit.L38

29.2.1.2.2.39 protein.synthesis.ribosomal protein.eukaryotic.60S subunit.L39

29.2.1.2.2.40 protein.synthesis.ribosomal protein.eukaryotic.60S subunit.L40

29.2.1.2.2.41 protein.synthesis.ribosomal protein.eukaryotic.60S subunit.L41

29.2.1.2.2.44 protein.synthesis.ribosomal protein.eukaryotic.60S subunit.L44

29.2.1.2.2.5 protein.synthesis.ribosomal protein.eukaryotic.60S subunit.L5

29.2.1.2.2.510 protein.synthesis.ribosomal protein.eukaryotic.60S subunit.L10A

29.2.1.2.2.513 protein.synthesis.ribosomal protein.eukaryotic.60S subunit.L13A

29.2.1.2.2.518 protein.synthesis.ribosomal protein.eukaryotic.60S subunit.L18A

29.2.1.2.2.523 protein.synthesis.ribosomal protein.eukaryotic.60S subunit.L23A

29.2.1.2.2.527 protein.synthesis.ribosomal protein.eukaryotic.60S subunit.L27A

29.2.1.2.2.535 protein.synthesis.ribosomal protein.eukaryotic.60S subunit.L35A

29.2.1.2.2.536 protein.synthesis.ribosomal protein.eukaryotic.60S subunit.L36A

29.2.1.2.2.537 protein.synthesis.ribosomal protein.eukaryotic.60S subunit.L37A

29.2.1.2.2.57 protein.synthesis.ribosomal protein.eukaryotic.60S subunit.L7A

29.2.1.2.2.571 protein.synthesis.ribosomal protein.eukaryotic.60S subunit.L7A/NHP2/SNU13

29.2.1.2.2.6 protein.synthesis.ribosomal protein.eukaryotic.60S subunit.L6

29.2.1.2.2.7 protein.synthesis.ribosomal protein.eukaryotic.60S subunit.L7

29.2.1.2.2.8 protein.synthesis.ribosomal protein.eukaryotic.60S subunit.L8

29.2.1.2.2.80 protein.synthesis.ribosomal protein.eukaryotic.60S subunit.P0

29.2.1.2.2.81 protein.synthesis.ribosomal protein.eukaryotic.60S subunit.P1

29.2.1.2.2.82 protein.synthesis.ribosomal protein.eukaryotic.60S subunit.P2

29.2.1.2.2.83 protein.synthesis.ribosomal protein.eukaryotic.60S subunit.P3

29.2.1.2.2.9 protein.synthesis.ribosomal protein.eukaryotic.60S subunit.L9

29.2.1.2.2.99 protein.synthesis.ribosomal protein.eukaryotic.60S subunit.unknown

29.2.1.99 protein.synthesis.ribosomal protein.unknown

29.2.1.99.1 protein.synthesis.ribosomal protein.unknown.small subunit

29.2.1.99.1.1 protein.synthesis.ribosomal protein.unknown.small subunit.S1

29.2.1.99.1.18 protein.synthesis.ribosomal protein.unknown.small subunit.S18

29.2.1.99.1.19 protein.synthesis.ribosomal protein.unknown.small subunit.S19

29.2.1.99.1.2 protein.synthesis.ribosomal protein.unknown.small subunit.S2

29.2.1.99.1.205 protein.synthesis.ribosomal protein.unknown.small subunit.S2/S5

29.2.1.99.1.21 protein.synthesis.ribosomal protein.unknown.small subunit.S21

29.2.1.99.1.25 protein.synthesis.ribosomal protein.unknown.small subunit.S25

29.2.1.99.1.4 protein.synthesis.ribosomal protein.unknown.small subunit.S4

29.2.1.99.1.5 protein.synthesis.ribosomal protein.unknown.small subunit.S5

29.2.1.99.1.6 protein.synthesis.ribosomal protein.unknown.small subunit.S6

29.2.1.99.1.7 protein.synthesis.ribosomal protein.unknown.small subunit.S7

29.2.1.99.1.9 protein.synthesis.ribosomal protein.unknown.small subunit.S9

29.2.1.99.2 protein.synthesis.ribosomal protein.unknown.large subunit

29.2.1.99.2.1 protein.synthesis.ribosomal protein.unknown.large subunit.L1

29.2.1.99.2.1185 protein.synthesis.ribosomal protein.unknown.large subunit.L18/L5

29.2.1.99.2.16 protein.synthesis.ribosomal protein.unknown.large subunit.L16

29.2.1.99.2.18 protein.synthesis.ribosomal protein.unknown.large subunit.L18

29.2.1.99.2.19 protein.synthesis.ribosomal protein.unknown.large subunit.L19

29.2.1.99.2.2 protein.synthesis.ribosomal protein.unknown.large subunit.L2

29.2.1.99.2.23 protein.synthesis.ribosomal protein.unknown.large subunit.L23

29.2.1.99.2.27 protein.synthesis.ribosomal protein.unknown.large subunit.L27

29.2.1.99.2.34 protein.synthesis.ribosomal protein.unknown.large subunit.L34

29.2.1.99.2.36 protein.synthesis.ribosomal protein.unknown.large subunit.L36

29.2.1.99.2.39 protein.synthesis.ribosomal protein.unknown.large subunit.L39

29.2.1.99.2.4 protein.synthesis.ribosomal protein.unknown.large subunit.L4

29.2.1.99.2.5 protein.synthesis.ribosomal protein.unknown.large subunit.L5

29.2.1.99.99 protein.synthesis.ribosomal protein.unknown.unknown

29.2.2 protein.synthesis.ribosome biogenesis

29.2.2.1 protein.synthesis.ribosome biogenesis.export from nucleus

29.2.2.2 protein.synthesis.ribosome biogenesis.Assembly factors

29.2.2.2.1 protein.synthesis.ribosome biogenesis.Assembly factors.DExD-box helicases

29.2.2.2.2 protein.synthesis.ribosome biogenesis.Assembly factors.GTPases

29.2.2.2.3 protein.synthesis.ribosome biogenesis.Assembly factors.WD-repeat proteins

29.2.2.2.99 protein.synthesis.ribosome biogenesis.Assembly factors.misc

29.2.2.3 protein.synthesis.ribosome biogenesis.Pre-rRNA processing and modifications

29.2.2.3.1 protein.synthesis.ribosome biogenesis.Pre-rRNA processing and modifications.snoRNPs

29.2.2.3.2 protein.synthesis.ribosome biogenesis.Pre-rRNA processing and modifications.snoRNAs

29.2.2.3.3 protein.synthesis.ribosome biogenesis.Pre-rRNA processing and modifications.methylotransferases

29.2.2.3.4 protein.synthesis.ribosome biogenesis.Pre-rRNA processing and modifications.WD-repeat proteins

29.2.2.3.5 protein.synthesis.ribosome biogenesis.Pre-rRNA processing and modifications.DExD-box helicases

29.2.2.3.99 protein.synthesis.ribosome biogenesis.Pre-rRNA processing and modifications.misc

29.2.2.50 protein.synthesis.ribosome biogenesis.BRIX

29.2.2.99 protein.synthesis.ribosome biogenesis.misc

29.2.3 protein.synthesis.initiation

29.2.3.1 protein.synthesis.initiation.deoxyhypusine synthase

29.2.4 protein.synthesis.elongation

29.2.4.1 protein.synthesis.elongation.deoxyhypusine synthase

29.2.5 protein.synthesis.release

29.2.6 protein.synthesis.ribosomal RNA

29.2.7 protein.synthesis.transfer RNA

29.2.7.1 protein.synthesis.transfer RNA.nucleus

29.2.7.1.1 protein.synthesis.transfer RNA.nucleus.tRNA-Ala

29.2.7.1.10 protein.synthesis.transfer RNA.nucleus.tRNA-Ile

29.2.7.1.11 protein.synthesis.transfer RNA.nucleus.tRNA-Leu

29.2.7.1.12 protein.synthesis.transfer RNA.nucleus.tRNA-Lys

29.2.7.1.13 protein.synthesis.transfer RNA.nucleus.tRNA-Met

29.2.7.1.14 protein.synthesis.transfer RNA.nucleus.tRNA-Phe

29.2.7.1.15 protein.synthesis.transfer RNA.nucleus.tRNA-Pro

29.2.7.1.16 protein.synthesis.transfer RNA.nucleus.tRNA-Ser

29.2.7.1.17 protein.synthesis.transfer RNA.nucleus.tRNA-Thr

29.2.7.1.18 protein.synthesis.transfer RNA.nucleus.tRNA-Trp

29.2.7.1.19 protein.synthesis.transfer RNA.nucleus.tRNA-Tyr

29.2.7.1.2 protein.synthesis.transfer RNA.nucleus.tRNA-Arg

29.2.7.1.20 protein.synthesis.transfer RNA.nucleus.tRNA-Val

29.2.7.1.3 protein.synthesis.transfer RNA.nucleus.tRNA-Asn

29.2.7.1.4 protein.synthesis.transfer RNA.nucleus.tRNA-Asp

29.2.7.1.5 protein.synthesis.transfer RNA.nucleus.tRNA-Cys

29.2.7.1.6 protein.synthesis.transfer RNA.nucleus.tRNA-Glu

29.2.7.1.7 protein.synthesis.transfer RNA.nucleus.tRNA-Gln

29.2.7.1.8 protein.synthesis.transfer RNA.nucleus.tRNA-Gly

29.2.7.1.9 protein.synthesis.transfer RNA.nucleus.tRNA-His

29.2.7.2 protein.synthesis.transfer RNA.plastid

29.2.7.2.1 protein.synthesis.transfer RNA.plastid.tRNA-Ala

29.2.7.2.10 protein.synthesis.transfer RNA.plastid.tRNA-Ile

29.2.7.2.11 protein.synthesis.transfer RNA.plastid.tRNA-Leu

29.2.7.2.12 protein.synthesis.transfer RNA.plastid.tRNA-Lys

29.2.7.2.13 protein.synthesis.transfer RNA.plastid.tRNA-Met

29.2.7.2.14 protein.synthesis.transfer RNA.plastid.tRNA-Phe

29.2.7.2.15 protein.synthesis.transfer RNA.plastid.tRNA-Pro

29.2.7.2.16 protein.synthesis.transfer RNA.plastid.tRNA-Ser

29.2.7.2.17 protein.synthesis.transfer RNA.plastid.tRNA-Thr

29.2.7.2.18 protein.synthesis.transfer RNA.plastid.tRNA-Trp

29.2.7.2.19 protein.synthesis.transfer RNA.plastid.tRNA-Tyr

29.2.7.2.2 protein.synthesis.transfer RNA.plastid.tRNA-Arg

29.2.7.2.20 protein.synthesis.transfer RNA.plastid.tRNA-Val

29.2.7.2.3 protein.synthesis.transfer RNA.plastid.tRNA-Asn

29.2.7.2.4 protein.synthesis.transfer RNA.plastid.tRNA-Asp

29.2.7.2.5 protein.synthesis.transfer RNA.plastid.tRNA-Cys

29.2.7.2.6 protein.synthesis.transfer RNA.plastid.tRNA-Glu

29.2.7.2.7 protein.synthesis.transfer RNA.plastid.tRNA-Gln

29.2.7.2.8 protein.synthesis.transfer RNA.plastid.tRNA-Gly

29.2.7.2.9 protein.synthesis.transfer RNA.plastid.tRNA-His

29.2.7.3 protein.synthesis.transfer RNA.mitochondrion

29.2.7.3.10 protein.synthesis.transfer RNA.mitochondrion.tRNA-Ile

29.2.7.3.12 protein.synthesis.transfer RNA.mitochondrion.tRNA-Lys

29.2.7.3.13 protein.synthesis.transfer RNA.mitochondrion.tRNA-Met

29.2.7.3.15 protein.synthesis.transfer RNA.mitochondrion.tRNA-Pro

29.2.7.3.16 protein.synthesis.transfer RNA.mitochondrion.tRNA-Ser

29.2.7.3.18 protein.synthesis.transfer RNA.mitochondrion.tRNA-Trp

29.2.7.3.19 protein.synthesis.transfer RNA.mitochondrion.tRNA-Tyr

29.2.7.3.3 protein.synthesis.transfer RNA.mitochondrion.tRNA-Asn

29.2.7.3.4 protein.synthesis.transfer RNA.mitochondrion.tRNA-Asp

29.2.7.3.5 protein.synthesis.transfer RNA.mitochondrion.tRNA-Cys

29.2.7.3.6 protein.synthesis.transfer RNA.mitochondrion.tRNA-Glu

29.2.7.3.7 protein.synthesis.transfer RNA.mitochondrion.tRNA-Gln

29.2.7.3.8 protein.synthesis.transfer RNA.mitochondrion.tRNA-Gly

29.2.99 protein.synthesis.misc

29.3 protein.targeting

29.3.1 protein.targeting.nucleus

29.3.2 protein.targeting.mitochondria

29.3.3 protein.targeting.chloroplast

29.3.4 protein.targeting.secretory pathway

29.3.4.1 protein.targeting.secretory pathway.ER

29.3.4.2 protein.targeting.secretory pathway.golgi

29.3.4.3 protein.targeting.secretory pathway.vacuole

29.3.4.4 protein.targeting.secretory pathway.plasma membrane

29.3.4.99 protein.targeting.secretory pathway.unspecified

29.3.5 protein.targeting.peroxisomes

29.3.99 protein.targeting.unknown

29.4 protein.postranslational modification

29.4.1 protein.postranslational modification.kinase

29.4.1.51 protein.postranslational modification.kinase.receptor like cytoplasmatic kinase I

29.4.1.52 protein.postranslational modification.kinase.receptor like cytoplasmatic kinase II

29.4.1.53 protein.postranslational modification.kinase.receptor like cytoplasmatic kinase III

29.4.1.54 protein.postranslational modification.kinase.receptor like cytoplasmatic kinase IV

29.4.1.55 protein.postranslational modification.kinase.receptor like cytoplasmatic kinase V

29.4.1.56 protein.postranslational modification.kinase.receptor like cytoplasmatic kinase VI

29.4.1.57 protein.postranslational modification.kinase.receptor like cytoplasmatic kinase VII

29.4.1.58 protein.postranslational modification.kinase.receptor like cytoplasmatic kinase VIII

29.4.1.59 protein.postranslational modification.kinase.receptor like cytoplasmatic kinase IX

29.4.1.60 protein.postranslational modification.kinase.receptor like cytoplasmatic kinase X

29.4.1.61 protein.postranslational modification.kinase.receptor like cytoplasmatic kinase X

29.5 protein.degradation

29.5.1 protein.degradation.subtilases

29.5.11 protein.degradation.ubiquitin

29.5.11.1 protein.degradation.ubiquitin.ubiquitin

29.5.11.2 protein.degradation.ubiquitin.E1

29.5.11.20 protein.degradation.ubiquitin.proteasom

29.5.11.3 protein.degradation.ubiquitin.E2

29.5.11.4 protein.degradation.ubiquitin.E3

29.5.11.4.1 protein.degradation.ubiquitin.E3.HECT

29.5.11.4.2 protein.degradation.ubiquitin.E3.RING

29.5.11.4.3 protein.degradation.ubiquitin.E3.SCF

29.5.11.4.3.1 protein.degradation.ubiquitin.E3.SCF.SKP

29.5.11.4.3.2 protein.degradation.ubiquitin.E3.SCF.FBOX

29.5.11.4.3.3 protein.degradation.ubiquitin.E3.SCF.cullin

29.5.11.4.3.4 protein.degradation.ubiquitin.E3.SCF.RBX

29.5.11.4.4 protein.degradation.ubiquitin.E3.APC

29.5.11.4.5 protein.degradation.ubiquitin.E3.BTB/POZ Cullin3

29.5.11.4.5.1 protein.degradation.ubiquitin.E3.BTB/POZ Cullin3.Cullin3

29.5.11.4.5.2 protein.degradation.ubiquitin.E3.BTB/POZ Cullin3.BTB/POZ

29.5.11.4.6 protein.degradation.ubiquitin.E3.DCX

29.5.11.4.99 protein.degradation.ubiquitin.E3.unspecified

29.5.11.5 protein.degradation.ubiquitin.ubiquitin protease

29.5.2 protein.degradation.autophagy

29.5.3 protein.degradation.cysteine protease

29.5.30 protein.degradation.prolyl protease

29.5.31 protein.degradation.alanine protease

29.5.4 protein.degradation.aspartate protease

29.5.5 protein.degradation.serine protease

29.5.7 protein.degradation.metalloprotease

29.5.9 protein.degradation.AAA type

29.6 protein.folding

29.6.1 protein.folding.prefoldin and trigger factor

29.6.2 protein.folding.chaperones and co-chaperones

29.6.2.1 protein.folding.chaperones and co-chaperones.small HSPs

29.6.2.1.1 protein.folding.chaperones and co-chaperones.small HSPs.chaperones

29.6.2.1.2 protein.folding.chaperones and co-chaperones.small HSPs.co-chaperones

29.6.2.2 protein.folding.chaperones and co-chaperones.HSP60s

29.6.2.2.1 protein.folding.chaperones and co-chaperones.HSP60s.cytosolic

29.6.2.2.1.1 protein.folding.chaperones and co-chaperones.HSP60s.cytosolic.chaperones

29.6.2.2.1.2 protein.folding.chaperones and co-chaperones.HSP60s.cytosolic.co-chaperones

29.6.2.2.2 protein.folding.chaperones and co-chaperones.HSP60s.organelle

29.6.2.2.2.1 protein.folding.chaperones and co-chaperones.HSP60s.organelle.chaperones

29.6.2.2.2.2 protein.folding.chaperones and co-chaperones.HSP60s.organelle.co-chaperones

29.6.2.3 protein.folding.chaperones and co-chaperones.HSP70s

29.6.2.3.1 protein.folding.chaperones and co-chaperones.HSP70s.chaperones

29.6.2.3.2 protein.folding.chaperones and co-chaperones.HSP70s.co-chaperones

29.6.2.3.2.1 protein.folding.chaperones and co-chaperones.HSP70s.co-chaperones.nucleotide exchange factor

29.6.2.3.2.2 protein.folding.chaperones and co-chaperones.HSP70s.co-chaperones.J-domain

29.6.2.4 protein.folding.chaperones and co-chaperones.HSP90s

29.6.2.4.1 protein.folding.chaperones and co-chaperones.HSP90s.chaperones

29.6.2.4.2 protein.folding.chaperones and co-chaperones.HSP90s.co-chaperones

29.6.2.5 protein.folding.chaperones and co-chaperones.HSP100s

29.6.2.5.1 protein.folding.chaperones and co-chaperones.HSP100s.chaperones

29.6.2.5.2 protein.folding.chaperones and co-chaperones.HSP100s.co-chaperones

29.6.3 protein.folding.immunophilins (IMM)

29.6.3.1 protein.folding.immunophilins (IMM).FKBPs

29.6.3.2 protein.folding.immunophilins (IMM).cyclophilins

29.7 protein.glycosylation

29.7.1 protein.glycosylation.mannosyl-oligosaccharide glucosidase I

29.7.10 protein.glycosylation.alpha-1,3-fucosyltransferase(alpha-1,3-FucT)

29.7.11 protein.glycosylation.alpha-N-acetylglucosaminidase

29.7.12 protein.glycosylation.beta-1,3-galactosyltransferase(beta-1,3-GalT)

29.7.13 protein.glycosylation.alpha-1,4-fucosyltransferase(alpha-1,4-FucT)

29.7.2 protein.glycosylation.mannosyl-oligosaccharide glucosidase II

29.7.3 protein.glycosylation.mannosyl-oligosaccharide alpha-1,2-mannosidase

29.7.4 protein.glycosylation.UDP-glucose glycoprotein glucosyltransferase

29.7.5 protein.glycosylation.alpha-1,3-mannosyl-glycoprotein-beta-1,2-N-acetylglucosaminyltransferase(GnTI)

29.7.6 protein.glycosylation.beta-1,4-mannosyl-glucoprotein-beta-1,4-N-acetylglucosaminyltransferase

29.7.7 protein.glycosylation.mannosyl-oligosaccharide-1,3-1,6-alpha-mannosidase(GMII)

29.7.8 protein.glycosylation.alpha-1,6-mannosyl-glycoprotein-beta-1,2-N-acetylglucosaminyltransferase(GnTII)

29.7.9 protein.glycosylation.beta-1,2-xylosyltransferase(beta-1,2-XylT)

29.8 protein.assembly and cofactor ligation

3 minor CHO metabolism

3.1 minor CHO metabolism.raffinose family

3.1.1 minor CHO metabolism.raffinose family.galactinol synthases

3.1.1.1 minor CHO metabolism.raffinose family.galactinol synthases.known

3.1.1.2 minor CHO metabolism.raffinose family.galactinol synthases.putative

3.1.1001 minor CHO metabolism.raffinose family

3.1.1002 minor CHO metabolism.raffinose family

3.1.2 minor CHO metabolism.raffinose family.raffinose synthases

3.1.2.1 minor CHO metabolism.raffinose family.raffinose synthases.known

3.1.2.2 minor CHO metabolism.raffinose family.raffinose synthases.putative

3.1.3 minor CHO metabolism.raffinose family.stachyose synthases

3.2 minor CHO metabolism.trehalose

3.2.1 minor CHO metabolism.trehalose.TPS

3.2.1001 minor CHO metabolism.trehalose

3.2.2 minor CHO metabolism.trehalose.TPP

3.2.3 minor CHO metabolism.trehalose.potential TPS/TPP

3.2.4 minor CHO metabolism.trehalose.trehalase

3.3 minor CHO metabolism.sugar alcohols

3.3.1001 minor CHO metabolism.sugar alcohols

3.3.1002 minor CHO metabolism.sugar alcohols

3.3.1003 minor CHO metabolism.sugar alcohols

3.3.1004 minor CHO metabolism.sugar alcohols

3.3.1005 minor CHO metabolism.sugar alcohols

3.3.1006 minor CHO metabolism.sugar alcohols

3.3.1007 minor CHO metabolism.sugar alcohols

3.3.1008 minor CHO metabolism.sugar alcohols

3.3.1009 minor CHO metabolism.sugar alcohols

3.3.1010 minor CHO metabolism.sugar alcohols

3.3.1011 minor CHO metabolism.sugar alcohols

3.3.1012 minor CHO metabolism.sugar alcohols

3.4 minor CHO metabolism.myo-inositol

3.4.1 minor CHO metabolism.myo-inositol.poly-phosphatases

3.4.1001 minor CHO metabolism.myo-inositol

3.4.1002 minor CHO metabolism.myo-inositol

3.4.1003 minor CHO metabolism.myo-inositol

3.4.1004 minor CHO metabolism.myo-inositol

3.4.2 minor CHO metabolism.myo-inositol.InsP-Kinases

3.4.3 minor CHO metabolism.myo-inositol.InsP Synthases

3.4.4 minor CHO metabolism.myo-inositol.myo inositol oxygenases

3.4.5 minor CHO metabolism.myo-inositol.inositol phosphatase

3.5 minor CHO metabolism.others

3.5.1 minor CHO metabolism.others.Xylose isomerase

3.6 minor CHO metabolism.callose

3.7 minor CHO metabolism.sugar kinases

3.8 minor CHO metabolism.galactose

3.8.1 minor CHO metabolism.galactose.galactokinases

3.8.2 minor CHO metabolism.galactose.alpha-galactosidases

3.8.3 minor CHO metabolism.galactose.galactose-1-phosphate uridyl transferases

3.99 minor CHO metabolism.misc

3.99.1001 minor CHO metabolism.misc

3.99.1002 minor CHO metabolism.misc

3.99.1003 minor CHO metabolism.misc

3.99.1004 minor CHO metabolism.misc

3.99.1005 minor CHO metabolism.misc

3.99.1006 minor CHO metabolism.misc

3.99.1007 minor CHO metabolism.misc

3.99.1008 minor CHO metabolism.misc

3.99.1009 minor CHO metabolism.misc

3.99.1010 minor CHO metabolism.misc

3.99.1011 minor CHO metabolism.misc

3.99.1012 minor CHO metabolism.misc

3.99.1013 minor CHO metabolism.misc

3.99.1014 minor CHO metabolism.misc

3.99.1015 minor CHO metabolism.misc

3.99.1016 minor CHO metabolism.misc

3.99.1017 minor CHO metabolism.misc

3.99.1018 minor CHO metabolism.misc

3.99.1019 minor CHO metabolism.misc

3.99.1020 minor CHO metabolism.misc

3.99.1021 minor CHO metabolism.misc

3.99.1022 minor CHO metabolism.misc

3.99.1023 minor CHO metabolism.misc

3.99.1024 minor CHO metabolism.misc

3.99.1025 minor CHO metabolism.misc

3.99.1026 minor CHO metabolism.misc

3.99.1027 minor CHO metabolism.misc.Laminaribiose

30 signalling

30.1 signalling.in sugar and nutrient physiology

30.1.1 signalling.in sugar and nutrient physiology

30.1.2 signalling.in sugar and nutrient physiology.pyruvate dehydrogenase kinase

30.10 signalling.phosphorelay

30.1001 signaling

30.1002 signaling

30.1003 signaling

30.1004 signaling

30.1005 signaling

30.1006 signaling

30.1007 signaling

30.1008 signaling

30.1009 signaling

30.11 signalling.light

30.11.1 signalling.light.COP9 signalosome

30.12 signalling.gravity

30.13 signalling.stress

30.13.1 signalling.stress.biotic

30.13.2 signalling.stress.abiotic

30.14 signalling.cyclic nucleotides

30.2 signalling.receptor kinases

30.2.1 signalling.receptor kinases.leucine rich repeat I

30.2.10 signalling.receptor kinases.leucine rich repeat X

30.2.11 signalling.receptor kinases.leucine rich repeat XI

30.2.12 signalling.receptor kinases.leucine rich repeat XII

30.2.13 signalling.receptor kinases.leucine rich repeat XIII

30.2.14 signalling.receptor kinases.leucine rich repeat XIV

30.2.15 signalling.receptor kinases.thaumatin like

30.2.16 signalling.receptor kinases.Catharanthus roseus-like RLK1

30.2.17 signalling.receptor kinases.DUF 26

30.2.18 signalling.receptor kinases.extensin

30.2.19 signalling.receptor kinases.legume-lectin

30.2.2 signalling.receptor kinases.leucine rich repeat II

30.2.20 signalling.receptor kinases.wheat LRK10 like

30.2.21 signalling.receptor kinases.lysine motif

30.2.22 signalling.receptor kinases.proline extensin like

30.2.23 signalling.receptor kinases.RKF3 like

30.2.24 signalling.receptor kinases.S-locus glycoprotein like

30.2.25 signalling.receptor kinases.wall associated kinase

30.2.26 signalling.receptor kinases.crinkly like

30.2.27 signalling.receptor kinases.C-Lectin

30.2.3 signalling.receptor kinases.leucine rich repeat III

30.2.4 signalling.receptor kinases.leucine rich repeat IV

30.2.5 signalling.receptor kinases.leucine rich repeat V

30.2.6 signalling.receptor kinases.leucine rich repeat VI

30.2.7 signalling.receptor kinases.leucine rich repeat VII

30.2.8 signalling.receptor kinases.leucine rich repeat VIII

30.2.8.1 signalling.receptor kinases.leucine rich repeat VIII.VIII-1

30.2.8.2 signalling.receptor kinases.leucine rich repeat VIII.VIII-2

30.2.9 signalling.receptor kinases.leucine rich repeat IX

30.2.99 signalling.receptor kinases.misc

30.3 signalling.calcium

30.4 signalling.phosphinositides

30.4.1 signalling.phosphinositides.phosphatidylinositol-4-phosphate 5-kinase

30.4.2 signalling.phosphinositides.phosphatidylinositol 4-kinase

30.4.3 signalling.phosphinositides.bis(5''-nucleosyl)-tetraphosphatase

30.4.4 signalling.phosphinositides.phosphoinositide phospholipase C

30.4.5 signalling.phosphinositides.inositol-1,3,4-trisphosphate 5/6-kinase

30.4.6 signaling.phosphinositides.phosphatidylinositol-4,5-bisphosphate 3-kinase

30.4.7 signaling.phosphinositides.phosphatidylinositol-4-phosphate 3-kinase

30.4.8 signaling.phosphinositides.phosphatidylinositol phosphatidylinositol 3-kinase

30.5 signalling.G-proteins

30.6 signalling.MAP kinases

30.7 signalling.14-3-3 proteins

30.8 signalling.misc

30.9 signalling.lipids

30.99 signalling.unspecified

31 cell

31.1 cell.organisation

31.2 cell.division

31.2.5 cell.division.plastid

31.3 cell.cycle

31.3.1 cell.cycle.peptidylprolyl isomerase

31.4 cell.vesicle transport

31.5 cell.cell death

31.5.1 cell.cell death.plants

31.5.2 cell.cell death.apoptosis

31.6 cell.motility

31.6.1 cell.motility.eukaryotes

31.6.1.1 cell.motility.eukaryotes.basal bodies

31.6.1.10 cell.motility.eukaryotes.flagellar associated proteins

31.6.1.11 cell.motility.eukaryotes.other

31.6.1.2 cell.motility.eukaryotes.deflagellation

31.6.1.3 cell.motility.eukaryotes.intraflagellar transport

31.6.1.3.1 cell.motility.eukaryotes.intraflagellar transport.IFT motor protein

31.6.1.3.1.1 cell.motility.eukaryotes.intraflagellar transport.IFT motor protein.kinesin-2 subunits

31.6.1.3.1.2 cell.motility.eukaryotes.intraflagellar transport.IFT motor protein.cytoplasmic dynein 1b subunits

31.6.1.3.2 cell.motility.eukaryotes.intraflagellar transport.IFT particle protein

31.6.1.3.2.1 cell.motility.eukaryotes.intraflagellar transport.IFT particle protein.complex A

31.6.1.3.2.2 cell.motility.eukaryotes.intraflagellar transport.IFT particle protein.complex B

31.6.1.4 cell.motility.eukaryotes.axonemal dyneins

31.6.1.4.1 cell.motility.eukaryotes.axonemal dyneins.outer arm

31.6.1.4.2 cell.motility.eukaryotes.axonemal dyneins.inner arm

31.6.1.4.2.1 cell.motility.eukaryotes.axonemal dyneins.inner arm.monomeric species

31.6.1.5 cell.motility.eukaryotes.radial spoke

31.6.1.5.1 cell.motility.eukaryotes.radial spoke.head

31.6.1.5.2 cell.motility.eukaryotes.radial spoke.stalk

31.6.1.6 cell.motility.eukaryotes.central pair

31.6.1.6.1 cell.motility.eukaryotes.central pair.C1a

31.6.1.6.2 cell.motility.eukaryotes.central pair.C1b

31.6.1.6.3 cell.motility.eukaryotes.central pair.C1

31.6.1.6.4 cell.motility.eukaryotes.central pair.C2b

31.6.1.6.5 cell.motility.eukaryotes.central pair.C2c

31.6.1.6.6 cell.motility.eukaryotes.central pair.C1-C2 bridge

31.6.1.7 cell.motility.eukaryotes.dynein regulatory complex (DRC)

31.6.1.8 cell.motility.eukaryotes.flagellar membrane proteins

31.6.1.9 cell.motility.eukaryotes.flagellar adhesion and gamete fusion

31.6.2 cell.motility.prokaryotes

31.7 cell.development

31.7.1 cell.development.zygote

31.8 cell.contractile vacuole

31.9 cell.eyespot

31.99 cell.unspecified

32 micro RNA, natural antisense etc

33 development

33.1 development.storage proteins

33.2 development.late embryogenesis abundant

33.3 development.squamosa promoter binding like (SPL)

33.30 development.multitarget

33.30.1 development.multitarget.target of rapamycin

33.50 development.inhibitor proteins

33.99 development.unspecified

34 transport

34.1 transport.p- and v-ATPases

34.1.1 transport.p- and v-ATPases.H+-transporting two-sector ATPase

34.1.1.1 transport.p- and v-ATPases.H+-transporting two-sector ATPase.subunit B

34.1.1.2 transport.p- and v-ATPases.H+-transporting two-sector ATPase.subunit C

34.1.1.3 transport.p- and v-ATPases.H+-transporting two-sector ATPase.subunit D

34.1.1.4 transport.p- and v-ATPases.H+-transporting two-sector ATPase.subunit E

34.1.1.5 transport.p- and v-ATPases.H+-transporting two-sector ATPase.subunit F

34.1.1.6 transport.p- and v-ATPases.H+-transporting two-sector ATPase.subunit H

34.1.2 transport.p- and v-ATPases.H+-exporting ATPase

34.1.3 transport.p- and v-ATPases.inhibitor

34.10 transport.nucleotides

34.11 transport.NDP-sugars at the ER

34.12 transport.metal

34.13 transport.peptides and oligopeptides

34.14 transport.unspecified cations

34.15 transport.potassium

34.16 transport.ABC transporters and multidrug resistance systems

34.17 transport.peroxisomes

34.18 transport.unspecified anions

34.18.1 transport.unspecified anions.arsenite-transporting ATPase

34.19 transport.Major Intrinsic Proteins

34.19.1 transport.Major Intrinsic Proteins.PIP

34.19.2 transport.Major Intrinsic Proteins.TIP

34.19.3 transport.Major Intrinsic Proteins.NIP

34.19.4 transport.Major Intrinsic Proteins.SIP

34.19.99 transport.Major Intrinsic Proteins.unspecified

34.2 transport.sugars

34.2.1 transport.sugars.sucrose

34.20 transport.porins

34.21 transport.calcium

34.22 transport.cyclic nucleotide or calcium regulated channels

34.23 transport.hormones

34.23.1 transport.hormones.auxin

34.24 transport.nitrite

34.3 transport.amino acids

34.30 transport.H+ transporting pyrophosphatase

34.4 transport.nitrate

34.5 transport.ammonium

34.6 transport.sulphate

34.7 transport.phosphate

34.8 transport.metabolite transporters at the envelope membrane

34.9 transport.metabolite transporters at the mitochondrial membrane

34.98 transport.membrane system unknown

34.99 transport.misc

35 not assigned

35.1 not assigned.no ontology

35.1.1 not assigned.no ontology.ABC1 family protein

35.1.10 not assigned.no ontology.Dof-type zinc finger domain-containing protein

35.1.1001 not assigned.no ontology

35.1.1002 not assigned.no ontology

35.1.1003 not assigned.no ontology

35.1.1004 not assigned.no ontology

35.1.1005 not assigned.no ontology

35.1.1006 not assigned.no ontology

35.1.1007 not assigned.no ontology

35.1.1008 not assigned.no ontology

35.1.1009 not assigned.no ontology

35.1.1010 not assigned.no ontology

35.1.1011 not assigned.no ontology

35.1.1012 not assigned.no ontology

35.1.1013 not assigned.no ontology

35.1.1014 not assigned.no ontology

35.1.1015 not assigned.no ontology

35.1.1016 not assigned.no ontology

35.1.1017 not assigned.no ontology

35.1.1018 not assigned.no ontology

35.1.1019 not assigned.no ontology

35.1.1020 not assigned.no ontology

35.1.1021 not assigned.no ontology

35.1.1022 not assigned.no ontology

35.1.1023 not assigned.no ontology

35.1.1024 not assigned.no ontology.Kojic acid

35.1.1025 not assigned.no ontology.Hydroxyurea

35.1.11 not assigned.no ontology.double-stranded RNA-binding domain (DsRBD)-containing protein

35.1.12 not assigned.no ontology.pumilio/Puf RNA-binding domain-containing protein

35.1.13 not assigned.no ontology.SET domain-containing protein

35.1.14 not assigned.no ontology.S RNA-binding domain-containing protein

35.1.15 not assigned.no ontology.SAR DNA-binding protein

35.1.16 not assigned.no ontology.speckle-type POZ protein-related

35.1.17 not assigned.no ontology.transcription factor jumonji (jmjC) domain-containing protein

35.1.18 not assigned.no ontology.SNF2 domain-containing protein / helicase domain-containing protein

35.1.19 not assigned.no ontology.C2 domain-containing protein

35.1.2 not assigned.no ontology.agenet domain-containing protein

35.1.20 not assigned.no ontology.formin homology 2 domain-containing protein

35.1.21 not assigned.no ontology.epsin N-terminal homology (ENTH) domain-containing protein

35.1.22 not assigned.no ontology.late embryogenesis abundant domain-containing protein

35.1.23 not assigned.no ontology.aconitase C-terminal domain-containing protein

35.1.24 not assigned.no ontology.paired amphipathic helix repeat-containing protein

35.1.25 not assigned.no ontology.paired amphipathic helix repeat-containing protein

35.1.26 not assigned.no ontology.DC1 domain containing protein

35.1.27 not assigned.no ontology.tetratricopeptide repeat (TPR)

35.1.3 not assigned.no ontology.armadillo/beta-catenin repeat family protein

35.1.40 not assigned.no ontology.glycine rich proteins

35.1.41 not assigned.no ontology.hydroxyproline rich proteins

35.1.42 not assigned.no ontology.proline rich family

35.1.5 not assigned.no ontology.pentatricopeptide (PPR) repeat-containing protein

35.1.6 not assigned.no ontology.Toll-Interleukin-Resistance (TIR) domain-containing protein

35.1.7 not assigned.no ontology.AT hook motif-containing protein

35.1.8 not assigned.no ontology.BSD domain-containing protein

35.1.9 not assigned.no ontology.BTB/POZ domain-containing protein

35.1.999 not assigned.no ontology.formerly annotated based on articles which have been retracted

35.2 not assigned.unknown

35.2.1001 not assigned.unknown

35.3 not assigned.disagreeing hits

4 glycolysis

4.1 glycolysis.cytosolic branch

4.1.1 glycolysis.cytosolic branch.UGPase

4.1.10 glycolysis.cytosolic branch.aldolase

4.1.11 glycolysis.cytosolic branch.3-phosphoglycerate kinase (PGK)

4.1.12 glycolysis.cytosolic branch.phosphoglycerate mutase

4.1.13 glycolysis.cytosolic branch.enolase

4.1.14 glycolysis.cytosolic branch.pyruvate kinase (PK)

4.1.15 glycolysis.cytosolic branch.phospho-enol-pyruvate carboxylase (PEPC)

4.1.16 glycolysis.cytosolic branch.phospho-enol-pyruvate carboxylase kinase (PPCK)

4.1.2 glycolysis.cytosolic branch.phosphoglucomutase (PGM)

4.1.3 glycolysis.cytosolic branch.glucose-6-phosphate isomerase

4.1.4 glycolysis.cytosolic branch.phosphofructokinase (PFK)

4.1.5 glycolysis.cytosolic branch.pyrophosphate-fructose-6-P phosphotransferase

4.1.6 glycolysis.cytosolic branch.fructose-2,6-bisphosphatase (Fru2,6BisPase)

4.1.7 glycolysis.cytosolic branch.triosephosphate isomerase (TPI)

4.1.8 glycolysis.cytosolic branch.glyceraldehyde 3-phosphate dehydrogenase (GAP-DH)

4.1.9 glycolysis.cytosolic branch.non-phosphorylating glyceraldehyde 3-phosphate dehydrogenase (NPGAP-DH)

4.1001 glycolysis

4.1002 glycolysis

4.1003 glycolysis

4.1004 glycolysis

4.1005 glycolysis

4.1006 glycolysis

4.1007 glycolysis

4.2 glycolysis.plastid branch

4.2.1 glycolysis.plastid branch.UGPase

4.2.11 glycolysis.plastid branch.3-phosphoglycerate kinase (PGK)

4.2.12 glycolysis.plastid branch.phosphoglycerate mutase

4.2.13 glycolysis.plastid branch.enolase

4.2.14 glycolysis.plastid branch.pyruvate kinase (PK)

4.2.15 glycolysis.plastid branch.phospho-enol-pyruvate carboxylase (PEPC)

4.2.2 glycolysis.plastid branch.phosphoglucomutase (PGM)

4.2.3 glycolysis.plastid branch.glucose-6-phosphate isomerase

4.2.4 glycolysis.plastid branch.phosphofructokinase (PFK)

4.2.5 glycolysis.plastid branch.pyrophosphate-fructose-6-P phosphotransferase

4.2.6 glycolysis.plastid branch.fructose-2,6-bisphosphatase (Fru2,6BisPase)

4.2.7 glycolysis.plastid branch.triosephosphate isomerase (TPI)

4.2.8 glycolysis.plastid branch.glyceraldehyde 3-phosphate dehydrogenase (GAP-DH)

4.3 glycolysis.unclear/dually targeted

4.3.1 glycolysis.unclear/dually targeted.UGPase

4.3.10 glycolysis.unclear/dually targeted.aldolase

4.3.11 glycolysis.unclear/dually targeted.3-phosphoglycerate kinase (PGK)

4.3.12 glycolysis.unclear/dually targeted.phosphoglycerate mutase

4.3.13 glycolysis.unclear/dually targeted.enolase

4.3.14 glycolysis.unclear/dually targeted.pyruvate kinase (PK)

4.3.15 glycolysis.unclear/dually targeted.phospho-enol-pyruvate carboxylase (PEPC)

4.3.16 glycolysis.unclear/dually targeted.phospho-enol-pyruvate carboxylase kinase (PEPCK)

4.3.2 glycolysis.unclear/dually targeted.phosphoglucomutase (PGM)

4.3.3 glycolysis.unclear/dually targeted.glucose-6-phosphate isomerase

4.3.4 glycolysis.unclear/dually targeted.phosphofructokinase (PFK)

4.3.5 glycolysis.unclear/dually targeted.pyrophosphate-fructose-6-P phosphotransferase

4.3.6 glycolysis.unclear/dually targeted.fructose-2,6-bisphosphatase (Fru2,6BisPase)

4.3.7 glycolysis.unclear/dually targeted.triosephosphate isomerase (TPI)

4.3.8 glycolysis.unclear/dually targeted.glyceraldehyde 3-phosphate dehydrogenase (GAP-DH)

4.3.9 glycolysis.unclear/dually targeted.non-phosphorylating glyceraldehyde 3-phosphate dehydrogenase (NPGAP-DH)

5 fermentation

5.1 fermentation.LDH

5.10 fermentation.aldehyde dehydrogenase

5.1001 fermentation

5.1002 fermentation

5.2 fermentation.PDC

5.3 fermentation.ADH

5.5 fermentation.phosphate acetyltransferase

5.6 fermentation.acetate kinase

5.8 fermentation.pyruvate::ferredoxin oxidoreductase

6 gluconeogenesis / glyoxylate cycle

6.1 gluconeogenesis / glyoxylate cycle.citrate synthase

6.2 gluconeogenesis / glyoxylate cycle.malate synthase

6.3 gluconeogenesis / glyoxylate cycle.Malate DH

6.4 gluconeogenesis / glyoxylate cycle.PEPCK

6.5 gluconeogenesis / glyoxylate cycle.pyruvate dikinase

6.9 gluconeogenesis / glyoxylate cycle.isocitrate lyase

7 OPP

7.1 OPP.oxidative PP

7.1.1 OPP.oxidative PP.G6PD

7.1.1001 OPP.oxidative PP

7.1.2 OPP.oxidative PP.6-phosphogluconolactonase

7.1.3 OPP.oxidative PP.6-phosphogluconate dehydrogenase

7.2 OPP.non-reductive PP

7.2.1 OPP.non-reductive PP.transketolase

7.2.2 OPP.non-reductive PP.transaldolase

7.2.3 OPP.non-reductive PP.ribulose-phosphate 3-epimerase

7.2.4 OPP.non-reductive PP.ribose 5-phosphate isomerase

7.3 OPP.electron transfer

8 TCA / org transformation

8.1 TCA / org transformation.TCA

8.1.1 TCA / org transformation.TCA.pyruvate DH

8.1.1.1 TCA / org transformation.TCA.pyruvate DH.E1

8.1.1.2 TCA / org transformation.TCA.pyruvate DH.E2

8.1.1.3 TCA / org transformation.TCA.pyruvate DH.E3

8.1.1001 TCA / org transformation.TCA

8.1.1002 TCA / org transformation.TCA

8.1.1003 TCA / org transformation.TCA

8.1.1004 TCA / org transformation.TCA

8.1.1005 TCA / org transformation.TCA

8.1.1006 TCA / org transformation.TCA

8.1.1007 TCA / org transformation.TCA

8.1.1008 TCA / org transformation.TCA

8.1.2 TCA / org transformation.TCA.CS

8.1.3 TCA / org transformation.TCA.aconitase

8.1.4 TCA / org transformation.TCA.IDH

8.1.5 TCA / org transformation.TCA.2-oxoglutarate dehydrogenase

8.1.6 TCA / org transformation.TCA.succinyl-CoA ligase

8.1.7 TCA / org transformation.TCA.succinate dehydrogenase

8.1.8 TCA / org transformation.TCA.fumarase

8.1.9 TCA / org transformation.TCA.malate DH

8.1.99 TCA / org transformation.TCA.misc

8.2 TCA / org transformation.other organic acid transformations

8.2.10 TCA / org transformation.other organic acid transformatons.malic

8.2.1001 TCA / org transformation. Various

8.2.1002 TCA / org transformation. Various

8.2.1003 TCA / org transformation. Various

8.2.1004 TCA / org transformation. Various

8.2.1005 TCA / org transformation. Various

8.2.11 TCA / org transformation.other organic acid transformatons.atp-citrate lyase

8.2.3 TCA / org transformation.other organic acid transformatons.aconitase

8.2.4 TCA / org transformation.other organic acid transformatons.IDH

8.2.9 TCA / org transformation.other organic acid transformatons.cyt MDH

8.2.99 TCA / org transformation.other organic acid transformatons.misc

8.3 TCA / org transformation.carbonic anhydrases

9 mitochondrial electron transport / ATP synthesis

9.1 mitochondrial electron transport / ATP synthesis.NADH-DH

9.1.1 mitochondrial electron transport / ATP synthesis.NADH-DH.complex I

9.1.1.5 mitochondrial electron transport / ATP synthesis.NADH-DH.complex I.carbonic anhydrase

9.1.2 mitochondrial electron transport / ATP synthesis.NADH-DH.localisation not clear

9.1001 mitochondrial electron transport / ATP synthesis

9.2 mitochondrial electron transport / ATP synthesis.NADH-DH

9.2.1 mitochondrial electron transport / ATP synthesis.NADH-DH.type II

9.2.1.2 mitochondrial electron transport / ATP synthesis.NADH-DH.type II.external

9.2.1.3 mitochondrial electron transport / ATP synthesis.NADH-DH.type II.mitochondrial

9.2.1.4 mitochondrial electron transport / ATP synthesis.NADH-DH.type II.internal matrix

9.3 mitochondrial electron transport / ATP synthesis.electron transfer flavoprotein

9.4 mitochondrial electron transport / ATP synthesis.alternative oxidase

9.5 mitochondrial electron transport / ATP synthesis.cytochrome c reductase

9.6 mitochondrial electron transport / ATP synthesis.cytochrome c

9.7 mitochondrial electron transport / ATP synthesis.cytochrome c oxidase

9.8 mitochondrial electron transport / ATP synthesis.uncoupling protein

9.9 mitochondrial electron transport / ATP synthesis.F1-ATPase

9.99 mitochondrial electron transport / ATP synthesis.unspecified

991 Mineral Nutrition

991.1 Mineral Nutrition.phosphate

991.1.1001 Mineral Nutrition.phosphate

35.2 not assigned.unknown MDP0000218737 highly similar to ( 541) AT5G50890 | Symbols: | LOCATED IN: cellular_component unknown; EXPRESSED IN: 17 plant structures; EXPRESSED DURING: 13 growth stages; BEST Arabidopsis thaliana protein match is: lipase class 3 family protein (TAIR:AT2G05260.1); Has 115 Blast hits to 115 proteins in 7 species: Archae - 0; Bacteria - 0; Metazoa - 0; Fungi - 0; Plants - 115; Viruses - 0; Other Eukaryotes - 0 (source: NCBI BLink). | chr5:20702684-20704235 FORWARDmoderately similar to ( 465) loc_os01g54810 12001.m11643 protein expressed protein no original description T

29.4 protein.postranslational modification MDP0000722904 highly similar to ( 544) AT1G20930 | Symbols: CDKB2;2 | CDKB2;2 (CYCLIN-DEPENDENT KINASE B2;2); cyclin-dependent protein kinase/ kinase | chr1:7292752-7294664 REVERSEhighly similar to ( 529) CDC2D_ANTMA Cell division control protein 2 homolog D (EC 2.7.11.22) (EC 2.7.11.23) - Antirrhinum majus (Garden snapdragon)moderately similar to ( 496) loc_os08g40170 12008.m07997 protein cell division control protein 2 homolog D, putative, expressed no original description T

27.3.55 RNA.regulation of transcription.HDA MDP0000289428 moderately similar to ( 271) AT1G64980 | Symbols: | unknown protein | chr1:24137840-24138619 REVERSEweakly similar to ( 154) HD2A_MAIZE Histone deacetylase 2a (HD2a) (Zm-HD2a) (Nucleolar histone deacetylase HD2-p39) - Zea mays (Maize)moderately similar to ( 205) loc_os07g23120 12007.m29140 protein expressed protein no original description T

35.2 not assigned.unknown MDP0000310899 weakly similar to ( 175) AT1G72510 | Symbols: | unknown protein | chr1:27303906-27304403 FORWARDweakly similar to ( 104) loc_os07g42220 12007.m08458 protein expressed protein no original description T

33.99 development.unspecified MDP0000674266 moderately similar to ( 204) AT5G39790 | Symbols: | 5'-AMP-activated protein kinase beta-1 subunit-related | chr5:15932884-15935121 REVERSEweakly similar to ( 189) loc_os02g04330 12002.m100032 protein expressed protein no original description T

29.5.4 protein.degradation.aspartate protease MDP0000292917 highly similar to ( 635) AT5G22850 | Symbols: | aspartyl protease family protein | chr5:7633717-7636298 REVERSEweakly similar to ( 112) ASP1_ORYSA Aspartic proteinase Asp1 precursor (EC 3.4.23.-) (OsAsp1) (OSAP1) (Nucellin-like protein) - Oryza sativa (Rice)highly similar to ( 525) loc_os01g56930 12001.m150738 protein aspartic proteinase nepenthesin-2 precursor, putative, expressed no original description T

29.4 protein.postranslational modification MDP0000179084 highly similar to ( 529) AT1G72770 | Symbols: HAB1 | HAB1 (HOMOLOGY TO ABI1); catalytic/ protein serine/threonine phosphatase | chr1:27390998-27392413 FORWARDmoderately similar to ( 462) loc_os01g40094 12001.m10282 protein protein phosphatase 2C ABI2, putative, expressed no original description T

35.2 not assigned.unknown MDP0000580985 very weakly similar to (90.9) AT5G18310 | Symbols: | unknown protein | chr5:6061759-6062912 FORWARD no original description T

31.5.1 cell.cell death.plants MDP0000192636 moderately similar to ( 342) AT1G29690 | Symbols: CAD1 | CAD1 (constitutively activated cell death 1) | chr1:10379310-10381861 REVERSEmoderately similar to ( 315) loc_os01g54510 12001.m11614 protein MAC/Perforin domain containing protein, expressed no original description T

35.2 not assigned.unknown MDP0000229084 weakly similar to ( 168) AT5G59410 | Symbols: | FUNCTIONS IN: molecular_function unknown; INVOLVED IN: biological_process unknown; LOCATED IN: cellular_component unknown; EXPRESSED IN: 22 plant structures; EXPRESSED DURING: 13 growth stages; CONTAINS InterPro DOMAIN/s: Rab5-interacting (InterPro:IPR010742); BEST Arabidopsis thaliana protein match is: Rab5-interacting family protein (TAIR:AT2G29020.1); Has 154 Blast hits to 154 proteins in 67 species: Archae - 0; Bacteria - 0; Metazoa - 108; Fungi - 0; Plants - 26; Viruses - 0; Other Eukaryotes - 20 (source: NCBI BLink). | chr5:23959761-23960730 REVERSEweakly similar to ( 151) loc_os05g01994 12005.m83862 protein RAB5-interacting protein isoform a, putative, expressed no original description T

31.3 cell.cycle MDP0000139550 moderately similar to ( 279) AT2G44740 | Symbols: CYCP4;1 | CYCP4;1 (cyclin p4;1); cyclin-dependent protein kinase | chr2:18442287-18443304 REVERSEmoderately similar to ( 209) loc_os10g41430 12010.m06907 protein nuc-1 negative regulatory protein preg, putative, expressed no original description T

29.5.11.1 protein.degradation.ubiquitin.ubiquitin MDP0000265064 weakly similar to ( 172) AT3G13235 | Symbols: | ubiquitin family protein | chr3:4271492-4274348 REVERSEweakly similar to ( 145) loc_os02g10510 12002.m06349 protein DNA damage-inducible protein DDI1, putative, expressed no original description T

30.4 signalling.phosphinositides MDP0000217790 nearly identical (2146) AT3G59770 | Symbols: SAC9 | SAC9; inositol or phosphatidylinositol phosphatase | chr3:22079281-22084785 REVERSEhighly similar to ( 902) loc_os01g25330 12001.m150579 protein SAC9, putative, expressed no original description T

31.1 cell.organisation MDP0000474142 weakly similar to ( 198) AT2G19770 | Symbols: PRF5 | PRF5 (PROFILIN5); actin binding / actin monomer binding | chr2:8519885-8521119 REVERSEmoderately similar to ( 235) PROF1_MALDO Profilin-1 (GD4-1) (Pollen allergen Mal d 4.0301) - Malus domestica (Apple) (Malus sylvestris)moderately similar to ( 205) loc_os06g05880 12006.m05314 protein profilin-2, putative, expressed no original description T

35.2 not assigned.unknown MDP0000877136 moderately similar to ( 213) AT2G32280 | Symbols: | unknown protein | chr2:13713210-13713977 FORWARDweakly similar to ( 147) loc_os07g36110 12007.m07864 protein expressed protein no original description T

10.8.1 cell wall.pectin*esterases.PME MDP0000184228 moderately similar to ( 470) AT3G29090 | Symbols: PME31, ATPME31 | pectinesterase family protein | chr3:11073804-11075335 FORWARDweakly similar to ( 149) PME_DAUCA Pectinesterase (EC 3.1.1.11) (Pectin methylesterase) (PE) - Daucus carota (Carrot)moderately similar to ( 419) loc_os10g26680 12010.m21883 protein pectinesterase-1 precursor, putative, expressed no original description T

35.2 not assigned.unknown MDP0000882402 moderately similar to ( 453) loc_os05g06510 12005.m083616 protein 4Fe-4S ferredoxin, iron-sulfur binding protein, putative, expressed no original description T

30.11 signalling.light MDP0000258995 moderately similar to ( 375) AT4G01690 | Symbols: PPOX, HEMG1, PPO1 | PPOX; protoporphyrinogen oxidase | chr4:729929-732309 FORWARDmoderately similar to ( 296) PPOC_TOBAC Protoporphyrinogen oxidase, chloroplast precursor (EC 1.3.3.4) (PPO I) (Protoporphyrinogen IX oxidase isozyme I) (PPX I) - Nicotiana tabacum (Common tobacco)moderately similar to ( 500) loc_os01g18320 12001.m08374 protein protoporphyrinogen oxidase, chloroplast precursor, putative, expressed no original description T

27.3.3 RNA.regulation of transcription.AP2/EREBP, APETALA2/Ethylene-responsive element binding protein family MDP0000683814 weakly similar to ( 178) AT1G78080 | Symbols: RAP2.4 | RAP2.4 (related to AP2 4); DNA binding / transcription factor | chr1:29364790-29365794 FORWARDvery weakly similar to (63.5) ERF5_TOBAC Ethylene-responsive transcription factor 5 (Ethylene-responsive element-binding factor 5 homolog) (EREBP-4) (NtERF4) - Nicotiana tabacum (Common tobacco)weakly similar to ( 161) loc_os02g51670 12002.m10198 protein AP2 domain-containing protein, putative, expressed no original description T

27.2 RNA.transcription MDP0000342635 moderately similar to ( 254) AT5G09920 | Symbols: RPB15.9, ATRPB15.9, RPB15.9.9, NRPB4 | NRPB4; DNA-directed RNA polymerase | chr5:3096276-3097370 FORWARDmoderately similar to ( 214) loc_os02g02510 12002.m33507 protein DNA-directed RNA polymerase II 16 kDa polypeptide, putative, expressed no original description T

35.2 not assigned.unknown MDP0000207725 moderately similar to ( 312) AT1G51130 | Symbols: | FUNCTIONS IN: molecular_function unknown; INVOLVED IN: biological_process unknown; LOCATED IN: cellular_component unknown; CONTAINS InterPro DOMAIN/s: Nse4 (InterPro:IPR014854); BEST Arabidopsis thaliana protein match is: unknown protein (TAIR:AT3G20760.1); Has 200 Blast hits to 198 proteins in 89 species: Archae - 0; Bacteria - 0; Metazoa - 63; Fungi - 88; Plants - 37; Viruses - 0; Other Eukaryotes - 12 (source: NCBI BLink). | chr1:18939480-18941560 REVERSEmoderately similar to ( 303) loc_os02g10090 12002.m06306 protein expressed protein no original description T

26.21 misc.protease inhibitor/seed storage/lipid transfer protein (LTP) family protein MDP0000310247 very weakly similar to (85.5) AT5G48485 | Symbols: DIR1 | DIR1 (DEFECTIVE IN INDUCED RESISTANCE 1); lipid binding / lipid transporter | chr5:19646317-19646625 REVERSEvery weakly similar to (94.4) loc_os07g18750 12007.m06314 protein PVR3-like protein, putative, expressed no original description T

11.1.9 lipid metabolism.FA synthesis and FA elongation.long chain fatty acid CoA ligase MDP0000465035 highly similar to ( 610) AT2G47240 | Symbols: LACS1 | long-chain-fatty-acid--CoA ligase family protein / long-chain acyl-CoA synthetase family protein | chr2:19393835-19397616 FORWARDweakly similar to ( 107) 4CL1_ORYSA 4-coumarate--CoA ligase 1 (EC 6.2.1.12) (4CL 1) (4-coumaroyl-CoA synthase 1) - Oryza sativa (Rice)highly similar to ( 569) loc_os05g04170 12005.m083564 protein ACS-like protein, putative, expressed no original description T

27.1 RNA.processing MDP0000293960 nearly identical (1096) AT1G61010 | Symbols: CPSF73-I | CPSF73-I (CLEAVAGE AND POLYADENYLATION SPECIFICITY FACTOR 73-I); protein binding | chr1:22474954-22477660 REVERSEweakly similar to ( 130) CPSF2_ORYSA Cleavage and polyadenylation specificity factor 100 kDa subunit (CPSF 100 kDa subunit) - Oryza sativa (Rice)nearly identical (1038) loc_os03g63590 12003.m11227 protein cleavage and polyadenylation specificity factor, 73 kDa subunit, putative, expressed no original description T

29.4 protein.postranslational modification MDP0000290669 highly similar to ( 576) AT5G67380 | Symbols: CKA1, ATCKA1 | CKA1 (CASEIN KINASE ALPHA 1); kinase | chr5:26881156-26883383 REVERSEhighly similar to ( 560) CSK2A_MAIZE Casein kinase II subunit alpha (EC 2.7.11.1) (CK II) (CK2-alpha) - Zea mays (Maize)highly similar to ( 566) loc_os07g02350 12007.m04707 protein casein kinase II subunit alpha-2, putative, expressed MSP1_C no original description T

28.1.3 DNA.synthesis/chromatin structure.histone MDP0000336462 weakly similar to ( 162) AT5G59970 | Symbols: | histone H4 | chr5:24146352-24146663 REVERSEweakly similar to ( 162) H4_PEA Histone H4 - Pisum sativum (Garden pea)weakly similar to ( 162) loc_os10g39410 12010.m06709 protein histone H4, putative, expressed no original description T

35.1 not assigned.no ontology MDP0000153229 weakly similar to ( 104) AT4G25040 | Symbols: | integral membrane family protein | chr4:12868320-12869319 FORWARDvery weakly similar to (72.0) loc_os12g41690 12012.m07950 protein plant integral membrane protein TIGR01569 containing protein, expressed no original description T

35.1 not assigned.no ontology MDP0000183958 very weakly similar to (86.7) AT3G56880 | Symbols: | VQ motif-containing protein | chr3:21060044-21060781 FORWARDvery weakly similar to (56.6) loc_os01g17050 12001.m42970 protein VQ, putative, expressed no original description T

34.3 transport.amino acids MDP0000208046 highly similar to ( 666) AT1G25530 | Symbols: | lysine and histidine specific transporter, putative | chr1:8964827-8967391 REVERSEhighly similar to ( 562) loc_os08g03350 12008.m04478 protein LHT1, putative, expressed no original description T

1.1.4.7 PS.lightreaction.ATP synthase.delta chain MDP0000750535 moderately similar to ( 244) AT4G09650 | Symbols: ATPD | ATPD (ATP SYNTHASE DELTA-SUBUNIT GENE); hydrogen ion transporting ATP synthase, rotational mechanism / proton-transporting ATPase, rotational mechanism | chr4:6100799-6101503 FORWARDmoderately similar to ( 254) ATPD_TOBAC ATP synthase delta chain, chloroplast precursor (EC 3.6.3.14) - Nicotiana tabacum (Common tobacco)moderately similar to ( 201) loc_os02g51470 12002.m33873 protein ATP synthase delta chain, chloroplast precursor, putative, expressed no original description T

35.1 not assigned.no ontology MDP0000339213 moderately similar to ( 418) AT4G19045 | Symbols: | protein binding | chr4:10438213-10439788 REVERSEmoderately similar to ( 415) loc_os03g38020 12003.m08904 protein mps one binder kinase activator-like 1A, putative, expressed no original description T

31.4 cell.vesicle transport MDP0000036103 moderately similar to ( 215) AT4G35410 | Symbols: | clathrin adaptor complex small chain family protein | chr4:16832572-16833476 FORWARDweakly similar to ( 127) AP2S1_MAIZE AP-2 complex subunit sigma-1 (Clathrin coat assembly protein AP17) (Clathrin coat-associated protein AP17) (Plasma membrane adaptor AP-2 17 kDa protein) (Clathrin assembly protein 2 small chain) - Zea mays (Maize)moderately similar to ( 206) loc_os03g57040 12003.m78897 protein AP-1 complex subunit sigma-2, putative, expressed no original description T

35.2 not assigned.unknown MDP0000132972 weakly similar to ( 137) AT3G25130 | Symbols: | unknown protein | chr3:9152434-9153654 FORWARDvery weakly similar to (64.3) loc_os11g26990 12011.m06619 protein expressed protein no original description T

27.3.6 RNA.regulation of transcription.bHLH,Basic Helix-Loop-Helix family MDP0000371401 moderately similar to ( 353) AT2G27230 | Symbols: LHW | LHW (LONESOME HIGHWAY); protein homodimerization/ transcription activator/ transcription factor | chr2:11650895-11653840 FORWARDmoderately similar to ( 396) loc_os11g06010 12011.m04797 protein helix-loop-helix DNA-binding, putative, expressed no original description T

28.1 DNA.synthesis/chromatin structure MDP0000230833 highly similar to ( 923) AT5G55310 | Symbols: TOP1BETA, TOP1 | TOP1BETA (DNA TOPOISOMERASE 1 BETA); DNA topoisomerase type I | chr5:22430389-22434590 REVERSEhighly similar to ( 758) TOP1_DAUCA DNA topoisomerase 1 (EC 5.99.1.2) (DNA topoisomerase I) - Daucus carota (Carrot)highly similar to ( 892) loc_os08g05840 12008.m04725 protein DNA topoisomerase 1, putative, expressed no original description T

27.3.35 RNA.regulation of transcription.bZIP transcription factor family MDP0000219041 weakly similar to ( 129) AT3G17609 | Symbols: HYH | HYH (HY5-HOMOLOG); DNA binding / transcription factor | chr3:6023971-6024585 FORWARDvery weakly similar to (99.0) loc_os02g10860 12002.m06384 protein transcription factor HY5, putative, expressed no original description T

35.1 not assigned.no ontology MDP0000242101 weakly similar to ( 192) AT5G14030 | Symbols: | translocon-associated protein beta (TRAPB) family protein | chr5:4526878-4528253 FORWARDweakly similar to ( 179) loc_os01g01307 12001.m42517 protein translocon-associated protein beta containing protein, expressed no original description T

29.5.11.4.2 protein.degradation.ubiquitin.E3.RING MDP0000472519 highly similar to ( 671) AT5G42340 | Symbols: | binding / ubiquitin-protein ligase | chr5:16928086-16930367 REVERSEmoderately similar to ( 423) SPL11_ORYSA Spotted leaf protein 11 (Spotted leaf11) (Cell death-related protein SPL11) - Oryza sativa (Rice)highly similar to ( 632) loc_os08g37570 12008.m07743 protein spotted leaf protein 11, putative, expressed no original description T

34.21 transport.calcium MDP0000271818 weakly similar to ( 167) AT3G54900 | Symbols: CXIP1, ATGRXCP | CXIP1 (CAX INTERACTING PROTEIN 1); antiporter/ glutathione disulfide oxidoreductase | chr3:20341850-20342371 REVERSEweakly similar to ( 164) loc_os03g63420 12003.m11210 protein OsGrx_S14 - glutaredoxin subgroup II, expressed no original description T

10.5.3 cell wall.cell wall proteins.LRR MDP0000202743 very weakly similar to (55.8) AT4G13340 | Symbols: | leucine-rich repeat family protein / extensin family protein | chr4:7758610-7760892 FORWARDvery weakly similar to (52.0) loc_os01g07840 12001.m07411 protein transposon protein, putative, CACTA, En/Spm sub-class no original description T

26.1 misc.misc2 MDP0000284599 moderately similar to ( 345) AT3G23600 | Symbols: | dienelactone hydrolase family protein | chr3:8473833-8475655 FORWARDweakly similar to ( 154) E134_MAIZE Endo-1,3;1,4-beta-D-glucanase precursor (EC 3.2.1.-) - Zea mays (Maize)moderately similar to ( 321) loc_os05g33100 12005.m07542 protein endo-1,3;1,4-beta-D-glucanase precursor, putative, expressed no original description T

29.2.1.2.1.24 protein.synthesis.ribosomal protein.eukaryotic.40S subunit.S24 MDP0000360583 moderately similar to ( 225) AT3G04920 | Symbols: | 40S ribosomal protein S24 (RPS24A) | chr3:1360989-1362065 FORWARDmoderately similar to ( 216) loc_os01g52490 12001.m11423 protein 40S ribosomal protein S24, putative, expressed no original description T

26.3 misc.gluco-, galacto- and mannosidases MDP0000297569 moderately similar to ( 362) AT5G42260 | Symbols: BGLU12 | BGLU12 (BETA GLUCOSIDASE 12); catalytic/ cation binding / hydrolase, hydrolyzing O-glycosyl compounds | chr5:16898712-16900235 FORWARDmoderately similar to ( 253) BGLC_MAIZE Beta-glucosidase, chloroplast precursor (EC 3.2.1.21) (Gentiobiase) (Cellobiase) (Beta-D-glucoside glucohydrolase) - Zea mays (Maize)moderately similar to ( 397) loc_os04g39900 12004.m08975 protein non-cyanogenic beta-glucosidase precursor, putative, expressed no original description T

27.3.67 RNA.regulation of transcription.putative transcription regulator MDP0000131485 weakly similar to ( 170) AT2G45850 | Symbols: | DNA-binding family protein | chr2:18871901-18873457 REVERSEweakly similar to ( 167) loc_os10g42230 12010.m50380 protein AT-hook protein 1, putative, expressed no original description T

27.4 RNA.RNA binding MDP0000546021 moderately similar to ( 349) AT3G20890 | Symbols: | RNA binding / nucleic acid binding / nucleotide binding | chr3:7320218-7321054 FORWARDmoderately similar to ( 233) loc_os11g03890 12011.m04584 protein RNA binding protein, putative, expressed no original description T

35.1 not assigned.no ontology MDP0000668523 very weakly similar to (58.2) AT3G07600 | Symbols: | heavy-metal-associated domain-containing protein | chr3:2424300-2424954 REVERSEvery weakly similar to (58.9) loc_os09g09930 12009.m04258 protein heavy metal-associated domain containing protein, expressed no original description T

35.2 not assigned.unknown MDP0000328555 no original description T

30.1 signalling.in sugar and nutrient physiology MDP0000158047 moderately similar to ( 395) AT4G08950 | Symbols: EXO | EXO (EXORDIUM) | chr4:5740378-5741322 FORWARDmoderately similar to ( 355) loc_os02g52040 12002.m10235 protein phi-1-like phosphate-induced protein, putative, expressed no original description T

35.2 not assigned.unknown MDP0000163831 no original description T

35.2 not assigned.unknown MDP0000306598 very weakly similar to (85.5) AT3G09110 | Symbols: | unknown protein | chr3:2794850-2795963 REVERSEweakly similar to ( 185) loc_os01g06030 12001.m07234 protein conserved hypothetical protein no original description T

11.1.8 lipid metabolism.FA synthesis and FA elongation.acyl coa ligase MDP0000709799 highly similar to ( 807) AT5G16340 | Symbols: | AMP-binding protein, putative | chr5:5349255-5350907 REVERSEweakly similar to ( 174) 4CL1_PETCR 4-coumarate--CoA ligase 1 (EC 6.2.1.12) (4CL 1) (4-coumaroyl-CoA synthase 1) - Petroselinum crispum (Parsley) (Petroselinum hortense)highly similar to ( 751) loc_os04g57850 12004.m10669 protein AMP-binding protein, putative, expressed no original description T

29.5.11.2 protein.degradation.ubiquitin.E1 MDP0000141494 highly similar to ( 784) AT2G21470 | Symbols: SAE2, ATSAE2, EMB2764 | SAE2 (SUMO-ACTIVATING ENZYME 2); SUMO activating enzyme | chr2:9198752-9202136 FORWARDweakly similar to ( 104) UBE11_WHEAT Ubiquitin-activating enzyme E1 1 - Triticum aestivum (Wheat)highly similar to ( 746) loc_os07g39780 12007.m08220 protein ubiquitin-like 1-activating enzyme E1B, putative, expressed no original description T

35.2 not assigned.unknown MDP0000528775 weakly similar to ( 153) AT1G77540 | Symbols: | H3/H4 histone acetyltransferase | chr1:29137762-29138182 REVERSEvery weakly similar to (82.8) loc_os04g35200 12004.m08610 protein expressed protein no original description T

29.4 protein.postranslational modification MDP0000332757 highly similar to ( 527) AT5G56580 | Symbols: ATMKK6, ANQ1, MKK6 | MKK6 (MAP KINASE KINASE 6); MAP kinase kinase/ kinase | chr5:22904851-22906620 REVERSEmoderately similar to ( 463) M2K1_ORYSA Mitogen-activated protein kinase kinase 1 (EC 2.7.12.2) (MAP kinase kinase 1) (MAPKK1) (OsMEK1) - Oryza sativa (Rice)moderately similar to ( 463) loc_os01g32660 12001.m43029 protein OsMKK6 - putative MAPKK based on amino acid sequence homology, expressed no original description T

13.1.6.1.1 amino acid metabolism.synthesis.aromatic aa.chorismate.3-deoxy-D-arabino-heptulosonate 7-phosphate synthase MDP0000300043 highly similar to ( 804) AT1G22410 | Symbols: | 2-dehydro-3-deoxyphosphoheptonate aldolase, putative / 3-deoxy-D-arabino-heptulosonate 7-phosphate synthase, putative / DAHP synthetase, putative | chr1:7912120-7914742 FORWARDhighly similar to ( 817) AROG_SOLTU Phospho-2-dehydro-3-deoxyheptonate aldolase 2, chloroplast precursor (EC 2.5.1.54) (Phospho-2-keto-3-deoxyheptonate aldolase 2) (DAHP synthetase 2) (3-deoxy-D-arabino-heptulosonate 7-phosphate synthase 2) - Solanum tuberosum (Potato)highly similar to ( 812) loc_os07g42960 12007.m29007 protein phospho-2-dehydro-3-deoxyheptonate aldolase 1, chloroplast precursor, putative, expressed no original description T

35.2 not assigned.unknown MDP0000235045 highly similar to (1000) AT5G10840 | Symbols: | endomembrane protein 70, putative | chr5:3424910-3427797 REVERSEhighly similar to ( 931) loc_os08g44100 12008.m08384 protein transmembrane 9 superfamily protein member 2 precursor, putative, expressed no original description T

35.1 not assigned.no ontology MDP0000188529 moderately similar to ( 319) AT1G18440 | Symbols: | peptidyl-tRNA hydrolase family protein | chr1:6345994-6347686 FORWARDmoderately similar to ( 301) loc_os01g49900 12001.m11175 protein peptidyl-tRNA hydrolase, putative, expressed no original description T

26.21 misc.protease inhibitor/seed storage/lipid transfer protein (LTP) family protein MDP0000219404 weakly similar to ( 129) AT3G22142 | Symbols: | structural constituent of cell wall | chr3:7803604-7808046 REVERSEvery weakly similar to (85.5) 14KD_DAUCA 14 kDa proline-rich protein DC2.15 precursor - Daucus carota (Carrot)weakly similar to ( 115) loc_os06g07220 12006.m05445 protein 36.4 kDa proline-rich protein, putative, expressed no original description T

26.21 misc.protease inhibitor/seed storage/lipid transfer protein (LTP) family protein MDP0000781178 weakly similar to ( 158) AT1G55260 | Symbols: | lipid binding | chr1:20614663-20616158 FORWARDweakly similar to ( 144) loc_os03g07100 12003.m06227 protein lipid transfer protein, putative, expressed no original description T

23.1.2.8 nucleotide metabolism.synthesis.purine.SAICAR lyase MDP0000256558 highly similar to ( 713) AT1G36280 | Symbols: | adenylosuccinate lyase, putative / adenylosuccinase, putative | chr1:13640600-13642908 FORWARDhighly similar to ( 658) loc_os03g19930 12003.m35190 protein adenylosuccinate lyase, putative, expressed no original description T

10.2.1 cell wall.cellulose synthesis.cellulose synthase MDP0000448752 nearly identical (1835) AT5G05170 | Symbols: CESA3, IXR1, ATCESA3, ATH-B, CEV1 | CEV1 (CONSTITUTIVE EXPRESSION OF VSP 1); cellulose synthase/ transferase, transferring glycosyl groups | chr5:1530401-1535090 REVERSEnearly identical (1739) loc_os07g10770 12007.m05532 protein CESA8 - cellulose synthase, expressed no original description T

16.8.3.1 secondary metabolism.flavonoids.dihydroflavonols.dihydroflavonol 4-reductase MDP0000269421 weakly similar to ( 170) AT1G61720 | Symbols: BAN | BAN (BANYULS); oxidoreductase | chr1:22791326-22792757 REVERSEweakly similar to ( 157) DFRA_DIACA Dihydroflavonol-4-reductase (EC 1.1.1.219) (DFR) (Dihydrokaempferol 4-reductase) - Dianthus caryophyllus (Carnation) (Clove pink)moderately similar to ( 286) loc_os07g40986 12007.m79911 protein dihydroflavonol-4-reductase, putative, expressed no original description T

11.1.12 lipid metabolism.FA synthesis and FA elongation.ACP protein MDP0000752918 weakly similar to ( 122) AT4G25050 | Symbols: ACP4 | ACP4 (acyl carrier protein 4); acyl carrier | chr4:12870178-12871024 FORWARDweakly similar to ( 127) ACP2_HORVU Acyl carrier protein 2, chloroplast precursor (Acyl carrier protein II) (ACP II) - Hordeum vulgare (Barley)weakly similar to ( 123) loc_os12g34890 12012.m07282 protein acyl carrier protein 1, chloroplast precursor, putative, expressed no original description T

29.4 protein.postranslational modification MDP0000184748 highly similar to ( 741) AT3G45100 | Symbols: SETH2 | SETH2; transferase, transferring glycosyl groups | chr3:16504648-16506858 FORWARDhighly similar to ( 717) loc_os07g16960 12007.m06137 protein phosphatidylinositol N-acetylglucosaminyltransferase subunit A, putative, expressed no original description T

35.1 not assigned.no ontology MDP0000868063 weakly similar to ( 133) AT2G35230 | Symbols: | VQ motif-containing protein | chr2:14843192-14844079 FORWARD no original description T

30.2.17 signalling.receptor kinases.DUF 26 MDP0000314346 highly similar to ( 600) AT1G07650 | Symbols: | leucine-rich repeat transmembrane protein kinase, putative | chr1:2359817-2366423 REVERSEmoderately similar to ( 231) NORK_MEDTR Nodulation receptor kinase precursor (EC 2.7.11.1) (Does not make infections protein 2) (Symbiosis receptor-like kinase) (MtSYMRK) - Medicago truncatula (Barrel medic)highly similar to ( 607) loc_os09g17630 12009.m05021 protein receptor-like protein kinase 2, putative, expressed no original description T

29.4.1.57 protein.postranslational modification.kinase.receptor like cytoplasmatic kinase VII MDP0000314346 highly similar to ( 600) AT1G07650 | Symbols: | leucine-rich repeat transmembrane protein kinase, putative | chr1:2359817-2366423 REVERSEmoderately similar to ( 231) NORK_MEDTR Nodulation receptor kinase precursor (EC 2.7.11.1) (Does not make infections protein 2) (Symbiosis receptor-like kinase) (MtSYMRK) - Medicago truncatula (Barrel medic)highly similar to ( 607) loc_os09g17630 12009.m05021 protein receptor-like protein kinase 2, putative, expressed no original description T

23.1.1.10 nucleotide metabolism.synthesis.pyrimidine.CTP synthetase MDP0000326288 highly similar to ( 891) AT4G20320 | Symbols: | CTP synthase/ catalytic | chr4:10974980-10978998 FORWARDhighly similar to ( 863) loc_os01g43020 12001.m10565 protein CTP synthase, putative, expressed no original description T

29.5.11.4.3.2 protein.degradation.ubiquitin.E3.SCF.FBOX MDP0000283804 very weakly similar to (90.1) AT5G55150 | Symbols: | F-box family protein | chr5:22382275-22383392 REVERSEvery weakly similar to (93.6) loc_os06g35080 12006.m08045 protein F-box domain containing protein, expressed no original description T

35.2 not assigned.unknown MDP0000846861 highly similar to ( 739) AT3G15550 | Symbols: | unknown protein | chr3:5267351-5270095 FORWARDhighly similar to ( 597) loc_os01g08150 12001.m07440 protein expressed protein no original description T

34.16 transport.ABC transporters and multidrug resistance systems MDP0000309212 highly similar to ( 743) AT5G64840 | Symbols: ATGCN5 | ATGCN5 (A. THALIANA GENERAL CONTROL NON-REPRESSIBLE 5); transporter | chr5:25916956-25919693 REVERSEhighly similar to ( 696) loc_os11g39020 12011.m07750 protein expressed protein MSP1_C no original description T

29.4 protein.postranslational modification MDP0000457389 highly similar to ( 657) AT5G58350 | Symbols: WNK4, ZIK2 | WNK4 (WITH NO K (=LYSINE) 4); kinase/ protein kinase | chr5:23585505-23587681 FORWARDvery weakly similar to (84.7) M2K1_ORYSA Mitogen-activated protein kinase kinase 1 (EC 2.7.12.2) (MAP kinase kinase 1) (MAPKK1) (OsMEK1) - Oryza sativa (Rice)moderately similar to ( 491) loc_os12g02250 12012.m04226 protein mitogen-activated protein kinase, putative, expressed no original description T

27.3.25 RNA.regulation of transcription.MYB domain transcription factor family MDP0000887107 moderately similar to ( 219) AT2G47460 | Symbols: MYB12, ATMYB12, PFG1 | MYB12 (MYB DOMAIN PROTEIN 12); DNA binding / transcription activator/ transcription factor | chr2:19476438-19479242 FORWARDmoderately similar to ( 204) MYBC_MAIZE Anthocyanin regulatory C1 protein - Zea mays (Maize)moderately similar to ( 214) loc_os03g29614 12003.m08231 protein anthocyanin regulatory C1 protein, putative, expressed no original description T

11.1.13 lipid metabolism.FA synthesis and FA elongation.acyl-CoA binding protein MDP0000305778 weakly similar to ( 117) AT1G31812 | Symbols: ACBP6, ACBP | ACBP6 (acyl-CoA-binding protein 6); acyl-CoA binding / phosphatidylcholine binding | chr1:11411132-11412099 REVERSEweakly similar to ( 147) ACBP_RICCO Acyl-CoA-binding protein (ACBP) - Ricinus communis (Castor bean)weakly similar to ( 136) loc_os06g02490 12006.m04982 protein acyl-CoA-binding protein, putative, expressed no original description T

27.3.99 RNA.regulation of transcription.unclassified MDP0000296604 highly similar to ( 609) AT4G36860 | Symbols: | zinc ion binding | chr4:17358580-17361189 REVERSEhighly similar to ( 570) loc_os06g08400 12006.m32223 protein zinc ion binding protein, putative, expressed no original description T

33.99 development.unspecified MDP0000296604 highly similar to ( 609) AT4G36860 | Symbols: | zinc ion binding | chr4:17358580-17361189 REVERSEhighly similar to ( 570) loc_os06g08400 12006.m32223 protein zinc ion binding protein, putative, expressed no original description T

29.5.1 protein.degradation.subtilases MDP0000246674 highly similar to ( 643) AT1G32940 | Symbols: ATSBT3.5, SBT3.5 | SBT3.5; identical protein binding / serine-type endopeptidase | chr1:11937634-11940856 FORWARDhighly similar to ( 838) loc_os09g36110 12009.m06561 protein subtilisin-like protease precursor, putative, expressed no original description T

29.3.4.99 protein.targeting.secretory pathway.unspecified MDP0000231698 moderately similar to ( 364) AT5G14670 | Symbols: ATARFA1B | ATARFA1B (ADP-ribosylation factor A1B); GTP binding / phospholipase activator/ protein binding | chr5:4729319-4730495 FORWARDmoderately similar to ( 360) ARF2_ORYSA ADP-ribosylation factor 2 - Oryza sativa (Rice)moderately similar to ( 362) loc_os01g16030 12001.m150547 protein ADP-ribosylation factor, putative, expressed no original description T

16.8.2 secondary metabolism.flavonoids.chalcones MDP0000686661 highly similar to ( 645) AT5G13930 | Symbols: CHS, TT4, ATCHS | TT4 (TRANSPARENT TESTA 4); naringenin-chalcone synthase | chr5:4488762-4490035 FORWARDhighly similar to ( 694) CHSA_PETHY Chalcone synthase A (EC 2.3.1.74) (Naringenin-chalcone synthase A) - Petunia hybrida (Petunia)highly similar to ( 637) loc_os11g32650 12011.m07166 protein chalcone synthase, putative, expressed no original description T

29.5.11.4.2 protein.degradation.ubiquitin.E3.RING MDP0000569492 weakly similar to ( 191) AT5G05830 | Symbols: | zinc finger (C3HC4-type RING finger) family protein | chr5:1755910-1756825 FORWARDweakly similar to ( 177) loc_os05g37900 12005.m07969 protein zinc finger, C3HC4 type family protein, expressed no original description T

35.2 not assigned.unknown MDP0000180752 very weakly similar to (92.4) AT2G20820 | Symbols: | unknown protein | chr2:8964450-8965237 FORWARDvery weakly similar to (88.2) loc_os03g38520 12003.m08945 protein expressed protein no original description T

35.2 not assigned.unknown MDP0000879912 highly similar to ( 660) AT1G28520 | Symbols: VOZ1 | VOZ1 (VASCULAR PLANT ONE ZINC FINGER PROTEIN); transcription activator | chr1:10029713-10031479 FORWARDmoderately similar to ( 397) loc_os01g54930 12001.m11654 protein vascular plant one zinc finger protein, putative, expressed no original description T

35.1.5 not assigned.no ontology.pentatricopeptide (PPR) repeat-containing protein MDP0000436155 nearly identical (1056) AT4G33990 | Symbols: EMB2758 | EMB2758 (embryo defective 2758) | chr4:16290141-16292612 REVERSEweakly similar to ( 121) RF1_ORYSA Rf1 protein, mitochondrial precursor (PPR protein) (Fertility restorer) (Restorer for CMS) - Oryza sativa (Rice)highly similar to ( 860) loc_os03g27880 12003.m08112 protein EMB2758, putative no original description T

35.1.5 not assigned.no ontology.pentatricopeptide (PPR) repeat-containing protein MDP0000235197 moderately similar to ( 476) AT1G08070 | Symbols: | pentatricopeptide (PPR) repeat-containing protein | chr1:2514374-2516599 REVERSEvery weakly similar to (76.3) RF1_ORYSA Rf1 protein, mitochondrial precursor (PPR protein) (Fertility restorer) (Restorer for CMS) - Oryza sativa (Rice)moderately similar to ( 416) loc_os07g02280 12007.m04700 protein pentatricopeptide repeat protein PPR868-14, putative, expressed no original description T

35.2 not assigned.unknown MDP0000174100 weakly similar to ( 133) AT5G62550 | Symbols: | unknown protein | chr5:25105839-25107875 REVERSEweakly similar to ( 166) loc_os06g38960 12006.m32105 protein expressed protein no original description T

20.2.1 stress.abiotic.heat MDP0000500938 nearly identical (1274) AT1G79920 | Symbols: | ATP binding | chr1:30058935-30062224 REVERSEmoderately similar to ( 296) HSP7C_PETHY Heat shock cognate 70 kDa protein - Petunia hybrida (Petunia)nearly identical (1180) loc_os01g08560 12001.m150497 protein heat shock 70 kDa protein 4, putative, expressed no original description T

10.8.1 cell wall.pectin*esterases.PME MDP0000141033 moderately similar to ( 244) AT4G33220 | Symbols: PME44, ATPME44 | enzyme inhibitor/ pectinesterase | chr4:16022506-16026130 FORWARDmoderately similar to ( 270) PME_PRUPE Pectinesterase PPE8B precursor (EC 3.1.1.11) (Pectin methylesterase) (PE) - Prunus persica (Peach)weakly similar to ( 191) loc_os08g34900 12008.m07479 protein pectinesterase PPE8B precursor, putative, expressed no original description T

29.5.11 protein.degradation.ubiquitin MDP0000238587 very weakly similar to (79.3) AT5G15460 | Symbols: MUB2 | MUB2 (MEMBRANE-ANCHORED UBIQUITIN-FOLD PROTEIN 2) | chr5:5018947-5020105 REVERSEvery weakly similar to (75.9) MUB3_ORYSA Membrane-anchored ubiquitin-fold protein 3 precursor (Membrane-anchored ub-fold protein 3) (OsMUB3) - Oryza sativa (Rice)very weakly similar to (75.9) loc_os03g31290 12003.m08378 protein ubiquitin-fusion protein, putative, expressed no original description T

27.3.67 RNA.regulation of transcription.putative transcription regulator MDP0000190587 moderately similar to ( 436) AT1G04020 | Symbols: ATBARD1, BARD1 | BARD1 (BREAST CANCER ASSOCIATED RING 1); DNA binding / transcription coactivator | chr1:1036610-1040045 FORWARDmoderately similar to ( 355) loc_os04g43300 12004.m09294 protein ATBRCA1, putative, expressed no original description T

35.1 not assigned.no ontology MDP0000366057 weakly similar to ( 110) AT2G04410 | Symbols: | FUNCTIONS IN: molecular_function unknown; INVOLVED IN: biological_process unknown; LOCATED IN: plasma membrane; EXPRESSED IN: 24 plant structures; EXPRESSED DURING: 15 growth stages; CONTAINS InterPro DOMAIN/s: Defence response, Rin4 (InterPro:IPR008700); BEST Arabidopsis thaliana protein match is: NOI (TAIR:AT5G55850.1); Has 141 Blast hits to 140 proteins in 13 species: Archae - 0; Bacteria - 0; Metazoa - 0; Fungi - 0; Plants - 139; Viruses - 0; Other Eukaryotes - 2 (source: NCBI BLink). | chr2:1534012-1535040 REVERSEvery weakly similar to (98.2) loc_os09g07920 12009.m04061 protein NOI protein, putative, expressed no original description T

31.3 cell.cycle MDP0000419883 moderately similar to ( 245) AT2G26760 | Symbols: CYCB1;4 | CYCB1;4 (Cyclin B1;4); cyclin-dependent protein kinase regulator | chr2:11401551-11403205 FORWARDmoderately similar to ( 270) CCN2_ANTMA G2/mitotic-specific cyclin-2 - Antirrhinum majus (Garden snapdragon)moderately similar to ( 236) loc_os01g59120 12001.m42540 protein cyclin IaZm, putative, expressed no original description T

29.2.1.2.2.5 protein.synthesis.ribosomal protein.eukaryotic.60S subunit.L5 MDP0000010907 moderately similar to ( 465) AT3G25520 | Symbols: ATL5, PGY3, OLI5, RPL5A | ATL5 (A. THALIANA RIBOSOMAL PROTEIN L5); 5S rRNA binding / structural constituent of ribosome | chr3:9269573-9271327 REVERSEmoderately similar to ( 472) RL5_CUCSA 60S ribosomal protein L5 - Cucumis sativus (Cucumber)moderately similar to ( 444) loc_os01g67126 12001.m43540 protein 60S ribosomal protein L5-2, putative, expressed no original description T

35.2 not assigned.unknown MDP0000296668 moderately similar to ( 353) AT4G35920 | Symbols: MCA1 | MCA1 (mid1-complementing activity 1) | chr4:17012106-17014192 REVERSEmoderately similar to ( 367) loc_os03g06120 12003.m101195 protein domain of unknown function DUF614 containing protein, expressed no original description T

29.2.1.2.1.9 protein.synthesis.ribosomal protein.eukaryotic.40S subunit.S9 MDP0000251733 moderately similar to ( 202) AT5G39850 | Symbols: | 40S ribosomal protein S9 (RPS9C) | chr5:15950053-15951171 FORWARDvery weakly similar to (57.0) POLX_TOBAC Retrovirus-related Pol polyprotein from transposon TNT 1-94 [Includes: Protease (EC 3.4.23.-); Reverse transcriptase (EC 2.7.7.49); Endonuclease] - Nicotiana tabacum (Common tobacco)weakly similar to ( 191) loc_os11g38959 12011.m80083 protein 40S ribosomal protein S9, putative, expressed no original description T

35.2 not assigned.unknown MDP0000817919 highly similar to ( 739) AT3G16270 | Symbols: | INVOLVED IN: intracellular protein transport; LOCATED IN: membrane; EXPRESSED IN: 25 plant structures; EXPRESSED DURING: 15 growth stages; CONTAINS InterPro DOMAIN/s: VHS (InterPro:IPR002014), ENTH/VHS (InterPro:IPR008942); Has 113 Blast hits to 112 proteins in 49 species: Archae - 0; Bacteria - 3; Metazoa - 48; Fungi - 1; Plants - 23; Viruses - 0; Other Eukaryotes - 38 (source: NCBI BLink). | chr3:5513701-5516540 FORWARDhighly similar to ( 614) loc_os01g13160 12001.m07926 protein expressed protein no original description T

16.8.2 secondary metabolism.flavonoids.chalcones MDP0000233012 moderately similar to ( 254) AT1G53520 | Symbols: | chalcone-flavanone isomerase-related | chr1:19976485-19977915 REVERSEweakly similar to ( 153) loc_os02g21520 12002.m07391 protein chalcone isomerase 3, putative, expressed no original description T

31.1 cell.organisation MDP0000151345 highly similar to ( 616) AT3G01750 | Symbols: | ankyrin repeat family protein | chr3:270615-272691 FORWARDhighly similar to ( 591) loc_os05g23320 12005.m06627 protein expressed protein no original description T

35.2 not assigned.unknown MDP0000817973 moderately similar to ( 332) AT5G13720 | Symbols: | LOCATED IN: chloroplast, chloroplast inner membrane, chloroplast envelope; EXPRESSED IN: 21 plant structures; EXPRESSED DURING: 13 growth stages; CONTAINS InterPro DOMAIN/s: Uncharacterised conserved protein UCP022348 (InterPro:IPR016804), Uncharacterised protein family UPF0114 (InterPro:IPR005134); BEST Arabidopsis thaliana protein match is: unknown protein (TAIR:AT4G19390.1); Has 294 Blast hits to 294 proteins in 98 species: Archae - 18; Bacteria - 168; Metazoa - 0; Fungi - 0; Plants - 53; Viruses - 0; Other Eukaryotes - 55 (source: NCBI BLink). | chr5:4427960-4429029 FORWARDmoderately similar to ( 317) loc_os07g46330 12007.m08850 protein uncharacterized protein UPF0114, putative, expressed no original description T

11.1.12 lipid metabolism.FA synthesis and FA elongation.ACP protein MDP0000292177 weakly similar to ( 181) AT1G65290 | Symbols: mtACP2 | mtACP2 (mitochondrial acyl carrier protein 2); acyl carrier/ metal ion binding | chr1:24249088-24250366 REVERSEvery weakly similar to (58.2) ACP2_BRANA Acyl carrier protein, chloroplast precursor (ACP) (ACP09) (Clone 22C01) - Brassica napus (Rape)weakly similar to ( 163) loc_os03g22950 12003.m07693 protein acyl carrier protein, mitochondrial precursor, putative, expressed no original description T

34.11 transport.NDP-sugars at the ER MDP0000165524 highly similar to ( 580) AT1G14360 | Symbols: ATUTR3, UTR3 | UTR3 (UDP-GALACTOSE TRANSPORTER 3); pyrimidine nucleotide sugar transmembrane transporter | chr1:4911362-4913029 REVERSEmoderately similar to ( 497) loc_os06g39260 12006.m08464 protein solute carrier family 35 member B1, putative, expressed no original description T

35.2 not assigned.unknown MDP0000224533 moderately similar to ( 451) AT3G01680 | Symbols: | unknown protein | chr3:252033-255246 FORWARD no original description T

34.12 transport.metal MDP0000435937 moderately similar to ( 475) AT1G79520 | Symbols: | cation efflux family protein | chr1:29912369-29914515 REVERSEmoderately similar to ( 416) loc_os01g03914 12001.m150472 protein metal tolerance protein C3, putative, expressed no original description T

27.3.99 RNA.regulation of transcription.unclassified MDP0000205080 moderately similar to ( 443) AT1G67340 | Symbols: | zinc finger (MYND type) family protein / F-box family protein | chr1:25230323-25231622 FORWARDmoderately similar to ( 369) loc_os01g69270 12001.m13027 protein MYND finger family protein, expressed no original description T

29.2.1.2.2.11 protein.synthesis.ribosomal protein.eukaryotic.60S subunit.L11 MDP0000616695 moderately similar to ( 304) AT5G45775 | Symbols: | 60S ribosomal protein L11 (RPL11D) | chr5:18565281-18566496 REVERSEmoderately similar to ( 304) RL11_MEDSA 60S ribosomal protein L11 (L5) - Medicago sativa (Alfalfa)moderately similar to ( 298) loc_os02g14059 12002.m77785 protein 60S ribosomal protein L11-1, putative, expressed no original description T

35.2 not assigned.unknown MDP0000716596 weakly similar to ( 130) AT5G22430 | Symbols: | unknown protein | chr5:7433913-7434646 FORWARD no original description T

35.2 not assigned.unknown MDP0000517458 moderately similar to ( 286) AT1G18060 | Symbols: | unknown protein | chr1:6212065-6213314 REVERSEmoderately similar to ( 256) loc_os01g03040 12001.m06943 protein expressed protein no original description T

30.2.17 signalling.receptor kinases.DUF 26 MDP0000269570 highly similar to ( 703) AT2G41890 | Symbols: | curculin-like (mannose-binding) lectin family protein / PAN domain-containing protein | chr2:17478058-17480352 REVERSEweakly similar to ( 195) KPRO_MAIZE Putative receptor protein kinase ZmPK1 precursor (EC 2.7.11.1) - Zea mays (Maize)moderately similar to ( 303) loc_os03g62180 12003.m101559 protein ATP binding protein, putative, expressed no original description T

29.5.5 protein.degradation.serine protease MDP0000151521 moderately similar to ( 472) AT1G28110 | Symbols: SCPL45 | SCPL45 (SERINE CARBOXYPEPTIDASE-LIKE 45 PRECURSOR); serine-type carboxypeptidase | chr1:9804153-9806832 REVERSEmoderately similar to ( 279) CBP2_WHEAT Serine carboxypeptidase 2 (EC 3.4.16.6) (Serine carboxypeptidase II) (Carboxypeptidase D) (CPDW-II) (CP-WII) [Contains: Serine carboxypeptidase 2 chain A (Serine carboxypeptidase II chain A); Serine carboxypeptidase 2 chain B (Serine camoderately similar to ( 481) loc_os11g31980 12011.m07100 protein serine carboxypeptidase 1 precursor, putative, expressed no original description T

26.1 misc.misc2 MDP0000072936 moderately similar to ( 265) AT3G23600 | Symbols: | dienelactone hydrolase family protein | chr3:8473833-8475655 FORWARDweakly similar to ( 119) E134_MAIZE Endo-1,3;1,4-beta-D-glucanase precursor (EC 3.2.1.-) - Zea mays (Maize)moderately similar to ( 245) loc_os05g33100 12005.m07542 protein endo-1,3;1,4-beta-D-glucanase precursor, putative, expressed no original description T

35.2 not assigned.unknown MDP0000517479 weakly similar to ( 122) AT1G52140 | Symbols: | unknown protein | chr1:19407559-19408185 REVERSEweakly similar to ( 108) loc_os06g36070 12006.m08144 protein expressed protein no original description T

5.10 fermentation.aldehyde dehydrogenase MDP0000790166 highly similar to ( 779) AT1G54100 | Symbols: ALDH7B4 | ALDH7B4 (Aldehyde Dehydrogenase 7B4); 3-chloroallyl aldehyde dehydrogenase/ oxidoreductase | chr1:20195435-20198853 REVERSEhighly similar to ( 960) AL7A1_MALDO Aldehyde dehydrogenase family 7 member A1 (EC 1.2.1.3) (Antiquitin-1) (Matured fruit 60 kDa protein) (MF-60) - Malus domestica (Apple) (Malus sylvestris)highly similar to ( 794) loc_os09g26880 12009.m22098 protein aldehyde dehydrogenase family 7 member A1, putative, expressed no original description T

26.11 misc.alcohol dehydrogenases MDP0000375181 highly similar to ( 615) AT5G24760 | Symbols: | alcohol dehydrogenase, putative | chr5:8495035-8497090 REVERSEmoderately similar to ( 423) ADH1_MAIZE Alcohol dehydrogenase 1 (EC 1.1.1.1) - Zea mays (Maize)highly similar to ( 573) loc_os02g42520 12002.m33422 protein alcohol dehydrogenase 1, putative, expressed no original description T

17.2.1 hormone metabolism.auxin.synthesis-degradation MDP0000312316 highly similar to ( 715) AT1G10510 | Symbols: emb2004 | emb2004 (embryo defective 2004) | chr1:3461771-3465590 FORWARDhighly similar to ( 630) loc_os06g13050 12006.m06020 protein peroxidase family protein, expressed no original description T

35.2 not assigned.unknown MDP0000613174 highly similar to ( 531) AT1G54385 | Symbols: | binding | chr1:20301289-20303048 REVERSEmoderately similar to ( 444) loc_os11g37100 12011.m07558 protein expressed protein no original description T

27.3.67 RNA.regulation of transcription.putative transcription regulator MDP0000253359 highly similar to ( 714) AT1G56110 | Symbols: NOP56 | NOP56 (Arabidopsis homolog of nucleolar protein Nop56) | chr1:20984544-20986893 REVERSEmoderately similar to ( 389) KAD2_ORYSA Adenylate kinase B (EC 2.7.4.3) (ATP-AMP transphosphorylase) - Oryza sativa (Rice)highly similar to ( 699) loc_os03g22880 12003.m07686 protein nucleolar protein Nop56, putative, expressed no original description T

10.2.1 cell wall.cellulose synthesis.cellulose synthase MDP0000185368 nearly identical (1662) AT4G39350 | Symbols: CESA2, ATH-A, ATCESA2 | CESA2 (CELLULOSE SYNTHASE A2); cellulose synthase/ transferase, transferring glycosyl groups | chr4:18297078-18301890 FORWARDnearly identical (1504) loc_os03g62090 12003.m11087 protein CESA5 - cellulose synthase, expressed no original description T

26.22 misc.short chain dehydrogenase/reductase (SDR) MDP0000305623 moderately similar to ( 322) AT1G67730 | Symbols: YBR159, KCR1 | YBR159; ketoreductase/ oxidoreductase | chr1:25391676-25393365 FORWARDvery weakly similar to (50.1) FABG3_BRANA 3-oxoacyl-[acyl-carrier-protein] reductase 3, chloroplast precursor (EC 1.1.1.100) (3-ketoacyl-acyl carrier protein reductase 3) - Brassica napus (Rape)moderately similar to ( 259) loc_os04g40730 12004.m09053 protein steroid dehydrogenase SPM2, putative, expressed PRK07003 no original description T

34.2 transport.sugars MDP0000301544 moderately similar to ( 397) AT3G01280 | Symbols: VDAC1, ATVDAC1 | VDAC1 (VOLTAGE DEPENDENT ANION CHANNEL 1); voltage-gated anion channel | chr3:85754-87612 FORWARDmoderately similar to ( 437) VDAC_PEA Outer plastidial membrane protein porin (Voltage-dependent anion-selective channel protein) (VDAC) - Pisum sativum (Garden pea)moderately similar to ( 372) loc_os09g19734 12009.m50200 protein isochorismate synthase 1, chloroplast precursor, putative, expressed no original description T

26.2 misc.UDP glucosyl and glucoronyl transferases MDP0000854555 highly similar to ( 947) AT3G61130 | Symbols: GAUT1, LGT1 | GAUT1 (GALACTURONOSYLTRANSFERASE 1); polygalacturonate 4-alpha-galacturonosyltransferase/ transferase, transferring glycosyl groups | chr3:22622399-22625514 FORWARDhighly similar to ( 932) loc_os09g36190 12009.m06568 protein transferase, transferring glycosyl groups, putative, expressed no original description T

29.4 protein.postranslational modification MDP0000265859 weakly similar to ( 179) AT2G20450 | Symbols: | 60S ribosomal protein L14 (RPL14A) | chr2:8813923-8815071 FORWARDweakly similar to ( 175) RL14_PEA Probable 60 ribosomal protein L14 (Hydroxyproline-rich glycoprotein HRGP1) - Pisum sativum (Garden pea)weakly similar to ( 190) loc_os04g43540 12004.m09314 protein 60 ribosomal protein L14, putative, expressed no original description T

35.2 not assigned.unknown MDP0000418876 moderately similar to ( 269) AT5G37070 | Symbols: | unknown protein | chr5:14651091-14652147 FORWARDmoderately similar to ( 258) loc_os05g27950 12005.m07083 protein cp protein, putative, expressed no original description T

13.1.5.3.2 amino acid metabolism.synthesis.serine-glycine-cysteine group.cysteine.SAT MDP0000390627 moderately similar to ( 414) AT5G56760 | Symbols: ATSERAT1;1, SAT5, SAT-52 | ATSERAT1;1 (ARABIDOPSIS THALIANA SERINE ACETYLTRANSFERASE 1;1); serine O-acetyltransferase | chr5:22961498-22962582 REVERSEmoderately similar to ( 382) loc_os01g52260 12001.m11400 protein serine acetyltransferase 3, mitochondrial precursor, putative, expressed no original description T

29.2.1.2.1.8 protein.synthesis.ribosomal protein.eukaryotic.40S subunit.S8 MDP0000651852 moderately similar to ( 394) AT5G06360 | Symbols: | ribosomal protein S8e family protein | chr5:1944835-1946512 FORWARDmoderately similar to ( 382) loc_os07g47580 12007.m079786 protein TGF-beta-inducible nuclear protein 1, putative, expressed no original description T

27.3.37 RNA.regulation of transcription.AS2,Lateral Organ Boundaries Gene Family MDP0000317227 moderately similar to ( 226) AT5G67420 | Symbols: LBD37 | LBD37 (LOB DOMAIN-CONTAINING PROTEIN 37) | chr5:26904576-26905415 REVERSEweakly similar to ( 190) loc_os03g33090 12003.m08552 protein seed specific protein Bn15D17A, putative, expressed COG3889 no original description T

29.4 protein.postranslational modification MDP0000046978 highly similar to ( 823) AT3G50530 | Symbols: CRK | CRK (CDPK-related kinase); ATP binding / calcium ion binding / calcium-dependent protein serine/threonine phosphatase/ kinase/ protein kinase/ protein serine/threonine kinase | chr3:18753833-18756487 FORWARDhighly similar to ( 828) CRK_DAUCA CDPK-related protein kinase (EC 2.7.11.1) (PK421) - Daucus carota (Carrot)highly similar to ( 783) loc_os07g42770 12007.m08507 protein CDPK-related protein kinase, putative, expressed no original description T

35.2 not assigned.unknown MDP0000144712 no original description T

35.2 not assigned.unknown MDP0000294816 nearly identical (2128) AT3G50590 | Symbols: | nucleotide binding | chr3:18771292-18779220 FORWARDnearly identical (1115) loc_os09g24260 12009.m05582 protein nucleotide binding protein, putative, expressed no original description T

1.1.1.2 PS.lightreaction.photosystem II.PSII polypeptide subunits MDP0000858039 highly similar to ( 554) AT3G50820 | Symbols: PSBO2, PSBO-2, OEC33 | PSBO2 (PHOTOSYSTEM II SUBUNIT O-2); oxygen evolving/ poly(U) binding | chr3:18891008-18892311 REVERSEhighly similar to ( 579) PSBO_TOBAC Oxygen-evolving enhancer protein 1, chloroplast precursor (OEE1) (33 kDa subunit of oxygen evolving system of photosystem II) (OEC 33 kDa subunit) (33 kDa thylakoid membrane protein) - Nicotiana tabacum (Common tobacco)highly similar to ( 541) loc_os01g31690 12001.m43025 protein oxygen-evolving enhancer protein 1, chloroplast precursor, putative, expressed no original description T

34.8 transport.metabolite transporters at the envelope membrane MDP0000878404 highly similar to ( 551) AT3G11320 | Symbols: | organic anion transmembrane transporter | chr3:3547017-3548539 REVERSEvery weakly similar to (95.1) TPT_SOLTU Triose phosphate/phosphate translocator, chloroplast precursor (cTPT) (E29) - Solanum tuberosum (Potato)highly similar to ( 518) loc_os03g17740 12003.m07199 protein organic anion transporter, putative, expressed no original description T

29.2.1.1.1.2.27 protein.synthesis.ribosomal protein.prokaryotic.chloroplast.50S subunit.L27 MDP0000888078 moderately similar to ( 216) AT5G15220 | Symbols: | ribosomal protein L27 family protein | chr5:4941466-4942133 REVERSEvery weakly similar to (99.4) RK27_TOBAC 50S ribosomal protein L27, chloroplast precursor (CL27) - Nicotiana tabacum (Common tobacco)moderately similar to ( 202) loc_os08g31228 12008.m080153 protein 50S ribosomal protein L27, putative, expressed no original description T

29.5.7 protein.degradation.metalloprotease MDP0000145511 moderately similar to ( 300) AT2G36305 | Symbols: RCE1, ATFACE-2, ATFACE2, FACE2 | FACE2 (FARNESYLATED PROTEIN-CONVERTING ENZYME 2); endopeptidase | chr2:15214607-15216449 FORWARDmoderately similar to ( 236) loc_os05g28950 12005.m083699 protein CAAX prenyl protease 2, putative, expressed no original description T

9.5 mitochondrial electron transport / ATP synthesis.cytochrome c reductase MDP0000269746 weakly similar to ( 147) AT5G25450 | Symbols: | ubiquinol-cytochrome C reductase complex 14 kDa protein, putative | chr5:8857036-8857849 FORWARDweakly similar to ( 131) UCR6_SOLTU Ubiquinol-cytochrome c reductase complex 14 kDa protein (EC 1.10.2.2) (CR14) - Solanum tuberosum (Potato)weakly similar to ( 119) loc_os07g10500 12007.m05505 protein ubiquinol-cytochrome c reductase complex 14 kDa protein, putative no original description T

11.1.12 lipid metabolism.FA synthesis and FA elongation.ACP protein MDP0000174750 very weakly similar to (69.7) AT2G44620 | Symbols: MTACP-1, MTACP1 | MTACP-1 (MITOCHONDRIAL ACYL CARRIER PROTEIN 1); acyl carrier/ cofactor binding / phosphopantetheine binding | chr2:18414320-18415065 FORWARDvery weakly similar to (52.8) loc_os07g12150 12007.m05668 protein acyl carrier protein, mitochondrial precursor, putative, expressed no original description T

29.2.1.2.1.18 protein.synthesis.ribosomal protein.eukaryotic.40S subunit.S18 MDP0000560127 moderately similar to ( 270) AT4G09800 | Symbols: RPS18C | RPS18C (S18 RIBOSOMAL PROTEIN); RNA binding / nucleic acid binding / structural constituent of ribosome | chr4:6173818-6174963 FORWARDmoderately similar to ( 211) RS18_CHLRE 40S ribosomal protein S18 - Chlamydomonas reinhardtiimoderately similar to ( 254) loc_os07g07709 12007.m29370 protein 40S ribosomal protein S18, putative, expressed no original description T

29.2.1.1.1.2.3 protein.synthesis.ribosomal protein.prokaryotic.chloroplast.50S subunit.L3 MDP0000823145 moderately similar to ( 439) AT3G17465 | Symbols: RPL3P | RPL3P (RIBOSOMAL PROTEIN L3 PLASTID); structural constituent of ribosome | chr3:5978059-5979572 REVERSEweakly similar to ( 130) RK3_TOBAC 50S ribosomal protein L3, chloroplast precursor (Fragment) - Nicotiana tabacum (Common tobacco)moderately similar to ( 407) loc_os01g14830 12001.m08085 protein 50S ribosomal protein L3-2, chloroplast precursor, putative, expressed no original description T

35.1 not assigned.no ontology MDP0000307711 highly similar to ( 935) AT2G01970 | Symbols: | endomembrane protein 70, putative | chr2:452197-454819 REVERSEhighly similar to ( 885) loc_os03g13380 12003.m06783 protein transmembrane 9 superfamily protein member 1 precursor, putative, expressed no original description T

13.99 amino acid metabolism.misc MDP0000148984 moderately similar to ( 479) AT1G49820 | Symbols: ATMTK | ATMTK (ARABIDOPSIS THALIANA S-METHYL-5-THIORIBOSE KINASE); S-methyl-5-thioribose kinase | chr1:18443005-18444907 FORWARDmoderately similar to ( 447) loc_os04g57400 12004.m35509 protein methylthioribose kinase, putative, expressed no original description T

15.2 metal handling.binding, chelation and storage MDP0000251135 weakly similar to ( 120) AT1G06330 | Symbols: | copper-binding family protein | chr1:1931671-1932266 REVERSEweakly similar to ( 123) loc_os01g46390 12001.m10837 protein conserved hypothetical protein no original description T

31.3 cell.cycle MDP0000257591 moderately similar to ( 269) AT2G44740 | Symbols: CYCP4;1 | CYCP4;1 (cyclin p4;1); cyclin-dependent protein kinase | chr2:18442287-18443304 REVERSEmoderately similar to ( 204) loc_os10g41430 12010.m06907 protein nuc-1 negative regulatory protein preg, putative, expressed no original description T

29.4 protein.postranslational modification MDP0000003459 highly similar to ( 751) AT5G26751 | Symbols: ATSK11, SK 11 | ATSK11; protein kinase/ protein serine/threonine kinase | chr5:9399582-9401839 REVERSEhighly similar to ( 760) MSK3_MEDSA Glycogen synthase kinase-3 homolog MsK-3 (EC 2.7.11.1) - Medicago sativa (Alfalfa)highly similar to ( 744) loc_os01g14860 12001.m43459 protein glycogen synthase kinase-3 homolog MsK-3, putative, expressed no original description T

34.18 transport.unspecified anions MDP0000295912 nearly identical (1509) AT1G30450 | Symbols: CCC1, ATCCC1, HAP5 | CCC1 (CATION-CHLORIDE CO-TRANSPORTER 1); cation:chloride symporter/ sodium:potassium:chloride symporter | chr1:10762905-10769061 FORWARDnearly identical (1494) loc_os08g23440 12008.m06365 protein CCC1, putative, expressed no original description T

29.5.11.1 protein.degradation.ubiquitin.ubiquitin MDP0000141893 moderately similar to ( 259) AT1G31340 | Symbols: RUB1, NEDD8, ATRUB1 | RUB1 (RELATED TO UBIQUITIN 1); protein binding | chr1:11218076-11219417 REVERSEweakly similar to ( 150) UBIQ_WHEAT Ubiquitin - Triticum aestivum (Wheat)moderately similar to ( 259) loc_os09g25320 12009.m05684 protein polyubiquitin 2, putative, expressed no original description T

35.2 not assigned.unknown MDP0000894041 moderately similar to ( 219) AT2G15890 | Symbols: MEE14 | MEE14 (maternal effect embryo arrest 14) | chr2:6921196-6921978 REVERSEweakly similar to ( 144) loc_os03g55670 12003.m10479 protein expressed protein no original description T

17.3.1.2.2 hormone metabolism.brassinosteroid.synthesis-degradation.sterols.SMT2 MDP0000408791 highly similar to ( 675) AT1G20330 | Symbols: SMT2, CVP1, FRL1 | SMT2 (STEROL METHYLTRANSFERASE 2); S-adenosylmethionine-dependent methyltransferase | chr1:7038968-7040053 REVERSEhighly similar to ( 533) SMT2_ORYSA 24-methylenesterol C-methyltransferase 2 (EC 2.1.1.143) (24-sterol C-methyltransferase 2) (Sterol-C-methyltransferase 2) - Oryza sativa (Rice)highly similar to ( 533) loc_os03g04340 12003.m05964 protein 24-methylenesterol C-methyltransferase 2, putative, expressed no original description T

35.1 not assigned.no ontology MDP0000282000 highly similar to ( 591) AT2G01070 | Symbols: | FUNCTIONS IN: molecular_function unknown; INVOLVED IN: biological_process unknown; LOCATED IN: endomembrane system, integral to membrane; EXPRESSED IN: 22 plant structures; EXPRESSED DURING: 13 growth stages; CONTAINS InterPro DOMAIN/s: Transmembrane receptor, eukaryota (InterPro:IPR009637); BEST Arabidopsis thaliana protein match is: unknown protein (TAIR:AT1G72480.1); Has 487 Blast hits to 486 proteins in 125 species: Archae - 0; Bacteria - 0; Metazoa - 264; Fungi - 101; Plants - 89; Viruses - 0; Other Eukaryotes - 33 (source: NCBI BLink). | chr2:75596-77300 FORWARDhighly similar to ( 629) loc_os11g34360 12011.m07294 protein TMEM87A protein, putative, expressed no original description T

35.2 not assigned.unknown MDP0000150006 moderately similar to ( 388) AT1G71900 | Symbols: | FUNCTIONS IN: molecular_function unknown; INVOLVED IN: biological_process unknown; LOCATED IN: cellular_component unknown; EXPRESSED IN: 22 plant structures; EXPRESSED DURING: 13 growth stages; CONTAINS InterPro DOMAIN/s: Protein of unknown function DUF803 (InterPro:IPR008521); BEST Arabidopsis thaliana protein match is: permease-related (TAIR:AT1G34470.1); Has 875 Blast hits to 858 proteins in 150 species: Archae - 0; Bacteria - 55; Metazoa - 361; Fungi - 238; Plants - 146; Viruses - 0; Other Eukaryotes - 75 (source: NCBI BLink). | chr1:27061754-27064053 FORWARDmoderately similar to ( 416) loc_os05g35060 12005.m07736 protein non-imprinted in Prader-Willi/Angelman syndrome region protein 1homolog, putative, expressed no original description T

29.2.1.2.2.35 protein.synthesis.ribosomal protein.eukaryotic.60S subunit.L35 MDP0000292576 weakly similar to ( 107) AT2G39390 | Symbols: | 60S ribosomal protein L35 (RPL35B) | chr2:16450803-16451762 REVERSEweakly similar to ( 103) loc_os04g30730 12004.m35547 protein 60S ribosomal protein L35, putative, expressed no original description T

20.1.7.6.1 stress.biotic.PR-proteins.proteinase inhibitors.trypsin inhibitor MDP0000277095 highly similar to ( 726) AT1G19110 | Symbols: | inter-alpha-trypsin inhibitor heavy chain-related | chr1:6602270-6605766 FORWARDhighly similar to ( 639) loc_os07g42280 12007.m08463 protein von Willebrand factor type A domain containing protein, expressed no original description T

29.5.11.4.3.2 protein.degradation.ubiquitin.E3.SCF.FBOX MDP0000257953 highly similar to ( 540) AT1G21410 | Symbols: SKP2A | SKP2A | chr1:7497479-7499386 FORWARDmoderately similar to ( 462) loc_os05g35110 12005.m07741 protein F-box/LRR-repeat protein 2, putative, expressed no original description T

27.3.24 RNA.regulation of transcription.MADS box transcription factor family MDP0000270062 moderately similar to ( 205) AT4G22950 | Symbols: AGL19, GL19 | AGL19 (AGAMOUS-LIKE 19); transcription factor | chr4:12023946-12027421 REVERSEweakly similar to ( 189) MAD50_ORYSA MADS-box transcription factor 50 (OsMADS50) (Protein SUPPRESSOR OF CONSTANS OVEREXPRESSION 1-like) (OsSOC1) (Protein AGAMOUS-like 20) (RMADS208) - Oryza sativa (Rice)weakly similar to ( 182) loc_os10g39130 12010.m21952 protein MADS-box transcription factor 56, putative, expressed no original description T

34.11 transport.NDP-sugars at the ER MDP0000229128 highly similar to ( 502) AT4G23010 | Symbols: ATUTR2, UTR2 | UDP-galactose transporter-related | chr4:12060318-12062486 REVERSEmoderately similar to ( 498) loc_os03g02670 12003.m34768 protein solute carrier family 35 member B3, putative, expressed no original description T

35.2 not assigned.unknown MDP0000268973 highly similar to ( 796) AT3G48770 | Symbols: | ATP binding / DNA binding | chr3:18079261-18086817 REVERSEhighly similar to ( 763) loc_os11g31480 12011.m07051 protein DNA binding protein, putative, expressed no original description T

10.6.3 cell wall.degradation.pectate lyases and polygalacturonases MDP0000145734 highly similar to ( 664) AT1G04680 | Symbols: | pectate lyase family protein | chr1:1304052-1307780 REVERSEmoderately similar to ( 413) PEL_LILLO Pectate lyase precursor (EC 4.2.2.2) - Lilium longiflorum (Trumpet lily)highly similar to ( 649) loc_os04g05050 12004.m35243 protein pectate lyase 8 precursor, putative, expressed no original description T

35.2 not assigned.unknown MDP0000270344 highly similar to ( 550) AT4G37680 | Symbols: HHP4 | HHP4 (heptahelical protein 4); receptor | chr4:17701231-17702568 FORWARDhighly similar to ( 519) loc_os03g13040 12003.m06750 protein haemolysin-III related family protein, expressed no original description T

29.2.3 protein.synthesis.initiation MDP0000364164 moderately similar to ( 215) AT2G04520 | Symbols: | eukaryotic translation initiation factor 1A, putative / eIF-1A, putative / eIF-4C, putative | chr2:1574802-1575239 REVERSEweakly similar to ( 197) IF1A_WHEAT Eukaryotic translation initiation factor 1A (EIF-1A) (EIF-4C) - Triticum aestivum (Wheat)weakly similar to ( 198) loc_os02g19770 12002.m07219 protein eukaryotic translation initiation factor 1A, putative, expressed no original description T

30.3 signalling.calcium MDP0000604285 moderately similar to ( 459) AT4G00820 | Symbols: iqd17 | iqd17 (IQ-domain 17); calmodulin binding | chr4:349300-351307 FORWARDmoderately similar to ( 412) loc_os08g02250 12008.m04369 protein calmodulin binding protein, putative, expressed no original description T

20.2.99 stress.abiotic.unspecified MDP0000415257 moderately similar to ( 216) AT3G17020 | Symbols: | universal stress protein (USP) family protein | chr3:5802728-5804063 REVERSEmoderately similar to ( 211) loc_os05g06500 12005.m05177 protein USP family protein, putative, expressed no original description T

29.2.5 protein.synthesis.release MDP0000557551 highly similar to ( 746) AT3G26618 | Symbols: ERF1-3 | ERF1-3 (eukaryotic release factor 1-3); translation release factor | chr3:9788854-9790161 FORWARDhighly similar to ( 758) loc_os07g39870 12007.m079730 protein eukaryotic peptide chain release factor subunit 1-1, putative, expressed no original description T

35.2 not assigned.unknown MDP0000150959 moderately similar to ( 259) AT5G61340 | Symbols: | unknown protein | chr5:24662064-24663044 REVERSEweakly similar to ( 183) loc_os12g38810 12012.m07666 protein expressed protein no original description T

29.5.11.4.2 protein.degradation.ubiquitin.E3.RING MDP0000460903 highly similar to ( 969) AT1G24330 | Symbols: | armadillo/beta-catenin repeat family protein / U-box domain-containing family protein | chr1:8631779-8634835 FORWARDweakly similar to ( 177) SPL11_ORYSA Spotted leaf protein 11 (Spotted leaf11) (Cell death-related protein SPL11) - Oryza sativa (Rice)highly similar to ( 792) loc_os01g67500 12001.m42788 protein ubiquitin-protein ligase, putative, expressed no original description T

26.1 misc.misc2 MDP0000329703 highly similar to ( 679) AT1G13560 | Symbols: AAPT1, ATAAPT1 | AAPT1 (AMINOALCOHOLPHOSPHOTRANSFERASE 1); phosphatidyltransferase/ phosphotransferase, for other substituted phosphate groups | chr1:4638834-4641691 REVERSEhighly similar to ( 642) loc_os02g02750 12002.m33990 protein ethanolaminephosphotransferase, putative, expressed no original description T

29.2.1.2.1.8 protein.synthesis.ribosomal protein.eukaryotic.40S subunit.S8 MDP0000201376 moderately similar to ( 341) AT5G06360 | Symbols: | ribosomal protein S8e family protein | chr5:1944835-1946512 FORWARDmoderately similar to ( 310) loc_os07g47580 12007.m079786 protein TGF-beta-inducible nuclear protein 1, putative, expressed no original description T

29.4 protein.postranslational modification MDP0000274900 highly similar to ( 679) AT1G72710 | Symbols: CKL2 | CKL2 (CASEIN KINASE 1-LIKE PROTEIN 2); ATP binding / kinase/ protein kinase/ protein serine/threonine kinase | chr1:27372553-27376178 FORWARDvery weakly similar to (62.8) KC47_ORYSA CDC2+/CDC28-related protein kinase R2 (EC 2.7.11.22) - Oryza sativa (Rice)highly similar to ( 677) loc_os01g51200 12001.m43159 protein casein kinase I isoform delta-like, putative, expressed no original description T

34.16 transport.ABC transporters and multidrug resistance systems MDP0000286528 nearly identical (1841) AT4G25960 | Symbols: PGP2 | PGP2 (P-GLYCOPROTEIN 2); ATPase, coupled to transmembrane movement of substances | chr4:13177438-13183425 FORWARDnearly identical (1006) MDR_ORYSA Putative multidrug resistance protein (P-glycoprotein) - Oryza sativa (Rice)nearly identical (1788) loc_os02g46680 12002.m33438 protein multidrug resistance protein 2, putative, expressed no original description T

27.3.99 RNA.regulation of transcription.unclassified MDP0000231017 weakly similar to ( 194) AT1G51200 | Symbols: | zinc finger (AN1-like) family protein | chr1:18985690-18986211 FORWARDweakly similar to ( 120) ISAP1_ORYSA Multiple stress-responsive zinc-finger protein ISAP1 (Stress-associated protein 1) (OsISAP1) - Oryza sativa (Rice)moderately similar to ( 220) loc_os06g41010 12006.m31898 protein zinc finger A20 and AN1 domains-containing protein, putative, expressed no original description T

13.1.1.2.1 amino acid metabolism.synthesis.central amino acid metabolism.aspartate.aspartate aminotransferase MDP0000280867 highly similar to ( 861) AT5G25757 | Symbols: | unknown protein | chr5:8965515-8967460 REVERSEmoderately similar to ( 447) AATC_DAUCA Aspartate aminotransferase, cytoplasmic (EC 2.6.1.1) (Transaminase A) - Daucus carota (Carrot)highly similar to ( 792) loc_os05g13950 12005.m083655 protein eukaryotic translation initiation factor 3 subunit 6-interacting protein, putative, expressed no original description T

27.3.71 RNA.regulation of transcription.SNF7 MDP0000230990 moderately similar to ( 260) AT1G03950 | Symbols: VPS2.3 | VPS2.3 (VACUOLAR PROTEIN SORTING-ASSOCIATED PROTEIN 2.3) | chr1:1011388-1013212 REVERSEmoderately similar to ( 248) loc_os10g33660 12010.m06194 protein charged multivesicular body protein 2b, putative, expressed no original description T

29.2.1.1.1.2.14 protein.synthesis.ribosomal protein.prokaryotic.chloroplast.50S subunit.L14 MDP0000231430 moderately similar to ( 281) AT3G04400 | Symbols: emb2171 | emb2171 (embryo defective 2171); structural constituent of ribosome | chr3:1167339-1168308 FORWARDmoderately similar to ( 279) RL23_TOBAC 60S ribosomal protein L23 - Nicotiana tabacum (Common tobacco)moderately similar to ( 281) loc_os10g32920 12010.m06128 protein 60S ribosomal protein L23, putative, expressed no original description T

17.1.1.1.10 hormone metabolism.abscisic acid.synthesis-degradation.synthesis.9-cis-epoxycarotenoid dioxygenase MDP0000164529 highly similar to ( 877) AT3G63520 | Symbols: CCD1, ATCCD1, ATNCED1, NCED1 | CCD1 (CAROTENOID CLEAVAGE DIOXYGENASE 1); 9-cis-epoxycarotenoid dioxygenase | chr3:23452940-23455896 FORWARDhighly similar to ( 834) loc_os12g44310 12012.m26998 protein 9,10-9,10 carotenoid cleavage dioxygenase 1, putative, expressed no original description T

27.3.3 RNA.regulation of transcription.AP2/EREBP, APETALA2/Ethylene-responsive element binding protein family MDP0000871361 moderately similar to ( 244) AT3G57600 | Symbols: | AP2 domain-containing transcription factor, putative | chr3:21332841-21333674 FORWARDvery weakly similar to (62.8) ERF1_ORYSA Ethylene-responsive transcription factor 1 (Ethylene-responsive element-binding factor 1) (EREBP-1) (OsEREBP1) - Oryza sativa (Rice)weakly similar to ( 186) loc_os03g07830 12003.m06295 protein dehydration-responsive element-binding protein 2F, putative no original description T

4.3.5 glycolysis.unclear/dually targeted.pyrophosphate-fructose-6-P phosphotransferase MDP0000293776 highly similar to ( 794) AT1G12000 | Symbols: | pyrophosphate--fructose-6-phosphate 1-phosphotransferase beta subunit, putative / pyrophosphate-dependent 6-phosphofructose-1-kinase, putative | chr1:4050159-4053727 REVERSEhighly similar to ( 813) PFPB_RICCO Pyrophosphate--fructose 6-phosphate 1-phosphotransferase subunit beta (EC 2.7.1.90) (PFP) (6-phosphofructokinase, pyrophosphate dependent) (Pyrophosphate-dependent 6-phosphofructose-1-kinase) (PPi-PFK) - Ricinus communis (Castor bean)highly similar to ( 766) loc_os06g13810 12006.m31864 protein pyrophosphate--fructose 6-phosphate 1-phosphotransferase beta subunit, putative, expressed no original description T

4.2.4 glycolysis.plastid branch.phosphofructokinase (PFK) MDP0000293776 highly similar to ( 794) AT1G12000 | Symbols: | pyrophosphate--fructose-6-phosphate 1-phosphotransferase beta subunit, putative / pyrophosphate-dependent 6-phosphofructose-1-kinase, putative | chr1:4050159-4053727 REVERSEhighly similar to ( 813) PFPB_RICCO Pyrophosphate--fructose 6-phosphate 1-phosphotransferase subunit beta (EC 2.7.1.90) (PFP) (6-phosphofructokinase, pyrophosphate dependent) (Pyrophosphate-dependent 6-phosphofructose-1-kinase) (PPi-PFK) - Ricinus communis (Castor bean)highly similar to ( 766) loc_os06g13810 12006.m31864 protein pyrophosphate--fructose 6-phosphate 1-phosphotransferase beta subunit, putative, expressed no original description T

29.5.11.4.2 protein.degradation.ubiquitin.E3.RING MDP0000248895 weakly similar to ( 107) AT5G59350 | Symbols: | unknown protein | chr5:23941132-23941995 FORWARDvery weakly similar to (73.6) EL5_ORYSA E3 ubiquitin-protein ligase EL5 (EC 6.3.2.-) - Oryza sativa (Rice)weakly similar to ( 110) loc_os11g47730 12011.m28698 protein expressed protein no original description T

29.7 protein.glycosylation MDP0000666177 moderately similar to ( 332) AT1G27600 | Symbols: | glycosyl transferase family 43 protein | chr1:9604083-9605881 REVERSEmoderately similar to ( 307) loc_os01g48440 12001.m43143 protein galactosylgalactosylxylosylprotein 3-beta-glucuronosyltransferase 1, putative, expressed no original description T

34.3 transport.amino acids MDP0000328893 moderately similar to ( 306) AT2G39130 | Symbols: | amino acid transporter family protein | chr2:16323171-16326744 REVERSEmoderately similar to ( 257) loc_os02g01100 12002.m100064 protein amino acid permease, putative, expressed no original description T

35.2 not assigned.unknown MDP0000271350 moderately similar to ( 238) AT1G06190 | Symbols: | ATP binding / ATPase, coupled to transmembrane movement of ions, phosphorylative mechanism | chr1:1892589-1894078 REVERSEweakly similar to ( 185) BP73_ORYSA SAP-like protein BP-73 (OsBP-73) (Riaa1) - Oryza sativa (Rice)moderately similar to ( 234) loc_os04g01240 12004.m05444 protein expressed protein no original description T

29.3.4.99 protein.targeting.secretory pathway.unspecified MDP0000149551 moderately similar to ( 360) AT1G10630 | Symbols: ATARFA1F | ATARFA1F (ARABIDOPSIS THALIANA ADP-RIBOSYLATION FACTOR A1F); GTP binding / copper ion binding / phospholipase activator/ protein binding | chr1:3513189-3514230 REVERSEmoderately similar to ( 362) ARF_VIGUN ADP-ribosylation factor - Vigna unguiculata (Cowpea)moderately similar to ( 362) loc_os01g59790 12001.m43240 protein ADP-ribosylation factor, putative, expressed no original description T

3.8.2 minor CHO metabolism.galactose.alpha-galactosidases MDP0000253567 highly similar to ( 713) AT3G26380 | Symbols: | glycosyl hydrolase family protein 27 / alpha-galactosidase family protein / melibiase family protein | chr3:9660140-9663145 FORWARDvery weakly similar to (81.6) AGAL_ORYSA Alpha-galactosidase precursor (EC 3.2.1.22) (Melibiase) (Alpha-D-galactoside galactohydrolase) - Oryza sativa (Rice)highly similar to ( 597) loc_os01g33420 12001.m09656 protein alpha-galactosidase/ hydrolase, hydrolyzing O-glycosyl compounds, putative, expressed no original description T

21.2 redox.ascorbate and glutathione MDP0000232692 weakly similar to ( 127) AT5G53560 | Symbols: ATB5-A, B5 #2, ATCB5-E, CB5-E | CB5-E (CYTOCHROME B5 ISOFORM E); heme binding | chr5:21759628-21760353 FORWARDweakly similar to ( 125) CYB5_TOBAC Cytochrome b5 - Nicotiana tabacum (Common tobacco)weakly similar to ( 123) loc_os10g37420 12010.m21998 protein cytochrome b5, putative, expressed no original description T

20.2 stress.abiotic MDP0000506346 moderately similar to ( 493) AT1G16560 | Symbols: | Per1-like family protein | chr1:5669234-5670343 FORWARDmoderately similar to ( 465) loc_os05g13330 12005.m27743 protein CAB2, putative, expressed no original description T

29.3.3 protein.targeting.chloroplast MDP0000491236 highly similar to ( 796) AT5G20300 | Symbols: | chloroplast outer membrane protein, putative | chr5:6853544-6855541 REVERSEweakly similar to ( 105) TOC34_PEA Translocase of chloroplast 34 (EC 3.6.5.-) (34 kDa chloroplast outer envelope protein) (GTP-binding protein OEP34) (GTP-binding protein IAP34) - Pisum sativum (Garden pea)highly similar to ( 663) loc_os12g09570 12012.m26861 protein chloroplast outer envelope protein 86, putative, expressed no original description T

21.2 redox.ascorbate and glutathione MDP0000329095 moderately similar to ( 220) AT2G32720 | Symbols: B5 #4, ATCB5-B, CB5-B | CB5-B (CYTOCHROME B5 ISOFORM B); heme binding | chr2:13877013-13878447 REVERSEmoderately similar to ( 222) CYB5_TOBAC Cytochrome b5 - Nicotiana tabacum (Common tobacco)moderately similar to ( 204) loc_os05g01820 12005.m04718 protein cytochrome b5, putative, expressed no original description T

21.1 redox.thioredoxin MDP0000232591 moderately similar to ( 293) AT3G25580 | Symbols: | thioredoxin-related | chr3:9292557-9294148 FORWARDmoderately similar to ( 275) loc_os08g15204 12008.m26827 protein thioredoxin domain-containing protein 9, putative, expressed no original description T

20.1 stress.biotic MDP0000312812 moderately similar to ( 238) AT4G27190 | Symbols: | disease resistance protein (NBS-LRR class), putative | chr4:13620977-13623934 REVERSEmoderately similar to ( 252) loc_os11g29090 12011.m06821 protein NB-ARC domain containing protein no original description T

33.99 development.unspecified MDP0000926586 very weakly similar to (60.8) AT3G44735 | Symbols: PSK1, ATPSK3 | PSK1; growth factor | chr3:16291388-16292118 REVERSEvery weakly similar to (53.9) PSK5_ORYSA Phytosulfokines 5 precursor (Secretory protein SH27A) [Contains: Phytosulfokine-alpha (PSK-alpha) (Phytosulfokine-a); Phytosulfokine-beta (PSK-beta) (Phytosulfokine-b)] - Oryza sativa (Rice)very weakly similar to (53.9) loc_os12g05260 12012.m04520 protein phytosulfokines 5 precursor, putative, expressed no original description T

30.3 signalling.calcium MDP0000159130 nearly identical (1689) AT1G07670 | Symbols: ATECA4, ECA4 | calcium-transporting ATPase | chr1:2370305-2374196 REVERSEmoderately similar to ( 358) ACA5_ORYSA Probable calcium-transporting ATPase 5, plasma membrane-type (EC 3.6.3.8) (Ca(2+)-ATPase isoform 5) - Oryza sativa (Rice)nearly identical (1591) loc_os03g17310 12003.m07160 protein calcium-transporting ATPase 2, endoplasmic reticulum-type, putative, expressed no original description T

30.5 signalling.G-proteins MDP0000506551 highly similar to ( 993) AT3G43300 | Symbols: ATMIN7 | ATMIN7 (ARABIDOPSIS THALIANA HOPM INTERACTOR 7); guanyl-nucleotide exchange factor/ protein binding | chr3:15234235-15245034 REVERSEhighly similar to ( 929) loc_os07g37750 12007.m08025 protein guanyl-nucleotide exchange factor, putative, expressed no original description T

20.1 stress.biotic MDP0000951714 moderately similar to ( 361) AT1G18250 | Symbols: ATLP-1 | ATLP-1 | chr1:6277024-6278005 REVERSEmoderately similar to ( 206) TLP1_PRUPE Thaumatin-like protein 1 precursor (PpAZ44) - Prunus persica (Peach)moderately similar to ( 292) loc_os06g47600 12006.m09286 protein thaumatin-like protein precursor, putative, expressed no original description T

26.2 misc.UDP glucosyl and glucoronyl transferases MDP0000551467 moderately similar to ( 328) AT5G59580 | Symbols: UGT76E1 | UGT76E1 (UDP-GLUCOSYL TRANSFERASE 76E1); UDP-glycosyltransferase/ quercetin 3-O-glucosyltransferase/ quercetin 7-O-glucosyltransferase | chr5:24006239-24007689 REVERSEweakly similar to ( 147) ZOX_PHAVU Zeatin O-xylosyltransferase (EC 2.4.2.40) (Zeatin O-beta-D-xylosyltransferase) - Phaseolus vulgaris (Kidney bean) (French bean)moderately similar to ( 262) loc_os07g13634 12007.m05808 protein cytokinin-N-glucosyltransferase 1, putative, expressed no original description T

29.4 protein.postranslational modification MDP0000270117 highly similar to ( 535) AT5G27930 | Symbols: | protein phosphatase 2C, putative / PP2C, putative | chr5:9958199-9960219 REVERSEmoderately similar to ( 416) loc_os05g29030 12005.m07189 protein catalytic/ protein phosphatase type 2C, putative, expressed no original description T

35.2 not assigned.unknown MDP0000420727 highly similar to ( 653) AT3G19540 | Symbols: | unknown protein | chr3:6780762-6782633 FORWARDhighly similar to ( 608) loc_os12g39160 12012.m07701 protein expressed protein no original description T

30.5 signalling.G-proteins MDP0000129000 moderately similar to ( 364) AT5G54840 | Symbols: SGP1 | SGP1; GTP binding | chr5:22277361-22278328 REVERSEvery weakly similar to (69.3) YPTM1_MAIZE GTP-binding protein YPTM1 - Zea mays (Maize)moderately similar to ( 345) loc_os07g33850 12007.m07643 protein septum-promoting GTP-binding protein 1, putative, expressed no original description T

31.4 cell.vesicle transport MDP0000696104 highly similar to ( 725) AT1G31730 | Symbols: | epsilon-adaptin, putative | chr1:11359907-11363916 FORWARDhighly similar to ( 667) loc_os01g68750 12001.m12977 protein clathrin binding protein, putative, expressed no original description T

27.3.23 RNA.regulation of transcription.HSF,Heat-shock transcription factor family MDP0000155667 moderately similar to ( 228) AT5G62020 | Symbols: AT-HSFB2A, HSFB2A | AT-HSFB2A; DNA binding / transcription factor | chr5:24916212-24917194 FORWARDmoderately similar to ( 216) loc_os08g43334 12008.m61974 protein heat shock factor protein 7, putative, expressed no original description T

35.2 not assigned.unknown MDP0000259468 moderately similar to ( 242) AT1G34350 | Symbols: | unknown protein | chr1:12534585-12535927 FORWARDmoderately similar to ( 205) loc_os08g01350 12008.m26548 protein transmembrane protein 18, putative, expressed no original description T

10.1.5 cell wall.precursor synthesis.UXS MDP0000925883 highly similar to ( 613) AT3G46440 | Symbols: UXS5 | UXS5; UDP-glucuronate decarboxylase/ catalytic | chr3:17089268-17091611 REVERSEweakly similar to ( 104) GME2_ORYSA GDP-mannose 3,5-epimerase 2 (EC 5.1.3.18) (GDP-Man 3,5-epimerase 2) - Oryza sativa (Rice)highly similar to ( 597) loc_os03g16980 12003.m34836 protein UDP-glucuronic acid decarboxylase 1, putative, expressed no original description T

29.3.4.99 protein.targeting.secretory pathway.unspecified MDP0000024218 highly similar to ( 673) AT1G56590 | Symbols: | clathrin adaptor complexes medium subunit family protein | chr1:21202250-21204697 REVERSEhighly similar to ( 620) loc_os05g31780 12005.m27805 protein AP-3 complex subunit mu-2, putative, expressed no original description T

16.8.2 secondary metabolism.flavonoids.chalcones MDP0000252589 moderately similar to ( 230) AT5G05270 | Symbols: | chalcone-flavanone isomerase family protein | chr5:1563543-1564827 FORWARDvery weakly similar to (55.1) CFI_VITVI Chalcone--flavonone isomerase (EC 5.5.1.6) (Chalcone isomerase) - Vitis vinifera (Grape)moderately similar to ( 213) loc_os11g02440 12011.m28712 protein chalcone--flavonone isomerase, putative, expressed no original description T

35.1 not assigned.no ontology MDP0000231182 moderately similar to ( 231) AT2G40600 | Symbols: | appr-1-p processing enzyme family protein | chr2:16947012-16948537 REVERSEweakly similar to ( 191) loc_os03g21830 12003.m07587 protein protein LRP16, putative, expressed no original description T

13.2.5.2 amino acid metabolism.degradation.serine-glycine-cysteine group.glycine MDP0000805894 highly similar to ( 733) AT1G11860 | Symbols: | aminomethyltransferase, putative | chr1:4001801-4003245 FORWARDhighly similar to ( 741) GCST_SOLTU Aminomethyltransferase, mitochondrial precursor (EC 2.1.2.10) (Glycine cleavage system T protein) (GCVT) - Solanum tuberosum (Potato)highly similar to ( 655) loc_os04g53230 12004.m10214 protein aminomethyltransferase, mitochondrial precursor, putative, expressed no original description T

35.1.5 not assigned.no ontology.pentatricopeptide (PPR) repeat-containing protein MDP0000290485 highly similar to ( 810) AT4G33400 | Symbols: | dem protein-related / defective embryo and meristems protein-related | chr4:16078189-16080410 REVERSEweakly similar to ( 164) CB12_PETHY Chlorophyll a-b binding protein, chloroplast precursor (LHCI type II CAB) - Petunia hybrida (Petunia)highly similar to ( 785) loc_os02g08190 12002.m06165 protein protein CYPRO4, putative, expressed no original description T

35.2 not assigned.unknown MDP0000323047 no original description T

29.5.11.4.2 protein.degradation.ubiquitin.E3.RING MDP0000451740 highly similar to ( 807) AT1G29340 | Symbols: PUB17, ATPUB17 | PUB17 (PLANT U-BOX 17); ubiquitin-protein ligase | chr1:10264412-10266601 FORWARDmoderately similar to ( 231) SPL11_ORYSA Spotted leaf protein 11 (Spotted leaf11) (Cell death-related protein SPL11) - Oryza sativa (Rice)moderately similar to ( 425) loc_os02g13960 12002.m06642 protein spotted leaf protein 11, putative, expressed no original description T

27.3.67 RNA.regulation of transcription.putative transcription regulator MDP0000313603 moderately similar to ( 422) AT2G29570 | Symbols: PCNA2, ATPCNA2 | PCNA2 (PROLIFERATING CELL NUCLEAR ANTIGEN 2); DNA binding / DNA polymerase processivity factor | chr2:12650139-12651598 REVERSEmoderately similar to ( 442) PCNA_TOBAC Proliferating cell nuclear antigen (PCNA) - Nicotiana tabacum (Common tobacco)moderately similar to ( 416) loc_os02g56130 12002.m10637 protein proliferating cell nuclear antigen, putative, expressed no original description T

29.5.9 protein.degradation.AAA type MDP0000207573 nearly identical (1068) AT3G19740 | Symbols: | ATP binding / ATPase/ nucleoside-triphosphatase/ nucleotide binding | chr3:6855944-6862930 REVERSEweakly similar to ( 147) CDC48_CAPAN Cell division cycle protein 48 homolog - Capsicum annuum (Bell pepper)highly similar to ( 949) loc_os06g12160 12006.m05933 protein ATP binding protein, putative, expressed no original description T

24.2 Biodegradation of Xenobiotics.lactoylglutathione lyase MDP0000407382 weakly similar to ( 166) AT1G15380 | Symbols: | lactoylglutathione lyase family protein / glyoxalase I family protein | chr1:5290955-5292287 FORWARDweakly similar to ( 162) loc_os01g07850 12001.m07412 protein lactoylglutathione lyase, putative no original description T

30.3 signalling.calcium MDP0000389244 moderately similar to ( 285) AT5G40190 | Symbols: | calmodulin-binding protein | chr5:16069298-16069849 REVERSEweakly similar to ( 195) loc_os09g20860 12009.m05293 protein calmodulin binding protein, putative, expressed no original description T

26.6 misc.O-methyl transferases MDP0000140067 moderately similar to ( 421) AT4G31790 | Symbols: | diphthine synthase, putative (DPH5) | chr4:15377479-15378530 FORWARDmoderately similar to ( 398) loc_os03g23970 12003.m35236 protein diphthine synthase, putative, expressed no original description T

35.2 not assigned.unknown MDP0000286732 moderately similar to ( 356) AT3G06670 | Symbols: | binding | chr3:2105908-2113136 REVERSEmoderately similar to ( 261) loc_os01g23590 12001.m08832 protein expressed protein MSP1_C no original description T

8.2.9 TCA / org transformation.other organic acid transformatons.cyt MDH MDP0000170418 moderately similar to ( 496) AT1G04410 | Symbols: | malate dehydrogenase, cytosolic, putative | chr1:1189418-1191267 REVERSEhighly similar to ( 507) MDHC_MAIZE Malate dehydrogenase, cytoplasmic (EC 1.1.1.37) - Zea mays (Maize)moderately similar to ( 498) loc_os10g33800 12010.m21921 protein malate dehydrogenase, cytoplasmic, putative, expressed no original description T

29.4 protein.postranslational modification MDP0000151935 highly similar to ( 617) AT4G28880 | Symbols: ckl3 | ckl3 (Casein Kinase I-like 3); ATP binding / kinase/ protein kinase/ protein serine/threonine kinase | chr4:14251351-14254048 FORWARDvery weakly similar to (67.4) CDPK_DAUCA Calcium-dependent protein kinase (EC 2.7.11.1) (CDPK) - Daucus carota (Carrot)highly similar to ( 560) loc_os02g17910 12002.m33640 protein casein kinase I isoform delta-like, putative, expressed no original description T

34.3 transport.amino acids MDP0000089124 highly similar to ( 781) AT2G38120 | Symbols: AUX1, WAV5, PIR1, MAP1 | AUX1 (AUXIN RESISTANT 1); amino acid transmembrane transporter/ auxin binding / auxin influx transmembrane transporter/ transporter | chr2:15973493-15976792 FORWARDhighly similar to ( 807) LAX2_MEDTR Auxin transporter-like protein 2 (AUX1-like protein 2) (MtLAX2) - Medicago truncatula (Barrel medic)highly similar to ( 771) loc_os01g63770 12001.m43433 protein auxin transporter-like protein 1, putative, expressed no original description T

29.3.1 protein.targeting.nucleus MDP0000303332 weakly similar to ( 150) AT1G71480 | Symbols: | nuclear transport factor 2 (NTF2) family protein | chr1:26932079-26933086 FORWARDweakly similar to ( 148) loc_os09g09320 12009.m21974 protein retrotransposon protein, putative, unclassified, expressed no original description T

17.7.1.4 hormone metabolism.jasmonate.synthesis-degradation.allene oxidase cyclase MDP0000899281 moderately similar to ( 268) AT1G13280 | Symbols: AOC4 | AOC4 (ALLENE OXIDE CYCLASE 4); allene-oxide cyclase | chr1:4547624-4548552 FORWARDmoderately similar to ( 256) loc_os03g32314 12003.m08473 protein allene oxide cyclase 4, chloroplast precursor, putative, expressed no original description T

31.1 cell.organisation MDP0000137341 moderately similar to ( 284) AT1G52080 | Symbols: AR791 | AR791; actin binding | chr1:19369788-19371862 FORWARDmoderately similar to ( 233) loc_os05g09380 12005.m083641 protein AR791, putative, expressed no original description T

35.2 not assigned.unknown MDP0000130219 moderately similar to ( 234) AT5G04830 | Symbols: | unknown protein | chr5:1402294-1403671 REVERSEmoderately similar to ( 215) loc_os01g73500 12001.m13378 protein expressed protein no original description T

17.4.2 hormone metabolism.cytokinin.signal transduction MDP0000155347 nearly identical (1329) AT1G27320 | Symbols: AHK3 | AHK3 (ARABIDOPSIS HISTIDINE KINASE 3); cytokinin receptor/ osmosensor/ protein histidine kinase | chr1:9487780-9492027 FORWARDweakly similar to ( 139) NTF2_ORYSA Nuclear transport factor 2 (NTF-2) - Oryza sativa (Rice)nearly identical (1126) loc_os01g69920 12001.m42798 protein histidine kinase 2, putative, expressed no original description T

35.2 not assigned.unknown MDP0000829294 moderately similar to ( 497) AT1G20410 | Symbols: | unknown protein | chr1:7079049-7082504 REVERSEmoderately similar to ( 428) loc_os04g47380 12004.m09683 protein RNA-binding protein, containing THUMP domain, putative, expressed no original description T

29.3.4.99 protein.targeting.secretory pathway.unspecified MDP0000703330 moderately similar to ( 328) AT1G14820 | Symbols: | SEC14 cytosolic factor family protein / phosphoglyceride transfer family protein | chr1:5105237-5106454 REVERSEweakly similar to ( 144) loc_os01g70210 12001.m13069 protein phosphatidylinositol transfer protein CSR1, putative, expressed no original description T

13.1.4.1 amino acid metabolism.synthesis.branched chain group.common MDP0000234363 highly similar to ( 757) AT3G58610 | Symbols: | ketol-acid reductoisomerase | chr3:21671561-21674639 FORWARDhighly similar to ( 771) ILV5_PEA Ketol-acid reductoisomerase, chloroplast precursor (EC 1.1.1.86) (Acetohydroxy-acid reductoisomerase) (Alpha-keto-beta-hydroxylacil reductoisomerase) - Pisum sativum (Garden pea)highly similar to ( 761) loc_os05g49800 12005.m09050 protein ketol-acid reductoisomerase, chloroplast precursor, putative, expressed no original description T

29.1.3 protein.aa activation.threonine-tRNA ligase MDP0000566415 moderately similar to ( 338) AT2G04842 | Symbols: EMB2761 | EMB2761 (EMBRYO DEFECTIVE 2761); ATP binding / aminoacyl-tRNA ligase/ ligase, forming aminoacyl-tRNA and related compounds / nucleotide binding / threonine-tRNA ligase | chr2:1698466-1701271 REVERSEmoderately similar to ( 327) loc_os02g33500 12002.m33712 protein threonyl-tRNA synthetase, putative, expressed no original description T

16.1.2.1 secondary metabolism.isoprenoids.mevalonate pathway.acetyl-CoA C-acyltransferase MDP0000231084 highly similar to ( 602) AT5G48230 | Symbols: EMB1276, ACAT2 | ACAT2 (ACETOACETYL-COA THIOLASE 2); acetyl-CoA C-acetyltransferase/ catalytic | chr5:19552570-19555030 REVERSEhighly similar to ( 564) loc_os09g07830 12009.m059940 protein acetyl-CoA acetyltransferase, cytosolic 1, putative, expressed no original description T

35.1 not assigned.no ontology MDP0000318284 very weakly similar to (99.8) AT5G04820 | Symbols: ATOFP13, OFP13 | OFP13 (ARABIDOPSIS THALIANA OVATE FAMILY PROTEIN 13) | chr5:1399685-1400467 FORWARDvery weakly similar to (85.5) loc_os05g36990 12005.m07880 protein plant-specific domain TIGR01568 family protein, expressed no original description T

34.16 transport.ABC transporters and multidrug resistance systems MDP0000234709 nearly identical (2296) AT1G04120 | Symbols: ATMRP5, MRP5 | ATMRP5; ATPase, coupled to transmembrane movement of substances / sulfonylurea receptor | chr1:1064848-1070396 REVERSEweakly similar to ( 181) MDR_ORYSA Putative multidrug resistance protein (P-glycoprotein) - Oryza sativa (Rice)nearly identical (2036) loc_os03g04920 12003.m06018 protein multidrug resistance-associated protein 5, putative, expressed TrbL no original description T

35.2 not assigned.unknown MDP0000147066 moderately similar to ( 438) AT5G19050 | Symbols: | unknown protein | chr5:6369307-6371726 FORWARDmoderately similar to ( 394) loc_os07g11060 12007.m28958 protein expressed protein no original description T

27.3.32 RNA.regulation of transcription.WRKY domain transcription factor family MDP0000496268 moderately similar to ( 236) AT2G30590 | Symbols: WRKY21 | WRKY21; calmodulin binding / transcription factor | chr2:13033891-13035303 FORWARDweakly similar to ( 193) loc_os12g40570 12012.m07841 protein WRKY transcription factor 21, putative, expressed no original description T

29.2.1.2.1.17 protein.synthesis.ribosomal protein.eukaryotic.40S subunit.S17 MDP0000313341 moderately similar to ( 225) AT5G04800 | Symbols: | 40S ribosomal protein S17 (RPS17D) | chr5:1389217-1389642 FORWARDmoderately similar to ( 212) loc_os03g01900 12003.m05734 protein 40S ribosomal protein S17, putative, expressed no original description T

29.3.1 protein.targeting.nucleus MDP0000259553 nearly identical (1532) AT2G31660 | Symbols: SAD2, URM9 | SAD2 (SUPER SENSITIVE TO ABA AND DROUGHT2); binding / protein transporter | chr2:13464519-13471353 FORWARDnearly identical (1161) loc_os04g26841 12004.m07810 protein importin-8, putative no original description T

29.5.11.4.2 protein.degradation.ubiquitin.E3.RING MDP0000222238 moderately similar to ( 315) AT3G05545 | Symbols: | transcription factor, putative / zinc finger (C3HC4 type RING finger) family protein | chr3:1609436-1612133 FORWARDmoderately similar to ( 264) loc_os05g06270 12005.m28008 protein VIP2 protein, putative, expressed no original description T

20.2.3 stress.abiotic.drought/salt MDP0000138427 highly similar to ( 983) AT4G14360 | Symbols: | dehydration-responsive protein-related | chr4:8267869-8270191 REVERSEhighly similar to ( 931) loc_os10g33720 12010.m21920 protein ankyrin-like protein, putative, expressed no original description T

20.2.99 stress.abiotic.unspecified MDP0000427722 weakly similar to ( 184) AT1G24020 | Symbols: MLP423 | MLP423 (MLP-LIKE PROTEIN 423) | chr1:8500653-8501458 REVERSEvery weakly similar to (82.0) RNS1_PANGI Ribonuclease 1 (EC 3.1.-.-) - Panax ginseng (Korean ginseng)weakly similar to ( 166) loc_os04g39150 12004.m35333 protein major latex protein 22, putative, expressed no original description T

27.2 RNA.transcription MDP0000831937 highly similar to ( 627) AT5G24120 | Symbols: SIGE, SIG5, ATSIG5 | SIGE (SIGMA FACTOR E); DNA binding / DNA-directed RNA polymerase/ sigma factor/ transcription factor | chr5:8157794-8159746 REVERSEmoderately similar to ( 456) loc_os05g50930 12005.m083829 protein RNA polymerase sigma factor rpoD, putative, expressedPRK07003 no original description T

4.1.13 glycolysis.cytosolic branch.enolase MDP0000939989 highly similar to ( 750) AT2G36530 | Symbols: LOS2 | LOS2; copper ion binding / phosphopyruvate hydratase | chr2:15321081-15323786 REVERSEhighly similar to ( 781) ENO_RICCO Enolase (EC 4.2.1.11) (2-phosphoglycerate dehydratase) (2-phospho-D-glycerate hydro-lyase) - Ricinus communis (Castor bean)highly similar to ( 763) loc_os03g14450 12003.m101271 protein enolase 2, putative, expressed no original description T

35.2 not assigned.unknown MDP0000791532 highly similar to ( 625) AT5G01620 | Symbols: | unknown protein | chr5:232882-234821 FORWARDmoderately similar to ( 384) loc_os03g60350 12003.m10924 protein expressed protein no original description T

27.3.11 RNA.regulation of transcription.C2H2 zinc finger family MDP0000254206 moderately similar to ( 366) AT3G52200 | Symbols: LTA3 | LTA3; ATP binding / dihydrolipoyllysine-residue acetyltransferase | chr3:19360317-19366091 FORWARDvery weakly similar to (71.6) ZFP1_WHEAT Zinc-finger protein 1 (WZF1) - Triticum aestivum (Wheat)moderately similar to ( 204) loc_os06g30460 12006.m091708 protein dihydrolipoyllysine-residue acetyltransferase component of pyruvatedehydrogenase complex, mitochondrial precursor, putative, expressed no original description T

29.2.1.2.1.12 protein.synthesis.ribosomal protein.eukaryotic.40S subunit.S12 MDP0000152496 weakly similar to ( 188) AT2G32060 | Symbols: | 40S ribosomal protein S12 (RPS12C) | chr2:13639228-13640104 REVERSEmoderately similar to ( 208) RS12_HORVU 40S ribosomal protein S12 - Hordeum vulgare (Barley)moderately similar to ( 214) loc_os07g12650 12007.m079809 protein 40S ribosomal protein S12, putative, expressed no original description T

31.1 cell.organisation MDP0000262534 highly similar to ( 564) AT3G04710 | Symbols: | ankyrin repeat family protein | chr3:1278229-1280942 FORWARDvery weakly similar to (73.6) STIP_SOYBN Heat shock protein STI (Stress-inducible protein) (GmSTI) - Glycine max (Soybean)highly similar to ( 508) loc_os05g01310 12005.m27662 protein ankyrin-1, putative, expressed no original description T

29.2.2.3.99 protein.synthesis.ribosome biogenesis.Pre-rRNA processing and modifications.misc MDP0000262685 nearly identical (1021) AT1G42440 | Symbols: | FUNCTIONS IN: molecular_function unknown; INVOLVED IN: ribosome biogenesis; LOCATED IN: nucleus; EXPRESSED IN: 22 plant structures; EXPRESSED DURING: 13 growth stages; CONTAINS InterPro DOMAIN/s: AARP2CN (InterPro:IPR012948), Protein of unknown function DUF663 (InterPro:IPR007034); BEST Arabidopsis thaliana protein match is: unknown protein (TAIR:AT1G06720.1); Has 2447 Blast hits to 1812 proteins in 205 species: Archae - 0; Bacteria - 115; Metazoa - 903; Fungi - 423; Plants - 100; Viruses - 53; Other Eukaryotes - 853 (source: NCBI BLink). | chr1:15895528-15899939 REVERSEhighly similar to ( 932) loc_os11g19250 12011.m05999 protein expressed protein no original description T

29.2.2.2.2 protein.synthesis.ribosome biogenesis.Assembly factors.GTPases MDP0000262685 nearly identical (1021) AT1G42440 | Symbols: | FUNCTIONS IN: molecular_function unknown; INVOLVED IN: ribosome biogenesis; LOCATED IN: nucleus; EXPRESSED IN: 22 plant structures; EXPRESSED DURING: 13 growth stages; CONTAINS InterPro DOMAIN/s: AARP2CN (InterPro:IPR012948), Protein of unknown function DUF663 (InterPro:IPR007034); BEST Arabidopsis thaliana protein match is: unknown protein (TAIR:AT1G06720.1); Has 2447 Blast hits to 1812 proteins in 205 species: Archae - 0; Bacteria - 115; Metazoa - 903; Fungi - 423; Plants - 100; Viruses - 53; Other Eukaryotes - 853 (source: NCBI BLink). | chr1:15895528-15899939 REVERSEhighly similar to ( 932) loc_os11g19250 12011.m05999 protein expressed protein no original description T

33.99 development.unspecified MDP0000299377 nearly identical (1835) AT3G05680 | Symbols: EMB2016 | EMB2016 (embryo defective 2016) | chr3:1660802-1672015 REVERSEhighly similar to ( 832) loc_os03g35340 12003.m08675 protein expressed protein no original description T

29.2.1.2.2.14 protein.synthesis.ribosomal protein.eukaryotic.60S subunit.L14 MDP0000641747 moderately similar to ( 224) AT2G20450 | Symbols: | 60S ribosomal protein L14 (RPL14A) | chr2:8813923-8815071 FORWARDmoderately similar to ( 221) RL14_PEA Probable 60 ribosomal protein L14 (Hydroxyproline-rich glycoprotein HRGP1) - Pisum sativum (Garden pea)moderately similar to ( 233) loc_os04g43540 12004.m09314 protein 60 ribosomal protein L14, putative, expressed no original description T

26.4.1 misc.beta 1,3 glucan hydrolases.glucan endo-1,3-beta-glucosidase MDP0000275579 weakly similar to ( 170) AT3G56290 | Symbols: | unknown protein | chr3:20878743-20879541 REVERSEvery weakly similar to (96.7) GUB_NICPL Lichenase precursor (EC 3.2.1.73) (Endo-beta-1,3-1,4 glucanase) - Nicotiana plumbaginifolia (Leadwort-leaved tobacco)weakly similar to ( 168) loc_os01g60830 12001.m12211 protein expressed protein no original description T

26.6 misc.O-methyl transferases MDP0000152520 highly similar to ( 538) AT1G04870 | Symbols: PRMT10, ATPRMT10 | PRMT10; [myelin basic protein]-arginine N-methyltransferase/ histone-arginine N-methyltransferase/ methyltransferase/ protein-arginine omega-N asymmetric methyltransferase/ protein-arginine omega-N monomethyltransferase | chr1:1373485-1375598 REVERSEhighly similar to ( 511) loc_os06g05090 12006.m091600 protein methyltransferase, putative, expressed no original description T

29.2.1.2.2.32 protein.synthesis.ribosomal protein.eukaryotic.60S subunit.L32 MDP0000208709 moderately similar to ( 228) AT4G18100 | Symbols: | 60S ribosomal protein L32 (RPL32A) | chr4:10035715-10036475 REVERSEvery weakly similar to (73.2) RL32_MAIZE 60S ribosomal protein L32 (Fragment) - Zea mays (Maize)moderately similar to ( 245) loc_os08g41300 12008.m08108 protein 60S ribosomal protein L32, putative, expressed no original description T

31.2 cell.division MDP0000159598 highly similar to ( 593) AT3G52750 | Symbols: FTSZ2-2 | FTSZ2-2; GTP binding / GTPase/ structural molecule | chr3:19549841-19552435 REVERSEhighly similar to ( 537) loc_os03g44420 12003.m09481 protein cell division protein ftsZ, putative, expressed no original description T

29.5.11.4.2 protein.degradation.ubiquitin.E3.RING MDP0000762575 weakly similar to ( 165) AT3G23280 | Symbols: | zinc finger (C3HC4-type RING finger) family protein / ankyrin repeat family protein | chr3:8321588-8324109 FORWARDweakly similar to ( 163) loc_os07g26490 12007.m079670 protein protein binding protein, putative, expressed no original description T

29.5.11.1 protein.degradation.ubiquitin.ubiquitin MDP0000209028 moderately similar to ( 443) AT2G17200 | Symbols: | ubiquitin family protein | chr2:7482133-7485090 REVERSEmoderately similar to ( 455) loc_os03g03920 12003.m101175 protein ubiquilin-1, putative, expressed no original description T

35.2 not assigned.unknown MDP0000120095 moderately similar to ( 406) AT5G14790 | Symbols: | binding | chr5:4784058-4785508 FORWARDmoderately similar to ( 378) loc_os02g20310 12002.m100182 protein HEAT repeat family protein, expressed no original description T

27.3.40 RNA.regulation of transcription.Aux/IAA family MDP0000295589 moderately similar to ( 229) AT1G04240 | Symbols: SHY2, IAA3 | SHY2 (SHORT HYPOCOTYL 2); transcription factor | chr1:1128564-1129319 REVERSEmoderately similar to ( 226) AX22B_PHAAU Auxin-induced protein 22B (Indole-3-acetic acid-induced protein ARG4) - Phaseolus aureus (Mung bean) (Vigna radiata)weakly similar to ( 172) loc_os12g40900 12012.m07873 protein OsIAA31 - Auxin-responsive Aux/IAA gene family member, expressed no original description T

27.3.67 RNA.regulation of transcription.putative transcription regulator MDP0000124449 moderately similar to ( 436) AT1G04020 | Symbols: ATBARD1, BARD1 | BARD1 (BREAST CANCER ASSOCIATED RING 1); DNA binding / transcription coactivator | chr1:1036610-1040045 FORWARDmoderately similar to ( 358) loc_os04g43300 12004.m09294 protein ATBRCA1, putative, expressed no original description T

27.3.21 RNA.regulation of transcription.GRAS transcription factor family MDP0000575908 highly similar to ( 625) AT5G66770 | Symbols: | scarecrow transcription factor family protein | chr5:26660723-26662477 FORWARDweakly similar to ( 199) CIGR2_ORYSA Chitin-inducible gibberellin-responsive protein 2 - Oryza sativa (Rice)moderately similar to ( 433) loc_os03g51330 12003.m10114 protein DELLA protein SLR1, putative, expressed no original description T

33.99 development.unspecified MDP0000575908 highly similar to ( 625) AT5G66770 | Symbols: | scarecrow transcription factor family protein | chr5:26660723-26662477 FORWARDweakly similar to ( 199) CIGR2_ORYSA Chitin-inducible gibberellin-responsive protein 2 - Oryza sativa (Rice)moderately similar to ( 433) loc_os03g51330 12003.m10114 protein DELLA protein SLR1, putative, expressed no original description T

20.1.7 stress.biotic.PR-proteins MDP0000316559 moderately similar to ( 309) AT5G66900 | Symbols: | disease resistance protein (CC-NBS-LRR class), putative | chr5:26714931-26717757 REVERSEmoderately similar to ( 232) loc_os12g39620 12012.m073872 protein disease resistance protein, putative, expressed no original description T

8.1.1.1 TCA / org transformation.TCA.pyruvate DH.E1 MDP0000146411 highly similar to ( 613) AT5G50850 | Symbols: MAB1 | MAB1 (MACCI-BOU); catalytic/ pyruvate dehydrogenase (acetyl-transferring) | chr5:20689671-20692976 FORWARDhighly similar to ( 606) ODPB_PEA Pyruvate dehydrogenase E1 component subunit beta, mitochondrial precursor (EC 1.2.4.1) (PDHE1-B) - Pisum sativum (Garden pea)highly similar to ( 641) loc_os08g42410 12008.m080219 protein pyruvate dehydrogenase E1 component subunit beta, mitochondrial precursor, putative, expressed no original description T

26.2 misc.UDP glucosyl and glucoronyl transferases MDP0000210431 highly similar to ( 876) AT5G47780 | Symbols: GAUT4 | GAUT4 (Galacturonosyltransferase 4); polygalacturonate 4-alpha-galacturonosyltransferase/ transferase, transferring glycosyl groups | chr5:19347991-19350517 FORWARDhighly similar to ( 802) loc_os08g23780 12008.m06398 protein transferase, transferring glycosyl groups, putative, expressed no original description T

28.1.3 DNA.synthesis/chromatin structure.histone MDP0000759996 weakly similar to ( 181) AT1G54690 | Symbols: HTA3, H2AXB, G-H2AX, GAMMA-H2AX | GAMMA-H2AX (GAMMA HISTONE VARIANT H2AX); DNA binding | chr1:20414607-20415206 REVERSEweakly similar to ( 188) H2AX_CICAR Histone H2AX - Cicer arietinum (Chickpea) (Garbanzo)weakly similar to ( 174) loc_os12g34510 12012.m07245 protein histone H2A, putative, expressed no original description T

30.5 signalling.G-proteins MDP0000155158 highly similar to ( 616) AT5G05940 | Symbols: ATROPGEF5, ROPGEF5 | ROPGEF5 (ROP GUANINE NUCLEOTIDE EXCHANGE FACTOR 5); Rho guanyl-nucleotide exchange factor | chr5:1786028-1788363 FORWARDhighly similar to ( 554) loc_os01g62990 12001.m12417 protein pollen-specific kinase partner protein, putative, expressed MSP1_C no original description T

29.5.11.5 protein.degradation.ubiquitin.ubiquitin protease MDP0000128587 nearly identical (1179) AT3G20630 | Symbols: UBP14, TTN6, ATUBP14 | UBP14 (UBIQUITIN-SPECIFIC PROTEASE 14); ubiquitin-specific protease | chr3:7203001-7208340 REVERSEnearly identical (1087) loc_os01g08200 12001.m07445 protein ubiquitin carboxyl-terminal hydrolase 14, putative, expressed no original description T

35.2 not assigned.unknown MDP0000230088 moderately similar to ( 258) AT5G03420 | Symbols: | FUNCTIONS IN: molecular_function unknown; INVOLVED IN: biological_process unknown; LOCATED IN: cellular_component unknown; EXPRESSED IN: 22 plant structures; EXPRESSED DURING: 13 growth stages; BEST Arabidopsis thaliana protein match is: 5'-AMP-activated protein kinase-related (TAIR:AT1G27070.1); Has 1365 Blast hits to 895 proteins in 182 species: Archae - 6; Bacteria - 436; Metazoa - 414; Fungi - 132; Plants - 56; Viruses - 2; Other Eukaryotes - 319 (source: NCBI BLink). | chr5:845641-848705 FORWARDmoderately similar to ( 242) loc_os03g04470 12003.m34722 protein expressed protein TrbL no original description T

35.2 not assigned.unknown MDP0000779067 very weakly similar to (71.2) AT1G52565 | Symbols: | unknown protein | chr1:19580061-19580511 REVERSE no original description T

27.3.68 RNA.regulation of transcription.PWWP domain protein MDP0000498180 moderately similar to ( 201) AT3G54760 | Symbols: | dentin sialophosphoprotein-related | chr3:20269659-20272037 REVERSEmoderately similar to ( 290) loc_os05g38810 12005.m08060 protein PWWP domain containing protein, expressed no original description T

35.2 not assigned.unknown MDP0000143680 highly similar to ( 612) AT5G50150 | Symbols: | unknown protein | chr5:20402444-20406375 REVERSEhighly similar to ( 541) loc_os06g28000 12006.m07345 protein carboxyl-terminal proteinase, putative, expressed no original description T

29.3.4.99 protein.targeting.secretory pathway.unspecified MDP0000262734 moderately similar to ( 327) AT3G62290 | Symbols: ATARFA1E | ATARFA1E (ADP-ribosylation factor A1E); GTP binding / phospholipase activator/ protein binding | chr3:23052287-23053545 FORWARDmoderately similar to ( 323) ARF2_ORYSA ADP-ribosylation factor 2 - Oryza sativa (Rice)moderately similar to ( 326) loc_os07g12200 12007.m05673 protein ADP-ribosylation factor 1, putative, expressed no original description T

13.1.2.3.21 amino acid metabolism.synthesis.glutamate family.arginine.ornithine carbamoyltransferase MDP0000290884 highly similar to ( 526) AT1G75330 | Symbols: OTC | OTC (ORNITHINE CARBAMOYLTRANSFERASE); amino acid binding / carboxyl- or carbamoyltransferase/ ornithine carbamoyltransferase | chr1:28266457-28268383 REVERSEhighly similar to ( 531) OTC_PEA Ornithine carbamoyltransferase, chloroplast precursor (EC 2.1.3.3) (OTCase) (Ornithine transcarbamylase) - Pisum sativum (Garden pea)highly similar to ( 509) loc_os02g47590 12002.m09793 protein ornithine carbamoyltransferase, chloroplast precursor, putative, expressed no original description T

11.1.1.2.4 lipid metabolism.FA synthesis and FA elongation.Acetyl CoA Carboxylation.heteromeric Complex.Biotin Carboxylase MDP0000321496 highly similar to ( 846) AT5G35360 | Symbols: CAC2 | CAC2; acetyl-CoA carboxylase/ biotin carboxylase | chr5:13584300-13587827 FORWARDmoderately similar to ( 420) MCCA_ORYSA Methylcrotonoyl-CoA carboxylase subunit alpha, mitochondrial precursor (EC 6.4.1.4) (3-methylcrotonyl-CoA carboxylase 1) (MCCase subunit alpha) (3-methylcrotonyl-CoA:carbon dioxide ligase subunit alpha) - Oryza sativa (Rice)moderately similar to ( 420) loc_os12g41250 12012.m26967 protein methylcrotonoyl-CoA carboxylase subunit alpha, mitochondrial precursor, putative, expressed no original description T

26.10 misc.cytochrome P450 MDP0000827657 moderately similar to ( 497) AT4G31940 | Symbols: CYP82C4 | CYP82C4; electron carrier/ heme binding / iron ion binding / monooxygenase/ oxygen binding | chr4:15452040-15453966 FORWARDhighly similar to ( 572) C82A3_SOYBN Cytochrome P450 82A3 (EC 1.14.-.-) (P450 CP6) - Glycine max (Soybean)moderately similar to ( 347) loc_os09g26960 12009.m05848 protein flavonoid 3-monooxygenase, putative, expressed no original description T

35.2 not assigned.unknown MDP0000590105 no original description T

35.2 not assigned.unknown MDP0000235858 no original description T

34.16 transport.ABC transporters and multidrug resistance systems MDP0000257415 highly similar to ( 968) AT2G28070 | Symbols: | ABC transporter family protein | chr2:11956432-11959782 FORWARDweakly similar to ( 119) PDR15_ORYSA Pleiotropic drug resistance protein 15 - Oryza sativa (Rice)highly similar to ( 792) loc_os03g64200 12003.m101582 protein ATPase, coupled to transmembrane movement of substances, putative, expressed no original description T

29.7 protein.glycosylation MDP0000284475 moderately similar to ( 406) AT1G20575 | Symbols: | dolichyl-phosphate beta-D-mannosyltransferase, putative / dolichol-phosphate mannosyltransferase, putative / mannose-P-dolichol synthase, putative | chr1:7126987-7128677 REVERSEmoderately similar to ( 383) loc_os03g60939 12003.m35486 protein dolichol-phosphate mannosyltransferase, putative, expressed no original description T

34.1.1 transport.p- and v-ATPases.H+-transporting two-sector ATPase MDP0000123144 weakly similar to ( 168) AT1G19910 | Symbols: AVA-P2, AVA-2PE, ATVHA-C2 | AVA-P2; ATPase/ proton-transporting ATPase, rotational mechanism | chr1:6913317-6914322 FORWARDweakly similar to ( 169) VATL_GOSHI Vacuolar ATP synthase 16 kDa proteolipid subunit (EC 3.6.3.14) - Gossypium hirsutum (Upland cotton)weakly similar to ( 166) loc_os11g06890 12011.m28741 protein vacuolar ATP synthase 16 kDa proteolipid subunit, putative, expressed no original description T

20.2.99 stress.abiotic.unspecified MDP0000678612 weakly similar to ( 143) AT1G68300 | Symbols: | universal stress protein (USP) family protein | chr1:25598518-25599261 REVERSEweakly similar to ( 136) loc_os01g32780 12001.m09597 protein ethylene-responsive protein, putative, expressed no original description T

29.5.7 protein.degradation.metalloprotease MDP0000227554 highly similar to ( 617) AT5G35690 | Symbols: | LOCATED IN: cellular_component unknown; CONTAINS InterPro DOMAIN/s: WLM (InterPro:IPR013536), PUG (InterPro:IPR006567); BEST Arabidopsis thaliana protein match is: zinc ion binding (TAIR:AT1G55915.1); Has 335 Blast hits to 326 proteins in 115 species: Archae - 0; Bacteria - 24; Metazoa - 37; Fungi - 138; Plants - 58; Viruses - 2; Other Eukaryotes - 76 (source: NCBI BLink). | chr5:13865027-13868117 REVERSEhighly similar to ( 594) loc_os08g39150 12008.m07897 protein expressed protein no original description T

20.2.99 stress.abiotic.unspecified MDP0000295908 weakly similar to ( 177) AT5G15780 | Symbols: | pollen Ole e 1 allergen and extensin family protein | chr5:5144898-5146297 REVERSEvery weakly similar to (68.6) loc_os10g13850 12010.m04638 protein expressed protein no original description T

35.2 not assigned.unknown MDP0000946670 very weakly similar to (99.8) AT1G03170 | Symbols: | unknown protein | chr1:769805-770527 FORWARDvery weakly similar to (76.3) loc_os03g49830 12003.m09981 protein expressed protein TrbL no original description T

30.2.99 signalling.receptor kinases.misc MDP0000031416 highly similar to ( 919) AT5G16000 | Symbols: NIK1 | NIK1 (NSP-INTERACTING KINASE 1); kinase | chr5:5224264-5227003 FORWARDmoderately similar to ( 276) PSKR_DAUCA Phytosulfokine receptor precursor (EC 2.7.11.1) (Phytosulfokine LRR receptor kinase) - Daucus carota (Carrot)highly similar to ( 804) loc_os06g16330 12006.m06346 protein BRASSINOSTEROID INSENSITIVE 1-associated receptor kinase 1 precursor, putative, expressed no original description T

27.3.70 RNA.regulation of transcription.Silencing Group MDP0000693646 moderately similar to ( 236) AT1G66740 | Symbols: AtSP7, SP7, SGA2, ASF1A | SGA2 | chr1:24892586-24893548 FORWARDmoderately similar to ( 231) loc_os05g48030 12005.m27955 protein anti-silencing protein 1, putative, expressed no original description T

35.1 not assigned.no ontology MDP0000237666 nearly identical (1233) AT5G19820 | Symbols: emb2734 | emb2734 (embryo defective 2734); binding / lyase | chr5:6695731-6701247 REVERSEnearly identical (1095) loc_os07g38760 12007.m08121 protein importin beta-3, putative, expressed no original description T

11.10.1 lipid metabolism.glycolipid synthesis.MGDG synthase MDP0000244574 highly similar to ( 712) AT4G31780 | Symbols: MGD1, MGDA | MGD1 (MONOGALACTOSYL DIACYLGLYCEROL SYNTHASE 1); 1,2-diacylglycerol 3-beta-galactosyltransferase/ UDP-galactosyltransferase/ UDP-glycosyltransferase/ transferase, transferring glycosyl groups | chr4:15374222-15376873 FORWARDhighly similar to ( 696) loc_os09g25580 12009.m05710 protein MGDG synthase type A, putative, expressed TrbL no original description T

29.2.1.2.2.34 protein.synthesis.ribosomal protein.eukaryotic.60S subunit.L34 MDP0000142785 weakly similar to ( 109) AT1G26880 | Symbols: | 60S ribosomal protein L34 (RPL34A) | chr1:9315798-9316681 REVERSEweakly similar to ( 111) RL34_PEA 60S ribosomal protein L34 - Pisum sativum (Garden pea)weakly similar to ( 105) loc_os09g24690 12009.m05623 protein 60S ribosomal protein L34, putative, expressed no original description T

35.2 not assigned.unknown MDP0000225543 weakly similar to ( 143) AT4G25670 | Symbols: | unknown protein | chr4:13085431-13085997 REVERSEweakly similar to ( 119) loc_os02g01360 12002.m05486 protein expressed protein no original description T

31.3 cell.cycle MDP0000310564 moderately similar to ( 240) AT1G70210 | Symbols: CYCD1;1 | CYCD1;1 (CYCLIN D1;1); cyclin-dependent protein kinase regulator | chr1:26440015-26441980 FORWARDweakly similar to ( 148) loc_os09g21450 12009.m05352 protein cyclin delta-2, putative, expressed no original description T

35.2 not assigned.unknown MDP0000133633 weakly similar to ( 137) AT4G28025 | Symbols: | unknown protein | chr4:13935836-13937367 REVERSEweakly similar to ( 105) loc_os02g39730 12002.m33303 protein expressed protein no original description T

29.2.1.2.1.515 protein.synthesis.ribosomal protein.eukaryotic.40S subunit.S15A MDP0000182870 moderately similar to ( 251) AT5G59850 | Symbols: | 40S ribosomal protein S15A (RPS15aF) | chr5:24112499-24113084 REVERSEmoderately similar to ( 248) RS15A_BRANA 40S ribosomal protein S15a (PPCB8) - Brassica napus (Rape)moderately similar to ( 247) loc_os02g27760 12002.m07920 protein 40S ribosomal protein S15a, putative, expressed no original description T

17.2.3 hormone metabolism.auxin.induced-regulated-responsive-activated MDP0000263867 highly similar to ( 606) AT1G04690 | Symbols: KAB1, KV-BETA1 | KAB1 (POTASSIUM CHANNEL BETA SUBUNIT); oxidoreductase/ potassium channel | chr1:1313662-1315420 FORWARDhighly similar to ( 582) KCAB_ORYSA Probable voltage-gated potassium channel subunit beta (K(+) channel subunit beta) - Oryza sativa (Rice)highly similar to ( 582) loc_os02g57240 12002.m10746 protein voltage-gated potassium channel beta subunit, putative, expressed no original description T

3.5 minor CHO metabolism.others MDP0000263867 highly similar to ( 606) AT1G04690 | Symbols: KAB1, KV-BETA1 | KAB1 (POTASSIUM CHANNEL BETA SUBUNIT); oxidoreductase/ potassium channel | chr1:1313662-1315420 FORWARDhighly similar to ( 582) KCAB_ORYSA Probable voltage-gated potassium channel subunit beta (K(+) channel subunit beta) - Oryza sativa (Rice)highly similar to ( 582) loc_os02g57240 12002.m10746 protein voltage-gated potassium channel beta subunit, putative, expressed no original description T

29.2.1.2.2.9 protein.synthesis.ribosomal protein.eukaryotic.60S subunit.L9 MDP0000263658 moderately similar to ( 322) AT1G33140 | Symbols: PGY2 | PGY2 (PIGGYBACK2); structural constituent of ribosome | chr1:12023360-12024502 FORWARDmoderately similar to ( 318) RL9_PEA 60S ribosomal protein L9 (Gibberellin-regulated protein GA) - Pisum sativum (Garden pea)moderately similar to ( 301) loc_os02g01332 12002.m77755 protein 60S ribosomal protein L9, putative, expressed COG3889 no original description T

26.16 misc.myrosinases-lectin-jacalin MDP0000168857 weakly similar to ( 187) AT5G01090 | Symbols: | legume lectin family protein | chr5:33055-34116 FORWARDvery weakly similar to (66.6) loc_os06g17490 12006.m06461 protein protein kinase, putative, expressed no original description T

35.2 not assigned.unknown MDP0000320025 weakly similar to ( 157) AT5G47830 | Symbols: | unknown protein | chr5:19373013-19374300 REVERSEweakly similar to ( 135) loc_os02g06390 12002.m05987 protein expressed protein no original description T

27.4 RNA.RNA binding MDP0000126558 weakly similar to ( 178) AT3G26420 | Symbols: ATRZ-1A | ATRZ-1A; RNA binding / nucleotide binding | chr3:9671953-9673055 FORWARDweakly similar to ( 119) GRP2_SINAL Glycine-rich RNA-binding protein GRP2A - Sinapis alba (White mustard) (Brassica hirta)weakly similar to ( 156) loc_os03g61990 12003.m101556 protein glycine-rich RNA-binding protein 8, putative, expressed no original description T

20.2.1 stress.abiotic.heat MDP0000308285 highly similar to ( 592) AT2G29970 | Symbols: | heat shock protein-related | chr2:12776601-12779784 FORWARDvery weakly similar to (60.1) HS101_ORYSA Heat shock protein 101 - Oryza sativa (Rice)highly similar to ( 542) loc_os11g01330 12011.m04338 protein expressed protein no original description T

35.1 not assigned.no ontology MDP0000288274 moderately similar to ( 447) AT1G21070 | Symbols: | transporter-related | chr1:7376148-7377810 REVERSEmoderately similar to ( 438) loc_os07g39280 12007.m08170 protein integral membrane protein like, putative, expressed no original description T

35.1 not assigned.no ontology MDP0000179533 highly similar to ( 503) AT4G09810 | Symbols: | transporter-related | chr4:6175415-6176892 REVERSEmoderately similar to ( 458) loc_os05g12490 12005.m05713 protein integral membrane protein like, putative, expressed no original description T

35.1 not assigned.no ontology MDP0000126232 moderately similar to ( 445) AT1G25375 | Symbols: | metallo-beta-lactamase family protein | chr1:8900279-8903220 REVERSEmoderately similar to ( 423) loc_os01g04200 12001.m150474 protein metallo-beta-lactamase superfamily protein, expressed no original description T

31.2 cell.division MDP0000197068 moderately similar to ( 370) AT5G03340 | Symbols: | cell division cycle protein 48, putative / CDC48, putative | chr5:810091-813133 REVERSEmoderately similar to ( 370) CDC48_SOYBN Cell division cycle protein 48 homolog (Valosin-containing protein homolog) (VCP) - Glycine max (Soybean)moderately similar to ( 360) loc_os03g05730 12003.m06095 protein cell division control protein 48 homolog E, putative, expressed no original description T

35.2 not assigned.unknown MDP0000121380 moderately similar to ( 274) AT3G61870 | Symbols: | unknown protein | chr3:22902702-22903895 FORWARDmoderately similar to ( 271) loc_os02g03010 12002.m05651 protein expressed protein no original description T

31.4 cell.vesicle transport MDP0000125037 very weakly similar to (85.5) AT4G32760 | Symbols: | protein transporter | chr4:15799376-15803832 FORWARDvery weakly similar to (77.8) loc_os06g22650 12006.m06919 protein VHS and GAT domain protein, putative, expressed no original description T

11.1.12 lipid metabolism.FA synthesis and FA elongation.ACP protein MDP0000201048 weakly similar to ( 159) AT2G44620 | Symbols: MTACP-1, MTACP1 | MTACP-1 (MITOCHONDRIAL ACYL CARRIER PROTEIN 1); acyl carrier/ cofactor binding / phosphopantetheine binding | chr2:18414320-18415065 FORWARDweakly similar to ( 132) loc_os07g12150 12007.m05668 protein acyl carrier protein, mitochondrial precursor, putative, expressed no original description T

35.1 not assigned.no ontology MDP0000844309 highly similar to ( 625) AT4G12650 | Symbols: | LOCATED IN: integral to membrane, Golgi apparatus, plasma membrane, vacuole; EXPRESSED IN: 25 plant structures; EXPRESSED DURING: 13 growth stages; CONTAINS InterPro DOMAIN/s: Nonaspanin (TM9SF) (InterPro:IPR004240); BEST Arabidopsis thaliana protein match is: unknown protein (TAIR:AT5G35160.1); Has 983 Blast hits to 980 proteins in 161 species: Archae - 0; Bacteria - 0; Metazoa - 434; Fungi - 144; Plants - 228; Viruses - 0; Other Eukaryotes - 177 (source: NCBI BLink). | chr4:7468207-7470165 REVERSEhighly similar to ( 622) loc_os08g38820 12008.m080200 protein transmembrane 9 superfamily protein member 2 precursor, putative, expressed no original description T

16.1.2.4 secondary metabolism.isoprenoids.mevalonate pathway.mevalonate kinase MDP0000056935 moderately similar to ( 452) AT5G27450 | Symbols: MVK, MK | MK (MEVALONATE KINASE); mevalonate kinase | chr5:9691051-9692975 FORWARDmoderately similar to ( 413) loc_os10g18220 12010.m04902 protein mevalonate kinase, putative, expressed no original description T

27.2 RNA.transcription MDP0000524927 nearly identical (1684) AT3G49500 | Symbols: RDR6, SGS2, SDE1 | RDR6 (RNA-DEPENDENT RNA POLYMERASE 6); RNA-directed RNA polymerase/ nucleic acid binding | chr3:18349193-18353205 REVERSEnearly identical (1433) loc_os01g34350 12001.m09741 protein RNA-dependent RNA polymerase homolog 1, putative, expressed no original description T

29.5.4 protein.degradation.aspartate protease MDP0000318500 highly similar to ( 748) AT5G60160 | Symbols: | aspartyl aminopeptidase, putative | chr5:24223887-24226783 REVERSEvery weakly similar to (91.3) APEA_BORBU Probable M18-family aminopeptidase 1 (EC 3.4.11.-) - Borrelia burgdorferi (Lyme disease spirochete)highly similar to ( 715) loc_os12g13390 12012.m073796 protein aspartyl aminopeptidase, putative, expressed no original description T

29.4 protein.postranslational modification MDP0000314872 highly similar to ( 516) AT3G17510 | Symbols: CIPK1, SnRK3.16 | CIPK1 (CBL-INTERACTING PROTEIN KINASE 1); kinase/ protein binding | chr3:5989309-5992627 REVERSEmoderately similar to ( 370) CIPK1_ORYSA CIPK-like protein 1 (EC 2.7.11.1) (OsCK1) - Oryza sativa (Rice)moderately similar to ( 483) loc_os01g18800 12001.m150556 protein CBL-interacting serine/threonine-protein kinase 1, putative, expressed no original description T

28.1 DNA.synthesis/chromatin structure MDP0000293040 highly similar to ( 516) AT5G27740 | Symbols: EMB2775 | EMB2775 (EMBRYO DEFECTIVE 2775); DNA binding / nucleoside-triphosphatase/ nucleotide binding | chr5:9823831-9826869 FORWARDmoderately similar to ( 492) loc_os03g57870 12003.m10699 protein replication factor C subunit 5, putative, expressed no original description T

10.5.5 cell wall.cell wall proteins.RGP MDP0000811808 weakly similar to ( 162) AT3G08900 | Symbols: RGP3, RGP | RGP3 (REVERSIBLY GLYCOSYLATED POLYPEPTIDE 3); transferase, transferring hexosyl groups | chr3:2708347-2709714 REVERSEweakly similar to ( 170) UPTG2_SOLTU Alpha-1,4-glucan-protein synthase [UDP-forming] 2 (EC 2.4.1.112) (UDP-glucose:protein transglucosylase 2) (UPTG 2) - Solanum tuberosum (Potato)weakly similar to ( 171) loc_os07g41360 12007.m08372 protein alpha-1,4-glucan-protein synthase 1, putative, expressed no original description T

35.2 not assigned.unknown MDP0000231385 nearly identical (2292) AT5G51200 | Symbols: | unknown protein | chr5:20804926-20819408 FORWARDnearly identical (1256) loc_os02g10790 12002.m100709 protein expressed protein no original description T

9.7 mitochondrial electron transport / ATP synthesis.cytochrome c oxidase MDP0000336861 very weakly similar to (94.4) AT4G37830 | Symbols: | cytochrome c oxidase-related | chr4:17787672-17788762 REVERSEvery weakly similar to (76.6) loc_os03g56190 12003.m10530 protein expressed protein no original description T

30.11 signalling.light MDP0000385091 highly similar to ( 528) AT5G65540 | Symbols: | unknown protein | chr5:26195710-26198112 FORWARDmoderately similar to ( 292) loc_os03g58780 12003.m34945 protein expressed protein no original description T

35.2 not assigned.unknown MDP0000321155 nearly identical (1023) AT5G49830 | Symbols: | EXPRESSED IN: 22 plant structures; EXPRESSED DURING: 13 growth stages; CONTAINS InterPro DOMAIN/s: Vps51/Vps67 (InterPro:IPR014812); BEST Arabidopsis thaliana protein match is: unknown protein (TAIR:AT1G10385.1). | chr5:20250803-20254766 REVERSEhighly similar to ( 921) loc_os07g10110 12007.m05466 protein expressed protein no original description T

26.8 misc.nitrilases, *nitrile lyases, berberine bridge enzymes, reticuline oxidases, troponine reductases MDP0000291707 moderately similar to ( 237) AT1G07440 | Symbols: | tropinone reductase, putative / tropine dehydrogenase, putative | chr1:2286700-2287665 REVERSEvery weakly similar to (70.5) GRDH_ORYSA Glucose and ribitol dehydrogenase homolog (EC 1.1.1.-) - Oryza sativa (Rice)moderately similar to ( 217) loc_os03g16230 12003.m34832 protein tropinone reductase, putative, expressed no original description T

31.3 cell.cycle MDP0000471541 very weakly similar to (80.5) AT3G21870 | Symbols: CYCP2;1 | CYCP2;1 (cyclin p2;1); cyclin-dependent protein kinase | chr3:7703927-7704813 REVERSE no original description T

35.2 not assigned.unknown MDP0000319422 moderately similar to ( 304) AT5G06990 | Symbols: | unknown protein | chr5:2169699-2170484 FORWARDmoderately similar to ( 232) loc_os01g59200 12001.m12057 protein plant-specific domain TIGR01570 family protein, expressed no original description T

33.99 development.unspecified MDP0000465844 moderately similar to ( 358) AT2G39210 | Symbols: | nodulin family protein | chr2:16366287-16368231 REVERSEmoderately similar to ( 315) loc_os03g58580 12003.m10763 protein nitrate and chloride transporter, putative, expressed no original description T

34.19.1 transport.Major Intrinsic Proteins.PIP MDP0000479436 highly similar to ( 504) AT4G00430 | Symbols: TMP-C, PIP1;4, PIP1E | PIP1;4 (PLASMA MEMBRANE INTRINSIC PROTEIN 1;4); water channel | chr4:186143-187531 REVERSEhighly similar to ( 501) PIP11_VICFA Aquaporin PIP1.1 (Plasma membrane intrinsic protein 1a) (PIP1a) (Aquaporin 1) (Plasma membrane aquaporin 1) - Vicia faba (Broad bean)moderately similar to ( 499) loc_os02g44630 12002.m33812 protein aquaporin PIP1.1, putative, expressed no original description T

35.1 not assigned.no ontology MDP0000430391 moderately similar to ( 280) AT3G24420 | Symbols: | hydrolase, alpha/beta fold family protein | chr3:8863111-8864883 REVERSEmoderately similar to ( 212) loc_os03g10620 12003.m35082 protein sigma factor sigB regulation protein rsbQ, putative, expressed no original description T

29.2.1.2.2.15 protein.synthesis.ribosomal protein.eukaryotic.60S subunit.L15 MDP0000149936 moderately similar to ( 360) AT4G16720 | Symbols: | 60S ribosomal protein L15 (RPL15A) | chr4:9400156-9401315 REVERSEmoderately similar to ( 355) RL15_PETHY 60S ribosomal protein L15 - Petunia hybrida (Petunia)moderately similar to ( 359) loc_os05g19370 12005.m06340 protein 60S ribosomal protein L15, putative, expressed no original description T

35.2 not assigned.unknown MDP0000272832 moderately similar to ( 338) AT1G32120 | Symbols: | unknown protein | chr1:11552926-11558608 FORWARDmoderately similar to ( 216) loc_os02g21280 12002.m07368 protein expressed protein no original description T

27.3.47 RNA.regulation of transcription.ELF3 MDP0000272100 moderately similar to ( 387) AT2G25930 | Symbols: ELF3, PYK20 | ELF3 (EARLY FLOWERING 3); protein C-terminus binding / transcription factor | chr2:11059459-11063178 FORWARDmoderately similar to ( 288) loc_os06g05060 12006.m05235 protein early flowering 3, putative, expressed no original description T

35.1.41 not assigned.no ontology.hydroxyproline rich proteins MDP0000177378 moderately similar to ( 483) AT4G18570 | Symbols: | proline-rich family protein | chr4:10231439-10234534 FORWARDmoderately similar to ( 391) loc_os01g46340 12001.m10832 protein pherophorin like protein, putative, expressed no original description T

29.5.11.4.2 protein.degradation.ubiquitin.E3.RING MDP0000167786 moderately similar to ( 253) AT1G73760 | Symbols: | zinc finger (C3HC4-type RING finger) family protein | chr1:27739366-27741161 REVERSEvery weakly similar to (51.2) EL5_ORYSA E3 ubiquitin-protein ligase EL5 (EC 6.3.2.-) - Oryza sativa (Rice)weakly similar to ( 179) loc_os01g49770 12001.m11162 protein protein binding protein, putative, expressed no original description T

17.7.1.2 hormone metabolism.jasmonate.synthesis-degradation.lipoxygenase MDP0000178268 very weakly similar to ( 100) AT3G45140 | Symbols: LOX2, ATLOX2 | LOX2 (LIPOXYGENASE 2); lipoxygenase | chr3:16525437-16529233 FORWARDweakly similar to ( 101) LOX23_HORVU Lipoxygenase 2.3, chloroplast precursor (EC 1.13.11.12) (LOX2:Hv:3) - Hordeum vulgare (Barley)weakly similar to ( 106) loc_os02g10120 12002.m06309 protein lipoxygenase 2.3, chloroplast precursor, putative, expressed no original description T

29.4.1.57 protein.postranslational modification.kinase.receptor like cytoplasmatic kinase VII MDP0000303573 highly similar to ( 658) AT1G80870 | Symbols: | protein kinase family protein | chr1:30392133-30394211 FORWARDweakly similar to ( 122) CRI4_MAIZE Putative receptor protein kinase CRINKLY4 precursor (EC 2.7.11.1) - Zea mays (Maize)moderately similar to ( 241) loc_os08g17320 12008.m05851 protein ATP binding protein, putative, expressed no original description T

27.3.99 RNA.regulation of transcription.unclassified MDP0000199093 moderately similar to ( 443) AT1G67340 | Symbols: | zinc finger (MYND type) family protein / F-box family protein | chr1:25230323-25231622 FORWARDmoderately similar to ( 369) loc_os01g69270 12001.m13027 protein MYND finger family protein, expressed no original description T

35.1 not assigned.no ontology MDP0000156100 highly similar to ( 688) AT1G19100 | Symbols: | ATP-binding region, ATPase-like domain-containing protein-related | chr1:6595560-6601159 FORWARDmoderately similar to ( 499) loc_os01g36840 12001.m150620 protein MORC family CW-type zinc finger 3, putative, expressed no original description T

10.7 cell wall.modification MDP0000120539 weakly similar to ( 113) AT4G28250 | Symbols: ATEXPB3, EXPB3, ATHEXP BETA 1.6 | ATEXPB3 (ARABIDOPSIS THALIANA EXPANSIN B3) | chr4:14000446-14001945 REVERSEweakly similar to ( 101) EXB17_ORYSA Expansin-B17 precursor (OsEXPB17) (Beta-expansin-17) (OsaEXPb1.13) - Oryza sativa (Rice)very weakly similar to ( 100) loc_os02g42650 12002.m09300 protein beta-expansin 3 precursor, putative, expressed no original description T

19.2 tetrapyrrole synthesis.glu-tRNA reductase MDP0000656101 highly similar to ( 669) AT1G58290 | Symbols: HEMA1 | HEMA1; glutamyl-tRNA reductase | chr1:21624028-21626051 REVERSEhighly similar to ( 689) HEM11_CUCSA Glutamyl-tRNA reductase 1, chloroplast precursor (EC 1.2.1.70) (GluTR) - Cucumis sativus (Cucumber)highly similar to ( 599) loc_os10g35840 12010.m06402 protein glutamyl-tRNA reductase, chloroplast precursor, putative, expressed no original description T

29.5.11.4.2 protein.degradation.ubiquitin.E3.RING MDP0000797453 weakly similar to ( 158) AT4G03510 | Symbols: RMA1 | RMA1; protein binding / ubiquitin-protein ligase/ zinc ion binding | chr4:1557905-1558654 REVERSEweakly similar to ( 105) loc_os01g56070 12001.m150727 protein RING finger protein 5, putative, expressed no original description T

27.4 RNA.RNA binding MDP0000379391 moderately similar to ( 354) AT2G25970 | Symbols: | KH domain-containing protein | chr2:11071844-11075604 REVERSEmoderately similar to ( 278) loc_os02g13130 12002.m100758 protein DNA-directed RNA polymerase, putative, expressed no original description T

29.4.1.57 protein.postranslational modification.kinase.receptor like cytoplasmatic kinase VII MDP0000188157 highly similar to ( 578) AT5G59010 | Symbols: | protein kinase-related | chr5:23820578-23822499 REVERSEweakly similar to ( 111) CRI4_MAIZE Putative receptor protein kinase CRINKLY4 precursor (EC 2.7.11.1) - Zea mays (Maize)moderately similar to ( 481) loc_os03g04050 12003.m05937 protein ATP binding protein, putative, expressed no original description T

27.4 RNA.RNA binding MDP0000129321 moderately similar to ( 205) AT5G64200 | Symbols: ATSC35, SC35 | ATSC35; RNA binding / nucleic acid binding / nucleotide binding | chr5:25681849-25683553 REVERSEvery weakly similar to (59.3) ROC1_NICSY 29 kDa ribonucleoprotein A, chloroplast precursor (CP29A) - Nicotiana sylvestris (Wood tobacco)moderately similar to ( 205) loc_os07g43050 12007.m079757 protein splicing factor, arginine/serine-rich 2, putative, expressed no original description T

29.4 protein.postranslational modification MDP0000207028 weakly similar to ( 176) AT5G50200 | Symbols: WR3, ATNRT3.1, NRT3.1 | WR3 (WOUND-RESPONSIVE 3); nitrate transmembrane transporter | chr5:20436612-20437535 FORWARDweakly similar to ( 159) loc_os02g38230 12002.m08911 protein component of high affinity nitrate transporter, putative, expressed no original description T

34.4 transport.nitrate MDP0000207028 weakly similar to ( 176) AT5G50200 | Symbols: WR3, ATNRT3.1, NRT3.1 | WR3 (WOUND-RESPONSIVE 3); nitrate transmembrane transporter | chr5:20436612-20437535 FORWARDweakly similar to ( 159) loc_os02g38230 12002.m08911 protein component of high affinity nitrate transporter, putative, expressed no original description T

27.2 RNA.transcription MDP0000311504 weakly similar to ( 126) AT2G29540 | Symbols: ATRPC14, ATRPAC14 | ATRPC14 (RNA POLYMERASE 14 KDA SUBUNIT); DNA binding / DNA-directed RNA polymerase/ protein dimerization | chr2:12643288-12643828 FORWARDweakly similar to ( 158) loc_os12g09280 12012.m073788 protein DNA-directed RNA polymerases I and III 14 kDa polypeptide, putative, expressed no original description T

29.5.11.20 protein.degradation.ubiquitin.proteasom MDP0000141365 moderately similar to ( 427) AT5G42790 | Symbols: PAF1, ATPSM30 | PAF1; endopeptidase/ peptidase/ threonine-type endopeptidase | chr5:17159270-17160975 REVERSEmoderately similar to ( 369) PSA1_ORYSA Proteasome subunit alpha type 1 (EC 3.4.25.1) (20S proteasome alpha subunit F) (20S proteasome subunit alpha-6) (Proteasome component C2) - Oryza sativa (Rice)moderately similar to ( 369) loc_os02g04100 12002.m05759 protein proteasome subunit alpha type 1, putative, expressed no original description T

34.99 transport.misc MDP0000221451 highly similar to ( 712) AT2G26510 | Symbols: PDE135 | PDE135 (pigment defective embryo 135); transmembrane transporter | chr2:11274118-11277464 FORWARDhighly similar to ( 632) loc_os12g39420 12012.m073719 protein solute carrier family 23 member 2, putative, expressed no original description T

26.2 misc.UDP glucosyl and glucoronyl transferases MDP0000950422 highly similar to ( 586) AT1G22360 | Symbols: AtUGT85A2 | AtUGT85A2 (UDP-glucosyl transferase 85A2); UDP-glycosyltransferase/ glucuronosyltransferase/ transferase, transferring glycosyl groups | chr1:7895068-7897527 REVERSEweakly similar to ( 178) UFO1_MAIZE Anthocyanidin 3-O-glucosyltransferase (EC 2.4.1.115) (Flavonol 3-O-glucosyltransferase) (UDP-glucose flavonoid 3-O-glucosyltransferase) (Bronze-1) (Bz-McC allele) - Zea mays (Maize)highly similar to ( 510) loc_os02g51910 12002.m10222 protein cytokinin-O-glucosyltransferase 2, putative, expressed TrbL MSP1_C no original description T

29.5.1 protein.degradation.subtilases MDP0000264894 highly similar to ( 949) AT5G45650 | Symbols: | subtilase family protein | chr5:18513520-18518790 REVERSEhighly similar to ( 852) loc_os01g52750 12001.m11448 protein subtilisin-like protease precursor, putative, expressed no original description T

29.2.1.2.2.14 protein.synthesis.ribosomal protein.eukaryotic.60S subunit.L14 MDP0000176124 moderately similar to ( 220) AT4G27090 | Symbols: | 60S ribosomal protein L14 (RPL14B) | chr4:13594104-13595187 REVERSEmoderately similar to ( 212) RL14_PEA Probable 60 ribosomal protein L14 (Hydroxyproline-rich glycoprotein HRGP1) - Pisum sativum (Garden pea)moderately similar to ( 225) loc_os04g43540 12004.m09314 protein 60 ribosomal protein L14, putative, expressed no original description T

29.5.11.4.2 protein.degradation.ubiquitin.E3.RING MDP0000186701 highly similar to ( 846) AT1G57820 | Symbols: VIM1, ORTH2 | VIM1 (VARIANT IN METHYLATION 1); DNA binding / chromatin binding / double-stranded methylated DNA binding / histone binding / methyl-CpG binding / methyl-CpNpG binding / methyl-CpNpN binding / ubiquitin-protein ligase | chr1:21414342-21417902 REVERSEhighly similar to ( 835) loc_os05g01230 12005.m04664 protein DNA binding protein, putative, expressed no original description T

20.1.7 stress.biotic.PR-proteins MDP0000139821 moderately similar to ( 321) AT5G66900 | Symbols: | disease resistance protein (CC-NBS-LRR class), putative | chr5:26714931-26717757 REVERSEmoderately similar to ( 279) loc_os12g39620 12012.m073872 protein disease resistance protein, putative, expressed no original description T

29.5.11.4.3.2 protein.degradation.ubiquitin.E3.SCF.FBOX MDP0000269848 highly similar to ( 858) AT2G44900 | Symbols: | armadillo/beta-catenin repeat family protein / F-box family protein | chr2:18511719-18515762 REVERSEmoderately similar to ( 352) loc_os10g41360 12010.m06900 protein ubiquitin-protein ligase, putative, expressed no original description T

20.2.1 stress.abiotic.heat MDP0000203499 nearly identical (1151) AT4G24190 | Symbols: SHD | SHD (SHEPHERD); ATP binding / unfolded protein binding | chr4:12551902-12555851 REVERSEnearly identical (1179) ENPL_CATRO Endoplasmin homolog precursor (GRP94 homolog) - Catharanthus roseus (Rosy periwinkle) (Madagascar periwinkle)highly similar to ( 767) loc_os06g50300 12006.m31934 protein endoplasmin homolog precursor, putative, expressed no original description T

29.2.1.2.1.12 protein.synthesis.ribosomal protein.eukaryotic.40S subunit.S12 MDP0000195426 very weakly similar to (92.8) AT2G32060 | Symbols: | 40S ribosomal protein S12 (RPS12C) | chr2:13639228-13640104 REVERSEvery weakly similar to ( 100) RS12_HORVU 40S ribosomal protein S12 - Hordeum vulgare (Barley)weakly similar to ( 106) loc_os07g12650 12007.m079809 protein 40S ribosomal protein S12, putative, expressed no original description T

16.1.4.3 secondary metabolism.isoprenoids.carotenoids.zeta-carotene desaturase MDP0000308095 highly similar to ( 900) AT3G04870 | Symbols: ZDS, PDE181 | ZDS (ZETA-CAROTENE DESATURASE); carotene 7,8-desaturase | chr3:1342842-1346189 FORWARDhighly similar to ( 937) ZDS_CAPAN Zeta-carotene desaturase, chloroplast precursor (EC 1.14.99.30) (Carotene 7,8-desaturase) - Capsicum annuum (Bell pepper)highly similar to ( 913) loc_os07g10490 12007.m05504 protein zeta-carotene desaturase, chloroplast precursor, putative, expressed no original description T

26.28 misc.GDSL-motif lipase MDP0000586088 weakly similar to ( 123) AT1G28580 | Symbols: | GDSL-motif lipase, putative | chr1:10044603-10045874 REVERSEvery weakly similar to (79.0) EST_HEVBR Esterase precursor (EC 3.1.1.-) (Early nodule-specific protein homolog) (Latex allergen Hev b 13) - Hevea brasiliensis (Para rubber tree)weakly similar to ( 114) loc_os01g11710 12001.m07788 protein esterase precursor, putative, expressed no original description T

27.3.67 RNA.regulation of transcription.putative transcription regulator MDP0000191009 weakly similar to ( 169) AT1G63480 | Symbols: | DNA-binding family protein | chr1:23539872-23541685 REVERSEweakly similar to ( 126) loc_os04g49990 12004.m35192 protein AT-hook protein 1, putative, expressed no original description T

9.7 mitochondrial electron transport / ATP synthesis.cytochrome c oxidase MDP0000155797 moderately similar to ( 331) AT1G28140 | Symbols: | unknown protein | chr1:9833029-9834390 REVERSEmoderately similar to ( 291) loc_os03g63360 12003.m11203 protein integral membrane protein, putative, expressed no original description T

35.2 not assigned.unknown MDP0000794528 no original description T

21.1 redox.thioredoxin MDP0000318969 highly similar to ( 716) AT5G03430 | Symbols: | phosphoadenosine phosphosulfate (PAPS) reductase family protein | chr5:849237-852867 REVERSEvery weakly similar to (59.7) TRXH_ORYSA Thioredoxin H-type (TRX-H) (Phloem sap 13 kDa protein 1) - Oryza sativa (Rice)highly similar to ( 655) loc_os01g15490 12001.m150544 protein transferase, putative, expressed no original description T

29.5.11.4.3.2 protein.degradation.ubiquitin.E3.SCF.FBOX MDP0000247014 moderately similar to ( 359) AT3G54480 | Symbols: SKIP5, SKP5 | SKIP5 (SKP1/ASK-INTERACTING PROTEIN 5) | chr3:20172518-20173325 REVERSE no original description T

35.2 not assigned.unknown MDP0000203270 highly similar to ( 691) AT2G47010 | Symbols: | unknown protein | chr2:19317505-19319252 FORWARDhighly similar to ( 605) loc_os05g19030 12005.m06307 protein expressed protein no original description T

35.2 not assigned.unknown MDP0000199155 very weakly similar to (72.8) AT3G52740 | Symbols: | unknown protein | chr3:19546446-19546868 REVERSEvery weakly similar to (64.3) loc_os04g33610 12004.m08456 protein expressed protein no original description T

16.1.2.2 secondary metabolism.isoprenoids.mevalonate pathway.HMG-CoA synthase MDP0000138071 highly similar to ( 729) AT4G11820 | Symbols: MVA1 | MVA1; acetyl-CoA C-acetyltransferase/ hydroxymethylglutaryl-CoA synthase | chr4:7109124-7111901 REVERSEhighly similar to ( 707) loc_os09g34960 12009.m06496 protein hydroxymethylglutaryl-CoA synthase, putative, expressed no original description T

1.1.1.2 PS.lightreaction.photosystem II.PSII polypeptide subunits MDP0000715912 moderately similar to ( 250) AT4G05180 | Symbols: PSBQ, PSBQ-2, PSII-Q | PSBQ-2; calcium ion binding | chr4:2672093-2673170 REVERSEmoderately similar to ( 252) PSBQ_SPIOL Oxygen-evolving enhancer protein 3, chloroplast precursor (OEE3) (16 kDa subunit of oxygen evolving system of photosystem II) (OEC 16 kDa subunit) - Spinacia oleracea (Spinach)weakly similar to ( 199) loc_os07g36080 12007.m079706 protein oxygen-evolving enhancer protein 3-1, chloroplast precursor, putative, expressed no original description T

35.2 not assigned.unknown MDP0000233319 weakly similar to ( 167) AT1G42480 | Symbols: | unknown protein | chr1:15936297-15938692 FORWARDweakly similar to ( 151) loc_os02g02524 12002.m100702 protein MIR-interacting saposin-like protein precursor, putative, expressed no original description T

13.1.3.4 amino acid metabolism.synthesis.aspartate family.methionine MDP0000793077 nearly identical (1277) AT5G17920 | Symbols: ATCIMS | ATMS1; 5-methyltetrahydropteroyltriglutamate-homocysteine S-methyltransferase/ methionine synthase | chr5:5935771-5939195 FORWARDnearly identical (1277) METE_CATRO 5-methyltetrahydropteroyltriglutamate--homocysteine methyltransferase (EC 2.1.1.14) (Vitamin-B12-independent methionine synthase isozyme) (Cobalamin-independent methionine synthase isozyme) - Catharanthus roseus (Rosy periwinkle) (Madagnearly identical (1271) loc_os12g42876 12012.m073894 protein 5-methyltetrahydropteroyltriglutamate--homocysteine methyltransferase, putative, expressed no original description T

29.2.2.50 protein.synthesis.ribosome biogenesis.BRIX MDP0000316217 moderately similar to ( 468) AT1G63780 | Symbols: IMP4 | IMP4 | chr1:23665045-23667243 REVERSEmoderately similar to ( 485) loc_os08g05880 12008.m04729 protein ribosome production factor 1, putative, expressed no original description T

35.2 not assigned.unknown MDP0000278894 weakly similar to ( 177) AT1G50020 | Symbols: | unknown protein | chr1:18520144-18521600 REVERSEweakly similar to ( 139) loc_os11g34870 12011.m07342 protein tubulin alpha-6 chain, putative, expressed no original description T

34.1 transport.p- and v-ATPases MDP0000319016 weakly similar to ( 190) AT2G07560 | Symbols: AHA6 | AHA6 (Arabidopsis H(+)-ATPase 6); ATPase | chr2:3170394-3173952 REVERSEweakly similar to ( 192) PMA1_ORYSA Plasma membrane ATPase (EC 3.6.3.6) (Proton pump) - Oryza sativa (Rice)weakly similar to ( 194) loc_os07g09340 12007.m05390 protein plasma membrane ATPase 1, putative, expressed no original description T

26.10 misc.cytochrome P450 MDP0000306273 highly similar to ( 776) AT5G23190 | Symbols: CYP86B1 | CYP86B1; electron carrier/ heme binding / iron ion binding / monooxygenase/ oxygen binding | chr5:7803478-7805659 REVERSEweakly similar to ( 121) C97B2_SOYBN Cytochrome P450 97B2 (EC 1.14.-.-) - Glycine max (Soybean)highly similar to ( 723) loc_os10g34480 12010.m06272 protein cytochrome P450 86A2, putative, expressed no original description T

35.2 not assigned.unknown MDP0000155747 moderately similar to ( 291) AT1G18490 | Symbols: | unknown protein | chr1:6367116-6368640 FORWARDmoderately similar to ( 236) loc_os08g03870 12008.m26565 protein cupin, RmlC-type, putative, expressed no original description T

31.3 cell.cycle MDP0000286130 moderately similar to ( 277) AT4G34160 | Symbols: CYCD3;1, CYCD3 | CYCD3;1 (CYCLIN D3;1); cyclin-dependent protein kinase regulator/ protein binding | chr4:16357903-16359304 FORWARDvery weakly similar to (54.7) CCNAL_DAUCA G2/mitotic-specific cyclin C13-1 (A-like cyclin) (Fragment) - Daucus carota (Carrot)weakly similar to ( 156) loc_os03g27420 12003.m08069 protein retrotransposon protein, putative, unclassified no original description T

29.2.4 protein.synthesis.elongation MDP0000903484 weakly similar to ( 135) AT2G18110 | Symbols: | elongation factor 1-beta, putative / EF-1-beta, putative | chr2:7872636-7873713 FORWARDweakly similar to ( 134) EF1B_ORYSA Elongation factor 1-beta (EF-1-beta) (Elongation factor 1B-alpha 2) (eEF-1B alpha) (Elongation factor 1-beta') (EF-1-beta') - Oryza sativa (Rice)weakly similar to ( 134) loc_os07g46750 12007.m08892 protein elongation factor 1-beta, putative, expressed no original description T

34.3 transport.amino acids MDP0000230263 moderately similar to ( 391) AT3G13620 | Symbols: | amino acid permease family protein | chr3:4450904-4452556 REVERSEmoderately similar to ( 341) loc_os03g25920 12003.m07936 protein cationic amino acid transporter, putative no original description T

35.2 not assigned.unknown MDP0000273325 no original description T

35.2 not assigned.unknown MDP0000947451 no original description T

27.1.19 RNA.processing.ribonucleases MDP0000941174 nearly identical (1281) AT1G75660 | Symbols: XRN3 | XRN3; 5'-3' exoribonuclease | chr1:28408289-28414825 FORWARDvery weakly similar to (95.5) CR2_HORVU Cold-regulated protein 2 (Fragment) - Hordeum vulgare (Barley)nearly identical (1366) loc_os01g65220 12001.m12635 protein XRN3, putative, expressed MSP1_C no original description T

15.1 metal handling.acquisition MDP0000742438 highly similar to ( 589) AT5G23980 | Symbols: ATFRO4, FRO4 | FRO4 (FERRIC REDUCTION OXIDASE 4); ferric-chelate reductase | chr5:8098167-8101282 REVERSEmoderately similar to ( 419) loc_os04g48930 12004.m101632 protein ferric-chelate reductase, putative, expressed no original description T

11.1.5 lipid metabolism.FA synthesis and FA elongation.beta hydroxyacyl ACP dehydratase MDP0000232809 moderately similar to ( 306) AT5G10160 | Symbols: | beta-hydroxyacyl-ACP dehydratase, putative | chr5:3185819-3187159 FORWARDmoderately similar to ( 282) loc_os05g36000 12005.m27841 protein beta-hydroxyacyl-ACP dehydratase, putative, expressed no original description T

25 C1-metabolism MDP0000900653 highly similar to ( 833) AT2G16370 | Symbols: THY-1 | THY-1 (THYMIDYLATE SYNTHASE 1); dihydrofolate reductase/ thymidylate synthase | chr2:7082038-7084333 REVERSEhighly similar to ( 865) DRTS_SOYBN Bifunctional dihydrofolate reductase-thymidylate synthase (DHFR-TS) [Includes: Dihydrofolate reductase (EC 1.5.1.3); Thymidylate synthase (EC 2.1.1.45)] - Glycine max (Soybean)highly similar to ( 832) loc_os11g29390 12011.m06849 protein bifunctional dihydrofolate reductase-thymidylate synthase, putative, expressed no original description T

29.3.4.2 protein.targeting.secretory pathway.golgi MDP0000823692 moderately similar to ( 300) AT2G27460 | Symbols: | sec23/sec24 transport family protein | chr2:11740670-11744867 FORWARDmoderately similar to ( 234) loc_os05g37120 12005.m27849 protein sec23/Sec24 trunk domain containing protein, expressed no original description T

27.3.22 RNA.regulation of transcription.HB,Homeobox transcription factor family MDP0000143173 moderately similar to ( 370) AT5G41410 | Symbols: BEL1 | BEL1 (BELL 1); DNA binding / protein binding / transcription factor | chr5:16580424-16583770 FORWARDvery weakly similar to (54.7) KNOX3_MAIZE Homeobox protein knotted-1-like 3 (Fragment) - Zea mays (Maize)moderately similar to ( 317) loc_os10g39030 12010.m06673 protein bell-like homeodomain protein 3, putative, expressed no original description T

35.1.42 not assigned.no ontology.proline rich family MDP0000179960 weakly similar to ( 133) AT5G07020 | Symbols: | proline-rich family protein | chr5:2180669-2182284 REVERSEweakly similar to ( 125) loc_os02g35090 12002.m08597 protein expressed protein no original description T

35.2 not assigned.unknown MDP0000343801 moderately similar to ( 460) AT4G21570 | Symbols: | unknown protein | chr4:11471126-11472269 REVERSEmoderately similar to ( 473) loc_os07g32230 12007.m29186 protein MAPK activating protein, putative, expressed no original description T

35.2 not assigned.unknown MDP0000256303 highly similar to ( 513) AT1G01430 | Symbols: | unknown protein | chr1:156953-158536 REVERSEmoderately similar to ( 472) loc_os05g12380 12005.m05703 protein expressed protein no original description T

30.2.11 signalling.receptor kinases.leucine rich repeat XI MDP0000064909 nearly identical (1274) AT2G41820 | Symbols: | leucine-rich repeat transmembrane protein kinase, putative | chr2:17447170-17449914 FORWARDmoderately similar to ( 417) RPK1_IPONI Receptor-like protein kinase precursor (EC 2.7.11.1) - Ipomoea nil (Japanese morning glory) (Pharbitis nil)highly similar to ( 967) loc_os07g10630 12007.m05518 protein ATP binding protein, putative, expressed no original description T

35.2 not assigned.unknown MDP0000315378 moderately similar to ( 467) AT1G48880 | Symbols: | unknown protein | chr1:18081033-18082650 FORWARDmoderately similar to ( 456) loc_os05g35190 12005.m07749 protein expressed protein no original description T

29.4 protein.postranslational modification MDP0000224925 highly similar to ( 823) AT3G53380 | Symbols: | lectin protein kinase family protein | chr3:19789204-19791351 REVERSEweakly similar to ( 189) PSKR_DAUCA Phytosulfokine receptor precursor (EC 2.7.11.1) (Phytosulfokine LRR receptor kinase) - Daucus carota (Carrot)moderately similar to ( 468) loc_os05g03450 12005.m04879 protein carbohydrate binding protein, putative, expressed no original description T

29.4.1.57 protein.postranslational modification.kinase.receptor like cytoplasmatic kinase VII MDP0000224925 highly similar to ( 823) AT3G53380 | Symbols: | lectin protein kinase family protein | chr3:19789204-19791351 REVERSEweakly similar to ( 189) PSKR_DAUCA Phytosulfokine receptor precursor (EC 2.7.11.1) (Phytosulfokine LRR receptor kinase) - Daucus carota (Carrot)moderately similar to ( 468) loc_os05g03450 12005.m04879 protein carbohydrate binding protein, putative, expressed no original description T

30.5 signalling.G-proteins MDP0000210199 weakly similar to ( 181) AT3G11730 | Symbols: ATFP8, ATRABD1, RABD1 | ATFP8; GTP binding / GTP-dependent protein binding / myosin XI tail binding | chr3:3709490-3711397 REVERSEweakly similar to ( 167) RIC1_ORYSA Ras-related protein RIC1 - Oryza sativa (Rice)weakly similar to ( 171) loc_os05g01490 12005.m83918 protein GTP-binding protein YPTM1, putative, expressed no original description T

21.1 redox.thioredoxin MDP0000160720 highly similar to ( 600) AT1G04980 | Symbols: ATPDIL2-2, ATPDI10, PDI10 | ATPDIL2-2 (PDI-LIKE 2-2); protein disulfide isomerase | chr1:1413869-1416120 REVERSEweakly similar to ( 128) PDIA6_MEDSA Probable protein disulfide-isomerase A6 precursor (EC 5.3.4.1) (P5) - Medicago sativa (Alfalfa)highly similar to ( 611) loc_os09g27830 12009.m22113 protein OsPDIL2-3 - Oryza sativa protein disulfide isomerase, expressed no original description T

35.2 not assigned.unknown MDP0000177692 very weakly similar to (55.8) loc_os01g52890 12001.m11462 protein expressed protein no original description T

28.1 DNA.synthesis/chromatin structure MDP0000136041 highly similar to ( 948) AT4G12620 | Symbols: ORC1B, ATORC1B, UNE13 | ORC1B (ORIGIN OF REPLICATION COMPLEX 1B); DNA binding / double-stranded methylated DNA binding / protein binding | chr4:7459812-7462253 REVERSEhighly similar to ( 914) loc_os06g08790 12006.m05598 protein origin recognition complex subunit 1, putative, expressed Sporozoite_P67 no original description T

28.99 DNA.unspecified MDP0000136041 highly similar to ( 948) AT4G12620 | Symbols: ORC1B, ATORC1B, UNE13 | ORC1B (ORIGIN OF REPLICATION COMPLEX 1B); DNA binding / double-stranded methylated DNA binding / protein binding | chr4:7459812-7462253 REVERSEhighly similar to ( 914) loc_os06g08790 12006.m05598 protein origin recognition complex subunit 1, putative, expressed Sporozoite_P67 no original description T

2.1.2.2 major CHO metabolism.synthesis.starch.starch synthase MDP0000095637 highly similar to ( 849) AT1G32900 | Symbols: | starch synthase, putative | chr1:11920582-11923506 REVERSEhighly similar to ( 910) SSG1_ANTMA Granule-bound starch synthase 1, chloroplast precursor (EC 2.4.1.21) (Granule-bound starch synthase I) (GBSS-I) - Antirrhinum majus (Garden snapdragon)highly similar to ( 772) loc_os07g22930 12007.m29139 protein granule-bound starch synthase 1b, chloroplast precursor, putative, expressed no original description T

16.2 secondary metabolism.phenylpropanoids MDP0000167955 highly similar to ( 949) AT4G30210 | Symbols: ATR2, AR2 | ATR2 (ARABIDOPSIS P450 REDUCTASE 2); NADPH-hemoprotein reductase | chr4:14796900-14800578 FORWARDhighly similar to ( 957) NCPR_CATRO NADPH--cytochrome P450 reductase (EC 1.6.2.4) (CPR) (P450R) - Catharanthus roseus (Rosy periwinkle) (Madagascar periwinkle)highly similar to ( 902) loc_os09g38620 12009.m06803 protein NADPH--cytochrome P450 reductase, putative, expressed MSP1_C no original description T

29.5.11.3 protein.degradation.ubiquitin.E2 MDP0000465760 moderately similar to ( 269) AT3G57870 | Symbols: AHUS5, EMB1637, SCE1, SCE1A, ATSCE1 | SCE1 (SUMO CONJUGATION ENZYME 1); SUMO ligase | chr3:21428831-21430110 REVERSEvery weakly similar to (95.5) UBC2_MEDSA Ubiquitin-conjugating enzyme E2-17 kDa (EC 6.3.2.19) (Ubiquitin-protein ligase) (Ubiquitin carrier protein) - Medicago sativa (Alfalfa)moderately similar to ( 285) loc_os03g03130 12003.m05850 protein ubiquitin-conjugating enzyme E2 I, putative, expressed no original description T

29.4 protein.postranslational modification MDP0000314856 moderately similar to ( 374) AT1G09020 | Symbols: SNF4, ATSNF4 | SNF4 (HOMOLOG OF YEAST SUCROSE NONFERMENTING 4); protein kinase activator | chr1:2900149-2904212 REVERSEmoderately similar to ( 335) loc_os03g63940 12003.m11263 protein protein kinase AKINbetagamma-2, putative, expressed no original description T

29.2.1.1.1.1.530 protein.synthesis.ribosomal protein.prokaryotic.chloroplast.30S subunit.S30A MDP0000248766 moderately similar to ( 328) AT5G24490 | Symbols: | 30S ribosomal protein, putative | chr5:8365690-8367178 FORWARDmoderately similar to ( 318) RR30_SPIOL Plastid-specific 30S ribosomal protein 1, chloroplast precursor (PSRP-1) (CS-S5) (CS5) (S22) (Ribosomal protein 1) - Spinacia oleracea (Spinach)moderately similar to ( 252) loc_os03g63950 12003.m35647 protein plastid-specific 30S ribosomal protein 1, chloroplast precursor, putative, expressed no original description T

35.2 not assigned.unknown MDP0000202827 moderately similar to ( 281) AT3G04780 | Symbols: | Encodes a protein with little sequence identity with any other protein of known structure or function. Part of this protein shows a 42% sequence identity with the C-terminal domain of the 32-kD human thioredoxin-like protein. | chr3:1311447-1313013 REVERSEmoderately similar to ( 245) loc_os03g58130 12003.m10725 protein thioredoxin-like protein 1, putative, expressed no original description T

29.5 protein.degradation MDP0000197449 weakly similar to ( 194) AT2G03120 | Symbols: ATSPP | ATSPP (ARABIDOPSIS SIGNAL PEPTIDE PEPTIDASE); aspartic-type endopeptidase | chr2:937554-940083 FORWARDweakly similar to ( 184) loc_os02g02530 12002.m77764 protein minor histocompatibility antigen H13, putative, expressed no original description T

27.4 RNA.RNA binding MDP0000260339 weakly similar to ( 125) AT2G21660 | Symbols: ATGRP7, CCR2 | CCR2 (COLD, CIRCADIAN RHYTHM, AND RNA BINDING 2); RNA binding / double-stranded DNA binding / single-stranded DNA binding | chr2:9265477-9266316 REVERSEweakly similar to ( 124) GRP1_DAUCA Glycine-rich RNA-binding protein - Daucus carota (Carrot)weakly similar to ( 127) loc_os12g43600 12012.m26989 protein glycine-rich RNA-binding protein 2, putative, expressed no original description T

35.1 not assigned.no ontology MDP0000343311 weakly similar to ( 152) AT5G53940 | Symbols: | yippee family protein | chr5:21897164-21898589 REVERSEweakly similar to ( 127) YIPL_SOLTU Protein yippee-like - Solanum tuberosum (Potato)weakly similar to ( 129) loc_os03g49150 12003.m101452 protein protein yippee-like, putative, expressed no original description T

35.2 not assigned.unknown MDP0000764712 moderately similar to ( 332) AT1G22030 | Symbols: | unknown protein | chr1:7759337-7760415 REVERSEmoderately similar to ( 214) loc_os01g65420 12001.m12654 protein expressed protein no original description T

29.5.9 protein.degradation.AAA type MDP0000503228 nearly identical (1440) AT5G03340 | Symbols: | cell division cycle protein 48, putative / CDC48, putative | chr5:810091-813133 REVERSEnearly identical (1465) CDC48_SOYBN Cell division cycle protein 48 homolog (Valosin-containing protein homolog) (VCP) - Glycine max (Soybean)nearly identical (1432) loc_os03g05730 12003.m06095 protein cell division control protein 48 homolog E, putative, expressed no original description T

26.2 misc.UDP glucosyl and glucoronyl transferases MDP0000204575 highly similar to ( 582) AT4G22580 | Symbols: | exostosin family protein | chr4:11889382-11890689 REVERSEmoderately similar to ( 255) KATAM_ORYSA Xyloglucan galactosyltransferase KATAMARI1 homolog (EC 2.4.1.-) - Oryza sativa (Rice)moderately similar to ( 432) loc_os10g40559 12010.m06823 protein xyloglucan galactosyltransferase KATAMARI 1, putative, expressed TrbL PRK08026 no original description T

27.3.25 RNA.regulation of transcription.MYB domain transcription factor family MDP0000209761 moderately similar to ( 221) AT4G38620 | Symbols: ATMYB4, MYB4 | MYB4; DNA binding / transcription factor | chr4:18053866-18054876 FORWARDmoderately similar to ( 220) MYB1_HORVU Myb-related protein Hv1 - Hordeum vulgare (Barley)moderately similar to ( 226) loc_os05g35500 12005.m07780 protein mybHv5, putative, expressed no original description T

1.1.40 PS.lightreaction.cyclic electron flow-chlororespiration MDP0000149416 moderately similar to ( 399) AT4G22890 | Symbols: PGR5-LIKE A | PGR5-LIKE A | chr4:12007157-12009175 FORWARDmoderately similar to ( 371) loc_os08g41460 12008.m080214 protein expressed protein no original description T

35.1 not assigned.no ontology MDP0000885773 very weakly similar to (65.1) AT1G03820 | Symbols: | unknown protein | chr1:960008-960676 REVERSE MSP1_C no original description T

35.2 not assigned.unknown MDP0000334396 weakly similar to ( 167) AT3G57785 | Symbols: | unknown protein | chr3:21404835-21405179 REVERSEweakly similar to ( 163) loc_os09g31260 12009.m06240 protein expressed protein no original description T

30.4.4 signalling.phosphinositides.phosphoinositide phospholipase C MDP0000239522 highly similar to ( 847) AT3G08510 | Symbols: | ATPLC2 (PHOSPHOLIPASE C 2); phospholipase C | chr3:2582626-2585556 REVERSEhighly similar to ( 700) loc_os07g49330 12007.m29351 protein phosphoinositide-specific phospholipase C, putative, expressed no original description T

26.8 misc.nitrilases, *nitrile lyases, berberine bridge enzymes, reticuline oxidases, troponine reductases MDP0000188304 highly similar to ( 558) AT4G38220 | Symbols: | aminoacylase, putative / N-acyl-L-amino-acid amidohydrolase, putative | chr4:17925251-17926919 FORWARDhighly similar to ( 541) loc_os08g40110 12008.m07991 protein aminoacylase-1, putative, expressed no original description T

29.1.3 protein.aa activation.threonine-tRNA ligase MDP0000149241 nearly identical (1035) AT5G26830 | Symbols: | threonyl-tRNA synthetase / threonine--tRNA ligase (THRRS) | chr5:9437351-9441568 FORWARDnearly identical (1014) loc_os08g19850 12008.m06052 protein threonyl-tRNA synthetase, mitochondrial precursor, putative, expressed no original description T

10.1.6 cell wall.precursor synthesis.GAE MDP0000216173 highly similar to ( 728) AT3G23820 | Symbols: GAE6 | GAE6 (UDP-D-GLUCURONATE 4-EPIMERASE 6); UDP-glucuronate 4-epimerase/ catalytic | chr3:8603645-8605027 FORWARDvery weakly similar to (92.0) GALE1_PEA UDP-glucose 4-epimerase (EC 5.1.3.2) (Galactowaldenase) (UDP-galactose 4-epimerase) - Pisum sativum (Garden pea)highly similar to ( 558) loc_os09g32670 12009.m06354 protein protein capI, putative, expressed COG4907 no original description T

16.1.1 secondary metabolism.isoprenoids.non-mevalonate pathway MDP0000302490 weakly similar to ( 159) AT1G74470 | Symbols: | geranylgeranyl reductase | chr1:27991248-27992845 FORWARDweakly similar to ( 148) loc_os02g51080 12002.m33869 protein geranylgeranyl hydrogenase, putative, expressed MSP1_C no original description T

9.1.1 mitochondrial electron transport / ATP synthesis.NADH-DH.complex I MDP0000226218 moderately similar to ( 443) AT4G28510 | Symbols: ATPHB1 | ATPHB1 (PROHIBITIN 1) | chr4:14084970-14086372 REVERSEmoderately similar to ( 432) loc_os07g15880 12007.m06032 protein mitochondrial prohibitin complex protein 2, putative, expressed no original description T

21.1 redox.thioredoxin MDP0000448333 weakly similar to ( 136) AT5G39950 | Symbols: ATTRX2, ATH2, ATTRXH2, TRXH2 | ATTRX2 (THIOREDOXIN 2); oxidoreductase, acting on sulfur group of donors, disulfide as acceptor | chr5:15990885-15991881 REVERSEweakly similar to ( 108) TRXH1_TOBAC Thioredoxin H-type 1 (TRX-H1) - Nicotiana tabacum (Common tobacco)weakly similar to ( 114) loc_os03g58630 12003.m10768 protein thioredoxin H-type 5, putative, expressed no original description T

27.3.32 RNA.regulation of transcription.WRKY domain transcription factor family MDP0000273851 moderately similar to ( 238) AT5G15130 | Symbols: WRKY72, ATWRKY72 | WRKY72; transcription factor | chr5:4904426-4906879 FORWARDmoderately similar to ( 239) loc_os06g05380 12006.m91873 protein OsWRKY73 - Superfamily of rice TFs having WRKY and zinc finger domains, expressed no original description T

29.4 protein.postranslational modification MDP0000212424 nearly identical (1745) AT5G18700 | Symbols: EMB3013 | EMB3013 (embryo defective 3013); ATP binding / binding / kinase/ protein kinase/ protein serine/threonine kinase | chr5:6235387-6240733 REVERSEweakly similar to ( 112) M2K1_ORYSA Mitogen-activated protein kinase kinase 1 (EC 2.7.12.2) (MAP kinase kinase 1) (MAPKK1) (OsMEK1) - Oryza sativa (Rice)nearly identical (1573) loc_os01g15480 12001.m08146 protein EMB3013, putative, expressed no original description T

27.3.32 RNA.regulation of transcription.WRKY domain transcription factor family MDP0000175240 weakly similar to ( 142) AT3G56400 | Symbols: WRKY70, ATWRKY70 | WRKY70; transcription factor/ transcription repressor | chr3:20909082-20910409 REVERSEweakly similar to ( 118) loc_os05g25770 12005.m06869 protein OsWRKY45 - Superfamily of rice TFs having WRKY and zinc finger domains, expressed no original description T

35.2 not assigned.unknown MDP0000285566 moderately similar to ( 455) AT1G32160 | Symbols: | unknown protein | chr1:11568701-11570241 FORWARDmoderately similar to ( 407) loc_os04g50860 12004.m09983 protein expressed protein no original description T

35.2 not assigned.unknown MDP0000671726 highly similar to ( 684) AT3G60320 | Symbols: | DNA binding | chr3:22292073-22295228 REVERSEvery weakly similar to (68.6) RK27_TOBAC 50S ribosomal protein L27, chloroplast precursor (CL27) - Nicotiana tabacum (Common tobacco)highly similar to ( 526) loc_os10g41310 12010.m06895 protein bZIP-like protein, putative, expressed no original description T

26.1 misc.misc2 MDP0000731356 moderately similar to ( 384) AT4G02340 | Symbols: | epoxide hydrolase, putative | chr4:1035722-1037403 FORWARDmoderately similar to ( 385) loc_os05g19150 12005.m06318 protein epoxide hydrolase 2, putative, expressed no original description T

10.6.3 cell wall.degradation.pectate lyases and polygalacturonases MDP0000266603 highly similar to ( 614) AT1G67750 | Symbols: | pectate lyase family protein | chr1:25401660-25403165 FORWARDmoderately similar to ( 400) PEL_LILLO Pectate lyase precursor (EC 4.2.2.2) - Lilium longiflorum (Trumpet lily)highly similar to ( 549) loc_os04g05050 12004.m35243 protein pectate lyase 8 precursor, putative, expressed no original description T

29.4 protein.postranslational modification MDP0000574890 highly similar to ( 553) AT3G51630 | Symbols: WNK5, ZIK1, ATWNK5 | WNK5 (WITH NO LYSINE (K) KINASE 5); protein kinase | chr3:19149487-19151924 FORWARDvery weakly similar to (74.7) NTF3_TOBAC Mitogen-activated protein kinase homolog NTF3 (EC 2.7.11.24) (P43) - Nicotiana tabacum (Common tobacco)moderately similar to ( 489) loc_os11g02300 12011.m28642 protein mitogen-activated protein kinase, putative, expressed no original description T

35.2 not assigned.unknown MDP0000904537 no original description T

27.3.20 RNA.regulation of transcription.G2-like transcription factor family, GARP MDP0000124053 weakly similar to ( 186) AT1G69580 | Symbols: | transcription factor | chr1:26172127-26173612 FORWARDweakly similar to ( 147) loc_os03g20900 12003.m07498 protein transfactor-like, putative, expressed no original description T

29.3.3 protein.targeting.chloroplast MDP0000155675 highly similar to ( 743) AT2G24820 | Symbols: TIC55 | TIC55 (TRANSLOCON AT THE INNER ENVELOPE MEMBRANE OF CHLOROPLASTS 55); 2 iron, 2 sulfur cluster binding / electron carrier/ oxidoreductase | chr2:10575038-10576829 FORWARDweakly similar to ( 117) CAO_CHLRE Chlorophyllide a oxygenase, chloroplast precursor (EC 1.13.12.14) (Chlorophyll a oxygenase) (Chlorophyll b synthase) - Chlamydomonas reinhardtiihighly similar to ( 676) loc_os02g54980 12002.m10525 protein pheophorbide a oxygenase, chloroplast precursor, putative, expressed no original description T

29.2.1.2.2.5 protein.synthesis.ribosomal protein.eukaryotic.60S subunit.L5 MDP0000321309 moderately similar to ( 461) AT3G25520 | Symbols: ATL5, PGY3, OLI5, RPL5A | ATL5 (A. THALIANA RIBOSOMAL PROTEIN L5); 5S rRNA binding / structural constituent of ribosome | chr3:9269573-9271327 REVERSEmoderately similar to ( 463) RL5_CUCSA 60S ribosomal protein L5 - Cucumis sativus (Cucumber)moderately similar to ( 440) loc_os01g67126 12001.m43540 protein 60S ribosomal protein L5-2, putative, expressed no original description T

29.5.3 protein.degradation.cysteine protease MDP0000203756 weakly similar to ( 125) AT3G12490 | Symbols: ATCYSB | cysteine protease inhibitor, putative / cystatin, putative | chr3:3960523-3961777 REVERSEweakly similar to ( 118) CYTI_VIGUN Cysteine proteinase inhibitor (Cystatin) - Vigna unguiculata (Cowpea)weakly similar to ( 122) loc_os01g16430 12001.m08239 protein multidomain cystatin, putative, expressed no original description T

30.2.11 signalling.receptor kinases.leucine rich repeat XI MDP0000267318 moderately similar to ( 406) AT3G47570 | Symbols: | leucine-rich repeat transmembrane protein kinase, putative | chr3:17527611-17530748 FORWARDmoderately similar to ( 265) RPK1_IPONI Receptor-like protein kinase precursor (EC 2.7.11.1) - Ipomoea nil (Japanese morning glory) (Pharbitis nil)moderately similar to ( 386) loc_os11g07230 12011.m079952 protein receptor-like protein kinase 5 precursor, putative, expressed no original description T

35.2 not assigned.unknown MDP0000185489 no original description T

29.3.4.99 protein.targeting.secretory pathway.unspecified MDP0000914572 highly similar to ( 592) AT3G51670 | Symbols: | SEC14 cytosolic factor family protein / phosphoglyceride transfer family protein | chr3:19168912-19170848 FORWARDhighly similar to ( 541) loc_os05g27820 12005.m07070 protein patellin-5, putative, expressed no original description T

35.2 not assigned.unknown MDP0000124016 highly similar to ( 577) AT5G19540 | Symbols: | unknown protein | chr5:6595748-6597724 FORWARDhighly similar to ( 509) loc_os08g29780 12008.m06980 protein expressed protein MSP1_C no original description T

27.3.6 RNA.regulation of transcription.bHLH,Basic Helix-Loop-Helix family MDP0000137325 moderately similar to ( 211) AT4G17880 | Symbols: | basic helix-loop-helix (bHLH) family protein | chr4:9933702-9935471 REVERSEvery weakly similar to (81.3) ARRS_MAIZE Anthocyanin regulatory R-S protein - Zea mays (Maize)weakly similar to ( 138) loc_os10g42430 12010.m06996 protein transcription factor MYC7E, putative, expressed no original description T

27.3.26 RNA.regulation of transcription.MYB-related transcription factor family MDP0000209471 moderately similar to ( 296) AT2G38090 | Symbols: | myb family transcription factor | chr2:15945278-15946775 FORWARDmoderately similar to ( 246) loc_os05g37730 12005.m07952 protein MYB-like transcription factor DIVARICATA, putative, expressed no original description T

29.5.11.4.3.2 protein.degradation.ubiquitin.E3.SCF.FBOX MDP0000430258 very weakly similar to (70.9) AT5G49610 | Symbols: | F-box family protein | chr5:20131448-20132527 REVERSEvery weakly similar to (93.6) loc_os03g56500 12003.m10559 protein expressed protein no original description T

30.2.3 signalling.receptor kinases.leucine rich repeat III MDP0000158428 highly similar to ( 618) AT4G23740 | Symbols: | leucine-rich repeat transmembrane protein kinase, putative | chr4:12367063-12369159 FORWARDmoderately similar to ( 211) PSKR_DAUCA Phytosulfokine receptor precursor (EC 2.7.11.1) (Phytosulfokine LRR receptor kinase) - Daucus carota (Carrot)highly similar to ( 584) loc_os01g12390 12001.m07854 protein ATP binding protein, putative, expressed no original description T

29.5.11.4.2 protein.degradation.ubiquitin.E3.RING MDP0000696497 very weakly similar to (95.1) AT5G53110 | Symbols: | FUNCTIONS IN: molecular_function unknown; INVOLVED IN: biological_process unknown; LOCATED IN: endomembrane system; EXPRESSED IN: 8 plant structures; EXPRESSED DURING: 4 anthesis, C globular stage, petal differentiation and expansion stage; BEST Arabidopsis thaliana protein match is: zinc finger (C3HC4-type RING finger) family protein (TAIR:AT2G46495.1); Has 118 Blast hits to 116 proteins in 13 species: Archae - 0; Bacteria - 0; Metazoa - 0; Fungi - 0; Plants - 118; Viruses - 0; Other Eukaryotes - 0 (source: NCBI BLink). | chr5:21529022-21529744 FORWARDvery weakly similar to (58.5) loc_os01g02290 12001.m06871 protein Ser/Thr receptor-like kinase, putative, expressed no original description T

35.1 not assigned.no ontology MDP0000321007 highly similar to ( 913) AT5G08500 | Symbols: | transmembrane CLPTM1 family protein | chr5:2748261-2751590 FORWARDhighly similar to ( 908) loc_os04g36050 12004.m08694 protein cleft lip and palate transmembrane 1 protein, putative, expressed no original description T

35.2 not assigned.unknown MDP0000279785 very weakly similar to (92.8) AT5G42330 | Symbols: | unknown protein | chr5:16926672-16927370 FORWARDvery weakly similar to (56.2) loc_os07g29190 12007.m07190 protein expressed protein no original description T

17.3.2.2 hormone metabolism.brassinosteroid.signal transduction.BZR MDP0000233714 moderately similar to ( 277) AT1G75080 | Symbols: BZR1 | BZR1 (BRASSINAZOLE-RESISTANT 1); DNA binding / transcription regulator/ transcription repressor | chr1:28185709-28187063 FORWARDmoderately similar to ( 265) loc_os07g39220 12007.m08164 protein BES1/BZR1 homolog protein 1, putative, expressed TrbL no original description T

35.2 not assigned.unknown MDP0000338689 no original description T

16.2.1.1 secondary metabolism.phenylpropanoids.lignin biosynthesis.PAL MDP0000787168 nearly identical (1014) AT2G37040 | Symbols: pal1, ATPAL1 | pal1 (Phe ammonia lyase 1); phenylalanine ammonia-lyase | chr2:15557602-15560237 REVERSEnearly identical (1054) PAL1_DAUCA Phenylalanine ammonia-lyase 1 (EC 4.3.1.5) - Daucus carota (Carrot)highly similar to ( 949) loc_os04g43800 12004.m09339 protein phenylalanine ammonia-lyase, putative, expressed no original description T

26.9 misc.glutathione S transferases MDP0000175443 moderately similar to ( 218) AT1G65820 | Symbols: | microsomal glutathione s-transferase, putative | chr1:24485213-24486682 FORWARDweakly similar to ( 198) loc_os03g50130 12003.m35389 protein microsomal glutathione S-transferase 3, putative, expressed no original description T

27.3.11 RNA.regulation of transcription.C2H2 zinc finger family MDP0000131949 moderately similar to ( 360) AT1G08290 | Symbols: | zinc finger (C2H2 type) protein (WIP3) | chr1:2610680-2613180 REVERSEmoderately similar to ( 316) loc_os06g40960 12006.m08631 protein zinc finger protein, putative, expressed no original description T

23.2 nucleotide metabolism.degradation MDP0000178729 nearly identical (1860) AT4G34890 | Symbols: ATXDH1, XDH1 | XDH1 (XANTHINE DEHYDROGENASE 1); xanthine dehydrogenase | chr4:16618736-16624983 REVERSEmoderately similar to ( 439) ALDOL_ORYSA Putative aldehyde oxidase-like protein - Oryza sativa (Rice)nearly identical (1701) loc_os03g31550 12003.m34889 protein xanthine dehydrogenase/oxidase, putative, expressed no original description T

13.1.6.1.10 amino acid metabolism.synthesis.aromatic aa.chorismate.dehydroquinate/shikimate dehydrogenase MDP0000279446 highly similar to ( 713) AT3G06350 | Symbols: EMB3004, MEE32 | MEE32 (MATERNAL EFFECT EMBRYO ARREST 32); 3-dehydroquinate dehydratase/ NADP or NADPH binding / binding / catalytic/ shikimate 5-dehydrogenase | chr3:1924536-1927701 REVERSEhighly similar to ( 612) loc_os12g34874 12012.m73984 protein shikimate dehydrogenase, putative, expressed no original description T

4.2.14 glycolysis.plastid branch.pyruvate kinase (PK) MDP0000260603 highly similar to ( 728) AT1G32440 | Symbols: PKp3 | PKp3 (plastidial pyruvate kinase 3); pyruvate kinase | chr1:11712205-11714963 FORWARDhighly similar to ( 793) KPYG_TOBAC Pyruvate kinase isozyme G, chloroplast precursor (EC 2.7.1.40) - Nicotiana tabacum (Common tobacco)highly similar to ( 719) loc_os10g42100 12010.m06967 protein pyruvate kinase isozyme G, chloroplast precursor, putative, expressed no original description T

26.21 misc.protease inhibitor/seed storage/lipid transfer protein (LTP) family protein MDP0000921319 weakly similar to ( 159) AT2G10940 | Symbols: | protease inhibitor/seed storage/lipid transfer protein (LTP) family protein | chr2:4311160-4312035 REVERSEvery weakly similar to (87.0) 14KD_DAUCA 14 kDa proline-rich protein DC2.15 precursor - Daucus carota (Carrot)weakly similar to ( 159) loc_os06g43600 12006.m08890 protein 36.4 kDa proline-rich protein, putative, expressed no original description T

16.1.1.1 secondary metabolism.isoprenoids.non-mevalonate pathway.DXS MDP0000793656 highly similar to ( 976) AT4G15560 | Symbols: CLA1, DEF, CLA, DXS, DXPS2 | CLA1 (CLOROPLASTOS ALTERADOS 1); 1-deoxy-D-xylulose-5-phosphate synthase | chr4:8884218-8887254 FORWARDhighly similar to ( 986) DXS_ORYSA Probable 1-deoxy-D-xylulose-5-phosphate synthase, chloroplast precursor (EC 2.2.1.7) (1-deoxyxylulose-5-phosphate synthase) (DXP synthase) (DXPS) - Oryza sativa (Rice)nearly identical (1116) loc_os07g09190 12007.m05375 protein 1-deoxy-D-xylulose-5-phosphate synthase, chloroplast precursor, putative, expressed no original description T

26.7 misc.oxidases - copper, flavone etc MDP0000769741 highly similar to ( 797) AT2G43020 | Symbols: ATPAO2 | ATPAO2 (Polyamine oxidase 2); amine oxidase/ electron carrier/ oxidoreductase | chr2:17891945-17894440 FORWARDweakly similar to ( 151) PAO_MAIZE Polyamine oxidase precursor (EC 1.5.3.11) - Zea mays (Maize)highly similar to ( 715) loc_os04g53190 12004.m35201 protein lysine-specific histone demethylase 1, putative, expressed no original description T

35.2 not assigned.unknown MDP0000339917 no original description T

35.2 not assigned.unknown MDP0000132362 weakly similar to ( 136) AT2G03420 | Symbols: | unknown protein | chr2:1035100-1035937 REVERSEweakly similar to ( 129) loc_os03g11230 12003.m06574 protein expressed protein no original description T

35.2 not assigned.unknown MDP0000311884 moderately similar to ( 273) AT1G54520 | Symbols: | unknown protein | chr1:20363565-20365874 FORWARDmoderately similar to ( 272) loc_os03g48920 12003.m35367 protein membrane protein, putative, expressed no original description T

34.13 transport.peptides and oligopeptides MDP0000606453 highly similar to ( 845) AT1G68570 | Symbols: | proton-dependent oligopeptide transport (POT) family protein | chr1:25746811-25750110 FORWARDhighly similar to ( 684) loc_os06g15370 12006.m06250 protein peptide transporter PTR2, putative, expressed no original description T

29.2.1.2.1.24 protein.synthesis.ribosomal protein.eukaryotic.40S subunit.S24 MDP0000445815 moderately similar to ( 231) AT3G04920 | Symbols: | 40S ribosomal protein S24 (RPS24A) | chr3:1360989-1362065 FORWARDmoderately similar to ( 216) loc_os01g52490 12001.m11423 protein 40S ribosomal protein S24, putative, expressed no original description T

20.1.1 stress.biotic.respiratory burst MDP0000273819 nearly identical (1008) AT1G19230 | Symbols: | respiratory burst oxidase protein E (RbohE) / NADPH oxidase | chr1:6644189-6649149 FORWARDvery weakly similar to (75.5) BAS1_HORVU 2-cys peroxiredoxin BAS1, chloroplast precursor (EC 1.11.1.15) (Thiol-specific antioxidant protein) (Fragment) - Hordeum vulgare (Barley)highly similar to ( 974) loc_os09g26660 12009.m05818 protein calcium ion binding protein, putative, expressed no original description T

29.2.1.1.1.2.18 protein.synthesis.ribosomal protein.prokaryotic.chloroplast.50S subunit.L18 MDP0000834632 moderately similar to ( 227) AT1G48350 | Symbols: | ribosomal protein L18 family protein | chr1:17867269-17868215 FORWARDweakly similar to ( 183) RK18_ORYSA 50S ribosomal protein L18, chloroplast precursor (CL18) - Oryza sativa (Rice)weakly similar to ( 183) loc_os03g61260 12003.m35497 protein 50S ribosomal protein L18, chloroplast precursor, putative, expressed no original description T

20.2.99 stress.abiotic.unspecified MDP0000521048 moderately similar to ( 254) AT1G72610 | Symbols: GLP1, ATGER1, GER1 | GER1 (GERMIN-LIKE PROTEIN 1); oxalate oxidase | chr1:27339302-27339928 REVERSEmoderately similar to ( 276) AB19A_PRUPE Auxin-binding protein ABP19a precursor - Prunus persica (Peach)moderately similar to ( 225) loc_os08g35760 12008.m07565 protein auxin-binding protein ABP20 precursor, putative, expressed no original description T

29.5.11.3 protein.degradation.ubiquitin.E2 MDP0000222938 weakly similar to ( 141) AT1G50490 | Symbols: UBC20 | UBC20 (ubiquitin-conjugating enzyme 20); ubiquitin-protein ligase | chr1:18705005-18706303 REVERSEvery weakly similar to (73.6) UBC2_MEDSA Ubiquitin-conjugating enzyme E2-17 kDa (EC 6.3.2.19) (Ubiquitin-protein ligase) (Ubiquitin carrier protein) - Medicago sativa (Alfalfa)weakly similar to ( 139) loc_os01g16650 12001.m08261 protein ubiquitin-conjugating enzyme X, putative, expressed no original description T

29.6 protein.folding MDP0000631455 highly similar to ( 886) AT2G28000 | Symbols: CPN60A, CH-CPN60A, SLP | CPN60A (CHAPERONIN-60ALPHA); ATP binding / protein binding | chr2:11926603-11929184 FORWARDhighly similar to ( 909) RUBA_PEA RuBisCO large subunit-binding protein subunit alpha, chloroplast precursor (60 kDa chaperonin subunit alpha) (CPN-60 alpha) - Pisum sativum (Garden pea)highly similar to ( 850) loc_os03g64210 12003.m101142 protein ruBisCO large subunit-binding protein subunit alpha, chloroplast precursor, putative, expressed no original description T

29.2.1.1.2.2.37 protein.synthesis.ribosomal protein.prokaryotic.mitochondrion.50S subunit.L37 MDP0000345456 weakly similar to ( 155) AT3G01740 | Symbols: | FUNCTIONS IN: molecular_function unknown; INVOLVED IN: biological_process unknown; EXPRESSED IN: 22 plant structures; EXPRESSED DURING: 13 growth stages; CONTAINS InterPro DOMAIN/s: Ribosomal protein L37, mitochondrial (InterPro:IPR013870); BEST Arabidopsis thaliana protein match is: unknown protein (TAIR:AT5G14290.1); Has 117 Blast hits to 117 proteins in 58 species: Archae - 0; Bacteria - 0; Metazoa - 56; Fungi - 22; Plants - 27; Viruses - 0; Other Eukaryotes - 12 (source: NCBI BLink). | chr3:268118-268498 FORWARDweakly similar to ( 131) loc_os12g14080 12012.m073800 protein expressed protein no original description T

17.2.3 hormone metabolism.auxin.induced-regulated-responsive-activated MDP0000596615 weakly similar to ( 112) AT1G17345 | Symbols: | auxin-responsive protein-related | chr1:5940525-5940920 FORWARDweakly similar to ( 110) loc_os08g35110 12008.m07500 protein OsSAUR33 - Auxin-responsive SAUR gene family member, expressed no original description T

26.24 misc.GCN5-related N-acetyltransferase MDP0000258868 moderately similar to ( 261) AT1G24040 | Symbols: | GCN5-related N-acetyltransferase (GNAT) family protein | chr1:8505794-8506753 REVERSEweakly similar to ( 150) loc_os04g39140 12004.m08903 protein acetyltransferase, GNAT family protein, expressed TrbL no original description T

13.1.5.1.1 amino acid metabolism.synthesis.serine-glycine-cysteine group.serine.phosphoglycerate dehydrogenase MDP0000949486 highly similar to ( 852) AT4G34200 | Symbols: EDA9 | EDA9 (embryo sac development arrest 9); ATP binding | chr4:16374041-16376561 REVERSEvery weakly similar to (98.6) DHGY_CUCSA Glycerate dehydrogenase (EC 1.1.1.29) (NADH-dependent hydroxypyruvate reductase) (HPR) (GDH) - Cucumis sativus (Cucumber)highly similar to ( 845) loc_os04g55720 12004.m35214 protein D-3-phosphoglycerate dehydrogenase, chloroplast precursor, putative, expressed no original description T

28.2 DNA.repair MDP0000672583 moderately similar to ( 437) AT5G57970 | Symbols: | methyladenine glycosylase family protein | chr5:23467316-23468910 FORWARDmoderately similar to ( 355) loc_os04g42290 12004.m09201 protein DNA-3-methyladenine glycosylase I, putative, expressed no original description T

26.17 misc.dynamin MDP0000311912 highly similar to ( 922) AT1G14830 | Symbols: ADL1C, ADL5, DRP1C | ADL1C (ARABIDOPSIS DYNAMIN-LIKE PROTEIN 1C); GTP binding / GTPase | chr1:5107699-5111470 REVERSEhighly similar to ( 884) loc_os03g50520 12003.m35393 protein dynamin-related protein 1C, putative, expressed no original description T

1.1.1.2 PS.lightreaction.photosystem II.PSII polypeptide subunits MDP0000281971 very weakly similar to (94.7) AT2G30570 | Symbols: PSBW | PSBW (PHOTOSYSTEM II REACTION CENTER W) | chr2:13019326-13020053 REVERSEweakly similar to ( 106) PSBW_SPIOL Photosystem II reaction center W protein, chloroplast precursor (PSII 6.1 kDa protein) - Spinacia oleracea (Spinach)very weakly similar to (76.6) loc_os05g43310 12005.m08459 protein photosystem II reaction center W protein, chloroplast precursor, putative, expressed no original description T

15.1 metal handling.acquisition MDP0000138686 highly similar to ( 742) AT5G49740 | Symbols: ATFRO7, FRO7 | ATFRO7 (FERRIC REDUCTION OXIDASE 7); ferric-chelate reductase/ oxidoreductase | chr5:20205549-20208628 REVERSEhighly similar to ( 723) loc_os04g36720 12004.m08712 protein ferric reductase-like transmembrane component, putative, expressed no original description T

31.3.1 cell.cycle.peptidylprolyl isomerase MDP0000186561 moderately similar to ( 280) AT4G32420 | Symbols: | peptidyl-prolyl cis-trans isomerase cyclophilin-type family protein | chr4:15647550-15650678 REVERSEweakly similar to ( 181) CYPH_MAIZE Peptidyl-prolyl cis-trans isomerase (EC 5.2.1.8) (PPIase) (Rotamase) (Cyclophilin) (Cyclosporin A-binding protein) - Zea mays (Maize)moderately similar to ( 226) loc_os02g10970 12002.m33611 protein peptidyl-prolyl cis-trans isomerase 1, putative, expressed no original description T

29.5.5 protein.degradation.serine protease MDP0000259633 highly similar to ( 725) AT3G07990 | Symbols: SCPL27 | SCPL27 (serine carboxypeptidase-like 27); serine-type carboxypeptidase | chr3:2552544-2554644 FORWARDhighly similar to ( 582) CBP2_HORVU Serine carboxypeptidase 2 precursor (EC 3.4.16.6) (Serine carboxypeptidase II) (Carboxypeptidase D) (CP-MII) [Contains: Serine carboxypeptidase 2 chain A (Serine carboxypeptidase II chain A); Serine carboxypeptidase 2 chain B (Serine cahighly similar to ( 684) loc_os01g61690 12001.m12294 protein virulence-related protein Nf314, putative, expressed no original description T

27.3.7 RNA.regulation of transcription.C2C2(Zn) CO-like, Constans-like zinc finger family MDP0000158886 weakly similar to ( 141) AT1G07050 | Symbols: | CONSTANS-like protein-related | chr1:2164327-2165133 REVERSEvery weakly similar to (55.8) PRR95_ORYSA Two-component response regulator-like PRR95 (Pseudo-response regulator 95) (OsPRR95) - Oryza sativa (Rice)very weakly similar to (90.9) loc_os02g05470 12002.m05895 protein CCT motif family protein, expressed no original description T

23.5.4 nucleotide metabolism.deoxynucleotide metabolism.ribonucleoside-diphosphate reductase MDP0000225340 moderately similar to ( 373) AT3G23580 | Symbols: RNR2, RNR2A | RNR2A (RIBONUCLEOTIDE REDUCTASE 2A); ribonucleoside-diphosphate reductase | chr3:8460261-8462574 FORWARDmoderately similar to ( 350) RIR2_TOBAC Ribonucleoside-diphosphate reductase small chain (EC 1.17.4.1) (Ribonucleotide reductase small subunit) (Ribonucleoside-diphosphate reductase R2 subunit) - Nicotiana tabacum (Common tobacco)moderately similar to ( 340) loc_os06g14620 12006.m06177 protein ribonucleoside-diphosphate reductase small chain, putative, expressed no original description T

30.5 signalling.G-proteins MDP0000600003 moderately similar to ( 367) AT2G44610 | Symbols: RAB6, ATRABH1B, ATRAB6A, RAB6A | RAB6A; GTP binding / protein binding | chr2:18411778-18413883 REVERSEweakly similar to ( 150) RHN1_NICPL Ras-related protein RHN1 - Nicotiana plumbaginifolia (Leadwort-leaved tobacco)moderately similar to ( 365) loc_os07g31370 12007.m07406 protein ras-related protein Rab-6A, putative, expressed no original description T

35.2 not assigned.unknown MDP0000172773 moderately similar to ( 256) AT2G40070 | Symbols: | FUNCTIONS IN: molecular_function unknown; INVOLVED IN: biological_process unknown; LOCATED IN: cellular_component unknown; EXPRESSED IN: 17 plant structures; EXPRESSED DURING: 7 growth stages; BEST Arabidopsis thaliana protein match is: proline-rich family protein (TAIR:AT3G09000.1); Has 92805 Blast hits to 48882 proteins in 1559 species: Archae - 225; Bacteria - 11081; Metazoa - 37135; Fungi - 20962; Plants - 3300; Viruses - 2664; Other Eukaryotes - 17438 (source: NCBI BLink). | chr2:16728378-16731040 REVERSEmoderately similar to ( 219) loc_os01g04880 12001.m42855 protein transposon protein, putative, CACTA, En/Spm sub-class, expressed no original description T

29.3.4.1 protein.targeting.secretory pathway.ER MDP0000311017 moderately similar to ( 344) AT3G25040 | Symbols: | ER lumen protein retaining receptor, putative / HDEL receptor, putative | chr3:9124479-9126051 FORWARDmoderately similar to ( 358) ERD2_PETHY ER lumen protein retaining receptor (HDEL receptor) (PGP169-12) - Petunia hybrida (Petunia)moderately similar to ( 338) loc_os11g28340 12011.m079996 protein ER lumen protein retaining receptor, putative, expressed no original description T

18.5.2.5 Co-factor and vitamine metabolism.folate & vitamine K.vitamine K.naphthoate synthase MDP0000943789 highly similar to ( 501) AT1G60550 | Symbols: ECHID, DHNS | ECHID (ENOYL-COA HYDRATASE/ISOMERASE D); catalytic/ naphthoate synthase | chr1:22305988-22308092 REVERSEvery weakly similar to (72.4) MFP_ORYSA Peroxisomal fatty acid beta-oxidation multifunctional protein (MFP) [Includes: Enoyl-CoA hydratase (EC 4.2.1.17); 3-2-trans-enoyl-CoA isomerase (EC 5.3.3.8); 3-hydroxybutyryl-CoA epimerase (EC 5.1.2.3); 3-hydroxyacyl-CoA dehydrogenase (Emoderately similar to ( 488) loc_os01g47350 12001.m10930 protein naphthoate synthase, putative, expressed no original description T

29.4 protein.postranslational modification MDP0000893433 highly similar to ( 791) AT5G47750 | Symbols: D6PKL2, PK5 | D6PKL2 (D6 PROTEIN KINASE LIKE 2); kinase | chr5:19339947-19341864 REVERSEhighly similar to ( 711) G11A_ORYSA Protein kinase G11A (EC 2.7.11.1) - Oryza sativa (Rice)highly similar to ( 756) loc_os08g38320 12008.m080198 protein protein kinase G11A, putative, expressed no original description T

26.7 misc.oxidases - copper, flavone etc MDP0000214959 moderately similar to ( 300) AT5G61510 | Symbols: | NADP-dependent oxidoreductase, putative | chr5:24737084-24738975 REVERSEmoderately similar to ( 279) loc_os10g41170 12010.m06881 protein quinone oxidoreductase, putative, expressed no original description T

33.99 development.unspecified MDP0000362286 weakly similar to ( 128) AT1G28330 | Symbols: | DYL1 (DORMANCY-ASSOCIATED PROTEIN-LIKE 1) | chr1:9934134-9935216 REVERSEweakly similar to ( 161) 12KD_FRAAN Auxin-repressed 12.5 kDa protein - Fragaria ananassa (Strawberry)weakly similar to ( 123) loc_os11g44810 12011.m28867 protein auxin-repressed 12.5 kDa protein, putative, expressed no original description T

13.1.6.1.5 amino acid metabolism.synthesis.aromatic aa.chorismate.shikimate kinase MDP0000107676 moderately similar to ( 286) AT2G21940 | Symbols: | shikimate kinase, putative | chr2:9351106-9352881 FORWARDmoderately similar to ( 292) loc_os04g54800 12004.m35210 protein shikimate kinase, chloroplast precursor, putative, expressed no original description T

31.1 cell.organisation MDP0000238674 highly similar to ( 607) AT3G16060 | Symbols: | kinesin motor family protein | chr3:5447503-5451196 FORWARDweakly similar to ( 134) K125_TOBAC 125 kDa kinesin-related protein - Nicotiana tabacum (Common tobacco)highly similar to ( 605) loc_os01g43580 12001.m10620 protein BY-2 kinesin-like protein 10, putative, expressed no original description T

29.4.1.57 protein.postranslational modification.kinase.receptor like cytoplasmatic kinase VII MDP0000379697 moderately similar to ( 379) AT1G21240 | Symbols: WAK3 | WAK3 (wall associated kinase 3); kinase/ protein serine/threonine kinase | chr1:7434303-7436702 FORWARDmoderately similar to ( 218) CRI4_MAIZE Putative receptor protein kinase CRINKLY4 precursor (EC 2.7.11.1) - Zea mays (Maize)moderately similar to ( 366) loc_os02g56630 12002.m10685 protein OsWAK24 - OsWAK receptor-like protein kinase no original description T

34.99 transport.misc MDP0000139929 weakly similar to ( 152) AT1G20925 | Symbols: | auxin efflux carrier family protein | chr1:7290612-7292507 FORWARDweakly similar to ( 126) loc_os09g31478 12009.m06269 protein auxin hydrogen symporter, putative, expressed no original description T

1.3.7 PS.calvin cycle.FBPase MDP0000237918 moderately similar to ( 245) AT1G43670 | Symbols: | fructose-1,6-bisphosphatase, putative / D-fructose-1,6-bisphosphate 1-phosphohydrolase, putative / FBPase, putative | chr1:16468184-16470347 FORWARDmoderately similar to ( 266) F16P2_SACHY Fructose-1,6-bisphosphatase, cytosolic (EC 3.1.3.11) (D-fructose-1,6-bisphosphate 1-phosphohydrolase) (FBPase) - Saccharum hybrid (Sugarcane)moderately similar to ( 255) loc_os01g64660 12001.m43295 protein fructose-1,6-bisphosphatase, cytosolic, putative, expressed no original description T

31.4 cell.vesicle transport MDP0000140237 nearly identical (1415) AT4G11380 | Symbols: | beta-adaptin, putative | chr4:6920608-6925444 FORWARDnearly identical (1395) loc_os03g23950 12003.m07741 protein AP-1 complex subunit beta-1, expressed no original description T

13.2.6.3 amino acid metabolism.degradation.aromatic aa.tryptophan MDP0000313624 highly similar to ( 502) AT1G06550 | Symbols: | enoyl-CoA hydratase/isomerase family protein | chr1:2003834-2006564 REVERSEmoderately similar to ( 300) RL18A_ORYSA 60S ribosomal protein L18a - Oryza sativa (Rice)moderately similar to ( 471) loc_os01g54860 12001.m11647 protein 3-hydroxyisobutyryl-CoA hydrolase/ catalytic, putative, expressed no original description T

29.4 protein.postranslational modification MDP0000145009 highly similar to ( 617) AT4G28880 | Symbols: ckl3 | ckl3 (Casein Kinase I-like 3); ATP binding / kinase/ protein kinase/ protein serine/threonine kinase | chr4:14251351-14254048 FORWARDvery weakly similar to (67.4) CDPK_DAUCA Calcium-dependent protein kinase (EC 2.7.11.1) (CDPK) - Daucus carota (Carrot)highly similar to ( 560) loc_os02g17910 12002.m33640 protein casein kinase I isoform delta-like, putative, expressed no original description T

17.2.2 hormone metabolism.auxin.signal transduction MDP0000297331 moderately similar to ( 472) AT5G16530 | Symbols: PIN5 | PIN5 (PIN-FORMED 5); auxin:hydrogen symporter/ transporter | chr5:5400735-5402626 FORWARDmoderately similar to ( 406) PIN6_ORYSA Probable auxin efflux carrier component 6 (OsPIN6) - Oryza sativa (Rice)moderately similar to ( 406) loc_os01g69070 12001.m13008 protein auxin efflux carrier component 6, putative, expressed no original description T

26.4.1 misc.beta 1,3 glucan hydrolases.glucan endo-1,3-beta-glucosidase MDP0000281216 weakly similar to ( 170) AT3G56290 | Symbols: | unknown protein | chr3:20878743-20879541 REVERSEvery weakly similar to (96.7) GUB_NICPL Lichenase precursor (EC 3.2.1.73) (Endo-beta-1,3-1,4 glucanase) - Nicotiana plumbaginifolia (Leadwort-leaved tobacco)weakly similar to ( 168) loc_os01g60830 12001.m12211 protein expressed protein no original description T

35.2 not assigned.unknown MDP0000365159 no original description T

29.5.11.4.3.2 protein.degradation.ubiquitin.E3.SCF.FBOX MDP0000282778 very weakly similar to (77.8) AT4G14103 | Symbols: | F-box family protein | chr4:8126948-8128305 FORWARDvery weakly similar to (72.4) loc_os01g57920 12001.m11940 protein F-box domain containing protein, expressed no original description T

11.9.4.5 lipid metabolism.lipid degradation.beta-oxidation.acyl-CoA thioesterase MDP0000309977 weakly similar to ( 184) AT1G04290 | Symbols: | thioesterase family protein | chr1:1147721-1148352 REVERSEweakly similar to ( 145) loc_os04g35590 12004.m08648 protein thioesterase superfamily member 2, putative, expressed no original description T

35.2 not assigned.unknown MDP0000077736 highly similar to ( 726) AT1G12640 | Symbols: | membrane bound O-acyl transferase (MBOAT) family protein | chr1:4303586-4305666 REVERSEhighly similar to ( 659) loc_os02g45344 12002.m09571 protein acyltransferase, putative, expressed no original description T

26.10 misc.cytochrome P450 MDP0000874252 highly similar to ( 762) AT5G04660 | Symbols: CYP77A4 | CYP77A4; electron carrier/ heme binding / iron ion binding / monooxygenase/ oxygen binding | chr5:1336049-1337587 FORWARDhighly similar to ( 773) C77A3_SOYBN Cytochrome P450 77A3 (EC 1.14.-.-) - Glycine max (Soybean)highly similar to ( 529) loc_os04g33370 12004.m08433 protein cytochrome P450 77A2, putative, expressed no original description T

35.2 not assigned.unknown MDP0000377836 moderately similar to ( 219) AT2G15760 | Symbols: | calmodulin-binding protein | chr2:6865807-6866754 REVERSEweakly similar to ( 103) loc_os07g42740 12007.m08504 protein calmodulin binding protein, putative, expressed no original description T

27.3.21 RNA.regulation of transcription.GRAS transcription factor family MDP0000827871 moderately similar to ( 345) AT3G49950 | Symbols: | scarecrow transcription factor family protein | chr3:18522570-18523802 FORWARDweakly similar to ( 154) CIGR2_ORYSA Chitin-inducible gibberellin-responsive protein 2 - Oryza sativa (Rice)moderately similar to ( 412) loc_os05g42130 12005.m08386 protein protein MONOCULM 1, putative, expressed no original description T

30.2.3 signalling.receptor kinases.leucine rich repeat III MDP0000259272 highly similar to ( 856) AT3G24660 | Symbols: TMKL1 | TMKL1 (transmembrane kinase-like 1); ATP binding / kinase/ protein serine/threonine kinase | chr3:9003641-9005751 FORWARDweakly similar to ( 187) RPK1_IPONI Receptor-like protein kinase precursor (EC 2.7.11.1) - Ipomoea nil (Japanese morning glory) (Pharbitis nil)highly similar to ( 508) loc_os08g45060 12008.m08476 protein protein Kinase-like protein TMKL1 precursor, putative, expressed no original description T

35.2 not assigned.unknown MDP0000756650 very weakly similar to (53.1) AT5G53880 | Symbols: | unknown protein | chr5:21872995-21873195 REVERSE no original description T

35.2 not assigned.unknown MDP0000405179 weakly similar to ( 118) AT3G02680 | Symbols: NBS1, ATNBS1 | NBS1 (NIJMEGEN BREAKAGE SYNDROME 1) | chr3:576378-579226 FORWARDvery weakly similar to (96.3) loc_os10g34580 12010.m065293 protein FHA domain containing protein, expressed no original description T

35.2 not assigned.unknown MDP0000317371 weakly similar to ( 125) loc_os01g12110 12001.m07826 protein expressed protein no original description T

29.5.11.3 protein.degradation.ubiquitin.E2 MDP0000325082 moderately similar to ( 265) AT3G46460 | Symbols: UBC13 | UBC13 (ubiquitin-conjugating enzyme 13); ubiquitin-protein ligase | chr3:17096120-17097315 REVERSEmoderately similar to ( 239) UBC7_WHEAT Ubiquitin-conjugating enzyme E2 7 (EC 6.3.2.19) (Ubiquitin-protein ligase 7) (Ubiquitin carrier protein 7) - Triticum aestivum (Wheat)moderately similar to ( 259) loc_os01g62244 12001.m43509 protein ubiquitin-conjugating enzyme E2 7, putative, expressed no original description T

29.3.3 protein.targeting.chloroplast MDP0000356050 moderately similar to ( 491) AT3G20320 | Symbols: TGD2 | TGD2 (TRIGALACTOSYLDIACYLGLYCEROL2); lipid transporter/ phospholipid binding | chr3:7087657-7089640 REVERSEmoderately similar to ( 399) loc_os01g74280 12001.m13455 protein ABC-type transport system involved in resistance to organic solvents, periplasmic component, putative, expressed no original description T

35.2 not assigned.unknown MDP0000126049 moderately similar to ( 377) AT4G00440 | Symbols: | unknown protein | chr4:195176-198221 FORWARDweakly similar to ( 155) loc_os04g47180 12004.m35411 protein expressed protein no original description T

34.1 transport.p- and v-ATPases MDP0000916321 nearly identical (1639) AT1G68710 | Symbols: | haloacid dehalogenase-like hydrolase family protein | chr1:25793498-25797975 REVERSEvery weakly similar to (71.2) ACA2_ORYSA Calcium-transporting ATPase 2, plasma membrane-type (EC 3.6.3.8) (Ca(2+)-ATPase isoform 2) - Oryza sativa (Rice)nearly identical (1515) loc_os06g29380 12006.m07482 protein phospholipid-transporting ATPase 12, putative, expressed no original description T

30.2.3 signalling.receptor kinases.leucine rich repeat III MDP0000280908 highly similar to ( 685) AT1G48480 | Symbols: RKL1 | RKL1; ATP binding / kinase/ protein serine/threonine kinase | chr1:17918475-17920743 FORWARDweakly similar to ( 172) PSKR_DAUCA Phytosulfokine receptor precursor (EC 2.7.11.1) (Phytosulfokine LRR receptor kinase) - Daucus carota (Carrot)highly similar to ( 608) loc_os03g12250 12003.m06675 protein atypical receptor-like kinase MARK, putative, expressed no original description T

26.1 misc.misc2 MDP0000254363 moderately similar to ( 362) AT3G23600 | Symbols: | dienelactone hydrolase family protein | chr3:8473833-8475655 FORWARDweakly similar to ( 156) E134_MAIZE Endo-1,3;1,4-beta-D-glucanase precursor (EC 3.2.1.-) - Zea mays (Maize)moderately similar to ( 337) loc_os05g33100 12005.m07542 protein endo-1,3;1,4-beta-D-glucanase precursor, putative, expressed no original description T

4.1.15 glycolysis.cytosolic branch.phospho-enol-pyruvate carboxylase (PEPC) MDP0000291654 nearly identical (1701) AT1G53310 | Symbols: ATPPC1 | ATPPC1 (PHOSPHOENOLPYRUVATE CARBOXYLASE 1); catalytic/ phosphoenolpyruvate carboxylase | chr1:19884261-19888070 REVERSEnearly identical (1720) CAPP1_SOYBN Phosphoenolpyruvate carboxylase, housekeeping isozyme (EC 4.1.1.31) (PEPCase) (PEPC 1) - Glycine max (Soybean)nearly identical (1694) loc_os08g27840 12008.m26491 protein phosphoenolpyruvate carboxylase 2, putative, expressed no original description T

35.2 not assigned.unknown MDP0000130884 moderately similar to ( 484) AT5G25770 | Symbols: | unknown protein | chr5:8969308-8971806 REVERSEmoderately similar to ( 479) loc_os06g06770 12006.m31988 protein expressed protein no original description T

28.1.3 DNA.synthesis/chromatin structure.histone MDP0000164539 weakly similar to ( 166) AT1G07790 | Symbols: HTB1 | HTB1; DNA binding | chr1:2413049-2413495 FORWARDweakly similar to ( 167) H2B1_MEDTR Probable histone H2B.1 - Medicago truncatula (Barrel medic)weakly similar to ( 162) loc_os01g62230 12001.m12346 protein histone H2B.4, putative, expressed no original description T

35.2 not assigned.unknown MDP0000208326 nearly identical (1085) AT5G12950 | Symbols: | catalytic | chr5:4093117-4096806 FORWARDhighly similar to ( 944) loc_os02g10190 12002.m06317 protein secreted protein, putative, expressed no original description T

29.2.3 protein.synthesis.initiation MDP0000201872 moderately similar to ( 288) AT1G13950 | Symbols: EIF-5A, ELF5A-1, ATELF5A-1, EIF5A | ELF5A-1 (EUKARYOTIC ELONGATION FACTOR 5A-1); translation initiation factor | chr1:4773631-4774668 FORWARDmoderately similar to ( 312) IF5A2_NICPL Eukaryotic translation initiation factor 5A-2 (eIF-5A-2) (eIF-4D) - Nicotiana plumbaginifolia (Leadwort-leaved tobacco)moderately similar to ( 304) loc_os12g32240 12012.m07030 protein eukaryotic translation initiation factor 5A-2, putative, expressed no original description T

13.1.5.1.2 amino acid metabolism.synthesis.serine-glycine-cysteine group.serine.phosphoserine aminotransferase MDP0000555175 highly similar to ( 636) AT4G35630 | Symbols: PSAT | PSAT; O-phospho-L-serine:2-oxoglutarate aminotransferase | chr4:16904205-16905497 FORWARDhighly similar to ( 582) SERC_SPIOL Phosphoserine aminotransferase, chloroplast precursor (EC 2.6.1.52) (PSAT) - Spinacia oleracea (Spinach)highly similar to ( 531) loc_os03g06200 12003.m06140 protein phosphoserine aminotransferase, chloroplast precursor, putative, expressed no original description T

35.1 not assigned.no ontology MDP0000440005 moderately similar to ( 454) AT3G44190 | Symbols: | pyridine nucleotide-disulphide oxidoreductase family protein | chr3:15902004-15903402 REVERSEmoderately similar to ( 407) loc_os02g05680 12002.m05916 protein disulfide oxidoreductase/ electron carrier/ oxidoreductase, putative, expressed no original description T

29.5.11.4.3.2 protein.degradation.ubiquitin.E3.SCF.FBOX MDP0000297094 weakly similar to ( 154) AT1G13570 | Symbols: | F-box family protein | chr1:4642528-4643930 REVERSEvery weakly similar to (84.7) loc_os09g27090 12009.m05861 protein F-box domain containing protein no original description T

35.2 not assigned.unknown MDP0000125594 moderately similar to ( 496) AT2G46890 | Symbols: | oxidoreductase, acting on the CH-CH group of donors | chr2:19266879-19268134 REVERSEmoderately similar to ( 420) loc_os04g01510 12004.m05468 protein membrane protein, putative, expressed no original description T

35.1 not assigned.no ontology MDP0000662344 moderately similar to ( 409) AT2G41250 | Symbols: | haloacid dehalogenase-like hydrolase family protein | chr2:17200862-17202551 REVERSEmoderately similar to ( 400) loc_os07g46520 12007.m08869 protein rhythmically expressed gene 2 protein, putative, expressed no original description T

35.2 not assigned.unknown MDP0000369052 no original description T

35.1 not assigned.no ontology MDP0000122563 moderately similar to ( 207) AT1G28280 | Symbols: | VQ motif-containing protein | chr1:9886297-9887395 REVERSEweakly similar to ( 165) loc_os05g44270 12005.m08553 protein DNA-binding WRKY, putative, expressed no original description T

33.99 development.unspecified MDP0000129392 weakly similar to ( 112) AT3G26640 | Symbols: LWD2 | LWD2 (LIGHT-REGULATED WD 2); nucleotide binding | chr3:9793276-9794316 FORWARDweakly similar to ( 108) loc_os02g32430 12002.m08336 protein WD40 repeat protein, putative, expressed no original description T

29.5.11.4.2 protein.degradation.ubiquitin.E3.RING MDP0000327154 weakly similar to ( 152) AT2G15580 | Symbols: | zinc finger (C3HC4-type RING finger) family protein | chr2:6797687-6798815 FORWARDvery weakly similar to (62.4) EL5_ORYSA E3 ubiquitin-protein ligase EL5 (EC 6.3.2.-) - Oryza sativa (Rice)weakly similar to ( 132) loc_os02g55480 12002.m77860 protein protein binding protein, putative, expressed no original description T

33.99 development.unspecified MDP0000305070 highly similar to ( 811) AT3G01930 | Symbols: | nodulin family protein | chr3:319289-321488 REVERSEhighly similar to ( 818) loc_os08g42010 12008.m08179 protein nodulin-like protein, putative, expressed no original description T

3.6 minor CHO metabolism.callose MDP0000695519 highly similar to ( 552) AT2G36850 | Symbols: ATGSL08, GSL8, GSL08, ATGSL8 | GSL8 (GLUCAN SYNTHASE-LIKE 8); 1,3-beta-glucan synthase/ transferase, transferring glycosyl groups | chr2:15454935-15469666 REVERSEhighly similar to ( 545) loc_os06g02260 12006.m71394 protein callose synthase catalytic subunit, putative, expressed no original description T

11.1.8 lipid metabolism.FA synthesis and FA elongation.acyl coa ligase MDP0000319265 highly similar to ( 961) AT1G55320 | Symbols: AAE18 | AAE18 (ACYL-ACTIVATING ENZYME 18); catalytic/ ligase | chr1:20634194-20636659 FORWARDhighly similar to ( 867) loc_os03g59080 12003.m10809 protein acyl-activating enzyme 18, putative, expressed no original description T

35.2 not assigned.unknown MDP0000194587 moderately similar to ( 488) AT5G59500 | Symbols: | unknown protein | chr5:23986024-23987214 FORWARD no original description T

31.1 cell.organisation MDP0000044499 nearly identical (1445) AT4G30160 | Symbols: VLN4, ATVLN4 | VLN4 (ARABIDOPSIS THALIANA VILLIN 4); actin binding | chr4:14754528-14759511 FORWARDnearly identical (1328) loc_os06g44890 12006.m09016 protein villin-4, putative, expressed no original description T

35.1 not assigned.no ontology MDP0000775229 weakly similar to ( 173) AT1G17140 | Symbols: | tropomyosin-related | chr1:5856740-5857861 REVERSEvery weakly similar to (62.4) loc_os01g55280 12001.m11687 protein expressed protein no original description T

7.1.3 OPP.oxidative PP.6-phosphogluconate dehydrogenase MDP0000191398 highly similar to ( 854) AT3G02360 | Symbols: | 6-phosphogluconate dehydrogenase family protein | chr3:482498-483958 FORWARDhighly similar to ( 820) loc_os06g02144 12006.m091574 protein 6-phosphogluconate dehydrogenase, decarboxylating, putative, expressed no original description T

35.1 not assigned.no ontology MDP0000154653 moderately similar to ( 213) AT3G29760 | Symbols: | NLI interacting factor (NIF) family protein | chr3:11590064-11591833 REVERSEmoderately similar to ( 266) loc_os07g01850 12007.m04663 protein NLI interacting factor-like phosphatase family protein, expressed no original description T

29.2.1.2.2.17 protein.synthesis.ribosomal protein.eukaryotic.60S subunit.L17 MDP0000140221 moderately similar to ( 280) AT1G27400 | Symbols: | 60S ribosomal protein L17 (RPL17A) | chr1:9515230-9516725 FORWARDmoderately similar to ( 270) RL17_MAIZE 60S ribosomal protein L17 - Zea mays (Maize)moderately similar to ( 270) loc_os09g08430 12009.m04110 protein 60S ribosomal protein L17, putative, expressed no original description T

28.1 DNA.synthesis/chromatin structure MDP0000208675 moderately similar to ( 306) AT1G74560 | Symbols: NRP1 | NRP1 (NAP1-RELATED PROTEIN 1); DNA binding / chromatin binding / histone binding | chr1:28017763-28019900 REVERSEmoderately similar to ( 263) loc_os02g36710 12002.m08758 protein protein SET, putative, expressed no original description T

35.1.5 not assigned.no ontology.pentatricopeptide (PPR) repeat-containing protein MDP0000230038 moderately similar to ( 407) AT1G08070 | Symbols: | pentatricopeptide (PPR) repeat-containing protein | chr1:2514374-2516599 REVERSEweakly similar to ( 101) RF1_ORYSA Rf1 protein, mitochondrial precursor (PPR protein) (Fertility restorer) (Restorer for CMS) - Oryza sativa (Rice)highly similar to ( 527) loc_os11g14980 12011.m05634 protein selenium-binding protein-like, putative, expressed no original description T

29.3.3 protein.targeting.chloroplast MDP0000755889 moderately similar to ( 276) AT2G15290 | Symbols: ATTIC21, TIC21, CIA5, PIC1 | TIC21 (TRANSLOCON AT INNER MEMBRANE OF CHLOROPLASTS 21); copper uptake transmembrane transporter/ iron ion transmembrane transporter/ protein homodimerization | chr2:6642512-6644011 REVERSEmoderately similar to ( 256) loc_os02g09470 12002.m06244 protein expressed protein no original description T

11.9.4.13 lipid metabolism.lipid degradation.beta-oxidation.acyl CoA reductase MDP0000120742 moderately similar to ( 324) AT4G33790 | Symbols: CER4, G7, FAR3 | CER4 (ECERIFERUM 4); fatty acyl-CoA reductase (alcohol-forming)/ oxidoreductase, acting on the CH-CH group of donors | chr4:16204325-16207891 REVERSEmoderately similar to ( 259) loc_os08g20200 12008.m06087 protein male sterility protein 2, putative, expressed no original description T

20.2.1 stress.abiotic.heat MDP0000152564 weakly similar to ( 127) AT1G54050 | Symbols: | 17.4 kDa class III heat shock protein (HSP17.4-CIII) | chr1:20179558-20180122 REVERSEvery weakly similar to (79.7) HSP21_SOYBN 17.9 kDa class II heat shock protein - Glycine max (Soybean)very weakly similar to ( 100) loc_os02g54140 12002.m10443 protein 17.5 kDa class II heat shock protein, putative, expressed no original description T

11.9.2.1 lipid metabolism.lipid degradation.lipases.triacylglycerol lipase MDP0000217952 moderately similar to ( 437) AT4G10955 | Symbols: | lipase class 3 family protein | chr4:6713778-6715372 REVERSEmoderately similar to ( 407) loc_os02g18480 12002.m07091 protein triacylglycerol lipase, putative, expressed no original description T

35.2 not assigned.unknown MDP0000322577 weakly similar to ( 112) AT1G64690 | Symbols: | unknown protein | chr1:24038069-24038890 FORWARDvery weakly similar to (92.0) loc_os10g41300 12010.m06894 protein expressed protein no original description T

23.1.2.20 nucleotide metabolism.synthesis.purine.adenylosuccinate synthase MDP0000849495 highly similar to ( 779) AT3G57610 | Symbols: ADSS | ADSS (ADENYLOSUCCINATE SYNTHASE); adenylosuccinate synthase | chr3:21334519-21336603 REVERSEhighly similar to ( 726) PURA_WHEAT Adenylosuccinate synthetase, chloroplast precursor (EC 6.3.4.4) (IMP--aspartate ligase) (AdSS) (AMPSase) (Fragment) - Triticum aestivum (Wheat)highly similar to ( 717) loc_os03g07840 12003.m06296 protein adenylosuccinate synthetase, chloroplast precursor, putative, expressed no original description T

34.9 transport.metabolite transporters at the mitochondrial membrane MDP0000321445 moderately similar to ( 380) AT3G51870 | Symbols: | binding / transporter | chr3:19243978-19246611 FORWARDvery weakly similar to (82.8) ADT1_MAIZE ADP,ATP carrier protein 1, mitochondrial precursor (ADP/ATP translocase 1) (Adenine nucleotide translocator 1) (ANT 1) - Zea mays (Maize)moderately similar to ( 319) loc_os01g16040 12001.m08201 protein protein brittle-1, chloroplast precursor, putative, expressed no original description T

26.10 misc.cytochrome P450 MDP0000941955 highly similar to ( 788) AT2G45970 | Symbols: CYP86A8, LCR | CYP86A8; fatty acid (omega-1)-hydroxylase/ oxygen binding | chr2:18912548-18914161 REVERSEweakly similar to ( 107) F3PH_PETHY Flavonoid 3'-monooxygenase (EC 1.14.13.21) (Flavonoid 3'-hydroxylase) (Cytochrome P450 75B2) - Petunia hybrida (Petunia)highly similar to ( 732) loc_os04g47250 12004.m09671 protein cytochrome P450 86A2, putative, expressed no original description T

26.10 misc.cytochrome P450 MDP0000241006 very weakly similar to (65.1) AT3G26180 | Symbols: CYP71B20 | CYP71B20; electron carrier/ heme binding / iron ion binding / monooxygenase/ oxygen binding | chr3:9578407-9579993 REVERSEvery weakly similar to (62.4) C71DB_LOTJA Cytochrome P450 71D11 (EC 1.14.-.-) (Fragment) - Lotus japonicusvery weakly similar to (61.6) loc_os03g37080 12003.m08820 protein cytochrome P450 71E1, putative no original description T

35.2 not assigned.unknown MDP0000272426 no original description T

29.4 protein.postranslational modification MDP0000195541 highly similar to ( 874) AT3G56760 | Symbols: | calcium-dependent protein kinase, putative / CDPK, putative | chr3:21020661-21023756 REVERSEhighly similar to ( 759) CRK_DAUCA CDPK-related protein kinase (EC 2.7.11.1) (PK421) - Daucus carota (Carrot)highly similar to ( 832) loc_os07g44710 12007.m08692 protein CDPK-related protein kinase, putative, expressed no original description T

35.2 not assigned.unknown MDP0000369858 weakly similar to ( 177) AT3G25400 | Symbols: | FUNCTIONS IN: molecular_function unknown; INVOLVED IN: biological_process unknown; LOCATED IN: cellular_component unknown; EXPRESSED IN: 11 plant structures; EXPRESSED DURING: 6 growth stages; CONTAINS InterPro DOMAIN/s: NTP Pyrophosphohydrolase MazG-related, RS21-C6 (InterPro:IPR011394), EAR (InterPro:IPR009039), NTP pyrophosphohydrolase MazG, putative catalytic core (InterPro:IPR004518); Has 572 Blast hits to 572 proteins in 181 species: Archae - 11; Bacteria - 286; Metazoa - 71; Fungi - 1; Plants - 31; Viruses - 0; Other Eukaryotes - 172 (source: NCBI BLink). | chr3:9213236-9214144 FORWARDweakly similar to ( 169) loc_os02g27810 12002.m07925 protein RS21-C6 protein, putative, expressed no original description T

29.2.1.2.2.37 protein.synthesis.ribosomal protein.eukaryotic.60S subunit.L37 MDP0000836948 weakly similar to ( 173) AT1G15250 | Symbols: | 60S ribosomal protein L37 (RPL37A) | chr1:5248825-5249381 REVERSEweakly similar to ( 176) loc_os02g56990 12002.m10721 protein 60S ribosomal protein L37, putative, expressed no original description T

23.5.5 nucleotide metabolism.deoxynucleotide metabolism.dUTP diphosphatase MDP0000170290 moderately similar to ( 226) AT3G46940 | Symbols: | deoxyuridine 5'-triphosphate nucleotidohydrolase family | chr3:17288367-17288867 REVERSEweakly similar to ( 198) loc_os03g46640 12003.m09682 protein deoxyuridine 5-triphosphate nucleotidohydrolase, putative, expressed no original description T

35.2 not assigned.unknown MDP0000716430 weakly similar to ( 129) AT3G07565 | Symbols: | DNA binding | chr3:2413823-2415872 FORWARDweakly similar to ( 117) loc_os05g24000 12005.m06694 protein expressed protein MSP1_C no original description T

35.2 not assigned.unknown MDP0000176579 moderately similar to ( 343) AT3G21200 | Symbols: | unknown protein | chr3:7436091-7437845 FORWARDmoderately similar to ( 278) loc_os08g15500 12008.m05673 protein expressed protein no original description T

35.2 not assigned.unknown MDP0000246935 very weakly similar to (56.6) AT1G11120 | Symbols: | unknown protein | chr1:3715229-3717320 FORWARD no original description T

35.2 not assigned.unknown MDP0000154070 no original description T

27.1 RNA.processing MDP0000933608 weakly similar to ( 124) AT4G03120 | Symbols: | proline-rich family protein | chr4:1385747-1387331 FORWARDweakly similar to ( 122) loc_os02g16640 12002.m06907 protein WW domain-binding protein 11, putative, expressed no original description T

17.5.3 hormone metabolism.ethylene.induced-regulated-responsive-activated MDP0000168695 weakly similar to ( 200) AT1G27660 | Symbols: | ethylene-responsive protein -related | chr1:9621701-9625666 FORWARDweakly similar to ( 149) loc_os01g01600 12001.m06805 protein expressed protein no original description T

29.3.4.3 protein.targeting.secretory pathway.vacuole MDP0000274790 moderately similar to ( 321) AT4G05000 | Symbols: VPS28-1 | VPS28-2; transporter | chr4:2563953-2564585 FORWARDmoderately similar to ( 246) loc_os01g57260 12001.m11879 protein VPS28 protein homolog 2, putative, expressed no original description T

29.3.4.99 protein.targeting.secretory pathway.unspecified MDP0000274790 moderately similar to ( 321) AT4G05000 | Symbols: VPS28-1 | VPS28-2; transporter | chr4:2563953-2564585 FORWARDmoderately similar to ( 246) loc_os01g57260 12001.m11879 protein VPS28 protein homolog 2, putative, expressed no original description T

29.2.1.2.2.28 protein.synthesis.ribosomal protein.eukaryotic.60S subunit.L28 MDP0000667499 moderately similar to ( 224) AT2G19730 | Symbols: | 60S ribosomal protein L28 (RPL28A) | chr2:8511752-8512995 FORWARDmoderately similar to ( 223) loc_os01g51020 12001.m11283 protein 60S ribosomal protein L28, putative, expressed no original description T

30.2.17 signalling.receptor kinases.DUF 26 MDP0000578117 moderately similar to ( 312) AT5G38280 | Symbols: PR5K | PR5K; kinase/ transmembrane receptor protein serine/threonine kinase | chr5:15293325-15295838 REVERSEweakly similar to ( 177) KPRO_MAIZE Putative receptor protein kinase ZmPK1 precursor (EC 2.7.11.1) - Zea mays (Maize)moderately similar to ( 357) loc_os01g02790 12001.m06919 protein receptor kinase LRK14, putative, expressed no original description T

27.3.35 RNA.regulation of transcription.bZIP transcription factor family MDP0000286846 moderately similar to ( 453) AT2G35530 | Symbols: | bZIP transcription factor family protein | chr2:14923280-14926025 REVERSEmoderately similar to ( 270) HBP1A_WHEAT Transcription factor HBP-1a (Histone-specific transcription factor HBP1) - Triticum aestivum (Wheat)moderately similar to ( 351) loc_os12g13170 12012.m26746 protein transcription factor HBP-1a, putative, expressed MSP1_C no original description T

29.2.3 protein.synthesis.initiation MDP0000217867 nearly identical (1037) AT4G11420 | Symbols: EIF3A, ATEIF3A-1, EIF3A-1, ATTIF3A1, TIF3A1 | EIF3A (EUKARYOTIC TRANSLATION INITIATION FACTOR 3A); translation initiation factor | chr4:6947834-6952053 REVERSEhighly similar to ( 993) IF3A_TOBAC Eukaryotic translation initiation factor 3 subunit 10 (eIF-3 theta) (Eukaryotic translation initiation factor 3 large subunit) (eIF3a) (PNLA-35) - Nicotiana tabacum (Common tobacco)highly similar to ( 944) loc_os01g03070 12001.m42594 protein eukaryotic translation initiation factor 3 subunit 10, putative, expressed PRK07003 no original description T

34.10 transport.nucleotides MDP0000330970 moderately similar to ( 240) AT3G26670 | Symbols: | FUNCTIONS IN: molecular_function unknown; INVOLVED IN: biological_process unknown; LOCATED IN: cellular_component unknown; EXPRESSED IN: 23 plant structures; EXPRESSED DURING: 15 growth stages; CONTAINS InterPro DOMAIN/s: Protein of unknown function DUF803 (InterPro:IPR008521); BEST Arabidopsis thaliana protein match is: permease-related (TAIR:AT3G23870.1); Has 715 Blast hits to 711 proteins in 119 species: Archae - 0; Bacteria - 0; Metazoa - 298; Fungi - 244; Plants - 119; Viruses - 2; Other Eukaryotes - 52 (source: NCBI BLink). | chr3:9798236-9800562 REVERSEmoderately similar to ( 222) loc_os02g31874 12002.m08280 protein expressed protein no original description T

19.10 tetrapyrrole synthesis.magnesium chelatase MDP0000639265 highly similar to ( 595) AT4G18480 | Symbols: CHLI1, CH42, CH-42, CHL11, CHLI-1 | CHLI1; ATPase/ magnesium chelatase | chr4:10201897-10203361 REVERSEhighly similar to ( 645) CHLI_SOYBN Magnesium-chelatase subunit chlI, chloroplast precursor (EC 6.6.1.1) (Mg-protoporphyrin IX chelatase) - Glycine max (Soybean)highly similar to ( 569) loc_os03g36540 12003.m08774 protein magnesium-chelatase subunit chlI, chloroplast precursor, putative, expressed no original description T

29.1.19 protein.aa activation.arginine-tRNA ligase MDP0000340127 moderately similar to ( 374) AT4G26300 | Symbols: emb1027 | emb1027 (embryo defective 1027); ATP binding / aminoacyl-tRNA ligase/ arginine-tRNA ligase/ nucleotide binding | chr4:13308400-13313109 REVERSEmoderately similar to ( 350) loc_os05g07030 12005.m083624 protein arginyl-tRNA synthetase, putative, expressed no original description T

33.2 development.late embryogenesis abundant MDP0000294557 weakly similar to ( 193) AT1G01470 | Symbols: LEA14, LSR3 | LEA14 (LATE EMBRYOGENESIS ABUNDANT 14) | chr1:172295-172826 REVERSEmoderately similar to ( 227) LEA14_SOYBN Desiccation protectant protein Lea14 homolog - Glycine max (Soybean)weakly similar to ( 177) loc_os05g50710 12005.m09138 protein late embryogenesis abundant protein Lea14-A, putative, expressed no original description T

26.3 misc.gluco-, galacto- and mannosidases MDP0000234499 moderately similar to ( 476) AT5G42260 | Symbols: BGLU12 | BGLU12 (BETA GLUCOSIDASE 12); catalytic/ cation binding / hydrolase, hydrolyzing O-glycosyl compounds | chr5:16898712-16900235 FORWARDmoderately similar to ( 382) BGLC_MAIZE Beta-glucosidase, chloroplast precursor (EC 3.2.1.21) (Gentiobiase) (Cellobiase) (Beta-D-glucoside glucohydrolase) - Zea mays (Maize)highly similar to ( 570) loc_os04g39880 12004.m35159 protein non-cyanogenic beta-glucosidase precursor, putative, expressed no original description T

9.9 mitochondrial electron transport / ATP synthesis.F1-ATPase MDP0000448896 moderately similar to ( 242) AT5G13450 | Symbols: | ATP synthase delta chain, mitochondrial, putative / H(+)-transporting two-sector ATPase, delta (OSCP) subunit, putative | chr5:4310558-4311941 REVERSEmoderately similar to ( 297) ATPO_IPOBA ATP synthase delta chain, mitochondrial precursor (EC 3.6.3.14) (Oligomycin sensitivity conferral protein) (OSCP) - Ipomoea batatas (Sweet potato) (Batate)moderately similar to ( 246) loc_os06g43850 12006.m32124 protein ATP synthase delta chain, mitochondrial precursor, putative, expressed no original description T

27.3.50 RNA.regulation of transcription.General Transcription MDP0000777771 weakly similar to ( 189) AT4G10920 | Symbols: KELP | KELP; DNA binding / transcription coactivator/ transcription regulator | chr4:6697894-6699103 REVERSEweakly similar to ( 157) loc_os01g50960 12001.m11277 protein RNA polymerase II transcriptional coactivator KELP, putative, expressed no original description T

20.2.2 stress.abiotic.cold MDP0000202739 weakly similar to ( 110) AT2G21060 | Symbols: ATGRP2B | ATGRP2B (GLYCINE-RICH PROTEIN 2B); DNA binding / nucleic acid binding / zinc ion binding | chr2:9036983-9037588 REVERSEweakly similar to ( 120) GRP2_NICSY Glycine-rich protein 2 - Nicotiana sylvestris (Wood tobacco)weakly similar to ( 108) loc_os02g02870 12002.m05637 protein glycine-rich protein 2, putative, expressed no original description T

11.8.1.1 lipid metabolism.'exotics' (steroids, squalene etc).sphingolipids.ceramidase MDP0000221217 nearly identical (1072) AT2G38010 | Symbols: | ceramidase family protein | chr2:15906862-15909867 FORWARDnearly identical (1031) loc_os01g43520 12001.m150425 protein ceramidase, putative, expressed no original description T

27.1.19 RNA.processing.ribonucleases MDP0000216907 very weakly similar to (67.4) AT1G24020 | Symbols: MLP423 | MLP423 (MLP-LIKE PROTEIN 423) | chr1:8500653-8501458 REVERSEmoderately similar to ( 249) MAL12_MALDO Major allergen Mal d 1 (Mal d I) (AP15) - Malus domestica (Apple) (Malus sylvestris)very weakly similar to (96.7) loc_os03g18850 12003.m07305 protein pathogenesis-related protein 1, putative, expressed no original description T

20.2.99 stress.abiotic.unspecified MDP0000216907 very weakly similar to (67.4) AT1G24020 | Symbols: MLP423 | MLP423 (MLP-LIKE PROTEIN 423) | chr1:8500653-8501458 REVERSEmoderately similar to ( 249) MAL12_MALDO Major allergen Mal d 1 (Mal d I) (AP15) - Malus domestica (Apple) (Malus sylvestris)very weakly similar to (96.7) loc_os03g18850 12003.m07305 protein pathogenesis-related protein 1, putative, expressed no original description T

35.2 not assigned.unknown MDP0000173780 highly similar to ( 538) AT5G12900 | Symbols: | unknown protein | chr5:4072151-4074445 REVERSEhighly similar to ( 559) loc_os08g39740 12008.m07954 protein expressed protein no original description T

35.2 not assigned.unknown MDP0000716032 moderately similar to ( 276) AT5G11000 | Symbols: | unknown protein | chr5:3479166-3480335 REVERSEweakly similar to ( 173) loc_os03g59200 12003.m10820 protein expressed protein TrbL no original description T

10.1.5 cell wall.precursor synthesis.UXS MDP0000331463 very weakly similar to (96.3) AT2G28760 | Symbols: UXS6 | NAD-dependent epimerase/dehydratase family protein | chr2:12336469-12338642 REVERSEvery weakly similar to (84.3) loc_os03g16980 12003.m34836 protein UDP-glucuronic acid decarboxylase 1, putative, expressed no original description T

11.8.2 lipid metabolism.'exotics' (steroids, squalene etc).methylsterol monooxygenase MDP0000320408 moderately similar to ( 494) AT4G12110 | Symbols: SMO1-1, ATSMO1, ATSMO1-1 | SMO1-1 (STEROL-4ALPHA-METHYL OXIDASE 1-1); 4,4-dimethyl-9beta,19-cyclopropylsterol-4alpha-methyl oxidase/ C-4 methylsterol oxidase/ catalytic | chr4:7254197-7256004 FORWARDmoderately similar to ( 439) loc_os10g39810 12010.m065330 protein C-4 methylsterol oxidase, putative, expressed no original description T

27.3.3 RNA.regulation of transcription.AP2/EREBP, APETALA2/Ethylene-responsive element binding protein family MDP0000292965 weakly similar to ( 170) AT1G64380 | Symbols: | AP2 domain-containing transcription factor, putative | chr1:23890981-23891988 REVERSEvery weakly similar to (62.0) ERF1_ORYSA Ethylene-responsive transcription factor 1 (Ethylene-responsive element-binding factor 1) (EREBP-1) (OsEREBP1) - Oryza sativa (Rice)weakly similar to ( 120) loc_os05g49700 12005.m09040 protein DNA binding protein, putative, expressed no original description T

16.4.1 secondary metabolism.N misc.alkaloid-like MDP0000850540 highly similar to ( 577) AT2G20340 | Symbols: | tyrosine decarboxylase, putative | chr2:8779804-8782490 FORWARDhighly similar to ( 639) TYDC4_PETCR Tyrosine decarboxylase 4 (EC 4.1.1.25) - Petroselinum crispum (Parsley) (Petroselinum hortense)highly similar to ( 526) loc_os07g25590 12007.m06837 protein tyrosine decarboxylase 1, putative, expressed no original description T

28.1 DNA.synthesis/chromatin structure MDP0000304488 highly similar to ( 544) AT5G16270 | Symbols: ATRAD21.3, SYN4 | SYN4 (SISTER CHROMATID COHESION 1 PROTEIN 4) | chr5:5316783-5322330 FORWARDmoderately similar to ( 336) loc_os01g67250 12001.m42786 protein N terminus of Rad21 / Rec8 like protein, expressed no original description T

29.5.11.4.3.2 protein.degradation.ubiquitin.E3.SCF.FBOX MDP0000278038 very weakly similar to (79.3) AT4G14103 | Symbols: | F-box family protein | chr4:8126948-8128305 FORWARDvery weakly similar to (61.6) loc_os08g09750 12008.m05109 protein F-box domain containing protein, expressed no original description T

29.5 protein.degradation MDP0000202199 moderately similar to ( 304) AT5G22860 | Symbols: | serine carboxypeptidase S28 family protein | chr5:7639907-7642945 REVERSEmoderately similar to ( 216) loc_os01g56150 12001.m11771 protein lysosomal Pro-X carboxypeptidase precursor, putative, expressed no original description T

35.2 not assigned.unknown MDP0000292018 no original description T

27.3.50 RNA.regulation of transcription.General Transcription MDP0000814056 weakly similar to ( 124) AT4G37740 | Symbols: AtGRF2 | AtGRF2 (GROWTHREGULATING FACTOR 2); transcription activator | chr4:17725533-17727609 REVERSEweakly similar to ( 122) loc_os12g29980 12012.m073831 protein atGRF2, putative, expressed no original description T

35.1.40 not assigned.no ontology.glycine rich proteins MDP0000710378 weakly similar to ( 145) AT4G02450 | Symbols: | glycine-rich protein | chr4:1073987-1075765 REVERSEweakly similar to ( 154) loc_os08g27070 12008.m06720 protein co-chaperone protein SBA1, putative, expressed no original description T

29.3.4.99 protein.targeting.secretory pathway.unspecified MDP0000322453 highly similar to ( 849) AT2G21520 | Symbols: | transporter | chr2:9215956-9218953 FORWARDhighly similar to ( 738) loc_os02g04020 12002.m05751 protein phosphatidylinositol transfer-like protein III, putative, expressed no original description T

21.4 redox.glutaredoxins MDP0000804078 weakly similar to ( 167) AT3G62950 | Symbols: | glutaredoxin family protein | chr3:23266303-23266614 FORWARDvery weakly similar to (68.9) GLRX_RICCO Glutaredoxin - Ricinus communis (Castor bean)weakly similar to ( 108) loc_os01g27140 12001.m09165 protein glutaredoxin, putative, expressed no original description T

35.2 not assigned.unknown MDP0000433653 weakly similar to ( 143) AT4G18590 | Symbols: | FUNCTIONS IN: molecular_function unknown; INVOLVED IN: biological_process unknown; LOCATED IN: cellular_component unknown; EXPRESSED IN: 23 plant structures; EXPRESSED DURING: 13 growth stages; CONTAINS InterPro DOMAIN/s: Replication factor A protein 3 (InterPro:IPR013970), Nucleic acid-binding, OB-fold-like (InterPro:IPR016027); BEST Arabidopsis thaliana protein match is: unknown protein (TAIR:AT3G52630.2); Has 58 Blast hits to 58 proteins in 25 species: Archae - 0; Bacteria - 0; Metazoa - 26; Fungi - 2; Plants - 26; Viruses - 0; Other Eukaryotes - 4 (source: NCBI BLink). | chr4:10236524-10236940 FORWARDweakly similar to ( 122) loc_os01g14980 12001.m150414 protein pollen-specific protein like, putative, expressed no original description T

26.21 misc.protease inhibitor/seed storage/lipid transfer protein (LTP) family protein MDP0000932449 weakly similar to ( 152) AT1G62510 | Symbols: | protease inhibitor/seed storage/lipid transfer protein (LTP) family protein | chr1:23136632-23137081 REVERSEweakly similar to ( 124) 14KD_DAUCA 14 kDa proline-rich protein DC2.15 precursor - Daucus carota (Carrot)weakly similar to ( 142) loc_os03g01320 12003.m34972 protein NT16 polypeptide, putative, expressed no original description T

15.2 metal handling.binding, chelation and storage MDP0000189389 moderately similar to ( 275) AT5G01600 | Symbols: ATFER1, FER1 | ATFER1; ferric iron binding / iron ion binding | chr5:228149-229594 REVERSEmoderately similar to ( 278) FRI3_SOYBN Ferritin-3, chloroplast precursor (EC 1.16.3.1) (SFerH-3) - Glycine max (Soybean)moderately similar to ( 255) loc_os12g01530 12012.m26727 protein ferritin-1, chloroplast precursor, putative, expressed no original description T

29.5.11.20 protein.degradation.ubiquitin.proteasom MDP0000592685 highly similar to ( 732) AT5G58290 | Symbols: RPT3 | RPT3 (REGULATORY PARTICLE TRIPLE-A ATPASE 3); ATPase | chr5:23569155-23571116 FORWARDhighly similar to ( 734) PRS6B_SOLTU 26S protease regulatory subunit 6B homolog - Solanum tuberosum (Potato)highly similar to ( 724) loc_os02g21970 12002.m07435 protein 26S protease regulatory subunit 6B, putative, expressed no original description T

29.5.9 protein.degradation.AAA type MDP0000592685 highly similar to ( 732) AT5G58290 | Symbols: RPT3 | RPT3 (REGULATORY PARTICLE TRIPLE-A ATPASE 3); ATPase | chr5:23569155-23571116 FORWARDhighly similar to ( 734) PRS6B_SOLTU 26S protease regulatory subunit 6B homolog - Solanum tuberosum (Potato)highly similar to ( 724) loc_os02g21970 12002.m07435 protein 26S protease regulatory subunit 6B, putative, expressed no original description T

35.2 not assigned.unknown MDP0000322608 weakly similar to ( 162) AT3G25400 | Symbols: | FUNCTIONS IN: molecular_function unknown; INVOLVED IN: biological_process unknown; LOCATED IN: cellular_component unknown; EXPRESSED IN: 11 plant structures; EXPRESSED DURING: 6 growth stages; CONTAINS InterPro DOMAIN/s: NTP Pyrophosphohydrolase MazG-related, RS21-C6 (InterPro:IPR011394), EAR (InterPro:IPR009039), NTP pyrophosphohydrolase MazG, putative catalytic core (InterPro:IPR004518); Has 572 Blast hits to 572 proteins in 181 species: Archae - 11; Bacteria - 286; Metazoa - 71; Fungi - 1; Plants - 31; Viruses - 0; Other Eukaryotes - 172 (source: NCBI BLink). | chr3:9213236-9214144 FORWARDweakly similar to ( 157) loc_os02g27810 12002.m07925 protein RS21-C6 protein, putative, expressed no original description T

35.2 not assigned.unknown MDP0000347520 no original description T

10.7 cell wall.modification MDP0000670959 moderately similar to ( 333) AT2G03090 | Symbols: ATEXPA15, EXP15, ATEXP15, ATHEXP ALPHA 1.3 | ATEXPA15 (ARABIDOPSIS THALIANA EXPANSIN A15) | chr2:917361-918554 REVERSEmoderately similar to ( 300) EXPA4_ORYSA Expansin-A4 precursor (OsEXPA4) (Alpha-expansin-4) (OsEXP4) (OsaEXPa1.22) - Oryza sativa (Rice)moderately similar to ( 300) loc_os05g39990 12005.m08176 protein alpha-expansin 1 precursor, putative, expressed no original description T

29.3.4.2 protein.targeting.secretory pathway.golgi MDP0000797875 moderately similar to ( 417) AT2G27460 | Symbols: | sec23/sec24 transport family protein | chr2:11740670-11744867 FORWARDmoderately similar to ( 353) loc_os05g37120 12005.m27849 protein sec23/Sec24 trunk domain containing protein, expressed no original description T

9.9 mitochondrial electron transport / ATP synthesis.F1-ATPase MDP0000336421 highly similar to ( 518) AT2G33040 | Symbols: | ATP synthase gamma chain, mitochondrial (ATPC) | chr2:14018978-14021047 REVERSEhighly similar to ( 541) ATPG3_IPOBA ATP synthase gamma chain, mitochondrial precursor (EC 3.6.3.14) - Ipomoea batatas (Sweet potato) (Batate)highly similar to ( 502) loc_os10g17280 12010.m04815 protein ATP synthase gamma chain, mitochondrial precursor, putative, expressedMSP1_C no original description T

35.2 not assigned.unknown MDP0000304878 weakly similar to ( 155) AT4G16410 | Symbols: | unknown protein | chr4:9262019-9262576 REVERSEweakly similar to ( 113) loc_os07g41630 12007.m08399 protein expressed protein no original description T

29.7.3 protein.glycosylation.mannosyl-oligosaccharide alpha-1,2-mannosidase MDP0000857857 highly similar to ( 862) AT1G51590 | Symbols: | mannosyl-oligosaccharide 1,2-alpha-mannosidase, putative | chr1:19128315-19131406 REVERSEhighly similar to ( 815) loc_os04g51690 12004.m10064 protein mannosyl-oligosaccharide 1,2-alpha-mannosidase IA, putative, expressed no original description T

30.2.11 signalling.receptor kinases.leucine rich repeat XI MDP0000851478 moderately similar to ( 498) AT5G61240 | Symbols: | protein binding | chr5:24629485-24631958 FORWARDweakly similar to ( 120) RPK1_IPONI Receptor-like protein kinase precursor (EC 2.7.11.1) - Ipomoea nil (Japanese morning glory) (Pharbitis nil)moderately similar to ( 449) loc_os03g11340 12003.m06585 protein leucine-rich repeat resistance protein, putative, expressed no original description T

26.8 misc.nitrilases, *nitrile lyases, berberine bridge enzymes, reticuline oxidases, troponine reductases MDP0000727481 moderately similar to ( 353) AT1G12570 | Symbols: | glucose-methanol-choline (GMC) oxidoreductase family protein | chr1:4278192-4280753 REVERSEmoderately similar to ( 412) loc_os04g48400 12004.m09776 protein protein HOTHEAD precursor, putative, expressed no original description T

34.99 transport.misc MDP0000524493 moderately similar to ( 464) AT1G64890 | Symbols: | integral membrane transporter family protein | chr1:24109752-24111165 FORWARDmoderately similar to ( 409) loc_os05g32320 12005.m07465 protein transporter, folate-biopterin transporter, putative, expressed no original description T

35.2 not assigned.unknown MDP0000807837 moderately similar to ( 212) AT2G30170 | Symbols: | catalytic | chr2:12879802-12881046 REVERSEmoderately similar to ( 209) loc_os01g07090 12001.m07336 protein protein phosphatase 2C homolog 7, putative, expressed no original description T

2.1.2.2 major CHO metabolism.synthesis.starch.starch synthase MDP0000122981 highly similar to ( 909) AT5G24300 | Symbols: SSI1, SSI, ATSS1 | SSI1 (SUPPRESSOR OF SALICYLIC ACID INSENSITIVITY 1); starch synthase/ transferase, transferring glycosyl groups | chr5:8266934-8270860 FORWARDhighly similar to ( 901) SSY1_SOLTU Soluble starch synthase 1, chloroplast precursor (EC 2.4.1.21) (SS I) (Soluble starch synthase I) - Solanum tuberosum (Potato)highly similar to ( 817) loc_os06g06560 12006.m05381 protein soluble starch synthase 1, chloroplast precursor, putative, expressed no original description T

27.3.46 RNA.regulation of transcription.DNA methyltransferases MDP0000251234 highly similar to ( 805) AT1G69770 | Symbols: CMT3 | CMT3 (chromomethylase 3); DNA (cytosine-5-)-methyltransferase | chr1:26248496-26253519 REVERSEhighly similar to ( 796) CMT1_MAIZE DNA (cytosine-5)-methyltransferase 1 (EC 2.1.1.37) (Chromomethylase 1) (Zea methyltransferase2) (Zmet2) (DNA cytosine methyltransferase MET2a) - Zea mays (Maize)highly similar to ( 766) loc_os03g12570 12003.m06707 protein DNA cytosine methyltransferase MET2a, putative, expressed PRK08026 TrbL no original description T

29.2.1.2.2.38 protein.synthesis.ribosomal protein.eukaryotic.60S subunit.L38 MDP0000153255 very weakly similar to ( 100) AT3G59540 | Symbols: | 60S ribosomal protein L38 (RPL38B) | chr3:21995897-21996742 REVERSEvery weakly similar to (97.4) loc_os11g24610 12011.m06387 protein 60S ribosomal protein L38, putative, expressed no original description T

35.2 not assigned.unknown MDP0000564469 no original description T

35.2 not assigned.unknown MDP0000532078 highly similar to ( 820) AT5G25757 | Symbols: | unknown protein | chr5:8965515-8967460 REVERSEhighly similar to ( 761) loc_os05g13950 12005.m083655 protein eukaryotic translation initiation factor 3 subunit 6-interacting protein, putative, expressed no original description T

35.2 not assigned.unknown MDP0000003818 nearly identical (2110) AT2G35110 | Symbols: GRL, NAP1, NAPP | GRL (GNARLED); transcription activator | chr2:14795970-14803525 REVERSEnearly identical (1838) NCKP1_ORYSA Probable protein NAP1 (Nck-associated protein 1) (P125Nap1) (NAP of plants) - Oryza sativa (Rice)nearly identical (1838) loc_os08g43130 12008.m08289 protein protein NAP1, putative, expressed no original description T

29.2.1.2.2.57 protein.synthesis.ribosomal protein.eukaryotic.60S subunit.L7A MDP0000424205 moderately similar to ( 381) AT3G62870 | Symbols: | 60S ribosomal protein L7A (RPL7aB) | chr3:23242862-23244273 REVERSEmoderately similar to ( 389) RL7A_ORYSA 60S ribosomal protein L7a - Oryza sativa (Rice)moderately similar to ( 389) loc_os09g32976 12009.m22160 protein 60S ribosomal protein L7a, putative, expressed no original description T

27.3.99 RNA.regulation of transcription.unclassified MDP0000238087 moderately similar to ( 486) AT1G01300 | Symbols: | aspartyl protease family protein | chr1:117065-118522 FORWARDvery weakly similar to (62.0) ASP1_ORYSA Aspartic proteinase Asp1 precursor (EC 3.4.23.-) (OsAsp1) (OSAP1) (Nucellin-like protein) - Oryza sativa (Rice)moderately similar to ( 381) loc_os01g41550 12001.m10423 protein aspartic proteinase nepenthesin-2 precursor, putative, expressed no original description T

23.3.2.2 nucleotide metabolism.salvage.nucleoside kinases.uridine kinase MDP0000260928 highly similar to ( 774) AT1G73980 | Symbols: | phosphoribulokinase/uridine kinase family protein | chr1:27820292-27823527 REVERSEvery weakly similar to (63.2) KPPR_MESCR Phosphoribulokinase, chloroplast precursor (EC 2.7.1.19) (Phosphopentokinase) (PRKase) (PRK) - Mesembryanthemum crystallinum (Common ice plant)highly similar to ( 767) loc_os02g47860 12002.m100333 protein uridine kinase, putative, expressed no original description T

29.4 protein.postranslational modification MDP0000183142 highly similar to ( 974) AT1G04210 | Symbols: | leucine-rich repeat family protein / protein kinase family protein | chr1:1114696-1119383 FORWARDvery weakly similar to (59.7) RPK1_IPONI Receptor-like protein kinase precursor (EC 2.7.11.1) - Ipomoea nil (Japanese morning glory) (Pharbitis nil)highly similar to ( 621) loc_os03g53250 12003.m10298 protein protein kinase domain containing protein, expressed no original description T

27.4 RNA.RNA binding MDP0000283985 highly similar to ( 752) AT1G49760 | Symbols: PAB8, PABP8 | PAB8 (POLY(A) BINDING PROTEIN 8); RNA binding / translation initiation factor | chr1:18416740-18419753 FORWARDvery weakly similar to (83.6) ROC1_NICSY 29 kDa ribonucleoprotein A, chloroplast precursor (CP29A) - Nicotiana sylvestris (Wood tobacco)highly similar to ( 769) loc_os09g02700 12009.m03642 protein polyadenylate-binding protein 2, putative, expressed no original description T

35.2 not assigned.unknown MDP0000298446 moderately similar to ( 401) AT5G10060 | Symbols: | FUNCTIONS IN: molecular_function unknown; INVOLVED IN: biological_process unknown; LOCATED IN: endomembrane system; CONTAINS InterPro DOMAIN/s: Protein of unknown function DUF618 (InterPro:IPR006903), Regulation of nuclear pre-mRNA protein (InterPro:IPR006569), ENTH/VHS (InterPro:IPR008942); BEST Arabidopsis thaliana protein match is: unknown protein (TAIR:AT5G65180.1); Has 4531 Blast hits to 4161 proteins in 412 species: Archae - 14; Bacteria - 404; Metazoa - 1999; Fungi - 666; Plants - 262; Viruses - 36; Other Eukaryotes - 1150 (source: NCBI BLink). | chr5:3145994-3148049 REVERSEmoderately similar to ( 344) loc_os04g54420 12004.m10333 protein expressed protein no original description T

35.2 not assigned.unknown MDP0000255843 moderately similar to ( 469) AT3G48860 | Symbols: | unknown protein | chr3:18117619-18120865 FORWARDvery weakly similar to (67.4) ACP1_SPIOL Acyl carrier protein 1, chloroplast precursor (Acyl carrier protein I) (ACP I) - Spinacia oleracea (Spinach)moderately similar to ( 439) loc_os01g70320 12001.m13080 protein expressed protein MSP1_C no original description T

16.4.1 secondary metabolism.N misc.alkaloid-like MDP0000166893 very weakly similar to (89.7) AT3G57030 | Symbols: | strictosidine synthase family protein | chr3:21101653-21103204 REVERSEvery weakly similar to (55.5) STSY_CATRO Strictosidine synthase precursor (EC 4.3.3.2) - Catharanthus roseus (Rosy periwinkle) (Madagascar periwinkle)very weakly similar to (76.6) loc_os12g04424 12012.m56468 protein strictosidine synthase 3 precursor, putative, expressed no original description T

33.99 development.unspecified MDP0000283081 moderately similar to ( 278) AT5G13170 | Symbols: SAG29 | SAG29 (SENESCENCE-ASSOCIATED PROTEIN 29) | chr5:4181331-4183171 REVERSEmoderately similar to ( 258) loc_os11g31190 12011.m07024 protein mtN3-like protein, putative, expressed no original description T

28.99 DNA.unspecified MDP0000261748 moderately similar to ( 309) AT3G18940 | Symbols: | clast3-related | chr3:6527081-6529050 REVERSEmoderately similar to ( 285) loc_os03g55784 12003.m10490 protein tumor necrosis factor superfamily, member 5-induced protein 1, putative, expressed no original description T

29.3.4.1 protein.targeting.secretory pathway.ER MDP0000920009 moderately similar to ( 270) AT2G21600 | Symbols: ATRER1B | ATRER1B | chr2:9243550-9244579 FORWARDmoderately similar to ( 287) loc_os06g49460 12006.m09470 protein RER1A protein, putative, expressed no original description T

19.2 tetrapyrrole synthesis.glu-tRNA reductase MDP0000172827 highly similar to ( 736) AT1G58290 | Symbols: HEMA1 | HEMA1; glutamyl-tRNA reductase | chr1:21624028-21626051 REVERSEhighly similar to ( 786) HEM11_CUCSA Glutamyl-tRNA reductase 1, chloroplast precursor (EC 1.2.1.70) (GluTR) - Cucumis sativus (Cucumber)highly similar to ( 635) loc_os10g35840 12010.m06402 protein glutamyl-tRNA reductase, chloroplast precursor, putative, expressed no original description T

30.2.11 signalling.receptor kinases.leucine rich repeat XI MDP0000131687 highly similar to ( 565) AT1G80080 | Symbols: TMM, AtRLP17 | TMM (TOO MANY MOUTHS); protein binding / receptor | chr1:30128073-30129563 REVERSEweakly similar to ( 134) RPK1_IPONI Receptor-like protein kinase precursor (EC 2.7.11.1) - Ipomoea nil (Japanese morning glory) (Pharbitis nil)moderately similar to ( 466) loc_os01g43440 12001.m10606 protein too many mouths protein precursor, putative no original description T

30.2.17 signalling.receptor kinases.DUF 26 MDP0000261851 highly similar to ( 876) AT4G27290 | Symbols: | ATP binding / protein kinase/ protein serine/threonine kinase/ protein tyrosine kinase/ sugar binding | chr4:13666281-13669202 FORWARDmoderately similar to ( 367) SLSG6_BRAOL S-locus-specific glycoprotein S6 precursor (SLSG-6) - Brassica oleracea (Wild cabbage)highly similar to ( 703) loc_os01g57560 12001.m11907 protein serine/threonine-protein kinase receptor precursor, putative, expressed no original description T

30.2.24 signalling.receptor kinases.S-locus glycoprotein like MDP0000261851 highly similar to ( 876) AT4G27290 | Symbols: | ATP binding / protein kinase/ protein serine/threonine kinase/ protein tyrosine kinase/ sugar binding | chr4:13666281-13669202 FORWARDmoderately similar to ( 367) SLSG6_BRAOL S-locus-specific glycoprotein S6 precursor (SLSG-6) - Brassica oleracea (Wild cabbage)highly similar to ( 703) loc_os01g57560 12001.m11907 protein serine/threonine-protein kinase receptor precursor, putative, expressed no original description T

35.2 not assigned.unknown MDP0000361998 no original description T

30.2.11 signalling.receptor kinases.leucine rich repeat XI MDP0000277933 nearly identical (1258) AT1G78920 | Symbols: AVP2, AVPL1 | AVP2 (ARABIDOPSIS VACUOLAR H+-PYROPHOSPHATASE 2); hydrogen-translocating pyrophosphatase | chr1:29672340-29676761 FORWARDmoderately similar to ( 402) AVP_HORVU Pyrophosphate-energized vacuolar membrane proton pump (EC 3.6.1.1) (Pyrophosphate-energized inorganic pyrophosphatase) (H(+)-PPase) - Hordeum vulgare (Barley)nearly identical (1216) loc_os02g33490 12002.m08442 protein pyrophosphate-energized membrane proton pump 3, putative, expressed no original description T

29.5.3 protein.degradation.cysteine protease MDP0000179185 moderately similar to ( 209) AT3G22260 | Symbols: | OTU-like cysteine protease family protein | chr3:7871489-7873393 FORWARDweakly similar to ( 185) loc_os02g07210 12002.m100113 protein cysteine-type peptidase, putative, expressed no original description T

29.2.1.2.2.17 protein.synthesis.ribosomal protein.eukaryotic.60S subunit.L17 MDP0000227849 moderately similar to ( 293) AT1G67430 | Symbols: | 60S ribosomal protein L17 (RPL17B) | chr1:25262209-25263627 FORWARDmoderately similar to ( 288) RL172_HORVU 60S ribosomal protein L17-2 - Hordeum vulgare (Barley)moderately similar to ( 285) loc_os09g08430 12009.m04110 protein 60S ribosomal protein L17, putative, expressed no original description T

13.1.3.4 amino acid metabolism.synthesis.aspartate family.methionine MDP0000153762 nearly identical (1332) AT5G17920 | Symbols: ATCIMS | ATMS1; 5-methyltetrahydropteroyltriglutamate-homocysteine S-methyltransferase/ methionine synthase | chr5:5935771-5939195 FORWARDnearly identical (1333) METE_CATRO 5-methyltetrahydropteroyltriglutamate--homocysteine methyltransferase (EC 2.1.1.14) (Vitamin-B12-independent methionine synthase isozyme) (Cobalamin-independent methionine synthase isozyme) - Catharanthus roseus (Rosy periwinkle) (Madagnearly identical (1317) loc_os12g42876 12012.m073894 protein 5-methyltetrahydropteroyltriglutamate--homocysteine methyltransferase, putative, expressed no original description T

29.2.3 protein.synthesis.initiation MDP0000287358 highly similar to ( 814) AT4G20980 | Symbols: | eukaryotic translation initiation factor 3 subunit 7, putative / eIF-3 zeta, putative / eIF3d, putative | chr4:11216997-11218772 FORWARDhighly similar to ( 710) loc_os05g49150 12005.m08985 protein eukaryotic translation initiation factor 3 subunit 7, putative, expressed no original description T

29.5.11.4.2 protein.degradation.ubiquitin.E3.RING MDP0000282421 highly similar to ( 528) AT3G07360 | Symbols: PUB9, ATPUB9 | PUB9 (PLANT U-BOX 9) | chr3:2355636-2356613 FORWARDmoderately similar to ( 264) SPL11_ORYSA Spotted leaf protein 11 (Spotted leaf11) (Cell death-related protein SPL11) - Oryza sativa (Rice)moderately similar to ( 408) loc_os02g28720 12002.m08016 protein spotted leaf protein 11, putative, expressed no original description T

20.1 stress.biotic MDP0000185570 weakly similar to ( 196) AT1G78780 | Symbols: | pathogenesis-related family protein | chr1:29621447-29622431 REVERSEmoderately similar to ( 210) PRPX_HORVU Pathogen-related protein - Hordeum vulgare (Barley)moderately similar to ( 226) loc_os01g14590 12001.m150539 protein pathogen-related protein, putative, expressed no original description T

29.2.1.2.2.28 protein.synthesis.ribosomal protein.eukaryotic.60S subunit.L28 MDP0000557465 moderately similar to ( 216) AT2G19730 | Symbols: | 60S ribosomal protein L28 (RPL28A) | chr2:8511752-8512995 FORWARDmoderately similar to ( 215) loc_os01g51020 12001.m11283 protein 60S ribosomal protein L28, putative, expressed no original description T

35.2 not assigned.unknown MDP0000132692 moderately similar to ( 267) AT4G32480 | Symbols: | unknown protein | chr4:15676488-15677776 FORWARDmoderately similar to ( 209) loc_os01g54340 12001.m43190 protein plant-specific domain TIGR01615 family protein, expressed no original description T

30.3 signalling.calcium MDP0000606583 moderately similar to ( 224) AT2G15680 | Symbols: | calmodulin-related protein, putative | chr2:6831024-6831587 FORWARDvery weakly similar to (90.1) CALM_CHLRE Calmodulin (CaM) - Chlamydomonas reinhardtiiweakly similar to ( 118) loc_os05g13580 12005.m05773 protein calmodulin-related protein 2, touch-induced, putative, expressed no original description T

21.2.1 redox.ascorbate and glutathione.ascorbate MDP0000236168 weakly similar to ( 158) AT1G19570 | Symbols: DHAR1, ATDHAR1 | DHAR1 (dehydroascorbate reductase); glutathione dehydrogenase (ascorbate) | chr1:6773462-6774413 REVERSEweakly similar to ( 167) loc_os05g02530 12005.m04789 protein chloride intracellular channel 6, putative, expressed no original description T

29.4 protein.postranslational modification MDP0000282499 nearly identical (1009) AT4G19120 | Symbols: ERD3 | ERD3 (early-responsive to dehydration 3) | chr4:10460665-10463034 REVERSEmoderately similar to ( 226) CDC22_MEDSA Cell division control protein 2 homolog 2 (EC 2.7.11.22) - Medicago sativa (Alfalfa)highly similar to ( 539) loc_os06g01450 12006.m04884 protein methyltransferase, putative, expressed no original description T

29.3.1 protein.targeting.nucleus MDP0000211834 nearly identical (1381) AT5G53480 | Symbols: | importin beta-2, putative | chr5:21714016-21716709 FORWARDnearly identical (1328) loc_os12g38110 12012.m07597 protein importin beta-1 subunit, putative, expressed no original description T

35.2 not assigned.unknown MDP0000650702 moderately similar to ( 351) AT4G39840 | Symbols: | unknown protein | chr4:18485268-18486623 FORWARDweakly similar to ( 181) loc_os09g20540 12009.m05261 protein expressed protein no original description T

16.8.3.1 secondary metabolism.flavonoids.dihydroflavonols.dihydroflavonol 4-reductase MDP0000243196 moderately similar to ( 429) AT1G61720 | Symbols: BAN | BAN (BANYULS); oxidoreductase | chr1:22791326-22792757 REVERSEmoderately similar to ( 285) DFRA_VITVI Dihydroflavonol-4-reductase (EC 1.1.1.219) (DFR) (Dihydrokaempferol 4-reductase) - Vitis vinifera (Grape)moderately similar to ( 336) loc_os04g53850 12004.m35472 protein leucoanthocyanidin reductase, putative, expressed no original description T

28.2 DNA.repair MDP0000176889 highly similar to ( 508) AT5G04560 | Symbols: DME | DME (DEMETER); DNA N-glycosylase/ DNA-(apurinic or apyrimidinic site) lyase | chr5:1309786-1318091 FORWARDmoderately similar to ( 385) loc_os02g29230 12002.m08067 protein conserved hypothetical protein no original description T
27.4	RNA.RNA binding	MDP0000871411	 highly similar to ( 583) AT1G17370 | Symbols: UBP1B | UBP1B (oligouridylate binding protein 1B); mRNA 3'-UTR binding | chr1:5951842-5954825 REVERSEvery weakly similar to (77.8) ROC1_SPIOL 28 kDa ribonucleoprotein, chloroplast (28RNP) - Spinacia oleracea (Spinach)highly similar to ( 532) loc_os07g42380 12007.m08471 protein nucleolysin TIAR, putative, expressed  no original description 	T
29.5	protein.degradation	MDP0000254490	 weakly similar to ( 175) AT5G22860 | Symbols:  | serine carboxypeptidase S28 family protein | chr5:7639907-7642945 REVERSEweakly similar to ( 160) loc_os06g43930 12006.m32127 protein lysosomal Pro-X carboxypeptidase precursor, putative, expressed no original description 	T
22.1.6	polyamine metabolism.synthesis.spermidine synthase	MDP0000125216	 moderately similar to ( 478) AT5G19530 | Symbols: ACL5 | ACL5 (ACAULIS 5); spermine synthase/ thermospermine synthase | chr5:6589176-6591070 REVERSEweakly similar to ( 119) SPD1_PEA Spermidine synthase 1 (EC 2.5.1.16) (Putrescine aminopropyltransferase 1) (SPDSY 1) - Pisum sativum (Garden pea)moderately similar to ( 444) loc_os02g14190 12002.m06665 protein spermidine synthase, putative, expressed no original description 	T
30.2.99	signalling.receptor kinases.misc	MDP0000276990	 weakly similar to ( 121) AT1G66880 | Symbols:  | serine/threonine protein kinase family protein | chr1:24946928-24955438 FORWARDvery weakly similar to (78.6) CRI4_MAIZE Putative receptor protein kinase CRINKLY4 precursor (EC 2.7.11.1) - Zea mays (Maize)weakly similar to ( 124) loc_os02g02120 12002.m05562 protein OsWAK11 - OsWAK receptor-like protein kinase, expressed  no original description 	T
31.1	cell.organisation	MDP0000509076	 moderately similar to ( 260) AT3G01750 | Symbols:  | ankyrin repeat family protein | chr3:270615-272691 FORWARDmoderately similar to ( 289) loc_os05g23320 12005.m06627 protein expressed protein  no original description 	T
30.6	signalling.MAP kinases	MDP0000385801	 moderately similar to ( 369) AT5G19010 | Symbols: MPK16 | MPK16; MAP kinase | chr5:6345096-6347676 REVERSEmoderately similar to ( 348) MPK14_ORYSA Mitogen-activated protein kinase 14 (EC 2.7.11.24) (MAP kinase 14) - Oryza sativa (Rice)moderately similar to ( 348) loc_os05g05160 12005.m05043 protein extracellular signal-regulated kinase 1, putative, expressed  no original description 	T
29.1.5	protein.aa activation.isoleucine-tRNA ligase	MDP0000176251	 nearly identical (1747) AT4G10320 | Symbols:  | isoleucyl-tRNA synthetase, putative / isoleucine--tRNA ligase, putative | chr4:6397526-6404509 REVERSEnearly identical (1628) loc_os06g43760 12006.m08906 protein ATP binding protein, putative, expressed  no original description 	T
27.3.63	RNA.regulation of transcription.PHD finger transcription factor	MDP0000203957	 moderately similar to ( 474) AT1G05380 | Symbols:  | DNA binding | chr1:1577231-1582190 FORWARDmoderately similar to ( 211) RS18_CHLRE 40S ribosomal protein S18 - Chlamydomonas reinhardtiimoderately similar to ( 424) loc_os07g07690 12007.m29081 protein DNA binding protein, putative, expressed     TrbL PRK07735  no original description 	T
35.2	not assigned.unknown	MDP0000775552	 weakly similar to ( 169) AT2G47115 | Symbols:  | unknown protein | chr2:19345376-19346864 REVERSEweakly similar to ( 142) loc_os07g46430 12007.m08860 protein expressed protein  no original description 	T
34.99	transport.misc	MDP0000255048	 highly similar to ( 598) AT5G65380 | Symbols:  | ripening-responsive protein, putative | chr5:26123241-26126352 REVERSEhighly similar to ( 516) loc_os03g08900 12003.m06397 protein transparent testa 12 protein, putative, expressed  no original description 	T
33.99	development.unspecified	MDP0000621646	 weakly similar to ( 110) AT5G04410 | Symbols: NAC2, anac078 | NAC2; transcription factor | chr5:1243980-1246416 FORWARD  no original description 	T
29.4	protein.postranslational modification	MDP0000897373	 highly similar to ( 632) AT3G08720 | Symbols: ATPK19, ATPK2 | S6K2 (ARABIDOPSIS THALIANA SERINE/THREONINE PROTEIN KINASE 2); kinase/ protein kinase | chr3:2648625-2650407 REVERSEweakly similar to ( 151) CIPK1_ORYSA CIPK-like protein 1 (EC 2.7.11.1) (OsCK1) - Oryza sativa (Rice)highly similar to ( 608) loc_os03g21620 12003.m07566 protein serine/threonine-protein kinase AtPK19, putative, expressed  no original description 	T
26.12	misc.peroxidases	MDP0000509949	 moderately similar to ( 464) AT1G05260 | Symbols: RCI3, RCI3A | RCI3 (RARE COLD INDUCIBLE GENE 3); peroxidase | chr1:1529827-1531271 FORWARDmoderately similar to ( 318) PER1_ORYSA Peroxidase 1 precursor (EC 1.11.1.7) - Oryza sativa (Rice)moderately similar to ( 376) loc_os06g46799 12006.m09205 protein peroxidase 39 precursor, putative, expressed  no original description 	T
35.2	not assigned.unknown	MDP0000756425	 very weakly similar to (51.2) AT5G50335 | Symbols:  | unknown protein | chr5:20489391-20489615 REVERSE no original description 	T
29.4.1.57	protein.postranslational modification.kinase.receptor like cytoplasmatic kinase VII	MDP0000930295	 highly similar to ( 630) AT1G01540 | Symbols:  | protein kinase family protein | chr1:195980-198383 FORWARDmoderately similar to ( 239) PSKR_DAUCA Phytosulfokine receptor precursor (EC 2.7.11.1) (Phytosulfokine LRR receptor kinase) - Daucus carota (Carrot)highly similar to ( 547) loc_os03g03410 12003.m05874 protein BRASSINOSTEROID INSENSITIVE 1-associated receptor kinase 1 precursor, putative, expressed    no original description 	T
21.2	redox.ascorbate and glutathione	MDP0000896377	 moderately similar to ( 243) AT3G48890 | Symbols: ATMP2, ATMAPR3, MSBP2, MAPR3 | ATMP2; heme binding | chr3:18129669-18131353 FORWARDmoderately similar to ( 256) loc_os10g35870 12010.m06404 protein membrane steroid-binding protein 1, putative, expressed   no original description 	T
30.2.99	signalling.receptor kinases.misc	MDP0000412849	 moderately similar to ( 296) AT5G43980 | Symbols: PDLP1, PDLP1A | PDLP1 (PLASMODESMATA-LOCATED PROTEIN 1) | chr5:17694817-17696458 REVERSEweakly similar to ( 145) loc_os04g45460 12004.m09499 protein secretory protein-like, putative, expressed    no original description 	T
26.10	misc.cytochrome P450	MDP0000146894	 moderately similar to ( 494) AT1G74110 | Symbols: CYP78A10 | CYP78A10; electron carrier/ heme binding / iron ion binding / monooxygenase/ oxygen binding | chr1:27866667-27868368 REVERSEmoderately similar to ( 457) C78A3_SOYBN Cytochrome P450 78A3 (EC 1.14.-.-) - Glycine max (Soybean)moderately similar to ( 494) loc_os11g29720 12011.m06881 protein cytochrome P450 78A4, putative, expressed   PRK08026  no original description 	T
35.2	not assigned.unknown	MDP0000090454	 moderately similar to ( 484) AT2G37940 | Symbols:  | FUNCTIONS IN: molecular_function unknown; INVOLVED IN: biological_process unknown; EXPRESSED IN: 22 plant structures; EXPRESSED DURING: 13 growth stages; BEST Arabidopsis thaliana protein match is: phosphatidic acid phosphatase-related / PAP2-related (TAIR:AT3G54020.1); Has 316 Blast hits to 316 proteins in 75 species: Archae - 0; Bacteria - 0; Metazoa - 213; Fungi - 0; Plants - 48; Viruses - 0; Other Eukaryotes - 55 (source: NCBI BLink). | chr2:15877169-15879411 FORWARDmoderately similar to ( 467) loc_os01g63060 12001.m12424 protein expressed protein no original description 	T
1.1.1.2	PS.lightreaction.photosystem II.PSII polypeptide subunits	MDP0000132436	 very weakly similar to (71.6) AT1G51400 | Symbols:  | photosystem II 5 kD protein | chr1:19052172-19052492 REVERSEvery weakly similar to (61.6) PST2_GOSHI Photosystem II 5 kDa protein, chloroplast precursor (PSII-T) (Light-regulated unknown 11 kDa protein) - Gossypium hirsutum (Upland cotton) no original description 	T
12.2.2	N-metabolism.ammonia metabolism.glutamine synthetase	MDP0000139493	 weakly similar to ( 168) AT5G35630 | Symbols: GS2, GLN2, ATGSL1 | GS2 (GLUTAMINE SYNTHETASE 2); glutamate-ammonia ligase | chr5:13831220-13833239 FORWARDmoderately similar to ( 219) GLNA2_DAUCA Glutamine synthetase, chloroplast precursor (EC 6.3.1.2) (Glutamate--ammonia ligase) (GS2) - Daucus carota (Carrot)weakly similar to ( 149) loc_os04g56400 12004.m79000 protein glutamine synthetase, chloroplast precursor, putative, expressed  no original description 	T
29.2.1.2.2.5	protein.synthesis.ribosomal protein.eukaryotic.60S subunit.L5	MDP0000719216	 moderately similar to ( 471) AT5G39740 | Symbols: OLI7, RPL5B | 60S ribosomal protein L5 (RPL5B) | chr5:15903365-15905185 FORWARDmoderately similar to ( 469) RL5_CUCSA 60S ribosomal protein L5 - Cucumis sativus (Cucumber)moderately similar to ( 447) loc_os01g67126 12001.m43540 protein 60S ribosomal protein L5-2, putative, expressed  no original description 	T
35.1	not assigned.no ontology	MDP0000186977	 weakly similar to ( 115) AT1G01490 | Symbols:  | heavy-metal-associated domain-containing protein | chr1:180401-182066 REVERSEvery weakly similar to (95.9) loc_os04g57200 12004.m10605 protein metal ion binding protein, putative, expressed no original description 	T
35.2	not assigned.unknown	MDP0000798964	 moderately similar to ( 298) AT4G32480 | Symbols:  | unknown protein | chr4:15676488-15677776 FORWARDmoderately similar to ( 251) loc_os01g54340 12001.m43190 protein plant-specific domain TIGR01615 family protein, expressed no original description 	T
31.3	cell.cycle	MDP0000135392	 moderately similar to ( 326) AT4G34160 | Symbols: CYCD3;1, CYCD3 | CYCD3;1 (CYCLIN D3;1); cyclin-dependent protein kinase regulator/ protein binding | chr4:16357903-16359304 FORWARDvery weakly similar to (57.4) CCNB2_ORYSA G2/mitotic-specific cyclin-2 (B-like cyclin) (CycOs2) - Oryza sativa (Rice)weakly similar to ( 169) loc_os07g42860 12007.m08515 protein cyclin delta-2, putative, expressed   no original description 	T
33.99	development.unspecified	MDP0000700875	 weakly similar to ( 169) AT5G11460 | Symbols:  | senescence-associated protein-related | chr5:3657064-3658388 REVERSEweakly similar to ( 145) loc_os01g08520 12001.m07477 protein expressed protein  no original description 	T
34.99	transport.misc	MDP0000260152	 moderately similar to ( 255) AT1G71090 | Symbols:  | auxin efflux carrier family protein | chr1:26812551-26813924 FORWARDmoderately similar to ( 257) loc_os08g09190 12008.m05054 protein auxin Efflux Carrier family protein, expressed  no original description 	T
16.2	secondary metabolism.phenylpropanoids	MDP0000325800	 highly similar to ( 949) AT4G24520 | Symbols: ATR1, AR1 | ATR1 (ARABIDOPSIS P450 REDUCTASE 1); NADPH-hemoprotein reductase | chr4:12663065-12667066 REVERSEnearly identical (1050) NCPR_PHAAU NADPH--cytochrome P450 reductase (EC 1.6.2.4) (CPR) (P450R) - Phaseolus aureus (Mung bean) (Vigna radiata)highly similar to ( 887) loc_os04g55960 12004.m10487 protein NADPH--cytochrome P450 reductase, putative, expressed  MSP1_C  no original description 	T
27.3.14	RNA.regulation of transcription.CCAAT box binding factor family, HAP2	MDP0000223651	 weakly similar to ( 141) AT1G72830 | Symbols: HAP2C, ATHAP2C, NF-YA3 | NF-YA3 (NUCLEAR FACTOR Y, SUBUNIT A3); transcription factor | chr1:27405699-27407088 REVERSEweakly similar to ( 140) loc_os03g07880 12003.m35594 protein nuclear transcription factor Y subunit A-3, putative, expressed no original description 	T
27.3.3	RNA.regulation of transcription.AP2/EREBP, APETALA2/Ethylene-responsive element binding protein family	MDP0000258562	 weakly similar to ( 151) AT2G23340 | Symbols:  | AP2 domain-containing transcription factor, putative | chr2:9938186-9938716 FORWARDvery weakly similar to (80.1) ERF4_TOBAC Ethylene-responsive transcription factor 4 (Ethylene-responsive element-binding factor 4 homolog) (EREBP-3) (NtERF3) - Nicotiana tabacum (Common tobacco)weakly similar to ( 142) loc_os04g55520 12004.m10443 protein dehydration responsive element binding protein, putative, expressed  no original description 	T
26.12	misc.peroxidases	MDP0000319048	 moderately similar to ( 303) AT5G05340 | Symbols:  | peroxidase, putative | chr5:1579142-1580819 REVERSEmoderately similar to ( 357) PER1_ARAHY Cationic peroxidase 1 precursor (EC 1.11.1.7) (PNPC1) - Arachis hypogaea (Peanut)moderately similar to ( 332) loc_os11g02100 12011.m04410 protein cationic peroxidase 1 precursor, putative, expressed no original description 	T
27.3.40	RNA.regulation of transcription.Aux/IAA family	MDP0000396750	 highly similar to ( 626) AT5G25510 | Symbols:  | serine/threonine protein phosphatase 2A (PP2A) regulatory subunit B', putative | chr5:8882728-8884325 REVERSEweakly similar to ( 121) IAA11_ORYSA Auxin-responsive protein IAA11 (Indoleacetic acid-induced protein 11) - Oryza sativa (Rice)highly similar to ( 593) loc_os05g48150 12005.m08886 protein transposon protein, putative, unclassified, expressed  no original description 	T
27.3.99	RNA.regulation of transcription.unclassified	MDP0000118897	 weakly similar to ( 174) AT5G16540 | Symbols: ZFN3 | ZFN3 (ZINC FINGER NUCLEASE 3); DNA binding / nuclease/ nucleic acid binding | chr5:5403437-5405034 FORWARDweakly similar to ( 192) ZFNL_PEA Zinc finger CCCH domain-containing protein ZFN-like - Pisum sativum (Garden pea)weakly similar to ( 170) loc_os11g28270 12011.m079994 protein zinc finger CCCH type domain-containing protein ZFN-like, putative, expressed no original description 	T
27.1.1	RNA.processing.splicing	MDP0000833543	 weakly similar to ( 188) AT2G37340 | Symbols: RSZ33, ATRSZ33 | RSZ33; nucleic acid binding / nucleotide binding / zinc ion binding | chr2:15670372-15672331 REVERSEmoderately similar to ( 201) loc_os05g02880 12005.m27529 protein splicing factor, arginine/serine-rich 7, putative, expressed  no original description 	T
11.1.10	lipid metabolism.FA synthesis and FA elongation.beta ketoacyl CoA synthase	MDP0000654110	 moderately similar to ( 282) AT2G26250 | Symbols: FDH, KCS10 | KCS10 (3-KETOACYL-COA SYNTHASE 10); acyltransferase/ catalytic/ transferase, transferring acyl groups other than amino-acyl groups | chr2:11170799-11173059 REVERSEmoderately similar to ( 272) loc_os03g08360 12003.m35563 protein fiddlehead-like protein, putative, expressed  no original description 	T
26.4.1	misc.beta 1,3 glucan hydrolases.glucan endo-1,3-beta-glucosidase	MDP0000319911	 highly similar to ( 597) AT3G13560 | Symbols:  | glycosyl hydrolase family 17 protein | chr3:4425484-4427284 REVERSEmoderately similar to ( 211) E13B_WHEAT Glucan endo-1,3-beta-glucosidase precursor (EC 3.2.1.39) ((1->3)-beta-glucan endohydrolase) ((1->3)-beta-glucanase) (Beta-1,3-endoglucanase) - Triticum aestivum (Wheat)moderately similar to ( 463) loc_os07g38930 12007.m08137 protein glucan endo-1,3-beta-glucosidase 4 precursor, putative, expressed   no original description 	T
35.1.9	not assigned.no ontology.BTB/POZ domain-containing protein	MDP0000181535	 highly similar to ( 870) AT3G61600 | Symbols: ATPOB1 | ATPOB1; protein binding | chr3:22795704-22798069 FORWARDhighly similar to ( 674) loc_os06g31100 12006.m07652 protein GAMYB-binding protein, putative, expressed  no original description 	T
27.3.99	RNA.regulation of transcription.unclassified	MDP0000244738	 highly similar to ( 723) AT4G36860 | Symbols:  | zinc ion binding | chr4:17358580-17361189 REVERSEhighly similar to ( 650) loc_os06g08400 12006.m32223 protein zinc ion binding protein, putative, expressed  no original description 	T
35.1.5	not assigned.no ontology.pentatricopeptide (PPR) repeat-containing protein	MDP0000273602	 highly similar to ( 748) AT1G71210 | Symbols:  | pentatricopeptide (PPR) repeat-containing protein | chr1:26838850-26841489 REVERSEweakly similar to ( 167) RF1_ORYSA Rf1 protein, mitochondrial precursor (PPR protein) (Fertility restorer) (Restorer for CMS) - Oryza sativa (Rice)highly similar to ( 624) loc_os02g03530 12002.m05703 protein expressed protein   no original description 	T
35.1	not assigned.no ontology	MDP0000255237	 moderately similar to ( 233) AT1G18720 | Symbols:  | unknown protein | chr1:6458020-6459658 REVERSEmoderately similar to ( 221) loc_os02g36870 12002.m33734 protein YGL010w-like protein, putative, expressed no original description 	T
35.2	not assigned.unknown	MDP0000198534	 moderately similar to ( 466) AT3G21295 | Symbols:  | FUNCTIONS IN: molecular_function unknown; INVOLVED IN: biological_process unknown; LOCATED IN: cellular_component unknown; CONTAINS InterPro DOMAIN/s: PWWP (InterPro:IPR000313); BEST Arabidopsis thaliana protein match is: unknown protein (TAIR:AT1G51745.1); Has 428 Blast hits to 314 proteins in 77 species: Archae - 0; Bacteria - 111; Metazoa - 87; Fungi - 55; Plants - 63; Viruses - 0; Other Eukaryotes - 112 (source: NCBI BLink). | chr3:7489635-7492296 FORWARDmoderately similar to ( 324) loc_os01g37790 12001.m10065 protein expressed protein   no original description 	T
27.3.50	RNA.regulation of transcription.General Transcription	MDP0000178691	 moderately similar to ( 289) AT3G13940 | Symbols:  | DNA binding / DNA-directed RNA polymerase | chr3:4600647-4602412 REVERSEweakly similar to ( 175) loc_os11g40090 12011.m07856 protein A49-like RNA polymerase I associated factor family protein, expressed  no original description 	T
29.5	protein.degradation	MDP0000860584	 moderately similar to ( 333) AT3G51800 | Symbols: ATG2 | ATG2; aminopeptidase/ metalloexopeptidase | chr3:19211261-19213568 REVERSEmoderately similar to ( 354) loc_os05g28280 12005.m07114 protein proliferation-associated protein 2G4, putative, expressed  no original description 	T
35.2	not assigned.unknown	MDP0000276541	 highly similar to ( 558) AT1G71110 | Symbols:  | unknown protein | chr1:26818244-26820852 FORWARDmoderately similar to ( 388) loc_os01g03110 12001.m06950 protein expressed protein  no original description 	T
11.1.1.1	lipid metabolism.FA synthesis and FA elongation.Acetyl CoA Carboxylation.homomeric Enzyme	MDP0000571465	 nearly identical (2293) AT1G36160 | Symbols: ACC1, AT-ACC1, EMB22, GK, PAS3 | ACC1 (ACETYL-COENZYME A CARBOXYLASE 1); acetyl-CoA carboxylase | chr1:13534196-13543773 FORWARDnearly identical (2100) loc_os10g21910 12010.m65459 protein acetyl-coenzyme A carboxylase ACC1A, putative, expressed  no original description 	T
29.5.11.20	protein.degradation.ubiquitin.proteasom	MDP0000141874	 nearly identical (1442) AT2G32730 | Symbols:  | 26S proteasome regulatory subunit, putative | chr2:13880189-13885464 FORWARDnearly identical (1286) loc_os08g12820 12008.m26623 protein 26S proteasome non-ATPase regulatory subunit 1, putative, expressed   no original description 	T
28.99	DNA.unspecified	MDP0000270931	 moderately similar to ( 278) AT4G32175 | Symbols:  | RNA binding | chr4:15535555-15537537 FORWARDmoderately similar to ( 288) loc_os01g66730 12001.m150798 protein exosome complex exonuclease RRP40, putative, expressed  no original description 	T
29.7	protein.glycosylation	MDP0000150532	 moderately similar to ( 414) AT3G57220 | Symbols:  | UDP-GlcNAc:dolichol phosphate N-acetylglucosamine-1-phosphate transferase, putative | chr3:21174516-21176904 FORWARDmoderately similar to ( 380) loc_os07g46640 12007.m08881 protein UDP-N-acetylglucosamine--dolichyl-phosphate N-acetylglucosaminephosphotransferase, putative, expressed  no original description 	T
31.3	cell.cycle	MDP0000175067	 weakly similar to ( 200) AT2G44740 | Symbols: CYCP4;1 | CYCP4;1 (cyclin p4;1); cyclin-dependent protein kinase | chr2:18442287-18443304 REVERSEweakly similar to ( 154) loc_os10g41430 12010.m06907 protein nuc-1 negative regulatory protein preg, putative, expressed  no original description 	T
30.5	signalling.G-proteins	MDP0000137580	 highly similar to ( 981) AT4G07410 | Symbols:  | transducin family protein / WD-40 repeat family protein | chr4:4201465-4204576 REVERSEhighly similar to ( 832) loc_os03g52470 12003.m10223 protein expressed protein  no original description 	T
20.2.1	stress.abiotic.heat	MDP0000168587	 highly similar to ( 820) AT3G52490 | Symbols:  | heat shock protein-related | chr3:19455850-19458721 REVERSEvery weakly similar to (78.2) HS101_ORYSA Heat shock protein 101 - Oryza sativa (Rice)highly similar to ( 602) loc_os04g33980 12004.m08492 protein transposon protein, putative, Mutator sub-class, expressed   TrbL  no original description 	T
35.2	not assigned.unknown	MDP0000158717	 moderately similar to ( 395) AT3G13000 | Symbols:  | transcription factor | chr3:4158214-4160818 REVERSEmoderately similar to ( 364) loc_os06g49060 12006.m09431 protein ternary complex factor MIP1, putative, expressed  no original description 	T
30.6	signalling.MAP kinases	MDP0000232111	 highly similar to ( 603) AT5G67130 | Symbols:  | phospholipase C/ phosphoric diester hydrolase | chr5:26791599-26793649 FORWARDhighly similar to ( 533) loc_os09g02729 12009.m059932 protein MAP3K-like protein kinase, putative, expressed   no original description 	T
29.5.11.4.2	protein.degradation.ubiquitin.E3.RING	MDP0000538280	 weakly similar to ( 121) AT5G22000 | Symbols: RHF2A | RHF2A (RING-H2 GROUP F2A); protein binding / zinc ion binding | chr5:7277436-7279553 FORWARDweakly similar to ( 133) loc_os11g36430 12011.m28683 protein zinc finger, RING-type, putative, expressed   no original description 	T
35.2	not assigned.unknown	MDP0000148366	 moderately similar to ( 318) AT3G04860 | Symbols:  | unknown protein | chr3:1339349-1340218 REVERSEmoderately similar to ( 331) loc_os03g59200 12003.m10820 protein expressed protein  no original description 	T
35.2	not assigned.unknown	MDP0000121763	 moderately similar to ( 364) AT4G12590 | Symbols:  | unknown protein | chr4:7451291-7452976 REVERSEmoderately similar to ( 331) loc_os09g32220 12009.m06311 protein protein pob, putative, expressed  no original description 	T
26.7	misc.oxidases - copper, flavone etc	MDP0000295428	 moderately similar to ( 496) AT5G16990 | Symbols:  | NADP-dependent oxidoreductase, putative | chr5:5581831-5583849 REVERSEmoderately similar to ( 440) loc_os12g12580 12012.m05231 protein NADP-dependent oxidoreductase P2, putative, expressed  no original description 	T
29.8	protein.assembly and cofactor ligation	MDP0000327390	 moderately similar to ( 276) AT5G17170 | Symbols: ENH1 | ENH1 (enhancer of sos3-1); metal ion binding / protein binding | chr5:5649335-5650835 FORWARDmoderately similar to ( 275) loc_os08g06530 12008.m04792 protein rubredoxin family protein, expressed  no original description 	T
29.4	protein.postranslational modification	MDP0000136806	 highly similar to ( 646) AT5G35410 | Symbols: SOS2, SnRK3.11, CIPK24 | SOS2 (SALT OVERLY SENSITIVE 2); kinase/ protein kinase | chr5:13634933-13638062 FORWARDmoderately similar to ( 497) CIPK1_ORYSA CIPK-like protein 1 (EC 2.7.11.1) (OsCK1) - Oryza sativa (Rice)highly similar to ( 617) loc_os06g40370 12006.m08573 protein CBL-interacting serine/threonine-protein kinase 24, putative, expressed  no original description 	T
21.5	redox.peroxiredoxin	MDP0000247659	 moderately similar to ( 265) AT3G26060 | Symbols: ATPRX Q | ATPRX Q; antioxidant/ peroxiredoxin | chr3:9524807-9526123 FORWARDvery weakly similar to (74.3) BAS1_HORVU 2-cys peroxiredoxin BAS1, chloroplast precursor (EC 1.11.1.15) (Thiol-specific antioxidant protein) (Fragment) - Hordeum vulgare (Barley)moderately similar to ( 265) loc_os06g09610 12006.m05679 protein peroxiredoxin bcp, putative, expressed  no original description 	T
27.3.26	RNA.regulation of transcription.MYB-related transcription factor family	MDP0000656112	 weakly similar to ( 150) AT1G53345 | Symbols:  | unknown protein | chr1:19902561-19903538 FORWARDweakly similar to ( 141) loc_os02g45950 12002.m09632 protein expressed protein  no original description 	T
29.1.7	protein.aa activation.alanine-tRNA ligase	MDP0000299239	 moderately similar to ( 300) AT3G16565 | Symbols:  | ATP binding / alanine-tRNA ligase/ ligase, forming aminoacyl-tRNA and related compounds / nucleic acid binding / nucleotide binding | chr3:5640531-5642675 REVERSEweakly similar to ( 130) RS21_ORYSA 40S ribosomal protein S21 - Oryza sativa (Rice)moderately similar to ( 275) loc_os01g03030 12001.m06942 protein alanyl-tRNA synthetase, putative, expressed  no original description 	T
35.2	not assigned.unknown	MDP0000249400	 moderately similar to ( 388) AT3G49720 | Symbols:  | unknown protein | chr3:18440192-18441655 REVERSEmoderately similar to ( 337) loc_os01g05070 12001.m42860 protein expressed protein  no original description 	T
33.99	development.unspecified	MDP0000022760	 moderately similar to ( 473) AT3G61790 | Symbols:  | seven in absentia (SINA) family protein | chr3:22871974-22873543 REVERSEweakly similar to ( 149) SINAL_DROME Probable E3 ubiquitin-protein ligase sina-like CG13030 (EC 6.3.2.-) - Drosophila melanogaster (Fruit fly)highly similar to ( 558) loc_os07g46560 12007.m08873 protein ubiquitin ligase SINAT5, putative, expressed   no original description 	T
27.3.6	RNA.regulation of transcription.bHLH,Basic Helix-Loop-Helix family	MDP0000290263	 moderately similar to ( 234) AT1G09530 | Symbols: PIF3, POC1, PAP3 | PIF3 (PHYTOCHROME INTERACTING FACTOR 3); DNA binding / protein binding / transcription factor/ transcription regulator | chr1:3077216-3079367 FORWARDvery weakly similar to (56.6) ARRS_MAIZE Anthocyanin regulatory R-S protein - Zea mays (Maize)weakly similar to ( 179) loc_os01g18290 12001.m42972 protein basic helix-loop-helix dimerisation region bHLH, putative, expressed   no original description 	T
35.2	not assigned.unknown	MDP0000318270	 weakly similar to ( 106) AT3G49210 | Symbols:  | unknown protein | chr3:18238216-18241161 REVERSEweakly similar to ( 108) loc_os01g48874 12001.m11075 protein expressed protein no original description 	T
4.2.14	glycolysis.plastid branch.pyruvate kinase (PK)	MDP0000171628	 highly similar to ( 579) AT3G22960 | Symbols: PKP1, PKP-ALPHA | PKP-ALPHA; pyruvate kinase | chr3:8139369-8141771 FORWARDhighly similar to ( 584) KPYA_RICCO Pyruvate kinase isozyme A, chloroplast precursor (EC 2.7.1.40) - Ricinus communis (Castor bean)highly similar to ( 653) loc_os03g46910 12003.m09706 protein pyruvate kinase isozyme A, chloroplast precursor, putative, expressed   no original description 	T
11.1.10	lipid metabolism.FA synthesis and FA elongation.beta ketoacyl CoA synthase	MDP0000134273	 highly similar to ( 830) AT2G26250 | Symbols: FDH, KCS10 | KCS10 (3-KETOACYL-COA SYNTHASE 10); acyltransferase/ catalytic/ transferase, transferring acyl groups other than amino-acyl groups | chr2:11170799-11173059 REVERSEhighly similar to ( 769) loc_os03g08360 12003.m35563 protein fiddlehead-like protein, putative, expressed   no original description 	T
30.11	signalling.light	MDP0000270526	 moderately similar to ( 361) AT3G49900 | Symbols:  | BTB/POZ domain-containing protein | chr3:18500635-18502614 REVERSEvery weakly similar to (65.5) NPH3_ORYSA Coleoptile phototropism protein 1 (Non-phototropic hypocotyl 3-like protein) (NPH3-like protein) - Oryza sativa (Rice)weakly similar to ( 189) loc_os07g39850 12007.m08227 protein expressed protein   no original description 	T
1.1.5.2	PS.lightreaction.other electron carrier (ox/red).ferredoxin	MDP0000155343	 weakly similar to ( 182) AT4G14890 | Symbols:  | ferredoxin family protein | chr4:8520887-8521351 FORWARDvery weakly similar to (91.3) FER2_RAPSA Ferredoxin, root R-B2 - Raphanus sativus (Radish)weakly similar to ( 159) loc_os03g45710 12003.m09594 protein ferredoxin, chloroplast precursor, putative, expressed  no original description 	T
29.4.1.57	protein.postranslational modification.kinase.receptor like cytoplasmatic kinase VII	MDP0000297560	 highly similar to ( 622) AT2G23200 | Symbols:  | protein kinase family protein | chr2:9879351-9881855 FORWARDmoderately similar to ( 255) NORK_MEDTR Nodulation receptor kinase precursor (EC 2.7.11.1) (Does not make infections protein 2) (Symbiosis receptor-like kinase) (MtSYMRK) - Medicago truncatula (Barrel medic)highly similar to ( 553) loc_os05g06990 12005.m05225 protein protein kinase, putative, expressed   no original description 	T
17.2.2	hormone metabolism.auxin.signal transduction	MDP0000301913	 moderately similar to ( 374) AT5G16530 | Symbols: PIN5 | PIN5 (PIN-FORMED 5); auxin:hydrogen symporter/ transporter | chr5:5400735-5402626 FORWARDmoderately similar to ( 308) PIN6_ORYSA Probable auxin efflux carrier component 6 (OsPIN6) - Oryza sativa (Rice)moderately similar to ( 308) loc_os01g69070 12001.m13008 protein auxin efflux carrier component 6, putative, expressed no original description 	T
35.2	not assigned.unknown	MDP0000582214	 moderately similar to ( 378) AT4G25030 | Symbols:  | unknown protein | chr4:12865336-12866638 FORWARDmoderately similar to ( 340) loc_os10g43060 12010.m22001 protein expressed protein  no original description 	T
17.4.1	hormone metabolism.cytokinin.synthesis-degradation	MDP0000232324	 moderately similar to ( 364) AT5G19040 | Symbols: ATIPT5, IPT5 | IPT5; ATP binding / tRNA isopentenyltransferase/ transferase, transferring alkyl or aryl (other than methyl) groups | chr5:6362389-6363381 REVERSEmoderately similar to ( 278) loc_os03g59570 12003.m10854 protein cytokinin synthase, putative, expressed  no original description 	T
30.5	signalling.G-proteins	MDP0000306514	 moderately similar to ( 363) AT1G06400 | Symbols: ARA2, ATRABA1A, ATRAB11E, ARA-2 | ARA-2; GTP binding | chr1:1951089-1952686 REVERSEmoderately similar to ( 387) RB11D_LOTJA Ras-related protein Rab11D - Lotus japonicusmoderately similar to ( 352) loc_os06g35814 12006.m08118 protein ras-related protein RIC2, putative, expressed  no original description 	T
31.1	cell.organisation	MDP0000568668	 moderately similar to ( 357) AT5G60210 | Symbols:  | LOCATED IN: plasma membrane; EXPRESSED IN: 22 plant structures; EXPRESSED DURING: 12 growth stages; BEST Arabidopsis thaliana protein match is: myosin heavy chain-related (TAIR:AT2G37080.1); Has 77946 Blast hits to 39215 proteins in 1793 species: Archae - 845; Bacteria - 8877; Metazoa - 39997; Fungi - 6049; Plants - 2988; Viruses - 248; Other Eukaryotes - 18942 (source: NCBI BLink). | chr5:24243529-24245889 REVERSEvery weakly similar to (55.1) MFP1_TOBAC MAR-binding filament-like protein 1-1 - Nicotiana tabacum (Common tobacco)moderately similar to ( 266) loc_os05g03120 12005.m27530 protein myosin heavy chain-like protein, putative, expressed   no original description 	T
35.2	not assigned.unknown	MDP0000300910	 moderately similar to ( 235) AT4G31980 | Symbols:  | unknown protein | chr4:15464905-15469204 FORWARDweakly similar to ( 174) loc_os01g21710 12001.m08655 protein conserved hypothetical protein  no original description 	T
20.2.3	stress.abiotic.drought/salt	MDP0000785636	 highly similar to ( 910) AT1G77260 | Symbols:  | dehydration-responsive protein-related | chr1:29023961-29026699 REVERSEhighly similar to ( 809) loc_os01g62800 12001.m12400 protein ankyrin-like protein, putative, expressed    no original description 	T
35.2	not assigned.unknown	MDP0000145329	 moderately similar to ( 203) AT4G22310 | Symbols:  | unknown protein | chr4:11791443-11792638 FORWARDmoderately similar to ( 201) loc_os07g26700 12007.m06947 protein brain protein 44, putative, expressed no original description 	T
29.5.11.4.2	protein.degradation.ubiquitin.E3.RING	MDP0000291345	 weakly similar to ( 112) AT3G46510 | Symbols: PUB13, ATPUB13 | PUB13 (PLANT U-BOX 13); ubiquitin-protein ligase | chr3:17124106-17126539 REVERSEvery weakly similar to (55.5) SPL11_ORYSA Spotted leaf protein 11 (Spotted leaf11) (Cell death-related protein SPL11) - Oryza sativa (Rice)very weakly similar to (83.2) loc_os03g16824 12003.m07115 protein spotted leaf protein 11, putative, expressed COG3889  no original description 	T
21.99	redox.misc	MDP0000223944	 very weakly similar to (81.6) AT1G31170 | Symbols: ATSRX, SRX | SRX (SULFIREDOXIN); DNA binding / oxidoreductase, acting on sulfur group of donors | chr1:11133521-11134228 FORWARDvery weakly similar to (82.8) loc_os06g07760 12006.m05498 protein sulfiredoxin-1, putative, expressed  no original description 	T
33.99	development.unspecified	MDP0000784909	 moderately similar to ( 403) AT4G00150 | Symbols:  | scarecrow-like transcription factor 6 (SCL6) | chr4:57429-59105 REVERSEweakly similar to ( 126) GAIP_CUCMA DELLA protein GAIP (Gibberellic acid-insensitive phloem protein) (GAIP) (CmGAIP) - Cucurbita maxima (Pumpkin) (Winter squash)moderately similar to ( 330) loc_os04g46860 12004.m09634 protein scarecrow-like 6, putative, expressed   no original description 	T
35.2	not assigned.unknown	MDP0000322057	 moderately similar to ( 367) AT1G28760 | Symbols:  | unknown protein | chr1:10103158-10104588 FORWARDmoderately similar to ( 357) loc_os03g45180 12003.m35338 protein expressed protein   no original description 	T
11.2.3	lipid metabolism.FA desaturation.omega 3 desaturase	MDP0000127630	 highly similar to ( 691) AT5G05580 | Symbols: FAD8 | FAD8 (FATTY ACID DESATURASE 8); omega-3 fatty acid desaturase | chr5:1664331-1666345 FORWARDhighly similar to ( 706) FAD3C_RICCO Omega-3 fatty acid desaturase, chloroplast precursor (EC 1.14.19.-) - Ricinus communis (Castor bean)highly similar to ( 632) loc_os03g18070 12003.m07232 protein omega-3 fatty acid desaturase, chloroplast precursor, putative, expressed   no original description 	T
29.2.1.2.1.9	protein.synthesis.ribosomal protein.eukaryotic.40S subunit.S9	MDP0000681199	 moderately similar to ( 335) AT5G39850 | Symbols:  | 40S ribosomal protein S9 (RPS9C) | chr5:15950053-15951171 FORWARDweakly similar to ( 114) RS9_TOBAC 40S ribosomal protein S9 (S4) (Fragment) - Nicotiana tabacum (Common tobacco)moderately similar to ( 325) loc_os11g38959 12011.m80083 protein 40S ribosomal protein S9, putative, expressed  no original description 	T
35.2	not assigned.unknown	MDP0000365908	 moderately similar to ( 215) AT4G33625 | Symbols:  | unknown protein | chr4:16153114-16154543 REVERSEweakly similar to ( 199) loc_os07g02710 12007.m04743 protein expressed protein  no original description 	T
27.1.19	RNA.processing.ribonucleases	MDP0000304293	 moderately similar to ( 429) AT2G32070 | Symbols:  | CCR4-NOT transcription complex protein, putative | chr2:13640829-13641656 REVERSEmoderately similar to ( 390) loc_os02g55300 12002.m10556 protein CCR4-NOT transcription complex subunit 7, putative, expressed no original description 	T
10.8.1	cell wall.pectin*esterases.PME	MDP0000161463	 very weakly similar to (96.7) AT2G43050 | Symbols: ATPMEPCRD | ATPMEPCRD; enzyme inhibitor/ pectinesterase | chr2:17902508-17904174 FORWARDvery weakly similar to (72.4) PME_PRUPE Pectinesterase PPE8B precursor (EC 3.1.1.11) (Pectin methylesterase) (PE) - Prunus persica (Peach)very weakly similar to (78.2) loc_os06g09340 12006.m05653 protein pectinesterase U1 precursor, putative, expressed  MSP1_C  no original description 	T
31.1	cell.organisation	MDP0000657053	 highly similar to ( 832) AT4G14960 | Symbols: TUA6 | TUA6; structural constituent of cytoskeleton | chr4:8548769-8550319 REVERSEhighly similar to ( 838) TBA1_PEA Tubulin alpha-1 chain - Pisum sativum (Garden pea)highly similar to ( 827) loc_os11g14220 12011.m28666 protein tubulin alpha-3 chain, putative, expressed   no original description 	T
35.2	not assigned.unknown	MDP0000575203	 moderately similar to ( 316) AT4G39840 | Symbols:  | unknown protein | chr4:18485268-18486623 FORWARDweakly similar to ( 176) loc_os09g20540 12009.m05261 protein expressed protein   no original description 	T
30.2.11	signalling.receptor kinases.leucine rich repeat XI	MDP0000231635	 highly similar to ( 835) AT5G14210 | Symbols:  | leucine-rich repeat transmembrane protein kinase, putative | chr5:4578503-4581374 REVERSEweakly similar to ( 128) NORK_MEDTR Nodulation receptor kinase precursor (EC 2.7.11.1) (Does not make infections protein 2) (Symbiosis receptor-like kinase) (MtSYMRK) - Medicago truncatula (Barrel medic)highly similar to ( 514) loc_os11g20360 12011.m28791 protein ATP binding protein, putative, expressed  no original description 	T
29.5.11.4.3.2	protein.degradation.ubiquitin.E3.SCF.FBOX	MDP0000843303	 moderately similar to ( 214) AT2G44130 | Symbols:  | kelch repeat-containing F-box family protein | chr2:18253731-18254960 FORWARDweakly similar to ( 200) loc_os11g14140 12011.m05551 protein protein kinase Kelch repeat:Kelch, putative, expressed    no original description 	T
29.2.1.2.1.15	protein.synthesis.ribosomal protein.eukaryotic.40S subunit.S15	MDP0000281535	 moderately similar to ( 362) AT1G04280 | Symbols:  | unknown protein | chr1:1143643-1146541 REVERSEmoderately similar to ( 277) RS15_ORYSA 40S ribosomal protein S15 - Oryza sativa (Rice)moderately similar to ( 361) loc_os05g43300 12005.m27906 protein expressed protein  no original description 	T
29.3.4.3	protein.targeting.secretory pathway.vacuole	MDP0000191311	 nearly identical (1039) AT2G14740 | Symbols: ATVSR3 | ATVSR3 (ARABIDOPSIS THALIANA VACULOLAR SORTING RECEPTOR 3); calcium ion binding | chr2:6308895-6312303 FORWARDnearly identical (1030) VSR1_PEA Vacuolar sorting receptor 1 precursor (BP-80) (80 kDa proaleurein-binding protein) - Pisum sativum (Garden pea)highly similar to ( 942) loc_os10g20630 12010.m05084 protein vacuolar sorting receptor 3 precursor, putative, expressed no original description 	T
29.4	protein.postranslational modification	MDP0000326332	 highly similar to ( 608) AT1G10430 | Symbols: PP2A-2 | PP2A-2; protein serine/threonine phosphatase | chr1:3428705-3430437 REVERSEhighly similar to ( 615) PP2A_HEVBR Serine/threonine-protein phosphatase PP2A catalytic subunit (EC 3.1.3.16) - Hevea brasiliensis (Para rubber tree)highly similar to ( 600) loc_os06g37660 12006.m08301 protein OsPP2Ac-1 - Phosphatase 2A isoform 1 belonging to family 1, expressed  no original description 	T
34.9	transport.metabolite transporters at the mitochondrial membrane	MDP0000796883	 highly similar to ( 620) AT3G08580 | Symbols: AAC1 | AAC1 (ADP/ATP CARRIER 1); ATP:ADP antiporter/ binding | chr3:2605706-2607030 REVERSEhighly similar to ( 638) ADT1_GOSHI ADP,ATP carrier protein 1, mitochondrial precursor (ADP/ATP translocase 1) (Adenine nucleotide translocator 1) (ANT 1) - Gossypium hirsutum (Upland cotton)highly similar to ( 570) loc_os02g48720 12002.m33839 protein ADP,ATP carrier protein, mitochondrial precursor, putative, expressed no original description 	T
1.1.1.2	PS.lightreaction.photosystem II.PSII polypeptide subunits	MDP0000174182	 very weakly similar to (78.2) AT3G01440 | Symbols:  | oxygen evolving enhancer 3 (PsbQ) family protein | chr3:168478-169407 FORWARDvery weakly similar to (70.9) PSBQ_SPIOL Oxygen-evolving enhancer protein 3, chloroplast precursor (OEE3) (16 kDa subunit of oxygen evolving system of photosystem II) (OEC 16 kDa subunit) - Spinacia oleracea (Spinach)very weakly similar to (97.4) loc_os04g44190 12004.m78971 protein expressed protein   no original description 	T
20.1.7	stress.biotic.PR-proteins	MDP0000291035	 highly similar to ( 535) AT5G66900 | Symbols:  | disease resistance protein (CC-NBS-LRR class), putative | chr5:26714931-26717757 REVERSEmoderately similar to ( 394) loc_os12g39620 12012.m073872 protein disease resistance protein, putative, expressed  no original description 	T
29.3.4.99	protein.targeting.secretory pathway.unspecified	MDP0000316688	 moderately similar to ( 312) AT1G01630 | Symbols:  | SEC14 cytosolic factor, putative / phosphoglyceride transfer protein, putative | chr1:229206-230675 FORWARDmoderately similar to ( 227) loc_os03g63370 12003.m11204 protein polyphosphoinositide binding protein Ssh2p, putative, expressed no original description 	T
26.10	misc.cytochrome P450	MDP0000298697	 moderately similar to ( 459) AT3G48280 | Symbols: CYP71A25 | CYP71A25; electron carrier/ heme binding / iron ion binding / monooxygenase/ oxygen binding | chr3:17879594-17881164 FORWARDmoderately similar to ( 454) C71A4_SOLME Cytochrome P450 71A4 (EC 1.14.-.-) (CYPLXXIA4) (P-450EG2) - Solanum melongena (Eggplant) (Aubergine)moderately similar to ( 358) loc_os01g12760 12001.m07889 protein cytochrome P450 71A6, putative, expressed  no original description 	T
29.5.11.4.2	protein.degradation.ubiquitin.E3.RING	MDP0000256623	 very weakly similar to (88.6) AT5G20885 | Symbols:  | zinc finger (C3HC4-type RING finger) family protein | chr5:7084133-7084663 REVERSEvery weakly similar to (54.3) EL5_ORYSA E3 ubiquitin-protein ligase EL5 (EC 6.3.2.-) - Oryza sativa (Rice)very weakly similar to (85.1) loc_os08g06090 12008.m04748 protein RING zinc finger protein-like, putative, expressed no original description 	T
34.8	transport.metabolite transporters at the envelope membrane	MDP0000462809	 highly similar to ( 522) AT4G32390 | Symbols:  | phosphate translocator-related | chr4:15636550-15637602 FORWARDvery weakly similar to (61.2) TPT_SPIOL Triose phosphate/phosphate translocator, chloroplast precursor (cTPT) (p36) (E29) - Spinacia oleracea (Spinach)moderately similar to ( 456) loc_os05g41480 12005.m27616 protein plastidic phosphate translocator-like protein1, putative, expressed   no original description 	T
31.3	cell.cycle	MDP0000258414	 very weakly similar to (77.8) AT1G49620 | Symbols: KRP7, ICN6, ICK5 | ICK5; cyclin binding / cyclin-dependent protein kinase inhibitor | chr1:18365692-18366559 REVERSEvery weakly similar to (59.7) loc_os11g40030 12011.m07850 protein cyclin-dependent kinase inhibitor 2, putative, expressed no original description 	T
35.1	not assigned.no ontology	MDP0000119794	 moderately similar to ( 206) AT1G10600 | Symbols:  | FUNCTIONS IN: molecular_function unknown; INVOLVED IN: ubiquitin-dependent protein catabolic process; LOCATED IN: chloroplast; EXPRESSED IN: cultured cell; CONTAINS InterPro DOMAIN/s: Mov34/MPN/PAD-1 (InterPro:IPR000555); BEST Arabidopsis thaliana protein match is: unknown protein (TAIR:AT4G16144.1); Has 771 Blast hits to 770 proteins in 170 species: Archae - 0; Bacteria - 0; Metazoa - 366; Fungi - 201; Plants - 115; Viruses - 0; Other Eukaryotes - 89 (source: NCBI BLink). | chr1:3503765-3505190 FORWARDweakly similar to ( 172) loc_os01g47262 12001.m10921 protein STAM-binding protein, putative, expressed  no original description 	T
29.4	protein.postranslational modification	MDP0000527695	 highly similar to ( 560) AT3G05580 | Symbols:  | serine/threonine protein phosphatase, putative | chr3:1618216-1619850 REVERSEhighly similar to ( 566) PP1_PHAVU Serine/threonine-protein phosphatase PP1 (EC 3.1.3.16) - Phaseolus vulgaris (Kidney bean) (French bean)highly similar to ( 506) loc_os01g24750 12001.m08941 protein serine/threonine-protein phosphatase PP1, putative, expressed  no original description 	T
15.2	metal handling.binding, chelation and storage	MDP0000252706	 moderately similar to ( 288) AT2G40300 | Symbols: ATFER4 | ATFER4 (ferritin 4); binding / ferric iron binding / oxidoreductase/ transition metal ion binding | chr2:16831501-16833214 REVERSEmoderately similar to ( 317) FRI3_SOYBN Ferritin-3, chloroplast precursor (EC 1.16.3.1) (SFerH-3) - Glycine max (Soybean)moderately similar to ( 274) loc_os12g01530 12012.m26727 protein ferritin-1, chloroplast precursor, putative, expressed  no original description 	T
18.7	Co-factor and vitamine metabolism.iron-sulphur clusters	MDP0000120058	 moderately similar to ( 235) AT4G22220 | Symbols: ISU1, ATISU1 | ISU1; structural molecule | chr4:11759444-11760881 REVERSEmoderately similar to ( 241) loc_os01g47340 12001.m42683 protein nifU-like N-terminal domain-containing protein, mitochondrial precursor, putative, expressed  no original description 	T
35.1	not assigned.no ontology	MDP0000312665	 very weakly similar to (67.0) AT3G11910 | Symbols: UBP13 | UBP13 (UBIQUITIN-SPECIFIC PROTEASE 13); ubiquitin thiolesterase/ ubiquitin-specific protease | chr3:3761758-3770290 REVERSEvery weakly similar to (79.3) loc_os11g36470 12011.m07499 protein cysteine-type endopeptidase/ double-stranded DNA binding protein, putative, expressed   no original description 	T
35.1	not assigned.no ontology	MDP0000137043	 moderately similar to ( 492) AT3G18140 | Symbols:  | transducin family protein / WD-40 repeat family protein | chr3:6213225-6214567 REVERSEvery weakly similar to (79.0) GBLP_CHLRE Guanine nucleotide-binding protein subunit beta-like protein - Chlamydomonas reinhardtiihighly similar to ( 505) loc_os03g47780 12003.m101438 protein WD-repeat protein pop3, putative, expressed  no original description 	T
27.3.3	RNA.regulation of transcription.AP2/EREBP, APETALA2/Ethylene-responsive element binding protein family	MDP0000848905	 weakly similar to ( 128) AT3G16770 | Symbols: RAP2.3, ATEBP, ERF72 | ATEBP (ETHYLENE-RESPONSIVE ELEMENT BINDING PROTEIN); DNA binding / protein binding / transcription activator/ transcription factor | chr3:5705784-5706768 FORWARDvery weakly similar to (99.4) ERF1_ORYSA Ethylene-responsive transcription factor 1 (Ethylene-responsive element-binding factor 1) (EREBP-1) (OsEREBP1) - Oryza sativa (Rice)weakly similar to ( 121) loc_os07g47790 12007.m29326 protein ERF-like protein, putative, expressed  no original description 	T
10.6.1	cell wall.degradation.cellulases and beta -1,4-glucanases	MDP0000740981	 highly similar to ( 714) AT4G39010 | Symbols: AtGH9B18 | AtGH9B18 (Arabidopsis thaliana glycosyl hydrolase 9B18); catalytic/ hydrolase, hydrolyzing O-glycosyl compounds | chr4:18176162-18179102 REVERSEhighly similar to ( 674) GUN23_ORYSA Endoglucanase 23 precursor (EC 3.2.1.4) (Endo-1,4-beta glucanase 23) (OsGLU12) - Oryza sativa (Rice)highly similar to ( 674) loc_os09g36060 12009.m06556 protein endoglucanase 1 precursor, putative, expressed   no original description 	T
26.3.4	misc.gluco-, galacto- and mannosidases.endoglucanase	MDP0000740981	 highly similar to ( 714) AT4G39010 | Symbols: AtGH9B18 | AtGH9B18 (Arabidopsis thaliana glycosyl hydrolase 9B18); catalytic/ hydrolase, hydrolyzing O-glycosyl compounds | chr4:18176162-18179102 REVERSEhighly similar to ( 674) GUN23_ORYSA Endoglucanase 23 precursor (EC 3.2.1.4) (Endo-1,4-beta glucanase 23) (OsGLU12) - Oryza sativa (Rice)highly similar to ( 674) loc_os09g36060 12009.m06556 protein endoglucanase 1 precursor, putative, expressed   no original description 	T
29.5.9	protein.degradation.AAA type	MDP0000647571	 moderately similar to ( 442) AT2G34560 | Symbols:  | katanin, putative | chr2:14560266-14562695 FORWARDweakly similar to ( 155) CDC48_CAPAN Cell division cycle protein 48 homolog - Capsicum annuum (Bell pepper)moderately similar to ( 451) loc_os01g55260 12001.m11685 protein katanin p60 ATPase-containing subunit A-like 1, putative, expressed no original description 	T
29.2.3	protein.synthesis.initiation	MDP0000881736	 highly similar to ( 802) AT4G20980 | Symbols:  | eukaryotic translation initiation factor 3 subunit 7, putative / eIF-3 zeta, putative / eIF3d, putative | chr4:11216997-11218772 FORWARDhighly similar to ( 701) loc_os05g49150 12005.m08985 protein eukaryotic translation initiation factor 3 subunit 7, putative, expressed   no original description 	T
29.2.1.2.2.510	protein.synthesis.ribosomal protein.eukaryotic.60S subunit.L10A	MDP0000314549	 moderately similar to ( 295) AT2G27530 | Symbols: PGY1 | PGY1 (PIGGYBACK1); RNA binding / structural constituent of ribosome | chr2:11763443-11764570 REVERSEmoderately similar to ( 209) RL10A_CHLRE 60S ribosomal protein L10a - Chlamydomonas reinhardtiimoderately similar to ( 303) loc_os01g64090 12001.m43287 protein 60S ribosomal protein L10a-1, putative, expressed  no original description 	T
20.2.1	stress.abiotic.heat	MDP0000272952	 weakly similar to ( 126) AT3G09520 | Symbols: ATEXO70H4 | ATEXO70H4 (exocyst subunit EXO70 family protein H4); protein binding | chr3:2923743-2925629 FORWARDweakly similar to ( 127) loc_os02g52270 12002.m10258 protein chaperone protein dnaJ, putative, expressed  no original description 	T
26.28	misc.GDSL-motif lipase	MDP0000928830	 highly similar to ( 514) AT3G16370 | Symbols:  | GDSL-motif lipase/hydrolase family protein | chr3:5556928-5558351 FORWARDweakly similar to ( 195) APG_BRANA Anter-specific proline-rich protein APG (Protein CEX) (Fragment) - Brassica napus (Rape)moderately similar to ( 490) loc_os06g05550 12006.m05282 protein anther-specific proline-rich protein APG precursor, putative, expressed  no original description 	T
23.1.1.3	nucleotide metabolism.synthesis.pyrimidine.dihydroorotase	MDP0000144714	 highly similar to ( 584) AT4G22930 | Symbols: PYR4, DHOASE | PYR4 (PYRIMIDIN 4); dihydroorotase/ hydrolase/ hydrolase, acting on carbon-nitrogen (but not peptide) bonds, in cyclic amides | chr4:12019315-12021200 FORWARDhighly similar to ( 565) loc_os01g54370 12001.m11601 protein dihydroorotase, mitochondrial precursor, putative, expressed  no original description 	T
20.2.99	stress.abiotic.unspecified	MDP0000922599	 weakly similar to ( 115) AT2G47710 | Symbols:  | universal stress protein (USP) family protein | chr2:19555045-19555956 REVERSEweakly similar to ( 119) loc_os12g36640 12012.m07450 protein fiber protein Fb19, putative, expressed no original description 	T
28.1	DNA.synthesis/chromatin structure	MDP0000315900	 nearly identical (1343) AT1G44900 | Symbols: MCM2 | ATP binding / DNA binding / DNA-dependent ATPase | chr1:16970291-16974457 FORWARDmoderately similar to ( 327) MCM3_MAIZE DNA replication licensing factor MCM3 homolog (Replication origin activator) (ROA protein) (Fragment) - Zea mays (Maize)nearly identical (1397) loc_os11g29380 12011.m06848 protein DNA replication licensing factor Mcm2, putative, expressed  no original description 	T
27.3.7	RNA.regulation of transcription.C2C2(Zn) CO-like, Constans-like zinc finger family	MDP0000815065	 weakly similar to ( 136) AT5G24470 | Symbols: APRR5, PRR5 | APRR5 (ARABIDOPSIS PSEUDO-RESPONSE REGULATOR 5); transcription regulator/ two-component response regulator | chr5:8356204-8358873 REVERSEweakly similar to ( 110) PRR95_ORYSA Two-component response regulator-like PRR95 (Pseudo-response regulator 95) (OsPRR95) - Oryza sativa (Rice)weakly similar to ( 110) loc_os09g36220 12009.m06571 protein two-component response regulator-like PRR95, putative, expressed  no original description 	T
20.1	stress.biotic	MDP0000145677	 very weakly similar to (55.1) AT3G14470 | Symbols:  | disease resistance protein (NBS-LRR class), putative | chr3:4857940-4861104 FORWARDvery weakly similar to (62.0) loc_os01g33684 12001.m150608 protein disease resistance RPP13-like protein 1, putative, expressed no original description 	T
30.2.11	signalling.receptor kinases.leucine rich repeat XI	MDP0000703915	 highly similar to ( 634) AT2G02220 | Symbols: ATPSKR1, PSKR1 | PSKR1 (PHYTOSULFOKIN RECEPTOR 1); ATP binding / peptide receptor/ protein serine/threonine kinase | chr2:584098-587124 REVERSEhighly similar to ( 599) PSKR_DAUCA Phytosulfokine receptor precursor (EC 2.7.11.1) (Phytosulfokine LRR receptor kinase) - Daucus carota (Carrot)moderately similar to ( 449) loc_os02g41890 12002.m09226 protein phytosulfokine receptor precursor, putative, expressed   no original description 	T
27.3.25	RNA.regulation of transcription.MYB domain transcription factor family	MDP0000274301	 moderately similar to ( 308) AT3G01140 | Symbols: MYB106, AtMYB106 | MYB106 (myb domain protein 106); DNA binding / transcription factor | chr3:46619-48143 REVERSEmoderately similar to ( 206) ODO1_PETHY ODORANT1 protein (MYB-like protein ODO1) - Petunia hybrida (Petunia)moderately similar to ( 303) loc_os08g33660 12008.m07356 protein MYB1, putative, expressed   no original description 	T
33.99	development.unspecified	MDP0000259556	 weakly similar to ( 116) AT1G05835 | Symbols:  | unknown protein | chr1:1762248-1762702 FORWARDvery weakly similar to (61.6) NAC67_ORYSA NAC domain-containing protein 67 (ONAC067) - Oryza sativa (Rice)very weakly similar to (68.6) loc_os07g09830 12007.m05438 protein no apical meristem protein   no original description 	T
35.1	not assigned.no ontology	MDP0000762227	 highly similar to ( 530) AT3G01990 | Symbols: ACR6 | ACR6; amino acid binding | chr3:330256-332066 FORWARDmoderately similar to ( 454) loc_os03g29980 12003.m34884 protein ACR4, putative, expressed no original description 	T
29.6	protein.folding	MDP0000576908	 moderately similar to ( 296) AT1G01230 | Symbols:  | ORMDL family protein | chr1:97620-99046 FORWARDmoderately similar to ( 295) loc_os02g45180 12002.m09557 protein ORM1-like protein 2, putative, expressed  no original description 	T
27.3.67	RNA.regulation of transcription.putative transcription regulator	MDP0000325245	 moderately similar to ( 204) AT4G12080 | Symbols:  | DNA-binding family protein | chr4:7239466-7241246 FORWARDweakly similar to ( 172) loc_os08g02490 12008.m04393 protein DNA-binding protein, putative, expressed   no original description 	T
26.28	misc.GDSL-motif lipase	MDP0000184619	 highly similar to ( 524) AT3G04290 | Symbols: ATLTL1, LTL1 | LTL1 (LI-TOLERANT LIPASE 1); carboxylesterase/ hydrolase, acting on ester bonds | chr3:1133620-1136223 REVERSEweakly similar to ( 173) APG_BRANA Anter-specific proline-rich protein APG (Protein CEX) (Fragment) - Brassica napus (Rape)moderately similar to ( 492) loc_os02g40440 12002.m09130 protein anther-specific proline-rich protein APG precursor, putative, expressed  no original description 	T
27.3.99	RNA.regulation of transcription.unclassified	MDP0000037858	 highly similar to ( 889) AT3G02320 | Symbols:  | RNA binding / tRNA (guanine-N2-)-methyltransferase | chr3:470097-473492 FORWARDhighly similar to ( 778) loc_os03g57280 12003.m10646 protein N-dimethylguanosine tRNA methyltransferase, putative, expressed   no original description 	T
30.2.3	signalling.receptor kinases.leucine rich repeat III	MDP0000620422	 highly similar to ( 758) AT1G68400 | Symbols:  | leucine-rich repeat transmembrane protein kinase, putative | chr1:25646401-25648916 REVERSEmoderately similar to ( 228) PSKR_DAUCA Phytosulfokine receptor precursor (EC 2.7.11.1) (Phytosulfokine LRR receptor kinase) - Daucus carota (Carrot)highly similar to ( 613) loc_os01g04230 12001.m07059 protein receptor kinase, putative, expressed   no original description 	T
30.3	signalling.calcium	MDP0000373964	 moderately similar to ( 282) AT5G40190 | Symbols:  | calmodulin-binding protein | chr5:16069298-16069849 REVERSEweakly similar to ( 197) loc_os09g20860 12009.m05293 protein calmodulin binding protein, putative, expressed  no original description 	T
26.18	misc.invertase/pectin methylesterase inhibitor family protein	MDP0000249105	 weakly similar to ( 107) AT3G17130 | Symbols:  | invertase/pectin methylesterase inhibitor family protein | chr3:5844495-5845046 REVERSEvery weakly similar to (59.7) PMEI_ACTCH Pectinesterase inhibitor (Pectin methylesterase inhibitor) (PMEI) - Actinidia chinensis (Kiwi) (Yangtao)very weakly similar to (67.8) loc_os01g20970 12001.m08581 protein c/VIF2, putative, expressed  no original description 	T
16.8.3.1	secondary metabolism.flavonoids.dihydroflavonols.dihydroflavonol 4-reductase	MDP0000269612	 moderately similar to ( 376) AT5G58490 | Symbols:  | cinnamoyl-CoA reductase family | chr5:23643068-23644455 FORWARDweakly similar to ( 155) DFRA_DIACA Dihydroflavonol-4-reductase (EC 1.1.1.219) (DFR) (Dihydrokaempferol 4-reductase) - Dianthus caryophyllus (Carnation) (Clove pink)moderately similar to ( 307) loc_os03g60380 12003.m10928 protein dihydroflavonol-4-reductase, putative, expressed  no original description 	T
10.7	cell wall.modification	MDP0000504183	 moderately similar to ( 475) AT2G28950 | Symbols: ATEXPA6, ATEXP6, ATHEXP ALPHA 1.8 | ATEXPA6 (ARABIDOPSIS THALIANA EXPANSIN A6) | chr2:12431840-12433482 REVERSEmoderately similar to ( 449) EXPA7_ORYSA Expansin-A7 precursor (OsEXPA7) (Alpha-expansin-7) (OsEXP7) (OsaEXPa1.26) - Oryza sativa (Rice)moderately similar to ( 449) loc_os03g60720 12003.m10961 protein alpha-expansin 6 precursor, putative, expressed  no original description 	T
35.2	not assigned.unknown	MDP0000397884	 very weakly similar to (78.2) AT4G28690 | Symbols:  | LOCATED IN: cellular_component unknown; BEST Arabidopsis thaliana protein match is: RIN13 (RPM1 INTERACTING PROTEIN 13) (TAIR:AT2G20310.1); Has 370 Blast hits to 352 proteins in 105 species: Archae - 0; Bacteria - 10; Metazoa - 143; Fungi - 82; Plants - 80; Viruses - 3; Other Eukaryotes - 52 (source: NCBI BLink). | chr4:14159093-14160876 FORWARDvery weakly similar to (72.4) loc_os10g32850 12010.m21790 protein expressed protein  no original description 	T
35.1.26	not assigned.no ontology.DC1 domain containing protein	MDP0000858290	 moderately similar to ( 387) AT2G16050 | Symbols:  | FUNCTIONS IN: molecular_function unknown; INVOLVED IN: biological_process unknown; LOCATED IN: cellular_component unknown; CONTAINS InterPro DOMAIN/s: DC1 (InterPro:IPR004146); BEST Arabidopsis thaliana protein match is: DC1 domain-containing protein (TAIR:AT1G20990.1); Has 719 Blast hits to 448 proteins in 12 species: Archae - 0; Bacteria - 0; Metazoa - 0; Fungi - 2; Plants - 717; Viruses - 0; Other Eukaryotes - 0 (source: NCBI BLink). | chr2:6980450-6981939 FORWARDmoderately similar to ( 210) loc_os12g40470 12012.m07831 protein C1-like domain containing protein, expressed  no original description 	T
29.5.11.4.3.2	protein.degradation.ubiquitin.E3.SCF.FBOX	MDP0000302015	 weakly similar to ( 105) AT3G06240 | Symbols:  | F-box family protein | chr3:1887336-1888619 FORWARDvery weakly similar to (63.2) loc_os12g06740 12012.m26845 protein F-box protein interaction domain containing protein, expressed   no original description 	T
31.1	cell.organisation	MDP0000878209	 highly similar to ( 503) AT5G20490 | Symbols: XIK, ATXIK, XI-17 | XIK; motor/ protein binding | chr5:6927064-6936079 REVERSEmoderately similar to ( 469) loc_os06g29350 12006.m07479 protein XIE, putative, expressed  no original description 	T
21.2	redox.ascorbate and glutathione	MDP0000581631	 moderately similar to ( 225) AT5G53560 | Symbols: ATB5-A, B5 #2, ATCB5-E, CB5-E | CB5-E (CYTOCHROME B5 ISOFORM E); heme binding | chr5:21759628-21760353 FORWARDweakly similar to ( 196) CYB5_TOBAC Cytochrome b5 - Nicotiana tabacum (Common tobacco)moderately similar to ( 207) loc_os10g37420 12010.m21998 protein cytochrome b5, putative, expressed no original description 	T
35.2	not assigned.unknown	MDP0000238581	 highly similar to ( 625) AT4G37680 | Symbols: HHP4 | HHP4 (heptahelical protein 4); receptor | chr4:17701231-17702568 FORWARDhighly similar to ( 602) loc_os03g13040 12003.m06750 protein haemolysin-III related family protein, expressed  no original description 	T
35.2	not assigned.unknown	MDP0000762392	 weakly similar to ( 175) AT1G29195 | Symbols:  | unknown protein | chr1:10202673-10203254 REVERSEweakly similar to ( 154) loc_os01g55370 12001.m11696 protein expressed protein  no original description 	T
20.2.99	stress.abiotic.unspecified	MDP0000166732	 weakly similar to ( 194) AT2G47710 | Symbols:  | universal stress protein (USP) family protein | chr2:19555045-19555956 REVERSEweakly similar to ( 179) loc_os12g36640 12012.m07450 protein fiber protein Fb19, putative, expressed  no original description 	T
35.1	not assigned.no ontology	MDP0000150861	 highly similar to ( 537) AT4G18905 | Symbols:  | transducin family protein / WD-40 repeat family protein | chr4:10360234-10362991 FORWARDvery weakly similar to (53.5) GBB_MAIZE Guanine nucleotide-binding protein subunit beta - Zea mays (Maize)moderately similar to ( 487) loc_os01g69970 12001.m13046 protein periodic tryptophan protein 1, putative, expressed no original description 	T
27.3.25	RNA.regulation of transcription.MYB domain transcription factor family	MDP0000708334	 moderately similar to ( 433) AT2G37630 | Symbols: ATPHAN, AS1, ATMYB91, MYB91 | AS1 (ASYMMETRIC LEAVES 1); DNA binding / protein homodimerization/ transcription factor | chr2:15781798-15782901 REVERSEvery weakly similar to ( 100) GAM1_ORYSA Transcription factor GAMYB (OsGAMyb) - Oryza sativa (Rice)moderately similar to ( 361) loc_os12g38400 12012.m26938 protein protein ASYMMETRIC LEAVES1, putative, expressed  no original description 	T
33.99	development.unspecified	MDP0000623346	 highly similar to ( 535) AT3G58040 | Symbols: SINAT2 | SINAT2 (SEVEN IN ABSENTIA OF ARABIDOPSIS 2); protein binding / ubiquitin-protein ligase/ zinc ion binding | chr3:21489612-21491085 FORWARDweakly similar to ( 150) SINAL_DROME Probable E3 ubiquitin-protein ligase sina-like CG13030 (EC 6.3.2.-) - Drosophila melanogaster (Fruit fly)moderately similar to ( 479) loc_os01g13370 12001.m07946 protein ubiquitin ligase SINAT3, putative, expressed no original description 	T
29.2.3	protein.synthesis.initiation	MDP0000138307	 moderately similar to ( 347) AT4G33250 | Symbols: EIF3K, TIF3K1, ATTIF3K1 | EIF3K (EUKARYOTIC TRANSLATION INITIATION FACTOR 3K); translation initiation factor | chr4:16039066-16040617 REVERSEmoderately similar to ( 359) IF3C_ORYSA Eukaryotic translation initiation factor 3 subunit 12 (eIF-3 p25) (eIF3k) - Oryza sativa (Rice)moderately similar to ( 359) loc_os03g08450 12003.m06353 protein eukaryotic translation initiation factor 3 subunit 12, putative, expressed  no original description 	T
21.3	redox.heme	MDP0000312359	 moderately similar to ( 249) AT2G16060 | Symbols: GLB1, AHB1, ARATH GLB1, NSHB1, ATGLB1 | AHB1 (ARABIDOPSIS HEMOGLOBIN 1); oxygen binding / oxygen transporter | chr2:6982782-6983522 REVERSEmoderately similar to ( 267) HBL1_GOSHI Non-symbiotic hemoglobin 1 (Hb1) (GOShi GLB1) - Gossypium hirsutum (Upland cotton)moderately similar to ( 239) loc_os03g12510 12003.m06701 protein non-symbiotic hemoglobin 2, putative, expressed no original description 	T
1.1.1.1	PS.lightreaction.photosystem II.LHC-II	MDP0000135220	 moderately similar to ( 276) AT1G61520 | Symbols: LHCA3 | LHCA3; chlorophyll binding | chr1:22700493-22701149 FORWARDvery weakly similar to (94.4) CB12_PETHY Chlorophyll a-b binding protein, chloroplast precursor (LHCI type II CAB) - Petunia hybrida (Petunia)moderately similar to ( 254) loc_os02g10390 12002.m33354 protein chlorophyll a-b binding protein 8, chloroplast precursor, putative, expressed   no original description 	T
33.99	development.unspecified	MDP0000309933	 highly similar to ( 820) AT4G33400 | Symbols:  | dem protein-related / defective embryo and meristems protein-related | chr4:16078189-16080410 REVERSEvery weakly similar to (53.5) CB12_PETHY Chlorophyll a-b binding protein, chloroplast precursor (LHCI type II CAB) - Petunia hybrida (Petunia)highly similar to ( 766) loc_os02g08190 12002.m06165 protein protein CYPRO4, putative, expressed    no original description 	T
10.6.3	cell wall.degradation.pectate lyases and polygalacturonases	MDP0000277149	 highly similar to ( 614) AT1G67750 | Symbols:  | pectate lyase family protein | chr1:25401660-25403165 FORWARDmoderately similar to ( 400) PEL_LILLO Pectate lyase precursor (EC 4.2.2.2) - Lilium longiflorum (Trumpet lily)highly similar to ( 549) loc_os04g05050 12004.m35243 protein pectate lyase 8 precursor, putative, expressed   no original description 	T
29.4	protein.postranslational modification	MDP0000418062	 moderately similar to ( 342) AT1G20930 | Symbols: CDKB2;2 | CDKB2;2 (CYCLIN-DEPENDENT KINASE B2;2); cyclin-dependent protein kinase/ kinase | chr1:7292752-7294664 REVERSEmoderately similar to ( 335) CDC2D_ANTMA Cell division control protein 2 homolog D (EC 2.7.11.22) (EC 2.7.11.23) - Antirrhinum majus (Garden snapdragon)moderately similar to ( 317) loc_os08g40170 12008.m07997 protein cell division control protein 2 homolog D, putative, expressed  no original description 	T
29.2.4	protein.synthesis.elongation	MDP0000626096	 weakly similar to ( 142) AT5G19510 | Symbols:  | elongation factor 1B alpha-subunit 2 (eEF1Balpha2) | chr5:6581854-6583137 REVERSEweakly similar to ( 144) EF1B_ORYSA Elongation factor 1-beta (EF-1-beta) (Elongation factor 1B-alpha 2) (eEF-1B alpha) (Elongation factor 1-beta') (EF-1-beta') - Oryza sativa (Rice)weakly similar to ( 144) loc_os07g46750 12007.m08892 protein elongation factor 1-beta, putative, expressed  no original description 	T
16.7	secondary metabolism.wax	MDP0000461409	 highly similar to ( 828) AT1G02205 | Symbols: CER1 | CER1 (ECERIFERUM 1); octadecanal decarbonylase | chr1:418818-422154 FORWARDhighly similar to ( 760) loc_os10g33250 12010.m21915 protein CER1, putative, expressed  no original description 	T
29.2.1.1.3.2.1712	protein.synthesis.ribosomal protein.prokaryotic.unknown organellar.50S subunit.L7/L12	MDP0000329721	 moderately similar to ( 232) AT1G68660 | Symbols:  | FUNCTIONS IN: molecular_function unknown; INVOLVED IN: protein catabolic process; EXPRESSED IN: 23 plant structures; EXPRESSED DURING: 14 growth stages; CONTAINS InterPro DOMAIN/s: Adaptor protein ClpS, core (InterPro:IPR003769), Ribosomal protein L7/L12, C-terminal/adaptor protein ClpS-like (InterPro:IPR014719); Has 79 Blast hits to 79 proteins in 34 species: Archae - 0; Bacteria - 46; Metazoa - 0; Fungi - 0; Plants - 30; Viruses - 0; Other Eukaryotes - 3 (source: NCBI BLink). | chr1:25778058-25779083 REVERSEweakly similar to ( 196) loc_os08g33540 12008.m080165 protein ATP-dependent Clp protease adaptor protein ClpS containing protein, expressed no original description 	T
29.5.5	protein.degradation.serine protease	MDP0000120995	 moderately similar to ( 318) AT1G63120 | Symbols: ATRBL2 | ATRBL2 (Arabidopsis thaliana Rhomboid-like 2); serine-type endopeptidase | chr1:23409054-23410725 REVERSEmoderately similar to ( 293) loc_os09g35730 12009.m06524 protein membrane protein, putative, expressed  no original description 	T
26.2	misc.UDP glucosyl and glucoronyl transferases	MDP0000120543	 highly similar to ( 898) AT3G25140 | Symbols: GAUT8, QUA1 | QUA1 (QUASIMODO 1); polygalacturonate 4-alpha-galacturonosyltransferase/ transferase, transferring glycosyl groups / transferase, transferring hexosyl groups | chr3:9154748-9156642 FORWARDhighly similar to ( 659) loc_os02g29530 12002.m08096 protein transferase, transferring glycosyl groups, putative, expressed   no original description 	T
1.1.1.2	PS.lightreaction.photosystem II.PSII polypeptide subunits	MDP0000596458	 moderately similar to ( 305) AT1G44575 | Symbols: NPQ4, PSBS | NPQ4 (NONPHOTOCHEMICAL QUENCHING); chlorophyll binding / xanthophyll binding | chr1:16871768-16872548 FORWARDmoderately similar to ( 325) PSBS_SPIOL Photosystem II 22 kDa protein, chloroplast precursor (CP22) - Spinacia oleracea (Spinach)moderately similar to ( 290) loc_os01g64960 12001.m150788 protein photosystem II 22 kDa protein, chloroplast precursor, putative, expressed   no original description 	T
35.2	not assigned.unknown	MDP0000285782	 weakly similar to ( 160) AT1G11440 | Symbols:  | FUNCTIONS IN: molecular_function unknown; INVOLVED IN: biological_process unknown; LOCATED IN: cellular_component unknown; EXPRESSED IN: 22 plant structures; EXPRESSED DURING: 13 growth stages; BEST Arabidopsis thaliana protein match is: glycine-rich protein (TAIR:AT3G29075.1); Has 22740 Blast hits to 8622 proteins in 417 species: Archae - 22; Bacteria - 573; Metazoa - 11353; Fungi - 2147; Plants - 905; Viruses - 631; Other Eukaryotes - 7109 (source: NCBI BLink). | chr1:3849418-3850509 FORWARDvery weakly similar to (65.1) loc_os06g12580 12006.m32024 protein pro-resilin precursor, putative, expressed  no original description 	T
10.1.21	cell wall.precursor synthesis.phosphomannomutase	MDP0000164473	 moderately similar to ( 423) AT2G45790 | Symbols: ATPMM, PMM | PMM (PHOSPHOMANNOMUTASE); phosphomannomutase | chr2:18855876-18857753 FORWARDmoderately similar to ( 429) loc_os04g58580 12004.m101698 protein phosphomannomutase, putative, expressed  no original description 	T
29.5.11.4.2	protein.degradation.ubiquitin.E3.RING	MDP0000479856	 moderately similar to ( 217) AT3G48070 | Symbols:  | protein binding / zinc ion binding | chr3:17750878-17752297 FORWARDweakly similar to ( 140) loc_os04g38970 12004.m08886 protein expressed protein  no original description 	T
35.2	not assigned.unknown	MDP0000183967	 highly similar to ( 604) AT4G14950 | Symbols:  | unknown protein | chr4:8545517-8547722 FORWARDhighly similar to ( 544) loc_os01g57066 12001.m42516 protein transmembrane protein 49, putative, expressed no original description 	T
16.8.3.1	secondary metabolism.flavonoids.dihydroflavonols.dihydroflavonol 4-reductase	MDP0000174748	 moderately similar to ( 429) AT1G61720 | Symbols: BAN | BAN (BANYULS); oxidoreductase | chr1:22791326-22792757 REVERSEmoderately similar to ( 286) DFRA_VITVI Dihydroflavonol-4-reductase (EC 1.1.1.219) (DFR) (Dihydrokaempferol 4-reductase) - Vitis vinifera (Grape)moderately similar to ( 337) loc_os04g53850 12004.m35472 protein leucoanthocyanidin reductase, putative, expressed  no original description 	T
35.2	not assigned.unknown	MDP0000360438	 weakly similar to ( 110) MAL11_MALDO Major allergen Mal d 1 (Mal d I) - Malus domestica (Apple) (Malus sylvestris) no original description 	T
35.2	not assigned.unknown	MDP0000186666	 moderately similar to ( 296) AT2G39650 | Symbols:  | unknown protein | chr2:16528094-16529274 REVERSEmoderately similar to ( 286) loc_os01g74250 12001.m13452 protein plant-specific domain TIGR01615 family protein, expressed no original description 	T
29.2.1.2.1.31	protein.synthesis.ribosomal protein.eukaryotic.40S subunit.SA	MDP0000286465	 moderately similar to ( 407) AT1G72370 | Symbols: P40, AP40, RP40, RPSAA | P40; structural constituent of ribosome | chr1:27243148-27244842 REVERSEmoderately similar to ( 423) RSSA_SOYBN 40S ribosomal protein SA (p40) - Glycine max (Soybean)moderately similar to ( 414) loc_os03g08440 12003.m35048 protein 40S ribosomal protein SA, putative, expressed no original description 	T
26.10	misc.cytochrome P450	MDP0000218244	 highly similar to ( 577) AT3G14690 | Symbols: CYP72A15 | CYP72A15; electron carrier/ heme binding / iron ion binding / monooxygenase/ oxygen binding | chr3:4937410-4939310 FORWARDhighly similar to ( 558) C72A1_CATRO Cytochrome P450 72A1 (EC 1.3.3.9) (CYPLXXII) (Secologanin synthase) (SLS) - Catharanthus roseus (Rosy periwinkle) (Madagascar periwinkle)highly similar to ( 523) loc_os01g43750 12001.m10637 protein cytochrome P450 72A1, putative, expressed  no original description 	T
35.2	not assigned.unknown	MDP0000150022	 moderately similar to ( 228) AT2G38430 | Symbols:  | unknown protein | chr2:16092950-16094810 REVERSEvery weakly similar to (80.1) loc_os02g30140 12002.m77811 protein expressed protein  no original description 	T
35.2	not assigned.unknown	MDP0000123077	 very weakly similar to (79.0) AT5G02440 | Symbols:  | unknown protein | chr5:531066-532725 FORWARD no original description 	T
35.1	not assigned.no ontology	MDP0000653461	 highly similar to ( 514) AT1G69840 | Symbols:  | band 7 family protein | chr1:26293932-26295150 REVERSEmoderately similar to ( 489) loc_os08g30790 12008.m26682 protein hypersensitive-induced response protein, putative, expressed no original description 	T
29.6	protein.folding	MDP0000201420	 highly similar to ( 867) AT3G03960 | Symbols:  | chaperonin, putative | chr3:1024432-1027604 FORWARDweakly similar to ( 177) TCPE1_AVESA T-complex protein 1 subunit epsilon (TCP-1-epsilon) (CCT-epsilon) (TCP-K19) - Avena sativa (Oat)highly similar to ( 903) loc_os03g59020 12003.m10803 protein T-complex protein 1 subunit theta, putative, expressed  no original description 	T
16.2	secondary metabolism.phenylpropanoids	MDP0000307780	 highly similar to ( 509) AT5G48930 | Symbols: HCT | HCT (HYDROXYCINNAMOYL-COA SHIKIMATE/QUINATE HYDROXYCINNAMOYL TRANSFERASE); quinate O-hydroxycinnamoyltransferase/ shikimate O-hydroxycinnamoyltransferase/ transferase | chr5:19836654-19838092 REVERSEmoderately similar to ( 240) HCBT2_DIACA Anthranilate N-benzoyltransferase protein 2 (EC 2.3.1.144) (Anthranilate N-hydroxycinnamoyl/benzoyltransferase 2) - Dianthus caryophyllus (Carnation) (Clove pink)moderately similar to ( 404) loc_os04g42250 12004.m35365 protein anthranilate N-benzoyltransferase protein 2, putative, expressed  no original description 	T
35.2	not assigned.unknown	MDP0000307644	 weakly similar to ( 108) AT4G35320 | Symbols:  | unknown protein | chr4:16806139-16806582 REVERSEweakly similar to ( 111) loc_os02g48470 12002.m09881 protein expressed protein   no original description 	T
34.16	transport.ABC transporters and multidrug resistance systems	MDP0000124006	 nearly identical (1560) AT1G27940 | Symbols: PGP13 | PGP13 (P-GLYCOPROTEIN 13); ATPase, coupled to transmembrane movement of substances | chr1:9733597-9738129 REVERSEhighly similar to ( 884) MDR_ORYSA Putative multidrug resistance protein (P-glycoprotein) - Oryza sativa (Rice)nearly identical (1111) loc_os04g38570 12004.m101543 protein multidrug resistance protein 11, putative, expressed   no original description 	T
29.5.11.4.3.2	protein.degradation.ubiquitin.E3.SCF.FBOX	MDP0000292564	 very weakly similar to (61.2) AT3G25750 | Symbols:  | F-box family protein | chr3:9400955-9402088 FORWARDvery weakly similar to (85.1) loc_os06g05580 12006.m32240 protein F-box domain containing protein, expressed no original description 	T
29.3.2	protein.targeting.mitochondria	MDP0000270353	 moderately similar to ( 217) AT3G20000 | Symbols: TOM40 | TOM40; P-P-bond-hydrolysis-driven protein transmembrane transporter/ voltage-gated anion channel | chr3:6967685-6970247 FORWARDmoderately similar to ( 219) loc_os03g20750 12003.m07482 protein mitochondrial import receptor subunit TOM40 homolog 1, putative, expressed no original description 	T
35.2	not assigned.unknown	MDP0000352268	   no original description 	T
23.3.3	nucleotide metabolism.salvage.NUDIX hydrolases	MDP0000307070	 moderately similar to ( 232) AT1G28960 | Symbols: ATNUDT15, ATNUDX15 | ATNUDX15 (ARABIDOPSIS THALIANA NUDIX HYDROLASE HOMOLOG 15); hydrolase | chr1:10110135-10111607 REVERSEmoderately similar to ( 229) loc_os08g28860 12008.m06891 protein peroxisomal coenzyme A diphosphatase NUDT7, putative, expressed  no original description 	T
31.2	cell.division	MDP0000120387	 highly similar to ( 593) AT2G38670 | Symbols: PECT1 | PECT1 (PHOSPHORYLETHANOLAMINE CYTIDYLYLTRANSFERASE 1); ethanolamine-phosphate cytidylyltransferase | chr2:16168979-16171680 FORWARDhighly similar to ( 578) loc_os11g03050 12011.m04501 protein ethanolamine-phosphate cytidylyltransferase, putative, expressed no original description 	T
33.99	development.unspecified	MDP0000120387	 highly similar to ( 593) AT2G38670 | Symbols: PECT1 | PECT1 (PHOSPHORYLETHANOLAMINE CYTIDYLYLTRANSFERASE 1); ethanolamine-phosphate cytidylyltransferase | chr2:16168979-16171680 FORWARDhighly similar to ( 578) loc_os11g03050 12011.m04501 protein ethanolamine-phosphate cytidylyltransferase, putative, expressed no original description 	T
13.1.3.5.4	amino acid metabolism.synthesis.aspartate family.lysine.diaminopimelate epimerase	MDP0000195636	 moderately similar to ( 448) AT3G53580 | Symbols:  | diaminopimelate epimerase family protein | chr3:19864784-19866907 FORWARDvery weakly similar to (53.9) RL8_TOBAC 60S ribosomal protein L8 (60S ribosomal protein L2) - Nicotiana tabacum (Common tobacco)moderately similar to ( 426) loc_os12g37960 12012.m26936 protein diaminopimelate epimerase, putative, expressed  no original description 	T
27.3.35	RNA.regulation of transcription.bZIP transcription factor family	MDP0000435971	 moderately similar to ( 403) AT1G06070 | Symbols:  | bZIP transcription factor, putative (bZIP69) | chr1:1835201-1837116 REVERSEmoderately similar to ( 276) RF2A_ORYSA Transcription factor RF2a - Oryza sativa (Rice)moderately similar to ( 276) loc_os09g34060 12009.m06455 protein transcription factor RF2a, putative, expressed   no original description 	T
35.1	not assigned.no ontology	MDP0000936748	 very weakly similar to (74.3) AT1G28400 | Symbols:  | unknown protein | chr1:9972732-9973739 REVERSEvery weakly similar to (78.2) E6_GOSHI Protein E6 - Gossypium hirsutum (Upland cotton) no original description 	T
35.2	not assigned.unknown	MDP0000230999	 highly similar to ( 689) AT4G21150 | Symbols: HAP6 | HAP6 (HAPLESS 6); dolichyl-diphosphooligosaccharide-protein glycotransferase | chr4:11278646-11283599 FORWARDhighly similar to ( 644) loc_os01g68324 12001.m12935 protein dolichyl-diphosphooligosaccharide--protein glycosyltransferase 63 kDasubunit precursor, putative, expressed  MSP1_C  no original description 	T
26.16	misc.myrosinases-lectin-jacalin	MDP0000260373	 weakly similar to ( 198) AT5G01090 | Symbols:  | legume lectin family protein | chr5:33055-34116 FORWARDvery weakly similar to (51.6) ARC1_PHAVU Arcelin-1 precursor - Phaseolus vulgaris (Kidney bean) (French bean)very weakly similar to (76.3) loc_os06g17490 12006.m06461 protein protein kinase, putative, expressed   no original description 	T
34.9	transport.metabolite transporters at the mitochondrial membrane	MDP0000716014	 highly similar to ( 562) AT4G28390 | Symbols: AAC3, ATAAC3 | AAC3 (ADP/ATP CARRIER 3); ATP:ADP antiporter/ binding | chr4:14041486-14042781 REVERSEhighly similar to ( 513) ADT1_SOLTU ADP,ATP carrier protein, mitochondrial precursor (ADP/ATP translocase) (Adenine nucleotide translocator) (ANT) - Solanum tuberosum (Potato)highly similar to ( 521) loc_os05g23720 12005.m06665 protein ADP,ATP carrier protein, mitochondrial precursor, putative, expressed no original description 	T
35.2	not assigned.unknown	MDP0000470064	 highly similar to ( 714) AT3G44330 | Symbols:  | INVOLVED IN: protein processing; LOCATED IN: mitochondrion, endoplasmic reticulum, plasma membrane, vacuole; EXPRESSED IN: 25 plant structures; EXPRESSED DURING: 13 growth stages; CONTAINS InterPro DOMAIN/s: Nicalin (InterPro:IPR016574), EF-HAND 1 (InterPro:IPR018247), Nicastrin (InterPro:IPR008710); Has 192 Blast hits to 192 proteins in 79 species: Archae - 6; Bacteria - 5; Metazoa - 124; Fungi - 0; Plants - 26; Viruses - 0; Other Eukaryotes - 31 (source: NCBI BLink). | chr3:16006754-16010671 FORWARDhighly similar to ( 653) loc_os08g01180 12008.m04267 protein nicalin precursor, putative, expressed  no original description 	T
29.2.3	protein.synthesis.initiation	MDP0000316345	 highly similar to ( 568) AT1G53900 | Symbols:  | GTP binding / translation initiation factor | chr1:20127634-20130834 FORWARDhighly similar to ( 513) loc_os12g31380 12012.m06947 protein translation initiation factor eIF-2B alpha subunit, putative, expressed   no original description 	T
11.1.12	lipid metabolism.FA synthesis and FA elongation.ACP protein	MDP0000258052	 very weakly similar to (99.8) AT4G25050 | Symbols: ACP4 | ACP4 (acyl carrier protein 4); acyl carrier | chr4:12870178-12871024 FORWARDvery weakly similar to (96.7) ACP1_SPIOL Acyl carrier protein 1, chloroplast precursor (Acyl carrier protein I) (ACP I) - Spinacia oleracea (Spinach)very weakly similar to (92.8) loc_os08g43580 12008.m26784 protein acyl carrier protein 3, chloroplast precursor, putative, expressed  no original description 	T
35.2	not assigned.unknown	MDP0000191802	 nearly identical (1007) AT5G10840 | Symbols:  | endomembrane protein 70, putative | chr5:3424910-3427797 REVERSEhighly similar to ( 933) loc_os08g44100 12008.m08384 protein transmembrane 9 superfamily protein member 2 precursor, putative, expressed  no original description 	T
13.1.5.3.1	amino acid metabolism.synthesis.serine-glycine-cysteine group.cysteine.OASTL	MDP0000274867	 moderately similar to ( 441) AT4G14880 | Symbols: OASA1, CYTACS1, ATCYS-3A | OASA1 (O-ACETYLSERINE (THIOL) LYASE (OAS-TL) ISOFORM A1); cysteine synthase | chr4:8518209-8520050 REVERSEmoderately similar to ( 473) CYSK_SPIOL Cysteine synthase (EC 2.5.1.47) (O-acetylserine sulfhydrylase) (O-acetylserine (Thiol)-lyase) (CSase A) (OAS-TL A) - Spinacia oleracea (Spinach)moderately similar to ( 462) loc_os03g53650 12003.m35628 protein cysteine synthase, putative, expressed no original description 	T
29.1.22	protein.aa activation.asparagine-tRNA ligase	MDP0000248906	 highly similar to ( 630) AT3G07420 | Symbols: NS2, ATNS2, SYNC2_ARATH, SYNC2 | NS2; asparagine-tRNA ligase | chr3:2374179-2376644 REVERSEmoderately similar to ( 478) loc_os12g22600 12012.m06103 protein asparaginyl-tRNA synthetase, cytoplasmic 2, putative, expressed  no original description 	T
30.5	signalling.G-proteins	MDP0000927251	 highly similar to ( 913) AT3G60860 | Symbols:  | guanine nucleotide exchange family protein | chr3:22484804-22491510 FORWARDhighly similar to ( 872) loc_os03g14260 12003.m101270 protein brefeldin A-inhibited guanine nucleotide-exchange protein 2, putative, expressed no original description 	T
35.2	not assigned.unknown	MDP0000245031	 highly similar to ( 907) AT2G23420 | Symbols: NAPRT2 | NAPRT2 (NICOTINATE PHOSPHORIBOSYLTRANSFERASE 2); nicotinate phosphoribosyltransferase | chr2:9971819-9974909 FORWARDhighly similar to ( 872) loc_os03g62110 12003.m34955 protein nicotinate phosphoribosyltransferase, putative, expressed no original description 	T
34.14	transport.unspecified cations	MDP0000242466	 moderately similar to ( 451) AT5G22830 | Symbols: ATMGT10, GMN10 | ATMGT10 (MAGNESIUM (MG) TRANSPORTER 10); magnesium ion transmembrane transporter | chr5:7627676-7630633 FORWARDmoderately similar to ( 419) loc_os03g48000 12003.m101439 protein GMN10, putative, expressed  no original description 	T
20.1.3.1	stress.biotic.signalling.MLO-like	MDP0000152684	 moderately similar to ( 251) AT3G45290 | Symbols: MLO3, ATMLO3 | MLO3 (MILDEW RESISTANCE LOCUS O 3); calmodulin binding | chr3:16617586-16621352 FORWARDmoderately similar to ( 215) MLOH1_HORVU MLO protein homolog 1 - Hordeum vulgare (Barley)moderately similar to ( 216) loc_os10g39520 12010.m06723 protein MLO-like protein 10, putative, expressed no original description 	T
27.4	RNA.RNA binding	MDP0000224531	 moderately similar to ( 211) AT5G50250 | Symbols:  | 31 kDa ribonucleoprotein, chloroplast, putative / RNA-binding protein RNP-T, putative / RNA-binding protein 1/2/3, putative / RNA-binding protein cp31, putative | chr5:20452677-20453965 REVERSEmoderately similar to ( 201) ROC1_SPIOL 28 kDa ribonucleoprotein, chloroplast (28RNP) - Spinacia oleracea (Spinach)moderately similar to ( 201) loc_os09g39180 12009.m06857 protein ribonucleoprotein, chloroplast precursor, putative, expressed   no original description 	T
29.4	protein.postranslational modification	MDP0000230989	 highly similar to ( 658) AT4G28540 | Symbols: CKL6, PAPK1 | CKL6 (CASEIN KINASE I-LIKE 6); kinase/ protein serine/threonine kinase | chr4:14107284-14110511 FORWARDvery weakly similar to (64.7) CDPK1_ORYSA Calcium-dependent protein kinase, isoform 1 (EC 2.7.11.1) (CDPK 1) - Oryza sativa (Rice)highly similar to ( 620) loc_os02g40860 12002.m09171 protein casein kinase I isoform delta-like, putative, expressed   no original description 	T
13.2.2.3	amino acid metabolism.degradation.glutamate family.arginine	MDP0000813339	 highly similar to ( 900) AT4G34710 | Symbols: ADC2, SPE2, ATADC2 | ADC2 (ARGININE DECARBOXYLASE 2); arginine decarboxylase | chr4:16560315-16562450 REVERSEhighly similar to ( 926) SPE1_PEA Arginine decarboxylase (EC 4.1.1.19) (ARGDC) (ADC) - Pisum sativum (Garden pea)highly similar to ( 807) loc_os06g04070 12006.m05138 protein arginine decarboxylase, putative, expressed   no original description 	T
22.1.3	polyamine metabolism.synthesis.arginine decarboxylase	MDP0000813339	 highly similar to ( 900) AT4G34710 | Symbols: ADC2, SPE2, ATADC2 | ADC2 (ARGININE DECARBOXYLASE 2); arginine decarboxylase | chr4:16560315-16562450 REVERSEhighly similar to ( 926) SPE1_PEA Arginine decarboxylase (EC 4.1.1.19) (ARGDC) (ADC) - Pisum sativum (Garden pea)highly similar to ( 807) loc_os06g04070 12006.m05138 protein arginine decarboxylase, putative, expressed   no original description 	T
35.1	not assigned.no ontology	MDP0000165386	 weakly similar to ( 109) AT5G28750 | Symbols:  | thylakoid assembly protein, putative | chr5:10784142-10785677 REVERSEvery weakly similar to (89.7) loc_os03g43430 12003.m09389 protein THA4, putative, expressed  no original description 	T
29.7	protein.glycosylation	MDP0000151273	 highly similar to ( 576) AT5G66680 | Symbols: DGL1 | DGL1; dolichyl-diphosphooligosaccharide-protein glycotransferase | chr5:26617840-26620581 REVERSEhighly similar to ( 561) loc_os07g10830 12007.m05538 protein dolichyl-diphosphooligosaccharide--protein glycosyltransferase 48 kDasubunit precursor, putative, expressed   no original description 	T
35.2	not assigned.unknown	MDP0000908678	 moderately similar to ( 335) AT2G46230 | Symbols:  | FUNCTIONS IN: molecular_function unknown; INVOLVED IN: biological_process unknown; LOCATED IN: cellular_component unknown; EXPRESSED IN: 22 plant structures; EXPRESSED DURING: 13 growth stages; CONTAINS InterPro DOMAIN/s: Protein of unknown function DUF652 (InterPro:IPR006984), Nucleotide binding protein, PINc (InterPro:IPR006596); BEST Arabidopsis thaliana protein match is: unknown protein (TAIR:AT1G26530.1); Has 441 Blast hits to 441 proteins in 161 species: Archae - 15; Bacteria - 0; Metazoa - 185; Fungi - 119; Plants - 49; Viruses - 0; Other Eukaryotes - 73 (source: NCBI BLink). | chr2:18984694-18985850 REVERSEmoderately similar to ( 357) loc_os06g06410 12006.m05367 protein nucleolar protein involved in pre-mRNA processing, putative, expressed no original description 	T
30.2.3	signalling.receptor kinases.leucine rich repeat III	MDP0000402658	 highly similar to ( 940) AT2G01210 | Symbols:  | leucine-rich repeat transmembrane protein kinase, putative | chr2:119509-121734 REVERSEmoderately similar to ( 238) PSKR_DAUCA Phytosulfokine receptor precursor (EC 2.7.11.1) (Phytosulfokine LRR receptor kinase) - Daucus carota (Carrot)highly similar to ( 838) loc_os01g33090 12001.m09626 protein ATP binding protein, putative, expressed    no original description 	T
34.18	transport.unspecified anions	MDP0000026235	 nearly identical (1068) AT3G62270 | Symbols:  | anion exchange family protein | chr3:23042528-23045633 REVERSEnearly identical (1051) loc_os12g37840 12012.m26773 protein boron transporter 1, putative, expressed MSP1_C  no original description 	T
35.1.1	not assigned.no ontology.ABC1 family protein	MDP0000818363	 highly similar to ( 570) AT5G05200 | Symbols:  | ABC1 family protein | chr5:1544206-1547082 REVERSEhighly similar to ( 543) loc_os07g12530 12007.m05705 protein ubiquinone biosynthesis protein ubiB, putative, expressed  no original description 	T
29.2.1.2.2.57	protein.synthesis.ribosomal protein.eukaryotic.60S subunit.L7A	MDP0000674102	 moderately similar to ( 388) AT3G62870 | Symbols:  | 60S ribosomal protein L7A (RPL7aB) | chr3:23242862-23244273 REVERSEmoderately similar to ( 395) RL7A_ORYSA 60S ribosomal protein L7a - Oryza sativa (Rice)moderately similar to ( 395) loc_os09g32976 12009.m22160 protein 60S ribosomal protein L7a, putative, expressed no original description 	T
29.4.1.57	protein.postranslational modification.kinase.receptor like cytoplasmatic kinase VII	MDP0000142616	 highly similar to ( 593) AT3G59350 | Symbols:  | serine/threonine protein kinase, putative | chr3:21932930-21934883 FORWARDweakly similar to ( 199) NORK_MEDTR Nodulation receptor kinase precursor (EC 2.7.11.1) (Does not make infections protein 2) (Symbiosis receptor-like kinase) (MtSYMRK) - Medicago truncatula (Barrel medic)highly similar to ( 503) loc_os03g62700 12003.m11147 protein protein kinase Pti1, putative, expressed  no original description 	T
35.2	not assigned.unknown	MDP0000326031	  no original description 	T
26.10	misc.cytochrome P450	MDP0000473817	 highly similar to ( 691) AT5G04660 | Symbols: CYP77A4 | CYP77A4; electron carrier/ heme binding / iron ion binding / monooxygenase/ oxygen binding | chr5:1336049-1337587 FORWARDhighly similar to ( 701) C77A3_SOYBN Cytochrome P450 77A3 (EC 1.14.-.-) - Glycine max (Soybean)moderately similar to ( 493) loc_os04g33370 12004.m08433 protein cytochrome P450 77A2, putative, expressed   no original description 	T
35.1	not assigned.no ontology	MDP0000252629	 highly similar to ( 933) AT1G14670 | Symbols:  | endomembrane protein 70, putative | chr1:5037669-5040199 FORWARDhighly similar to ( 877) loc_os03g13380 12003.m06783 protein transmembrane 9 superfamily protein member 1 precursor, putative, expressed  no original description 	T
29.5	protein.degradation	MDP0000347533	 very weakly similar to (91.3) AT5G22860 | Symbols:  | serine carboxypeptidase S28 family protein | chr5:7639907-7642945 REVERSEvery weakly similar to (93.2) loc_os01g56150 12001.m11771 protein lysosomal Pro-X carboxypeptidase precursor, putative, expressed no original description 	T
29.2.1.2.1.25	protein.synthesis.ribosomal protein.eukaryotic.40S subunit.S25	MDP0000197641	 very weakly similar to (68.6) AT2G21580 | Symbols:  | 40S ribosomal protein S25 (RPS25B) | chr2:9236629-9237510 FORWARDvery weakly similar to (64.7) loc_os09g39540 12009.m060074 protein 40S ribosomal protein S25, putative, expressed   no original description 	T
29.4	protein.postranslational modification	MDP0000139362	 moderately similar to ( 500) AT1G10830 | Symbols:  | sodium symporter-related | chr1:3606411-3607449 REVERSEmoderately similar to ( 478) loc_os12g21710 12012.m06016 protein nnrU, putative, expressed  COG4907  no original description 	T
35.1	not assigned.no ontology	MDP0000258630	 highly similar to ( 653) AT1G73170 | Symbols:  | ATP binding / ATP-dependent peptidase/ nucleoside-triphosphatase/ nucleotide binding / serine-type endopeptidase | chr1:27511722-27514395 REVERSEhighly similar to ( 649) loc_os06g06190 12006.m05345 protein expressed protein  no original description 	T
20.2.3	stress.abiotic.drought/salt	MDP0000339601	 moderately similar to ( 292) AT3G51070 | Symbols:  | dehydration-responsive protein-related | chr3:18969068-18972291 FORWARDmoderately similar to ( 279) loc_os01g66110 12001.m42777 protein ankyrin-like protein, putative, expressed no original description 	T
26.10	misc.cytochrome P450	MDP0000180684	 moderately similar to ( 245) AT5G63450 | Symbols: CYP94B1 | CYP94B1; electron carrier/ heme binding / iron ion binding / monooxygenase/ oxygen binding | chr5:25408987-25410519 REVERSEvery weakly similar to (93.2) C82A1_PEA Cytochrome P450 82A1 (EC 1.14.-.-) (CYPLXXXII) (Fragment) - Pisum sativum (Garden pea)moderately similar to ( 233) loc_os01g58960 12001.m12035 protein cytochrome P450 94A1, putative, expressed    no original description 	T
10.2.1	cell wall.cellulose synthesis.cellulose synthase	MDP0000292881	 nearly identical (1910) AT4G32410 | Symbols: CESA1, RSW1 | CESA1 (CELLULOSE SYNTHASE 1); cellulose synthase/ transferase, transferring glycosyl groups | chr4:15641009-15646388 REVERSEnearly identical (1844) loc_os05g08370 12005.m05354 protein CESA1 - cellulose synthase, expressed   no original description 	T
35.2	not assigned.unknown	MDP0000333990	  no original description 	T
29.5.9	protein.degradation.AAA type	MDP0000300046	 moderately similar to ( 412) AT5G17760 | Symbols:  | AAA-type ATPase family protein | chr5:5861276-5862301 REVERSEvery weakly similar to (57.8) PRS4_ORYSA 26S protease regulatory subunit 4 homolog (TAT-binding protein homolog 2) - Oryza sativa (Rice)moderately similar to ( 353) loc_os05g51130 12005.m09179 protein mitochondrial chaperone BCS1, putative, expressed   no original description 	T
26.2	misc.UDP glucosyl and glucoronyl transferases	MDP0000132973	 highly similar to ( 897) AT3G25140 | Symbols: GAUT8, QUA1 | QUA1 (QUASIMODO 1); polygalacturonate 4-alpha-galacturonosyltransferase/ transferase, transferring glycosyl groups / transferase, transferring hexosyl groups | chr3:9154748-9156642 FORWARDhighly similar to ( 656) loc_os02g29530 12002.m08096 protein transferase, transferring glycosyl groups, putative, expressed   no original description 	T
35.2	not assigned.unknown	MDP0000277011	 moderately similar to ( 456) AT2G40550 | Symbols: ETG1 | ETG1 (E2F TARGET GENE 1) | chr2:16934927-16938033 FORWARDmoderately similar to ( 420) loc_os01g07260 12001.m07352 protein expressed protein  no original description 	T
35.2	not assigned.unknown	MDP0000146812	 very weakly similar to (92.8) AT1G68350 | Symbols:  | unknown protein | chr1:25616516-25617004 FORWARDvery weakly similar to (63.9) loc_os01g04170 12001.m07053 protein expressed protein  no original description 	T
11.2.4	lipid metabolism.FA desaturation.omega 6 desaturase	MDP0000281451	 moderately similar to ( 327) AT3G12120 | Symbols: FAD2 | FAD2 (FATTY ACID DESATURASE 2); delta12-fatty acid dehydrogenase/ omega-6 fatty acid desaturase | chr3:3860592-3861743 REVERSEmoderately similar to ( 345) FD6E2_SOYBN Omega-6 fatty acid desaturase, endoplasmic reticulum isozyme 2 (EC 1.14.19.-) - Glycine max (Soybean)moderately similar to ( 305) loc_os02g48560 12002.m100054 protein omega-6 fatty acid desaturase, endoplasmic reticulum isozyme 2, putative, expressed  no original description 	T
11.2.3	lipid metabolism.FA desaturation.omega 3 desaturase	MDP0000281451	 moderately similar to ( 327) AT3G12120 | Symbols: FAD2 | FAD2 (FATTY ACID DESATURASE 2); delta12-fatty acid dehydrogenase/ omega-6 fatty acid desaturase | chr3:3860592-3861743 REVERSEmoderately similar to ( 345) FD6E2_SOYBN Omega-6 fatty acid desaturase, endoplasmic reticulum isozyme 2 (EC 1.14.19.-) - Glycine max (Soybean)moderately similar to ( 305) loc_os02g48560 12002.m100054 protein omega-6 fatty acid desaturase, endoplasmic reticulum isozyme 2, putative, expressed  no original description 	T
20.2.99	stress.abiotic.unspecified	MDP0000222188	 weakly similar to ( 189) AT3G62550 | Symbols:  | universal stress protein (USP) family protein | chr3:23136194-23137074 FORWARDweakly similar to ( 148) loc_os01g19820 12001.m150564 protein universal stress protein, putative, expressed  no original description 	T
35.2	not assigned.unknown	MDP0000200077	   no original description 	T
10.7	cell wall.modification	MDP0000158544	 moderately similar to ( 432) AT4G14130 | Symbols: XTR7 | XTR7 (XYLOGLUCAN ENDOTRANSGLYCOSYLASE 7); hydrolase, acting on glycosyl bonds / hydrolase, hydrolyzing O-glycosyl compounds / xyloglucan:xyloglucosyl transferase | chr4:8137161-8138196 REVERSEmoderately similar to ( 473) BRU1_SOYBN Brassinosteroid-regulated protein BRU1 precursor - Glycine max (Soybean)moderately similar to ( 365) loc_os06g48200 12006.m09346 protein xyloglucan endotransglucosylase/hydrolase protein 23 precursor, putative, expressed no original description 	T
29.5.11.4.2	protein.degradation.ubiquitin.E3.RING	MDP0000185406	 moderately similar to ( 206) AT5G45100 | Symbols:  | protein binding / zinc ion binding | chr5:18218938-18219741 FORWARDweakly similar to ( 160) loc_os03g46570 12003.m09676 protein ubiquitin-protein ligase/ zinc ion binding protein, putative, expressed   no original description 	T
30.2.3	signalling.receptor kinases.leucine rich repeat III	MDP0000577872	 highly similar to ( 691) AT1G48480 | Symbols: RKL1 | RKL1; ATP binding / kinase/ protein serine/threonine kinase | chr1:17918475-17920743 FORWARDweakly similar to ( 175) PSKR_DAUCA Phytosulfokine receptor precursor (EC 2.7.11.1) (Phytosulfokine LRR receptor kinase) - Daucus carota (Carrot)highly similar to ( 608) loc_os03g12250 12003.m06675 protein atypical receptor-like kinase MARK, putative, expressed   no original description 	T
30.3	signalling.calcium	MDP0000361567	 moderately similar to ( 298) AT3G43810 | Symbols: CAM7 | CAM7 (CALMODULIN 7); calcium ion binding | chr3:15664619-15666355 REVERSEmoderately similar to ( 296) CALM_LILLO Calmodulin (CaM) - Lilium longiflorum (Trumpet lily)moderately similar to ( 296) loc_os07g48780 12007.m29366 protein calmodulin, putative, expressed  no original description 	T
26.2	misc.UDP glucosyl and glucoronyl transferases	MDP0000120125	 moderately similar to ( 470) AT3G28340 | Symbols: GATL10 | GATL10 (Galacturonosyltransferase-like 10); polygalacturonate 4-alpha-galacturonosyltransferase/ transferase, transferring hexosyl groups | chr3:10589396-10590493 REVERSEmoderately similar to ( 426) loc_os03g18890 12003.m07309 protein transferase, transferring glycosyl groups, putative, expressed  no original description 	T
30.2.11	signalling.receptor kinases.leucine rich repeat XI	MDP0000163007	 moderately similar to ( 462) AT3G25670 | Symbols:  | leucine-rich repeat family protein | chr3:9344532-9346301 REVERSEweakly similar to ( 134) RPK1_IPONI Receptor-like protein kinase precursor (EC 2.7.11.1) - Ipomoea nil (Japanese morning glory) (Pharbitis nil)moderately similar to ( 448) loc_os01g02060 12001.m06848 protein too many mouths protein precursor, putative, expressed  no original description 	T
27.3.8	RNA.regulation of transcription.C2C2(Zn) DOF zinc finger family	MDP0000241840	 weakly similar to ( 137) AT5G60850 | Symbols: OBP4 | OBP4; DNA binding / transcription factor | chr5:24480578-24481501 FORWARDweakly similar to ( 115) PBF_MAIZE Dof zinc finger protein PBF (Prolamin box-binding factor) - Zea mays (Maize)weakly similar to ( 129) loc_os02g49440 12002.m09978 protein expressed protein  no original description 	T
29.4.1.57	protein.postranslational modification.kinase.receptor like cytoplasmatic kinase VII	MDP0000874088	 highly similar to ( 786) AT1G34210 | Symbols: SERK2, ATSERK2 | SERK2 (SOMATIC EMBRYOGENESIS RECEPTOR-LIKE KINASE 2); kinase | chr1:12459078-12462752 FORWARDmoderately similar to ( 226) PSKR_DAUCA Phytosulfokine receptor precursor (EC 2.7.11.1) (Phytosulfokine LRR receptor kinase) - Daucus carota (Carrot)highly similar to ( 764) loc_os08g07760 12008.m04913 protein BRASSINOSTEROID INSENSITIVE 1-associated receptor kinase 1 precursor, putative, expressed   no original description 	T
30.2.17	signalling.receptor kinases.DUF 26	MDP0000173609	 moderately similar to ( 264) AT1G66920 | Symbols:  | serine/threonine protein kinase, putative | chr1:24965410-24967432 REVERSEweakly similar to ( 144) KPRO_MAIZE Putative receptor protein kinase ZmPK1 precursor (EC 2.7.11.1) - Zea mays (Maize)moderately similar to ( 296) loc_os01g02560 12001.m06898 protein Ser/Thr receptor-like kinase, putative, expressed no original description 	T
29.4	protein.postranslational modification	MDP0000173609	 moderately similar to ( 264) AT1G66920 | Symbols:  | serine/threonine protein kinase, putative | chr1:24965410-24967432 REVERSEweakly similar to ( 144) KPRO_MAIZE Putative receptor protein kinase ZmPK1 precursor (EC 2.7.11.1) - Zea mays (Maize)moderately similar to ( 296) loc_os01g02560 12001.m06898 protein Ser/Thr receptor-like kinase, putative, expressed no original description 	T
29.8	protein.assembly and cofactor ligation	MDP0000319416	 highly similar to ( 685) AT3G24430 | Symbols: HCF101 | HCF101 (HIGH-CHLOROPHYLL-FLUORESCENCE 101); ATP binding | chr3:8868731-8872154 REVERSEvery weakly similar to (53.9) MIND_MESVI Putative septum site-determining protein minD - Mesostigma viridehighly similar to ( 674) loc_os01g52170 12001.m11391 protein HCF101, putative, expressed no original description 	T
11.3.3	lipid metabolism.Phospholipid synthesis.phosphatidate cytidylyltransferase	MDP0000290660	 highly similar to ( 696) AT4G22340 | Symbols:  | phosphatidate cytidylyltransferase, putative / CDP-diglyceride synthetase, putative | chr4:11800150-11802621 REVERSEhighly similar to ( 705) CDS1_SOLTU Phosphatidate cytidylyltransferase (EC 2.7.7.41) (CDP-diglyceride synthetase) (CDP-diglyceride pyrophosphorylase) (CDP-diacylglycerol synthase) (CDS) (CTP:phosphatidate cytidylyltransferase) (CDP-DAG synthase) (CDP-DG synthetase) - Solahighly similar to ( 662) loc_os01g55360 12001.m43197 protein phosphatidate cytidylyltransferase, putative, expressed  no original description 	T
27.3.67	RNA.regulation of transcription.putative transcription regulator	MDP0000477220	 moderately similar to ( 214) AT2G33620 | Symbols:  | DNA-binding family protein / AT-hook protein 1 (AHP1) | chr2:14234749-14236563 FORWARDmoderately similar to ( 230) loc_os08g40150 12008.m26527 protein AT-hook protein 1, putative, expressed   no original description 	T
35.2	not assigned.unknown	MDP0000444739	 moderately similar to ( 222) AT3G52220 | Symbols:  | unknown protein | chr3:19369580-19370980 REVERSEmoderately similar to ( 215) loc_os08g44440 12008.m08417 protein multiple myeloma tumor-associated protein 2, putative, expressed  no original description 	T
20.1	stress.biotic	MDP0000752582	 moderately similar to ( 477) AT3G14470 | Symbols:  | disease resistance protein (NBS-LRR class), putative | chr3:4857940-4861104 FORWARDmoderately similar to ( 398) loc_os05g41310 12005.m08306 protein disease resistance protein RGA2, putative, expressed   no original description 	T
35.2	not assigned.unknown	MDP0000271797	 highly similar to ( 608) AT2G25270 | Symbols:  | unknown protein | chr2:10759779-10762358 FORWARDhighly similar to ( 551) loc_os02g55590 12002.m10585 protein expressed protein  no original description 	T
29.2.4	protein.synthesis.elongation	MDP0000198451	 highly similar to ( 856) AT5G60390 | Symbols:  | elongation factor 1-alpha / EF-1-alpha | chr5:24289226-24290675 FORWARDhighly similar to ( 858) EF1A_MAIZE Elongation factor 1-alpha (EF-1-alpha) - Zea mays (Maize)highly similar to ( 857) loc_os03g08050 12003.m78808 protein elongation factor 1-alpha, putative, expressed   no original description 	T
28.1.3	DNA.synthesis/chromatin structure.histone	MDP0000161230	 moderately similar to ( 266) AT5G10980 | Symbols:  | histone H3 | chr5:3472591-3473349 REVERSEmoderately similar to ( 264) H33_VITVI Histone H3.3 - Vitis vinifera (Grape)moderately similar to ( 266) loc_os06g04030 12006.m05134 protein histone H3, putative, expressed no original description 	T
35.2	not assigned.unknown	MDP0000256566	 moderately similar to ( 399) AT5G50290 | Symbols:  | unknown protein | chr5:20461814-20463896 FORWARDmoderately similar to ( 341) loc_os05g33860 12005.m07618 protein conserved hypothetical protein   no original description 	T
29.4.1.57	protein.postranslational modification.kinase.receptor like cytoplasmatic kinase VII	MDP0000294124	 highly similar to ( 888) AT2G20300 | Symbols: ALE2 | ALE2 (Abnormal Leaf Shape 2); kinase | chr2:8756475-8759845 REVERSEmoderately similar to ( 242) NORK_MEDTR Nodulation receptor kinase precursor (EC 2.7.11.1) (Does not make infections protein 2) (Symbiosis receptor-like kinase) (MtSYMRK) - Medicago truncatula (Barrel medic)highly similar to ( 654) loc_os02g57080 12002.m33474 protein ATP binding protein, putative, expressed  no original description 	T
35.2	not assigned.unknown	MDP0000136717	 weakly similar to ( 169) AT2G06010 | Symbols: ORG4 | ORG4 (OBP3-RESPONSIVE GENE 4) | chr2:2336841-2338391 FORWARDweakly similar to ( 146) loc_os04g36850 12004.m08725 protein expressed protein    no original description 	T
33.99	development.unspecified	MDP0000141898	 highly similar to ( 514) AT2G46280 | Symbols: TRIP-1, TIF3I1 | TRIP-1 (TGF-BETA RECEPTOR INTERACTING PROTEIN 1); nucleotide binding / protein binding | chr2:19003656-19005393 REVERSEvery weakly similar to (65.9) PF20_CHLRE Flagellar WD repeat protein PF20 - Chlamydomonas reinhardtiihighly similar to ( 506) loc_os08g21660 12008.m06205 protein eukaryotic translation initiation factor 3 subunit 2, putative, expressed  no original description 	T
35.2	not assigned.unknown	MDP0000328107	  no original description 	T
29.4.1.57	protein.postranslational modification.kinase.receptor like cytoplasmatic kinase VII	MDP0000213799	 moderately similar to ( 244) AT1G21590 | Symbols:  | protein kinase family protein | chr1:7566613-7569694 REVERSEweakly similar to ( 164) NORK_PEA Nodulation receptor kinase precursor (EC 2.7.11.1) - Pisum sativum (Garden pea)moderately similar to ( 228) loc_os03g13820 12003.m101264 protein ATP binding protein, putative, expressed   no original description 	T
35.1.1	not assigned.no ontology.ABC1 family protein	MDP0000313744	 highly similar to ( 808) AT2G39190 | Symbols: ATATH8 | ATATH8; transporter | chr2:16350140-16355745 FORWARDhighly similar to ( 684) loc_os02g56200 12002.m33927 protein ATATH8, putative, expressed  no original description 	T
15.2	metal handling.binding, chelation and storage	MDP0000296387	 moderately similar to ( 254) AT4G38580 | Symbols: ATFP6, HIPP26 | ATFP6 (FARNESYLATED PROTEIN 6); metal ion binding | chr4:18034596-18035693 FORWARDmoderately similar to ( 205) loc_os04g17100 12004.m35260 protein farnesylated protein 1, putative, expressed   no original description 	T
29.2.1.99.99	protein.synthesis.ribosomal protein.unknown.unknown	MDP0000184668	 weakly similar to ( 118) AT1G16790 | Symbols:  | ribosomal protein-related | chr1:5744638-5745072 FORWARD   no original description 	T
35.2	not assigned.unknown	MDP0000362327	  no original description 	T
35.2	not assigned.unknown	MDP0000249527	 weakly similar to ( 189) AT3G15660 | Symbols: ATGRX4, GRX4 | GRX4 (GLUTAREDOXIN 4); metal ion binding | chr3:5308134-5309383 REVERSEweakly similar to ( 186) loc_os01g42700 12001.m43089 protein expressed protein  no original description 	T
31.1	cell.organisation	MDP0000135735	 moderately similar to ( 454) AT2G37080 | Symbols:  | myosin heavy chain-related | chr2:15581565-15584057 REVERSEvery weakly similar to (56.6) MFP1_TOBAC MAR-binding filament-like protein 1-1 - Nicotiana tabacum (Common tobacco)moderately similar to ( 393) loc_os05g03120 12005.m27530 protein myosin heavy chain-like protein, putative, expressed  no original description 	T
31.1	cell.organisation	MDP0000319081	 highly similar to ( 827) AT1G75780 | Symbols: TUB1 | TUB1; GTP binding / GTPase/ structural molecule | chr1:28451378-28453602 REVERSEhighly similar to ( 831) TBB6_GOSHI Tubulin beta-6 chain (Beta-6 tubulin) - Gossypium hirsutum (Upland cotton)highly similar to ( 827) loc_os03g56810 12003.m35436 protein tubulin beta-7 chain, putative, expressed  no original description 	T
26.2	misc.UDP glucosyl and glucoronyl transferases	MDP0000243946	 highly similar to ( 815) AT5G47780 | Symbols: GAUT4 | GAUT4 (Galacturonosyltransferase 4); polygalacturonate 4-alpha-galacturonosyltransferase/ transferase, transferring glycosyl groups | chr5:19347991-19350517 FORWARDhighly similar to ( 755) loc_os08g23780 12008.m06398 protein transferase, transferring glycosyl groups, putative, expressed  no original description 	T
20.2.1	stress.abiotic.heat	MDP0000867730	 weakly similar to ( 138) AT5G02490 | Symbols:  | heat shock cognate 70 kDa protein 2 (HSC70-2) (HSP70-2) | chr5:550296-552565 REVERSEweakly similar to ( 138) HSP7C_PETHY Heat shock cognate 70 kDa protein - Petunia hybrida (Petunia)weakly similar to ( 137) loc_os03g16860 12003.m101294 protein heat shock cognate 70 kDa protein 2, putative, expressed  no original description 	T
35.2	not assigned.unknown	MDP0000887072	  no original description 	T
29.5.11.4.2	protein.degradation.ubiquitin.E3.RING	MDP0000244758	 moderately similar to ( 385) AT2G37150 | Symbols:  | zinc finger (C3HC4-type RING finger) family protein | chr2:15603748-15605988 REVERSEvery weakly similar to (54.7) EL5_ORYSA E3 ubiquitin-protein ligase EL5 (EC 6.3.2.-) - Oryza sativa (Rice)weakly similar to ( 176) loc_os05g07070 12005.m083625 protein zinc finger, C3HC4 type family protein, expressed no original description 	T
35.2	not assigned.unknown	MDP0000302736	 weakly similar to ( 173) AT2G04039 | Symbols:  | unknown protein | chr2:1333464-1334457 FORWARD no original description 	T
34.99	transport.misc	MDP0000157345	 highly similar to ( 715) AT3G59030 | Symbols: TT12, ATTT12 | TT12 (TRANSPARENT TESTA 12); antiporter/ solute:hydrogen antiporter/ transmembrane transporter/ transporter | chr3:21819124-21821288 FORWARDhighly similar to ( 583) loc_os12g42130 12012.m07992 protein transparent testa 12 protein, putative, expressed  no original description 	T
10.5.5	cell wall.cell wall proteins.RGP	MDP0000232047	 highly similar to ( 666) AT3G08900 | Symbols: RGP3, RGP | RGP3 (REVERSIBLY GLYCOSYLATED POLYPEPTIDE 3); transferase, transferring hexosyl groups | chr3:2708347-2709714 REVERSEhighly similar to ( 681) UPTG_PEA Alpha-1,4-glucan-protein synthase [UDP-forming] (EC 2.4.1.112) (UDP-glucose:protein transglucosylase) (UPTG) (Reversibly glycosylated polypeptide) - Pisum sativum (Garden pea)highly similar to ( 673) loc_os07g41360 12007.m08372 protein alpha-1,4-glucan-protein synthase 1, putative, expressed   no original description 	T
26.28	misc.GDSL-motif lipase	MDP0000149267	 highly similar to ( 513) AT5G33370 | Symbols:  | GDSL-motif lipase/hydrolase family protein | chr5:12602864-12604670 REVERSEweakly similar to ( 164) APG_BRANA Anter-specific proline-rich protein APG (Protein CEX) (Fragment) - Brassica napus (Rape)moderately similar to ( 489) loc_os02g40440 12002.m09130 protein anther-specific proline-rich protein APG precursor, putative, expressed  no original description 	T
35.1	not assigned.no ontology	MDP0000257454	 weakly similar to ( 186) AT3G11780 | Symbols:  | MD-2-related lipid recognition domain-containing protein / ML domain-containing protein | chr3:3724326-3725476 REVERSEweakly similar to ( 139) loc_os03g57420 12003.m10660 protein ML domain protein, putative, expressed no original description 	T
10.7	cell wall.modification	MDP0000772420	 moderately similar to ( 295) AT2G40610 | Symbols: ATEXPA8, EXP8, ATEXP8, ATHEXP ALPHA 1.11 | ATEXPA8 (ARABIDOPSIS THALIANA EXPANSIN A8) | chr2:16949121-16950472 REVERSEmoderately similar to ( 247) EXPA6_ORYSA Expansin-A6 precursor (OsEXPA6) (Alpha-expansin-6) (OsEXP6) (OsaEXPa1.24) - Oryza sativa (Rice)moderately similar to ( 247) loc_os03g21820 12003.m07586 protein alpha-expansin 10 precursor, putative, expressed  no original description 	T
21.1	redox.thioredoxin	MDP0000303018	 weakly similar to ( 166) AT1G11530 | Symbols: ATCXXS1 | ATCXXS1 (C-terminal cysteine residue is changed to a serine 1); protein disulfide isomerase | chr1:3874518-3875311 FORWARDvery weakly similar to (88.2) TRXH2_TOBAC Thioredoxin H-type 2 (TRX-H2) - Nicotiana tabacum (Common tobacco)weakly similar to ( 129) loc_os04g53740 12004.m35046 protein thioredoxin-like 4, putative, expressed no original description 	T
29.5.11.20	protein.degradation.ubiquitin.proteasom	MDP0000219591	 highly similar to ( 655) AT5G09900 | Symbols: EMB2107, RPN5A, MSA | EMB2107 (EMBRYO DEFECTIVE 2107) | chr5:3089406-3092434 REVERSEhighly similar to ( 675) loc_os03g63430 12003.m101139 protein 26S proteasome non-ATPase regulatory subunit 12, putative, expressed   no original description 	T
30.2.24	signalling.receptor kinases.S-locus glycoprotein like	MDP0000817734	 highly similar to ( 542) AT1G78830 | Symbols:  | curculin-like (mannose-binding) lectin family protein | chr1:29637141-29638508 REVERSEmoderately similar to ( 278) EP1G_DAUCA Epidermis-specific secreted glycoprotein EP1 precursor (52/54 kDa medium protein) - Daucus carota (Carrot)weakly similar to ( 132) loc_os04g23640 12004.m07505 protein ATP binding protein, putative, expressed  no original description 	T
30.6	signalling.MAP kinases	MDP0000783234	 moderately similar to ( 303) AT3G18040 | Symbols: MPK9 | MPK9 (MAP KINASE 9); MAP kinase | chr3:6175741-6178150 FORWARDmoderately similar to ( 265) MPK12_ORYSA Mitogen-activated protein kinase 12 (EC 2.7.11.24) (MAP kinase 12) (OsBWMK1) (Blast- and wound-induced MAP kinase 1) (MAP kinase 1) (OsMAPK1) - Oryza sativa (Rice)moderately similar to ( 265) loc_os06g49430 12006.m31928 protein OsMPK17-1 - putative MAPK based on amino acid sequence homology, expressed  no original description 	T
1.1.40	PS.lightreaction.cyclic electron flow-chlororespiration	MDP0000236916	 weakly similar to ( 163) AT2G05620 | Symbols: PGR5 | PGR5 (proton gradient regulation 5); electron carrier | chr2:2081204-2081687 REVERSEweakly similar to ( 140) loc_os08g45190 12008.m08489 protein PGR5, putative, expressed no original description 	T
29.5.11.4.3.2	protein.degradation.ubiquitin.E3.SCF.FBOX	MDP0000808501	 very weakly similar to (90.9) AT5G55150 | Symbols:  | F-box family protein | chr5:22382275-22383392 REVERSEvery weakly similar to (92.8) loc_os06g35080 12006.m08045 protein F-box domain containing protein, expressed  no original description 	T
26.9	misc.glutathione S transferases	MDP0000189838	 moderately similar to ( 294) AT3G55040 | Symbols: GSTL2 | GSTL2 | chr3:20398718-20400305 REVERSEmoderately similar to ( 242) IN21_MAIZE IN2-1 protein - Zea mays (Maize)moderately similar to ( 310) loc_os03g17470 12003.m07174 protein IN2-1 protein, putative, expressed no original description 	T
34.1	transport.p- and v-ATPases	MDP0000472684	 highly similar to ( 896) AT1G17500 | Symbols:  | ATPase, coupled to transmembrane movement of ions, phosphorylative mechanism | chr1:6018757-6023201 FORWARDvery weakly similar to (53.1) ACA2_ORYSA Calcium-transporting ATPase 2, plasma membrane-type (EC 3.6.3.8) (Ca(2+)-ATPase isoform 2) - Oryza sativa (Rice)highly similar to ( 892) loc_os06g36990 12006.m08234 protein phospholipid-transporting ATPase 4, putative, expressed   no original description 	T
20.2.99	stress.abiotic.unspecified	MDP0000409061	 very weakly similar to (78.6) AT4G08685 | Symbols: SAH7 | SAH7 | chr4:5550563-5551259 FORWARDvery weakly similar to (53.1) PSC13_MAIZE Pollen-specific protein C13 precursor - Zea mays (Maize)very weakly similar to (54.3) loc_os10g22590 12010.m05266 protein pollen-specific protein C13 precursor, putative, expressedMSP1_C  no original description 	T
29.5.11.4.3.2	protein.degradation.ubiquitin.E3.SCF.FBOX	MDP0000256433	 moderately similar to ( 341) AT4G10925 | Symbols:  | F-box family protein | chr4:6702987-6704283 REVERSEmoderately similar to ( 311) loc_os01g36940 12001.m09990 protein F-box domain containing protein, expressed  no original description 	T
34.1	transport.p- and v-ATPases	MDP0000230812	 nearly identical (1551) AT5G44240 | Symbols:  | haloacid dehalogenase-like hydrolase family protein | chr5:17817619-17823598 FORWARDvery weakly similar to (90.5) ACA6_ORYSA Probable calcium-transporting ATPase 6, plasma membrane-type (EC 3.6.3.8) (Ca(2+)-ATPase isoform 6) - Oryza sativa (Rice)nearly identical (1067) loc_os11g25980 12011.m06519 protein phospholipid-transporting ATPase 2, putative, expressed  no original description 	T
20.2.99	stress.abiotic.unspecified	MDP0000218759	 weakly similar to ( 109) AT4G08685 | Symbols: SAH7 | SAH7 | chr4:5550563-5551259 FORWARDvery weakly similar to (77.0) OLEE1_BETVE Olee1-like protein precursor - Betula verrucosa (White birch) (Betula pendula)very weakly similar to (94.7) loc_os10g22590 12010.m05266 protein pollen-specific protein C13 precursor, putative, expressed  no original description 	T
35.2	not assigned.unknown	MDP0000272880	 very weakly similar to (75.9) AT3G14280 | Symbols:  | unknown protein | chr3:4761654-4762286 REVERSEvery weakly similar to (58.9) loc_os06g09350 12006.m32003 protein expressed protein  no original description 	T
29.7	protein.glycosylation	MDP0000365996	 weakly similar to ( 183) AT2G01720 | Symbols:  | ribophorin I family protein | chr2:317193-320016 REVERSEweakly similar to ( 141) loc_os05g23600 12005.m27570 protein dolichyl-diphosphooligosaccharide--protein glycosyltransferase 67 kDasubunit precursor, putative, expressed no original description 	T
29.2.1.2.1.20	protein.synthesis.ribosomal protein.eukaryotic.40S subunit.S20	MDP0000361260	 moderately similar to ( 228) AT3G47370 | Symbols:  | 40S ribosomal protein S20 (RPS20B) | chr3:17453671-17454437 REVERSEweakly similar to ( 194) RS20_ORYSA 40S ribosomal protein S20 - Oryza sativa (Rice)weakly similar to ( 199) loc_os10g08930 12010.m04240 protein 40S ribosomal protein S20, putative, expressed  no original description 	T
17.7.1.2	hormone metabolism.jasmonate.synthesis-degradation.lipoxygenase	MDP0000923670	 nearly identical (1155) AT1G55020 | Symbols: LOX1 | LOX1; lipoxygenase | chr1:20525798-20530143 FORWARDnearly identical (1164) LOX1_SOLTU Lipoxygenase 1 (EC 1.13.11.12) - Solanum tuberosum (Potato)highly similar to ( 962) loc_os03g49380 12003.m35371 protein lipoxygenase 4, putative, expressed  no original description 	T
35.1	not assigned.no ontology	MDP0000246491	 moderately similar to ( 214) AT3G60600 | Symbols: VAP27-1 | VAP (VESICLE ASSOCIATED PROTEIN); protein binding | chr3:22400537-22401669 FORWARDmoderately similar to ( 209) loc_os08g06020 12008.m04743 protein VAP27, putative, expressed no original description 	T
16.7	secondary metabolism.wax	MDP0000309240	 moderately similar to ( 269) AT1G02205 | Symbols: CER1 | CER1 (ECERIFERUM 1); octadecanal decarbonylase | chr1:418818-422154 FORWARDmoderately similar to ( 269) loc_os02g40784 12002.m34021 protein CER1, putative, expressed   no original description 	T
35.1	not assigned.no ontology	MDP0000156202	 weakly similar to ( 129) AT1G06920 | Symbols: ATOFP4, OFP4 | OFP4 (ARABIDOPSIS THALIANA OVATE FAMILY PROTEIN 4) | chr1:2124854-2125801 REVERSEweakly similar to ( 159) loc_os01g60810 12001.m12209 protein plant-specific domain TIGR01568 family protein, expressed  no original description 	T
35.1.5	not assigned.no ontology.pentatricopeptide (PPR) repeat-containing protein	MDP0000301848	 highly similar to ( 664) AT1G60770 | Symbols:  | pentatricopeptide (PPR) repeat-containing protein | chr1:22366959-22368648 REVERSEvery weakly similar to (69.7) RF1_ORYSA Rf1 protein, mitochondrial precursor (PPR protein) (Fertility restorer) (Restorer for CMS) - Oryza sativa (Rice)highly similar to ( 544) loc_os04g25410 12004.m07669 protein tetratricopeptide-like helical, putative, expressed   no original description 	T
33.99	development.unspecified	MDP0000190738	 moderately similar to ( 232) AT1G10200 | Symbols: WLIM1 | WLIM1; transcription factor/ zinc ion binding | chr1:3346677-3347763 REVERSEmoderately similar to ( 225) SF3_HELAN Pollen-specific protein SF3 - Helianthus annuus (Common sunflower)moderately similar to ( 235) loc_os12g32620 12012.m07062 protein pollen-specific protein SF3, putative, expressed no original description 	T
23.4.1	nucleotide metabolism.phosphotransfer and pyrophosphatases.adenylate kinase	MDP0000318499	 moderately similar to ( 394) AT5G63400 | Symbols: ADK1 | ADK1 (ADENYLATE KINASE 1); ATP binding / adenylate kinase/ nucleobase, nucleoside, nucleotide kinase | chr5:25393502-25394817 REVERSEmoderately similar to ( 424) KAD2_ORYSA Adenylate kinase B (EC 2.7.4.3) (ATP-AMP transphosphorylase) - Oryza sativa (Rice)moderately similar to ( 424) loc_os11g20790 12011.m06153 protein adenylate kinase B, putative, expressed  no original description 	T
27.3.29	RNA.regulation of transcription.TCP transcription factor family	MDP0000243495	 moderately similar to ( 235) AT3G15030 | Symbols: TCP4 | TCP4 (TCP family transcription factor 4); transcription factor | chr3:5062308-5063570 FORWARDmoderately similar to ( 202) loc_os01g11550 12001.m07772 protein mutant cincinnata, putative, expressed  no original description 	T
7.2.2	OPP.non-reductive PP.transaldolase	MDP0000316685	 highly similar to ( 627) AT5G13420 | Symbols:  | transaldolase, putative | chr5:4302080-4304212 REVERSEhighly similar to ( 629) loc_os01g70170 12001.m13065 protein transaldolase 2, putative, expressed   no original description 	T
10.6.3	cell wall.degradation.pectate lyases and polygalacturonases	MDP0000296416	 highly similar to ( 667) AT1G48100 | Symbols:  | glycoside hydrolase family 28 protein / polygalacturonase (pectinase) family protein | chr1:17766921-17769625 FORWARDmoderately similar to ( 284) PGLR_ACTCH Polygalacturonase precursor (EC 3.2.1.15) (PG) (Pectinase) - Actinidia chinensis (Kiwi) (Yangtao)highly similar to ( 601) loc_os05g20020 12005.m06402 protein polygalacturonase precursor, putative, expressed   no original description 	T
20.2.1	stress.abiotic.heat	MDP0000290189	 weakly similar to ( 112) AT5G59610 | Symbols:  | DNAJ heat shock N-terminal domain-containing protein | chr5:24013165-24014889 FORWARDvery weakly similar to (51.6) DNJH_CUCSA DnaJ protein homolog (DNAJ-1) - Cucumis sativus (Cucumber)weakly similar to ( 134) loc_os03g60790 12003.m10967 protein heat shock protein DnaJ, N-terminal, putative, expressed  no original description 	T
29.2.1.2.1.14	protein.synthesis.ribosomal protein.eukaryotic.40S subunit.S14	MDP0000163790	 moderately similar to ( 221) AT3G52580 | Symbols:  | 40S ribosomal protein S14 (RPS14C) | chr3:19503324-19504701 FORWARDmoderately similar to ( 219) RS142_MAIZE 40S ribosomal protein S14 (Clone MCH2) - Zea mays (Maize)moderately similar to ( 227) loc_os02g06700 12002.m06018 protein 40S ribosomal protein S14, putative, expressed no original description 	T
35.2	not assigned.unknown	MDP0000232978	 moderately similar to ( 352) AT5G16610 | Symbols:  | unknown protein | chr5:5445068-5447945 REVERSEmoderately similar to ( 318) loc_os03g12120 12003.m35099 protein no apical meristem protein, expressed   no original description 	T
27.3.7	RNA.regulation of transcription.C2C2(Zn) CO-like, Constans-like zinc finger family	MDP0000318891	 moderately similar to ( 216) AT5G57180 | Symbols: CIA2 | CIA2 (CHLOROPLAST IMPORT APPARATUS 2); transcription regulator | chr5:23168393-23169523 FORWARDweakly similar to ( 154) loc_os02g05470 12002.m05895 protein CCT motif family protein, expressed   no original description 	T
31.1	cell.organisation	MDP0000672731	 weakly similar to ( 126) AT1G10340 | Symbols:  | ankyrin repeat family protein | chr1:3390475-3392481 REVERSEvery weakly similar to ( 100) loc_os09g16550 12009.m04915 protein protein binding protein, putative, expressed  no original description 	T
9.1.2	mitochondrial electron transport / ATP synthesis.NADH-DH.localisation not clear	MDP0000666211	 weakly similar to ( 180) AT5G18800 | Symbols:  | NADH-ubiquinone oxidoreductase 19 kDa subunit (NDUFA8) family protein | chr5:6267304-6268393 FORWARDweakly similar to ( 166) loc_os03g18420 12003.m07265 protein NADH-ubiquinone oxidoreductase 19 kDa subunit, putative, expressed no original description 	T
34.3	transport.amino acids	MDP0000132207	 highly similar to ( 526) AT1G47670 | Symbols:  | amino acid transporter family protein | chr1:17536834-17539486 REVERSEvery weakly similar to (59.7) LAX5_MEDTR Auxin transporter-like protein 5 (AUX1-like protein 5) (MtLAX5) - Medicago truncatula (Barrel medic)highly similar to ( 528) loc_os04g47420 12004.m09687 protein expressed protein   no original description 	T
28.1	DNA.synthesis/chromatin structure	MDP0000142758	 highly similar to ( 813) AT2G16440 | Symbols: MCM4 | DNA replication licensing factor, putative | chr2:7126536-7130665 REVERSEmoderately similar to ( 295) MCM3_MAIZE DNA replication licensing factor MCM3 homolog (Replication origin activator) (ROA protein) (Fragment) - Zea mays (Maize)highly similar to ( 811) loc_os01g36390 12001.m09934 protein DNA replication licensing factor mcm4, putative, expressed  no original description 	T
29.2.4	protein.synthesis.elongation	MDP0000517914	 moderately similar to ( 248) AT3G08740 | Symbols:  | elongation factor P (EF-P) family protein | chr3:2654788-2656154 REVERSEmoderately similar to ( 246) loc_os11g02450 12011.m04444 protein elongation factor P, putative, expressed no original description 	T
11.8.1.3	lipid metabolism.''exotics'' (steroids, squalene etc).sphingolipids.ceramide glucosyltransferase	MDP0000035240	 highly similar to ( 809) AT2G19880 | Symbols:  | ceramide glucosyltransferase, putative | chr2:8581578-8585118 FORWARDhighly similar to ( 708) loc_os01g67870 12001.m12891 protein ceramide glucosyltransferase, putative, expressed no original description 	T
35.1	not assigned.no ontology	MDP0000156058	 moderately similar to ( 449) AT1G74300 | Symbols:  | esterase/lipase/thioesterase family protein | chr1:27935368-27936474 FORWARDmoderately similar to ( 412) loc_os02g32970 12002.m08390 protein catalytic/ hydrolase, putative, expressed   no original description 	T
29.4	protein.postranslational modification	MDP0000250399	 highly similar to ( 805) AT1G16900 | Symbols:  | sugar binding / transferase, transferring glycosyl groups | chr1:5779262-5782303 REVERSEhighly similar to ( 747) loc_os01g11070 12001.m07727 protein alpha-1,2-mannosyltransferase ALG9, putative, expressed   no original description 	T
26.10	misc.cytochrome P450	MDP0000225698	 highly similar to ( 515) AT2G30490 | Symbols: ATC4H, C4H, CYP73A5 | C4H (CINNAMATE-4-HYDROXYLASE); trans-cinnamate 4-monooxygenase | chr2:12993861-12995683 REVERSEhighly similar to ( 541) TCMO_PHAAU Trans-cinnamate 4-monooxygenase (EC 1.14.13.11) (Cinnamic acid 4-hydroxylase) (CA4H) (C4H) (P450C4H) (Cytochrome P450 73) - Phaseolus aureus (Mung bean) (Vigna radiata)moderately similar to ( 459) loc_os01g60450 12001.m12173 protein trans-cinnamate 4-monooxygenase, putative   no original description 	T
21.1	redox.thioredoxin	MDP0000203322	 moderately similar to ( 234) AT2G04700 | Symbols:  | ferredoxin thioredoxin reductase catalytic beta chain family protein | chr2:1646961-1648345 FORWARDmoderately similar to ( 246) FTRC_SOYBN Ferredoxin-thioredoxin reductase catalytic chain, chloroplast precursor (EC 1.18.-.-) (FTR-C) - Glycine max (Soybean)moderately similar to ( 202) loc_os09g07570 12009.m059937 protein ferredoxin-thioredoxin reductase catalytic chain, chloroplast precursor, putative, expressed  no original description 	T
35.2	not assigned.unknown	MDP0000334726	  no original description 	T
35.2	not assigned.unknown	MDP0000803096	 very weakly similar to (91.7) AT5G59050 | Symbols:  | unknown protein | chr5:23841849-23842519 REVERSEvery weakly similar to (58.2) loc_os01g63050 12001.m12423 protein expressed protein  no original description 	T
34.3	transport.amino acids	MDP0000790376	 highly similar to ( 771) AT1G77380 | Symbols: AAP3, ATAAP3 | AAP3; amino acid transmembrane transporter | chr1:29075201-29077252 REVERSEhighly similar to ( 695) loc_os05g34980 12005.m07728 protein amino acid carrier, putative, expressed  no original description 	T
34.9	transport.metabolite transporters at the mitochondrial membrane	MDP0000222932	 moderately similar to ( 469) AT3G20240 | Symbols:  | mitochondrial substrate carrier family protein | chr3:7057192-7058716 FORWARDmoderately similar to ( 206) BT1_MAIZE Protein brittle-1, chloroplast precursor - Zea mays (Maize)moderately similar to ( 466) loc_os09g32200 12009.m06309 protein protein brittle-1, chloroplast precursor, putative, expressed no original description 	T
29.5.3	protein.degradation.cysteine protease	MDP0000231666	 highly similar to ( 601) AT1G47128 | Symbols: RD21, RD21A | RD21 (responsive to dehydration 21); cysteine-type endopeptidase/ cysteine-type peptidase | chr1:17283139-17285609 REVERSEhighly similar to ( 571) ORYA_ORYSA Oryzain alpha chain precursor (EC 3.4.22.-) - Oryza sativa (Rice)moderately similar to ( 477) loc_os04g57440 12004.m101443 protein cysteine protease 1 precursor, putative, expressed  no original description 	T
29.5.11.4.2	protein.degradation.ubiquitin.E3.RING	MDP0000485736	 weakly similar to ( 148) AT1G67856 | Symbols:  | protein binding / zinc ion binding | chr1:25442486-25442887 FORWARDvery weakly similar to (80.9) loc_os08g38460 12008.m07830 protein RING-H2 finger protein, putative, expressed no original description 	T
27.3.69	RNA.regulation of transcription.SET-domain transcriptional regulator family	MDP0000321249	 highly similar to ( 641) AT1G26760 | Symbols: ATXR1, SDG35 | SDG35 (SET DOMAIN PROTEIN 35); binding | chr1:9248304-9249941 REVERSEmoderately similar to ( 488) loc_os04g40830 12004.m35345 protein APC2, putative, expressed    no original description 	T
31.1	cell.organisation	MDP0000321157	 highly similar to ( 823) AT5G23860 | Symbols: TUB8 | TUB8; structural constituent of cytoskeleton | chr5:8042962-8044528 FORWARDhighly similar to ( 822) TBB2_ORYSA Tubulin beta-2 chain (Beta-2 tubulin) - Oryza sativa (Rice)highly similar to ( 822) loc_os03g01530 12003.m05700 protein tubulin beta-2 chain, putative, expressed   no original description 	T
29.5	protein.degradation	MDP0000295860	 moderately similar to ( 486) AT1G78680 | Symbols: ATGGH2 | ATGGH2 (gamma-glutamyl hydrolase 2); omega peptidase | chr1:29593933-29596037 FORWARDmoderately similar to ( 368) GGH_SOYBN Gamma-glutamyl hydrolase precursor (EC 3.4.19.9) (Gamma-Glu-X carboxypeptidase) (Conjugase) (GH) - Glycine max (Soybean)moderately similar to ( 400) loc_os05g44130 12005.m27626 protein gamma-glutamyl hydrolase precursor, putative, expressed   no original description 	T
35.2	not assigned.unknown	MDP0000187921	 weakly similar to ( 172) AT1G19180 | Symbols: TIFY10A | JAZ1 (JASMONATE-ZIM-DOMAIN PROTEIN 1); protein binding | chr1:6622708-6623271 FORWARDweakly similar to ( 130) loc_os07g42370 12007.m29261 protein pnFL-2, putative, expressed no original description 	T
29.2.1.1.3.1.21	protein.synthesis.ribosomal protein.prokaryotic.unknown organellar.30S subunit.S21	MDP0000308332	 weakly similar to ( 153) AT3G27160 | Symbols: GHS1 | GHS1 (GLUCOSE HYPERSENSITIVE 1); structural constituent of ribosome | chr3:10017531-10018854 FORWARDvery weakly similar to (63.2) RR21_SPIOL Chloroplast 30S ribosomal protein S21 (Fragment) - Spinacia oleracea (Spinach)very weakly similar to (97.8) loc_os02g09590 12002.m06256 protein ribosome-like protein, putative, expressed   no original description 	T
35.2	not assigned.unknown	MDP0000237340	 moderately similar to ( 439) AT1G02145 | Symbols:  | transferase, transferring glycosyl groups | chr1:404915-408485 FORWARDmoderately similar to ( 387) loc_os04g46050 12004.m101602 protein dolichyl-P-Man Man, putative, expressed  no original description 	T
1.2.4.4	PS.photorespiration.glycine cleavage.H protein	MDP0000613170	 moderately similar to ( 253) AT1G32470 | Symbols:  | glycine cleavage system H protein, mitochondrial, putative | chr1:11739479-11740246 REVERSEmoderately similar to ( 250) GCSH_PEA Glycine cleavage system H protein, mitochondrial precursor - Pisum sativum (Garden pea)moderately similar to ( 234) loc_os10g37180 12010.m06515 protein glycine cleavage system H protein, mitochondrial precursor, putative, expressed no original description 	T
21.4	redox.glutaredoxins	MDP0000472203	 weakly similar to ( 177) AT5G18600 | Symbols:  | glutaredoxin family protein | chr5:6183466-6183774 REVERSEvery weakly similar to (69.3) GLRX_RICCO Glutaredoxin - Ricinus communis (Castor bean)weakly similar to ( 128) loc_os01g27140 12001.m09165 protein glutaredoxin, putative, expressed no original description 	T
29.5.11.4.2	protein.degradation.ubiquitin.E3.RING	MDP0000563233	 very weakly similar to (67.4) AT1G67180 | Symbols:  | zinc finger (C3HC4-type RING finger) family protein / BRCT domain-containing protein | chr1:25130099-25132169 FORWARDvery weakly similar to (77.4) loc_os08g31930 12008.m07187 protein ubiquitin-protein ligase/ zinc ion binding protein, putative, expressed   no original description 	T
34.3	transport.amino acids	MDP0000426374	 highly similar to ( 919) AT1G47670 | Symbols:  | amino acid transporter family protein | chr1:17536834-17539486 REVERSEhighly similar to ( 742) loc_os12g30040 12012.m06817 protein amino acid transporter-like protein 1, putative, expressed  no original description 	T
28.1.3.1	DNA.synthesis/chromatin structure.histone.H1	MDP0000320059	 very weakly similar to (77.8) AT2G30620 | Symbols:  | histone H1.2 | chr2:13045360-13046267 FORWARDvery weakly similar to (72.0) H1_MAIZE Histone H1 - Zea mays (Maize)very weakly similar to (73.2) loc_os03g58470 12003.m10753 protein histone H1, putative, expressed no original description 	T
31.1	cell.organisation	MDP0000183660	 highly similar to ( 719) AT3G12110 | Symbols: ACT11 | ACT11 (actin-11); structural constituent of cytoskeleton | chr3:3858116-3859609 FORWARDhighly similar to ( 724) ACT_GOSHI Actin - Gossypium hirsutum (Upland cotton)highly similar to ( 722) loc_os03g50890 12003.m10074 protein actin-1, putative, expressed  no original description 	T
29.2.1.2.1.24	protein.synthesis.ribosomal protein.eukaryotic.40S subunit.S24	MDP0000523595	 moderately similar to ( 231) AT3G04920 | Symbols:  | 40S ribosomal protein S24 (RPS24A) | chr3:1360989-1362065 FORWARDmoderately similar to ( 216) loc_os01g52490 12001.m11423 protein 40S ribosomal protein S24, putative, expressed  no original description 	T
29.6	protein.folding	MDP0000286941	 highly similar to ( 610) AT1G24510 | Symbols:  | T-complex protein 1 epsilon subunit, putative / TCP-1-epsilon, putative / chaperonin, putative | chr1:8685504-8687660 REVERSEhighly similar to ( 588) TCPE2_AVESA T-complex protein 1 subunit epsilon (TCP-1-epsilon) (CCT-epsilon) (TCP-K36) - Avena sativa (Oat)highly similar to ( 613) loc_os06g36700 12006.m08205 protein T-complex protein 1 subunit epsilon, putative, expressed  no original description 	T
27.3.29	RNA.regulation of transcription.TCP transcription factor family	MDP0000155433	 weakly similar to ( 171) AT5G60970 | Symbols: TCP5 | TCP5 (TEOSINTE BRANCHED1, CYCLOIDEA AND PCF TRANSCRIPTION FACTOR 5); transcription factor | chr5:24535570-24536652 REVERSEweakly similar to ( 139) loc_os01g55750 12001.m43206 protein TCP family transcription factor containing protein, expressed no original description 	T
31.4	cell.vesicle transport	MDP0000179513	 nearly identical (1206) AT5G51430 | Symbols: EYE | EYE (EMBRYO YELLOW) | chr5:20887044-20890795 REVERSEnearly identical (1115) loc_os06g45830 12006.m09111 protein expressed protein  no original description 	T
28.1	DNA.synthesis/chromatin structure	MDP0000893502	 moderately similar to ( 430) AT1G68290 | Symbols: ENDO 2 | ENDO 2 (endonuclease 2); T/G mismatch-specific endonuclease/ endonuclease/ nucleic acid binding / single-stranded DNA specific endodeoxyribonuclease | chr1:25596718-25598264 FORWARDmoderately similar to ( 388) loc_os01g03740 12001.m07010 protein nuclease PA3, putative, expressed no original description 	T
29.2.4	protein.synthesis.elongation	MDP0000219010	 highly similar to ( 856) AT5G60390 | Symbols:  | elongation factor 1-alpha / EF-1-alpha | chr5:24289226-24290675 FORWARDhighly similar to ( 858) EF1A_MAIZE Elongation factor 1-alpha (EF-1-alpha) - Zea mays (Maize)highly similar to ( 857) loc_os03g08050 12003.m78808 protein elongation factor 1-alpha, putative, expressed   no original description 	T
29.2.1.2.2.36	protein.synthesis.ribosomal protein.eukaryotic.60S subunit.L36	MDP0000156587	 weakly similar to ( 172) AT5G02450 | Symbols:  | 60S ribosomal protein L36 (RPL36C) | chr5:533501-534394 FORWARDweakly similar to ( 143) RL36_DAUCA 60S ribosomal protein L36 - Daucus carota (Carrot)weakly similar to ( 192) loc_os05g38520 12005.m27873 protein 60S ribosomal protein L36-2, putative, expressed   no original description 	T
7.2.4	OPP.non-reductive PP.ribose 5-phosphate isomerase	MDP0000271244	 moderately similar to ( 377) AT2G01290 | Symbols:  | ribose-5-phosphate isomerase | chr2:149192-149989 REVERSEmoderately similar to ( 305) loc_os07g08030 12007.m05261 protein ribose-5-phosphate isomerase, putative, expressed    no original description 	T
1.1.1.2	PS.lightreaction.photosystem II.PSII polypeptide subunits	MDP0000261330	 weakly similar to ( 127) AT5G02120 | Symbols: OHP | OHP (ONE HELIX PROTEIN) | chr5:419144-419633 FORWARDweakly similar to ( 127) loc_os05g22730 12005.m06569 protein expressed protein  no original description 	T
13.1.3.4	amino acid metabolism.synthesis.aspartate family.methionine	MDP0000656078	 highly similar to ( 731) AT1G02500 | Symbols: SAM1, SAM-1, MAT1 | SAM1 (S-ADENOSYLMETHIONINE SYNTHETASE 1); methionine adenosyltransferase | chr1:519037-520218 FORWARDhighly similar to ( 735) METL_CATRO S-adenosylmethionine synthetase 2 (EC 2.5.1.6) (Methionine adenosyltransferase 2) (AdoMet synthetase 2) - Catharanthus roseus (Rosy periwinkle) (Madagascar periwinkle)highly similar to ( 728) loc_os05g04510 12005.m27538 protein S-adenosylmethionine synthetase 1, putative, expressed  no original description 	T
30.7	signalling.14-3-3 proteins	MDP0000387626	 highly similar to ( 595) AT5G16040 | Symbols:  | regulator of chromosome condensation (RCC1) family protein | chr5:5240860-5243308 REVERSEmoderately similar to ( 442) 1433A_SOYBN 14-3-3-like protein A (SGF14A) - Glycine max (Soybean)highly similar to ( 560) loc_os08g33390 12008.m07329 protein HECT domain and RCC1-like domain-containing protein 2, putative, expressed   no original description 	T
27.3.99	RNA.regulation of transcription.unclassified	MDP0000294807	 moderately similar to ( 400) AT3G26935 | Symbols:  | zinc finger (DHHC type) family protein | chr3:9933001-9935218 REVERSEhighly similar to ( 502) loc_os08g42620 12008.m080224 protein palmitoyltransferase ZDHHC9, putative, expressed  no original description 	T
34.13	transport.peptides and oligopeptides	MDP0000600063	 highly similar to ( 691) AT5G62680 | Symbols:  | proton-dependent oligopeptide transport (POT) family protein | chr5:25165430-25167822 REVERSEhighly similar to ( 611) loc_os03g48180 12003.m101441 protein peptide transporter PTR2, putative, expressed  TrbL PRK07003  no original description 	T
35.2	not assigned.unknown	MDP0000811769	 weakly similar to ( 158) AT1G64700 | Symbols:  | unknown protein | chr1:24039931-24040542 REVERSE  no original description 	T
9.1.2	mitochondrial electron transport / ATP synthesis.NADH-DH.localisation not clear	MDP0000070106	 moderately similar to ( 419) AT4G04860 | Symbols: DER2.2 | DER2.2 (DERLIN-2.2) | chr4:2460135-2461378 FORWARDmoderately similar to ( 389) DERL2_ORYSA Derlin-2 (OsDerlin 2-1) - Oryza sativa (Rice)moderately similar to ( 389) loc_os03g63520 12003.m11220 protein derlin-2, putative, expressed no original description 	T
35.2	not assigned.unknown	MDP0000183959	 moderately similar to ( 481) AT3G08030 | Symbols:  | unknown protein | chr3:2564517-2565819 FORWARDmoderately similar to ( 422) loc_os03g59300 12003.m34946 protein expressed protein  no original description 	T
29.5.7	protein.degradation.metalloprotease	MDP0000193420	 highly similar to ( 667) AT3G59990 | Symbols: MAP2B | MAP2B (METHIONINE AMINOPEPTIDASE 2B); aminopeptidase/ metalloexopeptidase | chr3:22155921-22158551 REVERSEhighly similar to ( 641) loc_os12g43370 12012.m26987 protein methionine aminopeptidase 2B, putative, expressed  no original description 	T
29.4.1.57	protein.postranslational modification.kinase.receptor like cytoplasmatic kinase VII	MDP0000151537	 highly similar to ( 753) AT2G39360 | Symbols:  | protein kinase family protein | chr2:16437592-16440039 REVERSEmoderately similar to ( 232) NORK_MEDTR Nodulation receptor kinase precursor (EC 2.7.11.1) (Does not make infections protein 2) (Symbiosis receptor-like kinase) (MtSYMRK) - Medicago truncatula (Barrel medic)highly similar to ( 721) loc_os06g22810 12006.m06934 protein protein kinase, putative, expressed   no original description 	T
27.3.17	RNA.regulation of transcription.CPP(Zn),CPP1-related transcription factor family	MDP0000174095	 moderately similar to ( 207) AT4G14770 | Symbols: TCX2, ATTCX2 | TCX2 (TESMIN/TSO1-LIKE CXC 2); transcription factor | chr4:8481522-8484825 REVERSEmoderately similar to ( 212) loc_os07g07974 12007.m05257 protein TSO1, putative, expressed   no original description 	T
35.2	not assigned.unknown	MDP0000263883	 highly similar to ( 545) AT4G14950 | Symbols:  | unknown protein | chr4:8545517-8547722 FORWARDmoderately similar to ( 495) loc_os01g57066 12001.m42516 protein transmembrane protein 49, putative, expressed no original description 	T
29.2.3	protein.synthesis.initiation	MDP0000903096	 moderately similar to ( 230) AT3G60240 | Symbols: EIF4G, CUM2 | EIF4G (EUKARYOTIC TRANSLATION INITIATION FACTOR 4G); translation initiation factor | chr3:22261842-22268295 FORWARDweakly similar to ( 199) loc_os07g36940 12007.m079713 protein eukaryotic translation initiation factor 4G, putative, expressed no original description 	T
26.24	misc.GCN5-related N-acetyltransferase	MDP0000781028	 moderately similar to ( 279) AT2G39020 | Symbols:  | GCN5-related N-acetyltransferase (GNAT) family protein | chr2:16295392-16296102 FORWARDweakly similar to ( 132) THT11_TOBAC Tyramine N-feruloyltransferase 4/11 (EC 2.3.1.110) (Hydroxycinnamoyl-CoA: tyramine N-hydroxycinnamoyltransferase) - Nicotiana tabacum (Common tobacco)moderately similar to ( 204) loc_os03g05710 12003.m06093 protein N-acetyltransferase, putative, expressed   Sporozoite_P67 TrbL  no original description 	T
17.2.3	hormone metabolism.auxin.induced-regulated-responsive-activated	MDP0000209900	 weakly similar to ( 138) AT2G46690 | Symbols:  | auxin-responsive family protein | chr2:19180904-19181269 FORWARDvery weakly similar to (64.7) A10A5_SOYBN Auxin-induced protein 10A5 - Glycine max (Soybean)very weakly similar to (94.7) loc_os06g50040 12006.m09528 protein OsSAUR29 - Auxin-responsive SAUR gene family member, expressed  MSP1_C  no original description 	T
35.2	not assigned.unknown	MDP0000192776	 nearly identical (1365) AT2G25800 | Symbols:  | INVOLVED IN: biological_process unknown; EXPRESSED IN: 23 plant structures; EXPRESSED DURING: 13 growth stages; CONTAINS InterPro DOMAIN/s: Munc13 homology 1 (InterPro:IPR014770), Protein of unknown function DUF810 (InterPro:IPR008528), Munc13 homology 2 (InterPro:IPR014772); BEST Arabidopsis thaliana protein match is: unknown protein (TAIR:AT2G20010.2); Has 242 Blast hits to 156 proteins in 35 species: Archae - 0; Bacteria - 8; Metazoa - 36; Fungi - 18; Plants - 94; Viruses - 0; Other Eukaryotes - 86 (source: NCBI BLink). | chr2:11006138-11009728 REVERSEnearly identical (1128) loc_os09g17760 12009.m05034 protein expressed protein   no original description 	T
29.2.1.2.2.22	protein.synthesis.ribosomal protein.eukaryotic.60S subunit.L22	MDP0000291704	 weakly similar to ( 161) AT3G05560 | Symbols:  | 60S ribosomal protein L22-2 (RPL22B) | chr3:1614641-1615204 FORWARDweakly similar to ( 153) loc_os07g47710 12007.m08986 protein 60S ribosomal protein L22-2, putative, expressed   no original description 	T
29.6	protein.folding	MDP0000132736	 weakly similar to ( 188) AT2G34860 | Symbols: EDA3 | EDA3 (embryo sac development arrest 3); heat shock protein binding / unfolded protein binding | chr2:14708380-14709804 FORWARDweakly similar to ( 182) loc_os08g36140 12008.m07603 protein expressed protein  no original description 	T
29.5.2	protein.degradation.autophagy	MDP0000798204	 moderately similar to ( 215) AT2G05630 | Symbols: ATG8D | autophagy 8d (APG8d) | chr2:2083211-2084775 REVERSEmoderately similar to ( 217) loc_os08g09240 12008.m26825 protein autophagy-related protein 8 precursor, putative, expressed no original description 	T
29.5	protein.degradation	MDP0000289880	 nearly identical (1191) AT1G76140 | Symbols:  | serine-type endopeptidase/ serine-type peptidase | chr1:28571187-28574852 FORWARDnearly identical (1090) loc_os01g01830 12001.m06826 protein prolyl endopeptidase, putative, expressed  no original description 	T
21.1	redox.thioredoxin	MDP0000863789	 moderately similar to ( 232) AT3G06730 | Symbols:  | thioredoxin family protein | chr3:2124276-2125845 FORWARDvery weakly similar to (69.7) TRXM_SPIOL Thioredoxin M-type, chloroplast precursor (TRX-M) [Contains: Thioredoxin M-type Mc; Thioredoxin M-type Md] - Spinacia oleracea (Spinach)weakly similar to ( 189) loc_os08g29110 12008.m06915 protein thioredoxin, putative, expressed  no original description 	T
31.1	cell.organisation	MDP0000293126	 highly similar to ( 687) AT2G29890 | Symbols: VLN1, ATVLN1 | VLN1 (VILLIN-LIKE 1); actin binding / actin filament binding | chr2:12744597-12749474 FORWARDhighly similar to ( 526) loc_os08g14230 12008.m05548 protein villin-4, putative, expressed  no original description 	T
27.3.6	RNA.regulation of transcription.bHLH,Basic Helix-Loop-Helix family	MDP0000138102	 moderately similar to ( 243) AT2G41130 | Symbols:  | basic helix-loop-helix (bHLH) family protein | chr2:17143360-17144686 FORWARDvery weakly similar to (51.2) ARRS_MAIZE Anthocyanin regulatory R-S protein - Zea mays (Maize)weakly similar to ( 173) loc_os03g15440 12003.m06981 protein DNA binding protein, putative, expressed no original description 	T
35.1	not assigned.no ontology	MDP0000767832	 weakly similar to ( 149) AT1G17100 | Symbols:  | SOUL heme-binding family protein | chr1:5844766-5845539 FORWARDmoderately similar to ( 259) loc_os01g13420 12001.m07951 protein heme-binding protein 2, putative, expressed no original description 	T
27.3.25	RNA.regulation of transcription.MYB domain transcription factor family	MDP0000629440	 moderately similar to ( 263) AT2G23290 | Symbols: AtMYB70 | AtMYB70 (myb domain protein 70); DNA binding / transcription factor | chr2:9904895-9905824 REVERSEweakly similar to ( 114) GAM1_ORYSA Transcription factor GAMYB (OsGAMyb) - Oryza sativa (Rice)moderately similar to ( 236) loc_os09g01960 12009.m03570 protein sucrose responsive element binding protein, putative, expressed   no original description 	T
29.4.1.57	protein.postranslational modification.kinase.receptor like cytoplasmatic kinase VII	MDP0000092936	 highly similar to ( 605) AT5G45840 | Symbols:  | leucine-rich repeat transmembrane protein kinase, putative | chr5:18594080-18597221 REVERSEweakly similar to ( 124) CRI4_MAIZE Putative receptor protein kinase CRINKLY4 precursor (EC 2.7.11.1) - Zea mays (Maize)highly similar to ( 531) loc_os01g10900 12001.m42620 protein ATP binding protein, putative, expressed  no original description 	T
30.11	signalling.light	MDP0000308582	 moderately similar to ( 414) AT3G18890 | Symbols:  | binding / catalytic/ coenzyme binding | chr3:6511169-6514729 FORWARDmoderately similar to ( 349) loc_os11g35340 12011.m07386 protein S-adenosylmethionine-dependent methyltransferase/ catalytic, putative, expressed MSP1_C  no original description 	T
29.5.11.4.3.3	protein.degradation.ubiquitin.E3.SCF.cullin	MDP0000242092	 nearly identical (1210) AT4G02570 | Symbols: ATCUL1, CUL1, AXR6 | ATCUL1 (ARABIDOPSIS THALIANA CULLIN 1); protein binding | chr4:1129315-1133435 FORWARDnearly identical (1158) loc_os01g27150 12001.m42650 protein cullin-1, putative, expressed  no original description 	T
19.3	tetrapyrrole synthesis.GSA	MDP0000149467	 highly similar to ( 684) AT5G63570 | Symbols: GSA1 | GSA1 (GLUTAMATE-1-SEMIALDEHYDE-2,1-AMINOMUTASE); glutamate-1-semialdehyde 2,1-aminomutase | chr5:25451957-25453620 FORWARDhighly similar to ( 692) GSA_TOBAC Glutamate-1-semialdehyde 2,1-aminomutase, chloroplast precursor (EC 5.4.3.8) (GSA) (Glutamate-1-semialdehyde aminotransferase) (GSA-AT) - Nicotiana tabacum (Common tobacco)highly similar to ( 661) loc_os08g41990 12008.m08177 protein glutamate-1-semialdehyde 2,1-aminomutase, chloroplast precursor, putative, expressed  no original description 	T
35.1	not assigned.no ontology	MDP0000582810	 moderately similar to ( 456) AT1G76340 | Symbols:  | integral membrane family protein | chr1:28635188-28636306 REVERSEmoderately similar to ( 414) loc_os03g11590 12003.m101239 protein expressed protein no original description 	T
1.1.5.2	PS.lightreaction.other electron carrier (ox/red).ferredoxin	MDP0000178104	 weakly similar to ( 176) AT1G32550 | Symbols:  | ferredoxin family protein | chr1:11771969-11774117 REVERSEvery weakly similar to (64.7) FER1_RAPSA Ferredoxin, root R-B1 - Raphanus sativus (Radish)weakly similar to ( 176) loc_os03g48040 12003.m34755 protein ferredoxin-6, chloroplast precursor, putative, expressed   no original description 	T
29.5.11.20	protein.degradation.ubiquitin.proteasom	MDP0000162339	 moderately similar to ( 291) AT1G64520 | Symbols: RPN12a | RPN12a (Regulatory Particle non-ATPase 12a); peptidase | chr1:23956459-23958120 FORWARDmoderately similar to ( 293) loc_os07g25420 12007.m29149 protein 26S proteasome non-ATPase regulatory subunit 8, putative, expressed MSP1_C  no original description 	T
27.4	RNA.RNA binding	MDP0000216169	 weakly similar to ( 137) AT4G13850 | Symbols: ATGRP2 | GR-RBP2 (GLYCINE-RICH RNA-BINDING PROTEIN 2); ATP binding / RNA binding / double-stranded DNA binding / single-stranded DNA binding | chr4:8021314-8022065 FORWARDvery weakly similar to (89.0) GRP1_DAUCA Glycine-rich RNA-binding protein - Daucus carota (Carrot)weakly similar to ( 124) loc_os01g68790 12001.m43348 protein glycine-rich RNA-binding protein 2, mitochondrial precursor, putative, expressed no original description 	T
20.1	stress.biotic	MDP0000527355	 moderately similar to ( 494) AT1G05850 | Symbols: POM1, ERH2, ELP1, CTL1, ELP, HOT2, ATCTL1 | POM1 (POM-POM1); chitinase | chr1:1766833-1768117 REVERSEweakly similar to ( 192) CHI2_BRANA Endochitinase CH25 precursor (EC 3.2.1.14) - Brassica napus (Rape)moderately similar to ( 436) loc_os09g32080 12009.m060024 protein endochitinase A2 precursor, putative, expressed  no original description 	T
31.1	cell.organisation	MDP0000797241	 highly similar to ( 670) AT1G07710 | Symbols:  | ankyrin repeat family protein | chr1:2386275-2387986 REVERSEvery weakly similar to (53.1) AKT1_ORYSA Potassium channel AKT1 (OsAKT1) - Oryza sativa (Rice)highly similar to ( 613) loc_os03g17250 12003.m07155 protein protein binding protein, putative, expressed   no original description 	T
17.7.1.2	hormone metabolism.jasmonate.synthesis-degradation.lipoxygenase	MDP0000081005	 highly similar to ( 714) AT1G55020 | Symbols: LOX1 | LOX1; lipoxygenase | chr1:20525798-20530143 FORWARDhighly similar to ( 686) LOX1_SOLTU Lipoxygenase 1 (EC 1.13.11.12) - Solanum tuberosum (Potato)highly similar to ( 600) loc_os03g49380 12003.m35371 protein lipoxygenase 4, putative, expressed  no original description 	T
29.2.1.2.1.53	protein.synthesis.ribosomal protein.eukaryotic.40S subunit.S3A	MDP0000450792	 moderately similar to ( 373) AT3G04840 | Symbols:  | 40S ribosomal protein S3A (RPS3aA) | chr3:1329751-1331418 FORWARDmoderately similar to ( 373) RS3A_ORYSA 40S ribosomal protein S3a (CYC07 protein) - Oryza sativa (Rice)moderately similar to ( 378) loc_os12g21798 12012.m26700 protein 40S ribosomal protein S3a, putative, expressed no original description 	T
26.2	misc.UDP glucosyl and glucoronyl transferases	MDP0000226447	 moderately similar to ( 444) AT1G01420 | Symbols: UGT72B3 | UGT72B3 (UDP-GLUCOSYL TRANSFERASE 72B3); UDP-glycosyltransferase/ quercetin 3-O-glucosyltransferase/ transferase, transferring glycosyl groups | chr1:154566-156011 REVERSEweakly similar to ( 159) ZOX_PHAVU Zeatin O-xylosyltransferase (EC 2.4.2.40) (Zeatin O-beta-D-xylosyltransferase) - Phaseolus vulgaris (Kidney bean) (French bean)moderately similar to ( 344) loc_os11g38650 12011.m07713 protein hydroquinone glucosyltransferase, putative, expressed  TrbL  no original description 	T
35.2	not assigned.unknown	MDP0000272085	 moderately similar to ( 254) AT3G32940 | Symbols:  | RNA binding | chr3:13491089-13493630 REVERSEmoderately similar to ( 227) loc_os08g01070 12008.m080054 protein transcription or splicing factor-like protein, putative, expressed  COG3889  no original description 	T
29.4	protein.postranslational modification	MDP0000289545	 moderately similar to ( 453) AT3G51370 | Symbols:  | protein phosphatase 2C, putative / PP2C, putative | chr3:19070387-19071975 FORWARDmoderately similar to ( 456) loc_os06g50380 12006.m31935 protein protein phosphatase 2C, putative, expressed  no original description 	T
29.3.3	protein.targeting.chloroplast	MDP0000126130	 moderately similar to ( 319) AT2G15290 | Symbols: ATTIC21, TIC21, CIA5, PIC1 | TIC21 (TRANSLOCON AT INNER MEMBRANE OF CHLOROPLASTS 21); copper uptake transmembrane transporter/ iron ion transmembrane transporter/ protein homodimerization | chr2:6642512-6644011 REVERSEmoderately similar to ( 298) loc_os02g09470 12002.m06244 protein expressed protein   no original description 	T
25.1	C1-metabolism.glycine hydroxymethyltransferase	MDP0000316281	 highly similar to ( 852) AT4G13930 | Symbols: SHM4 | SHM4 (serine hydroxymethyltransferase 4); catalytic/ glycine hydroxymethyltransferase/ pyridoxal phosphate binding | chr4:8048013-8050021 REVERSEhighly similar to ( 531) GLYM_PEA Serine hydroxymethyltransferase, mitochondrial precursor (EC 2.1.2.1) (Serine methylase) (Glycine hydroxymethyltransferase) (SHMT) - Pisum sativum (Garden pea)highly similar to ( 780) loc_os12g22030 12012.m26898 protein serine hydroxymethyltransferase, mitochondrial precursor, putative, expressed   no original description 	T
29.2.1.2.1.53	protein.synthesis.ribosomal protein.eukaryotic.40S subunit.S3A	MDP0000304729	 moderately similar to ( 373) AT4G34670 | Symbols:  | 40S ribosomal protein S3A (RPS3aB) | chr4:16548724-16550222 FORWARDmoderately similar to ( 372) RS3A_CATRO 40S ribosomal protein S3a (CYC07 protein) - Catharanthus roseus (Rosy periwinkle) (Madagascar periwinkle)moderately similar to ( 376) loc_os12g21798 12012.m26700 protein 40S ribosomal protein S3a, putative, expressed    no original description 	T
29.8	protein.assembly and cofactor ligation	MDP0000297963	 highly similar to ( 898) AT4G04770 | Symbols: ATABC1, LAF6, ATNAP1 | ATABC1 (ATP BINDING CASSETTE PROTEIN 1); ATPase, coupled to transmembrane movement of substances / protein binding / transporter | chr4:2427998-2429785 REVERSEhighly similar to ( 728) loc_os01g61400 12001.m12266 protein expressed protein MSP1_C  no original description 	T
35.2	not assigned.unknown	MDP0000269836	 weakly similar to ( 187) AT5G43750 | Symbols:  | unknown protein | chr5:17580100-17581145 REVERSEweakly similar to ( 187) loc_os01g70400 12001.m43437 protein expressed protein  no original description 	T
26.3.2	misc.gluco-, galacto- and mannosidases.beta-galactosidase	MDP0000310582	 nearly identical (1418) AT4G36360 | Symbols: BGAL3 | BGAL3 (beta-galactosidase 3); beta-galactosidase/ catalytic/ cation binding / sugar binding | chr4:17176840-17181143 REVERSEnearly identical (1105) BGAL_ASPOF Beta-galactosidase precursor (EC 3.2.1.23) (Lactase) - Asparagus officinalis (Garden asparagus)nearly identical (1283) loc_os03g06940 12003.m35030 protein beta-galactosidase precursor, putative, expressed  no original description 	T
29.2.1.2.1.31	protein.synthesis.ribosomal protein.eukaryotic.40S subunit.SA	MDP0000231146	 moderately similar to ( 405) AT1G72370 | Symbols: P40, AP40, RP40, RPSAA | P40; structural constituent of ribosome | chr1:27243148-27244842 REVERSEmoderately similar to ( 436) RSSA_SOYBN 40S ribosomal protein SA (p40) - Glycine max (Soybean)moderately similar to ( 404) loc_os07g42450 12007.m08478 protein 40S ribosomal protein SA, putative, expressed  no original description 	T
35.2	not assigned.unknown	MDP0000794663	 moderately similar to ( 303) AT3G18050 | Symbols:  | unknown protein | chr3:6180931-6182593 FORWARDweakly similar to ( 170) loc_os07g37156 12007.m79903 protein expressed protein  no original description 	T
3.5	minor CHO metabolism.others	MDP0000253287	 moderately similar to ( 480) AT1G14520 | Symbols: MIOX1 | MIOX1 (MYO-INISITOL OXYGENASE); inositol oxygenase/ oxidoreductase | chr1:4968376-4969954 REVERSEmoderately similar to ( 459) MIOX_ORYSA Probable inositol oxygenase (EC 1.13.99.1) (Myo-inositol oxygenase) - Oryza sativa (Rice)moderately similar to ( 459) loc_os06g36560 12006.m32098 protein inositol oxygenase, putative, expressed   no original description 	T
3.4.4	minor CHO metabolism.myo-inositol.myo inositol oxygenases	MDP0000253287	 moderately similar to ( 480) AT1G14520 | Symbols: MIOX1 | MIOX1 (MYO-INISITOL OXYGENASE); inositol oxygenase/ oxidoreductase | chr1:4968376-4969954 REVERSEmoderately similar to ( 459) MIOX_ORYSA Probable inositol oxygenase (EC 1.13.99.1) (Myo-inositol oxygenase) - Oryza sativa (Rice)moderately similar to ( 459) loc_os06g36560 12006.m32098 protein inositol oxygenase, putative, expressed   no original description 	T
35.1.5	not assigned.no ontology.pentatricopeptide (PPR) repeat-containing protein	MDP0000305971	 nearly identical (1459) AT1G48410 | Symbols: AGO1 | AGO1 (ARGONAUTE 1); endoribonuclease/ miRNA binding / protein binding / siRNA binding | chr1:17886285-17891892 REVERSEweakly similar to ( 105) RF1_ORYSA Rf1 protein, mitochondrial precursor (PPR protein) (Fertility restorer) (Restorer for CMS) - Oryza sativa (Rice)nearly identical (1450) loc_os04g47870 12004.m09728 protein PINHEAD protein, putative, expressed   no original description 	T
1.3.9	PS.calvin cycle.seduheptulose bisphosphatase	MDP0000138073	 highly similar to ( 592) AT3G55800 | Symbols: SBPASE | SBPASE (sedoheptulose-bisphosphatase); phosphoric ester hydrolase/ sedoheptulose-bisphosphatase | chr3:20709640-20711421 FORWARDhighly similar to ( 562) S17P_SPIOL Sedoheptulose-1,7-bisphosphatase, chloroplast precursor (EC 3.1.3.37) (Sedoheptulose-bisphosphatase) (SBPase) (SED(1,7)P2ase) - Spinacia oleracea (Spinach)highly similar to ( 534) loc_os04g16680 12004.m06845 protein sedoheptulose-1,7-bisphosphatase, chloroplast precursor, putative, expressed  no original description 	T
33.99	development.unspecified	MDP0000384846	 highly similar to ( 558) AT1G18080 | Symbols: ATARCA, RACK1A_AT | ATARCA; nucleotide binding | chr1:6222325-6223901 FORWARDhighly similar to ( 575) GBLP_SOYBN Guanine nucleotide-binding protein subunit beta-like protein - Glycine max (Soybean)moderately similar to ( 472) loc_os01g49290 12001.m11115 protein guanine nucleotide-binding protein beta subunit-like protein, putative, expressed   no original description 	T
29.2.3	protein.synthesis.initiation	MDP0000296038	 weakly similar to ( 188) AT1G54290 | Symbols:  | eukaryotic translation initiation factor SUI1, putative | chr1:20268710-20269390 REVERSEweakly similar to ( 184) SUI1_BRAOL Protein translation factor SUI1 homolog (Translation initiation factor nps45) - Brassica oleracea (Wild cabbage)weakly similar to ( 183) loc_os05g41900 12005.m27899 protein protein translation factor SUI1, putative, expressed no original description 	T
10.6.1	cell wall.degradation.cellulases and beta -1,4-glucanases	MDP0000196385	 highly similar to ( 926) AT5G04885 | Symbols:  | glycosyl hydrolase family 3 protein | chr5:1423369-1426628 FORWARDvery weakly similar to (52.4) XYNB_PRUPE Putative beta-D-xylosidase (EC 3.2.1.-) (PpAz152) (Fragment) - Prunus persica (Peach)highly similar to ( 858) loc_os03g53800 12003.m101132 protein periplasmic beta-glucosidase precursor, putative, expressed no original description 	T
31.3	cell.cycle	MDP0000129333	 moderately similar to ( 326) AT4G34160 | Symbols: CYCD3;1, CYCD3 | CYCD3;1 (CYCLIN D3;1); cyclin-dependent protein kinase regulator/ protein binding | chr4:16357903-16359304 FORWARDvery weakly similar to (57.4) CCNB2_ORYSA G2/mitotic-specific cyclin-2 (B-like cyclin) (CycOs2) - Oryza sativa (Rice)weakly similar to ( 169) loc_os07g42860 12007.m08515 protein cyclin delta-2, putative, expressed   no original description 	T
31.3.1	cell.cycle.peptidylprolyl isomerase	MDP0000253745	 moderately similar to ( 268) AT3G56070 | Symbols: ROC2 | ROC2 (ROTAMASE CYCLOPHILIN 2); cyclosporin A binding / peptidyl-prolyl cis-trans isomerase | chr3:20806987-20807517 REVERSEmoderately similar to ( 258) CYPH_LUPLU Peptidyl-prolyl cis-trans isomerase (EC 5.2.1.8) (PPIase) (Rotamase) (Cyclophilin) (Cyclosporin A-binding protein) - Lupinus luteus (European yellow lupin)moderately similar to ( 276) loc_os09g39780 12009.m22203 protein peptidyl-prolyl cis-trans isomerase CYP19-3, putative, expressed  no original description 	T
29.5.11.3	protein.degradation.ubiquitin.E2	MDP0000140755	 moderately similar to ( 232) AT5G53300 | Symbols:  | UBC10 (ubiquitin-conjugating enzyme 10); ubiquitin-protein ligase | chr5:21633187-21633989 REVERSEweakly similar to ( 104) UBC2_WHEAT Ubiquitin-conjugating enzyme E2-17 kDa (EC 6.3.2.19) (Ubiquitin-protein ligase) (Ubiquitin carrier protein) - Triticum aestivum (Wheat)moderately similar to ( 229) loc_os01g60410 12001.m42744 protein ubiquitin-conjugating enzyme E2-17 kDa, putative, expressed  no original description 	T
26.21	misc.protease inhibitor/seed storage/lipid transfer protein (LTP) family protein	MDP0000141631	 weakly similar to ( 142) AT3G53980 | Symbols:  | protease inhibitor/seed storage/lipid transfer protein (LTP) family protein | chr3:19987760-19988203 REVERSEweakly similar to ( 134) loc_os05g06780 12005.m05205 protein lipid binding protein, putative, expressed  no original description 	T
29.4.1.57	protein.postranslational modification.kinase.receptor like cytoplasmatic kinase VII	MDP0000390677	 nearly identical (1005) AT3G51550 | Symbols: FER | FER (FERONIA); kinase/ protein kinase | chr3:19117877-19120564 REVERSEmoderately similar to ( 247) NORK_MEDTR Nodulation receptor kinase precursor (EC 2.7.11.1) (Does not make infections protein 2) (Symbiosis receptor-like kinase) (MtSYMRK) - Medicago truncatula (Barrel medic)highly similar to ( 901) loc_os03g21540 12003.m07558 protein protein kinase, putative, expressed  no original description 	T
8.1.1.2	TCA / org transformation.TCA.pyruvate DH.E2	MDP0000311469	 highly similar to ( 568) AT1G34430 | Symbols: EMB3003 | EMB3003 (embryo defective 3003); acyltransferase/ dihydrolipoyllysine-residue acetyltransferase/ protein binding | chr1:12588027-12590084 REVERSEhighly similar to ( 536) loc_os12g08170 12012.m04804 protein dihydrolipoyllysine-residue acetyltransferase component of pyruvatedehydrogenase complex, mitochondrial precursor, putative, expressed  no original description 	T
1.1.1.1	PS.lightreaction.photosystem II.LHC-II	MDP0000233260	 moderately similar to ( 389) AT1G76570 | Symbols:  | chlorophyll A-B binding family protein | chr1:28729132-28730754 FORWARDweakly similar to ( 156) CB23_HORVU Chlorophyll a-b binding protein of LHCII type III, chloroplast precursor (CAB) - Hordeum vulgare (Barley)moderately similar to ( 353) loc_os09g12540 12009.m04517 protein chlorophyll a-b binding protein, chloroplast precursor, putative, expressed   no original description 	T
27.3.6	RNA.regulation of transcription.bHLH,Basic Helix-Loop-Helix family	MDP0000900024	 moderately similar to ( 227) AT5G46830 | Symbols:  | basic helix-loop-helix (bHLH) family protein | chr5:19002719-19004254 FORWARDvery weakly similar to (79.0) ARLC_MAIZE Anthocyanin regulatory Lc protein - Zea mays (Maize)weakly similar to ( 162) loc_os10g42430 12010.m06996 protein transcription factor MYC7E, putative, expressed   no original description 	T
35.2	not assigned.unknown	MDP0000199268	 moderately similar to ( 366) AT5G61820 | Symbols:  | FUNCTIONS IN: molecular_function unknown; INVOLVED IN: biological_process unknown; LOCATED IN: vacuole; EXPRESSED IN: 24 plant structures; EXPRESSED DURING: 15 growth stages; CONTAINS InterPro DOMAIN/s: Stress up-regulated Nod 19 (InterPro:IPR011692); Has 27 Blast hits to 27 proteins in 10 species: Archae - 0; Bacteria - 0; Metazoa - 0; Fungi - 0; Plants - 26; Viruses - 0; Other Eukaryotes - 1 (source: NCBI BLink). | chr5:24834505-24836223 REVERSEmoderately similar to ( 373) loc_os08g42590 12008.m26771 protein mtN19-like protein, putative, expressed  no original description 	T
30.2.3	signalling.receptor kinases.leucine rich repeat III	MDP0000453826	 highly similar to ( 564) AT1G48480 | Symbols: RKL1 | RKL1; ATP binding / kinase/ protein serine/threonine kinase | chr1:17918475-17920743 FORWARDmoderately similar to ( 215) PSKR_DAUCA Phytosulfokine receptor precursor (EC 2.7.11.1) (Phytosulfokine LRR receptor kinase) - Daucus carota (Carrot)highly similar to ( 546) loc_os03g12250 12003.m06675 protein atypical receptor-like kinase MARK, putative, expressed     no original description 	T
27.3.99	RNA.regulation of transcription.unclassified	MDP0000612456	 highly similar to ( 635) AT2G29580 | Symbols:  | zinc finger (CCCH-type) family protein / RNA recognition motif (RRM)-containing protein | chr2:12652011-12654158 FORWARDhighly similar to ( 657) loc_os06g07350 12006.m05457 protein pre-mRNA-splicing factor SLT11, putative, expressed  no original description 	T
27.4	RNA.RNA binding	MDP0000232549	 moderately similar to ( 228) AT2G35410 | Symbols:  | 33 kDa ribonucleoprotein, chloroplast, putative / RNA-binding protein cp33, putative | chr2:14898341-14899590 FORWARDvery weakly similar to (97.4) ROC5_NICSY 33 kDa ribonucleoprotein, chloroplast precursor - Nicotiana sylvestris (Wood tobacco)weakly similar to ( 186) loc_os04g50110 12004.m09943 protein plastid-specific 30S ribosomal protein 2, chloroplast precursor, putative, expressed  no original description 	T
26.3.2	misc.gluco-, galacto- and mannosidases.beta-galactosidase	MDP0000202465	 highly similar to ( 953) AT5G63810 | Symbols: BGAL10 | BGAL10 (beta-galactosidase 10); beta-galactosidase/ catalytic/ cation binding | chr5:25537242-25541315 FORWARDhighly similar to ( 700) BGAL_ASPOF Beta-galactosidase precursor (EC 3.2.1.23) (Lactase) - Asparagus officinalis (Garden asparagus)highly similar to ( 939) loc_os01g65460 12001.m42774 protein beta-galactosidase precursor, putative, expressed  no original description 	T
35.1	not assigned.no ontology	MDP0000289460	 moderately similar to ( 426) AT3G60260 | Symbols:  | phagocytosis and cell motility protein ELMO1-related | chr3:22274303-22276839 FORWARDmoderately similar to ( 389) loc_os02g43590 12002.m09394 protein ELMO domain-containing protein 2, putative, expressed  no original description 	T
35.2	not assigned.unknown	MDP0000206216	 moderately similar to ( 260) AT4G27435 | Symbols:  | unknown protein | chr4:13723903-13724872 FORWARDmoderately similar to ( 231) loc_os07g27810 12007.m07055 protein fiber protein Fb34, putative, expressed no original description 	T
34.7	transport.phosphate	MDP0000233872	 highly similar to ( 572) AT5G20380 | Symbols: PHT4;5 | PHT4;5; inorganic phosphate transmembrane transporter | chr5:6887936-6892358 REVERSEmoderately similar to ( 325) loc_os09g38410 12009.m60241 protein sialin, putative, expressed  no original description 	T
35.2	not assigned.unknown	MDP0000119565	 moderately similar to ( 265) AT4G13750 | Symbols:  | ATP binding | chr4:7975191-7987558 FORWARDmoderately similar to ( 217) loc_os03g58910 12003.m35467 protein expressed protein  no original description 	T
27.3.25	RNA.regulation of transcription.MYB domain transcription factor family	MDP0000642761	 moderately similar to ( 201) AT5G14750 | Symbols: WER, ATMYB66, WER1 | ATMYB66 (MYB DOMAIN PROTEIN 66); DNA binding / protein binding / transcription factor/ transcription regulator | chr5:4763656-4764738 REVERSEweakly similar to ( 153) MYB1_HORVU Myb-related protein Hv1 - Hordeum vulgare (Barley)weakly similar to ( 152) loc_os06g10350 12006.m05753 protein anthocyanin regulatory C1 protein, putative, expressed   no original description 	T
34.15	transport.potassium	MDP0000853168	 nearly identical (1182) AT2G40540 | Symbols: KT2, ATKT2, SHY3, KUP2, ATKUP2, TRK2 | KT2 (POTASSIUM TRANSPORTER 2); potassium ion transmembrane transporter | chr2:16931445-16934516 FORWARDnearly identical (1074) HAK8_ORYSA Putative potassium transporter 8 (OsHAK8) - Oryza sativa (Rice)nearly identical (1074) loc_os03g21890 12003.m07593 protein potassium transporter 8, putative, expressed  no original description 	T
26.11.1	misc.alcohol dehydrogenases.cinnamyl alcohol dehydrogenase	MDP0000312930	 very weakly similar to (55.8) AT5G19440 | Symbols:  | cinnamyl-alcohol dehydrogenase, putative (CAD) | chr5:6556493-6558123 FORWARDvery weakly similar to (52.8) loc_os08g34280 12008.m07417 protein dihydroflavonol-4-reductase, putative, expressed no original description 	T
16.8.3.1	secondary metabolism.flavonoids.dihydroflavonols.dihydroflavonol 4-reductase	MDP0000312930	 very weakly similar to (55.8) AT5G19440 | Symbols:  | cinnamyl-alcohol dehydrogenase, putative (CAD) | chr5:6556493-6558123 FORWARDvery weakly similar to (52.8) loc_os08g34280 12008.m07417 protein dihydroflavonol-4-reductase, putative, expressed no original description 	T
35.2	not assigned.unknown	MDP0000791555	 moderately similar to ( 388) AT5G51920 | Symbols:  | catalytic/ pyridoxal phosphate binding | chr5:21097202-21098914 REVERSEhighly similar to ( 510) loc_os08g43180 12008.m08292 protein expressed protein  no original description 	T
26.7	misc.oxidases - copper, flavone etc	MDP0000683492	 weakly similar to ( 163) AT4G13010 | Symbols:  | oxidoreductase, zinc-binding dehydrogenase family protein | chr4:7600682-7602567 FORWARDweakly similar to ( 172) QORH_SPIOL Chloroplastic quinone-oxidoreductase homolog (EC 1.-.-.-) (ceQORH) - Spinacia oleracea (Spinach)weakly similar to ( 137) loc_os04g30420 12004.m08147 protein chloroplastic quinone-oxidoreductase, putative, expressed   no original description 	T
17.5.1	hormone metabolism.ethylene.synthesis-degradation	MDP0000229093	 moderately similar to ( 468) AT4G10490 | Symbols:  | oxidoreductase, 2OG-Fe(II) oxygenase family protein | chr4:6483900-6485179 FORWARDweakly similar to ( 194) FL3H_DIACA Naringenin,2-oxoglutarate 3-dioxygenase (EC 1.14.11.9) (Flavonone-3-hydroxylase) (F3H) (FHT) - Dianthus caryophyllus (Carnation) (Clove pink)moderately similar to ( 444) loc_os04g49210 12004.m09856 protein naringenin,2-oxoglutarate 3-dioxygenase, putative, expressed  no original description 	T
34.7	transport.phosphate	MDP0000232357	 highly similar to ( 625) AT5G35730 | Symbols:  | EXS family protein / ERD1/XPR1/SYG1 family protein | chr5:13893941-13896821 FORWARDhighly similar to ( 615) loc_os12g40340 12012.m07818 protein EXS, C-terminal, putative, expressed  no original description 	T
29.4.1.57	protein.postranslational modification.kinase.receptor like cytoplasmatic kinase VII	MDP0000156135	 highly similar to ( 870) AT1G77280 | Symbols:  | protein kinase family protein | chr1:29031468-29035882 REVERSEmoderately similar to ( 215) NORK_MEDTR Nodulation receptor kinase precursor (EC 2.7.11.1) (Does not make infections protein 2) (Symbiosis receptor-like kinase) (MtSYMRK) - Medicago truncatula (Barrel medic)highly similar to ( 502) loc_os12g07990 12012.m04786 protein ATP binding protein, putative no original description 	T
31.2	cell.division	MDP0000778575	 moderately similar to ( 361) AT3G25980 | Symbols:  | mitotic spindle checkpoint protein, putative (MAD2) | chr3:9503232-9504402 FORWARDmoderately similar to ( 355) MAD2_MAIZE Mitotic spindle checkpoint protein MAD2 - Zea mays (Maize)moderately similar to ( 367) loc_os04g40940 12004.m35349 protein mitotic spindle checkpoint protein MAD2, putative, expressed no original description 	T
29.5	protein.degradation	MDP0000143364	 moderately similar to ( 308) AT5G26110 | Symbols:  | ATP binding / protein kinase | chr5:9118239-9118990 REVERSEmoderately similar to ( 273) loc_os10g28640 12010.m05743 protein RIO1 family protein, expressed  no original description 	T
17.2.3	hormone metabolism.auxin.induced-regulated-responsive-activated	MDP0000234699	 highly similar to ( 662) AT5G65470 | Symbols:  | unknown protein | chr5:26172432-26174904 FORWARDhighly similar to ( 612) loc_os09g24570 12009.m05611 protein psRT17-1 like protein, putative, expressed  no original description 	T
35.2	not assigned.unknown	MDP0000506495	 weakly similar to ( 161) AT2G36145 | Symbols:  | unknown protein | chr2:15168571-15169373 FORWARDweakly similar to ( 175) loc_os01g64270 12001.m12542 protein expressed protein   no original description 	T
20.2.3	stress.abiotic.drought/salt	MDP0000507074	 highly similar to ( 885) AT1G26850 | Symbols:  | dehydration-responsive family protein | chr1:9301146-9303432 REVERSEhighly similar to ( 802) loc_os09g24900 12009.m22086 protein ankyrin protein kinase-like, putative, expressed   no original description 	T
13.1.6.1.10	amino acid metabolism.synthesis.aromatic aa.chorismate.dehydroquinate/shikimate dehydrogenase	MDP0000176374	 highly similar to ( 621) AT3G06350 | Symbols: EMB3004, MEE32 | MEE32 (MATERNAL EFFECT EMBRYO ARREST 32); 3-dehydroquinate dehydratase/ NADP or NADPH binding / binding / catalytic/ shikimate 5-dehydrogenase | chr3:1924536-1927701 REVERSEhighly similar to ( 584) loc_os12g34874 12012.m73984 protein shikimate dehydrogenase, putative, expressed no original description 	T
35.2	not assigned.unknown	MDP0000312770	 moderately similar to ( 484) AT2G41800 | Symbols:  | FUNCTIONS IN: molecular_function unknown; INVOLVED IN: biological_process unknown; LOCATED IN: cell wall, plant-type cell wall; EXPRESSED IN: 7 plant structures; EXPRESSED DURING: 4 anthesis, C globular stage, petal differentiation and expansion stage; CONTAINS InterPro DOMAIN/s: Protein of unknown function DUF642 (InterPro:IPR006946), Galactose-binding like (InterPro:IPR008979); BEST Arabidopsis thaliana protein match is: unknown protein (TAIR:AT2G41810.1); Has 156 Blast hits to 155 proteins in 11 species: Archae - 0; Bacteria - 0; Metazoa - 0; Fungi - 0; Plants - 156; Viruses - 0; Other Eukaryotes - 0 (source: NCBI BLink). | chr2:17436671-17438005 REVERSEmoderately similar to ( 395) loc_os03g59300 12003.m34946 protein expressed protein  no original description 	T
35.2	not assigned.unknown	MDP0000871101	 weakly similar to ( 117) AT5G59350 | Symbols:  | unknown protein | chr5:23941132-23941995 FORWARDvery weakly similar to (71.2) loc_os11g47730 12011.m28698 protein expressed protein  no original description 	T
20.2.1	stress.abiotic.heat	MDP0000254260	 nearly identical (1110) AT5G52640 | Symbols: HSP81-1, ATHS83, HSP81.1, HSP83, ATHSP90.1 | ATHSP90.1 (HEAT SHOCK PROTEIN 90.1); ATP binding / unfolded protein binding | chr5:21352542-21355147 FORWARDnearly identical (1101) HSP83_IPONI Heat shock protein 83 - Ipomoea nil (Japanese morning glory) (Pharbitis nil)nearly identical (1057) loc_os04g01740 12004.m05490 protein heat shock protein 82, putative, expressed   no original description 	T
35.2	not assigned.unknown	MDP0000588582	 moderately similar to ( 205) AT1G68220 | Symbols:  | unknown protein | chr1:25570322-25571562 FORWARDweakly similar to ( 161) loc_os08g16130 12008.m05736 protein fiber protein Fb34, putative, expressed  no original description 	T
10.8.1	cell wall.pectin*esterases.PME	MDP0000173284	 highly similar to ( 664) AT5G09760 | Symbols:  | pectinesterase family protein | chr5:3032446-3034364 FORWARDmoderately similar to ( 290) PME2_CITSI Pectinesterase-2 precursor (EC 3.1.1.11) (Pectin methylesterase) (PE) - Citrus sinensis (Sweet orange)moderately similar to ( 340) loc_os04g35770 12004.m08666 protein pectinesterase-2 precursor, putative, expressed   no original description 	T
35.2	not assigned.unknown	MDP0000139823	 weakly similar to ( 131) AT1G22270 | Symbols:  | unknown protein | chr1:7865548-7865922 FORWARDvery weakly similar to (98.6) loc_os07g43020 12007.m08531 protein expressed protein no original description 	T
26.21	misc.protease inhibitor/seed storage/lipid transfer protein (LTP) family protein	MDP0000137366	 moderately similar to ( 363) AT1G67325 | Symbols:  | binding / zinc ion binding | chr1:25209825-25212412 REVERSEmoderately similar to ( 287) loc_os08g41010 12008.m26752 protein Zn-finger, RanBP-type, containing protein, expressed no original description 	T
35.2	not assigned.unknown	MDP0000329147	 moderately similar to ( 330) AT2G37110 | Symbols:  | unknown protein | chr2:15592689-15593726 FORWARDmoderately similar to ( 291) loc_os01g06460 12001.m07276 protein SAT5, putative, expressed  no original description 	T
4.1.14	glycolysis.cytosolic branch.pyruvate kinase (PK)	MDP0000128626	 weakly similar to ( 172) AT3G52990 | Symbols:  | pyruvate kinase, putative | chr3:19649336-19652237 FORWARDvery weakly similar to (68.6) KPYC_TOBAC Pyruvate kinase, cytosolic isozyme (EC 2.7.1.40) (PK) - Nicotiana tabacum (Common tobacco)weakly similar to ( 172) loc_os12g05110 12012.m26837 protein pyruvate kinase, cytosolic isozyme, putative, expressed no original description 	T
33.99	development.unspecified	MDP0000554317	 weakly similar to ( 105) AT5G20700 | Symbols:  | senescence-associated protein-related | chr5:7006178-7007003 REVERSEweakly similar to ( 119) loc_os02g07820 12002.m06128 protein expressed protein  no original description 	T
34.8	transport.metabolite transporters at the envelope membrane	MDP0000321440	 highly similar to ( 571) AT5G46110 | Symbols: APE2, TPT | APE2 (ACCLIMATION OF PHOTOSYNTHESIS TO ENVIRONMENT 2); antiporter/ triose-phosphate transmembrane transporter | chr5:18697606-18700223 FORWARDhighly similar to ( 582) TPT_PEA Triose phosphate/phosphate translocator, chloroplast precursor (cTPT) (p36) (E30) - Pisum sativum (Garden pea)highly similar to ( 526) loc_os01g13770 12001.m150534 protein triose phosphate/phosphate translocator, chloroplast precursor, putative, expressed  TrbL  no original description 	T
31.3.1	cell.cycle.peptidylprolyl isomerase	MDP0000482092	 moderately similar to ( 372) AT3G15520 | Symbols:  | peptidyl-prolyl cis-trans isomerase TLP38, chloroplast / thylakoid lumen PPIase of 38 kDa / cyclophilin / rotamase | chr3:5249739-5252432 REVERSEvery weakly similar to (51.6) TLP40_SPIOL Peptidyl-prolyl cis-trans isomerase, chloroplast precursor (EC 5.2.1.8) (40 kDa thylakoid lumen PPIase) (40 kDa thylakoid lumen rotamase) - Spinacia oleracea (Spinach)moderately similar to ( 333) loc_os07g37830 12007.m08032 protein peptidyl-prolyl cis-trans isomerase CYP37, chloroplast precursor, putative, expressed  no original description 	T
9.9	mitochondrial electron transport / ATP synthesis.F1-ATPase 	MDP0000545884	 moderately similar to ( 256) AT3G52300 | Symbols: ATPQ | ATPQ (ATP SYNTHASE D CHAIN, MITOCHONDRIAL) | chr3:19396689-19397866 FORWARDmoderately similar to ( 238) loc_os08g37320 12008.m26713 protein ATP synthase D chain, mitochondrial, putative, expressed   no original description 	T
13.1.6.1.5	amino acid metabolism.synthesis.aromatic aa.chorismate.shikimate kinase	MDP0000303078	 moderately similar to ( 285) AT2G21940 | Symbols:  | shikimate kinase, putative | chr2:9351106-9352881 FORWARDmoderately similar to ( 277) loc_os04g54800 12004.m35210 protein shikimate kinase, chloroplast precursor, putative, expressed no original description 	T
29.4	protein.postranslational modification	MDP0000284260	 highly similar to ( 647) AT5G25510 | Symbols:  | serine/threonine protein phosphatase 2A (PP2A) regulatory subunit B', putative | chr5:8882728-8884325 REVERSEhighly similar to ( 609) loc_os05g48150 12005.m08886 protein transposon protein, putative, unclassified, expressed    no original description 	T
27.1.2	RNA.processing.RNA helicase	MDP0000315398	 moderately similar to ( 206) AT1G05460 | Symbols: SDE3 | SDE3 (SILENCING DEFECTIVE); RNA helicase | chr1:1601357-1604658 REVERSEmoderately similar to ( 217) loc_os03g06440 12003.m35028 protein RNA helicase SDE3, putative, expressed  no original description 	T
17.5.1	hormone metabolism.ethylene.synthesis-degradation	MDP0000711591	 weakly similar to ( 172) AT1G79760 | Symbols: DTA4 | DTA4 (DOWNSTREAM TARGET OF AGL15-4) | chr1:30012054-30013042 FORWARDvery weakly similar to (63.5) ACCO1_MALDO 1-aminocyclopropane-1-carboxylate oxidase 1 (EC 1.14.17.4) (ACC oxidase 1) (Ethylene-forming enzyme) (EFE) (Protein AP4) (PAE12) - Malus domestica (Apple) (Malus sylvestris)weakly similar to ( 141) loc_os01g54090 12001.m11573 protein expressed protein  no original description 	T
16.8.1	secondary metabolism.flavonoids.anthocyanins	MDP0000711591	 weakly similar to ( 172) AT1G79760 | Symbols: DTA4 | DTA4 (DOWNSTREAM TARGET OF AGL15-4) | chr1:30012054-30013042 FORWARDvery weakly similar to (63.5) ACCO1_MALDO 1-aminocyclopropane-1-carboxylate oxidase 1 (EC 1.14.17.4) (ACC oxidase 1) (Ethylene-forming enzyme) (EFE) (Protein AP4) (PAE12) - Malus domestica (Apple) (Malus sylvestris)weakly similar to ( 141) loc_os01g54090 12001.m11573 protein expressed protein  no original description 	T
35.2	not assigned.unknown	MDP0000939369	 moderately similar to ( 266) AT4G34340 | Symbols: TAF8 | TAF8 (TBP-ASSOCIATED FACTOR 8); DNA binding | chr4:16426771-16427832 REVERSEmoderately similar to ( 224) loc_os12g38620 12012.m26943 protein bromodomain associated family protein, expressed  no original description 	T
29.2.1.2.2.14	protein.synthesis.ribosomal protein.eukaryotic.60S subunit.L14	MDP0000319645	 moderately similar to ( 201) AT2G20450 | Symbols:  | 60S ribosomal protein L14 (RPL14A) | chr2:8813923-8815071 FORWARDmoderately similar to ( 201) RL14_PEA Probable 60 ribosomal protein L14 (Hydroxyproline-rich glycoprotein HRGP1) - Pisum sativum (Garden pea)moderately similar to ( 209) loc_os04g43540 12004.m09314 protein 60 ribosomal protein L14, putative, expressed  no original description 	T
30.2.11	signalling.receptor kinases.leucine rich repeat XI	MDP0000295496	 highly similar to ( 597) AT1G35710 | Symbols:  | leucine-rich repeat transmembrane protein kinase, putative | chr1:13220940-13224386 FORWARDmoderately similar to ( 329) RPK1_IPONI Receptor-like protein kinase precursor (EC 2.7.11.1) - Ipomoea nil (Japanese morning glory) (Pharbitis nil)highly similar to ( 555) loc_os10g02970 12010.m03714 protein receptor-like protein kinase precursor, putative CobN  no original description 	T
21.1	redox.thioredoxin	MDP0000924844	 highly similar to ( 530) AT2G47470 | Symbols: ATPDIL2-1, UNE5, MEE30, PDI11, ATPDI11 | UNE5 (UNFERTILIZED EMBRYO SAC 5); protein disulfide isomerase | chr2:19481503-19483683 FORWARDhighly similar to ( 554) PDIA6_MEDSA Probable protein disulfide-isomerase A6 precursor (EC 5.3.4.1) (P5) - Medicago sativa (Alfalfa)highly similar to ( 513) loc_os01g23740 12001.m43001 protein OsPDIL2-2 - Oryza sativa protein disulfide isomerase, expressed  no original description 	T
4.2.14	glycolysis.plastid branch.pyruvate kinase (PK)	MDP0000193946	 highly similar to ( 917) AT3G52990 | Symbols:  | pyruvate kinase, putative | chr3:19649336-19652237 FORWARDmoderately similar to ( 375) KPYC_SOLTU Pyruvate kinase, cytosolic isozyme (EC 2.7.1.40) (PK) - Solanum tuberosum (Potato)highly similar to ( 924) loc_os11g05110 12011.m079911 protein pyruvate kinase, cytosolic isozyme, putative, expressed   no original description 	T
26.28	misc.GDSL-motif lipase	MDP0000496435	 highly similar to ( 561) AT5G33370 | Symbols:  | GDSL-motif lipase/hydrolase family protein | chr5:12602864-12604670 REVERSEweakly similar to ( 164) APG_BRANA Anter-specific proline-rich protein APG (Protein CEX) (Fragment) - Brassica napus (Rape)highly similar to ( 504) loc_os02g40440 12002.m09130 protein anther-specific proline-rich protein APG precursor, putative, expressed   no original description 	T
30.2.11	signalling.receptor kinases.leucine rich repeat XI	MDP0000565292	 moderately similar to ( 270) AT2G33500 | Symbols:  | zinc finger (B-box type) family protein | chr2:14188159-14190046 REVERSEvery weakly similar to (75.1) RPK1_IPONI Receptor-like protein kinase precursor (EC 2.7.11.1) - Ipomoea nil (Japanese morning glory) (Pharbitis nil)weakly similar to ( 177) loc_os01g59440 12001.m43236 protein BRASSINOSTEROID INSENSITIVE 1-associated receptor kinase 1 precursor, putative, expressed    MSP1_C PRK08026  no original description 	T
27.3.7	RNA.regulation of transcription.C2C2(Zn) CO-like, Constans-like zinc finger family	MDP0000565292	 moderately similar to ( 270) AT2G33500 | Symbols:  | zinc finger (B-box type) family protein | chr2:14188159-14190046 REVERSEvery weakly similar to (75.1) RPK1_IPONI Receptor-like protein kinase precursor (EC 2.7.11.1) - Ipomoea nil (Japanese morning glory) (Pharbitis nil)weakly similar to ( 177) loc_os01g59440 12001.m43236 protein BRASSINOSTEROID INSENSITIVE 1-associated receptor kinase 1 precursor, putative, expressed    MSP1_C PRK08026  no original description 	T
30.2.11	signalling.receptor kinases.leucine rich repeat XI	MDP0000212313	 nearly identical (1121) AT5G49660 | Symbols:  | leucine-rich repeat transmembrane protein kinase, putative | chr5:20161401-20164534 REVERSEmoderately similar to ( 388) RPK1_IPONI Receptor-like protein kinase precursor (EC 2.7.11.1) - Ipomoea nil (Japanese morning glory) (Pharbitis nil)highly similar to ( 907) loc_os12g43660 12012.m08140 protein receptor-like protein kinase 5 precursor, putative, expressed  no original description 	T
35.1	not assigned.no ontology	MDP0000421222	 moderately similar to ( 362) AT2G45010 | Symbols:  | unknown protein | chr2:18568782-18569615 FORWARDmoderately similar to ( 355) loc_os03g01210 12003.m35650 protein domain of unknown function DUF614 containing protein, expressed   no original description 	T
29.2.1.2.1.5	protein.synthesis.ribosomal protein.eukaryotic.40S subunit.S5	MDP0000130128	 moderately similar to ( 322) AT2G37270 | Symbols: ATRPS5B | ATRPS5B (RIBOSOMAL PROTEIN 5B); structural constituent of ribosome | chr2:15647883-15649042 REVERSEmoderately similar to ( 325) RS5_CICAR 40S ribosomal protein S5 (Fragment) - Cicer arietinum (Chickpea) (Garbanzo)moderately similar to ( 318) loc_os11g29190 12011.m080000 protein 40S ribosomal protein S5, putative, expressed  no original description 	T
26.6	misc.O-methyl transferases	MDP0000185626	 highly similar to ( 555) AT4G29510 | Symbols: ATPRMT11, PRMT11, ATPRMT1B, PRMT1B | PRMT11 (ARGININE METHYLTRANSFERASE 11); protein-arginine N-methyltransferase | chr4:14491739-14493752 FORWARDhighly similar to ( 562) loc_os09g19560 12009.m05164 protein protein arginine N-methyltransferase 1, putative, expressed  no original description 	T
35.2	not assigned.unknown	MDP0000209004	 moderately similar to ( 357) AT4G39860 | Symbols:  | unknown protein | chr4:18499909-18501472 FORWARDmoderately similar to ( 305) loc_os02g36500 12002.m08737 protein expressed protein no original description 	T
27.3.25	RNA.regulation of transcription.MYB domain transcription factor family	MDP0000911051	 weakly similar to ( 158) AT3G01140 | Symbols: MYB106, AtMYB106 | MYB106 (myb domain protein 106); DNA binding / transcription factor | chr3:46619-48143 REVERSEvery weakly similar to (94.0) MYB1_PHYPA Myb-related protein Pp1 (Fragment) - Physcomitrella patens (Moss)weakly similar to ( 120) loc_os08g33660 12008.m07356 protein MYB1, putative, expressed  no original description 	T
15.2	metal handling.binding, chelation and storage	MDP0000325832	 moderately similar to ( 288) AT2G40300 | Symbols: ATFER4 | ATFER4 (ferritin 4); binding / ferric iron binding / oxidoreductase/ transition metal ion binding | chr2:16831501-16833214 REVERSEmoderately similar to ( 317) FRI3_SOYBN Ferritin-3, chloroplast precursor (EC 1.16.3.1) (SFerH-3) - Glycine max (Soybean)moderately similar to ( 274) loc_os12g01530 12012.m26727 protein ferritin-1, chloroplast precursor, putative, expressed   no original description 	T
10.2.1	cell wall.cellulose synthesis.cellulose synthase	MDP0000279461	 nearly identical (1342) AT4G32410 | Symbols: CESA1, RSW1 | CESA1 (CELLULOSE SYNTHASE 1); cellulose synthase/ transferase, transferring glycosyl groups | chr4:15641009-15646388 REVERSEnearly identical (1326) loc_os05g08370 12005.m05354 protein CESA1 - cellulose synthase, expressed   no original description 	T
17.6.3	hormone metabolism.gibberelin.induced-regulated-responsive-activated	MDP0000140078	 weakly similar to ( 104) AT1G75750 | Symbols: GASA1 | GASA1 (GAST1 PROTEIN HOMOLOG 1) | chr1:28441813-28442284 REVERSEvery weakly similar to (98.2) loc_os05g35690 12005.m07799 protein gibberellin-regulated protein 2 precursor, putative, expressed no original description 	T
29.5.5	protein.degradation.serine protease	MDP0000940110	 highly similar to ( 677) AT4G36195 | Symbols:  | serine carboxypeptidase S28 family protein | chr4:17127202-17129787 FORWARDhighly similar to ( 675) loc_os10g36760 12010.m06482 protein prolyl carboxypeptidase like protein, putative, expressed  no original description 	T
17.2.3	hormone metabolism.auxin.induced-regulated-responsive-activated	MDP0000148873	 moderately similar to ( 341) AT4G27450 | Symbols:  | unknown protein | chr4:13727665-13728683 REVERSEvery weakly similar to (95.5) TSJT1_TOBAC Stem-specific protein TSJT1 - Nicotiana tabacum (Common tobacco)weakly similar to ( 182) loc_os03g53270 12003.m35408 protein stem-specific protein TSJT1, putative, expressed  no original description 	T
35.2	not assigned.unknown	MDP0000174775	 moderately similar to ( 263) AT2G35790 | Symbols:  | unknown protein | chr2:15040735-15041976 FORWARDmoderately similar to ( 247) loc_os03g63680 12003.m11236 protein expressed protein no original description 	T
35.2	not assigned.unknown	MDP0000838695	 very weakly similar to (55.5) AT5G48480 | Symbols:  | unknown protein | chr5:19644814-19645658 FORWARD  no original description 	T
29.2.1.2.2.523	protein.synthesis.ribosomal protein.eukaryotic.60S subunit.L23A	MDP0000203623	 moderately similar to ( 207) AT2G39460 | Symbols: ATRPL23A, RPL23A, RPL23AA | RPL23AA (RIBOSOMAL PROTEIN L23AA); RNA binding / nucleotide binding / structural constituent of ribosome | chr2:16475049-16475904 FORWARDmoderately similar to ( 207) RL23A_DAUCA 60S ribosomal protein L23a - Daucus carota (Carrot)weakly similar to ( 178) loc_os04g42270 12004.m35367 protein 60S ribosomal protein L23a, putative, expressed no original description 	T
34.6	transport.sulphate	MDP0000167489	 nearly identical (1080) AT1G79990 | Symbols:  | protein binding / structural molecule | chr1:30085910-30091949 FORWARDmoderately similar to ( 339) NO70_SOYBN Early nodulin 70 - Glycine max (Soybean)nearly identical (1238) loc_os06g05180 12006.m71402 protein coatomer subunit beta, putative, expressed   no original description 	T
1.1.1.2	PS.lightreaction.photosystem II.PSII polypeptide subunits	MDP0000340790	 moderately similar to ( 342) AT5G11450 | Symbols:  | oxygen-evolving complex-related | chr5:3654475-3656357 FORWARDmoderately similar to ( 329) loc_os01g43070 12001.m42679 protein expressed protein no original description 	T
27.3.20	RNA.regulation of transcription.G2-like transcription factor family, GARP	MDP0000321302	 weakly similar to ( 111) AT2G40260 | Symbols:  | myb family transcription factor | chr2:16816818-16818473 REVERSEweakly similar to ( 112) loc_os04g47890 12004.m09729 protein myb-like DNA-binding domain, SHAQKYF class family protein, expressed no original description 	T
1.3.4	PS.calvin cycle.GAP	MDP0000152497	 highly similar to ( 915) AT2G24270 | Symbols: ALDH11A3 | ALDH11A3; 3-chloroallyl aldehyde dehydrogenase/ glyceraldehyde-3-phosphate dehydrogenase (NADP+) | chr2:10327325-10329601 REVERSEhighly similar to ( 920) GAPN_NICPL NADP-dependent glyceraldehyde-3-phosphate dehydrogenase (EC 1.2.1.9) (Non-phosphorylating glyceraldehyde 3-phosphate dehydrogenase) (Glyceraldehyde-3-phosphate dehydrogenase [NADP+]) (Triosephosphate dehydrogenase) - Nicotiana plumbaginhighly similar to ( 897) loc_os08g34210 12008.m26702 protein NADP-dependent glyceraldehyde-3-phosphate dehydrogenase, putative, expressed   no original description 	T
5.10	fermentation.aldehyde dehydrogenase	MDP0000152497	 highly similar to ( 915) AT2G24270 | Symbols: ALDH11A3 | ALDH11A3; 3-chloroallyl aldehyde dehydrogenase/ glyceraldehyde-3-phosphate dehydrogenase (NADP+) | chr2:10327325-10329601 REVERSEhighly similar to ( 920) GAPN_NICPL NADP-dependent glyceraldehyde-3-phosphate dehydrogenase (EC 1.2.1.9) (Non-phosphorylating glyceraldehyde 3-phosphate dehydrogenase) (Glyceraldehyde-3-phosphate dehydrogenase [NADP+]) (Triosephosphate dehydrogenase) - Nicotiana plumbaginhighly similar to ( 897) loc_os08g34210 12008.m26702 protein NADP-dependent glyceraldehyde-3-phosphate dehydrogenase, putative, expressed   no original description 	T
19.3	tetrapyrrole synthesis.GSA	MDP0000315935	 highly similar to ( 737) AT3G48730 | Symbols: GSA2 | GSA2 (glutamate-1-semialdehyde 2,1-aminomutase 2); catalytic/ glutamate-1-semialdehyde 2,1-aminomutase/ pyridoxal phosphate binding / transaminase | chr3:18049697-18051550 FORWARDhighly similar to ( 743) GSA_TOBAC Glutamate-1-semialdehyde 2,1-aminomutase, chloroplast precursor (EC 5.4.3.8) (GSA) (Glutamate-1-semialdehyde aminotransferase) (GSA-AT) - Nicotiana tabacum (Common tobacco)highly similar to ( 698) loc_os08g41990 12008.m08177 protein glutamate-1-semialdehyde 2,1-aminomutase, chloroplast precursor, putative, expressed   no original description 	T
26.3	misc.gluco-, galacto- and mannosidases	MDP0000047589	 highly similar to ( 544) AT2G44450 | Symbols: BGLU15 | BGLU15 (BETA GLUCOSIDASE 15); catalytic/ cation binding / hydrolase, hydrolyzing O-glycosyl compounds | chr2:18340966-18343744 FORWARDmoderately similar to ( 462) BGLC_MAIZE Beta-glucosidase, chloroplast precursor (EC 3.2.1.21) (Gentiobiase) (Cellobiase) (Beta-D-glucoside glucohydrolase) - Zea mays (Maize)highly similar to ( 639) loc_os04g39880 12004.m35159 protein non-cyanogenic beta-glucosidase precursor, putative, expressed  no original description 	T
27.1	RNA.processing	MDP0000131323	 highly similar to ( 509) AT2G34750 | Symbols:  | RNA polymerase I specific transcription initiation factor RRN3 family protein | chr2:14660449-14663792 REVERSEmoderately similar to ( 458) loc_os06g47350 12006.m31918 protein RNA polymerase I transcription factor, putative, expressed   no original description 	T
27.3.25	RNA.regulation of transcription.MYB domain transcription factor family	MDP0000124555	 weakly similar to ( 174) AT5G14750 | Symbols: WER, ATMYB66, WER1 | ATMYB66 (MYB DOMAIN PROTEIN 66); DNA binding / protein binding / transcription factor/ transcription regulator | chr5:4763656-4764738 REVERSEweakly similar to ( 174) MYBC_MAIZE Anthocyanin regulatory C1 protein - Zea mays (Maize)weakly similar to ( 168) loc_os03g29614 12003.m08231 protein anthocyanin regulatory C1 protein, putative, expressed  no original description 	T
13.2.3.2	amino acid metabolism.degradation.aspartate family.threonine	MDP0000176217	 moderately similar to ( 485) AT1G67280 | Symbols:  | lactoylglutathione lyase, putative / glyoxalase I, putative | chr1:25188563-25190134 REVERSEmoderately similar to ( 408) LGUL_ORYSA Lactoylglutathione lyase (EC 4.4.1.5) (Methylglyoxalase) (Aldoketomutase) (Glyoxalase I) (Glx I) (Ketone-aldehyde mutase) (S-D-lactoylglutathione methylglyoxal lyase) (Allergen Ory s ?) (Allergen Glb33) (PP33) - Oryza sativa (Rice)moderately similar to ( 476) loc_os05g14194 12005.m27746 protein lactoylglutathione lyase, putative, expressed  no original description 	T
26.2	misc.UDP glucosyl and glucoronyl transferases	MDP0000915151	 highly similar to ( 555) AT1G22400 | Symbols: UGT85A1, ATUGT85A1 | UGT85A1; UDP-glycosyltransferase/ cis-zeatin O-beta-D-glucosyltransferase/ glucuronosyltransferase/ trans-zeatin O-beta-D-glucosyltransferase/ transferase, transferring glycosyl groups / transferase, transferring hexosyl groups | chr1:7903851-7906607 REVERSEweakly similar to ( 181) UFO2_MAIZE Anthocyanidin 3-O-glucosyltransferase (EC 2.4.1.115) (Flavonol 3-O-glucosyltransferase) (UDP-glucose flavonoid 3-O-glucosyltransferase) (Bronze-1) (Bz-Mc2 allele) - Zea mays (Maize)highly similar to ( 521) loc_os02g51910 12002.m10222 protein cytokinin-O-glucosyltransferase 2, putative, expressed  no original description 	T
33.99	development.unspecified	MDP0000497953	 highly similar to ( 597) AT5G45275 | Symbols:  | FUNCTIONS IN: molecular_function unknown; INVOLVED IN: biological_process unknown; LOCATED IN: endomembrane system; EXPRESSED IN: cultured cell; CONTAINS InterPro DOMAIN/s: Nodulin-like (InterPro:IPR010658), Major facilitator superfamily, general substrate transporter (InterPro:IPR016196); BEST Arabidopsis thaliana protein match is: nodulin-related (TAIR:AT4G19450.1); Has 697 Blast hits to 659 proteins in 149 species: Archae - 19; Bacteria - 126; Metazoa - 5; Fungi - 146; Plants - 324; Viruses - 0; Other Eukaryotes - 77 (source: NCBI BLink). | chr5:18334476-18337240 REVERSEmoderately similar to ( 455) loc_os11g01590 12011.m04362 protein major Facilitator Superfamily protein, expressed   no original description 	T
10.6.3	cell wall.degradation.pectate lyases and polygalacturonases	MDP0000491898	 highly similar to ( 652) AT1G67750 | Symbols:  | pectate lyase family protein | chr1:25401660-25403165 FORWARDmoderately similar to ( 427) PEL_LILLO Pectate lyase precursor (EC 4.2.2.2) - Lilium longiflorum (Trumpet lily)highly similar to ( 581) loc_os04g05050 12004.m35243 protein pectate lyase 8 precursor, putative, expressed   no original description 	T
27.3.3	RNA.regulation of transcription.AP2/EREBP, APETALA2/Ethylene-responsive element binding protein family	MDP0000457509	 very weakly similar to (85.9) AT1G50640 | Symbols: ERF3, ATERF3 | ERF3 (ETHYLENE RESPONSIVE ELEMENT BINDING FACTOR 3); DNA binding / protein binding / transcription factor/ transcription repressor | chr1:18757602-18758279 REVERSEvery weakly similar to (87.0) ERF3_TOBAC Ethylene-responsive transcription factor 3 (Ethylene-responsive element-binding factor 3 homolog) (EREBP-5) (NtERF5) - Nicotiana tabacum (Common tobacco)very weakly similar to (84.3) loc_os05g41760 12005.m08350 protein ethylene-responsive transcription factor 12, putative, expressed  no original description 	T
29.2.3	protein.synthesis.initiation	MDP0000436207	 moderately similar to ( 217) AT1G69410 | Symbols: ATELF5A-3, ELF5A-3 | ELF5A-3 (EUKARYOTIC ELONGATION FACTOR 5A-3); translation initiation factor | chr1:26089301-26090194 FORWARDmoderately similar to ( 234) IF5A2_MEDSA Eukaryotic translation initiation factor 5A-2 (eIF-5A-2) - Medicago sativa (Alfalfa)moderately similar to ( 232) loc_os03g55150 12003.m35635 protein eukaryotic translation initiation factor 5A-2, putative, expressed no original description 	T
10.7	cell wall.modification	MDP0000311765	 moderately similar to ( 413) AT2G36870 | Symbols:  | xyloglucan:xyloglucosyl transferase, putative / xyloglucan endotransglycosylase, putative / endo-xyloglucan transferase, putative | chr2:15472869-15474630 REVERSEmoderately similar to ( 202) XTH_TOBAC Probable xyloglucan endotransglucosylase/hydrolase protein precursor (EC 2.4.1.207) - Nicotiana tabacum (Common tobacco)moderately similar to ( 373) loc_os10g39840 12010.m06755 protein xyloglucan endotransglucosylase/hydrolase protein 32 precursor, putative, expressed   no original description 	T
29.2.1.1.1.1.10	protein.synthesis.ribosomal protein.prokaryotic.chloroplast.30S subunit.S10	MDP0000451604	 moderately similar to ( 223) AT3G13120 | Symbols:  | 30S ribosomal protein S10, chloroplast, putative | chr3:4220310-4221526 REVERSEmoderately similar to ( 236) RR10_MESCR 30S ribosomal protein S10, chloroplast precursor - Mesembryanthemum crystallinum (Common ice plant)moderately similar to ( 201) loc_os03g10060 12003.m06460 protein chloroplast 30S ribosomal protein S10, putative, expressed no original description 	T
17.5.1	hormone metabolism.ethylene.synthesis-degradation	MDP0000322095	 weakly similar to ( 120) AT3G21420 | Symbols:  | oxidoreductase, 2OG-Fe(II) oxygenase family protein | chr3:7541579-7543221 FORWARDvery weakly similar to (85.5) FLS_SOLTU Flavonol synthase/flavanone 3-hydroxylase (EC 1.14.11.23) (EC 1.14.11.9) (FLS) - Solanum tuberosum (Potato)weakly similar to ( 126) loc_os10g40934 12010.m06860 protein flavonol synthase/flavanone 3-hydroxylase, putative, expressed  no original description 	T
29.3.1	protein.targeting.nucleus	MDP0000248706	 nearly identical (1129) AT3G08947 | Symbols:  | binding / protein transporter | chr3:2724007-2726722 FORWARDnearly identical (1046) loc_os05g28510 12005.m083698 protein importin beta-1 subunit, putative, expressed  no original description 	T
30.2.17	signalling.receptor kinases.DUF 26	MDP0000211661	 moderately similar to ( 315) AT5G38280 | Symbols: PR5K | PR5K; kinase/ transmembrane receptor protein serine/threonine kinase | chr5:15293325-15295838 REVERSEweakly similar to ( 167) KPRO_MAIZE Putative receptor protein kinase ZmPK1 precursor (EC 2.7.11.1) - Zea mays (Maize)moderately similar to ( 386) loc_os01g02290 12001.m06871 protein Ser/Thr receptor-like kinase, putative, expressed  no original description 	T
29.2.1.2.2.535	protein.synthesis.ribosomal protein.eukaryotic.60S subunit.L35A	MDP0000290560	 moderately similar to ( 216) AT1G74270 | Symbols:  | 60S ribosomal protein L35a (RPL35aC) | chr1:27928415-27929466 REVERSEweakly similar to ( 199) loc_os02g54470 12002.m100389 protein 60S ribosomal protein L33-B, putative, expressed  no original description 	T
1.1.2.2	PS.lightreaction.photosystem I.PSI polypeptide subunits	MDP0000128787	 weakly similar to ( 107) AT4G28750 | Symbols: PSAE-1 | PSAE-1 (PSA E1 KNOCKOUT); catalytic | chr4:14202951-14203888 REVERSEweakly similar to ( 120) PSAEB_NICSY Photosystem I reaction center subunit IV B, chloroplast precursor (PSI-E B) [Contains: Photosystem I reaction center subunit IV B isoform 2] - Nicotiana sylvestris (Wood tobacco)weakly similar to ( 105) loc_os07g25430 12007.m29150 protein photosystem I reaction center subunit IV A, chloroplast precursor, putative, expressed  no original description 	T
34.1.1	transport.p- and v-ATPases.H+-transporting two-sector ATPase	MDP0000143682	 moderately similar to ( 247) AT4G11150 | Symbols: TUF, emb2448, TUFF, VHA-E1 | TUF (VACUOLAR ATP SYNTHASE SUBUNIT E1); proton-transporting ATPase, rotational mechanism | chr4:6800091-6801696 FORWARDmoderately similar to ( 243) VATE_GOSHI Vacuolar ATP synthase subunit E (EC 3.6.3.14) (V-ATPase E subunit) (Vacuolar proton pump E subunit) - Gossypium hirsutum (Upland cotton)moderately similar to ( 244) loc_os01g46980 12001.m10894 protein vacuolar ATP synthase subunit E, putative, expressed  no original description 	T
30.3	signalling.calcium	MDP0000224985	 very weakly similar to (53.5) loc_os01g72100 12001.m13246 protein polcalcin Jun o 2, putative, expressed  no original description 	T
26.16	misc.myrosinases-lectin-jacalin	MDP0000232997	 weakly similar to ( 198) AT5G01090 | Symbols:  | legume lectin family protein | chr5:33055-34116 FORWARDvery weakly similar to (51.6) ARC1_PHAVU Arcelin-1 precursor - Phaseolus vulgaris (Kidney bean) (French bean)very weakly similar to (76.3) loc_os06g17490 12006.m06461 protein protein kinase, putative, expressed   no original description 	T
27.3.35	RNA.regulation of transcription.bZIP transcription factor family	MDP0000555457	 moderately similar to ( 268) AT2G42380 | Symbols: ATBZIP34, BZIP34 | bZIP transcription factor family protein | chr2:17647205-17648660 REVERSEvery weakly similar to (90.5) RF2B_ORYSA Transcription factor RF2b - Oryza sativa (Rice)weakly similar to ( 185) loc_os01g11350 12001.m07754 protein transcription factor RF2b, putative, expressed  no original description 	T
29.2.1.1.3.1.6	protein.synthesis.ribosomal protein.prokaryotic.unknown organellar.30S subunit.S6	MDP0000551907	 weakly similar to ( 183) AT3G18760 | Symbols:  | ribosomal protein S6 family protein | chr3:6457930-6459558 REVERSEweakly similar to ( 167) loc_os01g12090 12001.m07824 protein expressed protein no original description 	T
35.2	not assigned.unknown	MDP0000424873	 weakly similar to ( 175) AT3G09085 | Symbols:  | unknown protein | chr3:2782238-2782576 FORWARDweakly similar to ( 136) loc_os03g15910 12003.m07028 protein transmembrane protein, putative, expressed no original description 	T
35.2	not assigned.unknown	MDP0000196399	 moderately similar to ( 303) AT5G13240 | Symbols:  | transcription regulator | chr5:4225261-4227152 REVERSEmoderately similar to ( 252) loc_os04g56730 12004.m35220 protein repressor of RNA polymerase III transcription MAF1, putative, expressed  no original description 	T
27.2	RNA.transcription	MDP0000589856	 nearly identical (1320) AT1G14790 | Symbols: RDR1, ATRDRP1 | RDR1 (RNA-DEPENDENT RNA POLYMERASE 1); RNA-directed RNA polymerase/ nucleic acid binding | chr1:5094317-5097817 REVERSEhighly similar to ( 949) loc_os02g50330 12002.m10067 protein RNA-directed RNA polymerase 2, putative, expressed  no original description 	T
30.2.17	signalling.receptor kinases.DUF 26	MDP0000275440	 highly similar to ( 782) AT1G11410 | Symbols:  | S-locus protein kinase, putative | chr1:3841286-3844284 FORWARDmoderately similar to ( 283) SLSG3_BRAOL S-locus-specific glycoprotein S13 precursor (SLSG-13) (Fragment) - Brassica oleracea (Wild cabbage)highly similar to ( 658) loc_os07g36544 12007.m07905 protein serine/threonine-protein kinase receptor precursor, putative, expressed  no original description 	T
27.4	RNA.RNA binding	MDP0000270177	 moderately similar to ( 254) AT3G59550 | Symbols: SYN3, ATRAD21.2, ATSYN3 | SYN3 | chr3:21997262-22000496 FORWARDmoderately similar to ( 213) RRP2_SPIOL Plastid-specific 30S ribosomal protein 2, chloroplast precursor (PSRP-2) - Spinacia oleracea (Spinach)weakly similar to ( 193) loc_os08g16610 12008.m26637 protein sister chromatid cohesion 1 protein 3, putative, expressed   no original description 	T
34.16	transport.ABC transporters and multidrug resistance systems	MDP0000259421	 nearly identical (1597) AT1G15520 | Symbols: PDR12, ATPDR12 | PDR12 (PLEIOTROPIC DRUG RESISTANCE 12); ATPase, coupled to transmembrane movement of substances | chr1:5331993-5338175 REVERSEnearly identical (1657) PDR1_TOBAC Pleiotropic drug resistance protein 1 (NtPDR1) - Nicotiana tabacum (Common tobacco)nearly identical (1571) loc_os02g11760 12002.m06425 protein PDR5-like ABC transporter, putative, expressed   no original description 	T
26.10	misc.cytochrome P450	MDP0000155194	 highly similar to ( 574) AT3G56630 | Symbols: CYP94D2 | CYP94D2; electron carrier/ heme binding / iron ion binding / monooxygenase/ oxygen binding | chr3:20978953-20980512 FORWARDvery weakly similar to (94.0) C97B2_SOYBN Cytochrome P450 97B2 (EC 1.14.-.-) - Glycine max (Soybean)highly similar to ( 519) loc_os01g59020 12001.m12041 protein cytochrome P450 94A2, putative, expressed   no original description 	T
26.3	misc.gluco-, galacto- and mannosidases	MDP0000262060	 highly similar to ( 726) AT3G55270 | Symbols: MKP1, ATMKP1 | MKP1 (MITOGEN-ACTIVATED PROTEIN KINASE PHOSPHATASE 1); MAP kinase tyrosine/serine/threonine phosphatase | chr3:20496775-20499408 FORWARDmoderately similar to ( 386) BGLC_MAIZE Beta-glucosidase, chloroplast precursor (EC 3.2.1.21) (Gentiobiase) (Cellobiase) (Beta-D-glucoside glucohydrolase) - Zea mays (Maize)highly similar to ( 589) loc_os05g02510 12005.m04787 protein beta-hexosaminidase beta chain precursor, putative, expressed  no original description 	T
17.4.1	hormone metabolism.cytokinin.synthesis-degradation	MDP0000013380	 moderately similar to ( 339) AT3G63110 | Symbols: ATIPT3, IPT3 | ATIPT3 (ARABIDOPSIS THALIANA ISOPENTENYLTRANSFERASE 3); ATP binding / tRNA isopentenyltransferase/ transferase, transferring alkyl or aryl (other than methyl) groups | chr3:23318437-23319447 REVERSEmoderately similar to ( 262) loc_os03g59570 12003.m10854 protein cytokinin synthase, putative, expressed  no original description 	T
27.4	RNA.RNA binding	MDP0000302917	 weakly similar to ( 149) AT5G61030 | Symbols: GR-RBP3 | GR-RBP3 (glycine-rich RNA-binding protein 3); ATP binding / RNA binding | chr5:24560870-24562152 FORWARDvery weakly similar to (86.3) ROC3_NICSY 28 kDa ribonucleoprotein, chloroplast precursor (28RNP) - Nicotiana sylvestris (Wood tobacco)weakly similar to ( 124) loc_os10g17454 12010.m065240 protein RNA-binding protein precursor, putative, expressed  no original description 	T
29.2.1.1.3.2.32	protein.synthesis.ribosomal protein.prokaryotic.unknown organellar.50S subunit.L32	MDP0000214200	 very weakly similar to (99.0) AT1G26740 | Symbols:  | structural constituent of ribosome | chr1:9245280-9246555 REVERSEvery weakly similar to (63.9) loc_os10g26690 12010.m05608 protein ribosomal protein L32 containing protein, expressed   no original description 	T
9.9	mitochondrial electron transport / ATP synthesis.F1-ATPase 	MDP0000205940	 highly similar to ( 919) AT4G38510 | Symbols:  | vacuolar ATP synthase subunit B, putative / V-ATPase B subunit, putative / vacuolar proton pump B subunit, putative / V-ATPase 57 kDa subunit, putative | chr4:18011155-18014789 REVERSEhighly similar to ( 912) VATB2_HORVU Vacuolar ATP synthase subunit B isoform 2 (EC 3.6.3.14) (V-ATPase B subunit 2) (Vacuolar proton pump B subunit 2) - Hordeum vulgare (Barley)highly similar to ( 915) loc_os01g51380 12001.m150697 protein vacuolar ATP synthase subunit B isoform 1, putative, expressed   no original description 	T
34.1.1.1	transport.p- and v-ATPases.H+-transporting two-sector ATPase.subunit B	MDP0000205940	 highly similar to ( 919) AT4G38510 | Symbols:  | vacuolar ATP synthase subunit B, putative / V-ATPase B subunit, putative / vacuolar proton pump B subunit, putative / V-ATPase 57 kDa subunit, putative | chr4:18011155-18014789 REVERSEhighly similar to ( 912) VATB2_HORVU Vacuolar ATP synthase subunit B isoform 2 (EC 3.6.3.14) (V-ATPase B subunit 2) (Vacuolar proton pump B subunit 2) - Hordeum vulgare (Barley)highly similar to ( 915) loc_os01g51380 12001.m150697 protein vacuolar ATP synthase subunit B isoform 1, putative, expressed   no original description 	T
29.8	protein.assembly and cofactor ligation	MDP0000146962	 moderately similar to ( 418) AT5G54290 | Symbols:  | cytochrome c biogenesis protein family | chr5:22051097-22053923 FORWARDmoderately similar to ( 416) loc_os11g04500 12011.m04648 protein expressed protein no original description 	T
4.1.7	glycolysis.cytosolic branch.triosephosphate isomerase (TPI)	MDP0000924958	 moderately similar to ( 362) AT3G55440 | Symbols: ATCTIMC, TPI | TPI (TRIOSEPHOSPHATE ISOMERASE); triose-phosphate isomerase | chr3:20553794-20556078 FORWARDmoderately similar to ( 374) TPIS_PETHY Triosephosphate isomerase, cytosolic (EC 5.3.1.1) (TIM) (Triose-phosphate isomerase) - Petunia hybrida (Petunia)moderately similar to ( 363) loc_os01g05490 12001.m42862 protein triosephosphate isomerase, cytosolic, putative, expressed no original description 	T
29.4.1.57	protein.postranslational modification.kinase.receptor like cytoplasmatic kinase VII	MDP0000852480	 highly similar to ( 984) AT5G24010 | Symbols:  | protein kinase family protein | chr5:8113910-8116384 FORWARDmoderately similar to ( 242) CRI4_MAIZE Putative receptor protein kinase CRINKLY4 precursor (EC 2.7.11.1) - Zea mays (Maize)highly similar to ( 719) loc_os06g22810 12006.m06934 protein protein kinase, putative, expressed  no original description 	T
26.9	misc.glutathione S transferases	MDP0000096349	 moderately similar to ( 282) AT2G47730 | Symbols: ATGSTF8, ATGSTF5, GST6, GSTF8 | ATGSTF8 (ARABIDOPSIS THALIANA GLUTATHIONE S-TRANSFERASE PHI 8); glutathione binding / glutathione transferase | chr2:19558213-19559266 FORWARDmoderately similar to ( 292) GSTF2_TOBAC Glutathione S-transferase APIC (EC 2.5.1.18) (GST class-phi) - Nicotiana tabacum (Common tobacco)weakly similar to ( 187) loc_os01g70770 12001.m13122 protein glutathione S-transferase III, putative, expressed  no original description 	T
26.2	misc.UDP glucosyl and glucoronyl transferases	MDP0000617956	 moderately similar to ( 445) AT4G15550 | Symbols: IAGLU | IAGLU (INDOLE-3-ACETATE BETA-D-GLUCOSYLTRANSFERASE); UDP-glycosyltransferase/ transferase, transferring glycosyl groups | chr4:8877877-8879301 REVERSEmoderately similar to ( 265) IAAG_MAIZE Indole-3-acetate beta-glucosyltransferase (EC 2.4.1.121) (IAA-Glu synthetase) ((Uridine 5'-diphosphate-glucose:indol-3-ylacetyl)-beta-D-glucosyl transferase) - Zea mays (Maize)moderately similar to ( 361) loc_os01g08440 12001.m07469 protein indole-3-acetate beta-glucosyltransferase, putative, expressed     TrbL  no original description 	T
29.5.11.4.2	protein.degradation.ubiquitin.E3.RING	MDP0000311115	 weakly similar to ( 136) AT5G61550 | Symbols:  | protein kinase family protein | chr5:24748325-24751558 FORWARDvery weakly similar to (51.2) SPL11_ORYSA Spotted leaf protein 11 (Spotted leaf11) (Cell death-related protein SPL11) - Oryza sativa (Rice)weakly similar to ( 114) loc_os06g04880 12006.m31972 protein ATP binding protein, putative, expressed no original description 	T
13.1.6.1.1	amino acid metabolism.synthesis.aromatic aa.chorismate.3-deoxy-D-arabino-heptulosonate 7-phosphate synthase	MDP0000145064	 highly similar to ( 657) AT1G22410 | Symbols:  | 2-dehydro-3-deoxyphosphoheptonate aldolase, putative / 3-deoxy-D-arabino-heptulosonate 7-phosphate synthase, putative / DAHP synthetase, putative | chr1:7912120-7914742 FORWARDhighly similar to ( 665) AROF_TOBAC Phospho-2-dehydro-3-deoxyheptonate aldolase 1, chloroplast precursor (EC 2.5.1.54) (Phospho-2-keto-3-deoxyheptonate aldolase 1) (DAHP synthetase 1) (3-deoxy-D-arabino-heptulosonate 7-phosphate synthase 1) - Nicotiana tabacum (Common tobhighly similar to ( 638) loc_os07g42960 12007.m29007 protein phospho-2-dehydro-3-deoxyheptonate aldolase 1, chloroplast precursor, putative, expressed no original description 	T
29.3.4.99	protein.targeting.secretory pathway.unspecified	MDP0000290540	 highly similar to ( 693) AT1G55690 | Symbols:  | SEC14 cytosolic factor family protein / phosphoglyceride transfer family protein | chr1:20808622-20811831 REVERSEhighly similar to ( 554) loc_os08g25310 12008.m06549 protein phosphatidylinositol transfer-like protein III, putative, expressed no original description 	T
35.1.5	not assigned.no ontology.pentatricopeptide (PPR) repeat-containing protein	MDP0000297308	 highly similar to ( 850) AT4G35130 | Symbols:  | pentatricopeptide (PPR) repeat-containing protein | chr4:16721084-16723498 REVERSEweakly similar to ( 103) RF1_ORYSA Rf1 protein, mitochondrial precursor (PPR protein) (Fertility restorer) (Restorer for CMS) - Oryza sativa (Rice)highly similar to ( 724) loc_os03g04390 12003.m05969 protein vegetative storage protein, putative, expressed  no original description 	T
29.2.1.1.1.2.17	protein.synthesis.ribosomal protein.prokaryotic.chloroplast.50S subunit.L17	MDP0000159402	 moderately similar to ( 252) AT3G54210 | Symbols:  | ribosomal protein L17 family protein | chr3:20067672-20068385 REVERSEmoderately similar to ( 258) RK17_TOBAC 50S ribosomal protein L17, chloroplast precursor (CL17) - Nicotiana tabacum (Common tobacco)moderately similar to ( 230) loc_os03g60100 12003.m10904 protein 50S ribosomal protein L17, putative, expressed  no original description 	T
35.2	not assigned.unknown	MDP0000834585	 moderately similar to ( 214) AT3G44330 | Symbols:  | INVOLVED IN: protein processing; LOCATED IN: mitochondrion, endoplasmic reticulum, plasma membrane, vacuole; EXPRESSED IN: 25 plant structures; EXPRESSED DURING: 13 growth stages; CONTAINS InterPro DOMAIN/s: Nicalin (InterPro:IPR016574), EF-HAND 1 (InterPro:IPR018247), Nicastrin (InterPro:IPR008710); Has 192 Blast hits to 192 proteins in 79 species: Archae - 6; Bacteria - 5; Metazoa - 124; Fungi - 0; Plants - 26; Viruses - 0; Other Eukaryotes - 31 (source: NCBI BLink). | chr3:16006754-16010671 FORWARDmoderately similar to ( 205) loc_os08g01180 12008.m04267 protein nicalin precursor, putative, expressed  no original description 	T
35.2	not assigned.unknown	MDP0000364779	  no original description 	T
10.6.3	cell wall.degradation.pectate lyases and polygalacturonases	MDP0000718765	 highly similar to ( 682) AT4G24780 | Symbols:  | pectate lyase family protein | chr4:12770631-12772227 REVERSEmoderately similar to ( 440) PEL_LILLO Pectate lyase precursor (EC 4.2.2.2) - Lilium longiflorum (Trumpet lily)highly similar to ( 612) loc_os04g05050 12004.m35243 protein pectate lyase 8 precursor, putative, expressed   no original description 	T
23.4.1	nucleotide metabolism.phosphotransfer and pyrophosphatases.adenylate kinase	MDP0000606998	 moderately similar to ( 240) AT3G01820 | Symbols:  | adenylate kinase family protein | chr3:293981-295357 REVERSEweakly similar to ( 147) KADC_SOLTU Adenylate kinase, chloroplast precursor (EC 2.7.4.3) (ATP-AMP transphosphorylase) - Solanum tuberosum (Potato)moderately similar to ( 225) loc_os08g02540 12008.m04398 protein adenylate kinase, chloroplast precursor, putative, expressed   no original description 	T
10.2.1	cell wall.cellulose synthesis.cellulose synthase	MDP0000269350	 nearly identical (1858) AT5G05170 | Symbols: CESA3, IXR1, ATCESA3, ATH-B, CEV1 | CEV1 (CONSTITUTIVE EXPRESSION OF VSP 1); cellulose synthase/ transferase, transferring glycosyl groups | chr5:1530401-1535090 REVERSEnearly identical (1733) loc_os07g10770 12007.m05532 protein CESA8 - cellulose synthase, expressed   no original description 	T
29.2.1.2.2.8	protein.synthesis.ribosomal protein.eukaryotic.60S subunit.L8	MDP0000191034	 moderately similar to ( 496) AT4G36130 | Symbols:  | 60S ribosomal protein L8 (RPL8C) | chr4:17097613-17098656 FORWARDmoderately similar to ( 478) RL8_TOBAC 60S ribosomal protein L8 (60S ribosomal protein L2) - Nicotiana tabacum (Common tobacco)moderately similar to ( 490) loc_os12g38000 12012.m07586 protein 60S ribosomal protein L2, putative, expressed   no original description 	T
26.2	misc.UDP glucosyl and glucoronyl transferases	MDP0000316785	 highly similar to ( 723) AT5G61840 | Symbols: GUT1 | GUT1; catalytic/ glucuronoxylan glucuronosyltransferase | chr5:24839365-24841703 REVERSEhighly similar to ( 689) loc_os01g70200 12001.m13068 protein secondary cell wall-related glycosyltransferase family 47, putative, expressed MSP1_C  no original description 	T
29.5.11.4.2	protein.degradation.ubiquitin.E3.RING	MDP0000126653	 weakly similar to ( 124) AT5G05280 | Symbols:  | zinc finger (C3HC4-type RING finger) family protein | chr5:1565509-1566039 REVERSEvery weakly similar to (65.9) EL5_ORYSA E3 ubiquitin-protein ligase EL5 (EC 6.3.2.-) - Oryza sativa (Rice)weakly similar to ( 154) loc_os06g45580 12006.m09086 protein RING-H2 finger protein ATL5B precursor, putative, expressed  no original description 	T
35.1	not assigned.no ontology	MDP0000199442	 moderately similar to ( 321) AT3G48690 | Symbols: ATCXE12, CXE12 | CXE12; carboxylesterase | chr3:18037186-18038160 REVERSEweakly similar to ( 116) GID1_ORYSA Gibberellin receptor GID1 (EC 3.-.-.-) (Gibberellin-insensitive dwarf protein 1) (Protein GIBBERELLIN INSENSITIVE DWARF1) - Oryza sativa (Rice)moderately similar to ( 275) loc_os09g28740 12009.m06025 protein gibberellin receptor GID1L2, putative, expressed   no original description 	T
29.2.1.2.2.35	protein.synthesis.ribosomal protein.eukaryotic.60S subunit.L35	MDP0000242008	 weakly similar to ( 137) AT2G39390 | Symbols:  | 60S ribosomal protein L35 (RPL35B) | chr2:16450803-16451762 REVERSEvery weakly similar to (61.6) RL35_WHEAT 60S ribosomal protein L35 - Triticum aestivum (Wheat)weakly similar to ( 129) loc_os04g30730 12004.m35547 protein 60S ribosomal protein L35, putative, expressed   no original description 	T
31.1	cell.organisation	MDP0000282375	 moderately similar to ( 281) AT3G54070 | Symbols:  | ankyrin repeat family protein | chr3:20021330-20023603 REVERSEvery weakly similar to (90.1) loc_os03g17250 12003.m07155 protein protein binding protein, putative, expressed no original description 	T
35.2	not assigned.unknown	MDP0000415110	 very weakly similar to (70.1) AT3G13950 | Symbols:  | unknown protein | chr3:4604149-4605195 FORWARD no original description 	T
20.2.99	stress.abiotic.unspecified	MDP0000520923	 weakly similar to ( 198) AT4G08685 | Symbols: SAH7 | SAH7 | chr4:5550563-5551259 FORWARDweakly similar to ( 129) PSC13_MAIZE Pollen-specific protein C13 precursor - Zea mays (Maize)weakly similar to ( 155) loc_os10g22590 12010.m05266 protein pollen-specific protein C13 precursor, putative, expressed  no original description 	T
29.4	protein.postranslational modification	MDP0000377663	 highly similar to ( 920) AT2G44830 | Symbols:  | protein kinase, putative | chr2:18490398-18492779 FORWARDhighly similar to ( 631) G11A_ORYSA Protein kinase G11A (EC 2.7.11.1) - Oryza sativa (Rice)highly similar to ( 983) loc_os04g46180 12004.m101604 protein protein kinase G11A, putative, expressed  no original description 	T
35.2	not assigned.unknown	MDP0000247586	 weakly similar to ( 111) AT1G09812 | Symbols:  | unknown protein | chr1:3187817-3188580 FORWARDweakly similar to ( 103) loc_os01g65630 12001.m43435 protein expressed protein no original description 	T
35.2	not assigned.unknown	MDP0000647620	 very weakly similar to (95.9) AT5G41761 | Symbols:  | unknown protein | chr5:16711437-16711742 FORWARDweakly similar to ( 103) loc_os06g33200 12006.m07857 protein expressed protein no original description 	T
30.3	signalling.calcium	MDP0000282782	 nearly identical (2610) AT3G60860 | Symbols:  | guanine nucleotide exchange family protein | chr3:22484804-22491510 FORWARDvery weakly similar to (93.2) CALM3_PETHY Calmodulin-related protein - Petunia hybrida (Petunia)nearly identical (2370) loc_os03g14260 12003.m101270 protein brefeldin A-inhibited guanine nucleotide-exchange protein 2, putative, expressed MSP1_C  no original description 	T
20.2.3	stress.abiotic.drought/salt	MDP0000015338	 nearly identical (1129) AT4G22120 | Symbols:  | early-responsive to dehydration protein-related / ERD protein-related | chr4:11715976-11719144 REVERSEnearly identical (1059) loc_os01g35050 12001.m150613 protein HYP1, putative, expressed  no original description 	T
35.2	not assigned.unknown	MDP0000669792	   no original description 	T
29.3.4.99	protein.targeting.secretory pathway.unspecified	MDP0000767304	 moderately similar to ( 361) AT5G14670 | Symbols: ATARFA1B | ATARFA1B (ADP-ribosylation factor A1B); GTP binding / phospholipase activator/ protein binding | chr5:4729319-4730495 FORWARDmoderately similar to ( 360) ARF1_ORYSA ADP-ribosylation factor 1 (13 kDa cold-induced protein) - Oryza sativa (Rice)moderately similar to ( 362) loc_os01g59790 12001.m43240 protein ADP-ribosylation factor, putative, expressed   no original description 	T
30.3	signalling.calcium	MDP0000380330	 moderately similar to ( 259) AT1G12310 | Symbols:  | calmodulin, putative | chr1:4187500-4187946 REVERSEweakly similar to ( 156) CALM_LILLO Calmodulin (CaM) - Lilium longiflorum (Trumpet lily)moderately similar to ( 240) loc_os08g02420 12008.m04386 protein calmodulin, putative, expressed  no original description 	T
29.2.1.2.2.6	protein.synthesis.ribosomal protein.eukaryotic.60S subunit.L6	MDP0000158959	 moderately similar to ( 210) AT1G18540 | Symbols:  | 60S ribosomal protein L6 (RPL6A) | chr1:6377448-6378548 REVERSEmoderately similar to ( 211) RL6_MESCR 60S ribosomal protein L6 (YL16-like) - Mesembryanthemum crystallinum (Common ice plant)weakly similar to ( 141) loc_os04g39700 12004.m08956 protein 60S ribosomal protein L6, putative, expressed   no original description 	T
20.1.7	stress.biotic.PR-proteins	MDP0000446522	 moderately similar to ( 431) AT5G17680 | Symbols:  | disease resistance protein (TIR-NBS-LRR class), putative | chr5:5822999-5827153 FORWARDvery weakly similar to (83.2) loc_os12g29280 12012.m06742 protein disease resistance protein RGA3, putative, expressed   no original description 	T
35.1.19	not assigned.no ontology.C2 domain-containing protein	MDP0000231656	 moderately similar to ( 484) AT3G07940 | Symbols:  | zinc finger and C2 domain protein, putative | chr3:2529542-2531368 FORWARDmoderately similar to ( 392) loc_os01g72230 12001.m13258 protein GTPase activating protein, putative, expressed   no original description 	T
35.2	not assigned.unknown	MDP0000302206	  no original description 	T
23.5.4	nucleotide metabolism.deoxynucleotide metabolism.ribonucleoside-diphosphate reductase	MDP0000260116	 nearly identical (1398) AT2G21790 | Symbols: R1, RNR1, CLS8, ATRNR1 | RNR1 (RIBONUCLEOTIDE REDUCTASE 1); ATP binding / protein binding / ribonucleoside-diphosphate reductase | chr2:9293529-9297580 FORWARDnearly identical (1392) loc_os06g07210 12006.m31786 protein ribonucleoside-diphosphate reductase large subunit, putative, expressed  no original description 	T
29.5.9	protein.degradation.AAA type	MDP0000124724	 weakly similar to ( 190) AT1G04730 | Symbols:  | AAA-type ATPase family protein | chr1:1325385-1331086 REVERSEweakly similar to ( 197) loc_os03g15810 12003.m07018 protein ATP binding protein, putative, expressed  no original description 	T
29.2.1.2.2.32	protein.synthesis.ribosomal protein.eukaryotic.60S subunit.L32	MDP0000708895	 weakly similar to ( 199) AT4G18100 | Symbols:  | 60S ribosomal protein L32 (RPL32A) | chr4:10035715-10036475 REVERSEvery weakly similar to (71.6) RL32_MAIZE 60S ribosomal protein L32 (Fragment) - Zea mays (Maize)moderately similar to ( 207) loc_os08g41300 12008.m08108 protein 60S ribosomal protein L32, putative, expressed   no original description 	T
31.3.1	cell.cycle.peptidylprolyl isomerase	MDP0000253995	 moderately similar to ( 251) AT5G13120 | Symbols:  | peptidyl-prolyl cis-trans isomerase cyclophilin-type family protein | chr5:4162714-4164720 REVERSEmoderately similar to ( 201) CYPB_VICFA Peptidyl-prolyl cis-trans isomerase, chloroplast precursor (EC 5.2.1.8) (PPIase) (Rotamase) (Cyclophilin) (Cyclosporin A-binding protein) (CYP B) - Vicia faba (Broad bean)moderately similar to ( 254) loc_os05g01270 12005.m04668 protein peptidyl-prolyl cis-trans isomerase, chloroplast precursor, putative, expressed  no original description 	T
1.1.40	PS.lightreaction.cyclic electron flow-chlororespiration	MDP0000225351	 moderately similar to ( 442) AT1G64770 | Symbols: NDF2 | NDF2 (NDH-DEPENDENT CYCLIC ELECTRON FLOW 1) | chr1:24057549-24058565 FORWARDmoderately similar to ( 390) loc_os03g06230 12003.m06143 protein expressed protein  no original description 	T
10.6.3	cell wall.degradation.pectate lyases and polygalacturonases	MDP0000573746	 highly similar to ( 682) AT4G24780 | Symbols:  | pectate lyase family protein | chr4:12770631-12772227 REVERSEmoderately similar to ( 440) PEL_LILLO Pectate lyase precursor (EC 4.2.2.2) - Lilium longiflorum (Trumpet lily)highly similar to ( 612) loc_os04g05050 12004.m35243 protein pectate lyase 8 precursor, putative, expressed   no original description 	T
35.2	not assigned.unknown	MDP0000228579	 weakly similar to ( 144) AT2G44930 | Symbols:  | unknown protein | chr2:18529700-18531671 REVERSEvery weakly similar to (99.8) loc_os11g34090 12011.m07267 protein expressed protein   no original description 	T
27.3.99	RNA.regulation of transcription.unclassified	MDP0000213980	 moderately similar to ( 493) AT3G57170 | Symbols:  | N-acetylglucosaminyl transferase component family protein / Gpi1 family protein | chr3:21159620-21162259 REVERSEvery weakly similar to (84.3) loc_os05g22370 12005.m27768 protein N-acetylglucosaminyl transferase component, putative, expressed  no original description 	T
27.3.21	RNA.regulation of transcription.GRAS transcription factor family	MDP0000662303	 moderately similar to ( 411) AT1G14920 | Symbols: GAI, RGA2 | GAI (GIBBERELLIC ACID INSENSITIVE); transcription factor | chr1:5149414-5151015 FORWARDmoderately similar to ( 417) GAI1_VITVI DELLA protein GAI1 (Gibberellic acid-insensitive mutant protein 1) (VvGAI1) - Vitis vinifera (Grape)moderately similar to ( 428) loc_os01g45860 12001.m10786 protein DELLA protein RGA, putative, expressed   no original description 	T
35.2	not assigned.unknown	MDP0000292084	 weakly similar to ( 125) AT5G20190 | Symbols:  | binding | chr5:6814093-6815171 FORWARDweakly similar to ( 159) loc_os03g04710 12003.m101184 protein expressed protein     no original description 	T
8.1.1.1	TCA / org transformation.TCA.pyruvate DH.E1	MDP0000769597	 highly similar to ( 599) AT5G50850 | Symbols: MAB1 | MAB1 (MACCI-BOU); catalytic/ pyruvate dehydrogenase (acetyl-transferring) | chr5:20689671-20692976 FORWARDhighly similar to ( 588) ODPB_PEA Pyruvate dehydrogenase E1 component subunit beta, mitochondrial precursor (EC 1.2.4.1) (PDHE1-B) - Pisum sativum (Garden pea)highly similar to ( 623) loc_os08g42410 12008.m080219 protein pyruvate dehydrogenase E1 component subunit beta, mitochondrial precursor, putative, expressed   no original description 	T
31.1	cell.organisation	MDP0000304954	 moderately similar to ( 277) AT5G04700 | Symbols:  | FUNCTIONS IN: molecular_function unknown; INVOLVED IN: biological_process unknown; LOCATED IN: cellular_component unknown; CONTAINS InterPro DOMAIN/s: Ankyrin (InterPro:IPR002110); BEST Arabidopsis thaliana protein match is: unknown protein (TAIR:AT5G04680.1); Has 868 Blast hits to 744 proteins in 75 species: Archae - 0; Bacteria - 22; Metazoa - 164; Fungi - 15; Plants - 471; Viruses - 0; Other Eukaryotes - 196 (source: NCBI BLink). | chr5:1354240-1356754 REVERSEvery weakly similar to (67.4) loc_os11g14520 12011.m05588 protein conserved hypothetical protein  no original description 	T
35.1	not assigned.no ontology	MDP0000299243	 highly similar to ( 778) AT2G25530 | Symbols:  | AFG1-like ATPase family protein | chr2:10863059-10866597 FORWARDhighly similar to ( 731) loc_os03g63500 12003.m35538 protein ATPase, putative, expressed no original description 	T
11.1.10	lipid metabolism.FA synthesis and FA elongation.beta ketoacyl CoA synthase	MDP0000296089	 highly similar to ( 876) AT1G19440 | Symbols: KCS4 | KCS4 (3-KETOACYL-COA SYNTHASE 4); acyltransferase/ catalytic/ transferase, transferring acyl groups other than amino-acyl groups | chr1:6729119-6730669 FORWARDvery weakly similar to (61.6) CHSE_IPONI Chalcone synthase E (EC 2.3.1.74) (Naringenin-chalcone synthase E) (CHS-E) - Ipomoea nil (Japanese morning glory) (Pharbitis nil)highly similar to ( 833) loc_os05g49290 12005.m27982 protein acyltransferase, putative, expressed   no original description 	T
27.3.7	RNA.regulation of transcription.C2C2(Zn) CO-like, Constans-like zinc finger family	MDP0000297093	 moderately similar to ( 314) AT3G02380 | Symbols: COL2 | COL2 (constans-like 2); transcription factor/ zinc ion binding | chr3:487438-488624 REVERSEmoderately similar to ( 234) loc_os06g16370 12006.m06350 protein zinc finger protein CONSTANS, putative, expressed  no original description 	T
16.10	secondary metabolism.simple phenols	MDP0000237964	 highly similar to ( 925) AT5G60020 | Symbols: LAC17, ATLAC17 | LAC17 (laccase 17); laccase | chr5:24168072-24170223 FORWARDmoderately similar to ( 221) ASO_CUCSA L-ascorbate oxidase precursor (EC 1.10.3.3) (Ascorbase) (ASO) - Cucumis sativus (Cucumber)highly similar to ( 839) loc_os01g62490 12001.m12371 protein L-ascorbate oxidase precursor, putative, expressed   no original description 	T
35.2	not assigned.unknown	MDP0000901379	 moderately similar to ( 416) AT1G18180 | Symbols:  | oxidoreductase, acting on the CH-CH group of donors | chr1:6255903-6257737 FORWARDmoderately similar to ( 443) loc_os03g50070 12003.m10002 protein membrane protein, putative, expressed   no original description 	T
29.2.3	protein.synthesis.initiation	MDP0000246956	 moderately similar to ( 471) AT2G05830 | Symbols:  | eukaryotic translation initiation factor 2B family protein / eIF-2B family protein | chr2:2229964-2231839 FORWARDmoderately similar to ( 471) loc_os11g11050 12011.m28758 protein methylthioribose-1-phosphate isomerase, putative, expressed   no original description 	T
35.2	not assigned.unknown	MDP0000129361	   no original description 	T
17.5.3	hormone metabolism.ethylene.induced-regulated-responsive-activated	MDP0000129011	 moderately similar to ( 216) AT4G29100 | Symbols:  | ethylene-responsive family protein | chr4:14341140-14344575 FORWARDweakly similar to ( 179) loc_os02g17680 12002.m33639 protein helix-loop-helix DNA-binding, putative, expressed  no original description 	T
35.2	not assigned.unknown	MDP0000140204	 moderately similar to ( 222) AT4G27390 | Symbols:  | unknown protein | chr4:13703417-13704655 REVERSEweakly similar to ( 161) loc_os05g42280 12005.m083771 protein expressed protein    no original description 	T
26.28	misc.GDSL-motif lipase	MDP0000310262	 moderately similar to ( 420) AT5G03610 | Symbols:  | GDSL-motif lipase/hydrolase family protein | chr5:915650-918326 FORWARDweakly similar to ( 108) APG_BRANA Anter-specific proline-rich protein APG (Protein CEX) (Fragment) - Brassica napus (Rape)moderately similar to ( 226) loc_os09g39430 12009.m06881 protein carboxylic ester hydrolase/ hydrolase, acting on ester bonds, putative, expressed  no original description 	T
35.2	not assigned.unknown	MDP0000716241	 weakly similar to ( 126) AT3G50685 | Symbols:  | unknown protein | chr3:18834272-18834748 REVERSEvery weakly similar to (95.1) loc_os10g15300 12010.m04723 protein expressed protein  no original description 	T
29.5.11.20	protein.degradation.ubiquitin.proteasom	MDP0000405349	 moderately similar to ( 404) AT5G66140 | Symbols: PAD2 | PAD2 (PROTEASOME ALPHA SUBUNIT D 2); endopeptidase/ peptidase/ threonine-type endopeptidase | chr5:26437445-26438677 REVERSEmoderately similar to ( 414) PSA7_CICAR Proteasome subunit alpha type 7 (EC 3.4.25.1) (20S proteasome alpha subunit D) (20S proteasome subunit alpha-4) - Cicer arietinum (Chickpea) (Garbanzo)moderately similar to ( 401) loc_os09g36710 12009.m06620 protein proteasome subunit alpha type 7, putative, expressed  no original description 	T
34.5	transport.ammonium	MDP0000245173	 highly similar to ( 637) AT2G38290 | Symbols: ATAMT2, AMT2;1 | ATAMT2 (AMMONIUM TRANSPORTER 2); ammonium transmembrane transporter/ high affinity secondary active ammonium transmembrane transporter | chr2:16039672-16042291 REVERSEhighly similar to ( 766) loc_os01g65000 12001.m12614 protein ammonium transporter 2, putative, expressed   no original description 	T
8.2.9	TCA / org transformation.other organic acid transformatons.cyt MDH	MDP0000201006	 moderately similar to ( 202) AT5G43330 | Symbols:  | malate dehydrogenase, cytosolic, putative | chr5:17390552-17392449 FORWARDmoderately similar to ( 209) MDHC_MAIZE Malate dehydrogenase, cytoplasmic (EC 1.1.1.37) - Zea mays (Maize)moderately similar to ( 208) loc_os10g33800 12010.m21921 protein malate dehydrogenase, cytoplasmic, putative, expressed no original description 	T
35.2	not assigned.unknown	MDP0000916179	 weakly similar to ( 111) AT1G09812 | Symbols:  | unknown protein | chr1:3187817-3188580 FORWARDweakly similar to ( 103) loc_os01g65630 12001.m43435 protein expressed protein no original description 	T
26.10	misc.cytochrome P450	MDP0000229348	 highly similar to ( 825) AT2G30490 | Symbols: ATC4H, C4H, CYP73A5 | C4H (CINNAMATE-4-HYDROXYLASE); trans-cinnamate 4-monooxygenase | chr2:12993861-12995683 REVERSEhighly similar to ( 846) TCMO_CATRO Trans-cinnamate 4-monooxygenase (EC 1.14.13.11) (Cinnamic acid 4-hydroxylase) (CA4H) (C4H) (P450C4H) (Cytochrome P450 73) - Catharanthus roseus (Rosy periwinkle) (Madagascar periwinkle)highly similar to ( 697) loc_os01g60450 12001.m12173 protein trans-cinnamate 4-monooxygenase, putative   no original description 	T
31.4	cell.vesicle transport	MDP0000280952	 nearly identical (1953) AT1G62020 | Symbols:  | coatomer protein complex, subunit alpha, putative | chr1:22919814-22923728 FORWARDhighly similar to ( 583) 2AAA_PEA Protein phosphatase PP2A regulatory subunit A (PR65) (Fragment) - Pisum sativum (Garden pea)nearly identical (1846) loc_os09g04110 12009.m22040 protein coatomer subunit alpha, putative, expressed   no original description 	T
21.2.2	redox.ascorbate and glutathione.glutathione	MDP0000188669	 highly similar to ( 644) AT5G27380 | Symbols: GSH2, GSHB | GSH2 (GLUTATHIONE SYNTHETASE 2); glutathione synthase | chr5:9668211-9670912 REVERSEhighly similar to ( 647) GSHB_BRAJU Glutathione synthetase, chloroplast precursor (EC 6.3.2.3) (Glutathione synthase) (GSH synthetase) (GSH-S) - Brassica juncea (Leaf mustard) (Indian mustard)highly similar to ( 619) loc_os11g42350 12011.m28855 protein glutathione synthetase, chloroplast precursor, putative, expressed  no original description 	T
35.2	not assigned.unknown	MDP0000208200	  no original description 	T
31.1	cell.organisation	MDP0000120733	 moderately similar to ( 447) AT3G57890 | Symbols:  | tubulin-specific chaperone C-related | chr3:21438271-21441695 FORWARDmoderately similar to ( 347) loc_os06g41110 12006.m08646 protein expressed protein no original description 	T
20.1	stress.biotic	MDP0000203619	 weakly similar to ( 108) AT2G02100 | Symbols: LCR69, PDF2.2 | LCR69 (LOW-MOLECULAR-WEIGHT CYSTEINE-RICH 69); peptidase inhibitor | chr2:528397-528885 FORWARDweakly similar to ( 107) DEF2_CAPAN Defensin J1-2 precursor - Capsicum annuum (Bell pepper)weakly similar to ( 102) loc_os03g03810 12003.m05913 protein flower-specific gamma-thionin precursor, putative, expressed no original description 	T
16.8.3.1	secondary metabolism.flavonoids.dihydroflavonols.dihydroflavonol 4-reductase	MDP0000440654	 highly similar to ( 509) AT5G42800 | Symbols: DFR, TT3, M318 | DFR (DIHYDROFLAVONOL 4-REDUCTASE); dihydrokaempferol 4-reductase | chr5:17164296-17165864 REVERSEhighly similar to ( 557) DFRA_VITVI Dihydroflavonol-4-reductase (EC 1.1.1.219) (DFR) (Dihydrokaempferol 4-reductase) - Vitis vinifera (Grape)moderately similar to ( 313) loc_os01g44260 12001.m10683 protein dihydroflavonol-4-reductase, putative, expressed   no original description 	T
30.3	signalling.calcium	MDP0000368719	 weakly similar to ( 176) AT1G18210 | Symbols:  | calcium-binding protein, putative | chr1:6268273-6268785 REVERSEweakly similar to ( 104) CALM1_SOLTU Calmodulin-1 (CaM-1) - Solanum tuberosum (Potato)weakly similar to ( 144) loc_os05g13580 12005.m05773 protein calmodulin-related protein 2, touch-induced, putative, expressed   no original description 	T
30.4.4	signalling.phosphinositides.phosphoinositide phospholipase C	MDP0000200253	 highly similar to ( 701) AT2G40116 | Symbols:  | phosphoinositide-specific phospholipase C family protein | chr2:16751782-16754311 FORWARDhighly similar to ( 671) loc_os12g37560 12012.m26931 protein phospholipase C, putative, expressed   no original description 	T
29.4	protein.postranslational modification	MDP0000223519	 highly similar to ( 536) AT3G54180 | Symbols: CDC2B, CDKB1;1 | CDKB1;1 (CYCLIN-DEPENDENT KINASE B1;1); cyclin-dependent protein kinase/ kinase/ protein binding | chr3:20059882-20061250 FORWARDhighly similar to ( 551) CDC2C_ANTMA Cell division control protein 2 homolog C (EC 2.7.11.22) (EC 2.7.11.23) - Antirrhinum majus (Garden snapdragon)moderately similar to ( 464) loc_os01g67160 12001.m12819 protein cell division control protein 2 homolog C, putative, expressed  no original description 	T
10.5.5	cell wall.cell wall proteins.RGP	MDP0000631513	 highly similar to ( 533) AT5G16510 | Symbols:  | reversibly glycosylated polypeptide, putative | chr5:5393296-5394342 FORWARDmoderately similar to ( 380) UPTG_PEA Alpha-1,4-glucan-protein synthase [UDP-forming] (EC 2.4.1.112) (UDP-glucose:protein transglucosylase) (UPTG) (Reversibly glycosylated polypeptide) - Pisum sativum (Garden pea)moderately similar to ( 464) loc_os04g56520 12004.m35219 protein alpha-1,4-glucan-protein synthase, putative, expressed  no original description 	T
29.2.4	protein.synthesis.elongation	MDP0000596986	 weakly similar to ( 150) AT5G19510 | Symbols:  | elongation factor 1B alpha-subunit 2 (eEF1Balpha2) | chr5:6581854-6583137 REVERSEweakly similar to ( 148) EF1B_ORYSA Elongation factor 1-beta (EF-1-beta) (Elongation factor 1B-alpha 2) (eEF-1B alpha) (Elongation factor 1-beta') (EF-1-beta') - Oryza sativa (Rice)weakly similar to ( 148) loc_os07g46750 12007.m08892 protein elongation factor 1-beta, putative, expressed  no original description 	T
13.1.4.1.4	amino acid metabolism.synthesis.branched chain group.common.branched-chain amino acid aminotransferase	MDP0000309960	 moderately similar to ( 485) AT1G10070 | Symbols: ATBCAT-2 | ATBCAT-2 (ARABIDOPSIS THALIANA BRANCHED-CHAIN AMINO ACID TRANSAMINASE 2); branched-chain-amino-acid transaminase/ catalytic | chr1:3288672-3290164 FORWARDmoderately similar to ( 437) loc_os03g01600 12003.m05706 protein branched-chain-amino-acid aminotransferase, putative, expressed  no original description 	T
30.3	signalling.calcium	MDP0000281885	 weakly similar to ( 160) AT5G04170 | Symbols:  | calcium-binding EF hand family protein | chr5:1145579-1147519 FORWARDweakly similar to ( 161) loc_os12g04240 12012.m26827 protein grancalcin, putative, expressed no original description 	T
35.2	not assigned.unknown	MDP0000438699	 highly similar to ( 580) AT5G25260 | Symbols:  | FUNCTIONS IN: molecular_function unknown; INVOLVED IN: biological_process unknown; LOCATED IN: plasma membrane; EXPRESSED IN: cultured cell; CONTAINS InterPro DOMAIN/s: Band 7 protein (InterPro:IPR001107); BEST Arabidopsis thaliana protein match is: unknown protein (TAIR:AT5G25250.1); Has 4214 Blast hits to 3442 proteins in 652 species: Archae - 14; Bacteria - 1365; Metazoa - 1204; Fungi - 198; Plants - 130; Viruses - 8; Other Eukaryotes - 1295 (source: NCBI BLink). | chr5:8752751-8754282 FORWARDhighly similar to ( 578) loc_os10g34040 12010.m06234 protein nodulin, putative, expressed    no original description 	T
35.1	not assigned.no ontology	MDP0000330374	 highly similar to ( 506) AT1G27760 | Symbols: SAT32, ATSAT32 | interferon-related developmental regulator family protein / IFRD protein family | chr1:9668895-9671322 FORWARDmoderately similar to ( 339) loc_os07g39900 12007.m08232 protein interferon-related developmental regulator family protein, expressed  no original description 	T
26.7	misc.oxidases - copper, flavone etc	MDP0000259254	 moderately similar to ( 399) AT5G66060 | Symbols:  | iron ion binding / oxidoreductase/ oxidoreductase, acting on paired donors, with incorporation or reduction of molecular oxygen, 2-oxoglutarate as one donor, and incorporation of one atom each of oxygen into both donors | chr5:26419481-26421118 FORWARDmoderately similar to ( 381) loc_os02g58070 12002.m10828 protein prolyl 4-hydroxylase alpha-2 subunit precursor, putative, expressed   no original description 	T
29.3.4.99	protein.targeting.secretory pathway.unspecified	MDP0000045232	 highly similar to ( 621) AT4G24550 | Symbols:  | clathrin adaptor complexes medium subunit family protein | chr4:12675873-12678903 FORWARDhighly similar to ( 604) loc_os07g42810 12007.m29006 protein AP-4 complex subunit mu-1, putative, expressed   no original description 	T
26.28	misc.GDSL-motif lipase	MDP0000159572	 moderately similar to ( 240) AT1G29670 | Symbols:  | GDSL-motif lipase/hydrolase family protein | chr1:10375843-10377717 FORWARDvery weakly similar to (73.9) APG_BRANA Anter-specific proline-rich protein APG (Protein CEX) (Fragment) - Brassica napus (Rape)moderately similar to ( 240) loc_os01g54470 12001.m11611 protein anther-specific proline-rich protein APG, putative, expressed no original description 	T
21.5	redox.peroxiredoxin	MDP0000755936	 moderately similar to ( 257) AT3G26060 | Symbols: ATPRX Q | ATPRX Q; antioxidant/ peroxiredoxin | chr3:9524807-9526123 FORWARDvery weakly similar to (74.3) BAS1_HORVU 2-cys peroxiredoxin BAS1, chloroplast precursor (EC 1.11.1.15) (Thiol-specific antioxidant protein) (Fragment) - Hordeum vulgare (Barley)moderately similar to ( 262) loc_os06g09610 12006.m05679 protein peroxiredoxin bcp, putative, expressed no original description 	T
31.4	cell.vesicle transport	MDP0000319279	 nearly identical (1308) AT4G11380 | Symbols:  | beta-adaptin, putative | chr4:6920608-6925444 FORWARDvery weakly similar to (51.6) ROC4_NICSY 31 kDa ribonucleoprotein, chloroplast precursor - Nicotiana sylvestris (Wood tobacco)nearly identical (1294) loc_os03g23950 12003.m07741 protein AP-1 complex subunit beta-1, expressed  no original description 	T
30.2.17	signalling.receptor kinases.DUF 26	MDP0000211660	 moderately similar to ( 318) AT5G38280 | Symbols: PR5K | PR5K; kinase/ transmembrane receptor protein serine/threonine kinase | chr5:15293325-15295838 REVERSEweakly similar to ( 168) KPRO_MAIZE Putative receptor protein kinase ZmPK1 precursor (EC 2.7.11.1) - Zea mays (Maize)moderately similar to ( 356) loc_os01g02390 12001.m42838 protein TAK14, putative, expressed  no original description 	T
31.1	cell.organisation	MDP0000221331	 moderately similar to ( 397) AT3G47690 | Symbols: ATEB1A, EB1A, ATEB1H2 | ATEB1A; microtubule binding | chr3:17581353-17583346 FORWARDmoderately similar to ( 342) loc_os04g54940 12004.m10386 protein ATEB1A, putative, expressed  no original description 	T
29.5.11.4.2	protein.degradation.ubiquitin.E3.RING	MDP0000217861	 moderately similar to ( 352) AT3G05670 | Symbols:  | PHD finger family protein | chr3:1653888-1657022 FORWARDmoderately similar to ( 242) loc_os03g19020 12003.m35178 protein PHD-finger family protein, expressed   CobN  no original description 	T
1.1.5.2	PS.lightreaction.other electron carrier (ox/red).ferredoxin	MDP0000241545	 weakly similar to ( 145) AT1G02180 | Symbols:  | ferredoxin-related | chr1:413619-414505 REVERSEmoderately similar to ( 226) loc_os08g35710 12008.m07560 protein expressed protein  no original description 	T
26.10	misc.cytochrome P450	MDP0000151003	 moderately similar to ( 375) AT3G48280 | Symbols: CYP71A25 | CYP71A25; electron carrier/ heme binding / iron ion binding / monooxygenase/ oxygen binding | chr3:17879594-17881164 FORWARDmoderately similar to ( 382) C71A9_SOYBN Cytochrome P450 71A9 (EC 1.14.-.-) (P450 CP1) - Glycine max (Soybean)moderately similar to ( 343) loc_os09g26960 12009.m05848 protein flavonoid 3-monooxygenase, putative, expressed  no original description 	T
35.1	not assigned.no ontology	MDP0000923528	 weakly similar to ( 171) AT3G26510 | Symbols:  | octicosapeptide/Phox/Bem1p (PB1) domain-containing protein | chr3:9711886-9712542 REVERSEweakly similar to ( 131) loc_os11g25800 12011.m06501 protein octicosapeptide/Phox/Bem1p, putative, expressed  no original description 	T
35.2	not assigned.unknown	MDP0000343762	  no original description 	T
27.1	RNA.processing	MDP0000177787	 weakly similar to ( 177) AT1G20580 | Symbols:  | small nuclear ribonucleoprotein, putative / snRNP, putative / Sm protein, putative | chr1:7128979-7130371 FORWARDweakly similar to ( 178) loc_os02g01250 12002.m05475 protein small nuclear ribonucleoprotein Sm D3, putative, expressed   no original description 	T
29.5.11.3	protein.degradation.ubiquitin.E2	MDP0000273384	 moderately similar to ( 262) AT1G50490 | Symbols: UBC20 | UBC20 (ubiquitin-conjugating enzyme 20); ubiquitin-protein ligase | chr1:18705005-18706303 REVERSEweakly similar to ( 128) UBC2_MEDSA Ubiquitin-conjugating enzyme E2-17 kDa (EC 6.3.2.19) (Ubiquitin-protein ligase) (Ubiquitin carrier protein) - Medicago sativa (Alfalfa)moderately similar to ( 221) loc_os01g16650 12001.m08261 protein ubiquitin-conjugating enzyme X, putative, expressed    no original description 	T
1.1.1.1	PS.lightreaction.photosystem II.LHC-II	MDP0000132360	 moderately similar to ( 379) AT2G40100 | Symbols: LHCB4.3 | LHCB4.3 (light harvesting complex PSII); chlorophyll binding | chr2:16745884-16747190 FORWARDmoderately similar to ( 224) CB29_CHLRE Chlorophyll a-b binding protein CP29 - Chlamydomonas reinhardtiimoderately similar to ( 346) loc_os07g37240 12007.m29225 protein expressed protein  no original description 	T
20.2.1	stress.abiotic.heat	MDP0000676815	 highly similar to ( 599) AT5G18750 | Symbols:  | DNAJ heat shock N-terminal domain-containing protein | chr5:6255177-6257831 FORWARDhighly similar to ( 585) loc_os06g34440 12006.m07980 protein heat shock protein binding protein, putative, expressed  no original description 	T
1.1.40	PS.lightreaction.cyclic electron flow-chlororespiration	MDP0000334395	 moderately similar to ( 374) AT3G15840 | Symbols: PIFI | PIFI (post-illumination chlorophyll fluorescence increase) | chr3:5356782-5358070 REVERSEmoderately similar to ( 305) loc_os03g38950 12003.m35300 protein expressed protein  no original description 	T
30.2.3	signalling.receptor kinases.leucine rich repeat III	MDP0000248164	 moderately similar to ( 311) AT2G26730 | Symbols:  | leucine-rich repeat transmembrane protein kinase, putative | chr2:11388621-11391286 FORWARDweakly similar to ( 124) PSKR_DAUCA Phytosulfokine receptor precursor (EC 2.7.11.1) (Phytosulfokine LRR receptor kinase) - Daucus carota (Carrot)moderately similar to ( 276) loc_os01g42294 12001.m10495 protein atypical receptor-like kinase MARK, putative, expressed    COG4907  no original description 	T
35.2	not assigned.unknown	MDP0000314803	 moderately similar to ( 222) AT5G19855 | Symbols:  | unknown protein | chr5:6712166-6713445 REVERSEmoderately similar to ( 208) loc_os03g59320 12003.m10830 protein expressed protein no original description 	T
34.16	transport.ABC transporters and multidrug resistance systems	MDP0000231597	 nearly identical (1863) AT2G36910 | Symbols: ATPGP1, PGP1, ABCB1 | ABCB1 (ATP BINDING CASSETTE SUBFAMILY B1); ATPase, coupled to transmembrane movement of substances / auxin efflux transmembrane transporter/ calmodulin binding | chr2:15502162-15507050 FORWARDhighly similar to ( 812) MDR_ORYSA Putative multidrug resistance protein (P-glycoprotein) - Oryza sativa (Rice)nearly identical (1575) loc_os08g45030 12008.m08473 protein multidrug resistance protein 1, putative, expressed   no original description 	T
27.2	RNA.transcription	MDP0000603266	 moderately similar to ( 268) AT4G14660 | Symbols: NRPE7 | NRPE7; DNA-directed RNA polymerase | chr4:8406500-8407036 REVERSEvery weakly similar to (80.5) RPB7_SOYBN DNA-directed RNA polymerase II 19 kDa polypeptide (EC 2.7.7.6) (RNA polymerase II subunit 5) - Glycine max (Soybean)weakly similar to ( 197) loc_os05g06490 12005.m05176 protein DNA-directed RNA polymerase II 19 kDa polypeptide, putative, expressed no original description 	T
35.2	not assigned.unknown	MDP0000803194	 weakly similar to ( 166) AT1G61930 | Symbols:  | unknown protein | chr1:22893218-22893829 FORWARDvery weakly similar to (89.4) loc_os07g33270 12007.m07588 protein expressed protein   no original description 	T
33.3	development.squamosa promoter binding like (SPL)	MDP0000210138	 weakly similar to ( 188) AT5G50570 | Symbols:  | squamosa promoter-binding protein, putative | chr5:20582555-20583791 REVERSEvery weakly similar to ( 100) SBP2_ANTMA Squamosa promoter-binding protein 2 - Antirrhinum majus (Garden snapdragon)weakly similar to ( 186) loc_os08g41940 12008.m08172 protein teosinte glume architecture 1, putative, expressed   no original description 	T
35.2	not assigned.unknown	MDP0000279037	 highly similar to ( 827) AT1G63300 | Symbols:  | unknown protein | chr1:23482193-23486067 FORWARDvery weakly similar to (73.6) MFP1_TOBAC MAR-binding filament-like protein 1-1 - Nicotiana tabacum (Common tobacco)moderately similar to ( 271) loc_os10g36060 12010.m06420 protein chorion family 2 protein, expressed   no original description 	T
27.3.7	RNA.regulation of transcription.C2C2(Zn) CO-like, Constans-like zinc finger family	MDP0000177126	 moderately similar to ( 350) AT3G07650 | Symbols: COL9 | COL9 (CONSTANS-LIKE 9); transcription factor/ zinc ion binding | chr3:2442494-2443901 FORWARDmoderately similar to ( 327) loc_os06g19444 12006.m32050 protein CCT motif family protein, expressed  no original description 	T
34.2	transport.sugars	MDP0000188309	 moderately similar to ( 433) AT4G35335 | Symbols:  | nucleotide-sugar transmembrane transporter/ sugar:hydrogen symporter | chr4:16807286-16810015 FORWARDmoderately similar to ( 450) loc_os07g38610 12007.m08106 protein UDP-galactose translocator, putative, expressed no original description 	T
35.2	not assigned.unknown	MDP0000363760	 very weakly similar to (69.7) AT1G42960 | Symbols:  | expressed protein localized to the inner membrane of the chloroplast. | chr1:16125863-16127080 FORWARD  no original description 	T
35.1	not assigned.no ontology	MDP0000219992	 moderately similar to ( 225) AT1G11755 | Symbols: LEW1 | LEW1 (LEAF WILTING 1); dehydrodolichyl diphosphate synthase | chr1:3969987-3971488 REVERSEmoderately similar to ( 206) loc_os03g10080 12003.m35073 protein expressed protein no original description 	T
10.5.4	cell wall.cell wall proteins.HRGP	MDP0000256238	 highly similar to ( 577) AT3G22440 | Symbols:  | hydroxyproline-rich glycoprotein family protein | chr3:7959854-7961886 FORWARDmoderately similar to ( 367) loc_os03g58070 12003.m10719 protein hydroxyproline-rich glycoprotein, putative, expressed   no original description 	T
1.2.5	PS.photorespiration.serine hydroxymethyltransferase	MDP0000234570	 moderately similar to ( 322) AT4G37930 | Symbols: SHM1, STM, SHMT1 | SHM1 (SERINE TRANSHYDROXYMETHYLTRANSFERASE 1); glycine hydroxymethyltransferase/ poly(U) binding | chr4:17831891-17834742 REVERSEmoderately similar to ( 337) GLYN_FLAPR Serine hydroxymethyltransferase 2, mitochondrial precursor (EC 2.1.2.1) (Serine methylase) (Glycine hydroxymethyltransferase) (SHMT) - Flaveria pringleimoderately similar to ( 328) loc_os03g52840 12003.m101129 protein serine hydroxymethyltransferase, mitochondrial precursor, putative, expressed  no original description 	T
29.5.11.20	protein.degradation.ubiquitin.proteasom	MDP0000232788	 highly similar to ( 638) AT4G19006 | Symbols:  | 26S proteasome regulatory subunit, putative (RPN9) | chr4:10409349-10411347 REVERSEhighly similar to ( 580) loc_os01g32800 12001.m43033 protein 26S proteasome non-ATPase regulatory subunit 13, putative, expressed no original description 	T
17.5.1	hormone metabolism.ethylene.synthesis-degradation	MDP0000214625	 moderately similar to ( 363) AT1G49390 | Symbols:  | oxidoreductase, 2OG-Fe(II) oxygenase family protein | chr1:18279600-18280873 FORWARDweakly similar to ( 191) FLS_PETHY Flavonol synthase/flavanone 3-hydroxylase (EC 1.14.11.23) (EC 1.14.11.9) (FLS) - Petunia hybrida (Petunia)moderately similar to ( 251) loc_os06g08032 12006.m05526 protein flavonol synthase/flavanone 3-hydroxylase, putative, expressed  no original description 	T
35.2	not assigned.unknown	MDP0000903481	 moderately similar to ( 396) AT4G28100 | Symbols:  | unknown protein | chr4:13965300-13966697 REVERSEmoderately similar to ( 308) loc_os07g37156 12007.m79903 protein expressed protein   no original description 	T
17.3.2.2	hormone metabolism.brassinosteroid.signal transduction.BZR	MDP0000157809	 moderately similar to ( 282) AT1G75080 | Symbols: BZR1 | BZR1 (BRASSINAZOLE-RESISTANT 1); DNA binding / transcription regulator/ transcription repressor | chr1:28185709-28187063 FORWARDmoderately similar to ( 201) loc_os07g39220 12007.m08164 protein BES1/BZR1 homolog protein 1, putative, expressed   no original description 	T
29.5.11.20	protein.degradation.ubiquitin.proteasom	MDP0000934414	 highly similar to ( 780) AT4G29040 | Symbols: RPT2a | RPT2a (regulatory particle AAA-ATPase 2a); ATPase | chr4:14312369-14314386 FORWARDhighly similar to ( 765) PRS4_ORYSA 26S protease regulatory subunit 4 homolog (TAT-binding protein homolog 2) - Oryza sativa (Rice)highly similar to ( 768) loc_os03g18690 12003.m07290 protein 26S protease regulatory subunit 4, putative, expressed   no original description 	T
16.7	secondary metabolism.wax	MDP0000218683	 moderately similar to ( 474) AT1G02205 | Symbols: CER1 | CER1 (ECERIFERUM 1); octadecanal decarbonylase | chr1:418818-422154 FORWARDmoderately similar to ( 445) loc_os10g33250 12010.m21915 protein CER1, putative, expressed  no original description 	T
29.5.11.4.2	protein.degradation.ubiquitin.E3.RING	MDP0000202889	 moderately similar to ( 372) AT5G01980 | Symbols:  | zinc finger (C3HC4-type RING finger) family protein | chr5:375542-377023 FORWARDvery weakly similar to (53.9) EL5_ORYSA E3 ubiquitin-protein ligase EL5 (EC 6.3.2.-) - Oryza sativa (Rice)moderately similar to ( 263) loc_os11g02670 12011.m28608 protein protein binding protein, putative, expressed  no original description 	T
18.7	Co-factor and vitamine metabolism.iron-sulphur clusters	MDP0000180080	 moderately similar to ( 235) AT4G22220 | Symbols: ISU1, ATISU1 | ISU1; structural molecule | chr4:11759444-11760881 REVERSEmoderately similar to ( 241) loc_os01g47340 12001.m42683 protein nifU-like N-terminal domain-containing protein, mitochondrial precursor, putative, expressed  no original description 	T
29.2.1.2.1.515	protein.synthesis.ribosomal protein.eukaryotic.40S subunit.S15A	MDP0000320879	 moderately similar to ( 246) AT5G59850 | Symbols:  | 40S ribosomal protein S15A (RPS15aF) | chr5:24112499-24113084 REVERSEmoderately similar to ( 243) RS15A_BRANA 40S ribosomal protein S15a (PPCB8) - Brassica napus (Rape)moderately similar to ( 241) loc_os02g27760 12002.m07920 protein 40S ribosomal protein S15a, putative, expressed no original description 	T
31.4	cell.vesicle transport	MDP0000901016	 highly similar to ( 962) AT3G15980 | Symbols:  | coatomer protein complex, subunit beta 2 (beta prime), putative | chr3:5412015-5418313 REVERSEhighly similar to ( 951) loc_os06g05180 12006.m71402 protein coatomer subunit beta, putative, expressed  no original description 	T
16.5	secondary metabolism.sulfur-containing	MDP0000501868	 moderately similar to ( 222) AT1G10500 | Symbols: ATCPISCA | ATCPISCA (chloroplast-localized IscA-like protein); structural molecule | chr1:3460160-3461340 REVERSEweakly similar to ( 195) loc_os06g05400 12006.m31979 protein iron-sulfur assembly protein IscA, chloroplast precursor, putative, expressed no original description 	T
27.3.22	RNA.regulation of transcription.HB,Homeobox transcription factor family	MDP0000050082	 nearly identical (1260) AT2G34710 | Symbols: PHB, ATHB14, ATHB-14, PHB-1D | PHB (PHABULOSA); DNA binding / transcription factor | chr2:14639548-14643993 REVERSEnearly identical (1269) loc_os03g43930 12003.m09434 protein class III HD-Zip protein 4, putative, expressed  no original description 	T
17.2.3	hormone metabolism.auxin.induced-regulated-responsive-activated	MDP0000285927	 very weakly similar to (62.8) AT1G54070 | Symbols:  | dormancy/auxin associated protein-related | chr1:20182683-20183242 FORWARDvery weakly similar to (65.5) loc_os08g35190 12008.m26509 protein auxin-repressed protein, putative, expressed no original description 	T
34.16	transport.ABC transporters and multidrug resistance systems	MDP0000289874	 nearly identical (2167) AT1G04120 | Symbols: ATMRP5, MRP5 | ATMRP5; ATPase, coupled to transmembrane movement of substances / sulfonylurea receptor | chr1:1064848-1070396 REVERSEweakly similar to ( 149) MDR_ORYSA Putative multidrug resistance protein (P-glycoprotein) - Oryza sativa (Rice)nearly identical (1922) loc_os03g04920 12003.m06018 protein multidrug resistance-associated protein 5, putative, expressed TrbL  no original description 	T
10.7	cell wall.modification	MDP0000279459	 very weakly similar to (74.7) AT2G18660 | Symbols: EXLB3 | EXLB3 (EXPANSIN-LIKE B3 PRECURSOR) | chr2:8091032-8091644 REVERSEvery weakly similar to (86.7) loc_os06g47360 12006.m09262 protein blight-associated protein p12 precursor, putative, expressed  no original description 	T
30.11	signalling.light	MDP0000197415	 highly similar to ( 716) AT4G08920 | Symbols: CRY1, BLU1, HY4, OOP2, ATCRY1 | CRY1 (CRYPTOCHROME 1); ATP binding / blue light photoreceptor/ protein homodimerization/ protein kinase | chr4:5724260-5726905 FORWARDmoderately similar to ( 481) PHR1_SINAL Deoxyribodipyrimidine photo-lyase (EC 4.1.99.3) (DNA photolyase) (Photoreactivating enzyme) - Sinapis alba (White mustard) (Brassica hirta)highly similar to ( 661) loc_os04g37920 12004.m35145 protein cryptochrome 1 apoprotein, putative, expressed  no original description 	T
20.1.3	stress.biotic.signalling	MDP0000160311	 moderately similar to ( 366) AT3G48090 | Symbols: EDS1 | EDS1 (enhanced disease susceptibility 1); lipase/ signal transducer/ triacylglycerol lipase | chr3:17755553-17757292 REVERSEmoderately similar to ( 426) loc_os09g22450 12009.m05451 protein EDS1-like protein, putative, expressed  no original description 	T
29.3.1	protein.targeting.nucleus	MDP0000864511	 nearly identical (1862) AT5G17020 | Symbols: XPO1A, ATCRM1, ATXPO1, XPO1 | XPO1A; protein binding / protein transporter/ receptor | chr5:5594904-5602467 FORWARDnearly identical (1714) loc_os03g64080 12003.m11273 protein retrotransposon protein, putative, unclassified  no original description 	T
29.2.1.2.1.24	protein.synthesis.ribosomal protein.eukaryotic.40S subunit.S24	MDP0000243151	 moderately similar to ( 226) AT3G04920 | Symbols:  | 40S ribosomal protein S24 (RPS24A) | chr3:1360989-1362065 FORWARDmoderately similar to ( 215) loc_os01g52490 12001.m11423 protein 40S ribosomal protein S24, putative, expressed no original description 	T
26.21	misc.protease inhibitor/seed storage/lipid transfer protein (LTP) family protein	MDP0000764876	 weakly similar to ( 129) AT3G22142 | Symbols:  | structural constituent of cell wall | chr3:7803604-7808046 REVERSEvery weakly similar to (85.5) 14KD_DAUCA 14 kDa proline-rich protein DC2.15 precursor - Daucus carota (Carrot)weakly similar to ( 115) loc_os06g07220 12006.m05445 protein 36.4 kDa proline-rich protein, putative, expressed  no original description 	T
35.1.21	not assigned.no ontology.epsin N-terminal homology (ENTH) domain-containing protein	MDP0000311896	 moderately similar to ( 484) AT5G11710 | Symbols:  | epsin N-terminal homology (ENTH) domain-containing protein / clathrin assembly protein-related | chr5:3772981-3776316 FORWARDmoderately similar to ( 400) loc_os04g51120 12004.m35441 protein af10-protein, putative, expressed  no original description 	T
26.21	misc.protease inhibitor/seed storage/lipid transfer protein (LTP) family protein	MDP0000155446	 weakly similar to ( 115) AT1G62510 | Symbols:  | protease inhibitor/seed storage/lipid transfer protein (LTP) family protein | chr1:23136632-23137081 REVERSEvery weakly similar to (92.4) 14KD_DAUCA 14 kDa proline-rich protein DC2.15 precursor - Daucus carota (Carrot)weakly similar to ( 105) loc_os03g01320 12003.m34972 protein NT16 polypeptide, putative, expressed no original description 	T
20.2.3	stress.abiotic.drought/salt	MDP0000303986	 highly similar to ( 898) AT1G33170 | Symbols:  | dehydration-responsive family protein | chr1:12027262-12030397 FORWARDhighly similar to ( 910) loc_os06g01450 12006.m04884 protein methyltransferase, putative, expressed  no original description 	T
10.1.20	cell wall.precursor synthesis.phosphomannose isomerase	MDP0000448667	 moderately similar to ( 410) AT3G02570 | Symbols: MEE31, PMI1 | MEE31 (MATERNAL EFFECT EMBRYO ARREST 31); mannose-6-phosphate isomerase | chr3:543463-545478 REVERSEmoderately similar to ( 367) loc_os01g03710 12001.m42847 protein mannose-6-phosphate isomerase, putative, expressed   no original description 	T
29.4	protein.postranslational modification	MDP0000149108	 highly similar to ( 779) AT5G25510 | Symbols:  | serine/threonine protein phosphatase 2A (PP2A) regulatory subunit B', putative | chr5:8882728-8884325 REVERSEhighly similar to ( 675) loc_os05g48150 12005.m08886 protein transposon protein, putative, unclassified, expressed   no original description 	T
35.2	not assigned.unknown	MDP0000175229	  no original description 	T
27.4	RNA.RNA binding	MDP0000628982	 moderately similar to ( 403) AT3G11400 | Symbols: EIF3G1, ATEIF3G1 | eukaryotic translation initiation factor 3G / eIF3g | chr3:3578536-3580366 FORWARDvery weakly similar to (54.7) ROC1_SPIOL 28 kDa ribonucleoprotein, chloroplast (28RNP) - Spinacia oleracea (Spinach)moderately similar to ( 401) loc_os02g54700 12002.m10498 protein eukaryotic translation initiation factor 3 subunit 4, putative, expressed  no original description 	T
35.2	not assigned.unknown	MDP0000158751	 weakly similar to ( 192) AT5G62140 | Symbols:  | unknown protein | chr5:24954563-24955376 REVERSEmoderately similar to ( 201) loc_os02g45460 12002.m09583 protein expressed protein  no original description 	T
29.2.1.2.1.17	protein.synthesis.ribosomal protein.eukaryotic.40S subunit.S17	MDP0000283097	 moderately similar to ( 220) AT5G04800 | Symbols:  | 40S ribosomal protein S17 (RPS17D) | chr5:1389217-1389642 FORWARDmoderately similar to ( 210) loc_os10g27190 12010.m05653 protein 40S ribosomal protein S17-4, putative, expressed   no original description 	T
27.4	RNA.RNA binding	MDP0000266453	 nearly identical (1020) AT5G25060 | Symbols:  | RNA recognition motif (RRM)-containing protein | chr5:8634219-8639984 REVERSEmoderately similar to ( 264) GPX1_PEA Phospholipid hydroperoxide glutathione peroxidase, chloroplast precursor (EC 1.11.1.12) (PHGPx) - Pisum sativum (Garden pea)highly similar to ( 972) loc_os02g08360 12002.m06182 protein RNA binding protein, putative, expressed   no original description 	T
35.1.1	not assigned.no ontology.ABC1 family protein	MDP0000147600	 moderately similar to ( 416) AT5G24810 | Symbols:  | ABC1 family protein | chr5:8516902-8522616 REVERSEmoderately similar to ( 342) loc_os06g48770 12006.m32168 protein retrotransposon protein, putative, SINE subclass, expressedCOG3889  no original description 	T
35.2	not assigned.unknown	MDP0000731378	 weakly similar to ( 191) AT5G49800 | Symbols:  | FUNCTIONS IN: molecular_function unknown; INVOLVED IN: biological_process unknown; EXPRESSED IN: 12 plant structures; EXPRESSED DURING: 4 anthesis, F mature embryo stage, C globular stage, petal differentiation and expansion stage, D bilateral stage; CONTAINS InterPro DOMAIN/s: Lipid-binding START (InterPro:IPR002913); Has 156 Blast hits to 155 proteins in 50 species: Archae - 0; Bacteria - 14; Metazoa - 91; Fungi - 2; Plants - 15; Viruses - 0; Other Eukaryotes - 34 (source: NCBI BLink). | chr5:20234771-20236135 REVERSEweakly similar to ( 156) loc_os11g14070 12011.m05545 protein expressed protein no original description 	T
35.2	not assigned.unknown	MDP0000176370	 moderately similar to ( 395) AT4G31530 | Symbols:  | binding / catalytic | chr4:15282281-15284064 FORWARDmoderately similar to ( 391) loc_os03g23980 12003.m07744 protein 3-beta hydroxysteroid dehydrogenase/isomerase family protein, putative, expressed  no original description 	T
26.10	misc.cytochrome P450	MDP0000160444	 moderately similar to ( 454) AT2G45510 | Symbols: CYP704A2 | CYP704A2; electron carrier/ heme binding / iron ion binding / monooxygenase/ oxygen binding | chr2:18753085-18754944 FORWARDweakly similar to ( 122) C97B2_SOYBN Cytochrome P450 97B2 (EC 1.14.-.-) - Glycine max (Soybean)moderately similar to ( 483) loc_os10g38110 12010.m21797 protein cytochrome P450 94A2, putative, expressed  no original description 	T
10.7	cell wall.modification	MDP0000431696	 moderately similar to ( 400) AT2G40610 | Symbols: ATEXPA8, EXP8, ATEXP8, ATHEXP ALPHA 1.11 | ATEXPA8 (ARABIDOPSIS THALIANA EXPANSIN A8) | chr2:16949121-16950472 REVERSEmoderately similar to ( 368) EXPA4_ORYSA Expansin-A4 precursor (OsEXPA4) (Alpha-expansin-4) (OsEXP4) (OsaEXPa1.22) - Oryza sativa (Rice)moderately similar to ( 368) loc_os05g39990 12005.m08176 protein alpha-expansin 1 precursor, putative, expressed  no original description 	T
29.5.5	protein.degradation.serine protease	MDP0000162392	 weakly similar to ( 192) AT1G11750 | Symbols: CLPP6, NCLPP1, NCLPP6 | CLPP6; serine-type endopeptidase | chr1:3967609-3969535 FORWARDvery weakly similar to (58.9) CLPP_PANGI ATP-dependent Clp protease proteolytic subunit (EC 3.4.21.92) (Endopeptidase Clp) - Panax ginseng (Korean ginseng)weakly similar to ( 133) loc_os03g29810 12003.m08245 protein ATP-dependent Clp protease proteolytic subunit 3, putative, expressed  no original description 	T
30.11.1	signalling.light.COP9 signalosome	MDP0000904859	 highly similar to ( 652) AT3G61140 | Symbols: FUS6, ATFUS6, CSN1, COP11, EMB78, ATSK31, SK31 | FUS6 (FUSCA 6) | chr3:22626335-22628895 FORWARDvery weakly similar to (68.6) CSN1_BRAOL COP9 signalosome complex subunit 1 (CSN complex subunit 1) (Constitutive photomorphogenesis protein 11) (FUSCA protein 6) (FUSCA6) (Fragment) - Brassica oleracea (Wild cabbage)highly similar to ( 594) loc_os03g02540 12003.m05795 protein COP9 signalosome complex subunit 1, putative, expressed  no original description 	T
11.8.8	lipid metabolism.'exotics' (steroids, squalene etc).squalene synthase	MDP0000311820	 highly similar to ( 611) AT4G34640 | Symbols: SQS1, ERG9 | SQS1 (SQUALENE SYNTHASE 1); farnesyl-diphosphate farnesyltransferase | chr4:16538489-16541655 FORWARDhighly similar to ( 566) loc_os03g59040 12003.m101539 protein squalene synthetase, putative, expressed  no original description 	T
35.2	not assigned.unknown	MDP0000889414	 very weakly similar to (77.0) AT3G43520 | Symbols:  | unknown protein | chr3:15406088-15407699 FORWARDvery weakly similar to (88.6) loc_os04g55930 12004.m10484 protein expressed protein     no original description 	T
29.5.11.4.2	protein.degradation.ubiquitin.E3.RING	MDP0000319557	 highly similar to ( 795) AT1G73930 | Symbols:  | unknown protein | chr1:27795347-27797534 REVERSEmoderately similar to ( 236) MSH2_MAIZE DNA mismatch repair protein MSH2 (MUS1) - Zea mays (Maize)highly similar to ( 657) loc_os03g15390 12003.m06976 protein expressed protein   no original description 	T
29.5.11.1	protein.degradation.ubiquitin.ubiquitin	MDP0000280442	 moderately similar to ( 259) AT1G31340 | Symbols: RUB1, NEDD8, ATRUB1 | RUB1 (RELATED TO UBIQUITIN 1); protein binding | chr1:11218076-11219417 REVERSEweakly similar to ( 150) UBIQ_WHEAT Ubiquitin - Triticum aestivum (Wheat)moderately similar to ( 259) loc_os09g25320 12009.m05684 protein polyubiquitin 2, putative, expressed no original description 	T
10.7	cell wall.modification	MDP0000697206	 moderately similar to ( 472) AT2G28950 | Symbols: ATEXPA6, ATEXP6, ATHEXP ALPHA 1.8 | ATEXPA6 (ARABIDOPSIS THALIANA EXPANSIN A6) | chr2:12431840-12433482 REVERSEmoderately similar to ( 449) EXPA7_ORYSA Expansin-A7 precursor (OsEXPA7) (Alpha-expansin-7) (OsEXP7) (OsaEXPa1.26) - Oryza sativa (Rice)moderately similar to ( 449) loc_os03g60720 12003.m10961 protein alpha-expansin 6 precursor, putative, expressed  no original description 	T
35.2	not assigned.unknown	MDP0000240039	 highly similar to ( 914) AT1G09780 | Symbols:  | 2,3-biphosphoglycerate-independent phosphoglycerate mutase, putative / phosphoglyceromutase, putative | chr1:3165550-3167812 REVERSEhighly similar to ( 958) PMGI_MESCR 2,3-bisphosphoglycerate-independent phosphoglycerate mutase (EC 5.4.2.1) (Phosphoglyceromutase) (BPG-independent PGAM) (PGAM-I) - Mesembryanthemum crystallinum (Common ice plant)highly similar to ( 946) loc_os01g60190 12001.m42741 protein 2,3-bisphosphoglycerate-independent phosphoglycerate mutase, putative, expressed  no original description 	T
26.19	misc.plastocyanin-like	MDP0000759922	 weakly similar to ( 170) AT4G31840 | Symbols:  | plastocyanin-like domain-containing protein | chr4:15401798-15402426 FORWARDvery weakly similar to (89.0) NO552_SOYBN Early nodulin 55-2 precursor (N-55-2) (Nodulin-315) - Glycine max (Soybean)weakly similar to ( 111) loc_os08g17160 12008.m05836 protein early nodulin-like protein 1 precursor, putative, expressed no original description 	T
26.4.1	misc.beta 1,3 glucan hydrolases.glucan endo-1,3-beta-glucosidase	MDP0000251002	 highly similar to ( 622) AT3G13560 | Symbols:  | glycosyl hydrolase family 17 protein | chr3:4425484-4427284 REVERSEmoderately similar to ( 233) E13B_WHEAT Glucan endo-1,3-beta-glucosidase precursor (EC 3.2.1.39) ((1->3)-beta-glucan endohydrolase) ((1->3)-beta-glucanase) (Beta-1,3-endoglucanase) - Triticum aestivum (Wheat)moderately similar to ( 483) loc_os07g38930 12007.m08137 protein glucan endo-1,3-beta-glucosidase 4 precursor, putative, expressed   no original description 	T
27.3.3	RNA.regulation of transcription.AP2/EREBP, APETALA2/Ethylene-responsive element binding protein family	MDP0000453797	 weakly similar to ( 176) AT1G78080 | Symbols: RAP2.4 | RAP2.4 (related to AP2 4); DNA binding / transcription factor | chr1:29364790-29365794 FORWARDvery weakly similar to (63.5) ERF5_TOBAC Ethylene-responsive transcription factor 5 (Ethylene-responsive element-binding factor 5 homolog) (EREBP-4) (NtERF4) - Nicotiana tabacum (Common tobacco)weakly similar to ( 159) loc_os02g51670 12002.m10198 protein AP2 domain-containing protein, putative, expressed  no original description 	T
26.13	misc.acid and other phosphatases	MDP0000155698	 moderately similar to ( 222) AT4G25150 | Symbols:  | acid phosphatase, putative | chr4:12901736-12902882 REVERSEmoderately similar to ( 209) VSPA_SOYBN Stem 28 kDa glycoprotein precursor (Vegetative storage protein A) - Glycine max (Soybean)moderately similar to ( 220) loc_os06g04790 12006.m05208 protein acid phosphatase 1 precursor, putative, expressed  no original description 	T
27.3.25	RNA.regulation of transcription.MYB domain transcription factor family	MDP0000742771	 moderately similar to ( 344) AT3G01140 | Symbols: MYB106, AtMYB106 | MYB106 (myb domain protein 106); DNA binding / transcription factor | chr3:46619-48143 REVERSEmoderately similar to ( 205) MYB4_ORYSA Myb-related protein Myb4 (OsMyb4) (Transcription factor RLTR1) - Oryza sativa (Rice)moderately similar to ( 321) loc_os08g33660 12008.m07356 protein MYB1, putative, expressed   no original description 	T
30.2.24	signalling.receptor kinases.S-locus glycoprotein like	MDP0000316310	 moderately similar to ( 458) AT1G78860 | Symbols:  | curculin-like (mannose-binding) lectin family protein | chr1:29646168-29647499 REVERSEmoderately similar to ( 454) EP1G_DAUCA Epidermis-specific secreted glycoprotein EP1 precursor (52/54 kDa medium protein) - Daucus carota (Carrot)weakly similar to ( 163) loc_os04g23760 12004.m07515 protein ATP binding protein, putative  no original description 	T
26.9	misc.glutathione S transferases	MDP0000273588	 moderately similar to ( 308) AT1G10370 | Symbols: GST30, ATGSTU17, GST30B, ERD9 | ERD9 (EARLY-RESPONSIVE TO DEHYDRATION 9); glutathione transferase | chr1:3397274-3398273 REVERSEmoderately similar to ( 220) GSTU6_ORYSA Probable glutathione S-transferase GSTU6 (EC 2.5.1.18) (28 kDa cold-induced protein) - Oryza sativa (Rice)moderately similar to ( 241) loc_os10g38710 12010.m06639 protein glutathione S-transferase GSTU6, putative, expressed  no original description 	T
20.2.99	stress.abiotic.unspecified	MDP0000947031	 weakly similar to ( 118) AT1G70840 | Symbols: MLP31 | MLP31 (MLP-LIKE PROTEIN 31) | chr1:26713170-26714014 REVERSEvery weakly similar to (91.7) loc_os04g39150 12004.m35333 protein major latex protein 22, putative, expressed no original description 	T
35.2	not assigned.unknown	MDP0000345166	 weakly similar to ( 104) AT4G39300 | Symbols:  | unknown protein | chr4:18286835-18288156 FORWARDvery weakly similar to (52.0) loc_os06g22654 12006.m91834 protein expressed protein no original description 	T
35.2	not assigned.unknown	MDP0000209492	  no original description 	T
27.3.99	RNA.regulation of transcription.unclassified	MDP0000250153	 moderately similar to ( 334) AT3G52500 | Symbols:  | aspartyl protease family protein | chr3:19465644-19467053 REVERSEvery weakly similar to (53.9) 7SBG2_SOYBN Basic 7S globulin 2 precursor (Bg) (SBg7S) [Contains: Basic 7S globulin 2 high kDa subunit; Basic 7S globulin 2 low kDa subunit] - Glycine max (Soybean)moderately similar to ( 204) loc_os04g58840 12004.m10764 protein aspartic proteinase nepenthesin-2 precursor, putative, expressed  PRK08026  no original description 	T
29.1.19	protein.aa activation.arginine-tRNA ligase	MDP0000210518	 highly similar to ( 683) AT4G26300 | Symbols: emb1027 | emb1027 (embryo defective 1027); ATP binding / aminoacyl-tRNA ligase/ arginine-tRNA ligase/ nucleotide binding | chr4:13308400-13313109 REVERSEhighly similar to ( 648) loc_os05g07030 12005.m083624 protein arginyl-tRNA synthetase, putative, expressed  no original description 	T
35.2	not assigned.unknown	MDP0000224960	 moderately similar to ( 310) AT3G61700 | Symbols:  | unknown protein | chr3:22836414-22838803 FORWARDmoderately similar to ( 280) loc_os01g12080 12001.m42921 protein plant-specific domain TIGR01589 family protein, expressed  no original description 	T
26.2	misc.UDP glucosyl and glucoronyl transferases	MDP0000693745	 moderately similar to ( 498) AT1G74680 | Symbols:  | exostosin family protein | chr1:28059528-28060984 FORWARDvery weakly similar to (57.8) KATAM_ORYSA Xyloglucan galactosyltransferase KATAMARI1 homolog (EC 2.4.1.-) - Oryza sativa (Rice)moderately similar to ( 484) loc_os08g34020 12008.m080169 protein secondary cell wall-related glycosyltransferase family 47, putative, expressed  no original description 	T
29.5.2	protein.degradation.autophagy	MDP0000339918	 weakly similar to ( 188) AT3G15580 | Symbols: ATG8I, APG8H | APG8H (AUTOPHAGY 8H); APG8 activating enzyme/ APG8-specific protease/ Atg8 ligase/ microtubule binding | chr3:5274075-5275000 REVERSEweakly similar to ( 165) loc_os11g01010 12011.m04306 protein autophagy-related protein 8 precursor, putative, expressed no original description 	T
35.2	not assigned.unknown	MDP0000272331	 moderately similar to ( 478) AT1G07040 | Symbols:  | unknown protein | chr1:2161225-2163035 REVERSEmoderately similar to ( 459) loc_os10g32680 12010.m065282 protein expressed protein  no original description 	T
35.1	not assigned.no ontology	MDP0000144978	 weakly similar to ( 183) AT5G62100 | Symbols: ATBAG2 | ATBAG2 (ARABIDOPSIS THALIANA BCL-2-ASSOCIATED ATHANOGENE 2) | chr5:24940477-24941528 FORWARDmoderately similar to ( 264) loc_os04g52890 12004.m101657 protein BCL-2 binding anthanogene-1, putative, expressed no original description 	T
35.2	not assigned.unknown	MDP0000787137	 moderately similar to ( 301) AT1G35180 | Symbols:  | FUNCTIONS IN: molecular_function unknown; INVOLVED IN: biological_process unknown; LOCATED IN: integral to membrane; CONTAINS InterPro DOMAIN/s: TRAM, LAG1 and CLN8 homology (InterPro:IPR006634); BEST Arabidopsis thaliana protein match is: unknown protein (TAIR:AT1G35170.1); Has 128 Blast hits to 128 proteins in 42 species: Archae - 0; Bacteria - 0; Metazoa - 49; Fungi - 4; Plants - 41; Viruses - 3; Other Eukaryotes - 31 (source: NCBI BLink). | chr1:12877230-12878644 REVERSEmoderately similar to ( 275) loc_os01g15770 12001.m08175 protein expressed protein no original description 	T
29.2.1.2.2.535	protein.synthesis.ribosomal protein.eukaryotic.60S subunit.L35A	MDP0000245732	 weakly similar to ( 188) AT1G74270 | Symbols:  | 60S ribosomal protein L35a (RPL35aC) | chr1:27928415-27929466 REVERSEweakly similar to ( 175) loc_os02g54470 12002.m100389 protein 60S ribosomal protein L33-B, putative, expressed no original description 	T
30.11	signalling.light	MDP0000175481	 moderately similar to ( 492) AT3G15570 | Symbols:  | phototropic-responsive NPH3 family protein | chr3:5270267-5271700 REVERSEmoderately similar to ( 251) NPH3_ORYSA Coleoptile phototropism protein 1 (Non-phototropic hypocotyl 3-like protein) (NPH3-like protein) - Oryza sativa (Rice)moderately similar to ( 291) loc_os05g08530 12005.m05369 protein coleoptile phototropism protein 1, putative, expressed   no original description 	T
13.2.4.1	amino acid metabolism.degradation.branched chain group.shared	MDP0000124047	 highly similar to ( 551) AT1G55510 | Symbols: BCDH BETA1 | BCDH BETA1 (BRANCHED-CHAIN ALPHA-KETO ACID DECARBOXYLASE E1 BETA SUBUNIT); 3-methyl-2-oxobutanoate dehydrogenase (2-methylpropanoyl-transferring)/ catalytic | chr1:20723482-20725505 FORWARDweakly similar to ( 172) ODPB_PEA Pyruvate dehydrogenase E1 component subunit beta, mitochondrial precursor (EC 1.2.4.1) (PDHE1-B) - Pisum sativum (Garden pea)highly similar to ( 530) loc_os07g07470 12007.m05206 protein 3-methyl-2-oxobutanoate dehydrogenase, putative, expressed  no original description 	T
34.13	transport.peptides and oligopeptides	MDP0000260604	 highly similar to ( 833) AT1G32450 | Symbols: NRT1.5 | NRT1.5 (NITRATE TRANSPORTER 1.5); nitrate transmembrane transporter/ transporter | chr1:11715337-11719807 REVERSEhighly similar to ( 612) loc_os02g46460 12002.m09683 protein peptide transporter PTR2, putative, expressed  no original description 	T
22.1.6	polyamine metabolism.synthesis.spermidine synthase	MDP0000198590	 moderately similar to ( 499) AT1G23820 | Symbols: SPDS1 | SPDS1 (spermidine synthase 1); spermidine synthase | chr1:8420278-8422724 FORWARDhighly similar to ( 511) SPD1_PEA Spermidine synthase 1 (EC 2.5.1.16) (Putrescine aminopropyltransferase 1) (SPDSY 1) - Pisum sativum (Garden pea)highly similar to ( 501) loc_os07g22600 12007.m29134 protein spermidine synthase 1, putative, expressed  no original description 	T
35.2	not assigned.unknown	MDP0000260557	 moderately similar to ( 420) AT1G68390 | Symbols:  | unknown protein | chr1:25642838-25645484 FORWARDmoderately similar to ( 391) loc_os03g52120 12003.m10189 protein expressed protein MSP1_C  no original description 	T
26.7	misc.oxidases - copper, flavone etc	MDP0000138668	 moderately similar to ( 499) AT4G13010 | Symbols:  | oxidoreductase, zinc-binding dehydrogenase family protein | chr4:7600682-7602567 FORWARDmoderately similar to ( 474) QORH_SPIOL Chloroplastic quinone-oxidoreductase homolog (EC 1.-.-.-) (ceQORH) - Spinacia oleracea (Spinach)moderately similar to ( 419) loc_os04g30420 12004.m08147 protein chloroplastic quinone-oxidoreductase, putative, expressed no original description 	T
35.2	not assigned.unknown	MDP0000133920	  no original description 	T
20.2.1	stress.abiotic.heat	MDP0000191939	 weakly similar to ( 138) AT4G36040 | Symbols:  | DNAJ heat shock N-terminal domain-containing protein (J11) | chr4:17049708-17050193 REVERSEvery weakly similar to (50.4) DNJH_CUCSA DnaJ protein homolog (DNAJ-1) - Cucumis sativus (Cucumber)very weakly similar to ( 100) loc_os08g43490 12008.m08323 protein chaperone protein dnaJ 11, chloroplast precursor, putative, expressed  no original description 	T
35.1	not assigned.no ontology	MDP0000263586	 weakly similar to ( 144) AT5G14030 | Symbols:  | translocon-associated protein beta (TRAPB) family protein | chr5:4526878-4528253 FORWARDweakly similar to ( 139) loc_os01g01307 12001.m42517 protein translocon-associated protein beta containing protein, expressed  no original description 	T
31.1	cell.organisation	MDP0000302440	 highly similar to ( 718) AT3G12110 | Symbols: ACT11 | ACT11 (actin-11); structural constituent of cytoskeleton | chr3:3858116-3859609 FORWARDhighly similar to ( 723) ACT_GOSHI Actin - Gossypium hirsutum (Upland cotton)highly similar to ( 720) loc_os03g50890 12003.m10074 protein actin-1, putative, expressed  no original description 	T
31.1	cell.organisation	MDP0000572047	 highly similar to ( 750) AT5G09810 | Symbols: ACT2, ACT7 | ACT7 (ACTIN 7); structural constituent of cytoskeleton | chr5:3052809-3054220 FORWARDhighly similar to ( 742) ACT3_ORYSA Actin-3 - Oryza sativa (Rice)highly similar to ( 744) loc_os11g06390 12011.m28655 protein actin-7, putative, expressed  no original description 	T
26.19	misc.plastocyanin-like	MDP0000669625	 weakly similar to ( 170) AT5G26330 | Symbols:  | plastocyanin-like domain-containing protein / mavicyanin, putative | chr5:9241614-9242635 REVERSEvery weakly similar to (83.6) BCP_PEA Blue copper protein precursor - Pisum sativum (Garden pea)weakly similar to ( 106) loc_os07g38290 12007.m08076 protein copper ion binding protein, putative, expressed   no original description 	T
21.1	redox.thioredoxin	MDP0000323884	 weakly similar to ( 188) AT3G15360 | Symbols: ATHM4, TRX-M4, ATM4 | TRX-M4 (ARABIDOPSIS THIOREDOXIN M-TYPE 4); enzyme activator | chr3:5188448-5189457 FORWARDweakly similar to ( 171) TRXM_PEA Thioredoxin M-type, chloroplast precursor (TRX-M) - Pisum sativum (Garden pea)weakly similar to ( 172) loc_os04g44830 12004.m09440 protein thioredoxin M-type 2, chloroplast precursor, putative, expressed  no original description 	T
2.1.2.1	major CHO metabolism.synthesis.starch.AGPase	MDP0000258518	 highly similar to ( 768) AT5G19220 | Symbols: ADG2, APL1 | APL1 (ADP GLUCOSE PYROPHOSPHORYLASE LARGE SUBUNIT 1); glucose-1-phosphate adenylyltransferase | chr5:6463931-6466775 REVERSEhighly similar to ( 753) GLGL3_SOLTU Glucose-1-phosphate adenylyltransferase large subunit 3, chloroplast precursor (EC 2.7.7.27) (ADP-glucose synthase) (ADP-glucose pyrophosphorylase) (AGPase S) (Alpha-D-glucose-1-phosphate adenyl transferase) - Solanum tuberosum (Potatohighly similar to ( 711) loc_os03g52460 12003.m10222 protein glucose-1-phosphate adenylyltransferase large subunit 3, chloroplast precursor, putative, expressed  no original description 	T
29.8	protein.assembly and cofactor ligation	MDP0000137031	 moderately similar to ( 314) AT1G59840 | Symbols: CCB4 | CCB4 (COFACTOR ASSEMBLY OF COMPLEX C) | chr1:22026569-22028174 FORWARDmoderately similar to ( 265) loc_os04g55690 12004.m10460 protein expressed protein  no original description 	T
34.1.1	transport.p- and v-ATPases.H+-transporting two-sector ATPase	MDP0000832297	 very weakly similar to (60.8) AT4G34720 | Symbols: AVA-P1, VHA-C1, ATVHA-C1 | AVA-P1; ATPase/ proton-transporting ATPase, rotational mechanism | chr4:16568223-16569165 REVERSEvery weakly similar to (60.8) VATL_TOBAC Vacuolar ATP synthase 16 kDa proteolipid subunit (EC 3.6.3.14) - Nicotiana tabacum (Common tobacco)very weakly similar to (60.8) loc_os12g07140 12012.m04703 protein expressed protein  no original description 	T
27.3.23	RNA.regulation of transcription.HSF,Heat-shock transcription factor family	MDP0000319910	 moderately similar to ( 336) AT3G22830 | Symbols: AT-HSFA6B, HSFA6B | AT-HSFA6B; DNA binding / transcription factor | chr3:8078981-8080895 FORWARDmoderately similar to ( 309) ARD2_ORYSA 1,2-dihydroxy-3-keto-5-methylthiopentene dioxygenase 2 (EC 1.13.-.-) (Aci-reductone dioxygenase 2) (Submergence-induced protein 2A) - Oryza sativa (Rice)moderately similar to ( 309) loc_os03g06620 12003.m06180 protein 1,2-dihydroxy-3-keto-5-methylthiopentene dioxygenase 2, putative, expressed  no original description 	T
35.1	not assigned.no ontology	MDP0000479020	 highly similar to ( 685) AT3G27320 | Symbols:  | hydrolase | chr3:10090307-10092391 FORWARDweakly similar to ( 115) GID1_ORYSA Gibberellin receptor GID1 (EC 3.-.-.-) (Gibberellin-insensitive dwarf protein 1) (Protein GIBBERELLIN INSENSITIVE DWARF1) - Oryza sativa (Rice)highly similar to ( 542) loc_os01g67600 12001.m12864 protein esterase, putative, expressed   no original description 	T
33.99	development.unspecified	MDP0000022758	 moderately similar to ( 468) AT3G61790 | Symbols:  | seven in absentia (SINA) family protein | chr3:22871974-22873543 REVERSEweakly similar to ( 148) SINAL_DROME Probable E3 ubiquitin-protein ligase sina-like CG13030 (EC 6.3.2.-) - Drosophila melanogaster (Fruit fly)highly similar to ( 554) loc_os07g46560 12007.m08873 protein ubiquitin ligase SINAT5, putative, expressed  no original description 	T
35.2	not assigned.unknown	MDP0000134805	 moderately similar to ( 303) loc_os05g49060 12005.m08976 protein expressed protein no original description 	T
29.2.4	protein.synthesis.elongation	MDP0000532501	 highly similar to ( 853) AT5G60390 | Symbols:  | elongation factor 1-alpha / EF-1-alpha | chr5:24289226-24290675 FORWARDhighly similar to ( 854) EF1A_TOBAC Elongation factor 1-alpha (EF-1-alpha) (Vitronectin-like adhesion protein 1) (PVN1) - Nicotiana tabacum (Common tobacco)highly similar to ( 854) loc_os03g08050 12003.m78808 protein elongation factor 1-alpha, putative, expressed   no original description 	T
34.2	transport.sugars	MDP0000193050	 highly similar to ( 818) AT5G26340 | Symbols: MSS1, STP13 | MSS1; carbohydrate transmembrane transporter/ hexose:hydrogen symporter/ high-affinity hydrogen:glucose symporter/ sugar:hydrogen symporter | chr5:9243851-9246994 REVERSEhighly similar to ( 577) STC_RICCO Sugar carrier protein C - Ricinus communis (Castor bean)highly similar to ( 795) loc_os03g11900 12003.m06642 protein sugar transport protein 8, putative, expressed   no original description 	T
8.1.1.1	TCA / org transformation.TCA.pyruvate DH.E1	MDP0000178814	 highly similar to ( 619) AT5G09300 | Symbols:  | 2-oxoisovalerate dehydrogenase, putative / 3-methyl-2-oxobutanoate dehydrogenase, putative / branched-chain alpha-keto acid dehydrogenase E1 alpha subunit, putative | chr5:2884282-2886291 REVERSEweakly similar to ( 135) ODPA_SOLTU Pyruvate dehydrogenase E1 component subunit alpha, mitochondrial precursor (EC 1.2.4.1) (PDHE1-A) - Solanum tuberosum (Potato)highly similar to ( 632) loc_os12g08260 12012.m073783 protein pyruvate dehydrogenase E1 component, alpha subunit, putative, expressed  no original description 	T
26.4.1	misc.beta 1,3 glucan hydrolases.glucan endo-1,3-beta-glucosidase	MDP0000147201	 moderately similar to ( 494) AT2G27500 | Symbols:  | glycosyl hydrolase family 17 protein | chr2:11752364-11753514 REVERSEmoderately similar to ( 261) E13B_WHEAT Glucan endo-1,3-beta-glucosidase precursor (EC 3.2.1.39) ((1->3)-beta-glucan endohydrolase) ((1->3)-beta-glucanase) (Beta-1,3-endoglucanase) - Triticum aestivum (Wheat)moderately similar to ( 467) loc_os01g64170 12001.m150784 protein glucan endo-1,3-beta-glucosidase 7 precursor, putative, expressed   no original description 	T
35.2	not assigned.unknown	MDP0000911385	 nearly identical (1013) AT3G13772 | Symbols:  | endomembrane protein 70, putative | chr3:4521712-4524394 REVERSEhighly similar to ( 913) loc_os06g37160 12006.m08251 protein transmembrane 9 superfamily protein member 4, putative, expressed  no original description 	T
29.2.1.2.2.82	protein.synthesis.ribosomal protein.eukaryotic.60S subunit.P2	MDP0000874687	 weakly similar to ( 110) AT2G27710 | Symbols:  | 60S acidic ribosomal protein P2 (RPP2B) | chr2:11816929-11817670 FORWARDvery weakly similar to ( 100) RLA2B_MAIZE 60S acidic ribosomal protein P2B - Zea mays (Maize)weakly similar to ( 104) loc_os05g37330 12005.m07913 protein 60S acidic ribosomal protein P2B, putative, expressed  no original description 	T
13.1.6.1.1	amino acid metabolism.synthesis.aromatic aa.chorismate.3-deoxy-D-arabino-heptulosonate 7-phosphate synthase	MDP0000654068	 highly similar to ( 828) AT1G22410 | Symbols:  | 2-dehydro-3-deoxyphosphoheptonate aldolase, putative / 3-deoxy-D-arabino-heptulosonate 7-phosphate synthase, putative / DAHP synthetase, putative | chr1:7912120-7914742 FORWARDhighly similar to ( 898) AROF_TOBAC Phospho-2-dehydro-3-deoxyheptonate aldolase 1, chloroplast precursor (EC 2.5.1.54) (Phospho-2-keto-3-deoxyheptonate aldolase 1) (DAHP synthetase 1) (3-deoxy-D-arabino-heptulosonate 7-phosphate synthase 1) - Nicotiana tabacum (Common tobhighly similar to ( 826) loc_os07g42960 12007.m29007 protein phospho-2-dehydro-3-deoxyheptonate aldolase 1, chloroplast precursor, putative, expressed   no original description 	T
35.2	not assigned.unknown	MDP0000319067	 very weakly similar to (55.1) loc_os04g44070 12004.m09365 protein expressed protein  no original description 	T
20.2.3	stress.abiotic.drought/salt	MDP0000303602	 moderately similar to ( 268) AT1G47980 | Symbols:  | unknown protein | chr1:17691985-17693818 REVERSEmoderately similar to ( 267) loc_os03g22470 12003.m07649 protein desiccation-related protein PCC13-62 precursor, putative, expressed no original description 	T
29.5.3	protein.degradation.cysteine protease	MDP0000798978	 moderately similar to ( 236) AT5G03330 | Symbols:  | OTU-like cysteine protease family protein | chr5:807728-809608 FORWARDmoderately similar to ( 228) loc_os02g57410 12002.m33952 protein cysteine-type peptidase, putative, expressed no original description 	T
33.99	development.unspecified	MDP0000121700	 weakly similar to ( 132) AT1G32400 | Symbols: TOM2A | TOM2A (TOBAMOVIRUS MULTIPLICATION 2A); protein binding | chr1:11689393-11690873 REVERSEweakly similar to ( 104) loc_os08g18044 12008.m05923 protein tetraspanin family protein, expressed   no original description 	T
21.1	redox.thioredoxin	MDP0000898292	 highly similar to ( 716) AT4G04950 | Symbols:  | thioredoxin family protein | chr4:2517882-2519924 REVERSEvery weakly similar to (74.7) TRXH_ORYSA Thioredoxin H-type (TRX-H) (Phloem sap 13 kDa protein 1) - Oryza sativa (Rice)highly similar to ( 669) loc_os10g35720 12010.m22006 protein OsGrx_S17 - glutaredoxin subgroup II, expressed  no original description 	T
30.2.99	signalling.receptor kinases.misc	MDP0000189841	 highly similar to ( 600) AT5G63410 | Symbols:  | leucine-rich repeat transmembrane protein kinase, putative | chr5:25395173-25397768 REVERSEweakly similar to ( 133) PSKR_DAUCA Phytosulfokine receptor precursor (EC 2.7.11.1) (Phytosulfokine LRR receptor kinase) - Daucus carota (Carrot)moderately similar to ( 399) loc_os11g20360 12011.m28791 protein ATP binding protein, putative, expressed  no original description 	T
29.4.1.57	protein.postranslational modification.kinase.receptor like cytoplasmatic kinase VII	MDP0000189841	 highly similar to ( 600) AT5G63410 | Symbols:  | leucine-rich repeat transmembrane protein kinase, putative | chr5:25395173-25397768 REVERSEweakly similar to ( 133) PSKR_DAUCA Phytosulfokine receptor precursor (EC 2.7.11.1) (Phytosulfokine LRR receptor kinase) - Daucus carota (Carrot)moderately similar to ( 399) loc_os11g20360 12011.m28791 protein ATP binding protein, putative, expressed  no original description 	T
29.2.4	protein.synthesis.elongation	MDP0000701209	 moderately similar to ( 327) AT1G09640 | Symbols:  | elongation factor 1B-gamma, putative / eEF-1B gamma, putative | chr1:3120162-3122152 FORWARDmoderately similar to ( 330) EF1G2_ORYSA Elongation factor 1-gamma 2 (EF-1-gamma 2) (eEF-1B gamma 2) - Oryza sativa (Rice)moderately similar to ( 329) loc_os02g12794 12002.m100462 protein elongation factor 1-gamma 2, putative, expressed no original description 	T
35.2	not assigned.unknown	MDP0000258332	 moderately similar to ( 295) AT1G27595 | Symbols:  | EXPRESSED IN: 22 plant structures; EXPRESSED DURING: 15 growth stages; BEST Arabidopsis thaliana protein match is: ESP4 (ENHANCED SILENCING PHENOTYPE 4); binding (TAIR:AT5G01400.1); Has 529 Blast hits to 235 proteins in 85 species: Archae - 0; Bacteria - 3; Metazoa - 125; Fungi - 41; Plants - 31; Viruses - 0; Other Eukaryotes - 329 (source: NCBI BLink). | chr1:9596475-9602917 FORWARDmoderately similar to ( 328) loc_os01g49940 12001.m11179 protein expressed protein   no original description 	T
26.21	misc.protease inhibitor/seed storage/lipid transfer protein (LTP) family protein	MDP0000265942	 weakly similar to ( 130) AT2G45180 | Symbols:  | protease inhibitor/seed storage/lipid transfer protein (LTP) family protein | chr2:18626377-18626781 FORWARDweakly similar to ( 123) 14KD_DAUCA 14 kDa proline-rich protein DC2.15 precursor - Daucus carota (Carrot)weakly similar to ( 119) loc_os04g46820 12004.m09630 protein cortical cell-delineating protein precursor, putative  no original description 	T
27.3.99	RNA.regulation of transcription.unclassified	MDP0000153127	 moderately similar to ( 306) AT1G23280 | Symbols:  | MAK16 protein-related | chr1:8260865-8262650 REVERSEmoderately similar to ( 283) loc_os07g16950 12007.m079650 protein MAK16-like protein RBM13, putative, expressed  no original description 	T
30.5	signalling.G-proteins	MDP0000259167	 moderately similar to ( 286) AT5G59840 | Symbols:  | Ras-related GTP-binding family protein | chr5:24107450-24109049 REVERSEmoderately similar to ( 286) RB1BV_BETVU Ras-related protein RAB1BV - Beta vulgaris (Sugar beet)moderately similar to ( 285) loc_os07g13530 12007.m29111 protein ras-related protein ARA-3, putative, expressed  no original description 	T
34.13	transport.peptides and oligopeptides	MDP0000282789	 highly similar to ( 681) AT1G22540 | Symbols:  | proton-dependent oligopeptide transport (POT) family protein | chr1:7964202-7966222 FORWARDhighly similar to ( 560) loc_os05g35650 12005.m07797 protein peptide transporter PTR2-B, putative, expressed  no original description 	T
26.22	misc.short chain dehydrogenase/reductase (SDR)	MDP0000269546	 moderately similar to ( 402) AT3G12800 | Symbols: SDRB, DECR | SDRB (SHORT-CHAIN DEHYDROGENASE-REDUCTASE B); binding / catalytic/ oxidoreductase | chr3:4063463-4064757 REVERSEvery weakly similar to (98.6) GRDH_DAUCA Glucose and ribitol dehydrogenase (EC 1.1.1.-) (Carrot ABA-induced in somatic embryos 5 protein) - Daucus carota (Carrot)moderately similar to ( 400) loc_os04g52400 12004.m10134 protein peroxisomal 2,4-dienoyl-CoA reductase, putative, expressed      no original description 	T
31.1	cell.organisation	MDP0000691891	 moderately similar to ( 269) AT4G22240 | Symbols:  | plastid-lipid associated protein PAP, putative | chr4:11766090-11767227 REVERSEmoderately similar to ( 285) LIPC_SOLTU Light-induced protein, chloroplast precursor (Chloroplastic drought-induced stress protein CDSP-34) - Solanum tuberosum (Potato)moderately similar to ( 241) loc_os09g04790 12009.m03848 protein plastid-lipid-associated protein 2, chloroplast precursor, putative, expressed no original description 	T
5.10	fermentation.aldehyde dehydrogenase	MDP0000419420	 moderately similar to ( 397) AT4G34240 | Symbols: ALDH3I1, ALDH3 | ALDH3I1 (ALDEHYDE DEHYDROGENASE 3|1); 3-chloroallyl aldehyde dehydrogenase/ aldehyde dehydrogenase (NAD) | chr4:16389801-16392633 FORWARDvery weakly similar to (95.5) BADH_SPIOL Betaine-aldehyde dehydrogenase, chloroplast precursor (EC 1.2.1.8) (BADH) - Spinacia oleracea (Spinach)moderately similar to ( 373) loc_os11g08300 12011.m28749 protein aldehyde dehydrogenase, dimeric NADP-preferring, putative, expressed  no original description 	T
29.2.1.1.1.2.17	protein.synthesis.ribosomal protein.prokaryotic.chloroplast.50S subunit.L17	MDP0000378945	 moderately similar to ( 268) AT3G54210 | Symbols:  | ribosomal protein L17 family protein | chr3:20067672-20068385 REVERSEmoderately similar to ( 259) RK17_TOBAC 50S ribosomal protein L17, chloroplast precursor (CL17) - Nicotiana tabacum (Common tobacco)moderately similar to ( 231) loc_os03g60100 12003.m10904 protein 50S ribosomal protein L17, putative, expressed   no original description 	T
3.5	minor CHO metabolism.others	MDP0000122562	 highly similar to ( 711) AT5G53850 | Symbols:  | haloacid dehalogenase-like hydrolase family protein | chr5:21861617-21864817 REVERSEhighly similar to ( 690) loc_os11g29370 12011.m06847 protein hydrolase, putative, expressed   no original description 	T
28.2	DNA.repair	MDP0000166574	 moderately similar to ( 328) AT5G44680 | Symbols:  | methyladenine glycosylase family protein | chr5:18024461-18025893 REVERSEmoderately similar to ( 296) loc_os01g58550 12001.m150749 protein DNA-3-methyladenine glycosylase I, putative, expressed  no original description 	T
35.2	not assigned.unknown	MDP0000231813	 moderately similar to ( 340) AT3G62580 | Symbols:  | FUNCTIONS IN: molecular_function unknown; INVOLVED IN: biological_process unknown; LOCATED IN: endomembrane system; EXPRESSED IN: 23 plant structures; EXPRESSED DURING: 13 growth stages; BEST Arabidopsis thaliana protein match is: late embryogenesis abundant domain-containing protein / LEA domain-containing protein (TAIR:AT1G72100.1); Has 237 Blast hits to 236 proteins in 85 species: Archae - 0; Bacteria - 4; Metazoa - 103; Fungi - 69; Plants - 61; Viruses - 0; Other Eukaryotes - 0 (source: NCBI BLink). | chr3:23146931-23147735 FORWARDmoderately similar to ( 302) loc_os12g37060 12012.m07491 protein expressed protein TrbL  no original description 	T
35.2	not assigned.unknown	MDP0000226326	 moderately similar to ( 437) AT2G30900 | Symbols:  | unknown protein | chr2:13150484-13152417 FORWARDmoderately similar to ( 393) loc_os01g46350 12001.m10833 protein expressed protein  no original description 	T
27.3.3	RNA.regulation of transcription.AP2/EREBP, APETALA2/Ethylene-responsive element binding protein family	MDP0000413387	 weakly similar to ( 147) AT3G16770 | Symbols: RAP2.3, ATEBP, ERF72 | ATEBP (ETHYLENE-RESPONSIVE ELEMENT BINDING PROTEIN); DNA binding / protein binding / transcription activator/ transcription factor | chr3:5705784-5706768 FORWARDvery weakly similar to (92.8) ERF1_ORYSA Ethylene-responsive transcription factor 1 (Ethylene-responsive element-binding factor 1) (EREBP-1) (OsEREBP1) - Oryza sativa (Rice)very weakly similar to (99.4) loc_os01g21120 12001.m08596 protein ethylene-responsive factor-like protein 1, putative, expressed  no original description 	T
26.2	misc.UDP glucosyl and glucoronyl transferases	MDP0000164913	 nearly identical (1130) AT4G01210 | Symbols:  | transferase, transferring glycosyl groups | chr4:507738-512362 REVERSEhighly similar to ( 927) loc_os01g43380 12001.m10601 protein transferase, transferring glycosyl groups, putative, expressed  no original description 	T
9.3	mitochondrial electron transport / ATP synthesis.electron transfer flavoprotein	MDP0000264913	 moderately similar to ( 395) AT5G43430 | Symbols: ETFBETA | ETFBETA; electron carrier | chr5:17453831-17455111 FORWARDmoderately similar to ( 360) loc_os04g10400 12004.m06292 protein electron transfer flavoprotein beta-subunit, putative, expressed    no original description 	T
35.1.41	not assigned.no ontology.hydroxyproline rich proteins	MDP0000314476	 highly similar to ( 593) AT3G25690 | Symbols: CHUP1 | CHUP1 (CHLOROPLAST UNUSUAL POSITIONING 1) | chr3:9354061-9357757 FORWARDmoderately similar to ( 497) loc_os12g01449 12012.m04148 protein expressed protein   no original description 	T
10.6.3	cell wall.degradation.pectate lyases and polygalacturonases	MDP0000181608	 highly similar to ( 656) AT1G04680 | Symbols:  | pectate lyase family protein | chr1:1304052-1307780 REVERSEmoderately similar to ( 409) PEL_LILLO Pectate lyase precursor (EC 4.2.2.2) - Lilium longiflorum (Trumpet lily)highly similar to ( 640) loc_os04g05050 12004.m35243 protein pectate lyase 8 precursor, putative, expressed  no original description 	T
29.4	protein.postranslational modification	MDP0000175066	 moderately similar to ( 346) AT4G25130 | Symbols:  | peptide methionine sulfoxide reductase, putative | chr4:12898802-12899998 REVERSEmoderately similar to ( 355) MSRA_BRANA Peptide methionine sulfoxide reductase (EC 1.8.4.11) (Protein-methionine-S-oxide reductase) (Peptide-methionine (S)-S-oxide reductase) (Peptide Met(O) reductase) - Brassica napus (Rape)moderately similar to ( 318) loc_os10g41400 12010.m22009 protein peptide methionine sulfoxide reductase, putative, expressed  no original description 	T
8.1.6	TCA / org transformation.TCA.succinyl-CoA ligase	MDP0000091064	 moderately similar to ( 481) AT5G23250 | Symbols:  | succinyl-CoA ligase (GDP-forming) alpha-chain, mitochondrial, putative / succinyl-CoA synthetase, alpha chain, putative / SCS-alpha, putative | chr5:7830460-7832265 FORWARDmoderately similar to ( 477) SUCA_ORYSA Probable succinyl-CoA ligase [GDP-forming] subunit alpha, mitochondrial precursor (EC 6.2.1.4) (Succinyl-CoA synthetase subunit alpha) (SCS-alpha) - Oryza sativa (Rice)moderately similar to ( 477) loc_os07g38970 12007.m08141 protein succinyl-CoA ligase alpha-chain 2, mitochondrial precursor, putative, expressed  no original description 	T
29.3.4.2	protein.targeting.secretory pathway.golgi	MDP0000186940	 moderately similar to ( 440) AT1G73430 | Symbols:  | sec34-like family protein | chr1:27604096-27610829 FORWARDmoderately similar to ( 407) loc_os08g34970 12008.m07486 protein conserved oligomeric Golgi complex component 3, putative, expressed no original description 	T
35.2	not assigned.unknown	MDP0000148339	 moderately similar to ( 279) AT3G07660 | Symbols:  | unknown protein | chr3:2445275-2450372 REVERSEweakly similar to ( 195) loc_os09g08680 12009.m04135 protein expressed protein   no original description 	T
23.3.2.2	nucleotide metabolism.salvage.nucleoside kinases.uridine kinase	MDP0000263091	 highly similar to ( 897) AT1G73980 | Symbols:  | phosphoribulokinase/uridine kinase family protein | chr1:27820292-27823527 REVERSEvery weakly similar to (63.9) KPPR_WHEAT Phosphoribulokinase, chloroplast precursor (EC 2.7.1.19) (Phosphopentokinase) (PRKase) (PRK) - Triticum aestivum (Wheat)highly similar to ( 880) loc_os02g47860 12002.m100333 protein uridine kinase, putative, expressed  no original description 	T
31.1	cell.organisation	MDP0000134669	 moderately similar to ( 351) AT4G39920 | Symbols: POR, TFC C | POR (PORCINO); binding | chr4:18515882-18516919 FORWARDmoderately similar to ( 243) loc_os02g35110 12002.m08599 protein tubulin-specific chaperone C, putative, expressed   no original description 	T
4.2.14	glycolysis.plastid branch.pyruvate kinase (PK)	MDP0000124883	 highly similar to ( 920) AT3G52990 | Symbols:  | pyruvate kinase, putative | chr3:19649336-19652237 FORWARDmoderately similar to ( 375) KPYC_SOLTU Pyruvate kinase, cytosolic isozyme (EC 2.7.1.40) (PK) - Solanum tuberosum (Potato)highly similar to ( 925) loc_os11g05110 12011.m079911 protein pyruvate kinase, cytosolic isozyme, putative, expressed   no original description 	T
1.3.3	PS.calvin cycle.phosphoglycerate kinase	MDP0000784808	 highly similar to ( 674) AT1G79550 | Symbols: PGK | PGK (PHOSPHOGLYCERATE KINASE); phosphoglycerate kinase | chr1:29924347-29926295 REVERSEhighly similar to ( 676) PGKY_TOBAC Phosphoglycerate kinase, cytosolic (EC 2.7.2.3) - Nicotiana tabacum (Common tobacco)highly similar to ( 660) loc_os06g45710 12006.m09099 protein phosphoglycerate kinase, cytosolic, putative, expressed   no original description 	T
31.2	cell.division	MDP0000172418	 nearly identical (1303) AT3G12280 | Symbols: RBR1, RBR, RB, ATRBR1 | RBR1 (RETINOBLASTOMA-RELATED 1); transcription factor binding | chr3:3913671-3918433 REVERSEnearly identical (1156) loc_os08g42600 12008.m08238 protein retinoblastoma-related 3, putative, expressed   no original description 	T
30.11	signalling.light	MDP0000231411	 moderately similar to ( 372) AT2G37680 | Symbols:  | CONTAINS InterPro DOMAIN/s: Vacuolar import and degradation protein Vid24 (InterPro:IPR018618); Has 214 Blast hits to 214 proteins in 84 species: Archae - 0; Bacteria - 0; Metazoa - 57; Fungi - 124; Plants - 31; Viruses - 0; Other Eukaryotes - 2 (source: NCBI BLink). | chr2:15803500-15805515 REVERSEmoderately similar to ( 366) loc_os03g47550 12003.m09764 protein FHY1, putative, expressed MSP1_C  no original description 	T
29.6	protein.folding	MDP0000575276	 weakly similar to ( 158) AT1G14980 | Symbols: CPN10 | CPN10 (CHAPERONIN 10); chaperone binding | chr1:5165930-5166654 REVERSEweakly similar to ( 156) CH10_BRANA 10 kDa chaperonin (Protein CPN10) (Protein groES) - Brassica napus (Rape)weakly similar to ( 136) loc_os07g44740 12007.m08695 protein chaperonin, putative, expressed no original description 	T
30.2.17	signalling.receptor kinases.DUF 26	MDP0000297547	 highly similar to ( 661) AT1G11300 | Symbols:  | ATP binding / carbohydrate binding / kinase/ protein kinase/ protein serine/threonine kinase/ protein tyrosine kinase/ sugar binding | chr1:3794389-3800719 FORWARDmoderately similar to ( 277) SLSG6_BRAOL S-locus-specific glycoprotein S6 precursor (SLSG-6) - Brassica oleracea (Wild cabbage)highly similar to ( 599) loc_os09g37840 12009.m60217 protein ATP binding protein, putative    no original description 	T
30.2.24	signalling.receptor kinases.S-locus glycoprotein like	MDP0000297547	 highly similar to ( 661) AT1G11300 | Symbols:  | ATP binding / carbohydrate binding / kinase/ protein kinase/ protein serine/threonine kinase/ protein tyrosine kinase/ sugar binding | chr1:3794389-3800719 FORWARDmoderately similar to ( 277) SLSG6_BRAOL S-locus-specific glycoprotein S6 precursor (SLSG-6) - Brassica oleracea (Wild cabbage)highly similar to ( 599) loc_os09g37840 12009.m60217 protein ATP binding protein, putative    no original description 	T
35.2	not assigned.unknown	MDP0000157380	 highly similar to ( 672) AT1G55360 | Symbols:  | unknown protein | chr1:20666526-20668502 REVERSEhighly similar to ( 655) loc_os07g10530 12007.m05508 protein carboxyl-terminal peptidase, putative, expressed   no original description 	T
35.2	not assigned.unknown	MDP0000796452	  no original description 	T
13.2.6.3	amino acid metabolism.degradation.aromatic aa.tryptophan	MDP0000258402	 moderately similar to ( 385) AT4G16210 | Symbols: ECHIA, E-COAH-2 | ECHIA (ENOYL-COA HYDRATASE/ISOMERASE A); catalytic | chr4:9176864-9177978 REVERSEvery weakly similar to (93.6) MFPA_CUCSA Glyoxysomal fatty acid beta-oxidation multifunctional protein MFP-a [Includes: Enoyl-CoA hydratase (EC 4.2.1.17); 3-2-trans-enoyl-CoA isomerase (EC 5.3.3.8); 3-hydroxybutyryl-CoA epimerase (EC 5.1.2.3); 3-hydroxyacyl-CoA dehydrogenase (moderately similar to ( 344) loc_os03g19680 12003.m07384 protein 3-hydroxybutyryl-CoA dehydratase, putative, expressed   no original description 	T
26.4.1	misc.beta 1,3 glucan hydrolases.glucan endo-1,3-beta-glucosidase	MDP0000785544	 highly similar to ( 731) AT1G64760 | Symbols:  | glycosyl hydrolase family 17 protein | chr1:24054220-24056194 REVERSEmoderately similar to ( 209) E13B_WHEAT Glucan endo-1,3-beta-glucosidase precursor (EC 3.2.1.39) ((1->3)-beta-glucan endohydrolase) ((1->3)-beta-glucanase) (Beta-1,3-endoglucanase) - Triticum aestivum (Wheat)highly similar to ( 650) loc_os03g57880 12003.m101534 protein glucan endo-1,3-beta-glucosidase 5 precursor, putative, expressed  no original description 	T
34.14	transport.unspecified cations	MDP0000236774	 moderately similar to ( 368) AT5G23630 | Symbols:  | ATPase E1-E2 type family protein / haloacid dehalogenase-like hydrolase familiy protein | chr5:7960756-7967644 REVERSEmoderately similar to ( 355) loc_os05g33390 12005.m07571 protein cation-transporting ATPase, putative, expressed  no original description 	T
21.1	redox.thioredoxin	MDP0000161677	 weakly similar to ( 187) AT5G06690 | Symbols: WCRKC1 | WCRKC1 (WCRKC THIOREDOXIN 1) | chr5:2060852-2061956 REVERSEvery weakly similar to (56.6) TRXM_PEA Thioredoxin M-type, chloroplast precursor (TRX-M) - Pisum sativum (Garden pea)weakly similar to ( 161) loc_os02g53400 12002.m10369 protein thioredoxin-like 5, chloroplast precursor, putative, expressed no original description 	T
35.2	not assigned.unknown	MDP0000771505	  no original description 	T
29.5.11.20	protein.degradation.ubiquitin.proteasom	MDP0000695052	 moderately similar to ( 419) AT1G56450 | Symbols: PBG1 | PBG1; peptidase/ threonine-type endopeptidase | chr1:21141970-21144186 FORWARDmoderately similar to ( 378) loc_os09g33986 12009.m22255 protein proteasome subunit beta type 4 precursor, putative, expressed  no original description 	T
35.1	not assigned.no ontology	MDP0000312257	 highly similar to ( 857) AT1G67230 | Symbols: LINC1 | LINC1 (LITTLE NUCLEI1) | chr1:25151561-25156032 REVERSEvery weakly similar to (55.1) MFP1_TOBAC MAR-binding filament-like protein 1-1 - Nicotiana tabacum (Common tobacco)highly similar to ( 561) loc_os02g48010 12002.m09835 protein nuclear matrix constituent protein 1, putative, expressed  PRK07003  no original description 	T
35.1	not assigned.no ontology	MDP0000688643	 moderately similar to ( 314) AT5G53750 | Symbols:  | FUNCTIONS IN: molecular_function unknown; INVOLVED IN: response to wounding; LOCATED IN: cellular_component unknown; EXPRESSED IN: male gametophyte, pollen tube; EXPRESSED DURING: L mature pollen stage; CONTAINS InterPro DOMAIN/s: Cystathionine beta-synthase, core (InterPro:IPR000644); BEST Arabidopsis thaliana protein match is: CBS domain-containing protein (TAIR:AT4G27460.1); Has 79 Blast hits to 78 proteins in 10 species: Archae - 0; Bacteria - 0; Metazoa - 0; Fungi - 0; Plants - 79; Viruses - 0; Other Eukaryotes - 0 (source: NCBI BLink). | chr5:21817416-21818723 FORWARDmoderately similar to ( 244) loc_os10g35630 12010.m06383 protein expressed protein    no original description 	T
26.7	misc.oxidases - copper, flavone etc	MDP0000154720	 highly similar to ( 616) AT3G24200 | Symbols:  | FAD binding / monooxygenase/ oxidoreductase/ oxidoreductase, acting on paired donors, with incorporation or reduction of molecular oxygen, NADH or NADPH as one donor, and incorporation of one atom of oxygen | chr3:8748095-8751166 REVERSEhighly similar to ( 562) loc_os03g62260 12003.m11104 protein monooxygenase/ oxidoreductase, putative, expressed no original description 	T
29.2.1.99.1.4	protein.synthesis.ribosomal protein.unknown.small subunit.S4	MDP0000132703	 moderately similar to ( 251) AT5G15750 | Symbols:  | RNA-binding S4 domain-containing protein | chr5:5141449-5142701 FORWARDmoderately similar to ( 242) loc_os04g56670 12004.m10554 protein 40S ribosomal protein S4-like, putative, expressed  no original description 	T
26.22	misc.short chain dehydrogenase/reductase (SDR)	MDP0000301237	 moderately similar to ( 360) AT1G24470 | Symbols: KCR2 | short-chain dehydrogenase/reductase (SDR) family protein | chr1:8674056-8676277 FORWARDvery weakly similar to (68.2) SDR1_PICAB Short-chain type dehydrogenase/reductase (EC 1.-.-.-) - Picea abies (Norway spruce) (Picea excelsa)moderately similar to ( 319) loc_os02g38440 12002.m08932 protein steroid dehydrogenase KIK-I, putative, expressed   no original description 	T
31.1	cell.organisation	MDP0000492839	 moderately similar to ( 416) AT5G23860 | Symbols: TUB8 | TUB8; structural constituent of cytoskeleton | chr5:8042962-8044528 FORWARDmoderately similar to ( 416) TBB9_GOSHI Tubulin beta-9 chain (Beta-9 tubulin) - Gossypium hirsutum (Upland cotton)moderately similar to ( 414) loc_os06g46000 12006.m31916 protein tubulin beta-3 chain, putative, expressed   no original description 	T
29.6	protein.folding	MDP0000670888	 highly similar to ( 677) AT5G26360 | Symbols:  | chaperonin, putative | chr5:9255561-9258891 REVERSEweakly similar to ( 169) TCPE2_AVESA T-complex protein 1 subunit epsilon (TCP-1-epsilon) (CCT-epsilon) (TCP-K36) - Avena sativa (Oat)highly similar to ( 660) loc_os06g34690 12006.m08006 protein T-complex protein 1 subunit gamma, putative, expressed no original description 	T
27.3.2	RNA.regulation of transcription.Alfin-like	MDP0000147523	 moderately similar to ( 260) AT5G26210 | Symbols: AL4 | AL4 (ALFIN-LIKE 4); DNA binding / methylated histone residue binding | chr5:9158566-9160221 REVERSEmoderately similar to ( 247) loc_os11g14010 12011.m28769 protein PHD finger protein, putative, expressed   no original description 	T
29.2.1.2.2.19	protein.synthesis.ribosomal protein.eukaryotic.60S subunit.L19	MDP0000772737	 moderately similar to ( 310) AT1G02780 | Symbols: emb2386 | emb2386 (embryo defective 2386); structural constituent of ribosome | chr1:608120-609391 REVERSEweakly similar to ( 112) RL19_MAIZE 60S ribosomal protein L19 (Fragment) - Zea mays (Maize)moderately similar to ( 303) loc_os03g38260 12003.m08925 protein 60S ribosomal protein L19-3, putative, expressed   no original description 	T
35.2	not assigned.unknown	MDP0000782892	 highly similar to ( 627) AT3G14920 | Symbols:  | unknown protein | chr3:5018275-5020104 FORWARDhighly similar to ( 549) loc_os01g10930 12001.m07713 protein peptide-N4-asparagine amidase A, putative, expressed  no original description 	T
21.1	redox.thioredoxin	MDP0000166089	 weakly similar to ( 129) AT3G51030 | Symbols: ATTRX1, ATTRX H1 | ATTRX1; oxidoreductase, acting on sulfur group of donors, disulfide as acceptor | chr3:18951123-18951955 REVERSEweakly similar to ( 137) TRXH_RICCO Thioredoxin H-type (TRX-H) - Ricinus communis (Castor bean)weakly similar to ( 109) loc_os07g08840 12007.m079626 protein thioredoxin H-type, putative, expressed  no original description 	T
27.3.21	RNA.regulation of transcription.GRAS transcription factor family	MDP0000269044	 highly similar to ( 507) AT5G41920 | Symbols:  | scarecrow transcription factor family protein | chr5:16779982-16781199 FORWARDmoderately similar to ( 213) RL27_PEA 60S ribosomal protein L27 - Pisum sativum (Garden pea)highly similar to ( 546) loc_os07g38030 12007.m08052 protein nodulation signaling pathway 2 protein, putative, expressed    no original description 	T
35.2	not assigned.unknown	MDP0000656794	 weakly similar to ( 174) AT5G41050 | Symbols:  | unknown protein | chr5:16433478-16434076 REVERSEweakly similar to ( 148) loc_os09g32988 12009.m06392 protein pollen proteins Ole e I family protein, expressed no original description 	T
34.9	transport.metabolite transporters at the mitochondrial membrane	MDP0000525936	 highly similar to ( 505) AT5G19760 | Symbols:  | dicarboxylate/tricarboxylate carrier (DTC) | chr5:6679591-6681845 REVERSEvery weakly similar to (68.2) BT1_MAIZE Protein brittle-1, chloroplast precursor - Zea mays (Maize)highly similar to ( 509) loc_os05g11780 12005.m05647 protein mitochondrial 2-oxoglutarate/malate carrier protein, putative, expressed no original description 	T
10.2.1	cell wall.cellulose synthesis.cellulose synthase	MDP0000174239	 moderately similar to ( 228) AT5G05170 | Symbols: CESA3, IXR1, ATCESA3, ATH-B, CEV1 | CEV1 (CONSTITUTIVE EXPRESSION OF VSP 1); cellulose synthase/ transferase, transferring glycosyl groups | chr5:1530401-1535090 REVERSEmoderately similar to ( 228) loc_os03g59340 12003.m10832 protein CESA2 - cellulose synthase, expressed  no original description 	T
17.5.2	hormone metabolism.ethylene.signal transduction	MDP0000288468	 highly similar to ( 749) AT2G40940 | Symbols: ERS1, ERS | ERS1 (ETHYLENE RESPONSE SENSOR 1); ethylene binding / protein histidine kinase/ receptor | chr2:17084635-17086819 REVERSEhighly similar to ( 741) ETR1_PRUPE Ethylene receptor (EC 2.7.13.3) - Prunus persica (Peach)highly similar to ( 779) loc_os03g49500 12003.m34910 protein ethylene receptor, putative, expressed  no original description 	T
17.2.3	hormone metabolism.auxin.induced-regulated-responsive-activated	MDP0000811081	 highly similar to ( 704) AT4G03400 | Symbols: DFL2, GH3-10 | DFL2 (DWARF IN LIGHT 2) | chr4:1497675-1499729 REVERSEhighly similar to ( 509) GH35_ORYSA Probable indole-3-acetic acid-amido synthetase GH3.5 (EC 6.3.2.-) (Auxin-responsive GH3-like protein 5) (OsGH3-5) - Oryza sativa (Rice)highly similar to ( 509) loc_os05g50890 12005.m27991 protein indole-3-acetic acid-amido synthetase GH3.5, putative, expressed  no original description 	T
27.3.24	RNA.regulation of transcription.MADS box transcription factor family	MDP0000462145	 weakly similar to ( 131) AT5G62165 | Symbols: AGL42 | AGL42 (AGAMOUS LIKE 42); transcription factor | chr5:24965075-24968437 FORWARDweakly similar to ( 117) MAD50_ORYSA MADS-box transcription factor 50 (OsMADS50) (Protein SUPPRESSOR OF CONSTANS OVEREXPRESSION 1-like) (OsSOC1) (Protein AGAMOUS-like 20) (RMADS208) - Oryza sativa (Rice)weakly similar to ( 113) loc_os10g39130 12010.m21952 protein MADS-box transcription factor 56, putative, expressed no original description 	T
29.5.11.4.4	protein.degradation.ubiquitin.E3.APC	MDP0000127633	 nearly identical (1863) AT5G05560 | Symbols: EMB2771 | EMB2771 (EMBRYO DEFECTIVE 2771); ubiquitin-protein ligase | chr5:1641452-1654685 FORWARD   no original description 	T
29.2.1.2.2.518	protein.synthesis.ribosomal protein.eukaryotic.60S subunit.L18A	MDP0000396774	 moderately similar to ( 417) AT1G29970 | Symbols:  | RPL18AA (60S RIBOSOMAL PROTEIN L18A-1) | chr1:10499952-10501311 REVERSEmoderately similar to ( 318) RL18A_ORYSA 60S ribosomal protein L18a - Oryza sativa (Rice)moderately similar to ( 318) loc_os01g54870 12001.m11648 protein 60S ribosomal protein L18a, putative, expressed   no original description 	T
35.2	not assigned.unknown	MDP0000843477	 weakly similar to ( 189) AT5G43240 | Symbols:  | unknown protein | chr5:17353077-17354765 REVERSEweakly similar to ( 154) loc_os04g50800 12004.m09977 protein expressed protein no original description 	T
29.5.11.20	protein.degradation.ubiquitin.proteasom	MDP0000298783	 moderately similar to ( 453) AT3G26340 | Symbols:  | 20S proteasome beta subunit E, putative | chr3:9650600-9652572 REVERSEmoderately similar to ( 464) PSB5_SPIOL Proteasome subunit beta type 5 precursor (EC 3.4.25.1) (20S proteasome subunit E) (Proteasome epsilon chain) - Spinacia oleracea (Spinach)moderately similar to ( 402) loc_os06g06030 12006.m31985 protein proteasome subunit beta type 5 precursor, putative, expressed no original description 	T
31.1	cell.organisation	MDP0000255045	 nearly identical (2135) AT3G19050 | Symbols: POK2 | POK2 (PHRAGMOPLAST ORIENTING KINESIN 2); ATP binding / microtubule motor | chr3:6578047-6590106 FORWARDhighly similar to ( 556) ARGC_ORYSA Probable N-acetyl-gamma-glutamyl-phosphate reductase, chloroplast precursor (EC 1.2.1.38) (AGPR) (N-acetyl-glutamate semialdehyde dehydrogenase) (NAGSA dehydrogenase) - Oryza sativa (Rice)nearly identical (1826) loc_os12g39980 12012.m07782 protein kinesin like protein, putative, expressed   no original description 	T
4.1.15	glycolysis.cytosolic branch.phospho-enol-pyruvate carboxylase (PEPC)	MDP0000073810	 nearly identical (1675) AT1G53310 | Symbols: ATPPC1 | ATPPC1 (PHOSPHOENOLPYRUVATE CARBOXYLASE 1); catalytic/ phosphoenolpyruvate carboxylase | chr1:19884261-19888070 REVERSEnearly identical (1697) CAPP1_SOYBN Phosphoenolpyruvate carboxylase, housekeeping isozyme (EC 4.1.1.31) (PEPCase) (PEPC 1) - Glycine max (Soybean)nearly identical (1678) loc_os08g27840 12008.m26491 protein phosphoenolpyruvate carboxylase 2, putative, expressed  no original description 	T
20.2.99	stress.abiotic.unspecified	MDP0000725994	 weakly similar to ( 130) AT1G68300 | Symbols:  | universal stress protein (USP) family protein | chr1:25598518-25599261 REVERSEweakly similar to ( 112) loc_os01g32780 12001.m09597 protein ethylene-responsive protein, putative, expressed  no original description 	T
35.2	not assigned.unknown	MDP0000208034	 moderately similar to ( 217) AT5G10840 | Symbols:  | endomembrane protein 70, putative | chr5:3424910-3427797 REVERSEmoderately similar to ( 209) loc_os09g38530 12009.m06794 protein transmembrane 9 superfamily protein member 4, putative, expressed no original description 	T
35.2	not assigned.unknown	MDP0000124008	   no original description 	T
31.4	cell.vesicle transport	MDP0000175636	 nearly identical (1461) AT4G31480 | Symbols:  | coatomer beta subunit, putative / beta-coat protein, putative / beta-COP, putative | chr4:15264076-15267384 FORWARDnearly identical (1433) loc_os11g07280 12011.m28658 protein coatomer subunit beta, putative, expressed   no original description 	T
35.2	not assigned.unknown	MDP0000320442	 nearly identical (1353) AT1G76850 | Symbols: SEC5A | SEC5A (EXOCYST COMPLEX COMPONENT SEC5) | chr1:28848013-28854282 FORWARDnearly identical (1191) loc_os04g34450 12004.m08539 protein expressed protein   no original description 	T
28.1	DNA.synthesis/chromatin structure	MDP0000189373	 moderately similar to ( 283) AT1G74560 | Symbols: NRP1 | NRP1 (NAP1-RELATED PROTEIN 1); DNA binding / chromatin binding / histone binding | chr1:28017763-28019900 REVERSEmoderately similar to ( 273) loc_os02g36710 12002.m08758 protein protein SET, putative, expressedMSP1_C  no original description 	T
16.1.1.6	secondary metabolism.isoprenoids.non-mevalonate pathway.HDS	MDP0000340322	 nearly identical (1232) AT5G60600 | Symbols: GCPE, ISPG, CSB3, CLB4, HDS | HDS (4-HYDROXY-3-METHYLBUT-2-ENYL DIPHOSPHATE SYNTHASE); 4 iron, 4 sulfur cluster binding / 4-hydroxy-3-methylbut-2-en-1-yl diphosphate synthase | chr5:24359447-24363019 FORWARDnearly identical (1178) loc_os02g39160 12002.m09004 protein hydroxymethylbutenyl 4-diphosphate synthase, putative, expressed  no original description 	T
20.2.3	stress.abiotic.drought/salt	MDP0000258604	 nearly identical (1078) AT4G02900 | Symbols:  | early-responsive to dehydration protein-related / ERD protein-related | chr4:1284066-1287747 FORWARDvery weakly similar to (53.1) GLGB_ORYSA 1,4-alpha-glucan branching enzyme, chloroplast precursor (EC 2.4.1.18) (Starch branching enzyme) (Q-enzyme) - Oryza sativa (Rice)nearly identical (1009) loc_os05g32720 12005.m07505 protein ERD4 protein, putative, expressed  no original description 	T
35.2	not assigned.unknown	MDP0000274659	   no original description 	T
35.2	not assigned.unknown	MDP0000322543	 moderately similar to ( 337) AT4G22920 | Symbols: ATNYE1, NYE1 | NYE1 (NON-YELLOWING 1) | chr4:12016776-12017969 REVERSEmoderately similar to ( 299) loc_os09g36200 12009.m06569 protein senescence-inducible chloroplast stay-green protein 1, putative, expressed  no original description 	T
29.2.1.2.2.57	protein.synthesis.ribosomal protein.eukaryotic.60S subunit.L7A	MDP0000235646	 moderately similar to ( 368) AT3G62870 | Symbols:  | 60S ribosomal protein L7A (RPL7aB) | chr3:23242862-23244273 REVERSEmoderately similar to ( 370) RL7A_ORYSA 60S ribosomal protein L7a - Oryza sativa (Rice)moderately similar to ( 370) loc_os09g32976 12009.m22160 protein 60S ribosomal protein L7a, putative, expressed no original description 	T
11.3.3	lipid metabolism.Phospholipid synthesis.phosphatidate cytidylyltransferase	MDP0000217745	 moderately similar to ( 254) AT5G58560 | Symbols:  | phosphatidate cytidylyltransferase family protein | chr5:23670513-23672570 FORWARDmoderately similar to ( 261) PHYK3_SOYBN Probable phytol kinase 3, chloroplast precursor (EC 2.7.-.-) - Glycine max (Soybean)moderately similar to ( 248) loc_os01g61560 12001.m43251 protein phytol kinase 2, chloroplast precursor, putative, expressed no original description 	T
27.1	RNA.processing	MDP0000165292	 highly similar to ( 537) AT1G80670 | Symbols:  | transducin family protein / WD-40 repeat family protein | chr1:30320809-30323543 REVERSEvery weakly similar to (68.6) PF20_CHLRE Flagellar WD repeat protein PF20 - Chlamydomonas reinhardtiihighly similar to ( 525) loc_os08g07960 12008.m26595 protein rae1-like protein, putative, expressed   no original description 	T
28.1.3	DNA.synthesis/chromatin structure.histone	MDP0000361589	 weakly similar to ( 174) AT1G07790 | Symbols: HTB1 | HTB1; DNA binding | chr1:2413049-2413495 FORWARDweakly similar to ( 176) H2B1_MEDTR Probable histone H2B.1 - Medicago truncatula (Barrel medic)weakly similar to ( 170) loc_os01g62230 12001.m12346 protein histone H2B.4, putative, expressed no original description 	T
29.6	protein.folding	MDP0000331298	 weakly similar to ( 164) AT5G17840 | Symbols:  | chaperone protein dnaJ-related | chr5:5895879-5897055 REVERSEweakly similar to ( 157) loc_os03g45344 12003.m101618 protein expressed protein no original description 	T
29.4.1.57	protein.postranslational modification.kinase.receptor like cytoplasmatic kinase VII	MDP0000239500	 moderately similar to ( 370) AT5G38560 | Symbols:  | protein kinase family protein | chr5:15439844-15443007 FORWARDweakly similar to ( 179) PSKR_DAUCA Phytosulfokine receptor precursor (EC 2.7.11.1) (Phytosulfokine LRR receptor kinase) - Daucus carota (Carrot)moderately similar to ( 357) loc_os05g01040 12005.m27516 protein phytosulfokine receptor precursor, putative, expressed no original description 	T
31.1	cell.organisation	MDP0000220181	 nearly identical (1066) AT1G80260 | Symbols: emb1427 | emb1427 (embryo defective 1427); tubulin binding | chr1:30175924-30180511 FORWARDhighly similar to ( 910) loc_os02g32340 12002.m08327 protein transferase, transferring glycosyl groups, putative, expressed  no original description 	T
3.4.3	minor CHO metabolism.myo-inositol.InsP Synthases	MDP0000207103	 highly similar to ( 936) AT5G10170 | Symbols: ATMIPS3, MIPS3 | MIPS3 (MYO-INOSITOL-1-PHOSTPATE SYNTHASE 3); binding / catalytic/ inositol-3-phosphate synthase | chr5:3187538-3190161 REVERSEhighly similar to ( 949) INO1_TOBAC Inositol-3-phosphate synthase (EC 5.5.1.4) (Myo-inositol-1-phosphate synthase) (MI-1-P synthase) (IPS) - Nicotiana tabacum (Common tobacco)highly similar to ( 909) loc_os03g09250 12003.m35064 protein inositol-3-phosphate synthase, putative, expressed  no original description 	T
30.3	signalling.calcium	MDP0000243873	 moderately similar to ( 298) AT5G62390 | Symbols: ATBAG7 | ATBAG7 (ARABIDOPSIS THALIANA BCL-2-ASSOCIATED ATHANOGENE 7); calmodulin binding | chr5:25052377-25054170 REVERSEweakly similar to ( 189) loc_os06g01500 12006.m31819 protein IQ calmodulin-binding motif family protein, expressed    no original description 	T
30.5	signalling.G-proteins	MDP0000336261	 moderately similar to ( 287) AT3G49180 | Symbols: RID3 | RID3 (ROOT INITIATION DEFECTIVE 3); nucleotide binding | chr3:18229810-18231874 FORWARDvery weakly similar to (52.8) GBLP_NICPL Guanine nucleotide-binding protein subunit beta-like protein - Nicotiana plumbaginifolia (Leadwort-leaved tobacco)moderately similar to ( 202) loc_os07g22534 12007.m079655 protein WD-repeat protein 18, putative, expressed   no original description 	T
29.5	protein.degradation	MDP0000288622	 weakly similar to ( 138) AT5G22860 | Symbols:  | serine carboxypeptidase S28 family protein | chr5:7639907-7642945 REVERSEweakly similar to ( 136) loc_os06g43930 12006.m32127 protein lysosomal Pro-X carboxypeptidase precursor, putative, expressed no original description 	T
27.3.71	RNA.regulation of transcription.SNF7	MDP0000292973	 weakly similar to ( 173) AT2G19830 | Symbols: SNF7.2, VPS32 | SNF7.2 | chr2:8558101-8559389 REVERSEweakly similar to ( 165) loc_os09g09480 12009.m059945 protein charged multivesicular body protein 4b, putative, expressed no original description 	T
29.5.11.20	protein.degradation.ubiquitin.proteasom	MDP0000933477	 highly similar to ( 733) AT5G43010 | Symbols: RPT4A | RPT4A; ATPase | chr5:17248563-17251014 REVERSEmoderately similar to ( 325) PRS7_PRUPE 26S protease regulatory subunit 7 (26S proteasome subunit 7) (26S proteasome AAA-ATPase subunit RPT1) (Regulatory particle triple-A ATPase subunit 1) - Prunus persica (Peach)highly similar to ( 723) loc_os02g10640 12002.m06362 protein 26S protease regulatory subunit S10B, putative, expressed no original description 	T
29.5.9	protein.degradation.AAA type	MDP0000933477	 highly similar to ( 733) AT5G43010 | Symbols: RPT4A | RPT4A; ATPase | chr5:17248563-17251014 REVERSEmoderately similar to ( 325) PRS7_PRUPE 26S protease regulatory subunit 7 (26S proteasome subunit 7) (26S proteasome AAA-ATPase subunit RPT1) (Regulatory particle triple-A ATPase subunit 1) - Prunus persica (Peach)highly similar to ( 723) loc_os02g10640 12002.m06362 protein 26S protease regulatory subunit S10B, putative, expressed no original description 	T
29.5.1	protein.degradation.subtilases	MDP0000417582	 highly similar to ( 699) AT1G32940 | Symbols: ATSBT3.5, SBT3.5 | SBT3.5; identical protein binding / serine-type endopeptidase | chr1:11937634-11940856 FORWARDhighly similar to ( 565) loc_os09g36110 12009.m06561 protein subtilisin-like protease precursor, putative, expressed no original description 	T
35.1	not assigned.no ontology	MDP0000252267	 moderately similar to ( 342) AT2G45010 | Symbols:  | unknown protein | chr2:18568782-18569615 FORWARDmoderately similar to ( 340) loc_os03g01210 12003.m35650 protein domain of unknown function DUF614 containing protein, expressed  no original description 	T
12.1.1	N-metabolism.nitrate metabolism.NR	MDP0000164574	 moderately similar to ( 455) AT5G17770 | Symbols: ATCBR, CBR1 | ATCBR (ARABIDOPSIS THALIANA NADH:CYTOCHROME B5 REDUCTASE 1); cytochrome-b5 reductase | chr5:5864543-5866495 REVERSEweakly similar to ( 186) NIA_LOTJA Nitrate reductase [NADH] (EC 1.7.1.1) (NR) - Lotus japonicusmoderately similar to ( 426) loc_os05g40990 12005.m08275 protein NADH-cytochrome b5 reductase, putative, expressed no original description 	T
35.2	not assigned.unknown	MDP0000786486	   no original description 	T
5.10	fermentation.aldehyde dehydrogenase	MDP0000260947	 highly similar to ( 525) AT4G36250 | Symbols: ALDH3F1 | ALDH3F1 (Aldehyde Dehydrogenase 3F1); 3-chloroallyl aldehyde dehydrogenase/ aldehyde dehydrogenase (NAD) | chr4:17151029-17153381 FORWARDweakly similar to ( 124) BADH_SPIOL Betaine-aldehyde dehydrogenase, chloroplast precursor (EC 1.2.1.8) (BADH) - Spinacia oleracea (Spinach)moderately similar to ( 474) loc_os02g43194 12002.m34024 protein fatty aldehyde dehydrogenase, putative, expressed   no original description 	T
13.2.6.3	amino acid metabolism.degradation.aromatic aa.tryptophan	MDP0000204554	 highly similar to ( 511) AT5G65940 | Symbols: CHY1 | CHY1 (BETA-HYDROXYISOBUTYRYL-COA HYDROLASE 1); 3-hydroxyisobutyryl-CoA hydrolase | chr5:26376927-26379161 REVERSEmoderately similar to ( 479) loc_os12g16350 12012.m073804 protein CHY1, putative, expressed  no original description 	T
8.1.8	TCA / org transformation.TCA.fumarase	MDP0000202281	 highly similar to ( 855) AT2G47510 | Symbols: FUM1 | FUM1 (FUMARASE 1); catalytic/ fumarate hydratase | chr2:19498614-19502020 FORWARDhighly similar to ( 818) loc_os03g21950 12003.m07599 protein fumarate hydratase 1, mitochondrial precursor, putative, expressed   no original description 	T
20.1	stress.biotic	MDP0000945042	 moderately similar to ( 496) AT1G05850 | Symbols: POM1, ERH2, ELP1, CTL1, ELP, HOT2, ATCTL1 | POM1 (POM-POM1); chitinase | chr1:1766833-1768117 REVERSEweakly similar to ( 197) CHI2_BRANA Endochitinase CH25 precursor (EC 3.2.1.14) - Brassica napus (Rape)moderately similar to ( 447) loc_os09g32080 12009.m060024 protein endochitinase A2 precursor, putative, expressed  no original description 	T
10.2.1	cell wall.cellulose synthesis.cellulose synthase	MDP0000128267	 nearly identical (1306) AT4G32410 | Symbols: CESA1, RSW1 | CESA1 (CELLULOSE SYNTHASE 1); cellulose synthase/ transferase, transferring glycosyl groups | chr4:15641009-15646388 REVERSEnearly identical (1298) loc_os05g08370 12005.m05354 protein CESA1 - cellulose synthase, expressed  no original description 	T
29.2.1.1.3.2.35	protein.synthesis.ribosomal protein.prokaryotic.unknown organellar.50S subunit.L35	MDP0000264184	 very weakly similar to (64.3) AT5G45590 | Symbols:  | unknown protein | chr5:18484643-18486227 REVERSEvery weakly similar to (60.8) loc_os10g42860 12010.m07038 protein ribosomal protein L35 containing protein, expressed  no original description 	T
17.5.1	hormone metabolism.ethylene.synthesis-degradation	MDP0000777260	 weakly similar to ( 198) AT5G59530 | Symbols:  | 2-oxoglutarate-dependent dioxygenase, putative | chr5:23994434-23995715 REVERSEweakly similar to ( 173) DV4H_CATRO Desacetoxyvindoline 4-hydroxylase (EC 1.14.11.20) - Catharanthus roseus (Rosy periwinkle) (Madagascar periwinkle)weakly similar to ( 171) loc_os04g10350 12004.m06287 protein 1-aminocyclopropane-1-carboxylate oxidase, putative, expressed no original description 	T
35.2	not assigned.unknown	MDP0000941515	 moderately similar to ( 350) AT1G79510 | Symbols:  | unknown protein | chr1:29908271-29909175 REVERSEmoderately similar to ( 236) loc_os04g52330 12004.m10127 protein expressed protein  no original description 	T
35.2	not assigned.unknown	MDP0000362001	 very weakly similar to (76.6) AT1G26355 | Symbols: SP1L1 | SP1L1 (SPIRAL1-LIKE1) | chr1:9117950-9118611 REVERSEvery weakly similar to (68.2) loc_os03g30430 12003.m34882 protein NAP16kDa protein, putative, expressed  no original description 	T
26.2	misc.UDP glucosyl and glucoronyl transferases	MDP0000173397	 highly similar to ( 771) AT1G74380 | Symbols: XXT5 | XXT5 (XYLOGLUCAN XYLOSYLTRANSFERASE 5); transferase/ transferase, transferring glycosyl groups / xyloglucan 6-xylosyltransferase | chr1:27959848-27961221 FORWARDhighly similar to ( 647) loc_os02g32750 12002.m100223 protein glycosyltransferase 5, putative, expressed  Sporozoite_P67  no original description 	T
35.2	not assigned.unknown	MDP0000238718	  no original description 	T
19.7	tetrapyrrole synthesis.uroporphyrinogen decarboxylase	MDP0000364293	 highly similar to ( 545) AT2G40490 | Symbols: HEME2 | HEME2; uroporphyrinogen decarboxylase | chr2:16912961-16914988 FORWARDhighly similar to ( 564) DCUP_TOBAC Uroporphyrinogen decarboxylase, chloroplast precursor (EC 4.1.1.37) (URO-D) (UPD) - Nicotiana tabacum (Common tobacco)highly similar to ( 533) loc_os03g21900 12003.m07594 protein uroporphyrinogen decarboxylase, putative, expressed  no original description 	T
35.2	not assigned.unknown	MDP0000316329	 highly similar to ( 533) AT4G14950 | Symbols:  | unknown protein | chr4:8545517-8547722 FORWARDmoderately similar to ( 490) loc_os01g57066 12001.m42516 protein transmembrane protein 49, putative, expressed  no original description 	T
29.2.4	protein.synthesis.elongation	MDP0000255901	 highly similar to ( 860) AT5G60390 | Symbols:  | elongation factor 1-alpha / EF-1-alpha | chr5:24289226-24290675 FORWARDhighly similar to ( 863) EF1A_ORYSA Elongation factor 1-alpha (EF-1-alpha) - Oryza sativa (Rice)highly similar to ( 863) loc_os03g08050 12003.m78808 protein elongation factor 1-alpha, putative, expressed   no original description 	T
29.2	protein.synthesis	MDP0000291992	 nearly identical (3482) AT1G64790 | Symbols:  | binding | chr1:24065232-24081908 REVERSEnearly identical (1629) loc_os03g51140 12003.m10094 protein GCN1-like protein 1, putative, expressed  MSP1_C  no original description 	T
27.3.35	RNA.regulation of transcription.bZIP transcription factor family	MDP0000121258	 highly similar to ( 527) AT5G10030 | Symbols: TGA4, OBF4 | TGA4 (TGACG MOTIF-BINDING FACTOR 4); DNA binding / calmodulin binding / transcription factor | chr5:3137648-3139295 REVERSEmoderately similar to ( 448) TGA1A_TOBAC TGACG-sequence-specific DNA-binding protein TGA-1A (TGA1a) (ASF-1 protein) - Nicotiana tabacum (Common tobacco)moderately similar to ( 392) loc_os04g54474 12004.m101669 protein transcription factor TGA4, putative, expressed   no original description 	T
35.2	not assigned.unknown	MDP0000287146	   no original description 	T
11.1.8	lipid metabolism.FA synthesis and FA elongation.acyl coa ligase	MDP0000697764	 highly similar to ( 833) AT3G16910 | Symbols: AAE7, ACN1 | AAE7 (ACYL-ACTIVATING ENZYME 7); AMP binding / acetate-CoA ligase | chr3:5773231-5775411 REVERSEweakly similar to ( 119) 4CL2_ORYSA 4-coumarate--CoA ligase 2 (EC 6.2.1.12) (4CL 2) (4-coumaroyl-CoA synthase 2) - Oryza sativa (Rice)highly similar to ( 830) loc_os03g19240 12003.m07341 protein AMP-binding protein, putative, expressed   no original description 	T
35.2	not assigned.unknown	MDP0000423958	  no original description 	T
29.5.11.20	protein.degradation.ubiquitin.proteasom	MDP0000538118	 moderately similar to ( 462) AT5G40580 | Symbols: PBB2 | PBB2; endopeptidase/ peptidase/ threonine-type endopeptidase | chr5:16248511-16250451 REVERSEweakly similar to ( 115) PSB6_TOBAC Proteasome subunit beta type 6 precursor (EC 3.4.25.1) (Proteasome delta chain) (Tobacco cryptogein-induced protein 7) (tcI 7) - Nicotiana tabacum (Common tobacco)moderately similar to ( 422) loc_os05g09490 12005.m083567 protein proteasome subunit beta type 7-A precursor, putative, expressed  no original description 	T
33.1	development.storage proteins	MDP0000183055	 highly similar to ( 532) AT3G63200 | Symbols: PLP9, PLA IIIB | PLP9 (PATATIN-LIKE PROTEIN 9); nutrient reservoir | chr3:23346140-23347379 FORWARDvery weakly similar to (82.8) PAT0_SOLTU Patatin precursor (Potato tuber protein) - Solanum tuberosum (Potato)moderately similar to ( 221) loc_os03g57080 12003.m10628 protein patatin-like protein 3, putative, expressed  TrbL  no original description 	T
29.5.11.4.3.1	protein.degradation.ubiquitin.E3.SCF.SKP	MDP0000273940	 moderately similar to ( 221) AT5G42190 | Symbols: ASK2, SKP1B | ASK2 (ARABIDOPSIS SKP1-LIKE 2); protein binding / ubiquitin-protein ligase | chr5:16854495-16855516 REVERSEmoderately similar to ( 219) loc_os11g26910 12011.m06610 protein SKP1-like protein 1A, putative, expressed no original description 	T
26.19	misc.plastocyanin-like	MDP0000284556	 weakly similar to ( 155) AT3G27200 | Symbols:  | plastocyanin-like domain-containing protein | chr3:10043738-10044340 REVERSEvery weakly similar to (92.0) BCP_PEA Blue copper protein precursor - Pisum sativum (Garden pea)very weakly similar to (97.4) loc_os04g46130 12004.m09564 protein blue copper protein precursor, putative, expressed no original description 	T
29.3.2	protein.targeting.mitochondria	MDP0000227285	 moderately similar to ( 221) AT5G55510 | Symbols:  | P-P-bond-hydrolysis-driven protein transmembrane transporter/ protein transporter | chr5:22486525-22487916 REVERSEweakly similar to ( 190) loc_os03g09870 12003.m06442 protein protein translocase/ protein transporter, putative, expressed  no original description 	T
19.7	tetrapyrrole synthesis.uroporphyrinogen decarboxylase	MDP0000212188	 weakly similar to ( 187) AT2G40490 | Symbols: HEME2 | HEME2; uroporphyrinogen decarboxylase | chr2:16912961-16914988 FORWARDmoderately similar to ( 206) DCUP_TOBAC Uroporphyrinogen decarboxylase, chloroplast precursor (EC 4.1.1.37) (URO-D) (UPD) - Nicotiana tabacum (Common tobacco)weakly similar to ( 179) loc_os03g21900 12003.m07594 protein uroporphyrinogen decarboxylase, putative, expressed no original description 	T
31.3	cell.cycle	MDP0000315218	 nearly identical (1016) AT5G26850 | Symbols:  | INVOLVED IN: biological_process unknown; LOCATED IN: chloroplast; EXPRESSED IN: 23 plant structures; EXPRESSED DURING: 13 growth stages; BEST Arabidopsis thaliana protein match is: cyclin-related (TAIR:AT2G41830.1); Has 199 Blast hits to 197 proteins in 66 species: Archae - 0; Bacteria - 0; Metazoa - 113; Fungi - 19; Plants - 59; Viruses - 0; Other Eukaryotes - 8 (source: NCBI BLink). | chr5:9445950-9450584 FORWARDhighly similar to ( 870) loc_os03g19520 12003.m35181 protein expressed protein   no original description 	T
34.7	transport.phosphate	MDP0000173837	 highly similar to ( 791) AT4G00370 | Symbols: ANTR2, PHT4;4 | ANTR2; inorganic phosphate transmembrane transporter/ organic anion transmembrane transporter | chr4:163153-166111 REVERSEhighly similar to ( 751) loc_os09g39680 12009.m06906 protein sialin, putative, expressed  no original description 	T
35.2	not assigned.unknown	MDP0000321949	 weakly similar to ( 126) AT1G69980 | Symbols:  | unknown protein | chr1:26356559-26357647 REVERSEweakly similar to ( 114) loc_os09g38750 12009.m06816 protein expressed protein  no original description 	T
35.2	not assigned.unknown	MDP0000321031	 moderately similar to ( 282) AT3G59670 | Symbols:  | unknown protein | chr3:22040485-22042380 FORWARDweakly similar to ( 118) loc_os05g30340 12005.m27796 protein expressed protein no original description 	T
35.2	not assigned.unknown	MDP0000254470	 moderately similar to ( 309) AT3G49720 | Symbols:  | unknown protein | chr3:18440192-18441655 REVERSEmoderately similar to ( 269) loc_os01g05070 12001.m42860 protein expressed protein  no original description 	T
27.1	RNA.processing	MDP0000236223	 highly similar to ( 513) AT5G53180 | Symbols: ATPTB2, PTB2 | PTB2 (POLYPYRIMIDINE TRACT-BINDING PROTEIN 2); RNA binding / nucleotide binding | chr5:21568393-21571081 REVERSEmoderately similar to ( 450) loc_os01g43170 12001.m43096 protein polypyrimidine tract-binding protein homolog 2, putative, expressed  no original description 	T
35.2	not assigned.unknown	MDP0000279862	 moderately similar to ( 268) AT1G72270 | Symbols:  | unknown protein | chr1:27199733-27211122 REVERSEmoderately similar to ( 267) loc_os04g55130 12004.m10405 protein expressed protein  no original description 	T
1.1.5.2	PS.lightreaction.other electron carrier (ox/red).ferredoxin	MDP0000238096	 weakly similar to ( 144) AT1G02180 | Symbols:  | ferredoxin-related | chr1:413619-414505 REVERSEmoderately similar to ( 219) loc_os08g35710 12008.m07560 protein expressed protein  no original description 	T
20.2.1	stress.abiotic.heat	MDP0000507230	 nearly identical (1119) AT4G16660 | Symbols:  | heat shock protein 70, putative / HSP70, putative | chr4:9377225-9381232 FORWARDmoderately similar to ( 223) BIP5_TOBAC Luminal-binding protein 5 precursor (BiP 5) (78 kDa glucose-regulated protein homolog 5) (GRP 78-5) - Nicotiana tabacum (Common tobacco)nearly identical (1032) loc_os02g48110 12002.m09845 protein oxygen-regulated protein precursor, putative, expressed  no original description 	T
29.5.9	protein.degradation.AAA type	MDP0000277945	 highly similar to ( 566) AT2G34560 | Symbols:  | katanin, putative | chr2:14560266-14562695 FORWARDweakly similar to ( 181) CDC48_CAPAN Cell division cycle protein 48 homolog - Capsicum annuum (Bell pepper)highly similar to ( 587) loc_os01g55260 12001.m11685 protein katanin p60 ATPase-containing subunit A-like 1, putative, expressed  no original description 	T
10.8.1	cell wall.pectin*esterases.PME	MDP0000162904	 highly similar to ( 694) AT3G14310 | Symbols: ATPME3 | ATPME3; pectinesterase | chr3:4772214-4775095 REVERSEhighly similar to ( 769) PME3_CITSI Pectinesterase-3 precursor (EC 3.1.1.11) (Pectin methylesterase 3) (PE 3) - Citrus sinensis (Sweet orange)moderately similar to ( 387) loc_os07g47830 12007.m08998 protein pectinesterase-1 precursor, putative, expressed  no original description 	T
35.2	not assigned.unknown	MDP0000422411	 weakly similar to ( 135) AT3G25400 | Symbols:  | FUNCTIONS IN: molecular_function unknown; INVOLVED IN: biological_process unknown; LOCATED IN: cellular_component unknown; EXPRESSED IN: 11 plant structures; EXPRESSED DURING: 6 growth stages; CONTAINS InterPro DOMAIN/s: NTP Pyrophosphohydrolase MazG-related, RS21-C6 (InterPro:IPR011394), EAR (InterPro:IPR009039), NTP pyrophosphohydrolase MazG, putative catalytic core (InterPro:IPR004518); Has 572 Blast hits to 572 proteins in 181 species: Archae - 11; Bacteria - 286; Metazoa - 71; Fungi - 1; Plants - 31; Viruses - 0; Other Eukaryotes - 172 (source: NCBI BLink). | chr3:9213236-9214144 FORWARDweakly similar to ( 133) loc_os04g43550 12004.m09315 protein mazG nucleotide pyrophosphohydrolase domain protein, putative, expressed no original description 	T
26.4	misc.beta 1,3 glucan hydrolases	MDP0000131669	 weakly similar to ( 138) AT1G78520 | Symbols:  | FUNCTIONS IN: molecular_function unknown; INVOLVED IN: biological_process unknown; LOCATED IN: endomembrane system; CONTAINS InterPro DOMAIN/s: X8 (InterPro:IPR012946); BEST Arabidopsis thaliana protein match is: glycosyl hydrolase family protein 17 (TAIR:AT2G43670.1); Has 747 Blast hits to 711 proteins in 30 species: Archae - 0; Bacteria - 0; Metazoa - 0; Fungi - 0; Plants - 747; Viruses - 0; Other Eukaryotes - 0 (source: NCBI BLink). | chr1:29537976-29538656 REVERSEvery weakly similar to (90.9) E13B_WHEAT Glucan endo-1,3-beta-glucosidase precursor (EC 3.2.1.39) ((1->3)-beta-glucan endohydrolase) ((1->3)-beta-glucanase) (Beta-1,3-endoglucanase) - Triticum aestivum (Wheat)weakly similar to ( 124) loc_os01g14140 12001.m150537 protein glucan endo-1,3-beta-glucosidase 4 precursor, putative, expressed no original description 	T
27.3.99	RNA.regulation of transcription.unclassified	MDP0000259773	 moderately similar to ( 245) AT3G51950 | Symbols:  | zinc finger (CCCH-type) family protein / RNA recognition motif (RRM)-containing protein | chr3:19278244-19280407 REVERSEmoderately similar to ( 278) loc_os07g48410 12007.m29333 protein nucleic acid binding protein, putative, expressed   no original description 	T
27.3.9	RNA.regulation of transcription.C2C2(Zn) GATA transcription factor family	MDP0000166889	 weakly similar to ( 129) AT3G54810 | Symbols: BME3-ZF, BME3 | zinc finger (GATA type) family protein | chr3:20296957-20298236 FORWARDweakly similar to ( 132) loc_os02g56250 12002.m10648 protein GATA zinc finger family protein, expressed no original description 	T
29.2.1.2.1.515	protein.synthesis.ribosomal protein.eukaryotic.40S subunit.S15A	MDP0000140235	 moderately similar to ( 251) AT5G59850 | Symbols:  | 40S ribosomal protein S15A (RPS15aF) | chr5:24112499-24113084 REVERSEmoderately similar to ( 248) RS15A_BRANA 40S ribosomal protein S15a (PPCB8) - Brassica napus (Rape)moderately similar to ( 247) loc_os02g27760 12002.m07920 protein 40S ribosomal protein S15a, putative, expressed  no original description 	T
29.5.11.4.2	protein.degradation.ubiquitin.E3.RING	MDP0000261942	 highly similar to ( 669) AT5G42340 | Symbols:  | binding / ubiquitin-protein ligase | chr5:16928086-16930367 REVERSEmoderately similar to ( 353) SPL11_ORYSA Spotted leaf protein 11 (Spotted leaf11) (Cell death-related protein SPL11) - Oryza sativa (Rice)highly similar to ( 560) loc_os08g37570 12008.m07743 protein spotted leaf protein 11, putative, expressed   MSP1_C  no original description 	T
27.3.25	RNA.regulation of transcription.MYB domain transcription factor family	MDP0000216918	 very weakly similar to (67.4) AT2G46410 | Symbols: CPC | CPC (CAPRICE); DNA binding / transcription factor | chr2:19049315-19050185 REVERSEvery weakly similar to (56.6) MYBC_MAIZE Anthocyanin regulatory C1 protein - Zea mays (Maize)very weakly similar to (67.4) loc_os01g43180 12001.m10581 protein myb-like protein isolog, putative no original description 	T
27.3.22	RNA.regulation of transcription.HB,Homeobox transcription factor family	MDP0000005879	 nearly identical (1321) AT4G32880 | Symbols: ATHB-8, ATHB8 | ATHB-8 (HOMEOBOX GENE 8); DNA binding / transcription factor | chr4:15863587-15867822 REVERSEnearly identical (1150) loc_os03g43930 12003.m09434 protein class III HD-Zip protein 4, putative, expressed   no original description 	T
33.3	development.squamosa promoter binding like (SPL)	MDP0000919693	 highly similar to ( 613) AT3G60030 | Symbols: SPL12 | SPL12 (squamosa promoter-binding protein-like 12); transcription factor | chr3:22165856-22169410 REVERSEvery weakly similar to (96.3) SBP1_ANTMA Squamosa promoter-binding protein 1 - Antirrhinum majus (Garden snapdragon)highly similar to ( 526) loc_os03g61760 12003.m11057 protein squamosa promoter-binding-like protein 12, putative, expressed   no original description 	T
16.10	secondary metabolism.simple phenols	MDP0000127409	 moderately similar to ( 452) AT2G29130 | Symbols: LAC2, ATLAC2 | LAC2 (laccase 2); laccase | chr2:12525189-12527699 REVERSEvery weakly similar to (96.7) ASO_TOBAC L-ascorbate oxidase precursor (EC 1.10.3.3) (Ascorbase) (ASO) - Nicotiana tabacum (Common tobacco)moderately similar to ( 426) loc_os01g62490 12001.m12371 protein L-ascorbate oxidase precursor, putative, expressed  no original description 	T
35.2	not assigned.unknown	MDP0000909034	 weakly similar to ( 190) AT1G26750 | Symbols:  | unknown protein | chr1:9246857-9248053 FORWARDmoderately similar to ( 249) loc_os12g10650 12012.m05048 protein expressed protein no original description 	T
1.2.5	PS.photorespiration.serine hydroxymethyltransferase	MDP0000332597	 highly similar to ( 857) AT4G37930 | Symbols: SHM1, STM, SHMT1 | SHM1 (SERINE TRANSHYDROXYMETHYLTRANSFERASE 1); glycine hydroxymethyltransferase/ poly(U) binding | chr4:17831891-17834742 REVERSEhighly similar to ( 887) GLYM_SOLTU Serine hydroxymethyltransferase, mitochondrial precursor (EC 2.1.2.1) (Serine methylase) (Glycine hydroxymethyltransferase) (SHMT) - Solanum tuberosum (Potato)highly similar to ( 851) loc_os03g52840 12003.m101129 protein serine hydroxymethyltransferase, mitochondrial precursor, putative, expressed   no original description 	T
35.2	not assigned.unknown	MDP0000156578	 weakly similar to ( 130) AT1G10657 | Symbols:  | unknown protein | chr1:3530652-3531572 FORWARDvery weakly similar to (82.0) loc_os05g38680 12005.m08047 protein plant-specific domain TIGR01589 family protein, expressed no original description 	T
29.2.1.1.1.2.1	protein.synthesis.ribosomal protein.prokaryotic.chloroplast.50S subunit.L1	MDP0000650449	 moderately similar to ( 388) AT3G63490 | Symbols:  | ribosomal protein L1 family protein | chr3:23444269-23445777 FORWARDmoderately similar to ( 378) RK1_SPIOL 50S ribosomal protein L1, chloroplast precursor (CL1) - Spinacia oleracea (Spinach)moderately similar to ( 378) loc_os05g32220 12005.m07455 protein 50S ribosomal protein L1, putative, expressed  no original description 	T
35.2	not assigned.unknown	MDP0000336915	  no original description 	T
5.10	fermentation.aldehyde dehydrogenase	MDP0000305716	 weakly similar to ( 110) AT3G24503 | Symbols: ALDH2C4, ALDH1A, REF1 | ALDH2C4; 3-chloroallyl aldehyde dehydrogenase/ aldehyde dehydrogenase (NAD)/ coniferyl-aldehyde dehydrogenase | chr3:8919732-8923029 REVERSEvery weakly similar to (91.3) RR6_SPIOL 30S ribosomal protein S6 alpha, chloroplast precursor [Contains: 30S ribosomal protein S6 beta; 30S ribosomal protein S6 gamma; 30S ribosomal protein S6 delta; 30S ribosomal protein S6 epsilon] (Fragment) - Spinacia oleracea (Spinach)weakly similar to ( 107) loc_os01g40870 12001.m10356 protein aldehyde dehydrogenase, mitochondrial precursor, putative, expressed  no original description 	T
13.2.6.3	amino acid metabolism.degradation.aromatic aa.tryptophan	MDP0000233842	 moderately similar to ( 403) AT5G43280 | Symbols: ATDCI1 | ATDCI1 (DELTA(3,5),DELTA(2,4)-DIENOYL-COA ISOMERASE 1); delta3,5-delta2,4-dienoyl-CoA isomerase/ enoyl-CoA hydratase | chr5:17367947-17368750 FORWARDvery weakly similar to (83.6) MFPA_CUCSA Glyoxysomal fatty acid beta-oxidation multifunctional protein MFP-a [Includes: Enoyl-CoA hydratase (EC 4.2.1.17); 3-2-trans-enoyl-CoA isomerase (EC 5.3.3.8); 3-hydroxybutyryl-CoA epimerase (EC 5.1.2.3); 3-hydroxyacyl-CoA dehydrogenase (moderately similar to ( 300) loc_os01g70090 12001.m13057 protein delta3,5-delta2,4-dienoyl-CoA isomerase, mitochondrial precursor, putative, expressed  no original description 	T
35.1	not assigned.no ontology	MDP0000321032	 moderately similar to ( 225) AT1G05720 | Symbols:  | selenoprotein family protein | chr1:1717677-1718849 REVERSEmoderately similar to ( 206) loc_os01g66960 12001.m12801 protein selenoprotein precursor, putative, expressed  no original description 	T
12.4	N-metabolism.misc	MDP0000770156	 highly similar to ( 594) AT5G67220 | Symbols:  | nitrogen regulation family protein | chr5:26820276-26821926 REVERSEhighly similar to ( 561) loc_os06g49870 12006.m09511 protein tRNA-dihydrouridine synthase B, putative, expressed  no original description 	T
27.3.10	RNA.regulation of transcription.C2C2(Zn) YABBY family	MDP0000298333	 moderately similar to ( 205) AT2G45190 | Symbols: AFO, FIL, YAB1 | AFO (ABNORMAL FLORAL ORGANS); protein binding / transcription factor/ transcription regulator | chr2:18628450-18630552 REVERSEweakly similar to ( 116) YAB1_ORYSA Protein YABBY (OsYAB1) (Filamentous flower protein 1) - Oryza sativa (Rice)weakly similar to ( 193) loc_os02g42950 12002.m33795 protein yabby15 protein, putative, expressed no original description 	T
26.3.2	misc.gluco-, galacto- and mannosidases.beta-galactosidase	MDP0000265046	 nearly identical (1238) AT2G28470 | Symbols: BGAL8 | BGAL8 (beta-galactosidase 8); beta-galactosidase/ catalytic/ cation binding / sugar binding | chr2:12169047-12173164 REVERSEhighly similar to ( 946) BGAL_ASPOF Beta-galactosidase precursor (EC 3.2.1.23) (Lactase) - Asparagus officinalis (Garden asparagus)nearly identical (1174) loc_os03g15020 12003.m34824 protein beta-galactosidase precursor, putative, expressed   no original description 	T
26.2	misc.UDP glucosyl and glucoronyl transferases	MDP0000172933	 moderately similar to ( 320) AT2G22590 | Symbols:  | transferase, transferring glycosyl groups | chr2:9593012-9594424 FORWARDmoderately similar to ( 209) UFOG_PETHY Anthocyanidin 3-O-glucosyltransferase (EC 2.4.1.115) (Flavonol 3-O-glucosyltransferase) (UDP-glucose flavonoid 3-O-glucosyltransferase) (Anthocyanin rhamnosyl transferase) - Petunia hybrida (Petunia)moderately similar to ( 313) loc_os01g64540 12001.m12568 protein anthocyanidin 3-O-glucosyltransferase, putative, expressed  no original description 	T
35.2	not assigned.unknown	MDP0000548529	 very weakly similar to (53.1) AT5G53880 | Symbols:  | unknown protein | chr5:21872995-21873195 REVERSE no original description 	T
29.2.1.2.1.4	protein.synthesis.ribosomal protein.eukaryotic.40S subunit.S4	MDP0000930575	 moderately similar to ( 449) AT5G58420 | Symbols:  | 40S ribosomal protein S4 (RPS4D) | chr5:23619599-23620896 FORWARDmoderately similar to ( 451) RS4_SOLTU 40S ribosomal protein S4 - Solanum tuberosum (Potato)moderately similar to ( 443) loc_os02g01560 12002.m05506 protein 40S ribosomal protein S4, putative, expressed no original description 	T
35.2	not assigned.unknown	MDP0000676683	 moderately similar to ( 221) AT1G08480 | Symbols:  | unknown protein | chr1:2684340-2685395 FORWARDweakly similar to ( 133) loc_os08g02080 12008.m26555 protein rickettsia 17 kDa surface antigen family protein, expressed  no original description 	T
16.8.3.1	secondary metabolism.flavonoids.dihydroflavonols.dihydroflavonol 4-reductase	MDP0000275397	 moderately similar to ( 247) AT5G58490 | Symbols:  | cinnamoyl-CoA reductase family | chr5:23643068-23644455 FORWARDweakly similar to ( 107) DFRA_DIACA Dihydroflavonol-4-reductase (EC 1.1.1.219) (DFR) (Dihydrokaempferol 4-reductase) - Dianthus caryophyllus (Carnation) (Clove pink)moderately similar to ( 229) loc_os03g60380 12003.m10928 protein dihydroflavonol-4-reductase, putative, expressed   no original description 	T
26.28	misc.GDSL-motif lipase	MDP0000199327	 moderately similar to ( 454) AT3G48460 | Symbols:  | GDSL-motif lipase/hydrolase family protein | chr3:17949496-17951082 FORWARDweakly similar to ( 129) EST_HEVBR Esterase precursor (EC 3.1.1.-) (Early nodule-specific protein homolog) (Latex allergen Hev b 13) - Hevea brasiliensis (Para rubber tree)moderately similar to ( 392) loc_os03g62740 12003.m11151 protein esterase precursor, putative, expressed  no original description 	T
34.15	transport.potassium	MDP0000144878	 highly similar to ( 689) AT5G51710 | Symbols: KEA5, ATKEA5 | KEA5; potassium ion transmembrane transporter/ potassium:hydrogen antiporter | chr5:21004566-21008580 REVERSEhighly similar to ( 524) loc_os06g36590 12006.m32099 protein inner membrane protein ybaL, putative, expressed  no original description 	T
26.10	misc.cytochrome P450	MDP0000276798	 highly similar to ( 862) AT2G45970 | Symbols: CYP86A8, LCR | CYP86A8; fatty acid (omega-1)-hydroxylase/ oxygen binding | chr2:18912548-18914161 REVERSEweakly similar to ( 113) F3PH_PETHY Flavonoid 3'-monooxygenase (EC 1.14.13.21) (Flavonoid 3'-hydroxylase) (Cytochrome P450 75B2) - Petunia hybrida (Petunia)highly similar to ( 733) loc_os04g47250 12004.m09671 protein cytochrome P450 86A2, putative, expressed   no original description 	T
35.2	not assigned.unknown	MDP0000162679	 weakly similar to ( 137) AT5G52900 | Symbols:  | unknown protein | chr5:21452893-21453692 REVERSE no original description 	T
8.3	TCA / org transformation.carbonic anhydrases	MDP0000156439	 moderately similar to ( 255) AT3G52720 | Symbols: ATACA1, ACA1 | ACA1 (ALPHA CARBONIC ANHYDRASE 1); carbonate dehydratase/ zinc ion binding | chr3:19539617-19541116 REVERSEvery weakly similar to (70.9) CAH2_CHLRE Carbonic anhydrase 2 precursor (EC 4.2.1.1) (Carbonate dehydratase 2) (CA2) [Contains: Carbonic anhydrase 2 large chain; Carbonic anhydrase 2 small chain] - Chlamydomonas reinhardtiimoderately similar to ( 259) loc_os02g33030 12002.m08396 protein carbonic anhydrase precursor, putative, expressed no original description 	T
35.2	not assigned.unknown	MDP0000333495	  no original description 	T
10.2	cell wall.cellulose synthesis	MDP0000270189	 nearly identical (1061) AT4G31590 | Symbols: ATCSLC05, CSLC05, ATCSLC5 | ATCSLC5 (CELLULOSE-SYNTHASE LIKE C5); cellulose synthase/ transferase, transferring glycosyl groups | chr4:15309889-15312336 REVERSEhighly similar to ( 892) loc_os09g25900 12009.m05742 protein CSLC2 - cellulose synthase-like family C, expressed   no original description 	T
17.6.2	hormone metabolism.gibberelin.signal transduction	MDP0000319522	 highly similar to ( 512) AT3G63010 | Symbols: ATGID1B, GID1B | GID1B (GA INSENSITIVE DWARF1B); hydrolase | chr3:23289717-23290998 FORWARDmoderately similar to ( 399) GID1_ORYSA Gibberellin receptor GID1 (EC 3.-.-.-) (Gibberellin-insensitive dwarf protein 1) (Protein GIBBERELLIN INSENSITIVE DWARF1) - Oryza sativa (Rice)moderately similar to ( 399) loc_os05g33730 12005.m07605 protein gibberellin receptor GID1L2, putative, expressed  no original description 	T
35.2	not assigned.unknown	MDP0000696314	 weakly similar to ( 124) AT1G54860 | Symbols:  | unknown protein | chr1:20457522-20458434 REVERSEweakly similar to ( 114) loc_os07g45060 12007.m08726 protein GPI-anchored protein At5g19240 precursor, putative, expressed  no original description 	T
30.2.11	signalling.receptor kinases.leucine rich repeat XI	MDP0000456401	 moderately similar to ( 495) AT1G47890 | Symbols: AtRLP7 | AtRLP7 (Receptor Like Protein 7); kinase/ protein binding | chr1:17643976-17647035 FORWARDmoderately similar to ( 208) RPK1_IPONI Receptor-like protein kinase precursor (EC 2.7.11.1) - Ipomoea nil (Japanese morning glory) (Pharbitis nil)highly similar to ( 541) loc_os12g12120 12012.m05186 protein verticillium wilt disease resistance protein precursor, putative, expressed   no original description 	T
26.2	misc.UDP glucosyl and glucoronyl transferases	MDP0000185643	 highly similar to ( 587) AT1G70090 | Symbols: GATL9, LGT8 | LGT8 (GLUCOSYL TRANSFERASE FAMILY 8); polygalacturonate 4-alpha-galacturonosyltransferase/ transferase, transferring glycosyl groups / transferase, transferring hexosyl groups | chr1:26400927-26402099 FORWARDmoderately similar to ( 446) loc_os06g13760 12006.m06091 protein transferase, transferring glycosyl groups, putative, expressed  no original description 	T
35.2	not assigned.unknown	MDP0000259323	 highly similar to ( 517) AT5G13100 | Symbols:  | unknown protein | chr5:4156789-4158235 FORWARDmoderately similar to ( 420) loc_os01g01295 12001.m42534 protein expressed protein  no original description 	T
29.2.3	protein.synthesis.initiation	MDP0000139147	 moderately similar to ( 454) AT2G34970 | Symbols:  | eIF4-gamma/eIF5/eIF2-epsilon domain-containing protein | chr2:14746340-14748532 FORWARDmoderately similar to ( 382) loc_os02g56740 12002.m33930 protein translation initiation factor eIF-2B epsilon subunit, putative, expressed  no original description 	T
26.12	misc.peroxidases	MDP0000303101	 moderately similar to ( 478) AT2G37130 | Symbols:  | peroxidase 21 (PER21) (P21) (PRXR5) | chr2:15598225-15599734 REVERSEweakly similar to ( 196) PER2_ORYSA Peroxidase 2 precursor (EC 1.11.1.7) - Oryza sativa (Rice)moderately similar to ( 414) loc_os07g49360 12007.m09145 protein peroxidase 21 precursor, putative, expressed  no original description 	T
26.18	misc.invertase/pectin methylesterase inhibitor family protein	MDP0000264060	 weakly similar to ( 165) AT5G20740 | Symbols:  | invertase/pectin methylesterase inhibitor family protein | chr5:7025867-7026484 REVERSEweakly similar to ( 116) 21KD_DAUCA 21 kDa protein precursor (1.2 protein) - Daucus carota (Carrot)weakly similar to ( 108) loc_os06g49760 12006.m09500 protein ripening-related protein, putative, expressed   no original description 	T
10.1.10	cell wall.precursor synthesis.UDP-glucose 4,6-dehydratase	MDP0000193908	 highly similar to ( 511) AT1G63000 | Symbols: NRS/ER, UER1 | NRS/ER (NUCLEOTIDE-RHAMNOSE SYNTHASE/EPIMERASE-REDUCTASE); UDP-4-keto-6-deoxy-glucose-3,5-epimerase/ UDP-4-keto-rhamnose-4-keto-reductase/ dTDP-4-dehydrorhamnose 3,5-epimerase/ dTDP-4-dehydrorhamnose reductase | chr1:23342510-23343859 FORWARDhighly similar to ( 501) loc_os02g45540 12002.m09591 protein dTDP-4-dehydrorhamnose reductase, putative, expressed   no original description 	T
30.2.11	signalling.receptor kinases.leucine rich repeat XI	MDP0000255961	 highly similar to ( 521) AT1G34420 | Symbols:  | leucine-rich repeat family protein / protein kinase family protein | chr1:12584587-12587570 FORWARDmoderately similar to ( 238) RPK1_IPONI Receptor-like protein kinase precursor (EC 2.7.11.1) - Ipomoea nil (Japanese morning glory) (Pharbitis nil)moderately similar to ( 419) loc_os12g08180 12012.m04805 protein receptor-like protein kinase precursor, putative, expressed   no original description 	T
29.2.1.2.2.10	protein.synthesis.ribosomal protein.eukaryotic.60S subunit.L10	MDP0000422870	 moderately similar to ( 403) AT1G26910 | Symbols:  | 60S ribosomal protein L10 (RPL10B) | chr1:9321709-9322813 FORWARDmoderately similar to ( 410) RL10_SOLME 60S ribosomal protein L10 (EQM) - Solanum melongena (Eggplant) (Aubergine)moderately similar to ( 396) loc_os11g11390 12011.m05332 protein 60S ribosomal protein L10-1, putative, expressed  no original description 	T
26.13	misc.acid and other phosphatases	MDP0000283500	 moderately similar to ( 368) AT5G34850 | Symbols: ATPAP26, PAP26 | PAP26 (PURPLE ACID PHOSPHATASE 26); acid phosphatase/ protein serine/threonine phosphatase | chr5:13108475-13111217 REVERSEmoderately similar to ( 265) PPAF_SOYBN Purple acid phosphatase precursor (EC 3.1.3.2) (Manganese(II) purple acid phosphatase) - Glycine max (Soybean)moderately similar to ( 353) loc_os06g43640 12006.m31909 protein purple acid phosphatase precursor, putative, expressed  no original description 	T
35.2	not assigned.unknown	MDP0000432874	 very weakly similar to (75.9) loc_os01g34790 12001.m09783 protein expressed protein  no original description 	T
20.1	stress.biotic	MDP0000553692	 weakly similar to ( 107) AT3G14470 | Symbols:  | disease resistance protein (NBS-LRR class), putative | chr3:4857940-4861104 FORWARDvery weakly similar to (98.2) loc_os11g42090 12011.m08045 protein Leucine Rich Repeat family protein, expressed no original description 	T
29.2.3	protein.synthesis.initiation	MDP0000270113	 highly similar to ( 692) AT4G11420 | Symbols: EIF3A, ATEIF3A-1, EIF3A-1, ATTIF3A1, TIF3A1 | EIF3A (EUKARYOTIC TRANSLATION INITIATION FACTOR 3A); translation initiation factor | chr4:6947834-6952053 REVERSEhighly similar to ( 646) IF3A_TOBAC Eukaryotic translation initiation factor 3 subunit 10 (eIF-3 theta) (Eukaryotic translation initiation factor 3 large subunit) (eIF3a) (PNLA-35) - Nicotiana tabacum (Common tobacco)highly similar to ( 576) loc_os01g03070 12001.m42594 protein eukaryotic translation initiation factor 3 subunit 10, putative, expressed   no original description 	T
35.2	not assigned.unknown	MDP0000315511	 moderately similar to ( 247) AT4G31130 | Symbols:  | unknown protein | chr4:15137159-15138465 REVERSEmoderately similar to ( 206) loc_os05g02990 12005.m64177 protein expressed protein no original description 	T
34.6	transport.sulphate	MDP0000145668	 highly similar to ( 905) AT1G23090 | Symbols: AST91, SULTR3;3 | AST91 (SULFATE TRANSPORTER 91); sulfate transmembrane transporter | chr1:8185238-8188954 REVERSEmoderately similar to ( 357) NO70_SOYBN Early nodulin 70 - Glycine max (Soybean)highly similar to ( 878) loc_os04g55800 12004.m10471 protein sulfate transporter 3.3, putative, expressed   no original description 	T
31.1	cell.organisation	MDP0000652692	 highly similar to ( 673) AT5G09810 | Symbols: ACT2, ACT7 | ACT7 (ACTIN 7); structural constituent of cytoskeleton | chr5:3052809-3054220 FORWARDhighly similar to ( 677) ACT_GOSHI Actin - Gossypium hirsutum (Upland cotton)highly similar to ( 676) loc_os03g50890 12003.m10074 protein actin-1, putative, expressed no original description 	T
11.8.1	lipid metabolism.'exotics' (steroids, squalene etc).sphingolipids	MDP0000688638	 highly similar to ( 578) AT4G04930 | Symbols: DES-1-LIKE | DES-1-LIKE; oxidoreductase/ sphingolipid delta-4 desaturase | chr4:2509236-2510918 FORWARDhighly similar to ( 563) loc_os02g42660 12002.m09301 protein dihydroceramide delta, putative, expressed no original description 	T
16.8.1.21	secondary metabolism.flavonoids.anthocyanins.anthocyanin 5-aromatic acyltransferase	MDP0000496646	 moderately similar to ( 315) AT5G39090 | Symbols:  | transferase family protein | chr5:15643612-15644958 FORWARDvery weakly similar to (87.4) HCBT2_DIACA Anthranilate N-benzoyltransferase protein 2 (EC 2.3.1.144) (Anthranilate N-hydroxycinnamoyl/benzoyltransferase 2) - Dianthus caryophyllus (Carnation) (Clove pink)moderately similar to ( 241) loc_os02g57480 12002.m10770 protein anthocyanin 5-aromatic acyltransferase, putative, expressed   no original description 	T
29.2.4	protein.synthesis.elongation	MDP0000482030	 nearly identical (1566) AT1G56070 | Symbols: LOS1 | LOS1; copper ion binding / translation elongation factor/ translation factor, nucleic acid binding | chr1:20968245-20971077 REVERSEnearly identical (1543) EF2_BETVU Elongation factor 2 (EF-2) - Beta vulgaris (Sugar beet)nearly identical (1550) loc_os02g32030 12002.m08296 protein elongation factor 2, putative, expressed   no original description 	T
26.10	misc.cytochrome P450	MDP0000151762	 highly similar to ( 622) AT2G46660 | Symbols: CYP78A6 | CYP78A6; electron carrier/ heme binding / iron ion binding / monooxygenase/ oxygen binding | chr2:19153602-19155417 REVERSEhighly similar to ( 609) C78A3_SOYBN Cytochrome P450 78A3 (EC 1.14.-.-) - Glycine max (Soybean)highly similar to ( 546) loc_os08g43390 12008.m08313 protein cytochrome P450 78A3, putative, expressed  no original description 	T
27.3.6	RNA.regulation of transcription.bHLH,Basic Helix-Loop-Helix family	MDP0000301871	 moderately similar to ( 252) AT1G74530 | Symbols:  | unknown protein | chr1:28009723-28012044 REVERSEmoderately similar to ( 218) loc_os08g07430 12008.m04881 protein expressed protein  no original description 	T
35.2	not assigned.unknown	MDP0000838506	 highly similar to ( 630) AT1G09340 | Symbols: CRB, CSP41B | CRB (CHLOROPLAST RNA BINDING); binding / catalytic/ coenzyme binding | chr1:3015473-3018035 FORWARDhighly similar to ( 600) loc_os12g23180 12012.m26701 protein RNA binding protein, putative, expressed no original description 	T
28.2	DNA.repair	MDP0000827219	 moderately similar to ( 353) AT3G12710 | Symbols:  | methyladenine glycosylase family protein | chr3:4040572-4041828 REVERSEmoderately similar to ( 316) loc_os01g58550 12001.m150749 protein DNA-3-methyladenine glycosylase I, putative, expressed  no original description 	T
31.3.1	cell.cycle.peptidylprolyl isomerase	MDP0000779747	 moderately similar to ( 250) AT3G62030 | Symbols: ROC4 | peptidyl-prolyl cis-trans isomerase, chloroplast / cyclophilin / rotamase / cyclosporin A-binding protein (ROC4) | chr3:22973004-22975139 FORWARDmoderately similar to ( 246) CYPB_VICFA Peptidyl-prolyl cis-trans isomerase, chloroplast precursor (EC 5.2.1.8) (PPIase) (Rotamase) (Cyclophilin) (Cyclosporin A-binding protein) (CYP B) - Vicia faba (Broad bean)moderately similar to ( 271) loc_os01g18210 12001.m08363 protein peptidyl-prolyl cis-trans isomerase, chloroplast precursor, putative, expressed no original description 	T
31.1	cell.organisation	MDP0000774288	 highly similar to ( 749) AT5G09810 | Symbols: ACT2, ACT7 | ACT7 (ACTIN 7); structural constituent of cytoskeleton | chr5:3052809-3054220 FORWARDhighly similar to ( 741) ACT13_SOLTU Actin-101 - Solanum tuberosum (Potato)highly similar to ( 743) loc_os11g06390 12011.m28655 protein actin-7, putative, expressed  no original description 	T
35.2	not assigned.unknown	MDP0000143636	 highly similar to ( 681) AT1G55360 | Symbols:  | unknown protein | chr1:20666526-20668502 REVERSEhighly similar to ( 657) loc_os07g10530 12007.m05508 protein carboxyl-terminal peptidase, putative, expressed  no original description 	T
20.1.3	stress.biotic.signalling	MDP0000206773	 moderately similar to ( 387) AT3G48090 | Symbols: EDS1 | EDS1 (enhanced disease susceptibility 1); lipase/ signal transducer/ triacylglycerol lipase | chr3:17755553-17757292 REVERSEmoderately similar to ( 423) loc_os09g22450 12009.m05451 protein EDS1-like protein, putative, expressed  no original description 	T
1.1.4.9	PS.lightreaction.ATP synthase.subunit B_ (ATPX)	MDP0000291815	 weakly similar to ( 186) AT4G32260 | Symbols:  | ATP synthase family | chr4:15573859-15574586 REVERSEweakly similar to ( 184) ATPX_SPIOL ATP synthase B' chain, chloroplast precursor (EC 3.6.3.14) (Subunit II) - Spinacia oleracea (Spinach)weakly similar to ( 175) loc_os03g17070 12003.m07139 protein ATP synthase B chain, chloroplast precursor, putative, expressed    no original description 	T
10.8.1	cell wall.pectin*esterases.PME	MDP0000288901	 highly similar to ( 536) AT3G59010 | Symbols:  | pectinesterase family protein | chr3:21803015-21805098 REVERSEmoderately similar to ( 434) PME2_CITSI Pectinesterase-2 precursor (EC 3.1.1.11) (Pectin methylesterase) (PE) - Citrus sinensis (Sweet orange)moderately similar to ( 420) loc_os01g21034 12001.m42984 protein pectinesterase-2 precursor, putative, expressed  MSP1_C  no original description 	T
31.1	cell.organisation	MDP0000213737	 moderately similar to ( 436) AT2G17390 | Symbols: AKR2B | AKR2B (ANKYRIN REPEAT-CONTAINING 2B); protein binding / transcription regulator | chr2:7555870-7557743 FORWARDvery weakly similar to (73.6) SR43C_ORYSA Probable signal recognition particle 43 kDa protein, chloroplast precursor - Oryza sativa (Rice)moderately similar to ( 421) loc_os09g33810 12009.m060082 protein ankyrin repeat domain-containing protein 2, putative, expressed  no original description 	T
24.1	Biodegradation of Xenobiotics.hydroxyacylglutathione hydrolase	MDP0000234294	 moderately similar to ( 372) AT1G53580 | Symbols: GLX2-3, ETHE1, GLY3 | GLY3 (GLYOXALASE II 3); hydrolase/ hydroxyacylglutathione hydrolase | chr1:19991542-19993250 REVERSEmoderately similar to ( 356) loc_os01g47690 12001.m43132 protein hydroxyacylglutathione hydrolase 3, mitochondrial precursor, putative, expressed  no original description 	T
35.2	not assigned.unknown	MDP0000159869	 weakly similar to ( 187) AT5G62440 | Symbols:  | Encodes a protein DOMINO1 that belongs to a plant-specific gene family sharing a common motif present in the tomato DEFECTIVE CHLOROPLASTS AND LEAVES (LeDCL) protein.  DOMINO1 is located in the nucleus.  Arabidopsis embryos carrying the domino1 mutation grow slowly in comparison with wild type embryos and reach only the globular stage at desiccation. The primary defect of the mutation at the cellular level is the large size of the nucleolus that can be observed soon after fertilization in the nuclei of both the embryo and the endosperm.  DOMINO1 might have a role in ribosome biogenesis and in determining the rate of cell division. | chr5:25072620-25073917 REVERSEweakly similar to ( 155) loc_os03g08810 12003.m35052 protein expressed protein   no original description 	T
30.2.11	signalling.receptor kinases.leucine rich repeat XI	MDP0000320471	 highly similar to ( 891) AT5G06940 | Symbols:  | leucine-rich repeat family protein | chr5:2148078-2150771 REVERSEmoderately similar to ( 314) RPK1_IPONI Receptor-like protein kinase precursor (EC 2.7.11.1) - Ipomoea nil (Japanese morning glory) (Pharbitis nil)highly similar to ( 669) loc_os04g39650 12004.m08952 protein receptor protein kinase CLAVATA1 precursor, putative, expressed   no original description 	T
10.1.4	cell wall.precursor synthesis.UGD	MDP0000132475	 highly similar to ( 876) AT3G29360 | Symbols:  | UDP-glucose 6-dehydrogenase, putative | chr3:11267375-11268817 REVERSEhighly similar to ( 885) UGDH_SOYBN UDP-glucose 6-dehydrogenase (EC 1.1.1.22) (UDP-Glc dehydrogenase) (UDP-GlcDH) (UDPGDH) - Glycine max (Soybean)highly similar to ( 883) loc_os12g25700 12012.m26903 protein UDP-glucose 6-dehydrogenase, putative, expressed  no original description 	T
17.2.3	hormone metabolism.auxin.induced-regulated-responsive-activated	MDP0000253721	 highly similar to ( 721) AT5G15740 | Symbols:  | unknown protein | chr5:5134788-5136956 REVERSEhighly similar to ( 752) loc_os06g17390 12006.m06451 protein auxin-independent growth promoter, putative, expressed  no original description 	T
20.1.7	stress.biotic.PR-proteins	MDP0000293188	 moderately similar to ( 487) AT5G17680 | Symbols:  | disease resistance protein (TIR-NBS-LRR class), putative | chr5:5822999-5827153 FORWARDweakly similar to ( 123) loc_os01g15580 12001.m08156 protein disease resistance protein RGA2, putative, expressed   no original description 	T
35.1	not assigned.no ontology	MDP0000088659	 highly similar to ( 682) AT5G34940 | Symbols: AtGUS3 | AtGUS3 (Arabidopsis thaliana glucuronidase 3); beta-glucuronidase | chr5:13236566-13238718 REVERSEhighly similar to ( 637) loc_os02g55870 12002.m10611 protein heparanase-like protein 3 precursor, putative, expressed   no original description 	T
35.2	not assigned.unknown	MDP0000269988	 very weakly similar to (56.6) AT1G68660 | Symbols:  | FUNCTIONS IN: molecular_function unknown; INVOLVED IN: protein catabolic process; EXPRESSED IN: 23 plant structures; EXPRESSED DURING: 14 growth stages; CONTAINS InterPro DOMAIN/s: Adaptor protein ClpS, core (InterPro:IPR003769), Ribosomal protein L7/L12, C-terminal/adaptor protein ClpS-like (InterPro:IPR014719); Has 79 Blast hits to 79 proteins in 34 species: Archae - 0; Bacteria - 46; Metazoa - 0; Fungi - 0; Plants - 30; Viruses - 0; Other Eukaryotes - 3 (source: NCBI BLink). | chr1:25778058-25779083 REVERSEweakly similar to ( 166) loc_os01g61700 12001.m12295 protein ATP-dependent Clp protease adaptor protein ClpS containing protein no original description 	T
30.2.17	signalling.receptor kinases.DUF 26	MDP0000167747	 highly similar to ( 677) AT1G11340 | Symbols:  | S-locus lectin protein kinase family protein | chr1:3814116-3817420 REVERSEmoderately similar to ( 236) SLSG3_BRAOL S-locus-specific glycoprotein S13 precursor (SLSG-13) (Fragment) - Brassica oleracea (Wild cabbage)highly similar to ( 546) loc_os04g53994 12004.m10292 protein ATP binding protein, putative, expressed  no original description 	T
21.3	redox.heme	MDP0000145420	 moderately similar to ( 488) AT5G20140 | Symbols:  | SOUL heme-binding family protein | chr5:6799047-6800892 REVERSEmoderately similar to ( 433) loc_os06g39690 12006.m32107 protein SOUL heme-binding protein, expressed  no original description 	T
35.2	not assigned.unknown	MDP0000179768	 moderately similar to ( 208) AT1G29040 | Symbols:  | unknown protein | chr1:10134120-10135180 FORWARDmoderately similar to ( 203) loc_os01g42770 12001.m10541 protein type IIA topoisomerase, B subunit, putative, expressed  no original description 	T
26.18	misc.invertase/pectin methylesterase inhibitor family protein	MDP0000854242	 weakly similar to ( 193) AT5G62360 | Symbols:  | invertase/pectin methylesterase inhibitor family protein | chr5:25040699-25041310 FORWARDweakly similar to ( 152) 21KD_DAUCA 21 kDa protein precursor (1.2 protein) - Daucus carota (Carrot)weakly similar to ( 104) loc_os06g49760 12006.m09500 protein ripening-related protein, putative, expressed   no original description 	T
30.2.17	signalling.receptor kinases.DUF 26	MDP0000262753	 highly similar to ( 870) AT5G51560 | Symbols:  | leucine-rich repeat transmembrane protein kinase, putative | chr5:20945807-20948613 FORWARDweakly similar to ( 181) PSKR_DAUCA Phytosulfokine receptor precursor (EC 2.7.11.1) (Phytosulfokine LRR receptor kinase) - Daucus carota (Carrot)highly similar to ( 737) loc_os06g04370 12006.m05166 protein ATP binding protein, putative, expressed  no original description 	T
27.3.44	RNA.regulation of transcription.Chromatin Remodeling Factors	MDP0000178949	 highly similar to ( 717) AT5G14170 | Symbols: CHC1 | CHC1 | chr5:4568696-4570444 REVERSEhighly similar to ( 581) loc_os04g31320 12004.m08234 protein SWI/SNF-related matrix-associated actin-dependent regulator of chromatin subfamily D member 3, putative, expressed  no original description 	T
30.2.17	signalling.receptor kinases.DUF 26	MDP0000189901	 moderately similar to ( 404) AT5G60900 | Symbols: RLK1 | RLK1 (RECEPTOR-LIKE PROTEIN KINASE 1); ATP binding / carbohydrate binding / kinase/ protein kinase/ protein serine/threonine kinase/ protein tyrosine kinase/ sugar binding | chr5:24498467-24501494 REVERSEmoderately similar to ( 292) KPRO_MAIZE Putative receptor protein kinase ZmPK1 precursor (EC 2.7.11.1) - Zea mays (Maize)highly similar to ( 552) loc_os08g13870 12008.m05512 protein ATP binding protein, putative  no original description 	T
35.1	not assigned.no ontology	MDP0000940098	 weakly similar to ( 154) AT3G11910 | Symbols: UBP13 | UBP13 (UBIQUITIN-SPECIFIC PROTEASE 13); ubiquitin thiolesterase/ ubiquitin-specific protease | chr3:3761758-3770290 REVERSEweakly similar to ( 153) loc_os11g36470 12011.m07499 protein cysteine-type endopeptidase/ double-stranded DNA binding protein, putative, expressed TrbL  no original description 	T
29.5.11.4.3.2	protein.degradation.ubiquitin.E3.SCF.FBOX	MDP0000247849	 highly similar to ( 629) AT1G22040 | Symbols:  | kelch repeat-containing F-box family protein | chr1:7768370-7769797 FORWARDhighly similar to ( 541) loc_os02g36520 12002.m08739 protein kelch motif family protein, expressed   no original description 	T
29.2.1.1.1.1.6	protein.synthesis.ribosomal protein.prokaryotic.chloroplast.30S subunit.S6	MDP0000501172	 weakly similar to ( 135) AT1G64510 | Symbols:  | ribosomal protein S6 family protein | chr1:23954993-23956205 REVERSEweakly similar to ( 132) RR6_SPIOL 30S ribosomal protein S6 alpha, chloroplast precursor [Contains: 30S ribosomal protein S6 beta; 30S ribosomal protein S6 gamma; 30S ribosomal protein S6 delta; 30S ribosomal protein S6 epsilon] (Fragment) - Spinacia oleracea (Spinach)weakly similar to ( 115) loc_os03g62630 12003.m11140 protein structural constituent of ribosome, putative, expressed  no original description 	T
27.1.1	RNA.processing.splicing	MDP0000394917	 nearly identical (1815) AT3G55200 | Symbols:  | splicing factor, putative | chr3:20460533-20464361 FORWARDnearly identical (1038) loc_os02g04480 12002.m05797 protein splicing factor 3B subunit 3, putative, expressed  no original description 	T
31.1	cell.organisation	MDP0000176396	 weakly similar to ( 196) AT3G18670 | Symbols:  | ankyrin repeat family protein | chr3:6424135-6426471 REVERSEvery weakly similar to (52.8) loc_os05g45550 12005.m08680 protein retrotransposon protein, putative, unclassified  no original description 	T
31.4	cell.vesicle transport	MDP0000126756	 nearly identical (1188) AT1G50500 | Symbols: HIT1, ATVPS53, VPS53 | HIT1 (HEAT-INTOLERANT 1); transporter | chr1:18708217-18715597 REVERSEmoderately similar to ( 491) loc_os01g67880 12001.m12892 protein vacuolar protein sorting protein 53, putative, expressed  no original description 	T
26.6	misc.O-methyl transferases	MDP0000155204	 moderately similar to ( 445) AT5G42760 | Symbols:  | INVOLVED IN: biological_process unknown; LOCATED IN: cellular_component unknown; EXPRESSED IN: 21 plant structures; EXPRESSED DURING: 13 growth stages; CONTAINS InterPro DOMAIN/s: Conserved hypothetical protein CHP00027,  methylltransferase (InterPro:IPR011610), O-methyltransferase, N-terminal (InterPro:IPR003455); Has 888 Blast hits to 888 proteins in 139 species: Archae - 36; Bacteria - 741; Metazoa - 23; Fungi - 3; Plants - 27; Viruses - 0; Other Eukaryotes - 58 (source: NCBI BLink). | chr5:17148953-17150105 FORWARDmoderately similar to ( 375) loc_os03g56370 12003.m10546 protein O-methyltransferase, putative, expressed  no original description 	T
29.2.1.2.2.22	protein.synthesis.ribosomal protein.eukaryotic.60S subunit.L22	MDP0000152410	 weakly similar to ( 106) AT3G05560 | Symbols:  | 60S ribosomal protein L22-2 (RPL22B) | chr3:1614641-1615204 FORWARDvery weakly similar to (97.4) loc_os07g47710 12007.m08986 protein 60S ribosomal protein L22-2, putative, expressed  no original description 	T
35.2	not assigned.unknown	MDP0000018462	 moderately similar to ( 423) AT2G45990 | Symbols:  | unknown protein | chr2:18919402-18920690 FORWARDmoderately similar to ( 390) loc_os07g23520 12007.m29141 protein expressed protein no original description 	T
31.1	cell.organisation	MDP0000550088	 highly similar to ( 572) AT3G01750 | Symbols:  | ankyrin repeat family protein | chr3:270615-272691 FORWARDvery weakly similar to (53.9) AKT1_ORYSA Potassium channel AKT1 (OsAKT1) - Oryza sativa (Rice)highly similar to ( 648) loc_os05g23320 12005.m06627 protein expressed protein    no original description 	T
35.2	not assigned.unknown	MDP0000641449	 weakly similar to ( 142) AT1G73940 | Symbols:  | unknown protein | chr1:27798519-27799522 REVERSEweakly similar to ( 106) loc_os03g63550 12003.m11223 protein expressed protein no original description 	T
35.1	not assigned.no ontology	MDP0000262650	 nearly identical (1453) AT1G15750 | Symbols: WSIP1, TPL | TPL (TOPLESS); protein binding / protein homodimerization/ transcription repressor | chr1:5415086-5420359 REVERSEvery weakly similar to (60.1) PF20_CHLRE Flagellar WD repeat protein PF20 - Chlamydomonas reinhardtiinearly identical (1716) loc_os08g06480 12008.m04787 protein lissencephaly type-1-like homology motif, putative, expressed  no original description 	T
27.1	RNA.processing	MDP0000206547	 weakly similar to ( 191) AT3G62840 | Symbols:  | FUNCTIONS IN: molecular_function unknown; LOCATED IN: small nucleolar ribonucleoprotein complex, nucleus; EXPRESSED IN: 22 plant structures; EXPRESSED DURING: 13 growth stages; CONTAINS InterPro DOMAIN/s: Like-Sm ribonucleoprotein, core (InterPro:IPR001163), Like-Sm ribonucleoprotein, eukaryotic and archaea-type, core (InterPro:IPR006649), Like-Sm ribonucleoprotein-related, core (InterPro:IPR010920); BEST Arabidopsis thaliana protein match is: small nuclear ribonucleoprotein D2, putative / snRNP core protein D2, putative / Sm protein D2, putative (TAIR:AT2G47640.3); Has 537 Blast hits to 537 proteins in 165 species: Archae - 2; Bacteria - 0; Metazoa - 239; Fungi - 109; Plants - 78; Viruses - 0; Other Eukaryotes - 109 (source: NCBI BLink). | chr3:23235727-23236615 REVERSEweakly similar to ( 186) loc_os05g24970 12005.m083689 protein small nuclear ribonucleoprotein Sm D2, putative, expressed no original description 	T
13.1.5.3.1	amino acid metabolism.synthesis.serine-glycine-cysteine group.cysteine.OASTL	MDP0000250284	 moderately similar to ( 483) AT4G14880 | Symbols: OASA1, CYTACS1, ATCYS-3A | OASA1 (O-ACETYLSERINE (THIOL) LYASE (OAS-TL) ISOFORM A1); cysteine synthase | chr4:8518209-8520050 REVERSEhighly similar to ( 508) CYSK_SOLTU Cysteine synthase (EC 2.5.1.47) (O-acetylserine sulfhydrylase) (O-acetylserine (Thiol)-lyase) (CSase A) (CS-A) (OAS-TL A) - Solanum tuberosum (Potato)moderately similar to ( 485) loc_os03g53650 12003.m35628 protein cysteine synthase, putative, expressed no original description 	T
35.2	not assigned.unknown	MDP0000915810	 moderately similar to ( 388) AT5G10010 | Symbols:  | unknown protein | chr5:3128098-3131452 FORWARDmoderately similar to ( 350) loc_os04g56590 12004.m35061 protein ATP/GTP binding protein, putative, expressed  no original description 	T
27.3.46	RNA.regulation of transcription.DNA methyltransferases	MDP0000296459	 highly similar to ( 545) AT5G14620 | Symbols: DRM2, DMT7 | DRM2 (DOMAINS REARRANGED METHYLTRANSFERASE 2); N-methyltransferase | chr5:4715429-4718578 REVERSEhighly similar to ( 556) loc_os03g02010 12003.m34978 protein DNA cytosine methyltransferase Zmet3, putative, expressed  no original description 	T
30.5	signalling.G-proteins	MDP0000257331	 moderately similar to ( 306) AT3G07880 | Symbols:  | Rho GDP-dissociation inhibitor family protein | chr3:2514175-2515544 FORWARDmoderately similar to ( 288) loc_os02g48730 12002.m09907 protein rho GDP-dissociation inhibitor 1, putative, expressed   no original description 	T
34.13	transport.peptides and oligopeptides	MDP0000279462	 highly similar to ( 739) AT1G09930 | Symbols: ATOPT2 | ATOPT2; oligopeptide transporter | chr1:3227490-3230043 REVERSEhighly similar to ( 696) loc_os01g43940 12001.m10653 protein oligopeptide transporter 4, putative  no original description 	T
1.3.12	PS.calvin cycle.PRK	MDP0000322896	 highly similar to ( 648) AT1G32060 | Symbols: PRK | PRK (PHOSPHORIBULOKINASE); ATP binding / phosphoribulokinase/ protein binding | chr1:11532668-11534406 FORWARDhighly similar to ( 661) KPPR_MESCR Phosphoribulokinase, chloroplast precursor (EC 2.7.1.19) (Phosphopentokinase) (PRKase) (PRK) - Mesembryanthemum crystallinum (Common ice plant)highly similar to ( 655) loc_os02g47020 12002.m09738 protein phosphoribulokinase, chloroplast precursor, putative, expressed   no original description 	T
35.2	not assigned.unknown	MDP0000126654	 highly similar to ( 576) AT4G20050 | Symbols: QRT3 | QRT3 (QUARTET 3); polygalacturonase | chr4:10849911-10852090 REVERSEhighly similar to ( 518) loc_os04g52320 12004.m10126 protein QRT3, putative, expressed   no original description 	T
30.3	signalling.calcium	MDP0000146799	 weakly similar to ( 191) AT3G10190 | Symbols:  | calmodulin, putative | chr3:3155309-3155938 FORWARDweakly similar to ( 104) CALM_WHEAT Calmodulin (CaM) - Triticum aestivum (Wheat)weakly similar to ( 109) loc_os01g72100 12001.m13246 protein polcalcin Jun o 2, putative, expressed   no original description 	T
31.1	cell.organisation	MDP0000281415	 moderately similar to ( 335) AT4G31700 | Symbols: RPS6 | RPS6 (RIBOSOMAL PROTEIN S6); structural constituent of ribosome | chr4:15346306-15347075 REVERSEmoderately similar to ( 335) RS6_ASPOF 40S ribosomal protein S6 - Asparagus officinalis (Garden asparagus)moderately similar to ( 412) loc_os09g23160 12009.m059972 protein annexin A4, putative, expressed   no original description 	T
29.2.1.2.1.6	protein.synthesis.ribosomal protein.eukaryotic.40S subunit.S6	MDP0000281415	 moderately similar to ( 335) AT4G31700 | Symbols: RPS6 | RPS6 (RIBOSOMAL PROTEIN S6); structural constituent of ribosome | chr4:15346306-15347075 REVERSEmoderately similar to ( 335) RS6_ASPOF 40S ribosomal protein S6 - Asparagus officinalis (Garden asparagus)moderately similar to ( 412) loc_os09g23160 12009.m059972 protein annexin A4, putative, expressed   no original description 	T
29.5.11.4.2	protein.degradation.ubiquitin.E3.RING	MDP0000196401	 moderately similar to ( 207) AT2G01275 | Symbols:  | zinc finger (C3HC4-type RING finger) family protein | chr2:142610-143809 REVERSEmoderately similar to ( 207) loc_os01g03100 12001.m42845 protein protein binding protein, putative, expressed  no original description 	T
27.3.99	RNA.regulation of transcription.unclassified	MDP0000225862	 moderately similar to ( 338) AT1G21000 | Symbols:  | zinc-binding family protein | chr1:7338013-7339088 FORWARDmoderately similar to ( 259) loc_os06g41930 12006.m31902 protein expressed protein no original description 	T
29.6	protein.folding	MDP0000642692	 highly similar to ( 936) AT1G24510 | Symbols:  | T-complex protein 1 epsilon subunit, putative / TCP-1-epsilon, putative / chaperonin, putative | chr1:8685504-8687660 REVERSEhighly similar to ( 903) TCPE2_AVESA T-complex protein 1 subunit epsilon (TCP-1-epsilon) (CCT-epsilon) (TCP-K36) - Avena sativa (Oat)highly similar to ( 946) loc_os06g36700 12006.m08205 protein T-complex protein 1 subunit epsilon, putative, expressed  no original description 	T
27.3.22	RNA.regulation of transcription.HB,Homeobox transcription factor family	MDP0000564897	 moderately similar to ( 226) AT2G22430 | Symbols: ATHB6 | ATHB6; DNA binding / protein binding / sequence-specific DNA binding / transcription activator/ transcription factor | chr2:9526470-9527612 REVERSEweakly similar to ( 171) loc_os09g29460 12009.m06097 protein homeobox-leucine zipper protein ATHB-6, putative, expressed  no original description 	T
33.99	development.unspecified	MDP0000789873	 highly similar to ( 745) AT2G39210 | Symbols:  | nodulin family protein | chr2:16366287-16368231 REVERSEhighly similar to ( 633) loc_os03g58580 12003.m10763 protein nitrate and chloride transporter, putative, expressed   no original description 	T
26.12	misc.peroxidases	MDP0000451182	 moderately similar to ( 447) AT5G51890 | Symbols:  | peroxidase | chr5:21091163-21092335 REVERSEmoderately similar to ( 259) PER1_ORYSA Peroxidase 1 precursor (EC 1.11.1.7) - Oryza sativa (Rice)moderately similar to ( 293) loc_os02g58720 12002.m100428 protein peroxidase 64 precursor, putative, expressed  no original description 	T
29.2.1.2.2.12	protein.synthesis.ribosomal protein.eukaryotic.60S subunit.L12	MDP0000312991	 moderately similar to ( 290) AT5G60670 | Symbols:  | 60S ribosomal protein L12 (RPL12C) | chr5:24381066-24381566 REVERSEmoderately similar to ( 236) RL12_CHLRE 60S ribosomal protein L12 (Fragment) - Chlamydomonas reinhardtiimoderately similar to ( 301) loc_os04g50990 12004.m09996 protein 60S ribosomal protein L12, putative, expressed   no original description 	T
31.4	cell.vesicle transport	MDP0000290998	 nearly identical (2022) AT1G62020 | Symbols:  | coatomer protein complex, subunit alpha, putative | chr1:22919814-22923728 FORWARDhighly similar to ( 618) 2AAA_PEA Protein phosphatase PP2A regulatory subunit A (PR65) (Fragment) - Pisum sativum (Garden pea)nearly identical (1915) loc_os09g04110 12009.m22040 protein coatomer subunit alpha, putative, expressed    no original description 	T
29.5	protein.degradation	MDP0000272931	 weakly similar to ( 145) AT5G22860 | Symbols:  | serine carboxypeptidase S28 family protein | chr5:7639907-7642945 REVERSEweakly similar to ( 140) loc_os01g56150 12001.m11771 protein lysosomal Pro-X carboxypeptidase precursor, putative, expressed no original description 	T
29.5.3	protein.degradation.cysteine protease	MDP0000505556	 moderately similar to ( 374) AT1G09850 | Symbols: XBCP3 | XBCP3 (xylem bark cysteine peptidase 3); cysteine-type endopeptidase/ cysteine-type peptidase | chr1:3201848-3203875 FORWARDmoderately similar to ( 360) ORYA_ORYSA Oryzain alpha chain precursor (EC 3.4.22.-) - Oryza sativa (Rice)moderately similar to ( 365) loc_os05g43230 12005.m08451 protein cysteine proteinase RD21a precursor, putative, expressed  no original description 	T
35.2	not assigned.unknown	MDP0000168825	  no original description 	T
19.10	tetrapyrrole synthesis.magnesium chelatase	MDP0000639820	 highly similar to ( 585) AT5G45930 | Symbols: CHLI2, CHL I2, CHLI-2 | CHLI2 (MAGNESIUM CHELATASE I2); ATPase/ magnesium chelatase | chr5:18628095-18629565 FORWARDhighly similar to ( 614) CHLI_SOYBN Magnesium-chelatase subunit chlI, chloroplast precursor (EC 6.6.1.1) (Mg-protoporphyrin IX chelatase) - Glycine max (Soybean)highly similar to ( 580) loc_os03g36540 12003.m08774 protein magnesium-chelatase subunit chlI, chloroplast precursor, putative, expressed no original description 	T
29.4	protein.postranslational modification	MDP0000051653	 nearly identical (1079) AT3G58640 | Symbols:  | protein kinase family protein | chr3:21687153-21692675 REVERSEvery weakly similar to (92.4) CRI4_MAIZE Putative receptor protein kinase CRINKLY4 precursor (EC 2.7.11.1) - Zea mays (Maize)highly similar to ( 998) loc_os01g48330 12001.m11024 protein ATP binding protein, putative, expressed    no original description 	T
27.3.99	RNA.regulation of transcription.unclassified	MDP0000262367	 moderately similar to ( 270) AT5G07900 | Symbols:  | mitochondrial transcription termination factor family protein / mTERF family protein | chr5:2520188-2521405 FORWARDweakly similar to ( 182) loc_os05g33440 12005.m07576 protein mTERF family protein, expressed  no original description 	T
26.24	misc.GCN5-related N-acetyltransferase	MDP0000310632	 moderately similar to ( 271) AT5G11340 | Symbols:  | GCN5-related N-acetyltransferase (GNAT) family protein | chr5:3619226-3621068 FORWARDmoderately similar to ( 259) loc_os01g42470 12001.m150648 protein pre-mRNA-splicing factor cwc24, putative, expressed  no original description 	T
35.2	not assigned.unknown	MDP0000213488	 moderately similar to ( 302) AT4G15830 | Symbols:  | binding | chr4:8995285-8996548 REVERSEmoderately similar to ( 261) loc_os01g70560 12001.m13102 protein expressed protein   no original description 	T
29.4	protein.postranslational modification	MDP0000341745	 moderately similar to ( 201) AT3G58640 | Symbols:  | protein kinase family protein | chr3:21687153-21692675 REVERSEweakly similar to ( 194) loc_os01g48330 12001.m11024 protein ATP binding protein, putative, expressed no original description 	T
19.3	tetrapyrrole synthesis.GSA	MDP0000836141	 highly similar to ( 606) AT1G80600 | Symbols: WIN1 | WIN1 (HOPW1-1-INTERACTING 1); N2-acetyl-L-ornithine:2-oxoglutarate 5-aminotransferase/ catalytic/ pyridoxal phosphate binding / transaminase | chr1:30298675-30300513 REVERSEweakly similar to ( 124) GSA_BRANA Glutamate-1-semialdehyde 2,1-aminomutase, chloroplast precursor (EC 5.4.3.8) (GSA) (Glutamate-1-semialdehyde aminotransferase) (GSA-AT) - Brassica napus (Rape)highly similar to ( 536) loc_os05g03830 12005.m04917 protein acetylornithine aminotransferase, mitochondrial precursor, putative, expressed   no original description 	T
16.8.3.1	secondary metabolism.flavonoids.dihydroflavonols.dihydroflavonol 4-reductase	MDP0000322079	 weakly similar to ( 153) AT1G09490 | Symbols:  | cinnamyl-alcohol dehydrogenase family / CAD family | chr1:3064680-3065815 FORWARDweakly similar to ( 123) DFRA_DIACA Dihydroflavonol-4-reductase (EC 1.1.1.219) (DFR) (Dihydrokaempferol 4-reductase) - Dianthus caryophyllus (Carnation) (Clove pink)weakly similar to ( 140) loc_os01g34480 12001.m150871 protein dihydroflavonol-4-reductase, putative, expressed no original description 	T
26.2	misc.UDP glucosyl and glucoronyl transferases	MDP0000124584	 moderately similar to ( 370) AT1G22340 | Symbols: AtUGT85A7 | AtUGT85A7 (UDP-glucosyl transferase 85A7); UDP-glycosyltransferase/ glucuronosyltransferase/ transferase, transferring glycosyl groups | chr1:7890464-7892090 REVERSEweakly similar to ( 146) UFO2_MAIZE Anthocyanidin 3-O-glucosyltransferase (EC 2.4.1.115) (Flavonol 3-O-glucosyltransferase) (UDP-glucose flavonoid 3-O-glucosyltransferase) (Bronze-1) (Bz-Mc2 allele) - Zea mays (Maize)moderately similar to ( 353) loc_os02g51910 12002.m10222 protein cytokinin-O-glucosyltransferase 2, putative, expressed TrbL  no original description 	T
29.5.11.4.2	protein.degradation.ubiquitin.E3.RING	MDP0000129868	 moderately similar to ( 332) AT3G24800 | Symbols: PRT1 | PRT1 (PROTEOLYSIS 1); ubiquitin-protein ligase | chr3:9055653-9057795 FORWARDmoderately similar to ( 308) loc_os02g40810 12002.m09166 protein ubiquitin protein ligase PRT1, putative, expressed  no original description 	T
17.7.1.2	hormone metabolism.jasmonate.synthesis-degradation.lipoxygenase	MDP0000120271	 highly similar to ( 605) AT1G55020 | Symbols: LOX1 | LOX1; lipoxygenase | chr1:20525798-20530143 FORWARDhighly similar to ( 574) LOX1_SOLTU Lipoxygenase 1 (EC 1.13.11.12) - Solanum tuberosum (Potato)moderately similar to ( 472) loc_os03g49380 12003.m35371 protein lipoxygenase 4, putative, expressed no original description 	T
35.2	not assigned.unknown	MDP0000679173	 highly similar to ( 846) AT3G23810 | Symbols: SAHH2, ATSAHH2 | SAHH2 (S-ADENOSYL-L-HOMOCYSTEINE (SAH) HYDROLASE 2); adenosylhomocysteinase/ binding / catalytic | chr3:8588013-8589671 REVERSEhighly similar to ( 850) SAHH_MEDSA Adenosylhomocysteinase (EC 3.3.1.1) (S-adenosyl-L-homocysteine hydrolase) (AdoHcyase) - Medicago sativa (Alfalfa)highly similar to ( 781) loc_os11g26850 12011.m28797 protein adenosylhomocysteinase, putative, expressed   no original description 	T
35.2	not assigned.unknown	MDP0000337239	 moderately similar to ( 201) AT5G47570 | Symbols:  | unknown protein | chr5:19292869-19294666 REVERSEweakly similar to ( 182) loc_os10g40410 12010.m06809 protein expressed protein  no original description 	T
30.6	signalling.MAP kinases	MDP0000795117	 highly similar to ( 535) AT5G67130 | Symbols:  | phospholipase C/ phosphoric diester hydrolase | chr5:26791599-26793649 FORWARDmoderately similar to ( 474) loc_os09g02729 12009.m059932 protein MAP3K-like protein kinase, putative, expressed  no original description 	T
20.1	stress.biotic	MDP0000712620	 moderately similar to ( 332) AT1G05850 | Symbols: POM1, ERH2, ELP1, CTL1, ELP, HOT2, ATCTL1 | POM1 (POM-POM1); chitinase | chr1:1766833-1768117 REVERSEweakly similar to ( 163) CHI1_GOSHI Endochitinase 1 precursor (EC 3.2.1.14) - Gossypium hirsutum (Upland cotton)moderately similar to ( 295) loc_os08g41100 12008.m08088 protein endochitinase 1 precursor, putative, expressed  no original description 	T
27.2	RNA.transcription	MDP0000303619	 nearly identical (1042) AT1G14790 | Symbols: RDR1, ATRDRP1 | RDR1 (RNA-DEPENDENT RNA POLYMERASE 1); RNA-directed RNA polymerase/ nucleic acid binding | chr1:5094317-5097817 REVERSEhighly similar to ( 803) loc_os02g50330 12002.m10067 protein RNA-directed RNA polymerase 2, putative, expressed  no original description 	T
29.4	protein.postranslational modification	MDP0000179236	 moderately similar to ( 241) AT5G48545 | Symbols:  | histidine triad family protein / HIT family protein | chr5:19676228-19677945 FORWARDvery weakly similar to (53.1) ZB14_MAIZE 14 kDa zinc-binding protein (Protein kinase C inhibitor) (PKCI) - Zea mays (Maize)moderately similar to ( 204) loc_os11g18990 12011.m079983 protein expressed protein no original description 	T
3.3	minor CHO metabolism.sugar alcohols	MDP0000626329	 moderately similar to ( 231) AT4G30310 | Symbols:  | ribitol kinase, putative | chr4:14831913-14835092 FORWARDmoderately similar to ( 224) loc_os02g08130 12002.m33998 protein carbohydrate kinase, putative, expressed  no original description 	T
29.4	protein.postranslational modification	MDP0000138291	 highly similar to ( 553) AT4G11240 | Symbols: TOPP7 | TOPP7; protein serine/threonine phosphatase | chr4:6847333-6849032 FORWARDhighly similar to ( 514) PP1_ORYSA Serine/threonine-protein phosphatase PP1 (EC 3.1.3.16) - Oryza sativa (Rice)highly similar to ( 548) loc_os06g06880 12006.m05413 protein serine/threonine-protein phosphatase PP1, putative, expressed no original description 	T
29.5.11.1	protein.degradation.ubiquitin.ubiquitin	MDP0000315920	 moderately similar to ( 253) AT2G47110 | Symbols: UBQ6 | UBQ6; protein binding | chr2:19344701-19345174 FORWARDweakly similar to ( 150) UBIQ_WHEAT Ubiquitin - Triticum aestivum (Wheat)moderately similar to ( 246) loc_os01g22490 12001.m08729 protein 40S ribosomal protein S27a, putative, expressed   no original description 	T
29.4	protein.postranslational modification	MDP0000139060	 moderately similar to ( 483) AT4G13020 | Symbols: MHK | MHK; ATP binding / kinase/ protein kinase/ protein serine/threonine kinase | chr4:7603947-7606732 FORWARDweakly similar to ( 144) CDC2A_ANTMA Cell division control protein 2 homolog A (EC 2.7.11.22) (EC 2.7.11.23) - Antirrhinum majus (Garden snapdragon)moderately similar to ( 384) loc_os03g63020 12003.m35531 protein serine/threonine-protein kinase MHK, putative, expressed  no original description 	T
34.9	transport.metabolite transporters at the mitochondrial membrane	MDP0000297664	 highly similar to ( 509) AT5G19760 | Symbols:  | dicarboxylate/tricarboxylate carrier (DTC) | chr5:6679591-6681845 REVERSEvery weakly similar to (74.7) BT1_MAIZE Protein brittle-1, chloroplast precursor - Zea mays (Maize)highly similar to ( 509) loc_os05g11780 12005.m05647 protein mitochondrial 2-oxoglutarate/malate carrier protein, putative, expressed  no original description 	T
2.2.2.1.2	major CHO metabolism.degradation.starch.starch cleavage.beta amylase	MDP0000196961	 highly similar to ( 576) AT5G18670 | Symbols: BMY3, BAM9 | BMY3; beta-amylase/ catalytic/ cation binding | chr5:6226138-6227999 FORWARDmoderately similar to ( 275) AMYB_VIGUN Beta-amylase (EC 3.2.1.2) (1,4-alpha-D-glucan maltohydrolase) - Vigna unguiculata (Cowpea)moderately similar to ( 399) loc_os07g47120 12007.m08929 protein beta-amylase, putative, expressed   no original description 	T
35.2	not assigned.unknown	MDP0000504125	 very weakly similar to (59.7) AT1G65720 | Symbols:  | unknown protein | chr1:24440471-24441013 REVERSEvery weakly similar to (58.2) loc_os10g34270 12010.m06253 protein expressed protein no original description 	T
9.6	mitochondrial electron transport / ATP synthesis.cytochrome c	MDP0000237690	 moderately similar to ( 478) AT5G40810 | Symbols:  | cytochrome c1, putative | chr5:16340670-16342327 FORWARDmoderately similar to ( 459) CY12_SOLTU Cytochrome c1, heme protein, mitochondrial precursor (Clone PC18I) (Fragment) - Solanum tuberosum (Potato)moderately similar to ( 474) loc_os05g23620 12005.m64225 protein cytochrome c1, heme protein, mitochondrial precursor, putative, expressed  no original description 	T
29.2.1.2.2.523	protein.synthesis.ribosomal protein.eukaryotic.60S subunit.L23A	MDP0000137399	 moderately similar to ( 227) AT3G55280 | Symbols: RPL23AB | RPL23AB (RIBOSOMAL PROTEIN L23AB); RNA binding / nucleotide binding / structural constituent of ribosome | chr3:20500667-20501519 FORWARDmoderately similar to ( 230) RL23A_DAUCA 60S ribosomal protein L23a - Daucus carota (Carrot)moderately similar to ( 211) loc_os04g42270 12004.m35367 protein 60S ribosomal protein L23a, putative, expressed no original description 	T
23.5.4	nucleotide metabolism.deoxynucleotide metabolism.ribonucleoside-diphosphate reductase	MDP0000413972	 weakly similar to ( 137) AT3G27060 | Symbols: TSO2 | TSO2 (TSO meaning 'ugly' in Chinese); oxidoreductase/ ribonucleoside-diphosphate reductase/ transition metal ion binding | chr3:9979971-9981057 REVERSEweakly similar to ( 148) RIR2_TOBAC Ribonucleoside-diphosphate reductase small chain (EC 1.17.4.1) (Ribonucleotide reductase small subunit) (Ribonucleoside-diphosphate reductase R2 subunit) - Nicotiana tabacum (Common tobacco)weakly similar to ( 129) loc_os06g14620 12006.m06177 protein ribonucleoside-diphosphate reductase small chain, putative, expressed no original description 	T
34.16	transport.ABC transporters and multidrug resistance systems	MDP0000350535	 weakly similar to ( 170) AT2G28070 | Symbols:  | ABC transporter family protein | chr2:11956432-11959782 FORWARDweakly similar to ( 154) loc_os03g64200 12003.m101582 protein ATPase, coupled to transmembrane movement of substances, putative, expressed no original description 	T
29.1.20	protein.aa activation.phenylalanine-tRNA ligase	MDP0000263089	 highly similar to ( 834) AT1G72550 | Symbols:  | tRNA synthetase beta subunit family protein | chr1:27319999-27323908 REVERSEhighly similar to ( 734) loc_os05g48510 12005.m27969 protein phenylalanyl-tRNA synthetase beta chain, putative, expressed no original description 	T
28.1	DNA.synthesis/chromatin structure	MDP0000186665	 moderately similar to ( 260) AT3G12530 | Symbols: PSF2 | PSF2 | chr3:3972604-3973728 REVERSEmoderately similar to ( 265) loc_os01g14610 12001.m42941 protein DNA replication complex GINS protein PSF2, putative, expressed no original description 	T
35.2	not assigned.unknown	MDP0000190809	  no original description 	T
3.5	minor CHO metabolism.others	MDP0000857724	 moderately similar to ( 312) AT1G59950 | Symbols:  | aldo/keto reductase, putative | chr1:22068033-22070588 REVERSEmoderately similar to ( 311) NADO1_ORYSA Probable NAD(P)H-dependent oxidoreductase 1 (EC 1.1.1.-) - Oryza sativa (Rice)moderately similar to ( 323) loc_os04g37470 12004.m08736 protein NAD(P)H-dependent oxidoreductase, putative, expressed  no original description 	T
23.2	nucleotide metabolism.degradation	MDP0000787842	 moderately similar to ( 218) AT2G26230 | Symbols:  | uricase / urate oxidase / nodulin 35, putative | chr2:11164956-11166968 FORWARDmoderately similar to ( 246) URIC_PHAVU Uricase-2 (EC 1.7.3.3) (Uricase II) (Urate oxidase) (Nodule-specific uricase) - Phaseolus vulgaris (Kidney bean) (French bean)moderately similar to ( 214) loc_os01g64520 12001.m97562 protein uricase, putative, expressed no original description 	T
29.2.1.2.2.99	protein.synthesis.ribosomal protein.eukaryotic.60S subunit.unknown	MDP0000138653	 weakly similar to ( 147) AT5G39800 | Symbols:  | 60S ribosomal protein-related | chr5:15935580-15936936 FORWARDweakly similar to ( 137) loc_os02g40490 12002.m09135 protein conserved hypothetical protein no original description 	T
29.3.4.99	protein.targeting.secretory pathway.unspecified	MDP0000178054	 moderately similar to ( 361) AT5G14670 | Symbols: ATARFA1B | ATARFA1B (ADP-ribosylation factor A1B); GTP binding / phospholipase activator/ protein binding | chr5:4729319-4730495 FORWARDmoderately similar to ( 361) ARF2_ORYSA ADP-ribosylation factor 2 - Oryza sativa (Rice)moderately similar to ( 363) loc_os05g41060 12005.m083767 protein ADP-ribosylation factor, putative, expressed  no original description 	T
35.1.41	not assigned.no ontology.hydroxyproline rich proteins	MDP0000223447	 moderately similar to ( 305) AT4G01050 | Symbols:  | hydroxyproline-rich glycoprotein family protein | chr4:455874-458175 FORWARDmoderately similar to ( 223) loc_os02g15750 12002.m06818 protein expressed protein  no original description 	T
17.5.1	hormone metabolism.ethylene.synthesis-degradation	MDP0000788934	 highly similar to ( 504) AT4G22880 | Symbols: LDOX, TDS4, TT18, ANS | LDOX (LEUCOANTHOCYANIDIN DIOXYGENASE); leucocyanidin oxygenase | chr4:12004905-12006059 REVERSEhighly similar to ( 625) LDOX_MALDO Leucoanthocyanidin dioxygenase (EC 1.14.11.19) (LDOX) (Leucocyanidin oxygenase) (Leucoanthocyanidin hydroxylase) (Anthocyanidin synthase) - Malus domestica (Apple) (Malus sylvestris)moderately similar to ( 318) loc_os01g27490 12001.m09199 protein leucoanthocyanidin dioxygenase, putative, expressed  no original description 	T
30.7	signalling.14-3-3 proteins	MDP0000846709	 moderately similar to ( 451) AT1G78300 | Symbols: GRF2, 14-3-3OMEGA, GF14 OMEGA | GRF2 (GENERAL REGULATORY FACTOR 2); protein binding / protein phosphorylated amino acid binding | chr1:29461883-29463052 FORWARDmoderately similar to ( 471) 1433_OENHO 14-3-3-like protein - Oenothera hookeri (Hooker's evening primrose)moderately similar to ( 420) loc_os08g33370 12008.m26501 protein 14-3-3-like protein, putative, expressed no original description 	T
35.2	not assigned.unknown	MDP0000558157	  no original description 	T
29.3.4.99	protein.targeting.secretory pathway.unspecified	MDP0000170867	 moderately similar to ( 362) AT5G14670 | Symbols: ATARFA1B | ATARFA1B (ADP-ribosylation factor A1B); GTP binding / phospholipase activator/ protein binding | chr5:4729319-4730495 FORWARDmoderately similar to ( 358) ARF2_ORYSA ADP-ribosylation factor 2 - Oryza sativa (Rice)moderately similar to ( 360) loc_os01g16030 12001.m150547 protein ADP-ribosylation factor, putative, expressed no original description 	T
35.2	not assigned.unknown	MDP0000511838	 weakly similar to ( 162) AT4G10070 | Symbols:  | KH domain-containing protein | chr4:6295608-6299759 REVERSEvery weakly similar to (64.7) EXTN_TOBAC Extensin precursor (Cell wall hydroxyproline-rich glycoprotein) - Nicotiana tabacum (Common tobacco)very weakly similar to ( 100) loc_os08g01930 12008.m04341 protein far upstream element-binding protein 1, putative, expressed   no original description 	T
35.2	not assigned.unknown	MDP0000485866	 moderately similar to ( 304) AT5G14970 | Symbols:  | unknown protein | chr5:4847368-4848760 FORWARDweakly similar to ( 142) loc_os03g41080 12003.m09177 protein seed maturation protein PM23, putative, expressed  no original description 	T
29.4.1.57	protein.postranslational modification.kinase.receptor like cytoplasmatic kinase VII	MDP0000274017	 highly similar to ( 647) AT5G37790 | Symbols:  | protein kinase family protein | chr5:15008433-15011025 REVERSEweakly similar to ( 172) CRI4_MAIZE Putative receptor protein kinase CRINKLY4 precursor (EC 2.7.11.1) - Zea mays (Maize)moderately similar to ( 244) loc_os03g13820 12003.m101264 protein ATP binding protein, putative, expressed  no original description 	T
35.2	not assigned.unknown	MDP0000881647	 highly similar to ( 653) AT5G50420 | Symbols:  | unknown protein | chr5:20530453-20532635 REVERSEhighly similar to ( 574) loc_os07g38490 12007.m08095 protein GDP-fucose protein-O-fucosyltransferase 2, putative, expressed  MSP1_C  no original description 	T
11.1.8	lipid metabolism.FA synthesis and FA elongation.acyl coa ligase	MDP0000166116	 highly similar to ( 791) AT5G16340 | Symbols:  | AMP-binding protein, putative | chr5:5349255-5350907 REVERSEweakly similar to ( 168) 4CL1_PETCR 4-coumarate--CoA ligase 1 (EC 6.2.1.12) (4CL 1) (4-coumaroyl-CoA synthase 1) - Petroselinum crispum (Parsley) (Petroselinum hortense)highly similar to ( 749) loc_os04g57850 12004.m10669 protein AMP-binding protein, putative, expressed   no original description 	T
29.3.1	protein.targeting.nucleus	MDP0000666968	 nearly identical (1138) AT2G41620 | Symbols:  | nucleoporin interacting component family protein | chr2:17350419-17354997 REVERSEnearly identical (1068) loc_os03g22690 12003.m07668 protein nucleoporin interacting component, putative, expressed  no original description 	T
28.1	DNA.synthesis/chromatin structure	MDP0000203318	 moderately similar to ( 394) AT5G06260 | Symbols:  | nucleolar protein-related | chr5:1902755-1904835 REVERSEmoderately similar to ( 343) loc_os12g06100 12012.m04601 protein TLD family protein, expressed no original description 	T
6.3	gluconeogenesis / glyoxylate cycle.Malate DH	MDP0000334963	 highly similar to ( 561) AT2G22780 | Symbols: PMDH1 | PMDH1 (PEROXISOMAL NAD-MALATE DEHYDROGENASE 1); L-malate dehydrogenase/ binding / catalytic/ malate dehydrogenase/ oxidoreductase/ oxidoreductase, acting on the CH-OH group of donors, NAD or NADP as acceptor | chr2:9689995-9691923 REVERSEhighly similar to ( 569) MDHG_CUCSA Malate dehydrogenase, glyoxysomal precursor (EC 1.1.1.37) - Cucumis sativus (Cucumber)highly similar to ( 546) loc_os12g43630 12012.m26992 protein malate dehydrogenase, glyoxysomal precursor, putative, expressed   no original description 	T
26.21	misc.protease inhibitor/seed storage/lipid transfer protein (LTP) family protein	MDP0000893990	 very weakly similar to (85.5) AT5G48485 | Symbols: DIR1 | DIR1 (DEFECTIVE IN INDUCED RESISTANCE 1); lipid binding / lipid transporter | chr5:19646317-19646625 REVERSEvery weakly similar to (94.4) loc_os07g18750 12007.m06314 protein PVR3-like protein, putative, expressed  no original description 	T
15.3	metal handling.regulation	MDP0000663130	 moderately similar to ( 287) AT4G14710 | Symbols: ATARD2 | acireductone dioxygenase [iron(II)-requiring]/ metal ion binding | chr4:8424943-8426360 REVERSEmoderately similar to ( 260) ARD2_ORYSA 1,2-dihydroxy-3-keto-5-methylthiopentene dioxygenase 2 (EC 1.13.-.-) (Aci-reductone dioxygenase 2) (Submergence-induced protein 2A) - Oryza sativa (Rice)moderately similar to ( 260) loc_os03g06620 12003.m06180 protein 1,2-dihydroxy-3-keto-5-methylthiopentene dioxygenase 2, putative, expressed  no original description 	T
35.1	not assigned.no ontology	MDP0000156002	 moderately similar to ( 375) AT4G26860 | Symbols:  | pyridoxal phosphate binding | chr4:13503291-13504693 REVERSEmoderately similar to ( 341) loc_os05g05740 12005.m05101 protein proline synthetase co-transcribed bacterial homolog protein, putative, expressed  no original description 	T
35.2	not assigned.unknown	MDP0000215990	 highly similar to ( 622) AT1G27510 | Symbols:  | INVOLVED IN: response to singlet oxygen; LOCATED IN: thylakoid membrane; EXPRESSED IN: 22 plant structures; EXPRESSED DURING: 13 growth stages; BEST Arabidopsis thaliana protein match is: EX1 (EXECUTER1) (TAIR:AT4G33630.2); Has 213 Blast hits to 208 proteins in 76 species: Archae - 0; Bacteria - 13; Metazoa - 63; Fungi - 31; Plants - 69; Viruses - 0; Other Eukaryotes - 37 (source: NCBI BLink). | chr1:9554945-9558293 REVERSEhighly similar to ( 564) loc_os01g01790 12001.m42829 protein expressed protein   no original description 	T
30.2.11	signalling.receptor kinases.leucine rich repeat XI	MDP0000128521	 moderately similar to ( 494) AT5G61240 | Symbols:  | protein binding | chr5:24629485-24631958 FORWARDweakly similar to ( 120) RPK1_IPONI Receptor-like protein kinase precursor (EC 2.7.11.1) - Ipomoea nil (Japanese morning glory) (Pharbitis nil)moderately similar to ( 445) loc_os03g11340 12003.m06585 protein leucine-rich repeat resistance protein, putative, expressed  no original description 	T
35.2	not assigned.unknown	MDP0000780769	   no original description 	T
35.2	not assigned.unknown	MDP0000289668	  no original description 	T
27.3.99	RNA.regulation of transcription.unclassified	MDP0000233616	 moderately similar to ( 258) AT2G36480 | Symbols:  | zinc finger (C2H2-type) family protein | chr2:15302567-15306274 REVERSEweakly similar to ( 182) loc_os09g39270 12009.m06866 protein expressed protein   no original description 	T
35.1.5	not assigned.no ontology.pentatricopeptide (PPR) repeat-containing protein	MDP0000221486	 highly similar to ( 530) AT2G29760 | Symbols:  | pentatricopeptide (PPR) repeat-containing protein | chr2:12712884-12715100 FORWARDweakly similar to ( 116) RF1_ORYSA Rf1 protein, mitochondrial precursor (PPR protein) (Fertility restorer) (Restorer for CMS) - Oryza sativa (Rice)moderately similar to ( 454) loc_os03g27880 12003.m08112 protein EMB2758, putative   TrbL SPT5  no original description 	T
26.19	misc.plastocyanin-like	MDP0000951420	 weakly similar to ( 158) AT3G20570 | Symbols:  | plastocyanin-like domain-containing protein | chr3:7186754-7187453 REVERSEvery weakly similar to (77.8) NO552_SOYBN Early nodulin 55-2 precursor (N-55-2) (Nodulin-315) - Glycine max (Soybean)weakly similar to ( 124) loc_os01g57880 12001.m11936 protein early nodulin-like protein 3 precursor, putative, expressed   no original description 	T
27.3.5	RNA.regulation of transcription.ARR	MDP0000155328	 moderately similar to ( 327) AT4G16110 | Symbols: ARR2 | ARR2 (ARABIDOPSIS RESPONSE REGULATOR 2); transcription factor/ two-component response regulator | chr4:9112979-9115785 FORWARDvery weakly similar to (86.7) PRR95_ORYSA Two-component response regulator-like PRR95 (Pseudo-response regulator 95) (OsPRR95) - Oryza sativa (Rice)moderately similar to ( 442) loc_os03g12350 12003.m101249 protein two-component response regulator ARR1, putative, expressed   no original description 	T
27.3.20	RNA.regulation of transcription.G2-like transcription factor family, GARP	MDP0000155328	 moderately similar to ( 327) AT4G16110 | Symbols: ARR2 | ARR2 (ARABIDOPSIS RESPONSE REGULATOR 2); transcription factor/ two-component response regulator | chr4:9112979-9115785 FORWARDvery weakly similar to (86.7) PRR95_ORYSA Two-component response regulator-like PRR95 (Pseudo-response regulator 95) (OsPRR95) - Oryza sativa (Rice)moderately similar to ( 442) loc_os03g12350 12003.m101249 protein two-component response regulator ARR1, putative, expressed   no original description 	T
16.1.4.1	secondary metabolism.isoprenoids.carotenoids.phytoene synthase	MDP0000177623	 moderately similar to ( 487) AT5G17230 | Symbols:  | phytoene synthase (PSY) / geranylgeranyl-diphosphate geranylgeranyl transferase | chr5:5659839-5662087 REVERSEhighly similar to ( 510) PSY_DAUCA Phytoene synthase, chloroplast precursor (EC 2.5.1.-) - Daucus carota (Carrot)moderately similar to ( 484) loc_os12g43130 12012.m26789 protein phytoene synthase, chloroplast precursor, putative, expressed no original description 	T
28.1	DNA.synthesis/chromatin structure	MDP0000212806	 weakly similar to ( 127) AT1G09815 | Symbols: POLD4 | POLD4 (POLYMERASE DELTA 4); DNA-directed DNA polymerase | chr1:3189460-3190050 FORWARDvery weakly similar to (83.6) loc_os09g34850 12009.m22171 protein delta DNA polymerase, putative, expressed  no original description 	T
20.2.1	stress.abiotic.heat	MDP0000233092	 moderately similar to ( 282) AT4G09350 | Symbols:  | DNAJ heat shock N-terminal domain-containing protein | chr4:5931317-5932152 REVERSEmoderately similar to ( 248) loc_os11g10990 12011.m05292 protein heat shock protein binding protein, putative, expressed  no original description 	T
30.2.11	signalling.receptor kinases.leucine rich repeat XI	MDP0000121482	 highly similar to ( 818) AT5G14210 | Symbols:  | leucine-rich repeat transmembrane protein kinase, putative | chr5:4578503-4581374 REVERSEweakly similar to ( 125) NORK_MEDTR Nodulation receptor kinase precursor (EC 2.7.11.1) (Does not make infections protein 2) (Symbiosis receptor-like kinase) (MtSYMRK) - Medicago truncatula (Barrel medic)highly similar to ( 516) loc_os11g20360 12011.m28791 protein ATP binding protein, putative, expressed  no original description 	T
27.3.67	RNA.regulation of transcription.putative transcription regulator	MDP0000577746	 weakly similar to ( 188) AT3G04590 | Symbols:  | DNA-binding family protein | chr3:1239245-1241603 REVERSEvery weakly similar to (97.8) loc_os08g40150 12008.m26527 protein AT-hook protein 1, putative, expressed  no original description 	T
35.2	not assigned.unknown	MDP0000809801	  no original description 	T
29.2.1.2.2.537	protein.synthesis.ribosomal protein.eukaryotic.60S subunit.L37A	MDP0000938471	 weakly similar to ( 182) AT3G10950 | Symbols:  | 60S ribosomal protein L37a (RPL37aB) | chr3:3423893-3424566 FORWARDweakly similar to ( 186) RL37A_GOSHI 60S ribosomal protein L37a - Gossypium hirsutum (Upland cotton)weakly similar to ( 186) loc_os05g48320 12005.m27963 protein 60S ribosomal protein L37a, putative, expressed  no original description 	T
35.2	not assigned.unknown	MDP0000320108	 moderately similar to ( 369) AT4G27030 | Symbols:  | small conjugating protein ligase | chr4:13571951-13572922 FORWARDmoderately similar to ( 240) loc_os08g08850 12008.m05020 protein carF, putative, expressed   no original description 	T
10.8.1	cell wall.pectin*esterases.PME	MDP0000163228	 weakly similar to ( 163) AT3G59010 | Symbols:  | pectinesterase family protein | chr3:21803015-21805098 REVERSEvery weakly similar to (99.0) PME2_CITSI Pectinesterase-2 precursor (EC 3.1.1.11) (Pectin methylesterase) (PE) - Citrus sinensis (Sweet orange)weakly similar to ( 110) loc_os01g13320 12001.m07941 protein pectinesterase-2 precursor, putative, expressed no original description 	T
1.1.5.2	PS.lightreaction.other electron carrier (ox/red).ferredoxin	MDP0000242154	 weakly similar to ( 144) AT1G02180 | Symbols:  | ferredoxin-related | chr1:413619-414505 REVERSEmoderately similar to ( 219) loc_os08g35710 12008.m07560 protein expressed protein  no original description 	T
35.2	not assigned.unknown	MDP0000190208	 weakly similar to ( 130) AT1G76200 | Symbols:  | unknown protein | chr1:28593351-28593980 FORWARDweakly similar to ( 112) loc_os05g03150 12005.m83955 protein expressed protein no original description 	T
33.99	development.unspecified	MDP0000123890	 moderately similar to ( 229) AT1G11430 | Symbols:  | plastid developmental protein DAG, putative | chr1:3847273-3848938 FORWARDmoderately similar to ( 229) DAG_ANTMA DAG protein, chloroplast precursor - Antirrhinum majus (Garden snapdragon)moderately similar to ( 226) loc_os08g04450 12008.m080079 protein DAG protein, chloroplast precursor, putative, expressed MSP1_C  no original description 	T
35.2	not assigned.unknown	MDP0000242477	 moderately similar to ( 402) AT3G03890 | Symbols:  | FMN binding | chr3:999837-1001996 REVERSEweakly similar to ( 161) RL10A_CHLRE 60S ribosomal protein L10a - Chlamydomonas reinhardtiimoderately similar to ( 383) loc_os07g38620 12007.m29233 protein root border cell-specific protein, putative, expressed  no original description 	T
30.2.99	signalling.receptor kinases.misc	MDP0000302448	 highly similar to ( 752) AT1G07705 | Symbols:  | transcription regulator | chr1:2382090-2385856 FORWARDvery weakly similar to (82.8) NORK_PEA Nodulation receptor kinase precursor (EC 2.7.11.1) - Pisum sativum (Garden pea)highly similar to ( 671) loc_os03g52594 12003.m35626 protein CCR4-NOT transcription complex subunit 2, putative, expressed   no original description 	T
26.28	misc.GDSL-motif lipase	MDP0000282119	 moderately similar to ( 245) AT3G48460 | Symbols:  | GDSL-motif lipase/hydrolase family protein | chr3:17949496-17951082 FORWARDvery weakly similar to (90.1) EST_HEVBR Esterase precursor (EC 3.1.1.-) (Early nodule-specific protein homolog) (Latex allergen Hev b 13) - Hevea brasiliensis (Para rubber tree)moderately similar to ( 210) loc_os03g62740 12003.m11151 protein esterase precursor, putative, expressed  no original description 	T
20.2.1	stress.abiotic.heat	MDP0000026750	 highly similar to ( 639) AT1G68370 | Symbols: ARG1 | ARG1 (ALTERED RESPONSE TO GRAVITY 1); cytoskeletal protein binding | chr1:25632046-25634527 REVERSEvery weakly similar to (69.3) DNJH_CUCSA DnaJ protein homolog (DNAJ-1) - Cucumis sativus (Cucumber)highly similar to ( 602) loc_os01g32870 12001.m43034 protein chaperone protein dnaJ 15, putative, expressed no original description 	T
29.4	protein.postranslational modification	MDP0000124959	 highly similar to ( 645) AT4G03415 | Symbols:  | catalytic/ protein serine/threonine phosphatase | chr4:1503789-1505510 REVERSEmoderately similar to ( 472) loc_os07g37890 12007.m08038 protein catalytic/ protein phosphatase type 2C, putative, expressed  no original description 	T
35.2	not assigned.unknown	MDP0000289300	 weakly similar to ( 134) AT5G22580 | Symbols:  | FUNCTIONS IN: molecular_function unknown; INVOLVED IN: biological_process unknown; LOCATED IN: chloroplast; EXPRESSED IN: 22 plant structures; EXPRESSED DURING: 13 growth stages; CONTAINS InterPro DOMAIN/s: Stress responsive alpha-beta barrel (InterPro:IPR013097), Dimeric alpha-beta barrel (InterPro:IPR011008); BEST Arabidopsis thaliana protein match is: HS1 (HEAT STABLE PROTEIN 1) (TAIR:AT3G17210.1); Has 231 Blast hits to 231 proteins in 57 species: Archae - 0; Bacteria - 89; Metazoa - 0; Fungi - 4; Plants - 98; Viruses - 0; Other Eukaryotes - 40 (source: NCBI BLink). | chr5:7502709-7503137 FORWARDweakly similar to ( 121) loc_os11g05290 12011.m04727 protein pop3 peptide, putative, expressed  no original description 	T
26.17	misc.dynamin	MDP0000466196	 highly similar to ( 757) AT1G60500 | Symbols:  | dynamin family protein | chr1:22291582-22293822 FORWARDhighly similar to ( 683) loc_os03g15420 12003.m06979 protein GTP binding protein, putative, expressed  no original description 	T
33.99	development.unspecified	MDP0000295766	 nearly identical (1327) AT1G80490 | Symbols: TPR1 | TPR1 (TOPLESS-RELATED 1) | chr1:30261094-30266446 REVERSEvery weakly similar to (62.8) PF20_CHLRE Flagellar WD repeat protein PF20 - Chlamydomonas reinhardtiinearly identical (1310) loc_os03g14980 12003.m101111 protein lissencephaly type-1-like homology motif, putative, expressed     no original description 	T
35.2	not assigned.unknown	MDP0000299780	 very weakly similar to (60.8) AT3G47070 | Symbols:  | unknown protein | chr3:17337205-17337507 REVERSEvery weakly similar to (50.4) loc_os07g13969 12007.m79892 protein expressed protein no original description 	T
34.7	transport.phosphate	MDP0000942873	 moderately similar to ( 322) AT2G38940 | Symbols: ATPT2, PHT1;4 | ATPT2 (ARABIDOPSIS THALIANA PHOSPHATE TRANSPORTER 2); carbohydrate transmembrane transporter/ inorganic phosphate transmembrane transporter/ phosphate transmembrane transporter/ sugar:hydrogen symporter | chr2:16258500-16260104 FORWARDmoderately similar to ( 269) loc_os08g45000 12008.m08471 protein inorganic phosphate transporter 1-7, putative, expressed  no original description 	T
16.8.3.1	secondary metabolism.flavonoids.dihydroflavonols.dihydroflavonol 4-reductase	MDP0000268045	 highly similar to ( 506) AT1G15950 | Symbols: CCR1, IRX4, ATCCR1 | CCR1 (CINNAMOYL COA REDUCTASE 1); cinnamoyl-CoA reductase | chr1:5478855-5481949 FORWARDmoderately similar to ( 220) DFRA_DIACA Dihydroflavonol-4-reductase (EC 1.1.1.219) (DFR) (Dihydrokaempferol 4-reductase) - Dianthus caryophyllus (Carnation) (Clove pink)moderately similar to ( 470) loc_os09g25150 12009.m22089 protein dihydroflavonol-4-reductase, putative, expressed  no original description 	T
35.2	not assigned.unknown	MDP0000184760	 weakly similar to ( 187) AT2G38640 | Symbols:  | unknown protein | chr2:16157725-16158474 REVERSEweakly similar to ( 168) loc_os05g24650 12005.m06759 protein expressed protein  no original description 	T
29.2.3	protein.synthesis.initiation	MDP0000160803	 moderately similar to ( 204) AT2G24060 | Symbols:  | translation initiation factor 3 (IF-3) family protein | chr2:10229453-10231390 FORWARDweakly similar to ( 176) loc_os02g55120 12002.m10538 protein translation initiation factor IF-3, chloroplast precursor, putative, expressed TrbL  no original description 	T
29.5.2	protein.degradation.autophagy	MDP0000655948	 weakly similar to ( 195) AT1G62040 | Symbols: ATG8C | ATG8C (autophagy 8c); microtubule binding | chr1:22933216-22934253 FORWARDweakly similar to ( 200) loc_os08g09240 12008.m26825 protein autophagy-related protein 8 precursor, putative, expressed  no original description 	T
33.99	development.unspecified	MDP0000141463	 moderately similar to ( 347) AT1G10200 | Symbols: WLIM1 | WLIM1; transcription factor/ zinc ion binding | chr1:3346677-3347763 REVERSEmoderately similar to ( 279) SF3_HELAN Pollen-specific protein SF3 - Helianthus annuus (Common sunflower)moderately similar to ( 325) loc_os12g32620 12012.m07062 protein pollen-specific protein SF3, putative, expressed no original description 	T
27.3.25	RNA.regulation of transcription.MYB domain transcription factor family	MDP0000211677	 moderately similar to ( 226) AT4G38620 | Symbols: ATMYB4, MYB4 | MYB4; DNA binding / transcription factor | chr4:18053866-18054876 FORWARDmoderately similar to ( 226) MYB1_HORVU Myb-related protein Hv1 - Hordeum vulgare (Barley)moderately similar to ( 227) loc_os09g36730 12009.m06622 protein myb-related protein Hv1, putative, expressed no original description 	T
29.2.1.1.1.2.85	protein.synthesis.ribosomal protein.prokaryotic.chloroplast.50S subunit.PSRP5	MDP0000196387	 very weakly similar to (58.9) AT3G56910 | Symbols: PSRP5 | PSRP5 (PLASTID-SPECIFIC 50S RIBOSOMAL PROTEIN 5) | chr3:21069558-21070257 REVERSEvery weakly similar to (65.1) PSRP5_PEA Plastid-specific 50S ribosomal protein 5, chloroplast precursor (PSRP-5) (50S ribosomal protein L18) (CL18) - Pisum sativum (Garden pea) no original description 	T
29.4	protein.postranslational modification	MDP0000320932	 highly similar to ( 847) AT3G01090 | Symbols: AKIN10 | AKIN10 (Arabidopsis SNF1 kinase homolog 10); protein binding / protein kinase | chr3:31437-33977 REVERSEhighly similar to ( 616) RKIN1_SECCE Carbon catabolite derepressing protein kinase (EC 2.7.11.1) - Secale cereale (Rye)highly similar to ( 742) loc_os05g45420 12005.m27631 protein SNF1-related protein kinase catalytic alpha subunit KIN10, putative, expressed  no original description 	T
35.2	not assigned.unknown	MDP0000243083	 very weakly similar to (79.7) AT5G59050 | Symbols:  | unknown protein | chr5:23841849-23842519 REVERSEvery weakly similar to (51.6) loc_os05g37930 12005.m07972 protein expressed protein no original description 	T
8.1.3	TCA / org transformation.TCA.aconitase	MDP0000259849	 highly similar to ( 741) AT4G13430 | Symbols: IIL1 | IIL1 (ISOPROPYL MALATE ISOMERASE LARGE SUBUNIT 1); 4 iron, 4 sulfur cluster binding / hydro-lyase/ lyase | chr4:7804194-7807789 REVERSEvery weakly similar to (78.2) ACOC_CUCMA Aconitate hydratase, cytoplasmic (EC 4.2.1.3) (Citrate hydro-lyase) (Aconitase) - Cucurbita maxima (Pumpkin) (Winter squash)highly similar to ( 759) loc_os02g03260 12002.m33530 protein 3-isopropylmalate dehydratase large subunit 2, putative, expressed   no original description 	T
35.2	not assigned.unknown	MDP0000205391	 weakly similar to ( 138) AT4G39840 | Symbols:  | unknown protein | chr4:18485268-18486623 FORWARDvery weakly similar to (93.2) loc_os09g20540 12009.m05261 protein expressed protein   no original description 	T
35.2	not assigned.unknown	MDP0000210038	 weakly similar to ( 139) AT4G20150 | Symbols:  | unknown protein | chr4:10888529-10889500 REVERSEweakly similar to ( 144) loc_os07g31490 12007.m079689 protein expressed protein no original description 	T
29.2.3	protein.synthesis.initiation	MDP0000240478	 moderately similar to ( 282) AT1G13950 | Symbols: EIF-5A, ELF5A-1, ATELF5A-1, EIF5A | ELF5A-1 (EUKARYOTIC ELONGATION FACTOR 5A-1); translation initiation factor | chr1:4773631-4774668 FORWARDmoderately similar to ( 309) IF5A2_NICPL Eukaryotic translation initiation factor 5A-2 (eIF-5A-2) (eIF-4D) - Nicotiana plumbaginifolia (Leadwort-leaved tobacco)moderately similar to ( 303) loc_os12g32240 12012.m07030 protein eukaryotic translation initiation factor 5A-2, putative, expressed no original description 	T
29.5.11.20	protein.degradation.ubiquitin.proteasom	MDP0000612763	 moderately similar to ( 383) AT1G64520 | Symbols: RPN12a | RPN12a (Regulatory Particle non-ATPase 12a); peptidase | chr1:23956459-23958120 FORWARDmoderately similar to ( 384) loc_os07g25420 12007.m29149 protein 26S proteasome non-ATPase regulatory subunit 8, putative, expressed  no original description 	T
30.6	signalling.MAP kinases	MDP0000879089	 highly similar to ( 853) AT5G19010 | Symbols: MPK16 | MPK16; MAP kinase | chr5:6345096-6347676 REVERSEhighly similar to ( 813) MPK14_ORYSA Mitogen-activated protein kinase 14 (EC 2.7.11.24) (MAP kinase 14) - Oryza sativa (Rice)highly similar to ( 813) loc_os05g05160 12005.m05043 protein extracellular signal-regulated kinase 1, putative, expressed   no original description 	T
29.4	protein.postranslational modification	MDP0000879089	 highly similar to ( 853) AT5G19010 | Symbols: MPK16 | MPK16; MAP kinase | chr5:6345096-6347676 REVERSEhighly similar to ( 813) MPK14_ORYSA Mitogen-activated protein kinase 14 (EC 2.7.11.24) (MAP kinase 14) - Oryza sativa (Rice)highly similar to ( 813) loc_os05g05160 12005.m05043 protein extracellular signal-regulated kinase 1, putative, expressed   no original description 	T
27.4	RNA.RNA binding	MDP0000188384	 moderately similar to ( 469) AT3G04610 | Symbols: FLK | FLK (flowering locus KH domain); RNA binding / nucleic acid binding | chr3:1250762-1254272 REVERSEmoderately similar to ( 392) loc_os12g40560 12012.m07840 protein KH domain containing protein, expressed   no original description 	T
26.22	misc.short chain dehydrogenase/reductase (SDR)	MDP0000171200	 moderately similar to ( 382) AT1G24360 | Symbols:  | 3-oxoacyl-(acyl-carrier protein) reductase, chloroplast / 3-ketoacyl-acyl carrier protein reductase | chr1:8640820-8643283 FORWARDmoderately similar to ( 408) FABG1_BRANA 3-oxoacyl-[acyl-carrier-protein] reductase 1, chloroplast precursor (EC 1.1.1.100) (3-ketoacyl-acyl carrier protein reductase 1) (Beta-keto acyl-carrier protein reductase 1) - Brassica napus (Rape)moderately similar to ( 411) loc_os04g30760 12004.m08180 protein 3-oxoacyl-reductase, chloroplast precursor, putative, expressed   no original description 	T
29.2.1.2.2.5	protein.synthesis.ribosomal protein.eukaryotic.60S subunit.L5	MDP0000525119	 moderately similar to ( 470) AT5G39740 | Symbols: OLI7, RPL5B | 60S ribosomal protein L5 (RPL5B) | chr5:15903365-15905185 FORWARDmoderately similar to ( 468) RL5_CUCSA 60S ribosomal protein L5 - Cucumis sativus (Cucumber)moderately similar to ( 447) loc_os01g67126 12001.m43540 protein 60S ribosomal protein L5-2, putative, expressed  no original description 	T
28.1	DNA.synthesis/chromatin structure	MDP0000271720	 moderately similar to ( 264) AT3G12530 | Symbols: PSF2 | PSF2 | chr3:3972604-3973728 REVERSEmoderately similar to ( 263) loc_os01g14610 12001.m42941 protein DNA replication complex GINS protein PSF2, putative, expressed  no original description 	T
35.2	not assigned.unknown	MDP0000908370	 weakly similar to ( 147) AT3G50685 | Symbols:  | unknown protein | chr3:18834272-18834748 REVERSEweakly similar to ( 113) loc_os10g15300 12010.m04723 protein expressed protein   no original description 	T
35.2	not assigned.unknown	MDP0000947876	 very weakly similar to (81.6) AT5G20900 | Symbols: JAZ12, TIFY3B | JAZ12 (JASMONATE-ZIM-DOMAIN PROTEIN 12) | chr5:7090883-7092201 FORWARDvery weakly similar to (83.6) loc_os04g55920 12004.m35599 protein ZIM motif family protein, expressed no original description 	T
31.1	cell.organisation	MDP0000192830	 highly similar to ( 826) AT1G75780 | Symbols: TUB1 | TUB1; GTP binding / GTPase/ structural molecule | chr1:28451378-28453602 REVERSEhighly similar to ( 831) TBB6_GOSHI Tubulin beta-6 chain (Beta-6 tubulin) - Gossypium hirsutum (Upland cotton)highly similar to ( 827) loc_os03g56810 12003.m35436 protein tubulin beta-7 chain, putative, expressed  no original description 	T
34.3	transport.amino acids	MDP0000231477	 moderately similar to ( 350) AT3G56200 | Symbols:  | amino acid transporter family protein | chr3:20850087-20851779 FORWARDmoderately similar to ( 322) loc_os01g61044 12001.m12231 protein amino acid-polyamine transporter, putative, expressed  no original description 	T
17.1.3	hormone metabolism.abscisic acid.induced-regulated-responsive-activated	MDP0000312509	 moderately similar to ( 260) AT1G74520 | Symbols: ATHVA22A | ATHVA22A | chr1:28008109-28009156 REVERSEweakly similar to ( 104) HVA22_HORVU Protein HVA22 - Hordeum vulgare (Barley)moderately similar to ( 228) loc_os02g51290 12002.m10163 protein HVA22-like protein a, putative, expressed no original description 	T
33.99	development.unspecified	MDP0000198044	 moderately similar to ( 415) AT3G61790 | Symbols:  | seven in absentia (SINA) family protein | chr3:22871974-22873543 REVERSEweakly similar to ( 152) SINAL_DROME Probable E3 ubiquitin-protein ligase sina-like CG13030 (EC 6.3.2.-) - Drosophila melanogaster (Fruit fly)moderately similar to ( 427) loc_os05g14860 12005.m05897 protein ubiquitin ligase SINAT3, putative, expressed   no original description 	T
35.2	not assigned.unknown	MDP0000200184	 moderately similar to ( 313) AT1G36990 | Symbols:  | unknown protein | chr1:14027594-14029711 REVERSEweakly similar to ( 103) loc_os04g37540 12004.m08743 protein expressed protein no original description 	T
35.2	not assigned.unknown	MDP0000602560	 nearly identical (1226) ATCG01280 | Symbols: YCF2.2 | hypothetical protein | chrC:145291-152175 REVERSEnearly identical (1259) YCF2_PANGI Protein ycf2 - Panax ginseng (Korean ginseng) no original description 	T
35.2	not assigned.unknown	MDP0000136295	 moderately similar to ( 355) AT3G63300 | Symbols:  | phosphoinositide binding | chr3:23385464-23387625 FORWARDmoderately similar to ( 282) loc_os12g41140 12012.m26965 protein expressed protein   no original description 	T
29.2.1.2.2.5	protein.synthesis.ribosomal protein.eukaryotic.60S subunit.L5	MDP0000163856	 moderately similar to ( 459) AT3G25520 | Symbols: ATL5, PGY3, OLI5, RPL5A | ATL5 (A. THALIANA RIBOSOMAL PROTEIN L5); 5S rRNA binding / structural constituent of ribosome | chr3:9269573-9271327 REVERSEmoderately similar to ( 471) RL5_CUCSA 60S ribosomal protein L5 - Cucumis sativus (Cucumber)moderately similar to ( 438) loc_os01g67126 12001.m43540 protein 60S ribosomal protein L5-2, putative, expressed no original description 	T
4.1.15	glycolysis.cytosolic branch.phospho-enol-pyruvate carboxylase (PEPC)	MDP0000185414	 weakly similar to ( 110) AT3G14940 | Symbols: ATPPC3 | ATPPC3 (PHOSPHOENOLPYRUVATE CARBOXYLASE 3); phosphoenolpyruvate carboxylase | chr3:5025584-5029476 FORWARDweakly similar to ( 113) CAPP1_SOYBN Phosphoenolpyruvate carboxylase, housekeeping isozyme (EC 4.1.1.31) (PEPCase) (PEPC 1) - Glycine max (Soybean)very weakly similar to (95.1) loc_os08g27840 12008.m26491 protein phosphoenolpyruvate carboxylase 2, putative, expressed  no original description 	T
20.2.3	stress.abiotic.drought/salt	MDP0000148807	 highly similar to ( 982) AT1G78240 | Symbols: TSD2, QUA2 | TSD2 (TUMOROUS SHOOT DEVELOPMENT 2); methyltransferase | chr1:29433173-29435815 REVERSEhighly similar to ( 764) loc_os02g51860 12002.m10217 protein ATP binding protein, putative, expressed     no original description 	T
35.2	not assigned.unknown	MDP0000191681	 highly similar to ( 961) AT1G22060 | Symbols:  | LOCATED IN: vacuole; EXPRESSED IN: 23 plant structures; EXPRESSED DURING: 13 growth stages; BEST Arabidopsis thaliana protein match is: F-box family protein (TAIR:AT1G22000.1); Has 67094 Blast hits to 30189 proteins in 1408 species: Archae - 880; Bacteria - 6261; Metazoa - 35085; Fungi - 5326; Plants - 2622; Viruses - 293; Other Eukaryotes - 16627 (source: NCBI BLink). | chr1:7773373-7780586 REVERSEhighly similar to ( 915) loc_os10g21940 12010.m05204 protein expressed protein   no original description 	T
35.1	not assigned.no ontology	MDP0000231035	 weakly similar to ( 109) AT5G27760 | Symbols:  | hypoxia-responsive family protein | chr5:9830396-9831559 FORWARDweakly similar to ( 102) loc_os07g47670 12007.m079790 protein hypoxia induced protein conserved region containing protein, expressed no original description 	T
29.2.1.2.1.9	protein.synthesis.ribosomal protein.eukaryotic.40S subunit.S9	MDP0000232369	 moderately similar to ( 338) AT5G39850 | Symbols:  | 40S ribosomal protein S9 (RPS9C) | chr5:15950053-15951171 FORWARDweakly similar to ( 112) RS9_TOBAC 40S ribosomal protein S9 (S4) (Fragment) - Nicotiana tabacum (Common tobacco)moderately similar to ( 326) loc_os11g38959 12011.m80083 protein 40S ribosomal protein S9, putative, expressed  no original description 	T
35.2	not assigned.unknown	MDP0000482479	  no original description 	T
30.3	signalling.calcium	MDP0000307702	 moderately similar to ( 215) AT3G24110 | Symbols:  | calcium-binding EF hand family protein | chr3:8704429-8705413 REVERSEvery weakly similar to (55.1) CDPK2_ORYSA Calcium-dependent protein kinase, isoform 2 (EC 2.7.11.1) (CDPK 2) - Oryza sativa (Rice)weakly similar to ( 196) loc_os06g40720 12006.m08607 protein calcium ion binding protein, putative, expressed  no original description 	T
26.1	misc.misc2	MDP0000209368	 moderately similar to ( 272) AT4G37550 | Symbols:  | formamidase, putative / formamide amidohydrolase, putative | chr4:17643684-17645249 FORWARDmoderately similar to ( 246) loc_os01g55950 12001.m11751 protein acetamidase, putative, expressed  no original description 	T
11.1.1.1	lipid metabolism.FA synthesis and FA elongation.Acetyl CoA Carboxylation.homomeric Enzyme	MDP0000219975	 nearly identical (3487) AT1G36160 | Symbols: ACC1, AT-ACC1, EMB22, GK, PAS3 | ACC1 (ACETYL-COENZYME A CARBOXYLASE 1); acetyl-CoA carboxylase | chr1:13534196-13543773 FORWARDmoderately similar to ( 215) MCCA_ORYSA Methylcrotonoyl-CoA carboxylase subunit alpha, mitochondrial precursor (EC 6.4.1.4) (3-methylcrotonyl-CoA carboxylase 1) (MCCase subunit alpha) (3-methylcrotonyl-CoA:carbon dioxide ligase subunit alpha) - Oryza sativa (Rice)nearly identical (3091) loc_os10g21910 12010.m65459 protein acetyl-coenzyme A carboxylase ACC1A, putative, expressed   no original description 	T
35.1	not assigned.no ontology	MDP0000186455	 highly similar to ( 833) AT3G06550 | Symbols:  | FUNCTIONS IN: molecular_function unknown; INVOLVED IN: biological_process unknown; LOCATED IN: endomembrane system; EXPRESSED IN: cultured cell; CONTAINS InterPro DOMAIN/s: Cas1p-like (InterPro:IPR012419); BEST Arabidopsis thaliana protein match is: O-acetyltransferase family protein (TAIR:AT2G34410.2); Has 185 Blast hits to 183 proteins in 57 species: Archae - 0; Bacteria - 0; Metazoa - 97; Fungi - 25; Plants - 56; Viruses - 0; Other Eukaryotes - 7 (source: NCBI BLink). | chr3:2039317-2042664 REVERSEhighly similar to ( 766) loc_os05g50510 12005.m09120 protein O-acetyltransferase, putative, expressed  no original description 	T
35.2	not assigned.unknown	MDP0000869126	 weakly similar to ( 120) AT5G16250 | Symbols:  | unknown protein | chr5:5310264-5310815 REVERSEvery weakly similar to (77.4) loc_os04g41900 12004.m09164 protein expressed protein   no original description 	T
35.2	not assigned.unknown	MDP0000300295	 very weakly similar to (94.0) AT1G13360 | Symbols:  | unknown protein | chr1:4577229-4577813 REVERSEvery weakly similar to (63.9) loc_os01g04010 12001.m07037 protein expressed protein   no original description 	T
30.2.11	signalling.receptor kinases.leucine rich repeat XI	MDP0000315498	 moderately similar to ( 446) AT3G12610 | Symbols: DRT100 | DRT100 (DNA-DAMAGE REPAIR/TOLERATION 100); nucleotide binding / protein binding | chr3:4006661-4007779 REVERSEweakly similar to ( 155) PGIP1_PHAVU Polygalacturonase inhibitor 1 precursor (Polygalacturonase-inhibiting protein) (PGIP-1) - Phaseolus vulgaris (Kidney bean) (French bean)moderately similar to ( 434) loc_os08g39550 12008.m07936 protein polygalacturonase inhibitor 2 precursor, putative, expressed   no original description 	T
2.2.2.3	major CHO metabolism.degradation.starch.glucan water dikinase	MDP0000321938	 nearly identical (1008) AT4G24450 | Symbols: GWD3, PWD, ATGWD2 | PWD (PHOSPHOGLUCAN, WATER DIKINASE); ATP binding / kinase | chr4:12635761-12642631 FORWARDhighly similar to ( 793) GWD1_SOLTU Alpha-glucan water dikinase, chloroplast precursor (EC 2.7.9.4) (EC 2.7.13.3) (Starch-related R1 protein) - Solanum tuberosum (Potato)highly similar to ( 711) loc_os06g30310 12006.m07575 protein alpha-glucan water dikinase, chloroplast precursor, putative, expressed  no original description 	T
30.2.17	signalling.receptor kinases.DUF 26	MDP0000202785	 highly similar to ( 816) AT5G16000 | Symbols: NIK1 | NIK1 (NSP-INTERACTING KINASE 1); kinase | chr5:5224264-5227003 FORWARDmoderately similar to ( 244) NORK_PEA Nodulation receptor kinase precursor (EC 2.7.11.1) - Pisum sativum (Garden pea)highly similar to ( 722) loc_os06g16330 12006.m06346 protein BRASSINOSTEROID INSENSITIVE 1-associated receptor kinase 1 precursor, putative, expressed   no original description 	T
30.2.99	signalling.receptor kinases.misc	MDP0000202785	 highly similar to ( 816) AT5G16000 | Symbols: NIK1 | NIK1 (NSP-INTERACTING KINASE 1); kinase | chr5:5224264-5227003 FORWARDmoderately similar to ( 244) NORK_PEA Nodulation receptor kinase precursor (EC 2.7.11.1) - Pisum sativum (Garden pea)highly similar to ( 722) loc_os06g16330 12006.m06346 protein BRASSINOSTEROID INSENSITIVE 1-associated receptor kinase 1 precursor, putative, expressed   no original description 	T
29.4	protein.postranslational modification	MDP0000202785	 highly similar to ( 816) AT5G16000 | Symbols: NIK1 | NIK1 (NSP-INTERACTING KINASE 1); kinase | chr5:5224264-5227003 FORWARDmoderately similar to ( 244) NORK_PEA Nodulation receptor kinase precursor (EC 2.7.11.1) - Pisum sativum (Garden pea)highly similar to ( 722) loc_os06g16330 12006.m06346 protein BRASSINOSTEROID INSENSITIVE 1-associated receptor kinase 1 precursor, putative, expressed   no original description 	T
29.4.1.57	protein.postranslational modification.kinase.receptor like cytoplasmatic kinase VII	MDP0000202785	 highly similar to ( 816) AT5G16000 | Symbols: NIK1 | NIK1 (NSP-INTERACTING KINASE 1); kinase | chr5:5224264-5227003 FORWARDmoderately similar to ( 244) NORK_PEA Nodulation receptor kinase precursor (EC 2.7.11.1) - Pisum sativum (Garden pea)highly similar to ( 722) loc_os06g16330 12006.m06346 protein BRASSINOSTEROID INSENSITIVE 1-associated receptor kinase 1 precursor, putative, expressed   no original description 	T
29.5.11.3	protein.degradation.ubiquitin.E2	MDP0000230564	 moderately similar to ( 272) AT1G16890 | Symbols: UBC36, UBC13B | ubiquitin-conjugating enzyme, putative | chr1:5776550-5778327 REVERSEweakly similar to ( 119) UBC2_WHEAT Ubiquitin-conjugating enzyme E2-17 kDa (EC 6.3.2.19) (Ubiquitin-protein ligase) (Ubiquitin carrier protein) - Triticum aestivum (Wheat)moderately similar to ( 268) loc_os01g48280 12001.m43137 protein ubiquitin-conjugating enzyme E2 N, putative, expressed no original description 	T
26.13	misc.acid and other phosphatases	MDP0000183910	 moderately similar to ( 365) AT1G73010 | Symbols:  | phosphatase | chr1:27464780-27466180 REVERSEmoderately similar to ( 311) loc_os01g52230 12001.m11397 protein phosphatase phospho1, putative, expressed  no original description 	T
29.5.5	protein.degradation.serine protease	MDP0000181372	 moderately similar to ( 303) AT5G38510 | Symbols:  | rhomboid family protein | chr5:15417839-15420002 REVERSEweakly similar to ( 166) loc_os02g22100 12002.m100193 protein rhomboid family protein, expressed  no original description 	T
29.5.11.3	protein.degradation.ubiquitin.E2	MDP0000223660	 moderately similar to ( 314) AT2G02760 | Symbols: ATUBC2, UBC2 | ATUBC2 (UBIQUITING-CONJUGATING ENZYME 2); ubiquitin-protein ligase | chr2:774271-775149 FORWARDmoderately similar to ( 314) UBC2_MEDSA Ubiquitin-conjugating enzyme E2-17 kDa (EC 6.3.2.19) (Ubiquitin-protein ligase) (Ubiquitin carrier protein) - Medicago sativa (Alfalfa)moderately similar to ( 315) loc_os07g07240 12007.m05183 protein ubiquitin-conjugating enzyme E2-17 kDa, putative, expressed no original description 	T
35.2	not assigned.unknown	MDP0000218824	 very weakly similar to (60.5) AT2G04515 | Symbols:  | unknown protein | chr2:1573721-1574311 FORWARD   no original description 	T
29.4.1.57	protein.postranslational modification.kinase.receptor like cytoplasmatic kinase VII	MDP0000887896	 highly similar to ( 886) AT5G16000 | Symbols: NIK1 | NIK1 (NSP-INTERACTING KINASE 1); kinase | chr5:5224264-5227003 FORWARDmoderately similar to ( 222) NORK_PEA Nodulation receptor kinase precursor (EC 2.7.11.1) - Pisum sativum (Garden pea)highly similar to ( 803) loc_os06g16330 12006.m06346 protein BRASSINOSTEROID INSENSITIVE 1-associated receptor kinase 1 precursor, putative, expressed   no original description 	T
17.7.1.2	hormone metabolism.jasmonate.synthesis-degradation.lipoxygenase	MDP0000312397	 nearly identical (1140) AT1G55020 | Symbols: LOX1 | LOX1; lipoxygenase | chr1:20525798-20530143 FORWARDnearly identical (1144) LOX1_SOLTU Lipoxygenase 1 (EC 1.13.11.12) - Solanum tuberosum (Potato)highly similar to ( 983) loc_os03g49380 12003.m35371 protein lipoxygenase 4, putative, expressed   no original description 	T
29.2.4	protein.synthesis.elongation	MDP0000180637	 highly similar to ( 742) AT4G02930 | Symbols:  | elongation factor Tu, putative / EF-Tu, putative | chr4:1295751-1298354 REVERSEhighly similar to ( 512) EFTUB_NICSY Elongation factor TuB, chloroplast precursor (EF-TuB) - Nicotiana sylvestris (Wood tobacco)highly similar to ( 703) loc_os03g63410 12003.m34958 protein elongation factor Tu, mitochondrial precursor, putative, expressed  no original description 	T
35.2	not assigned.unknown	MDP0000142484	 weakly similar to ( 163) AT1G67050 | Symbols:  | unknown protein | chr1:25028862-25029656 REVERSE  no original description 	T
34.13	transport.peptides and oligopeptides	MDP0000753658	 moderately similar to ( 367) AT1G22540 | Symbols:  | proton-dependent oligopeptide transport (POT) family protein | chr1:7964202-7966222 FORWARDmoderately similar to ( 273) loc_os05g35650 12005.m07797 protein peptide transporter PTR2-B, putative, expressed  no original description 	T
35.1	not assigned.no ontology	MDP0000874953	 highly similar to ( 663) AT1G08350 | Symbols:  | endomembrane protein 70 family protein | chr1:2632970-2635605 REVERSEhighly similar to ( 624) loc_os03g13380 12003.m06783 protein transmembrane 9 superfamily protein member 1 precursor, putative, expressed   no original description 	T
35.2	not assigned.unknown	MDP0000753333	 moderately similar to ( 292) AT3G04860 | Symbols:  | unknown protein | chr3:1339349-1340218 REVERSEmoderately similar to ( 313) loc_os03g59200 12003.m10820 protein expressed protein  no original description 	T
35.2	not assigned.unknown	MDP0000299955	 moderately similar to ( 405) AT5G24840 | Symbols:  | tRNA (guanine-N7-)-methyltransferase | chr5:8533897-8534652 REVERSEmoderately similar to ( 390) loc_os06g12990 12006.m06015 protein tRNA (guanine-N(1)-)-methyltransferase, putative, expressed  no original description 	T
31.4	cell.vesicle transport	MDP0000021204	 highly similar to ( 726) AT5G05010 | Symbols:  | clathrin adaptor complexes medium subunit-related | chr5:1477137-1479872 FORWARDhighly similar to ( 693) COPD_ORYSA Coatomer subunit delta (Delta-coat protein) (Delta-COP) (Archain) - Oryza sativa (Rice)highly similar to ( 716) loc_os05g24601 12005.m83866 protein coatomer subunit delta, putative, expressed  no original description 	T
35.1.5	not assigned.no ontology.pentatricopeptide (PPR) repeat-containing protein	MDP0000373251	 moderately similar to ( 465) AT1G61870 | Symbols: PPR336 | PPR336 (pentatricopeptide repeat 336) | chr1:22865326-22866552 REVERSEweakly similar to ( 111) RF1_ORYSA Rf1 protein, mitochondrial precursor (PPR protein) (Fertility restorer) (Restorer for CMS) - Oryza sativa (Rice)moderately similar to ( 335) loc_os01g58080 12001.m43225 protein membrane-associated salt-inducible protein, putative, expressed MSP1_C TrbL  no original description 	T
35.2	not assigned.unknown	MDP0000256033	 moderately similar to ( 356) AT1G09800 | Symbols:  | tRNA pseudouridine synthase family protein | chr1:3177121-3180336 REVERSEmoderately similar to ( 339) loc_os12g09290 12012.m04913 protein pseudouridylate synthase, putative, expressed  no original description 	T
26.7	misc.oxidases - copper, flavone etc	MDP0000869086	 highly similar to ( 700) AT5G07800 | Symbols:  | flavin-containing monooxygenase family protein / FMO family protein | chr5:2486717-2489241 REVERSEmoderately similar to ( 496) loc_os07g02140 12007.m04688 protein disulfide oxidoreductase/ monooxygenase/ oxidoreductase, putative, expressed  no original description 	T
35.2	not assigned.unknown	MDP0000302068	 weakly similar to ( 151) AT3G54310 | Symbols:  | unknown protein | chr3:20111153-20112944 REVERSEvery weakly similar to (65.5) loc_os06g17100 12006.m06422 protein retrotransposon protein, putative, unclassified  no original description 	T
31.4	cell.vesicle transport	MDP0000185731	 moderately similar to ( 378) AT1G08560 | Symbols: SYP111, KN, ATSYP111 | SYP111 (SYNTAXIN OF PLANTS 111); SNAP receptor | chr1:2709778-2710710 REVERSEmoderately similar to ( 347) loc_os03g52650 12003.m10239 protein syntaxin-related protein KNOLLE, putative, expressed     no original description 	T
35.2	not assigned.unknown	MDP0000766269	 moderately similar to ( 320) AT2G23360 | Symbols:  | transport protein-related | chr2:9949456-9952727 FORWARDmoderately similar to ( 303) loc_os04g55570 12004.m10448 protein expressed protein   no original description 	T
27.1.1	RNA.processing.splicing	MDP0000120033	 very weakly similar to (55.5) AT4G31580 | Symbols: SRZ-22, SRZ22, RSZP22 | SRZ-22; protein binding | chr4:15306983-15308064 FORWARD  no original description 	T
26.7	misc.oxidases - copper, flavone etc	MDP0000673779	 highly similar to ( 517) AT5G16990 | Symbols:  | NADP-dependent oxidoreductase, putative | chr5:5581831-5583849 REVERSEmoderately similar to ( 452) loc_os12g12580 12012.m05231 protein NADP-dependent oxidoreductase P2, putative, expressed no original description 	T
26.21	misc.protease inhibitor/seed storage/lipid transfer protein (LTP) family protein	MDP0000251626	 very weakly similar to ( 100) AT3G22142 | Symbols:  | structural constituent of cell wall | chr3:7803604-7808046 REVERSEvery weakly similar to (63.2) 14KD_DAUCA 14 kDa proline-rich protein DC2.15 precursor - Daucus carota (Carrot)very weakly similar to (87.4) loc_os06g07220 12006.m05445 protein 36.4 kDa proline-rich protein, putative, expressed  no original description 	T
31.1	cell.organisation	MDP0000623328	 weakly similar to ( 164) AT4G19840 | Symbols: ATPP2-A1, ATPP2A-1, PP2-A1 | ATPP2-A1; carbohydrate binding | chr4:10774336-10775701 FORWARDweakly similar to ( 158) loc_os01g06500 12001.m07280 protein lectin-like protein, putative, expressed no original description 	T
35.1	not assigned.no ontology	MDP0000242401	 moderately similar to ( 279) AT2G17570 | Symbols:  | undecaprenyl pyrophosphate synthetase family protein / UPP synthetase family protein | chr2:7645086-7645973 FORWARDvery weakly similar to (54.3) DR206_PEA Disease resistance response protein 206 - Pisum sativum (Garden pea)moderately similar to ( 213) loc_os06g07120 12006.m05436 protein dehydrodolichyl diphosphate synthase 6, putative, expressed   no original description 	T
29.2.1.2.2.6	protein.synthesis.ribosomal protein.eukaryotic.60S subunit.L6	MDP0000320323	 moderately similar to ( 323) AT1G18540 | Symbols:  | 60S ribosomal protein L6 (RPL6A) | chr1:6377448-6378548 REVERSEmoderately similar to ( 339) RL6_MESCR 60S ribosomal protein L6 (YL16-like) - Mesembryanthemum crystallinum (Common ice plant)moderately similar to ( 260) loc_os04g39700 12004.m08956 protein 60S ribosomal protein L6, putative, expressed  TrbL COG5283  no original description 	T
15.2	metal handling.binding, chelation and storage	MDP0000266039	 moderately similar to ( 382) AT2G31040 | Symbols:  | ATP synthase protein I -related | chr2:13209094-13211012 REVERSEmoderately similar to ( 303) loc_os01g48300 12001.m43139 protein expressed protein  no original description 	T
26.3	misc.gluco-, galacto- and mannosidases	MDP0000149337	 moderately similar to ( 407) AT4G21760 | Symbols: BGLU47 | BGLU47 (Beta-glucosidase 47); catalytic/ cation binding / hydrolase, hydrolyzing O-glycosyl compounds | chr4:11561229-11563871 FORWARDmoderately similar to ( 288) BGLC_MAIZE Beta-glucosidase, chloroplast precursor (EC 3.2.1.21) (Gentiobiase) (Cellobiase) (Beta-D-glucoside glucohydrolase) - Zea mays (Maize)moderately similar to ( 434) loc_os04g43360 12004.m101583 protein beta-glucosidase, chloroplast precursor, putative, expressed  no original description 	T
34.9	transport.metabolite transporters at the mitochondrial membrane	MDP0000841464	 highly similar to ( 561) AT5G24030 | Symbols: SLAH3 | SLAH3 (SLAC1 HOMOLOGUE 3); transporter | chr5:8118618-8120993 REVERSEhighly similar to ( 552) loc_os01g28840 12001.m09324 protein C4-dicarboxylate transporter/malic acid transport protein, expressed  no original description 	T
29.5.11.20	protein.degradation.ubiquitin.proteasom	MDP0000823038	 highly similar to ( 781) AT4G29040 | Symbols: RPT2a | RPT2a (regulatory particle AAA-ATPase 2a); ATPase | chr4:14312369-14314386 FORWARDhighly similar to ( 766) PRS4_ORYSA 26S protease regulatory subunit 4 homolog (TAT-binding protein homolog 2) - Oryza sativa (Rice)highly similar to ( 770) loc_os03g18690 12003.m07290 protein 26S protease regulatory subunit 4, putative, expressed  no original description 	T
35.1	not assigned.no ontology	MDP0000182259	 very weakly similar to (96.7) AT4G16380 | Symbols:  | LOCATED IN: cellular_component unknown; BEST Arabidopsis thaliana protein match is: metal ion binding (TAIR:AT1G49420.1); Has 3600 Blast hits to 3158 proteins in 308 species: Archae - 4; Bacteria - 309; Metazoa - 1646; Fungi - 490; Plants - 698; Viruses - 60; Other Eukaryotes - 393 (source: NCBI BLink). | chr4:9255148-9255955 FORWARD  no original description 	T
27.4	RNA.RNA binding	MDP0000360837	 weakly similar to ( 189) AT2G37510 | Symbols:  | RNA-binding protein, putative | chr2:15743112-15744504 REVERSEvery weakly similar to (75.9) GRP2_SORBI Glycine-rich RNA-binding protein 2 - Sorghum bicolor (Sorghum) (Sorghum vulgare)weakly similar to ( 172) loc_os03g17060 12003.m07138 protein RNA binding protein, putative, expressed  no original description 	T
35.1	not assigned.no ontology	MDP0000806945	 moderately similar to ( 435) AT1G30910 | Symbols:  | molybdenum cofactor sulfurase family protein | chr1:11000912-11002801 FORWARDweakly similar to ( 125) MOCOS_ORYSA Molybdenum cofactor sulfurase (EC 4.4.-.-) (MoCo sulfurase) (MOS) - Oryza sativa (Rice)moderately similar to ( 421) loc_os09g38772 12009.m06818 protein mo-molybdopterin cofactor sulfurase, putative, expressed   no original description 	T
26.28	misc.GDSL-motif lipase	MDP0000319412	 highly similar to ( 502) AT1G33811 | Symbols:  | GDSL-motif lipase/hydrolase family protein | chr1:12267918-12269690 FORWARDweakly similar to ( 157) APG_BRANA Anter-specific proline-rich protein APG (Protein CEX) (Fragment) - Brassica napus (Rape)moderately similar to ( 345) loc_os01g54470 12001.m11611 protein anther-specific proline-rich protein APG, putative, expressed  no original description 	T
31.1	cell.organisation	MDP0000808579	 moderately similar to ( 414) AT3G61710 | Symbols:  | autophagy protein Apg6 family | chr3:22840211-22841815 REVERSEmoderately similar to ( 377) loc_os01g48920 12001.m43146 protein beclin-1-like protein, putative, expressed MSP1_C  no original description 	T
29.5.11.20	protein.degradation.ubiquitin.proteasom	MDP0000265787	 weakly similar to ( 161) AT1G67250 | Symbols:  | proteasome maturation factor UMP1 family protein | chr1:25163808-25164967 REVERSEvery weakly similar to (99.4) loc_os02g55640 12002.m10589 protein proteasome maturation factor UMP1 family protein, expressed no original description 	T
29.2.1.2.2.15	protein.synthesis.ribosomal protein.eukaryotic.60S subunit.L15	MDP0000436496	 moderately similar to ( 359) AT4G16720 | Symbols:  | 60S ribosomal protein L15 (RPL15A) | chr4:9400156-9401315 REVERSEmoderately similar to ( 355) RL15_PETHY 60S ribosomal protein L15 - Petunia hybrida (Petunia)moderately similar to ( 358) loc_os05g19370 12005.m06340 protein 60S ribosomal protein L15, putative, expressed   no original description 	T
35.2	not assigned.unknown	MDP0000250605	 moderately similar to ( 487) AT1G49890 | Symbols:  | unknown protein | chr1:18470282-18473463 FORWARDmoderately similar to ( 289) loc_os03g10820 12003.m06534 protein expressed protein   no original description 	T
26.2	misc.UDP glucosyl and glucoronyl transferases	MDP0000580411	 moderately similar to ( 306) AT5G49690 | Symbols:  | UDP-glucoronosyl/UDP-glucosyl transferase family protein | chr5:20189968-20191350 REVERSEmoderately similar to ( 211) UFOG_PETHY Anthocyanidin 3-O-glucosyltransferase (EC 2.4.1.115) (Flavonol 3-O-glucosyltransferase) (UDP-glucose flavonoid 3-O-glucosyltransferase) (Anthocyanin rhamnosyl transferase) - Petunia hybrida (Petunia)moderately similar to ( 301) loc_os01g64540 12001.m12568 protein anthocyanidin 3-O-glucosyltransferase, putative, expressed  no original description 	T
30.3	signalling.calcium	MDP0000728603	 moderately similar to ( 355) AT1G09210 | Symbols:  | calreticulin 2 (CRT2) | chr1:2973217-2976655 REVERSEmoderately similar to ( 369) CALR_BETVU Calreticulin precursor - Beta vulgaris (Sugar beet)moderately similar to ( 337) loc_os07g14270 12007.m29118 protein calreticulin precursor, putative, expressed no original description 	T
6.5	gluconeogenesis / glyoxylate cycle.pyruvate dikinase	MDP0000244014	 nearly identical (1268) AT4G15530 | Symbols: PPDK | PPDK (pyruvate orthophosphate dikinase); kinase/ pyruvate, phosphate dikinase | chr4:8864828-8870748 REVERSEnearly identical (1315) PPDK_MESCR Pyruvate, phosphate dikinase, chloroplast precursor (EC 2.7.9.1) (Pyruvate, orthophosphate dikinase) - Mesembryanthemum crystallinum (Common ice plant)nearly identical (1249) loc_os05g33570 12005.m083718 protein pyruvate, phosphate dikinase, chloroplast precursor, putative, expressed    no original description 	T
34.13	transport.peptides and oligopeptides	MDP0000174770	 weakly similar to ( 127) AT3G54140 | Symbols: ATPTR1, PTR1 | PTR1 (PEPTIDE TRANSPORTER 1); dipeptide transporter/ transporter/ tripeptide transporter | chr3:20045885-20048154 REVERSEweakly similar to ( 125) loc_os01g04950 12001.m42857 protein peptide transporter PTR2, putative, expressed no original description 	T
30.2.11	signalling.receptor kinases.leucine rich repeat XI	MDP0000186555	 moderately similar to ( 372) AT2G19780 | Symbols:  | leucine-rich repeat family protein / extensin family protein | chr2:8522831-8524039 REVERSEweakly similar to ( 120) RPK1_IPONI Receptor-like protein kinase precursor (EC 2.7.11.1) - Ipomoea nil (Japanese morning glory) (Pharbitis nil)moderately similar to ( 303) loc_os05g09640 12005.m05477 protein BRASSINOSTEROID INSENSITIVE 1 precursor, putative, expressed   no original description 	T
26.1	misc.misc2	MDP0000157089	 moderately similar to ( 204) AT4G15955 | Symbols:  | epoxide hydrolase-related | chr4:9043872-9045544 REVERSEweakly similar to ( 189) loc_os03g61340 12003.m11017 protein epoxide hydrolase 2, putative, expressed  no original description 	T
27.3.6	RNA.regulation of transcription.bHLH,Basic Helix-Loop-Helix family	MDP0000853274	 weakly similar to ( 140) AT1G09250 | Symbols:  | transcription factor | chr1:2989509-2990132 FORWARDweakly similar to ( 116) loc_os03g19780 12003.m07393 protein expressed protein    no original description 	T
29.2.4	protein.synthesis.elongation	MDP0000470429	 highly similar to ( 852) AT5G60390 | Symbols:  | elongation factor 1-alpha / EF-1-alpha | chr5:24289226-24290675 FORWARDhighly similar to ( 858) EF1A_MAIZE Elongation factor 1-alpha (EF-1-alpha) - Zea mays (Maize)highly similar to ( 857) loc_os03g08050 12003.m78808 protein elongation factor 1-alpha, putative, expressed   no original description 	T
30.2.17	signalling.receptor kinases.DUF 26	MDP0000400679	 weakly similar to ( 147) AT1G70250 | Symbols:  | receptor serine/threonine kinase, putative | chr1:26452975-26456088 FORWARDvery weakly similar to (63.5) CRI4_MAIZE Putative receptor protein kinase CRINKLY4 precursor (EC 2.7.11.1) - Zea mays (Maize)weakly similar to ( 159) loc_os01g02400 12001.m06882 protein Ser/Thr receptor-like kinase, putative, expressed  no original description 	T
11.6	lipid metabolism.lipid transfer proteins etc	MDP0000039359	 weakly similar to ( 124) AT2G38540 | Symbols: LP1, LTP1, ATLTP1 | LP1; calmodulin binding | chr2:16130418-16130893 FORWARDweakly similar to ( 141) NLTP2_GOSHI Nonspecific lipid-transfer protein precursor (LTP) (GH3) - Gossypium hirsutum (Upland cotton)weakly similar to ( 117) loc_os12g02330 12012.m04234 protein nonspecific lipid-transfer protein precursor, putative, expressed no original description 	T
27.3.21	RNA.regulation of transcription.GRAS transcription factor family	MDP0000205236	 highly similar to ( 579) AT1G50600 | Symbols: SCL5 | SCL5; transcription factor | chr1:18737398-18739547 REVERSEhighly similar to ( 552) CIGR2_ORYSA Chitin-inducible gibberellin-responsive protein 2 - Oryza sativa (Rice)highly similar to ( 570) loc_os10g22430 12010.m05252 protein chitin-inducible gibberellin-responsive protein 2, putative, expressed   no original description 	T
29.2.4	protein.synthesis.elongation	MDP0000922511	 moderately similar to ( 291) AT1G57720 | Symbols:  | elongation factor 1B-gamma, putative / eEF-1B gamma, putative | chr1:21377873-21380114 FORWARDmoderately similar to ( 291) EF1G3_ORYSA Elongation factor 1-gamma 3 (EF-1-gamma 3) (eEF-1B gamma 3) - Oryza sativa (Rice)moderately similar to ( 291) loc_os06g37440 12006.m08279 protein elongation factor 1-gamma 3, putative, expressed no original description 	T
10.7	cell wall.modification	MDP0000435467	 weakly similar to ( 170) AT4G28250 | Symbols: ATEXPB3, EXPB3, ATHEXP BETA 1.6 | ATEXPB3 (ARABIDOPSIS THALIANA EXPANSIN B3) | chr4:14000446-14001945 REVERSEweakly similar to ( 161) EXB17_ORYSA Expansin-B17 precursor (OsEXPB17) (Beta-expansin-17) (OsaEXPb1.13) - Oryza sativa (Rice)weakly similar to ( 160) loc_os02g42650 12002.m09300 protein beta-expansin 3 precursor, putative, expressed   no original description 	T
17.6.3	hormone metabolism.gibberelin.induced-regulated-responsive-activated	MDP0000126347	 weakly similar to ( 139) AT3G10185 | Symbols:  | Encodes a Gibberellin-regulated GASA/GAST/Snakin family protein | chr3:3145579-3146199 FORWARDweakly similar to ( 148) loc_os05g31280 12005.m07361 protein GAST1 protein precursor, putative, expressed  no original description 	T
35.2	not assigned.unknown	MDP0000222576	 very weakly similar to (96.7) AT5G02020 | Symbols:  | unknown protein | chr5:386999-387921 REVERSE no original description 	T
29.3.4.3	protein.targeting.secretory pathway.vacuole	MDP0000912183	 highly similar to ( 883) AT3G54860 | Symbols: ATVPS33, VPS33 | vacuolar protein sorting protein, putative | chr3:20324286-20329841 REVERSEhighly similar to ( 817) loc_os04g14654 12004.m35255 protein vacuolar protein-sorting protein 33, putative, expressed  no original description 	T
31.2.5	cell.division.plastid	MDP0000666598	 moderately similar to ( 272) AT5G53280 | Symbols: PDV1 | PDV1 (PLASTID DIVISION1) | chr5:21607586-21609080 FORWARDmoderately similar to ( 214) loc_os02g16450 12002.m06888 protein expressed protein   no original description 	T
35.2	not assigned.unknown	MDP0000563232	 moderately similar to ( 499) AT5G15900 | Symbols:  | unknown protein | chr5:5189524-5192070 REVERSEmoderately similar to ( 400) loc_os06g16170 12006.m06330 protein expressed protein  no original description 	T
27.4	RNA.RNA binding	MDP0000841125	 highly similar to ( 513) AT5G61960 | Symbols: AML1 | AML1 (ARABIDOPSIS MEI2-LIKE PROTEIN 1); RNA binding / protein binding | chr5:24879001-24883483 REVERSEweakly similar to ( 126) PLA2_ORYSA Protein terminal ear1 homolog (Protein PLASTOCHRON2) (Protein LEAFY HEAD2) - Oryza sativa (Rice)moderately similar to ( 488) loc_os02g31290 12002.m33391 protein mei2-like protein, putative, expressed no original description 	T
29.2.1.2.2.18	protein.synthesis.ribosomal protein.eukaryotic.60S subunit.L18	MDP0000231958	 moderately similar to ( 337) AT3G05590 | Symbols: RPL18 | RPL18 (RIBOSOMAL PROTEIN L18); structural constituent of ribosome | chr3:1621511-1622775 FORWARDmoderately similar to ( 317) RL18_CICAR 60S ribosomal protein L18 (Fragment) - Cicer arietinum (Chickpea) (Garbanzo)moderately similar to ( 323) loc_os03g22180 12003.m07621 protein 60S ribosomal protein L18, putative, expressed   no original description 	T
34.99	transport.misc	MDP0000128682	 nearly identical (1377) AT1G72560 | Symbols: PSD | PSD (PAUSED); nucleobase, nucleoside, nucleotide and nucleic acid transmembrane transporter/ tRNA binding | chr1:27325026-27329467 REVERSEhighly similar to ( 916) loc_os07g42180 12007.m08454 protein PAUSED, putative, expressed  no original description 	T
29.2.1.2.2.31	protein.synthesis.ribosomal protein.eukaryotic.60S subunit.L31	MDP0000156683	 weakly similar to ( 134) AT2G19740 | Symbols:  | 60S ribosomal protein L31 (RPL31A) | chr2:8513577-8514346 FORWARDweakly similar to ( 125) RL31_PANGI 60S ribosomal protein L31 - Panax ginseng (Korean ginseng)weakly similar to ( 129) loc_os02g48660 12002.m09900 protein 60S ribosomal protein L31, putative, expressed  no original description 	T
16.1.1.6	secondary metabolism.isoprenoids.non-mevalonate pathway.HDS	MDP0000704997	 moderately similar to ( 332) AT5G60600 | Symbols: GCPE, ISPG, CSB3, CLB4, HDS | HDS (4-HYDROXY-3-METHYLBUT-2-ENYL DIPHOSPHATE SYNTHASE); 4 iron, 4 sulfur cluster binding / 4-hydroxy-3-methylbut-2-en-1-yl diphosphate synthase | chr5:24359447-24363019 FORWARDmoderately similar to ( 329) loc_os02g39160 12002.m09004 protein hydroxymethylbutenyl 4-diphosphate synthase, putative, expressed no original description 	T
20.2.3	stress.abiotic.drought/salt	MDP0000518147	 weakly similar to ( 153) AT2G41430 | Symbols: ERD15, LSR1, CID1 | ERD15 (EARLY RESPONSIVE TO DEHYDRATION 15); protein binding | chr2:17269736-17270314 FORWARDvery weakly similar to (92.8) loc_os03g23010 12003.m35608 protein expressed protein  no original description 	T
34.8	transport.metabolite transporters at the envelope membrane	MDP0000224440	 highly similar to ( 562) AT3G17430 | Symbols:  | phosphate translocator-related | chr3:5966597-5968962 FORWARDvery weakly similar to (58.9) TPT_FLAPR Triose phosphate/phosphate translocator, chloroplast precursor (cTPT) - Flaveria pringleihighly similar to ( 530) loc_os03g49570 12003.m09957 protein organic anion transporter, putative, expressed no original description 	T
20.1.7	stress.biotic.PR-proteins	MDP0000685425	 moderately similar to ( 387) AT5G17680 | Symbols:  | disease resistance protein (TIR-NBS-LRR class), putative | chr5:5822999-5827153 FORWARDweakly similar to ( 101) loc_os12g37760 12012.m07562 protein RGH1A, putative, expressed   no original description 	T
34.3	transport.amino acids	MDP0000280551	 nearly identical (1324) AT1G15690 | Symbols: AVP1, ATAVP3, AVP-3 | AVP1; ATPase/ hydrogen-translocating pyrophosphatase | chr1:5399115-5402185 FORWARDnearly identical (1330) AVP_PHAAU Pyrophosphate-energized vacuolar membrane proton pump (EC 3.6.1.1) (Pyrophosphate-energized inorganic pyrophosphatase) (H(+)-PPase) (Vacuolar H(+)-pyrophosphatase) - Phaseolus aureus (Mung bean) (Vigna radiata)nearly identical (1320) loc_os02g09150 12002.m100123 protein pyrophosphate-energized vacuolar membrane proton pump, putative, expressed   no original description 	T
30.2.11	signalling.receptor kinases.leucine rich repeat XI	MDP0000219538	 moderately similar to ( 288) AT1G72300 | Symbols:  | leucine-rich repeat transmembrane protein kinase, putative | chr1:27217679-27220966 REVERSEmoderately similar to ( 234) PSKR_DAUCA Phytosulfokine receptor precursor (EC 2.7.11.1) (Phytosulfokine LRR receptor kinase) - Daucus carota (Carrot)moderately similar to ( 278) loc_os02g05930 12002.m05941 protein phytosulfokine receptor precursor, putative, expressed  no original description 	T
34.1	transport.p- and v-ATPases	MDP0000815058	 moderately similar to ( 464) AT5G44240 | Symbols:  | haloacid dehalogenase-like hydrolase family protein | chr5:17817619-17823598 FORWARDmoderately similar to ( 357) loc_os11g25980 12011.m06519 protein phospholipid-transporting ATPase 2, putative, expressed  no original description 	T
29.5.1	protein.degradation.subtilases	MDP0000909991	 nearly identical (1192) AT3G14240 | Symbols:  | subtilase family protein | chr3:4741637-4743964 REVERSEnearly identical (1028) loc_os08g35090 12008.m07498 protein subtilisin-like protease precursor, putative, expressed   no original description 	T
30.11	signalling.light	MDP0000269627	 moderately similar to ( 470) AT3G10970 | Symbols:  | haloacid dehalogenase-like hydrolase family protein | chr3:3433276-3436077 REVERSEmoderately similar to ( 426) loc_os05g07632 12005.m27473 protein catalytic/ hydrolase, putative, expressed   no original description 	T
16.2	secondary metabolism.phenylpropanoids	MDP0000672454	 highly similar to ( 691) AT1G77670 | Symbols:  | aminotransferase class I and II family protein | chr1:29189043-29190901 REVERSEvery weakly similar to (71.6) 1A11_ORYSA 1-aminocyclopropane-1-carboxylate synthase 1 (EC 4.4.1.14) (ACC synthase 1) (S-adenosyl-L-methionine methylthioadenosine-lyase 1) - Oryza sativa (Rice)highly similar to ( 675) loc_os09g28050 12009.m22116 protein asparate aminotransferase, putative, expressed  no original description 	T
35.2	not assigned.unknown	MDP0000144618	 moderately similar to ( 232) AT4G30240 | Symbols:  | protein binding | chr4:14808359-14809745 REVERSEweakly similar to ( 141) loc_os08g14640 12008.m26634 protein expressed protein  no original description 	T
35.2	not assigned.unknown	MDP0000360394	 very weakly similar to (97.8) MAL12_MALDO Major allergen Mal d 1 (Mal d I) (AP15) - Malus domestica (Apple) (Malus sylvestris) no original description 	T
29.4	protein.postranslational modification	MDP0000152707	 highly similar to ( 932) AT1G53050 | Symbols:  | protein kinase family protein | chr1:19772574-19775531 FORWARDmoderately similar to ( 222) CDC2_VIGUN Cell division control protein 2 homolog (EC 2.7.11.22) (EC 2.7.11.23) (p34cdc2) - Vigna unguiculata (Cowpea)highly similar to ( 766) loc_os01g10430 12001.m42914 protein transposon protein, putative, unclassified, expressed   no original description 	T
29.4	protein.postranslational modification	MDP0000275922	 highly similar to ( 848) AT5G20930 | Symbols: TSL | TSL (TOUSLED); kinase/ protein serine/threonine kinase | chr5:7098213-7102970 FORWARDvery weakly similar to (98.6) CRK_DAUCA CDPK-related protein kinase (EC 2.7.11.1) (PK421) - Daucus carota (Carrot)highly similar to ( 860) loc_os03g53880 12003.m35581 protein tousled-like kinase 2, putative, expressed  no original description 	T
10.6.3	cell wall.degradation.pectate lyases and polygalacturonases	MDP0000150610	 highly similar to ( 644) AT3G07010 | Symbols:  | pectate lyase family protein | chr3:2212973-2216306 REVERSEmoderately similar to ( 417) PEL_LILLO Pectate lyase precursor (EC 4.2.2.2) - Lilium longiflorum (Trumpet lily)highly similar to ( 627) loc_os04g05050 12004.m35243 protein pectate lyase 8 precursor, putative, expressed  no original description 	T
29.4	protein.postranslational modification	MDP0000201061	 weakly similar to ( 153) AT5G50200 | Symbols: WR3, ATNRT3.1, NRT3.1 | WR3 (WOUND-RESPONSIVE 3); nitrate transmembrane transporter | chr5:20436612-20437535 FORWARDweakly similar to ( 138) loc_os02g38230 12002.m08911 protein component of high affinity nitrate transporter, putative, expressed no original description 	T
34.4	transport.nitrate	MDP0000201061	 weakly similar to ( 153) AT5G50200 | Symbols: WR3, ATNRT3.1, NRT3.1 | WR3 (WOUND-RESPONSIVE 3); nitrate transmembrane transporter | chr5:20436612-20437535 FORWARDweakly similar to ( 138) loc_os02g38230 12002.m08911 protein component of high affinity nitrate transporter, putative, expressed no original description 	T
30.3	signalling.calcium	MDP0000855506	 very weakly similar to (94.4) AT5G54490 | Symbols: PBP1 | PBP1 (PINOID-BINDING PROTEIN 1); calcium ion binding / protein binding | chr5:22121458-22121841 FORWARDvery weakly similar to (91.3) loc_os06g46950 12006.m09221 protein EF-hand Ca2+-binding protein CCD1, putative, expressed no original description 	T
30.5	signalling.G-proteins	MDP0000889408	 nearly identical (2409) AT3G43300 | Symbols: ATMIN7 | ATMIN7 (ARABIDOPSIS THALIANA HOPM INTERACTOR 7); guanyl-nucleotide exchange factor/ protein binding | chr3:15234235-15245034 REVERSEnearly identical (2159) loc_os07g37750 12007.m08025 protein guanyl-nucleotide exchange factor, putative, expressed   no original description 	T
35.2	not assigned.unknown	MDP0000244530	 moderately similar to ( 231) AT5G12470 | Symbols:  | unknown protein | chr5:4044950-4047290 REVERSEmoderately similar to ( 290) loc_os01g72710 12001.m13305 protein expressed protein   no original description 	T
4.3.12	glycolysis.unclear/dually targeted.phosphoglycerate mutase	MDP0000302731	 moderately similar to ( 457) AT1G22170 | Symbols:  | phosphoglycerate/bisphosphoglycerate mutase family protein | chr1:7826603-7828055 FORWARDmoderately similar to ( 447) loc_os02g51590 12002.m33276 protein 2,3-bisphosphoglycerate-dependent phosphoglycerate mutase, putative, expressed  no original description 	T
29.4	protein.postranslational modification	MDP0000928643	 highly similar to ( 542) AT1G20930 | Symbols: CDKB2;2 | CDKB2;2 (CYCLIN-DEPENDENT KINASE B2;2); cyclin-dependent protein kinase/ kinase | chr1:7292752-7294664 REVERSEhighly similar to ( 529) CDC2D_ANTMA Cell division control protein 2 homolog D (EC 2.7.11.22) (EC 2.7.11.23) - Antirrhinum majus (Garden snapdragon)moderately similar to ( 494) loc_os08g40170 12008.m07997 protein cell division control protein 2 homolog D, putative, expressed    no original description 	T
34.1	transport.p- and v-ATPases	MDP0000254558	 weakly similar to ( 193) AT3G42640 | Symbols: AHA8 | AHA8 (Arabidopsis H(+)-ATPase 8); ATPase | chr3:14724309-14728062 FORWARDweakly similar to ( 198) PMA1_ORYSA Plasma membrane ATPase (EC 3.6.3.6) (Proton pump) - Oryza sativa (Rice)weakly similar to ( 198) loc_os04g56160 12004.m101441 protein plasma membrane ATPase, putative, expressed   no original description 	T
35.2	not assigned.unknown	MDP0000206154	 weakly similar to ( 145) AT1G31200 | Symbols: ATPP2-A9 | ATPP2-A9 (Phloem protein 2-A9); carbohydrate binding | chr1:11146923-11147678 REVERSEvery weakly similar to (55.1) loc_os01g06500 12001.m07280 protein lectin-like protein, putative, expressed no original description 	T
35.2	not assigned.unknown	MDP0000150032	 weakly similar to ( 153) AT4G13220 | Symbols:  | unknown protein | chr4:7674431-7675275 REVERSEweakly similar to ( 136) loc_os12g17830 12012.m073811 protein expressed protein  no original description 	T
33.99	development.unspecified	MDP0000451154	 weakly similar to ( 131) AT1G32400 | Symbols: TOM2A | TOM2A (TOBAMOVIRUS MULTIPLICATION 2A); protein binding | chr1:11689393-11690873 REVERSEweakly similar to ( 106) loc_os08g18044 12008.m05923 protein tetraspanin family protein, expressed   no original description 	T
27.3.40	RNA.regulation of transcription.Aux/IAA family	MDP0000255223	 moderately similar to ( 220) AT3G16500 | Symbols: PAP1, IAA26 | PAP1 (PHYTOCHROME-ASSOCIATED PROTEIN 1); transcription factor | chr3:5612801-5614208 REVERSEweakly similar to ( 193) IAA7_ORYSA Auxin-responsive protein IAA7 (Indoleacetic acid-induced protein 7) - Oryza sativa (Rice)weakly similar to ( 193) loc_os02g13520 12002.m06598 protein OsIAA7 - Auxin-responsive Aux/IAA gene family member, expressed  no original description 	T
8.2.9	TCA / org transformation.other organic acid transformatons.cyt MDH	MDP0000744301	 very weakly similar to (94.7) AT5G58330 | Symbols:  | malate dehydrogenase (NADP), chloroplast, putative | chr5:23580010-23582287 REVERSEweakly similar to ( 108) MDHP_PEA Malate dehydrogenase [NADP], chloroplast precursor (EC 1.1.1.82) (NADP-MDH) - Pisum sativum (Garden pea)very weakly similar to (83.2) loc_os08g44810 12008.m08453 protein malate dehydrogenase 1, chloroplast precursor, putative, expressed no original description 	T
35.2	not assigned.unknown	MDP0000230223	 nearly identical (1570) AT3G23590 | Symbols: RFR1 | RFR1 (REF4-related 1) | chr3:8467638-8473469 FORWARDnearly identical (1225) loc_os07g11000 12007.m05555 protein expressed protein   no original description 	T
35.2	not assigned.unknown	MDP0000460644	 weakly similar to ( 107) AT3G06035 | Symbols:  | unknown protein | chr3:1823172-1824110 REVERSEvery weakly similar to (98.6) loc_os07g45060 12007.m08726 protein GPI-anchored protein At5g19240 precursor, putative, expressed  no original description 	T
20.1	stress.biotic	MDP0000166759	 moderately similar to ( 374) AT3G14470 | Symbols:  | disease resistance protein (NBS-LRR class), putative | chr3:4857940-4861104 FORWARDmoderately similar to ( 416) loc_os05g41290 12005.m08304 protein disease resistance RPP13-like protein 1, putative, expressed  no original description 	T
29.2.1.2.2.30	protein.synthesis.ribosomal protein.eukaryotic.60S subunit.L30	MDP0000783562	 weakly similar to ( 135) AT1G77940 | Symbols:  | 60S ribosomal protein L30 (RPL30B) | chr1:29304116-29305288 REVERSEweakly similar to ( 144) RL30_LUPLU 60S ribosomal protein L30 - Lupinus luteus (European yellow lupin)weakly similar to ( 129) loc_os01g16890 12001.m42968 protein 60S ribosomal protein L30, putative, expressed no original description 	T
20.2.1	stress.abiotic.heat	MDP0000449304	 weakly similar to ( 149) AT5G23240 | Symbols:  | DNAJ heat shock N-terminal domain-containing protein | chr5:7826857-7828534 REVERSEweakly similar to ( 191) loc_os06g28050 12006.m32226 protein 3Fe-4S ferredoxin, putative, expressed  no original description 	T
23.5.4	nucleotide metabolism.deoxynucleotide metabolism.ribonucleoside-diphosphate reductase	MDP0000230224	 highly similar to ( 558) AT3G23580 | Symbols: RNR2, RNR2A | RNR2A (RIBONUCLEOTIDE REDUCTASE 2A); ribonucleoside-diphosphate reductase | chr3:8460261-8462574 FORWARDhighly similar to ( 508) RIR2_TOBAC Ribonucleoside-diphosphate reductase small chain (EC 1.17.4.1) (Ribonucleotide reductase small subunit) (Ribonucleoside-diphosphate reductase R2 subunit) - Nicotiana tabacum (Common tobacco)moderately similar to ( 497) loc_os06g14620 12006.m06177 protein ribonucleoside-diphosphate reductase small chain, putative, expressed no original description 	T
29.2.1.2.2.6	protein.synthesis.ribosomal protein.eukaryotic.60S subunit.L6	MDP0000246810	 moderately similar to ( 319) AT1G18540 | Symbols:  | 60S ribosomal protein L6 (RPL6A) | chr1:6377448-6378548 REVERSEmoderately similar to ( 325) RL6_MESCR 60S ribosomal protein L6 (YL16-like) - Mesembryanthemum crystallinum (Common ice plant)moderately similar to ( 254) loc_os04g39700 12004.m08956 protein 60S ribosomal protein L6, putative, expressed  TrbL  no original description 	T
1.1.1.1	PS.lightreaction.photosystem II.LHC-II	MDP0000417927	 moderately similar to ( 474) AT2G34430 | Symbols: LHB1B1, LHCB1.4 | LHB1B1; chlorophyll binding | chr2:14524818-14525618 FORWARDmoderately similar to ( 499) CB23_SOYBN Chlorophyll a-b binding protein 3, chloroplast precursor (LHCII type I CAB-3) (LHCP) - Glycine max (Soybean)moderately similar to ( 471) loc_os01g41710 12001.m42672 protein chlorophyll a-b binding protein 2, chloroplast precursor, putative, expressed  no original description 	T
31.1	cell.organisation	MDP0000372527	 moderately similar to ( 224) AT4G19840 | Symbols: ATPP2-A1, ATPP2A-1, PP2-A1 | ATPP2-A1; carbohydrate binding | chr4:10774336-10775701 FORWARDweakly similar to ( 194) loc_os01g06500 12001.m07280 protein lectin-like protein, putative, expressed  no original description 	T
11.1.7	lipid metabolism.FA synthesis and FA elongation.ACP thioesterase	MDP0000200228	 moderately similar to ( 471) AT3G25110 | Symbols: AtFaTA | AtFaTA (Arabidopsis FatA acyl-ACP thioesterase); acyl carrier/ acyl-[acyl-carrier-protein] hydrolase | chr3:9146589-9148273 REVERSEmoderately similar to ( 236) FATB_GOSHI Myristoyl-acyl carrier protein thioesterase, chloroplast precursor (EC 3.1.2.-) (16:0-acyl-carrier protein thioesterase) (16:0-ACP thioesterase) (Acyl-[acyl-carrier-protein] hydrolase) (PATE) - Gossypium hirsutum (Upland cotton)moderately similar to ( 454) loc_os09g32760 12009.m060080 protein oleoyl-acyl carrier protein thioesterase, chloroplast precursor, putative, expressed no original description 	T
13.2.3.1.1	amino acid metabolism.degradation.aspartate family.asparagine.L-asparaginase	MDP0000658649	 moderately similar to ( 441) AT5G08100 | Symbols:  | L-asparaginase / L-asparagine amidohydrolase | chr5:2593242-2594586 REVERSEmoderately similar to ( 434) ASPG_LUPLU L-asparaginase precursor (EC 3.5.1.1) (L-asparagine amidohydrolase) [Contains: L-asparaginase subunit alpha; L-asparaginase subunit beta] - Lupinus luteus (European yellow lupin)moderately similar to ( 386) loc_os03g40070 12003.m09089 protein transposon protein, putative, unclassified, expressed no original description 	T
26.3.1	misc.gluco-, galacto- and mannosidases.alpha-galactosidase	MDP0000144090	 nearly identical (1355) AT5G63840 | Symbols: RSW3 | RSW3 (RADIAL SWELLING 3); glucosidase/ hydrolase, hydrolyzing O-glycosyl compounds | chr5:25545056-25548922 FORWARDmoderately similar to ( 276) AGLU_BETVU Alpha-glucosidase precursor (EC 3.2.1.20) (Maltase) - Beta vulgaris (Sugar beet)nearly identical (1159) loc_os03g11720 12003.m06624 protein neutral alpha-glucosidase AB precursor, putative, expressed  no original description 	T
35.1	not assigned.no ontology	MDP0000308625	 moderately similar to ( 395) AT1G80710 | Symbols:  | transducin family protein / WD-40 repeat family protein | chr1:30333499-30335796 REVERSEmoderately similar to ( 287) loc_os08g18150 12008.m05934 protein expressed protein  no original description 	T
35.2	not assigned.unknown	MDP0000317457	 very weakly similar to (60.8) loc_os09g28500 12009.m06001 protein EF hand family protein, expressed no original description 	T
34.9	transport.metabolite transporters at the mitochondrial membrane	MDP0000878094	 very weakly similar to (93.6) AT1G64220 | Symbols: TOM7-2 | TOM7-2 (TRANSLOCASE OF OUTER MEMBRANE 7 KDA SUBUNIT 2); P-P-bond-hydrolysis-driven protein transmembrane transporter | chr1:23833088-23833321 REVERSEvery weakly similar to (90.9) TOM7A_SOLTU Mitochondrial import receptor subunit TOM7-1 (Translocase of outer membrane 7 kDa subunit 1) - Solanum tuberosum (Potato)very weakly similar to (75.9) loc_os05g50654 12005.m83904 protein mitochondrial import receptor subunit TOM7-1, putative, expressed no original description 	T
1.1.1.1	PS.lightreaction.photosystem II.LHC-II	MDP0000337585	 moderately similar to ( 389) AT1G19150 | Symbols: LHCA6, LHCA2*1 | LHCA6; chlorophyll binding | chr1:6612806-6613799 FORWARDmoderately similar to ( 313) CB12_PETHY Chlorophyll a-b binding protein, chloroplast precursor (LHCI type II CAB) - Petunia hybrida (Petunia)moderately similar to ( 375) loc_os09g26810 12009.m05833 protein chlorophyll a-b binding protein, chloroplast precursor, putative, expressed  no original description 	T
27.3.21	RNA.regulation of transcription.GRAS transcription factor family	MDP0000165587	 highly similar to ( 568) AT4G37650 | Symbols: SHR, SGR7 | SHR (SHORT ROOT); protein binding / sequence-specific DNA binding / transcription factor | chr4:17691871-17693466 FORWARDweakly similar to ( 186) CIGR2_ORYSA Chitin-inducible gibberellin-responsive protein 2 - Oryza sativa (Rice)moderately similar to ( 460) loc_os03g31880 12003.m08434 protein SHR, putative, expressed   no original description 	T
35.2	not assigned.unknown	MDP0000265505	   no original description 	T
26.2	misc.UDP glucosyl and glucoronyl transferases	MDP0000191048	 moderately similar to ( 374) AT5G12890 | Symbols:  | UDP-glucoronosyl/UDP-glucosyl transferase family protein | chr5:4069658-4071124 REVERSEweakly similar to ( 179) ZOX_PHAVU Zeatin O-xylosyltransferase (EC 2.4.2.40) (Zeatin O-beta-D-xylosyltransferase) - Phaseolus vulgaris (Kidney bean) (French bean)moderately similar to ( 341) loc_os04g42690 12004.m09234 protein cytokinin-O-glucosyltransferase 3, putative, expressed TrbL Sporozoite_P67  no original description 	T
35.2	not assigned.unknown	MDP0000329773	 moderately similar to ( 266) AT3G23760 | Symbols:  | FUNCTIONS IN: molecular_function unknown; INVOLVED IN: biological_process unknown; LOCATED IN: vacuole; EXPRESSED IN: 20 plant structures; EXPRESSED DURING: 13 growth stages; BEST Arabidopsis thaliana protein match is: transferase, transferring glycosyl groups (TAIR:AT4G14100.1); Has 63 Blast hits to 60 proteins in 11 species: Archae - 0; Bacteria - 0; Metazoa - 10; Fungi - 0; Plants - 51; Viruses - 0; Other Eukaryotes - 2 (source: NCBI BLink). | chr3:8562965-8564640 REVERSEmoderately similar to ( 202) loc_os07g09150 12007.m05371 protein expressed protein   no original description 	T
27.3.17	RNA.regulation of transcription.CPP(Zn),CPP1-related transcription factor family	MDP0000292449	 moderately similar to ( 474) AT4G14770 | Symbols: TCX2, ATTCX2 | TCX2 (TESMIN/TSO1-LIKE CXC 2); transcription factor | chr4:8481522-8484825 REVERSEmoderately similar to ( 365) loc_os07g07974 12007.m05257 protein TSO1, putative, expressed  no original description 	T
27.3.3	RNA.regulation of transcription.AP2/EREBP, APETALA2/Ethylene-responsive element binding protein family	MDP0000756341	 weakly similar to ( 160) AT5G67190 | Symbols:  | AP2 domain-containing transcription factor, putative | chr5:26809135-26809689 REVERSEvery weakly similar to (81.3) ERF5_TOBAC Ethylene-responsive transcription factor 5 (Ethylene-responsive element-binding factor 5 homolog) (EREBP-4) (NtERF4) - Nicotiana tabacum (Common tobacco)weakly similar to ( 150) loc_os04g55520 12004.m10443 protein dehydration responsive element binding protein, putative, expressed  no original description 	T
20.1.7	stress.biotic.PR-proteins	MDP0000322399	 moderately similar to ( 479) AT5G19980 | Symbols:  | integral membrane family protein | chr5:6749907-6750932 REVERSEhighly similar to ( 501) loc_os09g14520 12009.m04714 protein expressed protein   no original description 	T
29.2.1.2.1.21	protein.synthesis.ribosomal protein.eukaryotic.40S subunit.S21	MDP0000733716	 weakly similar to ( 148) AT5G27700 | Symbols:  | structural constituent of ribosome | chr5:9807541-9808048 REVERSEweakly similar to ( 137) RS21_ORYSA 40S ribosomal protein S21 - Oryza sativa (Rice)weakly similar to ( 129) loc_os03g46490 12003.m09669 protein 40S ribosomal protein S21, putative  no original description 	T
29.3.5	protein.targeting.peroxisomes	MDP0000299043	 very weakly similar to (95.5) AT1G12830 | Symbols:  | unknown protein | chr1:4374412-4375053 REVERSE  no original description 	T
27.3.24	RNA.regulation of transcription.MADS box transcription factor family	MDP0000197958	 very weakly similar to (68.6) AT4G24540 | Symbols: AGL24 | AGL24 (AGAMOUS-LIKE 24); protein binding / protein heterodimerization/ protein homodimerization/ sequence-specific DNA binding / transcription factor | chr4:12671160-12673645 REVERSEvery weakly similar to (62.0) MAD47_ORYSA MADS-box transcription factor 47 (OsMADS47) - Oryza sativa (Rice)very weakly similar to (62.0) loc_os03g08754 12003.m35564 protein MADS-box transcription factor 47, putative, expressed  no original description 	T
29.5.1	protein.degradation.subtilases	MDP0000284537	 nearly identical (1097) AT4G20430 | Symbols:  | subtilase family protein | chr4:11017656-11021105 REVERSEnearly identical (1068) loc_os01g56320 12001.m11788 protein peptidase/ subtilase, putative, expressed   no original description 	T
10.1.4	cell wall.precursor synthesis.UGD	MDP0000193220	 highly similar to ( 870) AT5G15490 | Symbols:  | UDP-glucose 6-dehydrogenase, putative | chr5:5027872-5029314 REVERSEhighly similar to ( 874) UGDH_SOYBN UDP-glucose 6-dehydrogenase (EC 1.1.1.22) (UDP-Glc dehydrogenase) (UDP-GlcDH) (UDPGDH) - Glycine max (Soybean)highly similar to ( 879) loc_os12g25700 12012.m26903 protein UDP-glucose 6-dehydrogenase, putative, expressed no original description 	T
35.2	not assigned.unknown	MDP0000319787	 moderately similar to ( 379) AT3G10940 | Symbols:  | protein phosphatase-related | chr3:3422259-3423394 REVERSEmoderately similar to ( 344) loc_os12g02120 12012.m073738 protein dual-specificity protein-like phosphatase 3, putative, expressed  no original description 	T
35.1	not assigned.no ontology	MDP0000165446	 moderately similar to ( 358) AT3G24570 | Symbols:  | peroxisomal membrane 22 kDa family protein | chr3:8966675-8968530 REVERSEmoderately similar to ( 294) loc_os01g12800 12001.m07893 protein mpv17 protein, putative, expressed  no original description 	T
29.2.1.2.1.8	protein.synthesis.ribosomal protein.eukaryotic.40S subunit.S8	MDP0000145857	 moderately similar to ( 263) AT5G59240 | Symbols:  | 40S ribosomal protein S8 (RPS8B) | chr5:23902626-23903670 REVERSEmoderately similar to ( 277) RS8_ORYSA 40S ribosomal protein S8 - Oryza sativa (Rice)moderately similar to ( 279) loc_os04g28180 12004.m07935 protein 40S ribosomal protein S8, putative, expressed no original description 	T
28.99	DNA.unspecified	MDP0000671514	 very weakly similar to (89.0) AT3G15140 | Symbols:  | exonuclease family protein | chr3:5099712-5101717 REVERSEmoderately similar to ( 435) loc_os01g17279 12001.m150555 protein histone mRNA exonuclease 1, putative, expressed   no original description 	T
30.5	signalling.G-proteins	MDP0000306885	 highly similar to ( 632) AT4G38430 | Symbols: ATROPGEF1, ROPGEF1 | ROPGEF1; Rho guanyl-nucleotide exchange factor/ protein binding | chr4:17986643-17988659 FORWARDhighly similar to ( 689) loc_os09g37270 12009.m22184 protein pollen-specific kinase partner protein, putative, expressed  no original description 	T
29.3.3	protein.targeting.chloroplast	MDP0000751091	 nearly identical (1224) AT3G46740 | Symbols: TOC75-III | TOC75-III (TRANSLOCON AT THE OUTER ENVELOPE MEMBRANE OF CHLOROPLASTS 75-III); P-P-bond-hydrolysis-driven protein transmembrane transporter | chr3:17216104-17219296 REVERSEnearly identical (1172) TOC75_PEA Protein TOC75, chloroplast precursor (75 kDa translocon at the outer-envelope-membrane of chloroplasts) (Import intermediate associated protein of 75 kDa) (75 kDa chloroplast outer envelope protein) - Pisum sativum (Garden pea)nearly identical (1047) loc_os03g16440 12003.m34707 protein protein TOC75, chloroplast precursor, putative, expressed    Sporozoite_P67  no original description 	T
27.4	RNA.RNA binding	MDP0000223369	 moderately similar to ( 473) AT4G27000 | Symbols: ATRBP45C | ATRBP45C; RNA binding | chr4:13554983-13557763 REVERSEvery weakly similar to (81.3) ROC1_NICPL 30 kDa ribonucleoprotein, chloroplast precursor (CP-RBP30) - Nicotiana plumbaginifolia (Leadwort-leaved tobacco)moderately similar to ( 451) loc_os03g37270 12003.m08836 protein expressed protein PRK08026  no original description 	T
31.1	cell.organisation	MDP0000294477	 weakly similar to ( 169) AT5G09820 | Symbols:  | plastid-lipid associated protein PAP / fibrillin family protein | chr5:3056090-3057380 REVERSEweakly similar to ( 165) loc_os04g34460 12004.m08540 protein structural molecule, putative, expressed   no original description 	T
10.5.5	cell wall.cell wall proteins.RGP	MDP0000146639	 highly similar to ( 637) AT3G08900 | Symbols: RGP3, RGP | RGP3 (REVERSIBLY GLYCOSYLATED POLYPEPTIDE 3); transferase, transferring hexosyl groups | chr3:2708347-2709714 REVERSEhighly similar to ( 657) UPTG2_SOLTU Alpha-1,4-glucan-protein synthase [UDP-forming] 2 (EC 2.4.1.112) (UDP-glucose:protein transglucosylase 2) (UPTG 2) - Solanum tuberosum (Potato)highly similar to ( 649) loc_os03g40270 12003.m35306 protein alpha-1,4-glucan-protein synthase, putative, expressed   no original description 	T
1.1.1.2	PS.lightreaction.photosystem II.PSII polypeptide subunits	MDP0000248920	 highly similar to ( 556) AT3G50820 | Symbols: PSBO2, PSBO-2, OEC33 | PSBO2 (PHOTOSYSTEM II SUBUNIT O-2); oxygen evolving/ poly(U) binding | chr3:18891008-18892311 REVERSEhighly similar to ( 578) PSBO_TOBAC Oxygen-evolving enhancer protein 1, chloroplast precursor (OEE1) (33 kDa subunit of oxygen evolving system of photosystem II) (OEC 33 kDa subunit) (33 kDa thylakoid membrane protein) - Nicotiana tabacum (Common tobacco)highly similar to ( 538) loc_os01g31690 12001.m43025 protein oxygen-evolving enhancer protein 1, chloroplast precursor, putative, expressed   no original description 	T
29.5.11.20	protein.degradation.ubiquitin.proteasom	MDP0000328601	 nearly identical (1395) AT2G20580 | Symbols: RPN1A, ATRPN1A | RPN1A (26S PROTEASOME REGULATORY SUBUNIT S2 1A); binding / enzyme regulator | chr2:8859211-8864699 FORWARDnearly identical (1279) loc_os02g05340 12002.m05882 protein 26S proteasome regulatory subunit rpn1, putative, expressed  no original description 	T
35.2	not assigned.unknown	MDP0000309461	   no original description 	T
33.99	development.unspecified	MDP0000274583	 highly similar to ( 559) AT2G46280 | Symbols: TRIP-1, TIF3I1 | TRIP-1 (TGF-BETA RECEPTOR INTERACTING PROTEIN 1); nucleotide binding / protein binding | chr2:19003656-19005393 REVERSEvery weakly similar to (72.0) PF20_CHLRE Flagellar WD repeat protein PF20 - Chlamydomonas reinhardtiihighly similar to ( 550) loc_os08g21660 12008.m06205 protein eukaryotic translation initiation factor 3 subunit 2, putative, expressed   no original description 	T
35.2	not assigned.unknown	MDP0000250771	 moderately similar to ( 419) AT3G56900 | Symbols:  | aladin-related / adracalin-related | chr3:21066330-21069343 FORWARDmoderately similar to ( 382) loc_os12g03540 12012.m04352 protein cytochrome cd1-nitrite reductase-like, C-terminal haem d1, putative, expressed no original description 	T
29.5.7	protein.degradation.metalloprotease	MDP0000314814	 nearly identical (1395) AT5G56730 | Symbols:  | peptidase M16 family protein / insulinase family protein | chr5:22946906-22952576 REVERSEnearly identical (1362) loc_os01g37825 12001.m43422 protein peptidase M16 inactive domain containing protein, expressed  no original description 	T
2.1.1.2	major CHO metabolism.synthesis.sucrose.SPP	MDP0000369360	 moderately similar to ( 302) AT2G35840 | Symbols:  | sucrose-phosphatase 1 (SPP1) | chr2:15053952-15055776 FORWARDmoderately similar to ( 280) loc_os02g05030 12002.m05851 protein sucrose phosphate synthase, putative, expressed  no original description 	T
31.2	cell.division	MDP0000767927	 moderately similar to ( 290) AT5G40770 | Symbols: ATPHB3 | ATPHB3 (PROHIBITIN 3) | chr5:16315589-16316621 REVERSEmoderately similar to ( 259) loc_os02g37000 12002.m08788 protein mitochondrial prohibitin complex protein 1, putative, expressed no original description 	T
29.2.1.1.1.2.17	protein.synthesis.ribosomal protein.prokaryotic.chloroplast.50S subunit.L17	MDP0000392579	 moderately similar to ( 232) AT3G54210 | Symbols:  | ribosomal protein L17 family protein | chr3:20067672-20068385 REVERSEmoderately similar to ( 226) RK17_TOBAC 50S ribosomal protein L17, chloroplast precursor (CL17) - Nicotiana tabacum (Common tobacco)moderately similar to ( 209) loc_os03g60100 12003.m10904 protein 50S ribosomal protein L17, putative, expressed  no original description 	T
29.5.11.4.3.2	protein.degradation.ubiquitin.E3.SCF.FBOX	MDP0000716238	 very weakly similar to (70.9) AT5G49610 | Symbols:  | F-box family protein | chr5:20131448-20132527 REVERSEvery weakly similar to (93.6) loc_os03g56500 12003.m10559 protein expressed protein  no original description 	T
29.2.1.2.2.535	protein.synthesis.ribosomal protein.eukaryotic.60S subunit.L35A	MDP0000119667	 moderately similar to ( 215) AT1G74270 | Symbols:  | 60S ribosomal protein L35a (RPL35aC) | chr1:27928415-27929466 REVERSEweakly similar to ( 196) loc_os02g54470 12002.m100389 protein 60S ribosomal protein L33-B, putative, expressed no original description 	T
26.3.2	misc.gluco-, galacto- and mannosidases.beta-galactosidase	MDP0000227393	 nearly identical (1418) AT4G36360 | Symbols: BGAL3 | BGAL3 (beta-galactosidase 3); beta-galactosidase/ catalytic/ cation binding / sugar binding | chr4:17176840-17181143 REVERSEnearly identical (1105) BGAL_ASPOF Beta-galactosidase precursor (EC 3.2.1.23) (Lactase) - Asparagus officinalis (Garden asparagus)nearly identical (1283) loc_os03g06940 12003.m35030 protein beta-galactosidase precursor, putative, expressed  no original description 	T
11.3	lipid metabolism.Phospholipid synthesis	MDP0000326344	 highly similar to ( 546) AT3G57650 | Symbols: LPAT2 | LPAT2; 1-acylglycerol-3-phosphate O-acyltransferase | chr3:21349751-21352839 FORWARDhighly similar to ( 546) LPAT2_BRAOL 1-acyl-sn-glycerol-3-phosphate acyltransferase 2 (EC 2.3.1.51) (Lysophosphatidyl acyltransferase 2) - Brassica oleracea (Wild cabbage)moderately similar to ( 493) loc_os11g41900 12011.m08028 protein 1-acyl-sn-glycerol-3-phosphate acyltransferase PLS1, putative, expressed  no original description 	T
35.2	not assigned.unknown	MDP0000776908	 weakly similar to ( 150) AT1G64700 | Symbols:  | unknown protein | chr1:24039931-24040542 REVERSE  no original description 	T
35.2	not assigned.unknown	MDP0000568274	 moderately similar to ( 306) AT3G08640 | Symbols:  | alphavirus core protein family | chr3:2622992-2624005 FORWARDmoderately similar to ( 278) loc_os01g59740 12001.m12108 protein expressed protein   no original description 	T
13.2.2.3	amino acid metabolism.degradation.glutamate family.arginine	MDP0000284554	 moderately similar to ( 313) AT4G34710 | Symbols: ADC2, SPE2, ATADC2 | ADC2 (ARGININE DECARBOXYLASE 2); arginine decarboxylase | chr4:16560315-16562450 REVERSEmoderately similar to ( 319) SPE1_SOYBN Arginine decarboxylase (EC 4.1.1.19) (ARGDC) (ADC) - Glycine max (Soybean)moderately similar to ( 280) loc_os06g04070 12006.m05138 protein arginine decarboxylase, putative, expressed  no original description 	T
22.1.3	polyamine metabolism.synthesis.arginine decarboxylase	MDP0000284554	 moderately similar to ( 313) AT4G34710 | Symbols: ADC2, SPE2, ATADC2 | ADC2 (ARGININE DECARBOXYLASE 2); arginine decarboxylase | chr4:16560315-16562450 REVERSEmoderately similar to ( 319) SPE1_SOYBN Arginine decarboxylase (EC 4.1.1.19) (ARGDC) (ADC) - Glycine max (Soybean)moderately similar to ( 280) loc_os06g04070 12006.m05138 protein arginine decarboxylase, putative, expressed  no original description 	T
35.2	not assigned.unknown	MDP0000849985	 highly similar to ( 624) AT5G41950 | Symbols:  | binding | chr5:16785825-16789360 FORWARDhighly similar to ( 598) loc_os03g02270 12003.m05769 protein expressed protein   no original description 	T
19.21	tetrapyrrole synthesis.heme oxygenase	MDP0000700015	 moderately similar to ( 394) AT2G26670 | Symbols: ATHO1, HO1, TED4, GUN2, HY6 | TED4 (REVERSAL OF THE DET PHENOTYPE 4); heme oxygenase (decyclizing) | chr2:11341816-11343394 FORWARDmoderately similar to ( 337) loc_os06g40080 12006.m08545 protein heme oxygenase 1, putative, expressed   no original description 	T
29.2.1.1.1.1.11	protein.synthesis.ribosomal protein.prokaryotic.chloroplast.30S subunit.S11	MDP0000265320	 moderately similar to ( 223) AT3G20000 | Symbols: TOM40 | TOM40; P-P-bond-hydrolysis-driven protein transmembrane transporter/ voltage-gated anion channel | chr3:6967685-6970247 FORWARDvery weakly similar to (75.1) RT11_PROWI Mitochondrial ribosomal protein S11 - Prototheca wickerhamiimoderately similar to ( 246) loc_os03g20750 12003.m07482 protein mitochondrial import receptor subunit TOM40 homolog 1, putative, expressed   no original description 	T
29.2.1.1.2.1.11	protein.synthesis.ribosomal protein.prokaryotic.mitochondrion.30S subunit.S11	MDP0000265320	 moderately similar to ( 223) AT3G20000 | Symbols: TOM40 | TOM40; P-P-bond-hydrolysis-driven protein transmembrane transporter/ voltage-gated anion channel | chr3:6967685-6970247 FORWARDvery weakly similar to (75.1) RT11_PROWI Mitochondrial ribosomal protein S11 - Prototheca wickerhamiimoderately similar to ( 246) loc_os03g20750 12003.m07482 protein mitochondrial import receptor subunit TOM40 homolog 1, putative, expressed   no original description 	T
28.1.1.4	DNA.synthesis/chromatin structure.retrotransposon/transposase.hat-like transposase	MDP0000423776	 moderately similar to ( 238) AT1G74050 | Symbols:  | 60S ribosomal protein L6 (RPL6C) | chr1:27847256-27848680 REVERSEmoderately similar to ( 249) RL6_MESCR 60S ribosomal protein L6 (YL16-like) - Mesembryanthemum crystallinum (Common ice plant)moderately similar to ( 217) loc_os04g39700 12004.m08956 protein 60S ribosomal protein L6, putative, expressed MSP1_C  no original description 	T
30.5	signalling.G-proteins	MDP0000309489	 moderately similar to ( 215) AT1G07140 | Symbols: SIRANBP | SIRANBP; Ran GTPase binding | chr1:2192360-2193688 REVERSEmoderately similar to ( 202) loc_os03g18180 12003.m07243 protein ran-binding protein 1 homolog c, putative, expressed  no original description 	T
20.2.3	stress.abiotic.drought/salt	MDP0000182090	 moderately similar to ( 260) AT1G33170 | Symbols:  | dehydration-responsive family protein | chr1:12027262-12030397 FORWARDmoderately similar to ( 263) loc_os06g01450 12006.m04884 protein methyltransferase, putative, expressed no original description 	T
35.2	not assigned.unknown	MDP0000253476	  no original description 	T
35.2	not assigned.unknown	MDP0000492898	 weakly similar to ( 188) AT2G40435 | Symbols:  | EXPRESSED IN: 20 plant structures; EXPRESSED DURING: 13 growth stages; BEST Arabidopsis thaliana protein match is: transcription regulator (TAIR:AT3G56220.1); Has 165 Blast hits to 165 proteins in 20 species: Archae - 0; Bacteria - 0; Metazoa - 0; Fungi - 0; Plants - 165; Viruses - 0; Other Eukaryotes - 0 (source: NCBI BLink). | chr2:16887048-16888407 FORWARDweakly similar to ( 173) loc_os03g21970 12003.m07601 protein DNA binding protein, putative, expressed  no original description 	T
27.3.9	RNA.regulation of transcription.C2C2(Zn) GATA transcription factor family	MDP0000739900	 very weakly similar to ( 100) AT4G26150 | Symbols: CGA1 | CGA1 (CYTOKININ-RESPONSIVE GATA FACTOR 1); transcription factor | chr4:13253210-13254659 FORWARDvery weakly similar to (70.9) loc_os02g12790 12002.m06526 protein GATA transcription factor 20, putative, expressed   no original description 	T
29.5.3	protein.degradation.cysteine protease	MDP0000128517	 moderately similar to ( 253) AT3G12490 | Symbols: ATCYSB | cysteine protease inhibitor, putative / cystatin, putative | chr3:3960523-3961777 REVERSEweakly similar to ( 106) CYTI_VIGUN Cysteine proteinase inhibitor (Cystatin) - Vigna unguiculata (Cowpea)moderately similar to ( 258) loc_os01g16430 12001.m08239 protein multidomain cystatin, putative, expressed  no original description 	T
30.11	signalling.light	MDP0000812065	 highly similar to ( 933) AT1G30440 | Symbols:  | phototropic-responsive NPH3 family protein | chr1:10759475-10762199 FORWARDmoderately similar to ( 441) NPH3_ORYSA Coleoptile phototropism protein 1 (Non-phototropic hypocotyl 3-like protein) (NPH3-like protein) - Oryza sativa (Rice)highly similar to ( 910) loc_os03g43990 12003.m35330 protein transposon protein, putative, Mutator sub-class, expressed   no original description 	T
35.1.3	not assigned.no ontology.armadillo/beta-catenin repeat family protein	MDP0000207423	 highly similar to ( 573) AT2G05810 | Symbols:  | armadillo/beta-catenin repeat family protein | chr2:2215225-2216967 REVERSEvery weakly similar to (51.6) SPL11_ORYSA Spotted leaf protein 11 (Spotted leaf11) (Cell death-related protein SPL11) - Oryza sativa (Rice)moderately similar to ( 380) loc_os09g05030 12009.m22045 protein armadillo/beta-catenin-like repeat family protein, expressed   no original description 	T
17.3.1.2.2	hormone metabolism.brassinosteroid.synthesis-degradation.sterols.SMT2	MDP0000122791	 highly similar to ( 558) AT5G13710 | Symbols: SMT1, CPH | SMT1 (STEROL METHYLTRANSFERASE 1); sterol 24-C-methyltransferase | chr5:4424048-4426866 REVERSEhighly similar to ( 513) SMT1_ORYSA Cycloartenol-C-24-methyltransferase 1 (EC 2.1.1.41) (24-sterol C-methyltransferase 1) (Sterol C-methyltransferase 1) - Oryza sativa (Rice)highly similar to ( 513) loc_os07g10600 12007.m28956 protein cycloartenol-C-24-methyltransferase 1, putative, expressed no original description 	T
35.2	not assigned.unknown	MDP0000367327	  no original description 	T
29.2.1.2.2.5	protein.synthesis.ribosomal protein.eukaryotic.60S subunit.L5	MDP0000334081	 moderately similar to ( 471) AT5G39740 | Symbols: OLI7, RPL5B | 60S ribosomal protein L5 (RPL5B) | chr5:15903365-15905185 FORWARDmoderately similar to ( 469) RL5_CUCSA 60S ribosomal protein L5 - Cucumis sativus (Cucumber)moderately similar to ( 447) loc_os01g67126 12001.m43540 protein 60S ribosomal protein L5-2, putative, expressed  no original description 	T
29.4	protein.postranslational modification	MDP0000175680	 highly similar to ( 549) AT4G32830 | Symbols: AtAUR1 | AtAUR1 (ATAURORA1); histone kinase(H3-S10 specific) / kinase/ protein serine/threonine kinase | chr4:15842557-15844354 FORWARDweakly similar to ( 170) CDPK_DAUCA Calcium-dependent protein kinase (EC 2.7.11.1) (CDPK) - Daucus carota (Carrot)highly similar to ( 518) loc_os01g09580 12001.m07583 protein serine/threonine-protein kinase Eg2-like, putative, expressed  no original description 	T
27.3.9	RNA.regulation of transcription.C2C2(Zn) GATA transcription factor family	MDP0000220844	 moderately similar to ( 245) AT5G66320 | Symbols:  | zinc finger (GATA type) family protein | chr5:26496208-26497309 REVERSEweakly similar to ( 145) loc_os02g43150 12002.m100295 protein GATA zinc finger family protein, expressed  no original description 	T
26.28	misc.GDSL-motif lipase	MDP0000202137	 highly similar to ( 519) AT3G16370 | Symbols:  | GDSL-motif lipase/hydrolase family protein | chr3:5556928-5558351 FORWARDmoderately similar to ( 203) APG_BRANA Anter-specific proline-rich protein APG (Protein CEX) (Fragment) - Brassica napus (Rape)moderately similar to ( 483) loc_os06g05550 12006.m05282 protein anther-specific proline-rich protein APG precursor, putative, expressed   no original description 	T
31.1	cell.organisation	MDP0000270539	 moderately similar to ( 202) AT5G59880 | Symbols: ADF3 | ADF3 (ACTIN DEPOLYMERIZING FACTOR 3); actin binding | chr5:24120382-24121628 FORWARDmoderately similar to ( 212) ADF2_PETHY Actin-depolymerizing factor 2 (ADF 2) - Petunia hybrida (Petunia)moderately similar to ( 202) loc_os05g02250 12005.m64199 protein expressed protein  no original description 	T
29.2.4	protein.synthesis.elongation	MDP0000777793	 nearly identical (1573) AT1G56070 | Symbols: LOS1 | LOS1; copper ion binding / translation elongation factor/ translation factor, nucleic acid binding | chr1:20968245-20971077 REVERSEnearly identical (1553) EF2_BETVU Elongation factor 2 (EF-2) - Beta vulgaris (Sugar beet)nearly identical (1560) loc_os02g32030 12002.m08296 protein elongation factor 2, putative, expressed   no original description 	T
30.5	signalling.G-proteins	MDP0000347722	 moderately similar to ( 311) AT5G02040 | Symbols: PRA1.A1 | PRA1.A1 (PRENYLATED RAB ACCEPTOR 1.A1) | chr5:401163-402471 FORWARDmoderately similar to ( 333) loc_os01g62890 12001.m42756 protein PRA1 family protein, expressed no original description 	T
35.1	not assigned.no ontology	MDP0000281518	 moderately similar to ( 285) AT3G10260 | Symbols:  | reticulon family protein | chr3:3171413-3172568 REVERSEmoderately similar to ( 294) loc_os03g56900 12003.m101524 protein seed maturation protein, putative, expressed   no original description 	T
15.2	metal handling.binding, chelation and storage	MDP0000917650	 weakly similar to ( 119) AT1G06330 | Symbols:  | copper-binding family protein | chr1:1931671-1932266 REVERSEvery weakly similar to (98.6) loc_os01g55320 12001.m11691 protein copper ion binding protein, putative, expressed  no original description 	T
27.3.4	RNA.regulation of transcription.ARF, Auxin Response Factor family	MDP0000929655	 highly similar to ( 755) AT1G59750 | Symbols: ARF1 | ARF1 (AUXIN RESPONSE FACTOR 1); DNA binding / transcription factor | chr1:21980414-21984193 FORWARDhighly similar to ( 702) loc_os02g35140 12002.m08602 protein auxin response factor 1, putative, expressed  no original description 	T
35.2	not assigned.unknown	MDP0000189331	   no original description 	T
35.2	not assigned.unknown	MDP0000270796	 weakly similar to ( 200) AT5G42050 | Symbols:  | FUNCTIONS IN: molecular_function unknown; EXPRESSED IN: 22 plant structures; EXPRESSED DURING: 13 growth stages; CONTAINS InterPro DOMAIN/s: Development and cell death domain (InterPro:IPR013989), Kelch related (InterPro:IPR013089); BEST Arabidopsis thaliana protein match is: unknown protein (TAIR:AT3G27090.1); Has 5084 Blast hits to 2870 proteins in 85 species: Archae - 0; Bacteria - 24; Metazoa - 270; Fungi - 58; Plants - 157; Viruses - 8; Other Eukaryotes - 4567 (source: NCBI BLink). | chr5:16815630-16816932 FORWARDmoderately similar to ( 204) B2_DAUCA B2 protein - Daucus carota (Carrot)weakly similar to ( 197) loc_os01g36950 12001.m150623 protein N-rich protein, putative, expressed no original description 	T
29.4	protein.postranslational modification	MDP0000915350	 highly similar to ( 550) AT5G62710 | Symbols:  | leucine-rich repeat family protein / protein kinase family protein | chr5:25187438-25190325 FORWARDmoderately similar to ( 241) PSKR_DAUCA Phytosulfokine receptor precursor (EC 2.7.11.1) (Phytosulfokine LRR receptor kinase) - Daucus carota (Carrot)moderately similar to ( 461) loc_os12g44090 12012.m08180 protein ATP binding protein, putative, expressed  no original description 	T
29.4.1.57	protein.postranslational modification.kinase.receptor like cytoplasmatic kinase VII	MDP0000915350	 highly similar to ( 550) AT5G62710 | Symbols:  | leucine-rich repeat family protein / protein kinase family protein | chr5:25187438-25190325 FORWARDmoderately similar to ( 241) PSKR_DAUCA Phytosulfokine receptor precursor (EC 2.7.11.1) (Phytosulfokine LRR receptor kinase) - Daucus carota (Carrot)moderately similar to ( 461) loc_os12g44090 12012.m08180 protein ATP binding protein, putative, expressed  no original description 	T
27.3.99	RNA.regulation of transcription.unclassified	MDP0000782742	 highly similar to ( 672) AT3G43230 | Symbols:  | zinc finger (FYVE type) family protein | chr3:15207776-15209599 FORWARDhighly similar to ( 619) loc_os07g42500 12007.m08482 protein senescence-associated-like protein, putative, expressed  no original description 	T
30.3	signalling.calcium	MDP0000859814	 weakly similar to ( 183) AT2G41410 | Symbols:  | calmodulin, putative | chr2:17262085-17262735 REVERSEvery weakly similar to (92.8) CALM_WHEAT Calmodulin (CaM) - Triticum aestivum (Wheat)weakly similar to ( 106) loc_os01g72100 12001.m13246 protein polcalcin Jun o 2, putative, expressed  no original description 	T
30.3	signalling.calcium	MDP0000272522	 weakly similar to ( 109) AT2G41410 | Symbols:  | calmodulin, putative | chr2:17262085-17262735 REVERSEvery weakly similar to (66.2) CALM_WHEAT Calmodulin (CaM) - Triticum aestivum (Wheat)very weakly similar to (60.8) loc_os01g72100 12001.m13246 protein polcalcin Jun o 2, putative, expressed  no original description 	T
31.1	cell.organisation	MDP0000329597	 moderately similar to ( 409) AT2G24420 | Symbols:  | DNA repair ATPase-related | chr2:10380147-10382624 FORWARDmoderately similar to ( 366) loc_os08g40790 12008.m26747 protein expressed protein  no original description 	T
34.21	transport.calcium	MDP0000289952	 highly similar to ( 562) AT1G55730 | Symbols: ATCAX5, CAX5 | ATCAX5; calcium:cation antiporter/ cation:cation antiporter | chr1:20831387-20833941 REVERSEhighly similar to ( 526) CAX2_ORYSA Vacuolar cation/proton exchanger 2 (Ca(2+)/H(+) exchanger 2) (OsCAX2) - Oryza sativa (Rice)highly similar to ( 526) loc_os03g27960 12003.m08120 protein vacuolar cation/proton exchanger 2, putative, expressed no original description 	T
29.5.11.20	protein.degradation.ubiquitin.proteasom	MDP0000138792	 moderately similar to ( 390) AT2G39990 | Symbols: EIF2 | EIF2; translation initiation factor | chr2:16698332-16699929 REVERSEmoderately similar to ( 375) loc_os05g01450 12005.m27663 protein eukaryotic translation initiation factor 3 subunit 5, putative, expressed   no original description 	T
29.5.11.4.2	protein.degradation.ubiquitin.E3.RING	MDP0000884442	 moderately similar to ( 234) AT4G14220 | Symbols: RHF1A | RHF1A (RING-H2 GROUP F1A); protein binding / ubiquitin-protein ligase/ zinc ion binding | chr4:8196012-8198240 FORWARDweakly similar to ( 195) loc_os11g36430 12011.m28683 protein zinc finger, RING-type, putative, expressed   no original description 	T
29.2.1.2.2.39	protein.synthesis.ribosomal protein.eukaryotic.60S subunit.L39	MDP0000363784	 weakly similar to ( 110) AT4G31985 | Symbols:  | 60S ribosomal protein L39 (RPL39C) | chr4:15469931-15470366 FORWARDweakly similar to ( 107) RL39_ORYSA 60S ribosomal protein L39 - Oryza sativa (Rice)weakly similar to ( 107) loc_os06g08320 12006.m05553 protein 60S ribosomal protein L39, putative, expressed no original description 	T
29.5.11.20	protein.degradation.ubiquitin.proteasom	MDP0000524965	 moderately similar to ( 497) AT5G58290 | Symbols: RPT3 | RPT3 (REGULATORY PARTICLE TRIPLE-A ATPASE 3); ATPase | chr5:23569155-23571116 FORWARDmoderately similar to ( 497) PRS6B_SOLTU 26S protease regulatory subunit 6B homolog - Solanum tuberosum (Potato)moderately similar to ( 489) loc_os02g21970 12002.m07435 protein 26S protease regulatory subunit 6B, putative, expressed  no original description 	T
17.7.1.4	hormone metabolism.jasmonate.synthesis-degradation.allene oxidase cyclase	MDP0000239834	 moderately similar to ( 260) AT1G13280 | Symbols: AOC4 | AOC4 (ALLENE OXIDE CYCLASE 4); allene-oxide cyclase | chr1:4547624-4548552 FORWARDmoderately similar to ( 284) loc_os03g32314 12003.m08473 protein allene oxide cyclase 4, chloroplast precursor, putative, expressed   no original description 	T
26.2	misc.UDP glucosyl and glucoronyl transferases	MDP0000204168	 highly similar to ( 779) AT3G15940 | Symbols:  | glycosyl transferase family 1 protein | chr3:5393632-5396187 REVERSEweakly similar to ( 103) loc_os01g15780 12001.m42953 protein transferase, transferring glycosyl groups, putative, expressed  no original description 	T
10.1.1.1	cell wall.precursor synthesis.NDP sugar pyrophosphorylase.GDP mannose	MDP0000203812	 highly similar to ( 662) AT2G39770 | Symbols: CYT1, VTC1, SOZ1, EMB101, GMP1 | CYT1 (CYTOKINESIS DEFECTIVE 1); mannose-1-phosphate guanylyltransferase/ nucleotidyltransferase | chr2:16589401-16590741 FORWARDvery weakly similar to (50.8) GLGL1_MAIZE Glucose-1-phosphate adenylyltransferase large subunit 1, chloroplast precursor (EC 2.7.7.27) (ADP-glucose synthase) (ADP-glucose pyrophosphorylase) (AGPase S) (Alpha-D-glucose-1-phosphate adenyl transferase) (Shrunken-2) - Zea mays (Mahighly similar to ( 654) loc_os08g13930 12008.m26632 protein mannose-1-phosphate guanyltransferase, putative, expressed  no original description 	T
17.7.1.2	hormone metabolism.jasmonate.synthesis-degradation.lipoxygenase	MDP0000277666	 highly similar to ( 964) AT3G45140 | Symbols: LOX2, ATLOX2 | LOX2 (LIPOXYGENASE 2); lipoxygenase | chr3:16525437-16529233 FORWARDhighly similar to ( 936) LOX23_HORVU Lipoxygenase 2.3, chloroplast precursor (EC 1.13.11.12) (LOX2:Hv:3) - Hordeum vulgare (Barley)highly similar to ( 952) loc_os02g10120 12002.m06309 protein lipoxygenase 2.3, chloroplast precursor, putative, expressed   no original description 	T
35.2	not assigned.unknown	MDP0000161846	 weakly similar to ( 145) AT1G52140 | Symbols:  | unknown protein | chr1:19407559-19408185 REVERSEvery weakly similar to (90.9) loc_os06g36070 12006.m08144 protein expressed protein  no original description 	T
35.2	not assigned.unknown	MDP0000713652	 weakly similar to ( 135) AT1G73885 | Symbols:  | unknown protein | chr1:27786864-27787708 FORWARDweakly similar to ( 142) loc_os03g50270 12003.m10020 protein expressed protein  no original description 	T
35.2	not assigned.unknown	MDP0000627609	 moderately similar to ( 388) AT1G74450 | Symbols:  | unknown protein | chr1:27982737-27983930 FORWARDmoderately similar to ( 281) loc_os04g59140 12004.m10791 protein expressed protein    TrbL  no original description 	T
26.7	misc.oxidases - copper, flavone etc	MDP0000276954	 moderately similar to ( 342) AT5G17000 | Symbols:  | NADP-dependent oxidoreductase, putative | chr5:5584983-5586991 REVERSEmoderately similar to ( 371) loc_os06g40070 12006.m08544 protein NADP-dependent oxidoreductase P2, putative, expressed  no original description 	T
29.1	protein.aa activation	MDP0000188838	 moderately similar to ( 352) AT4G27490 | Symbols:  | 3' exoribonuclease family domain 1-containing protein | chr4:13739961-13741647 FORWARDmoderately similar to ( 330) loc_os03g62720 12003.m11149 protein exosome complex exonuclease 1, putative, expressed no original description 	T
35.2	not assigned.unknown	MDP0000321110	 highly similar to ( 722) AT5G06550 | Symbols:  | FUNCTIONS IN: molecular_function unknown; INVOLVED IN: cell surface receptor linked signal transduction; EXPRESSED IN: 19 plant structures; EXPRESSED DURING: 12 growth stages; CONTAINS InterPro DOMAIN/s: Cyclin-like F-box (InterPro:IPR001810), Transcription factor jumonji/aspartyl beta-hydroxylase (InterPro:IPR003347), Transcription factor jumonji (InterPro:IPR013129); BEST Arabidopsis thaliana protein match is: transferase, transferring glycosyl groups (TAIR:AT1G78280.1); Has 1299 Blast hits to 1289 proteins in 204 species: Archae - 0; Bacteria - 195; Metazoa - 777; Fungi - 106; Plants - 94; Viruses - 0; Other Eukaryotes - 127 (source: NCBI BLink). | chr5:2001246-2003068 REVERSEhighly similar to ( 616) loc_os11g36450 12011.m07497 protein transferase, transferring glycosyl groups, putative, expressed  no original description 	T
3.1.2.2	minor CHO metabolism.raffinose family.raffinose synthases.putative	MDP0000857666	 weakly similar to ( 188) AT3G57520 | Symbols: AtSIP2 | AtSIP2 (Arabidopsis thaliana seed imbibition 2); hydrolase, hydrolyzing O-glycosyl compounds | chr3:21289299-21292694 REVERSEvery weakly similar to (80.9) STSYN_PEA Stachyose synthase precursor (EC 2.4.1.67) (Galactinol--raffinose galactosyltransferase) - Pisum sativum (Garden pea)weakly similar to ( 183) loc_os03g59430 12003.m10841 protein stachyose synthase precursor, putative, expressed  no original description 	T
34.13	transport.peptides and oligopeptides	MDP0000896511	 highly similar to ( 789) AT1G32450 | Symbols: NRT1.5 | NRT1.5 (NITRATE TRANSPORTER 1.5); nitrate transmembrane transporter/ transporter | chr1:11715337-11719807 REVERSEhighly similar to ( 590) loc_os02g46460 12002.m09683 protein peptide transporter PTR2, putative, expressed   no original description 	T
27.3.71	RNA.regulation of transcription.SNF7	MDP0000410714	 moderately similar to ( 303) AT5G22950 | Symbols: VPS24.1 | VPS24.1 | chr5:7681380-7682720 FORWARDmoderately similar to ( 288) loc_os07g29630 12007.m07235 protein charged multivesicular body protein 3, putative, expressed no original description 	T
10.8.2	cell wall.pectin*esterases.acetyl esterase	MDP0000313863	 moderately similar to ( 485) AT4G19420 | Symbols:  | pectinacetylesterase family protein | chr4:10587504-10589807 REVERSEmoderately similar to ( 461) loc_os02g47400 12002.m100327 protein carboxylic ester hydrolase, putative, expressed no original description 	T
20.2.5	stress.abiotic.light	MDP0000918616	 nearly identical (1216) AT1G21640 | Symbols: NADK2, ATNADK2 | NADK2; NAD+ kinase/ calmodulin binding | chr1:7588726-7592669 FORWARDnearly identical (1122) NADK2_ORYSA Probable NAD kinase 2, chloroplast precursor (EC 2.7.1.23) - Oryza sativa (Rice)nearly identical (1122) loc_os11g08670 12011.m05064 protein NAD kinase 2, chloroplast precursor, putative, expressed MSP1_C  no original description 	T
30.5	signalling.G-proteins	MDP0000296959	 moderately similar to ( 439) AT1G18190 | Symbols: GC2 | GC2 (golgin candidate 2) | chr1:6257963-6261325 REVERSEmoderately similar to ( 243) RGP2_ORYSA Ras-related protein RGP2 (GTP-binding regulatory protein RGP2) - Oryza sativa (Rice)moderately similar to ( 325) loc_os04g55810 12004.m10472 protein expressed protein  no original description 	T
29.5.11.20	protein.degradation.ubiquitin.proteasom	MDP0000322547	 moderately similar to ( 395) AT5G66140 | Symbols: PAD2 | PAD2 (PROTEASOME ALPHA SUBUNIT D 2); endopeptidase/ peptidase/ threonine-type endopeptidase | chr5:26437445-26438677 REVERSEmoderately similar to ( 405) PSA7_CICAR Proteasome subunit alpha type 7 (EC 3.4.25.1) (20S proteasome alpha subunit D) (20S proteasome subunit alpha-4) - Cicer arietinum (Chickpea) (Garbanzo)moderately similar to ( 393) loc_os09g36710 12009.m06620 protein proteasome subunit alpha type 7, putative, expressed   no original description 	T
17.3.1.2.5	hormone metabolism.brassinosteroid.synthesis-degradation.sterols.HYD1	MDP0000131723	 moderately similar to ( 253) AT1G20050 | Symbols: HYD1 | HYD1 (HYDRA1); C-8 sterol isomerase | chr1:6949160-6950135 FORWARDmoderately similar to ( 238) EBP_ORYSA Probable 3-beta-hydroxysteroid-Delta(8),Delta(7)-isomerase (EC 5.3.3.5) (Cholestenol Delta-isomerase) (Delta(8)-Delta(7) sterol isomerase) (D8-D7 sterol isomerase) - Oryza sativa (Rice)moderately similar to ( 238) loc_os01g01369 12001.m150882 protein 3-beta-hydroxysteroid-delta-isomerase, putative, expressed   no original description 	T
26.16	misc.myrosinases-lectin-jacalin	MDP0000924331	 weakly similar to ( 127) AT4G19840 | Symbols: ATPP2-A1, ATPP2A-1, PP2-A1 | ATPP2-A1; carbohydrate binding | chr4:10774336-10775701 FORWARDweakly similar to ( 127) loc_os01g06500 12001.m07280 protein lectin-like protein, putative, expressed  no original description 	T
27.3.6	RNA.regulation of transcription.bHLH,Basic Helix-Loop-Helix family	MDP0000311721	 weakly similar to ( 112) AT4G20970 | Symbols:  | basic helix-loop-helix (bHLH) family protein | chr4:11215259-11216212 FORWARDvery weakly similar to (77.8) loc_os01g01870 12001.m06830 protein helix-loop-helix DNA-binding domain containing protein, expressed  no original description 	T
10.6.2	cell wall.degradation.mannan-xylose-arabinose-fucose	MDP0000321839	 highly similar to ( 628) AT5G01930 | Symbols:  | (1-4)-beta-mannan endohydrolase, putative | chr5:361189-362867 REVERSEmoderately similar to ( 492) loc_os05g25480 12005.m06841 protein hydrolase, hydrolyzing O-glycosyl compounds, putative, expressed  no original description 	T
20.1.7	stress.biotic.PR-proteins	MDP0000157225	 highly similar to ( 513) AT5G66900 | Symbols:  | disease resistance protein (CC-NBS-LRR class), putative | chr5:26714931-26717757 REVERSEmoderately similar to ( 397) loc_os12g39620 12012.m073872 protein disease resistance protein, putative, expressedMSP1_C  no original description 	T
35.2	not assigned.unknown	MDP0000139868	   no original description 	T
27.3.8	RNA.regulation of transcription.C2C2(Zn) DOF zinc finger family	MDP0000127431	 weakly similar to ( 127) AT1G28310 | Symbols:  | Dof-type zinc finger domain-containing protein | chr1:9912534-9913685 REVERSEweakly similar to ( 120) PBF_MAIZE Dof zinc finger protein PBF (Prolamin box-binding factor) - Zea mays (Maize)weakly similar to ( 125) loc_os07g32510 12007.m07514 protein expressed protein  no original description 	T
35.1	not assigned.no ontology	MDP0000206651	 weakly similar to ( 149) AT2G33845 | Symbols:  | DNA-binding protein-related | chr2:14317781-14318712 FORWARDweakly similar to ( 154) loc_os01g55030 12001.m11663 protein expressed protein   no original description 	T
35.2	not assigned.unknown	MDP0000157756	   no original description 	T
27.3.20	RNA.regulation of transcription.G2-like transcription factor family, GARP	MDP0000809773	 moderately similar to ( 339) AT4G13640 | Symbols: UNE16 | UNE16 (unfertilized embryo sac 16); transcription factor | chr4:7936864-7938497 REVERSEmoderately similar to ( 202) loc_os03g20900 12003.m07498 protein transfactor-like, putative, expressed no original description 	T
29.3.2	protein.targeting.mitochondria	MDP0000179083	 weakly similar to ( 184) AT1G72750 | Symbols: ATTIM23-2 | ATTIM23-2; P-P-bond-hydrolysis-driven protein transmembrane transporter/ protein transporter | chr1:27383577-27384143 FORWARDweakly similar to ( 167) loc_os03g02390 12003.m05781 protein mitochondrial import inner membrane translocase subunit tim23, putative, expressed   no original description 	T
35.2	not assigned.unknown	MDP0000179767	 highly similar to ( 506) AT1G29050 | Symbols:  | unknown protein | chr1:10136376-10139082 REVERSEmoderately similar to ( 441) loc_os01g46350 12001.m10833 protein expressed protein   no original description 	T
9.1.2	mitochondrial electron transport / ATP synthesis.NADH-DH.localisation not clear	MDP0000319798	 nearly identical (1160) AT5G37510 | Symbols: EMB1467 | EMB1467 (embryo defective 1467); NADH dehydrogenase (ubiquinone)/ NADH dehydrogenase/ electron carrier/ iron-sulfur cluster binding / oxidoreductase/ oxidoreductase, acting on NADH or NADPH | chr5:14897490-14900447 FORWARDnearly identical (1204) NDUS1_SOLTU NADH-ubiquinone oxidoreductase 75 kDa subunit, mitochondrial precursor (EC 1.6.5.3) (EC 1.6.99.3) (Complex I-75kD) (CI-75kD) (76 kDa mitochondrial complex I subunit) - Solanum tuberosum (Potato)nearly identical (1137) loc_os03g50540 12003.m10045 protein NADH-ubiquinone oxidoreductase 75 kDa subunit, mitochondrial precursor, putative, expressed  no original description 	T
30.2.17	signalling.receptor kinases.DUF 26	MDP0000214826	 moderately similar to ( 231) AT5G51560 | Symbols:  | leucine-rich repeat transmembrane protein kinase, putative | chr5:20945807-20948613 FORWARDvery weakly similar to (86.7) CRI4_MAIZE Putative receptor protein kinase CRINKLY4 precursor (EC 2.7.11.1) - Zea mays (Maize)weakly similar to ( 191) loc_os01g39600 12001.m150636 protein ATP binding protein, putative, expressed  no original description 	T
29.4.1.57	protein.postranslational modification.kinase.receptor like cytoplasmatic kinase VII	MDP0000214826	 moderately similar to ( 231) AT5G51560 | Symbols:  | leucine-rich repeat transmembrane protein kinase, putative | chr5:20945807-20948613 FORWARDvery weakly similar to (86.7) CRI4_MAIZE Putative receptor protein kinase CRINKLY4 precursor (EC 2.7.11.1) - Zea mays (Maize)weakly similar to ( 191) loc_os01g39600 12001.m150636 protein ATP binding protein, putative, expressed  no original description 	T
16.4.1	secondary metabolism.N misc.alkaloid-like	MDP0000159602	 weakly similar to ( 122) AT3G57030 | Symbols:  | strictosidine synthase family protein | chr3:21101653-21103204 REVERSEvery weakly similar to (63.9) STSY_CATRO Strictosidine synthase precursor (EC 4.3.3.2) - Catharanthus roseus (Rosy periwinkle) (Madagascar periwinkle)moderately similar to ( 204) loc_os07g42250 12007.m08460 protein strictosidine synthase 1 precursor, putative, expressed   COG4934 COG4907 CobN  no original description 	T
34.7	transport.phosphate	MDP0000238253	 highly similar to ( 625) AT5G44370 | Symbols: PHT4;6 | PHT4;6 (PHOSPHATE TRANSPORTER 4;6); carbohydrate transmembrane transporter/ inorganic phosphate transmembrane transporter/ organic anion transmembrane transporter/ sugar:hydrogen symporter | chr5:17875147-17876445 REVERSEhighly similar to ( 602) loc_os11g08370 12011.m05033 protein sialin, putative, expressed  no original description 	T
29.2.1.2.2.27	protein.synthesis.ribosomal protein.eukaryotic.60S subunit.L27	MDP0000322240	 moderately similar to ( 212) AT4G15000 | Symbols:  | 60S ribosomal protein L27 (RPL27C) | chr4:8571896-8572387 FORWARDmoderately similar to ( 214) RL27_PEA 60S ribosomal protein L27 - Pisum sativum (Garden pea)weakly similar to ( 197) loc_os02g18380 12002.m07081 protein 60S ribosomal protein L27, putative, expressed no original description 	T
28.1	DNA.synthesis/chromatin structure	MDP0000770205	 nearly identical (1353) AT1G44900 | Symbols: MCM2 | ATP binding / DNA binding / DNA-dependent ATPase | chr1:16970291-16974457 FORWARDmoderately similar to ( 330) MCM3_MAIZE DNA replication licensing factor MCM3 homolog (Replication origin activator) (ROA protein) (Fragment) - Zea mays (Maize)nearly identical (1408) loc_os11g29380 12011.m06848 protein DNA replication licensing factor Mcm2, putative, expressed   no original description 	T
35.2	not assigned.unknown	MDP0000135455	 moderately similar to ( 202) AT5G56170 | Symbols:  | FUNCTIONS IN: molecular_function unknown; INVOLVED IN: biological_process unknown; LOCATED IN: anchored to membrane; EXPRESSED IN: 22 plant structures; EXPRESSED DURING: 13 growth stages; BEST Arabidopsis thaliana protein match is: LRE (LORELEI) (TAIR:AT4G26466.1); Has 71 Blast hits to 71 proteins in 9 species: Archae - 0; Bacteria - 0; Metazoa - 0; Fungi - 0; Plants - 71; Viruses - 0; Other Eukaryotes - 0 (source: NCBI BLink). | chr5:22736072-22737108 FORWARDweakly similar to ( 174) loc_os02g48980 12002.m09932 protein GPI-anchored protein, putative, expressed  no original description 	T
29.4	protein.postranslational modification	MDP0000191016	 moderately similar to ( 350) AT2G25620 | Symbols:  | protein phosphatase 2C, putative / PP2C, putative | chr2:10903154-10904978 REVERSEmoderately similar to ( 472) loc_os02g55560 12002.m100396 protein DNA-binding protein phosphatase 2C, putative, expressed no original description 	T
33.99	development.unspecified	MDP0000198698	 highly similar to ( 798) AT5G52820 | Symbols:  | WD-40 repeat family protein / notchless protein, putative | chr5:21401423-21404203 FORWARDweakly similar to ( 115) GBLP_ORYSA Guanine nucleotide-binding protein subunit beta-like protein (GPB-LR) (RWD) - Oryza sativa (Rice)highly similar to ( 772) loc_os10g01540 12010.m21815 protein notchless homolog 1, putative, expressed no original description 	T
16.8.2	secondary metabolism.flavonoids.chalcones	MDP0000759336	 moderately similar to ( 296) AT3G55120 | Symbols: TT5, A11, CFI | TT5 (TRANSPARENT TESTA 5); chalcone isomerase | chr3:20430248-20431415 REVERSEmoderately similar to ( 325) CFI_VITVI Chalcone--flavonone isomerase (EC 5.5.1.6) (Chalcone isomerase) - Vitis vinifera (Grape)moderately similar to ( 265) loc_os03g60509 12003.m10939 protein expressed protein  no original description 	T
9.6	mitochondrial electron transport / ATP synthesis.cytochrome c	MDP0000262186	 moderately similar to ( 472) AT5G40810 | Symbols:  | cytochrome c1, putative | chr5:16340670-16342327 FORWARDmoderately similar to ( 453) CY12_SOLTU Cytochrome c1, heme protein, mitochondrial precursor (Clone PC18I) (Fragment) - Solanum tuberosum (Potato)moderately similar to ( 466) loc_os05g23620 12005.m64225 protein cytochrome c1, heme protein, mitochondrial precursor, putative, expressed no original description 	T
29.3.2	protein.targeting.mitochondria	MDP0000830613	 very weakly similar to (87.0) AT5G43970 | Symbols: TOM22-V, TOM9-2, ATTOM22-V | TOM22-V (TRANSLOCASE OF OUTER MEMBRANE 22-V); P-P-bond-hydrolysis-driven protein transmembrane transporter | chr5:17692888-17693187 FORWARDvery weakly similar to (76.3) loc_os02g29400 12002.m08084 protein mitochondrial import receptor subunit TOM22 homolog 2, putative, expressed no original description 	T
29.5	protein.degradation	MDP0000166885	 moderately similar to ( 351) AT5G22860 | Symbols:  | serine carboxypeptidase S28 family protein | chr5:7639907-7642945 REVERSEmoderately similar to ( 251) loc_os01g56150 12001.m11771 protein lysosomal Pro-X carboxypeptidase precursor, putative, expressed no original description 	T
15.2	metal handling.binding, chelation and storage	MDP0000908305	 moderately similar to ( 378) AT1G09240 | Symbols: NAS3, ATNAS3 | NAS3 (NICOTIANAMINE SYNTHASE 3); nicotianamine synthase | chr1:2984950-2985912 FORWARDmoderately similar to ( 306) NAS9_HORVU Nicotianamine synthase 9 (EC 2.5.1.43) (S-adenosyl-L-methionine:S-adenosyl-L-methionine:S-adenosyl-methionine 3-amino-3-carboxypropyltransferase 9) - Hordeum vulgare (Barley)moderately similar to ( 302) loc_os07g48980 12007.m09107 protein nicotianamine synthase 3, putative, expressed  no original description 	T
35.1	not assigned.no ontology	MDP0000649783	 weakly similar to ( 125) AT5G62150 | Symbols:  | peptidoglycan-binding LysM domain-containing protein | chr5:24958325-24958633 FORWARDweakly similar to ( 122) loc_os04g48380 12004.m09774 protein lysM domain containing protein no original description 	T
30.2.17	signalling.receptor kinases.DUF 26	MDP0000620420	 moderately similar to ( 359) AT1G66980 | Symbols:  | protein kinase family protein / glycerophosphoryl diester phosphodiesterase family protein | chr1:24997491-25001961 REVERSEweakly similar to ( 199) KPRO_MAIZE Putative receptor protein kinase ZmPK1 precursor (EC 2.7.11.1) - Zea mays (Maize)moderately similar to ( 385) loc_os01g49580 12001.m11145 protein protein kinase domain containing protein, expressed MSP1_C  no original description 	T
35.1.5	not assigned.no ontology.pentatricopeptide (PPR) repeat-containing protein	MDP0000140407	 highly similar to ( 721) AT1G19520 | Symbols: NFD5 | NFD5 (NUCLEAR FUSION DEFECTIVE 5) | chr1:6760032-6762581 FORWARDvery weakly similar to (97.1) RF1_ORYSA Rf1 protein, mitochondrial precursor (PPR protein) (Fertility restorer) (Restorer for CMS) - Oryza sativa (Rice)highly similar to ( 532) loc_os02g39820 12002.m09068 protein expressed protein   no original description 	T
35.2	not assigned.unknown	MDP0000159186	 moderately similar to ( 256) AT2G20930 | Symbols:  | FUNCTIONS IN: molecular_function unknown; INVOLVED IN: transport, ER to Golgi vesicle-mediated transport; LOCATED IN: intracellular; EXPRESSED IN: 24 plant structures; EXPRESSED DURING: 15 growth stages; CONTAINS InterPro DOMAIN/s: Sedlin (InterPro:IPR006722), Longin-like (InterPro:IPR011012); Has 370 Blast hits to 370 proteins in 116 species: Archae - 0; Bacteria - 0; Metazoa - 216; Fungi - 55; Plants - 47; Viruses - 0; Other Eukaryotes - 52 (source: NCBI BLink). | chr2:9000790-9001656 REVERSEmoderately similar to ( 261) loc_os09g36976 12009.m06647 protein hematopoietic stem/progenitor cells 176, putative, expressed   no original description 	T
9.1.2	mitochondrial electron transport / ATP synthesis.NADH-DH.localisation not clear	MDP0000178799	 moderately similar to ( 455) AT4G02580 | Symbols:  | NADH-ubiquinone oxidoreductase 24 kDa subunit, putative | chr4:1134586-1136906 FORWARDmoderately similar to ( 421) loc_os05g43360 12005.m08464 protein NADH-ubiquinone oxidoreductase 24 kDa subunit, mitochondrial precursor, putative, expressed  no original description 	T
27.1	RNA.processing	MDP0000290061	 weakly similar to ( 150) AT4G30220 | Symbols: RUXF | RUXF (SMALL NUCLEAR RIBONUCLEOPROTEIN F) | chr4:14803100-14804259 REVERSEweakly similar to ( 149) loc_os11g43620 12011.m28864 protein small nuclear ribonucleoprotein F, putative, expressed no original description 	T
29.4	protein.postranslational modification	MDP0000240040	 highly similar to ( 537) AT2G38620 | Symbols: CDKB1;2 | CDKB1;2 (cyclin-dependent kinase B1;2); cyclin binding / kinase/ protein serine/threonine kinase | chr2:16152551-16153866 FORWARDhighly similar to ( 556) CDC2C_ANTMA Cell division control protein 2 homolog C (EC 2.7.11.22) (EC 2.7.11.23) - Antirrhinum majus (Garden snapdragon)moderately similar to ( 464) loc_os01g67160 12001.m12819 protein cell division control protein 2 homolog C, putative, expressed  no original description 	T
35.2	not assigned.unknown	MDP0000916920	 moderately similar to ( 367) AT3G49720 | Symbols:  | unknown protein | chr3:18440192-18441655 REVERSEmoderately similar to ( 317) loc_os01g05070 12001.m42860 protein expressed protein no original description 	T
29.2.1.2.2.510	protein.synthesis.ribosomal protein.eukaryotic.60S subunit.L10A	MDP0000191873	 moderately similar to ( 311) AT5G22440 | Symbols:  | 60S ribosomal protein L10A (RPL10aC) | chr5:7435328-7436486 REVERSEmoderately similar to ( 227) RL10A_CHLRE 60S ribosomal protein L10a - Chlamydomonas reinhardtiimoderately similar to ( 317) loc_os01g64090 12001.m43287 protein 60S ribosomal protein L10a-1, putative, expressed  no original description 	T
4.2.14	glycolysis.plastid branch.pyruvate kinase (PK)	MDP0000173365	 highly similar to ( 853) AT5G52920 | Symbols: PKP1, PKP-BETA1, PKP2 | PKP-BETA1 (PLASTIDIC PYRUVATE KINASE BETA SUBUNIT 1); pyruvate kinase | chr5:21463680-21466612 FORWARDhighly similar to ( 682) KPYG_TOBAC Pyruvate kinase isozyme G, chloroplast precursor (EC 2.7.1.40) - Nicotiana tabacum (Common tobacco)highly similar to ( 828) loc_os01g47080 12001.m10904 protein pyruvate kinase isozyme G, chloroplast precursor, putative, expressed  PRK08026  no original description 	T
25.8	C1-metabolism.tetrahydrofolate synthase	MDP0000276411	 highly similar to ( 587) AT5G05980 | Symbols: ATDFB | ATDFB (A. THALIANA DHFS-FPGS HOMOLOG B); tetrahydrofolylpolyglutamate synthase | chr5:1799738-1804177 REVERSEhighly similar to ( 521) loc_os03g02030 12003.m101150 protein folylpolyglutamate synthase, mitochondrial precursor, putative, expressed   no original description 	T
35.1.5	not assigned.no ontology.pentatricopeptide (PPR) repeat-containing protein	MDP0000147925	 highly similar to ( 505) AT3G02650 | Symbols:  | pentatricopeptide (PPR) repeat-containing protein | chr3:566271-569865 FORWARDweakly similar to ( 116) RF1_ORYSA Rf1 protein, mitochondrial precursor (PPR protein) (Fertility restorer) (Restorer for CMS) - Oryza sativa (Rice)moderately similar to ( 384) loc_os01g67210 12001.m12824 protein expressed protein  no original description 	T
34.2	transport.sugars	MDP0000236004	 highly similar to ( 558) AT5G16150 | Symbols: GLT1, PGLCT | PGLCT (PLASTIDIC GLC TRANSLOCATOR); carbohydrate transmembrane transporter/ sugar:hydrogen symporter | chr5:5272904-5275678 FORWARDweakly similar to ( 159) STC_RICCO Sugar carrier protein C - Ricinus communis (Castor bean)highly similar to ( 537) loc_os01g04190 12001.m150473 protein arabinose-proton symporter, putative, expressed no original description 	T
27.1.19	RNA.processing.ribonucleases	MDP0000942514	 very weakly similar to (84.3) MAL12_MALDO Major allergen Mal d 1 (Mal d I) (AP15) - Malus domestica (Apple) (Malus sylvestris)very weakly similar to (55.5) loc_os03g18850 12003.m07305 protein pathogenesis-related protein 1, putative, expressed no original description 	T
20.2.99	stress.abiotic.unspecified	MDP0000942514	 very weakly similar to (84.3) MAL12_MALDO Major allergen Mal d 1 (Mal d I) (AP15) - Malus domestica (Apple) (Malus sylvestris)very weakly similar to (55.5) loc_os03g18850 12003.m07305 protein pathogenesis-related protein 1, putative, expressed no original description 	T
30.3	signalling.calcium	MDP0000718381	 nearly identical (1261) AT3G63380 | Symbols:  | calcium-transporting ATPase, plasma membrane-type, putative / Ca(2+)-ATPase, putative (ACA12) | chr3:23407112-23410213 REVERSEhighly similar to ( 727) ACA5_ORYSA Probable calcium-transporting ATPase 5, plasma membrane-type (EC 3.6.3.8) (Ca(2+)-ATPase isoform 5) - Oryza sativa (Rice)nearly identical (1017) loc_os10g28240 12010.m05706 protein calcium-transporting ATPase 13, plasma membrane-type, putative, expressed  no original description 	T
29.7	protein.glycosylation	MDP0000323739	 moderately similar to ( 475) AT1G53290 | Symbols:  | galactosyltransferase family protein | chr1:19871353-19873251 FORWARDmoderately similar to ( 430) loc_os06g06280 12006.m091613 protein beta-1,3-galactosyltransferase 6, putative, expressed no original description 	T
35.2	not assigned.unknown	MDP0000316683	 very weakly similar to (66.2) AT5G64680 | Symbols:  | unknown protein | chr5:25854433-25855331 FORWARDweakly similar to ( 108) loc_os08g32690 12008.m07261 protein expressed protein no original description 	T
35.1.1	not assigned.no ontology.ABC1 family protein	MDP0000148067	 highly similar to ( 700) AT3G07700 | Symbols:  | ABC1 family protein | chr3:2459696-2463241 REVERSEhighly similar to ( 674) loc_os09g07660 12009.m059938 protein ubiquinone biosynthesis protein ubiB, putative, expressed   no original description 	T
20.2.1	stress.abiotic.heat	MDP0000249262	 moderately similar to ( 329) AT2G20560 | Symbols:  | DNAJ heat shock family protein | chr2:8848353-8849815 REVERSEweakly similar to ( 186) DNJH_CUCSA DnaJ protein homolog (DNAJ-1) - Cucumis sativus (Cucumber)moderately similar to ( 285) loc_os02g20394 12002.m07281 protein dnaJ homolog subfamily B member 4, putative, expressed  no original description 	T
21.5	redox.peroxiredoxin	MDP0000258515	 moderately similar to ( 303) AT3G06050 | Symbols: PRXIIF, ATPRXIIF | PRXIIF (PEROXIREDOXIN IIF); antioxidant/ peroxidase | chr3:1826311-1827809 REVERSEmoderately similar to ( 281) loc_os01g16152 12001.m42520 protein peroxiredoxin-5, mitochondrial precursor, putative, expressed   no original description 	T
20.1	stress.biotic	MDP0000688401	 moderately similar to ( 211) AT1G78780 | Symbols:  | pathogenesis-related family protein | chr1:29621447-29622431 REVERSEmoderately similar to ( 229) PRPX_HORVU Pathogen-related protein - Hordeum vulgare (Barley)moderately similar to ( 246) loc_os01g14550 12001.m08059 protein pathogen-related protein, putative, expressed  no original description 	T
16.2	secondary metabolism.phenylpropanoids	MDP0000304620	 moderately similar to ( 386) AT4G31910 | Symbols:  | transferase family protein | chr4:15441125-15443696 FORWARDvery weakly similar to (93.6) HCBT1_DIACA Anthranilate N-benzoyltransferase protein 1 (EC 2.3.1.144) (Anthranilate N-hydroxycinnamoyl/benzoyltransferase 1) - Dianthus caryophyllus (Carnation) (Clove pink)weakly similar to ( 120) loc_os11g31090 12011.m07014 protein transferase, putative, expressed  no original description 	T
3.4.4	minor CHO metabolism.myo-inositol.myo inositol oxygenases	MDP0000287757	 moderately similar to ( 473) AT4G26260 | Symbols: MIOX4 | MIOX4; inositol oxygenase | chr4:13297939-13300146 FORWARDmoderately similar to ( 450) MIOX_ORYSA Probable inositol oxygenase (EC 1.13.99.1) (Myo-inositol oxygenase) - Oryza sativa (Rice)moderately similar to ( 450) loc_os06g36560 12006.m32098 protein inositol oxygenase, putative, expressed  no original description 	T
13.1.1.3.1	amino acid metabolism.synthesis.central amino acid metabolism.alanine.alanine aminotransferase	MDP0000250058	 highly similar to ( 878) AT1G70580 | Symbols: AOAT2, GGT2 | AOAT2 (ALANINE-2-OXOGLUTARATE AMINOTRANSFERASE 2); L-alanine:2-oxoglutarate aminotransferase/ glycine:2-oxoglutarate aminotransferase | chr1:26613222-26615845 FORWARDmoderately similar to ( 486) ALA2_HORVU Alanine aminotransferase 2 (EC 2.6.1.2) (GPT) (Glutamic--pyruvic transaminase 2) (Glutamic--alanine transaminase 2) (ALAAT-2) - Hordeum vulgare (Barley)highly similar to ( 855) loc_os07g01760 12007.m29036 protein alanine aminotransferase 2, putative, expressed no original description 	T
35.1.41	not assigned.no ontology.hydroxyproline rich proteins	MDP0000255495	 moderately similar to ( 376) AT1G54170 | Symbols: CID3 | CID3 (CTC-Interacting Domain 3) | chr1:20221353-20224919 REVERSEmoderately similar to ( 327) loc_os02g27950 12002.m07939 protein ataxin-2 N-terminal region family protein, expressed  no original description 	T
31.1	cell.organisation	MDP0000301930	 nearly identical (1680) AT5G65930 | Symbols: ZWI, PKCBP, KCBP | ZWI (ZWICHEL); calmodulin binding / microtubule motor | chr5:26370369-26376394 REVERSEmoderately similar to ( 231) FLA10_CHLRE Kinesin-like protein FLA10 (Protein KHP1) - Chlamydomonas reinhardtiinearly identical (1639) loc_os04g57140 12004.m10599 protein kinesin-like calmodulin binding protein, putative, expressed  no original description 	T
29.4	protein.postranslational modification	MDP0000297154	 highly similar to ( 660) AT5G19130 | Symbols:  | GPI transamidase component family protein / Gaa1-like family protein | chr5:6416128-6418993 REVERSEhighly similar to ( 573) loc_os01g48980 12001.m11087 protein GPI-anchor transamidase, putative, expressed   no original description 	T
5.10	fermentation.aldehyde dehydrogenase	MDP0000406860	 highly similar to ( 698) AT4G34240 | Symbols: ALDH3I1, ALDH3 | ALDH3I1 (ALDEHYDE DEHYDROGENASE 3|1); 3-chloroallyl aldehyde dehydrogenase/ aldehyde dehydrogenase (NAD) | chr4:16389801-16392633 FORWARDweakly similar to ( 157) BADH_SPIOL Betaine-aldehyde dehydrogenase, chloroplast precursor (EC 1.2.1.8) (BADH) - Spinacia oleracea (Spinach)highly similar to ( 634) loc_os11g08300 12011.m28749 protein aldehyde dehydrogenase, dimeric NADP-preferring, putative, expressed  no original description 	T
35.1	not assigned.no ontology	MDP0000648216	 moderately similar to ( 264) AT1G23550 | Symbols: SRO2 | SRO2 (SIMILAR TO RCD ONE 2); NAD+ ADP-ribosyltransferase | chr1:8350912-8352232 FORWARDweakly similar to ( 164) loc_os03g12820 12003.m35107 protein ATP8, putative, expressed no original description 	T
20.2.1	stress.abiotic.heat	MDP0000872143	 weakly similar to ( 105) AT2G17880 | Symbols:  | DNAJ heat shock protein, putative | chr2:7767176-7767658 REVERSEvery weakly similar to (53.1) DNJH_CUCSA DnaJ protein homolog (DNAJ-1) - Cucumis sativus (Cucumber)very weakly similar to (94.4) loc_os08g43490 12008.m08323 protein chaperone protein dnaJ 11, chloroplast precursor, putative, expressed no original description 	T
20	stress	MDP0000833745	 weakly similar to ( 128) AT4G34190 | Symbols: SEP1 | SEP1 (STRESS ENHANCED PROTEIN 1); chlorophyll binding | chr4:16372606-16373511 REVERSEvery weakly similar to (95.5) loc_os10g25570 12010.m21876 protein expressed protein no original description 	T
33.99	development.unspecified	MDP0000134838	 moderately similar to ( 395) AT5G46700 | Symbols: TET1, TRN2 | TRN2 (TORNADO 2) | chr5:18951035-18952439 FORWARDmoderately similar to ( 256) loc_os06g37510 12006.m08286 protein senescence-associated protein 5, putative, expressed no original description 	T
29.5.7	protein.degradation.metalloprotease	MDP0000146940	 nearly identical (1586) AT3G19170 | Symbols: ATPREP1, ATZNMP | ATPREP1 (PRESEQUENCE PROTEASE 1); metalloendopeptidase | chr3:6625578-6631874 REVERSEnearly identical (1532) loc_os02g52390 12002.m77746 protein protein hypA, putative, expressed  no original description 	T
29.4	protein.postranslational modification	MDP0000233137	 highly similar to ( 777) AT5G25510 | Symbols:  | serine/threonine protein phosphatase 2A (PP2A) regulatory subunit B', putative | chr5:8882728-8884325 REVERSEhighly similar to ( 669) loc_os05g48150 12005.m08886 protein transposon protein, putative, unclassified, expressed  no original description 	T
35.2	not assigned.unknown	MDP0000325781	   no original description 	T
30.3	signalling.calcium	MDP0000187969	 moderately similar to ( 202) AT2G15680 | Symbols:  | calmodulin-related protein, putative | chr2:6831024-6831587 FORWARDvery weakly similar to (85.1) CALM_CHLRE Calmodulin (CaM) - Chlamydomonas reinhardtiiweakly similar to ( 108) loc_os05g13580 12005.m05773 protein calmodulin-related protein 2, touch-induced, putative, expressed no original description 	T
26.3	misc.gluco-, galacto- and mannosidases	MDP0000217844	 highly similar to ( 828) AT3G18080 | Symbols: BGLU44 | BGLU44 (B-S GLUCOSIDASE 44); (R)-amygdalin beta-glucosidase/ 4-methylumbelliferyl-beta-D-glucopyranoside beta-glucosidase/ beta-gentiobiose beta-glucosidase/ cellobiose glucosidase/ esculin beta-glucosidase/ hydrolase, hydrolyzing O-glycosyl compounds | chr3:6191586-6194124 FORWARDmoderately similar to ( 394) BGLC_MAIZE Beta-glucosidase, chloroplast precursor (EC 3.2.1.21) (Gentiobiase) (Cellobiase) (Beta-D-glucoside glucohydrolase) - Zea mays (Maize)highly similar to ( 758) loc_os01g32364 12001.m09560 protein non-cyanogenic beta-glucosidase precursor, putative, expressed no original description 	T
26.8	misc.nitrilases, *nitrile lyases, berberine bridge enzymes, reticuline oxidases, troponine reductases	MDP0000748068	 moderately similar to ( 327) AT1G12570 | Symbols:  | glucose-methanol-choline (GMC) oxidoreductase family protein | chr1:4278192-4280753 REVERSEmoderately similar to ( 383) loc_os04g48400 12004.m09776 protein protein HOTHEAD precursor, putative, expressed  no original description 	T
35.2	not assigned.unknown	MDP0000147493	 weakly similar to ( 200) AT4G27390 | Symbols:  | unknown protein | chr4:13703417-13704655 REVERSEweakly similar to ( 149) loc_os05g42280 12005.m083771 protein expressed protein   no original description 	T
28.1.3	DNA.synthesis/chromatin structure.histone	MDP0000164957	 weakly similar to ( 181) AT1G54690 | Symbols: HTA3, H2AXB, G-H2AX, GAMMA-H2AX | GAMMA-H2AX (GAMMA HISTONE VARIANT H2AX); DNA binding | chr1:20414607-20415206 REVERSEweakly similar to ( 188) H2AX_CICAR Histone H2AX - Cicer arietinum (Chickpea) (Garbanzo)weakly similar to ( 174) loc_os12g34510 12012.m07245 protein histone H2A, putative, expressed  no original description 	T
27.3.10	RNA.regulation of transcription.C2C2(Zn) YABBY family	MDP0000192940	 moderately similar to ( 236) AT2G45190 | Symbols: AFO, FIL, YAB1 | AFO (ABNORMAL FLORAL ORGANS); protein binding / transcription factor/ transcription regulator | chr2:18628450-18630552 REVERSEweakly similar to ( 141) YAB1_ORYSA Protein YABBY (OsYAB1) (Filamentous flower protein 1) - Oryza sativa (Rice)moderately similar to ( 223) loc_os02g42950 12002.m33795 protein yabby15 protein, putative, expressed no original description 	T
27.3.22	RNA.regulation of transcription.HB,Homeobox transcription factor family	MDP0000943529	 nearly identical (1267) AT2G34710 | Symbols: PHB, ATHB14, ATHB-14, PHB-1D | PHB (PHABULOSA); DNA binding / transcription factor | chr2:14639548-14643993 REVERSEnearly identical (1276) loc_os03g43930 12003.m09434 protein class III HD-Zip protein 4, putative, expressed   no original description 	T
29.2.1.1.1.1.31	protein.synthesis.ribosomal protein.prokaryotic.chloroplast.30S subunit.S31	MDP0000796276	 very weakly similar to (97.1) AT2G38140 | Symbols: PSRP4 | PSRP4 (PLASTID-SPECIFIC RIBOSOMAL PROTEIN 4); structural constituent of ribosome | chr2:15980948-15981459 FORWARDvery weakly similar to (66.6) RR31_SPIOL 30S ribosomal protein S31, chloroplast precursor (Plastid-specific 30S ribosomal protein 4) (PSRP-4) - Spinacia oleracea (Spinach)very weakly similar to (72.4) loc_os05g09400 12005.m27555 protein PSRP4, putative, expressed no original description 	T
35.2	not assigned.unknown	MDP0000314698	 highly similar to ( 580) AT1G75110 | Symbols: RRA2 | RRA2 (REDUCED RESIDUAL ARABINOSE 2) | chr1:28194348-28195885 REVERSEhighly similar to ( 556) loc_os07g39840 12007.m08226 protein expressed protein   no original description 	T
29.5.3	protein.degradation.cysteine protease	MDP0000764121	 highly similar to ( 558) AT3G45310 | Symbols:  | cysteine proteinase, putative | chr3:16628704-16630473 REVERSEhighly similar to ( 526) ORYC_ORYSA Oryzain gamma chain precursor (EC 3.4.22.-) - Oryza sativa (Rice)highly similar to ( 526) loc_os09g27030 12009.m059986 protein thiol protease aleurain precursor, putative, expressed  no original description 	T
8.2.9	TCA / org transformation.other organic acid transformatons.cyt MDH	MDP0000532379	 weakly similar to ( 130) AT1G04410 | Symbols:  | malate dehydrogenase, cytosolic, putative | chr1:1189418-1191267 REVERSEweakly similar to ( 130) MDHC_MAIZE Malate dehydrogenase, cytoplasmic (EC 1.1.1.37) - Zea mays (Maize)weakly similar to ( 126) loc_os10g33800 12010.m21921 protein malate dehydrogenase, cytoplasmic, putative, expressed  no original description 	T
9.1.2	mitochondrial electron transport / ATP synthesis.NADH-DH.localisation not clear	MDP0000273948	 weakly similar to ( 149) AT5G47890 | Symbols:  | NADH-ubiquinone oxidoreductase B8 subunit, putative | chr5:19388806-19390409 FORWARDweakly similar to ( 134) loc_os04g24520 12004.m07583 protein NADH-ubiquinone oxidoreductase 10.5 kDa subunit, putative, expressed no original description 	T
31.1	cell.organisation	MDP0000151974	 highly similar to ( 699) AT5G27550 | Symbols:  | ATP binding / microtubule motor | chr5:9727634-9731323 REVERSEweakly similar to ( 133) K125_TOBAC 125 kDa kinesin-related protein - Nicotiana tabacum (Common tobacco)moderately similar to ( 462) loc_os05g33030 12005.m07535 protein kinesin-4, putative, expressed   no original description 	T
27.3.6	RNA.regulation of transcription.bHLH,Basic Helix-Loop-Helix family	MDP0000320691	 weakly similar to ( 125) AT5G39860 | Symbols: PRE1 | PRE1 (PACLOBUTRAZOL RESISTANCE1); DNA binding / transcription factor | chr5:15957689-15958303 FORWARDweakly similar to ( 102) loc_os10g26460 12010.m05586 protein DNA binding protein, putative no original description 	T
34	transport	MDP0000125469	 highly similar to ( 879) AT3G08960 | Symbols:  | binding / protein transporter | chr3:2730405-2736762 REVERSEhighly similar to ( 695) loc_os07g49260 12007.m09135 protein protein transporter, putative, expressed  no original description 	T
35.2	not assigned.unknown	MDP0000317465	 weakly similar to ( 145) AT2G26110 | Symbols:  | unknown protein | chr2:11120869-11121798 FORWARDvery weakly similar to (58.2) loc_os06g37020 12006.m08237 protein expressed protein  no original description 	T
19.7	tetrapyrrole synthesis.uroporphyrinogen decarboxylase	MDP0000269628	 highly similar to ( 520) AT2G40490 | Symbols: HEME2 | HEME2; uroporphyrinogen decarboxylase | chr2:16912961-16914988 FORWARDhighly similar to ( 555) DCUP_TOBAC Uroporphyrinogen decarboxylase, chloroplast precursor (EC 4.1.1.37) (URO-D) (UPD) - Nicotiana tabacum (Common tobacco)highly similar to ( 506) loc_os03g21900 12003.m07594 protein uroporphyrinogen decarboxylase, putative, expressed  no original description 	T
1.3.6	PS.calvin cycle.aldolase	MDP0000273688	 highly similar to ( 635) AT2G01140 | Symbols:  | fructose-bisphosphate aldolase, putative | chr2:95006-96491 REVERSEhighly similar to ( 572) ALFC_PEA Fructose-bisphosphate aldolase 1, chloroplast precursor (EC 4.1.2.13) (Fragment) - Pisum sativum (Garden pea)highly similar to ( 626) loc_os01g02880 12001.m06927 protein fructose-bisphosphate aldolase, chloroplast precursor, putative, expressed   no original description 	T
23.2	nucleotide metabolism.degradation	MDP0000208546	 highly similar to ( 728) AT4G04955 | Symbols: ATALN | ATALN (Arabidopsis allantoinase); allantoinase/ hydrolase | chr4:2522301-2525259 REVERSEmoderately similar to ( 290) loc_os04g58390 12004.m10721 protein allantoinase, putative, expressed  no original description 	T
35.1	not assigned.no ontology	MDP0000234325	 moderately similar to ( 428) AT1G32230 | Symbols:  | RCD1 (RADICAL-INDUCED CELL DEATH1); protein binding | chr1:11613427-11615748 FORWARDmoderately similar to ( 328) loc_os10g42710 12010.m21812 protein RCD1, putative, expressed  no original description 	T
35.1	not assigned.no ontology	MDP0000260408	 very weakly similar to (55.1) AT3G06880 | Symbols:  | nucleotide binding | chr3:2170589-2175686 REVERSEhighly similar to ( 577) loc_os02g07190 12002.m06066 protein expressed protein    no original description 	T
35.1	not assigned.no ontology	MDP0000260701	 moderately similar to ( 214) AT1G08370 | Symbols: DCP1, ATDCP1 | DCP1 (decapping 1); m7G(5')pppN diphosphatase/ protein homodimerization | chr1:2638355-2640367 FORWARDweakly similar to ( 194) loc_os11g05650 12011.m04762 protein dcp1-like decapping family protein, expressed   no original description 	T
27.3.4	RNA.regulation of transcription.ARF, Auxin Response Factor family	MDP0000153538	 highly similar to ( 984) AT1G30330 | Symbols: ARF6 | ARF6 (AUXIN RESPONSE FACTOR 6); transcription factor | chr1:10686125-10690036 REVERSEvery weakly similar to (54.3) IAA9_ORYSA Auxin-responsive protein IAA9 (Indoleacetic acid-induced protein 9) - Oryza sativa (Rice)highly similar to ( 995) loc_os02g06910 12002.m06038 protein auxin response factor 6, putative, expressed  no original description 	T
31.4	cell.vesicle transport	MDP0000318293	 weakly similar to ( 127) AT4G15790 | Symbols:  | unknown protein | chr4:8982390-8984069 FORWARDweakly similar to ( 114) loc_os07g09600 12007.m05415 protein vesicle-associated membrane protein 725, putative, expressed  no original description 	T
35.2	not assigned.unknown	MDP0000273148	 moderately similar to ( 404) AT5G26910 | Symbols:  | unknown protein | chr5:9466804-9469523 REVERSEmoderately similar to ( 382) loc_os03g19080 12003.m101318 protein expressed protein  no original description 	T
27.4	RNA.RNA binding	MDP0000441694	 moderately similar to ( 471) AT2G43410 | Symbols: FPA | FPA; RNA binding | chr2:18026397-18030989 REVERSEmoderately similar to ( 259) loc_os09g34070 12009.m22168 protein FPA, putative, expressed   no original description 	T
27.3.41	RNA.regulation of transcription.B3 transcription factor family	MDP0000238744	 moderately similar to ( 377) AT3G18990 | Symbols: VRN1, REM39 | VRN1 (REDUCED VERNALIZATION RESPONSE 1); transcription repressor | chr3:6549077-6551568 REVERSEweakly similar to ( 108) loc_os11g09160 12011.m05113 protein B3 DNA binding domain containing protein, expressed  no original description 	T
31.4	cell.vesicle transport	MDP0000899455	 weakly similar to ( 132) AT1G21380 | Symbols:  | VHS domain-containing protein / GAT domain-containing protein | chr1:7485806-7488032 REVERSEweakly similar to ( 118) loc_os11g09329 12011.m079960 protein VHS and GAT domain protein, putative, expressed  no original description 	T
34.19.2	transport.Major Intrinsic Proteins.TIP	MDP0000155416	 weakly similar to ( 191) AT4G01470 | Symbols: GAMMA-TIP3, TIP1;3 | TIP1;3 (TONOPLAST INTRINSIC PROTEIN 1;3); urea transmembrane transporter/ water channel | chr4:625092-625850 REVERSEweakly similar to ( 191) TIP12_ORYSA Probable aquaporin TIP1.2 (Tonoplast intrinsic protein 1.2) (OsTIP1.2) - Oryza sativa (Rice)weakly similar to ( 191) loc_os01g74450 12001.m13470 protein aquaporin TIP1.2, putative, expressed   no original description 	T
17.2.3	hormone metabolism.auxin.induced-regulated-responsive-activated	MDP0000228499	 highly similar to ( 540) AT1G60710 | Symbols: ATB2 | ATB2; oxidoreductase | chr1:22355073-22356627 REVERSEmoderately similar to ( 486) A115_TOBAC Auxin-induced protein PCNT115 - Nicotiana tabacum (Common tobacco)highly similar to ( 577) loc_os04g26910 12004.m07816 protein auxin-induced protein PCNT115, putative, expressed  no original description 	T
11.3.3	lipid metabolism.Phospholipid synthesis.phosphatidate cytidylyltransferase	MDP0000175026	 moderately similar to ( 254) AT5G58560 | Symbols:  | phosphatidate cytidylyltransferase family protein | chr5:23670513-23672570 FORWARDmoderately similar to ( 261) PHYK3_SOYBN Probable phytol kinase 3, chloroplast precursor (EC 2.7.-.-) - Glycine max (Soybean)moderately similar to ( 248) loc_os01g61560 12001.m43251 protein phytol kinase 2, chloroplast precursor, putative, expressed no original description 	T
31.2.5	cell.division.plastid	MDP0000330474	 highly similar to ( 532) AT2G21280 | Symbols: GC1, ATSULA, SULA | SULA; binding / catalytic/ coenzyme binding | chr2:9110502-9112679 REVERSEvery weakly similar to (53.5) DFRA_MAIZE Dihydroflavonol-4-reductase (EC 1.1.1.219) (DFR) (Dihydrokaempferol 4-reductase) - Zea mays (Maize)moderately similar to ( 461) loc_os02g58790 12002.m100430 protein expressed protein   no original description 	T
1.3.9	PS.calvin cycle.seduheptulose bisphosphatase	MDP0000244771	 highly similar to ( 627) AT3G55800 | Symbols: SBPASE | SBPASE (sedoheptulose-bisphosphatase); phosphoric ester hydrolase/ sedoheptulose-bisphosphatase | chr3:20709640-20711421 FORWARDhighly similar to ( 594) S17P_SPIOL Sedoheptulose-1,7-bisphosphatase, chloroplast precursor (EC 3.1.3.37) (Sedoheptulose-bisphosphatase) (SBPase) (SED(1,7)P2ase) - Spinacia oleracea (Spinach)highly similar to ( 579) loc_os04g16680 12004.m06845 protein sedoheptulose-1,7-bisphosphatase, chloroplast precursor, putative, expressed  no original description 	T
24.2	Biodegradation of Xenobiotics.lactoylglutathione lyase	MDP0000215478	 weakly similar to ( 164) AT1G15380 | Symbols:  | lactoylglutathione lyase family protein / glyoxalase I family protein | chr1:5290955-5292287 FORWARDweakly similar to ( 162) loc_os01g07850 12001.m07412 protein lactoylglutathione lyase, putative  no original description 	T
26.22	misc.short chain dehydrogenase/reductase (SDR)	MDP0000208027	 moderately similar to ( 269) AT4G09750 | Symbols:  | short-chain dehydrogenase/reductase (SDR) family protein | chr4:6146770-6148698 FORWARDmoderately similar to ( 249) loc_os06g39040 12006.m08442 protein retinol dehydrogenase 12, putative, expressed no original description 	T
29.4.1.57	protein.postranslational modification.kinase.receptor like cytoplasmatic kinase VII	MDP0000258353	 highly similar to ( 542) AT5G38560 | Symbols:  | protein kinase family protein | chr5:15439844-15443007 FORWARDmoderately similar to ( 263) PSKR_DAUCA Phytosulfokine receptor precursor (EC 2.7.11.1) (Phytosulfokine LRR receptor kinase) - Daucus carota (Carrot)moderately similar to ( 494) loc_os01g02040 12001.m42834 protein ATP binding protein, putative, expressed  TrbL  no original description 	T
29.4.1.57	protein.postranslational modification.kinase.receptor like cytoplasmatic kinase VII	MDP0000681462	 moderately similar to ( 417) AT2G07180 | Symbols:  | protein kinase, putative | chr2:2981082-2983271 REVERSEweakly similar to ( 182) CRI4_MAIZE Putative receptor protein kinase CRINKLY4 precursor (EC 2.7.11.1) - Zea mays (Maize)moderately similar to ( 390) loc_os06g45280 12006.m09055 protein protein kinase APK1B, chloroplast precursor, putative, expressed  no original description 	T
10.6.2	cell wall.degradation.mannan-xylose-arabinose-fucose	MDP0000709705	 highly similar to ( 665) AT2G20680 | Symbols:  | glycosyl hydrolase family 5 protein / cellulase family protein | chr2:8921024-8923066 FORWARDhighly similar to ( 518) loc_os01g54300 12001.m11594 protein hydrolase, hydrolyzing O-glycosyl compounds, putative, expressed  no original description 	T
1.2.5	PS.photorespiration.serine hydroxymethyltransferase	MDP0000132477	 moderately similar to ( 304) AT4G37930 | Symbols: SHM1, STM, SHMT1 | SHM1 (SERINE TRANSHYDROXYMETHYLTRANSFERASE 1); glycine hydroxymethyltransferase/ poly(U) binding | chr4:17831891-17834742 REVERSEmoderately similar to ( 318) GLYM_SOLTU Serine hydroxymethyltransferase, mitochondrial precursor (EC 2.1.2.1) (Serine methylase) (Glycine hydroxymethyltransferase) (SHMT) - Solanum tuberosum (Potato)moderately similar to ( 301) loc_os03g52840 12003.m101129 protein serine hydroxymethyltransferase, mitochondrial precursor, putative, expressed  no original description 	T
27.3.99	RNA.regulation of transcription.unclassified	MDP0000871812	 highly similar to ( 778) AT4G15080 | Symbols:  | zinc finger (DHHC type) family protein | chr4:8609085-8612229 REVERSEhighly similar to ( 588) loc_os09g38970 12009.m06837 protein metal ion binding protein, putative, expressed  no original description 	T
30.3	signalling.calcium	MDP0000290601	 moderately similar to ( 320) AT5G57580 | Symbols:  | calmodulin-binding protein | chr5:23314994-23317683 REVERSEmoderately similar to ( 312) loc_os02g35470 12002.m100044 protein calmodulin binding protein, putative, expressed   no original description 	T
30.11.1	signalling.light.COP9 signalosome	MDP0000145618	 highly similar to ( 582) AT1G71230 | Symbols: CSN5B, CSN5, AJH2 | CSN5B (COP9-SIGNALOSOME 5B); protein binding | chr1:26852560-26854255 FORWARDvery weakly similar to (75.5) CSN5B_BRAOL COP9 signalosome complex subunit 5b (EC 3.4.-.-) (Signalosome subunit 5b) (Jun activation domain-binding homolog 1) (Fragments) - Brassica oleracea (Wild cabbage)highly similar to ( 569) loc_os04g56070 12004.m101676 protein COP9 signalosome complex subunit 5b, putative, expressed   no original description 	T
33.99	development.unspecified	MDP0000145618	 highly similar to ( 582) AT1G71230 | Symbols: CSN5B, CSN5, AJH2 | CSN5B (COP9-SIGNALOSOME 5B); protein binding | chr1:26852560-26854255 FORWARDvery weakly similar to (75.5) CSN5B_BRAOL COP9 signalosome complex subunit 5b (EC 3.4.-.-) (Signalosome subunit 5b) (Jun activation domain-binding homolog 1) (Fragments) - Brassica oleracea (Wild cabbage)highly similar to ( 569) loc_os04g56070 12004.m101676 protein COP9 signalosome complex subunit 5b, putative, expressed   no original description 	T
35.1	not assigned.no ontology	MDP0000155311	 weakly similar to ( 117) AT2G18500 | Symbols: ATOFP7, OFP7 | OFP7 (ARABIDOPSIS THALIANA OVATE FAMILY PROTEIN 7) | chr2:8027102-8028049 FORWARDvery weakly similar to (84.7) loc_os10g38880 12010.m06655 protein plant-specific domain TIGR01568 family protein, expressed PRK08026 Sporozoite_P67  no original description 	T
27.3.4	RNA.regulation of transcription.ARF, Auxin Response Factor family	MDP0000634433	 weakly similar to ( 132) AT3G61830 | Symbols: ARF18 | ARF18 (AUXIN RESPONSE FACTOR 18); transcription factor | chr3:22888171-22891179 FORWARDvery weakly similar to (53.5) IAA20_ORYSA Auxin-responsive protein IAA20 (Indoleacetic acid-induced protein 20) - Oryza sativa (Rice)weakly similar to ( 106) loc_os02g35140 12002.m08602 protein auxin response factor 1, putative, expressed no original description 	T
27.3.67	RNA.regulation of transcription.putative transcription regulator	MDP0000153963	 moderately similar to ( 437) AT2G29570 | Symbols: PCNA2, ATPCNA2 | PCNA2 (PROLIFERATING CELL NUCLEAR ANTIGEN 2); DNA binding / DNA polymerase processivity factor | chr2:12650139-12651598 REVERSEmoderately similar to ( 457) PCNA_TOBAC Proliferating cell nuclear antigen (PCNA) - Nicotiana tabacum (Common tobacco)moderately similar to ( 433) loc_os02g56130 12002.m10637 protein proliferating cell nuclear antigen, putative, expressedMSP1_C  no original description 	T
35.1	not assigned.no ontology	MDP0000217095	 highly similar to ( 580) AT5G64500 | Symbols:  | membrane protein-related | chr5:25780511-25783474 FORWARDhighly similar to ( 521) loc_os04g44430 12004.m09402 protein carbohydrate transporter/ sugar porter/ transporter, putative, expressed  no original description 	T
31.1	cell.organisation	MDP0000194397	 nearly identical (1026) AT1G77550 | Symbols:  | protein binding / tubulin-tyrosine ligase | chr1:29138490-29142965 REVERSEhighly similar to ( 947) loc_os03g08140 12003.m06323 protein tubulin--tyrosine ligase-like protein 12, putative, expressed   no original description 	T
5.10	fermentation.aldehyde dehydrogenase	MDP0000140980	 highly similar to ( 866) AT3G48000 | Symbols: ALDH2B4, ALDH2, ALDH2A | ALDH2B4 (ALDEHYDE DEHYDROGENASE 2B4); 3-chloroallyl aldehyde dehydrogenase/ ATP binding / aldehyde dehydrogenase (NAD) | chr3:17717082-17719843 REVERSEmoderately similar to ( 370) BADH_BETVU Betaine-aldehyde dehydrogenase, chloroplast precursor (EC 1.2.1.8) (BADH) - Beta vulgaris (Sugar beet)highly similar to ( 847) loc_os06g15990 12006.m71425 protein aldehyde dehydrogenase, mitochondrial precursor, putative, expressed  no original description 	T
30.1	signalling.in sugar and nutrient physiology	MDP0000304854	 highly similar to ( 973) AT2G32400 | Symbols: GLR5, GLR3.7, ATGLR3.7 | GLR5 (GLUTAMATE RECEPTOR 5); intracellular ligand-gated ion channel | chr2:13752665-13756233 REVERSEhighly similar to ( 806) GLR31_ORYSA Glutamate receptor 3.1 precursor (Ligand-gated ion channel 3.1) - Oryza sativa (Rice)highly similar to ( 868) loc_os06g46670 12006.m32152 protein glutamate receptor 3.4 precursor, putative, expressed  no original description 	T
17.2.2	hormone metabolism.auxin.signal transduction	MDP0000250518	 moderately similar to ( 394) AT3G01280 | Symbols: VDAC1, ATVDAC1 | VDAC1 (VOLTAGE DEPENDENT ANION CHANNEL 1); voltage-gated anion channel | chr3:85754-87612 FORWARDmoderately similar to ( 432) VDAC1_SOLTU 34 kDa outer mitochondrial membrane protein porin (Voltage-dependent anion-selective channel protein) (VDAC) (POM 34) - Solanum tuberosum (Potato)moderately similar to ( 363) loc_os09g19734 12009.m50200 protein isochorismate synthase 1, chloroplast precursor, putative, expressed   no original description 	T
34.2	transport.sugars	MDP0000250518	 moderately similar to ( 394) AT3G01280 | Symbols: VDAC1, ATVDAC1 | VDAC1 (VOLTAGE DEPENDENT ANION CHANNEL 1); voltage-gated anion channel | chr3:85754-87612 FORWARDmoderately similar to ( 432) VDAC1_SOLTU 34 kDa outer mitochondrial membrane protein porin (Voltage-dependent anion-selective channel protein) (VDAC) (POM 34) - Solanum tuberosum (Potato)moderately similar to ( 363) loc_os09g19734 12009.m50200 protein isochorismate synthase 1, chloroplast precursor, putative, expressed   no original description 	T
19.3	tetrapyrrole synthesis.GSA	MDP0000174407	 highly similar to ( 739) AT5G63570 | Symbols: GSA1 | GSA1 (GLUTAMATE-1-SEMIALDEHYDE-2,1-AMINOMUTASE); glutamate-1-semialdehyde 2,1-aminomutase | chr5:25451957-25453620 FORWARDhighly similar to ( 743) GSA_BRANA Glutamate-1-semialdehyde 2,1-aminomutase, chloroplast precursor (EC 5.4.3.8) (GSA) (Glutamate-1-semialdehyde aminotransferase) (GSA-AT) - Brassica napus (Rape)highly similar to ( 696) loc_os08g41990 12008.m08177 protein glutamate-1-semialdehyde 2,1-aminomutase, chloroplast precursor, putative, expressed  no original description 	T
35.2	not assigned.unknown	MDP0000183935	 highly similar to ( 530) AT4G30060 | Symbols:  | unknown protein | chr4:14689420-14691519 REVERSEhighly similar to ( 511) loc_os06g45110 12006.m09038 protein expressed protein no original description 	T
1.1.2.1	PS.lightreaction.photosystem I.LHC-I	MDP0000260027	 very weakly similar to (77.0) AT3G47470 | Symbols: LHCA4, CAB4 | LHCA4 (LIGHT-HARVESTING CHLOROPHYLL-PROTEIN COMPLEX I SUBUNIT A4); chlorophyll binding | chr3:17493622-17494773 REVERSE  no original description 	T
35.1.20	not assigned.no ontology.formin homology 2 domain-containing protein	MDP0000667895	 highly similar to ( 514) AT3G25500 | Symbols: AFH1, FH1, AHF1, ATFH1 | AFH1 (FORMIN HOMOLOGY 1); actin binding / actin filament binding / protein binding | chr3:9251320-9254826 REVERSEhighly similar to ( 520) loc_os01g67240 12001.m12827 protein AFH1, putative, expressed no original description 	T
29.2.1.2.1.4	protein.synthesis.ribosomal protein.eukaryotic.40S subunit.S4	MDP0000148249	 moderately similar to ( 455) AT5G58420 | Symbols:  | 40S ribosomal protein S4 (RPS4D) | chr5:23619599-23620896 FORWARDmoderately similar to ( 453) RS4_SOLTU 40S ribosomal protein S4 - Solanum tuberosum (Potato)moderately similar to ( 449) loc_os02g01560 12002.m05506 protein 40S ribosomal protein S4, putative, expressed   no original description 	T
27.3.11	RNA.regulation of transcription.C2H2 zinc finger family	MDP0000373134	 moderately similar to ( 334) AT5G44160 | Symbols: NUC | NUC (nutcracker); nucleic acid binding / transcription factor/ zinc ion binding | chr5:17773091-17775513 FORWARDmoderately similar to ( 328) loc_os03g10140 12003.m06467 protein nucleic acid binding protein, putative, expressed  no original description 	T
28.1.3	DNA.synthesis/chromatin structure.histone	MDP0000332388	 moderately similar to ( 228) AT5G65360 | Symbols:  | histone H3 | chr5:26120099-26120509 REVERSEmoderately similar to ( 226) H32_WHEAT Histone H3.2 - Triticum aestivum (Wheat)moderately similar to ( 228) loc_os11g05730 12011.m04769 protein histone H3, putative, expressed  no original description 	T
35.2	not assigned.unknown	MDP0000201070	 moderately similar to ( 325) AT2G16990 | Symbols:  | tetracycline transporter | chr2:7383194-7387783 FORWARDweakly similar to ( 125) loc_os11g05390 12011.m04736 protein tetracycline transporter protein, putative, expressed  no original description 	T
27.3.25	RNA.regulation of transcription.MYB domain transcription factor family	MDP0000282792	 moderately similar to ( 388) AT2G37630 | Symbols: ATPHAN, AS1, ATMYB91, MYB91 | AS1 (ASYMMETRIC LEAVES 1); DNA binding / protein homodimerization/ transcription factor | chr2:15781798-15782901 REVERSEvery weakly similar to ( 100) GAM1_ORYSA Transcription factor GAMYB (OsGAMyb) - Oryza sativa (Rice)moderately similar to ( 326) loc_os12g38400 12012.m26938 protein protein ASYMMETRIC LEAVES1, putative, expressed  no original description 	T
20.2.99	stress.abiotic.unspecified	MDP0000529003	 very weakly similar to (81.3) AT1G20440 | Symbols: COR47, RD17 | COR47 (COLD-REGULATED 47) | chr1:7084722-7085664 REVERSEvery weakly similar to (66.6) ECP44_DAUCA Phosphoprotein ECPP44 - Daucus carota (Carrot)very weakly similar to (72.4) loc_os02g44870 12002.m09527 protein dehydrin COR410, putative, expressed   no original description 	T
10.5.1.1	cell wall.cell wall proteins.AGPs.AGP	MDP0000282305	 moderately similar to ( 435) AT4G12730 | Symbols: FLA2 | FLA2 (FASCICLIN-LIKE ARABINOGALACTAN 2) | chr4:7491598-7492809 REVERSEmoderately similar to ( 337) loc_os09g07350 12009.m04004 protein fasciclin-like arabinogalactan protein 8 precursor, putative, expressed   no original description 	T
35.2	not assigned.unknown	MDP0000261563	 moderately similar to ( 213) AT1G45688 | Symbols:  | unknown protein | chr1:17191502-17192870 FORWARDmoderately similar to ( 327) loc_os06g06780 12006.m05403 protein harpin-induced protein, putative, expressed   no original description 	T
35.2	not assigned.unknown	MDP0000252752	 weakly similar to ( 189) AT1G70950 | Symbols:  | FUNCTIONS IN: molecular_function unknown; INVOLVED IN: biological_process unknown; EXPRESSED IN: 6 plant structures; EXPRESSED DURING: 4 anthesis, petal differentiation and expansion stage, D bilateral stage, E expanded cotyledon stage; CONTAINS InterPro DOMAIN/s: Targeting for Xklp2 (InterPro:IPR009675); BEST Arabidopsis thaliana protein match is: unknown protein (TAIR:AT1G23060.1); Has 875 Blast hits to 771 proteins in 143 species: Archae - 0; Bacteria - 102; Metazoa - 281; Fungi - 65; Plants - 205; Viruses - 3; Other Eukaryotes - 219 (source: NCBI BLink). | chr1:26752779-26755242 FORWARDvery weakly similar to (73.6) loc_os02g10690 12002.m100132 protein seed specific protein Bn15D14A, putative, expressed  no original description 	T
35.2	not assigned.unknown	MDP0000132130	 moderately similar to ( 467) AT1G71110 | Symbols:  | unknown protein | chr1:26818244-26820852 FORWARDmoderately similar to ( 328) loc_os01g03110 12001.m06950 protein expressed protein no original description 	T
35.2	not assigned.unknown	MDP0000511481	 very weakly similar to (90.9) AT3G56870 | Symbols:  | unknown protein | chr3:21053077-21055518 FORWARDvery weakly similar to (75.5) loc_os03g39740 12003.m35305 protein expressed protein   no original description 	T
12.1.1	N-metabolism.nitrate metabolism.NR	MDP0000148564	 nearly identical (1373) AT1G77760 | Symbols: NIA1, GNR1, NR1 | NIA1 (NITRATE REDUCTASE 1); nitrate reductase | chr1:29236005-29239367 REVERSEnearly identical (1420) NIA_CUCMA Nitrate reductase [NADH] (EC 1.7.1.1) (NR) - Cucurbita maxima (Pumpkin) (Winter squash)nearly identical (1274) loc_os08g36480 12008.m07637 protein nitrate reductase 1, putative, expressed    no original description 	T
30.2.11	signalling.receptor kinases.leucine rich repeat XI	MDP0000182437	 weakly similar to ( 193) AT1G17240 | Symbols: AtRLP2 | AtRLP2 (Receptor Like Protein 2); protein binding / protein kinase | chr1:5896528-5898717 REVERSEweakly similar to ( 160) PSKR_DAUCA Phytosulfokine receptor precursor (EC 2.7.11.1) (Phytosulfokine LRR receptor kinase) - Daucus carota (Carrot)moderately similar to ( 212) loc_os02g05970 12002.m05945 protein phytosulfokine receptor precursor, putative, expressed  no original description 	T
16.8.2	secondary metabolism.flavonoids.chalcones	MDP0000361563	 very weakly similar to (92.8) AT5G13930 | Symbols: CHS, TT4, ATCHS | TT4 (TRANSPARENT TESTA 4); naringenin-chalcone synthase | chr5:4488762-4490035 FORWARDweakly similar to ( 102) CHSY_MALDO Chalcone synthase (EC 2.3.1.74) (Naregenin-chalcone synthase) (Fragment) - Malus domestica (Apple) (Malus sylvestris)very weakly similar to (88.6) loc_os11g32650 12011.m07166 protein chalcone synthase, putative, expressed no original description 	T
27.4	RNA.RNA binding	MDP0000656663	 moderately similar to ( 243) AT1G60000 | Symbols:  | 29 kDa ribonucleoprotein, chloroplast, putative / RNA-binding protein cp29, putative | chr1:22093704-22094540 REVERSEweakly similar to ( 140) ROC1_SPIOL 28 kDa ribonucleoprotein, chloroplast (28RNP) - Spinacia oleracea (Spinach)moderately similar to ( 254) loc_os02g57010 12002.m10723 protein ribonucleoprotein, chloroplast, putative, expressed   no original description 	T
21.2	redox.ascorbate and glutathione	MDP0000312256	 moderately similar to ( 271) AT5G38630 | Symbols: ACYB-1 | ACYB-1; carbon-monoxide oxygenase | chr5:15466141-15467511 FORWARDmoderately similar to ( 229) loc_os08g29520 12008.m06956 protein cytochrome b561, putative, expressed  no original description 	T
35.2	not assigned.unknown	MDP0000438765	 very weakly similar to (85.9) AT4G13870 | Symbols: WRNEXO | WRNEXO (WERNER SYNDROME-LIKE EXONUCLEASE); 3'-5' exonuclease/ nucleic acid binding / protein binding | chr4:8023563-8025542 REVERSEweakly similar to ( 143) loc_os01g47180 12001.m10913 protein 3-5 exonuclease/ nucleic acid binding protein, putative, expressed   no original description 	T
12.1.2	N-metabolism.nitrate metabolism.nitrite reductase	MDP0000722117	 highly similar to ( 923) AT2G15620 | Symbols: NIR1, NIR, ATHNIR | NIR1 (NITRITE REDUCTASE 1); ferredoxin-nitrate reductase/ nitrite reductase (NO-forming) | chr2:6810552-6812666 FORWARDhighly similar to ( 957) NIR_BETVE Ferredoxin--nitrite reductase, chloroplast precursor (EC 1.7.7.1) - Betula verrucosa (White birch) (Betula pendula)highly similar to ( 855) loc_os01g25484 12001.m43466 protein ferredoxin--nitrite reductase, chloroplast precursor, putative, expressed    no original description 	T
23.1.2.5	nucleotide metabolism.synthesis.purine.AIR synthase	MDP0000153621	 moderately similar to ( 473) AT3G55010 | Symbols: ATPURM, PUR5 | PUR5; ATP binding / phosphoribosylformylglycinamidine cyclo-ligase | chr3:20386818-20388549 FORWARDmoderately similar to ( 490) PUR5_VIGUN Phosphoribosylformylglycinamidine cyclo-ligase, chloroplast/mitochondrial precursor (EC 6.3.3.1) (AIRS) (Phosphoribosyl-aminoimidazole synthetase) (AIR synthase) (VUpur5) - Vigna unguiculata (Cowpea)moderately similar to ( 467) loc_os03g61600 12003.m11041 protein phosphoribosylformylglycinamidine cyclo-ligase,chloroplast/mitochondrial precursor, putative, expressed PRK08026  no original description 	T
35.2	not assigned.unknown	MDP0000321414	 moderately similar to ( 265) AT1G80690 | Symbols:  | unknown protein | chr1:30329283-30330524 REVERSEmoderately similar to ( 218) loc_os03g10200 12003.m35075 protein expressed protein  no original description 	T
34.12	transport.metal	MDP0000148742	 highly similar to ( 523) AT3G53720 | Symbols: ATCHX20, CHX20 | ATCHX20 (CATION/H+ EXCHANGER 20); monovalent cation:proton antiporter/ sodium:hydrogen antiporter | chr3:19905826-19910027 REVERSEmoderately similar to ( 462) loc_os05g02240 12005.m04760 protein ATCHX20, putative, expressed  no original description 	T
10.8.1	cell wall.pectin*esterases.PME	MDP0000168682	 highly similar to ( 652) AT1G53840 | Symbols: ATPME1 | ATPME1; pectinesterase | chr1:20101533-20103458 FORWARDhighly similar to ( 692) PME3_PHAVU Pectinesterase-3 precursor (EC 3.1.1.11) (Pectin methylesterase 3) (PE 3) - Phaseolus vulgaris (Kidney bean) (French bean)moderately similar to ( 375) loc_os04g38560 12004.m08845 protein pectinesterase-1 precursor, putative, expressed  CobN MSP1_C  no original description 	T
29.3.4.99	protein.targeting.secretory pathway.unspecified	MDP0000720186	 highly similar to ( 874) AT2G34250 | Symbols:  | protein transport protein sec61, putative | chr2:14462635-14464572 FORWARDhighly similar to ( 889) loc_os09g17840 12009.m05042 protein protein transport protein Sec61 alpha subunit isoform 2, putative, expressed  no original description 	T
30.5	signalling.G-proteins	MDP0000701112	 moderately similar to ( 231) AT1G52380 | Symbols:  | Ran-binding protein 1 domain-containing protein / RanBP1 domain-containing protein | chr1:19509979-19511301 FORWARDweakly similar to ( 193) loc_os05g28190 12005.m07106 protein ranBP1 domain containing protein, expressed   no original description 	T
29.3.4.2	protein.targeting.secretory pathway.golgi	MDP0000304608	 nearly identical (1226) AT3G07100 | Symbols:  | protein transport protein Sec24, putative | chr3:2245689-2250077 REVERSEnearly identical (1077) loc_os04g04020 12004.m05709 protein protein transport protein Sec24-like, putative, expressed   no original description 	T
33.99	development.unspecified	MDP0000147154	 nearly identical (1090) AT3G09090 | Symbols: DEX1 | DEX1 (DEFECTIVE IN EXINE FORMATION 1); calcium ion binding | chr3:2783165-2787552 REVERSEnearly identical (1004) loc_os03g61050 12003.m10989 protein DEX1, putative, expressed  no original description 	T
30.2.11	signalling.receptor kinases.leucine rich repeat XI	MDP0000297928	 nearly identical (1157) AT2G24230 | Symbols:  | leucine-rich repeat transmembrane protein kinase, putative | chr2:10301979-10304540 REVERSEmoderately similar to ( 289) PSKR_DAUCA Phytosulfokine receptor precursor (EC 2.7.11.1) (Phytosulfokine LRR receptor kinase) - Daucus carota (Carrot)highly similar to ( 881) loc_os08g17410 12008.m05860 protein BRASSINOSTEROID INSENSITIVE 1 precursor, putative, expressed   no original description 	T
35.2	not assigned.unknown	MDP0000208497	 weakly similar to ( 105) AT4G39900 | Symbols:  | unknown protein | chr4:18508569-18510289 REVERSE  no original description 	T
35.2	not assigned.unknown	MDP0000183257	 very weakly similar to (87.8) AT5G26230 | Symbols:  | unknown protein | chr5:9173517-9174542 REVERSEvery weakly similar to (67.4) loc_os05g32440 12005.m07477 protein expressed protein  no original description 	T
11.1.7	lipid metabolism.FA synthesis and FA elongation.ACP thioesterase	MDP0000943090	 moderately similar to ( 480) AT3G25110 | Symbols: AtFaTA | AtFaTA (Arabidopsis FatA acyl-ACP thioesterase); acyl carrier/ acyl-[acyl-carrier-protein] hydrolase | chr3:9146589-9148273 REVERSEmoderately similar to ( 242) FATB_GOSHI Myristoyl-acyl carrier protein thioesterase, chloroplast precursor (EC 3.1.2.-) (16:0-acyl-carrier protein thioesterase) (16:0-ACP thioesterase) (Acyl-[acyl-carrier-protein] hydrolase) (PATE) - Gossypium hirsutum (Upland cotton)moderately similar to ( 459) loc_os09g32760 12009.m060080 protein oleoyl-acyl carrier protein thioesterase, chloroplast precursor, putative, expressed  no original description 	T
13.1.1.2.1	amino acid metabolism.synthesis.central amino acid metabolism.aspartate.aspartate aminotransferase	MDP0000243374	 highly similar to ( 685) AT4G31990 | Symbols: ASP5, AAT3, ATAAT1 | ASP5 (ASPARTATE AMINOTRANSFERASE 5); L-aspartate:2-oxoglutarate aminotransferase | chr4:15470876-15473521 REVERSEmoderately similar to ( 428) AATC_ORYSA Aspartate aminotransferase, cytoplasmic (EC 2.6.1.1) (Transaminase A) - Oryza sativa (Rice)highly similar to ( 661) loc_os02g55420 12002.m10568 protein aspartate aminotransferase, chloroplast precursor, putative, expressed   no original description 	T
26.11	misc.alcohol dehydrogenases	MDP0000278916	 highly similar to ( 540) AT1G32780 | Symbols:  | alcohol dehydrogenase, putative | chr1:11869977-11872595 REVERSEmoderately similar to ( 408) ADH1_PETHY Alcohol dehydrogenase 1 (EC 1.1.1.1) - Petunia hybrida (Petunia)highly similar to ( 515) loc_os10g07229 12010.m04111 protein alcohol dehydrogenase 2, putative, expressed  no original description 	T
35.2	not assigned.unknown	MDP0000378517	 very weakly similar to (62.0) AT4G00525 | Symbols:  | unknown protein | chr4:231862-232769 FORWARD no original description 	T
27.3.67	RNA.regulation of transcription.putative transcription regulator	MDP0000545581	 highly similar to ( 711) AT1G56110 | Symbols: NOP56 | NOP56 (Arabidopsis homolog of nucleolar protein Nop56) | chr1:20984544-20986893 REVERSEhighly similar to ( 684) loc_os03g22880 12003.m07686 protein nucleolar protein Nop56, putative, expressed no original description 	T
4.1.8	glycolysis.cytosolic branch.glyceraldehyde 3-phosphate dehydrogenase (GAP-DH)	MDP0000198482	 highly similar to ( 561) AT1G16300 | Symbols: GAPCP-2 | GAPCP-2; NAD or NADH binding / binding / catalytic/ glyceraldehyde-3-phosphate dehydrogenase (phosphorylating)/ glyceraldehyde-3-phosphate dehydrogenase | chr1:5574433-5577406 FORWARDmoderately similar to ( 435) G3PC_GINBI Glyceraldehyde-3-phosphate dehydrogenase, cytosolic (EC 1.2.1.12) - Ginkgo biloba (Ginkgo)highly similar to ( 540) loc_os02g07490 12002.m06096 protein glyceraldehyde-3-phosphate dehydrogenase, cytosolic, putative, expressed  no original description 	T
17.2.3	hormone metabolism.auxin.induced-regulated-responsive-activated	MDP0000120398	 moderately similar to ( 410) AT4G17280 | Symbols:  | INVOLVED IN: multicellular organismal development; LOCATED IN: membrane; EXPRESSED IN: 11 plant structures; EXPRESSED DURING: 6 growth stages; CONTAINS InterPro DOMAIN/s: Uncharacterised conserved protein UCP037471 (InterPro:IPR017214), Protein of unknown function DUF568, DOMON-like (InterPro:IPR007613), DOMON related (InterPro:IPR005018), Cytochrome b561/ferric reductase transmembrane (InterPro:IPR006593); BEST Arabidopsis thaliana protein match is: auxin-responsive protein, putative (TAIR:AT5G47530.1); Has 203 Blast hits to 203 proteins in 14 species: Archae - 0; Bacteria - 0; Metazoa - 0; Fungi - 0; Plants - 203; Viruses - 0; Other Eukaryotes - 0 (source: NCBI BLink). | chr4:9678887-9680277 REVERSEmoderately similar to ( 349) loc_os08g41280 12008.m08106 protein membrane protein, putative, expressed   no original description 	T
29.5	protein.degradation	MDP0000051968	 nearly identical (1540) AT1G63770 | Symbols:  | peptidase M1 family protein | chr1:23657791-23663476 REVERSEnearly identical (1431) loc_os08g44860 12008.m080051 protein aminopeptidase N, putative, expressed   no original description 	T
29.2.2.3.3	protein.synthesis.ribosome biogenesis.Pre-rRNA processing and modifications.methylotransferases	MDP0000239646	 moderately similar to ( 452) AT4G25630 | Symbols: FIB2, ATFIB2 | FIB2 (FIBRILLARIN 2); snoRNA binding | chr4:13074239-13076205 FORWARDmoderately similar to ( 432) loc_os02g57590 12002.m33482 protein fibrillarin-2, putative, expressed    no original description 	T
27.3.99	RNA.regulation of transcription.unclassified	MDP0000210668	 moderately similar to ( 303) AT5G07900 | Symbols:  | mitochondrial transcription termination factor family protein / mTERF family protein | chr5:2520188-2521405 FORWARDweakly similar to ( 164) loc_os05g33460 12005.m083717 protein mTERF family protein, expressed  no original description 	T
26.2	misc.UDP glucosyl and glucoronyl transferases	MDP0000818140	 moderately similar to ( 414) AT3G50740 | Symbols: UGT72E1 | UGT72E1 (UDP-glucosyl transferase 72E1); UDP-glycosyltransferase/ coniferyl-alcohol glucosyltransferase/ transferase, transferring glycosyl groups | chr3:18855348-18856811 REVERSEweakly similar to ( 160) ZOX_PHAVU Zeatin O-xylosyltransferase (EC 2.4.2.40) (Zeatin O-beta-D-xylosyltransferase) - Phaseolus vulgaris (Kidney bean) (French bean)moderately similar to ( 290) loc_os11g38650 12011.m07713 protein hydroquinone glucosyltransferase, putative, expressed  no original description 	T
29.2.1.1.1.2.34	protein.synthesis.ribosomal protein.prokaryotic.chloroplast.50S subunit.L34	MDP0000179766	 very weakly similar to (53.1) RK34_SPIOL 50S ribosomal protein L34, chloroplast precursor (CL34) - Spinacia oleracea (Spinach)  no original description 	T
26.3.5	misc.gluco-, galacto- and mannosidases.glycosyl hydrolase family 5	MDP0000729817	 highly similar to ( 622) AT1G13130 | Symbols:  | glycosyl hydrolase family 5 protein / cellulase family protein | chr1:4474726-4477820 FORWARDhighly similar to ( 592) loc_os04g40510 12004.m101557 protein hydrolase, hydrolyzing O-glycosyl compounds, putative, expressed   no original description 	T
20.1	stress.biotic	MDP0000292031	 moderately similar to ( 350) AT4G11650 | Symbols: ATOSM34 | ATOSM34 (osmotin 34) | chr4:7025127-7026113 REVERSEmoderately similar to ( 364) P21_SOYBN Protein P21 - Glycine max (Soybean)moderately similar to ( 327) loc_os03g46070 12003.m09627 protein protein P21, putative, expressed  no original description 	T
27.2	RNA.transcription	MDP0000231797	 moderately similar to ( 206) AT4G16265 | Symbols: NRPB9B, NRPD9B, NRPE9B | NRPB9B; DNA binding / DNA-directed RNA polymerase/ nucleic acid binding / transcription regulator/ zinc ion binding | chr4:9202529-9203975 REVERSEmoderately similar to ( 204) loc_os12g37370 12012.m07522 protein DNA-directed RNA polymerase II 14.5 kDa polypeptide, putative, expressed  no original description 	T
8.2.9	TCA / org transformation.other organic acid transformatons.cyt MDH	MDP0000278198	 highly similar to ( 642) AT5G58330 | Symbols:  | malate dehydrogenase (NADP), chloroplast, putative | chr5:23580010-23582287 REVERSEhighly similar to ( 659) MDHP_PEA Malate dehydrogenase [NADP], chloroplast precursor (EC 1.1.1.82) (NADP-MDH) - Pisum sativum (Garden pea)highly similar to ( 639) loc_os08g44810 12008.m08453 protein malate dehydrogenase 1, chloroplast precursor, putative, expressed  no original description 	T
34.12	transport.metal	MDP0000152673	 moderately similar to ( 270) AT2G46800 | Symbols: ZAT, ATMTP1, MTP1, ZAT1, ATCDF1 | ZAT (ZINC TRANSPORTER OF ARABIDOPSIS THALIANA); inorganic anion transmembrane transporter/ metal ion transmembrane transporter/ zinc ion transmembrane transporter | chr2:19238128-19239324 FORWARDmoderately similar to ( 255) loc_os05g03780 12005.m04912 protein metal tolerance protein A2, putative, expressed  no original description 	T
20.2.3	stress.abiotic.drought/salt	MDP0000203574	 highly similar to ( 647) AT4G18030 | Symbols:  | dehydration-responsive family protein | chr4:10012850-10015267 REVERSEhighly similar to ( 589) loc_os09g24900 12009.m22086 protein ankyrin protein kinase-like, putative, expressed no original description 	T
26.28	misc.GDSL-motif lipase	MDP0000476508	 moderately similar to ( 317) AT5G40990 | Symbols: GLIP1 | GLIP1 (GDSL LIPASE1); carboxylesterase/ lipase | chr5:16418920-16420400 FORWARDweakly similar to ( 120) APG_BRANA Anter-specific proline-rich protein APG (Protein CEX) (Fragment) - Brassica napus (Rape)weakly similar to ( 160) loc_os09g04710 12009.m21966 protein anther-specific proline-rich protein APG precursor, putative, expressed no original description 	T
21.2	redox.ascorbate and glutathione	MDP0000266448	 weakly similar to ( 168) AT2G24940 | Symbols: AtMAPR2 | AtMAPR2 (Arabidopsis thaliana membrane-associated progesterone binding protein 2); heme binding | chr2:10609447-10609749 FORWARDweakly similar to ( 140) loc_os02g55060 12002.m100058 protein membrane steroid-binding protein 1, putative, expressed no original description 	T
21.4	redox.glutaredoxins	MDP0000725469	 weakly similar to ( 166) AT3G62950 | Symbols:  | glutaredoxin family protein | chr3:23266303-23266614 FORWARDvery weakly similar to (70.1) GLRX_RICCO Glutaredoxin - Ricinus communis (Castor bean)weakly similar to ( 107) loc_os12g35330 12012.m07326 protein OsGrx_C12 - glutaredoxin subgroup III no original description 	T
35.2	not assigned.unknown	MDP0000609131	 moderately similar to ( 399) AT5G63910 | Symbols: FCLY | FCLY (FARNESYLCYSTEINE LYASE); prenylcysteine oxidase | chr5:25572053-25574077 REVERSEvery weakly similar to ( 100) loc_os04g59630 12004.m10838 protein prenylcysteine oxidase precursor, putative, expressed   no original description 	T
28.1.3	DNA.synthesis/chromatin structure.histone	MDP0000581822	 moderately similar to ( 262) AT5G65360 | Symbols:  | histone H3 | chr5:26120099-26120509 REVERSEmoderately similar to ( 261) H32_WHEAT Histone H3.2 - Triticum aestivum (Wheat)moderately similar to ( 262) loc_os11g05730 12011.m04769 protein histone H3, putative, expressed no original description 	T
31.4	cell.vesicle transport	MDP0000136087	 nearly identical (1182) AT1G60070 | Symbols:  | binding / clathrin binding / protein binding / protein transporter | chr1:22142944-22149296 REVERSEnearly identical (1090) loc_os06g07090 12006.m091619 protein AP-1 complex subunit gamma-1, expressed  no original description 	T
31.1	cell.organisation	MDP0000175196	 moderately similar to ( 231) AT3G46010 | Symbols: ADF1, atadf, ATADF1 | ADF1 (ACTIN DEPOLYMERIZING FACTOR 1); actin binding | chr3:16909679-16910678 REVERSEmoderately similar to ( 240) ADF2_PETHY Actin-depolymerizing factor 2 (ADF 2) - Petunia hybrida (Petunia)moderately similar to ( 234) loc_os05g02250 12005.m64199 protein expressed protein no original description 	T
26.10	misc.cytochrome P450	MDP0000836708	 highly similar to ( 613) AT2G40890 | Symbols: CYP98A3 | CYP98A3 (cytochrome P450, family 98, subfamily A, polypeptide 3); monooxygenase/ p-coumarate 3-hydroxylase | chr2:17058291-17060532 REVERSEhighly similar to ( 630) C98A2_SOYBN Cytochrome P450 98A2 (EC 1.14.-.-) - Glycine max (Soybean)moderately similar to ( 483) loc_os05g41440 12005.m27896 protein cytochrome P450 98A1, putative, expressed  no original description 	T
15.1	metal handling.acquisition	MDP0000299273	 moderately similar to ( 436) AT5G49740 | Symbols: ATFRO7, FRO7 | ATFRO7 (FERRIC REDUCTION OXIDASE 7); ferric-chelate reductase/ oxidoreductase | chr5:20205549-20208628 REVERSEmoderately similar to ( 447) loc_os04g36720 12004.m08712 protein ferric reductase-like transmembrane component, putative, expressed  no original description 	T
35.2	not assigned.unknown	MDP0000281697	 moderately similar to ( 284) AT2G02590 | Symbols:  | FUNCTIONS IN: molecular_function unknown; INVOLVED IN: biological_process unknown; LOCATED IN: chloroplast; EXPRESSED IN: 21 plant structures; EXPRESSED DURING: 13 growth stages; CONTAINS InterPro DOMAIN/s: Putative small multi-drug export (InterPro:IPR009577); Has 213 Blast hits to 213 proteins in 89 species: Archae - 35; Bacteria - 146; Metazoa - 0; Fungi - 0; Plants - 15; Viruses - 0; Other Eukaryotes - 17 (source: NCBI BLink). | chr2:704977-706494 FORWARDmoderately similar to ( 255) loc_os03g58620 12003.m10767 protein small multi-drug export protein, putative, expressed  no original description 	T
20.1.3.1	stress.biotic.signalling.MLO-like	MDP0000208730	 moderately similar to ( 255) AT3G45290 | Symbols: MLO3, ATMLO3 | MLO3 (MILDEW RESISTANCE LOCUS O 3); calmodulin binding | chr3:16617586-16621352 FORWARDweakly similar to ( 188) MLOH1_ORYSA MLO protein homolog 1 (OsMLO1) - Oryza sativa (Rice)moderately similar to ( 211) loc_os10g39520 12010.m06723 protein MLO-like protein 10, putative, expressed  no original description 	T
35.2	not assigned.unknown	MDP0000949591	 very weakly similar to (75.5) AT5G40460 | Symbols:  | unknown protein | chr5:16202465-16202803 FORWARD no original description 	T
35.2	not assigned.unknown	MDP0000285798	 highly similar to ( 623) AT5G45540 | Symbols:  | unknown protein | chr5:18458294-18460705 REVERSEmoderately similar to ( 488) loc_os01g72380 12001.m13273 protein expressed protein  no original description 	T
34.99	transport.misc	MDP0000501447	 moderately similar to ( 281) AT1G14010 | Symbols:  | emp24/gp25L/p24 family protein | chr1:4800385-4801790 REVERSEmoderately similar to ( 281) loc_os07g43890 12007.m08613 protein transmembrane emp24 domain-containing protein 10 precursor, putative, expressed no original description 	T
16.2.1.10	secondary metabolism.phenylpropanoids.lignin biosynthesis.CAD	MDP0000241504	 highly similar to ( 582) AT5G63620 | Symbols:  | oxidoreductase, zinc-binding dehydrogenase family protein | chr5:25466380-25468296 REVERSEweakly similar to ( 133) ADH_MALDO Alcohol dehydrogenase (EC 1.1.1.1) - Malus domestica (Apple) (Malus sylvestris)highly similar to ( 531) loc_os08g01760 12008.m26554 protein S-glutathione dehydrogenase/class III alcohol dehydrogenase, putative, expressed  no original description 	T
26.11	misc.alcohol dehydrogenases	MDP0000241504	 highly similar to ( 582) AT5G63620 | Symbols:  | oxidoreductase, zinc-binding dehydrogenase family protein | chr5:25466380-25468296 REVERSEweakly similar to ( 133) ADH_MALDO Alcohol dehydrogenase (EC 1.1.1.1) - Malus domestica (Apple) (Malus sylvestris)highly similar to ( 531) loc_os08g01760 12008.m26554 protein S-glutathione dehydrogenase/class III alcohol dehydrogenase, putative, expressed  no original description 	T
35.2	not assigned.unknown	MDP0000269985	 highly similar to ( 646) AT3G19895 | Symbols:  | unknown protein | chr3:6917530-6921211 REVERSEmoderately similar to ( 486) loc_os02g58280 12002.m10849 protein expressed protein   SPT5  no original description 	T
35.1.41	not assigned.no ontology.hydroxyproline rich proteins	MDP0000179769	 moderately similar to ( 336) AT4G18570 | Symbols:  | proline-rich family protein | chr4:10231439-10234534 FORWARDmoderately similar to ( 321) loc_os01g46340 12001.m10832 protein pherophorin like protein, putative, expressed   no original description 	T
35.2	not assigned.unknown	MDP0000941476	 moderately similar to ( 241) AT5G66230 | Symbols:  | unknown protein | chr5:26462704-26463873 FORWARDweakly similar to ( 148) loc_os10g42660 12010.m07019 protein expressed protein   no original description 	T
26.8	misc.nitrilases, *nitrile lyases, berberine bridge enzymes, reticuline oxidases, troponine reductases	MDP0000157943	 highly similar to ( 583) AT1G30700 | Symbols:  | FAD-binding domain-containing protein | chr1:10892623-10894437 FORWARDmoderately similar to ( 410) loc_os06g45990 12006.m32146 protein patellin-5, putative, expressed  TrbL MSP1_C  no original description 	T
35.2	not assigned.unknown	MDP0000594737	   no original description 	T
29.2.1.1.1.1.17	protein.synthesis.ribosomal protein.prokaryotic.chloroplast.30S subunit.S17	MDP0000597906	 weakly similar to ( 149) AT1G79850 | Symbols: RPS17, CS17, PRPS17 | RPS17 (RIBOSOMAL PROTEIN S17); structural constituent of ribosome | chr1:30041473-30041922 REVERSEweakly similar to ( 120) RR17_PEA 30S ribosomal protein S17, chloroplast precursor (CS17) (Fragment) - Pisum sativum (Garden pea)very weakly similar to (97.1) loc_os04g59494 12004.m35538 protein transportin-2, putative, expressed   no original description 	T
21.4	redox.glutaredoxins	MDP0000155448	 weakly similar to ( 144) AT3G62930 | Symbols:  | glutaredoxin family protein | chr3:23261538-23261846 REVERSEvery weakly similar to (60.5) GLRX_RICCO Glutaredoxin - Ricinus communis (Castor bean)weakly similar to ( 108) loc_os01g27140 12001.m09165 protein glutaredoxin, putative, expressed   no original description 	T
35.1	not assigned.no ontology	MDP0000136296	 moderately similar to ( 363) AT1G03070 | Symbols:  | glutamate binding | chr1:730148-731379 FORWARDmoderately similar to ( 331) loc_os03g53400 12003.m10310 protein transmembrane BAX inhibitor motif-containing protein 4, putative, expressed  no original description 	T
27.2	RNA.transcription	MDP0000934360	 highly similar to ( 629) AT1G64860 | Symbols: SIGA, SIG1, SIG2, SIGB, RPOD1 | SIGA (SIGMA FACTOR A); DNA binding / DNA-directed RNA polymerase/ sigma factor/ transcription factor | chr1:24098497-24100746 FORWARDmoderately similar to ( 432) loc_os08g06630 12008.m26470 protein RNA polymerase sigma factor rpoD, putative, expressed  no original description 	T
13.2.7	amino acid metabolism.degradation.histidine	MDP0000124089	 highly similar to ( 812) AT1G43710 | Symbols: emb1075 | emb1075 (embryo defective 1075); carboxy-lyase/ catalytic/ pyridoxal phosphate binding | chr1:16486534-16488298 REVERSEvery weakly similar to (52.4) DCE_PETHY Glutamate decarboxylase (EC 4.1.1.15) (GAD) - Petunia hybrida (Petunia)highly similar to ( 759) loc_os02g33710 12002.m08462 protein histidine decarboxylase, putative, expressed  no original description 	T
20.1	stress.biotic	MDP0000218549	 highly similar to ( 509) AT1G05850 | Symbols: POM1, ERH2, ELP1, CTL1, ELP, HOT2, ATCTL1 | POM1 (POM-POM1); chitinase | chr1:1766833-1768117 REVERSEweakly similar to ( 200) CHI2_BRANA Endochitinase CH25 precursor (EC 3.2.1.14) - Brassica napus (Rape)moderately similar to ( 457) loc_os09g32080 12009.m060024 protein endochitinase A2 precursor, putative, expressed  no original description 	T
33.3	development.squamosa promoter binding like (SPL)	MDP0000176265	 weakly similar to ( 155) AT5G50570 | Symbols:  | squamosa promoter-binding protein, putative | chr5:20582555-20583791 REVERSEweakly similar to ( 115) SBP2_ANTMA Squamosa promoter-binding protein 2 - Antirrhinum majus (Garden snapdragon)weakly similar to ( 168) loc_os08g41940 12008.m08172 protein teosinte glume architecture 1, putative, expressed  no original description 	T
35.2	not assigned.unknown	MDP0000226935	     PRK07003  no original description 	T
35.2	not assigned.unknown	MDP0000287061	  no original description 	T
26.7	misc.oxidases - copper, flavone etc	MDP0000211617	 moderately similar to ( 496) AT3G03080 | Symbols:  | NADP-dependent oxidoreductase, putative | chr3:698530-700278 REVERSEmoderately similar to ( 440) loc_os12g12580 12012.m05231 protein NADP-dependent oxidoreductase P2, putative, expressed  no original description 	T
35.2	not assigned.unknown	MDP0000285384	 moderately similar to ( 423) AT3G54260 | Symbols:  | unknown protein | chr3:20085097-20086745 REVERSEmoderately similar to ( 238) loc_os03g19470 12003.m07363 protein expressed protein  no original description 	T
19.15	tetrapyrrole synthesis.chlorophyll synthase	MDP0000163757	 highly similar to ( 551) AT3G51820 | Symbols: ATG4, G4, CHLG | G4; chlorophyll synthetase | chr3:19216301-19218934 REVERSEhighly similar to ( 522) loc_os05g28200 12005.m083694 protein bacteriochlorophyll synthase 34 kDa chain, putative, expressed  no original description 	T
33.99	development.unspecified	MDP0000283921	 moderately similar to ( 326) AT2G45330 | Symbols: emb1067 | emb1067 (embryo defective 1067); tRNA 2'-phosphotransferase/ transferase, transferring phosphorus-containing groups | chr2:18686340-18687874 FORWARDmoderately similar to ( 329) loc_os06g04300 12006.m05159 protein tRNA 2phosphotransferase, putative, expressed  no original description 	T
29.2.1.1.1.1.20	protein.synthesis.ribosomal protein.prokaryotic.chloroplast.30S subunit.S20	MDP0000205210	 weakly similar to ( 166) AT3G15190 | Symbols:  | chloroplast 30S ribosomal protein S20, putative | chr3:5116216-5117412 FORWARDweakly similar to ( 119) loc_os01g48690 12001.m150433 protein ribosomal protein rpS20, putative, expressed  no original description 	T
27.3.99	RNA.regulation of transcription.unclassified	MDP0000804605	 moderately similar to ( 207) AT1G32540 | Symbols: LOL1 | LOL1 (LSD ONE LIKE 1); DNA binding | chr1:11768043-11769591 FORWARDmoderately similar to ( 204) loc_os08g06280 12008.m26469 protein LOL1, putative, expressed  no original description 	T
35.2	not assigned.unknown	MDP0000843800	    no original description 	T
29.2.1.1.1.1.7	protein.synthesis.ribosomal protein.prokaryotic.chloroplast.30S subunit.S7	MDP0000231363	 moderately similar to ( 355) AT2G37270 | Symbols: ATRPS5B | ATRPS5B (RIBOSOMAL PROTEIN 5B); structural constituent of ribosome | chr2:15647883-15649042 REVERSEmoderately similar to ( 358) RS5_CICAR 40S ribosomal protein S5 (Fragment) - Cicer arietinum (Chickpea) (Garbanzo)moderately similar to ( 351) loc_os11g29190 12011.m080000 protein 40S ribosomal protein S5, putative, expressed  no original description 	T
29.2.1.2.1.5	protein.synthesis.ribosomal protein.eukaryotic.40S subunit.S5	MDP0000231363	 moderately similar to ( 355) AT2G37270 | Symbols: ATRPS5B | ATRPS5B (RIBOSOMAL PROTEIN 5B); structural constituent of ribosome | chr2:15647883-15649042 REVERSEmoderately similar to ( 358) RS5_CICAR 40S ribosomal protein S5 (Fragment) - Cicer arietinum (Chickpea) (Garbanzo)moderately similar to ( 351) loc_os11g29190 12011.m080000 protein 40S ribosomal protein S5, putative, expressed  no original description 	T
29.2.1.2.1.23	protein.synthesis.ribosomal protein.eukaryotic.40S subunit.S23	MDP0000363108	 moderately similar to ( 275) AT5G02960 | Symbols:  | 40S ribosomal protein S23 (RPS23B) | chr5:693280-694396 REVERSEmoderately similar to ( 283) RS23_FRAAN 40S ribosomal protein S23 (S12) - Fragaria ananassa (Strawberry)moderately similar to ( 283) loc_os03g60400 12003.m10930 protein 40S ribosomal protein S23, putative, expressed   no original description 	T
35.2	not assigned.unknown	MDP0000197509	 moderately similar to ( 207) AT3G06035 | Symbols:  | unknown protein | chr3:1823172-1824110 REVERSEweakly similar to ( 184) loc_os07g45060 12007.m08726 protein GPI-anchored protein At5g19240 precursor, putative, expressed no original description 	T
28.1.3	DNA.synthesis/chromatin structure.histone	MDP0000263445	 moderately similar to ( 266) AT4G40030 | Symbols:  | histone H3.2 | chr4:18555840-18556417 REVERSEmoderately similar to ( 264) H33_VITVI Histone H3.3 - Vitis vinifera (Grape)moderately similar to ( 266) loc_os06g04030 12006.m05134 protein histone H3, putative, expressed no original description 	T
29.2.2	protein.synthesis.ribosome biogenesis	MDP0000233571	 moderately similar to ( 328) AT4G15770 | Symbols:  | RNA binding / protein binding | chr4:8978060-8978623 FORWARDmoderately similar to ( 261) loc_os07g47420 12007.m08959 protein 60S ribosome subunit biogenesis protein NIP7, putative, expressed   no original description 	T
27.3.19	RNA.regulation of transcription.EIN3-like(EIL) transcription factor family	MDP0000202831	 highly similar to ( 536) AT2G27050 | Symbols: EIL1 | EIL1 (ETHYLENE-INSENSITIVE3-LIKE 1); transcription factor/ transcription regulator | chr2:11546314-11548068 FORWARDmoderately similar to ( 498) loc_os03g20790 12003.m07486 protein ETHYLENE-INSENSITIVE3-like 1 protein, putative, expressed  no original description 	T
34.9	transport.metabolite transporters at the mitochondrial membrane	MDP0000313602	 moderately similar to ( 443) AT5G58970 | Symbols: ATUCP2 | ATUCP2 (UNCOUPLING PROTEIN 2); oxidative phosphorylation uncoupler | chr5:23809437-23811018 REVERSEvery weakly similar to (75.1) ADT_CHLRE ADP,ATP carrier protein (ADP/ATP translocase) (Adenine nucleotide translocator) (ANT) - Chlamydomonas reinhardtiimoderately similar to ( 404) loc_os11g48040 12011.m08602 protein mitochondrial uncoupling protein 3, putative, expressed   no original description 	T
28.1	DNA.synthesis/chromatin structure	MDP0000348749	 moderately similar to ( 234) AT1G18800 | Symbols: NRP2 | NRP2 (NAP1-RELATED PROTEIN 2); DNA binding / chromatin binding / histone binding | chr1:6481466-6483463 REVERSEmoderately similar to ( 207) loc_os02g36710 12002.m08758 protein protein SET, putative, expressed no original description 	T
30.5	signalling.G-proteins	MDP0000574151	 moderately similar to ( 305) AT1G56330 | Symbols: SAR1, ATSAR1, ATSARA1B, ATSAR1B | ATSAR1B (SECRETION-ASSOCIATED RAS 1 B); GTP binding | chr1:21086845-21088478 REVERSEmoderately similar to ( 301) SAR1A_BRACM GTP-binding protein SAR1A - Brassica campestris (Field mustard)moderately similar to ( 303) loc_os01g15010 12001.m08102 protein GTP-binding protein SAR1A, putative, expressed   no original description 	T
35.2	not assigned.unknown	MDP0000280508	 moderately similar to ( 246) AT5G50100 | Symbols:  | FUNCTIONS IN: molecular_function unknown; INVOLVED IN: biological_process unknown; LOCATED IN: mitochondrion; EXPRESSED IN: 22 plant structures; EXPRESSED DURING: 15 growth stages; CONTAINS InterPro DOMAIN/s: Thioredoxin-like fold (InterPro:IPR012336), Putative thiol-disulphide oxidoreductase DCC (InterPro:IPR007263); Has 598 Blast hits to 598 proteins in 152 species: Archae - 0; Bacteria - 266; Metazoa - 3; Fungi - 0; Plants - 27; Viruses - 0; Other Eukaryotes - 302 (source: NCBI BLink). | chr5:20371916-20373172 FORWARDmoderately similar to ( 233) loc_os04g57310 12004.m35507 protein PBng143, putative, expressed no original description 	T
29.3.3	protein.targeting.chloroplast	MDP0000742177	 highly similar to ( 752) AT2G24820 | Symbols: TIC55 | TIC55 (TRANSLOCON AT THE INNER ENVELOPE MEMBRANE OF CHLOROPLASTS 55); 2 iron, 2 sulfur cluster binding / electron carrier/ oxidoreductase | chr2:10575038-10576829 FORWARDweakly similar to ( 113) CAO_CHLRE Chlorophyllide a oxygenase, chloroplast precursor (EC 1.13.12.14) (Chlorophyll a oxygenase) (Chlorophyll b synthase) - Chlamydomonas reinhardtiihighly similar to ( 679) loc_os02g54980 12002.m10525 protein pheophorbide a oxygenase, chloroplast precursor, putative, expressed   no original description 	T
35.2	not assigned.unknown	MDP0000318588	 moderately similar to ( 319) AT3G10250 | Symbols:  | unknown protein | chr3:3168371-3170418 REVERSEmoderately similar to ( 218) loc_os02g04450 12002.m33545 protein plant-specific domain TIGR01589 family protein, expressed  no original description 	T
26.28	misc.GDSL-motif lipase	MDP0000250901	 moderately similar to ( 435) AT2G42990 | Symbols:  | GDSL-motif lipase/hydrolase family protein | chr2:17879056-17880200 FORWARDmoderately similar to ( 210) APG_BRANA Anter-specific proline-rich protein APG (Protein CEX) (Fragment) - Brassica napus (Rape)moderately similar to ( 363) loc_os06g43044 12006.m08834 protein anther-specific proline-rich protein APG, putative, expressed   no original description 	T
31.1	cell.organisation	MDP0000823528	 weakly similar to ( 142) AT4G27360 | Symbols:  | dynein light chain, putative | chr4:13694032-13694517 FORWARDvery weakly similar to (85.9) DYL1_CHLRE Dynein 8 kDa light chain, flagellar outer arm - Chlamydomonas reinhardtiiweakly similar to ( 109) loc_os01g55510 12001.m11709 protein dynein light chain LC6, flagellar outer arm, putative, expressed no original description 	T
35.1	not assigned.no ontology	MDP0000144927	 highly similar to ( 546) AT5G26180 | Symbols:  | NOL1/NOP2/sun family protein | chr5:9149253-9152595 FORWARDhighly similar to ( 504) loc_os02g12600 12002.m06507 protein NOL1R, putative, expressed  no original description 	T
31.3	cell.cycle	MDP0000241994	 moderately similar to ( 356) AT2G26760 | Symbols: CYCB1;4 | CYCB1;4 (Cyclin B1;4); cyclin-dependent protein kinase regulator | chr2:11401551-11403205 FORWARDmoderately similar to ( 363) CCN1_ANTMA G2/mitotic-specific cyclin-1 - Antirrhinum majus (Garden snapdragon)moderately similar to ( 339) loc_os01g59120 12001.m42540 protein cyclin IaZm, putative, expressed  no original description 	T
35.2	not assigned.unknown	MDP0000237173	    no original description 	T
20.2.3	stress.abiotic.drought/salt	MDP0000893254	 highly similar to ( 835) AT1G30360 | Symbols: ERD4 | ERD4 (early-responsive to dehydration 4) | chr1:10715892-10718799 FORWARDhighly similar to ( 771) loc_os07g05570 12007.m29066 protein ERD4 protein, putative, expressed   no original description 	T
20.2.99	stress.abiotic.unspecified	MDP0000243084	 moderately similar to ( 204) AT3G53990 | Symbols:  | universal stress protein (USP) family protein | chr3:19989658-19991019 REVERSEweakly similar to ( 194) loc_os05g37970 12005.m083738 protein USP family protein, putative, expressed no original description 	T
34.8	transport.metabolite transporters at the envelope membrane	MDP0000261082	 moderately similar to ( 473) AT5G33320 | Symbols: CUE1, PPT, ARAPPT | CUE1 (CAB UNDEREXPRESSED 1); antiporter/ triose-phosphate transmembrane transporter | chr5:12588950-12591408 FORWARDmoderately similar to ( 207) TPT_PEA Triose phosphate/phosphate translocator, chloroplast precursor (cTPT) (p36) (E30) - Pisum sativum (Garden pea)moderately similar to ( 482) loc_os09g12600 12009.m22060 protein triose phosphate/phosphate translocator, non-green plastid,chloroplast precursor, putative, expressed  no original description 	T
29.5.11.4.5.2	protein.degradation.ubiquitin.E3.BTB/POZ Cullin3.BTB/POZ	MDP0000786851	 nearly identical (1017) AT5G19330 | Symbols:  | armadillo/beta-catenin repeat family protein / BTB/POZ domain-containing protein | chr5:6508300-6512701 REVERSEvery weakly similar to (62.4) IMA1B_ORYSA Importin alpha-1b subunit - Oryza sativa (Rice)highly similar to ( 961) loc_os05g33050 12005.m07537 protein protein binding protein, putative, expressed no original description 	T
34.12	transport.metal	MDP0000284084	 moderately similar to ( 265) AT2G46800 | Symbols: ZAT, ATMTP1, MTP1, ZAT1, ATCDF1 | ZAT (ZINC TRANSPORTER OF ARABIDOPSIS THALIANA); inorganic anion transmembrane transporter/ metal ion transmembrane transporter/ zinc ion transmembrane transporter | chr2:19238128-19239324 FORWARDmoderately similar to ( 236) loc_os05g03780 12005.m04912 protein metal tolerance protein A2, putative, expressed  no original description 	T
34.21	transport.calcium	MDP0000320927	 highly similar to ( 528) AT3G13320 | Symbols: CAX2, atcax2 | CAX2 (CATION EXCHANGER 2); calcium:cation antiporter/ calcium:hydrogen antiporter | chr3:4315418-4317997 FORWARDhighly similar to ( 583) CAX2_ORYSA Vacuolar cation/proton exchanger 2 (Ca(2+)/H(+) exchanger 2) (OsCAX2) - Oryza sativa (Rice)highly similar to ( 583) loc_os03g27960 12003.m08120 protein vacuolar cation/proton exchanger 2, putative, expressed no original description 	T
29.3.4.1	protein.targeting.secretory pathway.ER	MDP0000187794	 moderately similar to ( 246) AT4G39220 | Symbols: ATRER1A | ATRER1A | chr4:18264280-18265531 FORWARDmoderately similar to ( 257) loc_os01g01640 12001.m06808 protein RER1A protein, putative, expressed  no original description 	T
27.3.22	RNA.regulation of transcription.HB,Homeobox transcription factor family	MDP0000219162	 weakly similar to ( 187) AT3G18010 | Symbols: WOX1 | WOX1 (WUSCHEL related homeobox 1); transcription factor | chr3:6161155-6163183 REVERSEweakly similar to ( 108) WOX3B_MAIZE WUSCHEL-related homeobox 3B (Narrow sheath protein 2) - Zea mays (Maize)weakly similar to ( 108) loc_os12g01120 12012.m04116 protein WUSCHEL-related homeobox 3B, putative  no original description 	T
29.2.1.2.1.29	protein.synthesis.ribosomal protein.eukaryotic.40S subunit.S29	MDP0000216511	 weakly similar to ( 114) AT4G33865 | Symbols:  | 40S ribosomal protein S29 (RPS29C) | chr4:16233395-16234114 REVERSEweakly similar to ( 121) loc_os12g32380 12012.m07041 protein 40S ribosomal protein S29, putative, expressed   no original description 	T
35.2	not assigned.unknown	MDP0000257355	 moderately similar to ( 213) AT5G16250 | Symbols:  | unknown protein | chr5:5310264-5310815 REVERSEweakly similar to ( 170) loc_os04g41900 12004.m09164 protein expressed protein   no original description 	T
29.4	protein.postranslational modification	MDP0000223686	 highly similar to ( 605) AT4G26100 | Symbols: CK1, CKL1 | CK1 (CASEIN KINASE 1); ATP binding / kinase/ protein kinase/ protein serine/threonine kinase | chr4:13227885-13230508 REVERSEvery weakly similar to (61.2) CIPK1_ORYSA CIPK-like protein 1 (EC 2.7.11.1) (OsCK1) - Oryza sativa (Rice)highly similar to ( 623) loc_os01g51200 12001.m43159 protein casein kinase I isoform delta-like, putative, expressed  no original description 	T
26.4.1	misc.beta 1,3 glucan hydrolases.glucan endo-1,3-beta-glucosidase	MDP0000327191	 moderately similar to ( 415) AT3G57270 | Symbols: BG1 | BG1 (BETA-1,3-GLUCANASE 1); catalytic/ cation binding / hydrolase, hydrolyzing O-glycosyl compounds | chr3:21191336-21193118 REVERSEhighly similar to ( 516) E13B_PRUPE Glucan endo-1,3-beta-glucosidase, basic isoform precursor (EC 3.2.1.39) ((1->3)-beta-glucan endohydrolase) ((1->3)-beta-glucanase) (Beta-1,3-endoglucanase) (PpGns1) - Prunus persica (Peach)moderately similar to ( 314) loc_os01g71340 12001.m13177 protein glucan endo-1,3-beta-glucosidase, acidic isoform precursor, putative, expressed        VAR1 COG5048 MopB_Res-Cmplx1_Nad11-M COG5099 SKN1 PRK08581 COG4886 PRK07133 YfhO Spore_YhcN_YlaJ Merozoite_SPAM DUF1976 ARG80 GAT1 PAP1 DUF1157 HSF1  no original description 	T
26.21	misc.protease inhibitor/seed storage/lipid transfer protein (LTP) family protein	MDP0000757585	 very weakly similar to (62.8) AT3G22120 | Symbols: CWLP | CWLP (CELL WALL-PLASMA MEMBRANE LINKER PROTEIN); lipid binding | chr3:7795283-7796287 REVERSEvery weakly similar to (56.2) PRP2_SOYBN Repetitive proline-rich cell wall protein 2 precursor - Glycine max (Soybean)very weakly similar to (54.7) loc_os12g38460 12012.m07632 protein RNA recognition motif family protein no original description 	T
11.2.3	lipid metabolism.FA desaturation.omega 3 desaturase	MDP0000209507	 highly similar to ( 558) AT2G29980 | Symbols: FAD3 | FAD3 (FATTY ACID DESATURASE 3); omega-3 fatty acid desaturase | chr2:12782257-12784946 REVERSEhighly similar to ( 568) FAD3E_BRANA Omega-3 fatty acid desaturase, endoplasmic reticulum (EC 1.14.19.-) - Brassica napus (Rape)highly similar to ( 535) loc_os03g18070 12003.m07232 protein omega-3 fatty acid desaturase, chloroplast precursor, putative, expressed no original description 	T
35.2	not assigned.unknown	MDP0000370504	 weakly similar to ( 195) AT4G28210 | Symbols: emb1923 | emb1923 (embryo defective 1923) | chr4:13990634-13991931 FORWARD   no original description 	T
27.3.99	RNA.regulation of transcription.unclassified	MDP0000874708	 moderately similar to ( 202) AT1G51200 | Symbols:  | zinc finger (AN1-like) family protein | chr1:18985690-18986211 FORWARDweakly similar to ( 121) ISAP1_ORYSA Multiple stress-responsive zinc-finger protein ISAP1 (Stress-associated protein 1) (OsISAP1) - Oryza sativa (Rice)moderately similar to ( 225) loc_os06g41010 12006.m31898 protein zinc finger A20 and AN1 domains-containing protein, putative, expressed no original description 	T
25	C1-metabolism	MDP0000362220	 moderately similar to ( 293) AT5G16450 | Symbols:  | dimethylmenaquinone methyltransferase family protein | chr5:5374357-5375342 FORWARDmoderately similar to ( 290) loc_os01g52460 12001.m11420 protein regulator of ribonuclease activity A, putative, expressed  no original description 	T
23.3.2.3	nucleotide metabolism.salvage.nucleoside kinases.thymidine kinase	MDP0000130389	 moderately similar to ( 286) AT3G07800 | Symbols:  | thymidine kinase, putative | chr3:2489944-2490935 REVERSEmoderately similar to ( 290) KITH_ORYSA Thymidine kinase (EC 2.7.1.21) - Oryza sativa (Rice)moderately similar to ( 295) loc_os03g02200 12003.m05763 protein thymidine kinase, putative, expressed   no original description 	T
30.2.11	signalling.receptor kinases.leucine rich repeat XI	MDP0000166536	 nearly identical (1071) AT1G12460 | Symbols:  | leucine-rich repeat transmembrane protein kinase, putative | chr1:4247703-4250444 FORWARDmoderately similar to ( 329) RPK1_IPONI Receptor-like protein kinase precursor (EC 2.7.11.1) - Ipomoea nil (Japanese morning glory) (Pharbitis nil)highly similar to ( 923) loc_os03g03570 12003.m101708 protein ATP binding protein, putative, expressed  no original description 	T
20.2.99	stress.abiotic.unspecified	MDP0000902360	 weakly similar to ( 198) AT4G08685 | Symbols: SAH7 | SAH7 | chr4:5550563-5551259 FORWARDweakly similar to ( 129) PSC13_MAIZE Pollen-specific protein C13 precursor - Zea mays (Maize)weakly similar to ( 155) loc_os10g22590 12010.m05266 protein pollen-specific protein C13 precursor, putative, expressed  no original description 	T
30.2.25	signalling.receptor kinases.wall associated kinase	MDP0000704594	 very weakly similar to (66.2) AT1G16260 | Symbols:  | protein kinase family protein | chr1:5559708-5562018 REVERSEvery weakly similar to (56.6) loc_os04g55760 12004.m10467 protein OsWAK55 - OsWAK receptor-like protein kinase no original description 	T
26.28	misc.GDSL-motif lipase	MDP0000696168	 highly similar to ( 521) AT3G04290 | Symbols: ATLTL1, LTL1 | LTL1 (LI-TOLERANT LIPASE 1); carboxylesterase/ hydrolase, acting on ester bonds | chr3:1133620-1136223 REVERSEweakly similar to ( 172) APG_BRANA Anter-specific proline-rich protein APG (Protein CEX) (Fragment) - Brassica napus (Rape)moderately similar to ( 492) loc_os02g40440 12002.m09130 protein anther-specific proline-rich protein APG precursor, putative, expressed   no original description 	T
29.2.3	protein.synthesis.initiation	MDP0000232642	 weakly similar to ( 191) AT5G54940 | Symbols:  | eukaryotic translation initiation factor SUI1, putative | chr5:22308420-22308758 REVERSEweakly similar to ( 172) SUI1_ORYSA Protein translation factor SUI1 homolog (Protein GOS2) (Translational initiation factor 1) (Protein eIF1) - Oryza sativa (Rice)weakly similar to ( 172) loc_os07g34589 12007.m29388 protein protein translation factor SUI1, putative, expressed no original description 	T
31.4	cell.vesicle transport	MDP0000915256	 nearly identical (1462) AT4G31480 | Symbols:  | coatomer beta subunit, putative / beta-coat protein, putative / beta-COP, putative | chr4:15264076-15267384 FORWARDnearly identical (1441) loc_os11g07280 12011.m28658 protein coatomer subunit beta, putative, expressed    no original description 	T
30.2.11	signalling.receptor kinases.leucine rich repeat XI	MDP0000167553	 highly similar to ( 938) AT2G27060 | Symbols:  | ATP binding / protein binding / protein kinase/ protein serine/threonine kinase/ protein tyrosine kinase | chr2:11551288-11554577 FORWARDweakly similar to ( 137) RPK1_IPONI Receptor-like protein kinase precursor (EC 2.7.11.1) - Ipomoea nil (Japanese morning glory) (Pharbitis nil)highly similar to ( 878) loc_os03g20450 12003.m07454 protein ATP binding protein, putative, expressed   no original description 	T
30.2.3	signalling.receptor kinases.leucine rich repeat III	MDP0000167553	 highly similar to ( 938) AT2G27060 | Symbols:  | ATP binding / protein binding / protein kinase/ protein serine/threonine kinase/ protein tyrosine kinase | chr2:11551288-11554577 FORWARDweakly similar to ( 137) RPK1_IPONI Receptor-like protein kinase precursor (EC 2.7.11.1) - Ipomoea nil (Japanese morning glory) (Pharbitis nil)highly similar to ( 878) loc_os03g20450 12003.m07454 protein ATP binding protein, putative, expressed   no original description 	T
35.2	not assigned.unknown	MDP0000205570	 moderately similar to ( 262) AT5G43750 | Symbols:  | unknown protein | chr5:17580100-17581145 REVERSEmoderately similar to ( 241) loc_os01g70400 12001.m43437 protein expressed protein  no original description 	T
27.3.55	RNA.regulation of transcription.HDA	MDP0000780720	 highly similar to ( 536) AT1G08460 | Symbols: HDA08, HDA8, ATHDA8 | HDA08; histone deacetylase | chr1:2672527-2674469 FORWARDvery weakly similar to ( 100) HDAC_MAIZE Probable histone deacetylase (RPD3 homolog) - Zea mays (Maize)highly similar to ( 550) loc_os05g36920 12005.m07873 protein histone deacetylase-like amidohydrolase, putative, expressed  no original description 	T
35.2	not assigned.unknown	MDP0000269361	 highly similar to ( 644) AT3G19540 | Symbols:  | unknown protein | chr3:6780762-6782633 FORWARDhighly similar to ( 597) loc_os03g40930 12003.m09164 protein expressed protein    no original description 	T
26.4.1	misc.beta 1,3 glucan hydrolases.glucan endo-1,3-beta-glucosidase	MDP0000235663	 highly similar to ( 620) AT3G07320 | Symbols:  | glycosyl hydrolase family 17 protein | chr3:2332324-2333925 REVERSEmoderately similar to ( 252) E13B_WHEAT Glucan endo-1,3-beta-glucosidase precursor (EC 3.2.1.39) ((1->3)-beta-glucan endohydrolase) ((1->3)-beta-glucanase) (Beta-1,3-endoglucanase) - Triticum aestivum (Wheat)moderately similar to ( 445) loc_os09g32550 12009.m060028 protein glucan endo-1,3-beta-glucosidase A6 precursor, putative, expressed   no original description 	T
35.2	not assigned.unknown	MDP0000208573	 weakly similar to ( 136) loc_os03g48110 12003.m09822 protein expressed protein no original description 	T
23.3.3	nucleotide metabolism.salvage.NUDIX hydrolases	MDP0000862091	 moderately similar to ( 236) AT3G12600 | Symbols: atnudt16 | atnudt16 (Arabidopsis thaliana Nudix hydrolase homolog 16); hydrolase | chr3:4004676-4005995 FORWARDmoderately similar to ( 231) loc_os03g59580 12003.m10855 protein nudix hydrolase 13, mitochondrial precursor, putative, expressed no original description 	T
35.1	not assigned.no ontology	MDP0000203723	 highly similar to ( 711) AT1G10950 | Symbols:  | endomembrane protein 70, putative | chr1:3659322-3663622 FORWARDhighly similar to ( 671) loc_os02g34690 12002.m08558 protein transmembrane 9 superfamily protein member 3 precursor, putative, expressed  no original description 	T
27.3.71	RNA.regulation of transcription.SNF7	MDP0000260590	 moderately similar to ( 279) AT2G06530 | Symbols: VPS2.1 | VPS2.1 | chr2:2588740-2590285 REVERSEmoderately similar to ( 268) loc_os07g13270 12007.m29110 protein charged multivesicular body protein 2a, putative, expressed  no original description 	T
29.3.99	protein.targeting.unknown	MDP0000237988	 highly similar to ( 927) AT3G06530 | Symbols:  | binding | chr3:2022596-2033637 FORWARDhighly similar to ( 697) loc_os04g46310 12004.m35403 protein HEAT repeat family protein, expressed   no original description 	T
34.19.1	transport.Major Intrinsic Proteins.PIP	MDP0000142957	 weakly similar to ( 144) AT2G16850 | Symbols: PIP3B, PIP2;8 | PIP2;8 (PLASMA MEMBRANE INTRINSIC PROTEIN 2;8); water channel | chr2:7301647-7303180 FORWARDweakly similar to ( 137) PIP26_ORYSA Probable aquaporin PIP2.6 (Plasma membrane intrinsic protein 2.6) (OsPIP2.6) - Oryza sativa (Rice)weakly similar to ( 137) loc_os04g16450 12004.m35259 protein aquaporin PIP2.6, putative, expressed  no original description 	T
34.19.4	transport.Major Intrinsic Proteins.SIP	MDP0000905147	 weakly similar to ( 184) AT3G04090 | Symbols: SIP1;1, SIP1A | SIP1A (SMALL AND BASIC INTRINSIC PROTEIN 1A); water channel | chr3:1072340-1074031 REVERSEvery weakly similar to (57.0) TIP43_ORYSA Probable aquaporin TIP4.3 (Tonoplast intrinsic protein 4.3) (OsTIP4.3) - Oryza sativa (Rice)moderately similar to ( 213) loc_os01g08660 12001.m07491 protein aquaporin SIP1.1, putative, expressed  no original description 	T
35.2	not assigned.unknown	MDP0000320341	 highly similar to ( 589) AT1G09340 | Symbols: CRB, CSP41B | CRB (CHLOROPLAST RNA BINDING); binding / catalytic/ coenzyme binding | chr1:3015473-3018035 FORWARDhighly similar to ( 570) loc_os12g23180 12012.m26701 protein RNA binding protein, putative, expressed   no original description 	T
33.2	development.late embryogenesis abundant	MDP0000324831	 very weakly similar to (81.6) AT4G15910 | Symbols: ATDI21, DI21 | ATDI21 (ARABIDOPSIS THALIANA DROUGHT-INDUCED 21) | chr4:9028657-9029269 FORWARDvery weakly similar to (81.3) LEA5A_GOSHI Late embryogenesis abundant protein Lea5-A - Gossypium hirsutum (Upland cotton)very weakly similar to (50.4) loc_os01g21250 12001.m08609 protein protein induced upon tuberization, putative, expressed  no original description 	T
33.99	development.unspecified	MDP0000288977	 highly similar to ( 644) AT2G19540 | Symbols:  | transducin family protein / WD-40 repeat family protein | chr2:8461804-8464347 FORWARDvery weakly similar to (56.6) GBB2_TOBAC Guanine nucleotide-binding protein subunit beta 2 - Nicotiana tabacum (Common tobacco)highly similar to ( 606) loc_os12g03822 12012.m56461 protein glutamate-rich WD repeat-containing protein 1, putative, expressed   no original description 	T
17.5.3	hormone metabolism.ethylene.induced-regulated-responsive-activated	MDP0000267538	 weakly similar to ( 194) AT3G24500 | Symbols: MBF1C, ATMBF1C | MBF1C (MULTIPROTEIN BRIDGING FACTOR 1C); DNA binding / transcription coactivator/ transcription factor | chr3:8918755-8919201 FORWARDweakly similar to ( 153) loc_os06g39240 12006.m091727 protein endothelial differentiation-related factor 1, putative, expressed no original description 	T
29.2.1.1.1.1.13	protein.synthesis.ribosomal protein.prokaryotic.chloroplast.30S subunit.S13	MDP0000140140	 moderately similar to ( 248) AT5G14320 | Symbols:  | 30S ribosomal protein S13, chloroplast (CS13) | chr5:4618189-4618772 REVERSEweakly similar to ( 138) RT13_SOYBN Small ribosomal subunit protein S13, mitochondrial precursor - Glycine max (Soybean)moderately similar to ( 201) loc_os03g49710 12003.m09970 protein 30S ribosomal protein S13, putative, expressed  no original description 	T
21.1	redox.thioredoxin	MDP0000201801	 highly similar to ( 538) AT2G47470 | Symbols: ATPDIL2-1, UNE5, MEE30, PDI11, ATPDI11 | UNE5 (UNFERTILIZED EMBRYO SAC 5); protein disulfide isomerase | chr2:19481503-19483683 FORWARDhighly similar to ( 558) PDIA6_MEDSA Probable protein disulfide-isomerase A6 precursor (EC 5.3.4.1) (P5) - Medicago sativa (Alfalfa)highly similar to ( 518) loc_os01g23740 12001.m43001 protein OsPDIL2-2 - Oryza sativa protein disulfide isomerase, expressed  no original description 	T
27.3.3	RNA.regulation of transcription.AP2/EREBP, APETALA2/Ethylene-responsive element binding protein family	MDP0000390121	 weakly similar to ( 115) AT5G07310 | Symbols:  | AP2 domain-containing transcription factor, putative | chr5:2305684-2306660 FORWARDvery weakly similar to (66.6) ERF1_ORYSA Ethylene-responsive transcription factor 1 (Ethylene-responsive element-binding factor 1) (EREBP-1) (OsEREBP1) - Oryza sativa (Rice)weakly similar to ( 106) loc_os04g32620 12004.m08360 protein DNA binding protein, putative, expressed  no original description 	T
29.2.1.2.1.19	protein.synthesis.ribosomal protein.eukaryotic.40S subunit.S19	MDP0000772832	 moderately similar to ( 248) AT5G61170 | Symbols:  | 40S ribosomal protein S19 (RPS19C) | chr5:24611158-24612202 FORWARDmoderately similar to ( 224) RS19_ORYSA 40S ribosomal protein S19 - Oryza sativa (Rice)moderately similar to ( 224) loc_os03g31090 12003.m08361 protein 40S ribosomal protein S19, putative, expressed   no original description 	T
1.3.7	PS.calvin cycle.FBPase	MDP0000251810	 highly similar to ( 603) AT1G43670 | Symbols:  | fructose-1,6-bisphosphatase, putative / D-fructose-1,6-bisphosphate 1-phosphohydrolase, putative / FBPase, putative | chr1:16468184-16470347 FORWARDhighly similar to ( 621) F16P2_SPIOL Fructose-1,6-bisphosphatase, cytosolic (EC 3.1.3.11) (D-fructose-1,6-bisphosphate 1-phosphohydrolase) (FBPase) - Spinacia oleracea (Spinach)highly similar to ( 611) loc_os01g64660 12001.m43295 protein fructose-1,6-bisphosphatase, cytosolic, putative, expressed   no original description 	T
11.9.2.1	lipid metabolism.lipid degradation.lipases.triacylglycerol lipase	MDP0000186513	 moderately similar to ( 328) AT5G14180 | Symbols: MPL1 | MPL1 (MYZUS PERSICAE-INDUCED LIPASE 1); catalytic | chr5:4571442-4574413 REVERSEmoderately similar to ( 338) loc_os06g42860 12006.m08816 protein gastric triacylglycerol lipase precursor, putative, expressed no original description 	T
1.3.7	PS.calvin cycle.FBPase	MDP0000591370	 moderately similar to ( 464) AT3G54050 | Symbols:  | fructose-1,6-bisphosphatase, putative / D-fructose-1,6-bisphosphate 1-phosphohydrolase, putative / FBPase, putative | chr3:20016951-20018527 FORWARDmoderately similar to ( 469) F16P1_ORYSA Fructose-1,6-bisphosphatase, chloroplast precursor (EC 3.1.3.11) (D-fructose-1,6-bisphosphate 1-phosphohydrolase) (FBPase) - Oryza sativa (Rice)moderately similar to ( 469) loc_os03g16050 12003.m07042 protein fructose-1,6-bisphosphatase, chloroplast precursor, putative, expressed no original description 	T
35.2	not assigned.unknown	MDP0000253706	 moderately similar to ( 206) AT5G53390 | Symbols:  | unknown protein | chr5:21656951-21659207 REVERSEmoderately similar to ( 230) loc_os01g48874 12001.m11075 protein expressed protein   no original description 	T
27.4	RNA.RNA binding	MDP0000149711	 highly similar to ( 652) AT1G17370 | Symbols: UBP1B | UBP1B (oligouridylate binding protein 1B); mRNA 3'-UTR binding | chr1:5951842-5954825 REVERSEvery weakly similar to (81.6) ROC3_NICSY 28 kDa ribonucleoprotein, chloroplast precursor (28RNP) - Nicotiana sylvestris (Wood tobacco)highly similar to ( 565) loc_os11g40510 12011.m28685 protein nucleolysin TIAR, putative, expressed  no original description 	T
2.2.1.1	major CHO metabolism.degradation.sucrose.fructokinase	MDP0000245124	 highly similar to ( 652) AT3G54090 | Symbols:  | pfkB-type carbohydrate kinase family protein | chr3:20028145-20029835 FORWARDweakly similar to ( 190) SCRK1_ORYSA Fructokinase-1 (EC 2.7.1.4) (Fructokinase I) (OsFKI) - Oryza sativa (Rice)highly similar to ( 554) loc_os01g63220 12001.m43514 protein protein kinase, putative, expressed   no original description 	T
29.2.1.2.2.34	protein.synthesis.ribosomal protein.eukaryotic.60S subunit.L34	MDP0000129561	 weakly similar to ( 186) AT1G26880 | Symbols:  | 60S ribosomal protein L34 (RPL34A) | chr1:9315798-9316681 REVERSEweakly similar to ( 191) RL34_PEA 60S ribosomal protein L34 - Pisum sativum (Garden pea)weakly similar to ( 179) loc_os09g24690 12009.m05623 protein 60S ribosomal protein L34, putative, expressed  no original description 	T
35.2	not assigned.unknown	MDP0000122540	 weakly similar to ( 182) AT2G42570 | Symbols:  | unknown protein | chr2:17717498-17719921 REVERSEweakly similar to ( 164) loc_os01g46350 12001.m10833 protein expressed protein  no original description 	T
35.1	not assigned.no ontology	MDP0000353448	 highly similar to ( 535) AT1G34020 | Symbols:  | transporter-related | chr1:12367359-12368965 FORWARDmoderately similar to ( 484) loc_os05g12490 12005.m05713 protein integral membrane protein like, putative, expressed  no original description 	T
21.4	redox.glutaredoxins	MDP0000406592	 weakly similar to ( 167) AT3G62950 | Symbols:  | glutaredoxin family protein | chr3:23266303-23266614 FORWARDvery weakly similar to (68.6) GLRX_RICCO Glutaredoxin - Ricinus communis (Castor bean)weakly similar to ( 108) loc_os01g27140 12001.m09165 protein glutaredoxin, putative, expressed no original description 	T
29.2.1.2.2.17	protein.synthesis.ribosomal protein.eukaryotic.60S subunit.L17	MDP0000305218	 moderately similar to ( 291) AT1G67430 | Symbols:  | 60S ribosomal protein L17 (RPL17B) | chr1:25262209-25263627 FORWARDmoderately similar to ( 277) RL17_MAIZE 60S ribosomal protein L17 - Zea mays (Maize)moderately similar to ( 277) loc_os09g08430 12009.m04110 protein 60S ribosomal protein L17, putative, expressed   no original description 	T
35.2	not assigned.unknown	MDP0000440685	  no original description 	T
29.2.1.2.2.14	protein.synthesis.ribosomal protein.eukaryotic.60S subunit.L14	MDP0000214560	 weakly similar to ( 171) AT4G27090 | Symbols:  | 60S ribosomal protein L14 (RPL14B) | chr4:13594104-13595187 REVERSEweakly similar to ( 163) RL14_PEA Probable 60 ribosomal protein L14 (Hydroxyproline-rich glycoprotein HRGP1) - Pisum sativum (Garden pea)weakly similar to ( 179) loc_os04g43540 12004.m09314 protein 60 ribosomal protein L14, putative, expressed no original description 	T
27.3.11	RNA.regulation of transcription.C2H2 zinc finger family	MDP0000160072	 weakly similar to ( 177) AT3G50700 | Symbols: AtIDD2 | AtIDD2 (Arabidopsis thaliana Indeterminate(ID)-Domain 2); nucleic acid binding / transcription factor/ zinc ion binding | chr3:18840945-18842829 FORWARDweakly similar to ( 169) loc_os01g09850 12001.m42906 protein zinc finger, C2H2-type, putative, expressed  no original description 	T
26.24	misc.GCN5-related N-acetyltransferase	MDP0000772208	 moderately similar to ( 218) AT3G22560 | Symbols:  | GCN5-related N-acetyltransferase (GNAT) family protein | chr3:7998915-7999442 REVERSEweakly similar to ( 151) loc_os03g58010 12003.m10713 protein N-acetyltransferase, putative, expressed  no original description 	T
30.99	signalling.unspecified	MDP0000281042	 nearly identical (1028) AT5G19390 | Symbols:  | pleckstrin homology (PH) domain-containing protein / RhoGAP domain-containing protein | chr5:6531906-6537143 FORWARDhighly similar to ( 989) loc_os03g24180 12003.m07764 protein rho GTPase activator, putative, expressed   no original description 	T
29.2.1.2.2.17	protein.synthesis.ribosomal protein.eukaryotic.60S subunit.L17	MDP0000178948	 moderately similar to ( 303) AT1G67430 | Symbols:  | 60S ribosomal protein L17 (RPL17B) | chr1:25262209-25263627 FORWARDmoderately similar to ( 299) RL172_HORVU 60S ribosomal protein L17-2 - Hordeum vulgare (Barley)moderately similar to ( 300) loc_os09g08430 12009.m04110 protein 60S ribosomal protein L17, putative, expressed no original description 	T
26.12	misc.peroxidases	MDP0000304104	 highly similar to ( 640) AT5G57960 | Symbols:  | GTP-binding family protein | chr5:23463133-23466481 REVERSEmoderately similar to ( 233) PER1_ARAHY Cationic peroxidase 1 precursor (EC 1.11.1.7) (PNPC1) - Arachis hypogaea (Peanut)highly similar to ( 580) loc_os11g38020 12011.m07649 protein GTP-binding protein hflX, putative, expressed   no original description 	T
23.3.2.1	nucleotide metabolism.salvage.nucleoside kinases.adenosine kinase	MDP0000225318	 highly similar to ( 529) AT5G03300 | Symbols: ADK2 | ADK2 (ADENOSINE KINASE 2); adenosine kinase/ copper ion binding / kinase | chr5:796573-798997 FORWARDmoderately similar to ( 401) ADK_PHYPA Adenosine kinase (EC 2.7.1.20) (AK) (Adenosine 5'-phosphotransferase) - Physcomitrella patens (Moss)highly similar to ( 510) loc_os04g43750 12004.m101587 protein adenosine kinase 2, putative, expressed  no original description 	T
27.3.35	RNA.regulation of transcription.bZIP transcription factor family	MDP0000279891	 weakly similar to ( 143) AT3G17609 | Symbols: HYH | HYH (HY5-HOMOLOG); DNA binding / transcription factor | chr3:6023971-6024585 FORWARDweakly similar to ( 112) loc_os02g10860 12002.m06384 protein transcription factor HY5, putative, expressed  no original description 	T
30.2.11	signalling.receptor kinases.leucine rich repeat XI	MDP0000172627	 nearly identical (1027) AT5G48940 | Symbols:  | leucine-rich repeat transmembrane protein kinase, putative | chr5:19839785-19843744 FORWARDhighly similar to ( 538) RPK1_IPONI Receptor-like protein kinase precursor (EC 2.7.11.1) - Ipomoea nil (Japanese morning glory) (Pharbitis nil)highly similar to ( 935) loc_os04g04330 12004.m101454 protein ATP binding protein, putative, expressed   no original description 	T
11.2.3	lipid metabolism.FA desaturation.omega 3 desaturase	MDP0000210620	 moderately similar to ( 420) AT5G05580 | Symbols: FAD8 | FAD8 (FATTY ACID DESATURASE 8); omega-3 fatty acid desaturase | chr5:1664331-1666345 FORWARDmoderately similar to ( 422) FAD3C_RICCO Omega-3 fatty acid desaturase, chloroplast precursor (EC 1.14.19.-) - Ricinus communis (Castor bean)moderately similar to ( 399) loc_os07g49310 12007.m29027 protein omega-3 fatty acid desaturase, chloroplast precursor, putative, expressed no original description 	T
26.11	misc.alcohol dehydrogenases	MDP0000045577	 highly similar to ( 588) AT1G77120 | Symbols: ADH1, ADH, ATADH, ATADH1 | ADH1 (ALCOHOL DEHYDROGENASE 1); alcohol dehydrogenase | chr1:28975509-28977216 FORWARDhighly similar to ( 654) ADH_MALDO Alcohol dehydrogenase (EC 1.1.1.1) - Malus domestica (Apple) (Malus sylvestris)highly similar to ( 606) loc_os11g10480 12011.m28756 protein alcohol dehydrogenase 1, putative, expressed  no original description 	T
35.2	not assigned.unknown	MDP0000330325	  no original description 	T
26.12	misc.peroxidases	MDP0000124622	 moderately similar to ( 316) AT3G17070 | Symbols:  | peroxidase, putative | chr3:5821048-5823165 FORWARDweakly similar to ( 190) PER2_ARAHY Cationic peroxidase 2 precursor (EC 1.11.1.7) (PNPC2) - Arachis hypogaea (Peanut)moderately similar to ( 261) loc_os05g14260 12005.m05837 protein peroxidase 29 precursor, putative, expressed  no original description 	T
35.2	not assigned.unknown	MDP0000662927	 weakly similar to ( 124) AT5G51720 | Symbols:  | unknown protein | chr5:21009645-21010227 FORWARDweakly similar to ( 127) loc_os07g28400 12007.m07112 protein kinesin like protein, putative, expressed no original description 	T
11.1.10	lipid metabolism.FA synthesis and FA elongation.beta ketoacyl CoA synthase	MDP0000632951	 highly similar to ( 794) AT1G01120 | Symbols: KCS1 | KCS1 (3-KETOACYL-COA SYNTHASE 1); acyltransferase/ fatty acid elongase | chr1:57392-58978 REVERSEvery weakly similar to (63.2) CHS6_SOYBN Chalcone synthase 6 (EC 2.3.1.74) (Naringenin-chalcone synthase 6) - Glycine max (Soybean)highly similar to ( 651) loc_os05g49900 12005.m09060 protein fatty acid elongase, putative, expressed   no original description 	T
9.9	mitochondrial electron transport / ATP synthesis.F1-ATPase 	MDP0000390327	 highly similar to ( 839) AT5G08690 | Symbols:  | ATP synthase beta chain 2, mitochondrial | chr5:2825739-2828352 FORWARDhighly similar to ( 899) ATPBM_ORYSA ATP synthase subunit beta, mitochondrial precursor (EC 3.6.3.14) - Oryza sativa (Rice)highly similar to ( 899) loc_os05g47980 12005.m08870 protein ATP synthase beta chain, mitochondrial precursor, putative, expressed   no original description 	T
33.99	development.unspecified	MDP0000178931	 moderately similar to ( 206) AT3G14770 | Symbols:  | nodulin MtN3 family protein | chr3:4957787-4959202 REVERSEweakly similar to ( 172) loc_os01g36070 12001.m09904 protein mtN3 protein-like, putative, expressed no original description 	T
30.2.99	signalling.receptor kinases.misc	MDP0000926265	 weakly similar to ( 175) AT5G01740 | Symbols:  | FUNCTIONS IN: molecular_function unknown; INVOLVED IN: biological_process unknown; LOCATED IN: cellular_component unknown; EXPRESSED IN: 18 plant structures; EXPRESSED DURING: 9 growth stages; CONTAINS InterPro DOMAIN/s: Wound-induced protein, Wun1 (InterPro:IPR009798); BEST Arabidopsis thaliana protein match is: SAG20 (SENESCENCE ASSOCIATED GENE 20) (TAIR:AT3G10985.1); Has 49 Blast hits to 49 proteins in 12 species: Archae - 0; Bacteria - 4; Metazoa - 0; Fungi - 0; Plants - 45; Viruses - 0; Other Eukaryotes - 0 (source: NCBI BLink). | chr5:280793-281281 FORWARDvery weakly similar to (68.6) WUN1_SOLTU Wound-induced protein 1 - Solanum tuberosum (Potato)weakly similar to ( 126) loc_os05g27590 12005.m07047 protein wound-induced protein WI12 containing protein, expressed   no original description 	T
13.1.5.3.1	amino acid metabolism.synthesis.serine-glycine-cysteine group.cysteine.OASTL	MDP0000325786	 highly similar to ( 536) AT3G61440 | Symbols: ATCYSC1, CYSC1 | CYSC1 (CYSTEINE SYNTHASE C1); L-3-cyanoalanine synthase/ cysteine synthase | chr3:22736383-22737792 FORWARDmoderately similar to ( 410) CYSKP_SPIOL Cysteine synthase, chloroplast precursor (EC 2.5.1.47) (O-acetylserine sulfhydrylase) (O-acetylserine (Thiol)-lyase) (CSase B) (CS-B) (OAS-TL B) - Spinacia oleracea (Spinach)moderately similar to ( 494) loc_os04g08350 12004.m35563 protein cysteine synthase, chloroplast precursor, putative, expressed  no original description 	T
35.2	not assigned.unknown	MDP0000120224	 moderately similar to ( 293) AT4G21110 | Symbols:  | G10 family protein | chr4:11267673-11268774 FORWARDmoderately similar to ( 294) BD31A_ORYSA Protein BUD31 homolog 1 (Protein G10 homolog 1) - Oryza sativa (Rice)moderately similar to ( 294) loc_os04g55290 12004.m101674 protein protein G10 homolog 1, putative, expressed  no original description 	T
35.1	not assigned.no ontology	MDP0000209598	 moderately similar to ( 280) AT2G17120 | Symbols: LYM2 | LYM2 (LYSM DOMAIN GPI-ANCHORED PROTEIN 2 PRECURSOR) | chr2:7459156-7460648 FORWARDmoderately similar to ( 288) loc_os09g37600 12009.m06707 protein erwinia induced protein 1, putative, expressed  no original description 	T
20.2.1	stress.abiotic.heat	MDP0000678359	 weakly similar to ( 148) AT5G23240 | Symbols:  | DNAJ heat shock N-terminal domain-containing protein | chr5:7826857-7828534 REVERSEweakly similar to ( 191) loc_os06g28050 12006.m32226 protein 3Fe-4S ferredoxin, putative, expressed  no original description 	T
27.4	RNA.RNA binding	MDP0000173934	 moderately similar to ( 234) AT4G14300 | Symbols:  | heterogeneous nuclear ribonucleoprotein, putative / hnRNP, putative | chr4:8231179-8232785 FORWARDvery weakly similar to (73.6) ROC1_NICPL 30 kDa ribonucleoprotein, chloroplast precursor (CP-RBP30) - Nicotiana plumbaginifolia (Leadwort-leaved tobacco)moderately similar to ( 221) loc_os10g33230 12010.m06153 protein heterogeneous nuclear ribonucleoprotein 27C, putative, expressed   no original description 	T
29.3.1	protein.targeting.nucleus	MDP0000208838	 highly similar to ( 621) AT5G40480 | Symbols: EMB3012 | EMB3012 (embryo defective 3012) | chr5:16213361-16223982 FORWARDmoderately similar to ( 480) loc_os09g39210 12009.m06860 protein nuclear pore protein-like, putative, expressed  no original description 	T
29.2.1.2.2.24	protein.synthesis.ribosomal protein.eukaryotic.60S subunit.L24	MDP0000155392	 moderately similar to ( 233) AT2G44860 | Symbols:  | 60S ribosomal protein L24, putative | chr2:18501034-18502128 REVERSEvery weakly similar to (79.3) RL24_CICAR 60S ribosomal protein L24 - Cicer arietinum (Chickpea) (Garbanzo)moderately similar to ( 222) loc_os07g19190 12007.m06356 protein ribosome biogenesis protein RLP24, putative, expressed  no original description 	T
29.2.1.2.2.34	protein.synthesis.ribosomal protein.eukaryotic.60S subunit.L34	MDP0000486185	 weakly similar to ( 186) AT1G69620 | Symbols: RPL34 | RPL34 (RIBOSOMAL PROTEIN L34); structural constituent of ribosome | chr1:26189900-26191081 FORWARDweakly similar to ( 190) RL34_PEA 60S ribosomal protein L34 - Pisum sativum (Garden pea)weakly similar to ( 176) loc_os09g24690 12009.m05623 protein 60S ribosomal protein L34, putative, expressed  no original description 	T
10.1.10	cell wall.precursor synthesis.UDP-glucose 4,6-dehydratase	MDP0000240463	 highly similar to ( 519) AT1G63000 | Symbols: NRS/ER, UER1 | NRS/ER (NUCLEOTIDE-RHAMNOSE SYNTHASE/EPIMERASE-REDUCTASE); UDP-4-keto-6-deoxy-glucose-3,5-epimerase/ UDP-4-keto-rhamnose-4-keto-reductase/ dTDP-4-dehydrorhamnose 3,5-epimerase/ dTDP-4-dehydrorhamnose reductase | chr1:23342510-23343859 FORWARDhighly similar to ( 507) loc_os02g45540 12002.m09591 protein dTDP-4-dehydrorhamnose reductase, putative, expressed   no original description 	T
29.5.11.4.3.2	protein.degradation.ubiquitin.E3.SCF.FBOX	MDP0000170855	 weakly similar to ( 164) AT4G24210 | Symbols: SLY1 | SLY1 (SLEEPY1) | chr4:12563658-12564113 FORWARDweakly similar to ( 106) GID2_ORYSA F-box protein GID2 (Gibberellin-insensitive dwarf protein 2) (Protein GIBBERELLIN INSENSITIVE DWARF2) - Oryza sativa (Rice)very weakly similar to (57.4) loc_os03g10040 12003.m06458 protein F-box protein GID2, putative, expressed  TrbL  no original description 	T
29.3.4.99	protein.targeting.secretory pathway.unspecified	MDP0000156618	 nearly identical (1312) AT3G63460 | Symbols:  | WD-40 repeat family protein | chr3:23431009-23437241 REVERSEnearly identical (1115) loc_os07g46370 12007.m08854 protein EMB2221, putative, expressed  no original description 	T
34.16	transport.ABC transporters and multidrug resistance systems	MDP0000094058	 highly similar to ( 976) AT3G21090 | Symbols:  | ABC transporter family protein | chr3:7391497-7394933 REVERSEweakly similar to ( 198) PDR15_ORYSA Pleiotropic drug resistance protein 15 - Oryza sativa (Rice)highly similar to ( 850) loc_os05g13520 12005.m05767 protein CER5, putative, expressed no original description 	T
26.3	misc.gluco-, galacto- and mannosidases	MDP0000938307	 moderately similar to ( 431) AT1G02850 | Symbols: BGLU11 | BGLU11 (BETA GLUCOSIDASE 11); catalytic/ cation binding / hydrolase, hydrolyzing O-glycosyl compounds | chr1:630569-633085 FORWARDmoderately similar to ( 254) BGLC_MAIZE Beta-glucosidase, chloroplast precursor (EC 3.2.1.21) (Gentiobiase) (Cellobiase) (Beta-D-glucoside glucohydrolase) - Zea mays (Maize)moderately similar to ( 397) loc_os05g30250 12005.m083706 protein non-cyanogenic beta-glucosidase precursor, putative, expressed no original description 	T
27.1	RNA.processing	MDP0000366587	 weakly similar to ( 160) AT1G21190 | Symbols:  | small nuclear ribonucleoprotein, putative / snRNP, putative / Sm protein, putative | chr1:7419989-7420856 REVERSEweakly similar to ( 166) loc_os01g64690 12001.m12583 protein U6 snRNA-associated Sm-like protein LSm3, putative, expressed  no original description 	T
29.4.1.57	protein.postranslational modification.kinase.receptor like cytoplasmatic kinase VII	MDP0000138626	 highly similar to ( 618) AT5G57670 | Symbols:  | protein kinase family protein | chr5:23360531-23363694 REVERSEmoderately similar to ( 201) NORK_MEDTR Nodulation receptor kinase precursor (EC 2.7.11.1) (Does not make infections protein 2) (Symbiosis receptor-like kinase) (MtSYMRK) - Medicago truncatula (Barrel medic)highly similar to ( 553) loc_os08g15060 12008.m05629 protein ATP binding protein, putative, expressed  no original description 	T
26.2	misc.UDP glucosyl and glucoronyl transferases	MDP0000124585	 moderately similar to ( 481) AT1G22380 | Symbols: AtUGT85A3 | AtUGT85A3 (UDP-glucosyl transferase 85A3); glucuronosyltransferase/ transcription factor/ transferase, transferring glycosyl groups | chr1:7900522-7902332 REVERSEweakly similar to ( 162) UFO3_MAIZE Anthocyanidin 3-O-glucosyltransferase (EC 2.4.1.115) (Flavonol 3-O-glucosyltransferase) (UDP-glucose flavonoid 3-O-glucosyltransferase) (Bronze-1) (Bz-W22 allele) - Zea mays (Maize)moderately similar to ( 452) loc_os04g37820 12004.m08771 protein cytokinin-O-glucosyltransferase 2, putative, expressed  no original description 	T
35.1	not assigned.no ontology	MDP0000310704	 moderately similar to ( 489) AT4G24140 | Symbols:  | hydrolase, alpha/beta fold family protein | chr4:12530032-12533664 REVERSEmoderately similar to ( 310) loc_os10g38860 12010.m06653 protein catalytic/ hydrolase, putative, expressedCOG3889  no original description 	T
27.3.6	RNA.regulation of transcription.bHLH,Basic Helix-Loop-Helix family	MDP0000271420	 moderately similar to ( 263) AT1G68810 | Symbols:  | basic helix-loop-helix (bHLH) family protein | chr1:25861289-25862882 FORWARDmoderately similar to ( 210) loc_os03g15440 12003.m06981 protein DNA binding protein, putative, expressed  no original description 	T
29.5.11.3	protein.degradation.ubiquitin.E2	MDP0000216487	 moderately similar to ( 285) AT3G57870 | Symbols: AHUS5, EMB1637, SCE1, SCE1A, ATSCE1 | SCE1 (SUMO CONJUGATION ENZYME 1); SUMO ligase | chr3:21428831-21430110 REVERSEweakly similar to ( 114) UBC2_MEDSA Ubiquitin-conjugating enzyme E2-17 kDa (EC 6.3.2.19) (Ubiquitin-protein ligase) (Ubiquitin carrier protein) - Medicago sativa (Alfalfa)moderately similar to ( 303) loc_os03g03130 12003.m05850 protein ubiquitin-conjugating enzyme E2 I, putative, expressed no original description 	T
11.1.1	lipid metabolism.FA synthesis and FA elongation.Acetyl CoA Carboxylation	MDP0000385310	 weakly similar to ( 177) AT3G56130 | Symbols:  | biotin/lipoyl attachment domain-containing protein | chr3:20827887-20829007 FORWARD  no original description 	T
30.4.1	signalling.phosphinositides.phosphatidylinositol-4-phosphate 5-kinase	MDP0000253374	 highly similar to ( 852) AT1G60890 | Symbols:  | phosphatidylinositol-4-phosphate 5-kinase family protein | chr1:22412452-22416338 REVERSEhighly similar to ( 667) PI5K1_ORYSA Phosphatidylinositol-4-phosphate 5-kinase 1 precursor (EC 2.7.1.68) (1-phosphatidylinositol-4-phosphate kinase) (PIP5K) (PtdIns(4)P-5-kinase) (Diphosphoinositide kinase) - Oryza sativa (Rice)highly similar to ( 675) loc_os03g49510 12003.m09950 protein phosphatidylinositol-4-phosphate 5-kinase 1 precursor, putative, expressed  no original description 	T
29.2.1.2.2.17	protein.synthesis.ribosomal protein.eukaryotic.60S subunit.L17	MDP0000170863	 moderately similar to ( 303) AT1G67430 | Symbols:  | 60S ribosomal protein L17 (RPL17B) | chr1:25262209-25263627 FORWARDmoderately similar to ( 299) RL172_HORVU 60S ribosomal protein L17-2 - Hordeum vulgare (Barley)moderately similar to ( 300) loc_os09g08430 12009.m04110 protein 60S ribosomal protein L17, putative, expressed no original description 	T
29.5.7	protein.degradation.metalloprotease	MDP0000759204	 highly similar to ( 538) AT2G32480 | Symbols:  | membrane-associated zinc metalloprotease, putative | chr2:13788679-13790022 REVERSEmoderately similar to ( 378) loc_os03g38230 12003.m08922 protein expressed protein  no original description 	T
29.2.1.1.1.2.5	protein.synthesis.ribosomal protein.prokaryotic.chloroplast.50S subunit.L5	MDP0000419618	 very weakly similar to (55.1) PSRP5_SPIOL Plastid-specific 50S ribosomal protein 5 alpha, chloroplast precursor (PSRP-5) (50S ribosomal protein L40) (CL40) [Contains: Plastid-specific 50S ribosomal protein 5 beta; Plastid-specific 50S ribosomal protein 5 gamma] - Spinacia oler no original description 	T
29.2.1.1.1.2.40	protein.synthesis.ribosomal protein.prokaryotic.chloroplast.50S subunit.L40	MDP0000419618	 very weakly similar to (55.1) PSRP5_SPIOL Plastid-specific 50S ribosomal protein 5 alpha, chloroplast precursor (PSRP-5) (50S ribosomal protein L40) (CL40) [Contains: Plastid-specific 50S ribosomal protein 5 beta; Plastid-specific 50S ribosomal protein 5 gamma] - Spinacia oler no original description 	T
30.3	signalling.calcium	MDP0000697565	 weakly similar to ( 121) AT5G54490 | Symbols: PBP1 | PBP1 (PINOID-BINDING PROTEIN 1); calcium ion binding / protein binding | chr5:22121458-22121841 FORWARDweakly similar to ( 125) loc_os06g46950 12006.m09221 protein EF-hand Ca2+-binding protein CCD1, putative, expressed no original description 	T
16.1.2.3	secondary metabolism.isoprenoids.mevalonate pathway.HMG-CoA reductase	MDP0000164847	 moderately similar to ( 282) AT1G76490 | Symbols: HMG1, HMGR1 | HMG1 (HYDROXY METHYLGLUTARYL COA REDUCTASE 1); hydroxymethylglutaryl-CoA reductase | chr1:28695801-28698206 FORWARDmoderately similar to ( 282) HMDH1_SOLTU 3-hydroxy-3-methylglutaryl-coenzyme A reductase 1 (EC 1.1.1.34) (HMG-CoA reductase 1) (HMGR1) (HMGR) - Solanum tuberosum (Potato)moderately similar to ( 227) loc_os09g31970 12009.m06287 protein 3-hydroxy-3-methylglutaryl-coenzyme A reductase 3, putative, expressed   no original description 	T
27.3.25	RNA.regulation of transcription.MYB domain transcription factor family	MDP0000231408	 weakly similar to ( 153) AT5G04110 | Symbols:  | DNA topoisomerase II family protein | chr5:1110757-1114112 REVERSEweakly similar to ( 139) loc_os04g08410 12004.m06129 protein expressed protein  no original description 	T
27.3.62	RNA.regulation of transcription.Nucleosome/chromatin assembly factor group	MDP0000362980	 weakly similar to ( 134) AT3G51880 | Symbols: HMGB1, NFD1 | HMGB1 (HIGH MOBILITY GROUP B 1); DNA binding / chromatin binding / structural constituent of chromatin / transcription factor | chr3:19247241-19248491 REVERSEweakly similar to ( 103) HMGL_SOYBN HMG1/2-like protein (SB11 protein) - Glycine max (Soybean)very weakly similar to (99.0) loc_os09g37910 12009.m22188 protein HMG1/2-like protein, putative, expressed no original description 	T
30.5	signalling.G-proteins	MDP0000312242	 highly similar to ( 667) AT5G19320 | Symbols: RANGAP2 | RANGAP2 (RAN GTPASE ACTIVATING PROTEIN 2); RAN GTPase activator | chr5:6505310-6506947 REVERSEhighly similar to ( 607) loc_os05g46560 12005.m27945 protein ran GTPase activating protein, putative, expressed no original description 	T
35.1	not assigned.no ontology	MDP0000663593	 weakly similar to ( 111) AT5G57370 | Symbols:  | unknown protein | chr5:23244811-23245470 FORWARDvery weakly similar to (96.3) loc_os02g26349 12002.m77802 protein splicing factor, arginine/serine-rich 7, putative, expressed  no original description 	T
30.2.17	signalling.receptor kinases.DUF 26	MDP0000165711	 highly similar to ( 894) AT1G11410 | Symbols:  | S-locus protein kinase, putative | chr1:3841286-3844284 FORWARDmoderately similar to ( 343) SLSG3_BRAOL S-locus-specific glycoprotein S13 precursor (SLSG-13) (Fragment) - Brassica oleracea (Wild cabbage)highly similar to ( 696) loc_os03g35600 12003.m08696 protein serine/threonine-protein kinase receptor precursor, putative, expressed   no original description 	T
30.2.24	signalling.receptor kinases.S-locus glycoprotein like	MDP0000165711	 highly similar to ( 894) AT1G11410 | Symbols:  | S-locus protein kinase, putative | chr1:3841286-3844284 FORWARDmoderately similar to ( 343) SLSG3_BRAOL S-locus-specific glycoprotein S13 precursor (SLSG-13) (Fragment) - Brassica oleracea (Wild cabbage)highly similar to ( 696) loc_os03g35600 12003.m08696 protein serine/threonine-protein kinase receptor precursor, putative, expressed   no original description 	T
34.2	transport.sugars	MDP0000381084	 highly similar to ( 915) AT4G35300 | Symbols:  | TMT2 (TONOPLAST MONOSACCHARIDE TRANSPORTER2); carbohydrate transmembrane transporter/ nucleoside transmembrane transporter/ sugar:hydrogen symporter | chr4:16796432-16799071 REVERSEvery weakly similar to (93.6) STA_RICCO Sugar carrier protein A - Ricinus communis (Castor bean)highly similar to ( 919) loc_os02g13560 12002.m100038 protein sugar transporter type 2a, putative, expressed   no original description 	T
35.2	not assigned.unknown	MDP0000239951	 moderately similar to ( 291) loc_os05g49060 12005.m08976 protein expressed protein  no original description 	T
20.2.1	stress.abiotic.heat	MDP0000795157	 moderately similar to ( 439) AT2G20560 | Symbols:  | DNAJ heat shock family protein | chr2:8848353-8849815 REVERSEweakly similar to ( 105) DNJH_CUCSA DnaJ protein homolog (DNAJ-1) - Cucumis sativus (Cucumber)moderately similar to ( 436) loc_os05g48810 12005.m27974 protein dnaJ homolog subfamily B member 5, putative, expressed   no original description 	T
23.4.10	nucleotide metabolism.phosphotransfer and pyrophosphatases.nucleoside diphosphate kinase	MDP0000322880	 moderately similar to ( 268) AT4G09320 | Symbols: NDPK1 | NDPK1; ATP binding / nucleoside diphosphate kinase | chr4:5923424-5924366 FORWARDmoderately similar to ( 270) NDK1_PEA Nucleoside diphosphate kinase 1 (EC 2.7.4.6) (Nucleoside diphosphate kinase I) (NDK I) (NDP kinase I) (NDPK I) - Pisum sativum (Garden pea)moderately similar to ( 249) loc_os07g30970 12007.m079686 protein nucleoside diphosphate kinase 1, putative, expressed no original description 	T
30.2.11	signalling.receptor kinases.leucine rich repeat XI	MDP0000256017	 moderately similar to ( 465) AT2G34930 | Symbols:  | disease resistance family protein | chr2:14737169-14739886 REVERSEweakly similar to ( 196) RPK1_IPONI Receptor-like protein kinase precursor (EC 2.7.11.1) - Ipomoea nil (Japanese morning glory) (Pharbitis nil)highly similar to ( 531) loc_os06g04810 12006.m05210 protein leucine-rich repeat receptor protein kinase EXS precursor, putative, expressed  no original description 	T
27.4	RNA.RNA binding	MDP0000193241	 very weakly similar to (81.3) AT3G62800 | Symbols:  | DRB4 (DOUBLE-STRANDED-RNA-BINDING PROTEIN 4); double-stranded RNA binding / protein binding | chr3:23225749-23227163 REVERSEweakly similar to ( 102) loc_os01g56520 12001.m11808 protein double-stranded RNA binding motif family protein, expressed  no original description 	T
35.2	not assigned.unknown	MDP0000350060	  no original description 	T
17.2.3	hormone metabolism.auxin.induced-regulated-responsive-activated	MDP0000126316	 moderately similar to ( 369) AT1G60710 | Symbols: ATB2 | ATB2; oxidoreductase | chr1:22355073-22356627 REVERSEmoderately similar to ( 359) A115_TOBAC Auxin-induced protein PCNT115 - Nicotiana tabacum (Common tobacco)moderately similar to ( 456) loc_os10g28320 12010.m22021 protein auxin-induced protein PCNT115, putative, expressed no original description 	T
29.5.11.4.3.2	protein.degradation.ubiquitin.E3.SCF.FBOX	MDP0000928355	 weakly similar to ( 164) AT3G06240 | Symbols:  | F-box family protein | chr3:1887336-1888619 FORWARDvery weakly similar to (87.4) loc_os09g27660 12009.m059992 protein ANTH domain containing protein, expressed  no original description 	T
35.2	not assigned.unknown	MDP0000449309	 moderately similar to ( 304) AT5G14970 | Symbols:  | unknown protein | chr5:4847368-4848760 FORWARDweakly similar to ( 142) loc_os03g41080 12003.m09177 protein seed maturation protein PM23, putative, expressed  no original description 	T
29.2.1.2.2.24	protein.synthesis.ribosomal protein.eukaryotic.60S subunit.L24	MDP0000712717	 moderately similar to ( 212) AT3G53020 | Symbols: STV1, RPL24B, RPL24 | STV1 (SHORT VALVE1); structural constituent of ribosome | chr3:19660749-19661912 REVERSEmoderately similar to ( 213) RL24_CICAR 60S ribosomal protein L24 - Cicer arietinum (Chickpea) (Garbanzo)moderately similar to ( 211) loc_os01g59990 12001.m43406 protein 60S ribosomal protein L24, putative, expressed no original description 	T
31.1	cell.organisation	MDP0000142804	 moderately similar to ( 480) AT1G18450 | Symbols: ATARP4, ARP4 | ATARP4; structural constituent of cytoskeleton | chr1:6348199-6351766 FORWARDweakly similar to ( 171) ACT_VOLCA Actin - Volvox carterimoderately similar to ( 478) loc_os08g04280 12008.m04572 protein actin-like protein 6A, putative, expressed  no original description 	T
10.7	cell wall.modification	MDP0000195798	 moderately similar to ( 468) AT2G39700 | Symbols: ATEXPA4, ATEXP4, ATHEXP ALPHA 1.6 | ATEXPA4 (ARABIDOPSIS THALIANA EXPANSIN A4) | chr2:16544246-16545434 REVERSEmoderately similar to ( 437) EXPA7_ORYSA Expansin-A7 precursor (OsEXPA7) (Alpha-expansin-7) (OsEXP7) (OsaEXPa1.26) - Oryza sativa (Rice)moderately similar to ( 437) loc_os03g60720 12003.m10961 protein alpha-expansin 6 precursor, putative, expressed  no original description 	T
29.2.1.2.2.82	protein.synthesis.ribosomal protein.eukaryotic.60S subunit.P2	MDP0000177670	 very weakly similar to (70.5) AT2G27720 | Symbols:  | 60S acidic ribosomal protein P2 (RPP2A) | chr2:11818696-11819370 FORWARDvery weakly similar to (60.5) RLA2B_MAIZE 60S acidic ribosomal protein P2B - Zea mays (Maize)very weakly similar to (60.5) loc_os02g32760 12002.m08369 protein 60S acidic ribosomal protein P2A, putative, expressed   no original description 	T
35.2	not assigned.unknown	MDP0000223410	 moderately similar to ( 317) AT5G25170 | Symbols:  | unknown protein | chr5:8693257-8694438 FORWARDmoderately similar to ( 229) loc_os03g10200 12003.m35075 protein expressed protein  no original description 	T
6.5	gluconeogenesis / glyoxylate cycle.pyruvate dikinase	MDP0000789992	 nearly identical (1511) AT4G15530 | Symbols: PPDK | PPDK (pyruvate orthophosphate dikinase); kinase/ pyruvate, phosphate dikinase | chr4:8864828-8870748 REVERSEnearly identical (1583) PPDK_MESCR Pyruvate, phosphate dikinase, chloroplast precursor (EC 2.7.9.1) (Pyruvate, orthophosphate dikinase) - Mesembryanthemum crystallinum (Common ice plant)nearly identical (1476) loc_os05g33570 12005.m083718 protein pyruvate, phosphate dikinase, chloroplast precursor, putative, expressed   no original description 	T
27.3.40	RNA.regulation of transcription.Aux/IAA family	MDP0000296324	 moderately similar to ( 206) AT3G15540 | Symbols: IAA19, MSG2 | IAA19 (INDOLE-3-ACETIC ACID INDUCIBLE 19); transcription factor | chr3:5264100-5265378 FORWARDmoderately similar to ( 249) AUX22_SOYBN Auxin-induced protein AUX22 - Glycine max (Soybean)weakly similar to ( 144) loc_os03g43400 12003.m09386 protein OsIAA11 - Auxin-responsive Aux/IAA gene family member, expressed no original description 	T
27.1.1	RNA.processing.splicing	MDP0000256723	 weakly similar to ( 156) AT1G23860 | Symbols: SRZ-21, SRZ21 | RSZP21 (RS-CONTAINING ZINC FINGER PROTEIN 21); protein binding | chr1:8428091-8429164 REVERSEweakly similar to ( 149) loc_os02g39720 12002.m33763 protein splicing factor, arginine/serine-rich 7, putative, expressed   no original description 	T
35.2	not assigned.unknown	MDP0000073670	 nearly identical (1262) AT5G12370 | Symbols: SEC10 | SEC10 (EXOCYST COMPLEX COMPONENT SEC10) | chr5:4003002-4008445 REVERSEnearly identical (1135) EXOC5_ORYSA Exocyst complex component 5 (Exocyst complex component Sec10) - Oryza sativa (Rice)nearly identical (1135) loc_os12g13590 12012.m26747 protein exocyst complex component 5, putative, expressed no original description 	T
29.4.1.57	protein.postranslational modification.kinase.receptor like cytoplasmatic kinase VII	MDP0000158475	 highly similar to ( 699) AT5G56890 | Symbols:  | protein kinase family protein | chr5:23010801-23015559 REVERSEmoderately similar to ( 224) NORK_PEA Nodulation receptor kinase precursor (EC 2.7.11.1) - Pisum sativum (Garden pea)highly similar to ( 665) loc_os01g09860 12001.m07610 protein ATP binding protein, putative, expressed  no original description 	T
1.1.1.2	PS.lightreaction.photosystem II.PSII polypeptide subunits	MDP0000764147	 moderately similar to ( 276) AT3G55330 | Symbols: PPL1 | PPL1 (PsbP-like protein 1); calcium ion binding | chr3:20514031-20515275 REVERSEvery weakly similar to (57.4) PSBP2_TOBAC Oxygen-evolving enhancer protein 2-2, chloroplast precursor (OEE2) (23 kDa subunit of oxygen evolving system of photosystem II) (OEC 23 kDa subunit) (23 kDa thylakoid membrane protein) - Nicotiana tabacum (Common tobacco)moderately similar to ( 266) loc_os08g25900 12008.m06605 protein chloroplast oxygen-evolving complex/thylakoid lumenal 25.6kDa protein, putative, expressed   no original description 	T
9.9	mitochondrial electron transport / ATP synthesis.F1-ATPase 	MDP0000655872	 moderately similar to ( 210) AT4G26210 | Symbols:  | mitochondrial ATP synthase g subunit family protein | chr4:13282370-13283118 FORWARDweakly similar to ( 187) loc_os01g41610 12001.m43082 protein hydrogen-transporting ATP synthase, rotational mechanism, putative, expressed  no original description 	T
35.2	not assigned.unknown	MDP0000534197	 moderately similar to ( 309) AT1G67330 | Symbols:  | unknown protein | chr1:25214118-25214993 FORWARDmoderately similar to ( 235) loc_os11g29780 12011.m06887 protein plant-specific domain TIGR01627 family protein, expressed   no original description 	T
28.1	DNA.synthesis/chromatin structure	MDP0000271168	 moderately similar to ( 483) AT4G29910 | Symbols: ORC5, ATORC5 | ORC5 (ORIGIN RECOGNITION COMPLEX PROTEIN 5); protein binding | chr4:14628154-14630055 REVERSEmoderately similar to ( 335) loc_os03g55200 12003.m10435 protein origin recognition complex subunit 5, putative, expressed no original description 	T
27.1.1	RNA.processing.splicing	MDP0000568145	 moderately similar to ( 298) AT3G27550 | Symbols:  | group II intron splicing factor CRS1-related | chr3:10208010-10209899 REVERSEmoderately similar to ( 275) loc_os05g05300 12005.m05057 protein CRS1 / YhbY domain containing protein, expressed  no original description 	T
34.99	transport.misc	MDP0000298711	 moderately similar to ( 452) AT5G01990 | Symbols:  | auxin efflux carrier family protein | chr5:377373-379600 REVERSEmoderately similar to ( 386) loc_os01g60230 12001.m150756 protein auxin hydrogen symporter, putative, expressed  no original description 	T
35.2	not assigned.unknown	MDP0000319029	 moderately similar to ( 358) AT5G17460 | Symbols:  | unknown protein | chr5:5757582-5758867 REVERSEmoderately similar to ( 331) loc_os01g03500 12001.m06988 protein expressed protein TrbL  no original description 	T
34.99	transport.misc	MDP0000144086	 moderately similar to ( 458) AT2G38330 | Symbols:  | MATE efflux family protein | chr2:16064571-16067318 FORWARDmoderately similar to ( 413) loc_os12g01580 12012.m26797 protein antiporter/ drug transporter, putative, expressed no original description 	T
29.5.11.4.2	protein.degradation.ubiquitin.E3.RING	MDP0000308051	 weakly similar to ( 107) AT5G59350 | Symbols:  | unknown protein | chr5:23941132-23941995 FORWARDvery weakly similar to (73.6) EL5_ORYSA E3 ubiquitin-protein ligase EL5 (EC 6.3.2.-) - Oryza sativa (Rice)weakly similar to ( 110) loc_os11g47730 12011.m28698 protein expressed protein   no original description 	T
34.22	transport.cyclic nucleotide or calcium regulated channels	MDP0000261979	 moderately similar to ( 370) AT5G53130 | Symbols: CNGC1, ATCNGC1 | CNGC1 (CYCLIC NUCLEOTIDE GATED CHANNEL 1); calmodulin binding / cation channel/ cyclic nucleotide binding / intracellular cyclic nucleotide activated cation channel/ inward rectifier potassium channel | chr5:21537830-21540490 REVERSEmoderately similar to ( 314) loc_os06g33570 12006.m07894 protein cyclic nucleotide-gated ion channel 1, putative, expressed  no original description 	T
30.3	signalling.calcium	MDP0000586415	 weakly similar to ( 164) AT1G66400 | Symbols:  | calmodulin-related protein, putative | chr1:24770856-24771329 REVERSEweakly similar to ( 104) CALM_CHLRE Calmodulin (CaM) - Chlamydomonas reinhardtiiweakly similar to ( 135) loc_os05g13580 12005.m05773 protein calmodulin-related protein 2, touch-induced, putative, expressed  no original description 	T
35.1	not assigned.no ontology	MDP0000320151	 moderately similar to ( 347) AT2G25110 | Symbols: SDF2 | SDF2 (STROMAL CELL-DERIVED FACTOR 2-LIKE PROTEIN PRECURSOR) | chr2:10684428-10685838 FORWARDmoderately similar to ( 330) loc_os08g17680 12008.m05888 protein stromal cell-derived factor 2-like protein 1 precursor, putative, expressed  no original description 	T
31.1	cell.organisation	MDP0000176073	 nearly identical (2590) AT2G35630 | Symbols: MOR1, GEM1 | MOR1 (MICROTUBULE ORGANIZATION 1); microtubule binding | chr2:14966828-14980361 FORWARDnearly identical (2481) loc_os01g60040 12001.m12135 protein cytoskeleton-associated protein 5, putative, expressed  no original description 	T
31.1	cell.organisation	MDP0000308678	 moderately similar to ( 209) AT5G59880 | Symbols: ADF3 | ADF3 (ACTIN DEPOLYMERIZING FACTOR 3); actin binding | chr5:24120382-24121628 FORWARDmoderately similar to ( 218) ADF2_PETHY Actin-depolymerizing factor 2 (ADF 2) - Petunia hybrida (Petunia)moderately similar to ( 208) loc_os05g02250 12005.m64199 protein expressed protein no original description 	T
35.1	not assigned.no ontology	MDP0000242152	 weakly similar to ( 197) AT5G21920 | Symbols:  | YGGT family protein | chr5:7241350-7242765 REVERSEweakly similar to ( 153) loc_os03g08080 12003.m06317 protein YGGT family protein, expressed  no original description 	T
27.3.6	RNA.regulation of transcription.bHLH,Basic Helix-Loop-Helix family	MDP0000231650	 moderately similar to ( 254) AT1G10120 | Symbols:  | DNA binding / transcription factor | chr1:3304228-3305984 REVERSEweakly similar to ( 186) loc_os06g16400 12006.m32045 protein BHLH transcription factor, putative, expressed no original description 	T
34.9	transport.metabolite transporters at the mitochondrial membrane	MDP0000317763	 moderately similar to ( 475) AT5G46800 | Symbols: BOU | BOU (A BOUT DE SOUFFLE); binding / transporter | chr5:18988779-18989810 REVERSEvery weakly similar to (92.8) BT1_MAIZE Protein brittle-1, chloroplast precursor - Zea mays (Maize)moderately similar to ( 500) loc_os10g42299 12010.m065349 protein mitochondrial carnitine/acylcarnitine carrier-like protein, putative, expressed no original description 	T
29.2.1.2.1.27	protein.synthesis.ribosomal protein.eukaryotic.40S subunit.S27	MDP0000155887	 weakly similar to ( 145) AT3G61110 | Symbols: ARS27A | ARS27A (ARABIDOPSIS RIBOSOMAL PROTEIN S27); structural constituent of ribosome | chr3:22611710-22612632 FORWARDweakly similar to ( 141) RS27_HORVU 40S ribosomal protein S27 (Manganese efficiency-related protein 1) - Hordeum vulgare (Barley)weakly similar to ( 139) loc_os02g27769 12002.m100798 protein 40S ribosomal protein S27, putative, expressed no original description 	T
35.2	not assigned.unknown	MDP0000190843	 moderately similar to ( 214) AT2G48070 | Symbols: RPH1 | RPH1 (RESISTANCE TO PHYTOPHTHORA 1) | chr2:19662863-19663773 FORWARDmoderately similar to ( 223) loc_os05g33520 12005.m07584 protein expressed protein no original description 	T
35.2	not assigned.unknown	MDP0000178042	   no original description 	T
27.3.62	RNA.regulation of transcription.Nucleosome/chromatin assembly factor group	MDP0000607529	 weakly similar to ( 152) AT1G20696 | Symbols: HMGB3, NFD3, NFD03 | HMGB3 (HIGH MOBILITY GROUP B 3); DNA binding / chromatin binding / structural constituent of chromatin / transcription factor | chr1:7179825-7181170 FORWARDweakly similar to ( 146) HMGL_SOYBN HMG1/2-like protein (SB11 protein) - Glycine max (Soybean)weakly similar to ( 130) loc_os09g37910 12009.m22188 protein HMG1/2-like protein, putative, expressed no original description 	T
29.5.11.4.5.2	protein.degradation.ubiquitin.E3.BTB/POZ Cullin3.BTB/POZ	MDP0000156128	 weakly similar to ( 155) AT4G08455 | Symbols:  | BTB/POZ domain-containing protein | chr4:5375891-5376922 FORWARDweakly similar to ( 130) loc_os07g15490 12007.m05993 protein TD and POZ domain-containing protein 1, putative, expressed no original description 	T
26.2	misc.UDP glucosyl and glucoronyl transferases	MDP0000058337	 moderately similar to ( 449) AT3G21760 | Symbols:  | UDP-glucoronosyl/UDP-glucosyl transferase family protein | chr3:7667099-7668556 FORWARDweakly similar to ( 146) ZOX_PHAVU Zeatin O-xylosyltransferase (EC 2.4.2.40) (Zeatin O-beta-D-xylosyltransferase) - Phaseolus vulgaris (Kidney bean) (French bean)moderately similar to ( 308) loc_os07g32010 12007.m07467 protein anthocyanidin 5,3-O-glucosyltransferase, putative, expressed   TrbL  no original description 	T
31.1	cell.organisation	MDP0000233014	 moderately similar to ( 463) AT1G18450 | Symbols: ATARP4, ARP4 | ATARP4; structural constituent of cytoskeleton | chr1:6348199-6351766 FORWARDweakly similar to ( 174) ACT3_ORYSA Actin-3 - Oryza sativa (Rice)moderately similar to ( 437) loc_os08g04280 12008.m04572 protein actin-like protein 6A, putative, expressed   no original description 	T
35.2	not assigned.unknown	MDP0000283400	  no original description 	T
34.14	transport.unspecified cations	MDP0000673414	 highly similar to ( 807) AT5G23630 | Symbols:  | ATPase E1-E2 type family protein / haloacid dehalogenase-like hydrolase familiy protein | chr5:7960756-7967644 REVERSEhighly similar to ( 796) loc_os05g33390 12005.m07571 protein cation-transporting ATPase, putative, expressed no original description 	T
30.5	signalling.G-proteins	MDP0000237668	 moderately similar to ( 443) AT4G03100 | Symbols:  | rac GTPase activating protein, putative | chr4:1374361-1375913 FORWARDmoderately similar to ( 410) loc_os12g34840 12012.m07277 protein rac GTPase activator, putative, expressed no original description 	T
29.2.4	protein.synthesis.elongation	MDP0000487255	 highly similar to ( 853) AT5G60390 | Symbols:  | elongation factor 1-alpha / EF-1-alpha | chr5:24289226-24290675 FORWARDhighly similar to ( 861) EF1A_ORYSA Elongation factor 1-alpha (EF-1-alpha) - Oryza sativa (Rice)highly similar to ( 861) loc_os03g08050 12003.m78808 protein elongation factor 1-alpha, putative, expressed   no original description 	T
31.1	cell.organisation	MDP0000152676	 weakly similar to ( 152) AT3G01750 | Symbols:  | ankyrin repeat family protein | chr3:270615-272691 FORWARDweakly similar to ( 145) loc_os05g23320 12005.m06627 protein expressed protein no original description 	T
27.3.99	RNA.regulation of transcription.unclassified	MDP0000229126	 highly similar to ( 566) AT5G07030 | Symbols:  | aspartic-type endopeptidase | chr5:2183600-2185717 REVERSEmoderately similar to ( 468) loc_os07g46480 12007.m08865 protein aspartic proteinase nepenthesin-1 precursor, putative, expressed   no original description 	T
34.13	transport.peptides and oligopeptides	MDP0000316682	 highly similar to ( 970) AT5G13400 | Symbols:  | proton-dependent oligopeptide transport (POT) family protein | chr5:4296854-4299079 REVERSEhighly similar to ( 739) loc_os01g01360 12001.m06782 protein peptide transporter PTR2, putative, expressed  no original description 	T
35.1	not assigned.no ontology	MDP0000242071	 highly similar to ( 656) AT5G07830 | Symbols: AtGUS2 | AtGUS2 (Arabidopsis thaliana glucuronidase 2); beta-glucuronidase | chr5:2504168-2506567 FORWARDhighly similar to ( 629) loc_os12g38920 12012.m073868 protein heparanase-like protein 2 precursor, putative, expressed MSP1_C  no original description 	T
30.2.17	signalling.receptor kinases.DUF 26	MDP0000227119	 very weakly similar to (56.6) AT2G46495 | Symbols:  | zinc finger (C3HC4-type RING finger) family protein | chr2:19080254-19085704 REVERSEvery weakly similar to (77.8) loc_os01g02300 12001.m06872 protein receptor kinase ORK10, putative, expressed  no original description 	T
30.2.3	signalling.receptor kinases.leucine rich repeat III	MDP0000425438	 highly similar to ( 663) AT2G23300 | Symbols:  | leucine-rich repeat transmembrane protein kinase, putative | chr2:9914608-9917130 FORWARDweakly similar to ( 127) RPK1_IPONI Receptor-like protein kinase precursor (EC 2.7.11.1) - Ipomoea nil (Japanese morning glory) (Pharbitis nil)highly similar to ( 601) loc_os09g02250 12009.m059931 protein transposon protein, putative, unclassified, expressed   no original description 	T
22.1.6	polyamine metabolism.synthesis.spermidine synthase	MDP0000027925	 highly similar to ( 522) AT1G23820 | Symbols: SPDS1 | SPDS1 (spermidine synthase 1); spermidine synthase | chr1:8420278-8422724 FORWARDhighly similar to ( 520) SPD2_PEA Spermidine synthase 2 (EC 2.5.1.16) (Putrescine aminopropyltransferase 2) (SPDSY 2) - Pisum sativum (Garden pea)highly similar to ( 509) loc_os07g22600 12007.m29134 protein spermidine synthase 1, putative, expressed no original description 	T
35.2	not assigned.unknown	MDP0000212784	 highly similar to ( 892) AT4G34260 | Symbols: FUC95A | FUC95A; 1,2-alpha-L-fucosidase | chr4:16398130-16401591 FORWARDhighly similar to ( 851) loc_os10g19880 12010.m05016 protein large secreted protein, putative, expressed  no original description 	T
35.2	not assigned.unknown	MDP0000134234	 moderately similar to ( 486) AT2G15860 | Symbols:  | unknown protein | chr2:6905712-6909282 REVERSEmoderately similar to ( 414) loc_os03g52780 12003.m10252 protein expressed protein   no original description 	T
29.2.1.2.2.39	protein.synthesis.ribosomal protein.eukaryotic.60S subunit.L39	MDP0000337690	 weakly similar to ( 110) AT4G31985 | Symbols:  | 60S ribosomal protein L39 (RPL39C) | chr4:15469931-15470366 FORWARDweakly similar to ( 107) RL39_ORYSA 60S ribosomal protein L39 - Oryza sativa (Rice)weakly similar to ( 107) loc_os06g08320 12006.m05553 protein 60S ribosomal protein L39, putative, expressed no original description 	T
35.2	not assigned.unknown	MDP0000289041	 moderately similar to ( 353) AT5G11950 | Symbols:  | protein homodimerization | chr5:3855072-3856815 FORWARDmoderately similar to ( 306) loc_os01g51210 12001.m11302 protein lysine decarboxylase-like protein, putative, expressed   no original description 	T
1.1.6	PS.lightreaction.NADH DH	MDP0000557856	 moderately similar to ( 253) AT4G37925 | Symbols: NDH-M | NDH-M (subunit NDH-M of NAD(P)H:plastoquinone dehydrogenase complex) | chr4:17830748-17831485 REVERSEmoderately similar to ( 225) loc_os04g45600 12004.m09513 protein expressed protein   no original description 	T
21.1	redox.thioredoxin	MDP0000165749	 moderately similar to ( 323) AT1G34780 | Symbols: ATAPRL4 | ATAPRL4 (APR-like 4) | chr1:12748835-12750124 REVERSEmoderately similar to ( 290) loc_os02g51850 12002.m33878 protein OsAPRL3 - Oryza sativa adenosine 5'-phosphosulfate reductase-like, expressed no original description 	T
8.2.10	TCA / org transformation.other organic acid transformatons.malic	MDP0000268037	 highly similar to ( 832) AT2G19900 | Symbols: ATNADP-ME1 | ATNADP-ME1 (NADP-malic enzyme 1); malate dehydrogenase (oxaloacetate-decarboxylating) (NADP+)/ malic enzyme/ oxidoreductase, acting on NADH or NADPH, NAD or NADP as acceptor | chr2:8592106-8595403 REVERSEhighly similar to ( 865) MAOX_VITVI NADP-dependent malic enzyme (EC 1.1.1.40) (NADP-ME) - Vitis vinifera (Grape)highly similar to ( 848) loc_os01g52500 12001.m42715 protein NADP-dependent malic enzyme, putative, expressed    COG4907  no original description 	T
31.1	cell.organisation	MDP0000282836	 moderately similar to ( 338) AT5G23910 | Symbols:  | ATP binding / microtubule motor | chr5:8068452-8072723 FORWARDweakly similar to ( 102) K125_TOBAC 125 kDa kinesin-related protein - Nicotiana tabacum (Common tobacco)moderately similar to ( 216) loc_os04g30720 12004.m35280 protein kinesin motor domain containing protein, expressed  no original description 	T
35.2	not assigned.unknown	MDP0000196904	 very weakly similar to (88.6) AT4G34560 | Symbols:  | unknown protein | chr4:16507923-16508588 FORWARD no original description 	T
29.3.3	protein.targeting.chloroplast	MDP0000210114	 highly similar to ( 557) AT1G75420 | Symbols:  | glycosyl transferase family 1 protein | chr1:28305469-28307317 FORWARDhighly similar to ( 518) loc_os01g15780 12001.m42953 protein transferase, transferring glycosyl groups, putative, expressed   no original description 	T
29.2.1.1.1.2.21	protein.synthesis.ribosomal protein.prokaryotic.chloroplast.50S subunit.L21	MDP0000221752	 weakly similar to ( 185) AT4G30930 | Symbols: NFD1 | NFD1 (NUCLEAR FUSION DEFECTIVE 1); RNA binding / structural constituent of ribosome | chr4:15050170-15051630 REVERSEvery weakly similar to (85.1) RK21_SPIOL 50S ribosomal protein L21, chloroplast precursor (CL21) (CS-L7) - Spinacia oleracea (Spinach)weakly similar to ( 179) loc_os05g48410 12005.m08912 protein 50S ribosomal protein L21, mitochondrial precursor, putative, expressed TrbL MSP1_C  no original description 	T
31.1	cell.organisation	MDP0000171197	 highly similar to ( 577) AT3G01750 | Symbols:  | ankyrin repeat family protein | chr3:270615-272691 FORWARDhighly similar to ( 636) loc_os05g23320 12005.m06627 protein expressed protein   no original description 	T
31.4	cell.vesicle transport	MDP0000610843	 moderately similar to ( 283) AT4G21450 | Symbols:  | vesicle-associated membrane family protein / VAMP family protein | chr4:11426136-11428125 FORWARDmoderately similar to ( 270) loc_os07g37270 12007.m07977 protein major sperm protein, putative, expressed no original description 	T
35.1	not assigned.no ontology	MDP0000306321	 moderately similar to ( 440) AT1G74300 | Symbols:  | esterase/lipase/thioesterase family protein | chr1:27935368-27936474 FORWARDmoderately similar to ( 392) loc_os02g32970 12002.m08390 protein catalytic/ hydrolase, putative, expressed  no original description 	T
11.1.9	lipid metabolism.FA synthesis and FA elongation.long chain fatty acid CoA ligase	MDP0000314713	 highly similar to ( 855) AT1G49430 | Symbols: LRD2, LACS2 | LACS2 (LONG-CHAIN ACYL-COA SYNTHETASE 2); long-chain-fatty-acid-CoA ligase | chr1:18291188-18295641 FORWARDvery weakly similar to (74.3) 4CL1_ORYSA 4-coumarate--CoA ligase 1 (EC 6.2.1.12) (4CL 1) (4-coumaroyl-CoA synthase 1) - Oryza sativa (Rice)highly similar to ( 862) loc_os11g35400 12011.m080023 protein acyl CoA synthetase, putative, expressed     Sporozoite_P67  no original description 	T
11.3	lipid metabolism.Phospholipid synthesis	MDP0000479163	 highly similar to ( 588) AT2G38110 | Symbols: ATGPAT6, GPAT6 | GPAT6 (GLYCEROL-3-PHOSPHATE ACYLTRANSFERASE 6); 1-acylglycerol-3-phosphate O-acyltransferase/ acyltransferase | chr2:15952816-15955364 REVERSEhighly similar to ( 542) loc_os01g63580 12001.m12474 protein glycerol-3-phosphate acyltransferase 8, putative, expressed  no original description 	T
26.4	misc.beta 1,3 glucan hydrolases	MDP0000289320	 weakly similar to ( 134) AT1G18650 | Symbols: PDCB3 | PDCB3 (PLASMODESMATA CALLOSE-BINDING PROTEIN 3); callose binding / polysaccharide binding | chr1:6419036-6420413 REVERSEvery weakly similar to (87.8) E13B_WHEAT Glucan endo-1,3-beta-glucosidase precursor (EC 3.2.1.39) ((1->3)-beta-glucan endohydrolase) ((1->3)-beta-glucanase) (Beta-1,3-endoglucanase) - Triticum aestivum (Wheat)weakly similar to ( 112) loc_os07g40940 12007.m08334 protein glucan endo-1,3-beta-glucosidase 3 precursor, putative, expressed  no original description 	T
34.1	transport.p- and v-ATPases	MDP0000231476	 highly similar to ( 545) AT1G12840 | Symbols: DET3, ATVHA-C | DET3 (DE-ETIOLATED 3); proton-transporting ATPase, rotational mechanism | chr1:4375584-4378220 FORWARDmoderately similar to ( 374) VATC_HORVU Vacuolar ATP synthase subunit C (EC 3.6.3.14) (V-ATPase C subunit) (Vacuolar proton pump C subunit) - Hordeum vulgare (Barley)highly similar to ( 515) loc_os05g51530 12005.m09215 protein vacuolar ATP synthase subunit C, putative, expressed   no original description 	T
29.5.11.4.2	protein.degradation.ubiquitin.E3.RING	MDP0000257529	 very weakly similar to ( 100) AT4G24204 | Symbols:  | protein binding / zinc ion binding | chr4:12562069-12562815 REVERSEvery weakly similar to (92.4) loc_os06g14200 12006.m06135 protein zinc finger, RING-type, putative no original description 	T
31.1	cell.organisation	MDP0000197971	 nearly identical (1665) AT5G65460 | Symbols:  | kinesin motor protein-related | chr5:26161831-26169001 REVERSEweakly similar to ( 151) FLA10_CHLRE Kinesin-like protein FLA10 (Protein KHP1) - Chlamydomonas reinhardtiihighly similar to ( 743) loc_os06g11380 12006.m05855 protein ATP binding protein, putative, expressed  no original description 	T
30.2.11	signalling.receptor kinases.leucine rich repeat XI	MDP0000432656	 moderately similar to ( 448) AT3G47570 | Symbols:  | leucine-rich repeat transmembrane protein kinase, putative | chr3:17527611-17530748 FORWARDmoderately similar to ( 279) PSKR_DAUCA Phytosulfokine receptor precursor (EC 2.7.11.1) (Phytosulfokine LRR receptor kinase) - Daucus carota (Carrot)highly similar to ( 505) loc_os11g47180 12011.m08498 protein receptor-like protein kinase precursor, putative, expressed   no original description 	T
27.3.5	RNA.regulation of transcription.ARR	MDP0000250737	 weakly similar to ( 181) AT1G59940 | Symbols: ARR3 | ARR3 (RESPONSE REGULATOR 3); transcription regulator/ two-component response regulator | chr1:22065894-22066895 REVERSEvery weakly similar to (67.8) PRR37_ORYSA Two-component response regulator-like PRR37 (Pseudo-response regulator 37) (OsPRR37) - Oryza sativa (Rice)weakly similar to ( 135) loc_os01g72330 12001.m13268 protein OsRR4 - Rice type-A response regulator, expressed   no original description 	T
35.2	not assigned.unknown	MDP0000545009	 weakly similar to ( 184) AT5G49210 | Symbols:  | unknown protein | chr5:19950093-19951464 FORWARDweakly similar to ( 136) loc_os11g05120 12011.m04710 protein expressed protein  CobN  no original description 	T
35.2	not assigned.unknown	MDP0000155726	 nearly identical (1125) AT3G24880 | Symbols:  | DNA binding | chr3:9086457-9095537 REVERSEmoderately similar to ( 310) loc_os08g08000 12008.m04936 protein DNA binding protein, putative, expressed   no original description 	T
31.4	cell.vesicle transport	MDP0000313014	 highly similar to ( 726) AT5G02100 | Symbols: UNE18, ORP3A | UNE18 (UNFERTILIZED EMBRYO SAC 18); oxysterol binding / sterol binding | chr5:413639-416016 FORWARDhighly similar to ( 705) loc_os03g16690 12003.m07102 protein oxysterol-binding protein homolog C2F12.05c, putative, expressed  no original description 	T
34.99	transport.misc	MDP0000152780	 nearly identical (1075) AT3G15380 | Symbols:  | choline transporter-related | chr3:5193319-5196435 FORWARDnearly identical (1026) loc_os04g58470 12004.m101697 protein expressed protein no original description 	T
35.1	not assigned.no ontology	MDP0000779091	 moderately similar to ( 246) AT3G12030 | Symbols:  | unknown protein | chr3:3834465-3835055 REVERSEweakly similar to ( 198) loc_os03g51160 12003.m101475 protein fb27, putative, expressed  no original description 	T
29.5.11.1	protein.degradation.ubiquitin.ubiquitin	MDP0000501162	 moderately similar to ( 253) AT2G47110 | Symbols: UBQ6 | UBQ6; protein binding | chr2:19344701-19345174 FORWARDweakly similar to ( 150) UBIQ_WHEAT Ubiquitin - Triticum aestivum (Wheat)moderately similar to ( 246) loc_os01g22490 12001.m08729 protein 40S ribosomal protein S27a, putative, expressed  no original description 	T
29.2.2.3.1	protein.synthesis.ribosome biogenesis.Pre-rRNA processing and modifications.snoRNPs	MDP0000255994	 weakly similar to ( 184) AT3G03920 | Symbols:  | Gar1 RNA-binding region family protein | chr3:1009123-1010379 REVERSEweakly similar to ( 189) loc_os11g37080 12011.m07556 protein h/ACA ribonucleoprotein complex subunit 1-like protein 1, putative, expressed no original description 	T
29.7	protein.glycosylation	MDP0000139743	 highly similar to ( 600) AT2G01720 | Symbols:  | ribophorin I family protein | chr2:317193-320016 REVERSEmoderately similar to ( 464) loc_os05g23600 12005.m27570 protein dolichyl-diphosphooligosaccharide--protein glycosyltransferase 67 kDasubunit precursor, putative, expressed  no original description 	T
34.20	transport.porins	MDP0000580900	 moderately similar to ( 402) AT5G67500 | Symbols: VDAC2, ATVDAC2 | VDAC2 (VOLTAGE DEPENDENT ANION CHANNEL 2); voltage-gated anion channel | chr5:26935223-26937123 FORWARDmoderately similar to ( 295) VDAC1_SOLTU 34 kDa outer mitochondrial membrane protein porin (Voltage-dependent anion-selective channel protein) (VDAC) (POM 34) - Solanum tuberosum (Potato)moderately similar to ( 345) loc_os03g10510 12003.m35080 protein outer mitochondrial membrane protein porin, putative, expressed  no original description 	T
34.2	transport.sugars	MDP0000580900	 moderately similar to ( 402) AT5G67500 | Symbols: VDAC2, ATVDAC2 | VDAC2 (VOLTAGE DEPENDENT ANION CHANNEL 2); voltage-gated anion channel | chr5:26935223-26937123 FORWARDmoderately similar to ( 295) VDAC1_SOLTU 34 kDa outer mitochondrial membrane protein porin (Voltage-dependent anion-selective channel protein) (VDAC) (POM 34) - Solanum tuberosum (Potato)moderately similar to ( 345) loc_os03g10510 12003.m35080 protein outer mitochondrial membrane protein porin, putative, expressed  no original description 	T
26.2	misc.UDP glucosyl and glucoronyl transferases	MDP0000127117	 weakly similar to ( 152) AT4G38040 | Symbols:  | exostosin family protein | chr4:17867501-17869131 FORWARDweakly similar to ( 163) loc_os02g09430 12002.m06240 protein exostosin-like, putative, expressed   no original description 	T
35.1	not assigned.no ontology	MDP0000753849	 highly similar to ( 576) AT2G01070 | Symbols:  | FUNCTIONS IN: molecular_function unknown; INVOLVED IN: biological_process unknown; LOCATED IN: endomembrane system, integral to membrane; EXPRESSED IN: 22 plant structures; EXPRESSED DURING: 13 growth stages; CONTAINS InterPro DOMAIN/s: Transmembrane receptor, eukaryota (InterPro:IPR009637); BEST Arabidopsis thaliana protein match is: unknown protein (TAIR:AT1G72480.1); Has 487 Blast hits to 486 proteins in 125 species: Archae - 0; Bacteria - 0; Metazoa - 264; Fungi - 101; Plants - 89; Viruses - 0; Other Eukaryotes - 33 (source: NCBI BLink). | chr2:75596-77300 FORWARDhighly similar to ( 601) loc_os11g34360 12011.m07294 protein TMEM87A protein, putative, expressed  no original description 	T
26.24	misc.GCN5-related N-acetyltransferase	MDP0000378275	 moderately similar to ( 257) AT1G26220 | Symbols:  | GCN5-related N-acetyltransferase (GNAT) family protein | chr1:9071157-9071750 FORWARDweakly similar to ( 178) loc_os08g01170 12008.m04266 protein expressed protein    no original description 	T
35.2	not assigned.unknown	MDP0000402527	 weakly similar to ( 144) AT5G60680 | Symbols:  | unknown protein | chr5:24386247-24386738 FORWARDvery weakly similar to (94.4) loc_os04g43990 12004.m09358 protein expressed protein   no original description 	T
16.1.1.10	secondary metabolism.isoprenoids.non-mevalonate pathway.geranylgeranyl pyrophosphate synthase	MDP0000668869	 moderately similar to ( 428) AT4G36810 | Symbols: GGPS1 | GGPS1 (GERANYLGERANYL PYROPHOSPHATE SYNTHASE 1); farnesyltranstransferase | chr4:17343513-17344628 FORWARDmoderately similar to ( 471) GGPPS_HEVBR Geranylgeranyl pyrophosphate synthetase, chloroplast precursor (GGPP synthetase) [Includes: Dimethylallyltranstransferase (EC 2.5.1.1); Geranyltranstransferase (EC 2.5.1.10); Farnesyltranstransferase (EC 2.5.1.29)] - Hevea brasiliensismoderately similar to ( 355) loc_os07g39270 12007.m28997 protein geranylgeranyl pyrophosphate synthetase 1, chloroplast precursor, putative, expressed   no original description 	T
35.2	not assigned.unknown	MDP0000321410	 moderately similar to ( 258) AT1G65230 | Symbols:  | unknown protein | chr1:24229167-24230764 FORWARDmoderately similar to ( 254) loc_os01g68450 12001.m12948 protein expressed protein  no original description 	T
34.9	transport.metabolite transporters at the mitochondrial membrane	MDP0000295963	 moderately similar to ( 426) AT3G51870 | Symbols:  | binding / transporter | chr3:19243978-19246611 FORWARDweakly similar to ( 101) BT1_MAIZE Protein brittle-1, chloroplast precursor - Zea mays (Maize)moderately similar to ( 366) loc_os01g16040 12001.m08201 protein protein brittle-1, chloroplast precursor, putative, expressed no original description 	T
18	Co-factor and vitamine metabolism	MDP0000275383	 highly similar to ( 508) AT5G01410 | Symbols: PDX1, ATPDX1.3, RSR4, PDX1.3, ATPDX1 | RSR4 (REDUCED SUGAR RESPONSE 4); protein heterodimerization/ protein homodimerization | chr5:172576-173505 REVERSEhighly similar to ( 508) PDX1_HEVBR Probable pyridoxin biosynthesis protein ER1 (PDX1 homolog) (Ethylene-inducible protein HEVER) - Hevea brasiliensis (Para rubber tree)moderately similar to ( 466) loc_os07g01020 12007.m04583 protein pyridoxin biosynthesis protein ER1, putative, expressed    no original description 	T
27.1	RNA.processing	MDP0000329254	 weakly similar to ( 145) AT2G23930 | Symbols: SNRNP-G | SNRNP-G (PROBABLE SMALL NUCLEAR RIBONUCLEOPROTEIN G) | chr2:10182496-10183026 FORWARDweakly similar to ( 139) RUXG_MEDSA Probable small nuclear ribonucleoprotein G (snRNP-G) (Sm protein G) (Sm-G) (SmG) - Medicago sativa (Alfalfa)weakly similar to ( 148) loc_os07g41790 12007.m079746 protein small nuclear ribonucleoprotein G, putative, expressed no original description 	T
35.2	not assigned.unknown	MDP0000774498	 weakly similar to ( 200) AT2G32910 | Symbols:  | FUNCTIONS IN: molecular_function unknown; INVOLVED IN: biological_process unknown; LOCATED IN: cellular_component unknown; EXPRESSED IN: 25 plant structures; EXPRESSED DURING: 15 growth stages; CONTAINS InterPro DOMAIN/s: Development and cell death domain (InterPro:IPR013989), Kelch related (InterPro:IPR013089); BEST Arabidopsis thaliana protein match is: unknown protein (TAIR:AT5G61910.3); Has 954 Blast hits to 804 proteins in 137 species: Archae - 0; Bacteria - 69; Metazoa - 446; Fungi - 64; Plants - 191; Viruses - 0; Other Eukaryotes - 184 (source: NCBI BLink). | chr2:13959685-13961989 FORWARDvery weakly similar to (60.5) B2_DAUCA B2 protein - Daucus carota (Carrot)weakly similar to ( 145) loc_os06g12260 12006.m091652 protein expressed protein PRK07003  no original description 	T
21.1	redox.thioredoxin	MDP0000686419	 weakly similar to ( 162) AT4G03520 | Symbols: ATHM2 | ATHM2; enzyme activator | chr4:1562585-1562803 REVERSEmoderately similar to ( 205) TRXM_PEA Thioredoxin M-type, chloroplast precursor (TRX-M) - Pisum sativum (Garden pea)weakly similar to ( 183) loc_os12g08730 12012.m04859 protein thioredoxin M-type, chloroplast precursor, putative, expressed no original description 	T
10.8.1	cell wall.pectin*esterases.PME	MDP0000206503	 moderately similar to ( 249) AT1G05310 | Symbols:  | pectinesterase family protein | chr1:1550615-1552434 REVERSEvery weakly similar to (95.1) PME3_CITSI Pectinesterase-3 precursor (EC 3.1.1.11) (Pectin methylesterase 3) (PE 3) - Citrus sinensis (Sweet orange)moderately similar to ( 244) loc_os08g10604 12008.m05193 protein pectinesterase-1 precursor, putative, expressed no original description 	T
35.2	not assigned.unknown	MDP0000192559	 moderately similar to ( 353) AT5G36290 | Symbols:  | unknown protein | chr5:14302977-14304972 FORWARDmoderately similar to ( 338) loc_os11g28300 12011.m28801 protein transmembrane protein FT27/PFT27-like, putative, expressed   no original description 	T
35.2	not assigned.unknown	MDP0000202208	  no original description 	T
29.5.4	protein.degradation.aspartate protease	MDP0000333631	 weakly similar to ( 105) AT4G35880 | Symbols:  | aspartyl protease family protein | chr4:16993339-16995721 FORWARDvery weakly similar to (84.0) loc_os04g15790 12004.m101470 protein aspartic-type endopeptidase/ pepsin A, putative, expressed  no original description 	T
33.99	development.unspecified	MDP0000264101	 highly similar to ( 522) AT4G05410 | Symbols:  | transducin family protein / WD-40 repeat family protein | chr4:2743229-2745521 REVERSEvery weakly similar to (88.2) GBLP_MEDSA Guanine nucleotide-binding protein subunit beta-like protein - Medicago sativa (Alfalfa)moderately similar to ( 493) loc_os03g42770 12003.m09329 protein expressed protein   PRK08026  no original description 	T
30.5	signalling.G-proteins	MDP0000264101	 highly similar to ( 522) AT4G05410 | Symbols:  | transducin family protein / WD-40 repeat family protein | chr4:2743229-2745521 REVERSEvery weakly similar to (88.2) GBLP_MEDSA Guanine nucleotide-binding protein subunit beta-like protein - Medicago sativa (Alfalfa)moderately similar to ( 493) loc_os03g42770 12003.m09329 protein expressed protein   PRK08026  no original description 	T
27.3.99	RNA.regulation of transcription.unclassified	MDP0000410971	 moderately similar to ( 358) AT4G22140 | Symbols:  | DNA binding / protein binding / zinc ion binding | chr4:11728093-11730230 REVERSEmoderately similar to ( 339) loc_os09g21770 12009.m05384 protein DNA binding protein, putative, expressed no original description 	T
27.3.44	RNA.regulation of transcription.Chromatin Remodeling Factors	MDP0000410971	 moderately similar to ( 358) AT4G22140 | Symbols:  | DNA binding / protein binding / zinc ion binding | chr4:11728093-11730230 REVERSEmoderately similar to ( 339) loc_os09g21770 12009.m05384 protein DNA binding protein, putative, expressed no original description 	T
29.4	protein.postranslational modification	MDP0000139194	 highly similar to ( 629) AT3G63280 | Symbols: ATNEK4 | ATNEK4 (NIMA-RELATED KINASE 4); ATP binding / kinase/ protein kinase/ protein serine/threonine kinase | chr3:23378582-23381362 FORWARDweakly similar to ( 107) M2K1_ORYSA Mitogen-activated protein kinase kinase 1 (EC 2.7.12.2) (MAP kinase kinase 1) (MAPKK1) (OsMEK1) - Oryza sativa (Rice)highly similar to ( 669) loc_os12g41180 12012.m07900 protein LSTK-1-like kinase, putative, expressed  no original description 	T
29.6	protein.folding	MDP0000145669	 weakly similar to ( 136) AT1G23100 | Symbols:  | 10 kDa chaperonin, putative | chr1:8195867-8196336 FORWARDweakly similar to ( 125) CH10_BRANA 10 kDa chaperonin (Protein CPN10) (Protein groES) - Brassica napus (Rape)weakly similar to ( 123) loc_os07g44740 12007.m08695 protein chaperonin, putative, expressed  no original description 	T
6.5	gluconeogenesis / glyoxylate cycle.pyruvate dikinase	MDP0000130296	 nearly identical (1490) AT4G15530 | Symbols: PPDK | PPDK (pyruvate orthophosphate dikinase); kinase/ pyruvate, phosphate dikinase | chr4:8864828-8870748 REVERSEnearly identical (1546) PPDK_MESCR Pyruvate, phosphate dikinase, chloroplast precursor (EC 2.7.9.1) (Pyruvate, orthophosphate dikinase) - Mesembryanthemum crystallinum (Common ice plant)nearly identical (1449) loc_os05g33570 12005.m083718 protein pyruvate, phosphate dikinase, chloroplast precursor, putative, expressed   no original description 	T
35.2	not assigned.unknown	MDP0000342608	  no original description 	T
27.3.5	RNA.regulation of transcription.ARR	MDP0000207048	 moderately similar to ( 215) AT5G62920 | Symbols: ARR6 | ARR6 (RESPONSE REGULATOR 6); transcription regulator/ two-component response regulator | chr5:25252745-25254158 REVERSEvery weakly similar to (72.0) PRR37_ORYSA Two-component response regulator-like PRR37 (Pseudo-response regulator 37) (OsPRR37) - Oryza sativa (Rice)weakly similar to ( 160) loc_os11g04720 12011.m04670 protein OsRR9 - Rice type-A response regulator, expressed no original description 	T
30.8	signalling.misc	MDP0000613004	 very weakly similar to ( 100) AT3G23805 | Symbols: RALFL24 | RALFL24 (ralf-like 24); signal transducer | chr3:8586467-8586823 FORWARDvery weakly similar to (73.2) loc_os12g35670 12012.m07360 protein RALF precursor, putative, expressed   no original description 	T
20.2.1	stress.abiotic.heat	MDP0000299975	 highly similar to ( 591) AT3G44110 | Symbols: ATJ3, ATJ | ATJ3; protein binding | chr3:15869179-15871059 REVERSEhighly similar to ( 590) DNJH_CUCSA DnaJ protein homolog (DNAJ-1) - Cucumis sativus (Cucumber)highly similar to ( 582) loc_os03g57340 12003.m10652 protein dnaJ protein, putative, expressed  no original description 	T
25	C1-metabolism	MDP0000291807	 highly similar to ( 604) AT5G14780 | Symbols: FDH | FDH (FORMATE DEHYDROGENASE); NAD or NADH binding / binding / catalytic/ cofactor binding / oxidoreductase, acting on the CH-OH group of donors, NAD or NADP as acceptor | chr5:4777043-4779190 FORWARDhighly similar to ( 633) FDH_SOLTU Formate dehydrogenase, mitochondrial precursor (EC 1.2.1.2) (NAD-dependent formate dehydrogenase) (FDH) - Solanum tuberosum (Potato)highly similar to ( 621) loc_os06g29180 12006.m07462 protein formate dehydrogenase 1, mitochondrial precursor, putative, expressed   no original description 	T
8.1.5	TCA / org transformation.TCA.2-oxoglutarate dehydrogenase	MDP0000089058	 nearly identical (1829) AT5G65750 | Symbols:  | 2-oxoglutarate dehydrogenase E1 component, putative / oxoglutarate decarboxylase, putative / alpha-ketoglutaric dehydrogenase, putative | chr5:26304212-26307947 FORWARDnearly identical (1732) loc_os07g49520 12007.m09160 protein 2-oxoglutarate dehydrogenase E1 component, mitochondrial precursor, putative, expressed  no original description 	T
35.2	not assigned.unknown	MDP0000143641	  no original description 	T
27.3.40	RNA.regulation of transcription.Aux/IAA family	MDP0000456476	 moderately similar to ( 297) AT5G65670 | Symbols: IAA9 | IAA9 (INDOLE-3-ACETIC ACID INDUCIBLE 9); transcription factor | chr5:26254463-26256134 FORWARDweakly similar to ( 173) IAA19_ORYSA Auxin-responsive protein IAA19 (Indoleacetic acid-induced protein 19) - Oryza sativa (Rice)weakly similar to ( 173) loc_os05g48590 12005.m08930 protein OsIAA19 - Auxin-responsive Aux/IAA gene family member, expressed   no original description 	T
29.5	protein.degradation	MDP0000332786	 moderately similar to ( 401) AT5G04200 | Symbols: AtMC9 | AtMC9 (metacaspase 9); cysteine-type peptidase | chr5:1153893-1154870 FORWARDmoderately similar to ( 332) loc_os11g04010 12011.m04596 protein caspase, putative, expressed no original description 	T
35.2	not assigned.unknown	MDP0000750789	  no original description 	T
29.2.1.2.2.6	protein.synthesis.ribosomal protein.eukaryotic.60S subunit.L6	MDP0000162548	 moderately similar to ( 302) AT1G18540 | Symbols:  | 60S ribosomal protein L6 (RPL6A) | chr1:6377448-6378548 REVERSEmoderately similar to ( 306) RL6_MESCR 60S ribosomal protein L6 (YL16-like) - Mesembryanthemum crystallinum (Common ice plant)moderately similar to ( 233) loc_os04g39700 12004.m08956 protein 60S ribosomal protein L6, putative, expressed  MSP1_C  no original description 	T
35.1	not assigned.no ontology	MDP0000207668	 highly similar to ( 753) AT3G07140 | Symbols:  | GPI transamidase component Gpi16 subunit family protein | chr3:2261278-2263632 FORWARDhighly similar to ( 703) loc_os11g28980 12011.m06813 protein GPI transamidase component PIG-T precursor, putative, expressed  no original description 	T
26.4.1	misc.beta 1,3 glucan hydrolases.glucan endo-1,3-beta-glucosidase	MDP0000295938	 highly similar to ( 605) AT4G31140 | Symbols:  | glycosyl hydrolase family 17 protein | chr4:15141581-15143188 FORWARDmoderately similar to ( 202) E13H_TOBAC Glucan endo-1,3-beta-glucosidase, acidic isoform PR-Q' precursor (EC 3.2.1.39) ((1->3)-beta-glucan endohydrolase) ((1->3)-beta-glucanase) (Beta-1,3-endoglucanase) (PR-35) - Nicotiana tabacum (Common tobacco)highly similar to ( 574) loc_os02g04670 12002.m05815 protein glucan endo-1,3-beta-glucosidase 6 precursor, putative, expressed no original description 	T
35.1	not assigned.no ontology	MDP0000334945	 weakly similar to ( 159) AT1G29395 | Symbols: COR414-TM1 | COR414-TM1 | chr1:10288345-10289539 REVERSEweakly similar to ( 140) loc_os05g49170 12005.m08987 protein cold acclimation WCOR413-like protein gamma form, putative, expressed  no original description 	T
29.6	protein.folding	MDP0000330296	 highly similar to ( 938) AT3G02530 | Symbols:  | chaperonin, putative | chr3:528806-532457 REVERSEmoderately similar to ( 213) TCPE2_AVESA T-complex protein 1 subunit epsilon (TCP-1-epsilon) (CCT-epsilon) (TCP-K36) - Avena sativa (Oat)highly similar to ( 934) loc_os05g05470 12005.m05074 protein T-complex protein 1 subunit zeta, putative, expressed  no original description 	T
29.5.11.3	protein.degradation.ubiquitin.E2	MDP0000319546	 moderately similar to ( 342) AT3G15355 | Symbols: UBC25, PFU1 | UBC25 (UBIQUITIN-CONJUGATING ENZYME 25); small conjugating protein ligase | chr3:5183858-5186669 FORWARDvery weakly similar to (63.5) UBC2_WHEAT Ubiquitin-conjugating enzyme E2-17 kDa (EC 6.3.2.19) (Ubiquitin-protein ligase) (Ubiquitin carrier protein) - Triticum aestivum (Wheat)moderately similar to ( 342) loc_os05g48380 12005.m08909 protein ubiquitin-conjugating enzyme E2, putative, expressed   no original description 	T
35.1	not assigned.no ontology	MDP0000126789	 moderately similar to ( 238) AT1G07230 | Symbols:  | hydrolase, acting on ester bonds | chr1:2220509-2222778 REVERSEmoderately similar to ( 256) loc_os03g61130 12003.m10997 protein hydrolase, acting on ester bonds, putative, expressed no original description 	T
29.4	protein.postranslational modification	MDP0000262599	 moderately similar to ( 351) AT2G23070 | Symbols:  | casein kinase II alpha chain, putative | chr2:9824162-9826871 REVERSEmoderately similar to ( 332) CSK2A_MAIZE Casein kinase II subunit alpha (EC 2.7.11.1) (CK II) (CK2-alpha) - Zea mays (Maize)moderately similar to ( 336) loc_os07g02350 12007.m04707 protein casein kinase II subunit alpha-2, putative, expressed  no original description 	T
33.99	development.unspecified	MDP0000270116	 nearly identical (1645) AT5G27030 | Symbols: TPR3 | TPR3 (TOPLESS-RELATED 3) | chr5:9508913-9515263 REVERSEvery weakly similar to (50.8) GBLP_BRANA Guanine nucleotide-binding protein subunit beta-like protein - Brassica napus (Rape)nearly identical (1580) loc_os01g15020 12001.m42634 protein lissencephaly type-1-like homology motif, putative, expressed   no original description 	T
9.1.1.5	mitochondrial electron transport / ATP synthesis.NADH-DH.complex I.carbonic anhydrase	MDP0000252362	 highly similar to ( 513) AT1G19600 | Symbols:  | pfkB-type carbohydrate kinase family protein | chr1:6779085-6780898 FORWARDvery weakly similar to (78.6) ADK_PHYPA Adenosine kinase (EC 2.7.1.20) (AK) (Adenosine 5'-phosphotransferase) - Physcomitrella patens (Moss)highly similar to ( 504) loc_os01g01620 12001.m06807 protein ribokinase, putative, expressed  PRK08026  no original description 	T
13.1.6.1.1	amino acid metabolism.synthesis.aromatic aa.chorismate.3-deoxy-D-arabino-heptulosonate 7-phosphate synthase	MDP0000259511	 highly similar to ( 848) AT4G33510 | Symbols: DHS2 | DHS2 (3-deoxy-d-arabino-heptulosonate 7-phosphate synthase); 3-deoxy-7-phosphoheptulonate synthase | chr4:16116496-16118233 FORWARDhighly similar to ( 830) AROF_TOBAC Phospho-2-dehydro-3-deoxyheptonate aldolase 1, chloroplast precursor (EC 2.5.1.54) (Phospho-2-keto-3-deoxyheptonate aldolase 1) (DAHP synthetase 1) (3-deoxy-D-arabino-heptulosonate 7-phosphate synthase 1) - Nicotiana tabacum (Common tobhighly similar to ( 816) loc_os07g42960 12007.m29007 protein phospho-2-dehydro-3-deoxyheptonate aldolase 1, chloroplast precursor, putative, expressed  no original description 	T
29.5.11.4.3.2	protein.degradation.ubiquitin.E3.SCF.FBOX	MDP0000786502	 highly similar to ( 563) AT3G61590 | Symbols: HWS, HS | HWS (HAWAIIAN SKIRT); ubiquitin-protein ligase | chr3:22792914-22794149 FORWARDmoderately similar to ( 497) loc_os01g47050 12001.m10901 protein kelch motif family protein, expressed  no original description 	T
34.22	transport.cyclic nucleotide or calcium regulated channels	MDP0000281334	 highly similar to ( 682) AT3G17700 | Symbols: CNBT1, CNGC20, ATCNGC20 | CNBT1 (CYCLIC NUCLEOTIDE-BINDING TRANSPORTER 1); calmodulin binding / cyclic nucleotide binding / ion channel | chr3:6049074-6052449 FORWARDhighly similar to ( 630) loc_os02g53340 12002.m100377 protein cyclic nucleotide-gated ion channel 19, putative, expressed  no original description 	T
29.5.11.1	protein.degradation.ubiquitin.ubiquitin	MDP0000263713	 moderately similar to ( 255) AT2G47110 | Symbols: UBQ6 | UBQ6; protein binding | chr2:19344701-19345174 FORWARDweakly similar to ( 150) UBIQ_WHEAT Ubiquitin - Triticum aestivum (Wheat)moderately similar to ( 246) loc_os01g22490 12001.m08729 protein 40S ribosomal protein S27a, putative, expressed  no original description 	T
29.5.11.4.2	protein.degradation.ubiquitin.E3.RING	MDP0000137556	 weakly similar to ( 187) AT1G20823 | Symbols:  | zinc finger (C3HC4-type RING finger) family protein | chr1:7238880-7239473 FORWARDvery weakly similar to (83.6) EL5_ORYSA E3 ubiquitin-protein ligase EL5 (EC 6.3.2.-) - Oryza sativa (Rice)weakly similar to ( 135) loc_os02g46100 12002.m09647 protein RING-H2 finger protein ATL1R, putative, expressed  no original description 	T
1.2.2	PS.photorespiration.glycolate oxydase	MDP0000282534	 moderately similar to ( 458) AT3G14130 | Symbols:  | (S)-2-hydroxy-acid oxidase, peroxisomal, putative / glycolate oxidase, putative / short chain alpha-hydroxy acid oxidase, putative | chr3:4685844-4687852 REVERSEmoderately similar to ( 369) GOX_SPIOL Peroxisomal (S)-2-hydroxy-acid oxidase (EC 1.1.3.15) (Glycolate oxidase) (GOX) (Short chain alpha-hydroxy acid oxidase) - Spinacia oleracea (Spinach)moderately similar to ( 390) loc_os07g42440 12007.m079750 protein hydroxyacid oxidase 1, putative, expressed  no original description 	T
29.5.11.4.2	protein.degradation.ubiquitin.E3.RING	MDP0000396222	 weakly similar to ( 154) AT4G03510 | Symbols: RMA1 | RMA1; protein binding / ubiquitin-protein ligase/ zinc ion binding | chr4:1557905-1558654 REVERSEweakly similar to ( 113) loc_os12g43930 12012.m08164 protein RING finger protein 5, putative, expressed  no original description 	T
31.4	cell.vesicle transport	MDP0000295664	 moderately similar to ( 409) AT5G08080 | Symbols: SYP132, ATSYP132 | SYP132 (SYNTAXIN OF PLANTS 132); SNAP receptor | chr5:2588532-2590488 FORWARDmoderately similar to ( 363) loc_os07g07000 12007.m05160 protein syntaxin 132, putative, expressed no original description 	T
35.2	not assigned.unknown	MDP0000492198	 moderately similar to ( 206) AT3G62630 | Symbols:  | FUNCTIONS IN: molecular_function unknown; INVOLVED IN: biological_process unknown; LOCATED IN: cellular_component unknown; EXPRESSED IN: 22 plant structures; EXPRESSED DURING: 14 growth stages; CONTAINS InterPro DOMAIN/s: Protein of unknown function DUF1645 (InterPro:IPR012442); BEST Arabidopsis thaliana protein match is: calmodulin-binding protein (TAIR:AT2G15760.1); Has 207 Blast hits to 197 proteins in 50 species: Archae - 3; Bacteria - 4; Metazoa - 52; Fungi - 9; Plants - 91; Viruses - 0; Other Eukaryotes - 48 (source: NCBI BLink). | chr3:23163937-23165079 REVERSEvery weakly similar to (90.5) loc_os03g19090 12003.m07327 protein expressed protein  no original description 	T
17.5.1	hormone metabolism.ethylene.synthesis-degradation	MDP0000163720	 moderately similar to ( 350) AT1G06620 | Symbols:  | 2-oxoglutarate-dependent dioxygenase, putative | chr1:2025618-2027094 FORWARDmoderately similar to ( 313) DV4H_CATRO Desacetoxyvindoline 4-hydroxylase (EC 1.14.11.20) - Catharanthus roseus (Rosy periwinkle) (Madagascar periwinkle)moderately similar to ( 298) loc_os04g10350 12004.m06287 protein 1-aminocyclopropane-1-carboxylate oxidase, putative, expressed  no original description 	T
35.1	not assigned.no ontology	MDP0000119568	 highly similar to ( 661) AT2G20190 | Symbols: ATCLASP, CLASP | CLASP (CLIP-ASSOCIATED PROTEIN); binding | chr2:8711862-8718813 REVERSEhighly similar to ( 552) loc_os04g42840 12004.m09249 protein HEAT repeat family protein, expressed  no original description 	T
10.8.1	cell wall.pectin*esterases.PME	MDP0000639167	 highly similar to ( 667) AT2G45220 | Symbols:  | pectinesterase family protein | chr2:18644281-18646394 REVERSEhighly similar to ( 676) PME2_CITSI Pectinesterase-2 precursor (EC 3.1.1.11) (Pectin methylesterase) (PE) - Citrus sinensis (Sweet orange)moderately similar to ( 426) loc_os01g21034 12001.m42984 protein pectinesterase-2 precursor, putative, expressed  no original description 	T
29.3.2	protein.targeting.mitochondria	MDP0000130462	 moderately similar to ( 256) AT2G42210 | Symbols: ATOEP16-3, OEP16-3 | mitochondrial import inner membrane translocase subunit Tim17/Tim22/Tim23 family protein | chr2:17590642-17591591 FORWARDmoderately similar to ( 208) loc_os02g48610 12002.m77845 protein protein translocase/ protein transporter, putative, expressed no original description 	T
31.1	cell.organisation	MDP0000196524	 moderately similar to ( 294) AT3G18670 | Symbols:  | ankyrin repeat family protein | chr3:6424135-6426471 REVERSEvery weakly similar to (76.6) loc_os07g09530 12007.m05408 protein protein binding protein, putative COG4907  no original description 	T
29.3.4.99	protein.targeting.secretory pathway.unspecified	MDP0000496812	 moderately similar to ( 225) AT2G34250 | Symbols:  | protein transport protein sec61, putative | chr2:14462635-14464572 FORWARDmoderately similar to ( 218) loc_os09g17840 12009.m05042 protein protein transport protein Sec61 alpha subunit isoform 2, putative, expressed  no original description 	T
24.2	Biodegradation of Xenobiotics.lactoylglutathione lyase	MDP0000319112	 moderately similar to ( 453) AT1G11840 | Symbols: ATGLX1 | ATGLX1 (GLYOXALASE I HOMOLOG); lactoylglutathione lyase/ metal ion binding | chr1:3996045-3997290 FORWARDmoderately similar to ( 427) LGUL_ORYSA Lactoylglutathione lyase (EC 4.4.1.5) (Methylglyoxalase) (Aldoketomutase) (Glyoxalase I) (Glx I) (Ketone-aldehyde mutase) (S-D-lactoylglutathione methylglyoxal lyase) (Allergen Ory s ?) (Allergen Glb33) (PP33) - Oryza sativa (Rice)moderately similar to ( 427) loc_os08g09250 12008.m26603 protein lactoylglutathione lyase, putative, expressed  no original description 	T
27.1	RNA.processing	MDP0000253718	 weakly similar to ( 194) AT3G62840 | Symbols:  | FUNCTIONS IN: molecular_function unknown; LOCATED IN: small nucleolar ribonucleoprotein complex, nucleus; EXPRESSED IN: 22 plant structures; EXPRESSED DURING: 13 growth stages; CONTAINS InterPro DOMAIN/s: Like-Sm ribonucleoprotein, core (InterPro:IPR001163), Like-Sm ribonucleoprotein, eukaryotic and archaea-type, core (InterPro:IPR006649), Like-Sm ribonucleoprotein-related, core (InterPro:IPR010920); BEST Arabidopsis thaliana protein match is: small nuclear ribonucleoprotein D2, putative / snRNP core protein D2, putative / Sm protein D2, putative (TAIR:AT2G47640.3); Has 537 Blast hits to 537 proteins in 165 species: Archae - 2; Bacteria - 0; Metazoa - 239; Fungi - 109; Plants - 78; Viruses - 0; Other Eukaryotes - 109 (source: NCBI BLink). | chr3:23235727-23236615 REVERSEweakly similar to ( 191) loc_os05g24970 12005.m083689 protein small nuclear ribonucleoprotein Sm D2, putative, expressed no original description 	T
26.2	misc.UDP glucosyl and glucoronyl transferases	MDP0000617184	 moderately similar to ( 421) AT5G49690 | Symbols:  | UDP-glucoronosyl/UDP-glucosyl transferase family protein | chr5:20189968-20191350 REVERSEmoderately similar to ( 205) UFOG_PETHY Anthocyanidin 3-O-glucosyltransferase (EC 2.4.1.115) (Flavonol 3-O-glucosyltransferase) (UDP-glucose flavonoid 3-O-glucosyltransferase) (Anthocyanin rhamnosyl transferase) - Petunia hybrida (Petunia)moderately similar to ( 301) loc_os01g64540 12001.m12568 protein anthocyanidin 3-O-glucosyltransferase, putative, expressed   no original description 	T
31.4	cell.vesicle transport	MDP0000243523	 moderately similar to ( 327) AT2G33120 | Symbols: SAR1, VAMP722, ATVAMP722 | SAR1 (SYNAPTOBREVIN-RELATED PROTEIN 1) | chr2:14043785-14045337 REVERSEmoderately similar to ( 332) loc_os07g09600 12007.m05415 protein vesicle-associated membrane protein 725, putative, expressed MSP1_C  no original description 	T
29.3.4.99	protein.targeting.secretory pathway.unspecified	MDP0000906354	 moderately similar to ( 268) AT3G09800 | Symbols:  | protein binding | chr3:3007153-3007916 REVERSEmoderately similar to ( 276) loc_os02g21250 12002.m07365 protein coatomer subunit zeta-1, putative, expressed no original description 	T
3.6	minor CHO metabolism.callose	MDP0000318573	 moderately similar to ( 460) AT3G07160 | Symbols: ATGSL10, gsl10 | ATGSL10 (glucan synthase-like 10); 1,3-beta-glucan synthase | chr3:2265142-2279383 REVERSEweakly similar to ( 139) loc_os06g51270 12006.m09650 protein CALS1, putative, expressed  no original description 	T
29.4	protein.postranslational modification	MDP0000589430	 highly similar to ( 724) AT3G44850 | Symbols:  | protein kinase-related | chr3:16374617-16376931 REVERSEvery weakly similar to (62.0) CDC2_MAIZE Cell division control protein 2 homolog (EC 2.7.11.22) (EC 2.7.11.23) (p34cdc2) - Zea mays (Maize)highly similar to ( 685) loc_os03g53720 12003.m10342 protein ATP binding protein, putative, expressed  no original description 	T
29.3.3	protein.targeting.chloroplast	MDP0000255224	 moderately similar to ( 388) AT2G45770 | Symbols: CPFTSY, FRD4 | CPFTSY; 7S RNA binding / GTP binding / nucleoside-triphosphatase/ nucleotide binding | chr2:18851248-18853402 FORWARDvery weakly similar to (70.1) SR541_HORVU Signal recognition particle 54 kDa protein 1 (SRP54) - Hordeum vulgare (Barley)moderately similar to ( 357) loc_os01g72800 12001.m43385 protein cell division protein ftsY, putative, expressed    no original description 	T
30.3	signalling.calcium	MDP0000193906	 moderately similar to ( 226) AT1G64850 | Symbols:  | calcium-binding EF hand family protein | chr1:24096548-24097574 REVERSEweakly similar to ( 199) loc_os04g51240 12004.m10020 protein EF hand family protein, expressed no original description 	T
27.1	RNA.processing	MDP0000340087	 weakly similar to ( 178) AT2G03870 | Symbols:  | small nuclear ribonucleoprotein, putative / snRNP, putative / Sm protein, putative | chr2:1180306-1181256 FORWARDvery weakly similar to (51.6) RUXG_MEDSA Probable small nuclear ribonucleoprotein G (snRNP-G) (Sm protein G) (Sm-G) (SmG) - Medicago sativa (Alfalfa)weakly similar to ( 170) loc_os08g08040 12008.m04939 protein LSM7 homolog, U6 small nuclear RNA associated, putative, expressed no original description 	T
34.21	transport.calcium	MDP0000141538	 highly similar to ( 525) AT3G51860 | Symbols: CAX3, ATHCX1, CAX1-LIKE, ATCAX3 | CAX3 (CATION EXCHANGER 3); calcium:cation antiporter/ calcium:hydrogen antiporter/ cation:cation antiporter | chr3:19239458-19242519 FORWARDmoderately similar to ( 485) CAX1A_ORYSA Vacuolar cation/proton exchanger 1a (Ca(2+)/H(+) exchanger 1a) (OsCAX1a) - Oryza sativa (Rice)moderately similar to ( 485) loc_os01g37690 12001.m42663 protein vacuolar cation/proton exchanger 1a, putative, expressed   no original description 	T
35.2	not assigned.unknown	MDP0000276796	 moderately similar to ( 205) AT4G29910 | Symbols: ORC5, ATORC5 | ORC5 (ORIGIN RECOGNITION COMPLEX PROTEIN 5); protein binding | chr4:14628154-14630055 REVERSEweakly similar to ( 177) loc_os03g55200 12003.m10435 protein origin recognition complex subunit 5, putative, expressed   no original description 	T
26.16	misc.myrosinases-lectin-jacalin	MDP0000780738	 weakly similar to ( 119) AT4G19840 | Symbols: ATPP2-A1, ATPP2A-1, PP2-A1 | ATPP2-A1; carbohydrate binding | chr4:10774336-10775701 FORWARDvery weakly similar to (97.4) loc_os01g06500 12001.m07280 protein lectin-like protein, putative, expressed no original description 	T
31.1	cell.organisation	MDP0000780738	 weakly similar to ( 119) AT4G19840 | Symbols: ATPP2-A1, ATPP2A-1, PP2-A1 | ATPP2-A1; carbohydrate binding | chr4:10774336-10775701 FORWARDvery weakly similar to (97.4) loc_os01g06500 12001.m07280 protein lectin-like protein, putative, expressed no original description 	T
29.2.1.1.2.1.14	protein.synthesis.ribosomal protein.prokaryotic.mitochondrion.30S subunit.S14	MDP0000146990	 weakly similar to ( 145) AT2G34520 | Symbols: RPS14 | RPS14 (mitochondrial ribosomal protein S14); structural constituent of ribosome | chr2:14548218-14548712 REVERSEweakly similar to ( 152) RT14_OENBE Mitochondrial ribosomal protein S14 - Oenothera bertiana (Bertero's evening primrose)weakly similar to ( 148) loc_os08g02640 12008.m26820 protein succinate dehydrogenase iron-sulfur protein,mitochondrial precursor, putative, expressed  no original description 	T
30.2.11	signalling.receptor kinases.leucine rich repeat XI	MDP0000812001	 nearly identical (1093) AT1G12460 | Symbols:  | leucine-rich repeat transmembrane protein kinase, putative | chr1:4247703-4250444 FORWARDmoderately similar to ( 344) RPK1_IPONI Receptor-like protein kinase precursor (EC 2.7.11.1) - Ipomoea nil (Japanese morning glory) (Pharbitis nil)highly similar to ( 921) loc_os03g03570 12003.m101708 protein ATP binding protein, putative, expressed  no original description 	T
30.2.17	signalling.receptor kinases.DUF 26	MDP0000284363	 highly similar to ( 745) AT4G28220 | Symbols: NDB1 | NDB1 (NAD(P)H dehydrogenase B1); NADH dehydrogenase/ disulfide oxidoreductase | chr4:13993078-13995651 FORWARDweakly similar to ( 160) SLSG3_BRAOL S-locus-specific glycoprotein S13 precursor (SLSG-13) (Fragment) - Brassica oleracea (Wild cabbage)highly similar to ( 711) loc_os06g47000 12006.m71453 protein expressed protein  no original description 	T
30.2.24	signalling.receptor kinases.S-locus glycoprotein like	MDP0000284363	 highly similar to ( 745) AT4G28220 | Symbols: NDB1 | NDB1 (NAD(P)H dehydrogenase B1); NADH dehydrogenase/ disulfide oxidoreductase | chr4:13993078-13995651 FORWARDweakly similar to ( 160) SLSG3_BRAOL S-locus-specific glycoprotein S13 precursor (SLSG-13) (Fragment) - Brassica oleracea (Wild cabbage)highly similar to ( 711) loc_os06g47000 12006.m71453 protein expressed protein  no original description 	T
35.1.5	not assigned.no ontology.pentatricopeptide (PPR) repeat-containing protein	MDP0000262290	 highly similar to ( 915) AT2G37230 | Symbols:  | pentatricopeptide (PPR) repeat-containing protein | chr2:15637177-15639450 REVERSEweakly similar to ( 163) RF1_ORYSA Rf1 protein, mitochondrial precursor (PPR protein) (Fertility restorer) (Restorer for CMS) - Oryza sativa (Rice)highly similar to ( 588) loc_os02g02020 12002.m05552 protein expressed protein  no original description 	T
35.1	not assigned.no ontology	MDP0000640395	 moderately similar to ( 389) AT4G28940 | Symbols:  | catalytic | chr4:14274414-14276920 FORWARDweakly similar to ( 135) BSPA_POPDE Bark storage protein A precursor - Populus deltoides (Poplar)moderately similar to ( 399) loc_os05g13970 12005.m083656 protein bark protein, putative, expressed  no original description 	T
29.3.3	protein.targeting.chloroplast	MDP0000672481	 nearly identical (1237) AT3G46740 | Symbols: TOC75-III | TOC75-III (TRANSLOCON AT THE OUTER ENVELOPE MEMBRANE OF CHLOROPLASTS 75-III); P-P-bond-hydrolysis-driven protein transmembrane transporter | chr3:17216104-17219296 REVERSEnearly identical (1176) TOC75_PEA Protein TOC75, chloroplast precursor (75 kDa translocon at the outer-envelope-membrane of chloroplasts) (Import intermediate associated protein of 75 kDa) (75 kDa chloroplast outer envelope protein) - Pisum sativum (Garden pea)nearly identical (1049) loc_os03g16440 12003.m34707 protein protein TOC75, chloroplast precursor, putative, expressed    no original description 	T
29.2.1.2.2.518	protein.synthesis.ribosomal protein.eukaryotic.60S subunit.L18A	MDP0000211254	 moderately similar to ( 339) AT2G34480 | Symbols:  | 60S ribosomal protein L18A (RPL18aB) | chr2:14532916-14534161 REVERSEmoderately similar to ( 329) RL18A_ORYSA 60S ribosomal protein L18a - Oryza sativa (Rice)moderately similar to ( 329) loc_os01g54870 12001.m11648 protein 60S ribosomal protein L18a, putative, expressed no original description 	T
34.8	transport.metabolite transporters at the envelope membrane	MDP0000050524	 highly similar to ( 568) AT1G77610 | Symbols:  | glucose-6-phosphate/phosphate translocator-related | chr1:29165489-29167486 FORWARDvery weakly similar to (97.4) TPT_MAIZE Triose phosphate/phosphate translocator, chloroplast precursor (cTPT) - Zea mays (Maize)highly similar to ( 565) loc_os08g01410 12008.m04290 protein organic anion transporter, putative, expressed no original description 	T
35.2	not assigned.unknown	MDP0000155233	 moderately similar to ( 285) AT1G19330 | Symbols:  | unknown protein | chr1:6680824-6683290 REVERSEmoderately similar to ( 260) loc_os10g21460 12010.m05162 protein expressed protein  no original description 	T
29.4	protein.postranslational modification	MDP0000939376	 nearly identical (1169) AT5G35980 | Symbols: YAK1 | protein kinase family protein | chr5:14128551-14134354 FORWARDweakly similar to ( 123) MPK4_ORYSA Mitogen-activated protein kinase 4 (EC 2.7.11.24) (MAP kinase 4) (OsMAP2) (Multiple stress-responsive MAP kinase 3) (OsMSRMK3) - Oryza sativa (Rice)highly similar to ( 783) loc_os02g47410 12002.m100053 protein ATP binding protein, putative, expressed  no original description 	T
35.2	not assigned.unknown	MDP0000685420	 weakly similar to ( 166) AT5G64230 | Symbols:  | unknown protein | chr5:25692572-25693909 FORWARDvery weakly similar to ( 100) loc_os02g51820 12002.m10213 protein expressed protein   no original description 	T
34.13	transport.peptides and oligopeptides	MDP0000182514	 highly similar to ( 719) AT2G37900 | Symbols:  | proton-dependent oligopeptide transport (POT) family protein | chr2:15864396-15866408 REVERSEhighly similar to ( 641) loc_os03g60850 12003.m101549 protein peptide transporter PTR2, putative, expressed  no original description 	T
29.5.11.4.2	protein.degradation.ubiquitin.E3.RING	MDP0000126755	 moderately similar to ( 348) AT5G60580 | Symbols:  | zinc finger (C3HC4-type RING finger) family protein | chr5:24354298-24356706 FORWARDmoderately similar to ( 441) loc_os06g46366 12006.m31777 protein protein binding protein, putative, expressed  no original description 	T
29.5.11.4.2	protein.degradation.ubiquitin.E3.RING	MDP0000248096	 moderately similar to ( 236) AT5G01520 | Symbols:  | zinc finger (C3HC4-type RING finger) family protein | chr5:206797-208399 FORWARDmoderately similar to ( 309) loc_os07g17400 12007.m06181 protein RNA-binding protein, putative, expressed TrbL  no original description 	T
29.2.1.2.1.13	protein.synthesis.ribosomal protein.eukaryotic.40S subunit.S13	MDP0000262752	 moderately similar to ( 253) AT5G62290 | Symbols:  | nucleotide-sensitive chloride conductance regulator (ICln) family protein | chr5:25019568-25020972 FORWARDmoderately similar to ( 224) RS13_SOYBN 40S ribosomal protein S13 - Glycine max (Soybean)moderately similar to ( 202) loc_os08g02410 12008.m04385 protein 40S ribosomal protein S13, putative, expressed no original description 	T
35.2	not assigned.unknown	MDP0000228792	 highly similar to ( 623) AT3G13000 | Symbols:  | transcription factor | chr3:4158214-4160818 REVERSEhighly similar to ( 550) loc_os06g49060 12006.m09431 protein ternary complex factor MIP1, putative, expressed   no original description 	T
35.2	not assigned.unknown	MDP0000151711	 weakly similar to ( 108) AT4G30010 | Symbols:  | unknown protein | chr4:14672947-14673219 FORWARDvery weakly similar to (84.3) loc_os02g07910 12002.m100775 protein fiber protein Fb15, putative, expressed no original description 	T
29.5.9	protein.degradation.AAA type	MDP0000853659	 nearly identical (1043) AT4G04910 | Symbols: NSF | NSF (N-ethylmaleimide sensitive factor); ATP binding / binding / nucleoside-triphosphatase/ nucleotide binding | chr4:2489696-2495666 REVERSEweakly similar to ( 171) CDC48_SOYBN Cell division cycle protein 48 homolog (Valosin-containing protein homolog) (VCP) - Glycine max (Soybean)highly similar to ( 994) loc_os05g44310 12005.m08557 protein vesicle-fusing ATPase, putative, expressed   no original description 	T
35.1.5	not assigned.no ontology.pentatricopeptide (PPR) repeat-containing protein	MDP0000175563	 highly similar to ( 936) AT3G46790 | Symbols: CRR2 | CRR2 (CHLORORESPIRATORY REDUCTION 2) | chr3:17231975-17233948 REVERSEvery weakly similar to (95.5) RF1_ORYSA Rf1 protein, mitochondrial precursor (PPR protein) (Fertility restorer) (Restorer for CMS) - Oryza sativa (Rice)highly similar to ( 694) loc_os01g62910 12001.m12411 protein CRR2, putative, expressed   no original description 	T
35.2	not assigned.unknown	MDP0000838190	 weakly similar to ( 117) AT1G71865 | Symbols:  | unknown protein | chr1:27030117-27030416 FORWARDweakly similar to ( 112) loc_os01g65094 12001.m150894 protein expressed protein no original description 	T
30.2.17	signalling.receptor kinases.DUF 26	MDP0000505642	 moderately similar to ( 328) AT1G66920 | Symbols:  | serine/threonine protein kinase, putative | chr1:24965410-24967432 REVERSEweakly similar to ( 183) KPRO_MAIZE Putative receptor protein kinase ZmPK1 precursor (EC 2.7.11.1) - Zea mays (Maize)moderately similar to ( 378) loc_os01g02560 12001.m06898 protein Ser/Thr receptor-like kinase, putative, expressed no original description 	T
29.4	protein.postranslational modification	MDP0000505642	 moderately similar to ( 328) AT1G66920 | Symbols:  | serine/threonine protein kinase, putative | chr1:24965410-24967432 REVERSEweakly similar to ( 183) KPRO_MAIZE Putative receptor protein kinase ZmPK1 precursor (EC 2.7.11.1) - Zea mays (Maize)moderately similar to ( 378) loc_os01g02560 12001.m06898 protein Ser/Thr receptor-like kinase, putative, expressed no original description 	T
10.7	cell wall.modification	MDP0000437664	 moderately similar to ( 312) AT4G28250 | Symbols: ATEXPB3, EXPB3, ATHEXP BETA 1.6 | ATEXPB3 (ARABIDOPSIS THALIANA EXPANSIN B3) | chr4:14000446-14001945 REVERSEmoderately similar to ( 301) EXB17_ORYSA Expansin-B17 precursor (OsEXPB17) (Beta-expansin-17) (OsaEXPb1.13) - Oryza sativa (Rice)moderately similar to ( 298) loc_os02g42650 12002.m09300 protein beta-expansin 3 precursor, putative, expressed   no original description 	T
27.3.20	RNA.regulation of transcription.G2-like transcription factor family, GARP	MDP0000323121	 moderately similar to ( 211) AT2G01060 | Symbols:  | myb family transcription factor | chr2:73456-74902 REVERSEmoderately similar to ( 252) loc_os05g41240 12005.m27895 protein MYB transcription factor, putative, expressed  no original description 	T
30.3	signalling.calcium	MDP0000292176	 highly similar to ( 567) AT1G08450 | Symbols: CRT3 | CRT3 (CALRETICULIN 3); calcium ion binding / unfolded protein binding | chr1:2668008-2671800 REVERSEmoderately similar to ( 482) CALR_NICPL Calreticulin precursor - Nicotiana plumbaginifolia (Leadwort-leaved tobacco)highly similar to ( 576) loc_os01g67054 12001.m43324 protein calreticulin-3 precursor, putative, expressed    no original description 	T
29.1.1	protein.aa activation.tyrosine-tRNA ligase	MDP0000244173	 highly similar to ( 636) AT3G02660 | Symbols: emb2768 | emb2768 (EMBRYO DEFECTIVE 2768); ATP binding / RNA binding / aminoacyl-tRNA ligase/ nucleotide binding / tyrosine-tRNA ligase | chr3:570221-571756 REVERSEhighly similar to ( 576) loc_os01g31610 12001.m09490 protein tyrosyl-tRNA synthetase, putative, expressed   no original description 	T
16.1.4.2	secondary metabolism.isoprenoids.carotenoids.phytoene dehydrogenase	MDP0000130369	 moderately similar to ( 236) loc_os04g47290 12004.m35413 protein expressed protein  no original description 	T
17.5.1	hormone metabolism.ethylene.synthesis-degradation	MDP0000128695	 moderately similar to ( 418) AT1G06620 | Symbols:  | 2-oxoglutarate-dependent dioxygenase, putative | chr1:2025618-2027094 FORWARDmoderately similar to ( 369) DV4H_CATRO Desacetoxyvindoline 4-hydroxylase (EC 1.14.11.20) - Catharanthus roseus (Rosy periwinkle) (Madagascar periwinkle)moderately similar to ( 337) loc_os04g10350 12004.m06287 protein 1-aminocyclopropane-1-carboxylate oxidase, putative, expressed  no original description 	T
29.5.9	protein.degradation.AAA type	MDP0000123181	 moderately similar to ( 454) AT3G28580 | Symbols:  | AAA-type ATPase family protein | chr3:10715736-10717238 FORWARDvery weakly similar to (57.8) CDC48_SOYBN Cell division cycle protein 48 homolog (Valosin-containing protein homolog) (VCP) - Glycine max (Soybean)moderately similar to ( 407) loc_os12g44190 12012.m08190 protein ATPase 3, putative, expressed MSP1_C  no original description 	T
17.3.1.2.7	hormone metabolism.brassinosteroid.synthesis-degradation.sterols.DWF5	MDP0000855347	 moderately similar to ( 406) AT1G50430 | Symbols: DWF5, PA, LE, ST7R, 7RED | DWF5 (DWARF 5); sterol delta7 reductase | chr1:18682175-18685555 REVERSEmoderately similar to ( 391) loc_os02g26650 12002.m34011 protein 7-dehydrocholesterol reductase, putative, expressed  no original description 	T
35.2	not assigned.unknown	MDP0000360414	  no original description 	T
20.1	stress.biotic	MDP0000860226	 moderately similar to ( 206) AT1G18250 | Symbols: ATLP-1 | ATLP-1 | chr1:6277024-6278005 REVERSEweakly similar to ( 125) TLP1_PRUPE Thaumatin-like protein 1 precursor (PpAZ44) - Prunus persica (Peach)weakly similar to ( 179) loc_os06g47600 12006.m09286 protein thaumatin-like protein precursor, putative, expressed  no original description 	T
28.1.3	DNA.synthesis/chromatin structure.histone	MDP0000460658	 moderately similar to ( 262) AT5G65360 | Symbols:  | histone H3 | chr5:26120099-26120509 REVERSEmoderately similar to ( 261) H32_WHEAT Histone H3.2 - Triticum aestivum (Wheat)moderately similar to ( 262) loc_os11g05730 12011.m04769 protein histone H3, putative, expressed  no original description 	T
27.3.67	RNA.regulation of transcription.putative transcription regulator	MDP0000417845	 moderately similar to ( 364) AT4G39680 | Symbols:  | SAP domain-containing protein | chr4:18414604-18416938 REVERSEmoderately similar to ( 303) loc_os03g26630 12003.m34873 protein DNA binding protein, putative, expressed  PRK08026  no original description 	T
31.1	cell.organisation	MDP0000268783	 highly similar to ( 700) AT2G31820 | Symbols:  | ankyrin repeat family protein | chr2:13530350-13532562 FORWARDhighly similar to ( 568) loc_os09g34280 12009.m060043 protein protein binding protein, putative, expressed  no original description 	T
27.4	RNA.RNA binding	MDP0000231846	 highly similar to ( 520) AT1G17370 | Symbols: UBP1B | UBP1B (oligouridylate binding protein 1B); mRNA 3'-UTR binding | chr1:5951842-5954825 REVERSEvery weakly similar to (79.0) ROC1_NICPL 30 kDa ribonucleoprotein, chloroplast precursor (CP-RBP30) - Nicotiana plumbaginifolia (Leadwort-leaved tobacco)highly similar to ( 546) loc_os08g40880 12008.m08067 protein mRNA 3-UTR binding protein, putative, expressed   no original description 	T
30.3	signalling.calcium	MDP0000764137	 nearly identical (1631) AT4G00900 | Symbols: ECA2, ATECA2 | ECA2 (ER-TYPE CA2+-ATPASE 2); calcium-transporting ATPase | chr4:382690-386226 REVERSEmoderately similar to ( 350) ACA2_ORYSA Calcium-transporting ATPase 2, plasma membrane-type (EC 3.6.3.8) (Ca(2+)-ATPase isoform 2) - Oryza sativa (Rice)nearly identical (1354) loc_os03g17310 12003.m07160 protein calcium-transporting ATPase 2, endoplasmic reticulum-type, putative, expressed   no original description 	T
21.4	redox.glutaredoxins	MDP0000295074	 weakly similar to ( 144) AT3G62930 | Symbols:  | glutaredoxin family protein | chr3:23261538-23261846 REVERSEvery weakly similar to (65.5) GLRX_RICCO Glutaredoxin - Ricinus communis (Castor bean)weakly similar to ( 103) loc_os01g27140 12001.m09165 protein glutaredoxin, putative, expressed no original description 	T
35.2	not assigned.unknown	MDP0000145847	 weakly similar to ( 138) AT1G50900 | Symbols:  | FUNCTIONS IN: molecular_function unknown; INVOLVED IN: biological_process unknown; LOCATED IN: chloroplast, chloroplast stroma; EXPRESSED IN: 22 plant structures; EXPRESSED DURING: 13 growth stages; CONTAINS InterPro DOMAIN/s: Ankyrin (InterPro:IPR002110); Has 30 Blast hits to 30 proteins in 12 species: Archae - 0; Bacteria - 0; Metazoa - 0; Fungi - 0; Plants - 27; Viruses - 0; Other Eukaryotes - 3 (source: NCBI BLink). | chr1:18866272-18867014 FORWARDweakly similar to ( 124) loc_os07g33660 12007.m07625 protein expressed protein no original description 	T
29.5.9	protein.degradation.AAA type	MDP0000297614	 moderately similar to ( 412) AT5G17760 | Symbols:  | AAA-type ATPase family protein | chr5:5861276-5862301 REVERSEvery weakly similar to (59.3) PRS4_ORYSA 26S protease regulatory subunit 4 homolog (TAT-binding protein homolog 2) - Oryza sativa (Rice)moderately similar to ( 354) loc_os05g51130 12005.m09179 protein mitochondrial chaperone BCS1, putative, expressed   no original description 	T
20.1	stress.biotic	MDP0000224397	 moderately similar to ( 428) AT3G12500 | Symbols: ATHCHIB, PR3, PR-3, CHI-B, B-CHI | ATHCHIB (ARABIDOPSIS THALIANA BASIC CHITINASE); chitinase | chr3:3962501-3963945 REVERSEmoderately similar to ( 486) CHIX_PEA Endochitinase precursor (EC 3.2.1.14) - Pisum sativum (Garden pea)moderately similar to ( 442) loc_os06g51050 12006.m09628 protein basic endochitinase 1 precursor, putative, expressed  no original description 	T
29.4	protein.postranslational modification	MDP0000813397	 moderately similar to ( 277) AT3G54030 | Symbols:  | protein kinase family protein | chr3:20011162-20013490 FORWARDmoderately similar to ( 234) loc_os04g58750 12004.m35529 protein protein kinase APK1B, chloroplast precursor, putative, expressed  no original description 	T
35.2	not assigned.unknown	MDP0000894465	 very weakly similar to (66.6) AT2G26520 | Symbols:  | unknown protein | chr2:11280280-11280717 FORWARD  no original description 	T
1.1.3	PS.lightreaction.cytochrome b6/f	MDP0000676293	 very weakly similar to (90.9) AT2G26500 | Symbols:  | cytochrome b6f complex subunit (petM), putative | chr2:11270370-11270747 FORWARDvery weakly similar to (52.0) PETM_SPIOL Cytochrome b6-f complex subunit 7 (Cytochrome b6-f complex subunit VII) (Cytochrome b6-f complex subunit petM) (Fragment) - Spinacia oleracea (Spinach)very weakly similar to (84.0) loc_os03g55720 12003.m10483 protein plastoquinol-plastocyanin reductase, putative, expressed  no original description 	T
13.1.1.3.1	amino acid metabolism.synthesis.central amino acid metabolism.alanine.alanine aminotransferase	MDP0000815368	 highly similar to ( 878) AT1G70580 | Symbols: AOAT2, GGT2 | AOAT2 (ALANINE-2-OXOGLUTARATE AMINOTRANSFERASE 2); L-alanine:2-oxoglutarate aminotransferase/ glycine:2-oxoglutarate aminotransferase | chr1:26613222-26615845 FORWARDmoderately similar to ( 486) ALA2_HORVU Alanine aminotransferase 2 (EC 2.6.1.2) (GPT) (Glutamic--pyruvic transaminase 2) (Glutamic--alanine transaminase 2) (ALAAT-2) - Hordeum vulgare (Barley)highly similar to ( 855) loc_os07g01760 12007.m29036 protein alanine aminotransferase 2, putative, expressed no original description 	T
35.2	not assigned.unknown	MDP0000400776	 weakly similar to ( 184) AT2G27290 | Symbols:  | unknown protein | chr2:11678586-11679765 REVERSEweakly similar to ( 168) loc_os04g33940 12004.m08488 protein expressed protein  no original description 	T
34.8	transport.metabolite transporters at the envelope membrane	MDP0000144687	 moderately similar to ( 484) AT5G46110 | Symbols: APE2, TPT | APE2 (ACCLIMATION OF PHOTOSYNTHESIS TO ENVIRONMENT 2); antiporter/ triose-phosphate transmembrane transporter | chr5:18697606-18700223 FORWARDmoderately similar to ( 488) TPT_SPIOL Triose phosphate/phosphate translocator, chloroplast precursor (cTPT) (p36) (E29) - Spinacia oleracea (Spinach)moderately similar to ( 479) loc_os01g13770 12001.m150534 protein triose phosphate/phosphate translocator, chloroplast precursor, putative, expressed  no original description 	T
35.2	not assigned.unknown	MDP0000316814	 weakly similar to ( 181) AT2G32380 | Symbols:  | FUNCTIONS IN: molecular_function unknown; INVOLVED IN: biological_process unknown; LOCATED IN: endomembrane system; EXPRESSED IN: 24 plant structures; EXPRESSED DURING: 15 growth stages; CONTAINS InterPro DOMAIN/s: Transmembrane protein 97, predicted (InterPro:IPR016964); BEST Arabidopsis thaliana protein match is: unknown protein (TAIR:AT1G05210.1); Has 102 Blast hits to 102 proteins in 33 species: Archae - 0; Bacteria - 0; Metazoa - 50; Fungi - 9; Plants - 41; Viruses - 0; Other Eukaryotes - 2 (source: NCBI BLink). | chr2:13746792-13747730 REVERSEweakly similar to ( 135) loc_os07g27450 12007.m07020 protein expressed protein no original description 	T
29.3.3	protein.targeting.chloroplast	MDP0000270246	 moderately similar to ( 430) AT2G28800 | Symbols: ALB3, ABL3 | ALB3 (ALBINO 3); P-P-bond-hydrolysis-driven protein transmembrane transporter | chr2:12357133-12359158 REVERSEmoderately similar to ( 451) PPF1_PEA Inner membrane protein PPF-1, chloroplast precursor (Post-floral-specific protein 1) - Pisum sativum (Garden pea)moderately similar to ( 408) loc_os01g05800 12001.m07211 protein inner membrane protein ALBINO3, chloroplast precursor, putative, expressed  no original description 	T
31.2.5	cell.division.plastid	MDP0000270246	 moderately similar to ( 430) AT2G28800 | Symbols: ALB3, ABL3 | ALB3 (ALBINO 3); P-P-bond-hydrolysis-driven protein transmembrane transporter | chr2:12357133-12359158 REVERSEmoderately similar to ( 451) PPF1_PEA Inner membrane protein PPF-1, chloroplast precursor (Post-floral-specific protein 1) - Pisum sativum (Garden pea)moderately similar to ( 408) loc_os01g05800 12001.m07211 protein inner membrane protein ALBINO3, chloroplast precursor, putative, expressed  no original description 	T
1.3.7	PS.calvin cycle.FBPase	MDP0000138730	 moderately similar to ( 278) AT3G54050 | Symbols:  | fructose-1,6-bisphosphatase, putative / D-fructose-1,6-bisphosphate 1-phosphohydrolase, putative / FBPase, putative | chr3:20016951-20018527 FORWARDmoderately similar to ( 291) F16P1_SOYBN Fructose-1,6-bisphosphatase, chloroplast precursor (EC 3.1.3.11) (D-fructose-1,6-bisphosphate 1-phosphohydrolase) (FBPase) - Glycine max (Soybean)moderately similar to ( 271) loc_os03g16050 12003.m07042 protein fructose-1,6-bisphosphatase, chloroplast precursor, putative, expressed no original description 	T
30.2.11	signalling.receptor kinases.leucine rich repeat XI	MDP0000922388	 nearly identical (1174) AT3G56370 | Symbols:  | leucine-rich repeat transmembrane protein kinase, putative | chr3:20899403-20902390 REVERSEmoderately similar to ( 351) RPK1_IPONI Receptor-like protein kinase precursor (EC 2.7.11.1) - Ipomoea nil (Japanese morning glory) (Pharbitis nil)nearly identical (1034) loc_os03g21730 12003.m07577 protein receptor-like protein kinase precursor, putative, expressed   no original description 	T
29.6	protein.folding	MDP0000363695	 moderately similar to ( 281) AT5G42000 | Symbols:  | ORMDL family protein | chr5:16799455-16799865 FORWARDmoderately similar to ( 273) loc_os02g45180 12002.m09557 protein ORM1-like protein 2, putative, expressed  no original description 	T
29.5	protein.degradation	MDP0000301453	 highly similar to ( 991) AT4G36760 | Symbols: ATAPP1, APP1 | ATAPP1; N-1-naphthylphthalamic acid binding / aminopeptidase | chr4:17326688-17329979 FORWARDhighly similar to ( 944) loc_os11g33330 12011.m07232 protein xaa-Pro aminopeptidase 1, putative, expressed     no original description 	T
30.5	signalling.G-proteins	MDP0000150710	 moderately similar to ( 313) AT3G07410 | Symbols: AtRABA5b | AtRABA5b (Arabidopsis Rab GTPase homolog A5b); GTP binding | chr3:2372485-2373482 REVERSEmoderately similar to ( 223) YPTC6_CHLRE Ras-related protein YPTC6 - Chlamydomonas reinhardtiimoderately similar to ( 292) loc_os08g41340 12008.m08112 protein ras-related protein ARA-4, putative, expressed   no original description 	T
28.1	DNA.synthesis/chromatin structure	MDP0000167767	 weakly similar to ( 175) AT4G29910 | Symbols: ORC5, ATORC5 | ORC5 (ORIGIN RECOGNITION COMPLEX PROTEIN 5); protein binding | chr4:14628154-14630055 REVERSEweakly similar to ( 164) loc_os03g55200 12003.m10435 protein origin recognition complex subunit 5, putative, expressed no original description 	T
29.4.1.57	protein.postranslational modification.kinase.receptor like cytoplasmatic kinase VII	MDP0000435842	 nearly identical (1234) AT1G66150 | Symbols: TMK1 | TMK1 (TRANSMEMBRANE KINASE 1); transmembrane receptor protein serine/threonine kinase | chr1:24631503-24634415 FORWARDmoderately similar to ( 221) CRI4_MAIZE Putative receptor protein kinase CRINKLY4 precursor (EC 2.7.11.1) - Zea mays (Maize)nearly identical (1060) loc_os03g50810 12003.m10067 protein receptor protein kinase TMK1 precursor, putative, expressed  no original description 	T
27.3.40	RNA.regulation of transcription.Aux/IAA family	MDP0000324919	 weakly similar to ( 137) AT1G04240 | Symbols: SHY2, IAA3 | SHY2 (SHORT HYPOCOTYL 2); transcription factor | chr1:1128564-1129319 REVERSEweakly similar to ( 137) AX22D_PHAAU Auxin-induced protein 22D (Indole-3-acetic acid-induced protein ARG13) - Phaseolus aureus (Mung bean) (Vigna radiata)weakly similar to ( 112) loc_os12g40900 12012.m07873 protein OsIAA31 - Auxin-responsive Aux/IAA gene family member, expressed  no original description 	T
35.1.5	not assigned.no ontology.pentatricopeptide (PPR) repeat-containing protein	MDP0000319929	 moderately similar to ( 224) AT2G35760 | Symbols:  | integral membrane family protein | chr2:15032059-15033094 FORWARDweakly similar to ( 143) loc_os12g32970 12012.m07095 protein membrane protein, putative, expressed no original description 	T
35.1	not assigned.no ontology	MDP0000521309	 moderately similar to ( 246) AT3G12030 | Symbols:  | unknown protein | chr3:3834465-3835055 REVERSEweakly similar to ( 198) loc_os03g51160 12003.m101475 protein fb27, putative, expressed  no original description 	T
29.4.1.57	protein.postranslational modification.kinase.receptor like cytoplasmatic kinase VII	MDP0000310218	 highly similar to ( 792) AT1G60780 | Symbols: HAP13 | HAP13 (HAPLESS 13); protein binding | chr1:22369289-22371885 REVERSEweakly similar to ( 117) CRI4_MAIZE Putative receptor protein kinase CRINKLY4 precursor (EC 2.7.11.1) - Zea mays (Maize)highly similar to ( 785) loc_os01g50770 12001.m42699 protein AP-1 complex subunit mu-1, putative, expressed   no original description 	T
35.2	not assigned.unknown	MDP0000844093	 highly similar to ( 566) AT5G47860 | Symbols:  | unknown protein | chr5:19381656-19384310 FORWARDhighly similar to ( 566) loc_os04g33280 12004.m101523 protein expressed protein no original description 	T
34.15	transport.potassium	MDP0000889811	 moderately similar to ( 454) AT4G22200 | Symbols: AKT2/3, AKT3, AKT2 | AKT2/3 (ARABIDOPSIS POTASSIUM TRANSPORT 2/3); cyclic nucleotide binding / inward rectifier potassium channel/ protein binding | chr4:11746666-11750091 REVERSEmoderately similar to ( 204) AKT1_ORYSA Potassium channel AKT1 (OsAKT1) - Oryza sativa (Rice)moderately similar to ( 392) loc_os05g35410 12005.m07771 protein potassium channel AKT2/3, putative, expressed  no original description 	T
34.3	transport.amino acids	MDP0000155074	 highly similar to ( 794) AT2G21050 | Symbols:  | amino acid permease, putative | chr2:9034289-9036439 FORWARDhighly similar to ( 783) LAX5_MEDTR Auxin transporter-like protein 5 (AUX1-like protein 5) (MtLAX5) - Medicago truncatula (Barrel medic)highly similar to ( 748) loc_os03g14080 12003.m06851 protein auxin transporter-like protein 3, putative, expressed  no original description 	T
33.99	development.unspecified	MDP0000214579	 highly similar to ( 745) AT2G39210 | Symbols:  | nodulin family protein | chr2:16366287-16368231 REVERSEhighly similar to ( 633) loc_os03g58580 12003.m10763 protein nitrate and chloride transporter, putative, expressed   no original description 	T
35.2	not assigned.unknown	MDP0000551040	 moderately similar to ( 223) AT5G47870 | Symbols:  | unknown protein | chr5:19384555-19385808 REVERSEweakly similar to ( 194) loc_os03g63450 12003.m11213 protein snRK1-interacting protein 1, putative, expressed  no original description 	T
26.10	misc.cytochrome P450	MDP0000450934	 moderately similar to ( 256) AT3G14690 | Symbols: CYP72A15 | CYP72A15; electron carrier/ heme binding / iron ion binding / monooxygenase/ oxygen binding | chr3:4937410-4939310 FORWARDmoderately similar to ( 233) C72A1_CATRO Cytochrome P450 72A1 (EC 1.3.3.9) (CYPLXXII) (Secologanin synthase) (SLS) - Catharanthus roseus (Rosy periwinkle) (Madagascar periwinkle)moderately similar to ( 233) loc_os01g43710 12001.m10633 protein cytochrome P450 72A1, putative, expressed  no original description 	T
30.5	signalling.G-proteins	MDP0000302770	 moderately similar to ( 381) AT1G09630 | Symbols: ATRAB11C, ATRABA2A, ATRAB-A2A, RAB-A2A | ATRAB11C (ARABIDOPSIS RAB GTPASE 11C); GTP binding | chr1:3118350-3119571 REVERSEmoderately similar to ( 328) RB2BV_BETVU Ras-related protein Rab2BV - Beta vulgaris (Sugar beet)moderately similar to ( 335) loc_os05g01480 12005.m04686 protein ras-related protein Rab11C, putative, expressed no original description 	T
35.2	not assigned.unknown	MDP0000240286	 weakly similar to ( 187) loc_os05g33290 12005.m07561 protein expressed protein  no original description 	T
29.7.4	protein.glycosylation.UDP-glucose glycoprotein glucosyltransferase	MDP0000231077	 nearly identical (2181) AT1G71220 | Symbols: EBS1 | EBS1 (EMS-mutagenized bri1 suppressor 1); UDP-glucose:glycoprotein glucosyltransferase/ transferase, transferring glycosyl groups / transferase, transferring hexosyl groups | chr1:26841664-26851730 FORWARDhighly similar to ( 958) loc_os02g44510 12002.m77741 protein UDP-glucose glycoprotein glucosyltransferase 1 precursor, putative, expressed  no original description 	T
35.2	not assigned.unknown	MDP0000246697	    Sporozoite_P67  no original description 	T
20.2.99	stress.abiotic.unspecified	MDP0000360716	 weakly similar to ( 194) AT4G08685 | Symbols: SAH7 | SAH7 | chr4:5550563-5551259 FORWARDweakly similar to ( 130) PSC13_MAIZE Pollen-specific protein C13 precursor - Zea mays (Maize)weakly similar to ( 157) loc_os10g22590 12010.m05266 protein pollen-specific protein C13 precursor, putative, expressed  no original description 	T
17.3.1.2.8	hormone metabolism.brassinosteroid.synthesis-degradation.sterols.DWF1	MDP0000682675	 highly similar to ( 937) AT3G19820 | Symbols: DWF1, DIM, EVE1, DIM1, CBB1 | DWF1 (DWARF 1); calmodulin binding / catalytic | chr3:6879835-6881616 REVERSEnearly identical (1006) DIM_PEA Cell elongation protein diminuto - Pisum sativum (Garden pea)highly similar to ( 971) loc_os10g25780 12010.m21783 protein cell elongation protein diminuto, putative, expressed   no original description 	T
20.1	stress.biotic	MDP0000733468	 moderately similar to ( 367) AT4G38660 | Symbols:  | thaumatin, putative | chr4:18066448-18067792 REVERSEmoderately similar to ( 233) TP1A_MALDO Thaumatin-like protein 1a precursor (Allergen Mal d 2) (Mdtl1) (Pathogenesis-related protein 5a) (PR-5a) - Malus domestica (Apple) (Malus sylvestris)moderately similar to ( 300) loc_os03g14030 12003.m06846 protein thaumatin-like protein 1 precursor, putative, expressed  no original description 	T
34.16	transport.ABC transporters and multidrug resistance systems	MDP0000150931	 highly similar to ( 606) AT1G17840 | Symbols: WBC11, ABCG11, DSO, COF1, ATWBC11 | WBC11 (WHITE-BROWN COMPLEX HOMOLOG PROTEIN 11); ATPase, coupled to transmembrane movement of substances / fatty acid transporter | chr1:6142870-6145894 FORWARDweakly similar to ( 183) PDR2_ORYSA Probable pleiotropic drug resistance protein 2 - Oryza sativa (Rice)highly similar to ( 768) loc_os09g29660 12009.m06117 protein ATP-binding cassette sub-family G member 2, putative, expressed   no original description 	T
35.1	not assigned.no ontology	MDP0000887328	 moderately similar to ( 250) AT4G23630 | Symbols: BTI1 | BTI1 (VIRB2-INTERACTING PROTEIN 1) | chr4:12318070-12319574 FORWARDmoderately similar to ( 251) loc_os01g53520 12001.m43176 protein seed maturation protein, putative, expressed   no original description 	T
8.1.7	TCA / org transformation.TCA.succinate dehydrogenase	MDP0000163246	 very weakly similar to ( 100) AT5G09600 | Symbols: SDH3-1 | SDH3-1; succinate dehydrogenase | chr5:2979777-2980527 FORWARDvery weakly similar to (78.6) C560_MARPO Succinate dehydrogenase cytochrome b560 subunit (Succinate dehydrogenase, subunit III) - Marchantia polymorpha (Liverwort)very weakly similar to (62.8) loc_os03g63410 12003.m34958 protein elongation factor Tu, mitochondrial precursor, putative, expressed  no original description 	T
35.1	not assigned.no ontology	MDP0000222943	 very weakly similar to (59.3) AT4G05030 | Symbols:  | heavy-metal-associated domain-containing protein | chr4:2576790-2577974 REVERSEvery weakly similar to (64.7) loc_os04g39010 12004.m08890 protein ATFP4, putative, expressed  no original description 	T
35.1.5	not assigned.no ontology.pentatricopeptide (PPR) repeat-containing protein	MDP0000309372	 highly similar to ( 549) AT5G08310 | Symbols:  | pentatricopeptide (PPR) repeat-containing protein | chr5:2670134-2675254 REVERSEweakly similar to ( 125) SYM_ORYSA Probable methionyl-tRNA synthetase (EC 6.1.1.10) (Methionine--tRNA ligase) (MetRS) - Oryza sativa (Rice)moderately similar to ( 471) loc_os10g30590 12010.m65449 protein tetratricopeptide-like helical, putative  no original description 	T
17.8.1	hormone metabolism.salicylic acid.synthesis-degradation	MDP0000292448	 moderately similar to ( 275) AT5G38020 | Symbols:  | S-adenosyl-L-methionine:carboxyl methyltransferase family protein | chr5:15165953-15167612 REVERSEmoderately similar to ( 263) BAMT_ANTMA Benzoate carboxyl methyltransferase (EC 2.1.1.-) (S-adenosyl-L-methionine:benzoic acid carboxyl methyltransferase) - Antirrhinum majus (Garden snapdragon)moderately similar to ( 251) loc_os02g48770 12002.m09911 protein jasmonate O-methyltransferase, putative, expressed  no original description 	T
27.3.16	RNA.regulation of transcription.CCAAT box binding factor family, HAP5	MDP0000146605	 moderately similar to ( 256) AT3G48590 | Symbols: HAP5A, ATHAP5A, NF-YC1 | NF-YC1 (NUCLEAR FACTOR Y, SUBUNIT C1); DNA binding / transcription activator/ transcription factor | chr3:18008893-18009938 REVERSEmoderately similar to ( 203) loc_os03g14669 12003.m101699 protein nuclear transcription factor Y subunit C-1, putative, expressed MSP1_C  no original description 	T
27.3.67	RNA.regulation of transcription.putative transcription regulator	MDP0000181488	 moderately similar to ( 203) AT3G57540 | Symbols:  | remorin family protein | chr3:21301623-21302924 REVERSEvery weakly similar to (58.5) REMO_SOLTU Remorin (pp34) - Solanum tuberosum (Potato)weakly similar to ( 190) loc_os07g38170 12007.m08065 protein DNA binding protein, putative, expressed  no original description 	T
30.11	signalling.light	MDP0000750922	 moderately similar to ( 323) AT4G38180 | Symbols: FRS5 | FRS5 (FAR1-related sequence 5); zinc ion binding | chr4:17906702-17909404 REVERSEmoderately similar to ( 455) loc_os12g03660 12012.m04363 protein transposon protein, putative, unclassified   no original description 	T
21.1	redox.thioredoxin	MDP0000415439	 weakly similar to ( 130) AT3G51030 | Symbols: ATTRX1, ATTRX H1 | ATTRX1; oxidoreductase, acting on sulfur group of donors, disulfide as acceptor | chr3:18951123-18951955 REVERSEweakly similar to ( 134) TRXH_RICCO Thioredoxin H-type (TRX-H) - Ricinus communis (Castor bean)weakly similar to ( 112) loc_os07g08840 12007.m079626 protein thioredoxin H-type, putative, expressed no original description 	T
8.1.1.2	TCA / org transformation.TCA.pyruvate DH.E2	MDP0000150127	 moderately similar to ( 223) AT3G52200 | Symbols: LTA3 | LTA3; ATP binding / dihydrolipoyllysine-residue acetyltransferase | chr3:19360317-19366091 FORWARDmoderately similar to ( 211) loc_os06g30460 12006.m091708 protein dihydrolipoyllysine-residue acetyltransferase component of pyruvatedehydrogenase complex, mitochondrial precursor, putative, expressed no original description 	T
34.19.2	transport.Major Intrinsic Proteins.TIP	MDP0000917582	 moderately similar to ( 382) AT3G16240 | Symbols: DELTA-TIP, TIP2;1, DELTA-TIP1, AQP1, ATTIP2;1 | DELTA-TIP; ammonia transporter/ methylammonium transmembrane transporter/ water channel | chr3:5505534-5506788 FORWARDmoderately similar to ( 363) TIP_ANTMA Probable aquaporin TIP-type (Tonoplast intrinsic protein DiP) (Dark intrinsic protein) - Antirrhinum majus (Garden snapdragon)moderately similar to ( 355) loc_os06g22960 12006.m06949 protein aquaporin TIP2.2, putative, expressed  no original description 	T
35.2	not assigned.unknown	MDP0000217893	 highly similar to ( 992) AT4G32910 | Symbols:  | INVOLVED IN: biological_process unknown; LOCATED IN: cellular_component unknown; EXPRESSED IN: 23 plant structures; EXPRESSED DURING: 13 growth stages; CONTAINS InterPro DOMAIN/s: Nucleoporin, Nup85-like (InterPro:IPR011502); Has 161 Blast hits to 158 proteins in 60 species: Archae - 0; Bacteria - 0; Metazoa - 128; Fungi - 10; Plants - 21; Viruses - 0; Other Eukaryotes - 2 (source: NCBI BLink). | chr4:15881364-15885210 FORWARDhighly similar to ( 885) loc_os01g54240 12001.m42721 protein nup85 Nucleoporin family protein, expressed   no original description 	T
29.2.1.1.1.2.3	protein.synthesis.ribosomal protein.prokaryotic.chloroplast.50S subunit.L3	MDP0000475073	 moderately similar to ( 377) AT2G43030 | Symbols:  | ribosomal protein L3 family protein | chr2:17894898-17895713 FORWARDmoderately similar to ( 372) RK3_TOBAC 50S ribosomal protein L3, chloroplast precursor (Fragment) - Nicotiana tabacum (Common tobacco)moderately similar to ( 348) loc_os02g04460 12002.m05795 protein 50S ribosomal protein L3, chloroplast precursor, putative, expressed  no original description 	T
1.2.1	PS.photorespiration.phosphoglycolate phosphatase	MDP0000245346	 moderately similar to ( 313) AT5G36700 | Symbols: ATPGLP1, PGLP1 | PGLP1 (2-PHOSPHOGLYCOLATE PHOSPHATASE 1); phosphoglycolate phosphatase | chr5:14422120-14424430 REVERSEmoderately similar to ( 303) loc_os04g41340 12004.m09113 protein 4-nitrophenylphosphatase, putative, expressed  no original description 	T
28.2	DNA.repair	MDP0000089368	 moderately similar to ( 493) AT1G79650 | Symbols: RAD23 | RAD23; damaged DNA binding | chr1:29972406-29975132 REVERSEmoderately similar to ( 322) RAD23_ORYSA Probable DNA repair protein RAD23 (OsRAD23) - Oryza sativa (Rice)moderately similar to ( 427) loc_os02g08300 12002.m06176 protein DNA repair protein RAD23-1, putative, expressed   no original description 	T
30.3	signalling.calcium	MDP0000328568	 moderately similar to ( 290) AT3G50360 | Symbols: ATCEN2, CEN1 | ATCEN2 (CENTRIN2); calcium ion binding | chr3:18674421-18675502 FORWARDmoderately similar to ( 204) CATR_CHLRE Caltractin (Centrin) (20 kDa calcium-binding protein) - Chlamydomonas reinhardtiimoderately similar to ( 261) loc_os07g42660 12007.m08497 protein caltractin, putative, expressed no original description 	T
29.5.11.4.2	protein.degradation.ubiquitin.E3.RING	MDP0000194613	 highly similar to ( 546) AT4G31890 | Symbols:  | armadillo/beta-catenin repeat family protein | chr4:15427290-15429049 REVERSEvery weakly similar to (65.5) SPL11_ORYSA Spotted leaf protein 11 (Spotted leaf11) (Cell death-related protein SPL11) - Oryza sativa (Rice)moderately similar to ( 334) loc_os09g25000 12009.m05654 protein spotted leaf protein 11, putative, expressed   no original description 	T
29.2.1.1.1.2.4	protein.synthesis.ribosomal protein.prokaryotic.chloroplast.50S subunit.L4	MDP0000299779	 moderately similar to ( 218) AT1G07320 | Symbols: RPL4 | RPL4; poly(U) binding / structural constituent of ribosome | chr1:2249190-2250173 FORWARDmoderately similar to ( 214) RK4_TOBAC 50S ribosomal protein L4, chloroplast precursor (CL4) (R-protein L4) - Nicotiana tabacum (Common tobacco)moderately similar to ( 214) loc_os03g15870 12003.m07024 protein 50S ribosomal protein L4, chloroplast precursor, putative, expressed Sporozoite_P67 TrbL  no original description 	T
26.28	misc.GDSL-motif lipase	MDP0000306888	 highly similar to ( 771) AT1G76160 | Symbols: sks5 | sks5 (SKU5 Similar 5); copper ion binding / oxidoreductase | chr1:28578211-28581020 REVERSEhighly similar to ( 545) ASO_BRANA L-ascorbate oxidase homolog precursor (EC 1.10.3.3) (Ascorbase) - Brassica napus (Rape)highly similar to ( 697) loc_os06g46500 12006.m09176 protein L-ascorbate oxidase homolog precursor, putative, expressed   no original description 	T
17.5.1	hormone metabolism.ethylene.synthesis-degradation	MDP0000133105	 moderately similar to ( 232) AT1G52820 | Symbols:  | 2-oxoglutarate-dependent dioxygenase, putative | chr1:19669216-19670321 FORWARDvery weakly similar to (83.2) LDOX_MALDO Leucoanthocyanidin dioxygenase (EC 1.14.11.19) (LDOX) (Leucocyanidin oxygenase) (Leucoanthocyanidin hydroxylase) (Anthocyanidin synthase) - Malus domestica (Apple) (Malus sylvestris)weakly similar to ( 126) loc_os08g32170 12008.m07211 protein oxidoreductase, putative, expressed  no original description 	T
35.1.5	not assigned.no ontology.pentatricopeptide (PPR) repeat-containing protein	MDP0000258737	 moderately similar to ( 368) AT4G37210 | Symbols:  | tetratricopeptide repeat (TPR)-containing protein | chr4:17512376-17514421 FORWARDmoderately similar to ( 267) loc_os07g03070 12007.m04778 protein TPR Domain containing protein, expressed  no original description 	T
29.3.4.99	protein.targeting.secretory pathway.unspecified	MDP0000329955	 weakly similar to ( 101) AT5G50460 | Symbols:  | protein transport protein SEC61 gamma subunit, putative | chr5:20552168-20552509 REVERSEweakly similar to ( 102) SC61G_ORYSA Protein transport protein SEC61 subunit gamma - Oryza sativa (Rice)weakly similar to ( 105) loc_os06g44374 12006.m91828 protein protein transport protein SEC61 gamma subunit, putative, expressed no original description 	T
4.1.10	glycolysis.cytosolic branch.aldolase	MDP0000120450	 moderately similar to ( 226) AT5G03690 | Symbols:  | fructose-bisphosphate aldolase, putative | chr5:963389-964982 REVERSEmoderately similar to ( 229) ALF_SPIOL Fructose-bisphosphate aldolase, cytoplasmic isozyme (EC 4.1.2.13) - Spinacia oleracea (Spinach)moderately similar to ( 229) loc_os01g67860 12001.m12890 protein fructose-bisphosphate aldolase cytoplasmic isozyme, putative, expressed   no original description 	T
35.2	not assigned.unknown	MDP0000171985	     no original description 	T
34.13	transport.peptides and oligopeptides	MDP0000274582	 highly similar to ( 657) AT2G02040 | Symbols: ATPTR2-B, NTR1, PTR2-B, PTR2, ATPTR2 | PTR2 (PEPTIDE TRANSPORTER 2); dipeptide transporter/ high affinity oligopeptide transporter/ nitrate transmembrane transporter/ peptide transporter/ transporter/ tripeptide transporter | chr2:487542-489707 FORWARDhighly similar to ( 648) loc_os03g51050 12003.m10087 protein peptide transporter PTR2, putative, expressed   no original description 	T
29.4	protein.postranslational modification	MDP0000278839	 highly similar to ( 530) AT5G58380 | Symbols: CIPK10, PKS2, SIP1, SNRK3.8 | SIP1 (SOS3-INTERACTING PROTEIN 1); ATP binding / kinase/ protein kinase/ protein serine/threonine kinase | chr5:23597092-23598531 REVERSEmoderately similar to ( 389) CIPK1_ORYSA CIPK-like protein 1 (EC 2.7.11.1) (OsCK1) - Oryza sativa (Rice)highly similar to ( 550) loc_os05g26820 12005.m06974 protein CBL-interacting serine/threonine-protein kinase 15, putative, expressed  MSP1_C  no original description 	T
35.2	not assigned.unknown	MDP0000202145	 very weakly similar to (63.9) AT5G22390 | Symbols:  | unknown protein | chr5:7418383-7418991 REVERSEvery weakly similar to (86.3) loc_os02g33090 12002.m08402 protein expressed protein MSP1_C  no original description 	T
35.1	not assigned.no ontology	MDP0000319790	 moderately similar to ( 254) AT3G10915 | Symbols:  | reticulon family protein | chr3:3416101-3417497 REVERSEmoderately similar to ( 234) loc_os03g21840 12003.m07588 protein seed maturation protein, putative, expressed no original description 	T
26.28	misc.GDSL-motif lipase	MDP0000149484	 moderately similar to ( 210) AT4G16230 | Symbols:  | carboxylesterase/ hydrolase, acting on ester bonds | chr4:9185298-9186447 FORWARDvery weakly similar to (84.0) APG_BRANA Anter-specific proline-rich protein APG (Protein CEX) (Fragment) - Brassica napus (Rape)moderately similar to ( 254) loc_os03g19670 12003.m35186 protein anther-specific proline-rich protein APG precursor, putative, expressed no original description 	T
16.2	secondary metabolism.phenylpropanoids	MDP0000123589	 highly similar to ( 582) AT1G27620 | Symbols:  | transferase family protein | chr1:9608444-9610432 FORWARDweakly similar to ( 168) HCBT1_DIACA Anthranilate N-benzoyltransferase protein 1 (EC 2.3.1.144) (Anthranilate N-hydroxycinnamoyl/benzoyltransferase 1) - Dianthus caryophyllus (Carnation) (Clove pink)moderately similar to ( 426) loc_os01g01520 12001.m06797 protein anthranilate N-benzoyltransferase protein 1, putative, expressed   no original description 	T
29.5.5	protein.degradation.serine protease	MDP0000595966	 moderately similar to ( 442) AT1G49970 | Symbols: CLPR1, NCLPP5, SVR2 | CLPR1; serine-type endopeptidase | chr1:18501936-18504462 REVERSEvery weakly similar to (69.7) CLPP_MESVI ATP-dependent Clp protease proteolytic subunit (EC 3.4.21.92) (Endopeptidase Clp) - Mesostigma viridemoderately similar to ( 315) loc_os05g51450 12005.m09210 protein endopeptidase Clp, putative, expressed  no original description 	T
31.1	cell.organisation	MDP0000292132	 moderately similar to ( 281) AT5G04700 | Symbols:  | FUNCTIONS IN: molecular_function unknown; INVOLVED IN: biological_process unknown; LOCATED IN: cellular_component unknown; CONTAINS InterPro DOMAIN/s: Ankyrin (InterPro:IPR002110); BEST Arabidopsis thaliana protein match is: unknown protein (TAIR:AT5G04680.1); Has 868 Blast hits to 744 proteins in 75 species: Archae - 0; Bacteria - 22; Metazoa - 164; Fungi - 15; Plants - 471; Viruses - 0; Other Eukaryotes - 196 (source: NCBI BLink). | chr5:1354240-1356754 REVERSEvery weakly similar to (60.1) loc_os11g14544 12011.m61739 protein expressed protein   no original description 	T
29.2.2.3.1	protein.synthesis.ribosome biogenesis.Pre-rRNA processing and modifications.snoRNPs	MDP0000165605	 weakly similar to ( 179) AT3G03920 | Symbols:  | Gar1 RNA-binding region family protein | chr3:1009123-1010379 REVERSEweakly similar to ( 185) loc_os11g37080 12011.m07556 protein h/ACA ribonucleoprotein complex subunit 1-like protein 1, putative, expressed no original description 	T
27.3.32	RNA.regulation of transcription.WRKY domain transcription factor family	MDP0000127976	 weakly similar to ( 158) AT2G47260 | Symbols: WRKY23, ATWRKY23 | WRKY23; transcription factor | chr2:19405045-19406446 REVERSEweakly similar to ( 144) loc_os07g02060 12007.m04680 protein OsWRKY29 - Superfamily of rice TFs having WRKY and zinc finger domains, expressed  no original description 	T
26.6	misc.O-methyl transferases	MDP0000275302	 moderately similar to ( 368) AT3G53140 | Symbols:  | O-diphenol-O-methyl transferase, putative | chr3:19695692-19697355 FORWARDweakly similar to ( 184) COMT1_CATRO Caffeic acid 3-O-methyltransferase (EC 2.1.1.68) (S-adenosysl-L-methionine:caffeic acid 3-O-methyltransferase) (COMT) (CAOMT) - Catharanthus roseus (Rosy periwinkle) (Madagascar periwinkle)moderately similar to ( 275) loc_os02g57760 12002.m10797 protein caffeic acid 3-O-methyltransferase, putative, expressed  no original description 	T
29.2.1.2.1.13	protein.synthesis.ribosomal protein.eukaryotic.40S subunit.S13	MDP0000753788	 moderately similar to ( 211) AT4G00100 | Symbols: ATRPS13A, RPS13, PFL2 | ATRPS13A (ARABIDOPSIS THALIANA RIBOSOMAL PROTEIN S13A); structural constituent of ribosome | chr4:37172-38123 FORWARDmoderately similar to ( 216) RS13_SOYBN 40S ribosomal protein S13 - Glycine max (Soybean)weakly similar to ( 188) loc_os08g02400 12008.m04384 protein 40S ribosomal protein S13, putative, expressed   no original description 	T
16.2.1.9	secondary metabolism.phenylpropanoids.lignin biosynthesis.COMT	MDP0000164000	 weakly similar to ( 116) AT5G54160 | Symbols: ATOMT1, OMT1 | ATOMT1 (O-METHYLTRANSFERASE 1); caffeate O-methyltransferase/ myricetin 3'-O-methyltransferase/ quercetin 3-O-methyltransferase | chr5:21982075-21984167 FORWARDweakly similar to ( 127) COMT1_CAPAN Caffeic acid 3-O-methyltransferase (EC 2.1.1.68) (S-adenosysl-L-methionine:caffeic acid 3-O-methyltransferase) (COMT) (CAOMT) - Capsicum annuum (Bell pepper)very weakly similar to (93.2) loc_os08g06100 12008.m04749 protein quercetin 3-O-methyltransferase 1, putative, expressed no original description 	T
35.1	not assigned.no ontology	MDP0000138170	 weakly similar to ( 111) AT5G57370 | Symbols:  | unknown protein | chr5:23244811-23245470 FORWARDvery weakly similar to (91.3) loc_os02g26349 12002.m77802 protein splicing factor, arginine/serine-rich 7, putative, expressed  no original description 	T
35.2	not assigned.unknown	MDP0000669769	 moderately similar to ( 318) AT2G23360 | Symbols:  | transport protein-related | chr2:9949456-9952727 FORWARDweakly similar to ( 165) loc_os04g55570 12004.m10448 protein expressed protein  no original description 	T
17.5.1	hormone metabolism.ethylene.synthesis-degradation	MDP0000248981	 highly similar to ( 511) AT5G51810 | Symbols: GA20OX2, AT2353, ATGA20OX2 | GA20OX2 (GIBBERELLIN 20 OXIDASE 2); gibberellin 20-oxidase | chr5:21055389-21056746 REVERSEmoderately similar to ( 484) GAO1A_WHEAT Gibberellin 20 oxidase 1-A (EC 1.14.11.-) (Gibberellin C-20 oxidase 1-B) (GA 20-oxidase 1-A) (Ta20ox1A) (TaGA20ox1-A) - Triticum aestivum (Wheat)moderately similar to ( 437) loc_os03g63970 12003.m11264 protein gibberellin 20 oxidase 1, putative, expressed  no original description 	T
29.5.11.4.2	protein.degradation.ubiquitin.E3.RING	MDP0000549038	 moderately similar to ( 316) AT1G69330 | Symbols:  | zinc finger (C3HC4-type RING finger) family protein | chr1:26064138-26064950 REVERSEmoderately similar to ( 290) loc_os03g31320 12003.m35286 protein RING zinc finger protein-like, putative, expressed no original description 	T
15.2	metal handling.binding, chelation and storage	MDP0000466190	 very weakly similar to (86.3) AT3G15353 | Symbols: MT3 | MT3 (METALLOTHIONEIN 3); copper ion binding | chr3:5180825-5181325 REVERSEweakly similar to ( 130) MT3_MALDO Metallothionein-like protein type 3 (MT-3) - Malus domestica (Apple) (Malus sylvestris)very weakly similar to (85.5) loc_os05g11320 12005.m05601 protein metallothionein-like protein type 3, putative, expressed no original description 	T
35.2	not assigned.unknown	MDP0000321946	 moderately similar to ( 400) AT3G53400 | Symbols:  | FUNCTIONS IN: molecular_function unknown; INVOLVED IN: biological_process unknown; BEST Arabidopsis thaliana protein match is: CPUORF47 (CONSERVED PEPTIDE UPSTREAM OPEN READING FRAME 47) (TAIR:AT5G03190.1); Has 120 Blast hits to 120 proteins in 12 species: Archae - 0; Bacteria - 0; Metazoa - 0; Fungi - 0; Plants - 115; Viruses - 0; Other Eukaryotes - 5 (source: NCBI BLink). | chr3:19798526-19799926 FORWARDweakly similar to ( 114) loc_os07g49210 12007.m09130 protein helicase conserved C-terminal domain containing protein, expressed no original description 	T
35.1.5	not assigned.no ontology.pentatricopeptide (PPR) repeat-containing protein	MDP0000312559	 moderately similar to ( 282) AT1G60420 | Symbols:  | DC1 domain-containing protein | chr1:22261978-22264243 FORWARDweakly similar to ( 122) CDPK2_MAIZE Calcium-dependent protein kinase 2 (EC 2.7.11.1) (CDPK 2) - Zea mays (Maize)moderately similar to ( 366) loc_os01g58194 12001.m11963 protein protein disulfide isomerase, putative, expressed     no original description 	T
27.3.8	RNA.regulation of transcription.C2C2(Zn) DOF zinc finger family	MDP0000808238	 weakly similar to ( 116) AT1G28310 | Symbols:  | Dof-type zinc finger domain-containing protein | chr1:9912534-9913685 REVERSEweakly similar to ( 104) PBF_MAIZE Dof zinc finger protein PBF (Prolamin box-binding factor) - Zea mays (Maize)weakly similar to ( 110) loc_os07g32510 12007.m07514 protein expressed protein  no original description 	T
29.4	protein.postranslational modification	MDP0000141686	 weakly similar to ( 130) AT3G17680 | Symbols:  | FUNCTIONS IN: molecular_function unknown; INVOLVED IN: biological_process unknown; LOCATED IN: cellular_component unknown; EXPRESSED IN: 16 plant structures; EXPRESSED DURING: 8 growth stages; CONTAINS InterPro DOMAIN/s: KIP1-like (InterPro:IPR011684); BEST Arabidopsis thaliana protein match is: unknown protein (TAIR:AT1G48405.1); Has 75 Blast hits to 73 proteins in 16 species: Archae - 0; Bacteria - 2; Metazoa - 5; Fungi - 2; Plants - 52; Viruses - 0; Other Eukaryotes - 14 (source: NCBI BLink). | chr3:6043138-6044267 REVERSEweakly similar to ( 188) loc_os03g01980 12003.m05742 protein protein Kinase interacting protein, putative, expressed  no original description 	T
35.1.1	not assigned.no ontology.ABC1 family protein	MDP0000182419	 highly similar to ( 997) AT4G31390 | Symbols:  | ABC1 family protein | chr4:15233126-15236764 FORWARDnearly identical (1011) loc_os11g11000 12011.m079963 protein ubiquinone biosynthesis protein ubiB, putative, expressed   no original description 	T
35.2	not assigned.unknown	MDP0000180449	 moderately similar to ( 226) AT2G02730 | Symbols:  | FUNCTIONS IN: molecular_function unknown; INVOLVED IN: biological_process unknown; LOCATED IN: endomembrane system; EXPRESSED IN: 19 plant structures; EXPRESSED DURING: 9 growth stages; CONTAINS InterPro DOMAIN/s: Protein of unknown function DUF1664 (InterPro:IPR012458); BEST Arabidopsis thaliana protein match is: bZIP family transcription factor (TAIR:AT1G27000.1); Has 86 Blast hits to 86 proteins in 10 species: Archae - 0; Bacteria - 0; Metazoa - 0; Fungi - 0; Plants - 86; Viruses - 0; Other Eukaryotes - 0 (source: NCBI BLink). | chr2:765280-767336 REVERSEmoderately similar to ( 201) loc_os05g08980 12005.m083639 protein expressed protein no original description 	T
30.3	signalling.calcium	MDP0000215758	 weakly similar to ( 140) AT2G15680 | Symbols:  | calmodulin-related protein, putative | chr2:6831024-6831587 FORWARDvery weakly similar to (79.7) CALM_CHLRE Calmodulin (CaM) - Chlamydomonas reinhardtiiweakly similar to ( 111) loc_os01g72100 12001.m13246 protein polcalcin Jun o 2, putative, expressed  no original description 	T
4.2.14	glycolysis.plastid branch.pyruvate kinase (PK)	MDP0000413866	 highly similar to ( 740) AT5G52920 | Symbols: PKP1, PKP-BETA1, PKP2 | PKP-BETA1 (PLASTIDIC PYRUVATE KINASE BETA SUBUNIT 1); pyruvate kinase | chr5:21463680-21466612 FORWARDhighly similar to ( 601) KPYG_TOBAC Pyruvate kinase isozyme G, chloroplast precursor (EC 2.7.1.40) - Nicotiana tabacum (Common tobacco)highly similar to ( 720) loc_os01g47080 12001.m10904 protein pyruvate kinase isozyme G, chloroplast precursor, putative, expressed  no original description 	T
26.10	misc.cytochrome P450	MDP0000163838	 weakly similar to ( 162) AT3G48320 | Symbols: CYP71A21 | CYP71A21; electron carrier/ heme binding / iron ion binding / monooxygenase/ oxygen binding | chr3:17891241-17892804 FORWARDweakly similar to ( 176) C71D8_SOYBN Cytochrome P450 71D8 (EC 1.14.-.-) (P450 CP7) - Glycine max (Soybean)weakly similar to ( 167) loc_os09g26970 12009.m05849 protein retrotransposon protein, putative, unclassified  no original description 	T
29.2.1.2.2.19	protein.synthesis.ribosomal protein.eukaryotic.60S subunit.L19	MDP0000194296	 moderately similar to ( 323) AT1G02780 | Symbols: emb2386 | emb2386 (embryo defective 2386); structural constituent of ribosome | chr1:608120-609391 REVERSEweakly similar to ( 112) RL19_MAIZE 60S ribosomal protein L19 (Fragment) - Zea mays (Maize)moderately similar to ( 320) loc_os03g21940 12003.m07598 protein 60S ribosomal protein L19-3, putative, expressed   no original description 	T
27.3.11	RNA.regulation of transcription.C2H2 zinc finger family	MDP0000827249	 moderately similar to ( 309) AT1G75710 | Symbols:  | zinc finger (C2H2 type) family protein | chr1:28428806-28431128 FORWARDmoderately similar to ( 317) loc_os04g59380 12004.m10814 protein nucleic acid binding protein, putative, expressed   no original description 	T
35.2	not assigned.unknown	MDP0000482160	 moderately similar to ( 224) AT1G45688 | Symbols:  | unknown protein | chr1:17191502-17192870 FORWARDmoderately similar to ( 338) loc_os06g06780 12006.m05403 protein harpin-induced protein, putative, expressed  no original description 	T
29.5.3	protein.degradation.cysteine protease	MDP0000185769	 highly similar to ( 565) AT4G39090 | Symbols: RD19, RD19A | RD19 (RESPONSIVE TO DEHYDRATION 19); cysteine-type endopeptidase/ cysteine-type peptidase | chr4:18215826-18217326 REVERSEhighly similar to ( 521) CYSP_PEA Cysteine proteinase 15A precursor (EC 3.4.22.-) (Turgor-responsive protein 15A) - Pisum sativum (Garden pea)moderately similar to ( 486) loc_os02g27030 12002.m07847 protein cysteine proteinase 1 precursor, putative, expressed  no original description 	T
35.1	not assigned.no ontology	MDP0000236521	 moderately similar to ( 444) AT5G39360 | Symbols: EDL2 | EDL2 (EID1-like 2) | chr5:15753313-15754062 REVERSEmoderately similar to ( 410) loc_os03g31310 12003.m101592 protein circadian clock coupling factor ZGT, putative, expressed   no original description 	T
29.2.3	protein.synthesis.initiation	MDP0000242464	 highly similar to ( 540) AT1G10840 | Symbols: TIF3H1 | TIF3H1; translation initiation factor | chr1:3607885-3610299 REVERSEhighly similar to ( 540) loc_os04g30780 12004.m35281 protein eukaryotic translation initiation factor 3 subunit 3, putative, expressed  no original description 	T
29.3.3	protein.targeting.chloroplast	MDP0000205699	 moderately similar to ( 336) AT1G08980 | Symbols: ATAMI1, AMI1, ATTOC64-I | AMI1 (AMIDASE 1); amidase/ hydrolase, acting on carbon-nitrogen (but not peptide) bonds / indoleacetamide hydrolase | chr1:2884455-2886430 FORWARDmoderately similar to ( 305) loc_os04g02754 12004.m05587 protein ATAMI1, putative, expressed no original description 	T
27.3.12	RNA.regulation of transcription.C3H zinc finger family	MDP0000699611	 highly similar to ( 549) AT5G58620 | Symbols:  | zinc finger (CCCH-type) family protein | chr5:23693959-23695782 FORWARDmoderately similar to ( 456) loc_os05g03760 12005.m27532 protein zinc finger transcription factor-like protein, putative, expressed  no original description 	T
35.2	not assigned.unknown	MDP0000308165	 weakly similar to ( 140) AT5G26230 | Symbols:  | unknown protein | chr5:9173517-9174542 REVERSEweakly similar to ( 103) loc_os05g32440 12005.m07477 protein expressed protein COG4907  no original description 	T
27.3.99	RNA.regulation of transcription.unclassified	MDP0000222269	 highly similar to ( 587) AT2G39830 | Symbols: DAR2 | DAR2 (DA1-RELATED PROTEIN 2); zinc ion binding | chr2:16619950-16623658 REVERSEhighly similar to ( 503) loc_os03g16090 12003.m101288 protein zinc ion binding protein, putative, expressed no original description 	T
35.1	not assigned.no ontology	MDP0000199451	 weakly similar to ( 149) AT5G40670 | Symbols:  | PQ-loop repeat family protein / transmembrane family protein | chr5:16285968-16287591 FORWARDweakly similar to ( 138) loc_os12g18110 12012.m073812 protein cystinosin, putative, expressed no original description 	T
34.3	transport.amino acids	MDP0000191097	 highly similar to ( 549) AT5G23810 | Symbols: AAP7 | AAP7; amino acid transmembrane transporter | chr5:8028461-8030138 FORWARDhighly similar to ( 555) loc_os04g39489 12004.m35579 protein AAP7, putative, expressed  no original description 	T
35.2	not assigned.unknown	MDP0000396621	 nearly identical (1226) AT5G56220 | Symbols:  | nucleoside-triphosphatase/ nucleotide binding | chr5:22754871-22757792 FORWARDnearly identical (1050) loc_os08g38970 12008.m07880 protein expressed protein   no original description 	T
35.2	not assigned.unknown	MDP0000218831	 moderately similar to ( 335) AT4G39860 | Symbols:  | unknown protein | chr4:18499909-18501472 FORWARDmoderately similar to ( 308) loc_os04g38310 12004.m101542 protein expressed protein   no original description 	T
35.1	not assigned.no ontology	MDP0000148803	 moderately similar to ( 360) AT2G39420 | Symbols:  | esterase/lipase/thioesterase family protein | chr2:16460442-16462872 FORWARDmoderately similar to ( 318) loc_os12g16180 12012.m05576 protein phospholipase, putative, expressed  no original description 	T
29.5.11.20	protein.degradation.ubiquitin.proteasom	MDP0000251636	 highly similar to ( 634) AT4G19006 | Symbols:  | 26S proteasome regulatory subunit, putative (RPN9) | chr4:10409349-10411347 REVERSEhighly similar to ( 579) loc_os01g32800 12001.m43033 protein 26S proteasome non-ATPase regulatory subunit 13, putative, expressed no original description 	T
35.1	not assigned.no ontology	MDP0000135937	 highly similar to ( 531) AT1G21880 | Symbols: LYM1 | LYM1 (LYSM DOMAIN GPI-ANCHORED PROTEIN 1 PRECURSOR) | chr1:7680689-7682526 FORWARDmoderately similar to ( 431) loc_os06g10660 12006.m05784 protein erwinia induced protein 1, putative, expressed   no original description 	T
26.7	misc.oxidases - copper, flavone etc	MDP0000191616	 weakly similar to ( 194) AT5G61510 | Symbols:  | NADP-dependent oxidoreductase, putative | chr5:24737084-24738975 REVERSEweakly similar to ( 184) loc_os03g01190 12003.m05670 protein quinone oxidoreductase, putative, expressed  no original description 	T
34.19.1	transport.Major Intrinsic Proteins.PIP	MDP0000258100	 moderately similar to ( 236) AT4G35890 | Symbols:  | La domain-containing protein | chr4:16997433-17000410 FORWARDweakly similar to ( 112) PIP26_ORYSA Probable aquaporin PIP2.6 (Plasma membrane intrinsic protein 2.6) (OsPIP2.6) - Oryza sativa (Rice)moderately similar to ( 204) loc_os09g36990 12009.m06648 protein expressed protein  PRK08026 TrbL SPT5  no original description 	T
26.28	misc.GDSL-motif lipase	MDP0000753221	 moderately similar to ( 397) AT5G14450 | Symbols:  | GDSL-motif lipase/hydrolase family protein | chr5:4658488-4660034 FORWARDmoderately similar to ( 273) EST_HEVBR Esterase precursor (EC 3.1.1.-) (Early nodule-specific protein homolog) (Latex allergen Hev b 13) - Hevea brasiliensis (Para rubber tree)moderately similar to ( 275) loc_os07g39750 12007.m079815 protein alpha-L-fucosidase 2 precursor, putative, expressed  no original description 	T
26.9	misc.glutathione S transferases	MDP0000266097	 moderately similar to ( 315) AT2G27490 | Symbols: ATCOAE | ATCOAE; ATP binding / dephospho-CoA kinase | chr2:11748087-11749009 REVERSEmoderately similar to ( 294) GSTF2_TOBAC Glutathione S-transferase APIC (EC 2.5.1.18) (GST class-phi) - Nicotiana tabacum (Common tobacco)moderately similar to ( 299) loc_os01g25880 12001.m09045 protein dephospho-CoA kinase, putative, expressed   no original description 	T
31.3	cell.cycle	MDP0000142166	 moderately similar to ( 234) AT4G03270 | Symbols: CYCD6;1 | CYCD6;1 (Cyclin D6;1); cyclin-dependent protein kinase | chr4:1432375-1433691 REVERSEweakly similar to ( 149) loc_os07g37010 12007.m29223 protein CYCD6, putative, expressed  no original description 	T
31.3.1	cell.cycle.peptidylprolyl isomerase	MDP0000703640	 moderately similar to ( 329) AT5G13120 | Symbols:  | peptidyl-prolyl cis-trans isomerase cyclophilin-type family protein | chr5:4162714-4164720 REVERSEmoderately similar to ( 245) CYPB_VICFA Peptidyl-prolyl cis-trans isomerase, chloroplast precursor (EC 5.2.1.8) (PPIase) (Rotamase) (Cyclophilin) (Cyclosporin A-binding protein) (CYP B) - Vicia faba (Broad bean)moderately similar to ( 332) loc_os05g01270 12005.m04668 protein peptidyl-prolyl cis-trans isomerase, chloroplast precursor, putative, expressed   no original description 	T
29.3.4.99	protein.targeting.secretory pathway.unspecified	MDP0000339620	 highly similar to ( 806) AT5G46630 | Symbols:  | clathrin adaptor complexes medium subunit family protein | chr5:18920580-18923060 FORWARDhighly similar to ( 809) loc_os02g46510 12002.m09688 protein AP-2 complex subunit mu, putative, expressed no original description 	T
27.3.24	RNA.regulation of transcription.MADS box transcription factor family	MDP0000060753	 moderately similar to ( 202) AT5G62165 | Symbols: AGL42 | AGL42 (AGAMOUS LIKE 42); transcription factor | chr5:24965075-24968437 FORWARDweakly similar to ( 170) MAD50_ORYSA MADS-box transcription factor 50 (OsMADS50) (Protein SUPPRESSOR OF CONSTANS OVEREXPRESSION 1-like) (OsSOC1) (Protein AGAMOUS-like 20) (RMADS208) - Oryza sativa (Rice)weakly similar to ( 160) loc_os10g39130 12010.m21952 protein MADS-box transcription factor 56, putative, expressed no original description 	T
26.4.1	misc.beta 1,3 glucan hydrolases.glucan endo-1,3-beta-glucosidase	MDP0000265527	 moderately similar to ( 334) AT4G16260 | Symbols:  | catalytic/ cation binding / hydrolase, hydrolyzing O-glycosyl compounds | chr4:9200180-9201441 REVERSEmoderately similar to ( 349) GUB_NICPL Lichenase precursor (EC 3.2.1.73) (Endo-beta-1,3-1,4 glucanase) - Nicotiana plumbaginifolia (Leadwort-leaved tobacco)moderately similar to ( 303) loc_os01g71400 12001.m13183 protein glucan endo-1,3-beta-glucosidase, acidic isoform precursor, putative   no original description 	T
28.1	DNA.synthesis/chromatin structure	MDP0000201427	 nearly identical (1142) AT4G31210 | Symbols:  | DNA topoisomerase family protein | chr4:15165280-15172534 FORWARDvery weakly similar to (63.9) ROC3_NICSY 28 kDa ribonucleoprotein, chloroplast precursor (28RNP) - Nicotiana sylvestris (Wood tobacco)highly similar to ( 977) loc_os06g16980 12006.m32047 protein DNA topoisomerase I family protein, expressed   no original description 	T
27.4	RNA.RNA binding	MDP0000201427	 nearly identical (1142) AT4G31210 | Symbols:  | DNA topoisomerase family protein | chr4:15165280-15172534 FORWARDvery weakly similar to (63.9) ROC3_NICSY 28 kDa ribonucleoprotein, chloroplast precursor (28RNP) - Nicotiana sylvestris (Wood tobacco)highly similar to ( 977) loc_os06g16980 12006.m32047 protein DNA topoisomerase I family protein, expressed   no original description 	T
35.2	not assigned.unknown	MDP0000265049	 weakly similar to ( 168) AT2G04039 | Symbols:  | unknown protein | chr2:1333464-1334457 FORWARD  no original description 	T
35.2	not assigned.unknown	MDP0000293367	 moderately similar to ( 298) AT2G35450 | Symbols:  | catalytic/ hydrolase | chr2:14903201-14905361 REVERSEmoderately similar to ( 222) loc_os04g32150 12004.m08313 protein amidohydrolase family protein, expressed  no original description 	T
35.1	not assigned.no ontology	MDP0000156411	 highly similar to ( 654) AT5G41120 | Symbols:  | esterase/lipase/thioesterase family protein | chr5:16455312-16459198 REVERSEhighly similar to ( 611) loc_os09g33530 12009.m22161 protein acyltransferase/ catalytic, putative, expressed   no original description 	T
29.5.5	protein.degradation.serine protease	MDP0000256481	 moderately similar to ( 366) AT2G29050 | Symbols: ATRBL1 | ATRBL1 (Arabidopsis thaliana Rhomboid-like 1) | chr2:12478245-12479653 FORWARDmoderately similar to ( 338) loc_os11g47840 12011.m08581 protein membrane protein, putative, expressed no original description 	T
29.5.3	protein.degradation.cysteine protease	MDP0000242006	 highly similar to ( 527) AT4G35350 | Symbols: XCP1 | XCP1 (XYLEM CYSTEINE PEPTIDASE 1); cysteine-type endopeptidase/ cysteine-type peptidase | chr4:16810529-16811578 FORWARDmoderately similar to ( 383) ORYA_ORYSA Oryzain alpha chain precursor (EC 3.4.22.-) - Oryza sativa (Rice)moderately similar to ( 470) loc_os01g73980 12001.m150856 protein xylem cysteine proteinase 2 precursor, putative, expressed  no original description 	T
33.99	development.unspecified	MDP0000312449	 moderately similar to ( 340) AT1G10200 | Symbols: WLIM1 | WLIM1; transcription factor/ zinc ion binding | chr1:3346677-3347763 REVERSEmoderately similar to ( 272) SF3_HELAN Pollen-specific protein SF3 - Helianthus annuus (Common sunflower)moderately similar to ( 320) loc_os12g32620 12012.m07062 protein pollen-specific protein SF3, putative, expressed  no original description 	T
27.4	RNA.RNA binding	MDP0000898896	 moderately similar to ( 350) AT3G21215 | Symbols:  | RNA-binding protein, putative | chr3:7442873-7445231 REVERSEmoderately similar to ( 294) loc_os08g15080 12008.m05631 protein cell wall integrity protein scw1, putative, expressed  no original description 	T
1.3.3	PS.calvin cycle.phosphoglycerate kinase	MDP0000456708	 highly similar to ( 650) AT1G79550 | Symbols: PGK | PGK (PHOSPHOGLYCERATE KINASE); phosphoglycerate kinase | chr1:29924347-29926295 REVERSEhighly similar to ( 656) PGKY_TOBAC Phosphoglycerate kinase, cytosolic (EC 2.7.2.3) - Nicotiana tabacum (Common tobacco)highly similar to ( 635) loc_os06g45710 12006.m09099 protein phosphoglycerate kinase, cytosolic, putative, expressed  no original description 	T
35.2	not assigned.unknown	MDP0000787725	 very weakly similar to (95.5) AT1G15260 | Symbols:  | unknown protein | chr1:5249834-5250370 FORWARD  no original description 	T
11.9.3.2	lipid metabolism.lipid degradation.lysophospholipases.carboxylesterase	MDP0000183429	 weakly similar to ( 185) AT4G22300 | Symbols: SOBER1 | SOBER1 (SUPPRESSOR OF AVRBST-ELICITED RESISTANCE 1); carboxylesterase | chr4:11787560-11791055 REVERSEweakly similar to ( 179) loc_os04g57370 12004.m10622 protein acyl-protein thioesterase 1, putative, expressed   no original description 	T
27.3.67	RNA.regulation of transcription.putative transcription regulator	MDP0000823144	 very weakly similar to (85.5) AT3G17460 | Symbols:  | PHD finger family protein | chr3:5976949-5977545 REVERSEvery weakly similar to (54.3) loc_os01g14330 12001.m08038 protein conserved hypothetical protein  no original description 	T
31.4	cell.vesicle transport	MDP0000254164	 nearly identical (1043) AT4G22540 | Symbols: ORP2A | ORP2A (OSBP(OXYSTEROL BINDING PROTEIN)-RELATED PROTEIN 2A); oxysterol binding / phosphoinositide binding | chr4:11860969-11863886 REVERSEhighly similar to ( 830) loc_os10g40590 12010.m06827 protein oxysterol-binding protein 1, putative, expressed   no original description 	T
26.12	misc.peroxidases	MDP0000149327	 moderately similar to ( 422) AT1G05260 | Symbols: RCI3, RCI3A | RCI3 (RARE COLD INDUCIBLE GENE 3); peroxidase | chr1:1529827-1531271 FORWARDmoderately similar to ( 288) PER1_ORYSA Peroxidase 1 precursor (EC 1.11.1.7) - Oryza sativa (Rice)moderately similar to ( 339) loc_os06g46799 12006.m09205 protein peroxidase 39 precursor, putative, expressed  no original description 	T
27.4	RNA.RNA binding	MDP0000488395	 moderately similar to ( 216) AT2G46780 | Symbols:  | RNA binding / nucleic acid binding / nucleotide binding | chr2:19229516-19231043 FORWARDvery weakly similar to (99.0) RLA1_MAIZE 60S acidic ribosomal protein P1 (L12) - Zea mays (Maize)weakly similar to ( 198) loc_os01g65550 12001.m43309 protein RNA-binding region-containing protein 1, putative, expressed   no original description 	T
29.1.1	protein.aa activation.tyrosine-tRNA ligase	MDP0000261642	 highly similar to ( 577) AT2G33840 | Symbols:  | tRNA synthetase class I (W and Y) family protein | chr2:14315011-14317329 FORWARDhighly similar to ( 561) loc_os08g05490 12008.m04692 protein tyrosyl-tRNA synthetase, putative, expressed  no original description 	T
35.2	not assigned.unknown	MDP0000875265	 moderately similar to ( 218) AT4G14746 | Symbols:  | INVOLVED IN: biological_process unknown; LOCATED IN: anchored to membrane; CONTAINS InterPro DOMAIN/s: EGF (InterPro:IPR006210); Has 201 Blast hits to 179 proteins in 41 species: Archae - 0; Bacteria - 0; Metazoa - 168; Fungi - 0; Plants - 31; Viruses - 0; Other Eukaryotes - 2 (source: NCBI BLink). | chr4:8462720-8464326 REVERSEweakly similar to ( 167) loc_os03g06580 12003.m06176 protein neurogenic locus notch protein homolog precursor, putative, expressed no original description 	T
8.1.1.2	TCA / org transformation.TCA.pyruvate DH.E2	MDP0000179082	 highly similar to ( 653) AT1G54220 | Symbols:  | dihydrolipoamide S-acetyltransferase, putative | chr1:20246460-20250208 REVERSEhighly similar to ( 605) loc_os07g22720 12007.m06556 protein dihydrolipoyllysine-residue acetyltransferase component of pyruvatedehydrogenase complex, mitochondrial precursor, putative, expressed   no original description 	T
30.2.11	signalling.receptor kinases.leucine rich repeat XI	MDP0000728755	 moderately similar to ( 425) AT5G06860 | Symbols: PGIP1, ATPGIP1 | PGIP1 (POLYGALACTURONASE INHIBITING PROTEIN 1); protein binding | chr5:2132373-2133434 FORWARDmoderately similar to ( 301) PGIP1_PHAVU Polygalacturonase inhibitor 1 precursor (Polygalacturonase-inhibiting protein) (PGIP-1) - Phaseolus vulgaris (Kidney bean) (French bean)moderately similar to ( 268) loc_os05g01370 12005.m04676 protein polygalacturonase inhibitor precursor, putative, expressed   no original description 	T
29.2.1.1.1.2.18	protein.synthesis.ribosomal protein.prokaryotic.chloroplast.50S subunit.L18	MDP0000279154	 moderately similar to ( 214) AT1G48350 | Symbols:  | ribosomal protein L18 family protein | chr1:17867269-17868215 FORWARDweakly similar to ( 169) RK18_ORYSA 50S ribosomal protein L18, chloroplast precursor (CL18) - Oryza sativa (Rice)weakly similar to ( 169) loc_os03g61260 12003.m35497 protein 50S ribosomal protein L18, chloroplast precursor, putative, expressed  no original description 	T
27.1	RNA.processing	MDP0000172287	 moderately similar to ( 392) AT1G01860 | Symbols: PFC1 | PFC1 (PALEFACE 1); mRNA (2'-O-methyladenosine-N6-)-methyltransferase | chr1:304439-306275 REVERSEmoderately similar to ( 206) loc_os02g03050 12002.m33527 protein dimethyladenosine transferase, putative, expressed   no original description 	T
34.14	transport.unspecified cations	MDP0000219395	 moderately similar to ( 219) AT1G78560 | Symbols:  | bile acid:sodium symporter family protein | chr1:29546846-29548764 REVERSEweakly similar to ( 173) loc_os04g31210 12004.m35291 protein bile acid sodium symporter/ transporter, putative, expressed  no original description 	T
35.2	not assigned.unknown	MDP0000319364	   no original description 	T
30.3	signalling.calcium	MDP0000169059	 highly similar to ( 514) AT3G52870 | Symbols:  | calmodulin-binding family protein | chr3:19593365-19595686 REVERSEmoderately similar to ( 482) loc_os12g05420 12012.m04535 protein calmodulin binding protein, putative, expressed  no original description 	T
35.2	not assigned.unknown	MDP0000623979	 moderately similar to ( 458) AT4G34260 | Symbols: FUC95A | FUC95A; 1,2-alpha-L-fucosidase | chr4:16398130-16401591 FORWARDmoderately similar to ( 438) loc_os10g19880 12010.m05016 protein large secreted protein, putative, expressed no original description 	T
35.2	not assigned.unknown	MDP0000441018	 weakly similar to ( 107) AT5G54540 | Symbols:  | FUNCTIONS IN: molecular_function unknown; INVOLVED IN: biological_process unknown; LOCATED IN: cellular_component unknown; EXPRESSED IN: 24 plant structures; EXPRESSED DURING: 15 growth stages; CONTAINS InterPro DOMAIN/s: Uncharacterised conserved protein UCP012943 (InterPro:IPR016606); BEST Arabidopsis thaliana protein match is: unknown protein (TAIR:AT4G25170.1); Has 60 Blast hits to 60 proteins in 11 species: Archae - 0; Bacteria - 2; Metazoa - 2; Fungi - 0; Plants - 56; Viruses - 0; Other Eukaryotes - 0 (source: NCBI BLink). | chr5:22156604-22157857 FORWARDweakly similar to ( 104) loc_os08g08190 12008.m04954 protein expressed protein   no original description 	T
29.6	protein.folding	MDP0000036030	 highly similar to ( 874) AT3G20050 | Symbols: ATTCP-1 | ATTCP-1; ATP binding / protein binding / unfolded protein binding | chr3:6998544-7002266 REVERSEmoderately similar to ( 226) TCPE1_AVESA T-complex protein 1 subunit epsilon (TCP-1-epsilon) (CCT-epsilon) (TCP-K19) - Avena sativa (Oat)highly similar to ( 866) loc_os04g46620 12004.m09610 protein T-complex protein 1 subunit alpha, putative, expressed  no original description 	T
29.2.1.2.1.12	protein.synthesis.ribosomal protein.eukaryotic.40S subunit.S12	MDP0000385481	 weakly similar to ( 191) AT2G32060 | Symbols:  | 40S ribosomal protein S12 (RPS12C) | chr2:13639228-13640104 REVERSEmoderately similar to ( 210) RS12_HORVU 40S ribosomal protein S12 - Hordeum vulgare (Barley)moderately similar to ( 217) loc_os07g12650 12007.m079809 protein 40S ribosomal protein S12, putative, expressed   no original description 	T
35.1	not assigned.no ontology	MDP0000235206	 moderately similar to ( 475) AT1G69040 | Symbols: ACR4 | ACR4 (ACT REPEAT 4); amino acid binding | chr1:25957843-25960079 FORWARDmoderately similar to ( 473) loc_os03g29980 12003.m34884 protein ACR4, putative, expressed  no original description 	T
35.1	not assigned.no ontology	MDP0000235102	 weakly similar to ( 186) AT5G06320 | Symbols: NHL3 | NHL3 | chr5:1931016-1931711 REVERSEweakly similar to ( 115) loc_os04g58850 12004.m10765 protein harpin inducing protein, putative, expressed no original description 	T
20.1.7	stress.biotic.PR-proteins	MDP0000309171	 weakly similar to ( 117) AT5G47280 | Symbols: ADR1-L3 | ADR1-L3 (ADR1-like 3); ATP binding / nucleoside-triphosphatase/ nucleotide binding / protein binding | chr5:19193157-19195559 FORWARDvery weakly similar to (94.7) loc_os12g39620 12012.m073872 protein disease resistance protein, putative, expressed  no original description 	T
30.4.5	signalling.phosphinositides.inositol-1,3,4-trisphosphate 5/6-kinase	MDP0000560630	 moderately similar to ( 329) AT5G16760 | Symbols:  | inositol 1,3,4-trisphosphate 5/6-kinase | chr5:5509890-5510849 FORWARDmoderately similar to ( 342) ITPK1_MAIZE Inositol-tetrakisphosphate 1-kinase 1 (EC 2.7.1.134) (Inositol-triphosphate 5/6-kinase 1) (EC 2.7.1.159) (Inositol 1,3,4-trisphosphate 5/6-kinase 1) (Ins(1,3,4)P(3) 5/6-kinase 1) (ZmIpk) (Low phytic acid protein 2) - Zea mays (Maize)moderately similar to ( 347) loc_os10g42550 12010.m07008 protein inositol-tetrakisphosphate 1-kinase 1, putative, expressed  COG4907  no original description 	T
35.2	not assigned.unknown	MDP0000125575	 highly similar to ( 534) AT1G77620 | Symbols:  | nucleoside-triphosphatase/ nucleotide binding | chr1:29167934-29172867 REVERSEmoderately similar to ( 367) loc_os03g15580 12003.m06995 protein AT hook motif family protein, expressed  no original description 	T
30.2.11	signalling.receptor kinases.leucine rich repeat XI	MDP0000493266	 highly similar to ( 746) AT3G47090 | Symbols:  | leucine-rich repeat transmembrane protein kinase, putative | chr3:17341512-17344645 REVERSEmoderately similar to ( 358) RPK1_IPONI Receptor-like protein kinase precursor (EC 2.7.11.1) - Ipomoea nil (Japanese morning glory) (Pharbitis nil)highly similar to ( 714) loc_os11g36180 12011.m07470 protein receptor-like protein kinase 5 precursor, putative  no original description 	T
35.2	not assigned.unknown	MDP0000301118	 moderately similar to ( 427) AT5G41950 | Symbols:  | binding | chr5:16785825-16789360 FORWARDmoderately similar to ( 430) loc_os03g02270 12003.m05769 protein expressed protein  no original description 	T
35.2	not assigned.unknown	MDP0000151375	 very weakly similar to (67.4) AT4G24190 | Symbols: SHD | SHD (SHEPHERD); ATP binding / unfolded protein binding | chr4:12551902-12555851 REVERSEvery weakly similar to (86.3) ENPL_CATRO Endoplasmin homolog precursor (GRP94 homolog) - Catharanthus roseus (Rosy periwinkle) (Madagascar periwinkle)very weakly similar to (69.3) loc_os06g50300 12006.m31934 protein endoplasmin homolog precursor, putative, expressed no original description 	T
29.5.11.4.2	protein.degradation.ubiquitin.E3.RING	MDP0000763211	 very weakly similar to (88.6) AT5G20885 | Symbols:  | zinc finger (C3HC4-type RING finger) family protein | chr5:7084133-7084663 REVERSEvery weakly similar to (54.3) EL5_ORYSA E3 ubiquitin-protein ligase EL5 (EC 6.3.2.-) - Oryza sativa (Rice)very weakly similar to (85.1) loc_os08g06090 12008.m04748 protein RING zinc finger protein-like, putative, expressed no original description 	T
35.2	not assigned.unknown	MDP0000241335	 moderately similar to ( 415) AT5G10060 | Symbols:  | FUNCTIONS IN: molecular_function unknown; INVOLVED IN: biological_process unknown; LOCATED IN: endomembrane system; CONTAINS InterPro DOMAIN/s: Protein of unknown function DUF618 (InterPro:IPR006903), Regulation of nuclear pre-mRNA protein (InterPro:IPR006569), ENTH/VHS (InterPro:IPR008942); BEST Arabidopsis thaliana protein match is: unknown protein (TAIR:AT5G65180.1); Has 4531 Blast hits to 4161 proteins in 412 species: Archae - 14; Bacteria - 404; Metazoa - 1999; Fungi - 666; Plants - 262; Viruses - 36; Other Eukaryotes - 1150 (source: NCBI BLink). | chr5:3145994-3148049 REVERSEmoderately similar to ( 348) loc_os04g54420 12004.m10333 protein expressed protein  no original description 	T
26.10	misc.cytochrome P450	MDP0000292868	 highly similar to ( 587) AT3G14660 | Symbols: CYP72A13 | CYP72A13; electron carrier/ heme binding / iron ion binding / monooxygenase/ oxygen binding | chr3:4924960-4926911 FORWARDhighly similar to ( 582) C72A1_CATRO Cytochrome P450 72A1 (EC 1.3.3.9) (CYPLXXII) (Secologanin synthase) (SLS) - Catharanthus roseus (Rosy periwinkle) (Madagascar periwinkle)highly similar to ( 515) loc_os01g43774 12001.m10639 protein cytochrome P450 72A1, putative, expressed  no original description 	T
27.1.19	RNA.processing.ribonucleases	MDP0000248778	 moderately similar to ( 214) AT3G20390 | Symbols:  | endoribonuclease L-PSP family protein | chr3:7110227-7111695 REVERSEmoderately similar to ( 215) loc_os07g33240 12007.m07585 protein expressed protein no original description 	T
29.5.11.1	protein.degradation.ubiquitin.ubiquitin	MDP0000880194	 moderately similar to ( 253) AT2G47110 | Symbols: UBQ6 | UBQ6; protein binding | chr2:19344701-19345174 FORWARDweakly similar to ( 150) UBIQ_WHEAT Ubiquitin - Triticum aestivum (Wheat)moderately similar to ( 246) loc_os01g22490 12001.m08729 protein 40S ribosomal protein S27a, putative, expressed no original description 	T
29.4	protein.postranslational modification	MDP0000932944	 moderately similar to ( 409) AT1G73500 | Symbols: ATMKK9, MKK9 | MKK9 (MAP KINASE KINASE 9); MAP kinase kinase/ kinase/ protein kinase activator | chr1:27639419-27640351 REVERSEmoderately similar to ( 204) M2K1_ORYSA Mitogen-activated protein kinase kinase 1 (EC 2.7.12.2) (MAP kinase kinase 1) (MAPKK1) (OsMEK1) - Oryza sativa (Rice)moderately similar to ( 287) loc_os03g12390 12003.m06689 protein OsMKK10-2 - putative MAPKK based on amino acid sequence homology, expressed   no original description 	T
27.3.25	RNA.regulation of transcription.MYB domain transcription factor family	MDP0000250597	 moderately similar to ( 244) AT4G09460 | Symbols: ATMYB6 | ATMYB6; DNA binding / transcription factor | chr4:5993019-5994038 FORWARDmoderately similar to ( 233) MYB38_MAIZE Myb-related protein Zm38 - Zea mays (Maize)moderately similar to ( 241) loc_os09g36730 12009.m06622 protein myb-related protein Hv1, putative, expressed no original description 	T
33.99	development.unspecified	MDP0000868556	 moderately similar to ( 335) AT2G17040 | Symbols: anac036 | anac036 (Arabidopsis NAC domain containing protein 36); transcription factor | chr2:7407123-7408120 FORWARDweakly similar to ( 163) NAC68_ORYSA NAC domain-containing protein 68 (ONAC068) - Oryza sativa (Rice)moderately similar to ( 280) loc_os03g04070 12003.m05939 protein NAC domain-containing protein 68, putative, expressed  no original description 	T
35.2	not assigned.unknown	MDP0000498703	 weakly similar to ( 121) AT5G65030 | Symbols:  | unknown protein | chr5:25975697-25976305 REVERSEvery weakly similar to (52.0) loc_os04g38320 12004.m08822 protein expressed protein   no original description 	T
29.4	protein.postranslational modification	MDP0000241811	 nearly identical (1071) AT3G25800 | Symbols: PDF1, PR 65, PP2AA2 | PP2AA2 (PROTEIN PHOSPHATASE 2A SUBUNIT A2); protein phosphatase type 2A regulator | chr3:9423036-9425783 REVERSEhighly similar to ( 680) 2AAA_PEA Protein phosphatase PP2A regulatory subunit A (PR65) (Fragment) - Pisum sativum (Garden pea)nearly identical (1052) loc_os09g07510 12009.m22048 protein serine/threonine-protein phosphatase 2A 65 kDa regulatory subunit Abeta isoform, putative, expressed   no original description 	T
29.5.11.4.3.2	protein.degradation.ubiquitin.E3.SCF.FBOX	MDP0000158085	 weakly similar to ( 199) AT1G21410 | Symbols: SKP2A | SKP2A | chr1:7497479-7499386 FORWARDweakly similar to ( 168) loc_os05g35110 12005.m07741 protein F-box/LRR-repeat protein 2, putative, expressed  no original description 	T
35.1	not assigned.no ontology	MDP0000351627	 weakly similar to ( 165) AT4G23930 | Symbols:  | FUNCTIONS IN: molecular_function unknown; INVOLVED IN: biological_process unknown; LOCATED IN: endomembrane system; EXPRESSED IN: 11 plant structures; EXPRESSED DURING: 6 growth stages; CONTAINS InterPro DOMAIN/s: Harpin-induced 1 (InterPro:IPR010847); BEST Arabidopsis thaliana protein match is: proline-rich family protein (TAIR:AT1G64450.1); Has 183 Blast hits to 177 proteins in 15 species: Archae - 0; Bacteria - 0; Metazoa - 0; Fungi - 0; Plants - 183; Viruses - 0; Other Eukaryotes - 0 (source: NCBI BLink). | chr4:12436131-12436694 REVERSEweakly similar to ( 144) loc_os05g14880 12005.m05899 protein expressed protein   no original description 	T
29.2.1.2.2.510	protein.synthesis.ribosomal protein.eukaryotic.60S subunit.L10A	MDP0000258483	 moderately similar to ( 309) AT5G22440 | Symbols:  | 60S ribosomal protein L10A (RPL10aC) | chr5:7435328-7436486 REVERSEmoderately similar to ( 226) RL10A_CHLRE 60S ribosomal protein L10a - Chlamydomonas reinhardtiimoderately similar to ( 316) loc_os01g64090 12001.m43287 protein 60S ribosomal protein L10a-1, putative, expressed  no original description 	T
35.1	not assigned.no ontology	MDP0000189871	 moderately similar to ( 257) AT1G74440 | Symbols:  | unknown protein | chr1:27979208-27980371 REVERSEmoderately similar to ( 229) loc_os02g36870 12002.m33734 protein YGL010w-like protein, putative, expressed  no original description 	T
31.4	cell.vesicle transport	MDP0000869097	 moderately similar to ( 296) AT5G61210 | Symbols: SNAP33, ATSNAP33, SNP33, ATSNAP33B | SNAP33 (SOLUBLE N-ETHYLMALEIMIDE-SENSITIVE FACTOR ADAPTOR PROTEIN 33); SNAP receptor/ protein binding | chr5:24624027-24625366 FORWARDmoderately similar to ( 254) loc_os02g24080 12002.m33379 protein SNAP25 homologous protein SNAP30, putative, expressed  no original description 	T
35.2	not assigned.unknown	MDP0000327491	 moderately similar to ( 384) AT4G25550 | Symbols:  | protein binding | chr4:13048519-13050873 FORWARDmoderately similar to ( 383) loc_os04g58640 12004.m35057 protein cleavage and polyadenylation specificity factor 5, putative, expressed MSP1_C  no original description 	T
35.2	not assigned.unknown	MDP0000806699	 moderately similar to ( 421) AT4G13750 | Symbols:  | ATP binding | chr4:7975191-7987558 FORWARDmoderately similar to ( 363) loc_os03g58910 12003.m35467 protein expressed protein no original description 	T
30.1	signalling.in sugar and nutrient physiology	MDP0000302137	 highly similar to ( 996) AT1G42540 | Symbols: ATGLR3.3, GLR3.3 | ATGLR3.3; intracellular ligand-gated ion channel | chr1:15973489-15976703 FORWARDhighly similar to ( 917) GLR31_ORYSA Glutamate receptor 3.1 precursor (Ligand-gated ion channel 3.1) - Oryza sativa (Rice)highly similar to ( 917) loc_os04g49570 12004.m101438 protein glutamate receptor 3.3 precursor, putative, expressed   no original description 	T
7.1.3	OPP.oxidative PP.6-phosphogluconate dehydrogenase	MDP0000250354	 highly similar to ( 852) AT3G02360 | Symbols:  | 6-phosphogluconate dehydrogenase family protein | chr3:482498-483958 FORWARDhighly similar to ( 820) loc_os06g02144 12006.m091574 protein 6-phosphogluconate dehydrogenase, decarboxylating, putative, expressed  no original description 	T
17.5.1	hormone metabolism.ethylene.synthesis-degradation	MDP0000503940	 moderately similar to ( 477) AT3G11180 | Symbols:  | oxidoreductase, 2OG-Fe(II) oxygenase family protein | chr3:3504249-3506688 FORWARDmoderately similar to ( 233) LDOX_MALDO Leucoanthocyanidin dioxygenase (EC 1.14.11.19) (LDOX) (Leucocyanidin oxygenase) (Leucoanthocyanidin hydroxylase) (Anthocyanidin synthase) - Malus domestica (Apple) (Malus sylvestris)moderately similar to ( 439) loc_os05g03640 12005.m04898 protein flavonol synthase/flavanone 3-hydroxylase, putative, expressed  no original description 	T
35.1.5	not assigned.no ontology.pentatricopeptide (PPR) repeat-containing protein	MDP0000284023	 moderately similar to ( 310) AT1G12250 | Symbols:  | thylakoid lumenal protein-related | chr1:4159623-4161269 FORWARDmoderately similar to ( 308) loc_os02g42960 12002.m09331 protein expressed protein  no original description 	T
10.1.3	cell wall.precursor synthesis.AXS	MDP0000265798	 highly similar to ( 656) AT1G08200 | Symbols: AXS2 | AXS2 (UDP-D-APIOSE/UDP-D-XYLOSE SYNTHASE 2); UDP-glucuronate decarboxylase | chr1:2574259-2576609 REVERSEvery weakly similar to (58.9) DFRA_DIACA Dihydroflavonol-4-reductase (EC 1.1.1.219) (DFR) (Dihydrokaempferol 4-reductase) - Dianthus caryophyllus (Carnation) (Clove pink)highly similar to ( 624) loc_os01g73790 12001.m13406 protein bifunctional polymyxin resistance arnA protein, putative, expressed  no original description 	T
33.99	development.unspecified	MDP0000747592	 weakly similar to ( 119) AT2G42840 | Symbols: PDF1 | PDF1 (PROTODERMAL FACTOR 1) | chr2:17826327-17827426 REVERSEvery weakly similar to ( 100) loc_os01g52690 12001.m11442 protein DNA-directed RNA polymerase II largest subunit, putative, expressed   no original description 	T
35.2	not assigned.unknown	MDP0000260705	 weakly similar to ( 196) AT3G57090 | Symbols: BIGYIN, FIS1A | BIGYIN; binding | chr3:21128627-21129981 FORWARDweakly similar to ( 183) loc_os03g24060 12003.m07752 protein mitochondria fission 1 protein, putative, expressed  no original description 	T
13.2.6.2	amino acid metabolism.degradation.aromatic aa.tyrosine	MDP0000148049	 moderately similar to ( 253) AT1G12050 | Symbols:  | fumarylacetoacetase, putative | chr1:4072904-4075856 FORWARDmoderately similar to ( 240) loc_os02g10310 12002.m100035 protein fumarylacetoacetase, putative, expressed no original description 	T
29.2.1.2.2.3	protein.synthesis.ribosomal protein.eukaryotic.60S subunit.L3	MDP0000897417	 highly similar to ( 655) AT1G61580 | Symbols: RPL3B, ARP2 | RPL3B (R-PROTEIN L3 B); structural constituent of ribosome | chr1:22720833-22722402 REVERSEhighly similar to ( 646) RL3_ORYSJ 60S ribosomal protein L3 OS=Oryza sativa subsp. japonica GN=RPL3 PE=2 SV=2highly similar to ( 646) loc_os12g07010 12012.m04691 protein 60S ribosomal protein L3, putative, expressed  no original description 	T
33.99	development.unspecified	MDP0000871412	 weakly similar to ( 123) AT5G20700 | Symbols:  | senescence-associated protein-related | chr5:7006178-7007003 REVERSEvery weakly similar to (77.0) loc_os02g07820 12002.m06128 protein expressed protein  no original description 	T
20.2.1	stress.abiotic.heat	MDP0000833769	 weakly similar to ( 167) AT4G13830 | Symbols: J20 | J20 (DNAJ-LIKE 20); heat shock protein binding | chr4:8011518-8012577 FORWARDvery weakly similar to (53.5) DNJH_CUCSA DnaJ protein homolog (DNAJ-1) - Cucumis sativus (Cucumber)weakly similar to ( 147) loc_os01g01160 12001.m06763 protein chaperone protein dnaJ 20, chloroplast precursor, putative, expressed  no original description 	T
1.1.1.1	PS.lightreaction.photosystem II.LHC-II	MDP0000757636	 moderately similar to ( 449) AT5G01530 | Symbols:  | chlorophyll A-B binding protein CP29 (LHCB4) | chr5:209084-210243 FORWARDmoderately similar to ( 248) CB29_CHLRE Chlorophyll a-b binding protein CP29 - Chlamydomonas reinhardtiimoderately similar to ( 438) loc_os07g37240 12007.m29225 protein expressed protein  no original description 	T
34.21	transport.calcium	MDP0000413871	 moderately similar to ( 215) AT3G51860 | Symbols: CAX3, ATHCX1, CAX1-LIKE, ATCAX3 | CAX3 (CATION EXCHANGER 3); calcium:cation antiporter/ calcium:hydrogen antiporter/ cation:cation antiporter | chr3:19239458-19242519 FORWARDweakly similar to ( 184) CAX1A_ORYSA Vacuolar cation/proton exchanger 1a (Ca(2+)/H(+) exchanger 1a) (OsCAX1a) - Oryza sativa (Rice)weakly similar to ( 184) loc_os01g37690 12001.m42663 protein vacuolar cation/proton exchanger 1a, putative, expressed   no original description 	T
35.2	not assigned.unknown	MDP0000264673	   no original description 	T
27.3.80	RNA.regulation of transcription.zf-HD	MDP0000372694	 weakly similar to ( 119) AT2G02540 | Symbols: ATHB21, ZFHD4 | ATHB21 (ARABIDOPSIS THALIANA HOMEOBOX PROTEIN 21); DNA binding / transcription activator/ transcription factor | chr2:684302-685234 FORWARDweakly similar to ( 108) loc_os09g29130 12009.m06064 protein ZF-HD protein dimerisation region containing protein, expressed  no original description 	T
30.2.11	signalling.receptor kinases.leucine rich repeat XI	MDP0000304719	 moderately similar to ( 451) AT1G45616 | Symbols: AtRLP6 | AtRLP6 (Receptor Like Protein 6); protein binding | chr1:17183550-17186534 REVERSEmoderately similar to ( 201) RPK1_IPONI Receptor-like protein kinase precursor (EC 2.7.11.1) - Ipomoea nil (Japanese morning glory) (Pharbitis nil)highly similar to ( 546) loc_os04g40440 12004.m09026 protein expressed protein  no original description 	T
29.2.1.1.1.1.9	protein.synthesis.ribosomal protein.prokaryotic.chloroplast.30S subunit.S9	MDP0000164974	 moderately similar to ( 283) AT1G74970 | Symbols: RPS9, TWN3 | RPS9 (RIBOSOMAL PROTEIN S9); structural constituent of ribosome | chr1:28157761-28159202 REVERSEmoderately similar to ( 278) RR9_SPIOL 30S ribosomal protein S9, chloroplast precursor (Fragment) - Spinacia oleracea (Spinach)moderately similar to ( 227) loc_os03g55930 12003.m10503 protein 30S ribosomal protein S9, chloroplast precursor, putative, expressed   no original description 	T
30.11	signalling.light	MDP0000773006	 moderately similar to ( 271) AT4G08920 | Symbols: CRY1, BLU1, HY4, OOP2, ATCRY1 | CRY1 (CRYPTOCHROME 1); ATP binding / blue light photoreceptor/ protein homodimerization/ protein kinase | chr4:5724260-5726905 FORWARDweakly similar to ( 105) PHR1_SINAL Deoxyribodipyrimidine photo-lyase (EC 4.1.99.3) (DNA photolyase) (Photoreactivating enzyme) - Sinapis alba (White mustard) (Brassica hirta)moderately similar to ( 253) loc_os04g37920 12004.m35145 protein cryptochrome 1 apoprotein, putative, expressed  no original description 	T
11.9.4.13	lipid metabolism.lipid degradation.beta-oxidation.acyl CoA reductase	MDP0000298128	 moderately similar to ( 217) AT4G33790 | Symbols: CER4, G7, FAR3 | CER4 (ECERIFERUM 4); fatty acyl-CoA reductase (alcohol-forming)/ oxidoreductase, acting on the CH-CH group of donors | chr4:16204325-16207891 REVERSEweakly similar to ( 151) loc_os08g20200 12008.m06087 protein male sterility protein 2, putative, expressed  no original description 	T
20.1	stress.biotic	MDP0000153378	 weakly similar to ( 181) AT4G31310 | Symbols:  | avirulence-responsive protein-related / avirulence induced gene (AIG) protein-related | chr4:15191325-15192337 FORWARDmoderately similar to ( 209) loc_os06g48960 12006.m09422 protein AIG2-like protein, putative, expressed  no original description 	T
34.3	transport.amino acids	MDP0000020317	 highly similar to ( 835) AT2G21050 | Symbols:  | amino acid permease, putative | chr2:9034289-9036439 FORWARDhighly similar to ( 819) LAX5_MEDTR Auxin transporter-like protein 5 (AUX1-like protein 5) (MtLAX5) - Medicago truncatula (Barrel medic)highly similar to ( 778) loc_os03g14080 12003.m06851 protein auxin transporter-like protein 3, putative, expressed  no original description 	T
35.2	not assigned.unknown	MDP0000164042	   no original description 	T
29.2.1.2.2.36	protein.synthesis.ribosomal protein.eukaryotic.60S subunit.L36	MDP0000327175	 weakly similar to ( 172) AT5G02450 | Symbols:  | 60S ribosomal protein L36 (RPL36C) | chr5:533501-534394 FORWARDweakly similar to ( 143) RL36_DAUCA 60S ribosomal protein L36 - Daucus carota (Carrot)weakly similar to ( 192) loc_os05g38520 12005.m27873 protein 60S ribosomal protein L36-2, putative, expressed   no original description 	T
29.2.1.1.1.2.12	protein.synthesis.ribosomal protein.prokaryotic.chloroplast.50S subunit.L12	MDP0000796252	 weakly similar to ( 155) AT3G27830 | Symbols: RPL12-A, RPL12 | RPL12-A (RIBOSOMAL PROTEIN L12-A); structural constituent of ribosome | chr3:10318576-10319151 FORWARDweakly similar to ( 141) RK12_SPIOL 50S ribosomal protein L12, chloroplast precursor (CL12) - Spinacia oleracea (Spinach)very weakly similar to (97.8) loc_os01g47330 12001.m10928 protein 50S ribosomal protein L12-1, chloroplast precursor, putative, expressed  no original description 	T
17.4.1	hormone metabolism.cytokinin.synthesis-degradation	MDP0000279125	 moderately similar to ( 478) AT5G21482 | Symbols: CKX7, ATCKX5 | CKX7 (CYTOKININ OXIDASE 7); cytokinin dehydrogenase/ oxidoreductase | chr5:7226842-7230052 FORWARDmoderately similar to ( 260) CKX1_MAIZE Cytokinin dehydrogenase 1 precursor (EC 1.5.99.12) (Cytokinin oxidase 1) (CKO 1) (COX 1) (ZmCKX1) - Zea mays (Maize)moderately similar to ( 384) loc_os08g35860 12008.m07575 protein cytokinin dehydrogenase 7, putative, expressed no original description 	T
33.3	development.squamosa promoter binding like (SPL)	MDP0000249364	 moderately similar to ( 335) AT1G62940 | Symbols: ACOS5 | ACOS5 (ACYL-COA SYNTHETASE 5); 4-coumarate-CoA ligase/ long-chain-fatty-acid-CoA ligase/ medium-chain-fatty-acid-CoA ligase | chr1:23310554-23312747 FORWARDweakly similar to ( 114) LG1_MAIZE LIGULELESS1 protein - Zea mays (Maize)moderately similar to ( 278) loc_os04g24530 12004.m101489 protein 4-coumarate--CoA ligase 2, putative, expressed  no original description 	T
20.1	stress.biotic	MDP0000317557	 moderately similar to ( 415) AT3G07040 | Symbols: RPM1, RPS3 | RPM1 (RESISTANCE TO P. SYRINGAE PV MACULICOLA 1); nucleotide binding / protein binding | chr3:2226244-2229024 REVERSEmoderately similar to ( 337) loc_os09g09490 12009.m04213 protein disease resistance protein RPM1, putative, expressed  no original description 	T
35.2	not assigned.unknown	MDP0000720790	 weakly similar to ( 106) AT3G04310 | Symbols:  | unknown protein | chr3:1141581-1142528 REVERSEvery weakly similar to (74.3) loc_os01g48820 12001.m11071 protein expressed protein  no original description 	T
29.3.2	protein.targeting.mitochondria	MDP0000261109	 moderately similar to ( 255) AT2G42210 | Symbols: ATOEP16-3, OEP16-3 | mitochondrial import inner membrane translocase subunit Tim17/Tim22/Tim23 family protein | chr2:17590642-17591591 FORWARDmoderately similar to ( 209) loc_os02g48610 12002.m77845 protein protein translocase/ protein transporter, putative, expressed no original description 	T
30.3	signalling.calcium	MDP0000168498	 moderately similar to ( 261) AT2G43290 | Symbols: MSS3 | MSS3 (multicopy suppressors of snf4 deficiency in yeast 3); calcium ion binding | chr2:17991308-17991955 REVERSEweakly similar to ( 112) CALM_CHLRE Calmodulin (CaM) - Chlamydomonas reinhardtiimoderately similar to ( 204) loc_os12g12730 12012.m05246 protein EF hand family protein, expressed   no original description 	T
35.1.41	not assigned.no ontology.hydroxyproline rich proteins	MDP0000834410	 very weakly similar to (92.0) AT5G65660 | Symbols:  | hydroxyproline-rich glycoprotein family protein | chr5:26245444-26246245 REVERSE no original description 	T
35.2	not assigned.unknown	MDP0000217829	  no original description 	T
35.2	not assigned.unknown	MDP0000249881	 highly similar to ( 828) AT1G79150 | Symbols:  | binding | chr1:29772716-29777874 REVERSEhighly similar to ( 738) loc_os06g30320 12006.m07576 protein nucleolar complex-associated protein, expressed  no original description 	T
35.2	not assigned.unknown	MDP0000238860	 very weakly similar to (60.5) AT5G35732 | Symbols:  | unknown protein | chr5:13897961-13898263 FORWARD no original description 	T
29.3.3	protein.targeting.chloroplast	MDP0000273683	 moderately similar to ( 396) AT2G01110 | Symbols: APG2, UNE3, PGA2, TATC | APG2 (ALBINO AND PALE GREEN 2); proton motive force dependent protein transmembrane transporter | chr2:83786-85088 REVERSEmoderately similar to ( 390) loc_os01g31680 12001.m43024 protein expressed protein no original description 	T
20.1	stress.biotic	MDP0000255700	 very weakly similar to (56.6) AT1G50180 | Symbols:  | disease resistance protein (CC-NBS-LRR class), putative | chr1:18584235-18587136 FORWARDvery weakly similar to (71.6) loc_os08g43050 12008.m08282 protein disease resistance protein RGA2, putative, expressed no original description 	T
35.2	not assigned.unknown	MDP0000803773	 weakly similar to ( 170) AT1G29195 | Symbols:  | unknown protein | chr1:10202673-10203254 REVERSEweakly similar to ( 152) loc_os01g55370 12001.m11696 protein expressed protein no original description 	T
20.2.3	stress.abiotic.drought/salt	MDP0000201702	 nearly identical (1189) AT5G19690 | Symbols: STT3A | STT3A (STAUROSPORIN AND TEMPERATURE SENSITIVE 3-LIKE A); oligosaccharyl transferase | chr5:6652649-6658214 FORWARDnearly identical (1159) loc_os05g44360 12005.m27627 protein oligosaccharyl transferase STT3 subunit, putative, expressed PRK07003  no original description 	T
34.22	transport.cyclic nucleotide or calcium regulated channels	MDP0000910032	 highly similar to ( 976) AT5G15410 | Symbols: DND1, ATCNGC2, CNGC2 | DND1 (DEFENSE NO DEATH 1); calcium channel/ calmodulin binding / cation channel/ cyclic nucleotide binding / intracellular cAMP activated cation channel/ intracellular cyclic nucleotide activated cation channel/ inward rectifier potassium channel | chr5:5003460-5006763 REVERSEhighly similar to ( 877) loc_os03g55100 12003.m10426 protein cyclic nucleotide-gated ion channel 2, putative, expressed   no original description 	T
29.4.1.57	protein.postranslational modification.kinase.receptor like cytoplasmatic kinase VII	MDP0000599231	 highly similar to ( 617) AT1G79620 | Symbols:  | leucine-rich repeat transmembrane protein kinase, putative | chr1:29957633-29962174 REVERSEmoderately similar to ( 243) NORK_MEDTR Nodulation receptor kinase precursor (EC 2.7.11.1) (Does not make infections protein 2) (Symbiosis receptor-like kinase) (MtSYMRK) - Medicago truncatula (Barrel medic)highly similar to ( 591) loc_os03g21230 12003.m07529 protein protein kinase, putative, expressed  no original description 	T
35.2	not assigned.unknown	MDP0000314684	 moderately similar to ( 263) AT5G45540 | Symbols:  | unknown protein | chr5:18458294-18460705 REVERSEmoderately similar to ( 371) loc_os11g42030 12011.m08039 protein expressed protein   no original description 	T
16.8.3.1	secondary metabolism.flavonoids.dihydroflavonols.dihydroflavonol 4-reductase	MDP0000119035	 moderately similar to ( 240) AT2G23910 | Symbols:  | cinnamoyl-CoA reductase-related | chr2:10177902-10179789 FORWARDweakly similar to ( 108) DFRA_MAIZE Dihydroflavonol-4-reductase (EC 1.1.1.219) (DFR) (Dihydrokaempferol 4-reductase) - Zea mays (Maize)moderately similar to ( 296) loc_os10g42620 12010.m07015 protein dihydroflavonol-4-reductase, putative, expressed  no original description 	T
29.5.4	protein.degradation.aspartate protease	MDP0000943304	 highly similar to ( 504) AT1G03220 | Symbols:  | extracellular dermal glycoprotein, putative / EDGP, putative | chr1:787143-788444 FORWARDmoderately similar to ( 286) 7SB1_SOYBN Basic 7S globulin precursor (Bg) (SBg7S) [Contains: Basic 7S globulin high kDa subunit; Basic 7S globulin low kDa subunit] - Glycine max (Soybean)moderately similar to ( 421) loc_os05g33400 12005.m07572 protein basic 7S globulin precursor, putative, expressed  no original description 	T
21.1	redox.thioredoxin	MDP0000288395	 moderately similar to ( 290) AT1G08570 | Symbols: ACHT4 | ACHT4 (ATYPICAL CYS HIS RICH THIOREDOXIN 4); oxidoreductase, acting on sulfur group of donors, disulfide as acceptor | chr1:2713604-2714312 FORWARDvery weakly similar to (53.9) TRXF_MESCR Thioredoxin F-type, chloroplast precursor (TRX-F) - Mesembryanthemum crystallinum (Common ice plant)moderately similar to ( 266) loc_os03g21000 12003.m35200 protein thioredoxin-like 1, putative, expressed   no original description 	T
21.4	redox.glutaredoxins	MDP0000287066	 highly similar to ( 697) AT1G72280 | Symbols: AERO1 | AERO1 (Arabidopsis endoplasmic reticulum oxidoreductins 1); FAD binding / electron carrier/ oxidoreductase, acting on sulfur group of donors, disulfide as acceptor / protein binding | chr1:27212039-27214506 REVERSEvery weakly similar to (68.2) GLRX_RICCO Glutaredoxin - Ricinus communis (Castor bean)highly similar to ( 696) loc_os03g52340 12003.m35402 protein endoplasmic oxidoreductin-1 precursor, putative, expressed   no original description 	T
35.2	not assigned.unknown	MDP0000255057	 highly similar to ( 754) AT5G48390 | Symbols: ATZIP4 | ATZIP4; binding | chr5:19612078-19615363 FORWARDhighly similar to ( 536) loc_os09g07380 12009.m04007 protein ABI3-interacting protein 2, putative, expressed   no original description 	T
29.2.1.2.2.35	protein.synthesis.ribosomal protein.eukaryotic.60S subunit.L35	MDP0000134764	 weakly similar to ( 147) AT2G39390 | Symbols:  | 60S ribosomal protein L35 (RPL35B) | chr2:16450803-16451762 REVERSEvery weakly similar to (71.2) RL35_WHEAT 60S ribosomal protein L35 - Triticum aestivum (Wheat)weakly similar to ( 140) loc_os04g30730 12004.m35547 protein 60S ribosomal protein L35, putative, expressed  no original description 	T
27.1.1	RNA.processing.splicing	MDP0000273752	 moderately similar to ( 248) AT1G02840 | Symbols: SR1, ATSRP34, SRP34 | SR1; RNA binding / nucleic acid binding / nucleotide binding | chr1:626918-629583 FORWARDmoderately similar to ( 263) loc_os05g30140 12005.m64232 protein pre-mRNA-splicing factor SF2, putative, expressed   no original description 	T
35.2	not assigned.unknown	MDP0000330403	 moderately similar to ( 205) AT2G32650 | Symbols:  | FUNCTIONS IN: molecular_function unknown; INVOLVED IN: biological_process unknown; CONTAINS InterPro DOMAIN/s: Cupin, RmlC-type (InterPro:IPR011051), Protein of unknown function DUF861, cupin-3 (InterPro:IPR008579), RmlC-like jelly roll fold (InterPro:IPR014710); BEST Arabidopsis thaliana protein match is: PTAC18 (PLASTID TRANSCRIPTIONALLY ACTIVE 18) (TAIR:AT2G32180.1); Has 169 Blast hits to 169 proteins in 46 species: Archae - 0; Bacteria - 71; Metazoa - 0; Fungi - 0; Plants - 81; Viruses - 0; Other Eukaryotes - 17 (source: NCBI BLink). | chr2:13851547-13851966 FORWARDweakly similar to ( 140) loc_os02g54360 12002.m10465 protein expressed protein  no original description 	T
34.2	transport.sugars	MDP0000138182	 highly similar to ( 622) AT5G59250 | Symbols:  | sugar transporter family protein | chr5:23903958-23906853 FORWARDweakly similar to ( 127) STC_RICCO Sugar carrier protein C - Ricinus communis (Castor bean)moderately similar to ( 485) loc_os10g42830 12010.m07035 protein D-xylose-proton symporter, putative, expressed  no original description 	T
35.2	not assigned.unknown	MDP0000187697	 moderately similar to ( 409) AT2G16760 | Symbols:  | FUNCTIONS IN: molecular_function unknown; LOCATED IN: endoplasmic reticulum; EXPRESSED IN: 10 plant structures; EXPRESSED DURING: 6 growth stages; CONTAINS InterPro DOMAIN/s: Six-bladed beta-propeller, TolB-like (InterPro:IPR011042); BEST Arabidopsis thaliana protein match is: unknown protein (TAIR:AT2G47370.1); Has 78 Blast hits to 78 proteins in 24 species: Archae - 0; Bacteria - 32; Metazoa - 0; Fungi - 0; Plants - 42; Viruses - 0; Other Eukaryotes - 4 (source: NCBI BLink). | chr2:7275739-7277122 FORWARDmoderately similar to ( 268) loc_os03g19452 12003.m78857 protein expressed protein  no original description 	T
33.99	development.unspecified	MDP0000560731	 very weakly similar to (85.1) AT1G72410 | Symbols:  | COP1-interacting protein-related | chr1:27251949-27256246 REVERSEmoderately similar to ( 289) loc_os03g21270 12003.m35204 protein expressed protein no original description 	T
26.4	misc.beta 1,3 glucan hydrolases	MDP0000319928	 weakly similar to ( 138) AT5G35740 | Symbols:  | glycosyl hydrolase family protein 17 | chr5:13910572-13911095 REVERSEvery weakly similar to (71.6) E13B_WHEAT Glucan endo-1,3-beta-glucosidase precursor (EC 3.2.1.39) ((1->3)-beta-glucan endohydrolase) ((1->3)-beta-glucanase) (Beta-1,3-endoglucanase) - Triticum aestivum (Wheat)very weakly similar to (90.1) loc_os07g32600 12007.m28981 protein glucan endo-1,3-beta-glucosidase 3 precursor, putative, expressed  no original description 	T
28.1.3	DNA.synthesis/chromatin structure.histone	MDP0000322229	 moderately similar to ( 262) AT5G65360 | Symbols:  | histone H3 | chr5:26120099-26120509 REVERSEmoderately similar to ( 261) H32_WHEAT Histone H3.2 - Triticum aestivum (Wheat)moderately similar to ( 262) loc_os11g05730 12011.m04769 protein histone H3, putative, expressed  no original description 	T
29.2.1.2.2.8	protein.synthesis.ribosomal protein.eukaryotic.60S subunit.L8	MDP0000290336	 highly similar to ( 507) AT4G36130 | Symbols:  | 60S ribosomal protein L8 (RPL8C) | chr4:17097613-17098656 FORWARDmoderately similar to ( 488) RL8_TOBAC 60S ribosomal protein L8 (60S ribosomal protein L2) - Nicotiana tabacum (Common tobacco)highly similar to ( 501) loc_os12g38000 12012.m07586 protein 60S ribosomal protein L2, putative, expressed   no original description 	T
29.3.2	protein.targeting.mitochondria	MDP0000188813	 moderately similar to ( 206) AT1G27390 | Symbols: TOM20-2 | TOM20-2 (TRANSLOCASE OUTER MEMBRANE 20-2); P-P-bond-hydrolysis-driven protein transmembrane transporter/ metal ion binding | chr1:9513469-9514912 REVERSEmoderately similar to ( 256) TOM20_SOLTU Mitochondrial import receptor subunit TOM20 (Translocase of outer membrane 20 kDa subunit) - Solanum tuberosum (Potato)weakly similar to ( 180) loc_os01g69250 12001.m43529 protein mitochondrial import receptor subunit TOM20, putative, expressed  no original description 	T
29.2.1.2.2.57	protein.synthesis.ribosomal protein.eukaryotic.60S subunit.L7A	MDP0000364277	 moderately similar to ( 202) AT5G20160 | Symbols:  | ribosomal protein L7Ae/L30e/S12e/Gadd45 family protein | chr5:6804653-6805102 REVERSEweakly similar to ( 200) loc_os03g13800 12003.m06824 protein NHP2-like protein 1, putative, expressed  no original description 	T
7.2.3	OPP.non-reductive PP.ribulose-phosphate 3-epimerase	MDP0000137234	 moderately similar to ( 457) AT5G61410 | Symbols: RPE | RPE; catalytic/ ribulose-phosphate 3-epimerase | chr5:24684085-24685836 REVERSEmoderately similar to ( 477) RPE_SPIOL Ribulose-phosphate 3-epimerase, chloroplast precursor (EC 5.1.3.1) (Pentose-5-phosphate 3-epimerase) (PPE) (RPE) (R5P3E) - Spinacia oleracea (Spinach)moderately similar to ( 427) loc_os03g07300 12003.m101203 protein ribulose-phosphate 3-epimerase, chloroplast precursor, putative, expressed  no original description 	T
1.3.3	PS.calvin cycle.phosphoglycerate kinase	MDP0000174843	 highly similar to ( 746) AT1G56190 | Symbols:  | phosphoglycerate kinase, putative | chr1:21028622-21030454 FORWARDhighly similar to ( 758) PGKH_TOBAC Phosphoglycerate kinase, chloroplast precursor (EC 2.7.2.3) - Nicotiana tabacum (Common tobacco)highly similar to ( 726) loc_os05g41640 12005.m08338 protein phosphoglycerate kinase, chloroplast precursor, putative, expressed  no original description 	T
35.2	not assigned.unknown	MDP0000833352	 weakly similar to ( 122) AT1G52140 | Symbols:  | unknown protein | chr1:19407559-19408185 REVERSEweakly similar to ( 108) loc_os06g36070 12006.m08144 protein expressed protein  no original description 	T
13.2.5.2	amino acid metabolism.degradation.serine-glycine-cysteine group.glycine	MDP0000570588	 moderately similar to ( 279) AT1G68010 | Symbols: HPR | HPR; glycerate dehydrogenase/ poly(U) binding | chr1:25493418-25495720 FORWARDmoderately similar to ( 325) DHGY_CUCSA Glycerate dehydrogenase (EC 1.1.1.29) (NADH-dependent hydroxypyruvate reductase) (HPR) (GDH) - Cucumis sativus (Cucumber)moderately similar to ( 268) loc_os02g01150 12002.m33493 protein hydroxypyruvate reductase, putative, expressed  no original description 	T
13.2.5.1	amino acid metabolism.degradation.serine-glycine-cysteine group.serine	MDP0000570588	 moderately similar to ( 279) AT1G68010 | Symbols: HPR | HPR; glycerate dehydrogenase/ poly(U) binding | chr1:25493418-25495720 FORWARDmoderately similar to ( 325) DHGY_CUCSA Glycerate dehydrogenase (EC 1.1.1.29) (NADH-dependent hydroxypyruvate reductase) (HPR) (GDH) - Cucumis sativus (Cucumber)moderately similar to ( 268) loc_os02g01150 12002.m33493 protein hydroxypyruvate reductase, putative, expressed  no original description 	T
1.2.6	PS.photorespiration.hydroxypyruvate reductase	MDP0000570588	 moderately similar to ( 279) AT1G68010 | Symbols: HPR | HPR; glycerate dehydrogenase/ poly(U) binding | chr1:25493418-25495720 FORWARDmoderately similar to ( 325) DHGY_CUCSA Glycerate dehydrogenase (EC 1.1.1.29) (NADH-dependent hydroxypyruvate reductase) (HPR) (GDH) - Cucumis sativus (Cucumber)moderately similar to ( 268) loc_os02g01150 12002.m33493 protein hydroxypyruvate reductase, putative, expressed  no original description 	T
16.2	secondary metabolism.phenylpropanoids	MDP0000157241	 weakly similar to ( 161) AT3G26040 | Symbols:  | transferase family protein | chr3:9519741-9521069 FORWARDvery weakly similar to (79.0) HCBT1_DIACA Anthranilate N-benzoyltransferase protein 1 (EC 2.3.1.144) (Anthranilate N-hydroxycinnamoyl/benzoyltransferase 1) - Dianthus caryophyllus (Carnation) (Clove pink)very weakly similar to (77.8) loc_os06g08580 12006.m05577 protein anthranilate N-benzoyltransferase protein 1, putative, expressed   no original description 	T
30.3	signalling.calcium	MDP0000230529	 nearly identical (1533) AT1G10130 | Symbols: ECA3, ATECA3 | ECA3 (ENDOPLASMIC RETICULUM-TYPE CALCIUM-TRANSPORTING ATPASE 3); calcium-transporting ATPase/ calmodulin binding / manganese-transporting ATPase/ peroxidase | chr1:3311139-3321941 FORWARDmoderately similar to ( 288) ACA1_ORYSA Calcium-transporting ATPase 1, plasma membrane-type (EC 3.6.3.8) (Ca(2+)-ATPase isoform 1) (Plastid envelope ATPase 1) - Oryza sativa (Rice)nearly identical (1428) loc_os03g52090 12003.m10186 protein calcium-transporting ATPase 3, endoplasmic reticulum-type, putative, expressed  no original description 	T
29.4	protein.postranslational modification	MDP0000230606	 highly similar to ( 606) AT1G10430 | Symbols: PP2A-2 | PP2A-2; protein serine/threonine phosphatase | chr1:3428705-3430437 REVERSEhighly similar to ( 617) PP2A_HELAN Serine/threonine-protein phosphatase PP2A catalytic subunit (EC 3.1.3.16) - Helianthus annuus (Common sunflower)highly similar to ( 600) loc_os06g37660 12006.m08301 protein OsPP2Ac-1 - Phosphatase 2A isoform 1 belonging to family 1, expressed   no original description 	T
35.1	not assigned.no ontology	MDP0000141181	 moderately similar to ( 327) AT5G10860 | Symbols:  | CBS domain-containing protein | chr5:3429173-3430142 REVERSEmoderately similar to ( 313) loc_os03g52690 12003.m101601 protein CBS domain protein, putative, expressed no original description 	T
26.28	misc.GDSL-motif lipase	MDP0000146003	 moderately similar to ( 402) AT1G75900 | Symbols:  | family II extracellular lipase 3 (EXL3) | chr1:28499179-28500943 FORWARDmoderately similar to ( 263) APG_BRANA Anter-specific proline-rich protein APG (Protein CEX) (Fragment) - Brassica napus (Rape)moderately similar to ( 335) loc_os03g64170 12003.m101141 protein anther-specific proline-rich protein APG precursor, putative, expressed no original description 	T
30.2.99	signalling.receptor kinases.misc	MDP0000320718	 nearly identical (1277) AT2G37050 | Symbols:  | kinase | chr2:15569290-15573477 FORWARDmoderately similar to ( 419) NORK_PEA Nodulation receptor kinase precursor (EC 2.7.11.1) - Pisum sativum (Garden pea)nearly identical (1123) loc_os12g01200 12012.m073729 protein transposon protein, putative, unclassified, expressed   no original description 	T
29.4	protein.postranslational modification	MDP0000192960	 highly similar to ( 778) AT1G68830 | Symbols: STN7 | STN7 (Stt7 homolog STN7); kinase/ protein kinase | chr1:25872654-25875473 REVERSEmoderately similar to ( 283) STT7_CHLRE Serine/threonine-protein kinase stt7, chloroplast precursor (EC 2.7.11.1) - Chlamydomonas reinhardtiihighly similar to ( 719) loc_os05g47560 12005.m08829 protein serine/threonine-protein kinase SNT7, chloroplast precursor, putative, expressed   no original description 	T
29.3.1	protein.targeting.nucleus	MDP0000582419	 moderately similar to ( 281) AT3G25150 | Symbols:  | nuclear transport factor 2 (NTF2) family protein / RNA recognition motif (RRM)-containing protein | chr3:9157161-9159362 REVERSEvery weakly similar to (51.6) NTF2_ORYSA Nuclear transport factor 2 (NTF-2) - Oryza sativa (Rice)moderately similar to ( 258) loc_os02g29480 12002.m08092 protein RNA-binding protein-like, putative, expressed  no original description 	T
29.4	protein.postranslational modification	MDP0000174607	 moderately similar to ( 453) AT4G27800 | Symbols:  | protein phosphatase 2C PPH1 / PP2C PPH1 (PPH1) | chr4:13852405-13854091 REVERSEmoderately similar to ( 406) loc_os01g37130 12001.m43052 protein protein phosphatase 2C PPH1, putative, expressed  no original description 	T
35.2	not assigned.unknown	MDP0000623980	 moderately similar to ( 429) AT4G34260 | Symbols: FUC95A | FUC95A; 1,2-alpha-L-fucosidase | chr4:16398130-16401591 FORWARDmoderately similar to ( 424) loc_os10g19880 12010.m05016 protein large secreted protein, putative, expressed  no original description 	T
31.4	cell.vesicle transport	MDP0000276262	 moderately similar to ( 218) AT1G79990 | Symbols:  | protein binding / structural molecule | chr1:30085910-30091949 FORWARDmoderately similar to ( 227) loc_os01g41040 12001.m10373 protein protein SYS1, putative, expressed no original description 	T
29.5.3	protein.degradation.cysteine protease	MDP0000153556	 highly similar to ( 506) AT4G16190 | Symbols:  | cysteine proteinase, putative | chr4:9171512-9172877 FORWARDhighly similar to ( 502) CYSP_PEA Cysteine proteinase 15A precursor (EC 3.4.22.-) (Turgor-responsive protein 15A) - Pisum sativum (Garden pea)moderately similar to ( 480) loc_os02g27030 12002.m07847 protein cysteine proteinase 1 precursor, putative, expressed  no original description 	T
26.22	misc.short chain dehydrogenase/reductase (SDR)	MDP0000871896	 highly similar to ( 550) AT4G27760 | Symbols: FEY, FEY3 | FEY (FOREVER YOUNG); binding / catalytic/ oxidoreductase | chr4:13844151-13846563 FORWARDvery weakly similar to (78.6) POR_DAUCA Protochlorophyllide reductase, chloroplast precursor (EC 1.3.1.33) (PCR) (NADPH-protochlorophyllide oxidoreductase) (POR) - Daucus carota (Carrot)highly similar to ( 533) loc_os02g02470 12002.m05596 protein WW domain-containing oxidoreductase, putative, expressed   no original description 	T
34.22	transport.cyclic nucleotide or calcium regulated channels	MDP0000593435	 weakly similar to ( 104) AT5G53130 | Symbols: CNGC1, ATCNGC1 | CNGC1 (CYCLIC NUCLEOTIDE GATED CHANNEL 1); calmodulin binding / cation channel/ cyclic nucleotide binding / intracellular cyclic nucleotide activated cation channel/ inward rectifier potassium channel | chr5:21537830-21540490 REVERSEweakly similar to ( 104) loc_os06g33610 12006.m07898 protein cyclic nucleotide-gated ion channel 1, putative, expressed no original description 	T
27.4	RNA.RNA binding	MDP0000243576	 moderately similar to ( 315) AT4G17720 | Symbols:  | RNA recognition motif (RRM)-containing protein | chr4:9862660-9864498 REVERSEmoderately similar to ( 271) loc_os04g50790 12004.m35440 protein nucleic acid binding protein, putative, expressed   no original description 	T
11.1.15	lipid metabolism.FA synthesis and FA elongation.ACP desaturase	MDP0000148102	 highly similar to ( 630) AT2G43710 | Symbols: SSI2, FAB2 | SSI2; acyl-[acyl-carrier-protein] desaturase/ stearoyl-CoA 9-desaturase | chr2:18120107-18122495 FORWARDhighly similar to ( 660) STAD_RICCO Acyl-[acyl-carrier-protein] desaturase, chloroplast precursor (EC 1.14.19.2) (Stearoyl-ACP desaturase) (Delta(9) stearoyl-acyl carrier protein desaturase) - Ricinus communis (Castor bean)highly similar to ( 616) loc_os01g69080 12001.m13009 protein acyl-desaturase, chloroplast precursor, putative, expressed  no original description 	T
29.5.5	protein.degradation.serine protease	MDP0000843722	 highly similar to ( 731) AT1G28110 | Symbols: SCPL45 | SCPL45 (SERINE CARBOXYPEPTIDASE-LIKE 45 PRECURSOR); serine-type carboxypeptidase | chr1:9804153-9806832 REVERSEmoderately similar to ( 342) CBP23_HORVU Serine carboxypeptidase II-3 precursor (EC 3.4.16.6) (CP-MII.3) [Contains: Serine carboxypeptidase II-3 chain A; Serine carboxypeptidase II-3 chain B] - Hordeum vulgare (Barley)highly similar to ( 690) loc_os11g31980 12011.m07100 protein serine carboxypeptidase 1 precursor, putative, expressed  no original description 	T
35.1	not assigned.no ontology	MDP0000166777	 moderately similar to ( 249) AT1G21880 | Symbols: LYM1 | LYM1 (LYSM DOMAIN GPI-ANCHORED PROTEIN 1 PRECURSOR) | chr1:7680689-7682526 FORWARDmoderately similar to ( 232) loc_os06g10660 12006.m05784 protein erwinia induced protein 1, putative, expressed  no original description 	T
31.3.1	cell.cycle.peptidylprolyl isomerase	MDP0000252743	 weakly similar to ( 191) AT5G48580 | Symbols: FKBP15-2 | FKBP15-2; FK506 binding / peptidyl-prolyl cis-trans isomerase | chr5:19696156-19697304 REVERSEweakly similar to ( 185) FKB15_VICFA FK506-binding protein 2 precursor (EC 5.2.1.8) (Peptidyl-prolyl cis-trans isomerase) (PPIase) (Rotamase) (15 kDa FKBP) (FKBP-15) - Vicia faba (Broad bean)weakly similar to ( 187) loc_os09g32526 12009.m22153 protein FK506-binding protein 2-1 precursor, putative, expressed  no original description 	T
29.2.1.2.2.57	protein.synthesis.ribosomal protein.eukaryotic.60S subunit.L7A	MDP0000272625	 weakly similar to ( 190) AT5G20160 | Symbols:  | ribosomal protein L7Ae/L30e/S12e/Gadd45 family protein | chr5:6804653-6805102 REVERSEweakly similar to ( 188) loc_os03g13800 12003.m06824 protein NHP2-like protein 1, putative, expressed no original description 	T
13.2.6.2	amino acid metabolism.degradation.aromatic aa.tyrosine	MDP0000194425	 highly similar to ( 595) AT1G12050 | Symbols:  | fumarylacetoacetase, putative | chr1:4072904-4075856 FORWARDhighly similar to ( 541) loc_os02g10310 12002.m100035 protein fumarylacetoacetase, putative, expressed   no original description 	T
29.5	protein.degradation	MDP0000244791	 moderately similar to ( 223) AT4G11740 | Symbols: SAY1 | SAY1 | chr4:7071955-7075256 FORWARDmoderately similar to ( 248) loc_os09g35710 12009.m22020 protein SAY1, putative, expressed   no original description 	T
27.4	RNA.RNA binding	MDP0000941113	 moderately similar to ( 235) AT1G01080 | Symbols:  | 33 kDa ribonucleoprotein, chloroplast, putative / RNA-binding protein cp33, putative | chr1:45503-46789 REVERSEweakly similar to ( 106) ROC5_NICSY 33 kDa ribonucleoprotein, chloroplast precursor - Nicotiana sylvestris (Wood tobacco)moderately similar to ( 203) loc_os08g02390 12008.m04383 protein ribonucleoprotein, chloroplast precursor, putative, expressed  no original description 	T
25.8	C1-metabolism.tetrahydrofolate synthase	MDP0000208825	 highly similar to ( 696) AT3G10160 | Symbols: ATDFC, DFC | DFC (DHFS-FPGS HOMOLOG C); tetrahydrofolylpolyglutamate synthase | chr3:3139588-3143949 REVERSEmoderately similar to ( 499) loc_os03g02030 12003.m101150 protein folylpolyglutamate synthase, mitochondrial precursor, putative, expressed  no original description 	T
35.2	not assigned.unknown	MDP0000951795	 moderately similar to ( 348) AT2G37960 | Symbols:  | unknown protein | chr2:15886962-15889180 REVERSE  no original description 	T
33.99	development.unspecified	MDP0000233467	 very weakly similar to (60.1) AT5G20700 | Symbols:  | senescence-associated protein-related | chr5:7006178-7007003 REVERSEvery weakly similar to (68.6) loc_os02g07820 12002.m06128 protein expressed protein  no original description 	T
27.3.2	RNA.regulation of transcription.Alfin-like	MDP0000183530	 moderately similar to ( 309) AT3G11200 | Symbols: AL2 | AL2 (ALFIN-LIKE 2); DNA binding / methylated histone residue binding | chr3:3508387-3510195 REVERSEmoderately similar to ( 288) loc_os05g07040 12005.m05230 protein PHD finger protein, putative, expressed  no original description 	T
35.2	not assigned.unknown	MDP0000208016	 weakly similar to ( 119) AT1G21065 | Symbols:  | unknown protein | chr1:7374210-7375644 FORWARDweakly similar to ( 109) loc_os06g43140 12006.m08844 protein expressed protein no original description 	T
29.7	protein.glycosylation	MDP0000819808	 moderately similar to ( 447) AT1G08280 | Symbols:  | glycosyl transferase family 29 protein / sialyltransferase family protein | chr1:2608408-2609604 FORWARDmoderately similar to ( 360) loc_os01g63970 12001.m12512 protein sialyltransferase-like protein, putative, expressed  MSP1_C  no original description 	T
10.6.3	cell wall.degradation.pectate lyases and polygalacturonases	MDP0000251956	 highly similar to ( 719) AT3G61490 | Symbols:  | glycoside hydrolase family 28 protein / polygalacturonase (pectinase) family protein | chr3:22758439-22760137 FORWARDvery weakly similar to (99.4) PGLR2_MAIZE Exopolygalacturonase precursor (EC 3.2.1.67) (ExoPG) (Pectinase) (Galacturan 1,4-alpha-galacturonidase) - Zea mays (Maize)highly similar to ( 558) loc_os03g03350 12003.m05870 protein glycoside hydrolase, family 28, putative, expressed  no original description 	T
25	C1-metabolism	MDP0000932736	 moderately similar to ( 207) AT5G14780 | Symbols: FDH | FDH (FORMATE DEHYDROGENASE); NAD or NADH binding / binding / catalytic/ cofactor binding / oxidoreductase, acting on the CH-OH group of donors, NAD or NADP as acceptor | chr5:4777043-4779190 FORWARDmoderately similar to ( 227) FDH_SOLTU Formate dehydrogenase, mitochondrial precursor (EC 1.2.1.2) (NAD-dependent formate dehydrogenase) (FDH) - Solanum tuberosum (Potato)moderately similar to ( 221) loc_os06g29180 12006.m07462 protein formate dehydrogenase 1, mitochondrial precursor, putative, expressed no original description 	T
29.5	protein.degradation	MDP0000757637	 weakly similar to ( 187) AT3G08980 | Symbols:  | signal peptidase I family protein | chr3:2741279-2742375 FORWARDweakly similar to ( 149) loc_os04g08340 12004.m06123 protein mitochondrial inner membrane protease subunit 2, putative, expressed no original description 	T
30.2.17	signalling.receptor kinases.DUF 26	MDP0000258335	 highly similar to ( 806) AT2G45340 | Symbols:  | leucine-rich repeat transmembrane protein kinase, putative | chr2:18691739-18694466 FORWARDweakly similar to ( 192) RPK1_IPONI Receptor-like protein kinase precursor (EC 2.7.11.1) - Ipomoea nil (Japanese morning glory) (Pharbitis nil)highly similar to ( 733) loc_os06g04370 12006.m05166 protein ATP binding protein, putative, expressed  no original description 	T
16.2.1.1	secondary metabolism.phenylpropanoids.lignin biosynthesis.PAL	MDP0000261492	 nearly identical (1169) AT2G37040 | Symbols: pal1, ATPAL1 | pal1 (Phe ammonia lyase 1); phenylalanine ammonia-lyase | chr2:15557602-15560237 REVERSEnearly identical (1199) PAL3_PETCR Phenylalanine ammonia-lyase 3 (EC 4.3.1.5) - Petroselinum crispum (Parsley) (Petroselinum hortense)nearly identical (1078) loc_os04g43800 12004.m09339 protein phenylalanine ammonia-lyase, putative, expressed  no original description 	T
21.2	redox.ascorbate and glutathione	MDP0000289918	 weakly similar to ( 144) AT1G60660 | Symbols: B5 #5, ATCB5LP, CB5LP | CB5LP (CYTOCHROME B5-LIKE PROTEIN); heme binding | chr1:22342589-22342954 REVERSEvery weakly similar to (80.1) CYB5_TOBAC Cytochrome b5 - Nicotiana tabacum (Common tobacco)weakly similar to ( 135) loc_os08g29669 12008.m80263 protein cytochrome b5, putative, expressed no original description 	T
35.2	not assigned.unknown	MDP0000719652	   no original description 	T
27.4	RNA.RNA binding	MDP0000635271	 weakly similar to ( 182) AT5G61030 | Symbols: GR-RBP3 | GR-RBP3 (glycine-rich RNA-binding protein 3); ATP binding / RNA binding | chr5:24560870-24562152 FORWARDvery weakly similar to (97.4) GRP2_SINAL Glycine-rich RNA-binding protein GRP2A - Sinapis alba (White mustard) (Brassica hirta)weakly similar to ( 153) loc_os10g17454 12010.m065240 protein RNA-binding protein precursor, putative, expressed no original description 	T
34.9	transport.metabolite transporters at the mitochondrial membrane	MDP0000462818	 moderately similar to ( 442) AT4G32400 | Symbols: SHS1, ATBT1 | SHS1 (SODIUM HYPERSENSITIVE 1); binding / nucleotide transmembrane transporter/ transporter | chr4:15638686-15640238 FORWARDmoderately similar to ( 337) BT1_MAIZE Protein brittle-1, chloroplast precursor - Zea mays (Maize)moderately similar to ( 405) loc_os05g07900 12005.m083634 protein protein brittle-1, chloroplast precursor, putative, expressed  no original description 	T
35.2	not assigned.unknown	MDP0000352267	   no original description 	T
35.2	not assigned.unknown	MDP0000785770	 weakly similar to ( 163) AT2G42975 | Symbols:  | unknown protein | chr2:17873998-17874820 FORWARDweakly similar to ( 132) loc_os01g47630 12001.m10957 protein expressed protein no original description 	T
29.2.1.2.2.26	protein.synthesis.ribosomal protein.eukaryotic.60S subunit.L26	MDP0000695520	 moderately similar to ( 211) AT3G49910 | Symbols:  | 60S ribosomal protein L26 (RPL26A) | chr3:18504311-18504751 FORWARDweakly similar to ( 191) RL26_BRARA 60S ribosomal protein L26 - Brassica rapa (Turnip)weakly similar to ( 190) loc_os12g05430 12012.m04536 protein 60S ribosomal protein L26-1, putative, expressed  no original description 	T
29.5.11.4.2	protein.degradation.ubiquitin.E3.RING	MDP0000511046	 moderately similar to ( 347) AT3G58030 | Symbols:  | zinc finger (C3HC4-type RING finger) family protein | chr3:21485527-21486837 FORWARDvery weakly similar to (53.5) COP1_PEA E3 ubiquitin ligase protein COP1 (EC 6.3.2.-) (Constitutive photomorphogenesis protein 1) - Pisum sativum (Garden pea)weakly similar to ( 134) loc_os08g06510 12008.m04790 protein zinc finger, C3HC4 type family protein, expressed  no original description 	T
33.99	development.unspecified	MDP0000128887	 weakly similar to ( 177) AT5G11460 | Symbols:  | senescence-associated protein-related | chr5:3657064-3658388 REVERSEweakly similar to ( 129) loc_os01g08520 12001.m07477 protein expressed protein  no original description 	T
6.3	gluconeogenesis / glyoxylate cycle.Malate DH	MDP0000277049	 highly similar to ( 554) AT2G22780 | Symbols: PMDH1 | PMDH1 (PEROXISOMAL NAD-MALATE DEHYDROGENASE 1); L-malate dehydrogenase/ binding / catalytic/ malate dehydrogenase/ oxidoreductase/ oxidoreductase, acting on the CH-OH group of donors, NAD or NADP as acceptor | chr2:9689995-9691923 REVERSEhighly similar to ( 570) MDHG_CUCSA Malate dehydrogenase, glyoxysomal precursor (EC 1.1.1.37) - Cucumis sativus (Cucumber)highly similar to ( 548) loc_os12g43630 12012.m26992 protein malate dehydrogenase, glyoxysomal precursor, putative, expressed  no original description 	T
21.1	redox.thioredoxin	MDP0000740655	 moderately similar to ( 377) AT1G76080 | Symbols: ATCDSP32, CDSP32 | CDSP32 (CHLOROPLASTIC DROUGHT-INDUCED STRESS PROTEIN OF 32 KD) | chr1:28548063-28549348 REVERSEvery weakly similar to (59.7) TRXH_WHEAT Thioredoxin H-type (TRX-H) (TrxTa) - Triticum aestivum (Wheat)moderately similar to ( 351) loc_os07g29410 12007.m29166 protein electron transporter/ thiol-disulfide exchange intermediate, putative, expressed  no original description 	T
28.1.3	DNA.synthesis/chromatin structure.histone	MDP0000205983	 weakly similar to ( 163) AT5G02560 | Symbols: HTA12 | HTA12; DNA binding | chr5:575437-576456 FORWARDweakly similar to ( 175) H2A3_MEDTR Probable histone H2A.3 - Medicago truncatula (Barrel medic)weakly similar to ( 168) loc_os03g17100 12003.m07141 protein histone H2A, putative, expressed  no original description 	T
27.3.22	RNA.regulation of transcription.HB,Homeobox transcription factor family	MDP0000423596	 moderately similar to ( 348) AT1G69780 | Symbols: ATHB13 | ATHB13; DNA binding / sequence-specific DNA binding / transcription factor | chr1:26259166-26260465 FORWARDvery weakly similar to (52.4) SUT2_ORYSA Sucrose transport protein 2 (Sucrose permease 2) (Sucrose-proton symporter 2) (Sucrose transporter 2) (SUC4-like protein) - Oryza sativa (Rice)moderately similar to ( 290) loc_os03g07450 12003.m06259 protein homeobox-leucine zipper protein HAT7, putative, expressed   no original description 	T
30.3	signalling.calcium	MDP0000231787	 highly similar to ( 591) AT5G61790 | Symbols: CNX1, ATCNX1 | calnexin 1 (CNX1) | chr5:24827394-24829642 REVERSEhighly similar to ( 625) CALX_SOYBN Calnexin homolog precursor - Glycine max (Soybean)highly similar to ( 581) loc_os04g32950 12004.m78935 protein calnexin homolog precursor, putative, expressed  no original description 	T
33.99	development.unspecified	MDP0000189244	 nearly identical (1258) AT1G52360 | Symbols:  | coatomer protein complex, subunit beta 2 (beta prime), putative | chr1:19499282-19505397 FORWARDvery weakly similar to (92.8) PF20_CHLRE Flagellar WD repeat protein PF20 - Chlamydomonas reinhardtiinearly identical (1254) loc_os06g05180 12006.m71402 protein coatomer subunit beta, putative, expressed no original description 	T
26.22	misc.short chain dehydrogenase/reductase (SDR)	MDP0000848695	 moderately similar to ( 263) AT1G67730 | Symbols: YBR159, KCR1 | YBR159; ketoreductase/ oxidoreductase | chr1:25391676-25393365 FORWARDvery weakly similar to (53.5) FABG3_BRANA 3-oxoacyl-[acyl-carrier-protein] reductase 3, chloroplast precursor (EC 1.1.1.100) (3-ketoacyl-acyl carrier protein reductase 3) - Brassica napus (Rape)moderately similar to ( 258) loc_os02g38440 12002.m08932 protein steroid dehydrogenase KIK-I, putative, expressed  no original description 	T
20.2.3	stress.abiotic.drought/salt	MDP0000776572	 highly similar to ( 919) AT4G14360 | Symbols:  | dehydration-responsive protein-related | chr4:8267869-8270191 REVERSEhighly similar to ( 885) loc_os10g33720 12010.m21920 protein ankyrin-like protein, putative, expressed  no original description 	T
30.7	signalling.14-3-3 proteins	MDP0000772373	 moderately similar to ( 431) AT1G78300 | Symbols: GRF2, 14-3-3OMEGA, GF14 OMEGA | GRF2 (GENERAL REGULATORY FACTOR 2); protein binding / protein phosphorylated amino acid binding | chr1:29461883-29463052 FORWARDmoderately similar to ( 450) 1433_OENHO 14-3-3-like protein - Oenothera hookeri (Hooker's evening primrose)moderately similar to ( 408) loc_os08g33370 12008.m26501 protein 14-3-3-like protein, putative, expressed no original description 	T
11.3.10	lipid metabolism.Phospholipid synthesis.(S)-coclaurine-N-methyltransferase	MDP0000828170	 highly similar to ( 532) AT4G33110 | Symbols:  | coclaurine N-methyltransferase, putative | chr4:15972497-15974531 REVERSEvery weakly similar to (56.6) PEAM1_ARATH Phosphoethanolamine N-methyltransferase 1 (EC 2.1.1.103) (PEAMT 1) (AtNMT1) - Arabidopsis thaliana (Mouse-ear cress)highly similar to ( 539) loc_os06g37610 12006.m08296 protein cyclopropane-fatty-acyl-phospholipid synthase, putative, expressed no original description 	T
26.28	misc.GDSL-motif lipase	MDP0000193364	 highly similar to ( 519) AT1G54790 | Symbols:  | GDSL-motif lipase/hydrolase family protein | chr1:20441124-20443997 REVERSEmoderately similar to ( 281) EST_HEVBR Esterase precursor (EC 3.1.1.-) (Early nodule-specific protein homolog) (Latex allergen Hev b 13) - Hevea brasiliensis (Para rubber tree)highly similar to ( 504) loc_os05g33270 12005.m07559 protein esterase precursor, putative, expressed  no original description 	T
26.23	misc.rhodanese	MDP0000154068	 moderately similar to ( 285) AT2G42220 | Symbols:  | rhodanese-like domain-containing protein | chr2:17592105-17593305 FORWARDmoderately similar to ( 255) loc_os09g10750 12009.m04339 protein rhodanese family protein, putative, expressed  no original description 	T
23.3.2.3	nucleotide metabolism.salvage.nucleoside kinases.thymidine kinase	MDP0000176234	 moderately similar to ( 286) AT3G07800 | Symbols:  | thymidine kinase, putative | chr3:2489944-2490935 REVERSEmoderately similar to ( 290) KITH_ORYSA Thymidine kinase (EC 2.7.1.21) - Oryza sativa (Rice)moderately similar to ( 295) loc_os03g02200 12003.m05763 protein thymidine kinase, putative, expressed   no original description 	T
27.4	RNA.RNA binding	MDP0000119699	 moderately similar to ( 201) AT5G04430 | Symbols: BTR1, BTR1S | BTR1L (BINDING TO TOMV RNA 1L (LONG FORM)); nucleic acid binding / single-stranded RNA binding | chr5:1250602-1253523 REVERSEweakly similar to ( 186) loc_os02g57640 12002.m10785 protein RNA-binding protein Nova-1, putative, expressed no original description 	T
26.3	misc.gluco-, galacto- and mannosidases	MDP0000306738	 highly similar to ( 561) AT4G21760 | Symbols: BGLU47 | BGLU47 (Beta-glucosidase 47); catalytic/ cation binding / hydrolase, hydrolyzing O-glycosyl compounds | chr4:11561229-11563871 FORWARDmoderately similar to ( 311) BGLC_MAIZE Beta-glucosidase, chloroplast precursor (EC 3.2.1.21) (Gentiobiase) (Cellobiase) (Beta-D-glucoside glucohydrolase) - Zea mays (Maize)highly similar to ( 520) loc_os04g43360 12004.m101583 protein beta-glucosidase, chloroplast precursor, putative, expressed  no original description 	T
33.99	development.unspecified	MDP0000918566	 very weakly similar to (95.5) AT1G54330 | Symbols: ANAC020 | ANAC020 (Arabidopsis NAC domain containing protein 20); transcription factor | chr1:20279715-20280889 REVERSEvery weakly similar to (92.8) NAC68_ORYSA NAC domain-containing protein 68 (ONAC068) - Oryza sativa (Rice)very weakly similar to (97.4) loc_os03g02800 12003.m05820 protein ANAC086, putative, expressed  no original description 	T
35.2	not assigned.unknown	MDP0000160665	 highly similar to ( 739) AT5G49570 | Symbols: AtPNG1 | AtPNG1 (Arabidopsis thaliana peptide-N-glycanase 1); catalytic/ peptide-N4-(N-acetyl-beta-glucosaminyl)asparagine amidase | chr5:20112071-20116125 REVERSEmoderately similar to ( 486) PNG1_ORYSA Peptide-N(4)-(N-acetyl-beta-glucosaminyl)asparagine amidase (EC 3.5.1.52) (Peptide:N-glycanase) - Oryza sativa (Rice)highly similar to ( 654) loc_os07g31460 12007.m07415 protein transglutaminase-like superfamily protein, expressed no original description 	T
27.3.22	RNA.regulation of transcription.HB,Homeobox transcription factor family	MDP0000838851	 moderately similar to ( 493) AT1G52150 | Symbols: ATHB-15, ATHB15, CNA, ICU4 | ATHB-15; DNA binding / transcription factor | chr1:19410118-19413961 REVERSEmoderately similar to ( 388) loc_os03g43930 12003.m09434 protein class III HD-Zip protein 4, putative, expressed no original description 	T
29.3.4.4	protein.targeting.secretory pathway.plasma membrane	MDP0000304491	 highly similar to ( 554) AT2G30050 | Symbols:  | transducin family protein / WD-40 repeat family protein | chr2:12825540-12826448 FORWARDvery weakly similar to (55.8) GBB_ORYSA Guanine nucleotide-binding protein subunit beta - Oryza sativa (Rice)highly similar to ( 535) loc_os02g04320 12002.m33334 protein SEC13-related protein, putative, expressed  no original description 	T
34.16	transport.ABC transporters and multidrug resistance systems	MDP0000942052	 highly similar to ( 504) AT2G28070 | Symbols:  | ABC transporter family protein | chr2:11956432-11959782 FORWARDvery weakly similar to ( 100) PDR13_ORYSA Pleiotropic drug resistance protein 13 - Oryza sativa (Rice)moderately similar to ( 358) loc_os03g64200 12003.m101582 protein ATPase, coupled to transmembrane movement of substances, putative, expressed  no original description 	T
30.11	signalling.light	MDP0000542944	 moderately similar to ( 204) AT3G22840 | Symbols: ELIP1, ELIP | ELIP1 (EARLY LIGHT-INDUCABLE PROTEIN); chlorophyll binding | chr3:8084628-8085445 REVERSEweakly similar to ( 177) ELI_PEA Early light-induced protein, chloroplast precursor (ELIP) - Pisum sativum (Garden pea)weakly similar to ( 140) loc_os07g08150 12007.m05272 protein early light-induced protein, chloroplast precursor, putative, expressed no original description 	T
29.5.1	protein.degradation.subtilases	MDP0000232884	 weakly similar to ( 121) AT1G71950 | Symbols:  | identical protein binding / serine-type endopeptidase | chr1:27080453-27081573 REVERSEweakly similar to ( 113) loc_os05g35740 12005.m27840 protein Pi starvation-induced protein, putative, expressed  no original description 	T
35.2	not assigned.unknown	MDP0000683341	 moderately similar to ( 330) AT2G42570 | Symbols:  | unknown protein | chr2:17717498-17719921 REVERSEmoderately similar to ( 285) loc_os01g46350 12001.m10833 protein expressed protein  no original description 	T
35.2	not assigned.unknown	MDP0000849143	 moderately similar to ( 351) AT3G07090 | Symbols:  | unknown protein | chr3:2243153-2244476 REVERSEmoderately similar to ( 336) loc_os02g56900 12002.m10712 protein thioredoxin, putative, expressed no original description 	T
8.3	TCA / org transformation.carbonic anhydrases	MDP0000326334	 weakly similar to ( 197) AT4G33580 | Symbols: ATBCA5, BCA5 | carbonic anhydrase family protein / carbonate dehydratase family protein | chr4:16139406-16141363 FORWARDweakly similar to ( 134) CAHC_TOBAC Carbonic anhydrase, chloroplast precursor (EC 4.2.1.1) (Carbonate dehydratase) - Nicotiana tabacum (Common tobacco)weakly similar to ( 177) loc_os09g28910 12009.m22242 protein carbonic anhydrase, chloroplast precursor, putative, expressed  no original description 	T
35.2	not assigned.unknown	MDP0000782093	 moderately similar to ( 491) AT1G26090 | Symbols:  | unknown protein | chr1:9020509-9022619 FORWARDweakly similar to ( 157) loc_os02g48130 12002.m09847 protein anion-transporting ATPase, putative, expressed   no original description 	T
35.2	not assigned.unknown	MDP0000363737	 very weakly similar to (76.6) AT5G64130 | Symbols:  | FUNCTIONS IN: molecular_function unknown; INVOLVED IN: biological_process unknown; LOCATED IN: plasma membrane; EXPRESSED IN: 24 plant structures; EXPRESSED DURING: 15 growth stages; CONTAINS InterPro DOMAIN/s: Lg106-like (InterPro:IPR012482); BEST Arabidopsis thaliana protein match is: unknown protein (TAIR:AT1G69510.3); Has 91 Blast hits to 91 proteins in 11 species: Archae - 0; Bacteria - 0; Metazoa - 0; Fungi - 0; Plants - 91; Viruses - 0; Other Eukaryotes - 0 (source: NCBI BLink). | chr5:25664547-25665339 REVERSEvery weakly similar to (71.2) loc_os01g47490 12001.m150682 protein negatively light-regulated protein, putative, expressed no original description 	T
27.4	RNA.RNA binding	MDP0000303178	 moderately similar to ( 278) AT5G40490 | Symbols:  | RNA recognition motif (RRM)-containing protein | chr5:16225483-16227423 FORWARDvery weakly similar to (87.0) ROC1_SPIOL 28 kDa ribonucleoprotein, chloroplast (28RNP) - Spinacia oleracea (Spinach)moderately similar to ( 239) loc_os08g23120 12008.m06333 protein heterogeneous nuclear ribonucleoprotein A3, putative, expressed    no original description 	T
35.2	not assigned.unknown	MDP0000182248	 moderately similar to ( 231) AT3G49290 | Symbols: ABIL2 | ABIL2 (ABL INTERACTOR-LIKE PROTEIN 2) | chr3:18273407-18275221 FORWARDweakly similar to ( 160) ABIL3_ORYSA Probable protein ABIL3 (Abl interactor-like protein 3) - Oryza sativa (Rice)weakly similar to ( 160) loc_os01g13530 12001.m07962 protein protein ABIL1, putative, expressed   no original description 	T
35.2	not assigned.unknown	MDP0000322404	 weakly similar to ( 192) AT5G24610 | Symbols:  | unknown protein | chr5:8428577-8429017 FORWARDweakly similar to ( 197) loc_os10g36550 12010.m065308 protein expressed protein no original description 	T
29.5.5	protein.degradation.serine protease	MDP0000260362	 highly similar to ( 635) AT4G12910 | Symbols: scpl20 | scpl20 (serine carboxypeptidase-like 20); serine-type carboxypeptidase | chr4:7550576-7553051 REVERSEhighly similar to ( 597) CBP1_ORYSA Serine carboxypeptidase 1 precursor (EC 3.4.16.5) (Serine carboxypeptidase I) (Carboxypeptidase C) - Oryza sativa (Rice)highly similar to ( 597) loc_os12g15470 12012.m073802 protein serine carboxypeptidase 1 precursor, putative, expressed  no original description 	T
29.5.11.20	protein.degradation.ubiquitin.proteasom	MDP0000885236	 highly similar to ( 540) AT5G05780 | Symbols: RPN8A, AE3, ATHMOV34 | RPN8A (RP NON-ATPASE SUBUNIT 8A) | chr5:1735862-1738176 FORWARDmoderately similar to ( 249) loc_os04g56646 12004.m10553 protein expressed protein no original description 	T
29.5	protein.degradation	MDP0000211603	 highly similar to ( 983) AT1G67420 | Symbols:  | peptidase | chr1:25255411-25260358 FORWARDmoderately similar to ( 474) loc_os07g19530 12007.m06388 protein peptidase family M28 containing protein, expressed no original description 	T
34.18	transport.unspecified anions	MDP0000877937	 nearly identical (1089) AT3G62270 | Symbols:  | anion exchange family protein | chr3:23042528-23045633 REVERSEnearly identical (1113) loc_os12g37840 12012.m26773 protein boron transporter 1, putative, expressed  no original description 	T
29.4.1.57	protein.postranslational modification.kinase.receptor like cytoplasmatic kinase VII	MDP0000155031	 nearly identical (1302) AT3G59420 | Symbols: ACR4 | ACR4 (ARABIDOPSIS CRINKLY4); kinase/ transmembrane receptor protein kinase | chr3:21959871-21962558 REVERSEnearly identical (1192) CRI4_MAIZE Putative receptor protein kinase CRINKLY4 precursor (EC 2.7.11.1) - Zea mays (Maize)nearly identical (1237) loc_os03g43670 12003.m09410 protein receptor protein kinase CRINKLY4 precursor, putative, expressed  no original description 	T
10.6.1	cell wall.degradation.cellulases and beta -1,4-glucanases	MDP0000151410	 moderately similar to ( 370) AT4G02290 | Symbols: AtGH9B13 | AtGH9B13 (Arabidopsis thaliana glycosyl hydrolase 9B13); catalytic/ hydrolase, hydrolyzing O-glycosyl compounds | chr4:1002654-1005125 REVERSEmoderately similar to ( 338) GUN3_ORYSA Endoglucanase 3 precursor (EC 3.2.1.4) (Endo-1,4-beta glucanase 3) (OsGLU8) - Oryza sativa (Rice)moderately similar to ( 338) loc_os01g21070 12001.m08591 protein endoglucanase 1 precursor, putative, expressed   no original description 	T
26.3.4	misc.gluco-, galacto- and mannosidases.endoglucanase	MDP0000151410	 moderately similar to ( 370) AT4G02290 | Symbols: AtGH9B13 | AtGH9B13 (Arabidopsis thaliana glycosyl hydrolase 9B13); catalytic/ hydrolase, hydrolyzing O-glycosyl compounds | chr4:1002654-1005125 REVERSEmoderately similar to ( 338) GUN3_ORYSA Endoglucanase 3 precursor (EC 3.2.1.4) (Endo-1,4-beta glucanase 3) (OsGLU8) - Oryza sativa (Rice)moderately similar to ( 338) loc_os01g21070 12001.m08591 protein endoglucanase 1 precursor, putative, expressed   no original description 	T
33.99	development.unspecified	MDP0000214900	 highly similar to ( 523) AT2G40360 | Symbols:  | transducin family protein / WD-40 repeat family protein | chr2:16853123-16856635 REVERSEhighly similar to ( 582) loc_os07g25440 12007.m079668 protein ribosome biogenesis protein BOP1, putative, expressed  no original description 	T
33.99	development.unspecified	MDP0000176629	 moderately similar to ( 240) AT1G11430 | Symbols:  | plastid developmental protein DAG, putative | chr1:3847273-3848938 FORWARDmoderately similar to ( 242) DAG_ANTMA DAG protein, chloroplast precursor - Antirrhinum majus (Garden snapdragon)moderately similar to ( 240) loc_os08g04450 12008.m080079 protein DAG protein, chloroplast precursor, putative, expressed MSP1_C  no original description 	T
26.13	misc.acid and other phosphatases	MDP0000238339	 weakly similar to ( 177) AT2G39920 | Symbols:  | acid phosphatase class B family protein | chr2:16663201-16664305 REVERSEvery weakly similar to (57.0) VSPB_SOYBN Stem 31 kDa glycoprotein precursor (Vegetative storage protein B) - Glycine max (Soybean)very weakly similar to (73.6) loc_os03g21480 12003.m07552 protein stem 31 kDa glycoprotein precursor, putative, expressed  no original description 	T
27.3.71	RNA.regulation of transcription.SNF7	MDP0000337339	 moderately similar to ( 254) AT5G63880 | Symbols: VPS20.1 | VPS20.1 | chr5:25563890-25565258 FORWARDmoderately similar to ( 224) loc_os02g38300 12002.m100264 protein charged multivesicular body protein 6, putative, expressed  no original description 	T
29.4	protein.postranslational modification	MDP0000202153	 nearly identical (1019) AT5G35980 | Symbols: YAK1 | protein kinase family protein | chr5:14128551-14134354 FORWARDweakly similar to ( 103) MSK3_MEDSA Glycogen synthase kinase-3 homolog MsK-3 (EC 2.7.11.1) - Medicago sativa (Alfalfa)highly similar to ( 666) loc_os02g47410 12002.m100053 protein ATP binding protein, putative, expressed  PRK08026 TrbL  no original description 	T
26.2	misc.UDP glucosyl and glucoronyl transferases	MDP0000261777	 highly similar to ( 972) AT3G61130 | Symbols: GAUT1, LGT1 | GAUT1 (GALACTURONOSYLTRANSFERASE 1); polygalacturonate 4-alpha-galacturonosyltransferase/ transferase, transferring glycosyl groups | chr3:22622399-22625514 FORWARDhighly similar to ( 955) loc_os09g36190 12009.m06568 protein transferase, transferring glycosyl groups, putative, expressed  no original description 	T
35.2	not assigned.unknown	MDP0000227031	 moderately similar to ( 214) AT5G51040 | Symbols:  | unknown protein | chr5:20750700-20751790 FORWARDweakly similar to ( 196) loc_os11g32480 12011.m07149 protein TPR repeat region family protein, expressed  no original description 	T
26.22	misc.short chain dehydrogenase/reductase (SDR)	MDP0000321133	 moderately similar to ( 302) AT4G05530 | Symbols: IBR1, SDRA | IBR1 (INDOLE-3-BUTYRIC ACID RESPONSE 1); binding / catalytic/ oxidoreductase | chr4:2816462-2818074 FORWARDvery weakly similar to (53.9) GRDH_DAUCA Glucose and ribitol dehydrogenase (EC 1.1.1.-) (Carrot ABA-induced in somatic embryos 5 protein) - Daucus carota (Carrot)moderately similar to ( 242) loc_os09g04730 12009.m22042 protein dehydrogenase/reductase SDR family member 2, putative, expressed  no original description 	T
35.2	not assigned.unknown	MDP0000539485	 very weakly similar to (96.3) AT1G15260 | Symbols:  | unknown protein | chr1:5249834-5250370 FORWARD  no original description 	T
35.2	not assigned.unknown	MDP0000270760	 moderately similar to ( 398) AT4G21210 | Symbols: ATRP1 | ATRP1 (PPDK REGULATORY PROTEIN); phosphoprotein phosphatase/ protein kinase | chr4:11307002-11308587 FORWARDmoderately similar to ( 401) loc_os07g34640 12007.m29204 protein ATP/GTP-binding protein, putative, expressed   no original description 	T
31.1	cell.organisation	MDP0000156606	 highly similar to ( 610) AT4G27430 | Symbols: CIP7 | CIP7 (COP1-INTERACTING PROTEIN 7); transcription activator | chr4:13718817-13722736 FORWARDmoderately similar to ( 273) loc_os07g26440 12007.m079669 protein CIP7, putative, expressed  no original description 	T
34.2	transport.sugars	MDP0000093999	 moderately similar to ( 464) AT1G79360 | Symbols: ATOCT2 | ATOCT2 (ARABIDOPSIS THALIANA ORGANIC CATION/CARNITINE TRANSPORTER 2); carbohydrate transmembrane transporter/ sugar:hydrogen symporter | chr1:29854140-29855723 REVERSEvery weakly similar to (63.2) STC_RICCO Sugar carrier protein C - Ricinus communis (Castor bean)moderately similar to ( 365) loc_os07g37510 12007.m08001 protein carbohydrate transporter/ sugar porter, putative   no original description 	T
35.2	not assigned.unknown	MDP0000942663	 moderately similar to ( 250) AT3G24730 | Symbols:  | catalytic | chr3:9030152-9030894 REVERSEmoderately similar to ( 242) loc_os11g19220 12011.m05996 protein thioredoxin-like protein 4B, putative, expressed   no original description 	T
35.2	not assigned.unknown	MDP0000881537	 moderately similar to ( 392) AT5G62960 | Symbols:  | unknown protein | chr5:25269444-25271533 FORWARDmoderately similar to ( 354) loc_os02g03790 12002.m05728 protein expressed protein no original description 	T
29.4.1.57	protein.postranslational modification.kinase.receptor like cytoplasmatic kinase VII	MDP0000303653	 highly similar to ( 839) AT4G02010 | Symbols:  | protein kinase family protein | chr4:881457-885222 FORWARDmoderately similar to ( 223) PSKR_DAUCA Phytosulfokine receptor precursor (EC 2.7.11.1) (Phytosulfokine LRR receptor kinase) - Daucus carota (Carrot)highly similar to ( 714) loc_os01g14932 12001.m42949 protein NAK-like ser/thr protein kinase, putative, expressed   no original description 	T
30.1	signalling.in sugar and nutrient physiology	MDP0000530198	 highly similar to ( 517) AT5G51550 | Symbols: EXL3 | EXL3 (EXORDIUM LIKE 3) | chr5:20939793-20940806 REVERSEmoderately similar to ( 416) loc_os06g04250 12006.m05154 protein phi-1-like phosphate-induced protein, putative, expressed   no original description 	T
33.99	development.unspecified	MDP0000192492	 weakly similar to ( 175) AT4G35770 | Symbols: SEN1, ATSEN1, DIN1 | SEN1 (SENESCENCE 1) | chr4:16945073-16945964 FORWARDweakly similar to ( 172) DIN1_RAPSA Senescence-associated protein DIN1 - Raphanus sativus (Radish)weakly similar to ( 158) loc_os06g50930 12006.m09617 protein senescence-associated protein DIN1, putative, expressed no original description 	T
33.99	development.unspecified	MDP0000780079	 very weakly similar to (60.8) AT3G44735 | Symbols: PSK1, ATPSK3 | PSK1; growth factor | chr3:16291388-16292118 REVERSEvery weakly similar to (53.9) PSK5_ORYSA Phytosulfokines 5 precursor (Secretory protein SH27A) [Contains: Phytosulfokine-alpha (PSK-alpha) (Phytosulfokine-a); Phytosulfokine-beta (PSK-beta) (Phytosulfokine-b)] - Oryza sativa (Rice)very weakly similar to (53.9) loc_os12g05260 12012.m04520 protein phytosulfokines 5 precursor, putative, expressed no original description 	T
26.5	misc.acyl transferases	MDP0000267283	 nearly identical (1224) AT1G80410 | Symbols: EMB2753 | EMB2753 (EMBRYO DEFECTIVE 2753); binding | chr1:30227963-30234832 REVERSEnearly identical (1192) loc_os01g43030 12001.m43094 protein NMDA receptor-regulated 1-like protein, putative, expressed  no original description 	T
26.2	misc.UDP glucosyl and glucoronyl transferases	MDP0000396630	 moderately similar to ( 485) AT2G30150 | Symbols:  | UDP-glucoronosyl/UDP-glucosyl transferase family protein | chr2:12874706-12876122 FORWARDweakly similar to ( 152) IAAG_MAIZE Indole-3-acetate beta-glucosyltransferase (EC 2.4.1.121) (IAA-Glu synthetase) ((Uridine 5'-diphosphate-glucose:indol-3-ylacetyl)-beta-D-glucosyl transferase) - Zea mays (Maize)moderately similar to ( 413) loc_os01g59110 12001.m12049 protein indole-3-acetate beta-glucosyltransferase, putative, expressed     TrbL PRK08026 COG4907  no original description 	T
35.1	not assigned.no ontology	MDP0000219552	 weakly similar to ( 178) AT3G54120 | Symbols:  | reticulon family protein (RTNLB12) | chr3:20041269-20042133 REVERSEweakly similar to ( 140) loc_os04g29920 12004.m08098 protein seed maturation protein, putative, expressed   no original description 	T
34.7	transport.phosphate	MDP0000166477	 highly similar to ( 701) AT4G00370 | Symbols: ANTR2, PHT4;4 | ANTR2; inorganic phosphate transmembrane transporter/ organic anion transmembrane transporter | chr4:163153-166111 REVERSEhighly similar to ( 657) loc_os09g39680 12009.m06906 protein sialin, putative, expressed no original description 	T
16.1.4.6	secondary metabolism.isoprenoids.carotenoids.carotenoid beta ring hydroxylase	MDP0000235597	 moderately similar to ( 379) AT5G52570 | Symbols: BETA-OHASE 2, B2, CHY2 | BETA-OHASE 2 (BETA-CAROTENE HYDROXYLASE 2); carotene beta-ring hydroxylase | chr5:21334911-21336887 REVERSEmoderately similar to ( 358) loc_os04g48880 12004.m09823 protein beta-carotene hydroxylase, putative, expressed   no original description 	T
35.2	not assigned.unknown	MDP0000221063	 weakly similar to ( 187) AT1G10522 | Symbols:  | unknown protein | chr1:3470009-3471291 FORWARDweakly similar to ( 129) loc_os09g21460 12009.m05353 protein expressed protein  no original description 	T
27.3.24	RNA.regulation of transcription.MADS box transcription factor family	MDP0000322567	 weakly similar to ( 112) AT4G24540 | Symbols: AGL24 | AGL24 (AGAMOUS-LIKE 24); protein binding / protein heterodimerization/ protein homodimerization/ sequence-specific DNA binding / transcription factor | chr4:12671160-12673645 REVERSEvery weakly similar to ( 100) MAD47_ORYSA MADS-box transcription factor 47 (OsMADS47) - Oryza sativa (Rice)very weakly similar to ( 100) loc_os03g08754 12003.m35564 protein MADS-box transcription factor 47, putative, expressed  no original description 	T
27.3.25	RNA.regulation of transcription.MYB domain transcription factor family	MDP0000121476	 moderately similar to ( 335) AT3G01140 | Symbols: MYB106, AtMYB106 | MYB106 (myb domain protein 106); DNA binding / transcription factor | chr3:46619-48143 REVERSEmoderately similar to ( 206) ODO1_PETHY ODORANT1 protein (MYB-like protein ODO1) - Petunia hybrida (Petunia)moderately similar to ( 304) loc_os08g33660 12008.m07356 protein MYB1, putative, expressed   no original description 	T
29.4	protein.postranslational modification	MDP0000290045	 highly similar to ( 762) AT2G42810 | Symbols: PP5, PP5.2 | PP5.2 (PROTEIN PHOSPHATASE 5.2); phosphoprotein phosphatase/ protein binding / protein serine/threonine phosphatase | chr2:17812336-17815896 REVERSEmoderately similar to ( 223) PP1_ORYSA Serine/threonine-protein phosphatase PP1 (EC 3.1.3.16) - Oryza sativa (Rice)highly similar to ( 541) loc_os05g11550 12005.m083649 protein serine/threonine-protein phosphatase 5, putative, expressed   no original description 	T
10.7	cell wall.modification	MDP0000361876	 moderately similar to ( 457) AT4G03210 | Symbols: XTH9 | XTH9 (XYLOGLUCAN ENDOTRANSGLUCOSYLASE/HYDROLASE 9); hydrolase, acting on glycosyl bonds / xyloglucan:xyloglucosyl transferase | chr4:1416107-1417197 FORWARDmoderately similar to ( 311) XTH_SOYBN Probable xyloglucan endotransglucosylase/hydrolase precursor (EC 2.4.1.207) (Fragment) - Glycine max (Soybean)moderately similar to ( 355) loc_os04g53950 12004.m35473 protein glycosyl hydrolases family 16 protein, expressed no original description 	T
29.4	protein.postranslational modification	MDP0000250124	 highly similar to ( 721) AT3G23310 | Symbols:  | protein kinase, putative | chr3:8339799-8343355 FORWARDweakly similar to ( 153) G11A_ORYSA Protein kinase G11A (EC 2.7.11.1) - Oryza sativa (Rice)highly similar to ( 768) loc_os05g43570 12005.m083775 protein serine/threonine-protein kinase 38-like, putative, expressed  no original description 	T
15.1	metal handling.acquisition	MDP0000287362	 moderately similar to ( 435) AT5G49740 | Symbols: ATFRO7, FRO7 | ATFRO7 (FERRIC REDUCTION OXIDASE 7); ferric-chelate reductase/ oxidoreductase | chr5:20205549-20208628 REVERSEmoderately similar to ( 446) loc_os04g36720 12004.m08712 protein ferric reductase-like transmembrane component, putative, expressed   no original description 	T
35.1	not assigned.no ontology	MDP0000276413	 weakly similar to ( 186) AT2G39805 | Symbols:  | integral membrane Yip1 family protein | chr2:16610216-16612353 REVERSEweakly similar to ( 167) loc_os05g38180 12005.m083742 protein yip1 domain family, member 2, putative, expressed   no original description 	T
35.2	not assigned.unknown	MDP0000173007	 nearly identical (1241) AT5G11560 | Symbols:  | catalytic | chr5:3709734-3713994 REVERSEnearly identical (1154) loc_os05g14170 12005.m05829 protein expressed protein  no original description 	T
10.2	cell wall.cellulose synthesis	MDP0000286908	 highly similar to ( 941) AT4G31590 | Symbols: ATCSLC05, CSLC05, ATCSLC5 | ATCSLC5 (CELLULOSE-SYNTHASE LIKE C5); cellulose synthase/ transferase, transferring glycosyl groups | chr4:15309889-15312336 REVERSEhighly similar to ( 841) loc_os08g15420 12008.m05665 protein CSLC3 - cellulose synthase-like family C, expressed   no original description 	T
27.3.67	RNA.regulation of transcription.putative transcription regulator	MDP0000142403	 moderately similar to ( 245) AT1G76010 | Symbols:  | nucleic acid binding | chr1:28528505-28530488 REVERSEmoderately similar to ( 235) loc_os09g37006 12009.m22212 protein ribonuclease P, putative, expressed  no original description 	T
29.2.1.2.1.3	protein.synthesis.ribosomal protein.eukaryotic.40S subunit.S3	MDP0000656640	 moderately similar to ( 402) AT5G35530 | Symbols:  | 40S ribosomal protein S3 (RPS3C) | chr5:13710355-13712192 REVERSEmoderately similar to ( 411) loc_os07g41750 12007.m29256 protein 40S ribosomal protein S3-A, putative, expressed   no original description 	T
34.2	transport.sugars	MDP0000807456	 highly similar to ( 788) AT4G16480 | Symbols: ATINT4, INT4 | INT4 (INOSITOL TRANSPORTER 4); carbohydrate transmembrane transporter/ myo-inositol:hydrogen symporter/ sugar:hydrogen symporter | chr4:9291246-9293083 FORWARDweakly similar to ( 132) STC_RICCO Sugar carrier protein C - Ricinus communis (Castor bean)highly similar to ( 674) loc_os04g43210 12004.m101578 protein proton myo-inositol cotransporter, putative, expressed  no original description 	T
17.2.1	hormone metabolism.auxin.synthesis-degradation	MDP0000281064	 moderately similar to ( 445) AT3G02875 | Symbols: ILR1 | ILR1 (IAA-LEUCINE RESISTANT 1); IAA-Leu conjugate hydrolase/ IAA-Phe conjugate hydrolase/ metallopeptidase | chr3:631993-633859 FORWARDmoderately similar to ( 470) loc_os03g62060 12003.m11084 protein IAA-amino acid hydrolase ILR1 precursor, putative, expressed   no original description 	T
34.14	transport.unspecified cations	MDP0000280653	 moderately similar to ( 327) AT3G14750 | Symbols:  | unknown protein | chr3:4953765-4955373 REVERSEmoderately similar to ( 244) loc_os04g31210 12004.m35291 protein bile acid sodium symporter/ transporter, putative, expressed   no original description 	T
30.5	signalling.G-proteins	MDP0000181506	 moderately similar to ( 434) AT3G49180 | Symbols: RID3 | RID3 (ROOT INITIATION DEFECTIVE 3); nucleotide binding | chr3:18229810-18231874 FORWARDvery weakly similar to (75.1) GBLP_CHLRE Guanine nucleotide-binding protein subunit beta-like protein - Chlamydomonas reinhardtiimoderately similar to ( 321) loc_os07g22534 12007.m079655 protein WD-repeat protein 18, putative, expressed  no original description 	T
29.3.4.2	protein.targeting.secretory pathway.golgi	MDP0000296003	 nearly identical (1248) AT4G01810 | Symbols:  | protein transport protein-related | chr4:776734-779802 REVERSEnearly identical (1154) loc_os01g21850 12001.m08669 protein protein binding protein, putative, expressed   no original description 	T
35.1.42	not assigned.no ontology.proline rich family	MDP0000277694	 weakly similar to ( 164) AT5G07020 | Symbols:  | proline-rich family protein | chr5:2180669-2182284 REVERSEweakly similar to ( 135) loc_os02g35090 12002.m08597 protein expressed protein  no original description 	T
26.10	misc.cytochrome P450	MDP0000270602	 highly similar to ( 618) AT1G74110 | Symbols: CYP78A10 | CYP78A10; electron carrier/ heme binding / iron ion binding / monooxygenase/ oxygen binding | chr1:27866667-27868368 REVERSEmoderately similar to ( 451) C78A3_SOYBN Cytochrome P450 78A3 (EC 1.14.-.-) - Glycine max (Soybean)moderately similar to ( 489) loc_os07g41240 12007.m08360 protein cytochrome P450 78A4, putative, expressed     TrbL SPT5 PRK08026 Sporozoite_P67  no original description 	T
27.3.37	RNA.regulation of transcription.AS2,Lateral Organ Boundaries Gene Family	MDP0000239820	 moderately similar to ( 235) AT5G67420 | Symbols: LBD37 | LBD37 (LOB DOMAIN-CONTAINING PROTEIN 37) | chr5:26904576-26905415 REVERSEmoderately similar to ( 207) loc_os03g33090 12003.m08552 protein seed specific protein Bn15D17A, putative, expressed  no original description 	T
7.2.4	OPP.non-reductive PP.ribose 5-phosphate isomerase	MDP0000284538	 moderately similar to ( 274) AT5G44520 | Symbols:  | ribose 5-phosphate isomerase-related | chr5:17934610-17936441 REVERSEmoderately similar to ( 263) loc_os03g56869 12003.m10603 protein ribose-5-phosphate isomerase, putative, expressed  no original description 	T
31.3	cell.cycle	MDP0000253549	 moderately similar to ( 426) AT1G16330 | Symbols: CYCB3;1 | CYCB3;1 (cyclin b3;1); cyclin-dependent protein kinase | chr1:5582387-5584457 REVERSEmoderately similar to ( 238) CCNB2_ORYSA G2/mitotic-specific cyclin-2 (B-like cyclin) (CycOs2) - Oryza sativa (Rice)moderately similar to ( 242) loc_os04g47580 12004.m101621 protein cyclin B2, putative, expressed  no original description 	T
29.2.4	protein.synthesis.elongation	MDP0000202507	 highly similar to ( 845) AT1G04170 | Symbols: EIF2 GAMMA | EIF2 GAMMA; translation factor, nucleic acid binding / translation initiation factor | chr1:1097423-1099702 FORWARDvery weakly similar to (91.3) EFTU_PROWI Elongation factor Tu (EF-Tu) - Prototheca wickerhamiihighly similar to ( 867) loc_os12g41400 12012.m07921 protein eukaryotic translation initiation factor 2 gamma subunit, putative, expressed  no original description 	T
33.99	development.unspecified	MDP0000212044	 moderately similar to ( 427) AT1G66330 | Symbols:  | senescence-associated family protein | chr1:24729880-24732144 REVERSEmoderately similar to ( 396) loc_os05g05600 12005.m27702 protein ATA15 protein, putative, expressed no original description 	T
29.4	protein.postranslational modification	MDP0000648845	 highly similar to ( 751) AT5G05970 | Symbols: NEDD1 | NEDD1 (NEURAL PRECURSOR CELL EXPRESSED, DEVELOPMENTALLY DOWN-REGULATED GENE 1); nucleotide binding | chr5:1795464-1799313 FORWARDhighly similar to ( 587) PP1_ORYSA Serine/threonine-protein phosphatase PP1 (EC 3.1.3.16) - Oryza sativa (Rice)highly similar to ( 652) loc_os09g09470 12009.m22054 protein nucleotide binding protein, putative, expressed  no original description 	T
28.99	DNA.unspecified	MDP0000276962	 highly similar to ( 656) AT4G34490 | Symbols: ATCAP1, CAP 1, CAP1 | ATCAP1 (ARABIDOPSIS THALIANA CYCLASE ASSOCIATED PROTEIN 1); actin binding | chr4:16484896-16487355 REVERSEhighly similar to ( 576) loc_os03g51250 12003.m10106 protein adenylyl cyclase-associated protein 1, putative, expressed  no original description 	T
10.2.2	cell wall.cellulose synthesis.COBRA	MDP0000236092	 highly similar to ( 814) AT3G20580 | Symbols: COBL10 | COBL10 (COBRA-LIKE PROTEIN 10 PRECURSOR) | chr3:7188063-7190416 REVERSEmoderately similar to ( 486) THI4_CITSI Thiazole biosynthetic enzyme, chloroplast precursor - Citrus sinensis (Sweet orange)highly similar to ( 704) loc_os06g47110 12006.m09237 protein COBRA-like protein 7 precursor, putative, expressed  TrbL  no original description 	T
13.2.6.2	amino acid metabolism.degradation.aromatic aa.tyrosine	MDP0000321003	 highly similar to ( 954) AT5G49150 | Symbols: ATGEX2, GEX2 | GEX2 (GAMETE EXPRESSED 2) | chr5:19924760-19928527 FORWARDhighly similar to ( 706) loc_os09g25650 12009.m05717 protein GEX2, putative, expressed  no original description 	T
35.1	not assigned.no ontology	MDP0000140406	 moderately similar to ( 305) AT4G34020 | Symbols:  | DJ-1 family protein | chr4:16298553-16300897 REVERSEmoderately similar to ( 273) loc_os05g44330 12005.m27915 protein 4-methyl-5-thiazole monophosphate biosynthesis protein, putative, expressed  no original description 	T
29.4	protein.postranslational modification	MDP0000296958	 highly similar to ( 997) AT1G73660 | Symbols:  | protein kinase family protein | chr1:27692247-27696718 REVERSEvery weakly similar to (86.7) PSKR_DAUCA Phytosulfokine receptor precursor (EC 2.7.11.1) (Phytosulfokine LRR receptor kinase) - Daucus carota (Carrot)highly similar to ( 755) loc_os06g12590 12006.m71421 protein ATP binding protein, putative, expressed   no original description 	T
17.2.3	hormone metabolism.auxin.induced-regulated-responsive-activated	MDP0000135436	 weakly similar to ( 179) AT5G20810 | Symbols:  | auxin-responsive protein, putative / small auxin up RNA (SAUR_B) | chr5:7044791-7045363 FORWARDvery weakly similar to (82.4) AXX15_SOYBN Auxin-induced protein X15 - Glycine max (Soybean)weakly similar to ( 142) loc_os09g26610 12009.m05813 protein OsSAUR38 - Auxin-responsive SAUR gene family member, expressed  no original description 	T
3.6	minor CHO metabolism.callose	MDP0000915307	 nearly identical (2499) AT4G04970 | Symbols: ATGSL1, ATGSL01, GSL01, GSL1 | GSL1 (GLUCAN SYNTHASE-LIKE 1); 1,3-beta-glucan synthase/ transferase, transferring glycosyl groups | chr4:2537039-2542434 FORWARDnearly identical (2297) loc_os01g55040 12001.m11664 protein ATGSL05, putative, expressed    no original description 	T
29.2.1.2.1.28	protein.synthesis.ribosomal protein.eukaryotic.40S subunit.S28	MDP0000364397	 very weakly similar to (86.3) AT5G64140 | Symbols: RPS28 | RPS28 (RIBOSOMAL PROTEIN S28); structural constituent of ribosome | chr5:25667529-25667723 REVERSEvery weakly similar to (88.2) RS28_MAIZE 40S ribosomal protein S28 - Zea mays (Maize)very weakly similar to (91.3) loc_os10g27174 12010.m65385 protein 40S ribosomal protein S28, putative, expressed  no original description 	T
34.99	transport.misc	MDP0000158714	 moderately similar to ( 405) AT1G32050 | Symbols:  | secretory carrier membrane protein (SCAMP) family protein | chr1:11528616-11530974 FORWARDmoderately similar to ( 372) loc_os04g50890 12004.m09986 protein SC3 protein, putative, expressed no original description 	T
4.1.10	glycolysis.cytosolic branch.aldolase	MDP0000362275	 weakly similar to ( 124) AT3G52930 | Symbols:  | fructose-bisphosphate aldolase, putative | chr3:19627383-19628874 REVERSEweakly similar to ( 130) ALF_ORYSA Fructose-bisphosphate aldolase cytoplasmic isozyme (EC 4.1.2.13) (Gravity-specific protein GSC 233) - Oryza sativa (Rice)weakly similar to ( 130) loc_os05g33380 12005.m07570 protein fructose-bisphosphate aldolase cytoplasmic isozyme, putative, expressed no original description 	T
35.2	not assigned.unknown	MDP0000289026	 weakly similar to ( 141) AT4G13500 | Symbols:  | unknown protein | chr4:7856987-7857874 REVERSEweakly similar to ( 133) loc_os03g39830 12003.m09066 protein expressed proteinMSP1_C  no original description 	T
29.5.11.4.2	protein.degradation.ubiquitin.E3.RING	MDP0000350960	 moderately similar to ( 244) AT5G01450 | Symbols:  | protein binding / zinc ion binding | chr5:183693-186244 REVERSEvery weakly similar to (82.8) loc_os05g44550 12005.m08581 protein ubiquitin-protein ligase/ zinc ion binding protein, putative no original description 	T
35.2	not assigned.unknown	MDP0000213812	 highly similar to ( 632) AT3G01060 | Symbols:  | unknown protein | chr3:16831-18782 REVERSEhighly similar to ( 614) loc_os05g43650 12005.m08492 protein expressed protein no original description 	T
27.3.99	RNA.regulation of transcription.unclassified	MDP0000799753	 moderately similar to ( 490) AT5G54180 | Symbols: PTAC15 | PTAC15 (PLASTID TRANSCRIPTIONALLY ACTIVE15) | chr5:21988544-21990183 FORWARDvery weakly similar to (80.1) loc_os07g04230 12007.m04892 protein EMB2219, putative, expressed   no original description 	T
26.10	misc.cytochrome P450	MDP0000310945	 highly similar to ( 757) AT5G04660 | Symbols: CYP77A4 | CYP77A4; electron carrier/ heme binding / iron ion binding / monooxygenase/ oxygen binding | chr5:1336049-1337587 FORWARDhighly similar to ( 778) C77A3_SOYBN Cytochrome P450 77A3 (EC 1.14.-.-) - Glycine max (Soybean)highly similar to ( 526) loc_os04g33370 12004.m08433 protein cytochrome P450 77A2, putative, expressed   no original description 	T
35.2	not assigned.unknown	MDP0000232608	 moderately similar to ( 248) AT3G16640 | Symbols: TCTP | TCTP (TRANSLATIONALLY CONTROLLED TUMOR PROTEIN) | chr3:5669709-5670729 REVERSEmoderately similar to ( 277) TCTP_ORYSA Translationally-controlled tumor protein homolog (TCTP) - Oryza sativa (Rice)moderately similar to ( 277) loc_os11g43900 12011.m08225 protein translationally-controlled tumor protein, putative, expressed  no original description 	T
2.1.2.2	major CHO metabolism.synthesis.starch.starch synthase	MDP0000247496	 highly similar to ( 864) AT1G32900 | Symbols:  | starch synthase, putative | chr1:11920582-11923506 REVERSEhighly similar to ( 906) SSG1_ANTMA Granule-bound starch synthase 1, chloroplast precursor (EC 2.4.1.21) (Granule-bound starch synthase I) (GBSS-I) - Antirrhinum majus (Garden snapdragon)highly similar to ( 814) loc_os07g22930 12007.m29139 protein granule-bound starch synthase 1b, chloroplast precursor, putative, expressed   no original description 	T
8.2.11	TCA / org transformation.other organic acid transformatons.atp-citrate lyase	MDP0000205540	 nearly identical (1115) AT3G06650 | Symbols: ACLB-1 | ACLB-1; ATP citrate synthase | chr3:2079247-2082633 REVERSEweakly similar to ( 104) SUCA_ORYSA Probable succinyl-CoA ligase [GDP-forming] subunit alpha, mitochondrial precursor (EC 6.2.1.4) (Succinyl-CoA synthetase subunit alpha) (SCS-alpha) - Oryza sativa (Rice)nearly identical (1107) loc_os01g19450 12001.m42644 protein ATP-citrate synthase subunit 1, putative, expressed   no original description 	T
35.1	not assigned.no ontology	MDP0000214906	 moderately similar to ( 374) AT1G80770 | Symbols: PDE318 | PDE318 (pigment defective 318); GTP binding | chr1:30355266-30357786 FORWARDmoderately similar to ( 345) loc_os08g06230 12008.m04762 protein nucleolar GTP-binding protein 1, putative, expressed  no original description 	T
35.2	not assigned.unknown	MDP0000924547	 very weakly similar to (62.0) AT2G28410 | Symbols:  | unknown protein | chr2:12156202-12156549 FORWARD  no original description 	T
35.2	not assigned.unknown	MDP0000206561	 very weakly similar to (79.0) AT2G20250 | Symbols:  | unknown protein | chr2:8734049-8736416 REVERSE  no original description 	T
27.1.20	RNA.processing.degradation dicer	MDP0000619907	 highly similar to ( 557) AT5G20320 | Symbols: DCL4, ATDCL4 | DCL4 (DICER-LIKE 4); RNA binding / protein binding / ribonuclease III | chr5:6859571-6869068 REVERSEmoderately similar to ( 462) loc_os04g43050 12004.m78969 protein dicer-like protein 4, putative, expressed  no original description 	T
35.2	not assigned.unknown	MDP0000155744	 moderately similar to ( 204) AT5G23040 | Symbols: CDF1 | CDF1 (CELL GROWTH DEFECT FACTOR 1) | chr5:7729465-7730930 REVERSEweakly similar to ( 189) loc_os02g49680 12002.m10002 protein calcium sensing receptor, putative, expressed  no original description 	T
35.1	not assigned.no ontology	MDP0000132610	 weakly similar to ( 167) AT1G18730 | Symbols: NDF6 | NDF6 (NDH DEPENDENT FLOW 6) | chr1:6460625-6461464 FORWARDweakly similar to ( 142) loc_os02g51020 12002.m10136 protein expressed protein  no original description 	T
20.2.4	stress.abiotic.touch/wounding	MDP0000334850	 very weakly similar to (77.8) AT4G28240 | Symbols:  | wound-responsive protein-related | chr4:13997979-13998239 REVERSE no original description 	T
35.2	not assigned.unknown	MDP0000167655	 very weakly similar to (97.1) AT4G08230 | Symbols:  | glycine-rich protein | chr4:5188047-5189574 REVERSEvery weakly similar to (89.0) loc_os03g15720 12003.m35135 protein expressed protein no original description 	T
35.2	not assigned.unknown	MDP0000151845	 weakly similar to ( 169) AT5G09840 | Symbols:  | unknown protein | chr5:3059027-3061970 FORWARDweakly similar to ( 136) loc_os12g06560 12012.m04646 protein expressed protein no original description 	T
10.6.3	cell wall.degradation.pectate lyases and polygalacturonases	MDP0000285495	 highly similar to ( 648) AT1G04680 | Symbols:  | pectate lyase family protein | chr1:1304052-1307780 REVERSEmoderately similar to ( 397) PEL_LILLO Pectate lyase precursor (EC 4.2.2.2) - Lilium longiflorum (Trumpet lily)highly similar to ( 632) loc_os04g05050 12004.m35243 protein pectate lyase 8 precursor, putative, expressed no original description 	T
35.2	not assigned.unknown	MDP0000159600	 weakly similar to ( 107) AT3G52740 | Symbols:  | unknown protein | chr3:19546446-19546868 REVERSEvery weakly similar to (85.9) loc_os04g33610 12004.m08456 protein expressed protein  no original description 	T
2.1.2	major CHO metabolism.synthesis.starch	MDP0000169375	 highly similar to ( 702) AT2G35710 | Symbols:  | glycogenin glucosyltransferase (glycogenin)-related | chr2:15010925-15012251 REVERSEhighly similar to ( 640) loc_os04g46750 12004.m35557 protein transferase, transferring glycosyl groups, putative, expressed  no original description 	T
29.4.1.57	protein.postranslational modification.kinase.receptor like cytoplasmatic kinase VII	MDP0000152433	 moderately similar to ( 267) AT2G37050 | Symbols:  | kinase | chr2:15569290-15573477 FORWARDweakly similar to ( 138) NORK_PEA Nodulation receptor kinase precursor (EC 2.7.11.1) - Pisum sativum (Garden pea)moderately similar to ( 219) loc_os12g01200 12012.m073729 protein transposon protein, putative, unclassified, expressed no original description 	T
10.6.3	cell wall.degradation.pectate lyases and polygalacturonases	MDP0000943790	 highly similar to ( 715) AT1G60590 | Symbols:  | polygalacturonase, putative / pectinase, putative | chr1:22314426-22316867 REVERSEmoderately similar to ( 277) PGLR_ACTCH Polygalacturonase precursor (EC 3.2.1.15) (PG) (Pectinase) - Actinidia chinensis (Kiwi) (Yangtao)highly similar to ( 593) loc_os01g44970 12001.m10703 protein polygalacturonase precursor, putative, expressed  no original description 	T
29.2.1.2.2.11	protein.synthesis.ribosomal protein.eukaryotic.60S subunit.L11	MDP0000300317	 weakly similar to ( 195) AT5G45775 | Symbols:  | 60S ribosomal protein L11 (RPL11D) | chr5:18565281-18566496 REVERSEweakly similar to ( 195) RL11_MEDSA 60S ribosomal protein L11 (L5) - Medicago sativa (Alfalfa)weakly similar to ( 193) loc_os02g14059 12002.m77785 protein 60S ribosomal protein L11-1, putative, expressed  no original description 	T
26.10	misc.cytochrome P450	MDP0000440922	 moderately similar to ( 418) AT5G36110 | Symbols: CYP716A1 | CYP716A1; electron carrier/ heme binding / iron ion binding / monooxygenase/ oxygen binding | chr5:14195377-14197613 FORWARDweakly similar to ( 191) C85A1_ORYSA Cytochrome P450 85A1 (EC 1.14.-.-) (C6-oxidase) (OsDWARF) (Dwarf protein) - Oryza sativa (Rice)moderately similar to ( 301) loc_os07g33580 12007.m07618 protein taxane 10-beta-hydroxylase, putative, expressed no original description 	T
29.5.11.4.3.2	protein.degradation.ubiquitin.E3.SCF.FBOX	MDP0000369237	 weakly similar to ( 163) AT4G07400 | Symbols: VFB3 | VFB3 (VIER F-BOX PROTEINE 3); ubiquitin-protein ligase | chr4:4197847-4199511 REVERSEweakly similar to ( 112) loc_os04g42670 12004.m09232 protein cyclin-like F-box, putative, expressed no original description 	T
26.12	misc.peroxidases	MDP0000128576	 weakly similar to ( 180) AT1G71695 | Symbols:  | peroxidase 12 (PER12) (P12) (PRXR6) | chr1:26964359-26966557 FORWARDweakly similar to ( 162) PER1_SORBI Cationic peroxidase SPC4 precursor (EC 1.11.1.7) (Fragment) - Sorghum bicolor (Sorghum) (Sorghum vulgare)weakly similar to ( 166) loc_os01g73220 12001.m13351 protein peroxidase 12 precursor, putative, expressed no original description 	T
29.1.19	protein.aa activation.arginine-tRNA ligase	MDP0000370315	 highly similar to ( 626) AT4G26300 | Symbols: emb1027 | emb1027 (embryo defective 1027); ATP binding / aminoacyl-tRNA ligase/ arginine-tRNA ligase/ nucleotide binding | chr4:13308400-13313109 REVERSEhighly similar to ( 597) loc_os05g07030 12005.m083624 protein arginyl-tRNA synthetase, putative, expressed no original description 	T
35.2	not assigned.unknown	MDP0000607654	   no original description 	T
29.7.9	protein.glycosylation.beta-1,2-xylosyltransferase(beta-1,2-XylT) 	MDP0000255676	 highly similar to ( 570) AT5G55500 | Symbols: ATXYLT, XYLT | XYLT (ARABIDOPSIS THALIANA BETA-1,2-XYLOSYLTRANSFERASE); xylosyltransferase | chr5:22482386-22484417 FORWARDhighly similar to ( 536) loc_os08g39380 12008.m07919 protein beta-2-xylosyltransferase, putative, expressed    TrbL  no original description 	T
29.2.4	protein.synthesis.elongation	MDP0000321320	 moderately similar to ( 449) AT4G11120 | Symbols:  | translation elongation factor Ts (EF-Ts), putative | chr4:6778066-6779934 FORWARDmoderately similar to ( 396) loc_os08g30884 12008.m07088 protein elongation factor Ts, putative, expressed  no original description 	T
27.3.40	RNA.regulation of transcription.Aux/IAA family	MDP0000131759	 moderately similar to ( 219) AT2G33310 | Symbols: IAA13 | IAA13; transcription factor | chr2:14114569-14115757 REVERSEweakly similar to ( 171) IAA10_ORYSA Auxin-responsive protein IAA10 (Indoleacetic acid-induced protein 10) - Oryza sativa (Rice)weakly similar to ( 171) loc_os02g57250 12002.m33477 protein OsIAA10 - Auxin-responsive Aux/IAA gene family member, expressed TrbL  no original description 	T
29.3.1	protein.targeting.nucleus	MDP0000935671	 nearly identical (1832) AT5G17020 | Symbols: XPO1A, ATCRM1, ATXPO1, XPO1 | XPO1A; protein binding / protein transporter/ receptor | chr5:5594904-5602467 FORWARDnearly identical (1682) loc_os03g64080 12003.m11273 protein retrotransposon protein, putative, unclassified  no original description 	T
26.2	misc.UDP glucosyl and glucoronyl transferases	MDP0000295443	 moderately similar to ( 298) AT1G03520 | Symbols:  | glycosyltransferase family 14 protein / core-2/I-branching enzyme family protein | chr1:877948-879286 REVERSEmoderately similar to ( 323) loc_os08g04790 12008.m04622 protein xylosyltransferase 2, putative, expressed   no original description 	T
35.2	not assigned.unknown	MDP0000280042	 highly similar to ( 931) AT2G40400 | Symbols:  | unknown protein | chr2:16869363-16872569 FORWARDhighly similar to ( 834) loc_os01g61120 12001.m12239 protein expressed protein   no original description 	T
35.2	not assigned.unknown	MDP0000174606	  no original description 	T
29.4.1.57	protein.postranslational modification.kinase.receptor like cytoplasmatic kinase VII	MDP0000783540	 weakly similar to ( 141) AT3G57830 | Symbols:  | leucine-rich repeat transmembrane protein kinase, putative | chr3:21419778-21422320 FORWARDvery weakly similar to (70.5) RPK1_IPONI Receptor-like protein kinase precursor (EC 2.7.11.1) - Ipomoea nil (Japanese morning glory) (Pharbitis nil)weakly similar to ( 125) loc_os08g39590 12008.m07940 protein ATP binding protein, putative, expressed no original description 	T
35.2	not assigned.unknown	MDP0000254535	 weakly similar to ( 181) AT1G02816 | Symbols:  | unknown protein | chr1:621637-622137 FORWARDweakly similar to ( 152) loc_os05g29900 12005.m07226 protein expressed protein    no original description 	T
31.1	cell.organisation	MDP0000681201	 highly similar to ( 828) AT1G50010 | Symbols: TUA2 | TUA2; structural constituent of cytoskeleton | chr1:18517737-18519729 FORWARDhighly similar to ( 831) TBA2_GOSHI Tubulin alpha-2 chain (Alpha-2 tubulin) - Gossypium hirsutum (Upland cotton)highly similar to ( 817) loc_os11g14220 12011.m28666 protein tubulin alpha-3 chain, putative, expressed   no original description 	T
8.1.6	TCA / org transformation.TCA.succinyl-CoA ligase	MDP0000304843	 highly similar to ( 654) AT2G20420 | Symbols:  | succinyl-CoA ligase (GDP-forming) beta-chain, mitochondrial, putative / succinyl-CoA synthetase, beta chain, putative / SCS-beta, putative | chr2:8805574-8807858 FORWARDhighly similar to ( 632) SUCB_ORYSA Succinyl-CoA ligase [GDP-forming] beta-chain, mitochondrial precursor (EC 6.2.1.4) (Succinyl-CoA synthetase, beta chain) (SCS-beta) - Oryza sativa (Rice)highly similar to ( 632) loc_os02g40830 12002.m100283 protein succinyl-CoA ligase beta-chain, mitochondrial precursor, putative, expressed  no original description 	T
9.6	mitochondrial electron transport / ATP synthesis.cytochrome c	MDP0000205322	 moderately similar to ( 214) AT1G22840 | Symbols: CYTC-1, ATCYTC-A | CYTC-1 (CYTOCHROME C-1); electron carrier/ heme binding / iron ion binding | chr1:8079384-8080246 FORWARDmoderately similar to ( 219) CYC_PHAAU Cytochrome c - Phaseolus aureus (Mung bean) (Vigna radiata)moderately similar to ( 212) loc_os05g34770 12005.m27830 protein cytochrome c, putative, expressed no original description 	T
35.2	not assigned.unknown	MDP0000208611	 moderately similar to ( 293) AT4G21110 | Symbols:  | G10 family protein | chr4:11267673-11268774 FORWARDmoderately similar to ( 294) BD31A_ORYSA Protein BUD31 homolog 1 (Protein G10 homolog 1) - Oryza sativa (Rice)moderately similar to ( 294) loc_os04g55290 12004.m101674 protein protein G10 homolog 1, putative, expressed  no original description 	T
26.9	misc.glutathione S transferases	MDP0000662880	 moderately similar to ( 271) AT1G10370 | Symbols: GST30, ATGSTU17, GST30B, ERD9 | ERD9 (EARLY-RESPONSIVE TO DEHYDRATION 9); glutathione transferase | chr1:3397274-3398273 REVERSEmoderately similar to ( 207) GSTU6_ORYSA Probable glutathione S-transferase GSTU6 (EC 2.5.1.18) (28 kDa cold-induced protein) - Oryza sativa (Rice)moderately similar to ( 219) loc_os10g38710 12010.m06639 protein glutathione S-transferase GSTU6, putative, expressed no original description 	T
27.3.55	RNA.regulation of transcription.HDA	MDP0000348722	 moderately similar to ( 232) AT3G18520 | Symbols: HDA15, ATHDA15 | HDA15; histone deacetylase | chr3:6361605-6365399 FORWARDmoderately similar to ( 207) loc_os07g06980 12007.m079613 protein histone deacetylase HDA110 isoform 1, putative, expressed no original description 	T
27.3.22	RNA.regulation of transcription.HB,Homeobox transcription factor family	MDP0000281696	 weakly similar to ( 178) AT1G27050 | Symbols: ATHB54 | ATHB54 (ARABIDOPSIS THALIANA HOMEOBOX PROTEIN 54); nucleic acid binding / transcription factor | chr1:9391893-9394408 FORWARDvery weakly similar to (75.9) loc_os01g56969 12001.m11854 protein nucleic acid binding protein, putative, expressed   no original description 	T
31.3	cell.cycle	MDP0000155259	 moderately similar to ( 363) AT5G67260 | Symbols: CYCD3;2 | CYCD3;2 (CYCLIN D3;2); cyclin-dependent protein kinase | chr5:26836313-26837665 FORWARDvery weakly similar to (56.2) CCNB2_ORYSA G2/mitotic-specific cyclin-2 (B-like cyclin) (CycOs2) - Oryza sativa (Rice)weakly similar to ( 167) loc_os09g29100 12009.m06061 protein cyclin delta-2, putative, expressed  no original description 	T
35.2	not assigned.unknown	MDP0000865961	 weakly similar to ( 116) AT1G52140 | Symbols:  | unknown protein | chr1:19407559-19408185 REVERSEweakly similar to ( 107) loc_os06g36070 12006.m08144 protein expressed protein  no original description 	T
29.4	protein.postranslational modification	MDP0000160039	 moderately similar to ( 433) AT3G04910 | Symbols: WNK1, ZIK4 | WNK1 (WITH NO LYSINE (K) 1); kinase/ protein kinase/ protein serine/threonine kinase | chr3:1356061-1358057 FORWARDvery weakly similar to (77.4) MPK10_ORYSA Mitogen-activated protein kinase 10 (EC 2.7.11.24) (MAP kinase 10) - Oryza sativa (Rice)moderately similar to ( 398) loc_os07g38530 12007.m29232 protein protein kinase domain containing protein, expressed  no original description 	T
27.3.59	RNA.regulation of transcription.Methyl binding domain proteins	MDP0000709648	 weakly similar to ( 144) AT1G22310 | Symbols: MBD8 | MBD8; methyl-CpG binding | chr1:7881714-7883644 REVERSEvery weakly similar to (92.0) loc_os09g29750 12009.m06126 protein expressed protein    PRK08026  no original description 	T
35.2	not assigned.unknown	MDP0000143025	 weakly similar to ( 108) AT1G20430 | Symbols:  | unknown protein | chr1:7083471-7083821 REVERSEvery weakly similar to (85.5) loc_os01g70550 12001.m43367 protein expressed protein  no original description 	T
29.2.1.2.1.3	protein.synthesis.ribosomal protein.eukaryotic.40S subunit.S3	MDP0000256937	 moderately similar to ( 403) AT5G35530 | Symbols:  | 40S ribosomal protein S3 (RPS3C) | chr5:13710355-13712192 REVERSEmoderately similar to ( 406) loc_os07g41750 12007.m29256 protein 40S ribosomal protein S3-A, putative, expressed  no original description 	T
35.2	not assigned.unknown	MDP0000300891	 very weakly similar to (64.3) AT1G21830 | Symbols:  | unknown protein | chr1:7661429-7662521 REVERSEvery weakly similar to (55.5) loc_os04g59420 12004.m101704 protein expressed protein  no original description 	T
35.1	not assigned.no ontology	MDP0000214577	 highly similar to ( 560) AT2G36840 | Symbols:  | ACT domain-containing protein | chr2:15451834-15453550 REVERSEhighly similar to ( 503) loc_os09g39740 12009.m22202 protein amino acid binding protein, putative, expressed   no original description 	T
35.2	not assigned.unknown	MDP0000262789	 very weakly similar to (62.8) AT2G46640 | Symbols:  | unknown protein | chr2:19148792-19150751 FORWARD  no original description 	T
35.2	not assigned.unknown	MDP0000344139	  no original description 	T
26.10	misc.cytochrome P450	MDP0000237148	 moderately similar to ( 235) AT4G31940 | Symbols: CYP82C4 | CYP82C4; electron carrier/ heme binding / iron ion binding / monooxygenase/ oxygen binding | chr4:15452040-15453966 FORWARDmoderately similar to ( 263) C82A1_PEA Cytochrome P450 82A1 (EC 1.14.-.-) (CYPLXXXII) (Fragment) - Pisum sativum (Garden pea)weakly similar to ( 157) loc_os09g26960 12009.m05848 protein flavonoid 3-monooxygenase, putative, expressed  no original description 	T
27.3.29	RNA.regulation of transcription.TCP transcription factor family	MDP0000320363	 weakly similar to ( 158) AT3G02150 | Symbols: PTF1, TFPD | PTF1 (PLASTID TRANSCRIPTION FACTOR 1); transcription factor | chr3:391522-392589 FORWARDweakly similar to ( 143) loc_os01g55750 12001.m43206 protein TCP family transcription factor containing protein, expressed   no original description 	T
28.1	DNA.synthesis/chromatin structure	MDP0000152995	 highly similar to ( 556) AT5G04130 | Symbols:  | DNA topoisomerase, ATP-hydrolyzing, putative / DNA topoisomerase II, putative / DNA gyrase, putative | chr5:1122084-1128031 REVERSEhighly similar to ( 552) GYRB_ORYSA DNA gyrase subunit B, chloroplast/mitochondrial precursor (EC 5.99.1.3) - Oryza sativa (Rice)highly similar to ( 552) loc_os01g16290 12001.m42965 protein DNA gyrase subunit B, putative, expressed   no original description 	T
1.1.5.2	PS.lightreaction.other electron carrier (ox/red).ferredoxin	MDP0000225335	 weakly similar to ( 185) AT2G27510 | Symbols: ATFD3 | ATFD3 (ferredoxin 3); 2 iron, 2 sulfur cluster binding / electron carrier/ iron-sulfur cluster binding | chr2:11758281-11758748 REVERSEweakly similar to ( 181) FER3_MAIZE Ferredoxin-3, chloroplast precursor (Ferredoxin III) (Fd III) - Zea mays (Maize)weakly similar to ( 177) loc_os05g37140 12005.m28058 protein ferredoxin-6, chloroplast precursor, putative, expressed   no original description 	T
30.2.3	signalling.receptor kinases.leucine rich repeat III	MDP0000237989	 moderately similar to ( 362) AT5G07620 | Symbols:  | protein kinase family protein | chr5:2407401-2409066 REVERSEmoderately similar to ( 204) VSPA_SOYBN Stem 28 kDa glycoprotein precursor (Vegetative storage protein A) - Glycine max (Soybean)moderately similar to ( 329) loc_os06g04790 12006.m05208 protein acid phosphatase 1 precursor, putative, expressed  no original description 	T
17.5.1	hormone metabolism.ethylene.synthesis-degradation	MDP0000309469	 moderately similar to ( 417) AT1G06650 | Symbols:  | 2-oxoglutarate-dependent dioxygenase, putative | chr1:2035909-2037186 FORWARDmoderately similar to ( 362) DV4H_CATRO Desacetoxyvindoline 4-hydroxylase (EC 1.14.11.20) - Catharanthus roseus (Rosy periwinkle) (Madagascar periwinkle)moderately similar to ( 340) loc_os04g10350 12004.m06287 protein 1-aminocyclopropane-1-carboxylate oxidase, putative, expressed  no original description 	T
31.1	cell.organisation	MDP0000150492	 highly similar to ( 788) AT1G68060 | Symbols: ATMAP70-1 | ATMAP70-1 (microtubule-associated proteins 70-1); microtubule binding | chr1:25511392-25514226 REVERSEhighly similar to ( 736) loc_os02g50320 12002.m10066 protein ATMAP70-3, putative, expressed   no original description 	T
20.2.1	stress.abiotic.heat	MDP0000129394	 moderately similar to ( 465) AT3G17830 | Symbols:  | DNAJ heat shock family protein | chr3:6101868-6104503 FORWARDweakly similar to ( 187) RL44_GOSHI 60S ribosomal protein L44 - Gossypium hirsutum (Upland cotton)moderately similar to ( 362) loc_os02g56040 12002.m10628 protein chaperone protein dnaJ, putative, expressed   no original description 	T
34.9	transport.metabolite transporters at the mitochondrial membrane	MDP0000137665	 highly similar to ( 556) AT5G14040 | Symbols:  | mitochondrial phosphate transporter | chr5:4531059-4532965 REVERSEvery weakly similar to (55.1) BT1_MAIZE Protein brittle-1, chloroplast precursor - Zea mays (Maize)highly similar to ( 541) loc_os02g52860 12002.m10316 protein phosphate carrier protein, mitochondrial precursor, putative, expressed  no original description 	T
35.2	not assigned.unknown	MDP0000316819	 weakly similar to ( 166) AT1G05205 | Symbols:  | unknown protein | chr1:1509352-1510249 REVERSEweakly similar to ( 177) loc_os06g46700 12006.m09196 protein expressed protein no original description 	T
35.2	not assigned.unknown	MDP0000930114	 highly similar to ( 611) AT4G25360 | Symbols:  | INVOLVED IN: biological_process unknown; EXPRESSED IN: 22 plant structures; EXPRESSED DURING: 13 growth stages; CONTAINS InterPro DOMAIN/s: Protein of unknown function DUF231, plant (InterPro:IPR004253); BEST Arabidopsis thaliana protein match is: YLS7 (TAIR:AT5G51640.1); Has 699 Blast hits to 688 proteins in 15 species: Archae - 0; Bacteria - 0; Metazoa - 0; Fungi - 0; Plants - 699; Viruses - 0; Other Eukaryotes - 0 (source: NCBI BLink). | chr4:12970187-12972404 FORWARDhighly similar to ( 581) loc_os01g68630 12001.m12965 protein expressed protein no original description 	T
16.2.1.10	secondary metabolism.phenylpropanoids.lignin biosynthesis.CAD	MDP0000874667	 highly similar to ( 598) AT5G51970 | Symbols:  | sorbitol dehydrogenase, putative / L-iditol 2-dehydrogenase, putative | chr5:21111820-21113284 FORWARDvery weakly similar to (95.9) MTDH_MESCR Probable mannitol dehydrogenase (EC 1.1.1.255) (NAD-dependent mannitol dehydrogenase) - Mesembryanthemum crystallinum (Common ice plant)highly similar to ( 560) loc_os08g43190 12008.m08293 protein sorbitol dehydrogenase, putative, expressed   no original description 	T
31.3	cell.cycle	MDP0000168026	 weakly similar to ( 128) AT2G42260 | Symbols: UVI4, PYM | UVI4 (UV-B-INSENSITIVE 4) | chr2:17602240-17603283 REVERSE no original description 	T
35.2	not assigned.unknown	MDP0000581775	 moderately similar to ( 234) AT4G31980 | Symbols:  | unknown protein | chr4:15464905-15469204 FORWARDweakly similar to ( 150) loc_os01g21710 12001.m08655 protein conserved hypothetical protein  no original description 	T
2.1.2.1	major CHO metabolism.synthesis.starch.AGPase	MDP0000252369	 highly similar to ( 735) AT1G27680 | Symbols: APL2 | APL2 (ADPGLC-PPASE LARGE SUBUNIT); glucose-1-phosphate adenylyltransferase | chr1:9631630-9634450 FORWARDhighly similar to ( 764) GLGL1_BETVU Glucose-1-phosphate adenylyltransferase large subunit, chloroplast precursor (EC 2.7.7.27) (ADP-glucose synthase) (ADP-glucose pyrophosphorylase) (AGPase S) (Alpha-D-glucose-1-phosphate adenyl transferase) - Beta vulgaris (Sugar beet)highly similar to ( 629) loc_os05g50380 12005.m083822 protein glucose-1-phosphate adenylyltransferase large subunit, chloroplast precursor, putative, expressed no original description 	T
27.3.9	RNA.regulation of transcription.C2C2(Zn) GATA transcription factor family	MDP0000190038	 weakly similar to ( 104) AT4G26150 | Symbols: CGA1 | CGA1 (CYTOKININ-RESPONSIVE GATA FACTOR 1); transcription factor | chr4:13253210-13254659 FORWARDvery weakly similar to (83.6) loc_os02g12790 12002.m06526 protein GATA transcription factor 20, putative, expressed   no original description 	T
26.2	misc.UDP glucosyl and glucoronyl transferases	MDP0000922600	 highly similar to ( 571) AT3G02350 | Symbols: GAUT9 | GAUT9 (Galacturonosyltransferase 9); polygalacturonate 4-alpha-galacturonosyltransferase/ transferase, transferring glycosyl groups / transferase, transferring hexosyl groups | chr3:479248-481178 FORWARDhighly similar to ( 504) loc_os02g29530 12002.m08096 protein transferase, transferring glycosyl groups, putative, expressed  no original description 	T
34.16	transport.ABC transporters and multidrug resistance systems	MDP0000282275	 moderately similar to ( 391) AT2G26910 | Symbols: PDR4, ATPDR4 | PDR4 (PLEIOTROPIC DRUG RESISTANCE 4); ATPase, coupled to transmembrane movement of substances | chr2:11481623-11487874 FORWARDmoderately similar to ( 367) PDR6_ORYSA Pleiotropic drug resistance protein 6 - Oryza sativa (Rice)moderately similar to ( 324) loc_os02g11760 12002.m06425 protein PDR5-like ABC transporter, putative, expressed  no original description 	T
30.2.17	signalling.receptor kinases.DUF 26	MDP0000602207	 moderately similar to ( 322) AT1G66920 | Symbols:  | serine/threonine protein kinase, putative | chr1:24965410-24967432 REVERSEweakly similar to ( 173) KPRO_MAIZE Putative receptor protein kinase ZmPK1 precursor (EC 2.7.11.1) - Zea mays (Maize)moderately similar to ( 367) loc_os01g02560 12001.m06898 protein Ser/Thr receptor-like kinase, putative, expressed VAR1  no original description 	T
16.1.4.5	secondary metabolism.isoprenoids.carotenoids.lycopene beta cyclase	MDP0000145663	 moderately similar to ( 500) AT3G10230 | Symbols: LYC | LYC (LYCOPENE CYCLASE); lycopene beta cyclase | chr3:3164340-3165449 REVERSEhighly similar to ( 714) CCS_CITSI Capsanthin/capsorubin synthase, chloroplast precursor (EC 5.3.99.8) - Citrus sinensis (Sweet orange)moderately similar to ( 472) loc_os02g09750 12002.m06272 protein lycopene beta cyclase, chloroplast precursor, putative, expressed  PRK08026  no original description 	T
30.2.17	signalling.receptor kinases.DUF 26	MDP0000177081	 moderately similar to ( 478) AT1G11410 | Symbols:  | S-locus protein kinase, putative | chr1:3841286-3844284 FORWARDmoderately similar to ( 204) NORK_MEDTR Nodulation receptor kinase precursor (EC 2.7.11.1) (Does not make infections protein 2) (Symbiosis receptor-like kinase) (MtSYMRK) - Medicago truncatula (Barrel medic)moderately similar to ( 422) loc_os07g36570 12007.m07908 protein KI domain interacting kinase 1, putative, expressed  no original description 	T
13.1.3.4	amino acid metabolism.synthesis.aspartate family.methionine	MDP0000310936	 highly similar to ( 738) AT1G02500 | Symbols: SAM1, SAM-1, MAT1 | SAM1 (S-ADENOSYLMETHIONINE SYNTHETASE 1); methionine adenosyltransferase | chr1:519037-520218 FORWARDhighly similar to ( 734) METK_CATRO S-adenosylmethionine synthetase 1 (EC 2.5.1.6) (Methionine adenosyltransferase 1) (AdoMet synthetase 1) - Catharanthus roseus (Rosy periwinkle) (Madagascar periwinkle)highly similar to ( 727) loc_os05g04510 12005.m27538 protein S-adenosylmethionine synthetase 1, putative, expressed  no original description 	T
29.5.3	protein.degradation.cysteine protease	MDP0000310811	 weakly similar to ( 124) AT3G12490 | Symbols: ATCYSB | cysteine protease inhibitor, putative / cystatin, putative | chr3:3960523-3961777 REVERSEweakly similar to ( 121) CYTI_VIGUN Cysteine proteinase inhibitor (Cystatin) - Vigna unguiculata (Cowpea)weakly similar to ( 122) loc_os01g16430 12001.m08239 protein multidomain cystatin, putative, expressed   no original description 	T
35.1	not assigned.no ontology	MDP0000669940	 weakly similar to ( 132) AT5G04820 | Symbols: ATOFP13, OFP13 | OFP13 (ARABIDOPSIS THALIANA OVATE FAMILY PROTEIN 13) | chr5:1399685-1400467 FORWARDweakly similar to ( 109) loc_os11g05770 12011.m04773 protein plant-specific domain TIGR01568 family protein, expressed  no original description 	T
35.1	not assigned.no ontology	MDP0000304097	 weakly similar to ( 187) AT3G11780 | Symbols:  | MD-2-related lipid recognition domain-containing protein / ML domain-containing protein | chr3:3724326-3725476 REVERSEweakly similar to ( 143) loc_os03g57420 12003.m10660 protein ML domain protein, putative, expressed no original description 	T
29.4	protein.postranslational modification	MDP0000159491	 moderately similar to ( 404) AT4G16360 | Symbols:  | AMP-activated protein kinase | chr4:9245662-9246833 FORWARDmoderately similar to ( 234) loc_os03g20340 12003.m07444 protein SNF1-related protein kinase regulatory subunit beta-1, putative, expressed   no original description 	T
29.7	protein.glycosylation	MDP0000149254	 highly similar to ( 607) AT1G32930 | Symbols:  | galactosyltransferase family protein | chr1:11931980-11934399 REVERSEhighly similar to ( 510) loc_os03g38050 12003.m08906 protein beta-1,3-galactosyltransferase sqv-2, putative, expressed  no original description 	T
17.3.1.2.6	hormone metabolism.brassinosteroid.synthesis-degradation.sterols.DWF7	MDP0000465581	 moderately similar to ( 477) AT3G02580 | Symbols: STE1, DWF7, BUL1 | STE1 (STEROL 1); C-5 sterol desaturase | chr3:547048-548615 FORWARDmoderately similar to ( 494) SC5D_TOBAC Delta(7)-sterol-C5(6)-desaturase (EC 1.3.3.-) (Delta-7-C-5 sterol desaturase) (Delta(7)-sterol-C5-desaturase) - Nicotiana tabacum (Common tobacco)moderately similar to ( 439) loc_os01g04260 12001.m07061 protein delta-7-sterol-C5, putative, expressed  no original description 	T
35.1	not assigned.no ontology	MDP0000941306	 weakly similar to ( 165) AT5G06600 | Symbols: UBP12 | UBP12 (UBIQUITIN-SPECIFIC PROTEASE 12); ubiquitin thiolesterase/ ubiquitin-specific protease | chr5:2020682-2027834 REVERSEweakly similar to ( 163) loc_os07g06950 12007.m05155 protein ubiquitin carboxyl-terminal hydrolase 7, putative, expressed  no original description 	T
29.5.11.20	protein.degradation.ubiquitin.proteasom	MDP0000176307	 moderately similar to ( 429) AT3G26340 | Symbols:  | 20S proteasome beta subunit E, putative | chr3:9650600-9652572 REVERSEmoderately similar to ( 444) PSB5_SPIOL Proteasome subunit beta type 5 precursor (EC 3.4.25.1) (20S proteasome subunit E) (Proteasome epsilon chain) - Spinacia oleracea (Spinach)moderately similar to ( 382) loc_os06g06030 12006.m31985 protein proteasome subunit beta type 5 precursor, putative, expressed no original description 	T
35.2	not assigned.unknown	MDP0000943161	  no original description 	T
27.3.35	RNA.regulation of transcription.bZIP transcription factor family	MDP0000198495	 very weakly similar to (89.0) AT5G44080 | Symbols:  | bZIP transcription factor family protein | chr5:17738787-17739734 REVERSEvery weakly similar to (50.4) CPRF2_PETCR Light-inducible protein CPRF-2 - Petroselinum crispum (Parsley) (Petroselinum hortense)very weakly similar to (97.8) loc_os01g64730 12001.m43299 protein G-box-binding factor 4, putative, expressed     no original description 	T
27.3.67	RNA.regulation of transcription.putative transcription regulator	MDP0000846874	 nearly identical (1327) AT1G02080 | Symbols:  | transcriptional regulator-related | chr1:373694-386682 FORWARDnearly identical (1102) loc_os10g40780 12010.m21965 protein transcription regulator, putative, expressed   no original description 	T
29.5.3	protein.degradation.cysteine protease	MDP0000179765	 moderately similar to ( 355) AT2G27420 | Symbols:  | cysteine proteinase, putative | chr2:11726311-11727519 REVERSEmoderately similar to ( 307) CYSP1_HORVU Cysteine proteinase EP-B 1 precursor (EC 3.4.22.-) - Hordeum vulgare (Barley)moderately similar to ( 343) loc_os12g17540 12012.m05711 protein vignain precursor, putative no original description 	T
26.7	misc.oxidases - copper, flavone etc	MDP0000283457	 highly similar to ( 928) AT5G20890 | Symbols:  | chaperonin, putative | chr5:7087020-7089906 REVERSEhighly similar to ( 584) ASO_BRANA L-ascorbate oxidase homolog precursor (EC 1.10.3.3) (Ascorbase) - Brassica napus (Rape)highly similar to ( 908) loc_os03g42220 12003.m35316 protein T-complex protein 1 subunit beta, putative, expressed   no original description 	T
23.4.2	nucleotide metabolism.phosphotransfer and pyrophosphatases.guanylate kinase	MDP0000235396	 highly similar to ( 525) AT3G57550 | Symbols: AGK2, GK-2 | AGK2 (GUANYLATE KINASE); guanylate kinase | chr3:21307269-21309193 FORWARDmoderately similar to ( 430) loc_os12g33100 12012.m07108 protein guanylate kinase, putative, expressed  no original description 	T
10.2.1	cell wall.cellulose synthesis.cellulose synthase	MDP0000184309	 highly similar to ( 623) AT2G32540 | Symbols: ATCSLB04, CSLB04, ATCSLB4 | ATCSLB04; cellulose synthase/ transferase/ transferase, transferring glycosyl groups | chr2:13814686-13818289 FORWARDhighly similar to ( 672) loc_os10g20090 12010.m21855 protein CSLH1 - cellulose synthase-like family H, expressed  no original description 	T
30.3	signalling.calcium	MDP0000859930	 moderately similar to ( 320) AT4G26470 | Symbols:  | calcium ion binding | chr4:13371377-13372197 FORWARDvery weakly similar to (70.1) CDPK2_MAIZE Calcium-dependent protein kinase 2 (EC 2.7.11.1) (CDPK 2) - Zea mays (Maize)moderately similar to ( 322) loc_os06g40720 12006.m08607 protein calcium ion binding protein, putative, expressed  no original description 	T
23.3.2.1	nucleotide metabolism.salvage.nucleoside kinases.adenosine kinase	MDP0000334473	 highly similar to ( 607) AT5G03300 | Symbols: ADK2 | ADK2 (ADENOSINE KINASE 2); adenosine kinase/ copper ion binding / kinase | chr5:796573-798997 FORWARDmoderately similar to ( 479) ADK_PHYPA Adenosine kinase (EC 2.7.1.20) (AK) (Adenosine 5'-phosphotransferase) - Physcomitrella patens (Moss)highly similar to ( 592) loc_os04g43750 12004.m101587 protein adenosine kinase 2, putative, expressed no original description 	T
29.3.4.3	protein.targeting.secretory pathway.vacuole	MDP0000256408	 moderately similar to ( 244) AT3G20210 | Symbols: DELTA-VPE, DELTAVPE | DELTA-VPE; cysteine-type endopeptidase | chr3:7052482-7054525 FORWARDmoderately similar to ( 246) VPE1_PHAVU Vacuolar-processing enzyme precursor (EC 3.4.22.-) (VPE) (Legumain-like proteinase) (LLP) - Phaseolus vulgaris (Kidney bean) (French bean)moderately similar to ( 246) loc_os01g37910 12001.m42664 protein vacuolar processing enzyme precursor, putative, expressed  no original description 	T
13.1.6.2.1	amino acid metabolism.synthesis.aromatic aa.phenylalanine and tyrosine.chorismate mutase	MDP0000179859	 moderately similar to ( 331) AT3G29200 | Symbols: CM1, ATCM1 | CM1 (CHORISMATE MUTASE 1); L-ascorbate peroxidase/ chorismate mutase | chr3:11164582-11166258 REVERSEmoderately similar to ( 287) loc_os01g55870 12001.m11743 protein chorismate mutase, chloroplast precursor, putative, expressed no original description 	T
18.4.5	Co-factor and vitamine metabolism.pantothenate.pantothenate kinase (PANK)	MDP0000863027	 highly similar to ( 610) AT4G35360 | Symbols:  | pantothenate kinase family protein | chr4:16812303-16814656 REVERSEhighly similar to ( 585) loc_os06g21980 12006.m091680 protein pantothenate kinase 4, putative, expressed  no original description 	T
26.2	misc.UDP glucosyl and glucoronyl transferases	MDP0000161200	 highly similar to ( 501) AT5G03795 | Symbols:  | LOCATED IN: membrane; EXPRESSED IN: embryo, sepal, flower; EXPRESSED DURING: C globular stage, petal differentiation and expansion stage; CONTAINS InterPro DOMAIN/s: Exostosin-like (InterPro:IPR004263); BEST Arabidopsis thaliana protein match is: exostosin family protein (TAIR:AT3G07620.1); Has 866 Blast hits to 860 proteins in 87 species: Archae - 0; Bacteria - 9; Metazoa - 265; Fungi - 4; Plants - 504; Viruses - 0; Other Eukaryotes - 84 (source: NCBI BLink). | chr5:1007554-1010373 REVERSEmoderately similar to ( 488) loc_os07g09050 12007.m28953 protein EXO, putative, expressed  no original description 	T
35.2	not assigned.unknown	MDP0000334210	 weakly similar to ( 131) AT4G30010 | Symbols:  | unknown protein | chr4:14672947-14673219 FORWARDvery weakly similar to (94.0) loc_os02g07910 12002.m100775 protein fiber protein Fb15, putative, expressed no original description 	T
27.3.99	RNA.regulation of transcription.unclassified	MDP0000286185	 weakly similar to ( 169) AT1G51200 | Symbols:  | zinc finger (AN1-like) family protein | chr1:18985690-18986211 FORWARDweakly similar to ( 104) ISAP1_ORYSA Multiple stress-responsive zinc-finger protein ISAP1 (Stress-associated protein 1) (OsISAP1) - Oryza sativa (Rice)weakly similar to ( 196) loc_os06g41010 12006.m31898 protein zinc finger A20 and AN1 domains-containing protein, putative, expressed no original description 	T
30.5	signalling.G-proteins	MDP0000175071	 moderately similar to ( 311) AT5G02040 | Symbols: PRA1.A1 | PRA1.A1 (PRENYLATED RAB ACCEPTOR 1.A1) | chr5:401163-402471 FORWARDmoderately similar to ( 332) loc_os01g62890 12001.m42756 protein PRA1 family protein, expressed  no original description 	T
35.2	not assigned.unknown	MDP0000343606	   no original description 	T
33.99	development.unspecified	MDP0000290562	 moderately similar to ( 322) AT3G55770 | Symbols:  | LIM domain-containing protein | chr3:20703799-20704614 FORWARDmoderately similar to ( 217) SF3_HELAN Pollen-specific protein SF3 - Helianthus annuus (Common sunflower)moderately similar to ( 315) loc_os03g15940 12003.m07031 protein pollen-specific protein SF3, putative, expressed no original description 	T
16.8.3.1	secondary metabolism.flavonoids.dihydroflavonols.dihydroflavonol 4-reductase	MDP0000283789	 moderately similar to ( 242) AT5G58490 | Symbols:  | cinnamoyl-CoA reductase family | chr5:23643068-23644455 FORWARDvery weakly similar to (84.3) DFRA_MAIZE Dihydroflavonol-4-reductase (EC 1.1.1.219) (DFR) (Dihydrokaempferol 4-reductase) - Zea mays (Maize)moderately similar to ( 209) loc_os03g60380 12003.m10928 protein dihydroflavonol-4-reductase, putative, expressed  no original description 	T
10.8.1	cell wall.pectin*esterases.PME	MDP0000196867	 highly similar to ( 674) AT5G09760 | Symbols:  | pectinesterase family protein | chr5:3032446-3034364 FORWARDmoderately similar to ( 286) PME2_CITSI Pectinesterase-2 precursor (EC 3.1.1.11) (Pectin methylesterase) (PE) - Citrus sinensis (Sweet orange)moderately similar to ( 333) loc_os04g35770 12004.m08666 protein pectinesterase-2 precursor, putative, expressed   no original description 	T
27.3.54	RNA.regulation of transcription.Histone acetyltransferases	MDP0000224377	 moderately similar to ( 445) AT5G56740 | Symbols: HAG2, HAC7, HAG02, HAC07 | HAG2 (HISTONE ACETYLTRANSFERASE OF THE GNAT FAMILY 2); H4 histone acetyltransferase/ histone acetyltransferase | chr5:22953009-22955577 REVERSEmoderately similar to ( 353) HAT1_ORYSA Probable acetyltransferase type B catalytic subunit (EC 2.3.1.48) (HAT B) - Oryza sativa (Rice)moderately similar to ( 353) loc_os09g17850 12009.m05043 protein acetyltransferase type B catalytic subunit, putative, expressed MSP1_C  no original description 	T
27.3.7	RNA.regulation of transcription.C2C2(Zn) CO-like, Constans-like zinc finger family	MDP0000609638	 weakly similar to ( 140) AT3G02380 | Symbols: COL2 | COL2 (constans-like 2); transcription factor/ zinc ion binding | chr3:487438-488624 REVERSEweakly similar to ( 126) loc_os06g16370 12006.m06350 protein zinc finger protein CONSTANS, putative, expressed no original description 	T
33.99	development.unspecified	MDP0000609638	 weakly similar to ( 140) AT3G02380 | Symbols: COL2 | COL2 (constans-like 2); transcription factor/ zinc ion binding | chr3:487438-488624 REVERSEweakly similar to ( 126) loc_os06g16370 12006.m06350 protein zinc finger protein CONSTANS, putative, expressed no original description 	T
29.5.11.20	protein.degradation.ubiquitin.proteasom	MDP0000221892	 nearly identical (1476) AT2G32730 | Symbols:  | 26S proteasome regulatory subunit, putative | chr2:13880189-13885464 FORWARDnearly identical (1303) loc_os08g12820 12008.m26623 protein 26S proteasome non-ATPase regulatory subunit 1, putative, expressed   no original description 	T
29.2.4	protein.synthesis.elongation	MDP0000667525	 nearly identical (1276) AT1G56070 | Symbols: LOS1 | LOS1; copper ion binding / translation elongation factor/ translation factor, nucleic acid binding | chr1:20968245-20971077 REVERSEnearly identical (1258) EF2_BETVU Elongation factor 2 (EF-2) - Beta vulgaris (Sugar beet)nearly identical (1264) loc_os02g32030 12002.m08296 protein elongation factor 2, putative, expressed   no original description 	T
29.3.99	protein.targeting.unknown	MDP0000120758	 weakly similar to ( 156) AT4G25370 | Symbols:  | Clp amino terminal domain-containing protein | chr4:12972747-12974580 FORWARDvery weakly similar to (50.8) CLPA_BRANA ATP-dependent Clp protease ATP-binding subunit clpA homolog, chloroplast precursor (Fragment) - Brassica napus (Rape)moderately similar to ( 203) loc_os03g14280 12003.m06870 protein ATP-dependent Clp protease ATP-binding subunit clpA, putative, expressed    PRK06958  no original description 	T
14.1	S-assimilation.ATPS	MDP0000263161	 highly similar to ( 770) AT3G22890 | Symbols: APS1 | APS1 (ATP SULFURYLASE 1); sulfate adenylyltransferase (ATP) | chr3:8112837-8114734 FORWARDhighly similar to ( 682) loc_os03g53230 12003.m10296 protein bifunctional 3-phosphoadenosine 5-phosphosulfate synthetase, putative, expressed  no original description 	T
27.3.24	RNA.regulation of transcription.MADS box transcription factor family	MDP0000259294	 weakly similar to ( 102) AT4G24540 | Symbols: AGL24 | AGL24 (AGAMOUS-LIKE 24); protein binding / protein heterodimerization/ protein homodimerization/ sequence-specific DNA binding / transcription factor | chr4:12671160-12673645 REVERSEvery weakly similar to (90.5) MAD47_ORYSA MADS-box transcription factor 47 (OsMADS47) - Oryza sativa (Rice)very weakly similar to (94.7) loc_os03g08754 12003.m35564 protein MADS-box transcription factor 47, putative, expressed   no original description 	T
35.2	not assigned.unknown	MDP0000947568	 weakly similar to ( 177) AT4G16450 | Symbols:  | unknown protein | chr4:9280132-9280541 FORWARDweakly similar to ( 186) loc_os08g33460 12008.m07336 protein expressed protein no original description 	T
29.2.1.2.1.25	protein.synthesis.ribosomal protein.eukaryotic.40S subunit.S25	MDP0000576521	 weakly similar to ( 136) AT2G21580 | Symbols:  | 40S ribosomal protein S25 (RPS25B) | chr2:9236629-9237510 FORWARDweakly similar to ( 121) loc_os08g44480 12008.m62036 protein 40S ribosomal protein S25-1, putative, expressed   no original description 	T
26.2	misc.UDP glucosyl and glucoronyl transferases	MDP0000730619	 moderately similar to ( 293) AT5G11130 | Symbols:  | exostosin family protein | chr5:3543622-3544724 FORWARDmoderately similar to ( 238) loc_os02g39960 12002.m09082 protein exostosin family protein, putative  no original description 	T
29.5.11	protein.degradation.ubiquitin	MDP0000220016	 highly similar to ( 680) AT1G78880 | Symbols:  | balbiani ring 1-related / BR1-related | chr1:29653068-29654819 REVERSEhighly similar to ( 695) loc_os08g13350 12008.m26625 protein cysteine-type endopeptidase/ ubiquitin thiolesterase, putative, expressed  no original description 	T
9.7	mitochondrial electron transport / ATP synthesis.cytochrome c oxidase	MDP0000263444	 weakly similar to ( 134) AT1G22450 | Symbols: COX6B, ATCOX6B2 | COX6B (CYTOCHROME C OXIDASE 6B); cytochrome-c oxidase | chr1:7925447-7926918 FORWARDweakly similar to ( 137) loc_os03g27290 12003.m08056 protein cytochrome c oxidase subunit, putative, expressed no original description 	T
26.9	misc.glutathione S transferases	MDP0000065895	 moderately similar to ( 318) AT2G30860 | Symbols: ATGSTF9, GLUTTR, ATGSTF7 | ATGSTF9 (GLUTATHIONE S-TRANSFERASE PHI 9); glutathione peroxidase/ glutathione transferase | chr2:13139132-13140079 FORWARDweakly similar to ( 179) GSTF1_TOBAC Glutathione S-transferase PARB (EC 2.5.1.18) (GST class-phi) - Nicotiana tabacum (Common tobacco)weakly similar to ( 196) loc_os03g04260 12003.m05957 protein glutathione S-transferase, putative, expressed  no original description 	T
35.2	not assigned.unknown	MDP0000347851	 weakly similar to ( 122) AT4G15802 | Symbols:  | FUNCTIONS IN: molecular_function unknown; INVOLVED IN: biological_process unknown; LOCATED IN: cellular_component unknown; CONTAINS InterPro DOMAIN/s: Heat shock factor binding 1 (InterPro:IPR009643); Has 178 Blast hits to 178 proteins in 62 species: Archae - 0; Bacteria - 0; Metazoa - 122; Fungi - 0; Plants - 37; Viruses - 0; Other Eukaryotes - 19 (source: NCBI BLink). | chr4:8986864-8988339 REVERSEvery weakly similar to (92.8) loc_os09g20830 12009.m60264 protein heat shock factor-binding protein 1, putative, expressed no original description 	T
35.2	not assigned.unknown	MDP0000285731	 very weakly similar to (90.1) AT4G23496 | Symbols: SP1L5 | SP1L5 (SPIRAL1-LIKE5) | chr4:12257914-12258297 REVERSEvery weakly similar to (75.5) loc_os03g30430 12003.m34882 protein NAP16kDa protein, putative, expressed no original description 	T
35.1	not assigned.no ontology	MDP0000441695	 moderately similar to ( 279) AT3G11660 | Symbols: NHL1 | NHL1 | chr3:3679031-3679660 REVERSEmoderately similar to ( 202) loc_os11g05860 12011.m04782 protein VAMP protein SEC22, putative, expressed no original description 	T
35.2	not assigned.unknown	MDP0000131022	 moderately similar to ( 299) AT4G32140 | Symbols:  | FUNCTIONS IN: molecular_function unknown; INVOLVED IN: biological_process unknown; LOCATED IN: endomembrane system, membrane; EXPRESSED IN: 24 plant structures; EXPRESSED DURING: 13 growth stages; CONTAINS InterPro DOMAIN/s: Protein of unknown function DUF6, transmembrane (InterPro:IPR000620); BEST Arabidopsis thaliana protein match is: membrane protein (TAIR:AT3G07080.1); Has 1078 Blast hits to 1078 proteins in 273 species: Archae - 20; Bacteria - 294; Metazoa - 244; Fungi - 147; Plants - 41; Viruses - 0; Other Eukaryotes - 332 (source: NCBI BLink). | chr4:15523037-15525195 REVERSEmoderately similar to ( 292) loc_os04g55260 12004.m10418 protein vacuolar membrane protein, putative, expressed  no original description 	T
12.2.2	N-metabolism.ammonia metabolism.glutamine synthetase	MDP0000083035	 nearly identical (1119) AT3G53180 | Symbols:  | catalytic/ glutamate-ammonia ligase | chr3:19707068-19711188 FORWARDnearly identical (1013) loc_os10g31820 12010.m06033 protein protein fluG, putative, expressed COG3889  no original description 	T
31.3	cell.cycle	MDP0000893879	 moderately similar to ( 206) AT3G63120 | Symbols: CYCP1;1 | CYCP1;1 (cyclin p1;1); cyclin-dependent protein kinase | chr3:23323143-23323893 REVERSEmoderately similar to ( 224) loc_os05g33040 12005.m083714 protein PREG-like protein, putative, expressed  no original description 	T
35.2	not assigned.unknown	MDP0000363273	 weakly similar to ( 114) AT3G08610 | Symbols:  | unknown protein | chr3:2616435-2616768 REVERSEweakly similar to ( 105) loc_os05g40300 12005.m83869 protein expressed protein no original description 	T
26.9	misc.glutathione S transferases	MDP0000122993	 moderately similar to ( 326) AT1G78380 | Symbols: ATGSTU19, GST8 | ATGSTU19 (GLUTATHIONE S-TRANSFERASE TAU 19); glutathione binding / glutathione transferase | chr1:29486659-29487819 REVERSEmoderately similar to ( 358) GSTX4_TOBAC Probable glutathione S-transferase (EC 2.5.1.18) (Auxin-induced protein PCNT107) - Nicotiana tabacum (Common tobacco)moderately similar to ( 256) loc_os03g57200 12003.m10639 protein glutathione S-transferase parA, putative, expressed  no original description 	T
35.2	not assigned.unknown	MDP0000243217	 very weakly similar to (89.0) AT1G69230 | Symbols: SP1L2 | SP1L2 (SPIRAL1-LIKE2) | chr1:26026543-26027085 REVERSEvery weakly similar to (89.4) loc_os11g41150 12011.m07958 protein SPR1, putative, expressed  no original description 	T
29.3.2	protein.targeting.mitochondria	MDP0000204238	 weakly similar to ( 129) AT2G29530 | Symbols:  | mitochondrial import inner membrane translocase (TIM10) | chr2:12641026-12642243 REVERSEweakly similar to ( 135) loc_os03g61019 12003.m101635 protein mitochondrial import inner membrane translocase subunit Tim10, putative, expressed no original description 	T
35.2	not assigned.unknown	MDP0000470756	 highly similar to ( 668) AT1G55360 | Symbols:  | unknown protein | chr1:20666526-20668502 REVERSEhighly similar to ( 652) loc_os07g10530 12007.m05508 protein carboxyl-terminal peptidase, putative, expressed   no original description 	T
16.2.1.10	secondary metabolism.phenylpropanoids.lignin biosynthesis.CAD	MDP0000037251	 moderately similar to ( 458) AT4G39330 | Symbols: ATCAD9, CAD9 | CAD9 (CINNAMYL ALCOHOL DEHYDROGENASE 9); binding / catalytic/ oxidoreductase/ zinc ion binding | chr4:18291268-18292740 FORWARDhighly similar to ( 506) MTDH_FRAAN Probable mannitol dehydrogenase (EC 1.1.1.255) (NAD-dependent mannitol dehydrogenase) - Fragaria ananassa (Strawberry)moderately similar to ( 424) loc_os10g29470 12010.m05819 protein mannitol dehydrogenase, putative, expressed  no original description 	T
34.8	transport.metabolite transporters at the envelope membrane	MDP0000139910	 highly similar to ( 507) AT1G61800 | Symbols: GPT2 | GPT2; antiporter/ glucose-6-phosphate transmembrane transporter | chr1:22824527-22826459 FORWARDmoderately similar to ( 243) TPT_SPIOL Triose phosphate/phosphate translocator, chloroplast precursor (cTPT) (p36) (E29) - Spinacia oleracea (Spinach)moderately similar to ( 486) loc_os08g08840 12008.m080099 protein glucose-6-phosphate/phosphate translocator 2, chloroplast precursor, putative, expressed  no original description 	T
26.4.1	misc.beta 1,3 glucan hydrolases.glucan endo-1,3-beta-glucosidase	MDP0000120761	 highly similar to ( 608) AT3G13560 | Symbols:  | glycosyl hydrolase family 17 protein | chr3:4425484-4427284 REVERSEmoderately similar to ( 212) E13B_WHEAT Glucan endo-1,3-beta-glucosidase precursor (EC 3.2.1.39) ((1->3)-beta-glucan endohydrolase) ((1->3)-beta-glucanase) (Beta-1,3-endoglucanase) - Triticum aestivum (Wheat)moderately similar to ( 479) loc_os07g38930 12007.m08137 protein glucan endo-1,3-beta-glucosidase 4 precursor, putative, expressed   no original description 	T
35.2	not assigned.unknown	MDP0000287049	   no original description 	T
35.2	not assigned.unknown	MDP0000239596	 weakly similar to ( 153) AT3G07760 | Symbols:  | FUNCTIONS IN: molecular_function unknown; INVOLVED IN: biological_process unknown; LOCATED IN: cellular_component unknown; EXPRESSED IN: 24 plant structures; EXPRESSED DURING: 15 growth stages; CONTAINS InterPro DOMAIN/s: Sterile alpha motif SAM (InterPro:IPR001660), Sterile alpha motif homology (InterPro:IPR010993), Sterile alpha motif homology 2 (InterPro:IPR011510); Has 31 Blast hits to 31 proteins in 12 species: Archae - 0; Bacteria - 0; Metazoa - 4; Fungi - 6; Plants - 19; Viruses - 0; Other Eukaryotes - 2 (source: NCBI BLink). | chr3:2476723-2477947 FORWARDweakly similar to ( 159) loc_os12g32992 12012.m07097 protein expressed protein  no original description 	T
26.10	misc.cytochrome P450	MDP0000147814	 highly similar to ( 580) AT3G14610 | Symbols: CYP72A7 | CYP72A7; electron carrier/ heme binding / iron ion binding / monooxygenase/ oxygen binding | chr3:4912565-4914503 FORWARDhighly similar to ( 571) C72A1_CATRO Cytochrome P450 72A1 (EC 1.3.3.9) (CYPLXXII) (Secologanin synthase) (SLS) - Catharanthus roseus (Rosy periwinkle) (Madagascar periwinkle)highly similar to ( 523) loc_os01g43710 12001.m10633 protein cytochrome P450 72A1, putative, expressed  CobN  no original description 	T
27.3.35	RNA.regulation of transcription.bZIP transcription factor family	MDP0000299504	 moderately similar to ( 455) AT2G40950 | Symbols: BZIP17 | BZIP17; DNA binding / transcription activator/ transcription factor | chr2:17087823-17090326 REVERSEweakly similar to ( 120) TGA1B_TOBAC TGACG-sequence-specific DNA-binding protein TGA-1B (TGA1b) (HSBF) (Fragment) - Nicotiana tabacum (Common tobacco)moderately similar to ( 296) loc_os05g34050 12005.m07637 protein DNA binding protein, putative, expressed   PRK08026  no original description 	T
34.99	transport.misc	MDP0000159221	 moderately similar to ( 471) AT1G19800 | Symbols: TGD1 | TGD1 (TRIGALACTOSYLDIACYLGLYCEROL 1); lipid transporter | chr1:6846812-6847954 FORWARDmoderately similar to ( 393) loc_os04g46700 12004.m35587 protein expressed protein  no original description 	T
35.2	not assigned.unknown	MDP0000626880	  no original description 	T
35.2	not assigned.unknown	MDP0000320910	 moderately similar to ( 208) AT5G28490 | Symbols: LSH1 | LSH1 (LIGHT-DEPENDENT SHORT HYPOCOTYLS 1) | chr5:10454541-10455113 REVERSEweakly similar to ( 191) loc_os10g33780 12010.m06207 protein expressed protein  no original description 	T
13.2.4.4	amino acid metabolism.degradation.branched chain group.leucine	MDP0000225981	 highly similar to ( 603) AT3G45300 | Symbols: IVD, ATIVD | IVD (ISOVALERYL-COA-DEHYDROGENASE); ATP binding / isovaleryl-CoA dehydrogenase | chr3:16621659-16624848 REVERSEhighly similar to ( 605) IVD2_SOLTU Isovaleryl-CoA dehydrogenase 2, mitochondrial precursor (EC 1.3.99.10) (IVD 2) (Fragment) - Solanum tuberosum (Potato)highly similar to ( 612) loc_os05g03480 12005.m083606 protein isovaleryl-CoA dehydrogenase, mitochondrial precursor, putative, expressed no original description 	T
20.2.99	stress.abiotic.unspecified	MDP0000742278	 weakly similar to ( 198) AT4G08685 | Symbols: SAH7 | SAH7 | chr4:5550563-5551259 FORWARDweakly similar to ( 129) PSC13_MAIZE Pollen-specific protein C13 precursor - Zea mays (Maize)weakly similar to ( 155) loc_os10g22590 12010.m05266 protein pollen-specific protein C13 precursor, putative, expressed  no original description 	T
8.1.1.2	TCA / org transformation.TCA.pyruvate DH.E2	MDP0000215942	 moderately similar to ( 216) AT1G34430 | Symbols: EMB3003 | EMB3003 (embryo defective 3003); acyltransferase/ dihydrolipoyllysine-residue acetyltransferase/ protein binding | chr1:12588027-12590084 REVERSEweakly similar to ( 184) loc_os12g08170 12012.m04804 protein dihydrolipoyllysine-residue acetyltransferase component of pyruvatedehydrogenase complex, mitochondrial precursor, putative, expressed   no original description 	T
30.2.3	signalling.receptor kinases.leucine rich repeat III	MDP0000242000	 moderately similar to ( 309) AT3G17840 | Symbols: RLK902 | RLK902; ATP binding / kinase/ protein serine/threonine kinase | chr3:6106092-6108430 FORWARDweakly similar to ( 137) PSKR_DAUCA Phytosulfokine receptor precursor (EC 2.7.11.1) (Phytosulfokine LRR receptor kinase) - Daucus carota (Carrot)moderately similar to ( 332) loc_os03g12250 12003.m06675 protein atypical receptor-like kinase MARK, putative, expressed no original description 	T
26.3.2	misc.gluco-, galacto- and mannosidases.beta-galactosidase	MDP0000188563	 nearly identical (1234) AT4G36360 | Symbols: BGAL3 | BGAL3 (beta-galactosidase 3); beta-galactosidase/ catalytic/ cation binding / sugar binding | chr4:17176840-17181143 REVERSEnearly identical (1042) BGAL_ASPOF Beta-galactosidase precursor (EC 3.2.1.23) (Lactase) - Asparagus officinalis (Garden asparagus)nearly identical (1233) loc_os03g06940 12003.m35030 protein beta-galactosidase precursor, putative, expressed   no original description 	T
23.3.1.1	nucleotide metabolism.salvage.phosphoribosyltransferases.aprt	MDP0000197456	 moderately similar to ( 310) AT1G27450 | Symbols: APT1, ATAPT1 | APT1 (ADENINE PHOSPHORIBOSYL TRANSFERASE 1); adenine phosphoribosyltransferase | chr1:9532421-9533807 FORWARDmoderately similar to ( 280) APT1_WHEAT Adenine phosphoribosyltransferase 1 (EC 2.4.2.7) (APRT 1) - Triticum aestivum (Wheat)moderately similar to ( 285) loc_os12g39860 12012.m07770 protein adenine phosphoribosyltransferase 1, putative, expressed   no original description 	T
35.1	not assigned.no ontology	MDP0000949756	 moderately similar to ( 374) AT3G17380 | Symbols:  | meprin and TRAF homology domain-containing protein / MATH domain-containing protein | chr3:5950240-5952124 FORWARDvery weakly similar to (92.4) loc_os07g06950 12007.m05155 protein ubiquitin carboxyl-terminal hydrolase 7, putative, expressed no original description 	T
20.2.99	stress.abiotic.unspecified	MDP0000266399	 weakly similar to ( 107) AT4G08685 | Symbols: SAH7 | SAH7 | chr4:5550563-5551259 FORWARDvery weakly similar to (72.8) OLEE1_BETVE Olee1-like protein precursor - Betula verrucosa (White birch) (Betula pendula)very weakly similar to (89.7) loc_os10g22590 12010.m05266 protein pollen-specific protein C13 precursor, putative, expressed  no original description 	T
29.2.1.2.2.535	protein.synthesis.ribosomal protein.eukaryotic.60S subunit.L35A	MDP0000199487	 moderately similar to ( 204) AT1G74270 | Symbols:  | 60S ribosomal protein L35a (RPL35aC) | chr1:27928415-27929466 REVERSEweakly similar to ( 189) loc_os02g54470 12002.m100389 protein 60S ribosomal protein L33-B, putative, expressed  no original description 	T
29.2.1.2.2.38	protein.synthesis.ribosomal protein.eukaryotic.60S subunit.L38	MDP0000593964	 weakly similar to ( 119) AT3G59540 | Symbols:  | 60S ribosomal protein L38 (RPL38B) | chr3:21995897-21996742 REVERSEweakly similar to ( 125) loc_os11g24610 12011.m06387 protein 60S ribosomal protein L38, putative, expressed no original description 	T
1.2.2	PS.photorespiration.glycolate oxydase	MDP0000917325	 highly similar to ( 616) AT3G14420 | Symbols:  | (S)-2-hydroxy-acid oxidase, peroxisomal, putative / glycolate oxidase, putative / short chain alpha-hydroxy acid oxidase, putative | chr3:4821899-4823899 FORWARDhighly similar to ( 610) GOX_SPIOL Peroxisomal (S)-2-hydroxy-acid oxidase (EC 1.1.3.15) (Glycolate oxidase) (GOX) (Short chain alpha-hydroxy acid oxidase) - Spinacia oleracea (Spinach)highly similar to ( 590) loc_os07g05820 12007.m29070 protein hydroxyacid oxidase 1, putative, expressed  no original description 	T
29.4	protein.postranslational modification	MDP0000313460	 highly similar to ( 571) AT5G58380 | Symbols: CIPK10, PKS2, SIP1, SNRK3.8 | SIP1 (SOS3-INTERACTING PROTEIN 1); ATP binding / kinase/ protein kinase/ protein serine/threonine kinase | chr5:23597092-23598531 REVERSEmoderately similar to ( 410) CIPK1_ORYSA CIPK-like protein 1 (EC 2.7.11.1) (OsCK1) - Oryza sativa (Rice)highly similar to ( 555) loc_os05g26820 12005.m06974 protein CBL-interacting serine/threonine-protein kinase 15, putative, expressed  no original description 	T
35.1	not assigned.no ontology	MDP0000176907	 weakly similar to ( 159) AT5G22870 | Symbols:  | harpin-induced protein-related / HIN1-related / harpin-responsive protein-related | chr5:7647056-7647679 REVERSEvery weakly similar to (87.4) loc_os02g44670 12002.m09507 protein harpin-induced 1, putative, expressed no original description 	T
4.1.10	glycolysis.cytosolic branch.aldolase	MDP0000329229	 weakly similar to ( 124) AT3G52930 | Symbols:  | fructose-bisphosphate aldolase, putative | chr3:19627383-19628874 REVERSEweakly similar to ( 130) ALF_ORYSA Fructose-bisphosphate aldolase cytoplasmic isozyme (EC 4.1.2.13) (Gravity-specific protein GSC 233) - Oryza sativa (Rice)weakly similar to ( 130) loc_os05g33380 12005.m07570 protein fructose-bisphosphate aldolase cytoplasmic isozyme, putative, expressed no original description 	T
30.11	signalling.light	MDP0000905414	 highly similar to ( 780) AT1G67900 | Symbols:  | phototropic-responsive NPH3 family protein | chr1:25467737-25469888 FORWARDmoderately similar to ( 306) NPH3_ORYSA Coleoptile phototropism protein 1 (Non-phototropic hypocotyl 3-like protein) (NPH3-like protein) - Oryza sativa (Rice)moderately similar to ( 455) loc_os02g38120 12002.m08900 protein photoreceptor-interacting protein-like, putative, expressed   no original description 	T
10.1.5	cell wall.precursor synthesis.UXS	MDP0000786569	 highly similar to ( 614) AT3G46440 | Symbols: UXS5 | UXS5; UDP-glucuronate decarboxylase/ catalytic | chr3:17089268-17091611 REVERSEweakly similar to ( 108) GME2_ORYSA GDP-mannose 3,5-epimerase 2 (EC 5.1.3.18) (GDP-Man 3,5-epimerase 2) - Oryza sativa (Rice)highly similar to ( 604) loc_os03g16980 12003.m34836 protein UDP-glucuronic acid decarboxylase 1, putative, expressed  no original description 	T
29.5.11.4.2	protein.degradation.ubiquitin.E3.RING	MDP0000508748	 weakly similar to ( 181) AT5G02750 | Symbols:  | zinc finger (C3HC4-type RING finger) family protein | chr5:620101-620952 FORWARDweakly similar to ( 102) loc_os05g33830 12005.m07615 protein zinc finger, C3HC4 type family protein, expressed  TrbL  no original description 	T
1.1.4.4	PS.lightreaction.ATP synthase.gamma chain	MDP0000385730	 highly similar to ( 567) AT4G04640 | Symbols: ATPC1 | ATPC1; enzyme regulator | chr4:2350761-2351882 REVERSEhighly similar to ( 592) ATPG_TOBAC ATP synthase gamma chain, chloroplast precursor (EC 3.6.3.14) - Nicotiana tabacum (Common tobacco)moderately similar to ( 483) loc_os07g32880 12007.m07550 protein ATP synthase gamma chain, chloroplast precursor, putative, expressed  no original description 	T
26.28	misc.GDSL-motif lipase	MDP0000175342	 moderately similar to ( 435) AT2G42990 | Symbols:  | GDSL-motif lipase/hydrolase family protein | chr2:17879056-17880200 FORWARDmoderately similar to ( 210) APG_BRANA Anter-specific proline-rich protein APG (Protein CEX) (Fragment) - Brassica napus (Rape)moderately similar to ( 363) loc_os06g43044 12006.m08834 protein anther-specific proline-rich protein APG, putative, expressed   no original description 	T
26.1	misc.misc2	MDP0000278072	 weakly similar to ( 137) AT3G23600 | Symbols:  | dienelactone hydrolase family protein | chr3:8473833-8475655 FORWARDvery weakly similar to (57.0) E134_MAIZE Endo-1,3;1,4-beta-D-glucanase precursor (EC 3.2.1.-) - Zea mays (Maize)weakly similar to ( 127) loc_os05g33100 12005.m07542 protein endo-1,3;1,4-beta-D-glucanase precursor, putative, expressed no original description 	T
29.2.1.2.2.8	protein.synthesis.ribosomal protein.eukaryotic.60S subunit.L8	MDP0000191839	 moderately similar to ( 496) AT4G36130 | Symbols:  | 60S ribosomal protein L8 (RPL8C) | chr4:17097613-17098656 FORWARDmoderately similar to ( 478) RL8_TOBAC 60S ribosomal protein L8 (60S ribosomal protein L2) - Nicotiana tabacum (Common tobacco)moderately similar to ( 490) loc_os12g38000 12012.m07586 protein 60S ribosomal protein L2, putative, expressed   no original description 	T
35.2	not assigned.unknown	MDP0000596604	 weakly similar to ( 163) AT1G57540 | Symbols:  | unknown protein | chr1:21310478-21311215 REVERSEweakly similar to ( 159) loc_os01g14790 12001.m08081 protein expressed protein no original description 	T
35.2	not assigned.unknown	MDP0000124063	 weakly similar to ( 164) AT2G27830 | Symbols:  | FUNCTIONS IN: molecular_function unknown; EXPRESSED IN: 22 plant structures; EXPRESSED DURING: 13 growth stages; BEST Arabidopsis thaliana protein match is: pentatricopeptide (PPR) repeat-containing protein (TAIR:AT4G22760.1); Has 68 Blast hits to 68 proteins in 11 species: Archae - 0; Bacteria - 0; Metazoa - 0; Fungi - 0; Plants - 68; Viruses - 0; Other Eukaryotes - 0 (source: NCBI BLink). | chr2:11860734-11861306 FORWARDweakly similar to ( 111) loc_os04g33310 12004.m08426 protein expressed protein  no original description 	T
24.2	Biodegradation of Xenobiotics.lactoylglutathione lyase	MDP0000893382	 moderately similar to ( 217) AT2G32090 | Symbols:  | lactoylglutathione lyase family protein / glyoxalase I family protein | chr2:13644720-13645608 FORWARDweakly similar to ( 138) loc_os03g45720 12003.m09595 protein lactoylglutathione lyase, putative, expressed  no original description 	T
29.2.1.1.1.1.31	protein.synthesis.ribosomal protein.prokaryotic.chloroplast.30S subunit.S31	MDP0000767901	 very weakly similar to (95.9) AT2G38140 | Symbols: PSRP4 | PSRP4 (PLASTID-SPECIFIC RIBOSOMAL PROTEIN 4); structural constituent of ribosome | chr2:15980948-15981459 FORWARDvery weakly similar to (67.8) RR31_SPIOL 30S ribosomal protein S31, chloroplast precursor (Plastid-specific 30S ribosomal protein 4) (PSRP-4) - Spinacia oleracea (Spinach)very weakly similar to (75.9) loc_os05g09400 12005.m27555 protein PSRP4, putative, expressed no original description 	T
30.2.17	signalling.receptor kinases.DUF 26	MDP0000212605	 highly similar to ( 815) AT4G27290 | Symbols:  | ATP binding / protein kinase/ protein serine/threonine kinase/ protein tyrosine kinase/ sugar binding | chr4:13666281-13669202 FORWARDmoderately similar to ( 355) SLSG6_BRAOL S-locus-specific glycoprotein S6 precursor (SLSG-6) - Brassica oleracea (Wild cabbage)highly similar to ( 657) loc_os01g57560 12001.m11907 protein serine/threonine-protein kinase receptor precursor, putative, expressed  no original description 	T
30.2.24	signalling.receptor kinases.S-locus glycoprotein like	MDP0000212605	 highly similar to ( 815) AT4G27290 | Symbols:  | ATP binding / protein kinase/ protein serine/threonine kinase/ protein tyrosine kinase/ sugar binding | chr4:13666281-13669202 FORWARDmoderately similar to ( 355) SLSG6_BRAOL S-locus-specific glycoprotein S6 precursor (SLSG-6) - Brassica oleracea (Wild cabbage)highly similar to ( 657) loc_os01g57560 12001.m11907 protein serine/threonine-protein kinase receptor precursor, putative, expressed  no original description 	T
29.4	protein.postranslational modification	MDP0000269951	 highly similar to ( 631) AT4G03415 | Symbols:  | catalytic/ protein serine/threonine phosphatase | chr4:1503789-1505510 REVERSEmoderately similar to ( 442) loc_os07g37890 12007.m08038 protein catalytic/ protein phosphatase type 2C, putative, expressed  no original description 	T
29.2.1.2.1.12	protein.synthesis.ribosomal protein.eukaryotic.40S subunit.S12	MDP0000006123	 weakly similar to ( 197) AT2G32060 | Symbols:  | 40S ribosomal protein S12 (RPS12C) | chr2:13639228-13640104 REVERSEmoderately similar to ( 212) RS12_HORVU 40S ribosomal protein S12 - Hordeum vulgare (Barley)moderately similar to ( 220) loc_os07g05580 12007.m05022 protein 40S ribosomal protein S12, putative, expressed  no original description 	T
29.5.11.20	protein.degradation.ubiquitin.proteasom	MDP0000949110	 highly similar to ( 732) AT5G43010 | Symbols: RPT4A | RPT4A; ATPase | chr5:17248563-17251014 REVERSEmoderately similar to ( 325) PRS7_PRUPE 26S protease regulatory subunit 7 (26S proteasome subunit 7) (26S proteasome AAA-ATPase subunit RPT1) (Regulatory particle triple-A ATPase subunit 1) - Prunus persica (Peach)highly similar to ( 721) loc_os02g10640 12002.m06362 protein 26S protease regulatory subunit S10B, putative, expressed  no original description 	T
29.5.9	protein.degradation.AAA type	MDP0000949110	 highly similar to ( 732) AT5G43010 | Symbols: RPT4A | RPT4A; ATPase | chr5:17248563-17251014 REVERSEmoderately similar to ( 325) PRS7_PRUPE 26S protease regulatory subunit 7 (26S proteasome subunit 7) (26S proteasome AAA-ATPase subunit RPT1) (Regulatory particle triple-A ATPase subunit 1) - Prunus persica (Peach)highly similar to ( 721) loc_os02g10640 12002.m06362 protein 26S protease regulatory subunit S10B, putative, expressed  no original description 	T
29.5.11.4.5.2	protein.degradation.ubiquitin.E3.BTB/POZ Cullin3.BTB/POZ	MDP0000120536	 highly similar to ( 550) AT3G06190 | Symbols: ATBPM2, BPM2 | BPM2 (BTB-POZ AND MATH DOMAIN 2); protein binding | chr3:1874577-1876575 REVERSEhighly similar to ( 552) loc_os07g46160 12007.m08834 protein speckle-type POZ protein, putative, expressed   no original description 	T
29.3.4.3	protein.targeting.secretory pathway.vacuole	MDP0000084203	 highly similar to ( 712) AT4G32940 | Symbols: GAMMA-VPE, GAMMAVPE | GAMMA-VPE (GAMMA VACUOLAR PROCESSING ENZYME); cysteine-type endopeptidase | chr4:15900557-15903161 REVERSEhighly similar to ( 766) VPE_CITSI Vacuolar-processing enzyme precursor (EC 3.4.22.-) (VPE) - Citrus sinensis (Sweet orange)highly similar to ( 656) loc_os01g37910 12001.m42664 protein vacuolar processing enzyme precursor, putative, expressed   no original description 	T
28.1	DNA.synthesis/chromatin structure	MDP0000208491	 highly similar to ( 840) AT5G08020 | Symbols: ATRPA70B, RPA70B | RPA70B (RPA70-KDA SUBUNIT B); DNA binding / nucleic acid binding | chr5:2572107-2574879 FORWARDhighly similar to ( 795) loc_os03g11540 12003.m06606 protein replication protein A 70 kDa DNA-binding subunit, putative, expressed no original description 	T
3.6	minor CHO metabolism.callose	MDP0000198973	 highly similar to ( 977) AT2G36850 | Symbols: ATGSL08, GSL8, GSL08, ATGSL8 | GSL8 (GLUCAN SYNTHASE-LIKE 8); 1,3-beta-glucan synthase/ transferase, transferring glycosyl groups | chr2:15454935-15469666 REVERSEhighly similar to ( 879) loc_os03g02756 12003.m101162 protein 1,3-beta-glucan synthase component bgs3, putative, expressed   no original description 	T
35.2	not assigned.unknown	MDP0000315565	 moderately similar to ( 459) AT4G39820 | Symbols:  | binding | chr4:18476698-18477924 REVERSEmoderately similar to ( 248) loc_os02g34540 12002.m08543 protein tetratricopeptide repeat protein 15, putative, expressed   no original description 	T
29.5.11.4.2	protein.degradation.ubiquitin.E3.RING	MDP0000281474	 highly similar to ( 829) AT3G46510 | Symbols: PUB13, ATPUB13 | PUB13 (PLANT U-BOX 13); ubiquitin-protein ligase | chr3:17124106-17126539 REVERSEhighly similar to ( 704) SPL11_ORYSA Spotted leaf protein 11 (Spotted leaf11) (Cell death-related protein SPL11) - Oryza sativa (Rice)highly similar to ( 721) loc_os03g16824 12003.m07115 protein spotted leaf protein 11, putative, expressed no original description 	T
29.1.7	protein.aa activation.alanine-tRNA ligase	MDP0000245659	 moderately similar to ( 300) AT3G16565 | Symbols:  | ATP binding / alanine-tRNA ligase/ ligase, forming aminoacyl-tRNA and related compounds / nucleic acid binding / nucleotide binding | chr3:5640531-5642675 REVERSEweakly similar to ( 134) RS21_ORYSA 40S ribosomal protein S21 - Oryza sativa (Rice)moderately similar to ( 275) loc_os01g03030 12001.m06942 protein alanyl-tRNA synthetase, putative, expressed  no original description 	T
33.99	development.unspecified	MDP0000281110	 nearly identical (1628) AT1G22770 | Symbols: GI, FB | GI (GIGANTEA) | chr1:8062398-8067447 FORWARDnearly identical (1515) GIGAN_ORYSA Protein GIGANTEA - Oryza sativa (Rice)nearly identical (1515) loc_os01g08700 12001.m42893 protein GIGANTEA protein, putative, expressed   no original description 	T
35.2	not assigned.unknown	MDP0000950316	 nearly identical (1768) AT2G02560 | Symbols: CAND1, ATCAND1, ETA2, TIP120, HVE | CAND1 (CULLIN-ASSOCIATED AND NEDDYLATION DISSOCIATED); binding | chr2:690345-697342 FORWARDnearly identical (1753) loc_os02g07120 12002.m06059 protein cullin-associated NEDD8-dissociated protein 1, putative, expressed  no original description 	T
35.1	not assigned.no ontology	MDP0000556339	 nearly identical (1079) AT4G12650 | Symbols:  | LOCATED IN: integral to membrane, Golgi apparatus, plasma membrane, vacuole; EXPRESSED IN: 25 plant structures; EXPRESSED DURING: 13 growth stages; CONTAINS InterPro DOMAIN/s: Nonaspanin (TM9SF) (InterPro:IPR004240); BEST Arabidopsis thaliana protein match is: unknown protein (TAIR:AT5G35160.1); Has 983 Blast hits to 980 proteins in 161 species: Archae - 0; Bacteria - 0; Metazoa - 434; Fungi - 144; Plants - 228; Viruses - 0; Other Eukaryotes - 177 (source: NCBI BLink). | chr4:7468207-7470165 REVERSEnearly identical (1051) loc_os08g38820 12008.m080200 protein transmembrane 9 superfamily protein member 2 precursor, putative, expressed  no original description 	T
35.2	not assigned.unknown	MDP0000142076	 moderately similar to ( 460) AT4G33945 | Symbols:  | armadillo/beta-catenin repeat family protein | chr4:16268119-16270514 FORWARDmoderately similar to ( 419) loc_os02g52370 12002.m10268 protein expressed protein  no original description 	T
35.2	not assigned.unknown	MDP0000139843	 weakly similar to ( 115) AT5G19875 | Symbols:  | unknown protein | chr5:6718412-6718783 FORWARDweakly similar to ( 101) loc_os09g27330 12009.m05885 protein expressed protein no original description 	T
20.2.99	stress.abiotic.unspecified	MDP0000848358	 weakly similar to ( 172) AT1G68300 | Symbols:  | universal stress protein (USP) family protein | chr1:25598518-25599261 REVERSEweakly similar to ( 156) loc_os01g32780 12001.m09597 protein ethylene-responsive protein, putative, expressed   no original description 	T
8.2.9	TCA / org transformation.other organic acid transformatons.cyt MDH	MDP0000142356	 weakly similar to ( 101) AT4G17620 | Symbols:  | glycine-rich protein | chr4:9822005-9824859 FORWARDvery weakly similar to (66.2) MDHC_MEDSA Malate dehydrogenase, cytoplasmic (EC 1.1.1.37) - Medicago sativa (Alfalfa)weakly similar to ( 101) loc_os05g32580 12005.m07491 protein expressed protein PRK08026  no original description 	T
20.1.7	stress.biotic.PR-proteins	MDP0000706371	 moderately similar to ( 229) AT5G66900 | Symbols:  | disease resistance protein (CC-NBS-LRR class), putative | chr5:26714931-26717757 REVERSEweakly similar to ( 124) loc_os12g39620 12012.m073872 protein disease resistance protein, putative, expressed   no original description 	T
20.1.7	stress.biotic.PR-proteins	MDP0000830772	 weakly similar to ( 177) AT1G64160 | Symbols:  | disease resistance-responsive family protein / dirigent family protein | chr1:23814063-23814611 FORWARDmoderately similar to ( 245) DR206_PEA Disease resistance response protein 206 - Pisum sativum (Garden pea)weakly similar to ( 151) loc_os07g44930 12007.m08714 protein disease resistance response protein 206, putative, expressed  no original description 	T
27.3.6	RNA.regulation of transcription.bHLH,Basic Helix-Loop-Helix family	MDP0000153000	 very weakly similar to (85.1) AT1G29950 | Symbols:  | transcription factor/ transcription regulator | chr1:10493461-10494216 FORWARDvery weakly similar to (77.4) loc_os02g21090 12002.m33374 protein expressed protein  no original description 	T
29.5.11.4.3.2	protein.degradation.ubiquitin.E3.SCF.FBOX	MDP0000191110	 moderately similar to ( 424) AT1G67480 | Symbols:  | kelch repeat-containing F-box family protein | chr1:25277294-25278529 FORWARDmoderately similar to ( 432) loc_os02g30210 12002.m100041 protein SKIP4, putative, expressed   no original description 	T
26.2	misc.UDP glucosyl and glucoronyl transferases	MDP0000315108	 highly similar to ( 585) AT1G57720 | Symbols:  | elongation factor 1B-gamma, putative / eEF-1B gamma, putative | chr1:21377873-21380114 FORWARDhighly similar to ( 620) EF1G2_ORYSA Elongation factor 1-gamma 2 (EF-1-gamma 2) (eEF-1B gamma 2) - Oryza sativa (Rice)highly similar to ( 619) loc_os02g12794 12002.m100462 protein elongation factor 1-gamma 2, putative, expressed  no original description 	T
26.17	misc.dynamin	MDP0000176025	 highly similar to ( 741) AT1G60500 | Symbols:  | dynamin family protein | chr1:22291582-22293822 FORWARDhighly similar to ( 683) loc_os03g15420 12003.m06979 protein GTP binding protein, putative, expressed  no original description 	T
35.2	not assigned.unknown	MDP0000287619	 moderately similar to ( 264) AT1G13530 | Symbols:  | unknown protein | chr1:4633740-4635129 REVERSEmoderately similar to ( 235) loc_os08g31890 12008.m07183 protein conserved hypothetical protein    no original description 	T
35.2	not assigned.unknown	MDP0000234099	 weakly similar to ( 171) AT1G26750 | Symbols:  | unknown protein | chr1:9246857-9248053 FORWARDmoderately similar to ( 228) loc_os12g10650 12012.m05048 protein expressed protein  no original description 	T
27.3.62	RNA.regulation of transcription.Nucleosome/chromatin assembly factor group	MDP0000205665	 weakly similar to ( 172) AT5G23420 | Symbols: HMGB6 | HMGB6; transcription factor | chr5:7888712-7890111 REVERSEvery weakly similar to (96.7) HMGL_VICFA HMG1/2-like protein - Vicia faba (Broad bean)weakly similar to ( 155) loc_os08g01100 12008.m04259 protein HMG1/2-like protein, putative, expressed  no original description 	T
34.2	transport.sugars	MDP0000365390	 moderately similar to ( 354) AT5G16150 | Symbols: GLT1, PGLCT | PGLCT (PLASTIDIC GLC TRANSLOCATOR); carbohydrate transmembrane transporter/ sugar:hydrogen symporter | chr5:5272904-5275678 FORWARDweakly similar to ( 132) STC_RICCO Sugar carrier protein C - Ricinus communis (Castor bean)moderately similar to ( 342) loc_os01g04190 12001.m150473 protein arabinose-proton symporter, putative, expressed no original description 	T
16.5.1.3.3	secondary metabolism.sulfur-containing.glucosinolates.degradation.nitrilase	MDP0000239810	 moderately similar to ( 390) AT5G22300 | Symbols: NIT4 | NIT4 (NITRILASE 4); 3-cyanoalanine hydratase/ cyanoalanine nitrilase/ indole-3-acetonitrile nitrilase/ nitrilase/ nitrile hydratase | chr5:7379401-7381764 FORWARDmoderately similar to ( 421) NRL4_TOBAC Nitrilase 4 (EC 3.5.5.1) - Nicotiana tabacum (Common tobacco)moderately similar to ( 410) loc_os02g42350 12002.m100293 protein nitrilase 4, putative, expressed no original description 	T
35.2	not assigned.unknown	MDP0000403302	 weakly similar to ( 116) AT5G17350 | Symbols:  | unknown protein | chr5:5718853-5719404 FORWARDweakly similar to ( 102) loc_os05g47960 12005.m08868 protein expressed protein   no original description 	T
35.1.14	not assigned.no ontology.S RNA-binding domain-containing protein	MDP0000154322	 moderately similar to ( 431) AT1G53120 | Symbols:  | RNA-binding S4 domain-containing protein | chr1:19792798-19794605 FORWARDmoderately similar to ( 376) loc_os01g54390 12001.m43191 protein RNA-binding S4, putative, expressed  no original description 	T
26.17	misc.dynamin	MDP0000069231	 highly similar to ( 995) AT3G60190 | Symbols: ADL4, ADLP2, EDR3, DRP1E, ADL1E | ADL1E (ARABIDOPSIS DYNAMIN-LIKE 1E); GTP binding / GTPase | chr3:22244367-22247651 REVERSEhighly similar to ( 983) loc_os10g41820 12010.m21978 protein dynamin-related protein 1C, putative, expressed  no original description 	T
35.2	not assigned.unknown	MDP0000122371	 highly similar to ( 571) AT1G53040 | Symbols:  | unknown protein | chr1:19764567-19766870 REVERSEhighly similar to ( 533) loc_os05g45490 12005.m08674 protein EMB2756, putative, expressed MSP1_C  no original description 	T
29.2.2.50	protein.synthesis.ribosome biogenesis.BRIX	MDP0000711630	 moderately similar to ( 232) AT1G52930 | Symbols:  | brix domain-containing protein | chr1:19711261-19713103 FORWARDmoderately similar to ( 231) loc_os02g37870 12002.m08875 protein brix domain-containing protein 2, putative, expressed no original description 	T
34.19.3	transport.Major Intrinsic Proteins.NIP	MDP0000281609	 moderately similar to ( 316) AT4G18910 | Symbols: NIP1;2, NLM2 | NIP1;2 (NOD26-LIKE INTRINSIC PROTEIN 1;2); arsenite transmembrane transporter/ water channel | chr4:10366211-10368179 FORWARDmoderately similar to ( 305) NO26_SOYBN Nodulin-26 (N-26) - Glycine max (Soybean)moderately similar to ( 326) loc_os02g13870 12002.m06633 protein aquaporin NIP1.2, putative, expressed  no original description 	T
17.3.1.2.2	hormone metabolism.brassinosteroid.synthesis-degradation.sterols.SMT2	MDP0000253134	 highly similar to ( 665) AT1G20330 | Symbols: SMT2, CVP1, FRL1 | SMT2 (STEROL METHYLTRANSFERASE 2); S-adenosylmethionine-dependent methyltransferase | chr1:7038968-7040053 REVERSEhighly similar to ( 528) SMT2_ORYSA 24-methylenesterol C-methyltransferase 2 (EC 2.1.1.143) (24-sterol C-methyltransferase 2) (Sterol-C-methyltransferase 2) - Oryza sativa (Rice)highly similar to ( 528) loc_os03g04340 12003.m05964 protein 24-methylenesterol C-methyltransferase 2, putative, expressed  no original description 	T
26.2	misc.UDP glucosyl and glucoronyl transferases	MDP0000370712	 highly similar to ( 575) AT3G62660 | Symbols: GATL7 | GATL7 (Galacturonosyltransferase-like 7); polygalacturonate 4-alpha-galacturonosyltransferase/ transferase, transferring glycosyl groups / transferase, transferring hexosyl groups | chr3:23173419-23174504 FORWARDhighly similar to ( 534) loc_os03g18890 12003.m07309 protein transferase, transferring glycosyl groups, putative, expressed   no original description 	T
13.1.3.1	amino acid metabolism.synthesis.aspartate family.asparagine	MDP0000119630	 highly similar to ( 967) AT3G47340 | Symbols: ASN1, DIN6, AT-ASN1 | ASN1 (GLUTAMINE-DEPENDENT ASPARAGINE SYNTHASE 1); asparagine synthase (glutamine-hydrolyzing) | chr3:17438445-17441043 REVERSEhighly similar to ( 978) ASNS1_LOTJA Asparagine synthetase [glutamine-hydrolyzing] 1 (EC 6.3.5.4) (Glutamine-dependent asparagine synthetase 1) - Lotus japonicushighly similar to ( 906) loc_os06g15420 12006.m06255 protein asparagine synthetase, putative, expressed COG3889  no original description 	T
10.8.1	cell wall.pectin*esterases.PME	MDP0000474161	 moderately similar to ( 491) AT5G47500 | Symbols:  | pectinesterase family protein | chr5:19271262-19272845 REVERSEweakly similar to ( 124) PME_DAUCA Pectinesterase (EC 3.1.1.11) (Pectin methylesterase) (PE) - Daucus carota (Carrot)moderately similar to ( 460) loc_os04g46740 12004.m09622 protein pectinesterase-4 precursor, putative, expressed   no original description 	T
29.2.1.1.1.1.7	protein.synthesis.ribosomal protein.prokaryotic.chloroplast.30S subunit.S7	MDP0000132523	 moderately similar to ( 483) AT3G11945 | Symbols: ATHST | homogentisate farnesyltransferase/ homogentisate geranylgeranyltransferase/ homogentisate solanesyltransferase | chr3:3780041-3782880 REVERSEmoderately similar to ( 358) RS5_CICAR 40S ribosomal protein S5 (Fragment) - Cicer arietinum (Chickpea) (Garbanzo)moderately similar to ( 461) loc_os07g38850 12007.m08130 protein prenyltransferase/ zinc ion binding protein, putative, expressed  no original description 	T
29.2.1.2.1.5	protein.synthesis.ribosomal protein.eukaryotic.40S subunit.S5	MDP0000132523	 moderately similar to ( 483) AT3G11945 | Symbols: ATHST | homogentisate farnesyltransferase/ homogentisate geranylgeranyltransferase/ homogentisate solanesyltransferase | chr3:3780041-3782880 REVERSEmoderately similar to ( 358) RS5_CICAR 40S ribosomal protein S5 (Fragment) - Cicer arietinum (Chickpea) (Garbanzo)moderately similar to ( 461) loc_os07g38850 12007.m08130 protein prenyltransferase/ zinc ion binding protein, putative, expressed  no original description 	T
34.21	transport.calcium	MDP0000334484	 moderately similar to ( 252) AT4G03560 | Symbols: ATTPC1, TPC1, ATCCH1, FOU2 | ATTPC1 (TWO-PORE CHANNEL 1); calcium channel/ voltage-gated calcium channel | chr4:1580253-1585691 FORWARDmoderately similar to ( 226) TPC1_ORYSA Probable voltage-dependent calcium channel protein TPC1 (Probable voltage-gated calcium channel) (Two-pore calcium channel protein) - Oryza sativa (Rice)moderately similar to ( 226) loc_os01g48680 12001.m42691 protein voltage-dependent calcium channel protein TPC1, putative, expressed  no original description 	T
27.3.67	RNA.regulation of transcription.putative transcription regulator	MDP0000319119	 moderately similar to ( 263) AT4G12080 | Symbols:  | DNA-binding family protein | chr4:7239466-7241246 FORWARDmoderately similar to ( 206) loc_os08g02490 12008.m04393 protein DNA-binding protein, putative, expressed  no original description 	T
35.2	not assigned.unknown	MDP0000361520	  no original description 	T
35.2	not assigned.unknown	MDP0000948665	  no original description 	T
27.3.21	RNA.regulation of transcription.GRAS transcription factor family	MDP0000254936	 highly similar to ( 585) AT4G17230 | Symbols: SCL13 | SCL13 (Scarecrow-like 13); transcription factor | chr4:9661218-9662807 REVERSEhighly similar to ( 508) CIGR2_ORYSA Chitin-inducible gibberellin-responsive protein 2 - Oryza sativa (Rice)highly similar to ( 510) loc_os10g22430 12010.m05252 protein chitin-inducible gibberellin-responsive protein 2, putative, expressed  no original description 	T
26.28	misc.GDSL-motif lipase	MDP0000312022	 moderately similar to ( 473) AT3G48460 | Symbols:  | GDSL-motif lipase/hydrolase family protein | chr3:17949496-17951082 FORWARDweakly similar to ( 156) EST_HEVBR Esterase precursor (EC 3.1.1.-) (Early nodule-specific protein homolog) (Latex allergen Hev b 13) - Hevea brasiliensis (Para rubber tree)moderately similar to ( 422) loc_os03g62740 12003.m11151 protein esterase precursor, putative, expressed  no original description 	T
35.1	not assigned.no ontology	MDP0000211549	 moderately similar to ( 351) AT3G47590 | Symbols:  | esterase/lipase/thioesterase family protein | chr3:17536133-17537515 REVERSEmoderately similar to ( 334) loc_os01g39790 12001.m43070 protein esterase, putative, expressed   no original description 	T
35.2	not assigned.unknown	MDP0000602145	 very weakly similar to (58.9) AT1G74055 | Symbols:  | unknown protein | chr1:27849279-27849713 FORWARD  no original description 	T
27.3.62	RNA.regulation of transcription.Nucleosome/chromatin assembly factor group	MDP0000757111	 weakly similar to ( 135) AT3G51880 | Symbols: HMGB1, NFD1 | HMGB1 (HIGH MOBILITY GROUP B 1); DNA binding / chromatin binding / structural constituent of chromatin / transcription factor | chr3:19247241-19248491 REVERSEweakly similar to ( 102) HMGL_SOYBN HMG1/2-like protein (SB11 protein) - Glycine max (Soybean)very weakly similar to (99.0) loc_os09g37910 12009.m22188 protein HMG1/2-like protein, putative, expressed no original description 	T
26.9	misc.glutathione S transferases	MDP0000755113	 moderately similar to ( 337) AT1G78380 | Symbols: ATGSTU19, GST8 | ATGSTU19 (GLUTATHIONE S-TRANSFERASE TAU 19); glutathione binding / glutathione transferase | chr1:29486659-29487819 REVERSEmoderately similar to ( 360) GSTX4_TOBAC Probable glutathione S-transferase (EC 2.5.1.18) (Auxin-induced protein PCNT107) - Nicotiana tabacum (Common tobacco)moderately similar to ( 260) loc_os03g57200 12003.m10639 protein glutathione S-transferase parA, putative, expressed no original description 	T
27.3.26	RNA.regulation of transcription.MYB-related transcription factor family	MDP0000537549	 weakly similar to ( 175) AT5G56840 | Symbols:  | DNA-binding family protein | chr5:22980789-22982152 FORWARDweakly similar to ( 187) loc_os01g09640 12001.m07588 protein MCB2 protein, putative, expressed  no original description 	T
26.1	misc.misc2	MDP0000361644	 moderately similar to ( 298) AT3G23600 | Symbols:  | dienelactone hydrolase family protein | chr3:8473833-8475655 FORWARDweakly similar to ( 145) E134_MAIZE Endo-1,3;1,4-beta-D-glucanase precursor (EC 3.2.1.-) - Zea mays (Maize)moderately similar to ( 285) loc_os05g33100 12005.m07542 protein endo-1,3;1,4-beta-D-glucanase precursor, putative, expressed   no original description 	T
33.99	development.unspecified	MDP0000314081	 weakly similar to ( 180) AT4G35770 | Symbols: SEN1, ATSEN1, DIN1 | SEN1 (SENESCENCE 1) | chr4:16945073-16945964 FORWARDweakly similar to ( 178) DIN1_RAPSA Senescence-associated protein DIN1 - Raphanus sativus (Radish)weakly similar to ( 165) loc_os06g50930 12006.m09617 protein senescence-associated protein DIN1, putative, expressed no original description 	T
35.2	not assigned.unknown	MDP0000179031	 weakly similar to ( 190) AT1G09310 | Symbols:  | unknown protein | chr1:3009109-3009648 FORWARDweakly similar to ( 166) loc_os12g37650 12012.m07550 protein expressed protein   no original description 	T
35.1	not assigned.no ontology	MDP0000259191	 highly similar to ( 518) AT5G22130 | Symbols: PNT1 | PNT1 (PEANUT 1); alpha-1,4-mannosyltransferase/ mannosyltransferase/ transferase, transferring glycosyl groups | chr5:7337486-7339831 REVERSEhighly similar to ( 518) PIGM_ARATH GPI mannosyltransferase 1 (EC 2.4.1.-) (GPI mannosyltransferase I) (GPI-MT-I) (Phosphatidylinositol-glycan biosynthesis class M protein) (PIG-M) (Protein PEANUT1) - Arabidopsis thaliana (Mouse-ear cress)highly similar to ( 502) loc_os03g46750 12003.m09691 protein PNT1, putative, expressed  no original description 	T
35.2	not assigned.unknown	MDP0000256442	 highly similar to ( 570) AT5G53620 | Symbols:  | unknown protein | chr5:21781217-21785061 FORWARDhighly similar to ( 544) loc_os08g43060 12008.m080226 protein expressed protein   no original description 	T
1.1.1.2	PS.lightreaction.photosystem II.PSII polypeptide subunits	MDP0000906067	 weakly similar to ( 183) AT1G03600 | Symbols:  | photosystem II family protein | chr1:898916-899440 FORWARDweakly similar to ( 183) loc_os03g21560 12003.m07560 protein photosystem II 11 kD protein, putative, expressed  no original description 	T
29.2.1.1.1.2.17	protein.synthesis.ribosomal protein.prokaryotic.chloroplast.50S subunit.L17	MDP0000158530	 moderately similar to ( 252) AT3G54210 | Symbols:  | ribosomal protein L17 family protein | chr3:20067672-20068385 REVERSEmoderately similar to ( 258) RK17_TOBAC 50S ribosomal protein L17, chloroplast precursor (CL17) - Nicotiana tabacum (Common tobacco)moderately similar to ( 230) loc_os03g60100 12003.m10904 protein 50S ribosomal protein L17, putative, expressed  no original description 	T
35.2	not assigned.unknown	MDP0000689889	 weakly similar to ( 145) AT3G55240 | Symbols:  | Overexpression leads to PEL (Pseudo-Etiolation in Light) phenotype. | chr3:20473876-20474705 REVERSEweakly similar to ( 116) loc_os05g38680 12005.m08047 protein plant-specific domain TIGR01589 family protein, expressed no original description 	T
29.2.1.2.2.510	protein.synthesis.ribosomal protein.eukaryotic.60S subunit.L10A	MDP0000129677	 moderately similar to ( 327) AT2G27530 | Symbols: PGY1 | PGY1 (PIGGYBACK1); RNA binding / structural constituent of ribosome | chr2:11763443-11764570 REVERSEmoderately similar to ( 247) RL10A_CHLRE 60S ribosomal protein L10a - Chlamydomonas reinhardtiimoderately similar to ( 340) loc_os01g64090 12001.m43287 protein 60S ribosomal protein L10a-1, putative, expressed  no original description 	T
29.2.1.2.2.8	protein.synthesis.ribosomal protein.eukaryotic.60S subunit.L8	MDP0000486544	 weakly similar to ( 168) AT4G36130 | Symbols:  | 60S ribosomal protein L8 (RPL8C) | chr4:17097613-17098656 FORWARDweakly similar to ( 168) RL8_TOBAC 60S ribosomal protein L8 (60S ribosomal protein L2) - Nicotiana tabacum (Common tobacco)weakly similar to ( 172) loc_os12g38000 12012.m07586 protein 60S ribosomal protein L2, putative, expressed no original description 	T
26.1	misc.misc2	MDP0000255705	 highly similar to ( 730) AT4G37560 | Symbols:  | formamidase, putative / formamide amidohydrolase, putative | chr4:17646489-17648457 FORWARDhighly similar to ( 724) loc_os01g55950 12001.m11751 protein acetamidase, putative, expressed  no original description 	T
35.2	not assigned.unknown	MDP0000780740	  no original description 	T
26.18	misc.invertase/pectin methylesterase inhibitor family protein	MDP0000254203	 weakly similar to ( 161) AT3G62820 | Symbols:  | invertase/pectin methylesterase inhibitor family protein | chr3:23229782-23230366 FORWARDvery weakly similar to (94.7) 21KD_DAUCA 21 kDa protein precursor (1.2 protein) - Daucus carota (Carrot)very weakly similar to (82.4) loc_os06g49760 12006.m09500 protein ripening-related protein, putative, expressed no original description 	T
35.2	not assigned.unknown	MDP0000181887	 weakly similar to ( 132) AT2G40435 | Symbols:  | EXPRESSED IN: 20 plant structures; EXPRESSED DURING: 13 growth stages; BEST Arabidopsis thaliana protein match is: transcription regulator (TAIR:AT3G56220.1); Has 165 Blast hits to 165 proteins in 20 species: Archae - 0; Bacteria - 0; Metazoa - 0; Fungi - 0; Plants - 165; Viruses - 0; Other Eukaryotes - 0 (source: NCBI BLink). | chr2:16887048-16888407 FORWARDweakly similar to ( 125) loc_os07g47960 12007.m09011 protein expressed protein  no original description 	T
35.2	not assigned.unknown	MDP0000330667	  no original description 	T
30.2.11	signalling.receptor kinases.leucine rich repeat XI	MDP0000593607	 weakly similar to ( 119) AT5G20480 | Symbols: EFR | EFR (EF-TU RECEPTOR); ATP binding / kinase/ protein serine/threonine kinase | chr5:6922497-6925679 FORWARDweakly similar to ( 106) RPK1_IPONI Receptor-like protein kinase precursor (EC 2.7.11.1) - Ipomoea nil (Japanese morning glory) (Pharbitis nil)weakly similar to ( 129) loc_os02g30540 12002.m08197 protein receptor-like protein kinase 5 precursor, putative  no original description 	T
35.2	not assigned.unknown	MDP0000498460	 moderately similar to ( 355) AT4G35730 | Symbols:  | unknown protein | chr4:16931111-16933241 FORWARDmoderately similar to ( 297) loc_os09g37510 12009.m06698 protein expressed protein  no original description 	T
35.2	not assigned.unknown	MDP0000241913	 moderately similar to ( 207) AT3G06035 | Symbols:  | unknown protein | chr3:1823172-1824110 REVERSEweakly similar to ( 184) loc_os07g45060 12007.m08726 protein GPI-anchored protein At5g19240 precursor, putative, expressed no original description 	T
28.1	DNA.synthesis/chromatin structure	MDP0000247378	 nearly identical (1008) AT5G46280 | Symbols: MCM3 | DNA replication licensing factor, putative | chr5:18769902-18773606 REVERSEhighly similar to ( 823) MCM3_MAIZE DNA replication licensing factor MCM3 homolog (Replication origin activator) (ROA protein) (Fragment) - Zea mays (Maize)nearly identical (1007) loc_os05g39850 12005.m08162 protein DNA replication licensing factor MCM3, putative, expressed   no original description 	T
34.12	transport.metal	MDP0000290114	 moderately similar to ( 285) AT2G01770 | Symbols: VIT1 | VIT1 (vacuolar iron transporter 1); iron ion transmembrane transporter | chr2:334775-336425 REVERSEmoderately similar to ( 280) loc_os04g38940 12004.m35331 protein protein CCC1, putative, expressed  no original description 	T
15.3	metal handling.regulation	MDP0000290114	 moderately similar to ( 285) AT2G01770 | Symbols: VIT1 | VIT1 (vacuolar iron transporter 1); iron ion transmembrane transporter | chr2:334775-336425 REVERSEmoderately similar to ( 280) loc_os04g38940 12004.m35331 protein protein CCC1, putative, expressed  no original description 	T
34.8	transport.metabolite transporters at the envelope membrane	MDP0000176939	 moderately similar to ( 499) AT5G04160 | Symbols:  | phosphate translocator-related | chr5:1143033-1144777 REVERSEvery weakly similar to (67.8) TPT_SPIOL Triose phosphate/phosphate translocator, chloroplast precursor (cTPT) (p36) (E29) - Spinacia oleracea (Spinach)moderately similar to ( 412) loc_os03g17740 12003.m07199 protein organic anion transporter, putative, expressed no original description 	T
17.3.1.2.8	hormone metabolism.brassinosteroid.synthesis-degradation.sterols.DWF1	MDP0000278275	 highly similar to ( 935) AT3G19820 | Symbols: DWF1, DIM, EVE1, DIM1, CBB1 | DWF1 (DWARF 1); calmodulin binding / catalytic | chr3:6879835-6881616 REVERSEnearly identical (1002) DIM_PEA Cell elongation protein diminuto - Pisum sativum (Garden pea)highly similar to ( 974) loc_os10g25780 12010.m21783 protein cell elongation protein diminuto, putative, expressed   no original description 	T
30.5	signalling.G-proteins	MDP0000414898	 moderately similar to ( 499) AT5G25460 | Symbols:  | unknown protein | chr5:8863430-8865394 FORWARDmoderately similar to ( 284) RAN_VICFA GTP-binding nuclear protein Ran/TC4 - Vicia faba (Broad bean)moderately similar to ( 441) loc_os01g42520 12001.m10516 protein expressed protein  no original description 	T
35.2	not assigned.unknown	MDP0000119431	 moderately similar to ( 221) AT5G24460 | Symbols:  | unknown protein | chr5:8354815-8355717 FORWARDweakly similar to ( 134) loc_os06g49780 12006.m09502 protein expressed protein  no original description 	T
10.6.3	cell wall.degradation.pectate lyases and polygalacturonases	MDP0000209147	 highly similar to ( 651) AT1G60590 | Symbols:  | polygalacturonase, putative / pectinase, putative | chr1:22314426-22316867 REVERSEmoderately similar to ( 228) PGLR_ACTCH Polygalacturonase precursor (EC 3.2.1.15) (PG) (Pectinase) - Actinidia chinensis (Kiwi) (Yangtao)highly similar to ( 540) loc_os01g44970 12001.m10703 protein polygalacturonase precursor, putative, expressed   no original description 	T
11.1.6	lipid metabolism.FA synthesis and FA elongation.enoyl ACP reductase	MDP0000327231	 highly similar to ( 541) AT2G05990 | Symbols: MOD1, ENR1 | MOD1 (MOSAIC DEATH 1); enoyl-[acyl-carrier-protein] reductase (NADH)/ enoyl-[acyl-carrier-protein] reductase/ oxidoreductase | chr2:2322876-2324867 FORWARDhighly similar to ( 528) FABI_BRANA Enoyl-[acyl-carrier-protein] reductase [NADH], chloroplast precursor (EC 1.3.1.9) (NADH-dependent enoyl-ACP reductase) - Brassica napus (Rape)moderately similar to ( 495) loc_os08g23810 12008.m26662 protein enoyl-[acyl-carrier-protein] reductase [NADH], chloroplast precursor, expressed  no original description 	T
20.1	stress.biotic	MDP0000237300	 moderately similar to ( 275) AT5G02140 | Symbols:  | thaumatin-like protein, putative | chr5:423384-424437 FORWARDweakly similar to ( 143) TP1A_MALDO Thaumatin-like protein 1a precursor (Allergen Mal d 2) (Mdtl1) (Pathogenesis-related protein 5a) (PR-5a) - Malus domestica (Apple) (Malus sylvestris)moderately similar to ( 229) loc_os11g47944 12011.m08592 protein thaumatin-like protein precursor, putative  no original description 	T
29.2.1.2.2.26	protein.synthesis.ribosomal protein.eukaryotic.60S subunit.L26	MDP0000757192	 moderately similar to ( 211) AT3G49910 | Symbols:  | 60S ribosomal protein L26 (RPL26A) | chr3:18504311-18504751 FORWARDweakly similar to ( 191) RL26_BRARA 60S ribosomal protein L26 - Brassica rapa (Turnip)weakly similar to ( 190) loc_os12g05430 12012.m04536 protein 60S ribosomal protein L26-1, putative, expressed  no original description 	T
29.6	protein.folding	MDP0000145314	 weakly similar to ( 101) AT4G39710 | Symbols:  | immunophilin, putative / FKBP-type peptidyl-prolyl cis-trans isomerase, putative | chr4:18427249-18428325 REVERSEvery weakly similar to (83.2) loc_os02g51570 12002.m10190 protein FKBP-type peptidyl-prolyl cis-trans isomerase 4, chloroplast precursor, putative, expressed  no original description 	T
35.2	not assigned.unknown	MDP0000290674	 moderately similar to ( 352) AT5G67370 | Symbols:  | unknown protein | chr5:26877667-26879465 REVERSEmoderately similar to ( 308) loc_os03g32490 12003.m08490 protein expressed protein  no original description 	T
31.1	cell.organisation	MDP0000437689	 weakly similar to ( 177) AT2G26770 | Symbols:  | plectin-related | chr2:11404257-11407743 REVERSEweakly similar to ( 135) loc_os01g59130 12001.m43504 protein expressed protein  no original description 	T
27.3.25	RNA.regulation of transcription.MYB domain transcription factor family	MDP0000193219	 moderately similar to ( 263) AT1G26580 | Symbols:  | FUNCTIONS IN: molecular_function unknown; INVOLVED IN: biological_process unknown; LOCATED IN: cellular_component unknown; EXPRESSED IN: 24 plant structures; EXPRESSED DURING: 15 growth stages; BEST Arabidopsis thaliana protein match is: myb family transcription factor / ELM2 domain-containing protein (TAIR:AT2G03470.1); Has 82 Blast hits to 82 proteins in 12 species: Archae - 0; Bacteria - 0; Metazoa - 0; Fungi - 4; Plants - 78; Viruses - 0; Other Eukaryotes - 0 (source: NCBI BLink). | chr1:9185620-9187213 FORWARDweakly similar to ( 171) loc_os03g31230 12003.m35283 protein DNA binding protein, putative, expressed  no original description 	T
26.5	misc.acyl transferases	MDP0000215941	 highly similar to ( 553) AT1G80410 | Symbols: EMB2753 | EMB2753 (EMBRYO DEFECTIVE 2753); binding | chr1:30227963-30234832 REVERSEhighly similar to ( 559) loc_os01g43030 12001.m43094 protein NMDA receptor-regulated 1-like protein, putative, expressed no original description 	T
35.2	not assigned.unknown	MDP0000135679	 moderately similar to ( 380) AT4G32140 | Symbols:  | FUNCTIONS IN: molecular_function unknown; INVOLVED IN: biological_process unknown; LOCATED IN: endomembrane system, membrane; EXPRESSED IN: 24 plant structures; EXPRESSED DURING: 13 growth stages; CONTAINS InterPro DOMAIN/s: Protein of unknown function DUF6, transmembrane (InterPro:IPR000620); BEST Arabidopsis thaliana protein match is: membrane protein (TAIR:AT3G07080.1); Has 1078 Blast hits to 1078 proteins in 273 species: Archae - 20; Bacteria - 294; Metazoa - 244; Fungi - 147; Plants - 41; Viruses - 0; Other Eukaryotes - 332 (source: NCBI BLink). | chr4:15523037-15525195 REVERSEmoderately similar to ( 369) loc_os04g55260 12004.m10418 protein vacuolar membrane protein, putative, expressed  no original description 	T
27.3.71	RNA.regulation of transcription.SNF7	MDP0000232670	 moderately similar to ( 393) AT3G10640 | Symbols: VPS60.1 | VPS60.1 | chr3:3323501-3324484 REVERSEmoderately similar to ( 369) loc_os05g01250 12005.m04666 protein charged multivesicular body protein 5, putative, expressed  no original description 	T
29.2.1.2.1.14	protein.synthesis.ribosomal protein.eukaryotic.40S subunit.S14	MDP0000783833	 weakly similar to ( 200) AT3G52580 | Symbols:  | 40S ribosomal protein S14 (RPS14C) | chr3:19503324-19504701 FORWARDmoderately similar to ( 201) RS142_MAIZE 40S ribosomal protein S14 (Clone MCH2) - Zea mays (Maize)moderately similar to ( 206) loc_os02g06700 12002.m06018 protein 40S ribosomal protein S14, putative, expressed no original description 	T
29.2.1.99.99	protein.synthesis.ribosomal protein.unknown.unknown	MDP0000801117	 weakly similar to ( 118) AT1G16790 | Symbols:  | ribosomal protein-related | chr1:5744638-5745072 FORWARD   no original description 	T
29.4.1.57	protein.postranslational modification.kinase.receptor like cytoplasmatic kinase VII	MDP0000463045	 moderately similar to ( 369) AT1G21230 | Symbols: WAK5 | WAK5 (WALL ASSOCIATED KINASE 5); kinase/ protein serine/threonine kinase | chr1:7429980-7432346 FORWARDmoderately similar to ( 215) CRI4_MAIZE Putative receptor protein kinase CRINKLY4 precursor (EC 2.7.11.1) - Zea mays (Maize)moderately similar to ( 352) loc_os02g56630 12002.m10685 protein OsWAK24 - OsWAK receptor-like protein kinase  no original description 	T
27.3.67	RNA.regulation of transcription.putative transcription regulator	MDP0000742985	 highly similar to ( 701) AT3G05060 | Symbols:  | SAR DNA-binding protein, putative | chr3:1413174-1415564 REVERSEhighly similar to ( 700) loc_os03g22740 12003.m07672 protein nucleolar protein NOP5, putative, expressed  PRK07003  no original description 	T
35.1	not assigned.no ontology	MDP0000267569	 weakly similar to ( 157) AT3G07560 | Symbols: APM2, PEX13 | PEX13 (PEROXIN 13); protein binding | chr3:2411616-2413310 REVERSEweakly similar to ( 130) loc_os07g05810 12007.m05045 protein glycine-rich protein, putative, expressed  no original description 	T
35.2	not assigned.unknown	MDP0000905593	 weakly similar to ( 177) AT1G61667 | Symbols:  | unknown protein | chr1:22767674-22768269 FORWARDweakly similar to ( 145) loc_os07g36410 12007.m07892 protein expressed protein   no original description 	T
26.22	misc.short chain dehydrogenase/reductase (SDR)	MDP0000124013	 highly similar to ( 636) AT4G13250 | Symbols:  | short-chain dehydrogenase/reductase (SDR) family protein | chr4:7684417-7686691 REVERSEvery weakly similar to (70.5) SDR1_PICAB Short-chain type dehydrogenase/reductase (EC 1.-.-.-) - Picea abies (Norway spruce) (Picea excelsa)highly similar to ( 651) loc_os01g12710 12001.m07884 protein oxidoreductase, putative, expressed   no original description 	T
26.19	misc.plastocyanin-like	MDP0000191805	 weakly similar to ( 169) AT4G31840 | Symbols:  | plastocyanin-like domain-containing protein | chr4:15401798-15402426 FORWARDweakly similar to ( 101) NO552_SOYBN Early nodulin 55-2 precursor (N-55-2) (Nodulin-315) - Glycine max (Soybean)weakly similar to ( 115) loc_os01g57880 12001.m11936 protein early nodulin-like protein 3 precursor, putative, expressed  no original description 	T
35.2	not assigned.unknown	MDP0000280698	 highly similar to ( 550) AT5G41970 | Symbols:  | FUNCTIONS IN: molecular_function unknown; INVOLVED IN: biological_process unknown; LOCATED IN: mitochondrion; EXPRESSED IN: 24 plant structures; EXPRESSED DURING: 13 growth stages; CONTAINS InterPro DOMAIN/s: Metal-dependent protein hydrolase (InterPro:IPR003226); BEST Arabidopsis thaliana protein match is: unknown protein (TAIR:AT3G49320.1); Has 464 Blast hits to 461 proteins in 209 species: Archae - 0; Bacteria - 122; Metazoa - 131; Fungi - 87; Plants - 29; Viruses - 0; Other Eukaryotes - 95 (source: NCBI BLink). | chr5:16791198-16792961 FORWARDhighly similar to ( 543) loc_os02g46150 12002.m09652 protein MYG1 protein, putative, expressed  no original description 	T
35.2	not assigned.unknown	MDP0000470984	 moderately similar to ( 238) AT3G53630 | Symbols:  | unknown protein | chr3:19883986-19885568 FORWARDweakly similar to ( 149) loc_os03g17140 12003.m07145 protein expressed protein   no original description 	T
35.1	not assigned.no ontology	MDP0000375867	 weakly similar to ( 145) AT4G02530 | Symbols:  | chloroplast thylakoid lumen protein | chr4:1112335-1114005 REVERSEweakly similar to ( 147) loc_os06g49160 12006.m09440 protein thylakoid lumenal 16.5 kDa protein, chloroplast precursor, putative, expressed no original description 	T
29.4	protein.postranslational modification	MDP0000223875	 highly similar to ( 544) AT4G32830 | Symbols: AtAUR1 | AtAUR1 (ATAURORA1); histone kinase(H3-S10 specific) / kinase/ protein serine/threonine kinase | chr4:15842557-15844354 FORWARDweakly similar to ( 169) CDPK_DAUCA Calcium-dependent protein kinase (EC 2.7.11.1) (CDPK) - Daucus carota (Carrot)highly similar to ( 513) loc_os01g09580 12001.m07583 protein serine/threonine-protein kinase Eg2-like, putative, expressed  no original description 	T
11.1.10	lipid metabolism.FA synthesis and FA elongation.beta ketoacyl CoA synthase	MDP0000282105	 highly similar to ( 664) AT2G28630 | Symbols: KCS12 | KCS12 (3-KETOACYL-COA SYNTHASE 12); acyltransferase/ catalytic/ transferase, transferring acyl groups other than amino-acyl groups | chr2:12275719-12277149 REVERSEvery weakly similar to (50.4) CHSY_PETCR Chalcone synthase (EC 2.3.1.74) (Naringenin-chalcone synthase) - Petroselinum crispum (Parsley) (Petroselinum hortense)highly similar to ( 506) loc_os10g33370 12010.m06165 protein acyltransferase, putative, expressed   no original description 	T
30.2.11	signalling.receptor kinases.leucine rich repeat XI	MDP0000127305	 moderately similar to ( 416) AT4G08850 | Symbols:  | kinase | chr4:5637467-5640496 REVERSEmoderately similar to ( 259) RPK1_IPONI Receptor-like protein kinase precursor (EC 2.7.11.1) - Ipomoea nil (Japanese morning glory) (Pharbitis nil)moderately similar to ( 456) loc_os10g02970 12010.m03714 protein receptor-like protein kinase precursor, putative   no original description 	T
1.1.2.2	PS.lightreaction.photosystem I.PSI polypeptide subunits	MDP0000909197	 weakly similar to ( 183) AT1G52230 | Symbols: PSAH2, PSAH-2, PSI-H | PSAH2 (PHOTOSYSTEM I SUBUNIT H2) | chr1:19454902-19455508 FORWARDweakly similar to ( 182) PSAH_SPIOL Photosystem I reaction center subunit VI, chloroplast precursor (PSI-H) (Light-harvesting complex I 11 kDa protein) - Spinacia oleracea (Spinach)weakly similar to ( 164) loc_os05g48630 12005.m08934 protein expressed protein no original description 	T
27.3.3	RNA.regulation of transcription.AP2/EREBP, APETALA2/Ethylene-responsive element binding protein family	MDP0000243375	 weakly similar to ( 191) AT5G25190 | Symbols:  | ethylene-responsive element-binding protein, putative | chr5:8707007-8707655 REVERSEvery weakly similar to (64.7) ERF3_TOBAC Ethylene-responsive transcription factor 3 (Ethylene-responsive element-binding factor 3 homolog) (EREBP-5) (NtERF5) - Nicotiana tabacum (Common tobacco)weakly similar to ( 125) loc_os02g55380 12002.m10564 protein ethylene response factor 1, putative, expressed   no original description 	T
17.5.1	hormone metabolism.ethylene.synthesis-degradation	MDP0000360447	 moderately similar to ( 500) AT4G22880 | Symbols: LDOX, TDS4, TT18, ANS | LDOX (LEUCOANTHOCYANIDIN DIOXYGENASE); leucocyanidin oxygenase | chr4:12004905-12006059 REVERSEhighly similar to ( 619) LDOX_MALDO Leucoanthocyanidin dioxygenase (EC 1.14.11.19) (LDOX) (Leucocyanidin oxygenase) (Leucoanthocyanidin hydroxylase) (Anthocyanidin synthase) - Malus domestica (Apple) (Malus sylvestris)moderately similar to ( 318) loc_os01g27490 12001.m09199 protein leucoanthocyanidin dioxygenase, putative, expressed   no original description 	T
35.1.5	not assigned.no ontology.pentatricopeptide (PPR) repeat-containing protein	MDP0000178906	 moderately similar to ( 380) AT5G60540 | Symbols: EMB2407, ATPDX2, PDX2 | PDX2 (PYRIDOXINE BIOSYNTHESIS 2); glutaminase/ glutaminyl-tRNA synthase (glutamine-hydrolyzing)/ protein heterodimerization | chr5:24336874-24338647 REVERSEvery weakly similar to (80.1) RF1_ORYSA Rf1 protein, mitochondrial precursor (PPR protein) (Fertility restorer) (Restorer for CMS) - Oryza sativa (Rice)moderately similar to ( 329) loc_os02g03740 12002.m05723 protein glutamine amidotransferase subunit pdxT, putative, expressed  no original description 	T
30.5	signalling.G-proteins	MDP0000296871	 highly similar to ( 623) AT3G49350 | Symbols:  | RAB GTPase activator | chr3:18297663-18299846 REVERSEhighly similar to ( 569) loc_os09g35960 12009.m06547 protein GTPase activator protein of Rab-like small GTPases-like protein, putative, expressed  MSP1_C  no original description 	T
8.2.10	TCA / org transformation.other organic acid transformatons.malic	MDP0000376988	 weakly similar to ( 107) AT5G25880 | Symbols: ATNADP-ME3 | ATNADP-ME3 (NADP-malic enzyme 3); malate dehydrogenase (oxaloacetate-decarboxylating) (NADP+)/ malic enzyme/ oxidoreductase, acting on NADH or NADPH, NAD or NADP as acceptor | chr5:9024549-9028260 FORWARDweakly similar to ( 112) MAOX_VITVI NADP-dependent malic enzyme (EC 1.1.1.40) (NADP-ME) - Vitis vinifera (Grape)weakly similar to ( 105) loc_os01g52500 12001.m42715 protein NADP-dependent malic enzyme, putative, expressed no original description 	T
30.5	signalling.G-proteins	MDP0000460066	 moderately similar to ( 267) AT3G07880 | Symbols:  | Rho GDP-dissociation inhibitor family protein | chr3:2514175-2515544 FORWARDmoderately similar to ( 254) loc_os02g48730 12002.m09907 protein rho GDP-dissociation inhibitor 1, putative, expressed   no original description 	T
29.5.11	protein.degradation.ubiquitin	MDP0000143483	 very weakly similar to (94.0) AT4G22290 | Symbols:  | ubiquitin thiolesterase | chr4:11783199-11785730 REVERSEweakly similar to ( 102) loc_os04g51710 12004.m10066 protein cysteine-type endopeptidase/ ubiquitin thiolesterase, putative, expressed no original description 	T
27.4	RNA.RNA binding	MDP0000596125	 moderately similar to ( 255) AT1G17370 | Symbols: UBP1B | UBP1B (oligouridylate binding protein 1B); mRNA 3'-UTR binding | chr1:5951842-5954825 REVERSEvery weakly similar to (53.1) ROC1_SPIOL 28 kDa ribonucleoprotein, chloroplast (28RNP) - Spinacia oleracea (Spinach)moderately similar to ( 276) loc_os08g40880 12008.m08067 protein mRNA 3-UTR binding protein, putative, expressed   no original description 	T
29.2.1.2.2.80	protein.synthesis.ribosomal protein.eukaryotic.60S subunit.P0	MDP0000216270	 moderately similar to ( 291) AT1G25260 | Symbols:  | acidic ribosomal protein P0-related | chr1:8854163-8855766 REVERSEmoderately similar to ( 279) loc_os11g01420 12011.m28710 protein mRNA turnover protein 4, putative, expressed  no original description 	T
1.1.1.2	PS.lightreaction.photosystem II.PSII polypeptide subunits	MDP0000208621	 moderately similar to ( 280) AT4G05180 | Symbols: PSBQ, PSBQ-2, PSII-Q | PSBQ-2; calcium ion binding | chr4:2672093-2673170 REVERSEmoderately similar to ( 279) PSBQ_SPIOL Oxygen-evolving enhancer protein 3, chloroplast precursor (OEE3) (16 kDa subunit of oxygen evolving system of photosystem II) (OEC 16 kDa subunit) - Spinacia oleracea (Spinach)moderately similar to ( 226) loc_os07g36080 12007.m079706 protein oxygen-evolving enhancer protein 3-1, chloroplast precursor, putative, expressed  no original description 	T
11.1.8	lipid metabolism.FA synthesis and FA elongation.acyl coa ligase	MDP0000243706	 highly similar to ( 584) AT3G16170 | Symbols:  | acyl-activating enzyme 13 (AAE13) | chr3:5476490-5480128 FORWARDvery weakly similar to (96.3) 4CL2_ORYSA 4-coumarate--CoA ligase 2 (EC 6.2.1.12) (4CL 2) (4-coumaroyl-CoA synthase 2) - Oryza sativa (Rice)highly similar to ( 578) loc_os01g55590 12001.m150955 protein long-chain-fatty-acid-CoA ligase-like protein, putative, expressed   no original description 	T
23.4.1	nucleotide metabolism.phosphotransfer and pyrophosphatases.adenylate kinase	MDP0000783760	 moderately similar to ( 397) AT2G37250 | Symbols: ADK, ATPADK1 | ADK (ADENOSINE KINASE); adenylate kinase/ nucleotide kinase | chr2:15641991-15643318 FORWARDmoderately similar to ( 377) KADC_SOLTU Adenylate kinase, chloroplast precursor (EC 2.7.4.3) (ATP-AMP transphosphorylase) - Solanum tuberosum (Potato)moderately similar to ( 374) loc_os03g03820 12003.m05914 protein adenylate kinase, chloroplast precursor, putative, expressed  no original description 	T
10.2	cell wall.cellulose synthesis	MDP0000210684	 highly similar to ( 546) AT2G35650 | Symbols: ATCSLA07, CSLA07, ATCSLA7, CSLA7 | ATCSLA07 (CELLULOSE SYNTHASE LIKE); mannan synthase/ transferase, transferring glycosyl groups | chr2:14985625-14988187 FORWARDmoderately similar to ( 480) loc_os03g07350 12003.m06249 protein CSLA4 - cellulose synthase-like family A; mannan synthase, expressed   no original description 	T
30.3	signalling.calcium	MDP0000841118	 highly similar to ( 613) AT5G61900 | Symbols: BON1, CPN1, BON | BON1 (BONZAI 1); calcium-dependent phospholipid binding | chr5:24855908-24859150 REVERSEhighly similar to ( 590) loc_os02g32160 12002.m33700 protein copine-3, putative, expressed no original description 	T
35.2	not assigned.unknown	MDP0000300385	 highly similar to ( 615) AT3G07210 | Symbols:  | unknown protein | chr3:2293000-2295119 REVERSEmoderately similar to ( 489) loc_os01g68830 12001.m12985 protein expressed protein  no original description 	T
26.2	misc.UDP glucosyl and glucoronyl transferases	MDP0000197595	 highly similar to ( 773) AT4G02500 | Symbols: ATXT2, XXT2, XT2 | XT2 (UDP-XYLOSYLTRANSFERASE 2); UDP-xylosyltransferase/ transferase/ transferase, transferring glycosyl groups / xyloglucan 6-xylosyltransferase | chr4:1101638-1103345 FORWARDhighly similar to ( 665) loc_os03g18820 12003.m101316 protein glycosyltransferase 5, putative, expressed   no original description 	T
30.3	signalling.calcium	MDP0000282919	 nearly identical (1629) AT1G07670 | Symbols: ATECA4, ECA4 | calcium-transporting ATPase | chr1:2370305-2374196 REVERSEmoderately similar to ( 358) ACA5_ORYSA Probable calcium-transporting ATPase 5, plasma membrane-type (EC 3.6.3.8) (Ca(2+)-ATPase isoform 5) - Oryza sativa (Rice)nearly identical (1522) loc_os03g17310 12003.m07160 protein calcium-transporting ATPase 2, endoplasmic reticulum-type, putative, expressed    no original description 	T
26.7	misc.oxidases - copper, flavone etc	MDP0000138851	 moderately similar to ( 358) AT1G48910 | Symbols: YUC10 | YUC10; FAD binding / monooxygenase/ oxidoreductase | chr1:18091681-18093774 FORWARDmoderately similar to ( 375) loc_os12g08780 12012.m04864 protein disulfide oxidoreductase/ monooxygenase/ oxidoreductase, putative   no original description 	T
35.2	not assigned.unknown	MDP0000141906	 very weakly similar to (80.1) AT5G67350 | Symbols:  | unknown protein | chr5:26868521-26869429 REVERSE  no original description 	T
30.2.3	signalling.receptor kinases.leucine rich repeat III	MDP0000189396	 highly similar to ( 708) AT5G58300 | Symbols:  | leucine-rich repeat transmembrane protein kinase, putative | chr5:23572821-23574871 FORWARDmoderately similar to ( 202) PSKR_DAUCA Phytosulfokine receptor precursor (EC 2.7.11.1) (Phytosulfokine LRR receptor kinase) - Daucus carota (Carrot)highly similar to ( 642) loc_os01g60330 12001.m12161 protein ATP binding protein, putative, expressed   no original description 	T
29.2.3	protein.synthesis.initiation	MDP0000362168	 moderately similar to ( 214) AT1G54290 | Symbols:  | eukaryotic translation initiation factor SUI1, putative | chr1:20268710-20269390 REVERSEmoderately similar to ( 208) SUI1_BRAOL Protein translation factor SUI1 homolog (Translation initiation factor nps45) - Brassica oleracea (Wild cabbage)moderately similar to ( 211) loc_os05g41900 12005.m27899 protein protein translation factor SUI1, putative, expressed no original description 	T
27.3.35	RNA.regulation of transcription.bZIP transcription factor family	MDP0000297791	 moderately similar to ( 250) AT2G40620 | Symbols:  | bZIP transcription factor family protein | chr2:16954804-16956872 REVERSEmoderately similar to ( 241) RF2B_ORYSA Transcription factor RF2b - Oryza sativa (Rice)moderately similar to ( 242) loc_os03g21800 12003.m101333 protein transcription factor RF2b, putative, expressed   no original description 	T
29.2.1.2.2.31	protein.synthesis.ribosomal protein.eukaryotic.60S subunit.L31	MDP0000174784	 weakly similar to ( 132) AT2G19740 | Symbols:  | 60S ribosomal protein L31 (RPL31A) | chr2:8513577-8514346 FORWARDweakly similar to ( 123) RL31_PANGI 60S ribosomal protein L31 - Panax ginseng (Korean ginseng)weakly similar to ( 128) loc_os06g21480 12006.m06805 protein 60S ribosomal protein L31, putative, expressed no original description 	T
20.2.3	stress.abiotic.drought/salt	MDP0000016671	 nearly identical (1035) AT4G19120 | Symbols: ERD3 | ERD3 (early-responsive to dehydration 3) | chr4:10460665-10463034 REVERSEhighly similar to ( 540) loc_os06g01450 12006.m04884 protein methyltransferase, putative, expressed   no original description 	T
35.2	not assigned.unknown	MDP0000286949	 moderately similar to ( 218) AT3G18800 | Symbols:  | unknown protein | chr3:6479746-6480339 FORWARDweakly similar to ( 149) loc_os04g59050 12004.m10783 protein expressed protein   no original description 	T
29.2.1.2.1.8	protein.synthesis.ribosomal protein.eukaryotic.40S subunit.S8	MDP0000202935	 moderately similar to ( 300) AT5G59240 | Symbols:  | 40S ribosomal protein S8 (RPS8B) | chr5:23902626-23903670 REVERSEmoderately similar to ( 318) RS8_ORYSA 40S ribosomal protein S8 - Oryza sativa (Rice)moderately similar to ( 321) loc_os04g28180 12004.m07935 protein 40S ribosomal protein S8, putative, expressed no original description 	T
35.1	not assigned.no ontology	MDP0000240313	 moderately similar to ( 414) AT1G15330 | Symbols:  | CBS domain-containing protein | chr1:5274368-5275499 FORWARDmoderately similar to ( 358) loc_os02g06410 12002.m05989 protein pv42p, putative, expressed   no original description 	T
29.2.1.1.1.2.22	protein.synthesis.ribosomal protein.prokaryotic.chloroplast.50S subunit.L22	MDP0000388315	 moderately similar to ( 268) AT4G28360 | Symbols:  | ribosomal protein L22 family  protein | chr4:14029294-14030926 REVERSEvery weakly similar to (76.6) RK22_MARPO Chloroplast 50S ribosomal protein L22 - Marchantia polymorpha (Liverwort)moderately similar to ( 259) loc_os01g42980 12001.m150656 protein structural constituent of ribosome, putative, expressed no original description 	T
21.2.2	redox.ascorbate and glutathione.glutathione	MDP0000311291	 moderately similar to ( 319) AT4G31870 | Symbols: ATGPX7 | ATGPX7 (glutathione peroxidase 7); glutathione peroxidase | chr4:15410205-15411617 FORWARDmoderately similar to ( 313) GPX1_PEA Phospholipid hydroperoxide glutathione peroxidase, chloroplast precursor (EC 1.11.1.12) (PHGPx) - Pisum sativum (Garden pea)moderately similar to ( 288) loc_os06g08670 12006.m05586 protein phospholipid hydroperoxide glutathione peroxidase 1, chloroplast precursor, putative, expressed no original description 	T
21.2.1	redox.ascorbate and glutathione.ascorbate	MDP0000320539	 highly similar to ( 724) AT3G27820 | Symbols: ATMDAR4, MDAR4 | MDAR4 (MONODEHYDROASCORBATE REDUCTASE 4); monodehydroascorbate reductase (NADH) | chr3:10315249-10317881 FORWARDmoderately similar to ( 450) MDARS_CUCSA Monodehydroascorbate reductase, seedling isozyme (EC 1.6.5.4) (MDAR seedling) (Ascorbate free radical reductase seedling) (AFR reductase seedling) - Cucumis sativus (Cucumber)highly similar to ( 644) loc_os02g47800 12002.m09814 protein monodehydroascorbate reductase, cytoplasmic isoform 2, putative, expressed  no original description 	T
30.2.11	signalling.receptor kinases.leucine rich repeat XI	MDP0000657568	 moderately similar to ( 490) AT5G05850 | Symbols:  | leucine-rich repeat family protein | chr5:1762691-1764609 REVERSEvery weakly similar to (72.0) RPK1_IPONI Receptor-like protein kinase precursor (EC 2.7.11.1) - Ipomoea nil (Japanese morning glory) (Pharbitis nil)moderately similar to ( 366) loc_os04g40080 12004.m08992 protein protein lap1, putative, expressed   no original description 	T
10.7	cell wall.modification	MDP0000302385	 moderately similar to ( 480) AT2G36870 | Symbols:  | xyloglucan:xyloglucosyl transferase, putative / xyloglucan endotransglycosylase, putative / endo-xyloglucan transferase, putative | chr2:15472869-15474630 REVERSEweakly similar to ( 200) XTH_TOBAC Probable xyloglucan endotransglucosylase/hydrolase protein precursor (EC 2.4.1.207) - Nicotiana tabacum (Common tobacco)moderately similar to ( 382) loc_os07g29750 12007.m07246 protein xyloglucan endotransglucosylase/hydrolase protein 32 precursor, putative, expressed   no original description 	T
35.1	not assigned.no ontology	MDP0000305096	 nearly identical (1084) AT5G35160 | Symbols:  | LOCATED IN: integral to membrane, Golgi apparatus; EXPRESSED IN: 24 plant structures; EXPRESSED DURING: 13 growth stages; CONTAINS InterPro DOMAIN/s: Nonaspanin (TM9SF) (InterPro:IPR004240); BEST Arabidopsis thaliana protein match is: unknown protein (TAIR:AT4G12650.1); Has 1053 Blast hits to 1004 proteins in 163 species: Archae - 0; Bacteria - 0; Metazoa - 478; Fungi - 143; Plants - 250; Viruses - 0; Other Eukaryotes - 182 (source: NCBI BLink). | chr5:13414945-13416921 FORWARDnearly identical (1073) loc_os02g55440 12002.m100394 protein transmembrane 9 superfamily protein member 2 precursor, putative, expressed  no original description 	T
31.3.1	cell.cycle.peptidylprolyl isomerase	MDP0000363054	 moderately similar to ( 326) AT5G58710 | Symbols: ROC7 | ROC7; peptidyl-prolyl cis-trans isomerase | chr5:23717840-23719495 FORWARDmoderately similar to ( 221) CYPH_CATRO Peptidyl-prolyl cis-trans isomerase (EC 5.2.1.8) (PPIase) (Rotamase) (Cyclophilin) (Cyclosporin A-binding protein) - Catharanthus roseus (Rosy periwinkle) (Madagascar periwinkle)moderately similar to ( 320) loc_os06g49480 12006.m32182 protein peptidyl-prolyl cis-trans isomerase CYP19-4 precursor, putative, expressed  no original description 	T
11.1.6	lipid metabolism.FA synthesis and FA elongation.enoyl ACP reductase	MDP0000160972	 highly similar to ( 536) AT2G05990 | Symbols: MOD1, ENR1 | MOD1 (MOSAIC DEATH 1); enoyl-[acyl-carrier-protein] reductase (NADH)/ enoyl-[acyl-carrier-protein] reductase/ oxidoreductase | chr2:2322876-2324867 FORWARDhighly similar to ( 523) FABI_BRANA Enoyl-[acyl-carrier-protein] reductase [NADH], chloroplast precursor (EC 1.3.1.9) (NADH-dependent enoyl-ACP reductase) - Brassica napus (Rape)moderately similar to ( 489) loc_os08g23810 12008.m26662 protein enoyl-[acyl-carrier-protein] reductase [NADH], chloroplast precursor, expressed  no original description 	T
30.4	signalling.phosphinositides	MDP0000201559	 weakly similar to ( 129) AT4G20260 | Symbols: ATPCAP1, PCAP1 | DREPP plasma membrane polypeptide family protein | chr4:10941593-10943227 FORWARDvery weakly similar to (80.5) SRS1_ORYSA Salt-stress root protein RS1 - Oryza sativa (Rice)weakly similar to ( 119) loc_os02g18410 12002.m07084 protein DREPP2 protein, putative, expressed   no original description 	T
20.2.3	stress.abiotic.drought/salt	MDP0000201559	 weakly similar to ( 129) AT4G20260 | Symbols: ATPCAP1, PCAP1 | DREPP plasma membrane polypeptide family protein | chr4:10941593-10943227 FORWARDvery weakly similar to (80.5) SRS1_ORYSA Salt-stress root protein RS1 - Oryza sativa (Rice)weakly similar to ( 119) loc_os02g18410 12002.m07084 protein DREPP2 protein, putative, expressed   no original description 	T
29.5.11.4.3.2	protein.degradation.ubiquitin.E3.SCF.FBOX	MDP0000308771	 moderately similar to ( 355) AT4G08980 | Symbols:  | F-box family protein (FBW2) | chr4:5758993-5760108 FORWARDmoderately similar to ( 330) loc_os02g52130 12002.m100364 protein ubiquitin-protein ligase, putative, expressed  no original description 	T
35.2	not assigned.unknown	MDP0000120180	   no original description 	T
35.2	not assigned.unknown	MDP0000120885	 highly similar to ( 558) AT2G12400 | Symbols:  | unknown protein | chr2:5005144-5008140 REVERSEhighly similar to ( 505) loc_os02g55590 12002.m10585 protein expressed protein  MSP1_C  no original description 	T
31.3	cell.cycle	MDP0000180375	 weakly similar to ( 184) AT2G42260 | Symbols: UVI4, PYM | UVI4 (UV-B-INSENSITIVE 4) | chr2:17602240-17603283 REVERSEvery weakly similar to (70.5) loc_os02g37850 12002.m100254 protein expressed protein  no original description 	T
35.2	not assigned.unknown	MDP0000484244	 very weakly similar to (68.9) AT5G05220 | Symbols:  | unknown protein | chr5:1550273-1550821 FORWARDvery weakly similar to (58.2) loc_os03g21650 12003.m07569 protein expressed protein   no original description 	T
35.2	not assigned.unknown	MDP0000204385	 very weakly similar to (87.0) AT4G15802 | Symbols:  | FUNCTIONS IN: molecular_function unknown; INVOLVED IN: biological_process unknown; LOCATED IN: cellular_component unknown; CONTAINS InterPro DOMAIN/s: Heat shock factor binding 1 (InterPro:IPR009643); Has 178 Blast hits to 178 proteins in 62 species: Archae - 0; Bacteria - 0; Metazoa - 122; Fungi - 0; Plants - 37; Viruses - 0; Other Eukaryotes - 19 (source: NCBI BLink). | chr4:8986864-8988339 REVERSEvery weakly similar to (56.2) loc_os09g20830 12009.m60264 protein heat shock factor-binding protein 1, putative, expressed no original description 	T
27.3.7	RNA.regulation of transcription.C2C2(Zn) CO-like, Constans-like zinc finger family	MDP0000298635	 moderately similar to ( 330) AT3G02380 | Symbols: COL2 | COL2 (constans-like 2); transcription factor/ zinc ion binding | chr3:487438-488624 REVERSEmoderately similar to ( 234) loc_os06g16370 12006.m06350 protein zinc finger protein CONSTANS, putative, expressed   no original description 	T
17.3.1.2.99	hormone metabolism.brassinosteroid.synthesis-degradation.sterols.other	MDP0000239289	 moderately similar to ( 405) AT1G58440 | Symbols: XF1, SQE1 | XF1; squalene monooxygenase | chr1:21714094-21717246 REVERSEmoderately similar to ( 428) ERG1_PANGI Squalene monooxygenase (EC 1.14.99.7) (Squalene epoxidase) (SE) - Panax ginseng (Korean ginseng)moderately similar to ( 431) loc_os03g12910 12003.m101256 protein squalene monooxygenase, putative, expressed   no original description 	T
27.3.11	RNA.regulation of transcription.C2H2 zinc finger family	MDP0000254462	 weakly similar to ( 166) AT3G02790 | Symbols:  | zinc finger (C2H2 type) family protein | chr3:604926-605243 FORWARDweakly similar to ( 172) loc_os06g49490 12006.m09473 protein expressed protein no original description 	T
29.5.11.4.2	protein.degradation.ubiquitin.E3.RING	MDP0000889931	 weakly similar to ( 158) AT4G27470 | Symbols:  | zinc finger (C3HC4-type RING finger) family protein | chr4:13735576-13736307 FORWARDweakly similar to ( 120) loc_os02g42690 12002.m33794 protein zinc finger, C3HC4 type family protein, expressed  no original description 	T
29.5.11.4.3.2	protein.degradation.ubiquitin.E3.SCF.FBOX	MDP0000891985	 highly similar to ( 560) AT5G07670 | Symbols:  | F-box family protein | chr5:2430421-2432065 FORWARDmoderately similar to ( 421) loc_os02g44104 12002.m100300 protein Leucine Rich Repeat family protein, expressed  no original description 	T
35.2	not assigned.unknown	MDP0000573321	 moderately similar to ( 388) AT5G10010 | Symbols:  | unknown protein | chr5:3128098-3131452 FORWARDmoderately similar to ( 348) loc_os04g56590 12004.m35061 protein ATP/GTP binding protein, putative, expressed no original description 	T
30.1	signalling.in sugar and nutrient physiology	MDP0000131279	 moderately similar to ( 214) AT4G01900 | Symbols: PII, GLB1 | GLB1 (GLNB1 HOMOLOG); acetylglutamate kinase regulator | chr4:821736-823294 FORWARDweakly similar to ( 196) loc_os05g04220 12005.m04955 protein nitrogen regulatory protein P-II, putative, expressed  no original description 	T
23.4.1	nucleotide metabolism.phosphotransfer and pyrophosphatases.adenylate kinase	MDP0000945557	 moderately similar to ( 458) AT5G35170 | Symbols:  | adenylate kinase family protein | chr5:13419278-13423381 FORWARDmoderately similar to ( 423) loc_os08g19140 12008.m05984 protein ATP binding protein, putative, expressed no original description 	T
34.9	transport.metabolite transporters at the mitochondrial membrane	MDP0000309670	 moderately similar to ( 463) AT4G27940 | Symbols: ATMTM1, MTM1 | mitochondrial substrate carrier family protein | chr4:13904745-13907036 FORWARDmoderately similar to ( 434) loc_os08g29720 12008.m26494 protein mitochondrial carrier protein CGI-69, putative, expressed   no original description 	T
35.2	not assigned.unknown	MDP0000764263	   no original description 	T
4.3.5	glycolysis.unclear/dually targeted.pyrophosphate-fructose-6-P phosphotransferase	MDP0000324386	 moderately similar to ( 373) AT1G12000 | Symbols:  | pyrophosphate--fructose-6-phosphate 1-phosphotransferase beta subunit, putative / pyrophosphate-dependent 6-phosphofructose-1-kinase, putative | chr1:4050159-4053727 REVERSEmoderately similar to ( 372) PFPB_RICCO Pyrophosphate--fructose 6-phosphate 1-phosphotransferase subunit beta (EC 2.7.1.90) (PFP) (6-phosphofructokinase, pyrophosphate dependent) (Pyrophosphate-dependent 6-phosphofructose-1-kinase) (PPi-PFK) - Ricinus communis (Castor bean)moderately similar to ( 340) loc_os06g13810 12006.m31864 protein pyrophosphate--fructose 6-phosphate 1-phosphotransferase beta subunit, putative, expressed  no original description 	T
26.4.1	misc.beta 1,3 glucan hydrolases.glucan endo-1,3-beta-glucosidase	MDP0000660874	 highly similar to ( 724) AT2G05790 | Symbols:  | glycosyl hydrolase family 17 protein | chr2:2199450-2201293 FORWARDmoderately similar to ( 288) E13B_WHEAT Glucan endo-1,3-beta-glucosidase precursor (EC 3.2.1.39) ((1->3)-beta-glucan endohydrolase) ((1->3)-beta-glucanase) (Beta-1,3-endoglucanase) - Triticum aestivum (Wheat)highly similar to ( 544) loc_os02g53200 12002.m10349 protein glucan endo-1,3-beta-glucosidase 7 precursor, putative, expressed   no original description 	T
26.22	misc.short chain dehydrogenase/reductase (SDR)	MDP0000285612	 moderately similar to ( 309) AT3G61220 | Symbols:  | short-chain dehydrogenase/reductase (SDR) family protein | chr3:22663025-22664316 FORWARDvery weakly similar to (56.2) SDR1_PICAB Short-chain type dehydrogenase/reductase (EC 1.-.-.-) - Picea abies (Norway spruce) (Picea excelsa)moderately similar to ( 275) loc_os04g44920 12004.m101595 protein short-chain dehydrogenase/reductase SDR, putative, expressed   no original description 	T
27.3.6	RNA.regulation of transcription.bHLH,Basic Helix-Loop-Helix family	MDP0000319726	 highly similar to ( 502) AT1G68820 | Symbols:  | membrane protein, putative | chr1:25865852-25868800 FORWARDmoderately similar to ( 447) loc_os03g49900 12003.m09988 protein protein binding protein, putative, expressed   no original description 	T
35.2	not assigned.unknown	MDP0000813361	 moderately similar to ( 261) AT3G07090 | Symbols:  | unknown protein | chr3:2243153-2244476 REVERSEmoderately similar to ( 245) loc_os02g56900 12002.m10712 protein thioredoxin, putative, expressed no original description 	T
29.5	protein.degradation	MDP0000339664	 moderately similar to ( 262) AT1G67700 | Symbols:  | unknown protein | chr1:25374295-25375810 FORWARDweakly similar to ( 186) loc_os02g38820 12002.m77827 protein expressed protein no original description 	T
35.2	not assigned.unknown	MDP0000286578	 nearly identical (2217) AT4G30990 | Symbols:  | binding | chr4:15084456-15097860 FORWARDnearly identical (1721) loc_os01g40250 12001.m10298 protein HEAT repeat family protein, expressed  no original description 	T
35.2	not assigned.unknown	MDP0000130479	 highly similar to ( 687) AT4G40050 | Symbols:  | signal transducer | chr4:18561206-18563727 REVERSEhighly similar to ( 573) loc_os09g29470 12009.m21999 protein expressed protein  no original description 	T
31.2	cell.division	MDP0000607749	 moderately similar to ( 440) AT5G40770 | Symbols: ATPHB3 | ATPHB3 (PROHIBITIN 3) | chr5:16315589-16316621 REVERSEmoderately similar to ( 393) loc_os02g37000 12002.m08788 protein mitochondrial prohibitin complex protein 1, putative, expressed   no original description 	T
35.2	not assigned.unknown	MDP0000438977	 weakly similar to ( 149) AT5G53390 | Symbols:  | unknown protein | chr5:21656951-21659207 REVERSEweakly similar to ( 154) loc_os01g48874 12001.m11075 protein expressed protein no original description 	T
29.4	protein.postranslational modification	MDP0000060858	 highly similar to ( 628) AT1G50370 | Symbols:  | serine/threonine protein phosphatase, putative | chr1:18658894-18661672 FORWARDmoderately similar to ( 380) PP2A3_ORYSA Serine/threonine-protein phosphatase PP2A-3 catalytic subunit (EC 3.1.3.16) - Oryza sativa (Rice)highly similar to ( 605) loc_os01g49690 12001.m11156 protein serine/threonine-protein phosphatase 6, putative, expressed no original description 	T
30.5	signalling.G-proteins	MDP0000215771	 moderately similar to ( 212) AT4G18800 | Symbols: ATHSGBP, ATRAB11B, ATRABA1D | ATRABA1D (ARABIDOPSIS RAB GTPASE HOMOLOG A1D); GTP binding | chr4:10320156-10321339 REVERSEmoderately similar to ( 212) RIC2_ORYSA Ras-related protein RIC2 - Oryza sativa (Rice)moderately similar to ( 212) loc_os06g35814 12006.m08118 protein ras-related protein RIC2, putative, expressed no original description 	T
10.7	cell wall.modification	MDP0000139485	 moderately similar to ( 477) AT1G32170 | Symbols: XTR4, XTH30 | XTR4 (XYLOGLUCAN ENDOTRANSGLYCOSYLASE 4); hydrolase, acting on glycosyl bonds / hydrolase, hydrolyzing O-glycosyl compounds / xyloglucan:xyloglucosyl transferase | chr1:11575434-11577776 FORWARDmoderately similar to ( 201) XTH_WHEAT Probable xyloglucan endotransglucosylase/hydrolase precursor (EC 2.4.1.207) - Triticum aestivum (Wheat)moderately similar to ( 383) loc_os02g03550 12002.m05705 protein xyloglucan endotransglucosylase/hydrolase protein 30 precursor, putative, expressed  COG3889  no original description 	T
26.13	misc.acid and other phosphatases	MDP0000219684	 highly similar to ( 838) AT5G50400 | Symbols: ATPAP27, PAP27 | PAP27 (PURPLE ACID PHOSPHATASE 27); acid phosphatase/ protein serine/threonine phosphatase | chr5:20523575-20526231 REVERSEhighly similar to ( 555) NPP_HORVU Nucleotide pyrophosphatase/phosphodiesterase (EC 3.-.-.-) (Fragments) - Hordeum vulgare (Barley)highly similar to ( 815) loc_os08g41880 12008.m08166 protein nucleotide pyrophosphatase/phosphodiesterase, putative, expressed  no original description 	T
10.2.2	cell wall.cellulose synthesis.COBRA	MDP0000288732	 highly similar to ( 771) AT5G60920 | Symbols: COB | COB (COBRA) | chr5:24511466-24513932 REVERSEhighly similar to ( 744) COBL3_ORYSA COBRA-like 3 protein precursor (BRITTLE CULM1-like 4 protein) - Oryza sativa (Rice)highly similar to ( 744) loc_os05g32110 12005.m083711 protein COBRA-like protein 4 precursor, putative, expressed no original description 	T
29.5.11.4.2	protein.degradation.ubiquitin.E3.RING	MDP0000171955	 moderately similar to ( 343) AT5G01450 | Symbols:  | protein binding / zinc ion binding | chr5:183693-186244 REVERSEweakly similar to ( 110) loc_os05g44550 12005.m08581 protein ubiquitin-protein ligase/ zinc ion binding protein, putative no original description 	T
29.2.1.1.1.1.6	protein.synthesis.ribosomal protein.prokaryotic.chloroplast.30S subunit.S6	MDP0000365305	 moderately similar to ( 222) AT1G64510 | Symbols:  | ribosomal protein S6 family protein | chr1:23954993-23956205 REVERSEmoderately similar to ( 214) RR6_SPIOL 30S ribosomal protein S6 alpha, chloroplast precursor [Contains: 30S ribosomal protein S6 beta; 30S ribosomal protein S6 gamma; 30S ribosomal protein S6 delta; 30S ribosomal protein S6 epsilon] (Fragment) - Spinacia oleracea (Spinach)weakly similar to ( 182) loc_os03g62630 12003.m11140 protein structural constituent of ribosome, putative, expressed  no original description 	T
35.2	not assigned.unknown	MDP0000270746	 weakly similar to ( 146) AT1G55265 | Symbols:  | unknown protein | chr1:20617326-20617853 FORWARDweakly similar to ( 113) loc_os03g48626 12003.m35577 protein expressed protein  no original description 	T
35.1	not assigned.no ontology	MDP0000244949	 moderately similar to ( 273) AT3G60600 | Symbols: VAP27-1 | VAP (VESICLE ASSOCIATED PROTEIN); protein binding | chr3:22400537-22401669 FORWARDmoderately similar to ( 268) loc_os08g06020 12008.m04743 protein VAP27, putative, expressed no original description 	T
27.3.32	RNA.regulation of transcription.WRKY domain transcription factor family	MDP0000202292	 weakly similar to ( 133) AT2G34830 | Symbols: WRKY35, MEE24, AtWRKY35 | WRKY35 (WRKY DNA-binding protein 35); transcription factor | chr2:14693839-14696378 REVERSEweakly similar to ( 134) loc_os02g47060 12002.m09741 protein OsWRKY66 - Superfamily of rice TFs having WRKY and zinc finger domains, expressed no original description 	T
29.2.1.2.2.7	protein.synthesis.ribosomal protein.eukaryotic.60S subunit.L7	MDP0000156365	 moderately similar to ( 398) AT2G01250 | Symbols:  | 60S ribosomal protein L7 (RPL7B) | chr2:133038-134264 REVERSEmoderately similar to ( 374) loc_os04g51630 12004.m35453 protein 60S ribosomal protein L7-2, putative, expressed  no original description 	T
29.4	protein.postranslational modification	MDP0000880912	 highly similar to ( 543) AT2G34650 | Symbols: PID, ABR | PID (PINOID); kinase/ protein kinase/ protein serine/threonine kinase | chr2:14589934-14591557 REVERSEmoderately similar to ( 357) G11A_ORYSA Protein kinase G11A (EC 2.7.11.1) - Oryza sativa (Rice)moderately similar to ( 426) loc_os12g42020 12012.m07983 protein protein kinase PVPK-1, putative, expressed  no original description 	T
29.2.2	protein.synthesis.ribosome biogenesis	MDP0000251050	 moderately similar to ( 309) AT4G15770 | Symbols:  | RNA binding / protein binding | chr4:8978060-8978623 FORWARDmoderately similar to ( 244) loc_os07g47420 12007.m08959 protein 60S ribosome subunit biogenesis protein NIP7, putative, expressed   no original description 	T
27.3.21	RNA.regulation of transcription.GRAS transcription factor family	MDP0000134341	 moderately similar to ( 412) AT1G14920 | Symbols: GAI, RGA2 | GAI (GIBBERELLIC ACID INSENSITIVE); transcription factor | chr1:5149414-5151015 FORWARDmoderately similar to ( 404) GAI1_VITVI DELLA protein GAI1 (Gibberellic acid-insensitive mutant protein 1) (VvGAI1) - Vitis vinifera (Grape)moderately similar to ( 415) loc_os01g45860 12001.m10786 protein DELLA protein RGA, putative, expressed   no original description 	T
5.10	fermentation.aldehyde dehydrogenase	MDP0000531301	 highly similar to ( 692) AT4G34240 | Symbols: ALDH3I1, ALDH3 | ALDH3I1 (ALDEHYDE DEHYDROGENASE 3|1); 3-chloroallyl aldehyde dehydrogenase/ aldehyde dehydrogenase (NAD) | chr4:16389801-16392633 FORWARDweakly similar to ( 157) BADH_SPIOL Betaine-aldehyde dehydrogenase, chloroplast precursor (EC 1.2.1.8) (BADH) - Spinacia oleracea (Spinach)highly similar to ( 634) loc_os11g08300 12011.m28749 protein aldehyde dehydrogenase, dimeric NADP-preferring, putative, expressed  no original description 	T
29.4.1.57	protein.postranslational modification.kinase.receptor like cytoplasmatic kinase VII	MDP0000202468	 highly similar to ( 542) AT5G18910 | Symbols:  | protein kinase family protein | chr5:6306994-6309396 REVERSEweakly similar to ( 185) NORK_MEDTR Nodulation receptor kinase precursor (EC 2.7.11.1) (Does not make infections protein 2) (Symbiosis receptor-like kinase) (MtSYMRK) - Medicago truncatula (Barrel medic)moderately similar to ( 401) loc_os06g45350 12006.m31915 protein ATP binding protein, putative, expressed  no original description 	T
35.2	not assigned.unknown	MDP0000912759	 moderately similar to ( 227) AT5G48120 | Symbols:  | binding | chr5:19508210-19514774 REVERSE no original description 	T
35.1	not assigned.no ontology	MDP0000834172	 moderately similar to ( 304) AT2G45600 | Symbols:  | hydrolase | chr2:18789799-18790788 FORWARDweakly similar to ( 128) GID1_ORYSA Gibberellin receptor GID1 (EC 3.-.-.-) (Gibberellin-insensitive dwarf protein 1) (Protein GIBBERELLIN INSENSITIVE DWARF1) - Oryza sativa (Rice)moderately similar to ( 241) loc_os08g43430 12008.m08317 protein gibberellin receptor GID1L2, putative, expressed   COG4907  no original description 	T
35.2	not assigned.unknown	MDP0000321075	 moderately similar to ( 366) AT4G09580 | Symbols:  | INVOLVED IN: biological_process unknown; LOCATED IN: endoplasmic reticulum; EXPRESSED IN: 24 plant structures; EXPRESSED DURING: 13 growth stages; CONTAINS InterPro DOMAIN/s: SNARE associated Golgi protein (InterPro:IPR015414); BEST Arabidopsis thaliana protein match is: unknown protein (TAIR:AT1G71940.1); Has 2023 Blast hits to 2023 proteins in 496 species: Archae - 2; Bacteria - 1014; Metazoa - 198; Fungi - 27; Plants - 131; Viruses - 0; Other Eukaryotes - 651 (source: NCBI BLink). | chr4:6052721-6054313 REVERSEmoderately similar to ( 289) loc_os03g49700 12003.m09969 protein gtk16 protein, putative, expressed no original description 	T
26.28	misc.GDSL-motif lipase	MDP0000771647	 highly similar to ( 531) AT5G14450 | Symbols:  | GDSL-motif lipase/hydrolase family protein | chr5:4658488-4660034 FORWARDmoderately similar to ( 335) EST_HEVBR Esterase precursor (EC 3.1.1.-) (Early nodule-specific protein homolog) (Latex allergen Hev b 13) - Hevea brasiliensis (Para rubber tree)moderately similar to ( 350) loc_os07g39740 12007.m08216 protein esterase precursor, putative, expressed   no original description 	T
29.5.3	protein.degradation.cysteine protease	MDP0000311720	 weakly similar to ( 127) AT5G60360 | Symbols: AALP | AALP (Arabidopsis aleurain-like protease); cysteine-type peptidase | chr5:24280044-24282157 FORWARDvery weakly similar to (94.0) ORYC_ORYSA Oryzain gamma chain precursor (EC 3.4.22.-) - Oryza sativa (Rice)very weakly similar to (94.0) loc_os09g27030 12009.m059986 protein thiol protease aleurain precursor, putative, expressed no original description 	T
35.2	not assigned.unknown	MDP0000250852	 moderately similar to ( 219) AT2G28305 | Symbols:  | unknown protein | chr2:12081186-12084307 FORWARDmoderately similar to ( 201) loc_os02g41770 12002.m09214 protein carboxy-lyase, putative no original description 	T
35.2	not assigned.unknown	MDP0000739019	 highly similar to ( 851) AT4G08810 | Symbols: SUB1 | SUB1; calcium ion binding | chr4:5616204-5617862 REVERSEhighly similar to ( 661) loc_os07g43990 12007.m08622 protein expressed protein  no original description 	T
26.3.2	misc.gluco-, galacto- and mannosidases.beta-galactosidase	MDP0000682327	 moderately similar to ( 325) AT2G28470 | Symbols: BGAL8 | BGAL8 (beta-galactosidase 8); beta-galactosidase/ catalytic/ cation binding / sugar binding | chr2:12169047-12173164 REVERSEmoderately similar to ( 211) BGAL_ASPOF Beta-galactosidase precursor (EC 3.2.1.23) (Lactase) - Asparagus officinalis (Garden asparagus)moderately similar to ( 313) loc_os03g15020 12003.m34824 protein beta-galactosidase precursor, putative, expressed  no original description 	T
29.5.11.4.3.2	protein.degradation.ubiquitin.E3.SCF.FBOX	MDP0000379288	 highly similar to ( 534) AT1G21410 | Symbols: SKP2A | SKP2A | chr1:7497479-7499386 FORWARDmoderately similar to ( 462) loc_os05g35110 12005.m07741 protein F-box/LRR-repeat protein 2, putative, expressed   no original description 	T
35.2	not assigned.unknown	MDP0000236634	 weakly similar to ( 101) AT2G03680 | Symbols: SPR1, SKU6 | SPR1 (SPIRAL1) | chr2:1121398-1121850 FORWARDvery weakly similar to ( 100) loc_os11g41150 12011.m07958 protein SPR1, putative, expressed   no original description 	T
27.3.99	RNA.regulation of transcription.unclassified	MDP0000287206	 weakly similar to ( 138) AT5G07900 | Symbols:  | mitochondrial transcription termination factor family protein / mTERF family protein | chr5:2520188-2521405 FORWARDweakly similar to ( 131) loc_os06g12060 12006.m05923 protein mTERF family protein, expressed  TrbL  no original description 	T
29.2.1.2.2.24	protein.synthesis.ribosomal protein.eukaryotic.60S subunit.L24	MDP0000161898	 moderately similar to ( 213) AT3G53020 | Symbols: STV1, RPL24B, RPL24 | STV1 (SHORT VALVE1); structural constituent of ribosome | chr3:19660749-19661912 REVERSEmoderately similar to ( 214) RL24_CICAR 60S ribosomal protein L24 - Cicer arietinum (Chickpea) (Garbanzo)moderately similar to ( 210) loc_os01g59990 12001.m43406 protein 60S ribosomal protein L24, putative, expressed no original description 	T
30.2.11	signalling.receptor kinases.leucine rich repeat XI	MDP0000322294	 nearly identical (1175) AT5G61480 | Symbols:  | leucine-rich repeat transmembrane protein kinase, putative | chr5:24724541-24727842 REVERSEmoderately similar to ( 455) RPK1_IPONI Receptor-like protein kinase precursor (EC 2.7.11.1) - Ipomoea nil (Japanese morning glory) (Pharbitis nil)highly similar to ( 847) loc_os08g05290 12008.m04672 protein receptor-like protein kinase 5 precursor, putative, expressed   no original description 	T
26.9	misc.glutathione S transferases	MDP0000169428	 moderately similar to ( 281) AT3G62760 | Symbols: ATGSTF13 | ATGSTF13; glutathione transferase | chr3:23217425-23218246 REVERSEmoderately similar to ( 232) GSTH1_ORYSA Probable glutathione S-transferase GSTF1 (EC 2.5.1.18) (GST-I) - Oryza sativa (Rice)moderately similar to ( 244) loc_os01g27260 12001.m09177 protein glutathione S-transferase GSTF1, putative, expressed no original description 	T
26.4.1	misc.beta 1,3 glucan hydrolases.glucan endo-1,3-beta-glucosidase	MDP0000321387	 moderately similar to ( 321) AT4G29360 | Symbols:  | glycosyl hydrolase family 17 protein | chr4:14451562-14453687 REVERSEmoderately similar to ( 280) E13B_WHEAT Glucan endo-1,3-beta-glucosidase precursor (EC 3.2.1.39) ((1->3)-beta-glucan endohydrolase) ((1->3)-beta-glucanase) (Beta-1,3-endoglucanase) - Triticum aestivum (Wheat)moderately similar to ( 330) loc_os03g46660 12003.m09685 protein glucan endo-1,3-beta-glucosidase 7 precursor, putative, expressed   no original description 	T
29.3.3	protein.targeting.chloroplast	MDP0000534726	 moderately similar to ( 270) AT3G23710 | Symbols:  | chloroplast inner membrane import protein Tic22, putative | chr3:8534061-8536148 FORWARDweakly similar to ( 189) loc_os07g19030 12007.m06342 protein protein translocase, putative, expressed  no original description 	T
35.2	not assigned.unknown	MDP0000215299	 highly similar to ( 643) AT5G50150 | Symbols:  | unknown protein | chr5:20402444-20406375 REVERSEhighly similar to ( 551) loc_os06g28000 12006.m07345 protein carboxyl-terminal proteinase, putative, expressed   no original description 	T
35.1	not assigned.no ontology	MDP0000319827	 moderately similar to ( 289) AT4G23630 | Symbols: BTI1 | BTI1 (VIRB2-INTERACTING PROTEIN 1) | chr4:12318070-12319574 FORWARDmoderately similar to ( 291) loc_os01g53520 12001.m43176 protein seed maturation protein, putative, expressed  no original description 	T
21.4	redox.glutaredoxins	MDP0000178508	 moderately similar to ( 254) AT3G57070 | Symbols:  | glutaredoxin family protein | chr3:21124174-21125427 FORWARDweakly similar to ( 189) loc_os03g24030 12003.m07749 protein electron transporter, putative, expressed  no original description 	T
27.4	RNA.RNA binding	MDP0000235584	 highly similar to ( 758) AT5G61960 | Symbols: AML1 | AML1 (ARABIDOPSIS MEI2-LIKE PROTEIN 1); RNA binding / protein binding | chr5:24879001-24883483 REVERSEweakly similar to ( 101) TE1_MAIZE Protein terminal ear1 - Zea mays (Maize)highly similar to ( 770) loc_os02g31290 12002.m33391 protein mei2-like protein, putative, expressed  no original description 	T
34.9	transport.metabolite transporters at the mitochondrial membrane	MDP0000228473	 moderately similar to ( 416) AT1G07030 | Symbols:  | mitochondrial substrate carrier family protein | chr1:2158631-2160524 REVERSEvery weakly similar to (90.5) BT1_MAIZE Protein brittle-1, chloroplast precursor - Zea mays (Maize)moderately similar to ( 358) loc_os03g18550 12003.m34847 protein mitochondrial RNA-splicing protein MRS3, putative, expressed  no original description 	T
31.1	cell.organisation	MDP0000648540	 weakly similar to ( 152) AT3G23400 | Symbols:  | plastid-lipid associated protein PAP / fibrillin family protein | chr3:8376636-8378225 REVERSEweakly similar to ( 154) loc_os11g38260 12011.m07673 protein harpin binding protein 1, putative, expressed no original description 	T
35.2	not assigned.unknown	MDP0000191996	 moderately similar to ( 355) AT3G03890 | Symbols:  | FMN binding | chr3:999837-1001996 REVERSEmoderately similar to ( 319) loc_os07g38620 12007.m29233 protein root border cell-specific protein, putative, expressed   no original description 	T
2.2.1.5	major CHO metabolism.degradation.sucrose.Susy	MDP0000277711	 nearly identical (1020) AT5G37180 | Symbols: SUS5, ATSUS5 | SUS5; UDP-glycosyltransferase/ sucrose synthase | chr5:14718238-14722913 FORWARDhighly similar to ( 808) SUS2_PEA Sucrose synthase 2 (EC 2.4.1.13) (Sucrose-UDP glucosyltransferase 2) - Pisum sativum (Garden pea)highly similar to ( 993) loc_os04g24430 12004.m07574 protein sucrose synthase 2, putative, expressed  no original description 	T
11.9.3.1	lipid metabolism.lipid degradation.lysophospholipases.phospholipase D	MDP0000304811	 moderately similar to ( 356) AT4G35790 | Symbols: ATPLDDELTA, PLDDELTA | ATPLDDELTA; phospholipase D | chr4:16955774-16959875 REVERSEmoderately similar to ( 271) PLDA1_MAIZE Phospholipase D alpha 1 (EC 3.1.4.4) (PLD alpha 1) (Choline phosphatase 1) (Phosphatidylcholine-hydrolyzing phospholipase D 1) - Zea mays (Maize)moderately similar to ( 339) loc_os07g15680 12007.m06012 protein phospholipase D beta 1, putative, expressed  MSP1_C  no original description 	T
26.28	misc.GDSL-motif lipase	MDP0000864726	 moderately similar to ( 407) AT3G26430 | Symbols:  | GDSL-motif lipase/hydrolase family protein | chr3:9674419-9675889 FORWARDmoderately similar to ( 344) EST_HEVBR Esterase precursor (EC 3.1.1.-) (Early nodule-specific protein homolog) (Latex allergen Hev b 13) - Hevea brasiliensis (Para rubber tree)moderately similar to ( 371) loc_os07g39750 12007.m079815 protein alpha-L-fucosidase 2 precursor, putative, expressed  no original description 	T
26.2	misc.UDP glucosyl and glucoronyl transferases	MDP0000168165	 highly similar to ( 842) AT5G15470 | Symbols: GAUT14 | GAUT14 (Galacturonosyltransferase 14); polygalacturonate 4-alpha-galacturonosyltransferase/ transferase, transferring glycosyl groups / transferase, transferring hexosyl groups | chr5:5021433-5024168 REVERSEhighly similar to ( 728) loc_os12g38930 12012.m07678 protein transferase, transferring glycosyl groups, putative, expressed no original description 	T
26.2	misc.UDP glucosyl and glucoronyl transferases	MDP0000132958	 moderately similar to ( 395) AT2G38150 | Symbols:  | transferase/ transferase, transferring glycosyl groups | chr2:15981700-15982917 REVERSEmoderately similar to ( 339) loc_os04g44740 12004.m09431 protein transferase/ transferase, transferring glycosyl groups, putative, expressed  no original description 	T
34.19.1	transport.Major Intrinsic Proteins.PIP	MDP0000196802	 weakly similar to ( 161) AT4G35100 | Symbols: PIP3, PIP3A, PIP2;7, SIMIP | PIP3 (PLASMA MEMBRANE INTRINSIC PROTEIN 3); water channel | chr4:16708672-16709958 FORWARDweakly similar to ( 159) PIP21_ORYSA Probable aquaporin PIP2.1 (Plasma membrane intrinsic protein 2a) (PIP2a) (OsPIP2.1) - Oryza sativa (Rice)moderately similar to ( 222) loc_os09g36930 12009.m22027 protein aquaporin PIP2.7, putative, expressed  no original description 	T
27.3.22	RNA.regulation of transcription.HB,Homeobox transcription factor family	MDP0000206752	 weakly similar to ( 125) AT3G01470 | Symbols: ATHB-1, ATHB1, HD-ZIP-1, HAT5 | ATHB-1 (ARABIDOPSIS THALIANA HOMEOBOX 1); DNA binding / protein homodimerization/ sequence-specific DNA binding / transcription activator/ transcription factor | chr3:182648-184034 REVERSEweakly similar to ( 116) loc_os02g49700 12002.m33448 protein homeodomain leucine zipper protein CPHB-5, putative, expressed   no original description 	T
27.4	RNA.RNA binding	MDP0000242810	 moderately similar to ( 220) AT1G51510 | Symbols: Y14 | Y14; RNA binding / protein binding | chr1:19103072-19104753 REVERSEvery weakly similar to (51.6) GRP1_DAUCA Glycine-rich RNA-binding protein - Daucus carota (Carrot)moderately similar to ( 214) loc_os05g04850 12005.m083613 protein RNA-binding protein 8A, putative, expressed  no original description 	T
29.2.4	protein.synthesis.elongation	MDP0000249658	 weakly similar to ( 161) AT3G49470 | Symbols: NACA2 | NACA2 (NASCENT POLYPEPTIDE-ASSOCIATED COMPLEX SUBUNIT ALPHA-LIKE PROTEIN 2) | chr3:18341072-18342273 FORWARDweakly similar to ( 162) loc_os03g02960 12003.m05834 protein nascent polypeptide-associated complex alpha subunit-like protein, putative, expressed   no original description 	T
35.2	not assigned.unknown	MDP0000701887	 very weakly similar to (75.1) AT3G49190 | Symbols:  | condensation domain-containing protein | chr3:18231988-18234376 REVERSEvery weakly similar to (65.9) loc_os01g56370 12001.m11793 protein expressed protein no original description 	T
29.2.1.1.1.1.10	protein.synthesis.ribosomal protein.prokaryotic.chloroplast.30S subunit.S10	MDP0000646401	 moderately similar to ( 223) AT3G13120 | Symbols:  | 30S ribosomal protein S10, chloroplast, putative | chr3:4220310-4221526 REVERSEmoderately similar to ( 236) RR10_MESCR 30S ribosomal protein S10, chloroplast precursor - Mesembryanthemum crystallinum (Common ice plant)moderately similar to ( 201) loc_os03g10060 12003.m06460 protein chloroplast 30S ribosomal protein S10, putative, expressed no original description 	T
35.2	not assigned.unknown	MDP0000214325	 moderately similar to ( 238) AT2G22125 | Symbols:  | binding | chr2:9406793-9414223 FORWARDmoderately similar to ( 221) loc_os06g11990 12006.m05916 protein armadillo/beta-catenin-like repeat family protein, expressed no original description 	T
18.7	Co-factor and vitamine metabolism.iron-sulphur clusters	MDP0000778166	 moderately similar to ( 247) AT4G22220 | Symbols: ISU1, ATISU1 | ISU1; structural molecule | chr4:11759444-11760881 REVERSEmoderately similar to ( 253) loc_os01g47340 12001.m42683 protein nifU-like N-terminal domain-containing protein, mitochondrial precursor, putative, expressed no original description 	T
9.1.1	mitochondrial electron transport / ATP synthesis.NADH-DH.complex I	MDP0000346226	 very weakly similar to (95.5) AT1G14450 | Symbols:  | unknown protein | chr1:4947337-4947558 REVERSEvery weakly similar to (94.7) loc_os02g35610 12002.m08649 protein expressed protein  no original description 	T
35.1.5	not assigned.no ontology.pentatricopeptide (PPR) repeat-containing protein	MDP0000928190	 highly similar to ( 775) AT5G08310 | Symbols:  | pentatricopeptide (PPR) repeat-containing protein | chr5:2670134-2675254 REVERSEmoderately similar to ( 202) RF1_ORYSA Rf1 protein, mitochondrial precursor (PPR protein) (Fertility restorer) (Restorer for CMS) - Oryza sativa (Rice)highly similar to ( 603) loc_os05g24930 12005.m06787 protein expressed protein  no original description 	T
29.5.5	protein.degradation.serine protease	MDP0000645731	 moderately similar to ( 441) AT1G47710 | Symbols:  | serpin, putative / serine protease inhibitor, putative | chr1:17558271-17560061 FORWARDmoderately similar to ( 340) PRTZ_HORVU Protein Z (Z4) (Major endosperm albumin) - Hordeum vulgare (Barley)moderately similar to ( 381) loc_os03g41438 12003.m09209 protein protein Z, putative, expressed   no original description 	T
33.99	development.unspecified	MDP0000303216	 moderately similar to ( 388) AT3G26640 | Symbols: LWD2 | LWD2 (LIGHT-REGULATED WD 2); nucleotide binding | chr3:9793276-9794316 FORWARDmoderately similar to ( 342) loc_os02g32430 12002.m08336 protein WD40 repeat protein, putative, expressed  no original description 	T
28.1	DNA.synthesis/chromatin structure	MDP0000297368	 nearly identical (2255) AT2G38770 | Symbols: EMB2765 | EMB2765 (EMBRYO DEFECTIVE 2765) | chr2:16203185-16210253 REVERSEnearly identical (2190) loc_os03g26960 12003.m08027 protein aquarius, putative, expressed   no original description 	T
34.1	transport.p- and v-ATPases	MDP0000378695	 moderately similar to ( 367) AT5G44240 | Symbols:  | haloacid dehalogenase-like hydrolase family protein | chr5:17817619-17823598 FORWARDvery weakly similar to (55.1) ACA1_ORYSA Calcium-transporting ATPase 1, plasma membrane-type (EC 3.6.3.8) (Ca(2+)-ATPase isoform 1) (Plastid envelope ATPase 1) - Oryza sativa (Rice)moderately similar to ( 335) loc_os11g25980 12011.m06519 protein phospholipid-transporting ATPase 2, putative, expressed  no original description 	T
34.12	transport.metal	MDP0000488686	 weakly similar to ( 146) AT5G59030 | Symbols: COPT1 | COPT1 (copper transporter 1); copper ion transmembrane transporter | chr5:23833945-23834457 REVERSEweakly similar to ( 117) loc_os01g56420 12001.m11798 protein copper transporter 1, putative, expressed  no original description 	T
28.99	DNA.unspecified	MDP0000295191	 weakly similar to ( 111) loc_os12g40530 12012.m07837 protein transposon protein, putative, Mutator sub-class, expressed no original description 	T
29.4	protein.postranslational modification	MDP0000717013	 highly similar to ( 724) AT3G44850 | Symbols:  | protein kinase-related | chr3:16374617-16376931 REVERSEvery weakly similar to (62.0) CDC2_MAIZE Cell division control protein 2 homolog (EC 2.7.11.22) (EC 2.7.11.23) (p34cdc2) - Zea mays (Maize)highly similar to ( 685) loc_os03g53720 12003.m10342 protein ATP binding protein, putative, expressed  no original description 	T
28.1.3	DNA.synthesis/chromatin structure.histone	MDP0000192236	 weakly similar to ( 163) AT5G02560 | Symbols: HTA12 | HTA12; DNA binding | chr5:575437-576456 FORWARDweakly similar to ( 171) H2A1_MEDTR Probable histone H2A.1 - Medicago truncatula (Barrel medic)weakly similar to ( 166) loc_os03g17100 12003.m07141 protein histone H2A, putative, expressed  no original description 	T
30.5	signalling.G-proteins	MDP0000120931	 moderately similar to ( 362) AT4G28950 | Symbols: ARAC7, ATROP9, ATRAC7, RAC7, ROP9 | ROP9 (RHO-RELATED PROTEIN FROM PLANTS 9); GTP binding | chr4:14278289-14279705 FORWARDmoderately similar to ( 342) RAC2_ORYSA Rac-like GTP-binding protein 2 (OsRac2) - Oryza sativa (Rice)moderately similar to ( 342) loc_os05g43820 12005.m27625 protein rac-like GTP-binding protein 2, putative, expressed no original description 	T
35.1.5	not assigned.no ontology.pentatricopeptide (PPR) repeat-containing protein	MDP0000194307	 highly similar to ( 899) AT1G25360 | Symbols:  | pentatricopeptide (PPR) repeat-containing protein | chr1:8894428-8896800 FORWARDhighly similar to ( 630) MSK1_TOBAC Shaggy-related protein kinase NtK-1 (EC 2.7.11.1) - Nicotiana tabacum (Common tobacco)highly similar to ( 786) loc_os07g39910 12007.m08233 protein pentatricopeptide repeat protein PPR1106-17, putative   no original description 	T
35.2	not assigned.unknown	MDP0000883284	    no original description 	T
20.2.99	stress.abiotic.unspecified	MDP0000530457	 moderately similar to ( 300) AT3G62020 | Symbols: GLP10 | GLP10 (GERMIN-LIKE PROTEIN 10); manganese ion binding / nutrient reservoir | chr3:22971443-22972192 REVERSEmoderately similar to ( 297) RHRE_PEA Rhicadhesin receptor precursor (Germin-like protein) - Pisum sativum (Garden pea)moderately similar to ( 303) loc_os05g19670 12005.m06370 protein rhicadhesin receptor precursor, putative, expressed  no original description 	T
35.2	not assigned.unknown	MDP0000150310	 weakly similar to ( 173) AT2G25737 | Symbols:  | unknown protein | chr2:10977174-10979677 FORWARDweakly similar to ( 157) loc_os09g17600 12009.m22230 protein expressed protein  no original description 	T
29.2.1.2.2.82	protein.synthesis.ribosomal protein.eukaryotic.60S subunit.P2	MDP0000323561	 weakly similar to ( 102) AT2G27720 | Symbols:  | 60S acidic ribosomal protein P2 (RPP2A) | chr2:11818696-11819370 FORWARDvery weakly similar to (94.7) RLA2B_MAIZE 60S acidic ribosomal protein P2B - Zea mays (Maize)very weakly similar to (96.7) loc_os05g37330 12005.m07913 protein 60S acidic ribosomal protein P2B, putative, expressed no original description 	T
35.1.21	not assigned.no ontology.epsin N-terminal homology (ENTH) domain-containing protein	MDP0000159850	 highly similar to ( 525) AT5G11710 | Symbols:  | epsin N-terminal homology (ENTH) domain-containing protein / clathrin assembly protein-related | chr5:3772981-3776316 FORWARDmoderately similar to ( 418) loc_os04g51120 12004.m35441 protein af10-protein, putative, expressed no original description 	T
33.99	development.unspecified	MDP0000174358	 moderately similar to ( 447) AT5G13570 | Symbols: DCP2, TDT, ATDCP2 | DCP2 (DECAPPING 2); hydrolase/ m7G(5')pppN diphosphatase/ mRNA binding / protein homodimerization | chr5:4367532-4369992 FORWARDmoderately similar to ( 404) loc_os02g56210 12002.m33973 protein mRNA decapping enzyme 2, putative, expressed no original description 	T
27.1	RNA.processing	MDP0000174358	 moderately similar to ( 447) AT5G13570 | Symbols: DCP2, TDT, ATDCP2 | DCP2 (DECAPPING 2); hydrolase/ m7G(5')pppN diphosphatase/ mRNA binding / protein homodimerization | chr5:4367532-4369992 FORWARDmoderately similar to ( 404) loc_os02g56210 12002.m33973 protein mRNA decapping enzyme 2, putative, expressed no original description 	T
11.9.3.4	lipid metabolism.lipid degradation.lysophospholipases.phospholipase A2	MDP0000249250	 weakly similar to ( 147) AT4G29460 | Symbols:  | phospholipase A2 gamma, secretory low molecular weight | chr4:14483066-14483930 REVERSEweakly similar to ( 153) loc_os02g58500 12002.m33321 protein phospholipase A2, putative, expressed no original description 	T
29.3.1	protein.targeting.nucleus	MDP0000681634	 nearly identical (1154) AT3G08947 | Symbols:  | binding / protein transporter | chr3:2724007-2726722 FORWARDnearly identical (1064) loc_os05g28510 12005.m083698 protein importin beta-1 subunit, putative, expressed  no original description 	T
31.1	cell.organisation	MDP0000331563	 moderately similar to ( 336) AT3G55000 | Symbols: TON1A, TON1 | TON1A (TONNEAU 1) | chr3:20381612-20383577 FORWARDmoderately similar to ( 335) loc_os12g01170 12012.m04121 protein tonneau 1b, putative, expressed  no original description 	T
29.4	protein.postranslational modification	MDP0000173897	 highly similar to ( 519) AT3G48750 | Symbols: CDKA;1, CDC2AAT, CDK2, CDC2, CDC2A, CDKA1 | CDC2 (CELL DIVISION CONTROL 2); cyclin-dependent protein kinase/ kinase/ protein binding / protein kinase | chr3:18072238-18074296 FORWARDhighly similar to ( 553) CDC2A_ANTMA Cell division control protein 2 homolog A (EC 2.7.11.22) (EC 2.7.11.23) - Antirrhinum majus (Garden snapdragon)highly similar to ( 516) loc_os03g02680 12003.m101097 protein cell division control protein 2 homolog 1, putative, expressed  no original description 	T
1.1.1.2	PS.lightreaction.photosystem II.PSII polypeptide subunits	MDP0000203627	 moderately similar to ( 242) AT2G39470 | Symbols: PPL2 | PPL2 (PsbP-like protein 2); calcium ion binding | chr2:16476335-16477402 FORWARDmoderately similar to ( 223) loc_os03g17174 12003.m101683 protein calcium ion binding protein, putative, expressed no original description 	T
35.2	not assigned.unknown	MDP0000217227	 highly similar to ( 568) AT5G26740 | Symbols:  | unknown protein | chr5:9292436-9294407 FORWARDmoderately similar to ( 438) loc_os03g61210 12003.m11005 protein expressed protein  no original description 	T
20.1	stress.biotic	MDP0000261585	 very weakly similar to (75.1) AT2G02100 | Symbols: LCR69, PDF2.2 | LCR69 (LOW-MOLECULAR-WEIGHT CYSTEINE-RICH 69); peptidase inhibitor | chr2:528397-528885 FORWARDvery weakly similar to (77.8) THGF_HELAN Flower-specific gamma-thionin precursor (Defensin SD2) - Helianthus annuus (Common sunflower)very weakly similar to (72.4) loc_os03g03810 12003.m05913 protein flower-specific gamma-thionin precursor, putative, expressed no original description 	T
26.7	misc.oxidases - copper, flavone etc	MDP0000239909	 highly similar to ( 526) AT1G62600 | Symbols:  | flavin-containing monooxygenase family protein / FMO family protein | chr1:23179542-23181411 FORWARDmoderately similar to ( 490) loc_os10g40570 12010.m06824 protein disulfide oxidoreductase/ monooxygenase, putative, expressed   no original description 	T
20.2.99	stress.abiotic.unspecified	MDP0000287357	 weakly similar to ( 139) AT2G34700 | Symbols:  | pollen Ole e 1 allergen and extensin family protein | chr2:14634298-14635597 REVERSEvery weakly similar to (94.0) PEXLP_TOBAC Pistil-specific extensin-like protein precursor (PELP) - Nicotiana tabacum (Common tobacco)very weakly similar to (69.7) loc_os05g45460 12005.m08671 protein pistil-specific extensin-like protein precursor, putative, expressed   no original description 	T
35.2	not assigned.unknown	MDP0000261986	 weakly similar to ( 128) AT5G02160 | Symbols:  | unknown protein | chr5:426392-427024 FORWARDweakly similar to ( 117) loc_os05g01950 12005.m04731 protein expressed protein no original description 	T
35.1	not assigned.no ontology	MDP0000697676	 weakly similar to ( 183) AT5G02230 | Symbols:  | haloacid dehalogenase-like hydrolase family protein | chr5:449132-450507 FORWARDweakly similar to ( 182) loc_os03g16670 12003.m35158 protein catalytic/ hydrolase, putative, expressed no original description 	T
35.2	not assigned.unknown	MDP0000327019	  no original description 	T
26.21	misc.protease inhibitor/seed storage/lipid transfer protein (LTP) family protein	MDP0000251966	 weakly similar to ( 106) AT3G18280 | Symbols:  | protease inhibitor/seed storage/lipid transfer protein (LTP) family protein | chr3:6267102-6267392 FORWARDvery weakly similar to ( 100) NLTP_VIGUN Probable nonspecific lipid-transfer protein AKCS9 precursor (LTP) - Vigna unguiculata (Cowpea)very weakly similar to (86.3) loc_os01g49640 12001.m11151 protein nonspecific lipid-transfer protein precursor, putative, expressed  no original description 	T
20.2.1	stress.abiotic.heat	MDP0000299415	 nearly identical (1264) AT1G79920 | Symbols:  | ATP binding | chr1:30058935-30062224 REVERSEmoderately similar to ( 264) HSP7C_PETHY Heat shock cognate 70 kDa protein - Petunia hybrida (Petunia)nearly identical (1165) loc_os01g08560 12001.m150497 protein heat shock 70 kDa protein 4, putative, expressed  no original description 	T
29.2.1.2.1.19	protein.synthesis.ribosomal protein.eukaryotic.40S subunit.S19	MDP0000274063	 moderately similar to ( 261) AT5G61170 | Symbols:  | 40S ribosomal protein S19 (RPS19C) | chr5:24611158-24612202 FORWARDmoderately similar to ( 245) RS19_ORYSA 40S ribosomal protein S19 - Oryza sativa (Rice)moderately similar to ( 245) loc_os03g31090 12003.m08361 protein 40S ribosomal protein S19, putative, expressed  no original description 	T
27.4	RNA.RNA binding	MDP0000205980	 very weakly similar to (99.8) AT3G46020 | Symbols:  | RNA-binding protein, putative | chr3:16912511-16913250 REVERSEvery weakly similar to (67.8) GRP1_DAUCA Glycine-rich RNA-binding protein - Daucus carota (Carrot)very weakly similar to (77.0) loc_os04g49440 12004.m09878 protein RNA binding protein, putative, expressed no original description 	T
29.2.1.99.2.23	protein.synthesis.ribosomal protein.unknown.large subunit.L23	MDP0000741562	 moderately similar to ( 204) AT4G39880 | Symbols:  | ribosomal protein L23 family protein | chr4:18504601-18505137 FORWARDweakly similar to ( 163) loc_os07g23244 12007.m06606 protein RNA binding protein, putative, expressed no original description 	T
33.99	development.unspecified	MDP0000131808	 very weakly similar to (99.8) AT1G75500 | Symbols:  | nodulin MtN21 family protein | chr1:28338282-28340091 REVERSEweakly similar to ( 151) loc_os03g05530 12003.m06076 protein nodulin-like protein, putative, expressed  no original description 	T
35.2	not assigned.unknown	MDP0000220754	 moderately similar to ( 496) AT4G36440 | Symbols:  | unknown protein | chr4:17207213-17209873 REVERSEmoderately similar to ( 429) loc_os01g15790 12001.m08177 protein expressed protein  no original description 	T
35.2	not assigned.unknown	MDP0000238949	 weakly similar to ( 198) AT3G07510 | Symbols:  | unknown protein | chr3:2393895-2394464 REVERSEweakly similar to ( 146) loc_os10g09930 12010.m04333 protein expressed protein   no original description 	T
30.3	signalling.calcium	MDP0000143754	 moderately similar to ( 409) AT1G19870 | Symbols: iqd32 | iqd32 (IQ-domain 32); calmodulin binding | chr1:6895400-6898539 REVERSEmoderately similar to ( 303) loc_os04g56740 12004.m35501 protein IQ calmodulin-binding motif family protein, expressed   no original description 	T
31.1	cell.organisation	MDP0000813111	 weakly similar to ( 153) AT4G19840 | Symbols: ATPP2-A1, ATPP2A-1, PP2-A1 | ATPP2-A1; carbohydrate binding | chr4:10774336-10775701 FORWARDweakly similar to ( 132) loc_os01g06500 12001.m07280 protein lectin-like protein, putative, expressedMSP1_C  no original description 	T
27.3.69	RNA.regulation of transcription.SET-domain transcriptional regulator family	MDP0000256841	 highly similar to ( 627) AT5G04940 | Symbols: SUVH1 | SUVH1 (SU(VAR)3-9 HOMOLOG 1); histone methyltransferase | chr5:1454616-1456628 REVERSEmoderately similar to ( 495) loc_os05g41172 12005.m083768 protein histone-lysine N-methyltransferase, H3 lysine-9 specific SUVH1, putative, expressed     no original description 	T
19.7	tetrapyrrole synthesis.uroporphyrinogen decarboxylase	MDP0000270629	 highly similar to ( 520) AT2G40490 | Symbols: HEME2 | HEME2; uroporphyrinogen decarboxylase | chr2:16912961-16914988 FORWARDhighly similar to ( 555) DCUP_TOBAC Uroporphyrinogen decarboxylase, chloroplast precursor (EC 4.1.1.37) (URO-D) (UPD) - Nicotiana tabacum (Common tobacco)highly similar to ( 506) loc_os03g21900 12003.m07594 protein uroporphyrinogen decarboxylase, putative, expressed  no original description 	T
33.99	development.unspecified	MDP0000785966	 very weakly similar to (80.9) AT5G47060 | Symbols:  | senescence-associated protein-related | chr5:19116843-19117639 FORWARDvery weakly similar to (73.9) loc_os04g49660 12004.m09899 protein expressed proteinMSP1_C  no original description 	T
35.1	not assigned.no ontology	MDP0000124204	 weakly similar to ( 150) AT4G14420 | Symbols:  | lesion inducing protein-related | chr4:8302171-8303738 REVERSEweakly similar to ( 147) loc_os03g05520 12003.m06075 protein nicotiana lesion-inducing like, putative, expressed no original description 	T
30.2.11	signalling.receptor kinases.leucine rich repeat XI	MDP0000580410	 moderately similar to ( 378) AT1G72300 | Symbols:  | leucine-rich repeat transmembrane protein kinase, putative | chr1:27217679-27220966 REVERSEmoderately similar to ( 267) PSKR_DAUCA Phytosulfokine receptor precursor (EC 2.7.11.1) (Phytosulfokine LRR receptor kinase) - Daucus carota (Carrot)moderately similar to ( 335) loc_os02g05930 12002.m05941 protein phytosulfokine receptor precursor, putative, expressed   no original description 	T
30.2.11	signalling.receptor kinases.leucine rich repeat XI	MDP0000250234	 moderately similar to ( 453) AT1G45616 | Symbols: AtRLP6 | AtRLP6 (Receptor Like Protein 6); protein binding | chr1:17183550-17186534 REVERSEweakly similar to ( 197) RPK1_IPONI Receptor-like protein kinase precursor (EC 2.7.11.1) - Ipomoea nil (Japanese morning glory) (Pharbitis nil)highly similar to ( 535) loc_os04g40440 12004.m09026 protein expressed protein   no original description 	T
33.99	development.unspecified	MDP0000186401	 weakly similar to ( 200) AT5G62200 | Symbols:  | embryo-specific protein-related | chr5:24984463-24985674 REVERSEweakly similar to ( 135) loc_os01g07780 12001.m07405 protein lipase/lipooxygenase, PLAT/LH2, putative, expressed  no original description 	T
26.7	misc.oxidases - copper, flavone etc	MDP0000290042	 moderately similar to ( 488) AT5G61510 | Symbols:  | NADP-dependent oxidoreductase, putative | chr5:24737084-24738975 REVERSEvery weakly similar to (60.1) QORH_SPIOL Chloroplastic quinone-oxidoreductase homolog (EC 1.-.-.-) (ceQORH) - Spinacia oleracea (Spinach)moderately similar to ( 442) loc_os03g01190 12003.m05670 protein quinone oxidoreductase, putative, expressed   no original description 	T
26.21	misc.protease inhibitor/seed storage/lipid transfer protein (LTP) family protein	MDP0000125336	 weakly similar to ( 151) AT1G55260 | Symbols:  | lipid binding | chr1:20614663-20616158 FORWARDvery weakly similar to (50.1) NLTP3_ORYSA Nonspecific lipid-transfer protein 3 precursor (LTP 3) - Oryza sativa (Rice)weakly similar to ( 133) loc_os03g07100 12003.m06227 protein lipid transfer protein, putative, expressed no original description 	T
30.5	signalling.G-proteins	MDP0000304114	 moderately similar to ( 367) AT4G18800 | Symbols: ATHSGBP, ATRAB11B, ATRABA1D | ATRABA1D (ARABIDOPSIS RAB GTPASE HOMOLOG A1D); GTP binding | chr4:10320156-10321339 REVERSEmoderately similar to ( 374) RIC2_ORYSA Ras-related protein RIC2 - Oryza sativa (Rice)moderately similar to ( 374) loc_os06g35814 12006.m08118 protein ras-related protein RIC2, putative, expressed no original description 	T
27.3.10	RNA.regulation of transcription.C2C2(Zn) YABBY family	MDP0000255415	 moderately similar to ( 238) AT2G26580 | Symbols: YAB5 | YAB5 (YABBY5); transcription factor | chr2:11303923-11306739 REVERSEweakly similar to ( 151) YAB1_ORYSA Protein YABBY (OsYAB1) (Filamentous flower protein 1) - Oryza sativa (Rice)weakly similar to ( 169) loc_os03g44710 12003.m101420 protein axial regulator YABBY2, putative, expressed  no original description 	T
29.2.4	protein.synthesis.elongation	MDP0000189047	 moderately similar to ( 212) AT5G19510 | Symbols:  | elongation factor 1B alpha-subunit 2 (eEF1Balpha2) | chr5:6581854-6583137 REVERSEmoderately similar to ( 203) EF1B_ORYSA Elongation factor 1-beta (EF-1-beta) (Elongation factor 1B-alpha 2) (eEF-1B alpha) (Elongation factor 1-beta') (EF-1-beta') - Oryza sativa (Rice)moderately similar to ( 205) loc_os07g46750 12007.m08892 protein elongation factor 1-beta, putative, expressed  no original description 	T
35.2	not assigned.unknown	MDP0000361818	  no original description 	T
9.5	mitochondrial electron transport / ATP synthesis.cytochrome c reductase	MDP0000763693	 weakly similar to ( 122) AT3G10860 | Symbols:  | ubiquinol-cytochrome C reductase complex ubiquinone-binding protein, putative / ubiquinol-cytochrome C reductase complex 8.2 kDa protein, putative | chr3:3399815-3400514 FORWARDweakly similar to ( 126) UCRQ_SOLTU Ubiquinol-cytochrome c reductase complex ubiquinone-binding protein QP-C (EC 1.10.2.2) (Ubiquinol-cytochrome c reductase complex 8.2 kDa protein) - Solanum tuberosum (Potato)weakly similar to ( 129) loc_os06g07969 12006.m05519 protein ubiquinol-cytochrome c reductase complex ubiquinone-binding proteinQP-C, putative, expressed no original description 	T
29.5.11.4.3.2	protein.degradation.ubiquitin.E3.SCF.FBOX	MDP0000252833	 highly similar to ( 517) AT1G21760 | Symbols: ATFBP7, FBP7 | FBP7 (F-BOX PROTEIN 7) | chr1:7649324-7651613 FORWARDmoderately similar to ( 401) loc_os04g35190 12004.m08609 protein F-box only protein 9, putative, expressed  no original description 	T
26.3	misc.gluco-, galacto- and mannosidases	MDP0000187687	 highly similar to ( 780) AT3G06510 | Symbols: SFR2 | SFR2 (SENSITIVE TO FREEZING 2); beta-glucosidase/ hydrolase, hydrolyzing O-glycosyl compounds | chr3:2016450-2019533 FORWARDweakly similar to ( 119) BGLC_MAIZE Beta-glucosidase, chloroplast precursor (EC 3.2.1.21) (Gentiobiase) (Cellobiase) (Beta-D-glucoside glucohydrolase) - Zea mays (Maize)highly similar to ( 820) loc_os11g45710 12011.m080066 protein SFR2, putative, expressed   no original description 	T
35.1.5	not assigned.no ontology.pentatricopeptide (PPR) repeat-containing protein	MDP0000850418	 highly similar to ( 838) AT4G14050 | Symbols:  | pentatricopeptide (PPR) repeat-containing protein | chr4:8103645-8105483 REVERSEvery weakly similar to (82.0) RF1_ORYSA Rf1 protein, mitochondrial precursor (PPR protein) (Fertility restorer) (Restorer for CMS) - Oryza sativa (Rice)highly similar to ( 683) loc_os11g43934 12011.m08228 protein pentatricopeptide repeat protein PPR868-14, putative, expressed  no original description 	T
26.10	misc.cytochrome P450	MDP0000166476	 highly similar to ( 812) AT2G45970 | Symbols: CYP86A8, LCR | CYP86A8; fatty acid (omega-1)-hydroxylase/ oxygen binding | chr2:18912548-18914161 REVERSEweakly similar to ( 103) F3PH_PETHY Flavonoid 3'-monooxygenase (EC 1.14.13.21) (Flavonoid 3'-hydroxylase) (Cytochrome P450 75B2) - Petunia hybrida (Petunia)highly similar to ( 686) loc_os04g47250 12004.m09671 protein cytochrome P450 86A2, putative, expressed    no original description 	T
27.3.22	RNA.regulation of transcription.HB,Homeobox transcription factor family	MDP0000546502	 highly similar to ( 502) AT2G35940 | Symbols: BLH1 | BLH1 (BEL1-LIKE HOMEODOMAIN 1); DNA binding / protein heterodimerization/ protein homodimerization/ transcription factor | chr2:15089171-15091699 REVERSEvery weakly similar to (60.8) KNAP1_MALDO Homeobox protein knotted-1-like 1 (KNAP1) - Malus domestica (Apple) (Malus sylvestris)moderately similar to ( 340) loc_os11g06020 12011.m04798 protein BEL1-like homeodomain transcription factor, putative, expressed   no original description 	T
35.2	not assigned.unknown	MDP0000268725	 weakly similar to ( 108) AT4G13530 | Symbols:  | unknown protein | chr4:7866808-7868555 FORWARDvery weakly similar to (59.3) loc_os02g35220 12002.m08610 protein expressed protein TrbL  no original description 	T
34.99	transport.misc	MDP0000208962	 moderately similar to ( 448) AT1G61250 | Symbols: SC3 | SC3 (SECRETORY CARRIER 3); transmembrane transporter | chr1:22586035-22588664 FORWARDmoderately similar to ( 412) loc_os01g57220 12001.m43501 protein SC3 protein, putative, expressed no original description 	T
21.1	redox.thioredoxin	MDP0000279178	 moderately similar to ( 235) AT4G29670 | Symbols: ACHT2 | thioredoxin family protein | chr4:14535983-14537108 REVERSEvery weakly similar to (59.3) TRXH_WHEAT Thioredoxin H-type (TRX-H) (TrxTa) - Triticum aestivum (Wheat)moderately similar to ( 238) loc_os05g11090 12005.m27738 protein thioredoxin-like 6, chloroplast precursor, putative, expressed no original description 	T
26.28	misc.GDSL-motif lipase	MDP0000296512	 moderately similar to ( 355) AT5G55050 | Symbols:  | GDSL-motif lipase/hydrolase family protein | chr5:22337745-22339741 FORWARDweakly similar to ( 181) APG_BRANA Anter-specific proline-rich protein APG (Protein CEX) (Fragment) - Brassica napus (Rape)moderately similar to ( 266) loc_os03g38470 12003.m08940 protein anther-specific proline-rich protein APG precursor, putative, expressed no original description 	T
34.7	transport.phosphate	MDP0000145529	 highly similar to ( 698) AT2G29650 | Symbols: ANTR1 | PHT4;1; carbohydrate transmembrane transporter/ inorganic diphosphate transmembrane transporter/ inorganic phosphate transmembrane transporter/ organic anion transmembrane transporter/ sugar:hydrogen symporter | chr2:12673685-12675722 REVERSEhighly similar to ( 684) loc_os01g17240 12001.m08317 protein sialin, putative, expressed   no original description 	T
26.10	misc.cytochrome P450	MDP0000835140	 highly similar to ( 584) AT3G56630 | Symbols: CYP94D2 | CYP94D2; electron carrier/ heme binding / iron ion binding / monooxygenase/ oxygen binding | chr3:20978953-20980512 FORWARDvery weakly similar to (92.4) C97B2_SOYBN Cytochrome P450 97B2 (EC 1.14.-.-) - Glycine max (Soybean)highly similar to ( 513) loc_os03g12260 12003.m06676 protein cytochrome P450 86A2, putative, expressed  no original description 	T
35.2	not assigned.unknown	MDP0000149992	 moderately similar to ( 318) AT4G21890 | Symbols:  | unknown protein | chr4:11611874-11612674 REVERSEweakly similar to ( 153) loc_os07g29770 12007.m079679 protein MYND finger family protein, expressed  no original description 	T
35.1	not assigned.no ontology	MDP0000304659	 weakly similar to ( 158) AT5G40300 | Symbols:  | integral membrane protein, putative | chr5:16110778-16112701 FORWARDweakly similar to ( 106) loc_os02g04180 12002.m05767 protein UL36 tegument protein, putative, expressed tolA  no original description 	T
35.2	not assigned.unknown	MDP0000167338	 moderately similar to ( 279) AT5G11000 | Symbols:  | unknown protein | chr5:3479166-3480335 REVERSEweakly similar to ( 171) loc_os03g59200 12003.m10820 protein expressed protein TrbL  no original description 	T
30.5	signalling.G-proteins	MDP0000690963	 very weakly similar to (50.4) AT2G30060 | Symbols:  | Ran-binding protein 1b (RanBP1b) | chr2:12827035-12828551 FORWARD no original description 	T
29.5.11.20	protein.degradation.ubiquitin.proteasom	MDP0000145084	 moderately similar to ( 447) AT1G53850 | Symbols: PAE1 | PAE1; endopeptidase/ peptidase/ threonine-type endopeptidase | chr1:20104131-20105792 REVERSEmoderately similar to ( 452) PSA5_SOYBN Proteasome subunit alpha type 5 (EC 3.4.25.1) (20S proteasome alpha subunit E) (20S proteasome subunit alpha-5) - Glycine max (Soybean)moderately similar to ( 451) loc_os11g40140 12011.m28848 protein proteasome subunit alpha type 5, putative, expressed   no original description 	T
29.5	protein.degradation	MDP0000759062	 moderately similar to ( 224) AT2G24200 | Symbols:  | cytosol aminopeptidase | chr2:10287017-10289450 REVERSEmoderately similar to ( 237) AMPL2_ORYSA Leucine aminopeptidase 2, chloroplast precursor (EC 3.4.11.1) (LAP 2) (Leucyl aminopeptidase 2) (Proline aminopeptidase 2) (EC 3.4.11.5) (Prolyl aminopeptidase 2) - Oryza sativa (Rice)moderately similar to ( 237) loc_os02g55140 12002.m10540 protein leucine aminopeptidase 3, chloroplast precursor, putative, expressed no original description 	T
35.2	not assigned.unknown	MDP0000337362	 moderately similar to ( 372) AT5G26770 | Symbols:  | unknown protein | chr5:9407981-9409735 REVERSEmoderately similar to ( 352) loc_os05g04530 12005.m04986 protein plant IF-like protein, putative, expressed  no original description 	T
29.6	protein.folding	MDP0000366511	 weakly similar to ( 130) AT5G43260 | Symbols:  | chaperone protein dnaJ-related | chr5:17357693-17357986 REVERSEvery weakly similar to (67.4) loc_os02g30320 12002.m08175 protein drought-induced protein 1, putative, expressed  no original description 	T
35.2	not assigned.unknown	MDP0000930948	 moderately similar to ( 209) AT5G44650 | Symbols:  | unknown protein | chr5:18013396-18015292 FORWARDmoderately similar to ( 223) loc_os01g58470 12001.m11989 protein expressed protein  no original description 	T
15.1	metal handling.acquisition	MDP0000272115	 weakly similar to ( 187) AT5G50160 | Symbols: ATFRO8, FRO8 | FRO8 (FERRIC REDUCTION OXIDASE 8); ferric-chelate reductase/ oxidoreductase | chr5:20415832-20418582 FORWARDvery weakly similar to (72.4) loc_os04g36720 12004.m08712 protein ferric reductase-like transmembrane component, putative, expressed  no original description 	T
31.1	cell.organisation	MDP0000248806	 weakly similar to ( 189) AT3G26070 | Symbols:  | plastid-lipid associated protein PAP / fibrillin family protein | chr3:9526904-9528199 FORWARDvery weakly similar to (53.1) PG1_PEA Plastoglobulin-1, chloroplast precursor - Pisum sativum (Garden pea)moderately similar to ( 239) loc_os03g49720 12003.m09971 protein structural molecule, putative, expressed  no original description 	T
27.3.3	RNA.regulation of transcription.AP2/EREBP, APETALA2/Ethylene-responsive element binding protein family	MDP0000119956	 weakly similar to ( 127) AT1G68550 | Symbols:  | AP2 domain-containing transcription factor, putative | chr1:25725810-25726784 REVERSEvery weakly similar to (80.5) ERF1_ORYSA Ethylene-responsive transcription factor 1 (Ethylene-responsive element-binding factor 1) (EREBP-1) (OsEREBP1) - Oryza sativa (Rice)very weakly similar to (88.6) loc_os09g13940 12009.m60262 protein AP2 domain containing protein, expressed  no original description 	T
31.1	cell.organisation	MDP0000276397	 highly similar to ( 688) AT5G51600 | Symbols: PLE, ATMAP65-3, MAP65-3 | PLE (PLEIADE); microtubule binding | chr5:20961061-20964080 REVERSEhighly similar to ( 628) loc_os01g49200 12001.m11107 protein microtubule-associated protein, putative, expressed   no original description 	T
29.1.14	protein.aa activation.glycine-tRNA ligase	MDP0000146975	 nearly identical (1058) AT1G29880 | Symbols:  | glycyl-tRNA synthetase / glycine--tRNA ligase | chr1:10459662-10462781 REVERSEnearly identical (1007) loc_os04g32650 12004.m08363 protein glycyl-tRNA synthetase 1, mitochondrial precursor, putative, expressed  no original description 	T
20.2.99	stress.abiotic.unspecified	MDP0000698024	 very weakly similar to (55.1) AT1G20450 | Symbols: LTI29, LTI45, ERD10 | ERD10 (EARLY RESPONSIVE TO DEHYDRATION 10); actin binding | chr1:7088235-7089107 REVERSE  no original description 	T
30.2.11	signalling.receptor kinases.leucine rich repeat XI	MDP0000140341	 moderately similar to ( 338) AT3G17640 | Symbols:  | leucine-rich repeat family protein | chr3:6032393-6033583 FORWARDvery weakly similar to (98.2) PGIP1_ORYSA Polygalacturonase inhibitor 1 precursor (Polygalacturonase-inhibiting protein) (Floral organ regulator 1) - Oryza sativa (Rice)moderately similar to ( 232) loc_os06g28480 12006.m07392 protein polygalacturonase inhibitor 1 precursor, putative, expressed   no original description 	T
26.18	misc.invertase/pectin methylesterase inhibitor family protein	MDP0000679993	 weakly similar to ( 167) AT5G20740 | Symbols:  | invertase/pectin methylesterase inhibitor family protein | chr5:7025867-7026484 REVERSEweakly similar to ( 107) 21KD_DAUCA 21 kDa protein precursor (1.2 protein) - Daucus carota (Carrot)weakly similar to ( 104) loc_os06g49760 12006.m09500 protein ripening-related protein, putative, expressed  no original description 	T
29.3.4.99	protein.targeting.secretory pathway.unspecified	MDP0000189652	 moderately similar to ( 347) AT5G05670 | Symbols:  | signal recognition particle binding | chr5:1695916-1697534 REVERSEmoderately similar to ( 313) loc_os08g41250 12008.m26756 protein signal recognition particle receptor beta subunit, putative, expressed no original description 	T
28.2	DNA.repair	MDP0000264615	 moderately similar to ( 290) AT1G75090 | Symbols:  | methyladenine glycosylase family protein | chr1:28187647-28189612 REVERSEmoderately similar to ( 248) loc_os03g10220 12003.m06475 protein DNA-3-methyladenine glycosylase I, putative, expressed no original description 	T
34.3	transport.amino acids	MDP0000132209	 moderately similar to ( 377) AT1G47670 | Symbols:  | amino acid transporter family protein | chr1:17536834-17539486 REVERSEvery weakly similar to (50.4) LAX5_MEDTR Auxin transporter-like protein 5 (AUX1-like protein 5) (MtLAX5) - Medicago truncatula (Barrel medic)moderately similar to ( 390) loc_os04g47420 12004.m09687 protein expressed protein   no original description 	T
29.5.3	protein.degradation.cysteine protease	MDP0000943270	 very weakly similar to (85.9) AT5G47550 | Symbols:  | cysteine protease inhibitor, putative / cystatin, putative | chr5:19286596-19286964 REVERSEvery weakly similar to (57.4) CYT1_ORYSA Cysteine proteinase inhibitor 1 (Oryzacystatin-1) (Oryzacystatin I) - Oryza sativa (Rice)very weakly similar to (76.6) loc_os03g31510 12003.m08398 protein cystatin, putative, expressed no original description 	T
26.10	misc.cytochrome P450	MDP0000133811	 moderately similar to ( 463) AT4G31940 | Symbols: CYP82C4 | CYP82C4; electron carrier/ heme binding / iron ion binding / monooxygenase/ oxygen binding | chr4:15452040-15453966 FORWARDmoderately similar to ( 493) C82A4_SOYBN Cytochrome P450 82A4 (EC 1.14.-.-) (P450 CP9) - Glycine max (Soybean)moderately similar to ( 287) loc_os09g26940 12009.m05846 protein flavonoid 3-monooxygenase, putative, expressed   no original description 	T
15.2	metal handling.binding, chelation and storage	MDP0000222300	 weakly similar to ( 187) AT1G71050 | Symbols:  | heavy-metal-associated domain-containing protein / copper chaperone (CCH)-related | chr1:26803322-26803946 REVERSEweakly similar to ( 162) loc_os01g32330 12001.m09558 protein farnesylated protein 2, putative, expressed  no original description 	T
23.2	nucleotide metabolism.degradation	MDP0000305350	 moderately similar to ( 393) AT4G04880 | Symbols:  | adenosine/AMP deaminase family protein | chr4:2465055-2467103 REVERSEmoderately similar to ( 327) loc_os07g46630 12007.m08880 protein deaminase, putative, expressed no original description 	T
35.2	not assigned.unknown	MDP0000802715	 moderately similar to ( 351) AT2G18630 | Symbols:  | unknown protein | chr2:8081154-8082496 FORWARDmoderately similar to ( 350) loc_os03g10240 12003.m06477 protein expressed protein  no original description 	T
20.2.99	stress.abiotic.unspecified	MDP0000292853	 weakly similar to ( 169) AT5G15780 | Symbols:  | pollen Ole e 1 allergen and extensin family protein | chr5:5144898-5146297 REVERSEvery weakly similar to (61.2) loc_os10g13850 12010.m04638 protein expressed protein  no original description 	T
29.3.4.3	protein.targeting.secretory pathway.vacuole	MDP0000298868	 nearly identical (1025) AT2G14740 | Symbols: ATVSR3 | ATVSR3 (ARABIDOPSIS THALIANA VACULOLAR SORTING RECEPTOR 3); calcium ion binding | chr2:6308895-6312303 FORWARDnearly identical (1010) VSR1_PEA Vacuolar sorting receptor 1 precursor (BP-80) (80 kDa proaleurein-binding protein) - Pisum sativum (Garden pea)highly similar to ( 922) loc_os10g20630 12010.m05084 protein vacuolar sorting receptor 3 precursor, putative, expressed no original description 	T
35.1	not assigned.no ontology	MDP0000249923	 weakly similar to ( 162) AT5G06660 | Symbols:  | unknown protein | chr5:2046863-2047453 FORWARDweakly similar to ( 118) loc_os03g51160 12003.m101475 protein fb27, putative, expressed  no original description 	T
34.1	transport.p- and v-ATPases	MDP0000303799	 nearly identical (1333) AT1G17260 | Symbols: AHA10 | AHA10 (Autoinhibited H(+)-ATPase isoform 10); ATPase/ ATPase, coupled to transmembrane movement of ions, phosphorylative mechanism / cation-transporting ATPase | chr1:5904058-5908898 FORWARDnearly identical (1280) PMA4_NICPL Plasma membrane ATPase 4 (EC 3.6.3.6) (Proton pump 4) - Nicotiana plumbaginifolia (Leadwort-leaved tobacco)nearly identical (1282) loc_os03g08560 12003.m06364 protein ATPase 10, plasma membrane-type, putative, expressed  no original description 	T
30.2.24	signalling.receptor kinases.S-locus glycoprotein like	MDP0000174381	 moderately similar to ( 296) AT5G60900 | Symbols: RLK1 | RLK1 (RECEPTOR-LIKE PROTEIN KINASE 1); ATP binding / carbohydrate binding / kinase/ protein kinase/ protein serine/threonine kinase/ protein tyrosine kinase/ sugar binding | chr5:24498467-24501494 REVERSEweakly similar to ( 107) KPRO_MAIZE Putative receptor protein kinase ZmPK1 precursor (EC 2.7.11.1) - Zea mays (Maize)moderately similar to ( 306) loc_os04g12600 12004.m06503 protein receptor-like protein kinase, putative  no original description 	T
27.3.24	RNA.regulation of transcription.MADS box transcription factor family	MDP0000289836	 moderately similar to ( 259) AT5G60910 | Symbols: AGL8, FUL | AGL8 (agamous-like 8); transcription factor | chr5:24502736-24504934 REVERSEmoderately similar to ( 295) AGL8_SOLTU Agamous-like MADS-box protein AGL8 homolog (POTM1-1) - Solanum tuberosum (Potato)moderately similar to ( 251) loc_os03g54160 12003.m35583 protein MADS-box transcription factor 14, putative, expressed no original description 	T
34.99	transport.misc	MDP0000155390	 highly similar to ( 980) AT2G27810 | Symbols:  | xanthine/uracil permease family protein | chr2:11852338-11855411 FORWARDhighly similar to ( 641) loc_os07g30810 12007.m07349 protein permease, putative, expressed   no original description 	T
35.2	not assigned.unknown	MDP0000570730	 moderately similar to ( 431) AT2G46080 | Symbols:  | INVOLVED IN: biological_process unknown; EXPRESSED IN: 23 plant structures; EXPRESSED DURING: 13 growth stages; BEST Arabidopsis thaliana protein match is: BPS1 (BYPASS 1) (TAIR:AT1G01550.2); Has 94 Blast hits to 83 proteins in 14 species: Archae - 0; Bacteria - 2; Metazoa - 1; Fungi - 0; Plants - 91; Viruses - 0; Other Eukaryotes - 0 (source: NCBI BLink). | chr2:18948317-18949360 REVERSEmoderately similar to ( 263) loc_os10g36950 12010.m06496 protein expressed protein   no original description 	T
30.2.17	signalling.receptor kinases.DUF 26	MDP0000139111	 weakly similar to ( 187) AT5G38280 | Symbols: PR5K | PR5K; kinase/ transmembrane receptor protein serine/threonine kinase | chr5:15293325-15295838 REVERSEweakly similar to ( 144) KPRO_MAIZE Putative receptor protein kinase ZmPK1 precursor (EC 2.7.11.1) - Zea mays (Maize)moderately similar to ( 301) loc_os04g56130 12004.m10504 protein ATP binding protein, putative  no original description 	T
33.99	development.unspecified	MDP0000203930	 weakly similar to ( 150) AT4G17670 | Symbols:  | senescence-associated protein-related | chr4:9833948-9834663 REVERSEvery weakly similar to (95.5) loc_os04g49660 12004.m09899 protein expressed protein no original description 	T
35.2	not assigned.unknown	MDP0000204316	 weakly similar to ( 103) AT1G60870 | Symbols: MEE9 | MEE9 (maternal effect embryo arrest 9) | chr1:22409712-22410227 FORWARD   no original description 	T
29.5.1	protein.degradation.subtilases	MDP0000310854	 highly similar to ( 600) AT5G59190 | Symbols:  | subtilase family protein | chr5:23885855-23888673 FORWARDmoderately similar to ( 485) loc_os09g36110 12009.m06561 protein subtilisin-like protease precursor, putative, expressed   no original description 	T
10.7	cell wall.modification	MDP0000568045	 moderately similar to ( 322) AT4G38400 | Symbols: ATEXLA2, EXPL2, ATEXPL2, ATHEXP BETA 2.2 | ATEXLA2 (ARABIDOPSIS THALIANA EXPANSIN-LIKE A2) | chr4:17978675-17979665 REVERSEmoderately similar to ( 279) EXLA2_ORYSA Expansin-like A2 precursor (OsEXLA2) (OsEXPL2) (OsaEXPb2.2) - Oryza sativa (Rice)moderately similar to ( 279) loc_os10g39640 12010.m06734 protein expansin-like 3 precursor, putative, expressed   no original description 	T
27.3.35	RNA.regulation of transcription.bZIP transcription factor family	MDP0000521934	 weakly similar to ( 130) AT1G75390 | Symbols: AtbZIP44 | AtbZIP44 (Arabidopsis thaliana basic leucine-zipper 44); DNA binding / protein heterodimerization/ transcription factor | chr1:28292224-28292665 FORWARDvery weakly similar to (89.7) OCS1_MAIZE Ocs element-binding factor 1 (OCSBF-1) - Zea mays (Maize)weakly similar to ( 111) loc_os02g03960 12002.m05745 protein ocs element-binding factor 1, putative, expressed  no original description 	T
30.4.5	signalling.phosphinositides.inositol-1,3,4-trisphosphate 5/6-kinase	MDP0000151118	 moderately similar to ( 334) AT5G16760 | Symbols:  | inositol 1,3,4-trisphosphate 5/6-kinase | chr5:5509890-5510849 FORWARDmoderately similar to ( 346) ITPK1_MAIZE Inositol-tetrakisphosphate 1-kinase 1 (EC 2.7.1.134) (Inositol-triphosphate 5/6-kinase 1) (EC 2.7.1.159) (Inositol 1,3,4-trisphosphate 5/6-kinase 1) (Ins(1,3,4)P(3) 5/6-kinase 1) (ZmIpk) (Low phytic acid protein 2) - Zea mays (Maize)moderately similar to ( 347) loc_os10g42550 12010.m07008 protein inositol-tetrakisphosphate 1-kinase 1, putative, expressed   no original description 	T
35.1	not assigned.no ontology	MDP0000133157	 moderately similar to ( 441) AT1G76340 | Symbols:  | integral membrane family protein | chr1:28635188-28636306 REVERSEmoderately similar to ( 406) loc_os03g11590 12003.m101239 protein expressed protein no original description 	T
29.2.3	protein.synthesis.initiation	MDP0000283642	 moderately similar to ( 215) AT2G04520 | Symbols:  | eukaryotic translation initiation factor 1A, putative / eIF-1A, putative / eIF-4C, putative | chr2:1574802-1575239 REVERSEweakly similar to ( 197) IF1A_WHEAT Eukaryotic translation initiation factor 1A (EIF-1A) (EIF-4C) - Triticum aestivum (Wheat)weakly similar to ( 198) loc_os02g19770 12002.m07219 protein eukaryotic translation initiation factor 1A, putative, expressed no original description 	T
16.8.2	secondary metabolism.flavonoids.chalcones	MDP0000134791	 moderately similar to ( 274) AT5G05270 | Symbols:  | chalcone-flavanone isomerase family protein | chr5:1563543-1564827 FORWARDvery weakly similar to (75.9) CFI_VITVI Chalcone--flavonone isomerase (EC 5.5.1.6) (Chalcone isomerase) - Vitis vinifera (Grape)moderately similar to ( 258) loc_os11g02440 12011.m28712 protein chalcone--flavonone isomerase, putative, expressed  no original description 	T
35.1	not assigned.no ontology	MDP0000317819	 moderately similar to ( 243) AT5G01580 | Symbols: OSH1 | OSH1 (OAS HIGH ACCUMULATION 1); catalytic | chr5:222760-223852 REVERSEmoderately similar to ( 219) loc_os01g61240 12001.m12251 protein gamma-interferon-inducible lysosomal thiol reductase precursor, putative no original description 	T
30.3	signalling.calcium	MDP0000284488	 moderately similar to ( 272) AT4G10630 | Symbols:  | glutaredoxin family protein | chr4:6566625-6567629 REVERSEmoderately similar to ( 263) loc_os08g02250 12008.m04369 protein calmodulin binding protein, putative, expressed    Sporozoite_P67  no original description 	T
35.2	not assigned.unknown	MDP0000253807	 weakly similar to ( 169) AT2G38550 | Symbols:  | unknown protein | chr2:16132178-16134229 FORWARDweakly similar to ( 141) loc_os01g60650 12001.m12193 protein non-green plastid inner envelope membrane protein, putative, expressed no original description 	T
10.6.3	cell wall.degradation.pectate lyases and polygalacturonases	MDP0000276859	 highly similar to ( 688) AT3G53190 | Symbols:  | pectate lyase family protein | chr3:19714171-19717537 FORWARDmoderately similar to ( 393) PEL_LILLO Pectate lyase precursor (EC 4.2.2.2) - Lilium longiflorum (Trumpet lily)highly similar to ( 531) loc_os10g31910 12010.m06042 protein pectate lyase 12 precursor, putative, expressed  no original description 	T
29.4	protein.postranslational modification	MDP0000340068	 highly similar to ( 573) AT5G06750 | Symbols:  | protein phosphatase 2C family protein / PP2C family protein | chr5:2086403-2088245 REVERSEhighly similar to ( 558) loc_os02g46080 12002.m77836 protein catalytic/ protein phosphatase type 2C, putative, expressed no original description 	T
30.2.17	signalling.receptor kinases.DUF 26	MDP0000412092	 moderately similar to ( 325) AT1G66920 | Symbols:  | serine/threonine protein kinase, putative | chr1:24965410-24967432 REVERSEweakly similar to ( 182) KPRO_MAIZE Putative receptor protein kinase ZmPK1 precursor (EC 2.7.11.1) - Zea mays (Maize)moderately similar to ( 363) loc_os01g02400 12001.m06882 protein Ser/Thr receptor-like kinase, putative, expressed no original description 	T
35.1	not assigned.no ontology	MDP0000335758	 weakly similar to ( 152) AT3G12180 | Symbols:  | cornichon family protein | chr3:3883464-3884570 FORWARDweakly similar to ( 125) loc_os12g32180 12012.m26719 protein ER-derived vesicles protein ERV14, putative, expressed no original description 	T
1.1.3.1	PS.lightreaction.cytochrome b6/f.iron sulfur subunit	MDP0000319969	 moderately similar to ( 352) AT4G03280 | Symbols: PETC, PGR1 | PETC (PHOTOSYNTHETIC ELECTRON TRANSFER C); electron transporter, transferring electrons from cytochrome b6/f complex of photosystem II | chr4:1440462-1441717 FORWARDmoderately similar to ( 355) UCRIA_SPIOL Cytochrome b6-f complex iron-sulfur subunit, chloroplast precursor (EC 1.10.99.1) (Rieske iron-sulfur protein) (Plastohydroquinone:plastocyanin oxidoreductase iron-sulfur protein) (ISP) (RISP) - Spinacia oleracea (Spinach)moderately similar to ( 321) loc_os07g37030 12007.m07953 protein cytochrome b6-f complex iron-sulfur subunit, chloroplast precursor, putative, expressed  no original description 	T
21.2	redox.ascorbate and glutathione	MDP0000951881	 moderately similar to ( 203) AT5G48810 | Symbols: ATB5-B, B5 #3, ATCB5-D, CB5-D | CB5-D (CYTOCHROME B5 ISOFORM D); heme binding | chr5:19789249-19790180 REVERSEmoderately similar to ( 228) CYB5_TOBAC Cytochrome b5 - Nicotiana tabacum (Common tobacco)moderately similar to ( 204) loc_os05g01820 12005.m04718 protein cytochrome b5, putative, expressed  no original description 	T
10.2.1	cell wall.cellulose synthesis.cellulose synthase	MDP0000847392	 moderately similar to ( 311) AT5G05170 | Symbols: CESA3, IXR1, ATCESA3, ATH-B, CEV1 | CEV1 (CONSTITUTIVE EXPRESSION OF VSP 1); cellulose synthase/ transferase, transferring glycosyl groups | chr5:1530401-1535090 REVERSEmoderately similar to ( 311) loc_os07g10770 12007.m05532 protein CESA8 - cellulose synthase, expressed no original description 	T
27.4	RNA.RNA binding	MDP0000321050	 weakly similar to ( 161) AT4G26650 | Symbols:  | RNA recognition motif (RRM)-containing protein | chr4:13445265-13447592 FORWARDvery weakly similar to (83.2) ROC1_NICSY 29 kDa ribonucleoprotein A, chloroplast precursor (CP29A) - Nicotiana sylvestris (Wood tobacco)weakly similar to ( 164) loc_os07g39560 12007.m29242 protein RNA-binding protein, putative, expressed  no original description 	T
28.1.3.1	DNA.synthesis/chromatin structure.histone.H1	MDP0000141691	 weakly similar to ( 109) AT1G14900 | Symbols: HMGA | HMGA (HIGH MOBILITY GROUP A); DNA binding | chr1:5138665-5139353 REVERSEweakly similar to ( 137) HMGYA_SOYBN HMG-Y-related protein A (SB16A protein) - Glycine max (Soybean)weakly similar to ( 112) loc_os08g33190 12008.m07310 protein linker histone H1 and H5 family protein, expressed  no original description 	T
10.3	cell wall.hemicellulose synthesis	MDP0000282228	 highly similar to ( 911) AT4G21060 | Symbols:  | galactosyltransferase family protein | chr4:11242003-11244860 FORWARDhighly similar to ( 806) loc_os08g03670 12008.m26563 protein galactosyltransferase family, putative, expressed  no original description 	T
26.2	misc.UDP glucosyl and glucoronyl transferases	MDP0000282228	 highly similar to ( 911) AT4G21060 | Symbols:  | galactosyltransferase family protein | chr4:11242003-11244860 FORWARDhighly similar to ( 806) loc_os08g03670 12008.m26563 protein galactosyltransferase family, putative, expressed  no original description 	T
15.2	metal handling.binding, chelation and storage	MDP0000292099	 very weakly similar to (94.4) AT2G28660 | Symbols:  | copper-binding family protein | chr2:12295419-12296944 FORWARDweakly similar to ( 120) loc_os08g31340 12008.m07132 protein heavy metal-associated domain containing protein, expressed  no original description 	T
13.1.6.1.1	amino acid metabolism.synthesis.aromatic aa.chorismate.3-deoxy-D-arabino-heptulosonate 7-phosphate synthase	MDP0000749719	 highly similar to ( 834) AT1G22410 | Symbols:  | 2-dehydro-3-deoxyphosphoheptonate aldolase, putative / 3-deoxy-D-arabino-heptulosonate 7-phosphate synthase, putative / DAHP synthetase, putative | chr1:7912120-7914742 FORWARDhighly similar to ( 914) AROF_TOBAC Phospho-2-dehydro-3-deoxyheptonate aldolase 1, chloroplast precursor (EC 2.5.1.54) (Phospho-2-keto-3-deoxyheptonate aldolase 1) (DAHP synthetase 1) (3-deoxy-D-arabino-heptulosonate 7-phosphate synthase 1) - Nicotiana tabacum (Common tobhighly similar to ( 846) loc_os07g42960 12007.m29007 protein phospho-2-dehydro-3-deoxyheptonate aldolase 1, chloroplast precursor, putative, expressed   no original description 	T
27.3.99	RNA.regulation of transcription.unclassified	MDP0000265706	 highly similar to ( 570) AT1G69420 | Symbols:  | zinc finger (DHHC type) family protein | chr1:26093690-26096736 FORWARDmoderately similar to ( 478) loc_os03g11110 12003.m06562 protein metal ion binding protein, putative, expressed no original description 	T
29.5.1	protein.degradation.subtilases	MDP0000122512	 highly similar to ( 525) AT1G32940 | Symbols: ATSBT3.5, SBT3.5 | SBT3.5; identical protein binding / serine-type endopeptidase | chr1:11937634-11940856 FORWARDhighly similar to ( 566) loc_os09g36110 12009.m06561 protein subtilisin-like protease precursor, putative, expressed no original description 	T
21.1	redox.thioredoxin	MDP0000135570	 weakly similar to ( 149) AT5G51010 | Symbols:  | rubredoxin family protein | chr5:20744615-20745344 FORWARDweakly similar to ( 133) loc_os11g32500 12011.m07151 protein rubredoxin family protein, expressed  no original description 	T
9.5	mitochondrial electron transport / ATP synthesis.cytochrome c reductase	MDP0000226879	 nearly identical (1090) AT1G29880 | Symbols:  | glycyl-tRNA synthetase / glycine--tRNA ligase | chr1:10459662-10462781 REVERSEmoderately similar to ( 399) UCRI_SOLTU Ubiquinol-cytochrome c reductase iron-sulfur subunit, mitochondrial precursor (EC 1.10.2.2) (Rieske iron-sulfur protein) (RISP) - Solanum tuberosum (Potato)nearly identical (1031) loc_os08g42560 12008.m08234 protein glycyl-tRNA synthetase 1, mitochondrial precursor, putative, expressed PRK07003  no original description 	T
29.1.14	protein.aa activation.glycine-tRNA ligase	MDP0000226879	 nearly identical (1090) AT1G29880 | Symbols:  | glycyl-tRNA synthetase / glycine--tRNA ligase | chr1:10459662-10462781 REVERSEmoderately similar to ( 399) UCRI_SOLTU Ubiquinol-cytochrome c reductase iron-sulfur subunit, mitochondrial precursor (EC 1.10.2.2) (Rieske iron-sulfur protein) (RISP) - Solanum tuberosum (Potato)nearly identical (1031) loc_os08g42560 12008.m08234 protein glycyl-tRNA synthetase 1, mitochondrial precursor, putative, expressed PRK07003  no original description 	T
34.21	transport.calcium	MDP0000148472	 weakly similar to ( 122) AT4G03560 | Symbols: ATTPC1, TPC1, ATCCH1, FOU2 | ATTPC1 (TWO-PORE CHANNEL 1); calcium channel/ voltage-gated calcium channel | chr4:1580253-1585691 FORWARDweakly similar to ( 118) TPC1_ORYSA Probable voltage-dependent calcium channel protein TPC1 (Probable voltage-gated calcium channel) (Two-pore calcium channel protein) - Oryza sativa (Rice)weakly similar to ( 118) loc_os01g48680 12001.m42691 protein voltage-dependent calcium channel protein TPC1, putative, expressed no original description 	T
20.2	stress.abiotic	MDP0000700402	 moderately similar to ( 419) AT4G05440 | Symbols: EDA35 | EDA35 (embryo sac development arrest 35) | chr4:2757274-2758117 FORWARD no original description 	T
27.3.22	RNA.regulation of transcription.HB,Homeobox transcription factor family	MDP0000128214	 moderately similar to ( 477) AT2G35940 | Symbols: BLH1 | BLH1 (BEL1-LIKE HOMEODOMAIN 1); DNA binding / protein heterodimerization/ protein homodimerization/ transcription factor | chr2:15089171-15091699 REVERSEvery weakly similar to (60.1) KNAP1_MALDO Homeobox protein knotted-1-like 1 (KNAP1) - Malus domestica (Apple) (Malus sylvestris)moderately similar to ( 319) loc_os11g06020 12011.m04798 protein BEL1-like homeodomain transcription factor, putative, expressed   no original description 	T
29.2.1.2.1.18	protein.synthesis.ribosomal protein.eukaryotic.40S subunit.S18	MDP0000165757	 moderately similar to ( 272) AT4G09800 | Symbols: RPS18C | RPS18C (S18 RIBOSOMAL PROTEIN); RNA binding / nucleic acid binding / structural constituent of ribosome | chr4:6173818-6174963 FORWARDmoderately similar to ( 211) RS18_CHLRE 40S ribosomal protein S18 - Chlamydomonas reinhardtiimoderately similar to ( 256) loc_os07g07709 12007.m29370 protein 40S ribosomal protein S18, putative, expressed  no original description 	T
33.99	development.unspecified	MDP0000635733	 highly similar to ( 721) AT4G33270 | Symbols: CDC20.1 | CDC20.1; signal transducer | chr4:16044545-16046590 REVERSEvery weakly similar to (58.2) GBB_ORYSA Guanine nucleotide-binding protein subunit beta - Oryza sativa (Rice)highly similar to ( 623) loc_os02g47180 12002.m33826 protein cell division cycle protein 20, putative, expressed no original description 	T
10.2.2	cell wall.cellulose synthesis.COBRA	MDP0000094767	 highly similar to ( 770) AT5G60920 | Symbols: COB | COB (COBRA) | chr5:24511466-24513932 REVERSEhighly similar to ( 738) COBL3_ORYSA COBRA-like 3 protein precursor (BRITTLE CULM1-like 4 protein) - Oryza sativa (Rice)highly similar to ( 738) loc_os05g32110 12005.m083711 protein COBRA-like protein 4 precursor, putative, expressed no original description 	T
31.1	cell.organisation	MDP0000157043	 moderately similar to ( 422) AT5G67270 | Symbols: ATEB1C, EB1C, ATEB1H1 | ATEB1C (microtubule end binding protein 1); microtubule binding | chr5:26840248-26841984 REVERSEmoderately similar to ( 408) loc_os10g35580 12010.m06382 protein microtubule-associated protein RP/EB family member 3, putative, expressed  no original description 	T
35.2	not assigned.unknown	MDP0000178270	 moderately similar to ( 299) AT1G62780 | Symbols:  | unknown protein | chr1:23249349-23251066 REVERSEmoderately similar to ( 290) loc_os04g01540 12004.m05471 protein expressed protein MSP1_C  no original description 	T
31.4	cell.vesicle transport	MDP0000242070	 moderately similar to ( 296) AT5G61210 | Symbols: SNAP33, ATSNAP33, SNP33, ATSNAP33B | SNAP33 (SOLUBLE N-ETHYLMALEIMIDE-SENSITIVE FACTOR ADAPTOR PROTEIN 33); SNAP receptor/ protein binding | chr5:24624027-24625366 FORWARDmoderately similar to ( 256) loc_os02g24080 12002.m33379 protein SNAP25 homologous protein SNAP30, putative, expressed  no original description 	T
27.3.3	RNA.regulation of transcription.AP2/EREBP, APETALA2/Ethylene-responsive element binding protein family	MDP0000288465	 weakly similar to ( 131) AT1G53910 | Symbols: RAP2.12 | RAP2.12; DNA binding / transcription factor | chr1:20135242-20136581 FORWARDweakly similar to ( 112) ERF1_ORYSA Ethylene-responsive transcription factor 1 (Ethylene-responsive element-binding factor 1) (EREBP-1) (OsEREBP1) - Oryza sativa (Rice)weakly similar to ( 123) loc_os01g21120 12001.m08596 protein ethylene-responsive factor-like protein 1, putative, expressed   no original description 	T
27.1	RNA.processing	MDP0000316637	 moderately similar to ( 479) AT2G47420 | Symbols:  | dimethyladenosine transferase, putative | chr2:19457574-19458777 FORWARDmoderately similar to ( 418) loc_os10g10434 12010.m04378 protein dimethyladenosine transferase, putative, expressed    no original description 	T
35.2	not assigned.unknown	MDP0000153787	 moderately similar to ( 286) AT2G43110 | Symbols:  | unknown protein | chr2:17922067-17924291 FORWARDmoderately similar to ( 231) loc_os04g28060 12004.m07925 protein expressed protein  no original description 	T
1.3	PS.calvin cycle	MDP0000325582	 weakly similar to ( 132) AT2G47400 | Symbols: CP12-1, CP12 | CP12-1 | chr2:19446889-19447263 FORWARDweakly similar to ( 126) loc_os03g19380 12003.m07355 protein CP12-2, putative, expressed  no original description 	T
35.2	not assigned.unknown	MDP0000143586	 very weakly similar to (82.8) AT4G13200 | Symbols:  | unknown protein | chr4:7668292-7669166 REVERSEvery weakly similar to (73.9) loc_os04g43350 12004.m09298 protein expressed protein  no original description 	T
30.11	signalling.light	MDP0000898346	 moderately similar to ( 476) AT3G22104 | Symbols:  | phototropic-responsive NPH3 protein-related | chr3:7789814-7792179 FORWARDweakly similar to ( 166) NPH3_ORYSA Coleoptile phototropism protein 1 (Non-phototropic hypocotyl 3-like protein) (NPH3-like protein) - Oryza sativa (Rice)moderately similar to ( 421) loc_os07g36230 12007.m079711 protein transposon protein, putative, Mutator sub-class, expressed  no original description 	T
29.3.1	protein.targeting.nucleus	MDP0000277391	 nearly identical (1428) AT5G53480 | Symbols:  | importin beta-2, putative | chr5:21714016-21716709 FORWARDnearly identical (1385) loc_os12g38110 12012.m07597 protein importin beta-1 subunit, putative, expressed  no original description 	T
29.2.1.2.2.13	protein.synthesis.ribosomal protein.eukaryotic.60S subunit.L13	MDP0000732600	 moderately similar to ( 240) AT3G01790 | Symbols:  | ribosomal protein L13 family protein | chr3:283880-285583 REVERSEvery weakly similar to (82.8) RK13_SPIOL 50S ribosomal protein L13, chloroplast precursor (CL13) - Spinacia oleracea (Spinach)moderately similar to ( 238) loc_os05g15370 12005.m05946 protein 50S ribosomal protein L13, putative, expressed  no original description 	T
35.2	not assigned.unknown	MDP0000296468	 moderately similar to ( 257) AT5G56120 | Symbols:  | unknown protein | chr5:22721483-22722193 FORWARDweakly similar to ( 155) loc_os08g38790 12008.m07862 protein conserved hypothetical protein  no original description 	T
35.2	not assigned.unknown	MDP0000302198	 highly similar to ( 747) AT4G27720 | Symbols:  | LOCATED IN: plasma membrane; EXPRESSED IN: 21 plant structures; EXPRESSED DURING: 13 growth stages; CONTAINS InterPro DOMAIN/s: Protein of unknown function DUF791 (InterPro:IPR008509), Major facilitator superfamily, general substrate transporter (InterPro:IPR016196); BEST Arabidopsis thaliana protein match is: unknown protein (TAIR:AT1G64650.1); Has 496 Blast hits to 491 proteins in 183 species: Archae - 5; Bacteria - 234; Metazoa - 75; Fungi - 33; Plants - 96; Viruses - 0; Other Eukaryotes - 53 (source: NCBI BLink). | chr4:13831203-13833521 FORWARDhighly similar to ( 687) loc_os03g02380 12003.m34984 protein major Facilitator Superfamily protein, expressed  no original description 	T
27.3.32	RNA.regulation of transcription.WRKY domain transcription factor family	MDP0000727570	 weakly similar to ( 132) AT4G23810 | Symbols: WRKY53, ATWRKY53 | WRKY53; DNA binding / protein binding / transcription activator/ transcription factor | chr4:12392666-12393739 REVERSEweakly similar to ( 122) loc_os08g29660 12008.m06969 protein OsWRKY69 - Superfamily of rice TFs having WRKY and zinc finger domains, expressed no original description 	T
31.1	cell.organisation	MDP0000477969	 moderately similar to ( 244) AT2G35490 | Symbols:  | plastid-lipid associated protein PAP, putative | chr2:14912309-14913797 REVERSEmoderately similar to ( 261) PG1_PEA Plastoglobulin-1, chloroplast precursor - Pisum sativum (Garden pea)moderately similar to ( 243) loc_os10g42500 12010.m07004 protein plastid-lipid associated protein 3, chloroplast precursor, putative, expressed    no original description 	T
33.99	development.unspecified	MDP0000142574	 moderately similar to ( 343) AT2G19580 | Symbols: TET2 | TET2 (TETRASPANIN2) | chr2:8472393-8475021 REVERSEmoderately similar to ( 292) loc_os06g37510 12006.m08286 protein senescence-associated protein 5, putative, expressed   no original description 	T
35.2	not assigned.unknown	MDP0000211241	 weakly similar to ( 137) AT2G01990 | Symbols:  | unknown protein | chr2:465197-466497 REVERSEvery weakly similar to (92.0) loc_os09g27590 12009.m05910 protein expressed protein   no original description 	T
35.1	not assigned.no ontology	MDP0000278288	 moderately similar to ( 489) AT4G39390 | Symbols: ATNST-KT1, NST-K1 | NST-K1 (NUCLEOTIDE SUGAR TRANSPORTER-KT 1); nucleotide-sugar transmembrane transporter | chr4:18316278-18317854 FORWARDmoderately similar to ( 471) loc_os02g40090 12002.m100277 protein integral membrane protein like, putative, expressed   no original description 	T
33.99	development.unspecified	MDP0000545006	 highly similar to ( 530) AT2G41500 | Symbols: LIS, EMB2776 | EMB2776; nucleotide binding | chr2:17304319-17306855 REVERSEweakly similar to ( 134) PF20_CHLRE Flagellar WD repeat protein PF20 - Chlamydomonas reinhardtiihighly similar to ( 530) loc_os03g23929 12003.m07739 protein vegetatible incompatibility protein HET-E-1, putative, expressed  no original description 	T
35.2	not assigned.unknown	MDP0000325984	  no original description 	T
35.1	not assigned.no ontology	MDP0000576905	 moderately similar to ( 335) AT1G01225 | Symbols:  | NC domain-containing protein-related | chr1:96064-97242 FORWARDmoderately similar to ( 302) loc_os09g35810 12009.m22022 protein NC domain containing protein, expressed  no original description 	T
35.2	not assigned.unknown	MDP0000069202	 moderately similar to ( 421) AT1G23190 | Symbols:  | phosphoglucomutase, cytoplasmic, putative / glucose phosphomutase, putative | chr1:8219946-8224186 FORWARDmoderately similar to ( 425) PGMC_SOLTU Phosphoglucomutase, cytoplasmic (EC 5.4.2.2) (Glucose phosphomutase) (PGM) - Solanum tuberosum (Potato)moderately similar to ( 419) loc_os03g50480 12003.m35578 protein phosphoglucomutase, cytoplasmic 2, putative, expressed  no original description 	T
13.1.3.5.2	amino acid metabolism.synthesis.aspartate family.lysine.dihydrodipicolinate reductase	MDP0000265388	 moderately similar to ( 387) AT3G59890 | Symbols:  | dihydrodipicolinate reductase family protein | chr3:22124497-22126491 REVERSEmoderately similar to ( 330) loc_os02g24020 12002.m07550 protein dihydrodipicolinate reductase, putative, expressed   no original description 	T
3.2.2	minor CHO metabolism.trehalose.TPP	MDP0000251585	 moderately similar to ( 500) AT5G51460 | Symbols: ATTPPA | ATTPPA; trehalose-phosphatase | chr5:20902266-20904292 FORWARDmoderately similar to ( 442) loc_os10g40550 12010.m22032 protein trehalose-phosphate phosphatase, putative, expressed  no original description 	T
1.2.4.4	PS.photorespiration.glycine cleavage.H protein	MDP0000618810	 moderately similar to ( 253) AT1G32470 | Symbols:  | glycine cleavage system H protein, mitochondrial, putative | chr1:11739479-11740246 REVERSEmoderately similar to ( 251) GCSH_PEA Glycine cleavage system H protein, mitochondrial precursor - Pisum sativum (Garden pea)moderately similar to ( 236) loc_os10g37180 12010.m06515 protein glycine cleavage system H protein, mitochondrial precursor, putative, expressed no original description 	T
21.6	redox.dismutases and catalases	MDP0000309331	 highly similar to ( 945) AT4G35090 | Symbols: CAT2 | CAT2 (CATALASE 2); catalase | chr4:16701105-16703215 REVERSEhighly similar to ( 992) CATA1_GOSHI Catalase isozyme 1 (EC 1.11.1.6) - Gossypium hirsutum (Upland cotton)highly similar to ( 927) loc_os03g03910 12003.m34998 protein catalase-1, putative, expressed  no original description 	T
17.2.3	hormone metabolism.auxin.induced-regulated-responsive-activated	MDP0000323263	 weakly similar to ( 174) AT1G75580 | Symbols:  | auxin-responsive protein, putative | chr1:28377530-28377856 FORWARDvery weakly similar to (89.7) AX15A_SOYBN Auxin-induced protein 15A - Glycine max (Soybean)weakly similar to ( 116) loc_os02g24740 12002.m07621 protein OsSAUR9 - Auxin-responsive SAUR gene family member, expressed no original description 	T
35.2	not assigned.unknown	MDP0000319338	 moderately similar to ( 341) AT2G43945 | Symbols:  | unknown protein | chr2:18197995-18199988 REVERSEmoderately similar to ( 342) loc_os01g23680 12001.m43000 protein cysteine sulfinate desulfinase/cysteine desulfurase and related enzymes, putative, expressed   no original description 	T
27.3.26	RNA.regulation of transcription.MYB-related transcription factor family	MDP0000875621	 weakly similar to ( 175) AT5G56840 | Symbols:  | DNA-binding family protein | chr5:22980789-22982152 FORWARDweakly similar to ( 197) loc_os01g09640 12001.m07588 protein MCB2 protein, putative, expressed no original description 	T
26.12	misc.peroxidases	MDP0000306867	 moderately similar to ( 449) AT5G05340 | Symbols:  | peroxidase, putative | chr5:1579142-1580819 REVERSEmoderately similar to ( 432) PERP7_BRARA Peroxidase P7 (EC 1.11.1.7) (TP7) - Brassica rapa (Turnip)moderately similar to ( 420) loc_os11g02130 12011.m04413 protein peroxidase 52 precursor, putative, expressed   no original description 	T
35.2	not assigned.unknown	MDP0000123555	 weakly similar to ( 157) AT2G35900 | Symbols:  | unknown protein | chr2:15070203-15071540 REVERSEweakly similar to ( 144) loc_os01g56470 12001.m11803 protein mal d 1-associated protein, putative, expressed  no original description 	T
30.2.17	signalling.receptor kinases.DUF 26	MDP0000145972	 moderately similar to ( 243) AT1G34300 | Symbols:  | lectin protein kinase family protein | chr1:12503450-12505939 FORWARDweakly similar to ( 163) KPRO_MAIZE Putative receptor protein kinase ZmPK1 precursor (EC 2.7.11.1) - Zea mays (Maize)moderately similar to ( 370) loc_os04g56130 12004.m10504 protein ATP binding protein, putative   no original description 	T
11.9.3.1	lipid metabolism.lipid degradation.lysophospholipases.phospholipase D	MDP0000300217	 nearly identical (1373) AT3G15730 | Symbols: PLDALPHA1, PLD | PLDALPHA1 (PHOSPHOLIPASE D ALPHA 1); phospholipase D | chr3:5330835-5333474 FORWARDnearly identical (1424) PLDA1_RICCO Phospholipase D alpha 1 precursor (EC 3.1.4.4) (PLD 1) (Choline phosphatase 1) (Phosphatidylcholine-hydrolyzing phospholipase D 1) - Ricinus communis (Castor bean)nearly identical (1346) loc_os01g07760 12001.m42610 protein phospholipase D alpha 1 precursor, putative, expressed   no original description 	T
29.2.2.50	protein.synthesis.ribosome biogenesis.BRIX	MDP0000044157	 moderately similar to ( 445) AT3G15460 | Symbols:  | brix domain-containing protein | chr3:5214279-5216188 REVERSEmoderately similar to ( 422) loc_os02g37870 12002.m08875 protein brix domain-containing protein 2, putative, expressed  no original description 	T
17.4.1	hormone metabolism.cytokinin.synthesis-degradation	MDP0000256199	 highly similar to ( 575) AT2G41510 | Symbols: ATCKX1, CKX1 | CKX1 (CYTOKININ OXIDASE/DEHYDROGENASE 1); cytokinin dehydrogenase | chr2:17314626-17316861 FORWARDmoderately similar to ( 310) CKX1_MAIZE Cytokinin dehydrogenase 1 precursor (EC 1.5.99.12) (Cytokinin oxidase 1) (CKO 1) (COX 1) (ZmCKX1) - Zea mays (Maize)highly similar to ( 554) loc_os05g31040 12005.m07338 protein cytokinin dehydrogenase 1 precursor, putative, expressed  no original description 	T
35.2	not assigned.unknown	MDP0000224068	 weakly similar to ( 171) AT2G42570 | Symbols:  | unknown protein | chr2:17717498-17719921 REVERSEweakly similar to ( 157) loc_os01g46350 12001.m10833 protein expressed protein  no original description 	T
16.1.4.5	secondary metabolism.isoprenoids.carotenoids.lycopene beta cyclase	MDP0000194622	 highly similar to ( 763) AT3G10230 | Symbols: LYC | LYC (LYCOPENE CYCLASE); lycopene beta cyclase | chr3:3164340-3165449 REVERSEhighly similar to ( 788) LCYB_CAPAN Lycopene beta cyclase, chloroplast precursor (EC 1.14.-.-) - Capsicum annuum (Bell pepper)highly similar to ( 646) loc_os02g09750 12002.m06272 protein lycopene beta cyclase, chloroplast precursor, putative, expressed  no original description 	T
27.3.50	RNA.regulation of transcription.General Transcription	MDP0000362681	 moderately similar to ( 262) AT1G73230 | Symbols:  | nascent polypeptide-associated complex (NAC) domain-containing protein | chr1:27540506-27541364 REVERSEmoderately similar to ( 250) loc_os10g34180 12010.m21923 protein transcription factor BTF3, putative, expressed no original description 	T
27.3.67	RNA.regulation of transcription.putative transcription regulator	MDP0000362681	 moderately similar to ( 262) AT1G73230 | Symbols:  | nascent polypeptide-associated complex (NAC) domain-containing protein | chr1:27540506-27541364 REVERSEmoderately similar to ( 250) loc_os10g34180 12010.m21923 protein transcription factor BTF3, putative, expressed no original description 	T
35.1	not assigned.no ontology	MDP0000150257	 highly similar to ( 529) AT1G27760 | Symbols: SAT32, ATSAT32 | interferon-related developmental regulator family protein / IFRD protein family | chr1:9668895-9671322 FORWARDmoderately similar to ( 344) loc_os07g39900 12007.m08232 protein interferon-related developmental regulator family protein, expressed  no original description 	T
10.6.1	cell wall.degradation.cellulases and beta -1,4-glucanases	MDP0000276676	 highly similar to ( 971) AT1G64390 | Symbols: AtGH9C2 | AtGH9C2 (Arabidopsis thaliana glycosyl hydrolase 9C2); carbohydrate binding / catalytic/ hydrolase, hydrolyzing O-glycosyl compounds | chr1:23911329-23914642 FORWARDhighly similar to ( 782) GUN2_ORYSA Endoglucanase 2 precursor (EC 3.2.1.4) (Endo-1,4-beta glucanase 2) (OsGLU5) - Oryza sativa (Rice)highly similar to ( 782) loc_os01g12070 12001.m07822 protein endoglucanase 1 precursor, putative, expressed   no original description 	T
26.3.4	misc.gluco-, galacto- and mannosidases.endoglucanase	MDP0000276676	 highly similar to ( 971) AT1G64390 | Symbols: AtGH9C2 | AtGH9C2 (Arabidopsis thaliana glycosyl hydrolase 9C2); carbohydrate binding / catalytic/ hydrolase, hydrolyzing O-glycosyl compounds | chr1:23911329-23914642 FORWARDhighly similar to ( 782) GUN2_ORYSA Endoglucanase 2 precursor (EC 3.2.1.4) (Endo-1,4-beta glucanase 2) (OsGLU5) - Oryza sativa (Rice)highly similar to ( 782) loc_os01g12070 12001.m07822 protein endoglucanase 1 precursor, putative, expressed   no original description 	T
29.5	protein.degradation	MDP0000286442	 highly similar to ( 751) AT5G05730 | Symbols: ASA1, TRP5, AMT1, WEI2 | ASA1 (ANTHRANILATE SYNTHASE ALPHA SUBUNIT 1); anthranilate synthase | chr5:1719740-1722811 REVERSEhighly similar to ( 696) loc_os03g61120 12003.m10996 protein anthranilate synthase component I-1, chloroplast precursor, putative, expressed     no original description 	T
29.3.4.2	protein.targeting.secretory pathway.golgi	MDP0000198525	 moderately similar to ( 270) AT4G14160 | Symbols:  | transport protein, putative | chr4:8167574-8173026 FORWARDmoderately similar to ( 294) loc_os08g36994 12008.m07687 protein protein transport protein Sec23B, putative, expressed no original description 	T
35.2	not assigned.unknown	MDP0000272009	 very weakly similar to (55.5) AT2G40475 | Symbols:  | unknown protein | chr2:16907222-16907803 REVERSEvery weakly similar to (53.1) loc_os01g07240 12001.m07350 protein expressed protein  no original description 	T
17.1.3	hormone metabolism.abscisic acid.induced-regulated-responsive-activated	MDP0000181297	 weakly similar to ( 192) AT2G22475 | Symbols: GEM | GEM (GL2-EXPRESSION MODULATOR) | chr2:9541523-9544778 FORWARDweakly similar to ( 194) loc_os06g11430 12006.m05860 protein FIP1, putative, expressed no original description 	T
35.2	not assigned.unknown	MDP0000265366	 moderately similar to ( 259) AT5G50100 | Symbols:  | FUNCTIONS IN: molecular_function unknown; INVOLVED IN: biological_process unknown; LOCATED IN: mitochondrion; EXPRESSED IN: 22 plant structures; EXPRESSED DURING: 15 growth stages; CONTAINS InterPro DOMAIN/s: Thioredoxin-like fold (InterPro:IPR012336), Putative thiol-disulphide oxidoreductase DCC (InterPro:IPR007263); Has 598 Blast hits to 598 proteins in 152 species: Archae - 0; Bacteria - 266; Metazoa - 3; Fungi - 0; Plants - 27; Viruses - 0; Other Eukaryotes - 302 (source: NCBI BLink). | chr5:20371916-20373172 FORWARDmoderately similar to ( 241) loc_os04g57310 12004.m35507 protein PBng143, putative, expressed  no original description 	T
28.2	DNA.repair	MDP0000267131	 nearly identical (1102) AT1G73430 | Symbols:  | sec34-like family protein | chr1:27604096-27610829 FORWARDhighly similar to ( 517) R51A2_MAIZE DNA repair protein RAD51 homolog B (Rad51-like protein B) (RAD51B) (ZmRAD51b) - Zea mays (Maize)nearly identical (1015) loc_os08g34970 12008.m07486 protein conserved oligomeric Golgi complex component 3, putative, expressed  no original description 	T
35.2	not assigned.unknown	MDP0000761358	  no original description 	T
20.1	stress.biotic	MDP0000285753	 weakly similar to ( 180) AT3G20600 | Symbols: NDR1 | NDR1 (non race-specific disease resistance 1); signal transducer | chr3:7194877-7195536 FORWARDvery weakly similar to (88.6) loc_os05g01330 12005.m04673 protein expressed protein  no original description 	T
18.1	Co-factor and vitamine metabolism.molybdenum cofactor	MDP0000163186	 weakly similar to ( 114) AT4G10100 | Symbols: CNX7, SIR5 | CNX7; catalytic | chr4:6309091-6309381 FORWARDweakly similar to ( 107) loc_os02g35200 12002.m08608 protein VP15, putative, expressed no original description 	T
29.5.11	protein.degradation.ubiquitin	MDP0000164649	 highly similar to ( 712) AT1G16860 | Symbols:  | merozoite surface protein-related | chr1:5768280-5770183 FORWARDhighly similar to ( 721) loc_os08g13350 12008.m26625 protein cysteine-type endopeptidase/ ubiquitin thiolesterase, putative, expressed   no original description 	T
27.3.5	RNA.regulation of transcription.ARR	MDP0000328874	 moderately similar to ( 234) AT1G59940 | Symbols: ARR3 | ARR3 (RESPONSE REGULATOR 3); transcription regulator/ two-component response regulator | chr1:22065894-22066895 REVERSEvery weakly similar to (73.9) PRR37_ORYSA Two-component response regulator-like PRR37 (Pseudo-response regulator 37) (OsPRR37) - Oryza sativa (Rice)weakly similar to ( 166) loc_os01g72330 12001.m13268 protein OsRR4 - Rice type-A response regulator, expressed  no original description 	T
26.2	misc.UDP glucosyl and glucoronyl transferases	MDP0000808264	 moderately similar to ( 331) AT1G61050 | Symbols:  | alpha 1,4-glycosyltransferase family protein / glycosyltransferase sugar-binding DXD motif-containing protein | chr1:22486736-22488043 FORWARDmoderately similar to ( 290) loc_os04g44740 12004.m09431 protein transferase/ transferase, transferring glycosyl groups, putative, expressed  no original description 	T
26.12	misc.peroxidases	MDP0000283650	 moderately similar to ( 351) AT5G05340 | Symbols:  | peroxidase, putative | chr5:1579142-1580819 REVERSEmoderately similar to ( 358) PER1_ARAHY Cationic peroxidase 1 precursor (EC 1.11.1.7) (PNPC1) - Arachis hypogaea (Peanut)moderately similar to ( 351) loc_os11g02130 12011.m04413 protein peroxidase 52 precursor, putative, expressed  no original description 	T
35.1	not assigned.no ontology	MDP0000149371	 nearly identical (1393) AT1G56500 | Symbols:  | haloacid dehalogenase-like hydrolase family protein | chr1:21159775-21167092 FORWARDnearly identical (1079) loc_os03g19760 12003.m35188 protein NHL repeat protein, putative, expressed   no original description 	T
27.3.3	RNA.regulation of transcription.AP2/EREBP, APETALA2/Ethylene-responsive element binding protein family	MDP0000231748	 moderately similar to ( 323) AT1G16060 | Symbols:  | ovule development protein, putative | chr1:5508563-5511609 FORWARDmoderately similar to ( 224) BBM2_BRANA Protein BABY BOOM 2 (BnBBM2) - Brassica napus (Rape)moderately similar to ( 327) loc_os09g25600 12009.m60258 protein protein BABY BOOM 2, putative, expressed  no original description 	T
26.6	misc.O-methyl transferases	MDP0000244230	 highly similar to ( 553) AT1G04870 | Symbols: PRMT10, ATPRMT10 | PRMT10; [myelin basic protein]-arginine N-methyltransferase/ histone-arginine N-methyltransferase/ methyltransferase/ protein-arginine omega-N asymmetric methyltransferase/ protein-arginine omega-N monomethyltransferase | chr1:1373485-1375598 REVERSEhighly similar to ( 504) loc_os06g05090 12006.m091600 protein methyltransferase, putative, expressed  no original description 	T
34.9	transport.metabolite transporters at the mitochondrial membrane	MDP0000293674	 moderately similar to ( 451) AT5G64970 | Symbols:  | mitochondrial substrate carrier family protein | chr5:25958806-25960443 REVERSEweakly similar to ( 104) BT1_MAIZE Protein brittle-1, chloroplast precursor - Zea mays (Maize)moderately similar to ( 440) loc_os03g09110 12003.m06417 protein protein brittle-1, chloroplast precursor, putative, expressed    no original description 	T
26.4	misc.beta 1,3 glucan hydrolases	MDP0000508660	 weakly similar to ( 133) AT1G09460 | Symbols:  | glucan endo-1,3-beta-glucosidase-related | chr1:3053901-3055090 FORWARDvery weakly similar to (84.0) E13B_WHEAT Glucan endo-1,3-beta-glucosidase precursor (EC 3.2.1.39) ((1->3)-beta-glucan endohydrolase) ((1->3)-beta-glucanase) (Beta-1,3-endoglucanase) - Triticum aestivum (Wheat)weakly similar to ( 122) loc_os05g50490 12005.m27988 protein expressed protein   no original description 	T
29.2.1.1.1.2.11	protein.synthesis.ribosomal protein.prokaryotic.chloroplast.50S subunit.L11	MDP0000238515	 moderately similar to ( 265) AT1G32990 | Symbols: PRPL11 | PRPL11 (PLASTID RIBOSOMAL PROTEIN L11); structural constituent of ribosome | chr1:11955827-11957139 FORWARDmoderately similar to ( 264) RK11_SPIOL 50S ribosomal protein L11, chloroplast precursor (CL11) - Spinacia oleracea (Spinach)moderately similar to ( 247) loc_os03g03020 12003.m05839 protein 50S ribosomal protein L11, chloroplast precursor, putative, expressed  no original description 	T
35.1.5	not assigned.no ontology.pentatricopeptide (PPR) repeat-containing protein	MDP0000708926	 highly similar to ( 541) AT5G65560 | Symbols:  | pentatricopeptide (PPR) repeat-containing protein | chr5:26201012-26203759 REVERSEmoderately similar to ( 322) RF1_ORYSA Rf1 protein, mitochondrial precursor (PPR protein) (Fertility restorer) (Restorer for CMS) - Oryza sativa (Rice)moderately similar to ( 480) loc_os08g21561 12008.m80312 protein EMB2745, putative  no original description 	T
35.1	not assigned.no ontology	MDP0000171453	 highly similar to ( 845) AT1G15490 | Symbols:  | hydrolase, alpha/beta fold family protein | chr1:5320859-5323422 REVERSEhighly similar to ( 697) loc_os01g45960 12001.m43116 protein hydrolase, putative, expressed   no original description 	T
34.99	transport.misc	MDP0000124654	 highly similar to ( 584) AT4G25640 | Symbols:  | MATE efflux family protein | chr4:13076953-13078965 REVERSEmoderately similar to ( 479) loc_os08g44870 12008.m080240 protein transparent testa 12 protein, putative, expressed  no original description 	T
26.23	misc.rhodanese	MDP0000205279	 moderately similar to ( 214) AT4G27700 | Symbols:  | rhodanese-like domain-containing protein | chr4:13826541-13827673 REVERSEvery weakly similar to (51.2) DIN1_RAPSA Senescence-associated protein DIN1 - Raphanus sativus (Radish)moderately similar to ( 226) loc_os09g36040 12009.m21934 protein rhodanese-like domain containing protein, expressed   no original description 	T
35.2	not assigned.unknown	MDP0000260735	 moderately similar to ( 379) AT4G25550 | Symbols:  | protein binding | chr4:13048519-13050873 FORWARDmoderately similar to ( 382) loc_os04g58640 12004.m35057 protein cleavage and polyadenylation specificity factor 5, putative, expressed no original description 	T
35.2	not assigned.unknown	MDP0000173807	 very weakly similar to (92.4) AT1G48780 | Symbols:  | unknown protein | chr1:18041989-18042744 FORWARD no original description 	T
29.4.1.57	protein.postranslational modification.kinase.receptor like cytoplasmatic kinase VII	MDP0000268423	 highly similar to ( 787) AT1G66150 | Symbols: TMK1 | TMK1 (TRANSMEMBRANE KINASE 1); transmembrane receptor protein serine/threonine kinase | chr1:24631503-24634415 FORWARDmoderately similar to ( 273) PSKR_DAUCA Phytosulfokine receptor precursor (EC 2.7.11.1) (Phytosulfokine LRR receptor kinase) - Daucus carota (Carrot)highly similar to ( 995) loc_os04g58910 12004.m10771 protein receptor protein kinase TMK1 precursor, putative, expressed   no original description 	T
35.2	not assigned.unknown	MDP0000787512	    no original description 	T
35.1	not assigned.no ontology	MDP0000320770	 weakly similar to ( 159) AT1G04290 | Symbols:  | thioesterase family protein | chr1:1147721-1148352 REVERSEweakly similar to ( 120) loc_os04g35590 12004.m08648 protein thioesterase superfamily member 2, putative, expressed   no original description 	T
29.5.11.4.2	protein.degradation.ubiquitin.E3.RING	MDP0000302491	 moderately similar to ( 280) AT5G01450 | Symbols:  | protein binding / zinc ion binding | chr5:183693-186244 REVERSEvery weakly similar to (74.7) loc_os05g44550 12005.m08581 protein ubiquitin-protein ligase/ zinc ion binding protein, putative  no original description 	T
13.1.4.1	amino acid metabolism.synthesis.branched chain group.common	MDP0000163713	 highly similar to ( 918) AT3G58610 | Symbols:  | ketol-acid reductoisomerase | chr3:21671561-21674639 FORWARDhighly similar to ( 922) ILV5_PEA Ketol-acid reductoisomerase, chloroplast precursor (EC 1.1.1.86) (Acetohydroxy-acid reductoisomerase) (Alpha-keto-beta-hydroxylacil reductoisomerase) - Pisum sativum (Garden pea)highly similar to ( 889) loc_os05g49800 12005.m09050 protein ketol-acid reductoisomerase, chloroplast precursor, putative, expressed  no original description 	T
26.24	misc.GCN5-related N-acetyltransferase	MDP0000318849	 moderately similar to ( 230) AT1G72030 | Symbols:  | GCN5-related N-acetyltransferase (GNAT) family protein | chr1:27110887-27111825 FORWARDmoderately similar to ( 217) loc_os07g16130 12007.m06056 protein histone acetyltransferase HPA2 and related acetyltransferases, putative, expressed TrbL  no original description 	T
35.1	not assigned.no ontology	MDP0000232309	 moderately similar to ( 323) AT4G15470 | Symbols:  | EXPRESSED IN: 24 plant structures; EXPRESSED DURING: 15 growth stages; CONTAINS InterPro DOMAIN/s: Uncharacterised protein family UPF0005 (InterPro:IPR006214); BEST Arabidopsis thaliana protein match is: glutamate binding (TAIR:AT1G03070.1); Has 4000 Blast hits to 3999 proteins in 955 species: Archae - 0; Bacteria - 1775; Metazoa - 750; Fungi - 92; Plants - 143; Viruses - 77; Other Eukaryotes - 1163 (source: NCBI BLink). | chr4:8843661-8845505 FORWARDvery weakly similar to (52.0) BI1_ORYSA Bax inhibitor 1 (BI-1) (OsBI-1) - Oryza sativa (Rice)moderately similar to ( 291) loc_os05g33360 12005.m27816 protein transmembrane BAX inhibitor motif-containing protein 4, putative, expressed  no original description 	T
35.2	not assigned.unknown	MDP0000349101	 weakly similar to ( 107) AT5G55125 | Symbols:  | unknown protein | chr5:22372737-22372967 FORWARDweakly similar to ( 102) loc_os11g31650 12011.m80153 protein expressed protein no original description 	T
35.2	not assigned.unknown	MDP0000133853	 nearly identical (1839) AT5G54440 | Symbols:  | unknown protein | chr5:22100056-22107695 FORWARDnearly identical (1694) loc_os12g36760 12012.m07461 protein expressed protein   no original description 	T
27.3.22	RNA.regulation of transcription.HB,Homeobox transcription factor family	MDP0000124827	 moderately similar to ( 234) AT5G15150 | Symbols: ATHB-3, HAT7, ATHB3 | ATHB-3 (ARABIDOPSIS THALIANA HOMEOBOX 3); DNA binding / sequence-specific DNA binding / transcription factor | chr5:4913951-4915609 REVERSEweakly similar to ( 177) loc_os03g07450 12003.m06259 protein homeobox-leucine zipper protein HAT7, putative, expressed   no original description 	T
34.8	transport.metabolite transporters at the envelope membrane	MDP0000455174	 moderately similar to ( 478) AT1G12500 | Symbols:  | phosphate translocator-related | chr1:4263542-4264957 REVERSEvery weakly similar to (66.2) TPT_PEA Triose phosphate/phosphate translocator, chloroplast precursor (cTPT) (p36) (E30) - Pisum sativum (Garden pea)moderately similar to ( 338) loc_os03g17740 12003.m07199 protein organic anion transporter, putative, expressed   no original description 	T
13.1.5.3.1	amino acid metabolism.synthesis.serine-glycine-cysteine group.cysteine.OASTL	MDP0000503728	 weakly similar to ( 142) AT4G14880 | Symbols: OASA1, CYTACS1, ATCYS-3A | OASA1 (O-ACETYLSERINE (THIOL) LYASE (OAS-TL) ISOFORM A1); cysteine synthase | chr4:8518209-8520050 REVERSEweakly similar to ( 158) CYSK_SOLTU Cysteine synthase (EC 2.5.1.47) (O-acetylserine sulfhydrylase) (O-acetylserine (Thiol)-lyase) (CSase A) (CS-A) (OAS-TL A) - Solanum tuberosum (Potato)weakly similar to ( 154) loc_os03g53650 12003.m35628 protein cysteine synthase, putative, expressed no original description 	T
26.2	misc.UDP glucosyl and glucoronyl transferases	MDP0000256891	 moderately similar to ( 213) AT4G38040 | Symbols:  | exostosin family protein | chr4:17867501-17869131 FORWARDmoderately similar to ( 223) loc_os02g09430 12002.m06240 protein exostosin-like, putative, expressed  no original description 	T
29.5.11.20	protein.degradation.ubiquitin.proteasom	MDP0000299188	 highly similar to ( 812) AT1G53750 | Symbols: RPT1A | RPT1A (REGULATORY PARTICLE TRIPLE-A 1A); ATPase | chr1:20065921-20068324 REVERSEhighly similar to ( 838) PRS7_PRUPE 26S protease regulatory subunit 7 (26S proteasome subunit 7) (26S proteasome AAA-ATPase subunit RPT1) (Regulatory particle triple-A ATPase subunit 1) - Prunus persica (Peach)highly similar to ( 813) loc_os06g09290 12006.m05648 protein 26S protease regulatory subunit 7, putative, expressed  no original description 	T
35.2	not assigned.unknown	MDP0000656610	 moderately similar to ( 279) AT5G41980 | Symbols:  | unknown protein | chr5:16793765-16794889 FORWARDmoderately similar to ( 260) loc_os12g20400 12012.m05986 protein transposon protein, putative, CACTA, En/Spm sub-class no original description 	T
31.4	cell.vesicle transport	MDP0000219580	 moderately similar to ( 356) AT3G09740 | Symbols: SYP71, ATSYP71 | SYP71 (SYNTAXIN OF PLANTS 71); protein transporter | chr3:2989615-2991354 FORWARDmoderately similar to ( 328) loc_os05g48020 12005.m08873 protein syntaxin 72, putative, expressed  no original description 	T
27.3.69	RNA.regulation of transcription.SET-domain transcriptional regulator family	MDP0000241310	 highly similar to ( 597) AT1G73100 | Symbols: SUVH3, SDG19 | SUVH3 (SU(VAR)3-9 HOMOLOG 3); histone methyltransferase | chr1:27491970-27493979 FORWARDhighly similar to ( 535) loc_os11g38900 12011.m07738 protein histone-lysine N-methyltransferase, H3 lysine-9 specific SUVH1, putative, expressed  no original description 	T
8.2.9	TCA / org transformation.other organic acid transformatons.cyt MDH	MDP0000197620	 highly similar to ( 543) AT1G04410 | Symbols:  | malate dehydrogenase, cytosolic, putative | chr1:1189418-1191267 REVERSEhighly similar to ( 557) MDHC_MAIZE Malate dehydrogenase, cytoplasmic (EC 1.1.1.37) - Zea mays (Maize)highly similar to ( 550) loc_os10g33800 12010.m21921 protein malate dehydrogenase, cytoplasmic, putative, expressed   no original description 	T
29.5.1	protein.degradation.subtilases	MDP0000950870	 nearly identical (1078) AT2G05920 | Symbols:  | subtilase family protein | chr2:2269831-2272207 REVERSEhighly similar to ( 867) loc_os10g25450 12010.m05491 protein subtilisin-like protease precursor, putative, expressed   no original description 	T
35.2	not assigned.unknown	MDP0000809657	 weakly similar to ( 188) AT5G46850 | Symbols:  | FUNCTIONS IN: molecular_function unknown; INVOLVED IN: GPI anchor biosynthetic process; LOCATED IN: endoplasmic reticulum membrane; EXPRESSED IN: 22 plant structures; EXPRESSED DURING: 13 growth stages; CONTAINS InterPro DOMAIN/s: PIG-X/PBN1 (InterPro:IPR013233); Has 52 Blast hits to 52 proteins in 24 species: Archae - 0; Bacteria - 0; Metazoa - 31; Fungi - 4; Plants - 13; Viruses - 0; Other Eukaryotes - 4 (source: NCBI BLink). | chr5:19010714-19012010 FORWARDweakly similar to ( 170) loc_os12g40500 12012.m26956 protein expressed protein no original description 	T
34.99	transport.misc	MDP0000231194	 moderately similar to ( 386) AT3G52640 | Symbols:  | nicastrin-related | chr3:19515625-19520290 REVERSEmoderately similar to ( 379) loc_os02g50360 12002.m10070 protein nicastrin family protein, expressed  no original description 	T
26.3.2	misc.gluco-, galacto- and mannosidases.beta-galactosidase	MDP0000895533	 moderately similar to ( 360) AT4G36360 | Symbols: BGAL3 | BGAL3 (beta-galactosidase 3); beta-galactosidase/ catalytic/ cation binding / sugar binding | chr4:17176840-17181143 REVERSEmoderately similar to ( 285) BGAL_ASPOF Beta-galactosidase precursor (EC 3.2.1.23) (Lactase) - Asparagus officinalis (Garden asparagus)moderately similar to ( 347) loc_os03g06940 12003.m35030 protein beta-galactosidase precursor, putative, expressed no original description 	T
10.6.1	cell wall.degradation.cellulases and beta -1,4-glucanases	MDP0000131267	 highly similar to ( 969) AT1G64390 | Symbols: AtGH9C2 | AtGH9C2 (Arabidopsis thaliana glycosyl hydrolase 9C2); carbohydrate binding / catalytic/ hydrolase, hydrolyzing O-glycosyl compounds | chr1:23911329-23914642 FORWARDhighly similar to ( 780) GUN2_ORYSA Endoglucanase 2 precursor (EC 3.2.1.4) (Endo-1,4-beta glucanase 2) (OsGLU5) - Oryza sativa (Rice)highly similar to ( 780) loc_os01g12070 12001.m07822 protein endoglucanase 1 precursor, putative, expressed   no original description 	T
26.3.4	misc.gluco-, galacto- and mannosidases.endoglucanase	MDP0000131267	 highly similar to ( 969) AT1G64390 | Symbols: AtGH9C2 | AtGH9C2 (Arabidopsis thaliana glycosyl hydrolase 9C2); carbohydrate binding / catalytic/ hydrolase, hydrolyzing O-glycosyl compounds | chr1:23911329-23914642 FORWARDhighly similar to ( 780) GUN2_ORYSA Endoglucanase 2 precursor (EC 3.2.1.4) (Endo-1,4-beta glucanase 2) (OsGLU5) - Oryza sativa (Rice)highly similar to ( 780) loc_os01g12070 12001.m07822 protein endoglucanase 1 precursor, putative, expressed   no original description 	T
9.1.1	mitochondrial electron transport / ATP synthesis.NADH-DH.complex I	MDP0000309083	 moderately similar to ( 423) AT4G28510 | Symbols: ATPHB1 | ATPHB1 (PROHIBITIN 1) | chr4:14084970-14086372 REVERSEmoderately similar to ( 417) loc_os07g15880 12007.m06032 protein mitochondrial prohibitin complex protein 2, putative, expressed  no original description 	T
35.1	not assigned.no ontology	MDP0000122432	 weakly similar to ( 147) AT3G10420 | Symbols:  | sporulation protein-related | chr3:3239307-3241571 FORWARDvery weakly similar to (99.8) loc_os07g08970 12007.m05353 protein expressed protein  no original description 	T
26.19	misc.plastocyanin-like	MDP0000197775	 very weakly similar to (91.3) AT3G27200 | Symbols:  | plastocyanin-like domain-containing protein | chr3:10043738-10044340 REVERSEvery weakly similar to (55.5) BCP_PEA Blue copper protein precursor - Pisum sativum (Garden pea)weakly similar to ( 122) loc_os02g48820 12002.m09916 protein uclacyanin-2 precursor, putative, expressed  no original description 	T
10.6.2	cell wall.degradation.mannan-xylose-arabinose-fucose	MDP0000197977	 nearly identical (1144) AT5G64570 | Symbols: XYL4, ATBXL4 | XYL4; hydrolase, hydrolyzing O-glycosyl compounds / xylan 1,4-beta-xylosidase | chr5:25810227-25813309 REVERSEmoderately similar to ( 444) XYNB_PRUPE Putative beta-D-xylosidase (EC 3.2.1.-) (PpAz152) (Fragment) - Prunus persica (Peach)nearly identical (1048) loc_os04g54810 12004.m10373 protein beta-D-xylosidase, putative, expressed   no original description 	T
35.2	not assigned.unknown	MDP0000232931	 moderately similar to ( 214) AT3G54750 | Symbols:  | unknown protein | chr3:20264833-20268416 REVERSEweakly similar to ( 149) loc_os12g42700 12012.m08049 protein expressed protein  no original description 	T
35.1	not assigned.no ontology	MDP0000549989	 moderately similar to ( 374) AT1G54780 | Symbols:  | thylakoid lumen 18.3 kDa protein | chr1:20439533-20440953 FORWARDmoderately similar to ( 343) loc_os05g33280 12005.m07560 protein beta-propeller domains of methanol dehydrogenase type, putative, expressed no original description 	T
29.2.1.2.2.82	protein.synthesis.ribosomal protein.eukaryotic.60S subunit.P2	MDP0000822395	 very weakly similar to (98.2) AT2G27710 | Symbols:  | 60S acidic ribosomal protein P2 (RPP2B) | chr2:11816929-11817670 FORWARDvery weakly similar to (91.7) RLA2B_MAIZE 60S acidic ribosomal protein P2B - Zea mays (Maize)very weakly similar to (91.3) loc_os05g37330 12005.m07913 protein 60S acidic ribosomal protein P2B, putative, expressed no original description 	T
35.2	not assigned.unknown	MDP0000460502	 weakly similar to ( 149) AT5G47870 | Symbols:  | unknown protein | chr5:19384555-19385808 REVERSEweakly similar to ( 123) loc_os09g33790 12009.m06428 protein snRK1-interacting protein 1, putative, expressed  no original description 	T
28.1.3	DNA.synthesis/chromatin structure.histone	MDP0000935120	 weakly similar to ( 137) AT5G27670 | Symbols: HTA7 | HTA7 (HISTONE H2A 7); DNA binding | chr5:9792807-9793365 REVERSEweakly similar to ( 144) H2A4_ORYSA Probable histone H2A.4 - Oryza sativa (Rice)weakly similar to ( 144) loc_os05g38640 12005.m08043 protein histone H2A, putative, expressed no original description 	T
35.2	not assigned.unknown	MDP0000274370	 very weakly similar to (62.8) AT3G01960 | Symbols:  | unknown protein | chr3:324581-325087 REVERSE no original description 	T
35.2	not assigned.unknown	MDP0000847267	 moderately similar to ( 263) AT1G76020 | Symbols:  | INVOLVED IN: biological_process unknown; LOCATED IN: cell wall, vacuole; EXPRESSED IN: 22 plant structures; EXPRESSED DURING: 13 growth stages; CONTAINS InterPro DOMAIN/s: Thioredoxin fold (InterPro:IPR012335), Thioredoxin-like fold (InterPro:IPR012336); BEST Arabidopsis thaliana protein match is: unknown protein (TAIR:AT1G20225.1); Has 118 Blast hits to 116 proteins in 37 species: Archae - 0; Bacteria - 51; Metazoa - 12; Fungi - 4; Plants - 28; Viruses - 0; Other Eukaryotes - 23 (source: NCBI BLink). | chr1:28532243-28533542 REVERSEmoderately similar to ( 218) loc_os09g37020 12009.m06651 protein expressed protein no original description 	T
29.2.1.2.2.13	protein.synthesis.ribosomal protein.eukaryotic.60S subunit.L13	MDP0000152308	 moderately similar to ( 311) AT3G49010 | Symbols: ATBBC1, BBC1, RSU2 | ATBBC1 (ARABIDOPSIS THALIANA BREAST BASIC CONSERVED 1); structural constituent of ribosome | chr3:18166971-18168047 REVERSEmoderately similar to ( 306) RL131_BRANA 60S ribosomal protein L13-1 (Cold-induced protein C24A) - Brassica napus (Rape)moderately similar to ( 305) loc_os03g37970 12003.m34891 protein 60S ribosomal protein L13-2, putative, expressed no original description 	T
11.3.3	lipid metabolism.Phospholipid synthesis.phosphatidate cytidylyltransferase	MDP0000197773	 highly similar to ( 695) AT4G22340 | Symbols:  | phosphatidate cytidylyltransferase, putative / CDP-diglyceride synthetase, putative | chr4:11800150-11802621 REVERSEhighly similar to ( 704) CDS1_SOLTU Phosphatidate cytidylyltransferase (EC 2.7.7.41) (CDP-diglyceride synthetase) (CDP-diglyceride pyrophosphorylase) (CDP-diacylglycerol synthase) (CDS) (CTP:phosphatidate cytidylyltransferase) (CDP-DAG synthase) (CDP-DG synthetase) - Solahighly similar to ( 663) loc_os01g55360 12001.m43197 protein phosphatidate cytidylyltransferase, putative, expressed  no original description 	T
29.5.5	protein.degradation.serine protease	MDP0000151096	 moderately similar to ( 274) AT3G07950 | Symbols:  | rhomboid protein-related | chr3:2531982-2534277 FORWARDmoderately similar to ( 242) loc_os07g46170 12007.m28913 protein transmembrane protein 115, putative, expressed no original description 	T
16.8.5.1	secondary metabolism.flavonoids.isoflavones.isoflavone reductase	MDP0000636927	 moderately similar to ( 238) AT4G39230 | Symbols:  | isoflavone reductase, putative | chr4:18266024-18267604 REVERSEmoderately similar to ( 245) IFRH_TOBAC Isoflavone reductase homolog A622 (EC 1.3.1.-) - Nicotiana tabacum (Common tobacco)moderately similar to ( 413) loc_os03g15360 12003.m06973 protein leucoanthocyanidin reductase, putative  no original description 	T
30.3	signalling.calcium	MDP0000519305	 moderately similar to ( 201) AT3G49260 | Symbols: iqd21 | iqd21 (IQ-domain 21); calmodulin binding | chr3:18262755-18265859 FORWARDvery weakly similar to (73.2) loc_os05g03190 12005.m04853 protein IQD1, putative, expressed   no original description 	T
16.4.1	secondary metabolism.N misc.alkaloid-like	MDP0000171619	 weakly similar to ( 121) AT3G57030 | Symbols:  | strictosidine synthase family protein | chr3:21101653-21103204 REVERSEvery weakly similar to (67.0) STSY_CATRO Strictosidine synthase precursor (EC 4.3.3.2) - Catharanthus roseus (Rosy periwinkle) (Madagascar periwinkle)moderately similar to ( 206) loc_os07g42250 12007.m08460 protein strictosidine synthase 1 precursor, putative, expressed no original description 	T
27.3.6	RNA.regulation of transcription.bHLH,Basic Helix-Loop-Helix family	MDP0000288670	 weakly similar to ( 133) AT5G61280 | Symbols:  | remorin family protein | chr5:24643625-24644910 REVERSEvery weakly similar to (54.3) REMO_SOLTU Remorin (pp34) - Solanum tuberosum (Potato)very weakly similar to ( 100) loc_os03g11300 12003.m06581 protein expressed protein  TrbL PRK07003  no original description 	T
30.3	signalling.calcium	MDP0000006177	 highly similar to ( 592) AT1G56340 | Symbols: CRT1 | CRT1 (CALRETICULIN 1); calcium ion binding / unfolded protein binding | chr1:21090022-21092630 REVERSEhighly similar to ( 613) CALR_BETVU Calreticulin precursor - Beta vulgaris (Sugar beet)highly similar to ( 559) loc_os07g14270 12007.m29118 protein calreticulin precursor, putative, expressed  no original description 	T
11.9.3.3	lipid metabolism.lipid degradation.lysophospholipases.glycerophosphodiester phosphodiesterase	MDP0000269941	 highly similar to ( 595) AT4G26690 | Symbols: SHV3, MRH5, GPDL2 | SHV3 (SHAVEN 3); glycerophosphodiester phosphodiesterase/ kinase | chr4:13456793-13459890 REVERSEhighly similar to ( 586) loc_os02g37590 12002.m08847 protein glycerophosphoryl diester phosphodiesterase family protein, putative, expressed no original description 	T
20.2.99	stress.abiotic.unspecified	MDP0000236390	 weakly similar to ( 114) AT3G05950 | Symbols:  | germin-like protein, putative | chr3:1781130-1781964 REVERSEweakly similar to ( 101) GLP1_MESCR Germin-like protein precursor - Mesembryanthemum crystallinum (Common ice plant)weakly similar to ( 108) loc_os04g52720 12004.m10165 protein germin-like protein subfamily 1 member 11 precursor, putative, expressed no original description 	T
33.99	development.unspecified	MDP0000269231	 highly similar to ( 647) AT5G14120 | Symbols:  | nodulin family protein | chr5:4556308-4558447 FORWARDhighly similar to ( 668) loc_os08g42010 12008.m08179 protein nodulin-like protein, putative, expressed no original description 	T
35.2	not assigned.unknown	MDP0000299695	 weakly similar to ( 141) AT1G73885 | Symbols:  | unknown protein | chr1:27786864-27787708 FORWARDweakly similar to ( 136) loc_os03g50270 12003.m10020 protein expressed protein  no original description 	T
34.99	transport.misc	MDP0000308201	 highly similar to ( 535) AT5G38030 | Symbols:  | MATE efflux family protein | chr5:15171486-15175302 REVERSEmoderately similar to ( 397) loc_os08g43654 12008.m08339 protein transparent testa 12 protein, putative, expressed TrbL  no original description 	T
31.1	cell.organisation	MDP0000144321	 nearly identical (1249) AT1G54560 | Symbols: XIE, ATXIE | XIE; motor/ protein binding | chr1:20371649-20379745 REVERSEnearly identical (1263) loc_os03g48140 12003.m09824 protein myosin XI, putative, expressed  MSP1_C  no original description 	T
31.4	cell.vesicle transport	MDP0000230516	 weakly similar to ( 124) AT4G23460 | Symbols:  | beta-adaptin, putative | chr4:12243899-12248898 REVERSEweakly similar to ( 113) loc_os03g23950 12003.m07741 protein AP-1 complex subunit beta-1, expressed  no original description 	T
27.3.22	RNA.regulation of transcription.HB,Homeobox transcription factor family	MDP0000144307	 weakly similar to ( 192) AT3G18010 | Symbols: WOX1 | WOX1 (WUSCHEL related homeobox 1); transcription factor | chr3:6161155-6163183 REVERSEweakly similar to ( 108) WOX3B_MAIZE WUSCHEL-related homeobox 3B (Narrow sheath protein 2) - Zea mays (Maize)weakly similar to ( 108) loc_os12g01120 12012.m04116 protein WUSCHEL-related homeobox 3B, putative  no original description 	T
35.2	not assigned.unknown	MDP0000266004	 weakly similar to ( 139) AT5G22580 | Symbols:  | FUNCTIONS IN: molecular_function unknown; INVOLVED IN: biological_process unknown; LOCATED IN: chloroplast; EXPRESSED IN: 22 plant structures; EXPRESSED DURING: 13 growth stages; CONTAINS InterPro DOMAIN/s: Stress responsive alpha-beta barrel (InterPro:IPR013097), Dimeric alpha-beta barrel (InterPro:IPR011008); BEST Arabidopsis thaliana protein match is: HS1 (HEAT STABLE PROTEIN 1) (TAIR:AT3G17210.1); Has 231 Blast hits to 231 proteins in 57 species: Archae - 0; Bacteria - 89; Metazoa - 0; Fungi - 4; Plants - 98; Viruses - 0; Other Eukaryotes - 40 (source: NCBI BLink). | chr5:7502709-7503137 FORWARDweakly similar to ( 122) loc_os11g05290 12011.m04727 protein pop3 peptide, putative, expressed  no original description 	T
10.2.2	cell wall.cellulose synthesis.COBRA	MDP0000360864	 moderately similar to ( 256) AT5G60920 | Symbols: COB | COB (COBRA) | chr5:24511466-24513932 REVERSEmoderately similar to ( 255) COBL1_ORYSA COBRA-like 1 protein precursor (BRITTLE CULM1-like 6 protein) - Oryza sativa (Rice)moderately similar to ( 255) loc_os07g41310 12007.m08367 protein COBRA-like protein 4 precursor, putative, expressed no original description 	T
29.2.1.2.1.15	protein.synthesis.ribosomal protein.eukaryotic.40S subunit.S15	MDP0000322130	 moderately similar to ( 265) AT5G09510 | Symbols:  | 40S ribosomal protein S15 (RPS15D) | chr5:2955698-2956353 REVERSEmoderately similar to ( 266) RS15_ORYSA 40S ribosomal protein S15 - Oryza sativa (Rice)moderately similar to ( 269) loc_os03g58430 12003.m10751 protein 40S ribosomal protein S15, putative, expressed no original description 	T
30.2.17	signalling.receptor kinases.DUF 26	MDP0000207348	 highly similar to ( 879) AT5G51560 | Symbols:  | leucine-rich repeat transmembrane protein kinase, putative | chr5:20945807-20948613 FORWARDweakly similar to ( 172) PSKR_DAUCA Phytosulfokine receptor precursor (EC 2.7.11.1) (Phytosulfokine LRR receptor kinase) - Daucus carota (Carrot)highly similar to ( 736) loc_os06g04370 12006.m05166 protein ATP binding protein, putative, expressed  no original description 	T
30.2.17	signalling.receptor kinases.DUF 26	MDP0000086517	 highly similar to ( 812) AT4G27290 | Symbols:  | ATP binding / protein kinase/ protein serine/threonine kinase/ protein tyrosine kinase/ sugar binding | chr4:13666281-13669202 FORWARDmoderately similar to ( 339) SLSG3_BRAOL S-locus-specific glycoprotein S13 precursor (SLSG-13) (Fragment) - Brassica oleracea (Wild cabbage)highly similar to ( 736) loc_os07g36544 12007.m07905 protein serine/threonine-protein kinase receptor precursor, putative, expressed  MSP1_C  no original description 	T
30.2.24	signalling.receptor kinases.S-locus glycoprotein like	MDP0000086517	 highly similar to ( 812) AT4G27290 | Symbols:  | ATP binding / protein kinase/ protein serine/threonine kinase/ protein tyrosine kinase/ sugar binding | chr4:13666281-13669202 FORWARDmoderately similar to ( 339) SLSG3_BRAOL S-locus-specific glycoprotein S13 precursor (SLSG-13) (Fragment) - Brassica oleracea (Wild cabbage)highly similar to ( 736) loc_os07g36544 12007.m07905 protein serine/threonine-protein kinase receptor precursor, putative, expressed  MSP1_C  no original description 	T
17.3.1.2.99	hormone metabolism.brassinosteroid.synthesis-degradation.sterols.other	MDP0000161955	 highly similar to ( 699) AT4G37760 | Symbols: SQE3 | SQE3 (squalene epoxidase 3); squalene monooxygenase | chr4:17744085-17746426 FORWARDhighly similar to ( 744) ERG1_PANGI Squalene monooxygenase (EC 1.14.99.7) (Squalene epoxidase) (SE) - Panax ginseng (Korean ginseng)highly similar to ( 730) loc_os03g12910 12003.m101256 protein squalene monooxygenase, putative, expressed no original description 	T
35.2	not assigned.unknown	MDP0000928193	 moderately similar to ( 271) AT1G62520 | Symbols:  | unknown protein | chr1:23144506-23145348 FORWARDweakly similar to ( 117) loc_os02g44280 12002.m09469 protein conserved hypothetical protein  no original description 	T
34.99	transport.misc	MDP0000320308	 moderately similar to ( 469) AT1G60030 | Symbols: ATNAT7, NAT7 | xanthine/uracil permease family protein | chr1:22113993-22116648 REVERSEmoderately similar to ( 447) loc_os02g50820 12002.m10116 protein permease I, putative, expressed  no original description 	T
29.5.11.4.2	protein.degradation.ubiquitin.E3.RING	MDP0000228255	 weakly similar to ( 196) AT3G47550 | Symbols:  | zinc finger (C3HC4-type RING finger) family protein | chr3:17523841-17525278 FORWARDweakly similar to ( 192) loc_os09g24650 12009.m059975 protein protein binding protein, putative, expressed  no original description 	T
31.3	cell.cycle	MDP0000130413	 moderately similar to ( 341) AT5G43080 | Symbols: CYCA3;1 | CYCA3;1 (Cyclin A3;1); cyclin-dependent protein kinase regulator | chr5:17293227-17294789 FORWARDmoderately similar to ( 311) CCNAL_DAUCA G2/mitotic-specific cyclin C13-1 (A-like cyclin) (Fragment) - Daucus carota (Carrot)moderately similar to ( 322) loc_os03g41100 12003.m09178 protein cyclin-A2, putative, expressed  no original description 	T
35.2	not assigned.unknown	MDP0000232393	 very weakly similar to (58.2) AT4G28210 | Symbols: emb1923 | emb1923 (embryo defective 1923) | chr4:13990634-13991931 FORWARD  no original description 	T
35.2	not assigned.unknown	MDP0000186396	   no original description 	T
35.2	not assigned.unknown	MDP0000773013	 moderately similar to ( 447) AT5G19540 | Symbols:  | unknown protein | chr5:6595748-6597724 FORWARDmoderately similar to ( 407) loc_os08g29780 12008.m06980 protein expressed protein  no original description 	T
35.1	not assigned.no ontology	MDP0000211072	 highly similar to ( 641) AT3G50780 | Symbols:  | INVOLVED IN: biological_process unknown; LOCATED IN: cellular_component unknown; EXPRESSED IN: 22 plant structures; EXPRESSED DURING: 13 growth stages; BEST Arabidopsis thaliana protein match is: PRLI-interacting factor-related (TAIR:AT1G63850.1); Has 173 Blast hits to 173 proteins in 14 species: Archae - 0; Bacteria - 0; Metazoa - 8; Fungi - 0; Plants - 164; Viruses - 0; Other Eukaryotes - 1 (source: NCBI BLink). | chr3:18875662-18877341 REVERSEhighly similar to ( 573) loc_os03g55950 12003.m101512 protein protein binding protein, putative, expressed   no original description 	T
30.2.11	signalling.receptor kinases.leucine rich repeat XI	MDP0000190265	 nearly identical (1463) AT2G01950 | Symbols: VH1, BRL2 | BRL2 (BRI1-LIKE 2); ATP binding / protein serine/threonine kinase/ transmembrane receptor protein serine/threonine kinase | chr2:440805-444236 REVERSEmoderately similar to ( 488) PSKR_DAUCA Phytosulfokine receptor precursor (EC 2.7.11.1) (Phytosulfokine LRR receptor kinase) - Daucus carota (Carrot)nearly identical (1239) loc_os10g02500 12010.m03669 protein serine/threonine-protein kinase BRI1-like 2 precursor, putative, expressed   no original description 	T
35.1.5	not assigned.no ontology.pentatricopeptide (PPR) repeat-containing protein	MDP0000183819	 highly similar to ( 888) AT3G13770 | Symbols:  | pentatricopeptide (PPR) repeat-containing protein | chr3:4519647-4521533 FORWARDvery weakly similar to (85.9) RF1_ORYSA Rf1 protein, mitochondrial precursor (PPR protein) (Fertility restorer) (Restorer for CMS) - Oryza sativa (Rice)highly similar to ( 723) loc_os06g41040 12006.m08639 protein pentatricopeptide repeat protein PPR1106-17, putative, expressed  MSP1_C  no original description 	T
30.11	signalling.light	MDP0000293898	 moderately similar to ( 482) AT2G47590 | Symbols: PHR2 | PHR2 (photolyase/blue-light receptor 2); DNA photolyase | chr2:19521888-19523732 FORWARDweakly similar to ( 170) CRYD_ORYSA Cryptochrome DASH, chloroplast/mitochondrial precursor - Oryza sativa (Rice)moderately similar to ( 348) loc_os03g22330 12003.m07635 protein photolyase/blue-light receptor PHR2, putative, expressed  TrbL  no original description 	T
34.2.1	transport.sugars.sucrose	MDP0000275743	 highly similar to ( 627) AT1G09960 | Symbols: SUT4, ATSUT4, suc4 | SUT4 (SUCROSE TRANSPORTER 4); carbohydrate transmembrane transporter/ sucrose transmembrane transporter/ sucrose:hydrogen symporter/ sugar:hydrogen symporter | chr1:3244258-3246718 FORWARDhighly similar to ( 547) SUT2_ORYSA Sucrose transport protein 2 (Sucrose permease 2) (Sucrose-proton symporter 2) (Sucrose transporter 2) (SUC4-like protein) - Oryza sativa (Rice)highly similar to ( 547) loc_os12g44380 12012.m27000 protein sucrose transport protein SUC4, putative, expressed TrbL  no original description 	T
35.2	not assigned.unknown	MDP0000289649	 weakly similar to ( 107) AT1G52220 | Symbols:  | FUNCTIONS IN: molecular_function unknown; INVOLVED IN: biological_process unknown; LOCATED IN: chloroplast thylakoid membrane, chloroplast; EXPRESSED IN: 23 plant structures; EXPRESSED DURING: 13 growth stages; BEST Arabidopsis thaliana protein match is: PSI-P (PHOTOSYSTEM I P SUBUNIT); DNA binding (TAIR:AT2G46820.2); Has 172 Blast hits to 172 proteins in 28 species: Archae - 0; Bacteria - 44; Metazoa - 0; Fungi - 0; Plants - 127; Viruses - 0; Other Eukaryotes - 1 (source: NCBI BLink). | chr1:19453770-19454605 REVERSEvery weakly similar to (69.3) loc_os07g28610 12007.m29162 protein thylakoid membrane phosphoprotein 14 kda, chloroplast precursor, putative, expressed   no original description 	T
27.3.7	RNA.regulation of transcription.C2C2(Zn) CO-like, Constans-like zinc finger family	MDP0000140484	 moderately similar to ( 330) AT3G02380 | Symbols: COL2 | COL2 (constans-like 2); transcription factor/ zinc ion binding | chr3:487438-488624 REVERSEmoderately similar to ( 234) loc_os06g16370 12006.m06350 protein zinc finger protein CONSTANS, putative, expressed   no original description 	T
35.1	not assigned.no ontology	MDP0000160372	 moderately similar to ( 210) AT1G67360 | Symbols:  | rubber elongation factor (REF) family protein | chr1:25237072-25237913 REVERSEvery weakly similar to (99.8) Y5940_ORYSA Putative REF/SRPP-like protein Os05g0151300 - Oryza sativa (Rice)weakly similar to ( 103) loc_os07g47510 12007.m079784 protein stress-related protein, putative, expressed no original description 	T
29.3.3	protein.targeting.chloroplast	MDP0000302761	 moderately similar to ( 411) AT1G08980 | Symbols: ATAMI1, AMI1, ATTOC64-I | AMI1 (AMIDASE 1); amidase/ hydrolase, acting on carbon-nitrogen (but not peptide) bonds / indoleacetamide hydrolase | chr1:2884455-2886430 FORWARDmoderately similar to ( 362) loc_os04g02780 12004.m05591 protein ATAMI1, putative, expressed no original description 	T
29.5.5	protein.degradation.serine protease	MDP0000231032	 moderately similar to ( 297) AT2G29050 | Symbols: ATRBL1 | ATRBL1 (Arabidopsis thaliana Rhomboid-like 1) | chr2:12478245-12479653 FORWARDmoderately similar to ( 291) loc_os11g47840 12011.m08581 protein membrane protein, putative, expressed no original description 	T
31.3	cell.cycle	MDP0000319510	 weakly similar to ( 136) AT5G48630 | Symbols:  | cyclin family protein | chr5:19721663-19723200 REVERSEweakly similar to ( 134) CCNC_ORYSA G1/S-specific cyclin C-type - Oryza sativa (Rice)weakly similar to ( 134) loc_os09g32680 12009.m22154 protein cyclin-C, putative, expressed no original description 	T
35.1.5	not assigned.no ontology.pentatricopeptide (PPR) repeat-containing protein	MDP0000307555	 highly similar to ( 693) AT3G48810 | Symbols:  | pentatricopeptide (PPR) repeat-containing protein | chr3:18097048-18099027 FORWARDmoderately similar to ( 250) RF1_ORYSA Rf1 protein, mitochondrial precursor (PPR protein) (Fertility restorer) (Restorer for CMS) - Oryza sativa (Rice)highly similar to ( 546) loc_os05g04160 12005.m04949 protein conserved hypothetical protein   no original description 	T
31.1	cell.organisation	MDP0000157737	 highly similar to ( 718) AT3G12110 | Symbols: ACT11 | ACT11 (actin-11); structural constituent of cytoskeleton | chr3:3858116-3859609 FORWARDhighly similar to ( 724) ACT1_ORYSA Actin-1 - Oryza sativa (Rice)highly similar to ( 724) loc_os03g50890 12003.m10074 protein actin-1, putative, expressed   no original description 	T
35.1.5	not assigned.no ontology.pentatricopeptide (PPR) repeat-containing protein	MDP0000271659	 highly similar to ( 774) AT5G57870 | Symbols:  | eukaryotic translation initiation factor 4F, putative / eIF-4F, putative | chr5:23439755-23443433 FORWARDhighly similar to ( 792) IF41_WHEAT Eukaryotic initiation factor iso-4F subunit p82-34 (eIF-(iso)4F p82-34) - Triticum aestivum (Wheat)highly similar to ( 800) loc_os04g42140 12004.m09187 protein eukaryotic initiation factor iso-4F subunit p82-34, putative, expressed  no original description 	T
27.3.3	RNA.regulation of transcription.AP2/EREBP, APETALA2/Ethylene-responsive element binding protein family	MDP0000308922	 moderately similar to ( 281) AT1G53910 | Symbols: RAP2.12 | RAP2.12; DNA binding / transcription factor | chr1:20135242-20136581 FORWARDmoderately similar to ( 218) ERF1_ORYSA Ethylene-responsive transcription factor 1 (Ethylene-responsive element-binding factor 1) (EREBP-1) (OsEREBP1) - Oryza sativa (Rice)moderately similar to ( 218) loc_os02g54160 12002.m10445 protein ethylene response element binding protein, putative, expressed  no original description 	T
35.2	not assigned.unknown	MDP0000126450	 weakly similar to ( 151) AT5G39210 | Symbols: CRR7 | CRR7 (CHLORORESPIRATORY REDUCTION 7) | chr5:15703231-15703999 FORWARD no original description 	T
17.2.3	hormone metabolism.auxin.induced-regulated-responsive-activated	MDP0000621904	 highly similar to ( 675) AT2G44500 | Symbols:  | unknown protein | chr2:18374447-18376435 FORWARDmoderately similar to ( 236) loc_os12g08820 12012.m26858 protein auxin-independent growth promoter-like protein, putative, expressed   no original description 	T
1.1.1.1	PS.lightreaction.photosystem II.LHC-II	MDP0000150729	 moderately similar to ( 454) AT1G61520 | Symbols: LHCA3 | LHCA3; chlorophyll binding | chr1:22700493-22701149 FORWARDweakly similar to ( 154) CB12_PETHY Chlorophyll a-b binding protein, chloroplast precursor (LHCI type II CAB) - Petunia hybrida (Petunia)moderately similar to ( 409) loc_os02g10390 12002.m33354 protein chlorophyll a-b binding protein 8, chloroplast precursor, putative, expressed   no original description 	T
30.5	signalling.G-proteins	MDP0000301199	 moderately similar to ( 362) AT1G06400 | Symbols: ARA2, ATRABA1A, ATRAB11E, ARA-2 | ARA-2; GTP binding | chr1:1951089-1952686 REVERSEmoderately similar to ( 392) RB11D_LOTJA Ras-related protein Rab11D - Lotus japonicusmoderately similar to ( 356) loc_os06g35814 12006.m08118 protein ras-related protein RIC2, putative, expressed   no original description 	T
10.6.2	cell wall.degradation.mannan-xylose-arabinose-fucose	MDP0000417632	 highly similar to ( 665) AT2G20680 | Symbols:  | glycosyl hydrolase family 5 protein / cellulase family protein | chr2:8921024-8923066 FORWARDhighly similar to ( 518) loc_os01g54300 12001.m11594 protein hydrolase, hydrolyzing O-glycosyl compounds, putative, expressed  no original description 	T
35.2	not assigned.unknown	MDP0000233503	 moderately similar to ( 253) AT2G03350 | Symbols:  | unknown protein | chr2:1019733-1021071 REVERSEmoderately similar to ( 207) loc_os03g11210 12003.m06572 protein cp protein, putative, expressed  no original description 	T
27.1.19	RNA.processing.ribonucleases	MDP0000131645	 very weakly similar to ( 100) MAL12_MALDO Major allergen Mal d 1 (Mal d I) (AP15) - Malus domestica (Apple) (Malus sylvestris)  no original description 	T
29.4.1.57	protein.postranslational modification.kinase.receptor like cytoplasmatic kinase VII	MDP0000669809	 highly similar to ( 974) AT1G66150 | Symbols: TMK1 | TMK1 (TRANSMEMBRANE KINASE 1); transmembrane receptor protein serine/threonine kinase | chr1:24631503-24634415 FORWARDmoderately similar to ( 232) PSKR_DAUCA Phytosulfokine receptor precursor (EC 2.7.11.1) (Phytosulfokine LRR receptor kinase) - Daucus carota (Carrot)highly similar to ( 979) loc_os03g50810 12003.m10067 protein receptor protein kinase TMK1 precursor, putative, expressed TrbL  no original description 	T
35.2	not assigned.unknown	MDP0000220009	 weakly similar to ( 176) AT5G22090 | Symbols:  | unknown protein | chr5:7315003-7316394 REVERSEvery weakly similar to (97.1) loc_os03g49830 12003.m09981 protein expressed protein   TrbL PRK07003 PRK08026 COG5283 PRK07735  no original description 	T
35.1	not assigned.no ontology	MDP0000230239	 weakly similar to ( 162) AT1G19980 | Symbols:  | cytomatrix protein-related | chr1:6933162-6934643 FORWARDvery weakly similar to (52.4) loc_os04g02680 12004.m05581 protein expressed protein no original description 	T
30.2.17	signalling.receptor kinases.DUF 26	MDP0000252094	 highly similar to ( 793) AT1G60800 | Symbols: NIK3 | NIK3 (NSP-INTERACTING KINASE 3); kinase | chr1:22383601-22386931 REVERSEmoderately similar to ( 256) PSKR_DAUCA Phytosulfokine receptor precursor (EC 2.7.11.1) (Phytosulfokine LRR receptor kinase) - Daucus carota (Carrot)highly similar to ( 785) loc_os01g07630 12001.m42609 protein BRASSINOSTEROID INSENSITIVE 1-associated receptor kinase 1 precursor, putative, expressed   TrbL  no original description 	T
30.2.99	signalling.receptor kinases.misc	MDP0000252094	 highly similar to ( 793) AT1G60800 | Symbols: NIK3 | NIK3 (NSP-INTERACTING KINASE 3); kinase | chr1:22383601-22386931 REVERSEmoderately similar to ( 256) PSKR_DAUCA Phytosulfokine receptor precursor (EC 2.7.11.1) (Phytosulfokine LRR receptor kinase) - Daucus carota (Carrot)highly similar to ( 785) loc_os01g07630 12001.m42609 protein BRASSINOSTEROID INSENSITIVE 1-associated receptor kinase 1 precursor, putative, expressed   TrbL  no original description 	T
29.4.1.57	protein.postranslational modification.kinase.receptor like cytoplasmatic kinase VII	MDP0000252094	 highly similar to ( 793) AT1G60800 | Symbols: NIK3 | NIK3 (NSP-INTERACTING KINASE 3); kinase | chr1:22383601-22386931 REVERSEmoderately similar to ( 256) PSKR_DAUCA Phytosulfokine receptor precursor (EC 2.7.11.1) (Phytosulfokine LRR receptor kinase) - Daucus carota (Carrot)highly similar to ( 785) loc_os01g07630 12001.m42609 protein BRASSINOSTEROID INSENSITIVE 1-associated receptor kinase 1 precursor, putative, expressed   TrbL  no original description 	T
29.4.1.57	protein.postranslational modification.kinase.receptor like cytoplasmatic kinase VII	MDP0000258724	 highly similar to ( 563) AT2G28250 | Symbols: NCRK | NCRK; kinase | chr2:12044004-12046339 FORWARDmoderately similar to ( 206) PSKR_DAUCA Phytosulfokine receptor precursor (EC 2.7.11.1) (Phytosulfokine LRR receptor kinase) - Daucus carota (Carrot)moderately similar to ( 492) loc_os02g02040 12002.m33504 protein protein kinase, putative, expressed  no original description 	T
27.3.16	RNA.regulation of transcription.CCAAT box binding factor family, HAP5	MDP0000190029	 moderately similar to ( 222) AT1G56170 | Symbols: HAP5B, ATHAP5B, NF-YC2 | NF-YC2 (NUCLEAR FACTOR Y, SUBUNIT C2); DNA binding / transcription activator/ transcription factor | chr1:21025118-21025717 FORWARDmoderately similar to ( 227) loc_os02g07450 12002.m33577 protein nuclear transcription factor Y subunit C-2, putative, expressed  no original description 	T
35.2	not assigned.unknown	MDP0000346782	   no original description 	T
26.3.2	misc.gluco-, galacto- and mannosidases.beta-galactosidase	MDP0000030527	 nearly identical (1257) AT4G36360 | Symbols: BGAL3 | BGAL3 (beta-galactosidase 3); beta-galactosidase/ catalytic/ cation binding / sugar binding | chr4:17176840-17181143 REVERSEnearly identical (1094) BGAL_ASPOF Beta-galactosidase precursor (EC 3.2.1.23) (Lactase) - Asparagus officinalis (Garden asparagus)nearly identical (1235) loc_os03g06940 12003.m35030 protein beta-galactosidase precursor, putative, expressed no original description 	T
27.3.6	RNA.regulation of transcription.bHLH,Basic Helix-Loop-Helix family	MDP0000200355	 moderately similar to ( 209) AT4G34530 | Symbols: CIB1 | CIB1 (CRYPTOCHROME-INTERACTING BASIC-HELIX-LOOP-HELIX 1); DNA binding / transcription factor | chr4:16498466-16499946 FORWARDvery weakly similar to (50.4) PGIP1_ORYSA Polygalacturonase inhibitor 1 precursor (Polygalacturonase-inhibiting protein) (Floral organ regulator 1) - Oryza sativa (Rice)weakly similar to ( 174) loc_os01g68700 12001.m12972 protein BHLH transcription factor, putative, expressed  no original description 	T
10.6.3	cell wall.degradation.pectate lyases and polygalacturonases	MDP0000302503	 highly similar to ( 601) AT3G07010 | Symbols:  | pectate lyase family protein | chr3:2212973-2216306 REVERSEmoderately similar to ( 384) PEL_LILLO Pectate lyase precursor (EC 4.2.2.2) - Lilium longiflorum (Trumpet lily)highly similar to ( 572) loc_os04g05050 12004.m35243 protein pectate lyase 8 precursor, putative, expressed  no original description 	T
33.99	development.unspecified	MDP0000245757	 nearly identical (1058) AT3G13870 | Symbols: RHD3 | RHD3 (ROOT HAIR DEFECTIVE 3); GTP binding | chr3:4565762-4571109 REVERSEnearly identical (1007) loc_os01g39310 12001.m43066 protein protein SEY1, putative, expressed   no original description 	T
26.8	misc.nitrilases, *nitrile lyases, berberine bridge enzymes, reticuline oxidases, troponine reductases	MDP0000184463	 moderately similar to ( 291) AT4G05530 | Symbols: IBR1, SDRA | IBR1 (INDOLE-3-BUTYRIC ACID RESPONSE 1); binding / catalytic/ oxidoreductase | chr4:2816462-2818074 FORWARDmoderately similar to ( 232) loc_os09g04730 12009.m22042 protein dehydrogenase/reductase SDR family member 2, putative, expressed  no original description 	T
35.2	not assigned.unknown	MDP0000672208	 moderately similar to ( 233) AT4G21110 | Symbols:  | G10 family protein | chr4:11267673-11268774 FORWARDmoderately similar to ( 241) BD31A_ORYSA Protein BUD31 homolog 1 (Protein G10 homolog 1) - Oryza sativa (Rice)moderately similar to ( 241) loc_os04g55290 12004.m101674 protein protein G10 homolog 1, putative, expressed no original description 	T
10.8.2	cell wall.pectin*esterases.acetyl esterase	MDP0000869939	 moderately similar to ( 314) AT2G46930 | Symbols:  | pectinacetylesterase, putative | chr2:19283625-19286090 FORWARDmoderately similar to ( 291) loc_os07g44070 12007.m29010 protein carboxylic ester hydrolase, putative, expressed  no original description 	T
1.2.2	PS.photorespiration.glycolate oxydase	MDP0000265684	 highly similar to ( 615) AT3G14420 | Symbols:  | (S)-2-hydroxy-acid oxidase, peroxisomal, putative / glycolate oxidase, putative / short chain alpha-hydroxy acid oxidase, putative | chr3:4821899-4823899 FORWARDhighly similar to ( 621) GOX_SPIOL Peroxisomal (S)-2-hydroxy-acid oxidase (EC 1.1.3.15) (Glycolate oxidase) (GOX) (Short chain alpha-hydroxy acid oxidase) - Spinacia oleracea (Spinach)highly similar to ( 583) loc_os07g05820 12007.m29070 protein hydroxyacid oxidase 1, putative, expressed  no original description 	T
27.3.9	RNA.regulation of transcription.C2C2(Zn) GATA transcription factor family	MDP0000338280	 weakly similar to ( 131) AT3G54810 | Symbols: BME3-ZF, BME3 | zinc finger (GATA type) family protein | chr3:20296957-20298236 FORWARDweakly similar to ( 133) loc_os02g56250 12002.m10648 protein GATA zinc finger family protein, expressed  no original description 	T
29.2.4	protein.synthesis.elongation	MDP0000213603	 highly similar to ( 857) AT5G60390 | Symbols:  | elongation factor 1-alpha / EF-1-alpha | chr5:24289226-24290675 FORWARDhighly similar to ( 863) EF1A_MAIZE Elongation factor 1-alpha (EF-1-alpha) - Zea mays (Maize)highly similar to ( 862) loc_os03g08050 12003.m78808 protein elongation factor 1-alpha, putative, expressed   no original description 	T
35.2	not assigned.unknown	MDP0000819605	 weakly similar to ( 184) AT1G61260 | Symbols:  | unknown protein | chr1:22593756-22594986 REVERSEweakly similar to ( 169) loc_os07g37620 12007.m08012 protein fiber expressed protein, putative, expressed   no original description 	T
16.8.3.1	secondary metabolism.flavonoids.dihydroflavonols.dihydroflavonol 4-reductase	MDP0000322755	 moderately similar to ( 469) AT1G15950 | Symbols: CCR1, IRX4, ATCCR1 | CCR1 (CINNAMOYL COA REDUCTASE 1); cinnamoyl-CoA reductase | chr1:5478855-5481949 FORWARDweakly similar to ( 198) DFRA_DIACA Dihydroflavonol-4-reductase (EC 1.1.1.219) (DFR) (Dihydrokaempferol 4-reductase) - Dianthus caryophyllus (Carnation) (Clove pink)moderately similar to ( 448) loc_os09g25150 12009.m22089 protein dihydroflavonol-4-reductase, putative, expressed   no original description 	T
30.5	signalling.G-proteins	MDP0000519967	 moderately similar to ( 369) AT5G45750 | Symbols: AtRABA1c | AtRABA1c (Arabidopsis Rab GTPase homolog A1c); GTP binding | chr5:18559318-18560639 FORWARDmoderately similar to ( 370) RIC2_ORYSA Ras-related protein RIC2 - Oryza sativa (Rice)moderately similar to ( 370) loc_os06g35814 12006.m08118 protein ras-related protein RIC2, putative, expressed no original description 	T
29.4	protein.postranslational modification	MDP0000052605	 highly similar to ( 581) AT1G10940 | Symbols: ASK1, SNRK2-4, SNRK2.4, SRK2A | SNRK2.4 (SNF1-RELATED PROTEIN KINASE 2.4); kinase | chr1:3656050-3658170 REVERSEhighly similar to ( 565) SAPK7_ORYSA Serine/threonine-protein kinase SAPK7 (EC 2.7.11.1) (Osmotic stress/abscisic acid-activated protein kinase 7) - Oryza sativa (Rice)highly similar to ( 565) loc_os04g35240 12004.m08614 protein serine/threonine-protein kinase SAPK7, putative, expressed  no original description 	T
21.1	redox.thioredoxin	MDP0000317051	 moderately similar to ( 288) AT3G51040 | Symbols: RTH | RTH (RTE1-HOMOLOG) | chr3:18952281-18953060 REVERSEmoderately similar to ( 209) TRXH_RICCO Thioredoxin H-type (TRX-H) - Ricinus communis (Castor bean)moderately similar to ( 262) loc_os03g58520 12003.m10758 protein expressed protein no original description 	T
30.2.11	signalling.receptor kinases.leucine rich repeat XI	MDP0000188220	 moderately similar to ( 306) AT1G28340 | Symbols: AtRLP4 | AtRLP4 (Receptor Like Protein 4); protein binding | chr1:9940175-9943252 FORWARDvery weakly similar to (80.9) PSKR_DAUCA Phytosulfokine receptor precursor (EC 2.7.11.1) (Phytosulfokine LRR receptor kinase) - Daucus carota (Carrot)moderately similar to ( 260) loc_os07g31840 12007.m07450 protein protein binding protein, putative, expressed  no original description 	T
29.4	protein.postranslational modification	MDP0000222098	 highly similar to ( 705) AT4G15415 | Symbols: ATB' GAMMA | ATB' GAMMA; poly(U) binding / protein phosphatase type 2A regulator | chr4:8817707-8819349 FORWARDhighly similar to ( 653) loc_os03g62730 12003.m101566 protein transposon protein, putative, unclassified   no original description 	T
17.1.1.1.10	hormone metabolism.abscisic acid.synthesis-degradation.synthesis.9-cis-epoxycarotenoid dioxygenase	MDP0000547655	 highly similar to ( 804) AT4G19170 | Symbols: NCED4 | NCED4 (NINE-CIS-EPOXYCAROTENOID DIOXYGENASE 4) | chr4:10481835-10483622 FORWARDhighly similar to ( 674) loc_os02g47510 12002.m09785 protein carotenoid cleavage dioxygenase 1, putative, expressed  no original description 	T
26.21	misc.protease inhibitor/seed storage/lipid transfer protein (LTP) family protein	MDP0000887949	 very weakly similar to (77.4) AT5G48485 | Symbols: DIR1 | DIR1 (DEFECTIVE IN INDUCED RESISTANCE 1); lipid binding / lipid transporter | chr5:19646317-19646625 REVERSEvery weakly similar to (89.7) loc_os07g18750 12007.m06314 protein PVR3-like protein, putative, expressed  no original description 	T
35.2	not assigned.unknown	MDP0000301602	 moderately similar to ( 283) AT2G02590 | Symbols:  | FUNCTIONS IN: molecular_function unknown; INVOLVED IN: biological_process unknown; LOCATED IN: chloroplast; EXPRESSED IN: 21 plant structures; EXPRESSED DURING: 13 growth stages; CONTAINS InterPro DOMAIN/s: Putative small multi-drug export (InterPro:IPR009577); Has 213 Blast hits to 213 proteins in 89 species: Archae - 35; Bacteria - 146; Metazoa - 0; Fungi - 0; Plants - 15; Viruses - 0; Other Eukaryotes - 17 (source: NCBI BLink). | chr2:704977-706494 FORWARDmoderately similar to ( 249) loc_os03g58620 12003.m10767 protein small multi-drug export protein, putative, expressed   no original description 	T
29.5.11.4.2	protein.degradation.ubiquitin.E3.RING	MDP0000264961	 very weakly similar to (87.0) AT5G20885 | Symbols:  | zinc finger (C3HC4-type RING finger) family protein | chr5:7084133-7084663 REVERSEvery weakly similar to (53.9) EL5_ORYSA E3 ubiquitin-protein ligase EL5 (EC 6.3.2.-) - Oryza sativa (Rice)very weakly similar to (80.9) loc_os02g45780 12002.m09615 protein protein binding protein, putative, expressed   no original description 	T
29.4.1.57	protein.postranslational modification.kinase.receptor like cytoplasmatic kinase VII	MDP0000252465	 nearly identical (1075) AT5G13300 | Symbols: SFC, VAN3, AGD3 | SFC (SCARFACE); ARF GTPase activator/ phosphoinositide binding | chr5:4255923-4262018 REVERSEweakly similar to ( 134) NORK_PEA Nodulation receptor kinase precursor (EC 2.7.11.1) - Pisum sativum (Garden pea)highly similar to ( 981) loc_os09g33600 12009.m06409 protein VAN3, putative, expressed  no original description 	T
18.1	Co-factor and vitamine metabolism.molybdenum cofactor	MDP0000245282	 moderately similar to ( 381) AT5G55130 | Symbols: CNX5, SIR1 | CNX5 (CO-FACTOR FOR NITRATE, REDUCTASE AND XANTHINE DEHYDROGENASE 5); Mo-molybdopterin cofactor sulfurase | chr5:22373374-22376028 REVERSEmoderately similar to ( 205) loc_os02g56050 12002.m10629 protein molybdenum cofactor synthesis protein 3, putative, expressed  no original description 	T
31.3	cell.cycle	MDP0000304147	 moderately similar to ( 449) AT1G20610 | Symbols: CYCB2;3 | CYCB2;3 (Cyclin B2;3); cyclin-dependent protein kinase regulator | chr1:7135073-7137273 REVERSEmoderately similar to ( 427) CCNB2_MEDSA G2/mitotic-specific cyclin-2 (B-like cyclin) (CycMs2) (Fragment) - Medicago sativa (Alfalfa)moderately similar to ( 412) loc_os06g51110 12006.m31938 protein cyclin IIIZm, putative, expressed  MSP1_C  no original description 	T
29.2.1.1.1.1.530	protein.synthesis.ribosomal protein.prokaryotic.chloroplast.30S subunit.S30A	MDP0000398785	 moderately similar to ( 297) AT5G24490 | Symbols:  | 30S ribosomal protein, putative | chr5:8365690-8367178 FORWARDmoderately similar to ( 291) RR30_SPIOL Plastid-specific 30S ribosomal protein 1, chloroplast precursor (PSRP-1) (CS-S5) (CS5) (S22) (Ribosomal protein 1) - Spinacia oleracea (Spinach)moderately similar to ( 225) loc_os03g63950 12003.m35647 protein plastid-specific 30S ribosomal protein 1, chloroplast precursor, putative, expressed no original description 	T
35.2	not assigned.unknown	MDP0000923601	  no original description 	T
35.2	not assigned.unknown	MDP0000815097	 weakly similar to ( 117) AT2G46150 | Symbols:  | FUNCTIONS IN: molecular_function unknown; INVOLVED IN: biological_process unknown; LOCATED IN: plasma membrane; EXPRESSED IN: 15 plant structures; EXPRESSED DURING: 7 growth stages; CONTAINS InterPro DOMAIN/s: Harpin-induced 1 (InterPro:IPR010847); BEST Arabidopsis thaliana protein match is: unknown protein (TAIR:AT3G54200.1); Has 137 Blast hits to 136 proteins in 12 species: Archae - 0; Bacteria - 0; Metazoa - 0; Fungi - 4; Plants - 133; Viruses - 0; Other Eukaryotes - 0 (source: NCBI BLink). | chr2:18961424-18962089 FORWARDvery weakly similar to (90.5) loc_os01g53470 12001.m11515 protein harpin-induced protein, putative, expressed  no original description 	T
11.1.13	lipid metabolism.FA synthesis and FA elongation.acyl-CoA binding protein	MDP0000310834	 highly similar to ( 929) AT3G05420 | Symbols: ACBP4 | ACBP4 (ACYL-COA BINDING PROTEIN 4); acyl-CoA binding | chr3:1561880-1567047 FORWARDvery weakly similar to (85.1) DYHA_CHLRE Dynein alpha chain, flagellar outer arm (DHC alpha) - Chlamydomonas reinhardtiihighly similar to ( 795) loc_os03g61930 12003.m35507 protein acyl-CoA binding protein, putative, expressed   no original description 	T
29.5.3	protein.degradation.cysteine protease	MDP0000131656	 moderately similar to ( 379) AT4G15880 | Symbols: ESD4 | ESD4 (EARLY IN SHORT DAYS 4); SUMO-specific protease/ cysteine-type peptidase | chr4:9012769-9015797 FORWARDmoderately similar to ( 271) loc_os01g25370 12001.m43010 protein SUMO protease, putative, expressed  no original description 	T
28.2	DNA.repair	MDP0000184480	 moderately similar to ( 224) AT2G24490 | Symbols: RPA2, ATRPA2, ROR1 | RPA2 (REPLICON PROTEIN A2); protein binding | chr2:10398651-10400416 REVERSEweakly similar to ( 164) loc_os02g42230 12002.m09260 protein replication factor A, putative, expressed  no original description 	T
13.1.4.1	amino acid metabolism.synthesis.branched chain group.common	MDP0000552989	 highly similar to ( 639) AT3G58610 | Symbols:  | ketol-acid reductoisomerase | chr3:21671561-21674639 FORWARDhighly similar to ( 642) ILV5_PEA Ketol-acid reductoisomerase, chloroplast precursor (EC 1.1.1.86) (Acetohydroxy-acid reductoisomerase) (Alpha-keto-beta-hydroxylacil reductoisomerase) - Pisum sativum (Garden pea)highly similar to ( 632) loc_os05g49800 12005.m09050 protein ketol-acid reductoisomerase, chloroplast precursor, putative, expressed  no original description 	T
26.12	misc.peroxidases	MDP0000208152	 moderately similar to ( 422) AT5G06720 | Symbols:  | peroxidase, putative | chr5:2077567-2078857 REVERSEmoderately similar to ( 368) PERX_TOBAC Lignin-forming anionic peroxidase precursor (EC 1.11.1.7) (TOPA) - Nicotiana tabacum (Common tobacco)moderately similar to ( 381) loc_os03g13200 12003.m06766 protein peroxidase 53 precursor, putative, expressed  no original description 	T
27.3.6	RNA.regulation of transcription.bHLH,Basic Helix-Loop-Helix family	MDP0000320739	 moderately similar to ( 298) AT5G42940 | Symbols:  | zinc finger (C3HC4-type RING finger) family protein | chr5:17216649-17219171 REVERSEvery weakly similar to (52.8) ARRS_MAIZE Anthocyanin regulatory R-S protein - Zea mays (Maize)moderately similar to ( 242) loc_os04g55510 12004.m10442 protein zinc finger, C3HC4 type family protein, expressed  no original description 	T
27.3.25	RNA.regulation of transcription.MYB domain transcription factor family	MDP0000578193	 moderately similar to ( 222) AT5G14750 | Symbols: WER, ATMYB66, WER1 | ATMYB66 (MYB DOMAIN PROTEIN 66); DNA binding / protein binding / transcription factor/ transcription regulator | chr5:4763656-4764738 REVERSEweakly similar to ( 163) MYBC_MAIZE Anthocyanin regulatory C1 protein - Zea mays (Maize)weakly similar to ( 164) loc_os06g10350 12006.m05753 protein anthocyanin regulatory C1 protein, putative, expressed   no original description 	T
29.5.11.4.3.2	protein.degradation.ubiquitin.E3.SCF.FBOX	MDP0000162018	 highly similar to ( 643) AT5G21040 | Symbols: FBX2 | FBX2 (F-BOX PROTEIN 2) | chr5:7145058-7146677 REVERSEvery weakly similar to (60.8) PF20_CHLRE Flagellar WD repeat protein PF20 - Chlamydomonas reinhardtiihighly similar to ( 538) loc_os04g52870 12004.m10180 protein F-box domain containing protein, expressed   no original description 	T
35.2	not assigned.unknown	MDP0000341605	  no original description 	T
35.1	not assigned.no ontology	MDP0000143864	 moderately similar to ( 314) AT4G15470 | Symbols:  | EXPRESSED IN: 24 plant structures; EXPRESSED DURING: 15 growth stages; CONTAINS InterPro DOMAIN/s: Uncharacterised protein family UPF0005 (InterPro:IPR006214); BEST Arabidopsis thaliana protein match is: glutamate binding (TAIR:AT1G03070.1); Has 4000 Blast hits to 3999 proteins in 955 species: Archae - 0; Bacteria - 1775; Metazoa - 750; Fungi - 92; Plants - 143; Viruses - 77; Other Eukaryotes - 1163 (source: NCBI BLink). | chr4:8843661-8845505 FORWARDvery weakly similar to (53.1) BI1_ORYSA Bax inhibitor 1 (BI-1) (OsBI-1) - Oryza sativa (Rice)moderately similar to ( 275) loc_os05g33360 12005.m27816 protein transmembrane BAX inhibitor motif-containing protein 4, putative, expressed  no original description 	T
35.1.5	not assigned.no ontology.pentatricopeptide (PPR) repeat-containing protein	MDP0000234257	 nearly identical (1758) AT3G52140 | Symbols:  | tetratricopeptide repeat (TPR)-containing protein | chr3:19333232-19341295 FORWARDnearly identical (1548) loc_os02g48620 12002.m33838 protein expressed protein  no original description 	T
27.4	RNA.RNA binding	MDP0000143543	 weakly similar to ( 195) AT3G01210 | Symbols:  | nucleic acid binding / oxidoreductase | chr3:72182-73159 FORWARDmoderately similar to ( 209) loc_os04g54870 12004.m10379 protein RNA recognition motif containing protein, putative, expressed no original description 	T
23.4.10	nucleotide metabolism.phosphotransfer and pyrophosphatases.nucleoside diphosphate kinase	MDP0000169163	 moderately similar to ( 331) AT4G11010 | Symbols: NDPK3 | NDPK3 (NUCLEOSIDE DIPHOSPHATE KINASE 3); ATP binding / nucleoside diphosphate kinase | chr4:6732780-6734298 REVERSEmoderately similar to ( 314) NDK4_SPIOL Nucleoside diphosphate kinase 4, chloroplast precursor (EC 2.7.4.6) (Nucleoside diphosphate kinase IV) (NDK IV) (NDP kinase IV) (NDPK IV) (Nucleoside diphosphate kinase III) - Spinacia oleracea (Spinach)moderately similar to ( 294) loc_os05g51700 12005.m083838 protein nucleoside diphosphate kinase 4, chloroplast precursor, putative, expressed  no original description 	T
35.2	not assigned.unknown	MDP0000334067	 weakly similar to ( 187) AT1G13380 | Symbols:  | unknown protein | chr1:4589218-4590362 REVERSEweakly similar to ( 187) loc_os09g10910 12009.m059949 protein fiber protein Fb34, putative, expressed no original description 	T
27.1	RNA.processing	MDP0000367870	 weakly similar to ( 143) AT3G11500 | Symbols:  | small nuclear ribonucleoprotein G, putative / snRNP-G, putative / Sm protein G, putative | chr3:3621626-3622474 REVERSEweakly similar to ( 133) RUXG_MEDSA Probable small nuclear ribonucleoprotein G (snRNP-G) (Sm protein G) (Sm-G) (SmG) - Medicago sativa (Alfalfa)weakly similar to ( 142) loc_os07g41790 12007.m079746 protein small nuclear ribonucleoprotein G, putative, expressed  no original description 	T
30.2.99	signalling.receptor kinases.misc	MDP0000196862	 highly similar to ( 800) AT1G60800 | Symbols: NIK3 | NIK3 (NSP-INTERACTING KINASE 3); kinase | chr1:22383601-22386931 REVERSEmoderately similar to ( 270) PSKR_DAUCA Phytosulfokine receptor precursor (EC 2.7.11.1) (Phytosulfokine LRR receptor kinase) - Daucus carota (Carrot)highly similar to ( 792) loc_os01g07630 12001.m42609 protein BRASSINOSTEROID INSENSITIVE 1-associated receptor kinase 1 precursor, putative, expressed  no original description 	T
29.4.1.57	protein.postranslational modification.kinase.receptor like cytoplasmatic kinase VII	MDP0000196862	 highly similar to ( 800) AT1G60800 | Symbols: NIK3 | NIK3 (NSP-INTERACTING KINASE 3); kinase | chr1:22383601-22386931 REVERSEmoderately similar to ( 270) PSKR_DAUCA Phytosulfokine receptor precursor (EC 2.7.11.1) (Phytosulfokine LRR receptor kinase) - Daucus carota (Carrot)highly similar to ( 792) loc_os01g07630 12001.m42609 protein BRASSINOSTEROID INSENSITIVE 1-associated receptor kinase 1 precursor, putative, expressed  no original description 	T
35.2	not assigned.unknown	MDP0000504522	 very weakly similar to (94.7) AT5G56550 | Symbols: OXS3, ATOXS3 | OXS3 (OXIDATIVE STRESS 3) | chr5:22895847-22896502 REVERSEvery weakly similar to (85.5) loc_os02g45930 12002.m09630 protein expressed protein no original description 	T
30.11	signalling.light	MDP0000220752	 moderately similar to ( 365) AT4G38180 | Symbols: FRS5 | FRS5 (FAR1-related sequence 5); zinc ion binding | chr4:17906702-17909404 REVERSEmoderately similar to ( 436) loc_os06g07010 12006.m05426 protein transposon protein, putative, unclassified, expressed   no original description 	T
35.2	not assigned.unknown	MDP0000299801	 weakly similar to ( 158) AT2G16900 | Symbols:  | FUNCTIONS IN: molecular_function unknown; INVOLVED IN: biological_process unknown; LOCATED IN: plasma membrane; EXPRESSED IN: 24 plant structures; EXPRESSED DURING: 13 growth stages; CONTAINS InterPro DOMAIN/s: Phospholipase-like, arabidopsis (InterPro:IPR007942); BEST Arabidopsis thaliana protein match is: unknown protein (TAIR:AT4G35110.4); Has 280 Blast hits to 196 proteins in 46 species: Archae - 2; Bacteria - 4; Metazoa - 40; Fungi - 6; Plants - 96; Viruses - 48; Other Eukaryotes - 84 (source: NCBI BLink). | chr2:7323857-7324886 REVERSEvery weakly similar to (53.1) loc_os06g49740 12006.m32188 protein GCIP-interacting protein, putative, expressed no original description 	T
10.1.10	cell wall.precursor synthesis.UDP-glucose 4,6-dehydratase	MDP0000179744	 nearly identical (1132) AT1G78570 | Symbols: RHM1, ROL1, ATRHM1 | RHM1 (RHAMNOSE BIOSYNTHESIS 1); UDP-L-rhamnose synthase/ UDP-glucose 4,6-dehydratase/ catalytic | chr1:29550110-29552207 FORWARDvery weakly similar to (97.8) ARAE2_ORYSA Probable UDP-arabinose 4-epimerase 2 (EC 5.1.3.5) (UDP-D-xylose 4-epimerase 2) (UDP-galactose 4-epimerase-like protein 2) (OsUEL-2) - Oryza sativa (Rice)nearly identical (1108) loc_os03g17000 12003.m35163 protein RHM1, putative, expressed   no original description 	T
29.4.1.57	protein.postranslational modification.kinase.receptor like cytoplasmatic kinase VII	MDP0000291093	 highly similar to ( 882) AT4G33430 | Symbols: BAK1, RKS10, SERK3, ELG, ATSERK3, ATBAK1 | BAK1 (BRI1-ASSOCIATED RECEPTOR KINASE); kinase/ protein binding / protein heterodimerization/ protein serine/threonine kinase | chr4:16086654-16090288 REVERSEmoderately similar to ( 226) NORK_PEA Nodulation receptor kinase precursor (EC 2.7.11.1) - Pisum sativum (Garden pea)highly similar to ( 758) loc_os04g38480 12004.m35325 protein BRASSINOSTEROID INSENSITIVE 1-associated receptor kinase 1 precursor, putative, expressed   no original description 	T
26.10	misc.cytochrome P450	MDP0000678982	 highly similar to ( 777) AT1G11680 | Symbols: CYP51G1, EMB1738, CYP51A2, CYP51 | CYP51G1 (CYTOCHROME P450 51G1); oxygen binding / sterol 14-demethylase | chr1:3938925-3940585 FORWARDhighly similar to ( 729) CP51_SORBI Cytochrome P450 51 (EC 1.14.13.70) (CYPLI) (P450-LIA1) (Obtusifoliol 14-alpha demethylase) - Sorghum bicolor (Sorghum) (Sorghum vulgare)highly similar to ( 724) loc_os11g32240 12011.m07126 protein cytochrome P450 51, putative, expressed  no original description 	T
21.2.2	redox.ascorbate and glutathione.glutathione	MDP0000243843	 moderately similar to ( 268) AT4G11600 | Symbols: ATGPX6, PHGPX, LSC803 | ATGPX6 (GLUTATHIONE PEROXIDASE 6); glutathione peroxidase | chr4:7010021-7011330 REVERSEmoderately similar to ( 265) GPX4_CITSI Probable phospholipid hydroperoxide glutathione peroxidase (EC 1.11.1.12) (PHGPx) (Salt-associated protein) - Citrus sinensis (Sweet orange)moderately similar to ( 272) loc_os04g46960 12004.m101614 protein phospholipid hydroperoxide glutathione peroxidase, putative, expressed no original description 	T
27.3.22	RNA.regulation of transcription.HB,Homeobox transcription factor family	MDP0000313776	 moderately similar to ( 445) AT1G62990 | Symbols: KNAT7, IXR11 | KNAT7 (KNOTTED-LIKE HOMEOBOX OF ARABIDOPSIS THALIANA 7); DNA binding / transcription activator/ transcription factor | chr1:23337468-23340348 FORWARDmoderately similar to ( 449) HD1_BRANA Homeobox protein HD1 - Brassica napus (Rape)moderately similar to ( 410) loc_os03g03164 12003.m35560 protein homeobox protein HD1, putative, expressed  MSP1_C  no original description 	T
18.5.2.5	Co-factor and vitamine metabolism.folate & vitamine K.vitamine K.naphthoate synthase	MDP0000797914	 highly similar to ( 518) AT1G60550 | Symbols: ECHID, DHNS | ECHID (ENOYL-COA HYDRATASE/ISOMERASE D); catalytic/ naphthoate synthase | chr1:22305988-22308092 REVERSEvery weakly similar to (70.1) MFP_ORYSA Peroxisomal fatty acid beta-oxidation multifunctional protein (MFP) [Includes: Enoyl-CoA hydratase (EC 4.2.1.17); 3-2-trans-enoyl-CoA isomerase (EC 5.3.3.8); 3-hydroxybutyryl-CoA epimerase (EC 5.1.2.3); 3-hydroxyacyl-CoA dehydrogenase (Emoderately similar to ( 499) loc_os01g47350 12001.m10930 protein naphthoate synthase, putative, expressed   no original description 	T
26.2	misc.UDP glucosyl and glucoronyl transferases	MDP0000142939	 highly similar to ( 578) AT3G07620 | Symbols:  | exostosin family protein | chr3:2433267-2434988 REVERSEmoderately similar to ( 479) loc_os07g09050 12007.m28953 protein EXO, putative, expressed  no original description 	T
35.1	not assigned.no ontology	MDP0000313132	 weakly similar to ( 158) AT5G22870 | Symbols:  | harpin-induced protein-related / HIN1-related / harpin-responsive protein-related | chr5:7647056-7647679 REVERSEvery weakly similar to (92.4) loc_os02g44670 12002.m09507 protein harpin-induced 1, putative, expressed no original description 	T
35.2	not assigned.unknown	MDP0000453458	  no original description 	T
29.5.11.4.2	protein.degradation.ubiquitin.E3.RING	MDP0000422586	 moderately similar to ( 439) AT1G02860 | Symbols: NLA | NLA (nitrogen limitation adaptation); ubiquitin-protein ligase | chr1:635474-637083 FORWARDmoderately similar to ( 445) loc_os07g47590 12007.m29409 protein expressed protein  no original description 	T
29.1.3	protein.aa activation.threonine-tRNA ligase	MDP0000261961	 nearly identical (1049) AT2G04842 | Symbols: EMB2761 | EMB2761 (EMBRYO DEFECTIVE 2761); ATP binding / aminoacyl-tRNA ligase/ ligase, forming aminoacyl-tRNA and related compounds / nucleotide binding / threonine-tRNA ligase | chr2:1698466-1701271 REVERSEnearly identical (1015) loc_os02g33500 12002.m33712 protein threonyl-tRNA synthetase, putative, expressed  no original description 	T
35.1	not assigned.no ontology	MDP0000167873	 moderately similar to ( 263) AT3G02700 | Symbols:  | NC domain-containing protein | chr3:581727-582813 REVERSEmoderately similar to ( 253) loc_os05g24560 12005.m06750 protein NC domain containing protein, expressed  no original description 	T
18.11	Co-factor and vitamine metabolism.lipoic acid	MDP0000284215	 highly similar to ( 544) AT5G08415 | Symbols:  | lipoic acid synthase family protein | chr5:2710983-2713101 REVERSEmoderately similar to ( 489) loc_os05g43576 12005.m27468 protein lipoic acid synthetase, mitochondrial precursor, putative, expressed   no original description 	T
34.14	transport.unspecified cations	MDP0000177842	 moderately similar to ( 357) AT2G03620 | Symbols:  | magnesium transporter CorA-like family protein (MRS2-5) | chr2:1100489-1102168 REVERSEmoderately similar to ( 304) loc_os06g44150 12006.m08943 protein MRS2-10, putative, expressed no original description 	T
35.2	not assigned.unknown	MDP0000916647	   no original description 	T
11.1.6	lipid metabolism.FA synthesis and FA elongation.enoyl ACP reductase	MDP0000350866	 moderately similar to ( 481) AT2G05990 | Symbols: MOD1, ENR1 | MOD1 (MOSAIC DEATH 1); enoyl-[acyl-carrier-protein] reductase (NADH)/ enoyl-[acyl-carrier-protein] reductase/ oxidoreductase | chr2:2322876-2324867 FORWARDmoderately similar to ( 474) FABI_BRANA Enoyl-[acyl-carrier-protein] reductase [NADH], chloroplast precursor (EC 1.3.1.9) (NADH-dependent enoyl-ACP reductase) - Brassica napus (Rape)moderately similar to ( 476) loc_os08g23810 12008.m26662 protein enoyl-[acyl-carrier-protein] reductase [NADH], chloroplast precursor, expressed no original description 	T
31.1	cell.organisation	MDP0000382436	 highly similar to ( 843) AT5G48460 | Symbols:  | fimbrin-like protein, putative | chr5:19636225-19639365 REVERSEhighly similar to ( 714) loc_os02g48740 12002.m09908 protein fimbrin 1, putative, expressed   no original description 	T
26.28	misc.GDSL-motif lipase	MDP0000468056	 moderately similar to ( 477) AT2G32900 | Symbols: ATZW10 | ATZW10 | chr2:13953807-13957470 FORWARDweakly similar to ( 167) APG_BRANA Anter-specific proline-rich protein APG (Protein CEX) (Fragment) - Brassica napus (Rape)moderately similar to ( 309) loc_os06g43044 12006.m08834 protein anther-specific proline-rich protein APG, putative, expressed    TrbL  no original description 	T
27.3.32	RNA.regulation of transcription.WRKY domain transcription factor family	MDP0000455180	 weakly similar to ( 132) AT4G23810 | Symbols: WRKY53, ATWRKY53 | WRKY53; DNA binding / protein binding / transcription activator/ transcription factor | chr4:12392666-12393739 REVERSEweakly similar to ( 122) loc_os08g29660 12008.m06969 protein OsWRKY69 - Superfamily of rice TFs having WRKY and zinc finger domains, expressed no original description 	T
29.2.1.2.1.29	protein.synthesis.ribosomal protein.eukaryotic.40S subunit.S29	MDP0000428281	 weakly similar to ( 114) AT4G33865 | Symbols:  | 40S ribosomal protein S29 (RPS29C) | chr4:16233395-16234114 REVERSEweakly similar to ( 121) loc_os12g32380 12012.m07041 protein 40S ribosomal protein S29, putative, expressed  no original description 	T
29.5.11.1	protein.degradation.ubiquitin.ubiquitin	MDP0000253224	 moderately similar to ( 255) AT3G52590 | Symbols: UBQ1, EMB2167, ERD16, HAP4 | UBQ1 (UBIQUITIN EXTENSION PROTEIN 1); protein binding / structural constituent of ribosome | chr3:19505668-19506681 FORWARDweakly similar to ( 150) UBIQ_WHEAT Ubiquitin - Triticum aestivum (Wheat)moderately similar to ( 255) loc_os09g39500 12009.m60242 protein ubiquitin fusion protein, putative, expressed no original description 	T
29.2.4	protein.synthesis.elongation	MDP0000163248	 highly similar to ( 771) AT4G02930 | Symbols:  | elongation factor Tu, putative / EF-Tu, putative | chr4:1295751-1298354 REVERSEhighly similar to ( 535) EFTUB_NICSY Elongation factor TuB, chloroplast precursor (EF-TuB) - Nicotiana sylvestris (Wood tobacco)highly similar to ( 735) loc_os03g63410 12003.m34958 protein elongation factor Tu, mitochondrial precursor, putative, expressed  no original description 	T
29.4	protein.postranslational modification	MDP0000262273	 nearly identical (1067) AT1G04210 | Symbols:  | leucine-rich repeat family protein / protein kinase family protein | chr1:1114696-1119383 FORWARDvery weakly similar to (60.1) RPK1_IPONI Receptor-like protein kinase precursor (EC 2.7.11.1) - Ipomoea nil (Japanese morning glory) (Pharbitis nil)highly similar to ( 639) loc_os03g53250 12003.m10298 protein protein kinase domain containing protein, expressed  no original description 	T
35.2	not assigned.unknown	MDP0000686092	 moderately similar to ( 235) AT4G31980 | Symbols:  | unknown protein | chr4:15464905-15469204 FORWARDweakly similar to ( 149) loc_os01g21710 12001.m08655 protein conserved hypothetical protein  no original description 	T
35.2	not assigned.unknown	MDP0000394035	  no original description 	T
13.2.4.1	amino acid metabolism.degradation.branched chain group.shared	MDP0000129664	 highly similar to ( 696) AT2G33150 | Symbols: PKT3, PED1, KAT2 | PKT3 (PEROXISOMAL 3-KETOACYL-COA THIOLASE 3); acetyl-CoA C-acyltransferase | chr2:14047814-14050983 REVERSEhighly similar to ( 627) loc_os02g57260 12002.m10748 protein 3-ketoacyl-CoA thiolase 2, peroxisomal precursor, putative, expressed  no original description 	T
29.5.11.20	protein.degradation.ubiquitin.proteasom	MDP0000291481	 moderately similar to ( 428) AT2G27020 | Symbols: PAG1 | PAG1; endopeptidase/ peptidase/ threonine-type endopeptidase | chr2:11528515-11530858 REVERSEmoderately similar to ( 435) PSA3_SPIOL Proteasome subunit alpha type 3 (EC 3.4.25.1) (20S proteasome alpha subunit G) (20S proteasome subunit alpha-7) (Proteasome component C8) - Spinacia oleracea (Spinach)moderately similar to ( 422) loc_os01g59600 12001.m43238 protein proteasome subunit alpha type 3, putative, expressed no original description 	T
35.1.12	not assigned.no ontology.pumilio/Puf RNA-binding domain-containing protein	MDP0000768248	 highly similar to ( 627) AT3G16810 | Symbols: APUM24 | APUM24 (Arabidopsis Pumilio 24); RNA binding / binding | chr3:5723436-5727539 REVERSEhighly similar to ( 512) loc_os03g61560 12003.m11037 protein expressed protein  no original description 	T
27.3.47	RNA.regulation of transcription.ELF3	MDP0000127365	 moderately similar to ( 372) AT2G25930 | Symbols: ELF3, PYK20 | ELF3 (EARLY FLOWERING 3); protein C-terminus binding / transcription factor | chr2:11059459-11063178 FORWARDmoderately similar to ( 269) loc_os01g38530 12001.m10136 protein early flowering 3, putative, expressed  no original description 	T
29.7	protein.glycosylation	MDP0000163119	 highly similar to ( 549) AT2G41490 | Symbols: GPT | GPT (UDP-GLCNAC%3ADOLICHOL+PHOSPHATE+GLCNAC-1-P+TRANSFERASE); UDP-N-acetylglucosamine-dolichyl-phosphate N-acetylglucosaminephosphotransferase | chr2:17301762-17304030 FORWARDmoderately similar to ( 472) loc_os07g46640 12007.m08881 protein UDP-N-acetylglucosamine--dolichyl-phosphate N-acetylglucosaminephosphotransferase, putative, expressed  no original description 	T
34.11	transport.NDP-sugars at the ER	MDP0000189783	 highly similar to ( 503) AT1G12600 | Symbols:  | FUNCTIONS IN: molecular_function unknown; INVOLVED IN: biological_process unknown; LOCATED IN: cellular_component unknown; EXPRESSED IN: leaf whorl, petal, male gametophyte, flower, pollen tube; EXPRESSED DURING: L mature pollen stage, 4 anthesis, petal differentiation and expansion stage; CONTAINS InterPro DOMAIN/s: UAA transporter (InterPro:IPR013657); BEST Arabidopsis thaliana protein match is: UDP-galactose transporter-related (TAIR:AT4G23010.1); Has 943 Blast hits to 943 proteins in 196 species: Archae - 2; Bacteria - 55; Metazoa - 506; Fungi - 148; Plants - 112; Viruses - 0; Other Eukaryotes - 120 (source: NCBI BLink). | chr1:4286414-4288859 REVERSEmoderately similar to ( 477) loc_os03g02670 12003.m34768 protein solute carrier family 35 member B3, putative, expressed  no original description 	T
34.1	transport.p- and v-ATPases	MDP0000330134	 highly similar to ( 568) AT4G39080 | Symbols: VHA-A3 | VHA-A3 (VACUOLAR PROTON ATPASE A3); ATPase | chr4:18209513-18214752 FORWARDhighly similar to ( 559) loc_os03g14690 12003.m06907 protein vacuolar ATP synthase 91 kDa subunit, putative, expressed no original description 	T
35.2	not assigned.unknown	MDP0000845594	 weakly similar to ( 186) AT3G53850 | Symbols:  | FUNCTIONS IN: molecular_function unknown; INVOLVED IN: biological_process unknown; LOCATED IN: endomembrane system; CONTAINS InterPro DOMAIN/s: Uncharacterised protein family UPF0497, trans-membrane plant (InterPro:IPR006702); BEST Arabidopsis thaliana protein match is: integral membrane protein, putative (TAIR:AT5G02060.1); Has 100 Blast hits to 100 proteins in 8 species: Archae - 0; Bacteria - 0; Metazoa - 0; Fungi - 0; Plants - 100; Viruses - 0; Other Eukaryotes - 0 (source: NCBI BLink). | chr3:19948262-19948994 REVERSEweakly similar to ( 174) loc_os09g07480 12009.m04017 protein expressed protein no original description 	T
27.2	RNA.transcription	MDP0000483776	 moderately similar to ( 330) AT5G59180 | Symbols: NRPB7 | NRPB7; DNA-directed RNA polymerase/ RNA binding | chr5:23883499-23884976 FORWARDmoderately similar to ( 345) RPB7_SOYBN DNA-directed RNA polymerase II 19 kDa polypeptide (EC 2.7.7.6) (RNA polymerase II subunit 5) - Glycine max (Soybean)moderately similar to ( 329) loc_os05g33240 12005.m27814 protein DNA-directed RNA polymerase II 19 kDa polypeptide, putative, expressed no original description 	T
29.4	protein.postranslational modification	MDP0000337353	 moderately similar to ( 334) AT5G10480 | Symbols: PAS2, PEP | PAS2 (PASTICCINO 2); enoyl-CoA hydratase/ protein tyrosine phosphatase | chr5:3298047-3299980 REVERSEmoderately similar to ( 350) loc_os04g20280 12004.m07185 protein PAS2, putative, expressed no original description 	T
35.2	not assigned.unknown	MDP0000878750	 moderately similar to ( 448) AT2G41530 | Symbols: ATSFGH, SFGH | SFGH (S-FORMYLGLUTATHIONE HYDROLASE); S-formylglutathione hydrolase/ hydrolase, acting on ester bonds | chr2:17323656-17325430 REVERSEmoderately similar to ( 418) loc_os01g71300 12001.m43439 protein esterase D, putative, expressed  no original description 	T
35.2	not assigned.unknown	MDP0000129874	 moderately similar to ( 260) AT4G27435 | Symbols:  | unknown protein | chr4:13723903-13724872 FORWARDmoderately similar to ( 231) loc_os07g27810 12007.m07055 protein fiber protein Fb34, putative, expressed no original description 	T
26.4.1	misc.beta 1,3 glucan hydrolases.glucan endo-1,3-beta-glucosidase	MDP0000630545	 highly similar to ( 657) AT5G56590 | Symbols:  | glycosyl hydrolase family 17 protein | chr5:22907521-22909436 FORWARDmoderately similar to ( 296) E13B_WHEAT Glucan endo-1,3-beta-glucosidase precursor (EC 3.2.1.39) ((1->3)-beta-glucan endohydrolase) ((1->3)-beta-glucanase) (Beta-1,3-endoglucanase) - Triticum aestivum (Wheat)highly similar to ( 675) loc_os01g53750 12001.m11541 protein glucan endo-1,3-beta-glucosidase 4 precursor, putative, expressed  no original description 	T
35.2	not assigned.unknown	MDP0000494807	 moderately similar to ( 459) AT4G39820 | Symbols:  | binding | chr4:18476698-18477924 REVERSEmoderately similar to ( 248) loc_os02g34540 12002.m08543 protein tetratricopeptide repeat protein 15, putative, expressed   no original description 	T
34.1.1	transport.p- and v-ATPases.H+-transporting two-sector ATPase	MDP0000237744	 weakly similar to ( 140) AT2G34770 | Symbols: FAH1 | FAH1 (FATTY ACID HYDROXYLASE 1); catalytic/ fatty acid alpha-hydroxylase | chr2:14666776-14668061 FORWARDvery weakly similar to (55.5) VATL_ORYSA Vacuolar ATP synthase 16 kDa proteolipid subunit (EC 3.6.3.14) - Oryza sativa (Rice)weakly similar to ( 148) loc_os03g56820 12003.m101521 protein inositolphosphorylceramide-B C-26 hydroxylase, putative, expressed  no original description 	T
35.2	not assigned.unknown	MDP0000333431	   no original description 	T
30.5	signalling.G-proteins	MDP0000156869	 moderately similar to ( 340) AT3G54840 | Symbols: ARA6, RABF1 | ARA6; GTP binding / GTPase | chr3:20318597-20320737 FORWARDmoderately similar to ( 211) RHN1_NICPL Ras-related protein RHN1 - Nicotiana plumbaginifolia (Leadwort-leaved tobacco)moderately similar to ( 311) loc_os10g30520 12010.m05913 protein ras-related protein RHN1, putative, expressed no original description 	T
35.1	not assigned.no ontology	MDP0000159170	 highly similar to ( 598) AT1G16570 | Symbols:  | glycosyl transferase family 1 protein | chr1:5670763-5673423 REVERSEhighly similar to ( 571) loc_os03g08300 12003.m101214 protein beta-1,4-mannosyltransferase, putative, expressed   no original description 	T
26.7	misc.oxidases - copper, flavone etc	MDP0000221906	 highly similar to ( 898) AT4G22010 | Symbols: sks4 | sks4 (SKU5 Similar 4); copper ion binding / oxidoreductase | chr4:11663429-11666463 FORWARDhighly similar to ( 589) ASO_BRANA L-ascorbate oxidase homolog precursor (EC 1.10.3.3) (Ascorbase) - Brassica napus (Rape)highly similar to ( 822) loc_os06g46500 12006.m09176 protein L-ascorbate oxidase homolog precursor, putative, expressed   no original description 	T
35.2	not assigned.unknown	MDP0000121614	 weakly similar to ( 138) AT2G43630 | Symbols:  | FUNCTIONS IN: molecular_function unknown; INVOLVED IN: biological_process unknown; LOCATED IN: chloroplast thylakoid membrane, chloroplast, nucleus, chloroplast envelope; EXPRESSED IN: 22 plant structures; EXPRESSED DURING: 13 growth stages; BEST Arabidopsis thaliana protein match is: glycine-rich protein (TAIR:AT3G59640.2); Has 48 Blast hits to 48 proteins in 14 species: Archae - 0; Bacteria - 2; Metazoa - 10; Fungi - 0; Plants - 32; Viruses - 2; Other Eukaryotes - 2 (source: NCBI BLink). | chr2:18096255-18097344 FORWARDvery weakly similar to (73.2) loc_os02g39340 12002.m100268 protein expressed protein  no original description 	T
35.1	not assigned.no ontology	MDP0000184167	 moderately similar to ( 310) AT1G80770 | Symbols: PDE318 | PDE318 (pigment defective 318); GTP binding | chr1:30355266-30357786 FORWARDmoderately similar to ( 275) loc_os08g06230 12008.m04762 protein nucleolar GTP-binding protein 1, putative, expressed no original description 	T
16.1.2.3	secondary metabolism.isoprenoids.mevalonate pathway.HMG-CoA reductase	MDP0000789333	 moderately similar to ( 382) AT1G76490 | Symbols: HMG1, HMGR1 | HMG1 (HYDROXY METHYLGLUTARYL COA REDUCTASE 1); hydroxymethylglutaryl-CoA reductase | chr1:28695801-28698206 FORWARDmoderately similar to ( 400) HMDH1_HEVBR 3-hydroxy-3-methylglutaryl-coenzyme A reductase 1 (EC 1.1.1.34) (HMG-CoA reductase 1) - Hevea brasiliensis (Para rubber tree)moderately similar to ( 389) loc_os08g40180 12008.m07998 protein 3-hydroxy-3-methylglutaryl-coenzyme A reductase 3, putative, expressed   no original description 	T
20.2.99	stress.abiotic.unspecified	MDP0000177386	 moderately similar to ( 363) AT5G61760 | Symbols: ATIPK2BETA, IPK2B, IPK2BETA | ATIPK2BETA; inositol or phosphatidylinositol kinase/ inositol trisphosphate 6-kinase | chr5:24813916-24814818 REVERSEweakly similar to ( 152) RHRE_PEA Rhicadhesin receptor precursor (Germin-like protein) - Pisum sativum (Garden pea)moderately similar to ( 282) loc_os02g32370 12002.m08330 protein inositol hexaphosphate kinase, putative, expressed   no original description 	T
3.4.2	minor CHO metabolism.myo-inositol.InsP-Kinases	MDP0000703056	 moderately similar to ( 355) AT5G61760 | Symbols: ATIPK2BETA, IPK2B, IPK2BETA | ATIPK2BETA; inositol or phosphatidylinositol kinase/ inositol trisphosphate 6-kinase | chr5:24813916-24814818 REVERSEmoderately similar to ( 278) loc_os02g32370 12002.m08330 protein inositol hexaphosphate kinase, putative, expressed no original description 	T
26.1	misc.misc2	MDP0000807347	 moderately similar to ( 427) AT3G51000 | Symbols:  | epoxide hydrolase, putative | chr3:18945258-18946499 REVERSEmoderately similar to ( 367) loc_os12g43970 12012.m08168 protein epoxide hydrolase 2, putative, expressed  no original description 	T
29.6	protein.folding	MDP0000614851	 highly similar to ( 887) AT2G28000 | Symbols: CPN60A, CH-CPN60A, SLP | CPN60A (CHAPERONIN-60ALPHA); ATP binding / protein binding | chr2:11926603-11929184 FORWARDhighly similar to ( 910) RUBA_PEA RuBisCO large subunit-binding protein subunit alpha, chloroplast precursor (60 kDa chaperonin subunit alpha) (CPN-60 alpha) - Pisum sativum (Garden pea)highly similar to ( 839) loc_os03g64210 12003.m101142 protein ruBisCO large subunit-binding protein subunit alpha, chloroplast precursor, putative, expressed  MSP1_C  no original description 	T
28.99	DNA.unspecified	MDP0000186626	 moderately similar to ( 439) AT5G17570 | Symbols:  | tatD-related deoxyribonuclease family protein | chr5:5792948-5794580 FORWARDmoderately similar to ( 382) loc_os04g48820 12004.m101631 protein deoxyribonuclease ycfH, putative, expressed  no original description 	T
29.2.1.1.1.2.12	protein.synthesis.ribosomal protein.prokaryotic.chloroplast.50S subunit.L12	MDP0000235602	 weakly similar to ( 155) AT3G27830 | Symbols: RPL12-A, RPL12 | RPL12-A (RIBOSOMAL PROTEIN L12-A); structural constituent of ribosome | chr3:10318576-10319151 FORWARDweakly similar to ( 141) RK12_SPIOL 50S ribosomal protein L12, chloroplast precursor (CL12) - Spinacia oleracea (Spinach)very weakly similar to (97.8) loc_os01g47330 12001.m10928 protein 50S ribosomal protein L12-1, chloroplast precursor, putative, expressed  no original description 	T
27.3.6	RNA.regulation of transcription.bHLH,Basic Helix-Loop-Helix family	MDP0000902699	 weakly similar to ( 127) AT4G00050 | Symbols: UNE10 | UNE10 (unfertilized embryo sac 10); DNA binding / transcription factor | chr4:17863-19848 FORWARDvery weakly similar to (51.6) ARRS_MAIZE Anthocyanin regulatory R-S protein - Zea mays (Maize)very weakly similar to (89.0) loc_os03g43810 12003.m35328 protein PIL5, putative, expressed no original description 	T
29.2.1.2.2.31	protein.synthesis.ribosomal protein.eukaryotic.60S subunit.L31	MDP0000263151	 weakly similar to ( 172) AT2G19740 | Symbols:  | 60S ribosomal protein L31 (RPL31A) | chr2:8513577-8514346 FORWARDweakly similar to ( 159) RL31_PANGI 60S ribosomal protein L31 - Panax ginseng (Korean ginseng)weakly similar to ( 175) loc_os08g39500 12008.m07931 protein 60S ribosomal protein L31, putative, expressed no original description 	T
29.4	protein.postranslational modification	MDP0000263170	 moderately similar to ( 266) AT4G25130 | Symbols:  | peptide methionine sulfoxide reductase, putative | chr4:12898802-12899998 REVERSEmoderately similar to ( 330) MSRA_FRAAN Peptide methionine sulfoxide reductase (EC 1.8.4.11) (Protein-methionine-S-oxide reductase) (Peptide-methionine (S)-S-oxide reductase) (Peptide Met(O) reductase) (Fruit-ripening protein E4) - Fragaria ananassa (Strawberry)moderately similar to ( 254) loc_os10g41400 12010.m22009 protein peptide methionine sulfoxide reductase, putative, expressed no original description 	T
29.5.11.4.5.2	protein.degradation.ubiquitin.E3.BTB/POZ Cullin3.BTB/POZ	MDP0000296225	 moderately similar to ( 421) AT1G05690 | Symbols: BT3 | BT3 (BTB AND TAZ DOMAIN PROTEIN 3); protein binding / transcription regulator | chr1:1707809-1709132 FORWARDmoderately similar to ( 401) loc_os01g66890 12001.m43320 protein protein binding protein, putative, expressed no original description 	T
29.5.11.4.3.2	protein.degradation.ubiquitin.E3.SCF.FBOX	MDP0000282995	 moderately similar to ( 280) AT1G23390 | Symbols:  | kelch repeat-containing F-box family protein | chr1:8309449-8310633 REVERSEweakly similar to ( 142) loc_os09g12150 12009.m04477 protein expressed protein   no original description 	T
30.5	signalling.G-proteins	MDP0000893876	 highly similar to ( 664) AT3G63130 | Symbols: RANGAP1 | RANGAP1 (RAN GTPASE ACTIVATING PROTEIN 1); RAN GTPase activator/ protein binding | chr3:23325108-23326715 FORWARDhighly similar to ( 660) loc_os05g46560 12005.m27945 protein ran GTPase activating protein, putative, expressed no original description 	T
35.2	not assigned.unknown	MDP0000830882	 moderately similar to ( 218) AT3G18800 | Symbols:  | unknown protein | chr3:6479746-6480339 FORWARDweakly similar to ( 149) loc_os04g59050 12004.m10783 protein expressed protein   no original description 	T
35.2	not assigned.unknown	MDP0000173271	 moderately similar to ( 416) AT4G03420 | Symbols:  | unknown protein | chr4:1512226-1513594 FORWARDmoderately similar to ( 286) loc_os10g35150 12010.m06338 protein expressed protein  no original description 	T
35.2	not assigned.unknown	MDP0000538450	   no original description 	T
35.1	not assigned.no ontology	MDP0000411286	 weakly similar to ( 188) AT1G79740 | Symbols:  | DNA binding / protein dimerization | chr1:30004367-30006715 REVERSEhighly similar to ( 685) loc_os06g44800 12006.m09007 protein transposon protein, putative, Ac/Ds sub-class, expressed no original description 	T
30.2.11	signalling.receptor kinases.leucine rich repeat XI	MDP0000433305	 moderately similar to ( 462) AT3G47570 | Symbols:  | leucine-rich repeat transmembrane protein kinase, putative | chr3:17527611-17530748 FORWARDmoderately similar to ( 306) RPK1_IPONI Receptor-like protein kinase precursor (EC 2.7.11.1) - Ipomoea nil (Japanese morning glory) (Pharbitis nil)highly similar to ( 504) loc_os11g47180 12011.m08498 protein receptor-like protein kinase precursor, putative, expressed   no original description 	T
20.1.7	stress.biotic.PR-proteins	MDP0000856947	 moderately similar to ( 219) AT1G55210 | Symbols:  | disease resistance response | chr1:20598057-20598620 REVERSEvery weakly similar to (70.1) DR206_PEA Disease resistance response protein 206 - Pisum sativum (Garden pea)weakly similar to ( 172) loc_os11g07830 12011.m04979 protein dirigent-like protein pDIR9, putative, expressed  no original description 	T
15	metal handling	MDP0000199671	 highly similar to ( 827) AT4G14040 | Symbols: EDA38, SBP2 | SBP2 (SELENIUM-BINDING PROTEIN 2); selenium binding | chr4:8100691-8102828 REVERSEhighly similar to ( 802) loc_os01g68770 12001.m12979 protein selenium-binding protein, putative, expressed no original description 	T
17.2.3	hormone metabolism.auxin.induced-regulated-responsive-activated	MDP0000912039	 weakly similar to ( 174) AT2G21220 | Symbols:  | auxin-responsive protein, putative | chr2:9089380-9089694 FORWARDvery weakly similar to (87.8) AXX15_SOYBN Auxin-induced protein X15 - Glycine max (Soybean)weakly similar to ( 116) loc_os02g24740 12002.m07621 protein OsSAUR9 - Auxin-responsive SAUR gene family member, expressed no original description 	T
33.99	development.unspecified	MDP0000870142	 highly similar to ( 505) AT3G13300 | Symbols: VCS | VCS (VARICOSE); nucleotide binding / protein homodimerization | chr3:4304085-4309949 FORWARDmoderately similar to ( 468) loc_os06g19660 12006.m06625 protein nucleotide binding protein, putative, expressed   no original description 	T
26.19	misc.plastocyanin-like	MDP0000923711	 weakly similar to ( 167) AT5G15350 | Symbols:  | plastocyanin-like domain-containing protein | chr5:4985184-4986154 REVERSEvery weakly similar to (65.9) BCP_PEA Blue copper protein precursor - Pisum sativum (Garden pea)very weakly similar to (99.4) loc_os09g38540 12009.m06795 protein blue copper protein precursor, putative, expressed no original description 	T
26.8	misc.nitrilases, *nitrile lyases, berberine bridge enzymes, reticuline oxidases, troponine reductases	MDP0000253475	 highly similar to ( 512) AT5G15610 | Symbols:  | proteasome family protein | chr5:5079579-5081929 FORWARDweakly similar to ( 159) PIR7A_ORYSA Probable esterase PIR7A (EC 3.1.-.-) - Oryza sativa (Rice)moderately similar to ( 479) loc_os04g01290 12004.m101446 protein PCI domain containing protein, expressed COG3889  no original description 	T
29.1.9	protein.aa activation.valine-tRNA ligase	MDP0000167080	 nearly identical (1480) AT1G14610 | Symbols: TWN2, VALRS | TWN2 (TWIN 2); ATP binding / aminoacyl-tRNA ligase/ nucleotide binding / valine-tRNA ligase | chr1:5008502-5014486 REVERSEnearly identical (1390) loc_os03g48850 12003.m35366 protein valyl-tRNA synthetase, putative, expressed  no original description 	T
24	Biodegradation of Xenobiotics	MDP0000449901	 moderately similar to ( 362) AT1G19900 | Symbols:  | glyoxal oxidase-related | chr1:6907038-6908684 REVERSEmoderately similar to ( 306) loc_os03g15320 12003.m06969 protein glyoxal oxidase, putative, expressed no original description 	T
26.13	misc.acid and other phosphatases	MDP0000264959	 moderately similar to ( 401) AT1G72880 | Symbols:  | acid phosphatase survival protein SurE, putative | chr1:27423678-27425928 REVERSEmoderately similar to ( 302) loc_os01g51280 12001.m43160 protein acid phosphatase, putative, expressed  no original description 	T
27.3.30	RNA.regulation of transcription.Trihelix, Triple-Helix transcription factor family	MDP0000209313	 moderately similar to ( 342) AT5G28300 | Symbols:  | trihelix DNA-binding protein, putative | chr5:10292789-10295101 REVERSEweakly similar to ( 168) loc_os04g45750 12004.m09526 protein expressed protein  no original description 	T
35.2	not assigned.unknown	MDP0000212216	 very weakly similar to (85.5) AT2G40475 | Symbols:  | unknown protein | chr2:16907222-16907803 REVERSEvery weakly similar to (78.6) loc_os01g07240 12001.m07350 protein expressed protein  no original description 	T
10.5.2	cell wall.cell wall proteins.proline rich proteins	MDP0000206705	 weakly similar to ( 144) AT4G38770 | Symbols: PRP4, ATPRP4 | PRP4 (PROLINE-RICH PROTEIN 4) | chr4:18097009-18098448 REVERSEvery weakly similar to (54.3) YCF1_CUCSA Putative membrane protein ycf1 - Cucumis sativus (Cucumber)very weakly similar to (94.4) loc_os10g05750 12010.m03978 protein proline-rich protein, putative, expressed  no original description 	T
10.7	cell wall.modification	MDP0000432497	 moderately similar to ( 332) AT2G03090 | Symbols: ATEXPA15, EXP15, ATEXP15, ATHEXP ALPHA 1.3 | ATEXPA15 (ARABIDOPSIS THALIANA EXPANSIN A15) | chr2:917361-918554 REVERSEmoderately similar to ( 300) EXPA4_ORYSA Expansin-A4 precursor (OsEXPA4) (Alpha-expansin-4) (OsEXP4) (OsaEXPa1.22) - Oryza sativa (Rice)moderately similar to ( 300) loc_os05g39990 12005.m08176 protein alpha-expansin 1 precursor, putative, expressed  no original description 	T
32	micro RNA, natural antisense etc	MDP0000148925	 very weakly similar to (58.5) AT1G67195 | Symbols: MIR414 | MIR414 (MICRORNA 414) | chr1:25137378-25137644 REVERSE  no original description 	T
17.5.1	hormone metabolism.ethylene.synthesis-degradation	MDP0000751780	 moderately similar to ( 206) AT3G60290 | Symbols:  | oxidoreductase/ oxidoreductase, acting on paired donors, with incorporation or reduction of molecular oxygen, 2-oxoglutarate as one donor, and incorporation of one atom each of oxygen into both donors | chr3:22282662-22284414 FORWARDweakly similar to ( 102) LDOX_MALDO Leucoanthocyanidin dioxygenase (EC 1.14.11.19) (LDOX) (Leucocyanidin oxygenase) (Leucoanthocyanidin hydroxylase) (Anthocyanidin synthase) - Malus domestica (Apple) (Malus sylvestris)moderately similar to ( 232) loc_os04g57180 12004.m10603 protein flavonol synthase/flavanone 3-hydroxylase, putative, expressed no original description 	T
29.5.11.4.3.2	protein.degradation.ubiquitin.E3.SCF.FBOX	MDP0000175577	 moderately similar to ( 431) AT1G16070 | Symbols: AtTLP8 | AtTLP8 (TUBBY LIKE PROTEIN 8); transcription factor | chr1:5511899-5513779 REVERSEmoderately similar to ( 370) loc_os02g08310 12002.m06177 protein tub family protein, expressed   no original description 	T
30.5	signalling.G-proteins	MDP0000193733	 moderately similar to ( 462) AT4G02790 | Symbols:  | GTP-binding family protein | chr4:1247416-1249351 FORWARDmoderately similar to ( 477) loc_os05g35540 12005.m07784 protein GTPase, putative, expressed  no original description 	T
26.19	misc.plastocyanin-like	MDP0000758655	 very weakly similar to (89.0) AT1G08500 | Symbols:  | plastocyanin-like domain-containing protein | chr1:2689110-2689881 FORWARDweakly similar to ( 103) loc_os12g26880 12012.m06506 protein uclacyanin-2 precursor, putative, expressed   no original description 	T
29.4	protein.postranslational modification	MDP0000226074	 highly similar to ( 585) AT5G10930 | Symbols: CIPK5, SnRK3.24 | CIPK5 (CBL-INTERACTING PROTEIN KINASE 5); ATP binding / kinase/ protein kinase/ protein serine/threonine kinase | chr5:3445569-3446906 REVERSEmoderately similar to ( 396) CIPK1_ORYSA CIPK-like protein 1 (EC 2.7.11.1) (OsCK1) - Oryza sativa (Rice)moderately similar to ( 483) loc_os09g25090 12009.m05662 protein CIPK-like protein 1, putative, expressed   no original description 	T
26.10	misc.cytochrome P450	MDP0000295142	 moderately similar to ( 377) AT3G26290 | Symbols: CYP71B26 | CYP71B26; electron carrier/ heme binding / iron ion binding / monooxygenase/ oxygen binding | chr3:9632770-9634439 REVERSEmoderately similar to ( 390) C71A9_SOYBN Cytochrome P450 71A9 (EC 1.14.-.-) (P450 CP1) - Glycine max (Soybean)moderately similar to ( 348) loc_os09g26960 12009.m05848 protein flavonoid 3-monooxygenase, putative, expressed  no original description 	T
27.1	RNA.processing	MDP0000120192	 weakly similar to ( 150) AT4G30220 | Symbols: RUXF | RUXF (SMALL NUCLEAR RIBONUCLEOPROTEIN F) | chr4:14803100-14804259 REVERSEweakly similar to ( 149) loc_os11g43620 12011.m28864 protein small nuclear ribonucleoprotein F, putative, expressed no original description 	T
27.1.19	RNA.processing.ribonucleases	MDP0000242287	 moderately similar to ( 429) AT2G32070 | Symbols:  | CCR4-NOT transcription complex protein, putative | chr2:13640829-13641656 REVERSEmoderately similar to ( 390) loc_os02g55300 12002.m10556 protein CCR4-NOT transcription complex subunit 7, putative, expressed no original description 	T
35.2	not assigned.unknown	MDP0000321798	 highly similar to ( 729) AT5G51600 | Symbols: PLE, ATMAP65-3, MAP65-3 | PLE (PLEIADE); microtubule binding | chr5:20961061-20964080 REVERSEhighly similar to ( 681) loc_os01g49200 12001.m11107 protein microtubule-associated protein, putative, expressed   no original description 	T
35.2	not assigned.unknown	MDP0000810262	  no original description 	T
35.2	not assigned.unknown	MDP0000257001	 very weakly similar to (78.6) AT4G16444 | Symbols:  | unknown protein | chr4:9274791-9276056 REVERSE  no original description 	T
13.2.4.1	amino acid metabolism.degradation.branched chain group.shared	MDP0000866160	 moderately similar to ( 252) AT2G33150 | Symbols: PKT3, PED1, KAT2 | PKT3 (PEROXISOMAL 3-KETOACYL-COA THIOLASE 3); acetyl-CoA C-acyltransferase | chr2:14047814-14050983 REVERSEmoderately similar to ( 255) loc_os10g31950 12010.m06045 protein 3-ketoacyl-CoA thiolase 2, peroxisomal precursor, putative, expressed no original description 	T
26.19	misc.plastocyanin-like	MDP0000157828	 weakly similar to ( 199) AT1G08500 | Symbols:  | plastocyanin-like domain-containing protein | chr1:2689110-2689881 FORWARDvery weakly similar to (60.1) BCP_PEA Blue copper protein precursor - Pisum sativum (Garden pea)weakly similar to ( 173) loc_os01g17470 12001.m08338 protein blue copper protein precursor, putative, expressed  no original description 	T
27.1.19	RNA.processing.ribonucleases	MDP0000151829	 very weakly similar to (63.9) AT1G24020 | Symbols: MLP423 | MLP423 (MLP-LIKE PROTEIN 423) | chr1:8500653-8501458 REVERSEmoderately similar to ( 237) MAL12_MALDO Major allergen Mal d 1 (Mal d I) (AP15) - Malus domestica (Apple) (Malus sylvestris)weakly similar to ( 106) loc_os03g18850 12003.m07305 protein pathogenesis-related protein 1, putative, expressed  no original description 	T
20.2.99	stress.abiotic.unspecified	MDP0000151829	 very weakly similar to (63.9) AT1G24020 | Symbols: MLP423 | MLP423 (MLP-LIKE PROTEIN 423) | chr1:8500653-8501458 REVERSEmoderately similar to ( 237) MAL12_MALDO Major allergen Mal d 1 (Mal d I) (AP15) - Malus domestica (Apple) (Malus sylvestris)weakly similar to ( 106) loc_os03g18850 12003.m07305 protein pathogenesis-related protein 1, putative, expressed  no original description 	T
20.2.1	stress.abiotic.heat	MDP0000567268	 moderately similar to ( 326) AT3G08910 | Symbols:  | DNAJ heat shock protein, putative | chr3:2710402-2711811 REVERSEweakly similar to ( 120) DNJH_CUCSA DnaJ protein homolog (DNAJ-1) - Cucumis sativus (Cucumber)moderately similar to ( 313) loc_os05g48810 12005.m27974 protein dnaJ homolog subfamily B member 5, putative, expressed no original description 	T
33.99	development.unspecified	MDP0000212760	 moderately similar to ( 459) AT1G79990 | Symbols:  | protein binding / structural molecule | chr1:30085910-30091949 FORWARDvery weakly similar to (97.1) PF20_CHLRE Flagellar WD repeat protein PF20 - Chlamydomonas reinhardtiimoderately similar to ( 456) loc_os06g05180 12006.m71402 protein coatomer subunit beta, putative, expressed no original description 	T
29.3.2	protein.targeting.mitochondria	MDP0000303749	 weakly similar to ( 190) AT4G26670 | Symbols:  | mitochondrial import inner membrane translocase subunit Tim17/Tim22/Tim23 family protein | chr4:13452257-13453579 FORWARDweakly similar to ( 160) loc_os12g33020 12012.m07100 protein protein translocase/ protein transporter, putative, expressed   no original description 	T
20.1.7	stress.biotic.PR-proteins	MDP0000240977	 moderately similar to ( 371) AT5G36930 | Symbols:  | disease resistance protein (TIR-NBS-LRR class), putative | chr5:14567771-14571916 REVERSEvery weakly similar to (82.0) loc_os11g12340 12011.m28760 protein disease resistance protein RPM1, putative, expressed  no original description 	T
29.2.1.2.2.80	protein.synthesis.ribosomal protein.eukaryotic.60S subunit.P0	MDP0000240422	 moderately similar to ( 461) AT2G40010 | Symbols:  | 60S acidic ribosomal protein P0 (RPP0A) | chr2:16708578-16710448 REVERSEmoderately similar to ( 467) RLA0_SOYBN 60S acidic ribosomal protein P0 - Glycine max (Soybean)moderately similar to ( 437) loc_os08g03640 12008.m04508 protein 60S acidic ribosomal protein P0, putative, expressed  no original description 	T
35.1	not assigned.no ontology	MDP0000195837	 weakly similar to ( 150) AT3G11910 | Symbols: UBP13 | UBP13 (UBIQUITIN-SPECIFIC PROTEASE 13); ubiquitin thiolesterase/ ubiquitin-specific protease | chr3:3761758-3770290 REVERSEweakly similar to ( 147) loc_os11g36470 12011.m07499 protein cysteine-type endopeptidase/ double-stranded DNA binding protein, putative, expressed TrbL  no original description 	T
2.2.2.1.2	major CHO metabolism.degradation.starch.starch cleavage.beta amylase	MDP0000199386	 highly similar to ( 572) AT5G18670 | Symbols: BMY3, BAM9 | BMY3; beta-amylase/ catalytic/ cation binding | chr5:6226138-6227999 FORWARDmoderately similar to ( 271) AMYB_VIGUN Beta-amylase (EC 3.2.1.2) (1,4-alpha-D-glucan maltohydrolase) - Vigna unguiculata (Cowpea)moderately similar to ( 394) loc_os07g47120 12007.m08929 protein beta-amylase, putative, expressed   no original description 	T
29.5.11.1	protein.degradation.ubiquitin.ubiquitin	MDP0000287424	 moderately similar to ( 240) AT3G52590 | Symbols: UBQ1, EMB2167, ERD16, HAP4 | UBQ1 (UBIQUITIN EXTENSION PROTEIN 1); protein binding / structural constituent of ribosome | chr3:19505668-19506681 FORWARDweakly similar to ( 150) UBIQ_WHEAT Ubiquitin - Triticum aestivum (Wheat)moderately similar to ( 240) loc_os09g39500 12009.m60242 protein ubiquitin fusion protein, putative, expressed  no original description 	T
29.2.2.3.4	protein.synthesis.ribosome biogenesis.Pre-rRNA processing and modifications.WD-repeat proteins	MDP0000556407	 highly similar to ( 538) AT5G14050 | Symbols:  | transducin family protein / WD-40 repeat family protein | chr5:4533539-4535179 FORWARDhighly similar to ( 516) loc_os07g41190 12007.m08355 protein WD-repeat protein 50, putative, expressed  no original description 	T
33.99	development.unspecified	MDP0000218910	 moderately similar to ( 437) AT2G30300 | Symbols:  | nodulin-related | chr2:12919401-12921222 FORWARDmoderately similar to ( 477) loc_os05g39800 12005.m08158 protein nodulin-like family protein, expressed  TrbL tolA Sporozoite_P67  no original description 	T
34.7	transport.phosphate	MDP0000296215	 nearly identical (1022) AT1G68740 | Symbols:  | EXS family protein / ERD1/XPR1/SYG1 family protein | chr1:25812735-25816574 REVERSEhighly similar to ( 887) loc_os06g29790 12006.m07523 protein phosphate transporter 1, putative, expressed  no original description 	T
29.2.1.1.1.2.5	protein.synthesis.ribosomal protein.prokaryotic.chloroplast.50S subunit.L5	MDP0000213335	 moderately similar to ( 291) AT4G01310 | Symbols:  | ribosomal protein L5 family protein | chr4:544166-545480 REVERSEmoderately similar to ( 265) RK5_SPIOL 50S ribosomal protein L5, chloroplast - Spinacia oleracea (Spinach)moderately similar to ( 226) loc_os03g03360 12003.m05871 protein 50S ribosomal protein L5, chloroplast precursor, putative, expressed  no original description 	T
33.99	development.unspecified	MDP0000281264	 weakly similar to ( 116) AT2G42840 | Symbols: PDF1 | PDF1 (PROTODERMAL FACTOR 1) | chr2:17826327-17827426 REVERSEweakly similar to ( 101) loc_os01g52690 12001.m11442 protein DNA-directed RNA polymerase II largest subunit, putative, expressed  no original description 	T
35.1	not assigned.no ontology	MDP0000826636	 moderately similar to ( 321) AT5G02230 | Symbols:  | haloacid dehalogenase-like hydrolase family protein | chr5:449132-450507 FORWARDmoderately similar to ( 295) loc_os03g16670 12003.m35158 protein catalytic/ hydrolase, putative, expressed no original description 	T
35.2	not assigned.unknown	MDP0000795631	 weakly similar to ( 168) loc_os07g06644 12007.m66710 protein expressed protein   no original description 	T
10.5.1.1	cell wall.cell wall proteins.AGPs.AGP	MDP0000707036	 very weakly similar to (75.1) AT3G61640 | Symbols: AGP20, AtAGP20 | AGP20 | chr3:22810283-22810629 REVERSEvery weakly similar to (64.3) loc_os05g12580 12005.m05721 protein AGP20, putative no original description 	T
29.5.11.4.2	protein.degradation.ubiquitin.E3.RING	MDP0000240829	 moderately similar to ( 402) AT1G10650 | Symbols:  | protein binding / zinc ion binding | chr1:3524122-3525060 REVERSEmoderately similar to ( 246) loc_os02g03760 12002.m33535 protein S-ribonuclease binding protein SBP1, putative, expressed no original description 	T
16.4.1	secondary metabolism.N misc.alkaloid-like	MDP0000182468	 weakly similar to ( 121) AT3G57030 | Symbols:  | strictosidine synthase family protein | chr3:21101653-21103204 REVERSEvery weakly similar to (67.0) STSY_CATRO Strictosidine synthase precursor (EC 4.3.3.2) - Catharanthus roseus (Rosy periwinkle) (Madagascar periwinkle)moderately similar to ( 211) loc_os07g42250 12007.m08460 protein strictosidine synthase 1 precursor, putative, expressed   COG4907 CobN COG4934  no original description 	T
35.2	not assigned.unknown	MDP0000130023	  no original description 	T
35.1	not assigned.no ontology	MDP0000280862	 moderately similar to ( 226) AT2G35760 | Symbols:  | integral membrane family protein | chr2:15032059-15033094 FORWARDweakly similar to ( 150) loc_os12g32970 12012.m07095 protein membrane protein, putative, expressed   no original description 	T
35.2	not assigned.unknown	MDP0000792050	   no original description 	T
16.10	secondary metabolism.simple phenols	MDP0000125288	 highly similar to ( 689) AT5G21100 | Symbols:  | L-ascorbate oxidase, putative | chr5:7168312-7170719 FORWARDhighly similar to ( 824) ASO_TOBAC L-ascorbate oxidase precursor (EC 1.10.3.3) (Ascorbase) (ASO) - Nicotiana tabacum (Common tobacco)highly similar to ( 756) loc_os06g37080 12006.m08243 protein L-ascorbate oxidase precursor, putative, expressed   no original description 	T
21.2	redox.ascorbate and glutathione	MDP0000152585	 moderately similar to ( 271) AT5G52240 | Symbols: MSBP1, ATMP1, AtMAPR5 | MSBP1 (membrane steroid binding protein 1); steroid binding | chr5:21213121-21214557 FORWARDmoderately similar to ( 272) loc_os10g35870 12010.m06404 protein membrane steroid-binding protein 1, putative, expressed  no original description 	T
30.3	signalling.calcium	MDP0000937817	 moderately similar to ( 305) AT5G24270 | Symbols: SOS3, CBL4 | SOS3 (SALT OVERLY SENSITIVE 3); calcium ion binding / calcium-dependent protein serine/threonine phosphatase | chr5:8238781-8240179 REVERSEvery weakly similar to (52.4) CALM_LILLO Calmodulin (CaM) - Lilium longiflorum (Trumpet lily)moderately similar to ( 281) loc_os02g18880 12002.m07131 protein calcineurin B-like protein 4, putative, expressed  no original description 	T
34.2	transport.sugars	MDP0000841918	 highly similar to ( 640) AT3G18830 | Symbols: ATPLT5 | ATPLT5 (POLYOL TRANSPORTER 5); D-ribose transmembrane transporter/ D-xylose transmembrane transporter/ carbohydrate transmembrane transporter/ galactose transmembrane transporter/ glucose transmembrane transporter/ glycerol transmembrane transporter/ mann | chr3:6489000-6491209 REVERSEweakly similar to ( 189) STC_RICCO Sugar carrier protein C - Ricinus communis (Castor bean)highly similar to ( 622) loc_os07g39350 12007.m08177 protein proton myo-inositol cotransporter, putative, expressed   no original description 	T
26.2	misc.UDP glucosyl and glucoronyl transferases	MDP0000261375	 moderately similar to ( 409) AT3G28340 | Symbols: GATL10 | GATL10 (Galacturonosyltransferase-like 10); polygalacturonate 4-alpha-galacturonosyltransferase/ transferase, transferring hexosyl groups | chr3:10589396-10590493 REVERSEmoderately similar to ( 362) loc_os03g18890 12003.m07309 protein transferase, transferring glycosyl groups, putative, expressed  no original description 	T
8.1.6	TCA / org transformation.TCA.succinyl-CoA ligase	MDP0000303254	 highly similar to ( 681) AT2G20420 | Symbols:  | succinyl-CoA ligase (GDP-forming) beta-chain, mitochondrial, putative / succinyl-CoA synthetase, beta chain, putative / SCS-beta, putative | chr2:8805574-8807858 FORWARDhighly similar to ( 662) SUCB_ORYSA Succinyl-CoA ligase [GDP-forming] beta-chain, mitochondrial precursor (EC 6.2.1.4) (Succinyl-CoA synthetase, beta chain) (SCS-beta) - Oryza sativa (Rice)highly similar to ( 662) loc_os02g40830 12002.m100283 protein succinyl-CoA ligase beta-chain, mitochondrial precursor, putative, expressed  no original description 	T
11.1.7	lipid metabolism.FA synthesis and FA elongation.ACP thioesterase	MDP0000951278	 highly similar to ( 583) AT1G08510 | Symbols: FATB | FATB (fatty acyl-ACP thioesterases B); acyl carrier/ acyl-[acyl-carrier-protein] hydrolase | chr1:2691546-2693409 REVERSEhighly similar to ( 582) FATB_GOSHI Myristoyl-acyl carrier protein thioesterase, chloroplast precursor (EC 3.1.2.-) (16:0-acyl-carrier protein thioesterase) (16:0-ACP thioesterase) (Acyl-[acyl-carrier-protein] hydrolase) (PATE) - Gossypium hirsutum (Upland cotton)highly similar to ( 511) loc_os06g05130 12006.m05242 protein myristoyl-acyl carrier protein thioesterase, chloroplast precursor, putative, expressed  no original description 	T
27.2	RNA.transcription	MDP0000269522	 highly similar to ( 865) AT1G14790 | Symbols: RDR1, ATRDRP1 | RDR1 (RNA-DEPENDENT RNA POLYMERASE 1); RNA-directed RNA polymerase/ nucleic acid binding | chr1:5094317-5097817 REVERSEhighly similar to ( 816) loc_os02g50330 12002.m10067 protein RNA-directed RNA polymerase 2, putative, expressed no original description 	T
29.5.11.5	protein.degradation.ubiquitin.ubiquitin protease	MDP0000542989	 highly similar to ( 744) AT3G20630 | Symbols: UBP14, TTN6, ATUBP14 | UBP14 (UBIQUITIN-SPECIFIC PROTEASE 14); ubiquitin-specific protease | chr3:7203001-7208340 REVERSEhighly similar to ( 667) loc_os01g08200 12001.m07445 protein ubiquitin carboxyl-terminal hydrolase 14, putative, expressed  no original description 	T
35.2	not assigned.unknown	MDP0000262757	 highly similar to ( 816) AT1G12380 | Symbols:  | unknown protein | chr1:4214499-4216880 REVERSEhighly similar to ( 721) loc_os02g25230 12002.m07670 protein expressed protein  no original description 	T
26.2	misc.UDP glucosyl and glucoronyl transferases	MDP0000294677	 weakly similar to ( 158) AT5G49690 | Symbols:  | UDP-glucoronosyl/UDP-glucosyl transferase family protein | chr5:20189968-20191350 REVERSEvery weakly similar to (91.3) UFOG_PETHY Anthocyanidin 3-O-glucosyltransferase (EC 2.4.1.115) (Flavonol 3-O-glucosyltransferase) (UDP-glucose flavonoid 3-O-glucosyltransferase) (Anthocyanin rhamnosyl transferase) - Petunia hybrida (Petunia)weakly similar to ( 139) loc_os07g10240 12007.m05479 protein anthocyanidin 3-O-glucosyltransferase, putative  no original description 	T
30.2.11	signalling.receptor kinases.leucine rich repeat XI	MDP0000926385	 moderately similar to ( 367) AT3G49750 | Symbols: AtRLP44 | AtRLP44 (Receptor Like Protein 44); protein binding | chr3:18450604-18451428 REVERSEvery weakly similar to (68.9) RPK1_IPONI Receptor-like protein kinase precursor (EC 2.7.11.1) - Ipomoea nil (Japanese morning glory) (Pharbitis nil)moderately similar to ( 311) loc_os09g04490 12009.m03818 protein BRASSINOSTEROID INSENSITIVE 1-associated receptor kinase 1 precursor, putative, expressed   no original description 	T
34.2	transport.sugars	MDP0000755167	 moderately similar to ( 427) AT3G18830 | Symbols: ATPLT5 | ATPLT5 (POLYOL TRANSPORTER 5); D-ribose transmembrane transporter/ D-xylose transmembrane transporter/ carbohydrate transmembrane transporter/ galactose transmembrane transporter/ glucose transmembrane transporter/ glycerol transmembrane transporter/ mann | chr3:6489000-6491209 REVERSEweakly similar to ( 129) HEX6_RICCO Hexose carrier protein HEX6 - Ricinus communis (Castor bean)moderately similar to ( 401) loc_os07g39350 12007.m08177 protein proton myo-inositol cotransporter, putative, expressed  no original description 	T
29.5.11.20	protein.degradation.ubiquitin.proteasom	MDP0000225175	 nearly identical (1396) AT2G20580 | Symbols: RPN1A, ATRPN1A | RPN1A (26S PROTEASOME REGULATORY SUBUNIT S2 1A); binding / enzyme regulator | chr2:8859211-8864699 FORWARDnearly identical (1274) loc_os02g05340 12002.m05882 protein 26S proteasome regulatory subunit rpn1, putative, expressed   no original description 	T
35.1.5	not assigned.no ontology.pentatricopeptide (PPR) repeat-containing protein	MDP0000281231	 highly similar to ( 661) AT3G47840 | Symbols:  | pentatricopeptide (PPR) repeat-containing protein | chr3:17651912-17654032 FORWARDweakly similar to ( 108) ERG_ANTMA GTP-binding protein ERG - Antirrhinum majus (Garden snapdragon)highly similar to ( 531) loc_os01g47400 12001.m10935 protein hydrolase, hydrolyzing O-glycosyl compounds, putative, expressed  MSP1_C  no original description 	T
35.2	not assigned.unknown	MDP0000816672	  no original description 	T
35.1	not assigned.no ontology	MDP0000232873	 moderately similar to ( 415) AT1G32230 | Symbols:  | RCD1 (RADICAL-INDUCED CELL DEATH1); protein binding | chr1:11613427-11615748 FORWARDmoderately similar to ( 306) loc_os10g42710 12010.m21812 protein RCD1, putative, expressed  no original description 	T
35.2	not assigned.unknown	MDP0000512591	 very weakly similar to (58.2) AT5G43150 | Symbols:  | unknown protein | chr5:17325076-17325689 FORWARDvery weakly similar to (78.6) loc_os04g51410 12004.m101641 protein expressed protein  no original description 	T
27.3.24	RNA.regulation of transcription.MADS box transcription factor family	MDP0000324166	 moderately similar to ( 345) AT4G18960 | Symbols: AG | AG (AGAMOUS); DNA binding / transcription factor | chr4:10383917-10388272 FORWARDmoderately similar to ( 353) AG_PANGI Floral homeotic protein AGAMOUS (GAG2) - Panax ginseng (Korean ginseng)moderately similar to ( 304) loc_os01g10504 12001.m150506 protein MADS-box transcription factor 3, putative, expressed  no original description 	T
27.3.99	RNA.regulation of transcription.unclassified	MDP0000443139	 moderately similar to ( 407) AT3G59520 | Symbols: ATRBL13 | ATRBL13 (ARABIDOPSIS RHOMBOID-LIKE PROTEIN 13) | chr3:21992054-21992863 FORWARDmoderately similar to ( 391) loc_os01g67040 12001.m43323 protein rhomboid family protein, expressed no original description 	T
29.5.11.3	protein.degradation.ubiquitin.E2	MDP0000443139	 moderately similar to ( 407) AT3G59520 | Symbols: ATRBL13 | ATRBL13 (ARABIDOPSIS RHOMBOID-LIKE PROTEIN 13) | chr3:21992054-21992863 FORWARDmoderately similar to ( 391) loc_os01g67040 12001.m43323 protein rhomboid family protein, expressed no original description 	T
35.2	not assigned.unknown	MDP0000178440	  no original description 	T
31.1	cell.organisation	MDP0000487384	 highly similar to ( 835) AT5G23860 | Symbols: TUB8 | TUB8; structural constituent of cytoskeleton | chr5:8042962-8044528 FORWARDhighly similar to ( 833) TBB2_ORYSA Tubulin beta-2 chain (Beta-2 tubulin) - Oryza sativa (Rice)highly similar to ( 833) loc_os03g01530 12003.m05700 protein tubulin beta-2 chain, putative, expressed   no original description 	T
35.2	not assigned.unknown	MDP0000233115	 moderately similar to ( 355) AT5G08540 | Symbols:  | unknown protein | chr5:2763914-2765431 FORWARDmoderately similar to ( 346) loc_os04g44560 12004.m09415 protein expressed protein no original description 	T
20.1	stress.biotic	MDP0000533075	 moderately similar to ( 254) AT3G14470 | Symbols:  | disease resistance protein (NBS-LRR class), putative | chr3:4857940-4861104 FORWARDmoderately similar to ( 215) loc_os05g41290 12005.m08304 protein disease resistance RPP13-like protein 1, putative, expressed  no original description 	T
35.2	not assigned.unknown	MDP0000149816	 moderately similar to ( 363) AT5G13610 | Symbols:  | unknown protein | chr5:4383073-4384808 FORWARDmoderately similar to ( 367) loc_os07g49410 12007.m079804 protein uncharacterized ACR, YagE family COG1723 containing protein, expressed PRK08026  no original description 	T
35.2	not assigned.unknown	MDP0000290670	 weakly similar to ( 142) AT2G23090 | Symbols:  | unknown protein | chr2:9829657-9830274 REVERSEweakly similar to ( 134) loc_os07g02340 12007.m04706 protein expressed protein no original description 	T
30.7	signalling.14-3-3 proteins	MDP0000137688	 moderately similar to ( 451) AT1G78300 | Symbols: GRF2, 14-3-3OMEGA, GF14 OMEGA | GRF2 (GENERAL REGULATORY FACTOR 2); protein binding / protein phosphorylated amino acid binding | chr1:29461883-29463052 FORWARDmoderately similar to ( 471) 1433_OENHO 14-3-3-like protein - Oenothera hookeri (Hooker's evening primrose)moderately similar to ( 420) loc_os08g33370 12008.m26501 protein 14-3-3-like protein, putative, expressed no original description 	T
35.2	not assigned.unknown	MDP0000940395	 very weakly similar to (58.5) AT1G70350 | Symbols:  | unknown protein | chr1:26507341-26508262 REVERSE  no original description 	T
27.1	RNA.processing	MDP0000250478	 weakly similar to ( 120) AT4G03120 | Symbols:  | proline-rich family protein | chr4:1385747-1387331 FORWARDweakly similar to ( 119) loc_os02g16640 12002.m06907 protein WW domain-binding protein 11, putative, expressed no original description 	T
26.19	misc.plastocyanin-like	MDP0000588940	 very weakly similar to (91.3) AT3G27200 | Symbols:  | plastocyanin-like domain-containing protein | chr3:10043738-10044340 REVERSEvery weakly similar to (63.2) BCP_PEA Blue copper protein precursor - Pisum sativum (Garden pea)weakly similar to ( 119) loc_os02g48820 12002.m09916 protein uclacyanin-2 precursor, putative, expressed  no original description 	T
35.2	not assigned.unknown	MDP0000128931	 nearly identical (1021) AT3G13772 | Symbols:  | endomembrane protein 70, putative | chr3:4521712-4524394 REVERSEhighly similar to ( 920) loc_os06g37160 12006.m08251 protein transmembrane 9 superfamily protein member 4, putative, expressed  no original description 	T
35.1	not assigned.no ontology	MDP0000321344	 weakly similar to ( 129) AT1G76560 | Symbols: CP12-3 | CP12-3 | chr1:28728285-28728689 FORWARDvery weakly similar to (94.4) loc_os08g40240 12008.m08004 protein CP12, putative, expressed   no original description 	T
11.8.2	lipid metabolism.'exotics' (steroids, squalene etc).methylsterol monooxygenase	MDP0000171011	 moderately similar to ( 396) AT1G07420 | Symbols: ATSMO2, SMO2-1 | SMO2-1 (STEROL 4-ALPHA-METHYL-OXIDASE 2-1); 4-alpha-methyl-delta7-sterol-4alpha-methyl oxidase/ C-4 methylsterol oxidase | chr1:2278918-2280033 FORWARDvery weakly similar to (55.8) SC5D_TOBAC Delta(7)-sterol-C5(6)-desaturase (EC 1.3.3.-) (Delta-7-C-5 sterol desaturase) (Delta(7)-sterol-C5-desaturase) - Nicotiana tabacum (Common tobacco)moderately similar to ( 374) loc_os11g48020 12011.m08600 protein C-4 methylsterol oxidase, putative, expressed no original description 	T
35.2	not assigned.unknown	MDP0000663723	  TrbL  no original description 	T
29.5	protein.degradation	MDP0000293458	 highly similar to ( 748) AT4G30920 | Symbols:  | cytosol aminopeptidase family protein | chr4:15046589-15049304 REVERSEhighly similar to ( 731) AMPL2_ORYSA Leucine aminopeptidase 2, chloroplast precursor (EC 3.4.11.1) (LAP 2) (Leucyl aminopeptidase 2) (Proline aminopeptidase 2) (EC 3.4.11.5) (Prolyl aminopeptidase 2) - Oryza sativa (Rice)highly similar to ( 731) loc_os02g55140 12002.m10540 protein leucine aminopeptidase 3, chloroplast precursor, putative, expressed no original description 	T
29.5.11.4.2	protein.degradation.ubiquitin.E3.RING	MDP0000278350	 highly similar to ( 510) AT5G62090 | Symbols:  | FUNCTIONS IN: molecular_function unknown; INVOLVED IN: biological_process unknown; LOCATED IN: cellular_component unknown; EXPRESSED IN: 17 plant structures; EXPRESSED DURING: 10 growth stages; BEST Arabidopsis thaliana protein match is: SLK1 (SEUSS-LIKE 1); transcription regulator (TAIR:AT4G25520.1); Has 38202 Blast hits to 15459 proteins in 742 species: Archae - 6; Bacteria - 1392; Metazoa - 14682; Fungi - 3752; Plants - 2341; Viruses - 407; Other Eukaryotes - 15622 (source: NCBI BLink). | chr5:24935221-24938540 REVERSEmoderately similar to ( 395) loc_os06g03600 12006.m05092 protein transcriptional corepressor SEUSS, putative, expressed   no original description 	T
35.2	not assigned.unknown	MDP0000141448	 weakly similar to ( 112) AT5G26230 | Symbols:  | unknown protein | chr5:9173517-9174542 REVERSEvery weakly similar to (72.4) loc_os05g32440 12005.m07477 protein expressed protein  no original description 	T
20.1	stress.biotic	MDP0000278475	 weakly similar to ( 189) AT5G24620 | Symbols:  | thaumatin-like protein, putative | chr5:8430918-8432289 FORWARDweakly similar to ( 122) TLP2_PRUPE Thaumatin-like protein 2 precursor (PpAZ8) - Prunus persica (Peach)weakly similar to ( 187) loc_os09g32280 12009.m06317 protein pathogenesis-related protein 5 precursor, putative   no original description 	T
35.2	not assigned.unknown	MDP0000222628	 weakly similar to ( 152) AT5G20190 | Symbols:  | binding | chr5:6814093-6815171 FORWARDweakly similar to ( 131) loc_os06g40440 12006.m08580 protein expressed protein   no original description 	T
35.2	not assigned.unknown	MDP0000341582	  no original description 	T
29.5.11.4.3.2	protein.degradation.ubiquitin.E3.SCF.FBOX	MDP0000525440	 highly similar to ( 693) AT5G42350 | Symbols:  | kelch repeat-containing F-box family protein | chr5:16932106-16933797 FORWARDmoderately similar to ( 389) loc_os09g38300 12009.m06771 protein kelch motif family protein, expressed  no original description 	T
30.7	signalling.14-3-3 proteins	MDP0000018128	 moderately similar to ( 377) AT1G34760 | Symbols: GRF11, GF14 OMICRON | GRF11 (GENERAL REGULATORY FACTOR 11); ATPase binding / amino acid binding / protein binding / protein phosphorylated amino acid binding | chr1:12743981-12745603 REVERSEmoderately similar to ( 374) 1433D_SOYBN 14-3-3-like protein D (SGF14D) - Glycine max (Soybean)moderately similar to ( 334) loc_os03g50290 12003.m35391 protein 14-3-3-like protein S94, putative, expressed  no original description 	T
34.1	transport.p- and v-ATPases	MDP0000602147	 nearly identical (1623) AT1G68710 | Symbols:  | haloacid dehalogenase-like hydrolase family protein | chr1:25793498-25797975 REVERSEvery weakly similar to (66.6) ACA2_ORYSA Calcium-transporting ATPase 2, plasma membrane-type (EC 3.6.3.8) (Ca(2+)-ATPase isoform 2) - Oryza sativa (Rice)nearly identical (1523) loc_os06g29380 12006.m07482 protein phospholipid-transporting ATPase 12, putative, expressed  PRK08026  no original description 	T
30.2.11	signalling.receptor kinases.leucine rich repeat XI	MDP0000201265	 moderately similar to ( 386) AT3G19320 | Symbols:  | leucine-rich repeat family protein | chr3:6696395-6698073 REVERSEweakly similar to ( 101) RPK1_IPONI Receptor-like protein kinase precursor (EC 2.7.11.1) - Ipomoea nil (Japanese morning glory) (Pharbitis nil)moderately similar to ( 324) loc_os04g42620 12004.m09229 protein protein binding protein, putative, expressed   no original description 	T
10.5.3	cell wall.cell wall proteins.LRR	MDP0000201265	 moderately similar to ( 386) AT3G19320 | Symbols:  | leucine-rich repeat family protein | chr3:6696395-6698073 REVERSEweakly similar to ( 101) RPK1_IPONI Receptor-like protein kinase precursor (EC 2.7.11.1) - Ipomoea nil (Japanese morning glory) (Pharbitis nil)moderately similar to ( 324) loc_os04g42620 12004.m09229 protein protein binding protein, putative, expressed   no original description 	T
26.13	misc.acid and other phosphatases	MDP0000376018	 weakly similar to ( 190) AT5G50400 | Symbols: ATPAP27, PAP27 | PAP27 (PURPLE ACID PHOSPHATASE 27); acid phosphatase/ protein serine/threonine phosphatase | chr5:20523575-20526231 REVERSEweakly similar to ( 146) NPP_HORVU Nucleotide pyrophosphatase/phosphodiesterase (EC 3.-.-.-) (Fragments) - Hordeum vulgare (Barley)moderately similar to ( 414) loc_os01g58640 12001.m12006 protein nucleotide pyrophosphatase/phosphodiesterase, putative, expressed no original description 	T
34.3	transport.amino acids	MDP0000688191	 nearly identical (1197) AT1G15690 | Symbols: AVP1, ATAVP3, AVP-3 | AVP1; ATPase/ hydrogen-translocating pyrophosphatase | chr1:5399115-5402185 FORWARDnearly identical (1191) AVP_HORVU Pyrophosphate-energized vacuolar membrane proton pump (EC 3.6.1.1) (Pyrophosphate-energized inorganic pyrophosphatase) (H(+)-PPase) - Hordeum vulgare (Barley)nearly identical (1187) loc_os06g08080 12006.m31844 protein pyrophosphate-energized vacuolar membrane proton pump, putative, expressed   no original description 	T
26.7	misc.oxidases - copper, flavone etc	MDP0000158853	 highly similar to ( 524) AT4G29720 | Symbols: ATPAO5 | ATPAO5 (Polyamine oxidase 5); amine oxidase/ electron carrier/ oxidoreductase | chr4:14553456-14555057 REVERSEvery weakly similar to (69.7) PAO_MAIZE Polyamine oxidase precursor (EC 1.5.3.11) - Zea mays (Maize)moderately similar to ( 361) loc_os01g51320 12001.m11313 protein peroxisomal N1-acetyl-spermine/spermidine oxidase, putative, expressed   no original description 	T
29.5	protein.degradation	MDP0000208437	 nearly identical (1133) AT1G50380 | Symbols:  | prolyl oligopeptidase family protein | chr1:18662480-18666185 FORWARDnearly identical (1071) loc_os06g51410 12006.m091806 protein protease 2, putative, expressed   PRK08026  no original description 	T
26.12	misc.peroxidases	MDP0000611163	 moderately similar to ( 382) AT5G06720 | Symbols:  | peroxidase, putative | chr5:2077567-2078857 REVERSEmoderately similar to ( 384) PERX_TOBAC Lignin-forming anionic peroxidase precursor (EC 1.11.1.7) (TOPA) - Nicotiana tabacum (Common tobacco)moderately similar to ( 325) loc_os03g13200 12003.m06766 protein peroxidase 53 precursor, putative, expressed no original description 	T
28.99	DNA.unspecified	MDP0000146862	 highly similar to ( 612) AT4G34490 | Symbols: ATCAP1, CAP 1, CAP1 | ATCAP1 (ARABIDOPSIS THALIANA CYCLASE ASSOCIATED PROTEIN 1); actin binding | chr4:16484896-16487355 REVERSEhighly similar to ( 546) loc_os03g51250 12003.m10106 protein adenylyl cyclase-associated protein 1, putative, expressed  no original description 	T
27.3.8	RNA.regulation of transcription.C2C2(Zn) DOF zinc finger family	MDP0000547254	 weakly similar to ( 179) AT2G28510 | Symbols:  | Dof-type zinc finger domain-containing protein | chr2:12199383-12200627 REVERSEweakly similar to ( 129) PBF_MAIZE Dof zinc finger protein PBF (Prolamin box-binding factor) - Zea mays (Maize)weakly similar to ( 130) loc_os03g16850 12003.m07117 protein conserved hypothetical protein   no original description 	T
30.11	signalling.light	MDP0000478356	 highly similar to ( 849) AT2G27110 | Symbols: FRS3 | FRS3 (FAR1-related sequence 3); zinc ion binding | chr2:11576969-11580259 REVERSEweakly similar to ( 142) BBM2_BRANA Protein BABY BOOM 2 (BnBBM2) - Brassica napus (Rape)moderately similar to ( 469) loc_os03g45300 12003.m09559 protein transposon protein, putative, unclassified, expressed   no original description 	T
35.2	not assigned.unknown	MDP0000194096	 moderately similar to ( 256) AT3G44150 | Symbols:  | unknown protein | chr3:15887617-15888728 FORWARDmoderately similar to ( 213) loc_os03g44560 12003.m09495 protein expp1 protein precursor, putative, expressed  no original description 	T
35.2	not assigned.unknown	MDP0000198860	 very weakly similar to (79.0) AT3G09110 | Symbols:  | unknown protein | chr3:2794850-2795963 REVERSEvery weakly similar to (59.7) loc_os04g50800 12004.m09977 protein expressed protein no original description 	T
35.2	not assigned.unknown	MDP0000655267	 weakly similar to ( 121) AT1G60870 | Symbols: MEE9 | MEE9 (maternal effect embryo arrest 9) | chr1:22409712-22410227 FORWARD   no original description 	T
27.3.3	RNA.regulation of transcription.AP2/EREBP, APETALA2/Ethylene-responsive element binding protein family	MDP0000675982	 weakly similar to ( 130) AT4G13040 | Symbols:  | AP2 domain-containing transcription factor family protein | chr4:7613720-7615310 FORWARDweakly similar to ( 138) loc_os02g29550 12002.m33690 protein DNA binding protein, putative, expressed  no original description 	T
35.2	not assigned.unknown	MDP0000297964	 moderately similar to ( 325) AT4G21890 | Symbols:  | unknown protein | chr4:11611874-11612674 REVERSEvery weakly similar to (50.4) HSP15_SOYBN 17.6 kDa class I heat shock protein (HSP 17.6-L) - Glycine max (Soybean)weakly similar to ( 136) loc_os07g29770 12007.m079679 protein MYND finger family protein, expressed  no original description 	T
27.3.24	RNA.regulation of transcription.MADS box transcription factor family	MDP0000314765	 moderately similar to ( 253) AT2G45660 | Symbols: AGL20, SOC1 | AGL20 (AGAMOUS-LIKE 20); transcription factor | chr2:18807799-18810193 REVERSEweakly similar to ( 192) MAD50_ORYSA MADS-box transcription factor 50 (OsMADS50) (Protein SUPPRESSOR OF CONSTANS OVEREXPRESSION 1-like) (OsSOC1) (Protein AGAMOUS-like 20) (RMADS208) - Oryza sativa (Rice)weakly similar to ( 175) loc_os10g39130 12010.m21952 protein MADS-box transcription factor 56, putative, expressed no original description 	T
30.5	signalling.G-proteins	MDP0000127031	 moderately similar to ( 330) AT1G52280 | Symbols: AtRABG3d | AtRABG3d (Arabidopsis Rab GTPase homolog G3d); GTP binding | chr1:19468150-19469449 REVERSEmoderately similar to ( 342) RAB7_GOSHI Ras-related protein Rab7 - Gossypium hirsutum (Upland cotton)moderately similar to ( 334) loc_os05g44050 12005.m08531 protein ras-related protein Rab7, putative, expressed  no original description 	T
11.6	lipid metabolism.lipid transfer proteins etc	MDP0000039357	 weakly similar to ( 124) AT2G38540 | Symbols: LP1, LTP1, ATLTP1 | LP1; calmodulin binding | chr2:16130418-16130893 FORWARDweakly similar to ( 141) NLTP2_GOSHI Nonspecific lipid-transfer protein precursor (LTP) (GH3) - Gossypium hirsutum (Upland cotton)weakly similar to ( 117) loc_os12g02330 12012.m04234 protein nonspecific lipid-transfer protein precursor, putative, expressed no original description 	T
29.5.9	protein.degradation.AAA type	MDP0000205276	 highly similar to ( 586) AT4G27680 | Symbols:  | MSP1 protein, putative / intramitochondrial sorting protein, putative | chr4:13821263-13823083 FORWARDweakly similar to ( 162) CDC48_SOYBN Cell division cycle protein 48 homolog (Valosin-containing protein homolog) (VCP) - Glycine max (Soybean)highly similar to ( 548) loc_os06g50050 12006.m32194 protein ATPase family AAA domain-containing protein 1, putative, expressed  no original description 	T
29.5.11.4.3.2	protein.degradation.ubiquitin.E3.SCF.FBOX	MDP0000945767	 moderately similar to ( 376) AT2G24540 | Symbols: AFR | AFR (ATTENUATED FAR-RED RESPONSE) | chr2:10426414-10427532 FORWARDmoderately similar to ( 256) loc_os06g44500 12006.m08978 protein kelch motif family protein, expressed  no original description 	T
27.3.35	RNA.regulation of transcription.bZIP transcription factor family	MDP0000138811	 moderately similar to ( 455) AT2G40950 | Symbols: BZIP17 | BZIP17; DNA binding / transcription activator/ transcription factor | chr2:17087823-17090326 REVERSEweakly similar to ( 120) TGA1B_TOBAC TGACG-sequence-specific DNA-binding protein TGA-1B (TGA1b) (HSBF) (Fragment) - Nicotiana tabacum (Common tobacco)moderately similar to ( 296) loc_os05g34050 12005.m07637 protein DNA binding protein, putative, expressed   PRK08026  no original description 	T
16.2.1.1	secondary metabolism.phenylpropanoids.lignin biosynthesis.PAL	MDP0000388769	 nearly identical (1159) AT2G37040 | Symbols: pal1, ATPAL1 | pal1 (Phe ammonia lyase 1); phenylalanine ammonia-lyase | chr2:15557602-15560237 REVERSEnearly identical (1181) PAL3_PETCR Phenylalanine ammonia-lyase 3 (EC 4.3.1.5) - Petroselinum crispum (Parsley) (Petroselinum hortense)nearly identical (1060) loc_os04g43800 12004.m09339 protein phenylalanine ammonia-lyase, putative, expressed   no original description 	T
17.3.1.2.2	hormone metabolism.brassinosteroid.synthesis-degradation.sterols.SMT2	MDP0000909546	 highly similar to ( 676) AT1G20330 | Symbols: SMT2, CVP1, FRL1 | SMT2 (STEROL METHYLTRANSFERASE 2); S-adenosylmethionine-dependent methyltransferase | chr1:7038968-7040053 REVERSEhighly similar to ( 534) SMT2_ORYSA 24-methylenesterol C-methyltransferase 2 (EC 2.1.1.143) (24-sterol C-methyltransferase 2) (Sterol-C-methyltransferase 2) - Oryza sativa (Rice)highly similar to ( 534) loc_os03g04340 12003.m05964 protein 24-methylenesterol C-methyltransferase 2, putative, expressed   no original description 	T
35.2	not assigned.unknown	MDP0000243608	 moderately similar to ( 258) AT2G25605 | Symbols:  | unknown protein | chr2:10899541-10900875 FORWARDmoderately similar to ( 253) loc_os05g49910 12005.m28019 protein expressed protein   no original description 	T
35.2	not assigned.unknown	MDP0000218460	 moderately similar to ( 294) AT4G15830 | Symbols:  | binding | chr4:8995285-8996548 REVERSEmoderately similar to ( 249) loc_os01g70560 12001.m13102 protein expressed protein  no original description 	T
23.1.2.1	nucleotide metabolism.synthesis.purine.amidophosphoribosyltransferase	MDP0000802170	 highly similar to ( 803) AT4G34740 | Symbols: ATASE2, CIA1, ATPURF2 | ATASE2 (GLN PHOSPHORIBOSYL PYROPHOSPHATE AMIDOTRANSFERASE 2); amidophosphoribosyltransferase | chr4:16574894-16576579 REVERSEhighly similar to ( 783) PUR1_SOYBN Amidophosphoribosyltransferase, chloroplast precursor (EC 2.4.2.14) (Glutamine phosphoribosylpyrophosphate amidotransferase) (ATASE) (GPAT) - Glycine max (Soybean)highly similar to ( 594) loc_os05g35580 12005.m07788 protein amidophosphoribosyltransferase, chloroplast precursor, putative, expressed  no original description 	T
34.8	transport.metabolite transporters at the envelope membrane	MDP0000485007	 highly similar to ( 506) AT4G32390 | Symbols:  | phosphate translocator-related | chr4:15636550-15637602 FORWARDvery weakly similar to (64.7) TPT_SPIOL Triose phosphate/phosphate translocator, chloroplast precursor (cTPT) (p36) (E29) - Spinacia oleracea (Spinach)moderately similar to ( 464) loc_os05g41480 12005.m27616 protein plastidic phosphate translocator-like protein1, putative, expressed   PRK08026  no original description 	T
35.2	not assigned.unknown	MDP0000923954	 moderately similar to ( 442) AT2G45320 | Symbols:  | unknown protein | chr2:18684182-18686107 REVERSEmoderately similar to ( 404) loc_os06g37430 12006.m32102 protein expressed protein  no original description 	T
35.2	not assigned.unknown	MDP0000311319	 moderately similar to ( 290) AT3G08600 | Symbols:  | unknown protein | chr3:2612646-2613596 FORWARDmoderately similar to ( 275) loc_os12g02550 12012.m04256 protein expressed protein   no original description 	T
35.1.41	not assigned.no ontology.hydroxyproline rich proteins	MDP0000298545	 very weakly similar to (66.2) AT3G02120 | Symbols:  | hydroxyproline-rich glycoprotein family protein | chr3:377122-377577 FORWARD no original description 	T
34.8	transport.metabolite transporters at the envelope membrane	MDP0000159173	 highly similar to ( 542) AT3G14400 | Symbols: UBP25 | UBP25 (UBIQUITIN-SPECIFIC PROTEASE 25); ubiquitin thiolesterase/ ubiquitin-specific protease | chr3:4811953-4815210 REVERSEvery weakly similar to (54.7) TPT_MAIZE Triose phosphate/phosphate translocator, chloroplast precursor (cTPT) - Zea mays (Maize)moderately similar to ( 413) loc_os08g04110 12008.m26566 protein phosphate/phosphoenolpyruvate translocator, putative, expressed   no original description 	T
29.2.1.2.2.82	protein.synthesis.ribosomal protein.eukaryotic.60S subunit.P2	MDP0000210824	 very weakly similar to (70.9) AT2G27720 | Symbols:  | 60S acidic ribosomal protein P2 (RPP2A) | chr2:11818696-11819370 FORWARDvery weakly similar to (60.8) RLA2B_MAIZE 60S acidic ribosomal protein P2B - Zea mays (Maize)very weakly similar to (60.5) loc_os05g37330 12005.m07913 protein 60S acidic ribosomal protein P2B, putative, expressed  no original description 	T
34.9	transport.metabolite transporters at the mitochondrial membrane	MDP0000222589	 highly similar to ( 605) AT3G08580 | Symbols: AAC1 | AAC1 (ADP/ATP CARRIER 1); ATP:ADP antiporter/ binding | chr3:2605706-2607030 REVERSEhighly similar to ( 622) ADT1_GOSHI ADP,ATP carrier protein 1, mitochondrial precursor (ADP/ATP translocase 1) (Adenine nucleotide translocator 1) (ANT 1) - Gossypium hirsutum (Upland cotton)highly similar to ( 553) loc_os02g48720 12002.m33839 protein ADP,ATP carrier protein, mitochondrial precursor, putative, expressed  no original description 	T
29.4	protein.postranslational modification	MDP0000309518	 highly similar to ( 587) AT3G58500 | Symbols: PP2A-3, EP7 | PP2A-3 (PROTEIN PHOSPHATASE 2A-3); protein serine/threonine phosphatase | chr3:21635503-21638911 REVERSEhighly similar to ( 582) PP2A_MEDSA Serine/threonine-protein phosphatase PP2A catalytic subunit (EC 3.1.3.16) - Medicago sativa (Alfalfa)highly similar to ( 582) loc_os03g59060 12003.m35470 protein OsPP2Ac-2 - Phosphatase 2A isoform 2 belonging to family 2, expressed  no original description 	T
35.2	not assigned.unknown	MDP0000276664	 weakly similar to ( 154) AT4G13220 | Symbols:  | unknown protein | chr4:7674431-7675275 REVERSEweakly similar to ( 138) loc_os12g17830 12012.m073811 protein expressed protein no original description 	T
30.11	signalling.light	MDP0000186762	 weakly similar to ( 199) AT2G02950 | Symbols: PKS1 | PKS1 (PHYTOCHROME KINASE SUBSTRATE 1); protein binding | chr2:855149-856468 REVERSEvery weakly similar to (66.2) loc_os10g26670 12010.m05606 protein expressed protein no original description 	T
35.2	not assigned.unknown	MDP0000300483	 nearly identical (2257) AT3G13330 | Symbols:  | binding | chr3:4319804-4330061 REVERSEnearly identical (1932) loc_os06g19730 12006.m06632 protein HEAT repeat family protein, expressed   no original description 	T
4.1.10	glycolysis.cytosolic branch.aldolase	MDP0000202904	 weakly similar to ( 162) AT5G03690 | Symbols:  | fructose-bisphosphate aldolase, putative | chr5:963389-964982 REVERSEweakly similar to ( 169) ALF_CICAR Fructose-bisphosphate aldolase, cytoplasmic isozyme (EC 4.1.2.13) - Cicer arietinum (Chickpea) (Garbanzo)weakly similar to ( 161) loc_os01g67860 12001.m12890 protein fructose-bisphosphate aldolase cytoplasmic isozyme, putative, expressed PRK07003 tolA TolA TrbL PRK07735  no original description 	T
1.3.6	PS.calvin cycle.aldolase	MDP0000202904	 weakly similar to ( 162) AT5G03690 | Symbols:  | fructose-bisphosphate aldolase, putative | chr5:963389-964982 REVERSEweakly similar to ( 169) ALF_CICAR Fructose-bisphosphate aldolase, cytoplasmic isozyme (EC 4.1.2.13) - Cicer arietinum (Chickpea) (Garbanzo)weakly similar to ( 161) loc_os01g67860 12001.m12890 protein fructose-bisphosphate aldolase cytoplasmic isozyme, putative, expressed PRK07003 tolA TolA TrbL PRK07735  no original description 	T
27.1	RNA.processing	MDP0000305272	 highly similar to ( 833) AT4G32850 | Symbols: nPAP | nPAP (NUCLEAR POLY(A) POLYMERASE); nucleotidyltransferase/ protein binding | chr4:15850134-15854266 FORWARDhighly similar to ( 852) loc_os06g36360 12006.m31883 protein poly, putative, expressed  no original description 	T
26.7	misc.oxidases - copper, flavone etc	MDP0000239956	 moderately similar to ( 469) AT1G23740 | Symbols:  | oxidoreductase, zinc-binding dehydrogenase family protein | chr1:8398245-8399656 REVERSEweakly similar to ( 108) QORH_SPIOL Chloroplastic quinone-oxidoreductase homolog (EC 1.-.-.-) (ceQORH) - Spinacia oleracea (Spinach)moderately similar to ( 438) loc_os08g29170 12008.m06921 protein quinone oxidoreductase-like protein At1g23740, chloroplast precursor, putative, expressed  no original description 	T
26.10	misc.cytochrome P450	MDP0000280105	 highly similar to ( 510) AT4G31940 | Symbols: CYP82C4 | CYP82C4; electron carrier/ heme binding / iron ion binding / monooxygenase/ oxygen binding | chr4:15452040-15453966 FORWARDhighly similar to ( 587) C82A3_SOYBN Cytochrome P450 82A3 (EC 1.14.-.-) (P450 CP6) - Glycine max (Soybean)moderately similar to ( 353) loc_os09g26960 12009.m05848 protein flavonoid 3-monooxygenase, putative, expressed    no original description 	T
21.6	redox.dismutases and catalases	MDP0000364366	 moderately similar to ( 236) AT5G18100 | Symbols: CSD3 | CSD3 (copper/zinc superoxide dismutase 3); superoxide dismutase | chr5:5987221-5988436 FORWARDmoderately similar to ( 254) SODC2_MESCR Superoxide dismutase [Cu-Zn] 2 (EC 1.15.1.1) - Mesembryanthemum crystallinum (Common ice plant)moderately similar to ( 235) loc_os03g11960 12003.m35095 protein superoxide dismutase 2, putative, expressed  no original description 	T
20.2.3	stress.abiotic.drought/salt	MDP0000187381	 nearly identical (1053) AT4G22120 | Symbols:  | early-responsive to dehydration protein-related / ERD protein-related | chr4:11715976-11719144 REVERSEhighly similar to ( 994) loc_os01g35050 12001.m150613 protein HYP1, putative, expressed   no original description 	T
17.3.1.2.99	hormone metabolism.brassinosteroid.synthesis-degradation.sterols.other	MDP0000413935	 highly similar to ( 749) AT1G58440 | Symbols: XF1, SQE1 | XF1; squalene monooxygenase | chr1:21714094-21717246 REVERSEhighly similar to ( 775) ERG1_PANGI Squalene monooxygenase (EC 1.14.99.7) (Squalene epoxidase) (SE) - Panax ginseng (Korean ginseng)highly similar to ( 811) loc_os03g12910 12003.m101256 protein squalene monooxygenase, putative, expressed    no original description 	T
35.2	not assigned.unknown	MDP0000854541	 very weakly similar to (68.9) AT1G25422 | Symbols:  | unknown protein | chr1:8919087-8919410 FORWARD no original description 	T
35.1	not assigned.no ontology	MDP0000630439	 moderately similar to ( 223) AT1G18720 | Symbols:  | unknown protein | chr1:6458020-6459658 REVERSEweakly similar to ( 194) loc_os02g36870 12002.m33734 protein YGL010w-like protein, putative, expressed no original description 	T
27.3.99	RNA.regulation of transcription.unclassified	MDP0000208667	 moderately similar to ( 335) AT5G63280 | Symbols:  | zinc finger (C2H2 type) family protein | chr5:25367085-25368848 FORWARDmoderately similar to ( 297) loc_os12g13130 12012.m56482 protein zinc finger protein, putative, expressed no original description 	T
27.3.11	RNA.regulation of transcription.C2H2 zinc finger family	MDP0000208667	 moderately similar to ( 335) AT5G63280 | Symbols:  | zinc finger (C2H2 type) family protein | chr5:25367085-25368848 FORWARDmoderately similar to ( 297) loc_os12g13130 12012.m56482 protein zinc finger protein, putative, expressed no original description 	T
20.1.7	stress.biotic.PR-proteins	MDP0000310094	 moderately similar to ( 468) AT5G66900 | Symbols:  | disease resistance protein (CC-NBS-LRR class), putative | chr5:26714931-26717757 REVERSEmoderately similar to ( 335) loc_os12g39620 12012.m073872 protein disease resistance protein, putative, expressed no original description 	T
14.1	S-assimilation.ATPS	MDP0000941173	 highly similar to ( 711) AT1G19920 | Symbols: APS2, ASA1 | APS2; sulfate adenylyltransferase (ATP) | chr1:6914835-6916657 REVERSEhighly similar to ( 601) loc_os03g53230 12003.m10296 protein bifunctional 3-phosphoadenosine 5-phosphosulfate synthetase, putative, expressed  no original description 	T
35.2	not assigned.unknown	MDP0000310920	 moderately similar to ( 469) AT3G54750 | Symbols:  | unknown protein | chr3:20264833-20268416 REVERSEmoderately similar to ( 383) loc_os12g42700 12012.m08049 protein expressed protein   no original description 	T
12.1.1	N-metabolism.nitrate metabolism.NR	MDP0000585462	 nearly identical (1381) AT1G77760 | Symbols: NIA1, GNR1, NR1 | NIA1 (NITRATE REDUCTASE 1); nitrate reductase | chr1:29236005-29239367 REVERSEnearly identical (1434) NIA_CUCMA Nitrate reductase [NADH] (EC 1.7.1.1) (NR) - Cucurbita maxima (Pumpkin) (Winter squash)nearly identical (1286) loc_os08g36480 12008.m07637 protein nitrate reductase 1, putative, expressed   no original description 	T
29.5.11.4.3.3	protein.degradation.ubiquitin.E3.SCF.cullin	MDP0000020140	 nearly identical (1224) AT4G02570 | Symbols: ATCUL1, CUL1, AXR6 | ATCUL1 (ARABIDOPSIS THALIANA CULLIN 1); protein binding | chr4:1129315-1133435 FORWARDnearly identical (1282) loc_os01g27150 12001.m42650 protein cullin-1, putative, expressed  no original description 	T
35.2	not assigned.unknown	MDP0000298322	 moderately similar to ( 219) AT2G20230 | Symbols:  | LOCATED IN: plasma membrane, vacuole; EXPRESSED IN: 24 plant structures; EXPRESSED DURING: 16 growth stages; CONTAINS InterPro DOMAIN/s: Tetraspanin (InterPro:IPR018499); BEST Arabidopsis thaliana protein match is: unknown protein (TAIR:AT4G28770.1); Has 91 Blast hits to 91 proteins in 12 species: Archae - 0; Bacteria - 0; Metazoa - 0; Fungi - 0; Plants - 90; Viruses - 0; Other Eukaryotes - 1 (source: NCBI BLink). | chr2:8725762-8727388 FORWARDweakly similar to ( 105) loc_os06g51480 12006.m32234 protein expressed protein  no original description 	T
35.2	not assigned.unknown	MDP0000579770	 moderately similar to ( 266) AT1G62520 | Symbols:  | unknown protein | chr1:23144506-23145348 FORWARDweakly similar to ( 117) loc_os10g40584 12010.m06826 protein nucleic acid binding protein, putative, expressed  no original description 	T
28.1.3	DNA.synthesis/chromatin structure.histone	MDP0000286160	 moderately similar to ( 210) AT1G08880 | Symbols: HTA5, H2AXA, G-H2AX, GAMMA-H2AX | H2AXA; DNA binding | chr1:2847144-2847676 REVERSEmoderately similar to ( 214) H2AX_CICAR Histone H2AX - Cicer arietinum (Chickpea) (Garbanzo)weakly similar to ( 196) loc_os12g34510 12012.m07245 protein histone H2A, putative, expressed  no original description 	T
26.22	misc.short chain dehydrogenase/reductase (SDR)	MDP0000243676	 moderately similar to ( 417) AT3G06060 | Symbols:  | short-chain dehydrogenase/reductase (SDR) family protein | chr3:1828296-1830160 REVERSEvery weakly similar to (79.7) FABG4_BRANA 3-oxoacyl-[acyl-carrier-protein] reductase 4 (EC 1.1.1.100) (3-ketoacyl-acyl carrier protein reductase 4) (Fragment) - Brassica napus (Rape)moderately similar to ( 395) loc_os02g47350 12002.m09770 protein 3-ketodihydrosphingosine reductase precursor, putative, expressed   no original description 	T
21.1	redox.thioredoxin	MDP0000200569	 moderately similar to ( 391) AT1G76080 | Symbols: ATCDSP32, CDSP32 | CDSP32 (CHLOROPLASTIC DROUGHT-INDUCED STRESS PROTEIN OF 32 KD) | chr1:28548063-28549348 REVERSEvery weakly similar to (59.7) TRXH2_BRANA Thioredoxin H-type 2 (TRX-H-2) - Brassica napus (Rape)moderately similar to ( 351) loc_os07g29410 12007.m29166 protein electron transporter/ thiol-disulfide exchange intermediate, putative, expressed  no original description 	T
29.2.1.2.1.21	protein.synthesis.ribosomal protein.eukaryotic.40S subunit.S21	MDP0000362177	 weakly similar to ( 148) AT5G27700 | Symbols:  | structural constituent of ribosome | chr5:9807541-9808048 REVERSEweakly similar to ( 137) RS21_ORYSA 40S ribosomal protein S21 - Oryza sativa (Rice)weakly similar to ( 129) loc_os03g46490 12003.m09669 protein 40S ribosomal protein S21, putative  no original description 	T
27.3.67	RNA.regulation of transcription.putative transcription regulator	MDP0000277912	 moderately similar to ( 357) AT2G38560 | Symbols: TFIIS | TFIIS (TRANSCRIPT ELONGATION FACTOR IIS); DNA binding / RNA polymerase II transcription elongation factor/ transcription factor | chr2:16134802-16136319 FORWARDmoderately similar to ( 360) loc_os03g60130 12003.m101543 protein transcription elongation factor A protein 2, putative, expressed  no original description 	T
29.2.1.2.1.27	protein.synthesis.ribosomal protein.eukaryotic.40S subunit.S27	MDP0000660188	 weakly similar to ( 136) AT2G47110 | Symbols: UBQ6 | UBQ6; protein binding | chr2:19344701-19345174 FORWARDweakly similar to ( 124) RS27A_SOLTU 40S ribosomal protein S27a - Solanum tuberosum (Potato)weakly similar to ( 129) loc_os01g22490 12001.m08729 protein 40S ribosomal protein S27a, putative, expressed no original description 	T
29.5.11.20	protein.degradation.ubiquitin.proteasom	MDP0000239113	 moderately similar to ( 384) AT2G39990 | Symbols: EIF2 | EIF2; translation initiation factor | chr2:16698332-16699929 REVERSEmoderately similar to ( 372) loc_os05g01450 12005.m27663 protein eukaryotic translation initiation factor 3 subunit 5, putative, expressed   no original description 	T
33.99	development.unspecified	MDP0000159656	 moderately similar to ( 246) AT4G27880 | Symbols:  | seven in absentia (SINA) family protein | chr4:13883623-13884927 FORWARDweakly similar to ( 118) SINAL_DROME Probable E3 ubiquitin-protein ligase sina-like CG13030 (EC 6.3.2.-) - Drosophila melanogaster (Fruit fly)moderately similar to ( 251) loc_os05g14860 12005.m05897 protein ubiquitin ligase SINAT3, putative, expressed  no original description 	T
35.2	not assigned.unknown	MDP0000319971	 moderately similar to ( 358) AT1G57680 | Symbols:  | FUNCTIONS IN: molecular_function unknown; INVOLVED IN: biological_process unknown; LOCATED IN: endomembrane system; EXPRESSED IN: 24 plant structures; EXPRESSED DURING: 15 growth stages; CONTAINS InterPro DOMAIN/s: Uncharacterised conserved protein UCP031277 (InterPro:IPR016971); Has 35 Blast hits to 35 proteins in 10 species: Archae - 0; Bacteria - 0; Metazoa - 0; Fungi - 0; Plants - 33; Viruses - 0; Other Eukaryotes - 2 (source: NCBI BLink). | chr1:21362749-21363837 REVERSEmoderately similar to ( 270) loc_os01g66190 12001.m42778 protein expressed protein no original description 	T
34.99	transport.misc	MDP0000154046	 moderately similar to ( 438) AT2G17500 | Symbols:  | auxin efflux carrier family protein | chr2:7607063-7608990 FORWARDmoderately similar to ( 375) loc_os09g38130 12009.m060062 protein auxin Efflux Carrier family protein, expressed  no original description 	T
26.28	misc.GDSL-motif lipase	MDP0000444114	 highly similar to ( 521) AT3G04290 | Symbols: ATLTL1, LTL1 | LTL1 (LI-TOLERANT LIPASE 1); carboxylesterase/ hydrolase, acting on ester bonds | chr3:1133620-1136223 REVERSEweakly similar to ( 174) APG_BRANA Anter-specific proline-rich protein APG (Protein CEX) (Fragment) - Brassica napus (Rape)moderately similar to ( 493) loc_os02g40440 12002.m09130 protein anther-specific proline-rich protein APG precursor, putative, expressed  no original description 	T
35.2	not assigned.unknown	MDP0000531576	 weakly similar to ( 134) AT3G25400 | Symbols:  | FUNCTIONS IN: molecular_function unknown; INVOLVED IN: biological_process unknown; LOCATED IN: cellular_component unknown; EXPRESSED IN: 11 plant structures; EXPRESSED DURING: 6 growth stages; CONTAINS InterPro DOMAIN/s: NTP Pyrophosphohydrolase MazG-related, RS21-C6 (InterPro:IPR011394), EAR (InterPro:IPR009039), NTP pyrophosphohydrolase MazG, putative catalytic core (InterPro:IPR004518); Has 572 Blast hits to 572 proteins in 181 species: Archae - 11; Bacteria - 286; Metazoa - 71; Fungi - 1; Plants - 31; Viruses - 0; Other Eukaryotes - 172 (source: NCBI BLink). | chr3:9213236-9214144 FORWARDweakly similar to ( 137) loc_os04g43550 12004.m09315 protein mazG nucleotide pyrophosphohydrolase domain protein, putative, expressed no original description 	T
26.1	misc.misc2	MDP0000161121	 moderately similar to ( 202) AT4G02340 | Symbols:  | epoxide hydrolase, putative | chr4:1035722-1037403 FORWARDmoderately similar to ( 370) loc_os10g35520 12010.m06376 protein epoxide hydrolase 2, putative, expressed  no original description 	T
26.5	misc.acyl transferases	MDP0000916748	 highly similar to ( 610) AT1G80410 | Symbols: EMB2753 | EMB2753 (EMBRYO DEFECTIVE 2753); binding | chr1:30227963-30234832 REVERSEhighly similar to ( 587) loc_os01g43030 12001.m43094 protein NMDA receptor-regulated 1-like protein, putative, expressed no original description 	T
29.2.1.2.2.13	protein.synthesis.ribosomal protein.eukaryotic.60S subunit.L13	MDP0000279617	 moderately similar to ( 304) AT3G49010 | Symbols: ATBBC1, BBC1, RSU2 | ATBBC1 (ARABIDOPSIS THALIANA BREAST BASIC CONSERVED 1); structural constituent of ribosome | chr3:18166971-18168047 REVERSEmoderately similar to ( 299) RL131_BRANA 60S ribosomal protein L13-1 (Cold-induced protein C24A) - Brassica napus (Rape)moderately similar to ( 298) loc_os03g37970 12003.m34891 protein 60S ribosomal protein L13-2, putative, expressed  no original description 	T
35.2	not assigned.unknown	MDP0000345084	  no original description 	T
35.2	not assigned.unknown	MDP0000636503	 very weakly similar to (59.7) AT1G71970 | Symbols:  | unknown protein | chr1:27093099-27093776 FORWARD  no original description 	T
33.99	development.unspecified	MDP0000120881	 moderately similar to ( 296) AT3G15510 | Symbols: ATNAC2, ANAC056, NARS1 | ATNAC2 (ARABIDOPSIS NAC DOMAIN CONTAINING PROTEIN 2); transcription factor | chr3:5243696-5245037 FORWARDmoderately similar to ( 241) NAC48_ORYSA NAC domain-containing protein 48 (ONAC048) - Oryza sativa (Rice)moderately similar to ( 281) loc_os07g37920 12007.m08041 protein NAM-like protein, putative, expressed  MSP1_C  no original description 	T
35.1	not assigned.no ontology	MDP0000302390	 moderately similar to ( 316) AT5G06570 | Symbols:  | hydrolase | chr5:2008075-2011013 REVERSEweakly similar to ( 149) GID1_ORYSA Gibberellin receptor GID1 (EC 3.-.-.-) (Gibberellin-insensitive dwarf protein 1) (Protein GIBBERELLIN INSENSITIVE DWARF1) - Oryza sativa (Rice)moderately similar to ( 361) loc_os07g06880 12007.m05148 protein gibberellin receptor GID1L2, putative, expressed  no original description 	T
13.2.2.1	amino acid metabolism.degradation.glutamate family.glutamine	MDP0000335197	 moderately similar to ( 296) AT5G13780 | Symbols:  | GCN5-related N-acetyltransferase, putative | chr5:4447610-4448603 REVERSEmoderately similar to ( 277) loc_os04g54330 12004.m101668 protein N-terminal acetyltransferase complex ARD1 subunit homolog A, putative, expressed  no original description 	T
11.1.12	lipid metabolism.FA synthesis and FA elongation.ACP protein	MDP0000608278	 weakly similar to ( 175) AT1G65290 | Symbols: mtACP2 | mtACP2 (mitochondrial acyl carrier protein 2); acyl carrier/ metal ion binding | chr1:24249088-24250366 REVERSEvery weakly similar to (60.1) ACP5_BRANA Acyl carrier protein, chloroplast precursor (ACP) (Clones 28F10, 10H11/11D11, 34F12 and 04F05/05E01) - Brassica napus (Rape)weakly similar to ( 166) loc_os03g22950 12003.m07693 protein acyl carrier protein, mitochondrial precursor, putative, expressed  no original description 	T
2.1.2	major CHO metabolism.synthesis.starch	MDP0000121520	 highly similar to ( 619) AT5G18480 | Symbols: PGSIP6 | PGSIP6 (PLANT GLYCOGENIN-LIKE STARCH INITIATION PROTEIN 6); transferase, transferring glycosyl groups / transferase, transferring hexosyl groups | chr5:6131307-6133787 REVERSEhighly similar to ( 530) loc_os02g41520 12002.m09188 protein glycosyl transferase family 8 protein, expressed   no original description 	T
15.2	metal handling.binding, chelation and storage	MDP0000267323	 very weakly similar to (68.9) AT5G63530 | Symbols: ATFP3, FP3 | ATFP3; metal ion binding / transition metal ion binding | chr5:25433423-25435614 FORWARDvery weakly similar to (95.5) loc_os10g14870 12010.m04684 protein ATFP3, putative, expressed no original description 	T
29.5.11.4.3.2	protein.degradation.ubiquitin.E3.SCF.FBOX	MDP0000176775	 very weakly similar to (73.6) AT5G07610 | Symbols:  | F-box family protein | chr5:2406068-2407330 FORWARDweakly similar to ( 101) loc_os03g56500 12003.m10559 protein expressed protein   no original description 	T
21.4	redox.glutaredoxins	MDP0000317056	 weakly similar to ( 172) AT3G21460 | Symbols:  | electron carrier/ protein disulfide oxidoreductase | chr3:7557567-7557875 REVERSEvery weakly similar to (73.9) GLRX_RICCO Glutaredoxin - Ricinus communis (Castor bean)weakly similar to ( 134) loc_os01g27140 12001.m09165 protein glutaredoxin, putative, expressed no original description 	T
30.5	signalling.G-proteins	MDP0000155528	 moderately similar to ( 352) AT4G17530 | Symbols: RAB1C, ATRAB1C, ATRABD2C | ATRAB1C; GTP binding | chr4:9773721-9775424 REVERSEmoderately similar to ( 351) YPTM2_MAIZE GTP-binding protein YPTM2 - Zea mays (Maize)moderately similar to ( 357) loc_os01g08450 12001.m07470 protein GTP-binding protein YPTM2, putative, expressed no original description 	T
30.2.3	signalling.receptor kinases.leucine rich repeat III	MDP0000223726	 moderately similar to ( 374) AT4G23740 | Symbols:  | leucine-rich repeat transmembrane protein kinase, putative | chr4:12367063-12369159 FORWARDweakly similar to ( 109) PSKR_DAUCA Phytosulfokine receptor precursor (EC 2.7.11.1) (Phytosulfokine LRR receptor kinase) - Daucus carota (Carrot)moderately similar to ( 310) loc_os01g12390 12001.m07854 protein ATP binding protein, putative, expressed  no original description 	T
27.3.15	RNA.regulation of transcription.CCAAT box binding factor family, HAP3	MDP0000324584	 moderately similar to ( 236) AT5G23090 | Symbols: NF-YB13 | NF-YB13 (NUCLEAR FACTOR Y, SUBUNIT B13); transcription factor | chr5:7749391-7750203 FORWARDvery weakly similar to (67.4) NFYB2_ORYSA Nuclear transcription factor Y subunit B-2 (OsNF-YB-2) (Transcriptional activator HAP3A) - Oryza sativa (Rice)moderately similar to ( 212) loc_os08g29500 12008.m06954 protein repressor protein, putative, expressed no original description 	T
26.21	misc.protease inhibitor/seed storage/lipid transfer protein (LTP) family protein	MDP0000215774	 weakly similar to ( 116) AT3G22142 | Symbols:  | structural constituent of cell wall | chr3:7803604-7808046 REVERSEvery weakly similar to (77.8) 14KD_DAUCA 14 kDa proline-rich protein DC2.15 precursor - Daucus carota (Carrot)weakly similar to ( 102) loc_os06g07220 12006.m05445 protein 36.4 kDa proline-rich protein, putative, expressed  no original description 	T
33.99	development.unspecified	MDP0000534460	 moderately similar to ( 406) AT5G44730 | Symbols:  | haloacid dehalogenase-like hydrolase family protein | chr5:18045588-18046519 REVERSEmoderately similar to ( 415) loc_os06g45440 12006.m32138 protein rhythmically expressed gene 2 protein, putative, expressed no original description 	T
35.2	not assigned.unknown	MDP0000592035	 highly similar to ( 911) AT1G73930 | Symbols:  | unknown protein | chr1:27795347-27797534 REVERSEhighly similar to ( 729) loc_os03g15390 12003.m06976 protein expressed protein  no original description 	T
13.1.6.1.1	amino acid metabolism.synthesis.aromatic aa.chorismate.3-deoxy-D-arabino-heptulosonate 7-phosphate synthase	MDP0000573537	 moderately similar to ( 341) AT4G33510 | Symbols: DHS2 | DHS2 (3-deoxy-d-arabino-heptulosonate 7-phosphate synthase); 3-deoxy-7-phosphoheptulonate synthase | chr4:16116496-16118233 FORWARDmoderately similar to ( 350) AROF_TOBAC Phospho-2-dehydro-3-deoxyheptonate aldolase 1, chloroplast precursor (EC 2.5.1.54) (Phospho-2-keto-3-deoxyheptonate aldolase 1) (DAHP synthetase 1) (3-deoxy-D-arabino-heptulosonate 7-phosphate synthase 1) - Nicotiana tabacum (Common tobmoderately similar to ( 349) loc_os07g42960 12007.m29007 protein phospho-2-dehydro-3-deoxyheptonate aldolase 1, chloroplast precursor, putative, expressed  no original description 	T
16.1.2.6	secondary metabolism.isoprenoids.mevalonate pathway.mevalonate diphosphate decarboxylase	MDP0000232665	 highly similar to ( 515) AT3G54250 | Symbols:  | mevalonate diphosphate decarboxylase, putative | chr3:20082468-20084688 REVERSEmoderately similar to ( 494) loc_os02g01920 12002.m05542 protein diphosphomevalonate decarboxylase, putative, expressed  no original description 	T
29.5.11.4.5.2	protein.degradation.ubiquitin.E3.BTB/POZ Cullin3.BTB/POZ	MDP0000286567	 moderately similar to ( 428) AT5G67480 | Symbols: BT4 | BT4 (BTB AND TAZ DOMAIN PROTEIN 4); protein binding / transcription regulator | chr5:26931055-26932529 REVERSEmoderately similar to ( 330) loc_os01g66890 12001.m43320 protein protein binding protein, putative, expressed  no original description 	T
29.5.5	protein.degradation.serine protease	MDP0000201966	 highly similar to ( 663) AT1G28110 | Symbols: SCPL45 | SCPL45 (SERINE CARBOXYPEPTIDASE-LIKE 45 PRECURSOR); serine-type carboxypeptidase | chr1:9804153-9806832 REVERSEmoderately similar to ( 294) CBP2_WHEAT Serine carboxypeptidase 2 (EC 3.4.16.6) (Serine carboxypeptidase II) (Carboxypeptidase D) (CPDW-II) (CP-WII) [Contains: Serine carboxypeptidase 2 chain A (Serine carboxypeptidase II chain A); Serine carboxypeptidase 2 chain B (Serine cahighly similar to ( 616) loc_os11g31980 12011.m07100 protein serine carboxypeptidase 1 precursor, putative, expressed MSP1_C  no original description 	T
26.10	misc.cytochrome P450	MDP0000149604	 highly similar to ( 533) AT5G24910 | Symbols: CYP714A1 | CYP714A1; electron carrier/ heme binding / iron ion binding / monooxygenase/ oxygen binding | chr5:8567674-8570260 REVERSEmoderately similar to ( 240) C72A1_CATRO Cytochrome P450 72A1 (EC 1.3.3.9) (CYPLXXII) (Secologanin synthase) (SLS) - Catharanthus roseus (Rosy periwinkle) (Madagascar periwinkle)moderately similar to ( 422) loc_os12g02640 12012.m04263 protein cytochrome P450 72A1, putative, expressed  no original description 	T
27.3.25	RNA.regulation of transcription.MYB domain transcription factor family	MDP0000210970	 moderately similar to ( 469) AT2G02820 | Symbols: MYB88 | MYB88 (myb domain protein 88); DNA binding / transcription factor | chr2:804716-807139 REVERSEvery weakly similar to (82.8) MYB4_ORYSA Myb-related protein Myb4 (OsMyb4) (Transcription factor RLTR1) - Oryza sativa (Rice)moderately similar to ( 303) loc_os07g43420 12007.m08570 protein myb, DNA-binding, putative, expressed   no original description 	T
29.5.11.4.2	protein.degradation.ubiquitin.E3.RING	MDP0000675059	 moderately similar to ( 216) AT5G64920 | Symbols: CIP8 | CIP8 (COP1-INTERACTING PROTEIN 8); protein binding / zinc ion binding | chr5:25944338-25945342 REVERSEvery weakly similar to (60.5) EL5_ORYSA E3 ubiquitin-protein ligase EL5 (EC 6.3.2.-) - Oryza sativa (Rice)weakly similar to ( 139) loc_os12g02620 12012.m26702 protein protein binding protein, putative, expressed    no original description 	T
30.2.17	signalling.receptor kinases.DUF 26	MDP0000267609	 highly similar to ( 896) AT1G11410 | Symbols:  | S-locus protein kinase, putative | chr1:3841286-3844284 FORWARDmoderately similar to ( 343) SLSG3_BRAOL S-locus-specific glycoprotein S13 precursor (SLSG-13) (Fragment) - Brassica oleracea (Wild cabbage)highly similar to ( 699) loc_os03g35600 12003.m08696 protein serine/threonine-protein kinase receptor precursor, putative, expressed   no original description 	T
30.2.24	signalling.receptor kinases.S-locus glycoprotein like	MDP0000267609	 highly similar to ( 896) AT1G11410 | Symbols:  | S-locus protein kinase, putative | chr1:3841286-3844284 FORWARDmoderately similar to ( 343) SLSG3_BRAOL S-locus-specific glycoprotein S13 precursor (SLSG-13) (Fragment) - Brassica oleracea (Wild cabbage)highly similar to ( 699) loc_os03g35600 12003.m08696 protein serine/threonine-protein kinase receptor precursor, putative, expressed   no original description 	T
11.8	lipid metabolism.'exotics'(steroids, squalene etc)	MDP0000857608	 moderately similar to ( 443) AT5G07010 | Symbols: ATST2A, ST2A | ST2A (SULFOTRANSFERASE 2A); hydroxyjasmonate sulfotransferase/ sulfotransferase | chr5:2174960-2176039 REVERSEmoderately similar to ( 271) loc_os02g45890 12002.m09626 protein flavonol 4-sulfotransferase, putative, expressed  no original description 	T
8.1.7	TCA / org transformation.TCA.succinate dehydrogenase	MDP0000439248	 moderately similar to ( 429) AT3G27380 | Symbols: SDH2-1 | SDH2-1; electron carrier/ succinate dehydrogenase | chr3:10131209-10132673 REVERSEmoderately similar to ( 388) loc_os08g02640 12008.m26820 protein succinate dehydrogenase iron-sulfur protein,mitochondrial precursor, putative, expressed  no original description 	T
27.3.1	RNA.regulation of transcription.ABI3/VP1-related B3-domain-containing transcription factor family	MDP0000288808	 moderately similar to ( 203) AT2G46870 | Symbols: NGA1 | NGA1 (NGATHA1); transcription factor | chr2:19261313-19262245 FORWARDvery weakly similar to (52.4) VIV1_MAIZE Regulatory protein viviparous-1 - Zea mays (Maize)moderately similar to ( 202) loc_os03g02900 12003.m05828 protein B3 DNA binding domain containing protein, expressed COG3889  no original description 	T
34.2	transport.sugars	MDP0000141961	 highly similar to ( 640) AT3G18830 | Symbols: ATPLT5 | ATPLT5 (POLYOL TRANSPORTER 5); D-ribose transmembrane transporter/ D-xylose transmembrane transporter/ carbohydrate transmembrane transporter/ galactose transmembrane transporter/ glucose transmembrane transporter/ glycerol transmembrane transporter/ mann | chr3:6489000-6491209 REVERSEweakly similar to ( 174) HEX6_RICCO Hexose carrier protein HEX6 - Ricinus communis (Castor bean)highly similar to ( 614) loc_os07g39350 12007.m08177 protein proton myo-inositol cotransporter, putative, expressed   no original description 	T
35.2	not assigned.unknown	MDP0000320681	 moderately similar to ( 331) AT3G21865 | Symbols: PEX22 | PEX22 (peroxin 22); protein binding | chr3:7701308-7703218 REVERSEmoderately similar to ( 300) loc_os04g53690 12004.m10262 protein expressed protein   no original description 	T
34.9	transport.metabolite transporters at the mitochondrial membrane	MDP0000169921	 moderately similar to ( 426) AT4G39460 | Symbols: SAMC1, SAMT1 | SAMC1 (S-ADENOSYLMETHIONINE CARRIER 1); S-adenosylmethionine transmembrane transporter/ binding | chr4:18356093-18358596 REVERSEvery weakly similar to (86.7) BT1_MAIZE Protein brittle-1, chloroplast precursor - Zea mays (Maize)moderately similar to ( 392) loc_os05g29860 12005.m27795 protein mitochondrial carrier C12B10.09, putative, expressed no original description 	T
35.2	not assigned.unknown	MDP0000167324	 moderately similar to ( 292) AT3G01680 | Symbols:  | unknown protein | chr3:252033-255246 FORWARD  no original description 	T
13.1.6.3.1	amino acid metabolism.synthesis.aromatic aa.phenylalanine.arogenate dehydratase / prephenate dehydratase	MDP0000134397	 moderately similar to ( 395) AT2G27820 | Symbols: PD1, ADT3 | PD1 (PREPHENATE DEHYDRATASE 1); arogenate dehydratase/ prephenate dehydratase | chr2:11856808-11858082 FORWARDmoderately similar to ( 361) loc_os09g39260 12009.m06865 protein prephenate dehydratase, putative, expressed   no original description 	T
29.5.11.20	protein.degradation.ubiquitin.proteasom	MDP0000219632	 highly similar to ( 729) AT5G19990 | Symbols: RPT6A, ATSUG1 | RPT6A (REGULATORY PARTICLE TRIPLE-A ATPASE 6A); ATPase | chr5:6752144-6754918 FORWARDmoderately similar to ( 333) PRS7_PRUPE 26S protease regulatory subunit 7 (26S proteasome subunit 7) (26S proteasome AAA-ATPase subunit RPT1) (Regulatory particle triple-A ATPase subunit 1) - Prunus persica (Peach)highly similar to ( 704) loc_os02g11050 12002.m06403 protein 26S protease regulatory subunit 8, putative, expressed  no original description 	T
29.5.9	protein.degradation.AAA type	MDP0000219632	 highly similar to ( 729) AT5G19990 | Symbols: RPT6A, ATSUG1 | RPT6A (REGULATORY PARTICLE TRIPLE-A ATPASE 6A); ATPase | chr5:6752144-6754918 FORWARDmoderately similar to ( 333) PRS7_PRUPE 26S protease regulatory subunit 7 (26S proteasome subunit 7) (26S proteasome AAA-ATPase subunit RPT1) (Regulatory particle triple-A ATPase subunit 1) - Prunus persica (Peach)highly similar to ( 704) loc_os02g11050 12002.m06403 protein 26S protease regulatory subunit 8, putative, expressed  no original description 	T
9.9	mitochondrial electron transport / ATP synthesis.F1-ATPase 	MDP0000945182	 highly similar to ( 917) AT4G38510 | Symbols:  | vacuolar ATP synthase subunit B, putative / V-ATPase B subunit, putative / vacuolar proton pump B subunit, putative / V-ATPase 57 kDa subunit, putative | chr4:18011155-18014789 REVERSEhighly similar to ( 908) VATB1_HORVU Vacuolar ATP synthase subunit B isoform 1 (EC 3.6.3.14) (V-ATPase B subunit 1) (Vacuolar proton pump B subunit 1) - Hordeum vulgare (Barley)highly similar to ( 912) loc_os01g51380 12001.m150697 protein vacuolar ATP synthase subunit B isoform 1, putative, expressed MSP1_C  no original description 	T
34.1.1.1	transport.p- and v-ATPases.H+-transporting two-sector ATPase.subunit B	MDP0000945182	 highly similar to ( 917) AT4G38510 | Symbols:  | vacuolar ATP synthase subunit B, putative / V-ATPase B subunit, putative / vacuolar proton pump B subunit, putative / V-ATPase 57 kDa subunit, putative | chr4:18011155-18014789 REVERSEhighly similar to ( 908) VATB1_HORVU Vacuolar ATP synthase subunit B isoform 1 (EC 3.6.3.14) (V-ATPase B subunit 1) (Vacuolar proton pump B subunit 1) - Hordeum vulgare (Barley)highly similar to ( 912) loc_os01g51380 12001.m150697 protein vacuolar ATP synthase subunit B isoform 1, putative, expressed MSP1_C  no original description 	T
17.5.1	hormone metabolism.ethylene.synthesis-degradation	MDP0000906115	 weakly similar to ( 181) AT2G36690 | Symbols:  | oxidoreductase, 2OG-Fe(II) oxygenase family protein | chr2:15379930-15381987 FORWARDweakly similar to ( 120) FLS_PETHY Flavonol synthase/flavanone 3-hydroxylase (EC 1.14.11.23) (EC 1.14.11.9) (FLS) - Petunia hybrida (Petunia)weakly similar to ( 198) loc_os08g37456 12008.m080186 protein flavonol synthase/flavanone 3-hydroxylase, putative, expressed no original description 	T
34.2	transport.sugars	MDP0000154362	 highly similar to ( 723) AT1G77210 | Symbols:  | sugar transporter, putative | chr1:29009036-29010980 REVERSEhighly similar to ( 540) STA_RICCO Sugar carrier protein A - Ricinus communis (Castor bean)highly similar to ( 685) loc_os09g15330 12009.m059918 protein sugar transport protein 14, putative, expressed  no original description 	T
31.5.1	cell.cell death.plants	MDP0000315206	 highly similar to ( 749) AT3G44880 | Symbols: ACD1, LLS1, PAO | ACD1 (ACCELERATED CELL DEATH 1); iron-sulfur cluster binding / pheophorbide a oxygenase | chr3:16383858-16386204 FORWARDvery weakly similar to (65.1) CAO_CHLRE Chlorophyllide a oxygenase, chloroplast precursor (EC 1.13.12.14) (Chlorophyll a oxygenase) (Chlorophyll b synthase) - Chlamydomonas reinhardtiihighly similar to ( 744) loc_os03g05310 12003.m06055 protein pheophorbide a oxygenase, chloroplast precursor, putative, expressed   no original description 	T
29.2.1.2.1.23	protein.synthesis.ribosomal protein.eukaryotic.40S subunit.S23	MDP0000245646	 moderately similar to ( 267) AT5G02960 | Symbols:  | 40S ribosomal protein S23 (RPS23B) | chr5:693280-694396 REVERSEmoderately similar to ( 275) RS23_FRAAN 40S ribosomal protein S23 (S12) - Fragaria ananassa (Strawberry)moderately similar to ( 275) loc_os03g60400 12003.m10930 protein 40S ribosomal protein S23, putative, expressed   no original description 	T
30.3	signalling.calcium	MDP0000238367	 moderately similar to ( 230) AT3G49260 | Symbols: iqd21 | iqd21 (IQ-domain 21); calmodulin binding | chr3:18262755-18265859 FORWARDvery weakly similar to (99.4) loc_os05g03190 12005.m04853 protein IQD1, putative, expressed  MSP1_C  no original description 	T
27.3.68	RNA.regulation of transcription.PWWP domain protein	MDP0000153032	 moderately similar to ( 201) AT3G54760 | Symbols:  | dentin sialophosphoprotein-related | chr3:20269659-20272037 REVERSEmoderately similar to ( 288) loc_os05g38810 12005.m08060 protein PWWP domain containing protein, expressed      no original description 	T
29.5.3	protein.degradation.cysteine protease	MDP0000315645	 weakly similar to ( 124) AT3G12490 | Symbols: ATCYSB | cysteine protease inhibitor, putative / cystatin, putative | chr3:3960523-3961777 REVERSEweakly similar to ( 121) CYTI_VIGUN Cysteine proteinase inhibitor (Cystatin) - Vigna unguiculata (Cowpea)weakly similar to ( 122) loc_os01g16430 12001.m08239 protein multidomain cystatin, putative, expressed  no original description 	T
35.2	not assigned.unknown	MDP0000172356	 very weakly similar to (60.5) AT1G16630 | Symbols:  | unknown protein | chr1:5682212-5684749 REVERSEvery weakly similar to (58.9) loc_os03g46920 12003.m09707 protein expressed protein   MSP1_C TrbL  no original description 	T
35.2	not assigned.unknown	MDP0000215367	 moderately similar to ( 484) AT2G42570 | Symbols:  | unknown protein | chr2:17717498-17719921 REVERSEmoderately similar to ( 421) loc_os01g46350 12001.m10833 protein expressed protein  no original description 	T
34.22	transport.cyclic nucleotide or calcium regulated channels	MDP0000141136	 nearly identical (1092) AT5G53130 | Symbols: CNGC1, ATCNGC1 | CNGC1 (CYCLIC NUCLEOTIDE GATED CHANNEL 1); calmodulin binding / cation channel/ cyclic nucleotide binding / intracellular cyclic nucleotide activated cation channel/ inward rectifier potassium channel | chr5:21537830-21540490 REVERSEhighly similar to ( 850) loc_os06g33570 12006.m07894 protein cyclic nucleotide-gated ion channel 1, putative, expressed  no original description 	T
34.99	transport.misc	MDP0000177114	 highly similar to ( 513) AT3G21690 | Symbols:  | MATE efflux family protein | chr3:7638750-7641861 FORWARDmoderately similar to ( 473) loc_os03g37640 12003.m35293 protein transparent testa 12 protein, putative, expressed   no original description 	T
35.2	not assigned.unknown	MDP0000169563	 moderately similar to ( 289) AT4G15830 | Symbols:  | binding | chr4:8995285-8996548 REVERSEmoderately similar to ( 244) loc_os01g70560 12001.m13102 protein expressed protein  no original description 	T
29.2.1.2.2.38	protein.synthesis.ribosomal protein.eukaryotic.60S subunit.L38	MDP0000951483	 weakly similar to ( 131) AT3G59540 | Symbols:  | 60S ribosomal protein L38 (RPL38B) | chr3:21995897-21996742 REVERSEweakly similar to ( 129) loc_os11g24610 12011.m06387 protein 60S ribosomal protein L38, putative, expressed no original description 	T
27.3.26	RNA.regulation of transcription.MYB-related transcription factor family	MDP0000241185	 moderately similar to ( 237) AT1G49010 | Symbols:  | myb family transcription factor | chr1:18132714-18133778 FORWARDmoderately similar to ( 206) loc_os01g34060 12001.m09717 protein DNA binding protein, putative, expressed  no original description 	T
35.2	not assigned.unknown	MDP0000171737	 moderately similar to ( 445) AT1G79270 | Symbols: ECT8 | ECT8 (evolutionarily conserved C-terminal region 8) | chr1:29816157-29818811 FORWARDmoderately similar to ( 447) loc_os01g48790 12001.m42694 protein YT521-B-like family protein, expressed  no original description 	T
35.2	not assigned.unknown	MDP0000367358	  no original description 	T
35.1	not assigned.no ontology	MDP0000122340	 moderately similar to ( 488) AT5G51570 | Symbols:  | band 7 family protein | chr5:20949511-20951234 FORWARDmoderately similar to ( 454) loc_os06g04460 12006.m05176 protein hypersensitive-induced reaction protein 4, putative, expressed   no original description 	T
29.2.1.2.2.518	protein.synthesis.ribosomal protein.eukaryotic.60S subunit.L18A	MDP0000132567	 moderately similar to ( 349) AT2G34480 | Symbols:  | 60S ribosomal protein L18A (RPL18aB) | chr2:14532916-14534161 REVERSEmoderately similar to ( 338) RL18A_ORYSA 60S ribosomal protein L18a - Oryza sativa (Rice)moderately similar to ( 338) loc_os01g54870 12001.m11648 protein 60S ribosomal protein L18a, putative, expressed no original description 	T
17.5.1	hormone metabolism.ethylene.synthesis-degradation	MDP0000281562	 moderately similar to ( 417) AT1G06650 | Symbols:  | 2-oxoglutarate-dependent dioxygenase, putative | chr1:2035909-2037186 FORWARDmoderately similar to ( 362) DV4H_CATRO Desacetoxyvindoline 4-hydroxylase (EC 1.14.11.20) - Catharanthus roseus (Rosy periwinkle) (Madagascar periwinkle)moderately similar to ( 330) loc_os04g10350 12004.m06287 protein 1-aminocyclopropane-1-carboxylate oxidase, putative, expressed no original description 	T
29.2.1.2.1.30	protein.synthesis.ribosomal protein.eukaryotic.40S subunit.S30	MDP0000329265	 weakly similar to ( 103) AT5G56670 | Symbols:  | 40S ribosomal protein S30 (RPS30C) | chr5:22935413-22935981 REVERSEweakly similar to ( 104) loc_os06g07580 12006.m05481 protein expressed protein no original description 	T
8.1.9	TCA / org transformation.TCA.malate DH	MDP0000159872	 highly similar to ( 568) AT3G47520 | Symbols: MDH | MDH (MALATE DEHYDROGENASE); L-malate dehydrogenase/ binding / catalytic/ malate dehydrogenase/ oxidoreductase/ oxidoreductase, acting on the CH-OH group of donors, NAD or NADP as acceptor | chr3:17513657-17514868 FORWARDmoderately similar to ( 405) MDHG_ORYSA Malate dehydrogenase, glyoxysomal precursor (EC 1.1.1.37) - Oryza sativa (Rice)highly similar to ( 569) loc_os08g33720 12008.m07362 protein malate dehydrogenase, mitochondrial precursor, putative, expressed  no original description 	T
6.3	gluconeogenesis / glyoxylate cycle.Malate DH	MDP0000159872	 highly similar to ( 568) AT3G47520 | Symbols: MDH | MDH (MALATE DEHYDROGENASE); L-malate dehydrogenase/ binding / catalytic/ malate dehydrogenase/ oxidoreductase/ oxidoreductase, acting on the CH-OH group of donors, NAD or NADP as acceptor | chr3:17513657-17514868 FORWARDmoderately similar to ( 405) MDHG_ORYSA Malate dehydrogenase, glyoxysomal precursor (EC 1.1.1.37) - Oryza sativa (Rice)highly similar to ( 569) loc_os08g33720 12008.m07362 protein malate dehydrogenase, mitochondrial precursor, putative, expressed  no original description 	T
27.3.4	RNA.regulation of transcription.ARF, Auxin Response Factor family	MDP0000194603	 highly similar to ( 652) AT4G23980 | Symbols: ARF9 | ARF9 (AUXIN RESPONSE FACTOR 9); transcription factor | chr4:12451592-12454737 FORWARDvery weakly similar to (58.5) IAA20_ORYSA Auxin-responsive protein IAA20 (Indoleacetic acid-induced protein 20) - Oryza sativa (Rice)highly similar to ( 594) loc_os02g35140 12002.m08602 protein auxin response factor 1, putative, expressed   no original description 	T
29.2.1.1.3.1.21	protein.synthesis.ribosomal protein.prokaryotic.unknown organellar.30S subunit.S21	MDP0000700350	 weakly similar to ( 153) AT3G27160 | Symbols: GHS1 | GHS1 (GLUCOSE HYPERSENSITIVE 1); structural constituent of ribosome | chr3:10017531-10018854 FORWARDvery weakly similar to (63.2) RR21_SPIOL Chloroplast 30S ribosomal protein S21 (Fragment) - Spinacia oleracea (Spinach)very weakly similar to (97.8) loc_os02g09590 12002.m06256 protein ribosome-like protein, putative, expressed   no original description 	T
20.2.99	stress.abiotic.unspecified	MDP0000132530	 weakly similar to ( 118) AT1G14930 | Symbols:  | major latex protein-related / MLP-related | chr1:5152465-5153035 REVERSEvery weakly similar to (71.6) loc_os04g39150 12004.m35333 protein major latex protein 22, putative, expressed no original description 	T
35.2	not assigned.unknown	MDP0000314967	 very weakly similar to (87.8) AT5G46020 | Symbols:  | unknown protein | chr5:18663075-18664629 REVERSEweakly similar to ( 101) loc_os01g54920 12001.m150439 protein heat- and acid-stable phosphoprotein, putative, expressed no original description 	T
35.2	not assigned.unknown	MDP0000241680	 weakly similar to ( 198) AT3G24160 | Symbols: PMP | PMP (PUTATIVE TYPE 1 MEMBRANE PROTEIN) | chr3:8726241-8729023 FORWARDweakly similar to ( 123) loc_os08g45220 12008.m26803 protein expressed protein  no original description 	T
20.2.3	stress.abiotic.drought/salt	MDP0000279220	 highly similar to ( 870) AT1G30360 | Symbols: ERD4 | ERD4 (early-responsive to dehydration 4) | chr1:10715892-10718799 FORWARDhighly similar to ( 817) loc_os07g05570 12007.m29066 protein ERD4 protein, putative, expressed  no original description 	T
27.3.21	RNA.regulation of transcription.GRAS transcription factor family	MDP0000247514	 moderately similar to ( 482) AT3G49950 | Symbols:  | scarecrow transcription factor family protein | chr3:18522570-18523802 FORWARDweakly similar to ( 115) NSP1_MEDTR Nodulation signaling pathway 1 protein - Medicago truncatula (Barrel medic)highly similar to ( 526) loc_os07g40020 12007.m08244 protein GRAS family transcription factor containing protein, expressed  no original description 	T
35.2	not assigned.unknown	MDP0000260553	 very weakly similar to (77.4) AT3G20898 | Symbols:  | unknown protein | chr3:7323796-7324203 FORWARD no original description 	T
29.5.5	protein.degradation.serine protease	MDP0000247270	 moderately similar to ( 443) AT2G27920 | Symbols: SCPL51 | SCPL51 (SERINE CARBOXYPEPTIDASE-LIKE 51); serine-type carboxypeptidase | chr2:11885777-11888732 REVERSEvery weakly similar to (65.9) CBP1_ORYSA Serine carboxypeptidase 1 precursor (EC 3.4.16.5) (Serine carboxypeptidase I) (Carboxypeptidase C) - Oryza sativa (Rice)moderately similar to ( 395) loc_os08g44640 12008.m26797 protein retinoid-inducible serine carboxypeptidase precursor, putative, expressed no original description 	T
27.3.71	RNA.regulation of transcription.SNF7	MDP0000323552	 moderately similar to ( 316) AT1G73030 | Symbols: VPS46.2 | VPS46.2 | chr1:27473938-27474848 FORWARDmoderately similar to ( 299) loc_os06g43590 12006.m091745 protein charged multivesicular body protein 1b, putative, expressed  no original description 	T
29.5.5	protein.degradation.serine protease	MDP0000073873	 highly similar to ( 734) AT5G42240 | Symbols: scpl42 | scpl42 (serine carboxypeptidase-like 42); serine-type carboxypeptidase | chr5:16888717-16890931 FORWARDmoderately similar to ( 329) CBP23_HORVU Serine carboxypeptidase II-3 precursor (EC 3.4.16.6) (CP-MII.3) [Contains: Serine carboxypeptidase II-3 chain A; Serine carboxypeptidase II-3 chain B] - Hordeum vulgare (Barley)highly similar to ( 705) loc_os11g10750 12011.m05268 protein serine carboxypeptidase F13S12.6 precursor, putative, expressed  no original description 	T
10.8.1	cell wall.pectin*esterases.PME	MDP0000370611	 highly similar to ( 594) AT1G11580 | Symbols: ATPMEPCRA, PMEPCRA | PMEPCRA (METHYLESTERASE PCR A); enzyme inhibitor/ pectinesterase | chr1:3888730-3890649 FORWARDmoderately similar to ( 429) PME1_CITSI Pectinesterase-1 precursor (EC 3.1.1.11) (Pectin methylesterase) (PE) - Citrus sinensis (Sweet orange)moderately similar to ( 497) loc_os01g57854 12001.m11933 protein pectinesterase-1 precursor, putative, expressed   no original description 	T
35.2	not assigned.unknown	MDP0000903665	 very weakly similar to (92.8) AT3G17950 | Symbols:  | unknown protein | chr3:6146651-6147169 FORWARDvery weakly similar to (61.6) loc_os06g14530 12006.m06168 protein expressed protein    no original description 	T
23.3.3	nucleotide metabolism.salvage.NUDIX hydrolases	MDP0000767960	 weakly similar to ( 108) AT5G45940 | Symbols: atnudt11 | atnudt11 (Arabidopsis thaliana Nudix hydrolase homolog 11); hydrolase | chr5:18629837-18630597 REVERSEweakly similar to ( 110) loc_os08g28860 12008.m06891 protein peroxisomal coenzyme A diphosphatase NUDT7, putative, expressed no original description 	T
35.1	not assigned.no ontology	MDP0000052269	 highly similar to ( 927) AT2G01970 | Symbols:  | endomembrane protein 70, putative | chr2:452197-454819 REVERSEhighly similar to ( 897) loc_os03g13380 12003.m06783 protein transmembrane 9 superfamily protein member 1 precursor, putative, expressed no original description 	T
35.2	not assigned.unknown	MDP0000500080	 moderately similar to ( 393) AT3G01670 | Symbols:  | unknown protein | chr3:247288-250261 FORWARDvery weakly similar to (51.6) EXTN_TOBAC Extensin precursor (Cell wall hydroxyproline-rich glycoprotein) - Nicotiana tabacum (Common tobacco)very weakly similar to (50.1) loc_os01g67390 12001.m12843 protein extensin-1 precursor, putative, expressed   no original description 	T
27.3.3	RNA.regulation of transcription.AP2/EREBP, APETALA2/Ethylene-responsive element binding protein family	MDP0000403580	 moderately similar to ( 280) AT1G53910 | Symbols: RAP2.12 | RAP2.12; DNA binding / transcription factor | chr1:20135242-20136581 FORWARDmoderately similar to ( 211) ERF1_ORYSA Ethylene-responsive transcription factor 1 (Ethylene-responsive element-binding factor 1) (EREBP-1) (OsEREBP1) - Oryza sativa (Rice)moderately similar to ( 211) loc_os02g54160 12002.m10445 protein ethylene response element binding protein, putative, expressed  no original description 	T
35.2	not assigned.unknown	MDP0000826832	 moderately similar to ( 377) AT1G48460 | Symbols:  | unknown protein | chr1:17911469-17913149 FORWARDweakly similar to ( 170) loc_os01g50830 12001.m11264 protein expressed protein   no original description 	T
35.1	not assigned.no ontology	MDP0000665070	 moderately similar to ( 246) AT3G12030 | Symbols:  | unknown protein | chr3:3834465-3835055 REVERSEweakly similar to ( 198) loc_os03g51160 12003.m101475 protein fb27, putative, expressed  no original description 	T
4.1.13	glycolysis.cytosolic branch.enolase	MDP0000179036	 highly similar to ( 785) AT2G36530 | Symbols: LOS2 | LOS2; copper ion binding / phosphopyruvate hydratase | chr2:15321081-15323786 REVERSEhighly similar to ( 818) ENO_RICCO Enolase (EC 4.2.1.11) (2-phosphoglycerate dehydratase) (2-phospho-D-glycerate hydro-lyase) - Ricinus communis (Castor bean)highly similar to ( 797) loc_os03g14450 12003.m101271 protein enolase 2, putative, expressed  no original description 	T
7.3	OPP.electron transfer	MDP0000593523	 highly similar to ( 618) AT1G30510 | Symbols: ATRFNR2 | ATRFNR2 (ROOT FNR 2); FAD binding / NADP or NADPH binding / electron carrier/ ferredoxin-NADP+ reductase/ oxidoreductase | chr1:10807150-10808984 REVERSEhighly similar to ( 656) FENR2_PEA Ferredoxin--NADP reductase, root isozyme, chloroplast precursor (EC 1.18.1.2) (FNR) - Pisum sativum (Garden pea)highly similar to ( 625) loc_os03g57120 12003.m35441 protein ferredoxin--NADP reductase, root isozyme, chloroplast precursor, putative, expressed no original description 	T
35.1	not assigned.no ontology	MDP0000131288	 highly similar to ( 504) AT5G57100 | Symbols:  | transporter-related | chr5:23106563-23108440 REVERSEvery weakly similar to (51.2) TPT_MAIZE Triose phosphate/phosphate translocator, chloroplast precursor (cTPT) - Zea mays (Maize)moderately similar to ( 275) loc_os02g49260 12002.m09960 protein expressed protein no original description 	T
29.2.1.2.2.32	protein.synthesis.ribosomal protein.eukaryotic.60S subunit.L32	MDP0000230607	 weakly similar to ( 199) AT4G18100 | Symbols:  | 60S ribosomal protein L32 (RPL32A) | chr4:10035715-10036475 REVERSEvery weakly similar to (71.2) RL32_MAIZE 60S ribosomal protein L32 (Fragment) - Zea mays (Maize)moderately similar to ( 207) loc_os08g41300 12008.m08108 protein 60S ribosomal protein L32, putative, expressed no original description 	T
27.3.26	RNA.regulation of transcription.MYB-related transcription factor family	MDP0000234807	 moderately similar to ( 228) AT5G67580 | Symbols: TRB2, ATTRB2, TBP3, ATTBP3 | TRB2; DNA binding / double-stranded telomeric DNA binding / protein homodimerization/ single-stranded telomeric DNA binding / transcription factor | chr5:26955843-26957073 REVERSEmoderately similar to ( 212) loc_os12g41920 12012.m26974 protein single myb histone 6, putative, expressed no original description 	T
34.13	transport.peptides and oligopeptides	MDP0000213770	 highly similar to ( 864) AT2G40460 | Symbols:  | proton-dependent oligopeptide transport (POT) family protein | chr2:16897123-16901171 FORWARDhighly similar to ( 582) loc_os03g04570 12003.m05985 protein peptide transporter PTR2, putative, expressed   no original description 	T
35.2	not assigned.unknown	MDP0000734649	 very weakly similar to (60.5) AT5G14410 | Symbols:  | unknown protein | chr5:4646897-4647398 REVERSEvery weakly similar to (50.4) loc_os01g71210 12001.m13164 protein expressed protein  no original description 	T
35.2	not assigned.unknown	MDP0000237180	 very weakly similar to (57.8) AT5G14410 | Symbols:  | unknown protein | chr5:4646897-4647398 REVERSE  no original description 	T
27.3.22	RNA.regulation of transcription.HB,Homeobox transcription factor family	MDP0000239684	 moderately similar to ( 361) AT4G34610 | Symbols: BLH6 | BLH6 (BELL1-LIKE HOMEODOMAIN 6); DNA binding / transcription factor | chr4:16530546-16532498 REVERSEvery weakly similar to (62.4) HLG3_MAIZE Homeobox protein liguleless 3 (Fragment) - Zea mays (Maize)moderately similar to ( 426) loc_os12g43950 12012.m073725 protein BEL1-related homeotic protein 30, putative, expressed  no original description 	T
27.3.22	RNA.regulation of transcription.HB,Homeobox transcription factor family	MDP0000304106	 moderately similar to ( 298) AT1G75430 | Symbols: BLH11 | BLH11 (BEL1-LIKE HOMEODOMAIN 11); transcription factor | chr1:28308121-28309517 REVERSEvery weakly similar to (63.2) RSH1_MAIZE Homeobox protein rough sheath 1 - Zea mays (Maize)moderately similar to ( 244) loc_os03g06930 12003.m06210 protein DNA binding protein, putative, expressed  no original description 	T
30.1	signalling.in sugar and nutrient physiology	MDP0000158045	 moderately similar to ( 416) AT4G08950 | Symbols: EXO | EXO (EXORDIUM) | chr4:5740378-5741322 FORWARDmoderately similar to ( 362) loc_os02g52040 12002.m10235 protein phi-1-like phosphate-induced protein, putative, expressed  no original description 	T
29.1.11	protein.aa activation.serine-tRNA ligase	MDP0000120290	 highly similar to ( 714) AT5G27470 | Symbols:  | seryl-tRNA synthetase / serine--tRNA ligase | chr5:9695087-9697154 FORWARDhighly similar to ( 746) SYS_HELAN Seryl-tRNA synthetase (EC 6.1.1.11) (Serine--tRNA ligase) (SerRS) - Helianthus annuus (Common sunflower)highly similar to ( 645) loc_os03g10190 12003.m06472 protein seryl-tRNA synthetase, putative, expressed no original description 	T
2.2.1.3.1	major CHO metabolism.degradation.sucrose.invertases.neutral	MDP0000261740	 highly similar to ( 817) AT5G22510 | Symbols:  | beta-fructofuranosidase, putative / invertase, putative / saccharase, putative / beta-fructosidase, putative | chr5:7474974-7477479 REVERSEhighly similar to ( 783) loc_os02g32730 12002.m08366 protein alkaline/neutral invertase, putative, expressed  no original description 	T
26.12	misc.peroxidases	MDP0000633623	 moderately similar to ( 268) AT5G14930 | Symbols: SAG101 | SAG101 (SENESCENCE-ASSOCIATED GENE 101); carboxylesterase/ triacylglycerol lipase | chr5:4828754-4830165 FORWARDvery weakly similar to (64.7) loc_os09g22450 12009.m05451 protein EDS1-like protein, putative, expressed   no original description 	T
35.2	not assigned.unknown	MDP0000177788	 nearly identical (1042) AT2G30600 | Symbols:  | BTB/POZ domain-containing protein | chr2:13037410-13041475 FORWARDhighly similar to ( 899) loc_os05g27880 12005.m27783 protein BTB/POZ domain containing protein, expressed  no original description 	T
35.1	not assigned.no ontology	MDP0000127844	 moderately similar to ( 429) AT4G37470 | Symbols:  | hydrolase, alpha/beta fold family protein | chr4:17617045-17618363 REVERSEmoderately similar to ( 419) loc_os03g32270 12003.m08471 protein sigma factor sigB regulation protein rsbQ, putative, expressed  no original description 	T
29.4	protein.postranslational modification	MDP0000253129	 highly similar to ( 539) AT4G38470 | Symbols:  | protein kinase family protein | chr4:17999432-18003551 FORWARDweakly similar to ( 118) NORK_MEDTR Nodulation receptor kinase precursor (EC 2.7.11.1) (Does not make infections protein 2) (Symbiosis receptor-like kinase) (MtSYMRK) - Medicago truncatula (Barrel medic)highly similar to ( 506) loc_os09g37230 12009.m22182 protein ATP binding protein, putative, expressed   no original description 	T
29.2.2.50	protein.synthesis.ribosome biogenesis.BRIX	MDP0000277059	 moderately similar to ( 432) AT3G23620 | Symbols:  | brix domain-containing protein | chr3:8480147-8481873 FORWARDmoderately similar to ( 410) BXDC1_ORYSA Brix domain-containing protein 1 homolog - Oryza sativa (Rice)moderately similar to ( 410) loc_os01g33030 12001.m09620 protein brix domain-containing protein 1, putative, expressed  no original description 	T
35.2	not assigned.unknown	MDP0000188386	 very weakly similar to (94.0) AT5G59080 | Symbols:  | unknown protein | chr5:23847691-23848403 REVERSE no original description 	T
16.8.5.1	secondary metabolism.flavonoids.isoflavones.isoflavone reductase	MDP0000601559	 moderately similar to ( 417) AT1G32100 | Symbols: ATPRR1, PRR1 | PRR1 (PINORESINOL REDUCTASE 1); pinoresinol reductase | chr1:11546472-11547953 REVERSEmoderately similar to ( 243) IFRH_SOLTU Isoflavone reductase homolog (EC 1.3.1.-) (CP100) - Solanum tuberosum (Potato)moderately similar to ( 409) loc_os12g16410 12012.m26884 protein isoflavone reductase, putative, expressed no original description 	T
26.28	misc.GDSL-motif lipase	MDP0000255711	 moderately similar to ( 409) AT1G09390 | Symbols:  | GDSL-motif lipase/hydrolase family protein | chr1:3031264-3033415 FORWARDmoderately similar to ( 221) EST_HEVBR Esterase precursor (EC 3.1.1.-) (Early nodule-specific protein homolog) (Latex allergen Hev b 13) - Hevea brasiliensis (Para rubber tree)moderately similar to ( 306) loc_os01g22660 12001.m42994 protein alpha-L-fucosidase 2 precursor, putative, expressed   no original description 	T
28.1	DNA.synthesis/chromatin structure	MDP0000340069	 moderately similar to ( 346) AT1G74560 | Symbols: NRP1 | NRP1 (NAP1-RELATED PROTEIN 1); DNA binding / chromatin binding / histone binding | chr1:28017763-28019900 REVERSEmoderately similar to ( 304) loc_os02g36710 12002.m08758 protein protein SET, putative, expressed  no original description 	T
29.2.4	protein.synthesis.elongation	MDP0000420164	 highly similar to ( 852) AT5G60390 | Symbols:  | elongation factor 1-alpha / EF-1-alpha | chr5:24289226-24290675 FORWARDhighly similar to ( 858) EF1A_MAIZE Elongation factor 1-alpha (EF-1-alpha) - Zea mays (Maize)highly similar to ( 857) loc_os03g08050 12003.m78808 protein elongation factor 1-alpha, putative, expressed   no original description 	T
34.13	transport.peptides and oligopeptides	MDP0000231369	 highly similar to ( 785) AT1G59740 | Symbols:  | proton-dependent oligopeptide transport (POT) family protein | chr1:21968227-21972312 FORWARDhighly similar to ( 664) loc_os11g12740 12011.m05466 protein peptide transporter PTR2, putative, expressed  no original description 	T
26.10	misc.cytochrome P450	MDP0000692160	 moderately similar to ( 480) AT4G31940 | Symbols: CYP82C4 | CYP82C4; electron carrier/ heme binding / iron ion binding / monooxygenase/ oxygen binding | chr4:15452040-15453966 FORWARDmoderately similar to ( 430) C82A1_PEA Cytochrome P450 82A1 (EC 1.14.-.-) (CYPLXXXII) (Fragment) - Pisum sativum (Garden pea)moderately similar to ( 350) loc_os09g26940 12009.m05846 protein flavonoid 3-monooxygenase, putative, expressed   no original description 	T
29.2.1.2.2.12	protein.synthesis.ribosomal protein.eukaryotic.60S subunit.L12	MDP0000689049	 moderately similar to ( 295) AT5G60670 | Symbols:  | 60S ribosomal protein L12 (RPL12C) | chr5:24381066-24381566 REVERSEmoderately similar to ( 240) RL12_CHLRE 60S ribosomal protein L12 (Fragment) - Chlamydomonas reinhardtiimoderately similar to ( 307) loc_os04g50990 12004.m09996 protein 60S ribosomal protein L12, putative, expressed  no original description 	T
35.2	not assigned.unknown	MDP0000263183	 moderately similar to ( 258) AT4G10090 | Symbols:  | unknown protein | chr4:6305455-6307286 FORWARDweakly similar to ( 187) loc_os03g17560 12003.m07181 protein expressed protein  no original description 	T
34.99	transport.misc	MDP0000314057	 highly similar to ( 937) AT2G27810 | Symbols:  | xanthine/uracil permease family protein | chr2:11852338-11855411 FORWARDhighly similar to ( 642) loc_os01g63870 12001.m12502 protein permease, putative, expressed  no original description 	T
30.2.11	signalling.receptor kinases.leucine rich repeat XI	MDP0000159463	 nearly identical (1309) AT5G07180 | Symbols: ERL2 | ERL2 (ERECTA-LIKE 2); kinase | chr5:2227787-2233232 REVERSEmoderately similar to ( 446) RPK1_IPONI Receptor-like protein kinase precursor (EC 2.7.11.1) - Ipomoea nil (Japanese morning glory) (Pharbitis nil)nearly identical (1247) loc_os06g03970 12006.m05128 protein receptor-like protein kinase 5 precursor, putative, expressed  no original description 	T
35.1	not assigned.no ontology	MDP0000136248	 highly similar to ( 515) AT5G53620 | Symbols:  | unknown protein | chr5:21781217-21785061 FORWARDhighly similar to ( 518) loc_os08g43060 12008.m080226 protein expressed protein   no original description 	T
35.1	not assigned.no ontology	MDP0000248768	 moderately similar to ( 401) AT1G64720 | Symbols: CP5 | CP5 | chr1:24046887-24048537 REVERSEmoderately similar to ( 372) loc_os02g03230 12002.m05673 protein nodule membrane protein, putative, expressed MSP1_C  no original description 	T
26.12	misc.peroxidases	MDP0000706473	 moderately similar to ( 381) AT5G06720 | Symbols:  | peroxidase, putative | chr5:2077567-2078857 REVERSEmoderately similar to ( 326) PERX_TOBAC Lignin-forming anionic peroxidase precursor (EC 1.11.1.7) (TOPA) - Nicotiana tabacum (Common tobacco)moderately similar to ( 337) loc_os03g13200 12003.m06766 protein peroxidase 53 precursor, putative, expressed no original description 	T
27.3.70	RNA.regulation of transcription.Silencing Group	MDP0000219093	 moderately similar to ( 288) AT1G66740 | Symbols: AtSP7, SP7, SGA2, ASF1A | SGA2 | chr1:24892586-24893548 FORWARDmoderately similar to ( 294) loc_os05g48030 12005.m27955 protein anti-silencing protein 1, putative, expressed  no original description 	T
30.2.11	signalling.receptor kinases.leucine rich repeat XI	MDP0000138583	 moderately similar to ( 443) AT2G34930 | Symbols:  | disease resistance family protein | chr2:14737169-14739886 REVERSEmoderately similar to ( 227) RPK1_IPONI Receptor-like protein kinase precursor (EC 2.7.11.1) - Ipomoea nil (Japanese morning glory) (Pharbitis nil)moderately similar to ( 370) loc_os11g35660 12011.m07418 protein leucine-rich repeat receptor protein kinase EXS precursor, putative  no original description 	T
27.3.25	RNA.regulation of transcription.MYB domain transcription factor family	MDP0000854094	 moderately similar to ( 234) AT5G45420 | Symbols:  | myb family transcription factor | chr5:18405668-18406597 REVERSEweakly similar to ( 157) loc_os08g06240 12008.m04763 protein dnajc2 protein, putative, expressed  no original description 	T
34.1.1	transport.p- and v-ATPases.H+-transporting two-sector ATPase	MDP0000494888	 moderately similar to ( 353) AT4G11150 | Symbols: TUF, emb2448, TUFF, VHA-E1 | TUF (VACUOLAR ATP SYNTHASE SUBUNIT E1); proton-transporting ATPase, rotational mechanism | chr4:6800091-6801696 FORWARDmoderately similar to ( 353) VATE_GOSHI Vacuolar ATP synthase subunit E (EC 3.6.3.14) (V-ATPase E subunit) (Vacuolar proton pump E subunit) - Gossypium hirsutum (Upland cotton)moderately similar to ( 356) loc_os01g46980 12001.m10894 protein vacuolar ATP synthase subunit E, putative, expressed  no original description 	T
26.28	misc.GDSL-motif lipase	MDP0000764366	 highly similar to ( 539) AT1G54790 | Symbols:  | GDSL-motif lipase/hydrolase family protein | chr1:20441124-20443997 REVERSEmoderately similar to ( 288) EST_HEVBR Esterase precursor (EC 3.1.1.-) (Early nodule-specific protein homolog) (Latex allergen Hev b 13) - Hevea brasiliensis (Para rubber tree)highly similar to ( 523) loc_os05g33270 12005.m07559 protein esterase precursor, putative, expressed  no original description 	T
29.2.1.2.2.18	protein.synthesis.ribosomal protein.eukaryotic.60S subunit.L18	MDP0000125401	 moderately similar to ( 337) AT3G05590 | Symbols: RPL18 | RPL18 (RIBOSOMAL PROTEIN L18); structural constituent of ribosome | chr3:1621511-1622775 FORWARDmoderately similar to ( 318) RL18_CICAR 60S ribosomal protein L18 (Fragment) - Cicer arietinum (Chickpea) (Garbanzo)moderately similar to ( 323) loc_os03g22180 12003.m07621 protein 60S ribosomal protein L18, putative, expressed  no original description 	T
26.10	misc.cytochrome P450	MDP0000119148	 highly similar to ( 723) AT5G23190 | Symbols: CYP86B1 | CYP86B1; electron carrier/ heme binding / iron ion binding / monooxygenase/ oxygen binding | chr5:7803478-7805659 REVERSEweakly similar to ( 127) C97B2_SOYBN Cytochrome P450 97B2 (EC 1.14.-.-) - Glycine max (Soybean)highly similar to ( 660) loc_os10g34480 12010.m06272 protein cytochrome P450 86A2, putative, expressed  no original description 	T
35.2	not assigned.unknown	MDP0000905600	 very weakly similar to (55.8) AT5G19855 | Symbols:  | unknown protein | chr5:6712166-6713445 REVERSE no original description 	T
27.3.6	RNA.regulation of transcription.bHLH,Basic Helix-Loop-Helix family	MDP0000662999	 moderately similar to ( 381) AT3G26744 | Symbols: ICE1, ATICE1, SCRM | ICE1 (INDUCER OF CBF EXPRESSION 1); DNA binding / transcription activator/ transcription factor | chr3:9832953-9834790 REVERSEvery weakly similar to (60.8) ARRS_MAIZE Anthocyanin regulatory R-S protein - Zea mays (Maize)moderately similar to ( 327) loc_os11g32100 12011.m07112 protein helix-loop-helix DNA-binding, putative, expressed   Sporozoite_P67 SPT5  no original description 	T
35.2	not assigned.unknown	MDP0000197247	 moderately similar to ( 273) AT2G34980 | Symbols: SETH1 | SETH1; phosphatidylinositol N-acetylglucosaminyltransferase/ transferase | chr2:14748967-14749878 FORWARDmoderately similar to ( 257) loc_os02g40870 12002.m33777 protein phosphatidylinositol N-acetylglucosaminyltransferase subunit C, putative, expressed  no original description 	T
17.3.1.2.4	hormone metabolism.brassinosteroid.synthesis-degradation.sterols.FACKEL	MDP0000285817	 moderately similar to ( 490) AT3G52940 | Symbols: FK, HYD2, ELL1 | FK (FACKEL); delta14-sterol reductase | chr3:19631030-19633112 REVERSEmoderately similar to ( 462) loc_os09g39220 12009.m06861 protein delta(14)-sterol reductase, putative, expressed  no original description 	T
35.2	not assigned.unknown	MDP0000145252	 weakly similar to ( 170) AT1G18730 | Symbols: NDF6 | NDF6 (NDH DEPENDENT FLOW 6) | chr1:6460625-6461464 FORWARDweakly similar to ( 140) loc_os02g51020 12002.m10136 protein expressed protein  no original description 	T
26.12	misc.peroxidases	MDP0000267123	 moderately similar to ( 251) AT5G14930 | Symbols: SAG101 | SAG101 (SENESCENCE-ASSOCIATED GENE 101); carboxylesterase/ triacylglycerol lipase | chr5:4828754-4830165 FORWARDvery weakly similar to (55.8) PERP7_BRARA Peroxidase P7 (EC 1.11.1.7) (TP7) - Brassica rapa (Turnip)very weakly similar to (75.5) loc_os06g13050 12006.m06020 protein peroxidase family protein, expressed   no original description 	T
29.5.7	protein.degradation.metalloprotease	MDP0000278259	 highly similar to ( 600) AT1G09300 | Symbols:  | metallopeptidase M24 family protein | chr1:3004485-3008131 FORWARDhighly similar to ( 532) loc_os12g37640 12012.m26934 protein peptidase C12B10.05, putative, expressed   no original description 	T
34.1.1	transport.p- and v-ATPases.H+-transporting two-sector ATPase	MDP0000290370	 highly similar to ( 600) AT3G28715 | Symbols:  | H+-transporting two-sector ATPase, putative | chr3:10778025-10780350 FORWARDhighly similar to ( 588) VA0D_ORYSA Probable vacuolar ATP synthase subunit d (EC 3.6.3.14) (V-ATPase d subunit) (Vacuolar proton pump subunit d) - Oryza sativa (Rice)highly similar to ( 588) loc_os01g40470 12001.m10318 protein vacuolar ATP synthase subunit d, putative, expressed  no original description 	T
29.7	protein.glycosylation	MDP0000297352	 moderately similar to ( 426) AT1G08280 | Symbols:  | glycosyl transferase family 29 protein / sialyltransferase family protein | chr1:2608408-2609604 FORWARDmoderately similar to ( 362) loc_os01g63970 12001.m12512 protein sialyltransferase-like protein, putative, expressed   no original description 	T
35.2	not assigned.unknown	MDP0000254135	 moderately similar to ( 286) AT3G27230 | Symbols:  | LOCATED IN: Golgi apparatus; EXPRESSED IN: 23 plant structures; EXPRESSED DURING: 13 growth stages; BEST Arabidopsis thaliana protein match is: methyltransferase (TAIR:AT5G40830.2); Has 184 Blast hits to 183 proteins in 14 species: Archae - 0; Bacteria - 2; Metazoa - 0; Fungi - 0; Plants - 182; Viruses - 0; Other Eukaryotes - 0 (source: NCBI BLink). | chr3:10054224-10055456 FORWARDvery weakly similar to (87.8) POLX_TOBAC Retrovirus-related Pol polyprotein from transposon TNT 1-94 [Includes: Protease (EC 3.4.23.-); Reverse transcriptase (EC 2.7.7.49); Endonuclease] - Nicotiana tabacum (Common tobacco)weakly similar to ( 178) loc_os06g04680 12006.m091595 protein expressed protein    no original description 	T
23.3.2.1	nucleotide metabolism.salvage.nucleoside kinases.adenosine kinase	MDP0000334474	 highly similar to ( 607) AT5G03300 | Symbols: ADK2 | ADK2 (ADENOSINE KINASE 2); adenosine kinase/ copper ion binding / kinase | chr5:796573-798997 FORWARDmoderately similar to ( 479) ADK_PHYPA Adenosine kinase (EC 2.7.1.20) (AK) (Adenosine 5'-phosphotransferase) - Physcomitrella patens (Moss)highly similar to ( 592) loc_os04g43750 12004.m101587 protein adenosine kinase 2, putative, expressed no original description 	T
35.2	not assigned.unknown	MDP0000463035	 weakly similar to ( 154) AT1G79770 | Symbols:  | unknown protein | chr1:30014371-30014874 FORWARDweakly similar to ( 138) loc_os06g36040 12006.m08141 protein expressed protein   no original description 	T
29.5.11.4.3.2	protein.degradation.ubiquitin.E3.SCF.FBOX	MDP0000176755	 highly similar to ( 552) AT3G54650 | Symbols: FBL17 | FBL17; ubiquitin-protein ligase | chr3:20226004-20228882 REVERSEmoderately similar to ( 468) loc_os03g43390 12003.m09385 protein Leucine Rich Repeat family protein, expressed  no original description 	T
4.1.8	glycolysis.cytosolic branch.glyceraldehyde 3-phosphate dehydrogenase (GAP-DH)	MDP0000757565	 highly similar to ( 556) AT1G13440 | Symbols: GAPC-2, GAPC2 | GAPC2 (GLYCERALDEHYDE-3-PHOSPHATE DEHYDROGENASE C2); NAD or NADH binding / binding / catalytic/ glyceraldehyde-3-phosphate dehydrogenase (phosphorylating)/ glyceraldehyde-3-phosphate dehydrogenase | chr1:4608465-4610494 REVERSEhighly similar to ( 555) G3PC_ANTMA Glyceraldehyde-3-phosphate dehydrogenase, cytosolic (EC 1.2.1.12) - Antirrhinum majus (Garden snapdragon)highly similar to ( 562) loc_os04g40950 12004.m35161 protein glyceraldehyde-3-phosphate dehydrogenase, cytosolic 3, putative, expressed   no original description 	T
35.1	not assigned.no ontology	MDP0000177488	 moderately similar to ( 471) AT2G36290 | Symbols:  | hydrolase, alpha/beta fold family protein | chr2:15208867-15210768 REVERSEmoderately similar to ( 424) loc_os04g33590 12004.m08454 protein catalytic/ hydrolase, putative, expressed  no original description 	T
30.3	signalling.calcium	MDP0000277474	 moderately similar to ( 268) AT3G43810 | Symbols: CAM7 | CAM7 (CALMODULIN 7); calcium ion binding | chr3:15664619-15666355 REVERSEmoderately similar to ( 278) CALM1_SOLTU Calmodulin-1 (CaM-1) - Solanum tuberosum (Potato)moderately similar to ( 270) loc_os07g48780 12007.m29366 protein calmodulin, putative, expressed no original description 	T
35.2	not assigned.unknown	MDP0000135568	 moderately similar to ( 221) AT5G51040 | Symbols:  | unknown protein | chr5:20750700-20751790 FORWARDmoderately similar to ( 205) loc_os11g32480 12011.m07149 protein TPR repeat region family protein, expressed  no original description 	T
29.3.1	protein.targeting.nucleus	MDP0000230603	 nearly identical (1200) AT5G62600 | Symbols:  | transportin-SR-related | chr5:25123337-25132473 REVERSEhighly similar to ( 972) loc_os10g33770 12010.m06205 protein importin-beta N-terminal domain containing protein   no original description 	T
35.1	not assigned.no ontology	MDP0000197580	 moderately similar to ( 287) AT5G53490 | Symbols:  | thylakoid lumenal 17.4 kDa protein, chloroplast | chr5:21723488-21724621 REVERSEmoderately similar to ( 291) loc_os10g35810 12010.m06399 protein uncharacterized low-complexity proteins, putative, expressed  no original description 	T
29.2.1.2.2.99	protein.synthesis.ribosomal protein.eukaryotic.60S subunit.unknown	MDP0000124728	 weakly similar to ( 150) AT5G39800 | Symbols:  | 60S ribosomal protein-related | chr5:15935580-15936936 FORWARDweakly similar to ( 139) loc_os02g40490 12002.m09135 protein conserved hypothetical protein no original description 	T
29.2.1.1.1.1.7	protein.synthesis.ribosomal protein.prokaryotic.chloroplast.30S subunit.S7	MDP0000184861	 moderately similar to ( 355) AT2G37270 | Symbols: ATRPS5B | ATRPS5B (RIBOSOMAL PROTEIN 5B); structural constituent of ribosome | chr2:15647883-15649042 REVERSEmoderately similar to ( 360) RS5_CICAR 40S ribosomal protein S5 (Fragment) - Cicer arietinum (Chickpea) (Garbanzo)moderately similar to ( 353) loc_os11g29190 12011.m080000 protein 40S ribosomal protein S5, putative, expressed no original description 	T
29.2.1.2.1.5	protein.synthesis.ribosomal protein.eukaryotic.40S subunit.S5	MDP0000184861	 moderately similar to ( 355) AT2G37270 | Symbols: ATRPS5B | ATRPS5B (RIBOSOMAL PROTEIN 5B); structural constituent of ribosome | chr2:15647883-15649042 REVERSEmoderately similar to ( 360) RS5_CICAR 40S ribosomal protein S5 (Fragment) - Cicer arietinum (Chickpea) (Garbanzo)moderately similar to ( 353) loc_os11g29190 12011.m080000 protein 40S ribosomal protein S5, putative, expressed no original description 	T
27.3.11	RNA.regulation of transcription.C2H2 zinc finger family	MDP0000776169	 weakly similar to ( 157) AT2G41940 | Symbols: ZFP8 | ZFP8 (ZINC FINGER PROTEIN 8); nucleic acid binding / transcription factor/ zinc ion binding | chr2:17507556-17508329 FORWARDweakly similar to ( 132) loc_os02g53530 12002.m10382 protein zinc finger protein, putative, expressed   no original description 	T
29.4.1.57	protein.postranslational modification.kinase.receptor like cytoplasmatic kinase VII	MDP0000190310	 weakly similar to ( 146) AT4G20270 | Symbols: BAM3 | BAM3 (BARELY ANY MERISTEM 3); ATP binding / protein binding / protein kinase/ protein serine/threonine kinase | chr4:10949822-10952924 FORWARDvery weakly similar to (58.5) NORK_PEA Nodulation receptor kinase precursor (EC 2.7.11.1) - Pisum sativum (Garden pea)weakly similar to ( 170) loc_os11g12620 12011.m05454 protein receptor protein kinase CLAVATA1 precursor, putative, expressed  no original description 	T
29.4.1.57	protein.postranslational modification.kinase.receptor like cytoplasmatic kinase VII	MDP0000513732	 highly similar to ( 843) AT1G66150 | Symbols: TMK1 | TMK1 (TRANSMEMBRANE KINASE 1); transmembrane receptor protein serine/threonine kinase | chr1:24631503-24634415 FORWARDmoderately similar to ( 248) PSKR_DAUCA Phytosulfokine receptor precursor (EC 2.7.11.1) (Phytosulfokine LRR receptor kinase) - Daucus carota (Carrot)highly similar to ( 817) loc_os03g50810 12003.m10067 protein receptor protein kinase TMK1 precursor, putative, expressed TrbL  no original description 	T
30.5	signalling.G-proteins	MDP0000207110	 highly similar to ( 786) AT3G54190 | Symbols:  | FUNCTIONS IN: molecular_function unknown; INVOLVED IN: biological_process unknown; EXPRESSED IN: 22 plant structures; EXPRESSED DURING: 13 growth stages; CONTAINS InterPro DOMAIN/s: WD40 repeat-like (InterPro:IPR011046); BEST Arabidopsis thaliana protein match is: unknown protein (TAIR:AT2G38630.1); Has 77 Blast hits to 77 proteins in 20 species: Archae - 0; Bacteria - 0; Metazoa - 0; Fungi - 0; Plants - 51; Viruses - 0; Other Eukaryotes - 26 (source: NCBI BLink). | chr3:20061759-20063880 REVERSEhighly similar to ( 752) loc_os05g40410 12005.m08217 protein expressed protein no original description 	T
34.15	transport.potassium	MDP0000283526	 highly similar to ( 954) AT1G31120 | Symbols: KUP10 | KUP10; potassium ion transmembrane transporter | chr1:11104375-11107361 REVERSEhighly similar to ( 910) HAK11_ORYSA Probable potassium transporter 11 (OsHAK11) - Oryza sativa (Rice)highly similar to ( 910) loc_os04g52390 12004.m10133 protein potassium transporter 11, putative, expressed   no original description 	T
35.2	not assigned.unknown	MDP0000122690	 moderately similar to ( 277) AT3G13410 | Symbols:  | unknown protein | chr3:4362149-4364032 REVERSEmoderately similar to ( 214) loc_os12g03060 12012.m26817 protein expressed protein  no original description 	T
31.4	cell.vesicle transport	MDP0000773526	 very weakly similar to (80.9) AT5G16490 | Symbols: RIC4 | RIC4 (ROP-INTERACTIVE CRIB MOTIF-CONTAINING PROTEIN 4); protein binding | chr5:5384468-5385205 REVERSEvery weakly similar to (55.8) loc_os03g63060 12003.m11178 protein wiscott-Aldrich syndrome, C-terminal, putative, expressedMSP1_C  no original description 	T
26.7	misc.oxidases - copper, flavone etc	MDP0000120008	 highly similar to ( 928) AT4G12420 | Symbols: SKU5 | SKU5; copper ion binding / oxidoreductase | chr4:7349941-7352868 REVERSEhighly similar to ( 515) ASO_BRANA L-ascorbate oxidase homolog precursor (EC 1.10.3.3) (Ascorbase) - Brassica napus (Rape)highly similar to ( 819) loc_os06g01490 12006.m04887 protein monocopper oxidase precursor, putative, expressed   no original description 	T
27.1	RNA.processing	MDP0000796160	 highly similar to ( 620) AT3G01150 | Symbols: PTB | PTB1 (POLYPYRIMIDINE TRACT-BINDING PROTEIN 1); RNA binding / nucleic acid binding / nucleotide binding | chr3:51732-54344 FORWARDhighly similar to ( 618) loc_os08g33830 12008.m26697 protein polypyrimidine tract-binding protein homolog 1, putative, expressed  no original description 	T
35.2	not assigned.unknown	MDP0000512279	 moderately similar to ( 278) AT1G28520 | Symbols: VOZ1 | VOZ1 (VASCULAR PLANT ONE ZINC FINGER PROTEIN); transcription activator | chr1:10029713-10031479 FORWARDmoderately similar to ( 243) loc_os05g43950 12005.m08521 protein expressed protein no original description 	T
23.3.1.3	nucleotide metabolism.salvage.phosphoribosyltransferases.upp	MDP0000249686	 highly similar to ( 788) AT4G26510 | Symbols:  | ATP binding / kinase/ phosphotransferase, alcohol group as acceptor / uracil phosphoribosyltransferase | chr4:13384503-13387920 FORWARDweakly similar to ( 106) ALF1_PEA Fructose-bisphosphate aldolase, cytoplasmic isozyme 1 (EC 4.1.2.13) - Pisum sativum (Garden pea)highly similar to ( 714) loc_os02g17320 12002.m33637 protein uridine/cytidine kinase-like 1, putative, expressed  no original description 	T
35.2	not assigned.unknown	MDP0000265744	 weakly similar to ( 172) AT1G75060 | Symbols:  | unknown protein | chr1:28181481-28183285 REVERSEmoderately similar to ( 228) loc_os04g08450 12004.m06132 protein expressed protein  no original description 	T
27.3.54	RNA.regulation of transcription.Histone acetyltransferases	MDP0000272113	 nearly identical (1850) AT1G79000 | Symbols: HAC1, PCAT2, ATHPCAT2 | HAC1 (HISTONE ACETYLTRANSFERASE OF THE CBP FAMILY 1); H3/H4 histone acetyltransferase/ histone acetyltransferase/ transcription cofactor | chr1:29716933-29723984 REVERSEnearly identical (1477) loc_os02g04490 12002.m05798 protein HAC1, putative, expressed   no original description 	T
29.2.1.1.1.2.17	protein.synthesis.ribosomal protein.prokaryotic.chloroplast.50S subunit.L17	MDP0000542367	 moderately similar to ( 261) AT3G54210 | Symbols:  | ribosomal protein L17 family protein | chr3:20067672-20068385 REVERSEmoderately similar to ( 258) RK17_TOBAC 50S ribosomal protein L17, chloroplast precursor (CL17) - Nicotiana tabacum (Common tobacco)moderately similar to ( 229) loc_os03g60100 12003.m10904 protein 50S ribosomal protein L17, putative, expressed  PRK07003  no original description 	T
3.8.2	minor CHO metabolism.galactose.alpha-galactosidases	MDP0000274090	 moderately similar to ( 490) AT5G08370 | Symbols: AtAGAL2 | AtAGAL2 (Arabidopsis thaliana ALPHA-GALACTOSIDASE 2); alpha-galactosidase/ catalytic/ hydrolase, hydrolyzing O-glycosyl compounds | chr5:2691116-2693441 REVERSEmoderately similar to ( 461) AGAL_ORYSA Alpha-galactosidase precursor (EC 3.2.1.22) (Melibiase) (Alpha-D-galactoside galactohydrolase) - Oryza sativa (Rice)moderately similar to ( 461) loc_os10g35110 12010.m065201 protein alpha-galactosidase precursor, putative, expressed  MSP1_C  no original description 	T
29.5.11.20	protein.degradation.ubiquitin.proteasom	MDP0000781483	 highly similar to ( 518) AT5G05780 | Symbols: RPN8A, AE3, ATHMOV34 | RPN8A (RP NON-ATPASE SUBUNIT 8A) | chr5:1735862-1738176 FORWARDmoderately similar to ( 211) loc_os04g56646 12004.m10553 protein expressed protein no original description 	T
17.2.3	hormone metabolism.auxin.induced-regulated-responsive-activated	MDP0000203028	 weakly similar to ( 171) AT1G75580 | Symbols:  | auxin-responsive protein, putative | chr1:28377530-28377856 FORWARDvery weakly similar to (89.0) ARG7_PHAAU Indole-3-acetic acid-induced protein ARG7 - Phaseolus aureus (Mung bean) (Vigna radiata)weakly similar to ( 116) loc_os02g24740 12002.m07621 protein OsSAUR9 - Auxin-responsive SAUR gene family member, expressed no original description 	T
34.8	transport.metabolite transporters at the envelope membrane	MDP0000656552	 highly similar to ( 516) AT4G32390 | Symbols:  | phosphate translocator-related | chr4:15636550-15637602 FORWARDvery weakly similar to (60.5) TPT_SPIOL Triose phosphate/phosphate translocator, chloroplast precursor (cTPT) (p36) (E29) - Spinacia oleracea (Spinach)moderately similar to ( 466) loc_os05g41480 12005.m27616 protein plastidic phosphate translocator-like protein1, putative, expressed   no original description 	T
27.4	RNA.RNA binding	MDP0000319817	 highly similar to ( 710) AT1G20200 | Symbols: EMB2719, HAP15 | EMB2719 (EMBRYO DEFECTIVE 2719); enzyme regulator | chr1:7001409-7004154 REVERSEhighly similar to ( 748) PSMD3_DAUCA Probable 26S proteasome non-ATPase regulatory subunit 3 (26S proteasome subunit S3) (Nuclear antigen 21D7) - Daucus carota (Carrot)highly similar to ( 684) loc_os08g43640 12008.m080046 protein 26S proteasome non-ATPase regulatory subunit 3, putative, expressed  TrbL PRK07003  no original description 	T
34.99	transport.misc	MDP0000744772	 moderately similar to ( 279) AT1G14010 | Symbols:  | emp24/gp25L/p24 family protein | chr1:4800385-4801790 REVERSEmoderately similar to ( 280) loc_os07g43890 12007.m08613 protein transmembrane emp24 domain-containing protein 10 precursor, putative, expressed  no original description 	T
35.2	not assigned.unknown	MDP0000570024	 highly similar to ( 681) AT3G04350 | Symbols:  | unknown protein | chr3:1153972-1156469 REVERSEhighly similar to ( 648) loc_os03g04930 12003.m35011 protein expressed protein   no original description 	T
10.7	cell wall.modification	MDP0000743239	 moderately similar to ( 450) AT2G28950 | Symbols: ATEXPA6, ATEXP6, ATHEXP ALPHA 1.8 | ATEXPA6 (ARABIDOPSIS THALIANA EXPANSIN A6) | chr2:12431840-12433482 REVERSEmoderately similar to ( 440) EXPA7_ORYSA Expansin-A7 precursor (OsEXPA7) (Alpha-expansin-7) (OsEXP7) (OsaEXPa1.26) - Oryza sativa (Rice)moderately similar to ( 440) loc_os03g60720 12003.m10961 protein alpha-expansin 6 precursor, putative, expressed   no original description 	T
30.2.11	signalling.receptor kinases.leucine rich repeat XI	MDP0000216262	 highly similar to ( 543) AT5G25930 | Symbols:  | leucine-rich repeat family protein / protein kinase family protein | chr5:9050880-9053978 FORWARDmoderately similar to ( 218) RPK1_IPONI Receptor-like protein kinase precursor (EC 2.7.11.1) - Ipomoea nil (Japanese morning glory) (Pharbitis nil)moderately similar to ( 481) loc_os05g44770 12005.m08602 protein receptor-like protein kinase 5 precursor, putative, expressed   no original description 	T
26.10	misc.cytochrome P450	MDP0000303875	 highly similar to ( 655) AT4G36380 | Symbols: ROT3 | ROT3 (ROTUNDIFOLIA 3); oxidoreductase, acting on paired donors, with incorporation or reduction of molecular oxygen, NADH or NADPH as one donor, and incorporation of one atom of oxygen / oxygen binding / steroid hydroxylase | chr4:17187973-17192202 REVERSEmoderately similar to ( 468) C90D2_ORYSA Cytochrome P450 90D2 (EC 1.14.-.-) (C6-oxidase) - Oryza sativa (Rice)moderately similar to ( 468) loc_os01g10040 12001.m42908 protein cytochrome P450 90D2, putative, expressed  no original description 	T
1.3.4	PS.calvin cycle.GAP	MDP0000362929	 very weakly similar to (75.5) AT1G42970 | Symbols: GAPB | GAPB (GLYCERALDEHYDE-3-PHOSPHATE DEHYDROGENASE B SUBUNIT); glyceraldehyde-3-phosphate dehydrogenase (NADP+)/ glyceraldehyde-3-phosphate dehydrogenase | chr1:16127552-16129584 FORWARDweakly similar to ( 104) G3PB_PEA Glyceraldehyde-3-phosphate dehydrogenase B, chloroplast precursor (EC 1.2.1.13) (NADP-dependent glyceraldehydephosphate dehydrogenase subunit B) - Pisum sativum (Garden pea)  no original description 	T
35.2	not assigned.unknown	MDP0000348818	 weakly similar to ( 194) AT5G42765 | Symbols:  | INVOLVED IN: biological_process unknown; LOCATED IN: thylakoid, chloroplast thylakoid membrane, chloroplast; EXPRESSED IN: 22 plant structures; EXPRESSED DURING: 13 growth stages; CONTAINS InterPro DOMAIN/s: Twin-arginine translocation pathway signal (InterPro:IPR006311); Has 13 Blast hits to 13 proteins in 4 species: Archae - 0; Bacteria - 0; Metazoa - 0; Fungi - 0; Plants - 13; Viruses - 0; Other Eukaryotes - 0 (source: NCBI BLink). | chr5:17150505-17151641 REVERSEweakly similar to ( 161) loc_os03g56320 12003.m35433 protein expressed protein  no original description 	T
29.5.11.4.2	protein.degradation.ubiquitin.E3.RING	MDP0000508676	 moderately similar to ( 316) AT1G69330 | Symbols:  | zinc finger (C3HC4-type RING finger) family protein | chr1:26064138-26064950 REVERSEmoderately similar to ( 290) loc_os03g31320 12003.m35286 protein RING zinc finger protein-like, putative, expressed no original description 	T
35.2	not assigned.unknown	MDP0000896898	 weakly similar to ( 112) AT5G11630 | Symbols:  | unknown protein | chr5:3740545-3741529 FORWARDvery weakly similar to (57.8) loc_os02g13630 12002.m100151 protein expressed protein  no original description 	T
29.2.1.2.1.7	protein.synthesis.ribosomal protein.eukaryotic.40S subunit.S7	MDP0000809965	 moderately similar to ( 306) AT1G48830 | Symbols:  | 40S ribosomal protein S7 (RPS7A) | chr1:18059854-18060935 REVERSEmoderately similar to ( 303) RS7_ORYSA 40S ribosomal protein S7 - Oryza sativa (Rice)moderately similar to ( 310) loc_os03g18580 12003.m07280 protein 40S ribosomal protein S7, putative, expressed  no original description 	T
34.21	transport.calcium	MDP0000652388	 moderately similar to ( 478) AT5G17860 | Symbols: CAX7 | CAX7 (calcium exchanger 7); calcium:sodium antiporter/ cation:cation antiporter | chr5:5902638-5904350 REVERSEmoderately similar to ( 407) loc_os03g08230 12003.m06332 protein cation cation antiporter, putative, expressed   no original description 	T
30.11.1	signalling.light.COP9 signalosome	MDP0000292286	 highly similar to ( 570) AT5G42970 | Symbols: COP8, FUS4, EMB134, COP14, CSN4, FUS8, ATS4 | COP8 (CONSTITUTIVE PHOTOMORPHOGENIC 8); protein binding | chr5:17237470-17240649 REVERSEhighly similar to ( 593) loc_os03g10120 12003.m06466 protein COP9 signalosome complex subunit 4, putative, expressed no original description 	T
34.99	transport.misc	MDP0000163588	 highly similar to ( 714) AT3G59030 | Symbols: TT12, ATTT12 | TT12 (TRANSPARENT TESTA 12); antiporter/ solute:hydrogen antiporter/ transmembrane transporter/ transporter | chr3:21819124-21821288 FORWARDhighly similar to ( 562) loc_os12g42130 12012.m07992 protein transparent testa 12 protein, putative, expressed  no original description 	T
29.5.5	protein.degradation.serine protease	MDP0000234291	 highly similar to ( 560) AT5G08260 | Symbols: scpl35 | scpl35 (serine carboxypeptidase-like 35); serine-type carboxypeptidase | chr5:2657236-2661272 FORWARDmoderately similar to ( 364) CBP23_HORVU Serine carboxypeptidase II-3 precursor (EC 3.4.16.6) (CP-MII.3) [Contains: Serine carboxypeptidase II-3 chain A; Serine carboxypeptidase II-3 chain B] - Hordeum vulgare (Barley)moderately similar to ( 458) loc_os02g42310 12002.m100291 protein lysosomal protective protein precursor, putative, expressed   no original description 	T
35.2	not assigned.unknown	MDP0000920394	 very weakly similar to (65.9) AT1G32928 | Symbols:  | unknown protein | chr1:11931332-11931586 FORWARD no original description 	T
35.2	not assigned.unknown	MDP0000310866	  no original description 	T
1.1.1.1	PS.lightreaction.photosystem II.LHC-II	MDP0000291218	 moderately similar to ( 423) AT3G47470 | Symbols: LHCA4, CAB4 | LHCA4 (LIGHT-HARVESTING CHLOROPHYLL-PROTEIN COMPLEX I SUBUNIT A4); chlorophyll binding | chr3:17493622-17494773 REVERSEmoderately similar to ( 226) CB12_PETHY Chlorophyll a-b binding protein, chloroplast precursor (LHCI type II CAB) - Petunia hybrida (Petunia)moderately similar to ( 370) loc_os08g33820 12008.m080044 protein chlorophyll a-b binding protein 4, chloroplast precursor, putative, expressed  no original description 	T
34.9	transport.metabolite transporters at the mitochondrial membrane	MDP0000943164	 highly similar to ( 512) AT5G14040 | Symbols:  | mitochondrial phosphate transporter | chr5:4531059-4532965 REVERSEhighly similar to ( 514) loc_os02g52860 12002.m10316 protein phosphate carrier protein, mitochondrial precursor, putative, expressed  no original description 	T
29.5.11.3	protein.degradation.ubiquitin.E2	MDP0000306090	 moderately similar to ( 258) AT1G64230 | Symbols: UBC28 | ubiquitin-conjugating enzyme, putative | chr1:23833792-23835220 FORWARDweakly similar to ( 122) UBC2_MEDSA Ubiquitin-conjugating enzyme E2-17 kDa (EC 6.3.2.19) (Ubiquitin-protein ligase) (Ubiquitin carrier protein) - Medicago sativa (Alfalfa)moderately similar to ( 252) loc_os04g57220 12004.m101686 protein ubiquitin-conjugating enzyme E2-17 kDa, putative, expressed no original description 	T
29.5.5	protein.degradation.serine protease	MDP0000264634	 highly similar to ( 709) AT3G07990 | Symbols: SCPL27 | SCPL27 (serine carboxypeptidase-like 27); serine-type carboxypeptidase | chr3:2552544-2554644 FORWARDhighly similar to ( 564) CBP2_HORVU Serine carboxypeptidase 2 precursor (EC 3.4.16.6) (Serine carboxypeptidase II) (Carboxypeptidase D) (CP-MII) [Contains: Serine carboxypeptidase 2 chain A (Serine carboxypeptidase II chain A); Serine carboxypeptidase 2 chain B (Serine cahighly similar to ( 660) loc_os01g61690 12001.m12294 protein virulence-related protein Nf314, putative, expressed   no original description 	T
29.2.1.2.1.19	protein.synthesis.ribosomal protein.eukaryotic.40S subunit.S19	MDP0000369361	 moderately similar to ( 262) AT5G61170 | Symbols:  | 40S ribosomal protein S19 (RPS19C) | chr5:24611158-24612202 FORWARDmoderately similar to ( 239) RS19_ORYSA 40S ribosomal protein S19 - Oryza sativa (Rice)moderately similar to ( 239) loc_os03g31090 12003.m08361 protein 40S ribosomal protein S19, putative, expressed   no original description 	T
35.2	not assigned.unknown	MDP0000283317	 moderately similar to ( 327) AT2G46230 | Symbols:  | FUNCTIONS IN: molecular_function unknown; INVOLVED IN: biological_process unknown; LOCATED IN: cellular_component unknown; EXPRESSED IN: 22 plant structures; EXPRESSED DURING: 13 growth stages; CONTAINS InterPro DOMAIN/s: Protein of unknown function DUF652 (InterPro:IPR006984), Nucleotide binding protein, PINc (InterPro:IPR006596); BEST Arabidopsis thaliana protein match is: unknown protein (TAIR:AT1G26530.1); Has 441 Blast hits to 441 proteins in 161 species: Archae - 15; Bacteria - 0; Metazoa - 185; Fungi - 119; Plants - 49; Viruses - 0; Other Eukaryotes - 73 (source: NCBI BLink). | chr2:18984694-18985850 REVERSEmoderately similar to ( 355) loc_os06g06410 12006.m05367 protein nucleolar protein involved in pre-mRNA processing, putative, expressed no original description 	T
26.8	misc.nitrilases, *nitrile lyases, berberine bridge enzymes, reticuline oxidases, troponine reductases	MDP0000178334	 highly similar to ( 699) AT1G12990 | Symbols:  | glycosyl transferase family 17 protein | chr1:4433973-4435552 FORWARDhighly similar to ( 605) loc_os02g38160 12002.m08904 protein N-acetylglucosaminyltransferase III, putative, expressed  no original description 	T
1.1.5.3	PS.lightreaction.other electron carrier (ox/red).ferredoxin reductase	MDP0000198078	 highly similar to ( 610) AT5G66190 | Symbols: ATLFNR1 | FNR1 (FERREDOXIN-NADP(+)-OXIDOREDUCTASE 1); NADPH dehydrogenase/ electron transporter, transferring electrons within the cyclic electron transport pathway of photosynthesis/ electron transporter, transferring electrons within the noncyclic electron transp | chr5:26451203-26452616 REVERSEhighly similar to ( 615) FENR_VICFA Ferredoxin--NADP reductase, chloroplast precursor (EC 1.18.1.2) (FNR) - Vicia faba (Broad bean)highly similar to ( 566) loc_os06g01850 12006.m04921 protein ferredoxin--NADP reductase, leaf isozyme, chloroplast precursor, putative, expressed  no original description 	T
29.7	protein.glycosylation	MDP0000825701	 moderately similar to ( 483) AT5G19980 | Symbols:  | integral membrane family protein | chr5:6749907-6750932 REVERSEhighly similar to ( 503) loc_os09g14520 12009.m04714 protein expressed protein  no original description 	T
34.2	transport.sugars	MDP0000688348	 highly similar to ( 694) AT3G18830 | Symbols: ATPLT5 | ATPLT5 (POLYOL TRANSPORTER 5); D-ribose transmembrane transporter/ D-xylose transmembrane transporter/ carbohydrate transmembrane transporter/ galactose transmembrane transporter/ glucose transmembrane transporter/ glycerol transmembrane transporter/ mann | chr3:6489000-6491209 REVERSEmoderately similar to ( 209) HEX6_RICCO Hexose carrier protein HEX6 - Ricinus communis (Castor bean)highly similar to ( 668) loc_os07g39350 12007.m08177 protein proton myo-inositol cotransporter, putative, expressed   no original description 	T
35.1	not assigned.no ontology	MDP0000748916	 moderately similar to ( 206) AT2G43120 | Symbols:  | pirin, putative | chr2:17927339-17928871 FORWARDmoderately similar to ( 232) loc_os03g62790 12003.m11157 protein pirin-like protein, putative, expressed no original description 	T
35.2	not assigned.unknown	MDP0000668140	 moderately similar to ( 378) AT4G25030 | Symbols:  | unknown protein | chr4:12865336-12866638 FORWARDmoderately similar to ( 340) loc_os10g43060 12010.m22001 protein expressed protein  no original description 	T
27.3.99	RNA.regulation of transcription.unclassified	MDP0000235992	 moderately similar to ( 396) AT4G24630 | Symbols:  | receptor/ zinc ion binding | chr4:12714919-12717111 FORWARDmoderately similar to ( 384) loc_os05g36090 12005.m07839 protein palmitoyltransferase ZDHHC9, putative, expressed  no original description 	T
13.2.3.1.1	amino acid metabolism.degradation.aspartate family.asparagine.L-asparaginase	MDP0000169087	 moderately similar to ( 497) AT3G16150 | Symbols:  | L-asparaginase, putative / L-asparagine amidohydrolase, putative | chr3:5471794-5473033 FORWARDmoderately similar to ( 318) ASPG_LUPLU L-asparaginase precursor (EC 3.5.1.1) (L-asparagine amidohydrolase) [Contains: L-asparaginase subunit alpha; L-asparaginase subunit beta] - Lupinus luteus (European yellow lupin)moderately similar to ( 474) loc_os04g55710 12004.m10462 protein transposon protein, putative, unclassified, expressed  no original description 	T
35.2	not assigned.unknown	MDP0000325652	  no original description 	T
35.2	not assigned.unknown	MDP0000543232	  no original description 	T
31.1	cell.organisation	MDP0000898327	 highly similar to ( 530) AT5G65860 | Symbols:  | ankyrin repeat family protein | chr5:26347706-26348686 REVERSEmoderately similar to ( 474) loc_os03g55330 12003.m10448 protein arginine N-methyltransferase 2, putative, expressed   no original description 	T
31.1	cell.organisation	MDP0000168646	 highly similar to ( 741) AT3G12110 | Symbols: ACT11 | ACT11 (actin-11); structural constituent of cytoskeleton | chr3:3858116-3859609 FORWARDhighly similar to ( 749) ACT1_TOBAC Actin - Nicotiana tabacum (Common tobacco)highly similar to ( 748) loc_os03g50890 12003.m10074 protein actin-1, putative, expressed   no original description 	T
35.1	not assigned.no ontology	MDP0000285682	 very weakly similar to (87.4) AT1G01490 | Symbols:  | heavy-metal-associated domain-containing protein | chr1:180401-182066 REVERSEweakly similar to ( 103) loc_os04g57200 12004.m10605 protein metal ion binding protein, putative, expressed no original description 	T
35.2	not assigned.unknown	MDP0000319122	    no original description 	T
29.2.3	protein.synthesis.initiation	MDP0000571038	 moderately similar to ( 340) AT4G33250 | Symbols: EIF3K, TIF3K1, ATTIF3K1 | EIF3K (EUKARYOTIC TRANSLATION INITIATION FACTOR 3K); translation initiation factor | chr4:16039066-16040617 REVERSEmoderately similar to ( 353) IF3C_ORYSA Eukaryotic translation initiation factor 3 subunit 12 (eIF-3 p25) (eIF3k) - Oryza sativa (Rice)moderately similar to ( 353) loc_os03g08450 12003.m06353 protein eukaryotic translation initiation factor 3 subunit 12, putative, expressed  no original description 	T
29.2.1.2.2.8	protein.synthesis.ribosomal protein.eukaryotic.60S subunit.L8	MDP0000221376	 moderately similar to ( 489) AT4G36130 | Symbols:  | 60S ribosomal protein L8 (RPL8C) | chr4:17097613-17098656 FORWARDmoderately similar to ( 470) RL8_TOBAC 60S ribosomal protein L8 (60S ribosomal protein L2) - Nicotiana tabacum (Common tobacco)moderately similar to ( 482) loc_os12g38000 12012.m07586 protein 60S ribosomal protein L2, putative, expressed   no original description 	T
35.2	not assigned.unknown	MDP0000234123	 moderately similar to ( 442) AT1G14590 | Symbols:  | FUNCTIONS IN: molecular_function unknown; INVOLVED IN: biological_process unknown; LOCATED IN: chloroplast thylakoid lumen; CONTAINS InterPro DOMAIN/s: Nucleotide-diphospho-sugar transferase, predicted (InterPro:IPR005069); BEST Arabidopsis thaliana protein match is: unknown protein (TAIR:AT2G02061.1); Has 179 Blast hits to 175 proteins in 14 species: Archae - 0; Bacteria - 0; Metazoa - 0; Fungi - 0; Plants - 172; Viruses - 0; Other Eukaryotes - 7 (source: NCBI BLink). | chr1:4998957-5000617 REVERSEmoderately similar to ( 403) loc_os04g49590 12004.m09892 protein regulatory protein, putative, expressed  no original description 	T
35.1	not assigned.no ontology	MDP0000166259	 moderately similar to ( 229) AT2G38740 | Symbols:  | haloacid dehalogenase-like hydrolase family protein | chr2:16194639-16195995 REVERSEmoderately similar to ( 227) loc_os10g41930 12010.m065348 protein catalytic/ hydrolase, putative, expressed no original description 	T
35.2	not assigned.unknown	MDP0000338330	 weakly similar to ( 115) loc_os02g39580 12002.m09046 protein expressed protein no original description 	T
30.4	signalling.phosphinositides	MDP0000148571	 nearly identical (2923) AT1G49340 | Symbols: ATPI4K ALPHA | ATPI4K ALPHA; 1-phosphatidylinositol 4-kinase/ inositol or phosphatidylinositol kinase | chr1:18252355-18263967 FORWARDweakly similar to ( 146) PI3K1_SOYBN Phosphatidylinositol 3-kinase, root isoform (EC 2.7.1.137) (PI3-kinase) (PtdIns-3-kinase) (PI3K) (SPI3K-5) - Glycine max (Soybean)nearly identical (1244) loc_os03g50320 12003.m10025 protein phosphatidylinositol 4-kinase PI4K, putative, expressed  no original description 	T
29.5.11.3	protein.degradation.ubiquitin.E2	MDP0000690401	 moderately similar to ( 266) AT1G17100 | Symbols:  | SOUL heme-binding family protein | chr1:5844766-5845539 FORWARDvery weakly similar to (55.5) UBC2_MEDSA Ubiquitin-conjugating enzyme E2-17 kDa (EC 6.3.2.19) (Ubiquitin-protein ligase) (Ubiquitin carrier protein) - Medicago sativa (Alfalfa)moderately similar to ( 257) loc_os01g11230 12001.m150508 protein heme-binding protein 2, putative, expressed no original description 	T
35.2	not assigned.unknown	MDP0000134680	 moderately similar to ( 441) AT5G12470 | Symbols:  | unknown protein | chr5:4044950-4047290 REVERSEmoderately similar to ( 427) loc_os05g32230 12005.m07456 protein expressed protein   no original description 	T
35.2	not assigned.unknown	MDP0000542322	 moderately similar to ( 300) AT2G34670 | Symbols:  | proline-rich family protein | chr2:14613239-14615231 REVERSEmoderately similar to ( 306) loc_os01g40330 12001.m42670 protein bZIP-like protein, putative, expressed   no original description 	T
33.99	development.unspecified	MDP0000282567	 moderately similar to ( 238) AT1G21140 | Symbols:  | nodulin, putative | chr1:7404464-7405066 FORWARDweakly similar to ( 176) NO21_SOYBN Nodulin 21 (N-21) - Glycine max (Soybean)weakly similar to ( 169) loc_os04g59020 12004.m10780 protein integral membrane protein, expressed  no original description 	T
29.2.1.2.1.11	protein.synthesis.ribosomal protein.eukaryotic.40S subunit.S11	MDP0000295156	 moderately similar to ( 240) AT5G23740 | Symbols: RPS11-BETA | RPS11-BETA (RIBOSOMAL PROTEIN S11-BETA); structural constituent of ribosome | chr5:8008251-8009330 REVERSEmoderately similar to ( 244) RS11_SOYBN 40S ribosomal protein S11 - Glycine max (Soybean)moderately similar to ( 246) loc_os07g37780 12007.m08027 protein 40S ribosomal protein S11, putative, expressed  no original description 	T
4.1.8	glycolysis.cytosolic branch.glyceraldehyde 3-phosphate dehydrogenase (GAP-DH)	MDP0000645828	 highly similar to ( 576) AT1G13440 | Symbols: GAPC-2, GAPC2 | GAPC2 (GLYCERALDEHYDE-3-PHOSPHATE DEHYDROGENASE C2); NAD or NADH binding / binding / catalytic/ glyceraldehyde-3-phosphate dehydrogenase (phosphorylating)/ glyceraldehyde-3-phosphate dehydrogenase | chr1:4608465-4610494 REVERSEhighly similar to ( 575) G3PC_ANTMA Glyceraldehyde-3-phosphate dehydrogenase, cytosolic (EC 1.2.1.12) - Antirrhinum majus (Garden snapdragon)highly similar to ( 566) loc_os04g40950 12004.m35161 protein glyceraldehyde-3-phosphate dehydrogenase, cytosolic 3, putative, expressed  no original description 	T
28.1	DNA.synthesis/chromatin structure	MDP0000186402	 highly similar to ( 657) AT5G11200 | Symbols:  | DEAD/DEAH box helicase, putative | chr5:3567389-3570686 FORWARDweakly similar to ( 180) IF4A_ORYSA Eukaryotic initiation factor 4A (EC 3.6.1.-) (ATP-dependent RNA helicase eIF4A) (eIF-4A) - Oryza sativa (Rice)highly similar to ( 553) loc_os01g36920 12001.m150622 protein spliceosome RNA helicase BAT1, putative, expressed  no original description 	T
27.1.2	RNA.processing.RNA helicase	MDP0000186402	 highly similar to ( 657) AT5G11200 | Symbols:  | DEAD/DEAH box helicase, putative | chr5:3567389-3570686 FORWARDweakly similar to ( 180) IF4A_ORYSA Eukaryotic initiation factor 4A (EC 3.6.1.-) (ATP-dependent RNA helicase eIF4A) (eIF-4A) - Oryza sativa (Rice)highly similar to ( 553) loc_os01g36920 12001.m150622 protein spliceosome RNA helicase BAT1, putative, expressed  no original description 	T
35.1	not assigned.no ontology	MDP0000188868	 moderately similar to ( 498) AT1G06890 | Symbols:  | transporter-related | chr1:2111728-2114038 REVERSEmoderately similar to ( 476) loc_os05g07670 12005.m05285 protein integral membrane protein like, putative, expressed no original description 	T
20.2.1	stress.abiotic.heat	MDP0000208958	 weakly similar to ( 182) AT5G37670 | Symbols:  | 15.7 kDa class I-related small heat shock protein-like (HSP15.7-CI) | chr5:14969035-14969448 FORWARDweakly similar to ( 121) HSP24_ORYSA 17.4 kDa class I heat shock protein 4 - Oryza sativa (Rice)weakly similar to ( 149) loc_os06g14240 12006.m06139 protein 17.4 kDa class I heat shock protein 3, putative, expressed  no original description 	T
27.3.29	RNA.regulation of transcription.TCP transcription factor family	MDP0000287069	 weakly similar to ( 144) AT4G18390 | Symbols:  | TCP family transcription factor, putative | chr4:10163212-10164309 REVERSEweakly similar to ( 138) loc_os07g05720 12007.m05036 protein TCP family transcription factor containing protein, expressed  no original description 	T
35.2	not assigned.unknown	MDP0000697264	 very weakly similar to (96.3) AT2G28410 | Symbols:  | unknown protein | chr2:12156202-12156549 FORWARD  no original description 	T
35.1	not assigned.no ontology	MDP0000908041	 nearly identical (2216) AT2G34680 | Symbols: AIR9 | AIR9; protein binding | chr2:14616947-14628300 REVERSEnearly identical (1028) loc_os07g05470 12007.m66708 protein AIR9, putative, expressed   no original description 	T
1.1.5.2	PS.lightreaction.other electron carrier (ox/red).ferredoxin	MDP0000771887	 weakly similar to ( 116) AT1G60950 | Symbols: FED A, ATFD2 | FED A; 2 iron, 2 sulfur cluster binding / electron carrier/ iron-sulfur cluster binding | chr1:22444565-22445011 FORWARDweakly similar to ( 124) FER1_MESCR Ferredoxin-1, chloroplast precursor (Ferredoxin I) - Mesembryanthemum crystallinum (Common ice plant)very weakly similar to (86.7) loc_os08g01380 12008.m080056 protein ferredoxin-1, chloroplast precursor, putative, expressed  no original description 	T
29.2.1.2.2.12	protein.synthesis.ribosomal protein.eukaryotic.60S subunit.L12	MDP0000311698	 moderately similar to ( 296) AT5G60670 | Symbols:  | 60S ribosomal protein L12 (RPL12C) | chr5:24381066-24381566 REVERSEmoderately similar to ( 242) RL12_CHLRE 60S ribosomal protein L12 (Fragment) - Chlamydomonas reinhardtiimoderately similar to ( 309) loc_os04g50990 12004.m09996 protein 60S ribosomal protein L12, putative, expressed  no original description 	T
17.2.2	hormone metabolism.auxin.signal transduction	MDP0000322329	 moderately similar to ( 416) AT5G16530 | Symbols: PIN5 | PIN5 (PIN-FORMED 5); auxin:hydrogen symporter/ transporter | chr5:5400735-5402626 FORWARDmoderately similar to ( 355) PIN6_ORYSA Probable auxin efflux carrier component 6 (OsPIN6) - Oryza sativa (Rice)moderately similar to ( 355) loc_os01g69070 12001.m13008 protein auxin efflux carrier component 6, putative, expressed no original description 	T
29.2.1.2.1.10	protein.synthesis.ribosomal protein.eukaryotic.40S subunit.S10	MDP0000178506	 moderately similar to ( 211) AT5G52650 | Symbols:  | 40S ribosomal protein S10 (RPS10C) | chr5:21355781-21357003 REVERSEmoderately similar to ( 207) RS10_ORYSA 40S ribosomal protein S10 - Oryza sativa (Rice)moderately similar to ( 207) loc_os04g35090 12004.m08600 protein 40S ribosomal protein S10, putative, expressed  no original description 	T
26.10	misc.cytochrome P450	MDP0000160416	 moderately similar to ( 470) AT3G48280 | Symbols: CYP71A25 | CYP71A25; electron carrier/ heme binding / iron ion binding / monooxygenase/ oxygen binding | chr3:17879594-17881164 FORWARDmoderately similar to ( 449) C71A4_SOLME Cytochrome P450 71A4 (EC 1.14.-.-) (CYPLXXIA4) (P-450EG2) - Solanum melongena (Eggplant) (Aubergine)moderately similar to ( 365) loc_os01g12760 12001.m07889 protein cytochrome P450 71A6, putative, expressed  no original description 	T
27.3.37	RNA.regulation of transcription.AS2,Lateral Organ Boundaries Gene Family	MDP0000881409	 weakly similar to ( 162) AT3G02550 | Symbols: LBD41 | LBD41 (LOB DOMAIN-CONTAINING PROTEIN 41) | chr3:536747-537650 REVERSEweakly similar to ( 125) loc_os01g32770 12001.m09596 protein seed specific protein Bn15D17A, putative, expressed  no original description 	T
16.2	secondary metabolism.phenylpropanoids	MDP0000205491	 moderately similar to ( 325) AT2G22570 | Symbols: ATNIC1, NIC1 | NIC1 (NICOTINAMIDASE 1); catalytic/ nicotinamidase | chr2:9590021-9590846 REVERSEmoderately similar to ( 291) loc_os02g39400 12002.m09028 protein isochorismatase hydrolase, putative, expressed MSP1_C  no original description 	T
30.5	signalling.G-proteins	MDP0000739171	 nearly identical (1173) AT3G60860 | Symbols:  | guanine nucleotide exchange family protein | chr3:22484804-22491510 FORWARDnearly identical (1128) loc_os03g14260 12003.m101270 protein brefeldin A-inhibited guanine nucleotide-exchange protein 2, putative, expressed MSP1_C  no original description 	T
20.2.1	stress.abiotic.heat	MDP0000379307	 moderately similar to ( 357) AT1G79920 | Symbols:  | ATP binding | chr1:30058935-30062224 REVERSEvery weakly similar to (58.5) BIP8_TOBAC Luminal-binding protein 8 (BiP 8) (78 kDa glucose-regulated protein homolog 8) (GRP 78-8) (Fragment) - Nicotiana tabacum (Common tobacco)moderately similar to ( 338) loc_os01g08560 12001.m150497 protein heat shock 70 kDa protein 4, putative, expressed   no original description 	T
35.2	not assigned.unknown	MDP0000722559	 moderately similar to ( 249) AT4G35730 | Symbols:  | unknown protein | chr4:16931111-16933241 FORWARDmoderately similar to ( 231) loc_os09g37510 12009.m06698 protein expressed protein   no original description 	T
35.2	not assigned.unknown	MDP0000771587	 moderately similar to ( 238) AT4G21150 | Symbols: HAP6 | HAP6 (HAPLESS 6); dolichyl-diphosphooligosaccharide-protein glycotransferase | chr4:11278646-11283599 FORWARDmoderately similar to ( 203) loc_os01g68324 12001.m12935 protein dolichyl-diphosphooligosaccharide--protein glycosyltransferase 63 kDasubunit precursor, putative, expressed no original description 	T
30.5	signalling.G-proteins	MDP0000285100	 highly similar to ( 777) AT3G54190 | Symbols:  | FUNCTIONS IN: molecular_function unknown; INVOLVED IN: biological_process unknown; EXPRESSED IN: 22 plant structures; EXPRESSED DURING: 13 growth stages; CONTAINS InterPro DOMAIN/s: WD40 repeat-like (InterPro:IPR011046); BEST Arabidopsis thaliana protein match is: unknown protein (TAIR:AT2G38630.1); Has 77 Blast hits to 77 proteins in 20 species: Archae - 0; Bacteria - 0; Metazoa - 0; Fungi - 0; Plants - 51; Viruses - 0; Other Eukaryotes - 26 (source: NCBI BLink). | chr3:20061759-20063880 REVERSEhighly similar to ( 741) loc_os05g40410 12005.m08217 protein expressed protein no original description 	T
10.7	cell wall.modification	MDP0000681724	 moderately similar to ( 394) AT1G26770 | Symbols: ATEXPA10, AT-EXP10, ATEXP10, ATHEXP ALPHA 1.1, EXP10 | ATEXPA10 (ARABIDOPSIS THALIANA EXPANSIN A 10); structural constituent of cell wall | chr1:9259775-9260792 FORWARDmoderately similar to ( 342) EXPA4_ORYSA Expansin-A4 precursor (OsEXPA4) (Alpha-expansin-4) (OsEXP4) (OsaEXPa1.22) - Oryza sativa (Rice)moderately similar to ( 342) loc_os05g39990 12005.m08176 protein alpha-expansin 1 precursor, putative, expressed  no original description 	T
28.1	DNA.synthesis/chromatin structure	MDP0000211229	 moderately similar to ( 471) AT5G04130 | Symbols:  | DNA topoisomerase, ATP-hydrolyzing, putative / DNA topoisomerase II, putative / DNA gyrase, putative | chr5:1122084-1128031 REVERSEmoderately similar to ( 431) GYRB_ORYSA DNA gyrase subunit B, chloroplast/mitochondrial precursor (EC 5.99.1.3) - Oryza sativa (Rice)moderately similar to ( 431) loc_os01g16290 12001.m42965 protein DNA gyrase subunit B, putative, expressed   no original description 	T
26.10	misc.cytochrome P450	MDP0000326412	 highly similar to ( 647) AT2G29090 | Symbols: CYP707A2 | CYP707A2; (+)-abscisic acid 8'-hydroxylase/ oxygen binding | chr2:12495038-12499080 REVERSEmoderately similar to ( 244) C724B_ORYSA Cytochrome P450 724B1 (EC 1.14.-.-) (OsDWARF11) (Dwarf protein 11) - Oryza sativa (Rice)highly similar to ( 534) loc_os02g47470 12002.m100328 protein cytochrome P450 90D2, putative, expressed MSP1_C  no original description 	T
29.3.4.99	protein.targeting.secretory pathway.unspecified	MDP0000216604	 weakly similar to ( 101) AT5G50460 | Symbols:  | protein transport protein SEC61 gamma subunit, putative | chr5:20552168-20552509 REVERSEweakly similar to ( 102) SC61G_ORYSA Protein transport protein SEC61 subunit gamma - Oryza sativa (Rice)weakly similar to ( 105) loc_os06g44374 12006.m91828 protein protein transport protein SEC61 gamma subunit, putative, expressed no original description 	T
20.1.7	stress.biotic.PR-proteins	MDP0000865880	 moderately similar to ( 445) AT1G61100 | Symbols:  | disease resistance protein (TIR class), putative | chr1:22508831-22512122 REVERSEmoderately similar to ( 243) loc_os07g26440 12007.m079669 protein CIP7, putative, expressed  no original description 	T
31.1	cell.organisation	MDP0000865880	 moderately similar to ( 445) AT1G61100 | Symbols:  | disease resistance protein (TIR class), putative | chr1:22508831-22512122 REVERSEmoderately similar to ( 243) loc_os07g26440 12007.m079669 protein CIP7, putative, expressed  no original description 	T
29.2.1.2.2.536	protein.synthesis.ribosomal protein.eukaryotic.60S subunit.L36A	MDP0000525424	 weakly similar to ( 179) AT4G14320 | Symbols:  | 60S ribosomal protein L36a/L44 (RPL36aB) | chr4:8242684-8243805 REVERSEweakly similar to ( 187) RL44_GOSHI 60S ribosomal protein L44 - Gossypium hirsutum (Upland cotton)weakly similar to ( 180) loc_os07g33997 12007.m07658 protein 60S ribosomal protein L44, putative, expressed no original description 	T
35.2	not assigned.unknown	MDP0000270654	 moderately similar to ( 348) AT3G05675 | Symbols:  | protein binding | chr3:1658425-1659859 REVERSEmoderately similar to ( 436) loc_os06g03840 12006.m05115 protein expressed protein  no original description 	T
29.2.1.2.2.7	protein.synthesis.ribosomal protein.eukaryotic.60S subunit.L7	MDP0000913491	 weakly similar to ( 191) AT1G80750 | Symbols:  | 60S ribosomal protein L7 (RPL7A) | chr1:30349052-30350434 FORWARDweakly similar to ( 195) loc_os04g51630 12004.m35453 protein 60S ribosomal protein L7-2, putative, expressed no original description 	T
17.4.2	hormone metabolism.cytokinin.signal transduction	MDP0000181429	 highly similar to ( 503) AT1G27320 | Symbols: AHK3 | AHK3 (ARABIDOPSIS HISTIDINE KINASE 3); cytokinin receptor/ osmosensor/ protein histidine kinase | chr1:9487780-9492027 FORWARDvery weakly similar to (51.2) ETR1_BRAOL Ethylene receptor (EC 2.7.13.3) - Brassica oleracea (Wild cabbage)moderately similar to ( 447) loc_os01g69920 12001.m42798 protein histidine kinase 2, putative, expressed  no original description 	T
29.2.1.2.1.8	protein.synthesis.ribosomal protein.eukaryotic.40S subunit.S8	MDP0000168216	 moderately similar to ( 300) AT5G59240 | Symbols:  | 40S ribosomal protein S8 (RPS8B) | chr5:23902626-23903670 REVERSEmoderately similar to ( 318) RS8_ORYSA 40S ribosomal protein S8 - Oryza sativa (Rice)moderately similar to ( 321) loc_os04g28180 12004.m07935 protein 40S ribosomal protein S8, putative, expressed no original description 	T
27.1.19	RNA.processing.ribonucleases	MDP0000942516	 very weakly similar to (53.1) AT1G24020 | Symbols: MLP423 | MLP423 (MLP-LIKE PROTEIN 423) | chr1:8500653-8501458 REVERSEmoderately similar to ( 298) MAL12_MALDO Major allergen Mal d 1 (Mal d I) (AP15) - Malus domestica (Apple) (Malus sylvestris)very weakly similar to (84.7) loc_os03g18850 12003.m07305 protein pathogenesis-related protein 1, putative, expressed no original description 	T
20.2.99	stress.abiotic.unspecified	MDP0000942516	 very weakly similar to (53.1) AT1G24020 | Symbols: MLP423 | MLP423 (MLP-LIKE PROTEIN 423) | chr1:8500653-8501458 REVERSEmoderately similar to ( 298) MAL12_MALDO Major allergen Mal d 1 (Mal d I) (AP15) - Malus domestica (Apple) (Malus sylvestris)very weakly similar to (84.7) loc_os03g18850 12003.m07305 protein pathogenesis-related protein 1, putative, expressed no original description 	T
27.3.6	RNA.regulation of transcription.bHLH,Basic Helix-Loop-Helix family	MDP0000646823	 moderately similar to ( 352) AT3G24140 | Symbols: FMA | FMA (FAMA); DNA binding / transcription activator/ transcription factor | chr3:8715525-8717772 REVERSEmoderately similar to ( 262) loc_os05g50900 12005.m09157 protein helix-loop-helix DNA-binding domain containing protein   no original description 	T
11.1.7	lipid metabolism.FA synthesis and FA elongation.ACP thioesterase	MDP0000912251	 moderately similar to ( 480) AT3G25110 | Symbols: AtFaTA | AtFaTA (Arabidopsis FatA acyl-ACP thioesterase); acyl carrier/ acyl-[acyl-carrier-protein] hydrolase | chr3:9146589-9148273 REVERSEmoderately similar to ( 242) FATB_GOSHI Myristoyl-acyl carrier protein thioesterase, chloroplast precursor (EC 3.1.2.-) (16:0-acyl-carrier protein thioesterase) (16:0-ACP thioesterase) (Acyl-[acyl-carrier-protein] hydrolase) (PATE) - Gossypium hirsutum (Upland cotton)moderately similar to ( 459) loc_os09g32760 12009.m060080 protein oleoyl-acyl carrier protein thioesterase, chloroplast precursor, putative, expressed  no original description 	T
26.22	misc.short chain dehydrogenase/reductase (SDR)	MDP0000312014	 moderately similar to ( 248) AT2G47140 | Symbols:  | short-chain dehydrogenase/reductase (SDR) family protein | chr2:19350970-19352059 REVERSEweakly similar to ( 195) TS2_MAIZE Sex determination protein tasselseed-2 - Zea mays (Maize)moderately similar to ( 260) loc_os03g61740 12003.m11055 protein sex determination protein tasselseed-2, putative, expressed   no original description 	T
20.1.7	stress.biotic.PR-proteins	MDP0000164725	 moderately similar to ( 285) AT5G66900 | Symbols:  | disease resistance protein (CC-NBS-LRR class), putative | chr5:26714931-26717757 REVERSEweakly similar to ( 196) loc_os12g39620 12012.m073872 protein disease resistance protein, putative, expressed  no original description 	T
35.2	not assigned.unknown	MDP0000251022	 moderately similar to ( 399) AT1G16080 | Symbols:  | unknown protein | chr1:5514394-5515761 FORWARDmoderately similar to ( 377) loc_os08g16570 12008.m26636 protein expressed protein  no original description 	T
33.99	development.unspecified	MDP0000147596	 moderately similar to ( 381) AT1G12910 | Symbols: ATAN11, LWD1 | ATAN11 (ANTHOCYANIN11); nucleotide binding | chr1:4395114-4396154 REVERSEmoderately similar to ( 341) loc_os02g32430 12002.m08336 protein WD40 repeat protein, putative, expressed  no original description 	T
35.2	not assigned.unknown	MDP0000244379	 moderately similar to ( 458) AT1G36310 | Symbols:  | methyltransferase | chr1:13669644-13670934 FORWARDmoderately similar to ( 457) loc_os02g51490 12002.m33275 protein expressed protein no original description 	T
35.2	not assigned.unknown	MDP0000140282	 highly similar to ( 577) AT5G19540 | Symbols:  | unknown protein | chr5:6595748-6597724 FORWARDhighly similar to ( 509) loc_os08g29780 12008.m06980 protein expressed protein MSP1_C  no original description 	T
35.2	not assigned.unknown	MDP0000180378	  no original description 	T
35.2	not assigned.unknown	MDP0000530792	 very weakly similar to (53.9) AT2G33850 | Symbols:  | unknown protein | chr2:14321221-14322024 REVERSEvery weakly similar to ( 100) E6_GOSHI Protein E6 - Gossypium hirsutum (Upland cotton)   no original description 	T
29.4	protein.postranslational modification	MDP0000301010	 highly similar to ( 667) AT4G28540 | Symbols: CKL6, PAPK1 | CKL6 (CASEIN KINASE I-LIKE 6); kinase/ protein serine/threonine kinase | chr4:14107284-14110511 FORWARDvery weakly similar to (64.7) CRK_DAUCA CDPK-related protein kinase (EC 2.7.11.1) (PK421) - Daucus carota (Carrot)highly similar to ( 626) loc_os02g40860 12002.m09171 protein casein kinase I isoform delta-like, putative, expressed  no original description 	T
30.3	signalling.calcium	MDP0000321833	 highly similar to ( 593) AT1G08450 | Symbols: CRT3 | CRT3 (CALRETICULIN 3); calcium ion binding / unfolded protein binding | chr1:2668008-2671800 REVERSEhighly similar to ( 513) CALR_NICPL Calreticulin precursor - Nicotiana plumbaginifolia (Leadwort-leaved tobacco)highly similar to ( 611) loc_os01g67054 12001.m43324 protein calreticulin-3 precursor, putative, expressed   no original description 	T
26.23	misc.rhodanese	MDP0000207557	 moderately similar to ( 249) AT4G27700 | Symbols:  | rhodanese-like domain-containing protein | chr4:13826541-13827673 REVERSEmoderately similar to ( 256) loc_os09g36040 12009.m21934 protein rhodanese-like domain containing protein, expressed no original description 	T
35.2	not assigned.unknown	MDP0000242903	 weakly similar to ( 187) loc_os05g33290 12005.m07561 protein expressed protein  no original description 	T
29.2.1.2.1.6	protein.synthesis.ribosomal protein.eukaryotic.40S subunit.S6	MDP0000872301	 moderately similar to ( 383) AT4G31700 | Symbols: RPS6 | RPS6 (RIBOSOMAL PROTEIN S6); structural constituent of ribosome | chr4:15346306-15347075 REVERSEmoderately similar to ( 382) RS6_ASPOF 40S ribosomal protein S6 - Asparagus officinalis (Garden asparagus)moderately similar to ( 371) loc_os07g42950 12007.m08524 protein 40S ribosomal protein S6, putative, expressed   no original description 	T
29.2.2.3.99	protein.synthesis.ribosome biogenesis.Pre-rRNA processing and modifications.misc	MDP0000238943	 moderately similar to ( 322) AT3G22660 | Symbols:  | rRNA processing protein-related | chr3:8016237-8017118 REVERSEmoderately similar to ( 319) loc_os07g29320 12007.m07203 protein rRNA-processing protein EBP2, putative, expressed  no original description 	T
20.2.3	stress.abiotic.drought/salt	MDP0000849502	 highly similar to ( 773) AT1G69450 | Symbols:  | LOCATED IN: endomembrane system, membrane; EXPRESSED IN: 23 plant structures; EXPRESSED DURING: 13 growth stages; CONTAINS InterPro DOMAIN/s: Protein of unknown function DUF221 (InterPro:IPR003864); BEST Arabidopsis thaliana protein match is: HYP1 (HYPOTHETICAL PROTEIN 1) (TAIR:AT3G01100.1); Has 911 Blast hits to 825 proteins in 137 species: Archae - 0; Bacteria - 0; Metazoa - 152; Fungi - 442; Plants - 243; Viruses - 0; Other Eukaryotes - 74 (source: NCBI BLink). | chr1:26107120-26110006 REVERSEhighly similar to ( 680) loc_os12g39320 12012.m07717 protein HYP1, putative, expressed no original description 	T
27.3.6	RNA.regulation of transcription.bHLH,Basic Helix-Loop-Helix family	MDP0000174388	 very weakly similar to (97.1) AT1G26945 | Symbols: KDR | KDR (KIDARI); transcription regulator | chr1:9351571-9352474 FORWARDvery weakly similar to (87.8) loc_os10g26460 12010.m05586 protein DNA binding protein, putative no original description 	T
29.5.11.4.2	protein.degradation.ubiquitin.E3.RING	MDP0000256628	 nearly identical (1455) AT1G47550 | Symbols:  | unknown protein | chr1:17457171-17463896 FORWARDnearly identical (1274) loc_os03g42750 12003.m34897 protein roothairless 1, putative, expressed   no original description 	T
20.2.1	stress.abiotic.heat	MDP0000277859	 moderately similar to ( 203) AT1G65820 | Symbols:  | microsomal glutathione s-transferase, putative | chr1:24485213-24486682 FORWARDvery weakly similar to (85.5) HSP11_HELAN 17.6 kDa class I heat shock protein - Helianthus annuus (Common sunflower)weakly similar to ( 177) loc_os03g50130 12003.m35389 protein microsomal glutathione S-transferase 3, putative, expressed  TrbL  no original description 	T
2.2.1.3.1	major CHO metabolism.degradation.sucrose.invertases.neutral	MDP0000186866	 highly similar to ( 963) AT4G34860 | Symbols:  | beta-fructofuranosidase, putative / invertase, putative / saccharase, putative / beta-fructosidase, putative | chr4:16609955-16612019 REVERSEhighly similar to ( 891) loc_os02g34560 12002.m33716 protein neutral/alkaline invertase, putative, expressed no original description 	T
26.28	misc.GDSL-motif lipase	MDP0000217651	 weakly similar to ( 197) AT5G08460 | Symbols:  | GDSL-motif lipase/hydrolase family protein | chr5:2733220-2735351 FORWARDweakly similar to ( 137) APG_BRANA Anter-specific proline-rich protein APG (Protein CEX) (Fragment) - Brassica napus (Rape)moderately similar to ( 273) loc_os06g36520 12006.m08188 protein anther-specific proline-rich protein APG precursor, putative, expressed  no original description 	T
20.2.99	stress.abiotic.unspecified	MDP0000166730	 weakly similar to ( 184) AT2G47710 | Symbols:  | universal stress protein (USP) family protein | chr2:19555045-19555956 REVERSEweakly similar to ( 173) loc_os12g36640 12012.m07450 protein fiber protein Fb19, putative, expressed   no original description 	T
23.2	nucleotide metabolism.degradation	MDP0000763924	 highly similar to ( 823) AT4G19180 | Symbols:  | nucleoside phosphatase family protein / GDA1/CD39 family protein | chr4:10485518-10487868 REVERSEvery weakly similar to (91.3) NTPA_PEA Nucleoside-triphosphatase (EC 3.6.1.15) (Nucleoside triphosphate phosphohydrolase) (NTPase) (Apyrase) - Pisum sativum (Garden pea)highly similar to ( 700) loc_os10g21000 12010.m05120 protein hydrolase, putative, expressed   no original description 	T
30.2.11	signalling.receptor kinases.leucine rich repeat XI	MDP0000206592	 moderately similar to ( 394) AT1G45616 | Symbols: AtRLP6 | AtRLP6 (Receptor Like Protein 6); protein binding | chr1:17183550-17186534 REVERSEmoderately similar to ( 213) RPK1_IPONI Receptor-like protein kinase precursor (EC 2.7.11.1) - Ipomoea nil (Japanese morning glory) (Pharbitis nil)moderately similar to ( 444) loc_os04g40440 12004.m09026 protein expressed protein  no original description 	T
26.7	misc.oxidases - copper, flavone etc	MDP0000239885	 moderately similar to ( 484) AT4G13010 | Symbols:  | oxidoreductase, zinc-binding dehydrogenase family protein | chr4:7600682-7602567 FORWARDmoderately similar to ( 486) QORH_SPIOL Chloroplastic quinone-oxidoreductase homolog (EC 1.-.-.-) (ceQORH) - Spinacia oleracea (Spinach)moderately similar to ( 402) loc_os04g30420 12004.m08147 protein chloroplastic quinone-oxidoreductase, putative, expressed  no original description 	T
27.3.9	RNA.regulation of transcription.C2C2(Zn) GATA transcription factor family	MDP0000131803	 weakly similar to ( 111) AT4G26150 | Symbols: CGA1 | CGA1 (CYTOKININ-RESPONSIVE GATA FACTOR 1); transcription factor | chr4:13253210-13254659 FORWARDvery weakly similar to (86.3) loc_os02g12790 12002.m06526 protein GATA transcription factor 20, putative, expressed  no original description 	T
29.2.1.1.1.2.3	protein.synthesis.ribosomal protein.prokaryotic.chloroplast.50S subunit.L3	MDP0000287275	 moderately similar to ( 377) AT2G43030 | Symbols:  | ribosomal protein L3 family protein | chr2:17894898-17895713 FORWARDmoderately similar to ( 372) RK3_TOBAC 50S ribosomal protein L3, chloroplast precursor (Fragment) - Nicotiana tabacum (Common tobacco)moderately similar to ( 340) loc_os02g04460 12002.m05795 protein 50S ribosomal protein L3, chloroplast precursor, putative, expressed   no original description 	T
35.2	not assigned.unknown	MDP0000145600	 moderately similar to ( 261) AT5G47310 | Symbols:  | unknown protein | chr5:19201325-19202674 FORWARDmoderately similar to ( 248) loc_os03g01130 12003.m05664 protein EREBP-4 like protein, putative, expressed  no original description 	T
29.4	protein.postranslational modification	MDP0000220797	 moderately similar to ( 467) AT2G39840 | Symbols: TOPP4 | TOPP4; protein serine/threonine phosphatase | chr2:16627430-16628789 FORWARDmoderately similar to ( 472) PP11_ACECL Serine/threonine-protein phosphatase PP1 isozyme 1 (EC 3.1.3.16) - Acetabularia cliftonii (Green alga)moderately similar to ( 472) loc_os03g16110 12003.m34828 protein serine/threonine-protein phosphatase PP1, putative, expressed no original description 	T
29.4	protein.postranslational modification	MDP0000376563	 moderately similar to ( 418) AT3G15260 | Symbols:  | protein phosphatase 2C, putative / PP2C, putative | chr3:5138842-5140242 FORWARDmoderately similar to ( 363) loc_os07g32380 12007.m07503 protein protein phosphatase 2C isoform epsilon, putative, expressed no original description 	T
30.2.3	signalling.receptor kinases.leucine rich repeat III	MDP0000884053	 highly similar to ( 714) AT5G67200 | Symbols:  | leucine-rich repeat transmembrane protein kinase, putative | chr5:26813893-26816555 REVERSEweakly similar to ( 129) PSKR_DAUCA Phytosulfokine receptor precursor (EC 2.7.11.1) (Phytosulfokine LRR receptor kinase) - Daucus carota (Carrot)highly similar to ( 520) loc_os04g55620 12004.m10453 protein receptor kinase, putative, expressed TrbL  no original description 	T
20.1	stress.biotic	MDP0000329063	 moderately similar to ( 365) AT2G28790 | Symbols:  | osmotin-like protein, putative | chr2:12354664-12355413 REVERSEweakly similar to ( 182) TP1A_MALDO Thaumatin-like protein 1a precursor (Allergen Mal d 2) (Mdtl1) (Pathogenesis-related protein 5a) (PR-5a) - Malus domestica (Apple) (Malus sylvestris)moderately similar to ( 347) loc_os01g62260 12001.m12349 protein osmotin-like protein precursor, putative, expressed  no original description 	T
16.8.3.1	secondary metabolism.flavonoids.dihydroflavonols.dihydroflavonol 4-reductase	MDP0000681549	 moderately similar to ( 412) AT5G19440 | Symbols:  | cinnamyl-alcohol dehydrogenase, putative (CAD) | chr5:6556493-6558123 FORWARDmoderately similar to ( 229) DFRA_DIACA Dihydroflavonol-4-reductase (EC 1.1.1.219) (DFR) (Dihydrokaempferol 4-reductase) - Dianthus caryophyllus (Carnation) (Clove pink)moderately similar to ( 378) loc_os01g34480 12001.m150871 protein dihydroflavonol-4-reductase, putative, expressed  no original description 	T
29.4	protein.postranslational modification	MDP0000151925	 highly similar to ( 551) AT3G58500 | Symbols: PP2A-3, EP7 | PP2A-3 (PROTEIN PHOSPHATASE 2A-3); protein serine/threonine phosphatase | chr3:21635503-21638911 REVERSEhighly similar to ( 550) PP2A2_ORYSA Serine/threonine-protein phosphatase PP2A-2 catalytic subunit (EC 3.1.3.16) - Oryza sativa (Rice)highly similar to ( 550) loc_os03g59060 12003.m35470 protein OsPP2Ac-2 - Phosphatase 2A isoform 2 belonging to family 2, expressed  no original description 	T
9.9	mitochondrial electron transport / ATP synthesis.F1-ATPase 	MDP0000785964	 weakly similar to ( 189) AT5G47030 | Symbols:  | ATP synthase delta' chain, mitochondrial | chr5:19090384-19092034 FORWARDmoderately similar to ( 209) ATP4_IPOBA ATP synthase delta' chain, mitochondrial precursor (EC 3.6.3.14) - Ipomoea batatas (Sweet potato) (Batate)weakly similar to ( 195) loc_os07g31300 12007.m07398 protein ATP synthase delta chain, mitochondrial precursor, putative, expressed no original description 	T
10.6.2	cell wall.degradation.mannan-xylose-arabinose-fucose	MDP0000166406	 moderately similar to ( 468) AT2G28100 | Symbols: ATFUC1 | ATFUC1 (alpha-L-fucosidase 1); alpha-L-fucosidase | chr2:11974803-11976489 FORWARDmoderately similar to ( 445) FUCO1_ORYSA Putative alpha-L-fucosidase 1 precursor (EC 3.2.1.51) (Alpha-L-fucoside fucohydrolase) - Oryza sativa (Rice)highly similar to ( 564) loc_os09g34920 12009.m06492 protein alpha-L-fucosidase 1 precursor, putative, expressed   no original description 	T
30.3	signalling.calcium	MDP0000157748	 weakly similar to ( 150) AT3G50770 | Symbols:  | calmodulin-related protein, putative | chr3:18873987-18874604 FORWARDvery weakly similar to (70.9) CALM1_SOLTU Calmodulin-1 (CaM-1) - Solanum tuberosum (Potato)weakly similar to ( 108) loc_os01g56030 12001.m11759 protein calmodulin-like protein 41, putative, expressed  no original description 	T
35.2	not assigned.unknown	MDP0000172234	   no original description 	T
35.1.40	not assigned.no ontology.glycine rich proteins	MDP0000252034	 moderately similar to ( 426) AT3G09070 | Symbols:  | glycine-rich protein | chr3:2768880-2770937 REVERSEmoderately similar to ( 218) loc_os01g63310 12001.m12447 protein expressed protein    no original description 	T
35.2	not assigned.unknown	MDP0000280711	 weakly similar to ( 127) AT5G40980 | Symbols:  | unknown protein | chr5:16414877-16415086 REVERSEweakly similar to ( 113) loc_os01g69890 12001.m13038 protein expressed protein  no original description 	T
26.2	misc.UDP glucosyl and glucoronyl transferases	MDP0000290004	 highly similar to ( 826) AT3G02350 | Symbols: GAUT9 | GAUT9 (Galacturonosyltransferase 9); polygalacturonate 4-alpha-galacturonosyltransferase/ transferase, transferring glycosyl groups / transferase, transferring hexosyl groups | chr3:479248-481178 FORWARDhighly similar to ( 587) loc_os02g29530 12002.m08096 protein transferase, transferring glycosyl groups, putative, expressed  no original description 	T
35.2	not assigned.unknown	MDP0000217265	   no original description 	T
35.2	not assigned.unknown	MDP0000133195	 weakly similar to ( 129) AT5G64400 | Symbols:  | unknown protein | chr5:25748922-25749947 FORWARDweakly similar to ( 138) loc_os03g48080 12003.m09819 protein p8MTCP1, putative, expressed no original description 	T
10.7	cell wall.modification	MDP0000521662	 moderately similar to ( 398) AT2G40610 | Symbols: ATEXPA8, EXP8, ATEXP8, ATHEXP ALPHA 1.11 | ATEXPA8 (ARABIDOPSIS THALIANA EXPANSIN A8) | chr2:16949121-16950472 REVERSEmoderately similar to ( 370) EXPA2_ORYSA Expansin-A2 precursor (OsEXPA2) (Alpha-expansin-2) (OsEXP2) (OsaEXPa1.23) (RiExB) (RiExC) - Oryza sativa (Rice)moderately similar to ( 370) loc_os01g60770 12001.m12205 protein alpha-expansin 10 precursor, putative, expressed  no original description 	T
35.2	not assigned.unknown	MDP0000330537	 moderately similar to ( 201) AT4G19140 | Symbols:  | unknown protein | chr4:10469830-10471275 FORWARDweakly similar to ( 176) loc_os04g51166 12004.m35051 protein expressed protein   no original description 	T
31.1	cell.organisation	MDP0000812416	 highly similar to ( 780) AT5G19780 | Symbols: TUA5 | TUA5; structural constituent of cytoskeleton | chr5:6687212-6688926 FORWARDhighly similar to ( 782) TBA6_MAIZE Tubulin alpha-6 chain (Alpha-6 tubulin) - Zea mays (Maize)highly similar to ( 780) loc_os07g38730 12007.m08118 protein tubulin alpha-1 chain, putative, expressed  no original description 	T
31.1	cell.organisation	MDP0000173902	 nearly identical (1014) AT3G44730 | Symbols:  | kinesin motor protein-related | chr3:16285888-16290852 FORWARDweakly similar to ( 164) FLA10_CHLRE Kinesin-like protein FLA10 (Protein KHP1) - Chlamydomonas reinhardtiihighly similar to ( 535) loc_os12g36100 12012.m073854 protein kinesin-4, putative, expressed   no original description 	T
30.3	signalling.calcium	MDP0000639465	 moderately similar to ( 313) AT5G13460 | Symbols: IQD11 | IQD11 (IQ-domain 11); calmodulin binding | chr5:4316323-4318247 FORWARDweakly similar to ( 148) loc_os03g23960 12003.m07742 protein SF16-like protein, putative, expressed  no original description 	T
27.3.22	RNA.regulation of transcription.HB,Homeobox transcription factor family	MDP0000423445	 moderately similar to ( 254) AT4G37790 | Symbols: HAT22 | HAT22; transcription factor | chr4:17768241-17769272 FORWARDmoderately similar to ( 209) loc_os08g36220 12008.m07611 protein homeobox-leucine zipper protein HAT14, putative, expressed no original description 	T
30.7	signalling.14-3-3 proteins	MDP0000270640	 moderately similar to ( 376) AT1G34760 | Symbols: GRF11, GF14 OMICRON | GRF11 (GENERAL REGULATORY FACTOR 11); ATPase binding / amino acid binding / protein binding / protein phosphorylated amino acid binding | chr1:12743981-12745603 REVERSEmoderately similar to ( 374) 1433_TOBAC 14-3-3-like protein - Nicotiana tabacum (Common tobacco)moderately similar to ( 331) loc_os02g36974 12002.m100253 protein 14-3-3-like protein B, putative, expressed  no original description 	T
13.1.3.5.4	amino acid metabolism.synthesis.aspartate family.lysine.diaminopimelate epimerase	MDP0000182066	 moderately similar to ( 392) AT3G53580 | Symbols:  | diaminopimelate epimerase family protein | chr3:19864784-19866907 FORWARDmoderately similar to ( 360) loc_os12g37960 12012.m26936 protein diaminopimelate epimerase, putative, expressed   no original description 	T
35.1.1	not assigned.no ontology.ABC1 family protein	MDP0000296681	 highly similar to ( 734) AT2G40090 | Symbols: ATATH9 | ATATH9; transporter | chr2:16737685-16740303 REVERSEmoderately similar to ( 298) loc_os11g48070 12011.m08606 protein isoamyl acetate-hydrolyzing esterase, putative, expressed   no original description 	T
35.1	not assigned.no ontology	MDP0000299424	 very weakly similar to (96.7) AT4G16380 | Symbols:  | LOCATED IN: cellular_component unknown; BEST Arabidopsis thaliana protein match is: metal ion binding (TAIR:AT1G49420.1); Has 3600 Blast hits to 3158 proteins in 308 species: Archae - 4; Bacteria - 309; Metazoa - 1646; Fungi - 490; Plants - 698; Viruses - 60; Other Eukaryotes - 393 (source: NCBI BLink). | chr4:9255148-9255955 FORWARD  no original description 	T
21.2	redox.ascorbate and glutathione	MDP0000271593	 weakly similar to ( 144) AT1G60660 | Symbols: B5 #5, ATCB5LP, CB5LP | CB5LP (CYTOCHROME B5-LIKE PROTEIN); heme binding | chr1:22342589-22342954 REVERSEvery weakly similar to (80.1) CYB5_TOBAC Cytochrome b5 - Nicotiana tabacum (Common tobacco)weakly similar to ( 135) loc_os08g29669 12008.m80263 protein cytochrome b5, putative, expressed no original description 	T
34.22	transport.cyclic nucleotide or calcium regulated channels	MDP0000259830	 highly similar to ( 907) AT1G19780 | Symbols: ATCNGC8, CNGC8 | ATCNGC8 (CYCLIC NUCLEOTIDE GATED CHANNEL 8); calmodulin binding / cyclic nucleotide binding / ion channel | chr1:6833885-6836578 REVERSEhighly similar to ( 752) loc_os04g55080 12004.m10400 protein cyclic nucleotide-gated ion channel 9, putative, expressed  TrbL  no original description 	T
31.1	cell.organisation	MDP0000398981	 highly similar to ( 820) AT5G55230 | Symbols: ATMAP65-1, MAP65-1 | ATMAP65-1 (MICROTUBULE-ASSOCIATED PROTEINS 65-1); microtubule binding | chr5:22402716-22405182 FORWARDhighly similar to ( 720) loc_os06g20370 12006.m06694 protein microtubule-associated protein MAP65-1a, putative, expressed no original description 	T
31.1	cell.organisation	MDP0000222020	 highly similar to ( 842) AT5G23860 | Symbols: TUB8 | TUB8; structural constituent of cytoskeleton | chr5:8042962-8044528 FORWARDhighly similar to ( 835) TBB2_ORYSA Tubulin beta-2 chain (Beta-2 tubulin) - Oryza sativa (Rice)highly similar to ( 835) loc_os03g01530 12003.m05700 protein tubulin beta-2 chain, putative, expressed   no original description 	T
34.6	transport.sulphate	MDP0000190006	 highly similar to ( 900) AT1G23090 | Symbols: AST91, SULTR3;3 | AST91 (SULFATE TRANSPORTER 91); sulfate transmembrane transporter | chr1:8185238-8188954 REVERSEmoderately similar to ( 353) NO70_SOYBN Early nodulin 70 - Glycine max (Soybean)highly similar to ( 862) loc_os04g55800 12004.m10471 protein sulfate transporter 3.3, putative, expressed   no original description 	T
31.2	cell.division	MDP0000230636	 highly similar to ( 758) AT1G78770 | Symbols: APC6 | cell division cycle family protein | chr1:29617421-29621273 FORWARDvery weakly similar to (50.4) SPY_ORYSA Probable UDP-N-acetylglucosamine--peptide N-acetylglucosaminyltransferase SPINDLY (EC 2.4.1.-) - Oryza sativa (Rice)highly similar to ( 678) loc_os03g13370 12003.m06782 protein cell division cycle protein 16, putative, expressed  no original description 	T
26.2	misc.UDP glucosyl and glucoronyl transferases	MDP0000304625	 moderately similar to ( 430) AT5G59590 | Symbols: UGT76E2 | UGT76E2 (UDP-GLUCOSYL TRANSFERASE 76E2); UDP-glycosyltransferase/ quercetin 3-O-glucosyltransferase/ quercetin 7-O-glucosyltransferase | chr5:24009152-24010585 REVERSEweakly similar to ( 158) UFOG_SOLME Anthocyanidin 3-O-glucosyltransferase (EC 2.4.1.115) (Flavonol 3-O-glucosyltransferase) (UDP-glucose flavonoid 3-O-glucosyltransferase) - Solanum melongena (Eggplant) (Aubergine)moderately similar to ( 332) loc_os03g60960 12003.m10983 protein cytokinin-N-glucosyltransferase 1, putative, expressed TrbL  no original description 	T
35.2	not assigned.unknown	MDP0000276174	 very weakly similar to (64.3) AT5G11550 | Symbols:  | binding | chr5:3708203-3709147 REVERSE   COG4907  no original description 	T
29.5.3	protein.degradation.cysteine protease	MDP0000643283	 highly similar to ( 542) AT5G50260 | Symbols:  | cysteine proteinase, putative | chr5:20455605-20456862 FORWARDhighly similar to ( 564) CYSEP_RICCO Vignain precursor (EC 3.4.22.-) (Cysteine endopeptidase) - Ricinus communis (Castor bean)moderately similar to ( 446) loc_os08g44270 12008.m08400 protein vignain precursor, putative, expressed  no original description 	T
35.2	not assigned.unknown	MDP0000254883	 weakly similar to ( 128) AT5G02160 | Symbols:  | unknown protein | chr5:426392-427024 FORWARDweakly similar to ( 116) loc_os05g01950 12005.m04731 protein expressed protein no original description 	T
27.3.99	RNA.regulation of transcription.unclassified	MDP0000169057	 moderately similar to ( 270) AT4G17600 | Symbols: LIL3:1 | LIL3:1; transcription factor | chr4:9803759-9804714 FORWARDmoderately similar to ( 272) loc_os02g03330 12002.m05683 protein lil3 protein, putative, expressed  no original description 	T
27.3.23	RNA.regulation of transcription.HSF,Heat-shock transcription factor family	MDP0000169057	 moderately similar to ( 270) AT4G17600 | Symbols: LIL3:1 | LIL3:1; transcription factor | chr4:9803759-9804714 FORWARDmoderately similar to ( 272) loc_os02g03330 12002.m05683 protein lil3 protein, putative, expressed  no original description 	T
35.2	not assigned.unknown	MDP0000161987	 highly similar to ( 536) AT3G04460 | Symbols: PEX12, ATPEX12, APM4 | PEX12; protein binding / zinc ion binding | chr3:1186641-1189094 FORWARDmoderately similar to ( 497) loc_os10g32960 12010.m06132 protein peroxisome assembly protein 12, putative, expressed  no original description 	T
35.2	not assigned.unknown	MDP0000309080	 nearly identical (3005) AT2G48060 | Symbols:  | unknown protein | chr2:19651560-19662386 REVERSEnearly identical (1821) loc_os01g29230 12001.m09360 protein expressed protein  COG3889  no original description 	T
16.2.1.10	secondary metabolism.phenylpropanoids.lignin biosynthesis.CAD	MDP0000236430	 moderately similar to ( 407) AT4G39330 | Symbols: ATCAD9, CAD9 | CAD9 (CINNAMYL ALCOHOL DEHYDROGENASE 9); binding / catalytic/ oxidoreductase/ zinc ion binding | chr4:18291268-18292740 FORWARDmoderately similar to ( 417) MTDH_MEDSA Probable mannitol dehydrogenase (EC 1.1.1.255) (NAD-dependent mannitol dehydrogenase) - Medicago sativa (Alfalfa)moderately similar to ( 323) loc_os10g29470 12010.m05819 protein mannitol dehydrogenase, putative, expressed  no original description 	T
28.1	DNA.synthesis/chromatin structure	MDP0000169601	 nearly identical (1023) AT4G31210 | Symbols:  | DNA topoisomerase family protein | chr4:15165280-15172534 FORWARDhighly similar to ( 854) loc_os06g16980 12006.m32047 protein DNA topoisomerase I family protein, expressed   no original description 	T
20.1	stress.biotic	MDP0000931048	 moderately similar to ( 409) AT1G18250 | Symbols: ATLP-1 | ATLP-1 | chr1:6277024-6278005 REVERSEmoderately similar to ( 240) TLP1_PRUPE Thaumatin-like protein 1 precursor (PpAZ44) - Prunus persica (Peach)moderately similar to ( 338) loc_os06g47600 12006.m09286 protein thaumatin-like protein precursor, putative, expressed  no original description 	T
27.3.6	RNA.regulation of transcription.bHLH,Basic Helix-Loop-Helix family	MDP0000210979	 weakly similar to ( 117) AT1G26945 | Symbols: KDR | KDR (KIDARI); transcription regulator | chr1:9351571-9352474 FORWARDweakly similar to ( 101) loc_os10g26460 12010.m05586 protein DNA binding protein, putative no original description 	T
18.3.1	Co-factor and vitamine metabolism.riboflavin.GTP cyclohydrolase II	MDP0000131646	 highly similar to ( 739) AT5G64300 | Symbols: ATGCH, GCH, ATRIBA1, RFD1 | ATGCH; 3,4-dihydroxy-2-butanone-4-phosphate synthase/ GTP cyclohydrolase II | chr5:25718459-25720790 FORWARDhighly similar to ( 665) loc_os02g36340 12002.m33397 protein riboflavin biosynthesis protein ribAB, chloroplast precursor, putative, expressed MSP1_C  no original description 	T
30.5	signalling.G-proteins	MDP0000550069	 moderately similar to ( 328) AT1G75840 | Symbols: ARAC5, ATGP3, ROP4, ATROP4 | ARAC5 (RAC-LIKE GTP BINDING PROTEIN 5); GTP binding / GTPase | chr1:28475964-28477377 FORWARDmoderately similar to ( 325) RAC1_LOTJA Rac-like GTP-binding protein RAC1 - Lotus japonicusmoderately similar to ( 323) loc_os02g58730 12002.m33488 protein rac-like GTP-binding protein 5, putative, expressed no original description 	T
30.11	signalling.light	MDP0000218935	 weakly similar to ( 117) AT3G26740 | Symbols: CCL | CCL (CCR-LIKE) | chr3:9827868-9828461 FORWARDweakly similar to ( 107) LIRP1_ORYSA Light-regulated protein precursor - Oryza sativa (Rice)weakly similar to ( 107) loc_os01g01340 12001.m42821 protein light-induced protein 1-like, putative, expressed   no original description 	T
35.2	not assigned.unknown	MDP0000361521	  no original description 	T
17.2.3	hormone metabolism.auxin.induced-regulated-responsive-activated	MDP0000214081	 highly similar to ( 550) AT4G03400 | Symbols: DFL2, GH3-10 | DFL2 (DWARF IN LIGHT 2) | chr4:1497675-1499729 REVERSEmoderately similar to ( 348) GH35_ORYSA Probable indole-3-acetic acid-amido synthetase GH3.5 (EC 6.3.2.-) (Auxin-responsive GH3-like protein 5) (OsGH3-5) - Oryza sativa (Rice)moderately similar to ( 348) loc_os05g50890 12005.m27991 protein indole-3-acetic acid-amido synthetase GH3.5, putative, expressed  no original description 	T
27.3.4	RNA.regulation of transcription.ARF, Auxin Response Factor family	MDP0000232417	 nearly identical (1112) AT1G30330 | Symbols: ARF6 | ARF6 (AUXIN RESPONSE FACTOR 6); transcription factor | chr1:10686125-10690036 REVERSEvery weakly similar to (52.0) IAA9_ORYSA Auxin-responsive protein IAA9 (Indoleacetic acid-induced protein 9) - Oryza sativa (Rice)nearly identical (1033) loc_os06g46410 12006.m091759 protein auxin response factor 6, putative, expressed   no original description 	T
27.3.23	RNA.regulation of transcription.HSF,Heat-shock transcription factor family	MDP0000191541	 moderately similar to ( 276) AT1G67970 | Symbols: AT-HSFA8, HSFA8 | AT-HSFA8; DNA binding / transcription factor | chr1:25484844-25486313 REVERSEweakly similar to ( 199) loc_os03g63750 12003.m11243 protein heat shock factor protein HSF8, putative, expressed no original description 	T
11.1.10	lipid metabolism.FA synthesis and FA elongation.beta ketoacyl CoA synthase	MDP0000433567	 highly similar to ( 775) AT1G04220 | Symbols: KCS2 | KCS2 (3-KETOACYL-COA SYNTHASE 2); fatty acid elongase | chr1:1119853-1122483 REVERSEvery weakly similar to (60.5) THS1_ARAHY Stilbene synthase 1 (EC 2.3.1.95) (Resveratrol synthase 1) (RS1) (Trihydroxystilbene synthase 1) - Arachis hypogaea (Peanut)highly similar to ( 782) loc_os06g39750 12006.m091730 protein fatty acid elongase, putative, expressed   no original description 	T
27.3.50	RNA.regulation of transcription.General Transcription	MDP0000336563	 very weakly similar to (98.2) AT4G00850 | Symbols: GIF3 | GIF3 (GRF1-INTERACTING FACTOR 3); protein binding / transcription coactivator | chr4:357675-358928 FORWARDvery weakly similar to (87.8) loc_os12g31350 12012.m06944 protein SSXT protein, putative, expressed   no original description 	T
29.5.11.4.3.2	protein.degradation.ubiquitin.E3.SCF.FBOX	MDP0000658885	 very weakly similar to (79.0) AT5G55150 | Symbols:  | F-box family protein | chr5:22382275-22383392 REVERSEvery weakly similar to (95.9) loc_os12g05709 12012.m04564 protein F-box domain containing protein  no original description 	T
16.2.1.6	secondary metabolism.phenylpropanoids.lignin biosynthesis.CCoAOMT	MDP0000265685	 moderately similar to ( 311) AT3G62000 | Symbols:  | O-methyltransferase family 3 protein | chr3:22959441-22961180 REVERSEweakly similar to ( 142) CAMT2_MAIZE Caffeoyl-CoA O-methyltransferase 2 (EC 2.1.1.104) (Trans-caffeoyl-CoA 3-O-methyltransferase 2) (CCoAMT-2) (CCoAOMT-2) - Zea mays (Maize)moderately similar to ( 268) loc_os08g05790 12008.m080088 protein caffeoyl-CoA O-methyltransferase 1, putative, expressed  no original description 	T
29.5.11.20	protein.degradation.ubiquitin.proteasom	MDP0000319486	 moderately similar to ( 327) AT4G31300 | Symbols: PBA1 | PBA1; endopeptidase/ peptidase/ threonine-type endopeptidase | chr4:15188927-15190935 FORWARDmoderately similar to ( 323) PSB6_TOBAC Proteasome subunit beta type 6 precursor (EC 3.4.25.1) (Proteasome delta chain) (Tobacco cryptogein-induced protein 7) (tcI 7) - Nicotiana tabacum (Common tobacco)moderately similar to ( 297) loc_os02g53060 12002.m10336 protein proteasome subunit beta type 6 precursor, putative, expressed MSP1_C  no original description 	T
35.2	not assigned.unknown	MDP0000324681	   no original description 	T
35.1	not assigned.no ontology	MDP0000142019	 highly similar to ( 541) AT4G12830 | Symbols:  | hydrolase, alpha/beta fold family protein | chr4:7531189-7533327 FORWARDhighly similar to ( 531) loc_os01g69060 12001.m150825 protein hydrolase-like protein, putative, expressed     PRK12678  no original description 	T
27.3.25	RNA.regulation of transcription.MYB domain transcription factor family	MDP0000872821	 moderately similar to ( 206) AT1G22640 | Symbols: ATMYB3, MYB3 | MYB3 (MYB DOMAIN PROTEIN 3); DNA binding / transcription factor | chr1:8006289-8007219 FORWARDmoderately similar to ( 205) MYB38_MAIZE Myb-related protein Zm38 - Zea mays (Maize)moderately similar to ( 205) loc_os01g65370 12001.m12650 protein transcription repressor MYB4, putative no original description 	T
29.5.11.4.2	protein.degradation.ubiquitin.E3.RING	MDP0000252100	 very weakly similar to (75.5) AT1G23980 | Symbols:  | zinc finger (C3HC4-type RING finger) family protein | chr1:8484879-8485988 REVERSEvery weakly similar to (63.5) EL5_ORYSA E3 ubiquitin-protein ligase EL5 (EC 6.3.2.-) - Oryza sativa (Rice)very weakly similar to (88.2) loc_os02g49710 12002.m10005 protein zinc finger, C3HC4 type family protein, expressed  no original description 	T
34.2	transport.sugars	MDP0000162816	 moderately similar to ( 394) AT3G01280 | Symbols: VDAC1, ATVDAC1 | VDAC1 (VOLTAGE DEPENDENT ANION CHANNEL 1); voltage-gated anion channel | chr3:85754-87612 FORWARDmoderately similar to ( 457) VDAC2_SOLTU 36 kDa outer mitochondrial membrane protein porin (Voltage-dependent anion-selective channel protein) (VDAC) (POM 36) - Solanum tuberosum (Potato)moderately similar to ( 369) loc_os09g19734 12009.m50200 protein isochorismate synthase 1, chloroplast precursor, putative, expressed   no original description 	T
30.5	signalling.G-proteins	MDP0000297746	 nearly identical (1787) AT3G55410 | Symbols:  | 2-oxoglutarate dehydrogenase E1 component, putative / oxoglutarate decarboxylase, putative / alpha-ketoglutaric dehydrogenase, putative | chr3:20541897-20545728 FORWARDmoderately similar to ( 323) RB1BV_BETVU Ras-related protein RAB1BV - Beta vulgaris (Sugar beet)nearly identical (1704) loc_os07g49520 12007.m09160 protein 2-oxoglutarate dehydrogenase E1 component, mitochondrial precursor, putative, expressed  no original description 	T
29.4	protein.postranslational modification	MDP0000307573	 moderately similar to ( 342) AT2G25620 | Symbols:  | protein phosphatase 2C, putative / PP2C, putative | chr2:10903154-10904978 REVERSEweakly similar to ( 138) RS11_SOYBN 40S ribosomal protein S11 - Glycine max (Soybean)moderately similar to ( 453) loc_os06g08140 12006.m05536 protein DNA-binding protein phosphatase 2C, putative, expressed   no original description 	T
35.2	not assigned.unknown	MDP0000314213	 moderately similar to ( 402) AT2G35260 | Symbols:  | unknown protein | chr2:14849642-14851601 FORWARD   no original description 	T
35.2	not assigned.unknown	MDP0000255483	 weakly similar to ( 178) AT4G31980 | Symbols:  | unknown protein | chr4:15464905-15469204 FORWARDweakly similar to ( 153) loc_os08g42570 12008.m08235 protein expressed protein  no original description 	T
35.2	not assigned.unknown	MDP0000268941	 moderately similar to ( 217) AT3G44380 | Symbols:  | FUNCTIONS IN: molecular_function unknown; INVOLVED IN: biological_process unknown; LOCATED IN: cellular_component unknown; EXPRESSED IN: 19 plant structures; EXPRESSED DURING: 13 growth stages; CONTAINS InterPro DOMAIN/s: Water Stress and Hypersensitive response (InterPro:IPR013990), Harpin-induced 1 (InterPro:IPR010847); BEST Arabidopsis thaliana protein match is: unknown protein (TAIR:AT1G52330.1); Has 88 Blast hits to 87 proteins in 8 species: Archae - 0; Bacteria - 0; Metazoa - 0; Fungi - 0; Plants - 88; Viruses - 0; Other Eukaryotes - 0 (source: NCBI BLink). | chr3:16036254-16036814 REVERSEweakly similar to ( 159) loc_os08g44410 12008.m08414 protein harpin-induced protein, putative   no original description 	T
29.4	protein.postranslational modification	MDP0000291595	 highly similar to ( 715) AT3G44850 | Symbols:  | protein kinase-related | chr3:16374617-16376931 REVERSEvery weakly similar to (63.9) MPK10_ORYSA Mitogen-activated protein kinase 10 (EC 2.7.11.24) (MAP kinase 10) - Oryza sativa (Rice)highly similar to ( 678) loc_os03g53720 12003.m10342 protein ATP binding protein, putative, expressed  no original description 	T
35.2	not assigned.unknown	MDP0000322107	 highly similar to ( 611) AT5G23850 | Symbols:  | FUNCTIONS IN: molecular_function unknown; INVOLVED IN: biological_process unknown; LOCATED IN: cellular_component unknown; EXPRESSED IN: 23 plant structures; EXPRESSED DURING: 13 growth stages; CONTAINS InterPro DOMAIN/s: Lipopolysaccharide-modifying protein (InterPro:IPR006598), Protein of unknown function DUF821, CAP10-like (InterPro:IPR008539); BEST Arabidopsis thaliana protein match is: unknown protein (TAIR:AT3G48980.1); Has 588 Blast hits to 567 proteins in 103 species: Archae - 2; Bacteria - 34; Metazoa - 301; Fungi - 69; Plants - 115; Viruses - 3; Other Eukaryotes - 64 (source: NCBI BLink). | chr5:8038126-8040741 FORWARDweakly similar to ( 148) ADF2_PETHY Actin-depolymerizing factor 2 (ADF 2) - Petunia hybrida (Petunia)highly similar to ( 511) loc_os02g42920 12002.m09327 protein lipopolysaccharide-modifying protein, putative, expressed  no original description 	T
33.99	development.unspecified	MDP0000433881	 highly similar to ( 575) AT1G18080 | Symbols: ATARCA, RACK1A_AT | ATARCA; nucleotide binding | chr1:6222325-6223901 FORWARDhighly similar to ( 591) GBLP_SOYBN Guanine nucleotide-binding protein subunit beta-like protein - Glycine max (Soybean)moderately similar to ( 487) loc_os01g49290 12001.m11115 protein guanine nucleotide-binding protein beta subunit-like protein, putative, expressed   no original description 	T
12.2.99	N-metabolism.ammonia metabolism.unspecified	MDP0000856758	 moderately similar to ( 335) AT4G25840 | Symbols: GPP1 | GPP1 (glycerol-3-phosphatase 1); catalytic/ hydrolase | chr4:13139026-13140719 FORWARDmoderately similar to ( 294) loc_os08g14580 12008.m05582 protein haloacid dehalogenase-like hydrolase domain-containing protein 1A, putative, expressed no original description 	T
11.8.1	lipid metabolism.'exotics' (steroids, squalene etc).sphingolipids	MDP0000231050	 highly similar to ( 630) AT3G61580 | Symbols:  | delta-8 sphingolipid desaturase (SLD1) | chr3:22786253-22787602 FORWARDvery weakly similar to (68.9) CYB5_TOBAC Cytochrome b5 - Nicotiana tabacum (Common tobacco)moderately similar to ( 449) loc_os09g16920 12009.m04952 protein desaturase/cytochrome b5 protein, putative, expressed   no original description 	T
35.2	not assigned.unknown	MDP0000920793	 moderately similar to ( 244) AT1G60060 | Symbols:  | unknown protein | chr1:22139282-22141585 FORWARDmoderately similar to ( 245) loc_os06g12740 12006.m05990 protein expressed protein   no original description 	T
29.2.1.2.1.5	protein.synthesis.ribosomal protein.eukaryotic.40S subunit.S5	MDP0000288917	 moderately similar to ( 324) AT2G37270 | Symbols: ATRPS5B | ATRPS5B (RIBOSOMAL PROTEIN 5B); structural constituent of ribosome | chr2:15647883-15649042 REVERSEmoderately similar to ( 327) RS5_CICAR 40S ribosomal protein S5 (Fragment) - Cicer arietinum (Chickpea) (Garbanzo)moderately similar to ( 320) loc_os11g29190 12011.m080000 protein 40S ribosomal protein S5, putative, expressed  no original description 	T
29.3.1	protein.targeting.nucleus	MDP0000258120	 nearly identical (1489) AT2G31660 | Symbols: SAD2, URM9 | SAD2 (SUPER SENSITIVE TO ABA AND DROUGHT2); binding / protein transporter | chr2:13464519-13471353 FORWARDnearly identical (1112) loc_os04g26841 12004.m07810 protein importin-8, putative  no original description 	T
35.2	not assigned.unknown	MDP0000230240	 very weakly similar to (84.3) AT5G42146 | Symbols:  | unknown protein | chr5:16844405-16844818 FORWARDvery weakly similar to (52.0) loc_os03g64100 12003.m11274 protein expressed protein no original description 	T
29.7.3	protein.glycosylation.mannosyl-oligosaccharide alpha-1,2-mannosidase 	MDP0000156362	 highly similar to ( 706) AT1G51590 | Symbols:  | mannosyl-oligosaccharide 1,2-alpha-mannosidase, putative | chr1:19128315-19131406 REVERSEhighly similar to ( 663) loc_os04g51690 12004.m10064 protein mannosyl-oligosaccharide 1,2-alpha-mannosidase IA, putative, expressed   no original description 	T
29.5.7	protein.degradation.metalloprotease	MDP0000489101	 moderately similar to ( 471) AT2G41790 | Symbols:  | peptidase M16 family protein / insulinase family protein | chr2:17429453-17436110 REVERSEmoderately similar to ( 426) loc_os07g38260 12007.m08073 protein insulin-degrading enzyme, putative, expressed  no original description 	T
34.99	transport.misc	MDP0000156343	 highly similar to ( 536) AT5G01990 | Symbols:  | auxin efflux carrier family protein | chr5:377373-379600 REVERSEmoderately similar to ( 467) loc_os01g60230 12001.m150756 protein auxin hydrogen symporter, putative, expressed  no original description 	T
35.2	not assigned.unknown	MDP0000206559	 highly similar to ( 659) AT4G28760 | Symbols:  | unknown protein | chr4:14208640-14211811 FORWARDmoderately similar to ( 366) loc_os04g42770 12004.m35169 protein expressed protein     no original description 	T
30.11	signalling.light	MDP0000713403	 highly similar to ( 755) AT5G66560 | Symbols:  | phototropic-responsive NPH3 family protein | chr5:26564368-26566662 FORWARDmoderately similar to ( 341) NPH3_ORYSA Coleoptile phototropism protein 1 (Non-phototropic hypocotyl 3-like protein) (NPH3-like protein) - Oryza sativa (Rice)highly similar to ( 574) loc_os03g55830 12003.m10494 protein transposon protein, putative, Mutator sub-class, expressed    no original description 	T
29.5.11.4.3.2	protein.degradation.ubiquitin.E3.SCF.FBOX	MDP0000672291	 weakly similar to ( 187) AT5G07610 | Symbols:  | F-box family protein | chr5:2406068-2407330 FORWARDweakly similar to ( 116) loc_os02g41910 12002.m09228 protein F-box domain containing protein, expressed MSP1_C  no original description 	T
29.4.1.57	protein.postranslational modification.kinase.receptor like cytoplasmatic kinase VII	MDP0000165526	 highly similar to ( 609) AT2G02800 | Symbols: APK2B | APK2B (PROTEIN KINASE 2B); ATP binding / kinase/ protein kinase/ protein serine/threonine kinase | chr2:796889-799250 REVERSEmoderately similar to ( 216) NORK_MEDTR Nodulation receptor kinase precursor (EC 2.7.11.1) (Does not make infections protein 2) (Symbiosis receptor-like kinase) (MtSYMRK) - Medicago truncatula (Barrel medic)highly similar to ( 579) loc_os03g07430 12003.m06257 protein serine/threonine-protein kinase NAK, putative, expressed   no original description 	T
26.22	misc.short chain dehydrogenase/reductase (SDR)	MDP0000217451	 moderately similar to ( 291) AT3G61220 | Symbols:  | short-chain dehydrogenase/reductase (SDR) family protein | chr3:22663025-22664316 FORWARDvery weakly similar to (58.5) SDR1_PICAB Short-chain type dehydrogenase/reductase (EC 1.-.-.-) - Picea abies (Norway spruce) (Picea excelsa)moderately similar to ( 276) loc_os04g44950 12004.m09449 protein carbonyl reductase 1, putative, expressed  no original description 	T
35.2	not assigned.unknown	MDP0000816544	 moderately similar to ( 409) AT4G13150 | Symbols:  | unknown protein | chr4:7650326-7652237 REVERSEmoderately similar to ( 341) loc_os02g09440 12002.m06241 protein expressed protein  no original description 	T
29.5.11.4.5.2	protein.degradation.ubiquitin.E3.BTB/POZ Cullin3.BTB/POZ	MDP0000176133	 nearly identical (1019) AT5G19330 | Symbols:  | armadillo/beta-catenin repeat family protein / BTB/POZ domain-containing protein | chr5:6508300-6512701 REVERSEvery weakly similar to (71.6) IMA1B_ORYSA Importin alpha-1b subunit - Oryza sativa (Rice)highly similar to ( 964) loc_os05g33050 12005.m07537 protein protein binding protein, putative, expressed   no original description 	T
21.1	redox.thioredoxin	MDP0000763506	 highly similar to ( 672) AT1G21750 | Symbols: ATPDIL1-1 | ATPDIL1-1 (PDI-LIKE 1-1); protein disulfide isomerase | chr1:7645767-7648695 FORWARDhighly similar to ( 726) PDI_RICCO Protein disulfide-isomerase precursor (EC 5.3.4.1) (PDI) - Ricinus communis (Castor bean)highly similar to ( 634) loc_os11g09280 12011.m28750 protein OsPDIL1-1 - Oryza sativa protein disulfide isomerase, expressed  no original description 	T
17.6.3	hormone metabolism.gibberelin.induced-regulated-responsive-activated	MDP0000150771	 weakly similar to ( 126) AT5G59845 | Symbols:  | gibberellin-regulated family protein | chr5:24111443-24111808 FORWARDweakly similar to ( 113) loc_os03g55290 12003.m10444 protein gibberellin-regulated protein 2 precursor, putative, expressed  no original description 	T
13.1.3.6.1.1	amino acid metabolism.synthesis.aspartate family.misc.homoserine.aspartate kinase	MDP0000304794	 moderately similar to ( 406) AT5G14060 | Symbols: CARAB-AK-LYS | CARAB-AK-LYS; amino acid binding / aspartate kinase | chr5:4536143-4538993 FORWARDweakly similar to ( 113) AKH2_MAIZE Bifunctional aspartokinase/homoserine dehydrogenase 2, chloroplast precursor (AK-HD 2) (AK-HSDH 2) [Includes: Aspartokinase (EC 2.7.2.4); Homoserine dehydrogenase (EC 1.1.1.3)] - Zea mays (Maize)moderately similar to ( 409) loc_os07g20544 12007.m29379 protein lysine-sensitive aspartokinase 3, putative, expressed  no original description 	T
30.5	signalling.G-proteins	MDP0000516451	 moderately similar to ( 372) AT2G38630 | Symbols:  | FUNCTIONS IN: molecular_function unknown; INVOLVED IN: biological_process unknown; LOCATED IN: chloroplast; CONTAINS InterPro DOMAIN/s: WD40 repeat-like (InterPro:IPR011046); BEST Arabidopsis thaliana protein match is: unknown protein (TAIR:AT3G54190.1); Has 79 Blast hits to 77 proteins in 21 species: Archae - 0; Bacteria - 0; Metazoa - 0; Fungi - 0; Plants - 53; Viruses - 0; Other Eukaryotes - 26 (source: NCBI BLink). | chr2:16154283-16156302 REVERSEmoderately similar to ( 363) loc_os05g40410 12005.m08217 protein expressed protein  no original description 	T
35.2	not assigned.unknown	MDP0000188253	 weakly similar to ( 121) AT5G16660 | Symbols:  | unknown protein | chr5:5465699-5467006 REVERSEvery weakly similar to (74.3) loc_os12g39370 12012.m073869 protein expressed protein no original description 	T
35.2	not assigned.unknown	MDP0000166304	 weakly similar to ( 105) AT5G63135 | Symbols:  | unknown protein | chr5:25325253-25326440 REVERSE no original description 	T
17.1.1.1.10	hormone metabolism.abscisic acid.synthesis-degradation.synthesis.9-cis-epoxycarotenoid dioxygenase	MDP0000813805	 highly similar to ( 843) AT3G14440 | Symbols: NCED3, ATNCED3, STO1, SIS7 | NCED3 (NINE-CIS-EPOXYCAROTENOID DIOXYGENASE 3); 9-cis-epoxycarotenoid dioxygenase | chr3:4831678-4833477 REVERSEhighly similar to ( 735) loc_os03g44380 12003.m09477 protein 9-cis-epoxycarotenoid dioxygenase 2, putative, expressed   no original description 	T
27.3.24	RNA.regulation of transcription.MADS box transcription factor family	MDP0000255146	 very weakly similar to (98.2) AT4G24540 | Symbols: AGL24 | AGL24 (AGAMOUS-LIKE 24); protein binding / protein heterodimerization/ protein homodimerization/ sequence-specific DNA binding / transcription factor | chr4:12671160-12673645 REVERSEvery weakly similar to (87.8) MAD47_ORYSA MADS-box transcription factor 47 (OsMADS47) - Oryza sativa (Rice)very weakly similar to (87.8) loc_os03g08754 12003.m35564 protein MADS-box transcription factor 47, putative, expressed   no original description 	T
35.2	not assigned.unknown	MDP0000357399	  no original description 	T
35.2	not assigned.unknown	MDP0000263024	 very weakly similar to (80.5) AT5G52180 | Symbols:  | unknown protein | chr5:21200165-21201541 REVERSE COG3889 PRK08026  no original description 	T
35.2	not assigned.unknown	MDP0000282203	 moderately similar to ( 408) AT2G30900 | Symbols:  | unknown protein | chr2:13150484-13152417 FORWARDmoderately similar to ( 374) loc_os01g46350 12001.m10833 protein expressed protein   no original description 	T
34.16	transport.ABC transporters and multidrug resistance systems	MDP0000200335	 nearly identical (1059) AT1G17840 | Symbols: WBC11, ABCG11, DSO, COF1, ATWBC11 | WBC11 (WHITE-BROWN COMPLEX HOMOLOG PROTEIN 11); ATPase, coupled to transmembrane movement of substances / fatty acid transporter | chr1:6142870-6145894 FORWARDweakly similar to ( 154) PDR2_ORYSA Probable pleiotropic drug resistance protein 2 - Oryza sativa (Rice)nearly identical (1001) loc_os10g35180 12010.m06341 protein ATP-binding cassette sub-family G member 2, putative, expressed   TrbL  no original description 	T
20.2.3	stress.abiotic.drought/salt	MDP0000783692	 highly similar to ( 982) AT1G33170 | Symbols:  | dehydration-responsive family protein | chr1:12027262-12030397 FORWARDhighly similar to ( 983) loc_os06g01450 12006.m04884 protein methyltransferase, putative, expressed   no original description 	T
11.1.12	lipid metabolism.FA synthesis and FA elongation.ACP protein	MDP0000548191	 weakly similar to ( 109) AT4G25050 | Symbols: ACP4 | ACP4 (acyl carrier protein 4); acyl carrier | chr4:12870178-12871024 FORWARDweakly similar to ( 117) ACP1_HORVU Acyl carrier protein 1, chloroplast precursor (Acyl carrier protein I) (ACP I) - Hordeum vulgare (Barley)weakly similar to ( 112) loc_os12g34890 12012.m07282 protein acyl carrier protein 1, chloroplast precursor, putative, expressed  no original description 	T
17.5.1	hormone metabolism.ethylene.synthesis-degradation	MDP0000798019	 moderately similar to ( 416) AT1G06650 | Symbols:  | 2-oxoglutarate-dependent dioxygenase, putative | chr1:2035909-2037186 FORWARDmoderately similar to ( 372) DV4H_CATRO Desacetoxyvindoline 4-hydroxylase (EC 1.14.11.20) - Catharanthus roseus (Rosy periwinkle) (Madagascar periwinkle)moderately similar to ( 333) loc_os04g10350 12004.m06287 protein 1-aminocyclopropane-1-carboxylate oxidase, putative, expressed  no original description 	T
35.1	not assigned.no ontology	MDP0000259250	 moderately similar to ( 294) AT4G11220 | Symbols: BTI2 | BTI2 (VIRB2-INTERACTING PROTEIN 2) | chr4:6838176-6839578 REVERSEmoderately similar to ( 284) loc_os05g45050 12005.m083785 protein seed maturation protein, putative, expressed no original description 	T
16.4.1	secondary metabolism.N misc.alkaloid-like	MDP0000141727	 highly similar to ( 572) AT1G08470 | Symbols:  | strictosidine synthase family protein | chr1:2682262-2683977 REVERSEweakly similar to ( 161) STSY_CATRO Strictosidine synthase precursor (EC 4.3.3.2) - Catharanthus roseus (Rosy periwinkle) (Madagascar periwinkle)highly similar to ( 502) loc_os03g53950 12003.m10360 protein strictosidine synthase precursor, putative, expressed TrbL  no original description 	T
30.2.11	signalling.receptor kinases.leucine rich repeat XI	MDP0000131641	 highly similar to ( 962) AT4G20940 | Symbols:  | leucine-rich repeat family protein | chr4:11202728-11206038 FORWARDmoderately similar to ( 254) PSKR_DAUCA Phytosulfokine receptor precursor (EC 2.7.11.1) (Phytosulfokine LRR receptor kinase) - Daucus carota (Carrot)nearly identical (1266) loc_os07g05190 12007.m04983 protein protein binding protein, putative, expressed  no original description 	T
34.99	transport.misc	MDP0000186482	 highly similar to ( 882) AT2G05760 | Symbols:  | xanthine/uracil permease family protein | chr2:2180978-2183710 FORWARDhighly similar to ( 753) loc_os01g55500 12001.m150722 protein solute carrier family 23 member 2, putative, expressed   no original description 	T
26.28	misc.GDSL-motif lipase	MDP0000218469	 moderately similar to ( 455) AT1G71691 | Symbols:  | GDSL-motif lipase/hydrolase family protein | chr1:26949395-26951173 REVERSEweakly similar to ( 112) APG_BRANA Anter-specific proline-rich protein APG (Protein CEX) (Fragment) - Brassica napus (Rape)moderately similar to ( 408) loc_os05g34700 12005.m27828 protein anther-specific proline-rich protein APG, putative, expressed no original description 	T
35.2	not assigned.unknown	MDP0000631736	  no original description 	T
29.2.4	protein.synthesis.elongation	MDP0000304140	 highly similar to ( 857) AT5G60390 | Symbols:  | elongation factor 1-alpha / EF-1-alpha | chr5:24289226-24290675 FORWARDhighly similar to ( 863) EF1A_MAIZE Elongation factor 1-alpha (EF-1-alpha) - Zea mays (Maize)highly similar to ( 862) loc_os03g08050 12003.m78808 protein elongation factor 1-alpha, putative, expressed   no original description 	T
35.2	not assigned.unknown	MDP0000827881	  no original description 	T
29.5.11.3	protein.degradation.ubiquitin.E2	MDP0000285227	 moderately similar to ( 308) AT1G64230 | Symbols: UBC28 | ubiquitin-conjugating enzyme, putative | chr1:23833792-23835220 FORWARDweakly similar to ( 143) UBC2_WHEAT Ubiquitin-conjugating enzyme E2-17 kDa (EC 6.3.2.19) (Ubiquitin-protein ligase) (Ubiquitin carrier protein) - Triticum aestivum (Wheat)moderately similar to ( 305) loc_os02g16040 12002.m100164 protein ubiquitin-conjugating enzyme E2-17 kDa, putative, expressed  no original description 	T
29.5.11.4.3.2	protein.degradation.ubiquitin.E3.SCF.FBOX	MDP0000265164	 moderately similar to ( 307) AT1G78650 | Symbols: POLD3 | POLD3; DNA-directed DNA polymerase | chr1:29582987-29585508 FORWARDmoderately similar to ( 285) loc_os01g10690 12001.m07689 protein expressed protein no original description 	T
35.2	not assigned.unknown	MDP0000133483	 weakly similar to ( 168) AT3G25400 | Symbols:  | FUNCTIONS IN: molecular_function unknown; INVOLVED IN: biological_process unknown; LOCATED IN: cellular_component unknown; EXPRESSED IN: 11 plant structures; EXPRESSED DURING: 6 growth stages; CONTAINS InterPro DOMAIN/s: NTP Pyrophosphohydrolase MazG-related, RS21-C6 (InterPro:IPR011394), EAR (InterPro:IPR009039), NTP pyrophosphohydrolase MazG, putative catalytic core (InterPro:IPR004518); Has 572 Blast hits to 572 proteins in 181 species: Archae - 11; Bacteria - 286; Metazoa - 71; Fungi - 1; Plants - 31; Viruses - 0; Other Eukaryotes - 172 (source: NCBI BLink). | chr3:9213236-9214144 FORWARDweakly similar to ( 175) loc_os04g43550 12004.m09315 protein mazG nucleotide pyrophosphohydrolase domain protein, putative, expressed no original description 	T
30.2.11	signalling.receptor kinases.leucine rich repeat XI	MDP0000144320	 nearly identical (1494) AT2G26330 | Symbols: ER, QRP1 | ER (ERECTA); transmembrane receptor protein kinase | chr2:11208367-11213895 REVERSEmoderately similar to ( 288) RPK1_IPONI Receptor-like protein kinase precursor (EC 2.7.11.1) - Ipomoea nil (Japanese morning glory) (Pharbitis nil)nearly identical (1364) loc_os06g10230 12006.m32010 protein receptor-like protein kinase 5 precursor, putative, expressed  no original description 	T
17.2.3	hormone metabolism.auxin.induced-regulated-responsive-activated	MDP0000233483	 highly similar to ( 737) AT2G46370 | Symbols: JAR1, FIN219 | JAR1 (JASMONATE RESISTANT 1); ATP binding / adenylyltransferase/ catalytic/ jasmonate-amino synthetase | chr2:19034579-19036369 FORWARDhighly similar to ( 760) GH35_ORYSA Probable indole-3-acetic acid-amido synthetase GH3.5 (EC 6.3.2.-) (Auxin-responsive GH3-like protein 5) (OsGH3-5) - Oryza sativa (Rice)highly similar to ( 760) loc_os05g50890 12005.m27991 protein indole-3-acetic acid-amido synthetase GH3.5, putative, expressed  no original description 	T
34.99	transport.misc	MDP0000312567	 moderately similar to ( 281) AT1G26690 | Symbols:  | emp24/gp25L/p24 family protein | chr1:9224299-9225682 REVERSEmoderately similar to ( 283) loc_os07g43890 12007.m08613 protein transmembrane emp24 domain-containing protein 10 precursor, putative, expressed no original description 	T
11.9.1	lipid metabolism.lipid degradation.palmitoyl[protein] hydrolase	MDP0000184385	 moderately similar to ( 439) AT3G60340 | Symbols:  | palmitoyl protein thioesterase family protein | chr3:22304395-22306389 FORWARDmoderately similar to ( 401) loc_os03g01150 12003.m05666 protein palmitoyl-protein thioesterase 1 precursor, putative, expressed  no original description 	T
27.3.24	RNA.regulation of transcription.MADS box transcription factor family	MDP0000144597	 weakly similar to ( 182) AT5G62165 | Symbols: AGL42 | AGL42 (AGAMOUS LIKE 42); transcription factor | chr5:24965075-24968437 FORWARDweakly similar to ( 152) MAD50_ORYSA MADS-box transcription factor 50 (OsMADS50) (Protein SUPPRESSOR OF CONSTANS OVEREXPRESSION 1-like) (OsSOC1) (Protein AGAMOUS-like 20) (RMADS208) - Oryza sativa (Rice)weakly similar to ( 143) loc_os10g39130 12010.m21952 protein MADS-box transcription factor 56, putative, expressed no original description 	T
4.3.5	glycolysis.unclear/dually targeted.pyrophosphate-fructose-6-P phosphotransferase	MDP0000265468	 highly similar to ( 865) AT1G12000 | Symbols:  | pyrophosphate--fructose-6-phosphate 1-phosphotransferase beta subunit, putative / pyrophosphate-dependent 6-phosphofructose-1-kinase, putative | chr1:4050159-4053727 REVERSEhighly similar to ( 888) PFPB_RICCO Pyrophosphate--fructose 6-phosphate 1-phosphotransferase subunit beta (EC 2.7.1.90) (PFP) (6-phosphofructokinase, pyrophosphate dependent) (Pyrophosphate-dependent 6-phosphofructose-1-kinase) (PPi-PFK) - Ricinus communis (Castor bean)highly similar to ( 842) loc_os06g13810 12006.m31864 protein pyrophosphate--fructose 6-phosphate 1-phosphotransferase beta subunit, putative, expressed   no original description 	T
30.2.17	signalling.receptor kinases.DUF 26	MDP0000278907	 highly similar to ( 813) AT1G56130 | Symbols:  | leucine-rich repeat family protein / protein kinase family protein | chr1:20994931-21000887 REVERSEmoderately similar to ( 216) NORK_PEA Nodulation receptor kinase precursor (EC 2.7.11.1) - Pisum sativum (Garden pea)highly similar to ( 797) loc_os04g52600 12004.m10153 protein protein kinase domain containing protein, expressed  no original description 	T
35.2	not assigned.unknown	MDP0000887913	  no original description 	T
29.5	protein.degradation	MDP0000172131	 moderately similar to ( 258) AT5G22860 | Symbols:  | serine carboxypeptidase S28 family protein | chr5:7639907-7642945 REVERSEweakly similar to ( 194) loc_os11g05760 12011.m04772 protein lysosomal Pro-X carboxypeptidase precursor, putative, expressed no original description 	T
35.2	not assigned.unknown	MDP0000820971	 moderately similar to ( 248) AT4G34340 | Symbols: TAF8 | TAF8 (TBP-ASSOCIATED FACTOR 8); DNA binding | chr4:16426771-16427832 REVERSEmoderately similar to ( 216) loc_os12g38620 12012.m26943 protein bromodomain associated family protein, expressed  no original description 	T
35.2	not assigned.unknown	MDP0000398863	 very weakly similar to (73.2) AT3G24535 | Symbols:  | unknown protein | chr3:8952006-8952572 FORWARD  no original description 	T
23.3.2.1	nucleotide metabolism.salvage.nucleoside kinases.adenosine kinase	MDP0000301789	 highly similar to ( 613) AT5G03300 | Symbols: ADK2 | ADK2 (ADENOSINE KINASE 2); adenosine kinase/ copper ion binding / kinase | chr5:796573-798997 FORWARDmoderately similar to ( 467) ADK_PHYPA Adenosine kinase (EC 2.7.1.20) (AK) (Adenosine 5'-phosphotransferase) - Physcomitrella patens (Moss)highly similar to ( 593) loc_os04g43750 12004.m101587 protein adenosine kinase 2, putative, expressed  no original description 	T
31.3.1	cell.cycle.peptidylprolyl isomerase	MDP0000124610	 moderately similar to ( 312) AT2G29960 | Symbols: CYP5, ATCYP5, CYP19-4 | CYP5 (CYCLOPHILIN 5); peptidyl-prolyl cis-trans isomerase | chr2:12769183-12770528 REVERSEmoderately similar to ( 222) CYPH_CATRO Peptidyl-prolyl cis-trans isomerase (EC 5.2.1.8) (PPIase) (Rotamase) (Cyclophilin) (Cyclosporin A-binding protein) - Catharanthus roseus (Rosy periwinkle) (Madagascar periwinkle)moderately similar to ( 310) loc_os06g49480 12006.m32182 protein peptidyl-prolyl cis-trans isomerase CYP19-4 precursor, putative, expressed  no original description 	T
34.3	transport.amino acids	MDP0000822921	 highly similar to ( 533) AT3G28960 | Symbols:  | amino acid transporter family protein | chr3:10984245-10985767 REVERSEmoderately similar to ( 458) loc_os04g38680 12004.m08857 protein amino acid/polyamine transporter II, putative, expressed  no original description 	T
29.2.1.2.2.29	protein.synthesis.ribosomal protein.eukaryotic.60S subunit.L29	MDP0000231729	 weakly similar to ( 110) AT3G06680 | Symbols:  | 60S ribosomal protein L29 (RPL29B) | chr3:2114402-2114724 REVERSEweakly similar to ( 106) loc_os05g28750 12005.m07160 protein 60S ribosomal protein L29, putative, expressed no original description 	T
29.6	protein.folding	MDP0000276724	 highly similar to ( 918) AT3G18190 | Symbols:  | chaperonin, putative | chr3:6232226-6233836 FORWARDmoderately similar to ( 316) TCPE2_AVESA T-complex protein 1 subunit epsilon (TCP-1-epsilon) (CCT-epsilon) (TCP-K36) - Avena sativa (Oat)highly similar to ( 840) loc_os02g22780 12002.m100196 protein T-complex protein 1 subunit delta, putative, expressed    no original description 	T
29.5	protein.degradation	MDP0000150964	 moderately similar to ( 462) AT1G02170 | Symbols: LOL3, ATMCPB1, MCP1B, AMC1, ATMC1 | AMC1 (METACASPASE 1); cysteine-type endopeptidase | chr1:411883-413426 FORWARDmoderately similar to ( 363) loc_os10g41534 12010.m21976 protein ICE-like protease p20 domain containing protein, expressed   no original description 	T
35.2	not assigned.unknown	MDP0000158437	 highly similar to ( 578) AT5G23850 | Symbols:  | FUNCTIONS IN: molecular_function unknown; INVOLVED IN: biological_process unknown; LOCATED IN: cellular_component unknown; EXPRESSED IN: 23 plant structures; EXPRESSED DURING: 13 growth stages; CONTAINS InterPro DOMAIN/s: Lipopolysaccharide-modifying protein (InterPro:IPR006598), Protein of unknown function DUF821, CAP10-like (InterPro:IPR008539); BEST Arabidopsis thaliana protein match is: unknown protein (TAIR:AT3G48980.1); Has 588 Blast hits to 567 proteins in 103 species: Archae - 2; Bacteria - 34; Metazoa - 301; Fungi - 69; Plants - 115; Viruses - 3; Other Eukaryotes - 64 (source: NCBI BLink). | chr5:8038126-8040741 FORWARDmoderately similar to ( 456) loc_os02g42920 12002.m09327 protein lipopolysaccharide-modifying protein, putative, expressed  no original description 	T
26.21	misc.protease inhibitor/seed storage/lipid transfer protein (LTP) family protein	MDP0000727860	 very weakly similar to (73.9) AT5G48485 | Symbols: DIR1 | DIR1 (DEFECTIVE IN INDUCED RESISTANCE 1); lipid binding / lipid transporter | chr5:19646317-19646625 REVERSEvery weakly similar to (68.2) loc_os07g18750 12007.m06314 protein PVR3-like protein, putative, expressed   no original description 	T
26.10	misc.cytochrome P450	MDP0000879787	 moderately similar to ( 462) AT4G31940 | Symbols: CYP82C4 | CYP82C4; electron carrier/ heme binding / iron ion binding / monooxygenase/ oxygen binding | chr4:15452040-15453966 FORWARDhighly similar to ( 518) C82A1_PEA Cytochrome P450 82A1 (EC 1.14.-.-) (CYPLXXXII) (Fragment) - Pisum sativum (Garden pea)moderately similar to ( 331) loc_os09g26980 12009.m05850 protein flavonoid 3-monooxygenase, putative, expressed   no original description 	T
27.3.26	RNA.regulation of transcription.MYB-related transcription factor family	MDP0000230601	 weakly similar to ( 192) AT1G74840 | Symbols:  | myb family transcription factor | chr1:28116201-28117317 REVERSEweakly similar to ( 124) loc_os10g41200 12010.m21804 protein ZmMybst1, putative, expressed no original description 	T
34.22	transport.cyclic nucleotide or calcium regulated channels	MDP0000251604	 moderately similar to ( 307) AT5G53130 | Symbols: CNGC1, ATCNGC1 | CNGC1 (CYCLIC NUCLEOTIDE GATED CHANNEL 1); calmodulin binding / cation channel/ cyclic nucleotide binding / intracellular cyclic nucleotide activated cation channel/ inward rectifier potassium channel | chr5:21537830-21540490 REVERSEmoderately similar to ( 251) loc_os06g33570 12006.m07894 protein cyclic nucleotide-gated ion channel 1, putative, expressed no original description 	T
29.5.11.4.2	protein.degradation.ubiquitin.E3.RING	MDP0000950987	 weakly similar to ( 158) AT4G03510 | Symbols: RMA1 | RMA1; protein binding / ubiquitin-protein ligase/ zinc ion binding | chr4:1557905-1558654 REVERSEweakly similar to ( 105) loc_os01g56070 12001.m150727 protein RING finger protein 5, putative, expressed   no original description 	T
35.1.5	not assigned.no ontology.pentatricopeptide (PPR) repeat-containing protein	MDP0000875888	 highly similar to ( 555) AT1G80550 | Symbols:  | pentatricopeptide (PPR) repeat-containing protein | chr1:30285358-30286704 REVERSEweakly similar to ( 127) RF1_ORYSA Rf1 protein, mitochondrial precursor (PPR protein) (Fertility restorer) (Restorer for CMS) - Oryza sativa (Rice)moderately similar to ( 445) loc_os04g55090 12004.m101673 protein rf1 protein, mitochondrial precursor, putative, expressed   no original description 	T
30.3	signalling.calcium	MDP0000183898	 moderately similar to ( 298) AT3G43810 | Symbols: CAM7 | CAM7 (CALMODULIN 7); calcium ion binding | chr3:15664619-15666355 REVERSEmoderately similar to ( 296) CALM_LILLO Calmodulin (CaM) - Lilium longiflorum (Trumpet lily)moderately similar to ( 296) loc_os07g48780 12007.m29366 protein calmodulin, putative, expressed no original description 	T
29.3.3	protein.targeting.chloroplast	MDP0000149360	 moderately similar to ( 388) AT1G04940 | Symbols: TIC20 | TIC20 (TRANSLOCON AT THE INNER ENVELOPE MEMBRANE OF CHLOROPLASTS 20) | chr1:1399588-1400616 FORWARDmoderately similar to ( 211) loc_os01g15660 12001.m97451 protein nucleoside-diphosphate-sugar epimerase, putative, expressed no original description 	T
26.28	misc.GDSL-motif lipase	MDP0000196666	 moderately similar to ( 481) AT3G26430 | Symbols:  | GDSL-motif lipase/hydrolase family protein | chr3:9674419-9675889 FORWARDmoderately similar to ( 406) EST_HEVBR Esterase precursor (EC 3.1.1.-) (Early nodule-specific protein homolog) (Latex allergen Hev b 13) - Hevea brasiliensis (Para rubber tree)moderately similar to ( 450) loc_os07g39750 12007.m079815 protein alpha-L-fucosidase 2 precursor, putative, expressed  no original description 	T
29.5.11.4.2	protein.degradation.ubiquitin.E3.RING	MDP0000637978	 nearly identical (3239) AT2G22125 | Symbols:  | binding | chr2:9406793-9414223 FORWARDvery weakly similar to (58.9) SPL11_ORYSA Spotted leaf protein 11 (Spotted leaf11) (Cell death-related protein SPL11) - Oryza sativa (Rice)nearly identical (1709) loc_os11g08090 12011.m05005 protein armadillo/beta-catenin-like repeat family protein, expressed   no original description 	T
26.9	misc.glutathione S transferases	MDP0000181272	 moderately similar to ( 215) AT2G30860 | Symbols: ATGSTF9, GLUTTR, ATGSTF7 | ATGSTF9 (GLUTATHIONE S-TRANSFERASE PHI 9); glutathione peroxidase/ glutathione transferase | chr2:13139132-13140079 FORWARDweakly similar to ( 114) GSTF3_MAIZE Glutathione S-transferase III (EC 2.5.1.18) (GST-III) (GST class-phi) - Zea mays (Maize)weakly similar to ( 132) loc_os03g04250 12003.m05956 protein glutathione S-transferase 6, putative, expressed no original description 	T
30.5	signalling.G-proteins	MDP0000824072	 moderately similar to ( 211) AT1G52380 | Symbols:  | Ran-binding protein 1 domain-containing protein / RanBP1 domain-containing protein | chr1:19509979-19511301 FORWARDweakly similar to ( 190) loc_os05g28190 12005.m07106 protein ranBP1 domain containing protein, expressed  no original description 	T
29.5.11.20	protein.degradation.ubiquitin.proteasom	MDP0000477688	 moderately similar to ( 446) AT4G24820 | Symbols:  | 26S proteasome regulatory subunit, putative (RPN7) | chr4:12790471-12792599 REVERSEmoderately similar to ( 444) PSMD6_ORYSA 26S proteasome non-ATPase regulatory subunit 6 (26S proteasome regulatory particle non-ATPase subunit 7) (OsRPN7) - Oryza sativa (Rice)moderately similar to ( 444) loc_os04g40850 12004.m35346 protein 26S proteasome non-ATPase regulatory subunit 6, putative, expressed  no original description 	T
35.2	not assigned.unknown	MDP0000171268	 moderately similar to ( 231) AT1G54520 | Symbols:  | unknown protein | chr1:20363565-20365874 FORWARDmoderately similar to ( 206) loc_os03g48920 12003.m35367 protein membrane protein, putative, expressed   no original description 	T
29.3.4.99	protein.targeting.secretory pathway.unspecified	MDP0000138028	 highly similar to ( 551) AT4G24550 | Symbols:  | clathrin adaptor complexes medium subunit family protein | chr4:12675873-12678903 FORWARDhighly similar to ( 538) loc_os07g42810 12007.m29006 protein AP-4 complex subunit mu-1, putative, expressed  no original description 	T
35.1	not assigned.no ontology	MDP0000230325	 moderately similar to ( 481) AT2G37730 | Symbols:  | fringe-related protein | chr2:15822186-15824538 REVERSEmoderately similar to ( 405) loc_os03g16334 12003.m07069 protein transferase, transferring glycosyl groups, putative, expressed   no original description 	T
35.2	not assigned.unknown	MDP0000271873	 weakly similar to ( 108) AT1G67080 | Symbols: ABA4 | ABA4 (abscisic acid (ABA)-deficient 4); intramolecular oxidoreductase | chr1:25045405-25046681 REVERSEvery weakly similar to (91.3) loc_os01g03750 12001.m07012 protein expressed protein no original description 	T
30.3	signalling.calcium	MDP0000664492	 moderately similar to ( 271) AT5G04170 | Symbols:  | calcium-binding EF hand family protein | chr5:1145579-1147519 FORWARDmoderately similar to ( 277) loc_os12g04240 12012.m26827 protein grancalcin, putative, expressed  no original description 	T
35.2	not assigned.unknown	MDP0000182420	  MSP1_C  no original description 	T
13.1.5.1.1	amino acid metabolism.synthesis.serine-glycine-cysteine group.serine.phosphoglycerate dehydrogenase	MDP0000309160	 highly similar to ( 774) AT1G17745 | Symbols: PGDH | PGDH (3-PHOSPHOGLYCERATE DEHYDROGENASE); phosphoglycerate dehydrogenase | chr1:6101157-6104979 FORWARDweakly similar to ( 110) DHGY_CUCSA Glycerate dehydrogenase (EC 1.1.1.29) (NADH-dependent hydroxypyruvate reductase) (HPR) (GDH) - Cucumis sativus (Cucumber)highly similar to ( 751) loc_os06g44460 12006.m08974 protein D-3-phosphoglycerate dehydrogenase, chloroplast precursor, putative, expressed  MSP1_C  no original description 	T
29.3.1	protein.targeting.nucleus	MDP0000123549	 moderately similar to ( 355) AT5G43960 | Symbols:  | nuclear transport factor 2 (NTF2) family protein / RNA recognition motif (RRM)-containing protein | chr5:17689154-17691653 REVERSEmoderately similar to ( 273) loc_os02g03890 12002.m05738 protein RNA binding protein, putative, expressed  no original description 	T
29.5.5	protein.degradation.serine protease	MDP0000203790	 moderately similar to ( 353) AT1G33540 | Symbols: scpl18 | scpl18 (serine carboxypeptidase-like 18); serine-type carboxypeptidase | chr1:12162349-12164700 REVERSEmoderately similar to ( 251) CBP1_ORYSA Serine carboxypeptidase 1 precursor (EC 3.4.16.5) (Serine carboxypeptidase I) (Carboxypeptidase C) - Oryza sativa (Rice)moderately similar to ( 335) loc_os04g09720 12004.m35249 protein serine carboxypeptidase 1 precursor, putative, expressed  no original description 	T
23.1.1.10	nucleotide metabolism.synthesis.pyrimidine.CTP synthetase	MDP0000137919	 highly similar to ( 716) AT4G20320 | Symbols:  | CTP synthase/ catalytic | chr4:10974980-10978998 FORWARDhighly similar to ( 687) loc_os01g43020 12001.m10565 protein CTP synthase, putative, expressed  no original description 	T
31.1	cell.organisation	MDP0000163222	 nearly identical (1029) AT5G48460 | Symbols:  | fimbrin-like protein, putative | chr5:19636225-19639365 REVERSEhighly similar to ( 872) loc_os02g48740 12002.m09908 protein fimbrin 1, putative, expressed  no original description 	T
3.5	minor CHO metabolism.others	MDP0000300285	 moderately similar to ( 351) AT5G58730 | Symbols:  | pfkB-type carbohydrate kinase family protein | chr5:23723627-23725324 REVERSEmoderately similar to ( 259) loc_os03g52760 12003.m10250 protein carbohydrate kinase, PfkB, putative, expressed   no original description 	T
29.4	protein.postranslational modification	MDP0000319801	 highly similar to ( 620) AT1G54510 | Symbols: ATNEK1 | ATNEK1 (ARABIDOPSIS THALIANA NIMA-RELATED SERINE/THREONINE KINASE 1); ATP binding / kinase/ protein kinase/ protein serine/threonine kinase | chr1:20358603-20362006 REVERSEvery weakly similar to ( 100) M2K1_ORYSA Mitogen-activated protein kinase kinase 1 (EC 2.7.12.2) (MAP kinase kinase 1) (MAPKK1) (OsMEK1) - Oryza sativa (Rice)highly similar to ( 610) loc_os12g41180 12012.m07900 protein LSTK-1-like kinase, putative, expressed   no original description 	T
30.10	signalling.phosphorelay	MDP0000061368	 weakly similar to ( 181) AT3G21510 | Symbols: AHP1 | AHP1 (HISTIDINE-CONTAINING PHOSPHOTRANSMITTER 1); histidine phosphotransfer kinase | chr3:7578432-7579537 REVERSEweakly similar to ( 116) HP1_ORYSA Histidine-containing phosphotransfer protein 1 (OsHP1) - Oryza sativa (Rice)weakly similar to ( 122) loc_os08g44350 12008.m08408 protein histidine-containing phosphotransfer protein 5, putative, expressed no original description 	T
35.2	not assigned.unknown	MDP0000130786	 weakly similar to ( 189) AT1G73840 | Symbols: ESP1 | ESP1 (ENHANCED SILENCING PHENOTYPE 1) | chr1:27763937-27766328 REVERSEweakly similar to ( 108) loc_os05g43780 12005.m08504 protein proline-rich protein precursor, putative, expressed   no original description 	T
30.2.17	signalling.receptor kinases.DUF 26	MDP0000197292	 moderately similar to ( 426) AT4G21410 | Symbols:  | protein kinase family protein | chr4:11402463-11405025 REVERSEweakly similar to ( 140) PSKR_DAUCA Phytosulfokine receptor precursor (EC 2.7.11.1) (Phytosulfokine LRR receptor kinase) - Daucus carota (Carrot)moderately similar to ( 373) loc_os05g41370 12005.m08312 protein receptor-like protein kinase homolog RK20-1, putative, expressed  no original description 	T
35.2	not assigned.unknown	MDP0000328020	  no original description 	T
25.7	C1-metabolism.GTP cyclohydrolase I	MDP0000256036	 highly similar to ( 608) AT3G07270 | Symbols:  | GTP cyclohydrolase I | chr3:2314005-2317059 FORWARDmoderately similar to ( 377) loc_os04g56710 12004.m10558 protein GTP cyclohydrolase I 1, putative, expressed no original description 	T
35.2	not assigned.unknown	MDP0000146787	 very weakly similar to (92.4) loc_os06g11030 12006.m05820 protein EF hand family protein no original description 	T
35.2	not assigned.unknown	MDP0000302894	 moderately similar to ( 361) AT4G17420 | Symbols:  | FUNCTIONS IN: molecular_function unknown; INVOLVED IN: biological_process unknown; LOCATED IN: cellular_component unknown; EXPRESSED IN: 23 plant structures; EXPRESSED DURING: 13 growth stages; CONTAINS InterPro DOMAIN/s: Protein of unknown function DUF124 (InterPro:IPR002838), Tryptophan RNA-binding attenuator protein-like (InterPro:IPR016031); BEST Arabidopsis thaliana protein match is: unknown protein (TAIR:AT5G47420.1). | chr4:9722378-9724443 REVERSEmoderately similar to ( 308) loc_os03g53600 12003.m101496 protein expressed protein   no original description 	T
11.8.1	lipid metabolism.'exotics' (steroids, squalene etc).sphingolipids	MDP0000124297	 moderately similar to ( 434) AT1G13580 | Symbols: LAG13 | LAG13 (LAG1 LONGEVITY ASSURANCE HOMOLOG 3) | chr1:4645006-4646765 REVERSEmoderately similar to ( 416) ASCL1_ORYSA ASC1-like protein 1 (Alternaria stem canker resistance-like protein 1) - Oryza sativa (Rice)moderately similar to ( 416) loc_os02g37080 12002.m08796 protein ASC1-like protein 1, putative, expressed   no original description 	T
27.4	RNA.RNA binding	MDP0000136330	 moderately similar to ( 429) AT1G47490 | Symbols: ATRBP47C | ATRBP47C (ARABIDOPSIS THALIANA RNA-BINDING PROTEIN 47C); RNA binding | chr1:17424801-17427182 FORWARDvery weakly similar to (84.7) ROC3_NICSY 28 kDa ribonucleoprotein, chloroplast precursor (28RNP) - Nicotiana sylvestris (Wood tobacco)moderately similar to ( 431) loc_os09g28810 12009.m06032 protein expressed protein MSP1_C  no original description 	T
35.2	not assigned.unknown	MDP0000792375	 moderately similar to ( 269) AT3G45900 | Symbols:  | unknown protein | chr3:16874566-16875984 REVERSEmoderately similar to ( 243) loc_os03g63660 12003.m11234 protein conserved hypothetical protein no original description 	T
17.6.2	hormone metabolism.gibberelin.signal transduction	MDP0000846748	 nearly identical (1460) AT3G11540 | Symbols: SPY | SPY (SPINDLY); protein N-acetylglucosaminyltransferase/ protein binding / transferase, transferring glycosyl groups | chr3:3632842-3637547 FORWARDnearly identical (1478) SPY_PETHY Probable UDP-N-acetylglucosamine--peptide N-acetylglucosaminyltransferase SPINDLY (EC 2.4.1.-) (PhSPY) - Petunia hybrida (Petunia)nearly identical (1352) loc_os08g44510 12008.m08424 protein UDP-N-acetylglucosamine--peptide N-acetylglucosaminyltransferase SPINDLY, putative, expressed   no original description 	T
13.1.3.1.1	amino acid metabolism.synthesis.aspartate family.asparagine.asparagine synthetase	MDP0000292272	 highly similar to ( 998) AT5G65010 | Symbols: ASN2 | ASN2 (ASPARAGINE SYNTHETASE 2); asparagine synthase (glutamine-hydrolyzing) | chr5:25969224-25972278 FORWARDhighly similar to ( 968) ASNS_ORYSA Asparagine synthetase [glutamine-hydrolyzing] (EC 6.3.5.4) (Glutamine-dependent asparagine synthetase) - Oryza sativa (Rice)highly similar to ( 970) loc_os06g15420 12006.m06255 protein asparagine synthetase, putative, expressed   no original description 	T
29.5.11.5	protein.degradation.ubiquitin.ubiquitin protease	MDP0000229858	 moderately similar to ( 371) AT5G46740 | Symbols: UBP21 | UBP21 (UBIQUITIN-SPECIFIC PROTEASE 21); ubiquitin thiolesterase/ ubiquitin-specific protease | chr5:18965410-18968492 REVERSEmoderately similar to ( 270) loc_os02g46650 12002.m09702 protein ubiquitin-specific protease 21, putative, expressed   no original description 	T
10.1.7	cell wall.precursor synthesis.GMD	MDP0000303268	 highly similar to ( 648) AT3G51160 | Symbols: MUR1, MUR_1, GMD2 | MUR1 (MURUS 1); GDP-mannose 4,6-dehydratase | chr3:19007232-19008353 REVERSEhighly similar to ( 578) loc_os06g04620 12006.m05192 protein GDP-mannose 4,6 dehydratase 2, putative, expressed  TrbL  no original description 	T
10.7	cell wall.modification	MDP0000208105	 moderately similar to ( 435) AT1G14720 | Symbols: XTR2, EXGT-A2, ATXTH28 | XTR2 (XYLOGLUCAN ENDOTRANSGLYCOSYLASE RELATED 2); hydrolase, acting on glycosyl bonds / xyloglucan:xyloglucosyl transferase | chr1:5066806-5068466 REVERSEweakly similar to ( 189) XTH_WHEAT Probable xyloglucan endotransglucosylase/hydrolase precursor (EC 2.4.1.207) - Triticum aestivum (Wheat)moderately similar to ( 361) loc_os03g13570 12003.m06802 protein xyloglucan endotransglucosylase/hydrolase protein 27 precursor, putative, expressed  no original description 	T
34.16	transport.ABC transporters and multidrug resistance systems	MDP0000149064	 nearly identical (1001) AT3G21090 | Symbols:  | ABC transporter family protein | chr3:7391497-7394933 REVERSEmoderately similar to ( 216) PDR15_ORYSA Pleiotropic drug resistance protein 15 - Oryza sativa (Rice)highly similar to ( 845) loc_os05g13520 12005.m05767 protein CER5, putative, expressed   no original description 	T
35.2	not assigned.unknown	MDP0000192468	   no original description 	T
27.3.67	RNA.regulation of transcription.putative transcription regulator	MDP0000197983	 moderately similar to ( 354) AT2G38560 | Symbols: TFIIS | TFIIS (TRANSCRIPT ELONGATION FACTOR IIS); DNA binding / RNA polymerase II transcription elongation factor/ transcription factor | chr2:16134802-16136319 FORWARDmoderately similar to ( 367) loc_os03g60130 12003.m101543 protein transcription elongation factor A protein 2, putative, expressed   no original description 	T
29.2.1.2.2.82	protein.synthesis.ribosomal protein.eukaryotic.60S subunit.P2	MDP0000293903	 very weakly similar to (62.4) AT3G44590 | Symbols:  | 60S acidic ribosomal protein P2 (RPP2D) | chr3:16163983-16164658 FORWARDvery weakly similar to (60.5) RLA2A_MAIZE 60S acidic ribosomal protein P2A (P2) - Zea mays (Maize)very weakly similar to (61.2) loc_os02g32760 12002.m08369 protein 60S acidic ribosomal protein P2A, putative, expressed  no original description 	T
35.2	not assigned.unknown	MDP0000787176	 weakly similar to ( 103) AT1G60870 | Symbols: MEE9 | MEE9 (maternal effect embryo arrest 9) | chr1:22409712-22410227 FORWARD   no original description 	T
27.3.20	RNA.regulation of transcription.G2-like transcription factor family, GARP	MDP0000234120	 weakly similar to ( 108) AT2G40260 | Symbols:  | myb family transcription factor | chr2:16816818-16818473 REVERSEweakly similar to ( 114) loc_os04g47890 12004.m09729 protein myb-like DNA-binding domain, SHAQKYF class family protein, expressed  TrbL  no original description 	T
20.2.1	stress.abiotic.heat	MDP0000213302	 moderately similar to ( 382) AT2G42750 | Symbols:  | DNAJ heat shock N-terminal domain-containing protein | chr2:17793404-17795419 FORWARDmoderately similar to ( 375) loc_os01g53020 12001.m11474 protein electron transporter/ heat shock protein binding protein, putative, expressed  no original description 	T
29.5.3	protein.degradation.cysteine protease	MDP0000197825	 very weakly similar to (88.6) AT5G47550 | Symbols:  | cysteine protease inhibitor, putative / cystatin, putative | chr5:19286596-19286964 REVERSEvery weakly similar to (65.9) CYT1_MAIZE Cystatin-1 precursor (Cystatin I) (Corn kernel cysteine proteinase inhibitor) - Zea mays (Maize)very weakly similar to (73.9) loc_os03g31510 12003.m08398 protein cystatin, putative, expressed  no original description 	T
35.2	not assigned.unknown	MDP0000199780	 nearly identical (1568) AT3G62360 | Symbols:  | carbohydrate binding | chr3:23073020-23080455 REVERSEnearly identical (1372) loc_os01g19470 12001.m08484 protein nodal modulator 1 precursor, putative, expressed   no original description 	T
30.2.99	signalling.receptor kinases.misc	MDP0000307165	 highly similar to ( 770) AT1G79620 | Symbols:  | leucine-rich repeat transmembrane protein kinase, putative | chr1:29957633-29962174 REVERSEmoderately similar to ( 239) NORK_MEDTR Nodulation receptor kinase precursor (EC 2.7.11.1) (Does not make infections protein 2) (Symbiosis receptor-like kinase) (MtSYMRK) - Medicago truncatula (Barrel medic)highly similar to ( 684) loc_os03g21230 12003.m07529 protein protein kinase, putative, expressed  no original description 	T
29.4.1.57	protein.postranslational modification.kinase.receptor like cytoplasmatic kinase VII	MDP0000307165	 highly similar to ( 770) AT1G79620 | Symbols:  | leucine-rich repeat transmembrane protein kinase, putative | chr1:29957633-29962174 REVERSEmoderately similar to ( 239) NORK_MEDTR Nodulation receptor kinase precursor (EC 2.7.11.1) (Does not make infections protein 2) (Symbiosis receptor-like kinase) (MtSYMRK) - Medicago truncatula (Barrel medic)highly similar to ( 684) loc_os03g21230 12003.m07529 protein protein kinase, putative, expressed  no original description 	T
35.2	not assigned.unknown	MDP0000500737	 very weakly similar to (56.2) AT2G40475 | Symbols:  | unknown protein | chr2:16907222-16907803 REVERSEvery weakly similar to (52.0) loc_os01g07240 12001.m07350 protein expressed protein  no original description 	T
35.2	not assigned.unknown	MDP0000470996	 moderately similar to ( 459) AT1G21370 | Symbols:  | unknown protein | chr1:7484250-7485451 FORWARDmoderately similar to ( 429) loc_os04g35780 12004.m08667 protein cytoplasm protein, putative, expressed no original description 	T
30.3	signalling.calcium	MDP0000306089	 moderately similar to ( 327) AT4G26470 | Symbols:  | calcium ion binding | chr4:13371377-13372197 FORWARDvery weakly similar to (71.2) CDPK2_MAIZE Calcium-dependent protein kinase 2 (EC 2.7.11.1) (CDPK 2) - Zea mays (Maize)moderately similar to ( 325) loc_os06g40720 12006.m08607 protein calcium ion binding protein, putative, expressed no original description 	T
35.2	not assigned.unknown	MDP0000232300	 moderately similar to ( 357) AT1G55480 | Symbols:  | binding / protein binding | chr1:20713822-20715351 FORWARDmoderately similar to ( 332) loc_os07g07540 12007.m28951 protein SHOOT1 protein, putative, expressed  no original description 	T
28.1	DNA.synthesis/chromatin structure	MDP0000226188	 moderately similar to ( 371) AT1G63160 | Symbols:  | replication factor C 40 kDa, putative | chr1:23422068-23423771 REVERSEmoderately similar to ( 209) RL24_HORVU 60S ribosomal protein L24 - Hordeum vulgare (Barley)moderately similar to ( 374) loc_os04g48060 12004.m09746 protein replication factor C subunit 4, putative, expressed   no original description 	T
29.2.1.2.2.24	protein.synthesis.ribosomal protein.eukaryotic.60S subunit.L24	MDP0000226188	 moderately similar to ( 371) AT1G63160 | Symbols:  | replication factor C 40 kDa, putative | chr1:23422068-23423771 REVERSEmoderately similar to ( 209) RL24_HORVU 60S ribosomal protein L24 - Hordeum vulgare (Barley)moderately similar to ( 374) loc_os04g48060 12004.m09746 protein replication factor C subunit 4, putative, expressed   no original description 	T
20.1.7	stress.biotic.PR-proteins	MDP0000139844	 weakly similar to ( 191) AT1G55210 | Symbols:  | disease resistance response | chr1:20598057-20598620 REVERSEvery weakly similar to (57.8) DR206_PEA Disease resistance response protein 206 - Pisum sativum (Garden pea)weakly similar to ( 150) loc_os11g07830 12011.m04979 protein dirigent-like protein pDIR9, putative, expressed  no original description 	T
34.13	transport.peptides and oligopeptides	MDP0000296517	 highly similar to ( 734) AT5G62680 | Symbols:  | proton-dependent oligopeptide transport (POT) family protein | chr5:25165430-25167822 REVERSEhighly similar to ( 635) loc_os03g48180 12003.m101441 protein peptide transporter PTR2, putative, expressed   no original description 	T
30.11	signalling.light	MDP0000283919	 very weakly similar to (65.5) AT3G61111 | Symbols:  | structural constituent of ribosome | chr3:22613230-22613919 REVERSEvery weakly similar to (73.6) RS27_HORVU 40S ribosomal protein S27 (Manganese efficiency-related protein 1) - Hordeum vulgare (Barley)very weakly similar to (73.6) loc_os02g27769 12002.m100798 protein 40S ribosomal protein S27, putative, expressed  no original description 	T
35.2	not assigned.unknown	MDP0000231819	 highly similar to ( 555) AT1G04950 | Symbols: TAFII59 | TAF6 (TBP-ASSOCIATED FACTOR 6); DNA binding / RNA polymerase II transcription factor/ transcription initiation factor | chr1:1403606-1407184 REVERSEhighly similar to ( 651) loc_os01g32750 12001.m09594 protein TBP-associated 59 kDa subunit protein, putative, expressed  no original description 	T
20.1.7	stress.biotic.PR-proteins	MDP0000744777	 moderately similar to ( 276) AT1G69545 | Symbols:  | leucine-rich repeat family protein | chr1:26148836-26150947 REVERSEvery weakly similar to (90.9) RPK1_IPONI Receptor-like protein kinase precursor (EC 2.7.11.1) - Ipomoea nil (Japanese morning glory) (Pharbitis nil)moderately similar to ( 202) loc_os01g71106 12001.m97573 protein NBS-LRR disease resistance protein, putative, expressed  no original description 	T
30.2.11	signalling.receptor kinases.leucine rich repeat XI	MDP0000744777	 moderately similar to ( 276) AT1G69545 | Symbols:  | leucine-rich repeat family protein | chr1:26148836-26150947 REVERSEvery weakly similar to (90.9) RPK1_IPONI Receptor-like protein kinase precursor (EC 2.7.11.1) - Ipomoea nil (Japanese morning glory) (Pharbitis nil)moderately similar to ( 202) loc_os01g71106 12001.m97573 protein NBS-LRR disease resistance protein, putative, expressed  no original description 	T
30.6	signalling.MAP kinases	MDP0000854535	 highly similar to ( 633) AT5G67130 | Symbols:  | phospholipase C/ phosphoric diester hydrolase | chr5:26791599-26793649 FORWARDhighly similar to ( 533) loc_os09g02729 12009.m059932 protein MAP3K-like protein kinase, putative, expressed   no original description 	T
35.2	not assigned.unknown	MDP0000308113	 weakly similar to ( 188) AT5G20090 | Symbols:  | unknown protein | chr5:6787543-6788000 REVERSEweakly similar to ( 186) loc_os09g20660 12009.m059970 protein brain protein 44-like protein, putative, expressed no original description 	T
29.4.1.57	protein.postranslational modification.kinase.receptor like cytoplasmatic kinase VII	MDP0000007813	 nearly identical (1085) AT3G46290 | Symbols:  | protein kinase, putative | chr3:17013009-17015501 FORWARDmoderately similar to ( 265) NORK_MEDTR Nodulation receptor kinase precursor (EC 2.7.11.1) (Does not make infections protein 2) (Symbiosis receptor-like kinase) (MtSYMRK) - Medicago truncatula (Barrel medic)highly similar to ( 967) loc_os06g22810 12006.m06934 protein protein kinase, putative, expressed  no original description 	T
30.5	signalling.G-proteins	MDP0000329986	 moderately similar to ( 313) AT3G07880 | Symbols:  | Rho GDP-dissociation inhibitor family protein | chr3:2514175-2515544 FORWARDmoderately similar to ( 295) loc_os02g48730 12002.m09907 protein rho GDP-dissociation inhibitor 1, putative, expressed  no original description 	T
26.18	misc.invertase/pectin methylesterase inhibitor family protein	MDP0000503246	 moderately similar to ( 206) AT5G62350 | Symbols:  | invertase/pectin methylesterase inhibitor family protein / DC 1.2 homolog (FL5-2I22) | chr5:25037504-25038112 FORWARDmoderately similar to ( 216) 21KD_DAUCA 21 kDa protein precursor (1.2 protein) - Daucus carota (Carrot)weakly similar to ( 157) loc_os06g49760 12006.m09500 protein ripening-related protein, putative, expressed  no original description 	T
35.2	not assigned.unknown	MDP0000187385	 moderately similar to ( 242) AT4G26550 | Symbols:  | FUNCTIONS IN: molecular_function unknown; INVOLVED IN: biological_process unknown; LOCATED IN: cellular_component unknown; EXPRESSED IN: 22 plant structures; EXPRESSED DURING: 13 growth stages; CONTAINS InterPro DOMAIN/s: SFT2-like (InterPro:IPR011691); BEST Arabidopsis thaliana protein match is: unknown protein (TAIR:AT5G56020.1); Has 452 Blast hits to 452 proteins in 136 species: Archae - 0; Bacteria - 0; Metazoa - 198; Fungi - 89; Plants - 61; Viruses - 0; Other Eukaryotes - 104 (source: NCBI BLink). | chr4:13402755-13404208 REVERSEmoderately similar to ( 232) loc_os06g40630 12006.m32220 protein protein SFT2, putative, expressed no original description 	T
17.5.1.1	hormone metabolism.ethylene.synthesis-degradation.1-aminocyclopropane-1-carboxylate synthase	MDP0000250254	 highly similar to ( 557) AT1G62960 | Symbols: ACS10 | ACS10 (ACC SYNTHASE 10); 1-aminocyclopropane-1-carboxylate synthase/ L-aspartate:2-oxoglutarate aminotransferase/ aromatic-amino-acid:2-oxoglutarate aminotransferase | chr1:23318271-23320229 REVERSEmoderately similar to ( 341) 1A1C_MALDO 1-aminocyclopropane-1-carboxylate synthase (EC 4.4.1.14) (ACC synthase) (S-adenosyl-L-methionine methylthioadenosine-lyase) - Malus domestica (Apple) (Malus sylvestris)highly similar to ( 506) loc_os06g03990 12006.m05130 protein aminotransferase ACS12, putative, expressed  no original description 	T
35.1.5	not assigned.no ontology.pentatricopeptide (PPR) repeat-containing protein	MDP0000313879	 highly similar to ( 932) AT4G16390 | Symbols:  | LOCATED IN: chloroplast; EXPRESSED IN: 22 plant structures; EXPRESSED DURING: 13 growth stages; CONTAINS InterPro DOMAIN/s: Pentatricopeptide repeat (InterPro:IPR002885), Smr protein/MutS2 C-terminal (InterPro:IPR002625); BEST Arabidopsis thaliana protein match is: pentatricopeptide (PPR) repeat-containing protein (TAIR:AT5G46580.1); Has 19821 Blast hits to 5782 proteins in 183 species: Archae - 2; Bacteria - 24; Metazoa - 276; Fungi - 350; Plants - 18437; Viruses - 0; Other Eukaryotes - 732 (source: NCBI BLink). | chr4:9257985-9260093 FORWARDweakly similar to ( 135) RF1_ORYSA Rf1 protein, mitochondrial precursor (PPR protein) (Fertility restorer) (Restorer for CMS) - Oryza sativa (Rice)highly similar to ( 728) loc_os03g11670 12003.m06619 protein salt-inducible protein, putative, expressed  MSP1_C  no original description 	T
35.2	not assigned.unknown	MDP0000294443	 highly similar to ( 530) AT5G18420 | Symbols:  | unknown protein | chr5:6105638-6109337 REVERSEmoderately similar to ( 444) loc_os11g13694 12011.m05508 protein expressed protein no original description 	T
20.1.7	stress.biotic.PR-proteins	MDP0000293686	 weakly similar to ( 152) AT1G55210 | Symbols:  | disease resistance response | chr1:20598057-20598620 REVERSEweakly similar to ( 107) loc_os12g07580 12012.m04747 protein disease resistance response protein 206, putative, expressed no original description 	T
17.5.1	hormone metabolism.ethylene.synthesis-degradation	MDP0000205515	 moderately similar to ( 390) AT1G06650 | Symbols:  | 2-oxoglutarate-dependent dioxygenase, putative | chr1:2035909-2037186 FORWARDmoderately similar to ( 342) DV4H_CATRO Desacetoxyvindoline 4-hydroxylase (EC 1.14.11.20) - Catharanthus roseus (Rosy periwinkle) (Madagascar periwinkle)moderately similar to ( 318) loc_os04g10350 12004.m06287 protein 1-aminocyclopropane-1-carboxylate oxidase, putative, expressed  no original description 	T
30.2.17	signalling.receptor kinases.DUF 26	MDP0000152330	 moderately similar to ( 457) AT1G67000 | Symbols:  | ATP binding / kinase/ protein kinase/ protein serine/threonine kinase | chr1:25004217-25007604 REVERSEweakly similar to ( 170) CRI4_MAIZE Putative receptor protein kinase CRINKLY4 precursor (EC 2.7.11.1) - Zea mays (Maize)moderately similar to ( 437) loc_os01g49614 12001.m43492 protein Ser/Thr protein kinase, putative, expressed  no original description 	T
35.2	not assigned.unknown	MDP0000950997	 moderately similar to ( 421) AT3G21360 | Symbols:  | electron carrier/ oxidoreductase | chr3:7522865-7524036 FORWARDmoderately similar to ( 379) loc_os01g09430 12001.m07568 protein syringomycin biosynthesis enzyme, putative, expressed  no original description 	T
28.1.3	DNA.synthesis/chromatin structure.histone	MDP0000481249	 weakly similar to ( 159) AT1G51060 | Symbols: HTA10 | HTA10; DNA binding | chr1:18926948-18927443 FORWARDweakly similar to ( 161) H2A2_ORYSA Probable histone H2A.2 - Oryza sativa (Rice)weakly similar to ( 161) loc_os07g36140 12007.m079709 protein histone H2A.3, putative, expressed no original description 	T
29.5.11.20	protein.degradation.ubiquitin.proteasom	MDP0000641719	 moderately similar to ( 220) AT2G05840 | Symbols: PAA2 | PAA2 (20S PROTEASOME SUBUNIT PAA2); endopeptidase/ peptidase/ threonine-type endopeptidase | chr2:2234226-2235973 FORWARDmoderately similar to ( 229) PSA6_TOBAC Proteasome subunit alpha type 6 (EC 3.4.25.1) (20S proteasome alpha subunit A) (20S proteasome subunit alpha-1) - Nicotiana tabacum (Common tobacco)moderately similar to ( 218) loc_os03g08280 12003.m101213 protein proteasome subunit alpha type 6, putative, expressed no original description 	T
35.2	not assigned.unknown	MDP0000212614	 moderately similar to ( 305) AT3G01680 | Symbols:  | unknown protein | chr3:252033-255246 FORWARD  no original description 	T
28.2	DNA.repair	MDP0000288182	 highly similar to ( 501) AT3G22880 | Symbols: ATDMC1, DMC1, ARLIM15 | DMC1 (DISRUPTION OF MEIOTIC CONTROL 1); ATP binding / DNA binding / DNA-dependent ATPase/ damaged DNA binding / nucleoside-triphosphatase/ nucleotide binding / protein binding | chr3:8097948-8100740 REVERSEhighly similar to ( 526) DMC1_SOYBN Meiotic recombination protein DMC1 homolog - Glycine max (Soybean)moderately similar to ( 481) loc_os11g04954 12011.m079904 protein meiotic recombination protein DMC1, putative, expressed no original description 	T
29.4.1	protein.postranslational modification.kinase	MDP0000242195	 moderately similar to ( 265) AT5G03420 | Symbols:  | FUNCTIONS IN: molecular_function unknown; INVOLVED IN: biological_process unknown; LOCATED IN: cellular_component unknown; EXPRESSED IN: 22 plant structures; EXPRESSED DURING: 13 growth stages; BEST Arabidopsis thaliana protein match is: 5'-AMP-activated protein kinase-related (TAIR:AT1G27070.1); Has 1365 Blast hits to 895 proteins in 182 species: Archae - 6; Bacteria - 436; Metazoa - 414; Fungi - 132; Plants - 56; Viruses - 2; Other Eukaryotes - 319 (source: NCBI BLink). | chr5:845641-848705 FORWARDvery weakly similar to (65.5) loc_os03g48170 12003.m09827 protein isoamylase N-terminal domain containing protein, expressed  MSP1_C  no original description 	T
8.2.11	TCA / org transformation.other organic acid transformatons.atp-citrate lyase	MDP0000240828	 highly similar to ( 708) AT1G10670 | Symbols: ACLA-1 | ACLA-1; ATP citrate synthase | chr1:3535787-3538098 FORWARDhighly similar to ( 671) loc_os11g47330 12011.m28910 protein ATP-citrate synthase, putative, expressed  no original description 	T
26.19	misc.plastocyanin-like	MDP0000516844	 very weakly similar to (68.9) AT5G15350 | Symbols:  | plastocyanin-like domain-containing protein | chr5:4985184-4986154 REVERSEweakly similar to ( 108) loc_os09g38540 12009.m06795 protein blue copper protein precursor, putative, expressed  no original description 	T
34.3	transport.amino acids	MDP0000893755	 highly similar to ( 917) AT1G58030 | Symbols: CAT2 | CAT2 (CATIONIC AMINO ACID TRANSPORTER 2); amino acid transmembrane transporter | chr1:21464185-21468141 REVERSEhighly similar to ( 835) loc_os12g42850 12012.m26983 protein high-affinity cationic amino acid transporter 1, putative, expressed  no original description 	T
26.8	misc.nitrilases, *nitrile lyases, berberine bridge enzymes, reticuline oxidases, troponine reductases	MDP0000163690	 weakly similar to ( 167) AT3G50440 | Symbols: ATMES10, MES10 | MES10 (METHYL ESTERASE 10); hydrolase/ hydrolase, acting on ester bonds / methyl jasmonate esterase | chr3:18717392-18718435 REVERSEweakly similar to ( 150) PIR7A_ORYSA Probable esterase PIR7A (EC 3.1.-.-) - Oryza sativa (Rice)weakly similar to ( 164) loc_os01g70840 12001.m13129 protein esterase PIR7B, putative, expressed no original description 	T
1.1.1.2	PS.lightreaction.photosystem II.PSII polypeptide subunits	MDP0000195878	 very weakly similar to (95.9) AT2G30570 | Symbols: PSBW | PSBW (PHOTOSYSTEM II REACTION CENTER W) | chr2:13019326-13020053 REVERSEvery weakly similar to ( 100) PSBW_SPIOL Photosystem II reaction center W protein, chloroplast precursor (PSII 6.1 kDa protein) - Spinacia oleracea (Spinach)very weakly similar to (73.6) loc_os01g56680 12001.m11825 protein photosystem II reaction center W protein, chloroplast precursor, putative, expressed no original description 	T
26.13	misc.acid and other phosphatases	MDP0000156048	 moderately similar to ( 397) AT2G01890 | Symbols: PAP8, ATPAP8 | PAP8 (PURPLE ACID PHOSPHATASE 8); acid phosphatase/ protein serine/threonine phosphatase | chr2:396985-399168 REVERSEmoderately similar to ( 401) loc_os03g13540 12003.m06799 protein purple acid phosphatase 1, putative, expressed no original description 	T
21.2	redox.ascorbate and glutathione	MDP0000228733	 moderately similar to ( 204) AT5G48810 | Symbols: ATB5-B, B5 #3, ATCB5-D, CB5-D | CB5-D (CYTOCHROME B5 ISOFORM D); heme binding | chr5:19789249-19790180 REVERSEmoderately similar to ( 231) CYB5_TOBAC Cytochrome b5 - Nicotiana tabacum (Common tobacco)moderately similar to ( 211) loc_os05g01820 12005.m04718 protein cytochrome b5, putative, expressed  no original description 	T
29.8	protein.assembly and cofactor ligation	MDP0000146870	 moderately similar to ( 427) AT5G54290 | Symbols:  | cytochrome c biogenesis protein family | chr5:22051097-22053923 FORWARDmoderately similar to ( 416) loc_os11g04500 12011.m04648 protein expressed protein no original description 	T
30.2.24	signalling.receptor kinases.S-locus glycoprotein like	MDP0000560468	 highly similar to ( 541) AT1G78830 | Symbols:  | curculin-like (mannose-binding) lectin family protein | chr1:29637141-29638508 REVERSEmoderately similar to ( 278) EP1G_DAUCA Epidermis-specific secreted glycoprotein EP1 precursor (52/54 kDa medium protein) - Daucus carota (Carrot)weakly similar to ( 138) loc_os04g23640 12004.m07505 protein ATP binding protein, putative, expressed  no original description 	T
27.3.69	RNA.regulation of transcription.SET-domain transcriptional regulator family	MDP0000146310	 highly similar to ( 598) AT3G21820 | Symbols: ATXR2, SDG36 | ATXR2 (HISTONE-LYSINE N-METHYLTRANSFERASE ATXR2); zinc ion binding | chr3:7688629-7691444 REVERSEhighly similar to ( 505) loc_os04g53700 12004.m10263 protein MYND finger family protein, expressed no original description 	T
34.16	transport.ABC transporters and multidrug resistance systems	MDP0000183294	 nearly identical (1963) AT2G36910 | Symbols: ATPGP1, PGP1, ABCB1 | ABCB1 (ATP BINDING CASSETTE SUBFAMILY B1); ATPase, coupled to transmembrane movement of substances / auxin efflux transmembrane transporter/ calmodulin binding | chr2:15502162-15507050 FORWARDhighly similar to ( 946) MDR_ORYSA Putative multidrug resistance protein (P-glycoprotein) - Oryza sativa (Rice)nearly identical (1691) loc_os08g45030 12008.m08473 protein multidrug resistance protein 1, putative, expressed   no original description 	T
35.1	not assigned.no ontology	MDP0000325723	 highly similar to ( 615) AT1G48790 | Symbols:  | mov34 family protein | chr1:18043925-18047427 REVERSEmoderately similar to ( 429) loc_os01g31470 12001.m09478 protein mov34/MPN/PAD-1 family protein, expressed  no original description 	T
29.6	protein.folding	MDP0000130405	 moderately similar to ( 260) AT1G18170 | Symbols:  | immunophilin / FKBP-type peptidyl-prolyl cis-trans isomerase family protein | chr1:6254291-6255507 FORWARDmoderately similar to ( 247) loc_os03g50080 12003.m10003 protein FK506 binding protein, putative, expressed   no original description 	T
10.6.1	cell wall.degradation.cellulases and beta -1,4-glucanases	MDP0000119414	 highly similar to ( 930) AT5G20950 | Symbols:  | glycosyl hydrolase family 3 protein | chr5:7107609-7110775 REVERSEhighly similar to ( 915) loc_os03g53800 12003.m101132 protein periplasmic beta-glucosidase precursor, putative, expressed  no original description 	T
30.5	signalling.G-proteins	MDP0000299673	 moderately similar to ( 359) AT2G17800 | Symbols: ARAC1, ATGP2, ATRAC1, ROP3, ATROP3 | ARAC1; GTP binding | chr2:7740313-7741942 FORWARDmoderately similar to ( 358) RAC1_BETVU Rac-like GTP-binding protein RHO1 (RHO1Bv) - Beta vulgaris (Sugar beet)moderately similar to ( 350) loc_os02g02840 12002.m05634 protein rac-like GTP-binding protein 6, putative, expressed  no original description 	T
10.6.3	cell wall.degradation.pectate lyases and polygalacturonases	MDP0000164054	 highly similar to ( 817) AT1G70370 | Symbols:  | BURP domain-containing protein / polygalacturonase, putative | chr1:26513003-26514998 REVERSEvery weakly similar to (57.4) EA92_VICFA Embryonic abundant protein USP92 precursor - Vicia faba (Broad bean)highly similar to ( 622) loc_os08g29200 12008.m06924 protein polygalacturonase-1 non-catalytic beta subunit precursor, putative, expressed   no original description 	T
35.2	not assigned.unknown	MDP0000127046	 moderately similar to ( 266) AT4G34340 | Symbols: TAF8 | TAF8 (TBP-ASSOCIATED FACTOR 8); DNA binding | chr4:16426771-16427832 REVERSEmoderately similar to ( 224) loc_os12g38620 12012.m26943 protein bromodomain associated family protein, expressed  no original description 	T
1.1.1.2	PS.lightreaction.photosystem II.PSII polypeptide subunits	MDP0000327226	 weakly similar to ( 181) ATCG00020 | Symbols: PSBA | Encodes chlorophyll binding protein D1, a part of the photosystem II reaction center core | chrC:383-1444 REVERSEweakly similar to ( 181) PSBA_WHEAT Photosystem Q(B) protein (32 kDa thylakoid membrane protein) (Photosystem II protein D1) - Triticum aestivum (Wheat)weakly similar to ( 181) loc_os08g35420 12008.m07531 protein photosystem Q, putative, expressed no original description 	T
21.1	redox.thioredoxin	MDP0000626628	 weakly similar to ( 188) AT3G15360 | Symbols: ATHM4, TRX-M4, ATM4 | TRX-M4 (ARABIDOPSIS THIOREDOXIN M-TYPE 4); enzyme activator | chr3:5188448-5189457 FORWARDweakly similar to ( 171) TRXM_PEA Thioredoxin M-type, chloroplast precursor (TRX-M) - Pisum sativum (Garden pea)weakly similar to ( 171) loc_os04g44830 12004.m09440 protein thioredoxin M-type 2, chloroplast precursor, putative, expressed  no original description 	T
27.3.67	RNA.regulation of transcription.putative transcription regulator	MDP0000233149	 highly similar to ( 883) AT5G18230 | Symbols:  | transcription regulator NOT2/NOT3/NOT5 family protein | chr5:6021610-6027031 REVERSEhighly similar to ( 838) loc_os03g44900 12003.m35336 protein CCR4-NOT transcription complex subunit 3, putative, expressed   no original description 	T
34.99	transport.misc	MDP0000268229	 highly similar to ( 504) AT1G33110 | Symbols:  | MATE efflux family protein | chr1:12005084-12008040 FORWARDmoderately similar to ( 500) loc_os12g03260 12012.m04324 protein transparent testa 12 protein, putative, expressed  no original description 	T
35.2	not assigned.unknown	MDP0000707824	 moderately similar to ( 261) AT4G31980 | Symbols:  | unknown protein | chr4:15464905-15469204 FORWARDweakly similar to ( 195) loc_os01g21710 12001.m08655 protein conserved hypothetical protein  no original description 	T
35.2	not assigned.unknown	MDP0000842137	 very weakly similar to (97.1) AT2G46225 | Symbols: ABIL1 | ABIL1 (Abi-1-like 1) | chr2:18982113-18984074 FORWARDweakly similar to ( 115) ABIL5_ORYSA Probable protein ABIL5 (Abl interactor-like protein 5) - Oryza sativa (Rice)weakly similar to ( 115) loc_os01g55560 12001.m11713 protein expressed protein no original description 	T
35.2	not assigned.unknown	MDP0000155087	 highly similar to ( 674) AT3G16200 | Symbols:  | unknown protein | chr3:5491619-5493605 REVERSEhighly similar to ( 595) loc_os01g31220 12001.m09453 protein expressed protein  no original description 	T
30.2.17	signalling.receptor kinases.DUF 26	MDP0000253714	 moderately similar to ( 369) AT1G04520 | Symbols: PDLP2 | PDLP2 (PLASMODESMATA-LOCATED PROTEIN 2) | chr1:1231852-1233787 FORWARDweakly similar to ( 114) loc_os04g45460 12004.m09499 protein secretory protein-like, putative, expressed    no original description 	T
30.2.99	signalling.receptor kinases.misc	MDP0000253714	 moderately similar to ( 369) AT1G04520 | Symbols: PDLP2 | PDLP2 (PLASMODESMATA-LOCATED PROTEIN 2) | chr1:1231852-1233787 FORWARDweakly similar to ( 114) loc_os04g45460 12004.m09499 protein secretory protein-like, putative, expressed    no original description 	T
29.5.11.3	protein.degradation.ubiquitin.E2	MDP0000240384	 moderately similar to ( 307) AT5G05080 | Symbols: UBC22 | UBC22 (ubiquitin-conjugating enzyme 22); ubiquitin-protein ligase | chr5:1498727-1499972 REVERSEvery weakly similar to (93.2) UBC2_MEDSA Ubiquitin-conjugating enzyme E2-17 kDa (EC 6.3.2.19) (Ubiquitin-protein ligase) (Ubiquitin carrier protein) - Medicago sativa (Alfalfa)moderately similar to ( 293) loc_os06g45000 12006.m32132 protein ubiquitin-conjugating enzyme E2S, putative, expressed  no original description 	T
35.2	not assigned.unknown	MDP0000263727	 moderately similar to ( 324) AT2G46230 | Symbols:  | FUNCTIONS IN: molecular_function unknown; INVOLVED IN: biological_process unknown; LOCATED IN: cellular_component unknown; EXPRESSED IN: 22 plant structures; EXPRESSED DURING: 13 growth stages; CONTAINS InterPro DOMAIN/s: Protein of unknown function DUF652 (InterPro:IPR006984), Nucleotide binding protein, PINc (InterPro:IPR006596); BEST Arabidopsis thaliana protein match is: unknown protein (TAIR:AT1G26530.1); Has 441 Blast hits to 441 proteins in 161 species: Archae - 15; Bacteria - 0; Metazoa - 185; Fungi - 119; Plants - 49; Viruses - 0; Other Eukaryotes - 73 (source: NCBI BLink). | chr2:18984694-18985850 REVERSEmoderately similar to ( 355) loc_os06g06410 12006.m05367 protein nucleolar protein involved in pre-mRNA processing, putative, expressed no original description 	T
34.19.4	transport.Major Intrinsic Proteins.SIP	MDP0000119818	 moderately similar to ( 316) AT3G56950 | Symbols: SIP2;1, SIP2 | SIP2;1; transporter | chr3:21078586-21079644 REVERSEvery weakly similar to (57.0) NO26_SOYBN Nodulin-26 (N-26) - Glycine max (Soybean)moderately similar to ( 240) loc_os03g20410 12003.m07450 protein aquaporin SIP2.1, putative, expressed  no original description 	T
35.2	not assigned.unknown	MDP0000499122	 weakly similar to ( 190) AT4G12590 | Symbols:  | unknown protein | chr4:7451291-7452976 REVERSEweakly similar to ( 164) loc_os09g32220 12009.m06311 protein protein pob, putative, expressed  no original description 	T
11.1.3	lipid metabolism.FA synthesis and FA elongation.ketoacyl ACP synthase	MDP0000074417	 highly similar to ( 742) AT5G46290 | Symbols: KAS I | KAS I (3-KETOACYL-ACYL CARRIER PROTEIN SYNTHASE I); catalytic/ fatty-acid synthase | chr5:18774439-18776629 REVERSEhighly similar to ( 711) KASC1_HORVU 3-oxoacyl-[acyl-carrier-protein] synthase I, chloroplast precursor (EC 2.3.1.41) (Beta-ketoacyl-ACP synthase I) (KAS I) - Hordeum vulgare (Barley)highly similar to ( 733) loc_os06g09630 12006.m05681 protein 3-oxoacyl-synthase I, chloroplast precursor, putative, expressed  no original description 	T
27.3.6	RNA.regulation of transcription.bHLH,Basic Helix-Loop-Helix family	MDP0000799392	 weakly similar to ( 120) AT1G26945 | Symbols: KDR | KDR (KIDARI); transcription regulator | chr1:9351571-9352474 FORWARDvery weakly similar to (96.7) loc_os10g26460 12010.m05586 protein DNA binding protein, putative no original description 	T
17.1.1	hormone metabolism.abscisic acid.synthesis-degradation	MDP0000124880	 moderately similar to ( 231) AT5G42560 | Symbols:  | abscisic acid-responsive HVA22 family protein | chr5:17015703-17016969 FORWARDweakly similar to ( 199) loc_os03g14040 12003.m101268 protein expressed protein  no original description 	T
35.1	not assigned.no ontology	MDP0000478556	 moderately similar to ( 320) AT3G23840 | Symbols:  | transferase family protein | chr3:8611177-8612559 FORWARDvery weakly similar to (62.8) HCBT1_DIACA Anthranilate N-benzoyltransferase protein 1 (EC 2.3.1.144) (Anthranilate N-hydroxycinnamoyl/benzoyltransferase 1) - Dianthus caryophyllus (Carnation) (Clove pink)moderately similar to ( 271) loc_os04g52164 12004.m10111 protein transferase, putative, expressed    no original description 	T
17.2.3	hormone metabolism.auxin.induced-regulated-responsive-activated	MDP0000170872	 moderately similar to ( 381) AT5G43830 | Symbols:  | unknown protein | chr5:17622593-17624239 REVERSEweakly similar to ( 127) TSJT1_TOBAC Stem-specific protein TSJT1 - Nicotiana tabacum (Common tobacco)moderately similar to ( 343) loc_os03g53270 12003.m35408 protein stem-specific protein TSJT1, putative, expressed  no original description 	T
30.2.3	signalling.receptor kinases.leucine rich repeat III	MDP0000271132	 highly similar to ( 542) AT5G58300 | Symbols:  | leucine-rich repeat transmembrane protein kinase, putative | chr5:23572821-23574871 FORWARDweakly similar to ( 167) PSKR_DAUCA Phytosulfokine receptor precursor (EC 2.7.11.1) (Phytosulfokine LRR receptor kinase) - Daucus carota (Carrot)highly similar to ( 517) loc_os01g60330 12001.m12161 protein ATP binding protein, putative, expressed   no original description 	T
30.2.3	signalling.receptor kinases.leucine rich repeat III	MDP0000937454	 highly similar to ( 830) AT3G51740 | Symbols: IMK2 | IMK2 (INFLORESCENCE MERISTEM RECEPTOR-LIKE KINASE 2); ATP binding / kinase/ protein kinase/ protein serine/threonine kinase | chr3:19189248-19191842 FORWARDweakly similar to ( 194) PSKR_DAUCA Phytosulfokine receptor precursor (EC 2.7.11.1) (Phytosulfokine LRR receptor kinase) - Daucus carota (Carrot)moderately similar to ( 469) loc_os03g18630 12003.m07284 protein receptor-like kinase RHG1, putative, expressed TrbL  no original description 	T
35.2	not assigned.unknown	MDP0000613143	 very weakly similar to ( 100) AT5G52220 | Symbols:  | FUNCTIONS IN: molecular_function unknown; INVOLVED IN: biological_process unknown; LOCATED IN: cellular_component unknown; EXPRESSED IN: 18 plant structures; EXPRESSED DURING: 13 growth stages; CONTAINS InterPro DOMAIN/s: Chromosome transmission fidelity protein 8 (InterPro:IPR018607); Has 85 Blast hits to 85 proteins in 45 species: Archae - 0; Bacteria - 0; Metazoa - 54; Fungi - 13; Plants - 11; Viruses - 0; Other Eukaryotes - 7 (source: NCBI BLink). | chr5:21207097-21207678 REVERSEvery weakly similar to (77.0) loc_os09g12780 12009.m04541 protein expressed protein no original description 	T
29.2.1.2.2.13	protein.synthesis.ribosomal protein.eukaryotic.60S subunit.L13	MDP0000234738	 moderately similar to ( 293) AT3G49010 | Symbols: ATBBC1, BBC1, RSU2 | ATBBC1 (ARABIDOPSIS THALIANA BREAST BASIC CONSERVED 1); structural constituent of ribosome | chr3:18166971-18168047 REVERSEmoderately similar to ( 287) RL132_BRANA 60S ribosomal protein L13-2 (Cold-induced protein C24B) - Brassica napus (Rape)moderately similar to ( 290) loc_os03g37970 12003.m34891 protein 60S ribosomal protein L13-2, putative, expressed  no original description 	T
33.99	development.unspecified	MDP0000180439	 nearly identical (1320) AT4G04940 | Symbols:  | transducin family protein / WD-40 repeat family protein | chr4:2511212-2517052 REVERSEvery weakly similar to (68.2) GBLP_BRANA Guanine nucleotide-binding protein subunit beta-like protein - Brassica napus (Rape)nearly identical (1080) loc_os06g43690 12006.m08899 protein WD repeat-containing protein 36, putative, expressed  no original description 	T
30.5	signalling.G-proteins	MDP0000180439	 nearly identical (1320) AT4G04940 | Symbols:  | transducin family protein / WD-40 repeat family protein | chr4:2511212-2517052 REVERSEvery weakly similar to (68.2) GBLP_BRANA Guanine nucleotide-binding protein subunit beta-like protein - Brassica napus (Rape)nearly identical (1080) loc_os06g43690 12006.m08899 protein WD repeat-containing protein 36, putative, expressed  no original description 	T
29.3.3	protein.targeting.chloroplast	MDP0000094843	 highly similar to ( 757) AT3G17970 | Symbols: atToc64-III | atToc64-III (Arabidopsis thaliana translocon at the outer membrane of chloroplasts 64-III); binding / carbon-nitrogen ligase, with glutamine as amido-N-donor | chr3:6148030-6151794 FORWARDvery weakly similar to (72.8) STIP_SOYBN Heat shock protein STI (Stress-inducible protein) (GmSTI) - Glycine max (Soybean)highly similar to ( 615) loc_os03g50010 12003.m35387 protein toc64, putative, expressed  no original description 	T
35.2	not assigned.unknown	MDP0000886459	 very weakly similar to (51.6) AT2G23440 | Symbols:  | unknown protein | chr2:9979482-9979730 FORWARD no original description 	T
10.5.1.1	cell wall.cell wall proteins.AGPs.AGP	MDP0000257330	 highly similar to ( 947) AT5G35700 | Symbols: FIM2 | FIM2 (FIMBRIN-LIKE PROTEIN 2); actin binding | chr5:13872833-13876432 REVERSEhighly similar to ( 847) loc_os02g48740 12002.m09908 protein fimbrin 1, putative, expressed   no original description 	T
35.1	not assigned.no ontology	MDP0000178426	 weakly similar to ( 185) AT2G17120 | Symbols: LYM2 | LYM2 (LYSM DOMAIN GPI-ANCHORED PROTEIN 2 PRECURSOR) | chr2:7459156-7460648 FORWARDweakly similar to ( 199) loc_os09g37600 12009.m06707 protein erwinia induced protein 1, putative, expressed no original description 	T
7.1.3	OPP.oxidative PP.6-phosphogluconate dehydrogenase	MDP0000149834	 moderately similar to ( 425) AT3G25530 | Symbols: GHBDH, ATGHBDH, GLYR1, GR1 | GLYR1 (GLYOXYLATE REDUCTASE 1); 3-hydroxybutyrate dehydrogenase/ phosphogluconate dehydrogenase (decarboxylating) | chr3:9271949-9273514 REVERSEmoderately similar to ( 427) loc_os02g35500 12002.m08638 protein 2-hydroxy-3-oxopropionate reductase, putative, expressed  no original description 	T
30.11	signalling.light	MDP0000240375	 moderately similar to ( 351) AT4G38180 | Symbols: FRS5 | FRS5 (FAR1-related sequence 5); zinc ion binding | chr4:17906702-17909404 REVERSEmoderately similar to ( 477) loc_os03g08370 12003.m06345 protein transposon protein, putative, unclassified, expressed  no original description 	T
16.2.1.10	secondary metabolism.phenylpropanoids.lignin biosynthesis.CAD	MDP0000250546	 highly similar to ( 596) AT5G51970 | Symbols:  | sorbitol dehydrogenase, putative / L-iditol 2-dehydrogenase, putative | chr5:21111820-21113284 FORWARDvery weakly similar to (97.1) ADHX_ORYSA Alcohol dehydrogenase class 3 (EC 1.1.1.1) (Alcohol dehydrogenase class III) (S-(hydroxymethyl)glutathione dehydrogenase) (EC 1.1.1.284) (Glutathione-dependent formaldehyde dehydrogenase) (FDH) (FALDH) (GSH-FDH) - Oryza sativa (Rice)highly similar to ( 556) loc_os08g43190 12008.m08293 protein sorbitol dehydrogenase, putative, expressed  no original description 	T
23.4.99	nucleotide metabolism.phosphotransfer and pyrophosphatases.misc	MDP0000281233	 moderately similar to ( 367) AT1G19580 | Symbols: GAMMA CA1 | GAMMA CA1 (GAMMA CARBONIC ANHYDRASE 1); carbonate dehydratase | chr1:6774937-6777092 FORWARDmoderately similar to ( 325) IPYR_ORYSA Soluble inorganic pyrophosphatase (EC 3.6.1.1) (Pyrophosphate phospho-hydrolase) (PPase) - Oryza sativa (Rice)moderately similar to ( 335) loc_os12g07220 12012.m04711 protein protein yrdA, putative, expressed no original description 	T
21.1.1	redox.thioredoxin.PDIL	MDP0000625137	 highly similar to ( 699) AT5G60640 | Symbols: ATPDIL1-4 | ATPDIL1-4 (PDI-LIKE 1-4); protein disulfide isomerase | chr5:24371416-24373993 REVERSEmoderately similar to ( 241) PDI_WHEAT Protein disulfide-isomerase precursor (EC 5.3.4.1) (PDI) - Triticum aestivum (Wheat)highly similar to ( 626) loc_os02g01010 12002.m05451 protein OsPDIL1-4 - Oryza sativa protein disulfide isomerase, expressed   no original description 	T
35.2	not assigned.unknown	MDP0000308779	 moderately similar to ( 491) AT3G54260 | Symbols:  | unknown protein | chr3:20085097-20086745 REVERSEmoderately similar to ( 285) loc_os03g19470 12003.m07363 protein expressed protein  no original description 	T
35.1	not assigned.no ontology	MDP0000138538	 very weakly similar to (87.0) AT1G01490 | Symbols:  | heavy-metal-associated domain-containing protein | chr1:180401-182066 REVERSEweakly similar to ( 102) loc_os04g57200 12004.m10605 protein metal ion binding protein, putative, expressed no original description 	T
16.8.4.1	secondary metabolism.flavonoids.flavonols.flavonol synthase (FLS)	MDP0000294667	 weakly similar to ( 185) AT5G08640 | Symbols: FLS | FLS (FLAVONOL SYNTHASE); flavonol synthase | chr5:2804009-2805175 FORWARDmoderately similar to ( 295) FLS_MALDO Flavonol synthase/flavanone 3-hydroxylase (EC 1.14.11.23) (EC 1.14.11.9) (FLS) - Malus domestica (Apple) (Malus sylvestris)weakly similar to ( 140) loc_os02g52840 12002.m10314 protein flavonol synthase/flavanone 3-hydroxylase, putative, expressed  no original description 	T
35.1	not assigned.no ontology	MDP0000199009	 weakly similar to ( 189) AT2G43120 | Symbols:  | pirin, putative | chr2:17927339-17928871 FORWARDmoderately similar to ( 209) loc_os03g62790 12003.m11157 protein pirin-like protein, putative, expressed no original description 	T
27.3.2	RNA.regulation of transcription.Alfin-like	MDP0000161840	 moderately similar to ( 334) AT3G11200 | Symbols: AL2 | AL2 (ALFIN-LIKE 2); DNA binding / methylated histone residue binding | chr3:3508387-3510195 REVERSEmoderately similar to ( 299) loc_os07g12910 12007.m079643 protein PHD finger protein, putative, expressed  no original description 	T
29.3.2	protein.targeting.mitochondria	MDP0000194107	 weakly similar to ( 166) AT2G28900 | Symbols: OEP16, ATOEP16-L, ATOEP16-1 | ATOEP16-1 (OUTER PLASTID ENVELOPE PROTEIN 16-1); P-P-bond-hydrolysis-driven protein transmembrane transporter/ protein transporter | chr2:12414423-12415459 REVERSEweakly similar to ( 198) loc_os05g02060 12005.m04742 protein amino acid selective channel protein, putative, expressed  no original description 	T
27.3.99	RNA.regulation of transcription.unclassified	MDP0000211757	 moderately similar to ( 297) AT5G07900 | Symbols:  | mitochondrial transcription termination factor family protein / mTERF family protein | chr5:2520188-2521405 FORWARDweakly similar to ( 175) loc_os05g33460 12005.m083717 protein mTERF family protein, expressed  no original description 	T
29.6	protein.folding	MDP0000684830	 highly similar to ( 939) AT1G24510 | Symbols:  | T-complex protein 1 epsilon subunit, putative / TCP-1-epsilon, putative / chaperonin, putative | chr1:8685504-8687660 REVERSEhighly similar to ( 904) TCPE2_AVESA T-complex protein 1 subunit epsilon (TCP-1-epsilon) (CCT-epsilon) (TCP-K36) - Avena sativa (Oat)highly similar to ( 952) loc_os06g36700 12006.m08205 protein T-complex protein 1 subunit epsilon, putative, expressed  no original description 	T
3.5	minor CHO metabolism.others	MDP0000755275	 moderately similar to ( 495) AT3G17940 | Symbols:  | aldose 1-epimerase family protein | chr3:6143707-6145244 REVERSEmoderately similar to ( 457) loc_os10g06720 12010.m21832 protein aldose 1-epimerase, putative, expressed  no original description 	T
27.3.50	RNA.regulation of transcription.General Transcription	MDP0000132483	 moderately similar to ( 262) AT1G73230 | Symbols:  | nascent polypeptide-associated complex (NAC) domain-containing protein | chr1:27540506-27541364 REVERSEmoderately similar to ( 250) loc_os10g34180 12010.m21923 protein transcription factor BTF3, putative, expressed no original description 	T
27.3.67	RNA.regulation of transcription.putative transcription regulator	MDP0000132483	 moderately similar to ( 262) AT1G73230 | Symbols:  | nascent polypeptide-associated complex (NAC) domain-containing protein | chr1:27540506-27541364 REVERSEmoderately similar to ( 250) loc_os10g34180 12010.m21923 protein transcription factor BTF3, putative, expressed no original description 	T
10.5.1.1	cell wall.cell wall proteins.AGPs.AGP	MDP0000126400	 very weakly similar to (68.9) AT2G46330 | Symbols: AGP16, ATAGP16 | AGP16 (ARABINOGALACTAN PROTEIN 16) | chr2:19018730-19019108 REVERSEvery weakly similar to (65.1) loc_os05g12580 12005.m05721 protein AGP20, putative no original description 	T
11.1.5	lipid metabolism.FA synthesis and FA elongation.beta hydroxyacyl ACP dehydratase	MDP0000322710	 weakly similar to ( 158) AT2G22230 | Symbols:  | beta-hydroxyacyl-ACP dehydratase, putative | chr2:9450042-9451427 FORWARDweakly similar to ( 135) loc_os05g36000 12005.m27841 protein beta-hydroxyacyl-ACP dehydratase, putative, expressed no original description 	T
1.1.2.2	PS.lightreaction.photosystem I.PSI polypeptide subunits	MDP0000128791	 weakly similar to ( 107) AT4G28750 | Symbols: PSAE-1 | PSAE-1 (PSA E1 KNOCKOUT); catalytic | chr4:14202951-14203888 REVERSEweakly similar to ( 120) PSAEB_NICSY Photosystem I reaction center subunit IV B, chloroplast precursor (PSI-E B) [Contains: Photosystem I reaction center subunit IV B isoform 2] - Nicotiana sylvestris (Wood tobacco)weakly similar to ( 105) loc_os07g25430 12007.m29150 protein photosystem I reaction center subunit IV A, chloroplast precursor, putative, expressed  no original description 	T
34.16	transport.ABC transporters and multidrug resistance systems	MDP0000158422	 highly similar to ( 917) AT3G59140 | Symbols: ATMRP14 | ATMRP14; ATPase, coupled to transmembrane movement of substances | chr3:21863519-21868701 REVERSEvery weakly similar to ( 100) MDR_ORYSA Putative multidrug resistance protein (P-glycoprotein) - Oryza sativa (Rice)highly similar to ( 861) loc_os06g06440 12006.m05369 protein multidrug resistance-associated protein 14, putative, expressed   no original description 	T
35.2	not assigned.unknown	MDP0000319760	 nearly identical (1165) AT5G16210 | Symbols:  | HEAT repeat-containing protein | chr5:5290999-5297779 REVERSEnearly identical (1069) loc_os01g33060 12001.m150606 protein HEAT repeat family protein, expressed  no original description 	T
35.2	not assigned.unknown	MDP0000278527	  no original description 	T
35.2	not assigned.unknown	MDP0000146856	 moderately similar to ( 332) AT1G75180 | Symbols:  | unknown protein | chr1:28216150-28217911 REVERSEmoderately similar to ( 296) loc_os03g56420 12003.m35584 protein expressed protein  no original description 	T
26.2	misc.UDP glucosyl and glucoronyl transferases	MDP0000186772	 highly similar to ( 776) AT1G74380 | Symbols: XXT5 | XXT5 (XYLOGLUCAN XYLOSYLTRANSFERASE 5); transferase/ transferase, transferring glycosyl groups / xyloglucan 6-xylosyltransferase | chr1:27959848-27961221 FORWARDhighly similar to ( 641) loc_os02g32750 12002.m100223 protein glycosyltransferase 5, putative, expressed  Sporozoite_P67  no original description 	T
13.1.1.3.1	amino acid metabolism.synthesis.central amino acid metabolism.alanine.alanine aminotransferase	MDP0000235318	 highly similar to ( 879) AT1G70580 | Symbols: AOAT2, GGT2 | AOAT2 (ALANINE-2-OXOGLUTARATE AMINOTRANSFERASE 2); L-alanine:2-oxoglutarate aminotransferase/ glycine:2-oxoglutarate aminotransferase | chr1:26613222-26615845 FORWARDmoderately similar to ( 483) ALA2_HORVU Alanine aminotransferase 2 (EC 2.6.1.2) (GPT) (Glutamic--pyruvic transaminase 2) (Glutamic--alanine transaminase 2) (ALAAT-2) - Hordeum vulgare (Barley)highly similar to ( 864) loc_os07g01760 12007.m29036 protein alanine aminotransferase 2, putative, expressed  no original description 	T
35.1	not assigned.no ontology	MDP0000201500	 highly similar to ( 808) AT4G12650 | Symbols:  | LOCATED IN: integral to membrane, Golgi apparatus, plasma membrane, vacuole; EXPRESSED IN: 25 plant structures; EXPRESSED DURING: 13 growth stages; CONTAINS InterPro DOMAIN/s: Nonaspanin (TM9SF) (InterPro:IPR004240); BEST Arabidopsis thaliana protein match is: unknown protein (TAIR:AT5G35160.1); Has 983 Blast hits to 980 proteins in 161 species: Archae - 0; Bacteria - 0; Metazoa - 434; Fungi - 144; Plants - 228; Viruses - 0; Other Eukaryotes - 177 (source: NCBI BLink). | chr4:7468207-7470165 REVERSEhighly similar to ( 784) loc_os08g38820 12008.m080200 protein transmembrane 9 superfamily protein member 2 precursor, putative, expressed  no original description 	T
30.3	signalling.calcium	MDP0000735902	 moderately similar to ( 379) AT1G74690 | Symbols: IQD31 | IQD31 (IQ-domain 31); calmodulin binding | chr1:28061498-28063924 REVERSEmoderately similar to ( 319) loc_os05g45930 12005.m083790 protein calmodulin binding protein, putative, expressed  no original description 	T
10.8.1	cell wall.pectin*esterases.PME	MDP0000222620	 moderately similar to ( 431) AT1G53830 | Symbols: ATPME2 | ATPME2; pectinesterase | chr1:20098562-20100745 FORWARDmoderately similar to ( 488) PME3_CITSI Pectinesterase-3 precursor (EC 3.1.1.11) (Pectin methylesterase 3) (PE 3) - Citrus sinensis (Sweet orange)moderately similar to ( 214) loc_os01g21034 12001.m42984 protein pectinesterase-2 precursor, putative, expressed  no original description 	T
2.2.1.4	major CHO metabolism.degradation.sucrose.hexokinase	MDP0000251415	 highly similar to ( 590) AT1G47840 | Symbols: HXK3 | HXK3 (HEXOKINASE 3); ATP binding / fructokinase/ glucokinase/ hexokinase | chr1:17616243-17618859 REVERSEhighly similar to ( 723) HXK2_TOBAC Hexokinase-2, chloroplast precursor (EC 2.7.1.1) (NtHxK2) - Nicotiana tabacum (Common tobacco)highly similar to ( 654) loc_os07g09890 12007.m05444 protein hexokinase-1, putative, expressed  no original description 	T
29.2.2.50	protein.synthesis.ribosome biogenesis.BRIX	MDP0000310109	 moderately similar to ( 446) AT4G01560 | Symbols: MEE49 | MEE49 (maternal effect embryo arrest 49) | chr4:677266-679164 REVERSEmoderately similar to ( 470) loc_os06g04600 12006.m31831 protein ribosome production factor 1, putative, expressed  no original description 	T
29.2.1.1.1.2.86	protein.synthesis.ribosomal protein.prokaryotic.chloroplast.50S subunit.PSRP6	MDP0000851946	 weakly similar to ( 103) AT5G17870 | Symbols: PSRP6 | PSRP6 (PLASTID-SPECIFIC 50S RIBOSOMAL PROTEIN 6) | chr5:5907817-5908137 FORWARDvery weakly similar to (92.8) PSRP6_SPIOL Plastid-specific 50S ribosomal protein 6, chloroplast precursor (PSRP-6) (CL25) - Spinacia oleracea (Spinach)very weakly similar to (85.1) loc_os11g34940 12011.m07348 protein plastid-specific ribosomal protein 6 precursor, putative, expressed no original description 	T
8.2.9	TCA / org transformation.other organic acid transformatons.cyt MDH	MDP0000174740	 highly similar to ( 580) AT1G04410 | Symbols:  | malate dehydrogenase, cytosolic, putative | chr1:1189418-1191267 REVERSEhighly similar to ( 593) MDHC_MAIZE Malate dehydrogenase, cytoplasmic (EC 1.1.1.37) - Zea mays (Maize)highly similar to ( 585) loc_os10g33800 12010.m21921 protein malate dehydrogenase, cytoplasmic, putative, expressed   no original description 	T
33.99	development.unspecified	MDP0000899294	 nearly identical (1338) AT1G15440 | Symbols:  | transducin family protein / WD-40 repeat family protein | chr1:5306159-5309460 REVERSEvery weakly similar to (95.5) PF20_CHLRE Flagellar WD repeat protein PF20 - Chlamydomonas reinhardtiinearly identical (1311) loc_os05g44320 12005.m083779 protein periodic tryptophan protein 2, putative, expressed    TrbL PRK08026  no original description 	T
1.2.5	PS.photorespiration.serine hydroxymethyltransferase	MDP0000174076	 highly similar to ( 784) AT5G26780 | Symbols: SHM2 | SHM2 (SERINE HYDROXYMETHYLTRANSFERASE 2); catalytic/ glycine hydroxymethyltransferase/ pyridoxal phosphate binding | chr5:9418299-9421725 FORWARDhighly similar to ( 795) GLYM_PEA Serine hydroxymethyltransferase, mitochondrial precursor (EC 2.1.2.1) (Serine methylase) (Glycine hydroxymethyltransferase) (SHMT) - Pisum sativum (Garden pea)highly similar to ( 767) loc_os03g52840 12003.m101129 protein serine hydroxymethyltransferase, mitochondrial precursor, putative, expressed no original description 	T
27.3.12	RNA.regulation of transcription.C3H zinc finger family	MDP0000120315	 weakly similar to ( 199) AT3G12130 | Symbols:  | KH domain-containing protein / zinc finger (CCCH type) family protein | chr3:3864486-3866406 REVERSEmoderately similar to ( 288) loc_os02g10080 12002.m33595 protein zinc finger C-x8-C-x5-C-x3-H type family protein, expressed no original description 	T
28.1	DNA.synthesis/chromatin structure	MDP0000141116	 highly similar to ( 614) AT1G67320 | Symbols:  | DNA primase, large subunit family | chr1:25205520-25208966 REVERSEhighly similar to ( 635) loc_os07g22400 12007.m079652 protein DNA primase large subunit, putative, expressed  no original description 	T
27.2	RNA.transcription	MDP0000141470	 moderately similar to ( 237) AT1G17440 | Symbols: EER4, TAF12B | EER4 (ENHANCED ETHYLENE RESPONSE 4); DNA binding / transcription initiation factor | chr1:5984407-5987663 REVERSEweakly similar to ( 162) loc_os01g62820 12001.m43273 protein TATA binding protein associated factor, putative, expressed   no original description 	T
29.7.8	protein.glycosylation.alpha-1,6-mannosyl-glycoprotein-beta-1,2-N-acetylglucosaminyltransferase(GnTII) 	MDP0000230423	 highly similar to ( 526) AT2G05320 | Symbols:  | beta-1,2-N-acetylglucosaminyltransferase II | chr2:1936016-1937308 FORWARDhighly similar to ( 525) loc_os02g55470 12002.m10573 protein alpha-1,6-mannosyl-glycoprotein 2-beta-N-acetylglucosaminyltransferase, putative, expressed no original description 	T
35.2	not assigned.unknown	MDP0000301927	 weakly similar to ( 106) AT5G20900 | Symbols: JAZ12, TIFY3B | JAZ12 (JASMONATE-ZIM-DOMAIN PROTEIN 12) | chr5:7090883-7092201 FORWARDweakly similar to ( 112) loc_os04g55920 12004.m35599 protein ZIM motif family protein, expressed  no original description 	T
35.2	not assigned.unknown	MDP0000126801	   no original description 	T
26.24	misc.GCN5-related N-acetyltransferase	MDP0000243609	 weakly similar to ( 185) AT5G11340 | Symbols:  | GCN5-related N-acetyltransferase (GNAT) family protein | chr5:3619226-3621068 FORWARDweakly similar to ( 190) loc_os01g42470 12001.m150648 protein pre-mRNA-splicing factor cwc24, putative, expressed  no original description 	T
26.4.1	misc.beta 1,3 glucan hydrolases.glucan endo-1,3-beta-glucosidase	MDP0000273111	 highly similar to ( 842) AT5G42740 | Symbols:  | glucose-6-phosphate isomerase, cytosolic (PGIC) | chr5:17136080-17140622 FORWARDhighly similar to ( 849) G6PI_SPIOL Glucose-6-phosphate isomerase, cytosolic (EC 5.3.1.9) (GPI) (Phosphoglucose isomerase) (PGI) (Phosphohexose isomerase) (PHI) - Spinacia oleracea (Spinach)highly similar to ( 796) loc_os06g14510 12006.m091669 protein glucose-6-phosphate isomerase, cytosolic B, putative, expressed   no original description 	T
34.3	transport.amino acids	MDP0000293465	 highly similar to ( 538) AT3G30390 | Symbols:  | amino acid transporter family protein | chr3:11977112-11978827 REVERSEmoderately similar to ( 479) loc_os06g42720 12006.m08803 protein amino acid transporter-like protein, putative, expressed  no original description 	T
20.1	stress.biotic	MDP0000663084	 moderately similar to ( 368) AT1G33970 | Symbols:  | avirulence-responsive protein, putative / avirulence induced gene protein, putative / AIG protein, putative | chr1:12349614-12350960 REVERSEmoderately similar to ( 286) loc_os02g35130 12002.m100235 protein protein AIG1, putative, expressed no original description 	T
29.2.1.2.1.14	protein.synthesis.ribosomal protein.eukaryotic.40S subunit.S14	MDP0000160747	 moderately similar to ( 250) AT4G18270 | Symbols: ATTRANS11, TRANS11 | ATTRANS11; P-P-bond-hydrolysis-driven protein transmembrane transporter/ catalytic | chr4:10099364-10101897 REVERSEvery weakly similar to (96.7) RS14_LUPLU 40S ribosomal protein S14 - Lupinus luteus (European yellow lupin)weakly similar to ( 157) loc_os01g55170 12001.m11677 protein RNA binding protein, putative, expressed   no original description 	T
34.16	transport.ABC transporters and multidrug resistance systems	MDP0000316000	 highly similar to ( 932) AT2G01320 | Symbols:  | ABC transporter family protein | chr2:154669-158063 REVERSEmoderately similar to ( 238) PDR3_ORYSA Pleiotropic drug resistance protein 3 - Oryza sativa (Rice)highly similar to ( 855) loc_os01g03144 12001.m06953 protein ABC-2 type transporter family protein, expressed   no original description 	T
23.3.3	nucleotide metabolism.salvage.NUDIX hydrolases	MDP0000289822	 moderately similar to ( 229) AT2G01670 | Symbols: atnudt17 | atnudt17 (Arabidopsis thaliana Nudix hydrolase homolog 17); hydrolase | chr2:296889-297818 REVERSEweakly similar to ( 183) loc_os02g50130 12002.m10047 protein nudix hydrolase 4, putative, expressed  no original description 	T
29.5.5	protein.degradation.serine protease	MDP0000654592	 highly similar to ( 609) AT3G27925 | Symbols: DEGP1, Deg1 | DEGP1 (DegP protease 1); serine-type endopeptidase/ serine-type peptidase | chr3:10366659-10368864 REVERSEhighly similar to ( 601) loc_os05g49380 12005.m083815 protein protease Do-like 1, chloroplast precursor, putative, expressed   no original description 	T
34.1	transport.p- and v-ATPases	MDP0000290422	 nearly identical (1332) AT1G17260 | Symbols: AHA10 | AHA10 (Autoinhibited H(+)-ATPase isoform 10); ATPase/ ATPase, coupled to transmembrane movement of ions, phosphorylative mechanism / cation-transporting ATPase | chr1:5904058-5908898 FORWARDnearly identical (1317) PMA4_NICPL Plasma membrane ATPase 4 (EC 3.6.3.6) (Proton pump 4) - Nicotiana plumbaginifolia (Leadwort-leaved tobacco)nearly identical (1306) loc_os04g56160 12004.m101441 protein plasma membrane ATPase, putative, expressed  no original description 	T
16.2.1.1	secondary metabolism.phenylpropanoids.lignin biosynthesis.PAL	MDP0000139075	 highly similar to ( 769) AT2G37040 | Symbols: pal1, ATPAL1 | pal1 (Phe ammonia lyase 1); phenylalanine ammonia-lyase | chr2:15557602-15560237 REVERSEhighly similar to ( 784) PAL3_PETCR Phenylalanine ammonia-lyase 3 (EC 4.3.1.5) - Petroselinum crispum (Parsley) (Petroselinum hortense)highly similar to ( 724) loc_os02g41650 12002.m33781 protein phenylalanine ammonia-lyase, putative, expressed   no original description 	T
20.2.4	stress.abiotic.touch/wounding	MDP0000764419	 moderately similar to ( 214) AT1G75380 | Symbols:  | wound-responsive protein-related | chr1:28281789-28283631 REVERSEweakly similar to ( 163) loc_os08g26870 12008.m080140 protein wound responsive protein, putative, expressed  no original description 	T
35.1.5	not assigned.no ontology.pentatricopeptide (PPR) repeat-containing protein	MDP0000317926	 highly similar to ( 681) AT1G52630 | Symbols:  | unknown protein | chr1:19606470-19607848 REVERSEweakly similar to ( 113) RF1_ORYSA Rf1 protein, mitochondrial precursor (PPR protein) (Fertility restorer) (Restorer for CMS) - Oryza sativa (Rice)highly similar to ( 594) loc_os08g42550 12008.m080223 protein AP2 domain containing protein, expressed  no original description 	T
10.2.1	cell wall.cellulose synthesis.cellulose synthase	MDP0000322053	 nearly identical (1235) AT4G39350 | Symbols: CESA2, ATH-A, ATCESA2 | CESA2 (CELLULOSE SYNTHASE A2); cellulose synthase/ transferase, transferring glycosyl groups | chr4:18297078-18301890 FORWARDnearly identical (1163) loc_os07g24190 12007.m28970 protein CESA3 - cellulose synthase, expressed   no original description 	T
35.1	not assigned.no ontology	MDP0000265028	 moderately similar to ( 352) AT2G38740 | Symbols:  | haloacid dehalogenase-like hydrolase family protein | chr2:16194639-16195995 REVERSEmoderately similar to ( 305) loc_os10g41930 12010.m065348 protein catalytic/ hydrolase, putative, expressed  no original description 	T
27.3	RNA.regulation of transcription	MDP0000246923	 moderately similar to ( 419) AT1G21600 | Symbols: PTAC6 | PTAC6 (PLASTID TRANSCRIPTIONALLY ACTIVE6) | chr1:7571514-7573782 REVERSEmoderately similar to ( 350) loc_os11g01890 12011.m079927 protein expressed protein  no original description 	T
28.1.1.4	DNA.synthesis/chromatin structure.retrotransposon/transposase.hat-like transposase	MDP0000394762	 highly similar to ( 820) AT3G42170 | Symbols:  | DNA binding | chr3:14321838-14323928 FORWARDmoderately similar to ( 259) TRA1_MAIZE Putative AC transposase (ORFA) - Zea mays (Maize)highly similar to ( 644) loc_os03g52310 12003.m10208 protein transposon protein, putative, unclassified, expressed  no original description 	T
27.3.5	RNA.regulation of transcription.ARR	MDP0000585643	 weakly similar to ( 155) AT3G57040 | Symbols: ARR9, ATRR4 | ARR9 (RESPONSE REGULATOR 9); transcription regulator/ two-component response regulator | chr3:21110059-21111228 FORWARDvery weakly similar to (66.2) PRR37_ORYSA Two-component response regulator-like PRR37 (Pseudo-response regulator 37) (OsPRR37) - Oryza sativa (Rice)weakly similar to ( 165) loc_os11g04720 12011.m04670 protein OsRR9 - Rice type-A response regulator, expressed no original description 	T
20.2.3	stress.abiotic.drought/salt	MDP0000589608	 nearly identical (1220) AT1G34130 | Symbols: STT3B | STT3B (staurosporin and temperature sensitive 3-like b); oligosaccharyl transferase | chr1:12430000-12432985 FORWARDnearly identical (1173) loc_os04g57890 12004.m10673 protein oligosaccharyl transferase STT3 subunit, putative, expressed   no original description 	T
26.4	misc.beta 1,3 glucan hydrolases	MDP0000299794	 weakly similar to ( 159) AT4G05430 | Symbols:  | FUNCTIONS IN: molecular_function unknown; INVOLVED IN: biological_process unknown; LOCATED IN: cellular_component unknown; CONTAINS InterPro DOMAIN/s: X8 (InterPro:IPR012946); BEST Arabidopsis thaliana protein match is: glucan endo-1,3-beta-glucosidase-related (TAIR:AT1G09460.1); Has 776 Blast hits to 740 proteins in 35 species: Archae - 0; Bacteria - 0; Metazoa - 0; Fungi - 3; Plants - 770; Viruses - 0; Other Eukaryotes - 3 (source: NCBI BLink). | chr4:2753582-2754324 FORWARDweakly similar to ( 142) PSAK_MEDSA Photosystem I reaction center subunit psaK, chloroplast precursor (Photosystem I subunit X) (PSI-K) - Medicago sativa (Alfalfa)weakly similar to ( 135) loc_os07g05480 12007.m079603 protein photosystem I reaction center subunit psaK, chloroplast precursor, putative, expressed  no original description 	T
29.2.1.1.3.2.4	protein.synthesis.ribosomal protein.prokaryotic.unknown organellar.50S subunit.L4	MDP0000847579	 moderately similar to ( 409) AT2G20060 | Symbols:  | ribosomal protein L4 family protein | chr2:8659384-8661227 FORWARDweakly similar to ( 107) RK4_TOBAC 50S ribosomal protein L4, chloroplast precursor (CL4) (R-protein L4) - Nicotiana tabacum (Common tobacco)moderately similar to ( 369) loc_os11g37510 12011.m07596 protein 50S ribosomal protein L4, putative, expressed  no original description 	T
17.5.1	hormone metabolism.ethylene.synthesis-degradation	MDP0000202141	 moderately similar to ( 215) AT1G17020 | Symbols: SRG1, ATSRG1 | SRG1 (SENESCENCE-RELATED GENE 1); oxidoreductase, acting on diphenols and related substances as donors, oxygen as acceptor / oxidoreductase, acting on paired donors, with incorporation or reduction of molecular oxygen, 2-oxoglutarate as one donor, and inc | chr1:5820258-5821741 FORWARDvery weakly similar to (89.0) LDOX_PETHY Leucoanthocyanidin dioxygenase (EC 1.14.11.19) (LDOX) (Leucocyanidin oxygenase) (Leucoanthocyanidin hydroxylase) - Petunia hybrida (Petunia)weakly similar to ( 137) loc_os01g25010 12001.m08967 protein flavonol synthase/flavanone 3-hydroxylase, putative, expressed no original description 	T
16.8.4	secondary metabolism.flavonoids.flavonols	MDP0000202141	 moderately similar to ( 215) AT1G17020 | Symbols: SRG1, ATSRG1 | SRG1 (SENESCENCE-RELATED GENE 1); oxidoreductase, acting on diphenols and related substances as donors, oxygen as acceptor / oxidoreductase, acting on paired donors, with incorporation or reduction of molecular oxygen, 2-oxoglutarate as one donor, and inc | chr1:5820258-5821741 FORWARDvery weakly similar to (89.0) LDOX_PETHY Leucoanthocyanidin dioxygenase (EC 1.14.11.19) (LDOX) (Leucocyanidin oxygenase) (Leucoanthocyanidin hydroxylase) - Petunia hybrida (Petunia)weakly similar to ( 137) loc_os01g25010 12001.m08967 protein flavonol synthase/flavanone 3-hydroxylase, putative, expressed no original description 	T
28.1.3	DNA.synthesis/chromatin structure.histone	MDP0000432128	 moderately similar to ( 228) AT5G65360 | Symbols:  | histone H3 | chr5:26120099-26120509 REVERSEmoderately similar to ( 226) H32_WHEAT Histone H3.2 - Triticum aestivum (Wheat)moderately similar to ( 228) loc_os11g05730 12011.m04769 protein histone H3, putative, expressed  no original description 	T
29.4	protein.postranslational modification	MDP0000152621	 highly similar to ( 675) AT3G04910 | Symbols: WNK1, ZIK4 | WNK1 (WITH NO LYSINE (K) 1); kinase/ protein kinase/ protein serine/threonine kinase | chr3:1356061-1358057 FORWARDvery weakly similar to (97.1) M2K1_ORYSA Mitogen-activated protein kinase kinase 1 (EC 2.7.12.2) (MAP kinase kinase 1) (MAPKK1) (OsMEK1) - Oryza sativa (Rice)highly similar to ( 656) loc_os07g38530 12007.m29232 protein protein kinase domain containing protein, expressed   no original description 	T
35.2	not assigned.unknown	MDP0000881783	 moderately similar to ( 397) AT1G47640 | Symbols:  | unknown protein | chr1:17518409-17519998 REVERSEmoderately similar to ( 388) loc_os03g55730 12003.m35637 protein seven transmembrane domain protein, putative, expressed  no original description 	T
28.2	DNA.repair	MDP0000313170	 highly similar to ( 910) AT4G02070 | Symbols: MSH6, MSH6-1, ATMSH6 | MSH6 (MUTS HOMOLOG 6); damaged DNA binding | chr4:906079-912930 FORWARDweakly similar to ( 191) MSH2_MAIZE DNA mismatch repair protein MSH2 (MUS1) - Zea mays (Maize)highly similar to ( 906) loc_os09g24220 12009.m05578 protein DNA mismatch repair protein MSH6-1, putative, expressed   no original description 	T
30.3	signalling.calcium	MDP0000277016	 moderately similar to ( 431) AT1G74690 | Symbols: IQD31 | IQD31 (IQ-domain 31); calmodulin binding | chr1:28061498-28063924 REVERSEmoderately similar to ( 357) loc_os05g45930 12005.m083790 protein calmodulin binding protein, putative, expressed  no original description 	T
35.2	not assigned.unknown	MDP0000426284	 weakly similar to ( 105) AT4G35905 | Symbols:  | unknown protein | chr4:17007284-17008067 REVERSEvery weakly similar to (94.0) loc_os10g30300 12010.m21898 protein expressed protein no original description 	T
26.4.1	misc.beta 1,3 glucan hydrolases.glucan endo-1,3-beta-glucosidase	MDP0000543964	 moderately similar to ( 476) AT3G55430 | Symbols:  | glycosyl hydrolase family 17 protein / beta-1,3-glucanase, putative | chr3:20549806-20552004 REVERSEmoderately similar to ( 411) E13B_WHEAT Glucan endo-1,3-beta-glucosidase precursor (EC 3.2.1.39) ((1->3)-beta-glucan endohydrolase) ((1->3)-beta-glucanase) (Beta-1,3-endoglucanase) - Triticum aestivum (Wheat)moderately similar to ( 424) loc_os11g47820 12011.m08580 protein glucan endo-1,3-beta-glucosidase precursor, putative, expressed   no original description 	T
30.11	signalling.light	MDP0000210133	 highly similar to ( 850) AT4G18810 | Symbols:  | binding / catalytic/ transcription repressor | chr4:10322622-10325735 REVERSEhighly similar to ( 777) loc_os02g13970 12002.m77732 protein UOS1, putative, expressed MSP1_C  no original description 	T
33.99	development.unspecified	MDP0000428265	 nearly identical (1461) AT1G56070 | Symbols: LOS1 | LOS1; copper ion binding / translation elongation factor/ translation factor, nucleic acid binding | chr1:20968245-20971077 REVERSEnearly identical (1439) EF2_BETVU Elongation factor 2 (EF-2) - Beta vulgaris (Sugar beet)nearly identical (1442) loc_os04g02820 12004.m101449 protein elongation factor 2, putative, expressed   no original description 	T
1.1.5.2	PS.lightreaction.other electron carrier (ox/red).ferredoxin	MDP0000467245	 moderately similar to ( 266) AT1G02180 | Symbols:  | ferredoxin-related | chr1:413619-414505 REVERSEweakly similar to ( 180) loc_os02g58740 12002.m10894 protein expressed protein  no original description 	T
29.5.9	protein.degradation.AAA type	MDP0000420252	 moderately similar to ( 461) AT3G01610 | Symbols: CDC48C, emb1354 | CDC48C; ATP binding / ATPase/ nucleoside-triphosphatase/ nucleotide binding | chr3:231787-235057 FORWARDmoderately similar to ( 260) CDC48_SOYBN Cell division cycle protein 48 homolog (Valosin-containing protein homolog) (VCP) - Glycine max (Soybean)highly similar to ( 528) loc_os06g01980 12006.m04934 protein cell division control protein 48 homolog C, putative, expressed   no original description 	T
35.2	not assigned.unknown	MDP0000184929	 weakly similar to ( 159) AT1G72110 | Symbols:  | unknown protein | chr1:27129253-27131354 REVERSEweakly similar to ( 154) loc_os01g48874 12001.m11075 protein expressed protein       Sporozoite_P67  no original description 	T
34.1.1	transport.p- and v-ATPases.H+-transporting two-sector ATPase	MDP0000186579	 highly similar to ( 681) AT3G28715 | Symbols:  | H+-transporting two-sector ATPase, putative | chr3:10778025-10780350 FORWARDhighly similar to ( 669) VA0D_ORYSA Probable vacuolar ATP synthase subunit d (EC 3.6.3.14) (V-ATPase d subunit) (Vacuolar proton pump subunit d) - Oryza sativa (Rice)highly similar to ( 669) loc_os01g40470 12001.m10318 protein vacuolar ATP synthase subunit d, putative, expressed no original description 	T
30.3	signalling.calcium	MDP0000201907	 highly similar to ( 946) AT4G37640 | Symbols: ACA2 | ACA2 (CALCIUM ATPASE 2); calcium ion transmembrane transporter/ calcium-transporting ATPase/ calmodulin binding | chr4:17683225-17686808 REVERSEhighly similar to ( 936) ACA1_ORYSA Calcium-transporting ATPase 1, plasma membrane-type (EC 3.6.3.8) (Ca(2+)-ATPase isoform 1) (Plastid envelope ATPase 1) - Oryza sativa (Rice)highly similar to ( 941) loc_os03g10640 12003.m06516 protein calcium-transporting ATPase 2, plasma membrane-type, putative, expressed   no original description 	T
10.6.3	cell wall.degradation.pectate lyases and polygalacturonases	MDP0000239958	 highly similar to ( 798) AT1G70370 | Symbols:  | BURP domain-containing protein / polygalacturonase, putative | chr1:26513003-26514998 REVERSEvery weakly similar to (57.0) EA92_VICFA Embryonic abundant protein USP92 precursor - Vicia faba (Broad bean)highly similar to ( 590) loc_os08g29200 12008.m06924 protein polygalacturonase-1 non-catalytic beta subunit precursor, putative, expressed   no original description 	T
34.99	transport.misc	MDP0000176870	 moderately similar to ( 414) AT1G61250 | Symbols: SC3 | SC3 (SECRETORY CARRIER 3); transmembrane transporter | chr1:22586035-22588664 FORWARDmoderately similar to ( 382) loc_os01g57220 12001.m43501 protein SC3 protein, putative, expressed no original description 	T
29.3.99	protein.targeting.unknown	MDP0000453329	 moderately similar to ( 252) AT5G02280 | Symbols:  | synbindin, putative | chr5:469377-470129 FORWARDmoderately similar to ( 236) loc_os07g27150 12007.m079581 protein trafficking protein particle complex subunit 4, putative, expressed  no original description 	T
27.4	RNA.RNA binding	MDP0000614064	 moderately similar to ( 473) AT4G27000 | Symbols: ATRBP45C | ATRBP45C; RNA binding | chr4:13554983-13557763 REVERSEvery weakly similar to (83.6) ROC1_NICPL 30 kDa ribonucleoprotein, chloroplast precursor (CP-RBP30) - Nicotiana plumbaginifolia (Leadwort-leaved tobacco)moderately similar to ( 453) loc_os03g37270 12003.m08836 protein expressed protein  no original description 	T
35.2	not assigned.unknown	MDP0000732599	 moderately similar to ( 318) AT5G14240 | Symbols:  | FUNCTIONS IN: molecular_function unknown; INVOLVED IN: biological_process unknown; LOCATED IN: cellular_component unknown; EXPRESSED IN: 23 plant structures; EXPRESSED DURING: 15 growth stages; CONTAINS InterPro DOMAIN/s: Thioredoxin fold (InterPro:IPR012335), Phosducin (InterPro:IPR001200), Thioredoxin-like fold (InterPro:IPR012336); Has 750 Blast hits to 750 proteins in 282 species: Archae - 0; Bacteria - 2; Metazoa - 486; Fungi - 126; Plants - 51; Viruses - 0; Other Eukaryotes - 85 (source: NCBI BLink). | chr5:4596223-4597568 REVERSEmoderately similar to ( 320) loc_os01g08960 12001.m07521 protein phosducin-like protein 3, putative, expressed  no original description 	T
21.4	redox.glutaredoxins	MDP0000804081	 weakly similar to ( 156) AT2G30540 | Symbols:  | glutaredoxin family protein | chr2:13011259-13011567 REVERSEvery weakly similar to (70.1) GLRX_RICCO Glutaredoxin - Ricinus communis (Castor bean)weakly similar to ( 126) loc_os12g35330 12012.m07326 protein OsGrx_C12 - glutaredoxin subgroup III no original description 	T
29.2.1.2.2.34	protein.synthesis.ribosomal protein.eukaryotic.60S subunit.L34	MDP0000845910	 weakly similar to ( 176) AT1G69620 | Symbols: RPL34 | RPL34 (RIBOSOMAL PROTEIN L34); structural constituent of ribosome | chr1:26189900-26191081 FORWARDweakly similar to ( 181) RL34_PEA 60S ribosomal protein L34 - Pisum sativum (Garden pea)weakly similar to ( 166) loc_os09g24690 12009.m05623 protein 60S ribosomal protein L34, putative, expressed  no original description 	T
26.4.1	misc.beta 1,3 glucan hydrolases.glucan endo-1,3-beta-glucosidase	MDP0000228162	 highly similar to ( 722) AT5G58090 | Symbols:  | glycosyl hydrolase family 17 protein | chr5:23505556-23507193 REVERSEmoderately similar to ( 218) E13B_WHEAT Glucan endo-1,3-beta-glucosidase precursor (EC 3.2.1.39) ((1->3)-beta-glucan endohydrolase) ((1->3)-beta-glucanase) (Beta-1,3-endoglucanase) - Triticum aestivum (Wheat)highly similar to ( 667) loc_os02g04670 12002.m05815 protein glucan endo-1,3-beta-glucosidase 6 precursor, putative, expressed no original description 	T
35.2	not assigned.unknown	MDP0000328793	 moderately similar to ( 220) AT2G38550 | Symbols:  | unknown protein | chr2:16132178-16134229 FORWARDweakly similar to ( 186) loc_os01g60650 12001.m12193 protein non-green plastid inner envelope membrane protein, putative, expressed  no original description 	T
35.2	not assigned.unknown	MDP0000281120	 very weakly similar to (68.9) AT1G06475 | Symbols:  | unknown protein | chr1:1973992-1974273 REVERSE  no original description 	T
29.4	protein.postranslational modification	MDP0000261968	 highly similar to ( 566) AT3G07790 | Symbols:  | DGCR14-related | chr3:2487491-2489017 FORWARDvery weakly similar to (96.3) M2K1_ORYSA Mitogen-activated protein kinase kinase 1 (EC 2.7.12.2) (MAP kinase kinase 1) (MAPKK1) (OsMEK1) - Oryza sativa (Rice)moderately similar to ( 446) loc_os06g30710 12006.m091709 protein DGCR14 protein, putative, expressed   no original description 	T
29.4	protein.postranslational modification	MDP0000291480	 moderately similar to ( 231) AT3G56490 | Symbols:  | zinc-binding protein, putative / protein kinase C inhibitor, putative | chr3:20941532-20943129 FORWARDmoderately similar to ( 213) ZB14_MAIZE 14 kDa zinc-binding protein (Protein kinase C inhibitor) (PKCI) - Zea mays (Maize)moderately similar to ( 231) loc_os01g59750 12001.m12109 protein zinc-binding protein, putative, expressed no original description 	T
17.2.3	hormone metabolism.auxin.induced-regulated-responsive-activated	MDP0000148780	 weakly similar to ( 109) AT4G38840 | Symbols:  | auxin-responsive protein, putative | chr4:18125174-18125473 REVERSEweakly similar to ( 124) AX6B_SOYBN Auxin-induced protein 6B - Glycine max (Soybean)very weakly similar to (80.9) loc_os08g02520 12008.m04396 protein OsSAUR31 - Auxin-responsive SAUR gene family member, expressed no original description 	T
17.2.3	hormone metabolism.auxin.induced-regulated-responsive-activated	MDP0000143958	 weakly similar to ( 192) AT5G19140 | Symbols: ATAILP1, AILP1 | AILP1 | chr5:6423398-6425785 FORWARDvery weakly similar to (57.0) TSJT1_TOBAC Stem-specific protein TSJT1 - Nicotiana tabacum (Common tobacco)weakly similar to ( 179) loc_os11g05050 12011.m28725 protein stem-specific protein TSJT1, putative, expressed  no original description 	T
31.4	cell.vesicle transport	MDP0000202577	 nearly identical (1206) AT1G62020 | Symbols:  | coatomer protein complex, subunit alpha, putative | chr1:22919814-22923728 FORWARDvery weakly similar to (77.4) PF20_CHLRE Flagellar WD repeat protein PF20 - Chlamydomonas reinhardtiinearly identical (1148) loc_os09g04110 12009.m22040 protein coatomer subunit alpha, putative, expressed  no original description 	T
35.2	not assigned.unknown	MDP0000206902	  no original description 	T
20.2.1	stress.abiotic.heat	MDP0000611170	 highly similar to ( 763) AT4G24190 | Symbols: SHD | SHD (SHEPHERD); ATP binding / unfolded protein binding | chr4:12551902-12555851 REVERSEnearly identical (1174) ENPL_CATRO Endoplasmin homolog precursor (GRP94 homolog) - Catharanthus roseus (Rosy periwinkle) (Madagascar periwinkle)highly similar to ( 757) loc_os06g50300 12006.m31934 protein endoplasmin homolog precursor, putative, expressed  no original description 	T
29.2.1.1.1.2.1	protein.synthesis.ribosomal protein.prokaryotic.chloroplast.50S subunit.L1	MDP0000136086	 moderately similar to ( 263) AT2G42710 | Symbols:  | ribosomal protein L1 family protein | chr2:17782352-17784830 FORWARDweakly similar to ( 148) RK1_SPIOL 50S ribosomal protein L1, chloroplast precursor (CL1) - Spinacia oleracea (Spinach)moderately similar to ( 256) loc_os06g35470 12006.m08084 protein 50S ribosomal protein L1, putative, expressed   no original description 	T
27.3.99	RNA.regulation of transcription.unclassified	MDP0000194689	 highly similar to ( 635) AT1G07705 | Symbols:  | transcription regulator | chr1:2382090-2385856 FORWARDhighly similar to ( 563) loc_os02g54120 12002.m100387 protein VIP2, putative, expressed  no original description 	T
20.1	stress.biotic	MDP0000140596	 weakly similar to ( 108) AT2G02100 | Symbols: LCR69, PDF2.2 | LCR69 (LOW-MOLECULAR-WEIGHT CYSTEINE-RICH 69); peptidase inhibitor | chr2:528397-528885 FORWARDweakly similar to ( 107) DEF2_CAPAN Defensin J1-2 precursor - Capsicum annuum (Bell pepper)weakly similar to ( 102) loc_os03g03810 12003.m05913 protein flower-specific gamma-thionin precursor, putative, expressed no original description 	T
35.1	not assigned.no ontology	MDP0000165731	 moderately similar to ( 461) AT3G10970 | Symbols:  | haloacid dehalogenase-like hydrolase family protein | chr3:3433276-3436077 REVERSEmoderately similar to ( 396) loc_os05g07632 12005.m27473 protein catalytic/ hydrolase, putative, expressed   no original description 	T
29.5.3	protein.degradation.cysteine protease	MDP0000141653	 moderately similar to ( 260) AT3G12490 | Symbols: ATCYSB | cysteine protease inhibitor, putative / cystatin, putative | chr3:3960523-3961777 REVERSEweakly similar to ( 107) CYTI_VIGUN Cysteine proteinase inhibitor (Cystatin) - Vigna unguiculata (Cowpea)moderately similar to ( 264) loc_os01g16430 12001.m08239 protein multidomain cystatin, putative, expressed  no original description 	T
26.3	misc.gluco-, galacto- and mannosidases	MDP0000306634	 moderately similar to ( 470) AT2G44480 | Symbols: BGLU17 | BGLU17 (BETA GLUCOSIDASE 17); catalytic/ cation binding / hydrolase, hydrolyzing O-glycosyl compounds | chr2:18360476-18363001 FORWARDmoderately similar to ( 381) MYRA_SINAL Myrosinase MA1 (EC 3.2.1.147) (Sinigrinase) (Thioglucosidase) - Sinapis alba (White mustard) (Brassica hirta)highly similar to ( 571) loc_os04g39880 12004.m35159 protein non-cyanogenic beta-glucosidase precursor, putative, expressed no original description 	T
1.1.5.3	PS.lightreaction.other electron carrier (ox/red).ferredoxin reductase	MDP0000618002	 moderately similar to ( 317) AT5G66190 | Symbols: ATLFNR1 | FNR1 (FERREDOXIN-NADP(+)-OXIDOREDUCTASE 1); NADPH dehydrogenase/ electron transporter, transferring electrons within the cyclic electron transport pathway of photosynthesis/ electron transporter, transferring electrons within the noncyclic electron transp | chr5:26451203-26452616 REVERSEmoderately similar to ( 320) FENR_MESCR Ferredoxin--NADP reductase, chloroplast precursor (EC 1.18.1.2) (FNR) - Mesembryanthemum crystallinum (Common ice plant)moderately similar to ( 306) loc_os06g01850 12006.m04921 protein ferredoxin--NADP reductase, leaf isozyme, chloroplast precursor, putative, expressed no original description 	T
27.3.99	RNA.regulation of transcription.unclassified	MDP0000553473	 moderately similar to ( 286) AT4G17900 | Symbols:  | zinc-binding family protein | chr4:9946813-9947697 FORWARDmoderately similar to ( 289) loc_os02g46610 12002.m09698 protein expressed protein no original description 	T
35.2	not assigned.unknown	MDP0000805832	 weakly similar to ( 108) AT5G08050 | Symbols:  | FUNCTIONS IN: molecular_function unknown; INVOLVED IN: biological_process unknown; LOCATED IN: thylakoid, chloroplast thylakoid membrane, chloroplast; EXPRESSED IN: 21 plant structures; EXPRESSED DURING: 13 growth stages; CONTAINS InterPro DOMAIN/s: Protein of unknown function DUF1118 (InterPro:IPR009500), Uncharacterised conserved protein UCP022207 (InterPro:IPR016801); Has 41 Blast hits to 41 proteins in 10 species: Archae - 0; Bacteria - 0; Metazoa - 0; Fungi - 0; Plants - 40; Viruses - 0; Other Eukaryotes - 1 (source: NCBI BLink). | chr5:2578418-2578964 FORWARDvery weakly similar to (86.7) loc_os12g38640 12012.m073865 protein expressed protein  no original description 	T
19.99	tetrapyrrole synthesis.unspecified	MDP0000708550	 moderately similar to ( 302) AT5G43860 | Symbols: ATCLH2, CLH2 | ATCLH2; chlorophyllase | chr5:17630492-17632184 FORWARDweakly similar to ( 172) CLH1_CITSI Chlorophyllase-1, chloroplast precursor (EC 3.1.1.14) (Chlorophyll-chlorophyllido hydrolase 1) (Chlase 1) - Citrus sinensis (Sweet orange)moderately similar to ( 237) loc_os10g28370 12010.m05717 protein chlorophyllase-2, chloroplast precursor, putative, expressed  no original description 	T
1.2.6	PS.photorespiration.hydroxypyruvate reductase	MDP0000172852	 highly similar to ( 606) AT1G68010 | Symbols: HPR | HPR; glycerate dehydrogenase/ poly(U) binding | chr1:25493418-25495720 FORWARDhighly similar to ( 613) DHGY_CUCSA Glycerate dehydrogenase (EC 1.1.1.29) (NADH-dependent hydroxypyruvate reductase) (HPR) (GDH) - Cucumis sativus (Cucumber)highly similar to ( 588) loc_os02g01150 12002.m33493 protein hydroxypyruvate reductase, putative, expressed  no original description 	T
27.4	RNA.RNA binding	MDP0000329401	 weakly similar to ( 187) AT1G76940 | Symbols:  | RNA recognition motif (RRM)-containing protein | chr1:28902707-28904085 REVERSEmoderately similar to ( 243) loc_os08g43360 12008.m080227 protein nucleic acid binding protein, putative, expressed  no original description 	T
26.7	misc.oxidases - copper, flavone etc	MDP0000185799	 highly similar to ( 827) AT4G25240 | Symbols: SKS1 | SKS1 (SKU5 SIMILAR 1); copper ion binding / oxidoreductase | chr4:12930539-12933563 FORWARDmoderately similar to ( 488) ASO_BRANA L-ascorbate oxidase homolog precursor (EC 1.10.3.3) (Ascorbase) - Brassica napus (Rape)highly similar to ( 795) loc_os06g01490 12006.m04887 protein monocopper oxidase precursor, putative, expressed no original description 	T
26.19	misc.plastocyanin-like	MDP0000296280	 weakly similar to ( 151) AT1G64640 | Symbols:  | plastocyanin-like domain-containing protein | chr1:24022482-24023151 REVERSEvery weakly similar to (62.0) NO16_MEDTR Early nodulin 16 precursor (N-16) - Medicago truncatula (Barrel medic)weakly similar to ( 128) loc_os03g02400 12003.m05782 protein early nodulin-like protein 1 precursor, putative, expressed no original description 	T
35.2	not assigned.unknown	MDP0000163249	 moderately similar to ( 268) AT4G02920 | Symbols:  | unknown protein | chr4:1292816-1294670 FORWARDmoderately similar to ( 208) loc_os05g05140 12005.m05041 protein expressed protein   no original description 	T
11.2.3	lipid metabolism.FA desaturation.omega 3 desaturase	MDP0000382134	 moderately similar to ( 224) AT3G12120 | Symbols: FAD2 | FAD2 (FATTY ACID DESATURASE 2); delta12-fatty acid dehydrogenase/ omega-6 fatty acid desaturase | chr3:3860592-3861743 REVERSEmoderately similar to ( 223) FAD6E_BRAJU Omega-6 fatty acid desaturase, endoplasmic reticulum (EC 1.14.19.-) (Delta(12) desaturase) - Brassica juncea (Leaf mustard) (Indian mustard)moderately similar to ( 216) loc_os02g48560 12002.m100054 protein omega-6 fatty acid desaturase, endoplasmic reticulum isozyme 2, putative, expressed no original description 	T
35.2	not assigned.unknown	MDP0000902602	 moderately similar to ( 484) AT1G29790 | Symbols:  | FUNCTIONS IN: molecular_function unknown; INVOLVED IN: biological_process unknown; LOCATED IN: Golgi apparatus, plasma membrane; EXPRESSED IN: 23 plant structures; EXPRESSED DURING: 12 growth stages; BEST Arabidopsis thaliana protein match is: methyltransferase (TAIR:AT5G40830.2); Has 127 Blast hits to 127 proteins in 12 species: Archae - 0; Bacteria - 0; Metazoa - 0; Fungi - 0; Plants - 127; Viruses - 0; Other Eukaryotes - 0 (source: NCBI BLink). | chr1:10430025-10431161 FORWARDmoderately similar to ( 332) loc_os10g42770 12010.m07029 protein expressed protein    TrbL Sporozoite_P67 PRK08026 COG4907  no original description 	T
26.3.1	misc.gluco-, galacto- and mannosidases.alpha-galactosidase	MDP0000455477	 nearly identical (1426) AT1G68560 | Symbols: ATXYL1, XYL1 | XYL1 (ALPHA-XYLOSIDASE 1); alpha-N-arabinofuranosidase/ hydrolase, hydrolyzing O-glycosyl compounds / xylan 1,4-beta-xylosidase | chr1:25734435-25737897 REVERSEhighly similar to ( 821) AGLU_ORYSA Probable alpha-glucosidase Os06g0675700 precursor (EC 3.2.1.20) (Maltase) - Oryza sativa (Rice)nearly identical (1269) loc_os01g03950 12001.m07031 protein alpha-D-xylosidase, putative, expressed  TrbL  no original description 	T
26.12	misc.peroxidases	MDP0000176436	 moderately similar to ( 417) AT1G71695 | Symbols:  | peroxidase 12 (PER12) (P12) (PRXR6) | chr1:26964359-26966557 FORWARDmoderately similar to ( 219) PER2_ORYSA Peroxidase 2 precursor (EC 1.11.1.7) - Oryza sativa (Rice)moderately similar to ( 379) loc_os01g73220 12001.m13351 protein peroxidase 12 precursor, putative, expressed  no original description 	T
23.3.3	nucleotide metabolism.salvage.NUDIX hydrolases	MDP0000264123	 weakly similar to ( 162) AT3G26690 | Symbols: ATNUDT13, ATNUDX13 | ATNUDX13 (ARABIDOPSIS THALIANA NUDIX HYDROLASE HOMOLOG 13); bis(5'-adenosyl)-pentaphosphatase/ hydrolase | chr3:9804418-9805398 REVERSEvery weakly similar to (85.5) RS27_HORVU 40S ribosomal protein S27 (Manganese efficiency-related protein 1) - Hordeum vulgare (Barley)weakly similar to ( 174) loc_os11g32750 12011.m28680 protein nudix hydrolase 13, mitochondrial precursor, putative, expressed no original description 	T
35.2	not assigned.unknown	MDP0000284049	 weakly similar to ( 142) AT2G23090 | Symbols:  | unknown protein | chr2:9829657-9830274 REVERSEweakly similar to ( 134) loc_os07g02340 12007.m04706 protein expressed protein no original description 	T
31.2	cell.division	MDP0000087507	 nearly identical (1452) AT2G17510 | Symbols: EMB2763 | EMB2763 (EMBRYO DEFECTIVE 2763); RNA binding / ribonuclease | chr2:7609395-7615881 REVERSEnearly identical (1426) loc_os03g03710 12003.m05904 protein exosome complex exonuclease RRP44, putative, expressed MSP1_C  no original description 	T
35.2	not assigned.unknown	MDP0000436956	 nearly identical (1052) ATCG01280 | Symbols: YCF2.2 | hypothetical protein | chrC:145291-152175 REVERSEnearly identical (1110) YCF2_GOSHI Protein ycf2 - Gossypium hirsutum (Upland cotton) no original description 	T
26.14	misc.oxygenases	MDP0000912144	 moderately similar to ( 446) AT3G18210 | Symbols:  | oxidoreductase, 2OG-Fe(II) oxygenase family protein | chr3:6238264-6240396 REVERSEmoderately similar to ( 475) loc_os01g31770 12001.m150600 protein oxidoreductase, putative, expressed  no original description 	T
29.2.1.1.3.2.1712	protein.synthesis.ribosomal protein.prokaryotic.unknown organellar.50S subunit.L7/L12	MDP0000262682	 moderately similar to ( 206) AT1G68660 | Symbols:  | FUNCTIONS IN: molecular_function unknown; INVOLVED IN: protein catabolic process; EXPRESSED IN: 23 plant structures; EXPRESSED DURING: 14 growth stages; CONTAINS InterPro DOMAIN/s: Adaptor protein ClpS, core (InterPro:IPR003769), Ribosomal protein L7/L12, C-terminal/adaptor protein ClpS-like (InterPro:IPR014719); Has 79 Blast hits to 79 proteins in 34 species: Archae - 0; Bacteria - 46; Metazoa - 0; Fungi - 0; Plants - 30; Viruses - 0; Other Eukaryotes - 3 (source: NCBI BLink). | chr1:25778058-25779083 REVERSEweakly similar to ( 181) loc_os08g33540 12008.m080165 protein ATP-dependent Clp protease adaptor protein ClpS containing protein, expressed no original description 	T
26.7	misc.oxidases - copper, flavone etc	MDP0000178612	 moderately similar to ( 410) AT3G03080 | Symbols:  | NADP-dependent oxidoreductase, putative | chr3:698530-700278 REVERSEmoderately similar to ( 378) loc_os12g12580 12012.m05231 protein NADP-dependent oxidoreductase P2, putative, expressed   no original description 	T
29.1.10	protein.aa activation.methionine-tRNA ligase	MDP0000139132	 weakly similar to ( 128) AT4G13780 | Symbols:  | methionine--tRNA ligase, putative / methionyl-tRNA synthetase, putative / MetRS, putative | chr4:7993366-7998433 REVERSEweakly similar to ( 123) SYM_ORYSA Probable methionyl-tRNA synthetase (EC 6.1.1.10) (Methionine--tRNA ligase) (MetRS) - Oryza sativa (Rice)weakly similar to ( 123) loc_os06g31210 12006.m07662 protein methionyl-tRNA synthetase, putative, expressed no original description 	T
27.3.67	RNA.regulation of transcription.putative transcription regulator	MDP0000537302	 moderately similar to ( 209) AT1G63470 | Symbols:  | DNA-binding family protein | chr1:23536831-23538863 REVERSEweakly similar to ( 158) loc_os02g57820 12002.m10803 protein DNA binding protein, putative, expressed   no original description 	T
29.4	protein.postranslational modification	MDP0000211527	 highly similar to ( 948) AT1G53050 | Symbols:  | protein kinase family protein | chr1:19772574-19775531 FORWARDmoderately similar to ( 219) CDC2_VIGUN Cell division control protein 2 homolog (EC 2.7.11.22) (EC 2.7.11.23) (p34cdc2) - Vigna unguiculata (Cowpea)highly similar to ( 776) loc_os01g10430 12001.m42914 protein transposon protein, putative, unclassified, expressed   no original description 	T
26.7	misc.oxidases - copper, flavone etc	MDP0000702799	 highly similar to ( 524) AT4G29720 | Symbols: ATPAO5 | ATPAO5 (Polyamine oxidase 5); amine oxidase/ electron carrier/ oxidoreductase | chr4:14553456-14555057 REVERSEvery weakly similar to (69.7) PAO_MAIZE Polyamine oxidase precursor (EC 1.5.3.11) - Zea mays (Maize)moderately similar to ( 361) loc_os01g51320 12001.m11313 protein peroxisomal N1-acetyl-spermine/spermidine oxidase, putative, expressed   no original description 	T
17.2.3	hormone metabolism.auxin.induced-regulated-responsive-activated	MDP0000193700	 highly similar to ( 705) AT2G46370 | Symbols: JAR1, FIN219 | JAR1 (JASMONATE RESISTANT 1); ATP binding / adenylyltransferase/ catalytic/ jasmonate-amino synthetase | chr2:19034579-19036369 FORWARDhighly similar to ( 721) GH35_ORYSA Probable indole-3-acetic acid-amido synthetase GH3.5 (EC 6.3.2.-) (Auxin-responsive GH3-like protein 5) (OsGH3-5) - Oryza sativa (Rice)highly similar to ( 721) loc_os05g50890 12005.m27991 protein indole-3-acetic acid-amido synthetase GH3.5, putative, expressed  no original description 	T
29.1.40	protein.aa activation.bifunctional aminoacyl-tRNA synthetase	MDP0000291077	 highly similar to ( 817) AT3G62120 | Symbols:  | tRNA synthetase class II (G, H, P and S) family protein | chr3:23001227-23003849 REVERSEhighly similar to ( 783) loc_os12g25710 12012.m073824 protein bifunctional aminoacyl-tRNA synthetase, putative, expressed  MSP1_C  no original description 	T
35.2	not assigned.unknown	MDP0000505478	 weakly similar to ( 170) AT3G50120 | Symbols:  | unknown protein | chr3:18585157-18587246 REVERSEweakly similar to ( 146) loc_os01g38380 12001.m10122 protein expressed protein no original description 	T
30.1	signalling.in sugar and nutrient physiology	MDP0000778016	 highly similar to ( 517) AT5G51550 | Symbols: EXL3 | EXL3 (EXORDIUM LIKE 3) | chr5:20939793-20940806 REVERSEmoderately similar to ( 416) loc_os06g04250 12006.m05154 protein phi-1-like phosphate-induced protein, putative, expressed   no original description 	T
8.1.4	TCA / org transformation.TCA.IDH	MDP0000389969	 highly similar to ( 603) AT4G35260 | Symbols: IDH1 | IDH1 (ISOCITRATE DEHYDROGENASE 1); isocitrate dehydrogenase (NAD+)/ oxidoreductase, acting on the CH-OH group of donors, NAD or NADP as acceptor | chr4:16774494-16776233 REVERSEmoderately similar to ( 276) LEU3_SOLTU 3-isopropylmalate dehydrogenase, chloroplast precursor (EC 1.1.1.85) (Beta-IPM dehydrogenase) (IMDH) (3-IPM-DH) - Solanum tuberosum (Potato)highly similar to ( 596) loc_os02g38200 12002.m08908 protein isocitrate dehydrogenase subunit 1, mitochondrial precursor, putative, expressed   no original description 	T
21.1	redox.thioredoxin	MDP0000789750	 highly similar to ( 650) AT1G04980 | Symbols: ATPDIL2-2, ATPDI10, PDI10 | ATPDIL2-2 (PDI-LIKE 2-2); protein disulfide isomerase | chr1:1413869-1416120 REVERSEweakly similar to ( 144) PDIA6_MEDSA Probable protein disulfide-isomerase A6 precursor (EC 5.3.4.1) (P5) - Medicago sativa (Alfalfa)highly similar to ( 680) loc_os09g27830 12009.m22113 protein OsPDIL2-3 - Oryza sativa protein disulfide isomerase, expressed   no original description 	T
3.8.2	minor CHO metabolism.galactose.alpha-galactosidases	MDP0000213588	 highly similar to ( 567) AT3G56310 | Symbols:  | alpha-galactosidase, putative / melibiase, putative / alpha-D-galactoside galactohydrolase, putative | chr3:20882886-20885745 FORWARDmoderately similar to ( 390) AGAL_ORYSA Alpha-galactosidase precursor (EC 3.2.1.22) (Melibiase) (Alpha-D-galactoside galactohydrolase) - Oryza sativa (Rice)highly similar to ( 522) loc_os07g48160 12007.m09030 protein alpha-galactosidase precursor, putative, expressed   no original description 	T
27.3.6	RNA.regulation of transcription.bHLH,Basic Helix-Loop-Helix family	MDP0000829998	 moderately similar to ( 214) AT2G46810 | Symbols:  | basic helix-loop-helix (bHLH) family protein | chr2:19239694-19242373 FORWARDweakly similar to ( 180) loc_os09g29360 12009.m06087 protein DNA binding protein, putative, expressed no original description 	T
29.5.4	protein.degradation.aspartate protease	MDP0000121524	 highly similar to ( 616) AT2G36670 | Symbols:  | aspartyl protease family protein | chr2:15364949-15368016 REVERSEweakly similar to ( 114) ASP1_ORYSA Aspartic proteinase Asp1 precursor (EC 3.4.23.-) (OsAsp1) (OSAP1) (Nucellin-like protein) - Oryza sativa (Rice)highly similar to ( 533) loc_os01g56930 12001.m150738 protein aspartic proteinase nepenthesin-2 precursor, putative, expressed  no original description 	T
35.1	not assigned.no ontology	MDP0000295077	 weakly similar to ( 179) AT1G04340 | Symbols:  | lesion inducing protein-related | chr1:1163345-1164737 REVERSEweakly similar to ( 196) loc_os03g05520 12003.m06075 protein nicotiana lesion-inducing like, putative, expressed  no original description 	T
34.11	transport.NDP-sugars at the ER	MDP0000190282	 very weakly similar to (70.1) AT4G09810 | Symbols:  | transporter-related | chr4:6175415-6176892 REVERSEvery weakly similar to (71.6) loc_os02g40090 12002.m100277 protein integral membrane protein like, putative, expressed  no original description 	T
27.3.11	RNA.regulation of transcription.C2H2 zinc finger family	MDP0000133494	 weakly similar to ( 144) AT2G45120 | Symbols:  | zinc finger (C2H2 type) family protein | chr2:18603680-18604624 FORWARDvery weakly similar to (52.4) ZFP1_WHEAT Zinc-finger protein 1 (WZF1) - Triticum aestivum (Wheat)weakly similar to ( 134) loc_os10g40660 12010.m06836 protein cys2/His2 zinc-finger transcription factor, putative, expressed   no original description 	T
30.7	signalling.14-3-3 proteins	MDP0000253447	 moderately similar to ( 417) AT1G78300 | Symbols: GRF2, 14-3-3OMEGA, GF14 OMEGA | GRF2 (GENERAL REGULATORY FACTOR 2); protein binding / protein phosphorylated amino acid binding | chr1:29461883-29463052 FORWARDmoderately similar to ( 436) 14335_SOLTU 14-3-3-like protein 16R - Solanum tuberosum (Potato)moderately similar to ( 406) loc_os03g50290 12003.m35391 protein 14-3-3-like protein S94, putative, expressed  no original description 	T
35.2	not assigned.unknown	MDP0000270083	 weakly similar to ( 132) AT5G13720 | Symbols:  | LOCATED IN: chloroplast, chloroplast inner membrane, chloroplast envelope; EXPRESSED IN: 21 plant structures; EXPRESSED DURING: 13 growth stages; CONTAINS InterPro DOMAIN/s: Uncharacterised conserved protein UCP022348 (InterPro:IPR016804), Uncharacterised protein family UPF0114 (InterPro:IPR005134); BEST Arabidopsis thaliana protein match is: unknown protein (TAIR:AT4G19390.1); Has 294 Blast hits to 294 proteins in 98 species: Archae - 18; Bacteria - 168; Metazoa - 0; Fungi - 0; Plants - 53; Viruses - 0; Other Eukaryotes - 55 (source: NCBI BLink). | chr5:4427960-4429029 FORWARDweakly similar to ( 127) loc_os07g46320 12007.m08849 protein uncharacterized protein UPF0114, putative, expressed  no original description 	T
29.5.11.1	protein.degradation.ubiquitin.ubiquitin	MDP0000840072	 weakly similar to ( 157) AT4G26840 | Symbols: SUM1, SUMO 1, SUMO1, ATSUMO1 | SUMO1 (SMALL UBIQUITIN-LIKE MODIFIER 1); protein binding / protein tag | chr4:13497466-13498458 FORWARDweakly similar to ( 149) SMT3_ORYSA Ubiquitin-like protein SMT3 - Oryza sativa (Rice)weakly similar to ( 149) loc_os01g68950 12001.m42793 protein ubiquitin-like protein SMT3, putative, expressed no original description 	T
20.2.99	stress.abiotic.unspecified	MDP0000142824	 weakly similar to ( 189) AT3G62550 | Symbols:  | universal stress protein (USP) family protein | chr3:23136194-23137074 FORWARDweakly similar to ( 148) loc_os01g19820 12001.m150564 protein universal stress protein, putative, expressed  no original description 	T
35.2	not assigned.unknown	MDP0000122917	 highly similar to ( 920) AT1G09780 | Symbols:  | 2,3-biphosphoglycerate-independent phosphoglycerate mutase, putative / phosphoglyceromutase, putative | chr1:3165550-3167812 REVERSEhighly similar to ( 965) PMGI_MESCR 2,3-bisphosphoglycerate-independent phosphoglycerate mutase (EC 5.4.2.1) (Phosphoglyceromutase) (BPG-independent PGAM) (PGAM-I) - Mesembryanthemum crystallinum (Common ice plant)highly similar to ( 952) loc_os01g60190 12001.m42741 protein 2,3-bisphosphoglycerate-independent phosphoglycerate mutase, putative, expressed  no original description 	T
26.2	misc.UDP glucosyl and glucoronyl transferases	MDP0000460504	 moderately similar to ( 216) AT4G38040 | Symbols:  | exostosin family protein | chr4:17867501-17869131 FORWARDmoderately similar to ( 232) loc_os02g09430 12002.m06240 protein exostosin-like, putative, expressed   no original description 	T
35.2	not assigned.unknown	MDP0000156789	 weakly similar to ( 156) AT1G42960 | Symbols:  | expressed protein localized to the inner membrane of the chloroplast. | chr1:16125863-16127080 FORWARDvery weakly similar to (65.5) loc_os12g39370 12012.m073869 protein expressed protein no original description 	T
29.2.1.2.2.5	protein.synthesis.ribosomal protein.eukaryotic.60S subunit.L5	MDP0000334079	 moderately similar to ( 474) AT3G25520 | Symbols: ATL5, PGY3, OLI5, RPL5A | ATL5 (A. THALIANA RIBOSOMAL PROTEIN L5); 5S rRNA binding / structural constituent of ribosome | chr3:9269573-9271327 REVERSEmoderately similar to ( 481) RL5_CUCSA 60S ribosomal protein L5 - Cucumis sativus (Cucumber)moderately similar to ( 448) loc_os01g67126 12001.m43540 protein 60S ribosomal protein L5-2, putative, expressed  no original description 	T
17.6.3	hormone metabolism.gibberelin.induced-regulated-responsive-activated	MDP0000232908	 weakly similar to ( 110) AT5G15230 | Symbols: GASA4 | GASA4 (GAST1 PROTEIN HOMOLOG 4) | chr5:4945286-4946025 FORWARDvery weakly similar to (87.4) loc_os09g24840 12009.m22085 protein GAST1 protein precursor, putative, expressed  no original description 	T
27.3.67	RNA.regulation of transcription.putative transcription regulator	MDP0000267648	 highly similar to ( 545) AT2G47790 | Symbols:  | transducin family protein / WD-40 repeat family protein | chr2:19571102-19572946 REVERSEvery weakly similar to (59.3) GBB_ORYSA Guanine nucleotide-binding protein subunit beta - Oryza sativa (Rice)moderately similar to ( 467) loc_os12g19590 12012.m26894 protein nucleotide binding protein, putative, expressed  no original description 	T
35.2	not assigned.unknown	MDP0000736035	     no original description 	T
1.1.5.2	PS.lightreaction.other electron carrier (ox/red).ferredoxin	MDP0000280246	 moderately similar to ( 206) AT3G16250 | Symbols: NDF4 | NDF4 (NDH-DEPENDENT CYCLIC ELECTRON FLOW 1); electron carrier/ iron-sulfur cluster binding | chr3:5507091-5508320 REVERSEweakly similar to ( 188) loc_os07g30670 12007.m079681 protein electron carrier/ electron transporter/ iron ion binding protein, putative, expressed  no original description 	T
34.2	transport.sugars	MDP0000896307	 highly similar to ( 667) AT2G16120 | Symbols:  | mannitol transporter, putative | chr2:6996727-6998441 REVERSEmoderately similar to ( 207) HEX6_RICCO Hexose carrier protein HEX6 - Ricinus communis (Castor bean)highly similar to ( 657) loc_os07g39350 12007.m08177 protein proton myo-inositol cotransporter, putative, expressed no original description 	T
10.2.1	cell wall.cellulose synthesis.cellulose synthase	MDP0000480237	 nearly identical (1757) AT4G39350 | Symbols: CESA2, ATH-A, ATCESA2 | CESA2 (CELLULOSE SYNTHASE A2); cellulose synthase/ transferase, transferring glycosyl groups | chr4:18297078-18301890 FORWARDnearly identical (1553) loc_os03g62090 12003.m11087 protein CESA5 - cellulose synthase, expressed  PRK07003  no original description 	T
29.4	protein.postranslational modification	MDP0000161427	 highly similar to ( 518) AT5G56580 | Symbols: ATMKK6, ANQ1, MKK6 | MKK6 (MAP KINASE KINASE 6); MAP kinase kinase/ kinase | chr5:22904851-22906620 REVERSEmoderately similar to ( 451) M2K1_ORYSA Mitogen-activated protein kinase kinase 1 (EC 2.7.12.2) (MAP kinase kinase 1) (MAPKK1) (OsMEK1) - Oryza sativa (Rice)moderately similar to ( 451) loc_os01g32660 12001.m43029 protein OsMKK6 - putative MAPKK based on amino acid sequence homology, expressed  no original description 	T
27.2	RNA.transcription	MDP0000172972	 moderately similar to ( 422) AT1G14790 | Symbols: RDR1, ATRDRP1 | RDR1 (RNA-DEPENDENT RNA POLYMERASE 1); RNA-directed RNA polymerase/ nucleic acid binding | chr1:5094317-5097817 REVERSEweakly similar to ( 121) loc_os04g39160 12004.m08905 protein RNA-dependent RNA polymerase homolog 1, putative, expressed  no original description 	T
28.1.3	DNA.synthesis/chromatin structure.histone	MDP0000364547	 moderately similar to ( 228) AT5G65360 | Symbols:  | histone H3 | chr5:26120099-26120509 REVERSEmoderately similar to ( 226) H32_WHEAT Histone H3.2 - Triticum aestivum (Wheat)moderately similar to ( 228) loc_os11g05730 12011.m04769 protein histone H3, putative, expressed no original description 	T
29.3	protein.targeting	MDP0000171639	 moderately similar to ( 329) AT2G20890 | Symbols: PSB29, THF1 | PSB29 | chr2:8987783-8989185 FORWARDmoderately similar to ( 321) THF1_SOLTU Protein THYLAKOID FORMATION1, chloroplast precursor - Solanum tuberosum (Potato)moderately similar to ( 330) loc_os07g37250 12007.m07975 protein chloroplast-localized Ptr ToxA-binding protein1, putative, expressed  no original description 	T
21.1.1	redox.thioredoxin.PDIL	MDP0000567216	 highly similar to ( 702) AT5G60640 | Symbols: ATPDIL1-4 | ATPDIL1-4 (PDI-LIKE 1-4); protein disulfide isomerase | chr5:24371416-24373993 REVERSEmoderately similar to ( 243) PDI_WHEAT Protein disulfide-isomerase precursor (EC 5.3.4.1) (PDI) - Triticum aestivum (Wheat)highly similar to ( 629) loc_os02g01010 12002.m05451 protein OsPDIL1-4 - Oryza sativa protein disulfide isomerase, expressed   no original description 	T
35.2	not assigned.unknown	MDP0000913740	 weakly similar to ( 175) AT4G02725 | Symbols:  | unknown protein | chr4:1206055-1207000 FORWARDvery weakly similar to (68.9) loc_os03g49450 12003.m09944 protein hypothetical protein no original description 	T
29.2.1.1.2.1.29	protein.synthesis.ribosomal protein.prokaryotic.mitochondrion.30S subunit.S29	MDP0000282183	 highly similar to ( 558) AT1G16870 | Symbols:  | mitochondrial 28S ribosomal protein S29-related | chr1:5770964-5773157 REVERSEmoderately similar to ( 488) loc_os07g10300 12007.m29094 protein expressed protein   no original description 	T
35.2	not assigned.unknown	MDP0000304723	 moderately similar to ( 370) AT2G39950 | Symbols:  | unknown protein | chr2:16676758-16679961 REVERSEmoderately similar to ( 323) loc_os07g01060 12007.m04587 protein expressed protein  no original description 	T
35.2	not assigned.unknown	MDP0000139404	 moderately similar to ( 474) AT1G79500 | Symbols:  | 2-dehydro-3-deoxyphosphooctonate aldolase / phospho-2-dehydro-3-deoxyoctonate aldolase / 3-deoxy-D-manno-octulosonic acid 8-phosphate synthetase (KDSA) | chr1:29903797-29907730 FORWARDmoderately similar to ( 476) KDSA_PEA 2-dehydro-3-deoxyphosphooctonate aldolase (EC 2.5.1.55) (Phospho-2-dehydro-3-deoxyoctonate aldolase) (3-deoxy-D-manno-octulosonic acid 8-phosphate synthetase) (KDO-8-phosphate synthetase) (KDO 8-P synthase) (KDOPS) - Pisum sativum (Gardenmoderately similar to ( 464) loc_os12g10784 12012.m073793 protein 2-dehydro-3-deoxyphosphooctonate aldolase, putative, expressed  no original description 	T
31.1	cell.organisation	MDP0000291916	 highly similar to ( 774) AT1G13180 | Symbols: DIS1, ARP3, ATARP3 | DIS1 (DISTORTED TRICHOMES 1); ATP binding / actin binding / protein binding / structural constituent of cytoskeleton | chr1:4495394-4498204 FORWARDmoderately similar to ( 224) ACT_VOLCA Actin - Volvox carterihighly similar to ( 741) loc_os02g38340 12002.m08922 protein actin-like protein 3, putative, expressed no original description 	T
9.1.2	mitochondrial electron transport / ATP synthesis.NADH-DH.localisation not clear	MDP0000191266	 highly similar to ( 853) AT5G08530 | Symbols: CI51 | CI51 (51 kDa subunit of complex I); 4 iron, 4 sulfur cluster binding / FMN binding / NAD or NADH binding / NADH dehydrogenase (ubiquinone)/ oxidoreductase, acting on NADH or NADPH | chr5:2759848-2761726 REVERSEhighly similar to ( 850) loc_os07g45090 12007.m079768 protein NADH-ubiquinone oxidoreductase 51 kDa subunit, mitochondrial precursor, putative, expressed no original description 	T
27.3.36	RNA.regulation of transcription.Argonaute	MDP0000260407	 moderately similar to ( 391) AT1G31280 | Symbols: AGO2 | AGO2 (argonaute 2); nucleic acid binding | chr1:11181777-11185112 FORWARDmoderately similar to ( 340) loc_os04g52540 12004.m10147 protein eukaryotic translation initiation factor 2C 2, putative, expressed         no original description 	T
21.1	redox.thioredoxin	MDP0000670699	 weakly similar to ( 183) AT1G50320 | Symbols: ATHX, ATX | ATHX; enzyme activator | chr1:18638606-18639464 REVERSEvery weakly similar to (92.4) TRXM_CHLRE Thioredoxin M-type, chloroplast precursor (TRX-M) (Thioredoxin CH2) - Chlamydomonas reinhardtiiweakly similar to ( 167) loc_os04g57930 12004.m10677 protein thioredoxin X, chloroplast precursor, putative, expressed  no original description 	T
3.8.2	minor CHO metabolism.galactose.alpha-galactosidases	MDP0000148840	 moderately similar to ( 300) AT5G08380 | Symbols: AtAGAL1 | AtAGAL1 (Arabidopsis thaliana ALPHA-GALACTOSIDASE 1); alpha-galactosidase/ catalytic/ hydrolase, hydrolyzing O-glycosyl compounds | chr5:2694851-2697616 REVERSEmoderately similar to ( 236) AGAL_ORYSA Alpha-galactosidase precursor (EC 3.2.1.22) (Melibiase) (Alpha-D-galactoside galactohydrolase) - Oryza sativa (Rice)moderately similar to ( 259) loc_os10g35070 12010.m065297 protein alpha-galactosidase precursor, putative, expressed no original description 	T
29.5.11.4.2	protein.degradation.ubiquitin.E3.RING	MDP0000864704	 weakly similar to ( 150) AT1G76410 | Symbols: ATL8 | ATL8; protein binding / zinc ion binding | chr1:28668915-28669472 FORWARDvery weakly similar to (80.1) EL5_ORYSA E3 ubiquitin-protein ligase EL5 (EC 6.3.2.-) - Oryza sativa (Rice)weakly similar to ( 107) loc_os02g46100 12002.m09647 protein RING-H2 finger protein ATL1R, putative, expressed  no original description 	T
13.2.6.2	amino acid metabolism.degradation.aromatic aa.tyrosine	MDP0000260790	 highly similar to ( 504) AT5G36160 | Symbols:  | aminotransferase-related | chr5:14233261-14235129 REVERSEvery weakly similar to (80.9) ALA2_HORVU Alanine aminotransferase 2 (EC 2.6.1.2) (GPT) (Glutamic--pyruvic transaminase 2) (Glutamic--alanine transaminase 2) (ALAAT-2) - Hordeum vulgare (Barley)highly similar to ( 512) loc_os11g35040 12011.m07358 protein tyrosine aminotransferase, putative, expressed   no original description 	T
34.3	transport.amino acids	MDP0000647348	 highly similar to ( 608) AT1G80510 | Symbols:  | amino acid transporter family protein | chr1:30273100-30274569 FORWARDhighly similar to ( 517) loc_os06g43700 12006.m08900 protein amino acid permease, putative, expressed  no original description 	T
11.3.3	lipid metabolism.Phospholipid synthesis.phosphatidate cytidylyltransferase	MDP0000167422	 moderately similar to ( 305) AT5G58560 | Symbols:  | phosphatidate cytidylyltransferase family protein | chr5:23670513-23672570 FORWARDmoderately similar to ( 302) PHYK3_SOYBN Probable phytol kinase 3, chloroplast precursor (EC 2.7.-.-) - Glycine max (Soybean)moderately similar to ( 272) loc_os01g61560 12001.m43251 protein phytol kinase 2, chloroplast precursor, putative, expressed  no original description 	T
29.5.11.20	protein.degradation.ubiquitin.proteasom	MDP0000301440	 highly similar to ( 658) AT4G24820 | Symbols:  | 26S proteasome regulatory subunit, putative (RPN7) | chr4:12790471-12792599 REVERSEhighly similar to ( 638) PSMD6_ORYSA 26S proteasome non-ATPase regulatory subunit 6 (26S proteasome regulatory particle non-ATPase subunit 7) (OsRPN7) - Oryza sativa (Rice)highly similar to ( 638) loc_os04g40850 12004.m35346 protein 26S proteasome non-ATPase regulatory subunit 6, putative, expressed   no original description 	T
34.99	transport.misc	MDP0000321341	 highly similar to ( 732) AT1G76550 | Symbols:  | pyrophosphate--fructose-6-phosphate 1-phosphotransferase alpha subunit, putative / pyrophosphate-dependent 6-phosphofructose-1-kinase, putative | chr1:28722900-28726929 REVERSEhighly similar to ( 769) PFPA_RICCO Pyrophosphate--fructose 6-phosphate 1-phosphotransferase alpha subunit (EC 2.7.1.90) (PFP) (6-phosphofructokinase, pyrophosphate dependent) (Pyrophosphate-dependent 6-phosphofructose-1-kinase) (PPi-PFK) - Ricinus communis (Castor bean)highly similar to ( 722) loc_os02g48360 12002.m100340 protein pyrophosphate--fructose 6-phosphate 1-phosphotransferase alpha subunit, putative, expressed  no original description 	T
11.3.5	lipid metabolism.Phospholipid synthesis.diacylglycerol kinase	MDP0000900186	 highly similar to ( 972) AT5G63770 | Symbols: ATDGK2 | ATDGK2 (Diacylglycerol kinase 2); diacylglycerol kinase | chr5:25519755-25522404 FORWARDhighly similar to ( 845) loc_os12g12260 12012.m05200 protein diacylglycerol kinase 1, putative, expressed  no original description 	T
10.7	cell wall.modification	MDP0000661960	 moderately similar to ( 469) AT5G65730 | Symbols:  | xyloglucan:xyloglucosyl transferase, putative / xyloglucan endotransglycosylase, putative / endo-xyloglucan transferase, putative | chr5:26299080-26300290 FORWARDmoderately similar to ( 315) XTH_TOBAC Probable xyloglucan endotransglucosylase/hydrolase protein precursor (EC 2.4.1.207) - Nicotiana tabacum (Common tobacco)moderately similar to ( 309) loc_os11g33270 12011.m07226 protein xyloglucan endotransglucosylase/hydrolase precursor, putative, expressedCOG3889  no original description 	T
34.22	transport.cyclic nucleotide or calcium regulated channels	MDP0000321613	 weakly similar to ( 183) AT5G53130 | Symbols: CNGC1, ATCNGC1 | CNGC1 (CYCLIC NUCLEOTIDE GATED CHANNEL 1); calmodulin binding / cation channel/ cyclic nucleotide binding / intracellular cyclic nucleotide activated cation channel/ inward rectifier potassium channel | chr5:21537830-21540490 REVERSEweakly similar to ( 173) loc_os02g15580 12002.m100777 protein cyclic nucleotide-gated ion channel 1, putative, expressed  no original description 	T
35.1	not assigned.no ontology	MDP0000239038	 highly similar to ( 633) AT2G20635 | Symbols:  | ATP binding / protein kinase/ protein serine/threonine kinase | chr2:8900410-8903384 FORWARDmoderately similar to ( 410) loc_os07g32480 12007.m07513 protein mitotic checkpoint serine/threonine-protein kinase BUB1, putative, expressed  no original description 	T
29.3.4.3	protein.targeting.secretory pathway.vacuole	MDP0000134944	 nearly identical (1262) AT2G38020 | Symbols: VCL1, MAN, EMB258 | VCL1 (VACUOLELESS 1) | chr2:15910272-15914190 REVERSEnearly identical (1021) loc_os01g47650 12001.m10958 protein VCL1, putative, expressed  no original description 	T
29.5.11.3	protein.degradation.ubiquitin.E2	MDP0000131917	 moderately similar to ( 374) AT3G17000 | Symbols: UBC32 | UBC32 (ubiquitin-conjugating enzyme 32); ubiquitin-protein ligase | chr3:5797296-5799467 FORWARDvery weakly similar to (62.0) UBC2_MEDSA Ubiquitin-conjugating enzyme E2-17 kDa (EC 6.3.2.19) (Ubiquitin-protein ligase) (Ubiquitin carrier protein) - Medicago sativa (Alfalfa)moderately similar to ( 336) loc_os03g19500 12003.m07367 protein ubiquitinating enzyme, putative, expressed no original description 	T
30.4.5	signalling.phosphinositides.inositol-1,3,4-trisphosphate 5/6-kinase	MDP0000161947	 moderately similar to ( 305) AT5G16760 | Symbols:  | inositol 1,3,4-trisphosphate 5/6-kinase | chr5:5509890-5510849 FORWARDmoderately similar to ( 278) ITPK1_MAIZE Inositol-tetrakisphosphate 1-kinase 1 (EC 2.7.1.134) (Inositol-triphosphate 5/6-kinase 1) (EC 2.7.1.159) (Inositol 1,3,4-trisphosphate 5/6-kinase 1) (Ins(1,3,4)P(3) 5/6-kinase 1) (ZmIpk) (Low phytic acid protein 2) - Zea mays (Maize)moderately similar to ( 284) loc_os10g42550 12010.m07008 protein inositol-tetrakisphosphate 1-kinase 1, putative, expressed   no original description 	T
1.3.5	PS.calvin cycle.TPI	MDP0000694943	 moderately similar to ( 396) AT3G55440 | Symbols: ATCTIMC, TPI | TPI (TRIOSEPHOSPHATE ISOMERASE); triose-phosphate isomerase | chr3:20553794-20556078 FORWARDmoderately similar to ( 411) TPIS_PETHY Triosephosphate isomerase, cytosolic (EC 5.3.1.1) (TIM) (Triose-phosphate isomerase) - Petunia hybrida (Petunia)moderately similar to ( 398) loc_os01g05490 12001.m42862 protein triosephosphate isomerase, cytosolic, putative, expressed no original description 	T
4.1.7	glycolysis.cytosolic branch.triosephosphate isomerase (TPI)	MDP0000694943	 moderately similar to ( 396) AT3G55440 | Symbols: ATCTIMC, TPI | TPI (TRIOSEPHOSPHATE ISOMERASE); triose-phosphate isomerase | chr3:20553794-20556078 FORWARDmoderately similar to ( 411) TPIS_PETHY Triosephosphate isomerase, cytosolic (EC 5.3.1.1) (TIM) (Triose-phosphate isomerase) - Petunia hybrida (Petunia)moderately similar to ( 398) loc_os01g05490 12001.m42862 protein triosephosphate isomerase, cytosolic, putative, expressed no original description 	T
29.2.1.2.2.39	protein.synthesis.ribosomal protein.eukaryotic.60S subunit.L39	MDP0000786306	 weakly similar to ( 110) AT4G31985 | Symbols:  | 60S ribosomal protein L39 (RPL39C) | chr4:15469931-15470366 FORWARDweakly similar to ( 107) RL39_ORYSA 60S ribosomal protein L39 - Oryza sativa (Rice)weakly similar to ( 107) loc_os06g08320 12006.m05553 protein 60S ribosomal protein L39, putative, expressed no original description 	T
31.3.1	cell.cycle.peptidylprolyl isomerase	MDP0000183239	 moderately similar to ( 306) AT4G34960 | Symbols:  | peptidyl-prolyl cis-trans isomerase, putative / cyclophilin, putative / rotamase, putative | chr4:16648865-16650646 FORWARDweakly similar to ( 174) CYPH_CATRO Peptidyl-prolyl cis-trans isomerase (EC 5.2.1.8) (PPIase) (Rotamase) (Cyclophilin) (Cyclosporin A-binding protein) - Catharanthus roseus (Rosy periwinkle) (Madagascar periwinkle)moderately similar to ( 279) loc_os09g36670 12009.m06616 protein peptidyl-prolyl cis-trans isomerase CYP19-4 precursor, putative, expressed no original description 	T
35.2	not assigned.unknown	MDP0000771589	 moderately similar to ( 361) AT1G03900 | Symbols: ATNAP4 | ATNAP4 (Arabidopsis thaliana non-intrinsic ABC protein 4); ATPase, coupled to transmembrane movement of substances / transporter | chr1:991252-992852 FORWARDmoderately similar to ( 367) loc_os10g33630 12010.m065199 protein adaptin ear-binding coat-associated protein 2, putative, expressed   no original description 	T
33.99	development.unspecified	MDP0000304459	 weakly similar to ( 132) AT3G63210 | Symbols: MARD1 | MARD1 | chr3:23354019-23354906 REVERSEweakly similar to ( 108) loc_os03g46260 12003.m101430 protein expressed protein  no original description 	T
26.3.1	misc.gluco-, galacto- and mannosidases.alpha-galactosidase	MDP0000178546	 highly similar to ( 849) AT5G63840 | Symbols: RSW3 | RSW3 (RADIAL SWELLING 3); glucosidase/ hydrolase, hydrolyzing O-glycosyl compounds | chr5:25545056-25548922 FORWARDmoderately similar to ( 265) AGLU_ORYSA Probable alpha-glucosidase Os06g0675700 precursor (EC 3.2.1.20) (Maltase) - Oryza sativa (Rice)highly similar to ( 674) loc_os03g11720 12003.m06624 protein neutral alpha-glucosidase AB precursor, putative, expressed  no original description 	T
35.2	not assigned.unknown	MDP0000347048	 moderately similar to ( 344) AT4G17790 | Symbols:  | INVOLVED IN: biological_process unknown; EXPRESSED IN: 23 plant structures; EXPRESSED DURING: 15 growth stages; CONTAINS InterPro DOMAIN/s: SNARE associated Golgi protein (InterPro:IPR015414); BEST Arabidopsis thaliana protein match is: unknown protein (TAIR:AT1G71940.1); Has 2258 Blast hits to 2258 proteins in 517 species: Archae - 4; Bacteria - 1129; Metazoa - 188; Fungi - 22; Plants - 104; Viruses - 0; Other Eukaryotes - 811 (source: NCBI BLink). | chr4:9891425-9892675 FORWARDmoderately similar to ( 345) loc_os02g46660 12002.m09703 protein gtk16 protein, putative, expressed no original description 	T
35.1	not assigned.no ontology	MDP0000151239	 moderately similar to ( 468) AT1G52870 | Symbols:  | peroxisomal membrane protein-related | chr1:19685856-19687678 FORWARDmoderately similar to ( 427) loc_os03g38730 12003.m35298 protein mpv17 / PMP22 family protein, expressed     no original description 	T
35.2	not assigned.unknown	MDP0000125328	 highly similar to ( 771) AT3G50590 | Symbols:  | nucleotide binding | chr3:18771292-18779220 FORWARDhighly similar to ( 701) loc_os09g24260 12009.m05582 protein nucleotide binding protein, putative, expressed   no original description 	T
35.1	not assigned.no ontology	MDP0000276802	 moderately similar to ( 278) AT5G41600 | Symbols: BTI3 | BTI3 (VIRB2-INTERACTING PROTEIN 3) | chr5:16636230-16637472 FORWARDmoderately similar to ( 268) loc_os05g45050 12005.m083785 protein seed maturation protein, putative, expressed  no original description 	T
29.2.1.2.2.30	protein.synthesis.ribosomal protein.eukaryotic.60S subunit.L30	MDP0000300610	 weakly similar to ( 171) AT1G36240 | Symbols:  | 60S ribosomal protein L30 (RPL30A) | chr1:13614890-13616233 FORWARDweakly similar to ( 178) RL30_LUPLU 60S ribosomal protein L30 - Lupinus luteus (European yellow lupin)weakly similar to ( 158) loc_os01g16890 12001.m42968 protein 60S ribosomal protein L30, putative, expressed no original description 	T
29.3.1	protein.targeting.nucleus	MDP0000847124	 nearly identical (1406) AT2G46520 | Symbols:  | cellular apoptosis susceptibility protein, putative / importin-alpha re-exporter, putative | chr2:19096867-19099785 FORWARDhighly similar to ( 964) loc_os01g13430 12001.m07952 protein importin-alpha re-exporter, putative, expressed   no original description 	T
20.1	stress.biotic	MDP0000701077	 moderately similar to ( 481) AT1G05850 | Symbols: POM1, ERH2, ELP1, CTL1, ELP, HOT2, ATCTL1 | POM1 (POM-POM1); chitinase | chr1:1766833-1768117 REVERSEweakly similar to ( 182) CHI2_BRANA Endochitinase CH25 precursor (EC 3.2.1.14) - Brassica napus (Rape)moderately similar to ( 426) loc_os09g32080 12009.m060024 protein endochitinase A2 precursor, putative, expressed no original description 	T
35.1	not assigned.no ontology	MDP0000228047	 moderately similar to ( 342) AT3G62040 | Symbols:  | catalytic/ hydrolase | chr3:22975328-22976518 REVERSEmoderately similar to ( 353) loc_os03g61829 12003.m101672 protein catalytic/ hydrolase, putative, expressed  no original description 	T
3.5	minor CHO metabolism.others	MDP0000737131	 moderately similar to ( 426) AT5G15140 | Symbols:  | aldose 1-epimerase family protein | chr5:4908686-4910670 FORWARDmoderately similar to ( 388) loc_os04g38530 12004.m08843 protein aldose 1-epimerase precursor, putative, expressed   no original description 	T
35.2	not assigned.unknown	MDP0000287127	 weakly similar to ( 178) AT5G01120 | Symbols:  | unknown protein | chr5:45281-46986 FORWARDweakly similar to ( 133) loc_os04g50800 12004.m09977 protein expressed protein no original description 	T
4.2.3	glycolysis.plastid branch.glucose-6-phosphate isomerase	MDP0000931200	 highly similar to ( 918) AT5G42740 | Symbols:  | glucose-6-phosphate isomerase, cytosolic (PGIC) | chr5:17136080-17140622 FORWARDhighly similar to ( 913) G6PI_SPIOL Glucose-6-phosphate isomerase, cytosolic (EC 5.3.1.9) (GPI) (Phosphoglucose isomerase) (PGI) (Phosphohexose isomerase) (PHI) - Spinacia oleracea (Spinach)highly similar to ( 871) loc_os03g56460 12003.m101518 protein glucose-6-phosphate isomerase, cytosolic A, putative, expressed   no original description 	T
27.3.6	RNA.regulation of transcription.bHLH,Basic Helix-Loop-Helix family	MDP0000181746	 weakly similar to ( 114) AT5G64340 | Symbols: SAC51 | SAC51 (SUPPRESSOR OF ACAULIS 51); transcription factor/ transcription regulator | chr5:25730890-25731936 REVERSEvery weakly similar to (91.3) loc_os03g27390 12003.m35261 protein expressed protein  no original description 	T
35.1.5	not assigned.no ontology.pentatricopeptide (PPR) repeat-containing protein	MDP0000135665	 moderately similar to ( 328) AT4G21190 | Symbols: emb1417 | emb1417 (embryo defective 1417) | chr4:11292493-11293763 REVERSEmoderately similar to ( 310) loc_os01g55290 12001.m11688 protein EMB1417, putative, expressed  no original description 	T
30.6	signalling.MAP kinases	MDP0000880208	 moderately similar to ( 205) AT4G01370 | Symbols: ATMPK4, MPK4 | ATMPK4 (ARABIDOPSIS THALIANA MAP KINASE 4); MAP kinase/ kinase | chr4:567219-568889 FORWARDmoderately similar to ( 210) MMK2_MEDSA Mitogen-activated protein kinase homolog MMK2 (EC 2.7.11.24) - Medicago sativa (Alfalfa)moderately similar to ( 201) loc_os10g38950 12010.m06663 protein OsMPK4 - putative MAPK based on amino acid sequence homology, expressed no original description 	T
35.2	not assigned.unknown	MDP0000208850	 moderately similar to ( 242) AT4G23890 | Symbols:  | unknown protein | chr4:12420593-12421345 REVERSEweakly similar to ( 177) loc_os07g09800 12007.m05435 protein expressed protein  no original description 	T
35.1	not assigned.no ontology	MDP0000311623	 moderately similar to ( 310) AT4G23630 | Symbols: BTI1 | BTI1 (VIRB2-INTERACTING PROTEIN 1) | chr4:12318070-12319574 FORWARDmoderately similar to ( 310) loc_os01g12650 12001.m07878 protein seed maturation protein, putative, expressed  no original description 	T
33.99	development.unspecified	MDP0000295408	 weakly similar to ( 179) AT4G14320 | Symbols:  | 60S ribosomal protein L36a/L44 (RPL36aB) | chr4:8242684-8243805 REVERSEweakly similar to ( 187) RL44_GOSHI 60S ribosomal protein L44 - Gossypium hirsutum (Upland cotton)weakly similar to ( 184) loc_os07g33860 12007.m07644 protein 60S ribosomal protein L44, putative, expressed  no original description 	T
1.2.5	PS.photorespiration.serine hydroxymethyltransferase	MDP0000332596	 highly similar to ( 857) AT4G37930 | Symbols: SHM1, STM, SHMT1 | SHM1 (SERINE TRANSHYDROXYMETHYLTRANSFERASE 1); glycine hydroxymethyltransferase/ poly(U) binding | chr4:17831891-17834742 REVERSEhighly similar to ( 887) GLYM_SOLTU Serine hydroxymethyltransferase, mitochondrial precursor (EC 2.1.2.1) (Serine methylase) (Glycine hydroxymethyltransferase) (SHMT) - Solanum tuberosum (Potato)highly similar to ( 851) loc_os03g52840 12003.m101129 protein serine hydroxymethyltransferase, mitochondrial precursor, putative, expressed   no original description 	T
30.7	signalling.14-3-3 proteins	MDP0000166687	 moderately similar to ( 348) AT5G65430 | Symbols: GRF8, GF14 KAPPA | GRF8 (GENERAL REGULATORY FACTOR 8); protein binding / protein phosphorylated amino acid binding | chr5:26148546-26150255 REVERSEmoderately similar to ( 334) 1433D_TOBAC 14-3-3-like protein D - Nicotiana tabacum (Common tobacco)moderately similar to ( 313) loc_os08g33370 12008.m26501 protein 14-3-3-like protein, putative, expressed  no original description 	T
29.8	protein.assembly and cofactor ligation	MDP0000773593	 weakly similar to ( 130) AT4G04770 | Symbols: ATABC1, LAF6, ATNAP1 | ATABC1 (ATP BINDING CASSETTE PROTEIN 1); ATPase, coupled to transmembrane movement of substances / protein binding / transporter | chr4:2427998-2429785 REVERSEweakly similar to ( 103) loc_os01g61400 12001.m12266 protein expressed protein no original description 	T
16.2	secondary metabolism.phenylpropanoids	MDP0000242601	 highly similar to ( 584) AT1G27620 | Symbols:  | transferase family protein | chr1:9608444-9610432 FORWARDweakly similar to ( 169) HCBT1_DIACA Anthranilate N-benzoyltransferase protein 1 (EC 2.3.1.144) (Anthranilate N-hydroxycinnamoyl/benzoyltransferase 1) - Dianthus caryophyllus (Carnation) (Clove pink)moderately similar to ( 426) loc_os01g01520 12001.m06797 protein anthranilate N-benzoyltransferase protein 1, putative, expressed   no original description 	T
35.1	not assigned.no ontology	MDP0000249571	 highly similar to ( 699) AT4G38650 | Symbols:  | glycosyl hydrolase family 10 protein | chr4:18063377-18065769 FORWARDhighly similar to ( 659) loc_os03g14010 12003.m34818 protein hydrolase, hydrolyzing O-glycosyl compounds, putative, expressed   no original description 	T
29.5	protein.degradation	MDP0000168289	 highly similar to ( 642) AT4G20070 | Symbols: ATAAH | ATAAH (Arabidopsis thaliana Allantoate Amidohydrolase); allantoate deiminase/ metallopeptidase | chr4:10861548-10864529 FORWARDhighly similar to ( 546) loc_os06g45480 12006.m32139 protein metallopeptidase, putative, expressed  no original description 	T
15.2	metal handling.binding, chelation and storage	MDP0000230387	 highly similar to ( 550) AT4G35050 | Symbols: MSI3, NFC3 | MSI3 (MULTICOPY SUPPRESSOR OF IRA1 3); protein binding | chr4:16682752-16684751 REVERSEvery weakly similar to (65.5) COP1_PEA E3 ubiquitin ligase protein COP1 (EC 6.3.2.-) (Constitutive photomorphogenesis protein 1) - Pisum sativum (Garden pea)moderately similar to ( 422) loc_os03g43890 12003.m09430 protein WD-40 repeat protein MSI1, putative, expressed   no original description 	T
33.99	development.unspecified	MDP0000230387	 highly similar to ( 550) AT4G35050 | Symbols: MSI3, NFC3 | MSI3 (MULTICOPY SUPPRESSOR OF IRA1 3); protein binding | chr4:16682752-16684751 REVERSEvery weakly similar to (65.5) COP1_PEA E3 ubiquitin ligase protein COP1 (EC 6.3.2.-) (Constitutive photomorphogenesis protein 1) - Pisum sativum (Garden pea)moderately similar to ( 422) loc_os03g43890 12003.m09430 protein WD-40 repeat protein MSI1, putative, expressed   no original description 	T
33.99	development.unspecified	MDP0000125733	 highly similar to ( 826) AT5G58230 | Symbols: MSI1, MEE70, ATMSI1 | MSI1 (MULTICOPY SUPRESSOR OF IRA1); protein binding | chr5:23556112-23557994 FORWARDvery weakly similar to (66.2) COP1_PEA E3 ubiquitin ligase protein COP1 (EC 6.3.2.-) (Constitutive photomorphogenesis protein 1) - Pisum sativum (Garden pea)highly similar to ( 796) loc_os03g43890 12003.m09430 protein WD-40 repeat protein MSI1, putative, expressed  no original description 	T
20.2.3	stress.abiotic.drought/salt	MDP0000277077	 highly similar to ( 998) AT5G64030 | Symbols:  | dehydration-responsive protein-related | chr5:25624965-25628257 FORWARDweakly similar to ( 166) NFYB_MAIZE Nuclear transcription factor Y subunit B (NF-YB) (CAAT-box DNA-binding protein subunit B) - Zea mays (Maize)highly similar to ( 948) loc_os01g66110 12001.m42777 protein ankyrin-like protein, putative, expressed  TrbL PRK07003  no original description 	T
34.1	transport.p- and v-ATPases	MDP0000250887	 nearly identical (1852) AT1G17500 | Symbols:  | ATPase, coupled to transmembrane movement of ions, phosphorylative mechanism | chr1:6018757-6023201 FORWARDvery weakly similar to (76.3) ACA2_ORYSA Calcium-transporting ATPase 2, plasma membrane-type (EC 3.6.3.8) (Ca(2+)-ATPase isoform 2) - Oryza sativa (Rice)nearly identical (1820) loc_os06g36990 12006.m08234 protein phospholipid-transporting ATPase 4, putative, expressed  no original description 	T
29.4	protein.postranslational modification	MDP0000291397	 highly similar to ( 704) AT4G20360 | Symbols: ATRAB8D, ATRABE1B | ATRABE1B (ARABIDOPSIS RAB GTPASE HOMOLOG E1B); GTP binding / GTPase/ translation elongation factor | chr4:10990036-10991466 FORWARDhighly similar to ( 742) EFTU2_SOYBN Elongation factor Tu, chloroplast precursor (EF-Tu) - Glycine max (Soybean)highly similar to ( 652) loc_os02g38210 12002.m08909 protein elongation factor Tu, chloroplast precursor, putative, expressed   no original description 	T
33.2	development.late embryogenesis abundant	MDP0000850643	 weakly similar to ( 138) AT5G06760 | Symbols:  | late embryogenesis abundant group 1 domain-containing protein / LEA group 1 domain-containing protein | chr5:2089754-2090313 REVERSEweakly similar to ( 132) PM1_SOYBN 18 kDa seed maturation protein - Glycine max (Soybean)very weakly similar to (72.8) loc_os08g23870 12008.m06407 protein seed maturation protein, putative, expressed  no original description 	T
26.22	misc.short chain dehydrogenase/reductase (SDR)	MDP0000804812	 weakly similar to ( 127) AT4G23420 | Symbols:  | short-chain dehydrogenase/reductase (SDR) family protein | chr4:12226288-12228562 FORWARDvery weakly similar to (92.8) loc_os11g07922 12011.m04988 protein retinol dehydrogenase 12, putative, expressed no original description 	T
31.3.1	cell.cycle.peptidylprolyl isomerase	MDP0000304470	 moderately similar to ( 243) AT3G66654 | Symbols:  | peptidyl-prolyl cis-trans isomerase cyclophilin-type family protein | chr3:2088694-2090297 FORWARDvery weakly similar to (55.8) CYPB_VICFA Peptidyl-prolyl cis-trans isomerase, chloroplast precursor (EC 5.2.1.8) (PPIase) (Rotamase) (Cyclophilin) (Cyclosporin A-binding protein) (CYP B) - Vicia faba (Broad bean)moderately similar to ( 220) loc_os07g29390 12007.m66741 protein peptidyl-prolyl cis-trans isomerase CYP21-3, mitochondrial precursor, putative, expressed  no original description 	T
24.1	Biodegradation of Xenobiotics.hydroxyacylglutathione hydrolase	MDP0000283739	 moderately similar to ( 214) AT2G31350 | Symbols: GLX2-5 | GLX2-5 (GLYOXALASE 2-5); hydroxyacylglutathione hydrolase/ iron ion binding / zinc ion binding | chr2:13368451-13370802 FORWARDweakly similar to ( 196) loc_os09g34100 12009.m06459 protein hydroxyacylglutathione hydrolase 2, mitochondrial precursor, putative, expressed no original description 	T
35.2	not assigned.unknown	MDP0000870498	 moderately similar to ( 233) AT5G66230 | Symbols:  | unknown protein | chr5:26462704-26463873 FORWARDweakly similar to ( 150) loc_os10g42660 12010.m07019 protein expressed protein  no original description 	T
27.3.11	RNA.regulation of transcription.C2H2 zinc finger family	MDP0000573176	 weakly similar to ( 167) AT1G27730 | Symbols: STZ, ZAT10 | STZ (salt tolerance zinc finger); nucleic acid binding / transcription factor/ transcription repressor/ zinc ion binding | chr1:9648302-9648985 REVERSEweakly similar to ( 115) ZFP1_WHEAT Zinc-finger protein 1 (WZF1) - Triticum aestivum (Wheat)weakly similar to ( 130) loc_os12g39400 12012.m07725 protein zinc-finger protein 1, putative, expressed  no original description 	T
34.2	transport.sugars	MDP0000261679	 highly similar to ( 828) AT5G26340 | Symbols: MSS1, STP13 | MSS1; carbohydrate transmembrane transporter/ hexose:hydrogen symporter/ high-affinity hydrogen:glucose symporter/ sugar:hydrogen symporter | chr5:9243851-9246994 REVERSEhighly similar to ( 577) STC_RICCO Sugar carrier protein C - Ricinus communis (Castor bean)highly similar to ( 788) loc_os03g11900 12003.m06642 protein sugar transport protein 8, putative, expressed   no original description 	T
33.99	development.unspecified	MDP0000285251	 highly similar to ( 928) AT2G32940 | Symbols: AGO6 | AGO6 (ARGONAUTE 6); nucleic acid binding | chr2:13972218-13976856 REVERSEmoderately similar to ( 313) ZFNL_PEA Zinc finger CCCH domain-containing protein ZFN-like - Pisum sativum (Garden pea)highly similar to ( 874) loc_os01g16870 12001.m150417 protein argonaute-like protein, putative, expressed   no original description 	T
26.7	misc.oxidases - copper, flavone etc	MDP0000459304	 highly similar to ( 847) AT1G76160 | Symbols: sks5 | sks5 (SKU5 Similar 5); copper ion binding / oxidoreductase | chr1:28578211-28581020 REVERSEhighly similar to ( 551) ASO_BRANA L-ascorbate oxidase homolog precursor (EC 1.10.3.3) (Ascorbase) - Brassica napus (Rape)highly similar to ( 740) loc_os07g32660 12007.m07529 protein L-ascorbate oxidase homolog precursor, putative, expressed   no original description 	T
29.5.11.4.3.2	protein.degradation.ubiquitin.E3.SCF.FBOX	MDP0000299479	 highly similar to ( 555) AT1G10780 | Symbols:  | F-box family protein | chr1:3592875-3594310 REVERSEmoderately similar to ( 480) loc_os09g28120 12009.m05963 protein F-box domain containing protein, expressed  no original description 	T
29.5	protein.degradation	MDP0000292636	 highly similar to ( 990) AT4G33090 | Symbols: APM1, ATAPM1 | APM1 (AMINOPEPTIDASE M1); aminopeptidase | chr4:15965915-15970418 REVERSEhighly similar to ( 912) loc_os02g12650 12002.m33362 protein puromycin-sensitive aminopeptidase, putative, expressed   no original description 	T
29.2.1.1.1.1.19	protein.synthesis.ribosomal protein.prokaryotic.chloroplast.30S subunit.S19	MDP0000930925	 very weakly similar to (89.4) AT5G47320 | Symbols: RPS19 | RPS19 (RIBOSOMAL PROTEIN S19); RNA binding | chr5:19203801-19204951 FORWARDvery weakly similar to (92.8) RT19_PETHY Mitochondrial ribosomal protein S19 - Petunia hybrida (Petunia)very weakly similar to (87.4) loc_osm1g00450 11706.m00038 protein Ribosomal protein S19 no original description 	T
26.2	misc.UDP glucosyl and glucoronyl transferases	MDP0000241559	 moderately similar to ( 290) AT3G45400 | Symbols:  | exostosin family protein | chr3:16651963-16653479 FORWARDmoderately similar to ( 251) loc_os12g03100 12012.m04308 protein secondary cell wall-related glycosyltransferase family 47, putative, expressed  no original description 	T
29.5.11.3	protein.degradation.ubiquitin.E2	MDP0000272292	 moderately similar to ( 314) AT2G02760 | Symbols: ATUBC2, UBC2 | ATUBC2 (UBIQUITING-CONJUGATING ENZYME 2); ubiquitin-protein ligase | chr2:774271-775149 FORWARDmoderately similar to ( 314) UBC2_MEDSA Ubiquitin-conjugating enzyme E2-17 kDa (EC 6.3.2.19) (Ubiquitin-protein ligase) (Ubiquitin carrier protein) - Medicago sativa (Alfalfa)moderately similar to ( 315) loc_os07g07240 12007.m05183 protein ubiquitin-conjugating enzyme E2-17 kDa, putative, expressed no original description 	T
26.17	misc.dynamin	MDP0000217322	 moderately similar to ( 294) AT1G60500 | Symbols:  | dynamin family protein | chr1:22291582-22293822 FORWARDmoderately similar to ( 253) loc_os03g15420 12003.m06979 protein GTP binding protein, putative, expressed  no original description 	T
30.2.17	signalling.receptor kinases.DUF 26	MDP0000319460	 moderately similar to ( 335) AT5G38260 | Symbols:  | serine/threonine protein kinase, putative | chr5:15283692-15285837 REVERSEweakly similar to ( 182) KPRO_MAIZE Putative receptor protein kinase ZmPK1 precursor (EC 2.7.11.1) - Zea mays (Maize)moderately similar to ( 402) loc_os01g02800 12001.m06920 protein receptor-like kinase ARK1AS, putative, expressed  no original description 	T
20.2.1	stress.abiotic.heat	MDP0000222555	 weakly similar to ( 121) AT1G80920 | Symbols: J8 | J8; heat shock protein binding / unfolded protein binding | chr1:30403863-30404549 REVERSEvery weakly similar to (62.4) loc_os06g44160 12006.m091751 protein dnaJ protein, putative, expressed no original description 	T
35.2	not assigned.unknown	MDP0000250688	 nearly identical (1053) AT4G13750 | Symbols:  | ATP binding | chr4:7975191-7987558 FORWARDhighly similar to ( 938) loc_os03g58910 12003.m35467 protein expressed protein   no original description 	T
29.5.11.4.3.2	protein.degradation.ubiquitin.E3.SCF.FBOX	MDP0000122624	 nearly identical (1059) AT4G33210 | Symbols:  | F-box family protein (FBL15) | chr4:16015971-16020697 REVERSEhighly similar to ( 995) loc_os10g25680 12010.m05512 protein ubiquitin-protein ligase, putative, expressed   no original description 	T
35.1.42	not assigned.no ontology.proline rich family	MDP0000322632	 weakly similar to ( 149) AT2G16630 | Symbols:  | proline-rich family protein | chr2:7209286-7210872 FORWARDweakly similar to ( 108) loc_os02g01190 12002.m05469 protein expressed protein   no original description 	T
35.2	not assigned.unknown	MDP0000119550	 weakly similar to ( 164) AT1G15215 | Symbols:  | FUNCTIONS IN: molecular_function unknown; INVOLVED IN: biological_process unknown; LOCATED IN: cellular_component unknown; BEST Arabidopsis thaliana protein match is: sequence-specific DNA binding / transcription factor (TAIR:AT3G18380.2); Has 41 Blast hits to 40 proteins in 9 species: Archae - 0; Bacteria - 0; Metazoa - 0; Fungi - 0; Plants - 41; Viruses - 0; Other Eukaryotes - 0 (source: NCBI BLink). | chr1:5238096-5239741 FORWARDweakly similar to ( 158) loc_os09g17770 12009.m22071 protein retrotransposon protein, putative, unclassified, expressed no original description 	T
35.2	not assigned.unknown	MDP0000185950	   no original description 	T
35.2	not assigned.unknown	MDP0000280386	 moderately similar to ( 446) AT4G14740 | Symbols:  | phosphoinositide binding | chr4:8450567-8452679 REVERSEmoderately similar to ( 382) loc_os03g43510 12003.m09397 protein expressed protein   no original description 	T
35.2	not assigned.unknown	MDP0000172895	  no original description 	T
11.9.3.2	lipid metabolism.lipid degradation.lysophospholipases.carboxylesterase	MDP0000255292	 moderately similar to ( 353) AT4G22300 | Symbols: SOBER1 | SOBER1 (SUPPRESSOR OF AVRBST-ELICITED RESISTANCE 1); carboxylesterase | chr4:11787560-11791055 REVERSEmoderately similar to ( 352) loc_os04g57370 12004.m10622 protein acyl-protein thioesterase 1, putative, expressed   no original description 	T
35.2	not assigned.unknown	MDP0000192000	 moderately similar to ( 209) AT1G55340 | Symbols:  | unknown protein | chr1:20652605-20653469 FORWARDweakly similar to ( 160) loc_os03g59250 12003.m10824 protein expressed protein  no original description 	T
30.2.11	signalling.receptor kinases.leucine rich repeat XI	MDP0000173845	 moderately similar to ( 404) AT1G72300 | Symbols:  | leucine-rich repeat transmembrane protein kinase, putative | chr1:27217679-27220966 REVERSEmoderately similar to ( 276) PSKR_DAUCA Phytosulfokine receptor precursor (EC 2.7.11.1) (Phytosulfokine LRR receptor kinase) - Daucus carota (Carrot)moderately similar to ( 373) loc_os06g47760 12006.m09302 protein phytosulfokine receptor precursor, putative, expressed  no original description 	T
30.2.3	signalling.receptor kinases.leucine rich repeat III	MDP0000741253	 highly similar to ( 582) AT5G58300 | Symbols:  | leucine-rich repeat transmembrane protein kinase, putative | chr5:23572821-23574871 FORWARDweakly similar to ( 164) PSKR_DAUCA Phytosulfokine receptor precursor (EC 2.7.11.1) (Phytosulfokine LRR receptor kinase) - Daucus carota (Carrot)highly similar to ( 546) loc_os01g60330 12001.m12161 protein ATP binding protein, putative, expressed    no original description 	T
27.3.22	RNA.regulation of transcription.HB,Homeobox transcription factor family	MDP0000233235	 highly similar to ( 902) AT1G79840 | Symbols: GL2 | GL2 (GLABRA 2); DNA binding / transcription factor | chr1:30037526-30041013 FORWARDhighly similar to ( 669) loc_os01g55549 12001.m150954 protein homeobox protein GLABRA2, putative, expressed    no original description 	T
26.4	misc.beta 1,3 glucan hydrolases	MDP0000323489	 weakly similar to ( 117) AT1G18650 | Symbols: PDCB3 | PDCB3 (PLASMODESMATA CALLOSE-BINDING PROTEIN 3); callose binding / polysaccharide binding | chr1:6419036-6420413 REVERSEvery weakly similar to (74.7) E13B_WHEAT Glucan endo-1,3-beta-glucosidase precursor (EC 3.2.1.39) ((1->3)-beta-glucan endohydrolase) ((1->3)-beta-glucanase) (Beta-1,3-endoglucanase) - Triticum aestivum (Wheat)weakly similar to ( 102) loc_os03g54910 12003.m10406 protein glucan endo-1,3-beta-glucosidase 3 precursor, putative, expressed no original description 	T
1.3.13	PS.calvin cycle.rubisco interacting	MDP0000321244	 highly similar to ( 731) AT2G39730 | Symbols: RCA | RCA (RUBISCO ACTIVASE); ADP binding / ATP binding / enzyme regulator/ ribulose-1,5-bisphosphate carboxylase/oxygenase activator | chr2:16571174-16573345 REVERSEhighly similar to ( 839) RCA_MALDO Ribulose bisphosphate carboxylase/oxygenase activase, chloroplast precursor (RuBisCO activase) (RA) - Malus domestica (Apple) (Malus sylvestris)highly similar to ( 721) loc_os11g47970 12011.m28706 protein ribulose bisphosphate carboxylase/oxygenase activase, chloroplast precursor, putative, expressed MSP1_C  no original description 	T
29.2.1.2.1.5	protein.synthesis.ribosomal protein.eukaryotic.40S subunit.S5	MDP0000273575	 moderately similar to ( 339) AT2G37270 | Symbols: ATRPS5B | ATRPS5B (RIBOSOMAL PROTEIN 5B); structural constituent of ribosome | chr2:15647883-15649042 REVERSEmoderately similar to ( 346) RS5_CICAR 40S ribosomal protein S5 (Fragment) - Cicer arietinum (Chickpea) (Garbanzo)moderately similar to ( 336) loc_os11g29190 12011.m080000 protein 40S ribosomal protein S5, putative, expressed no original description 	T
33.99	development.unspecified	MDP0000296113	 moderately similar to ( 343) AT3G55770 | Symbols:  | LIM domain-containing protein | chr3:20703799-20704614 FORWARDmoderately similar to ( 216) SF3_HELAN Pollen-specific protein SF3 - Helianthus annuus (Common sunflower)moderately similar to ( 325) loc_os03g15940 12003.m07031 protein pollen-specific protein SF3, putative, expressed  no original description 	T
4.1.6	glycolysis.cytosolic branch.fructose-2,6-bisphosphatase (Fru2,6BisPase)	MDP0000317977	 nearly identical (1086) AT1G07110 | Symbols: F2KP, ATF2KP, FKFBP | F2KP (FRUCTOSE-2,6-BISPHOSPHATASE); fructose-2,6-bisphosphate 2-phosphatase | chr1:2178363-2183980 REVERSEnearly identical (1001) loc_os05g07130 12005.m05239 protein fructose-6-phosphate-2-kinase/fructose-2, 6-bisphosphatase, putative, expressed no original description 	T
31.2	cell.division	MDP0000321176	 moderately similar to ( 498) AT5G55280 | Symbols: FTSZ1-1, ATFTSZ1-1, CPFTSZ | FTSZ1-1; protein binding / structural molecule | chr5:22420740-22422527 REVERSEmoderately similar to ( 473) loc_os04g56970 12004.m35221 protein cell division protein ftsZ homolog, chloroplast precursor, putative, expressed no original description 	T
35.2	not assigned.unknown	MDP0000692817	 moderately similar to ( 438) AT3G55350 | Symbols:  | unknown protein | chr3:20518518-20520690 FORWARDmoderately similar to ( 425) loc_os11g47650 12011.m08565 protein transposon protein, putative, CACTA, En/Spm sub-class, expressed  no original description 	T
26.2	misc.UDP glucosyl and glucoronyl transferases	MDP0000203016	 weakly similar to ( 168) AT2G30150 | Symbols:  | UDP-glucoronosyl/UDP-glucosyl transferase family protein | chr2:12874706-12876122 FORWARDweakly similar to ( 144) loc_os01g59110 12001.m12049 protein indole-3-acetate beta-glucosyltransferase, putative, expressed no original description 	T
35.2	not assigned.unknown	MDP0000146053	 moderately similar to ( 468) AT3G13990 | Symbols:  | unknown protein | chr3:4625708-4630383 REVERSEmoderately similar to ( 288) loc_os03g29750 12003.m08241 protein expressed protein   no original description 	T
28.1	DNA.synthesis/chromatin structure	MDP0000278398	 moderately similar to ( 209) AT5G49010 | Symbols: SLD5 | SLD5 (SYNTHETIC LETHALITY WITH DPB11-1 5) | chr5:19866464-19867762 FORWARDweakly similar to ( 198) loc_os05g05150 12005.m05042 protein SLD5, putative, expressed no original description 	T
35.2	not assigned.unknown	MDP0000464528	 moderately similar to ( 325) AT3G52610 | Symbols:  | unknown protein | chr3:19510513-19512547 FORWARDmoderately similar to ( 309) loc_os01g64256 12001.m42538 protein transposon protein, putative, Mutator sub-class, expressed   no original description 	T
1.1.2.2	PS.lightreaction.photosystem I.PSI polypeptide subunits	MDP0000137271	 weakly similar to ( 179) AT5G64040 | Symbols: PSAN | PSAN; calmodulin binding | chr5:25628690-25629409 REVERSEweakly similar to ( 155) PSAN_MAIZE Photosystem I reaction center subunit N, chloroplast precursor (PSI-N) (Fragment) - Zea mays (Maize)weakly similar to ( 157) loc_os12g08770 12012.m04863 protein photosystem I reaction center subunit N, chloroplast precursor, putative, expressed  no original description 	T
34.13	transport.peptides and oligopeptides	MDP0000278025	 moderately similar to ( 330) AT5G01230 | Symbols:  | FtsJ-like methyltransferase family protein | chr5:92789-95380 REVERSEmoderately similar to ( 333) loc_os06g49140 12006.m32178 protein ribosomal RNA methyltransferase 1, putative, expressed   TrbL  no original description 	T
16.2	secondary metabolism.phenylpropanoids	MDP0000168790	 moderately similar to ( 373) AT5G54160 | Symbols: ATOMT1, OMT1 | ATOMT1 (O-METHYLTRANSFERASE 1); caffeate O-methyltransferase/ myricetin 3'-O-methyltransferase/ quercetin 3-O-methyltransferase | chr5:21982075-21984167 FORWARDmoderately similar to ( 387) COMT1_CATRO Caffeic acid 3-O-methyltransferase (EC 2.1.1.68) (S-adenosysl-L-methionine:caffeic acid 3-O-methyltransferase) (COMT) (CAOMT) - Catharanthus roseus (Rosy periwinkle) (Madagascar periwinkle)moderately similar to ( 341) loc_os08g06100 12008.m04749 protein quercetin 3-O-methyltransferase 1, putative, expressed  no original description 	T
23.1.2.7	nucleotide metabolism.synthesis.purine.SAICAR synthetase	MDP0000294564	 moderately similar to ( 476) AT3G21110 | Symbols: PUR7, PURC | PUR7 (PURIN 7); phosphoribosylaminoimidazolesuccinocarboxamide synthase | chr3:7402696-7405273 REVERSEmoderately similar to ( 458) loc_os09g29190 12009.m22124 protein phosphoribosylaminoimidazole-succinocarboxamide synthase, chloroplast precursor, putative, expressed   no original description 	T
35.2	not assigned.unknown	MDP0000286353	 moderately similar to ( 235) AT5G23550 | Symbols:  | FUNCTIONS IN: molecular_function unknown; INVOLVED IN: biological_process unknown; LOCATED IN: cellular_component unknown; EXPRESSED IN: 22 plant structures; EXPRESSED DURING: 13 growth stages; CONTAINS InterPro DOMAIN/s: SFT2-like (InterPro:IPR011691); BEST Arabidopsis thaliana protein match is: unknown protein (TAIR:AT5G24170.1); Has 455 Blast hits to 455 proteins in 131 species: Archae - 0; Bacteria - 0; Metazoa - 195; Fungi - 96; Plants - 62; Viruses - 0; Other Eukaryotes - 102 (source: NCBI BLink). | chr5:7939914-7941479 REVERSEmoderately similar to ( 220) loc_os02g35640 12002.m08652 protein pRGR1, putative, expressed no original description 	T
26.17	misc.dynamin	MDP0000179659	 highly similar to ( 507) AT1G60500 | Symbols:  | dynamin family protein | chr1:22291582-22293822 FORWARDmoderately similar to ( 468) loc_os03g15420 12003.m06979 protein GTP binding protein, putative, expressed  no original description 	T
11.2.1	lipid metabolism.FA desaturation.desaturase	MDP0000240315	 moderately similar to ( 465) AT3G15850 | Symbols: FAD5, FADB, JB67, ADS3 | FAD5 (FATTY ACID DESATURASE 5); 16:0 monogalactosyldiacylglycerol desaturase/ oxidoreductase | chr3:5359087-5360998 FORWARDvery weakly similar to (79.0) HP1_ORYSA Histidine-containing phosphotransfer protein 1 (OsHP1) - Oryza sativa (Rice)very weakly similar to (86.3) loc_os01g54050 12001.m11569 protein histidine-containing phosphotransfer protein 4, putative, expressed  no original description 	T
29.5.1	protein.degradation.subtilases	MDP0000585315	 highly similar to ( 744) AT2G05920 | Symbols:  | subtilase family protein | chr2:2269831-2272207 REVERSEhighly similar to ( 590) loc_os03g55350 12003.m10450 protein subtilisin-like protease precursor, putative, expressed   no original description 	T
20.1.7	stress.biotic.PR-proteins	MDP0000214225	 moderately similar to ( 431) AT5G66900 | Symbols:  | disease resistance protein (CC-NBS-LRR class), putative | chr5:26714931-26717757 REVERSEmoderately similar to ( 323) loc_os12g39620 12012.m073872 protein disease resistance protein, putative, expressed  no original description 	T
29.5.11.20	protein.degradation.ubiquitin.proteasom	MDP0000419582	 moderately similar to ( 425) AT3G22110 | Symbols: PAC1 | PAC1; endopeptidase/ peptidase/ threonine-type endopeptidase | chr3:7792819-7793571 REVERSEmoderately similar to ( 436) PSA4_SPIOL Proteasome subunit alpha type 4 (EC 3.4.25.1) (20S proteasome alpha subunit C) (20S proteasome subunit alpha-3) (Proteasome 27 kDa subunit) - Spinacia oleracea (Spinach)moderately similar to ( 400) loc_os06g07140 12006.m05438 protein proteasome subunit alpha type 4, putative, expressed  no original description 	T
35.2	not assigned.unknown	MDP0000188209	    no original description 	T
35.2	not assigned.unknown	MDP0000176769	 moderately similar to ( 499) AT3G14870 | Symbols:  | unknown protein | chr3:5004040-5005586 FORWARDmoderately similar to ( 312) loc_os10g23220 12010.m21871 protein expressed protein  no original description 	T
10.2	cell wall.cellulose synthesis	MDP0000572242	 highly similar to ( 969) AT4G07960 | Symbols: ATCSLC12, CSLC12 | ATCSLC12 (CELLULOSE-SYNTHASE LIKE C12); cellulose synthase/ transferase, transferring glycosyl groups | chr4:4802628-4805114 REVERSEhighly similar to ( 873) loc_os05g43530 12005.m08480 protein CSLC7 - cellulose synthase-like family C, expressed  COG4907  no original description 	T
27.3.3	RNA.regulation of transcription.AP2/EREBP, APETALA2/Ethylene-responsive element binding protein family	MDP0000124316	 weakly similar to ( 197) AT1G15360 | Symbols: SHN1, WIN1 | SHN1 (SHINE 1); DNA binding / sequence-specific DNA binding / transcription factor | chr1:5283609-5284506 FORWARDvery weakly similar to (64.7) ERF4_TOBAC Ethylene-responsive transcription factor 4 (Ethylene-responsive element-binding factor 4 homolog) (EREBP-3) (NtERF3) - Nicotiana tabacum (Common tobacco)weakly similar to ( 193) loc_os02g10760 12002.m06374 protein ethylene responsive element, putative, expressed  no original description 	T
16.2.1.3	secondary metabolism.phenylpropanoids.lignin biosynthesis.4CL	MDP0000691789	 highly similar to ( 724) AT1G65060 | Symbols: 4CL3 | 4CL3; 4-coumarate-CoA ligase | chr1:24167927-24171457 REVERSEhighly similar to ( 815) 4CL2_SOYBN 4-coumarate--CoA ligase 2 (EC 6.2.1.12) (4CL 2) (4-coumaroyl-CoA synthase 2) (Clone 4CL16) - Glycine max (Soybean)highly similar to ( 701) loc_os02g46970 12002.m09733 protein 4-coumarate--CoA ligase 2, putative, expressed   no original description 	T
35.2	not assigned.unknown	MDP0000250382	 weakly similar to ( 154) AT1G79770 | Symbols:  | unknown protein | chr1:30014371-30014874 FORWARDweakly similar to ( 143) loc_os06g36040 12006.m08141 protein expressed protein  no original description 	T
27.3.80	RNA.regulation of transcription.zf-HD	MDP0000277477	 weakly similar to ( 126) AT1G75240 | Symbols: AtHB33 | AtHB33 (ARABIDOPSIS THALIANA HOMEOBOX PROTEIN 33); DNA binding / transcription factor | chr1:28241576-28242505 FORWARDweakly similar to ( 145) loc_os03g50920 12003.m10076 protein ZF-HD protein dimerisation region containing protein, expressed   no original description 	T
10.6.2	cell wall.degradation.mannan-xylose-arabinose-fucose	MDP0000140483	 nearly identical (1113) AT5G49360 | Symbols: BXL1, ATBXL1 | BXL1 (BETA-XYLOSIDASE 1); hydrolase, hydrolyzing O-glycosyl compounds | chr5:20012179-20016659 REVERSEhighly similar to ( 769) XYNB_PRUPE Putative beta-D-xylosidase (EC 3.2.1.-) (PpAz152) (Fragment) - Prunus persica (Peach)highly similar to ( 882) loc_os04g54810 12004.m10373 protein beta-D-xylosidase, putative, expressed   no original description 	T
35.2	not assigned.unknown	MDP0000119925	 weakly similar to ( 145) AT3G22290 | Symbols:  | unknown protein | chr3:7882082-7885260 FORWARDweakly similar to ( 127) loc_os03g57100 12003.m10630 protein serologically defined breast cancer antigen NY-BR-84, putative, expressed no original description 	T
15.2	metal handling.binding, chelation and storage	MDP0000325376	 weakly similar to ( 194) AT1G71050 | Symbols:  | heavy-metal-associated domain-containing protein / copper chaperone (CCH)-related | chr1:26803322-26803946 REVERSEweakly similar to ( 171) loc_os01g32330 12001.m09558 protein farnesylated protein 2, putative, expressed  no original description 	T
29.4	protein.postranslational modification	MDP0000095375	 moderately similar to ( 412) AT2G44680 | Symbols: CKB4 | CKB4 (CASEIN KINASE II BETA SUBUNIT 4); protein serine/threonine kinase | chr2:18426898-18428166 REVERSEmoderately similar to ( 404) loc_os07g31280 12007.m29180 protein casein kinase II subunit beta-4, putative, expressed   no original description 	T
35.2	not assigned.unknown	MDP0000252969	 moderately similar to ( 276) AT3G18050 | Symbols:  | unknown protein | chr3:6180931-6182593 FORWARDweakly similar to ( 152) loc_os07g37156 12007.m79903 protein expressed protein   no original description 	T
21.4	redox.glutaredoxins	MDP0000376239	 weakly similar to ( 176) AT5G18600 | Symbols:  | glutaredoxin family protein | chr5:6183466-6183774 REVERSEvery weakly similar to (70.5) GLRX_RICCO Glutaredoxin - Ricinus communis (Castor bean)weakly similar to ( 128) loc_os12g35330 12012.m07326 protein OsGrx_C12 - glutaredoxin subgroup III no original description 	T
30.3	signalling.calcium	MDP0000464941	 moderately similar to ( 298) AT5G62390 | Symbols: ATBAG7 | ATBAG7 (ARABIDOPSIS THALIANA BCL-2-ASSOCIATED ATHANOGENE 7); calmodulin binding | chr5:25052377-25054170 REVERSEweakly similar to ( 189) loc_os06g01500 12006.m31819 protein IQ calmodulin-binding motif family protein, expressed    no original description 	T
27.3.37	RNA.regulation of transcription.AS2,Lateral Organ Boundaries Gene Family	MDP0000220708	 weakly similar to ( 154) AT3G11090 | Symbols: LBD21 | LBD21 (LOB DOMAIN-CONTAINING PROTEIN 21) | chr3:3475036-3475533 FORWARDweakly similar to ( 130) loc_os05g27980 12005.m07086 protein LOB domain protein 12, putative, expressed  no original description 	T
35.1	not assigned.no ontology	MDP0000269554	 nearly identical (1255) AT3G01380 | Symbols:  | catalytic/ transferase | chr3:144288-149828 REVERSEhighly similar to ( 566) loc_os02g37050 12002.m08793 protein phosphatidylinositolglycan class N family protein, expressed   no original description 	T
3.5	minor CHO metabolism.others	MDP0000323702	 moderately similar to ( 441) AT2G37770 | Symbols:  | aldo/keto reductase family protein | chr2:15834888-15836659 FORWARDmoderately similar to ( 244) ALDR_HORVU Aldose reductase (EC 1.1.1.21) (AR) (Aldehyde reductase) - Hordeum vulgare (Barley)moderately similar to ( 410) loc_os01g62860 12001.m12406 protein aldose reductase, putative, expressed  no original description 	T
35.2	not assigned.unknown	MDP0000126011	 highly similar to ( 534) AT1G27595 | Symbols:  | EXPRESSED IN: 22 plant structures; EXPRESSED DURING: 15 growth stages; BEST Arabidopsis thaliana protein match is: ESP4 (ENHANCED SILENCING PHENOTYPE 4); binding (TAIR:AT5G01400.1); Has 529 Blast hits to 235 proteins in 85 species: Archae - 0; Bacteria - 3; Metazoa - 125; Fungi - 41; Plants - 31; Viruses - 0; Other Eukaryotes - 329 (source: NCBI BLink). | chr1:9596475-9602917 FORWARDmoderately similar to ( 463) loc_os01g49940 12001.m11179 protein expressed protein no original description 	T
13.1.1.2.1	amino acid metabolism.synthesis.central amino acid metabolism.aspartate.aspartate aminotransferase	MDP0000292164	 highly similar to ( 720) AT2G30970 | Symbols: ASP1 | ASP1 (ASPARTATE AMINOTRANSFERASE 1); L-aspartate:2-oxoglutarate aminotransferase | chr2:13179012-13181686 FORWARDmoderately similar to ( 407) AAT1_MEDSA Aspartate aminotransferase 1 (EC 2.6.1.1) (Transaminase A) - Medicago sativa (Alfalfa)highly similar to ( 731) loc_os06g35540 12006.m08091 protein aspartate aminotransferase, mitochondrial precursor, putative, expressed  no original description 	T
20.1	stress.biotic	MDP0000522745	 moderately similar to ( 242) AT5G66900 | Symbols:  | disease resistance protein (CC-NBS-LRR class), putative | chr5:26714931-26717757 REVERSEweakly similar to ( 132) loc_os12g39620 12012.m073872 protein disease resistance protein, putative, expressed  no original description 	T
26.10	misc.cytochrome P450	MDP0000194814	 highly similar to ( 560) AT3G56630 | Symbols: CYP94D2 | CYP94D2; electron carrier/ heme binding / iron ion binding / monooxygenase/ oxygen binding | chr3:20978953-20980512 FORWARDvery weakly similar to (84.3) C71DA_SOYBN Cytochrome P450 71D10 (EC 1.14.-.-) - Glycine max (Soybean)highly similar to ( 501) loc_os01g58990 12001.m12038 protein cytochrome P450 94A2, putative   no original description 	T
30.3	signalling.calcium	MDP0000293580	 highly similar to ( 955) AT2G43040 | Symbols: NPG1 | NPG1 (no pollen germination 1); calmodulin binding | chr2:17896049-17898524 REVERSEhighly similar to ( 847) loc_os12g37780 12012.m07564 protein calmodulin-binding protein MPCBP, putative, expressed  no original description 	T
27.3.3	RNA.regulation of transcription.AP2/EREBP, APETALA2/Ethylene-responsive element binding protein family	MDP0000137561	 moderately similar to ( 326) AT4G36920 | Symbols: AP2, FLO2, FL1 | AP2 (APETALA 2); transcription factor | chr4:17400998-17403140 FORWARDweakly similar to ( 139) BBM2_BRANA Protein BABY BOOM 2 (BnBBM2) - Brassica napus (Rape)moderately similar to ( 319) loc_os04g55560 12004.m35488 protein AP2 domain containing protein, expressed  no original description 	T
35.2	not assigned.unknown	MDP0000578300	 highly similar to ( 504) AT1G09980 | Symbols:  | INVOLVED IN: biological_process unknown; LOCATED IN: mitochondrion; EXPRESSED IN: 22 plant structures; EXPRESSED DURING: 13 growth stages; CONTAINS InterPro DOMAIN/s: Protein of unknown function DUF676, hydrolase-like (InterPro:IPR007751); BEST Arabidopsis thaliana protein match is: ZW18 (TAIR:AT1G58350.2); Has 342 Blast hits to 275 proteins in 70 species: Archae - 0; Bacteria - 0; Metazoa - 214; Fungi - 0; Plants - 45; Viruses - 0; Other Eukaryotes - 83 (source: NCBI BLink). | chr1:3256541-3260866 REVERSEmoderately similar to ( 448) loc_os12g06800 12012.m04670 protein serine esterase family protein, putative, expressed  no original description 	T
35.2	not assigned.unknown	MDP0000549333	 nearly identical (1694) AT3G48770 | Symbols:  | ATP binding / DNA binding | chr3:18079261-18086817 REVERSEnearly identical (1503) loc_os11g31480 12011.m07051 protein DNA binding protein, putative, expressed  no original description 	T
26.7	misc.oxidases - copper, flavone etc	MDP0000202087	 moderately similar to ( 426) AT5G16990 | Symbols:  | NADP-dependent oxidoreductase, putative | chr5:5581831-5583849 REVERSEmoderately similar to ( 392) loc_os12g12580 12012.m05231 protein NADP-dependent oxidoreductase P2, putative, expressed  no original description 	T
33.99	development.unspecified	MDP0000798833	 very weakly similar to (60.8) AT3G44735 | Symbols: PSK1, ATPSK3 | PSK1; growth factor | chr3:16291388-16292118 REVERSEvery weakly similar to (50.8) PSK5_ORYSA Phytosulfokines 5 precursor (Secretory protein SH27A) [Contains: Phytosulfokine-alpha (PSK-alpha) (Phytosulfokine-a); Phytosulfokine-beta (PSK-beta) (Phytosulfokine-b)] - Oryza sativa (Rice)very weakly similar to (50.8) loc_os12g05260 12012.m04520 protein phytosulfokines 5 precursor, putative, expressed no original description 	T
35.2	not assigned.unknown	MDP0000482871	 moderately similar to ( 249) AT4G16144 | Symbols:  | FUNCTIONS IN: molecular_function unknown; INVOLVED IN: biological_process unknown; LOCATED IN: cellular_component unknown; CONTAINS InterPro DOMAIN/s: Mov34/MPN/PAD-1 (InterPro:IPR000555); BEST Arabidopsis thaliana protein match is: mov34 family protein (TAIR:AT1G48790.1); Has 622 Blast hits to 459 proteins in 132 species: Archae - 0; Bacteria - 0; Metazoa - 326; Fungi - 156; Plants - 82; Viruses - 0; Other Eukaryotes - 58 (source: NCBI BLink). | chr4:9138053-9141422 REVERSEweakly similar to ( 149) loc_os01g23640 12001.m42998 protein mov34/MPN/PAD-1 family protein, expressed   no original description 	T
28.1.3	DNA.synthesis/chromatin structure.histone	MDP0000258816	 moderately similar to ( 266) AT5G10980 | Symbols:  | histone H3 | chr5:3472591-3473349 REVERSEmoderately similar to ( 264) H33_VITVI Histone H3.3 - Vitis vinifera (Grape)moderately similar to ( 266) loc_os06g04030 12006.m05134 protein histone H3, putative, expressed no original description 	T
29.2.1.2.1.11	protein.synthesis.ribosomal protein.eukaryotic.40S subunit.S11	MDP0000325964	 moderately similar to ( 270) AT5G23740 | Symbols: RPS11-BETA | RPS11-BETA (RIBOSOMAL PROTEIN S11-BETA); structural constituent of ribosome | chr5:8008251-8009330 REVERSEmoderately similar to ( 273) RS11_SOYBN 40S ribosomal protein S11 - Glycine max (Soybean)moderately similar to ( 270) loc_os07g37780 12007.m08027 protein 40S ribosomal protein S11, putative, expressed  no original description 	T
31.1	cell.organisation	MDP0000153650	 highly similar to ( 882) AT3G61650 | Symbols: TUBG1 | TUBG1 (GAMMA-TUBULIN); GTP binding / GTPase/ structural molecule | chr3:22812601-22815011 REVERSEhighly similar to ( 848) TBG_PHYPA Tubulin gamma chain (Gamma tubulin) - Physcomitrella patens (Moss)highly similar to ( 841) loc_os05g06450 12005.m27549 protein tubulin gamma-2 chain, putative, expressed  no original description 	T
35.2	not assigned.unknown	MDP0000274266	 weakly similar to ( 114) AT3G12410 | Symbols:  | 3'-5' exonuclease/ nucleic acid binding | chr3:3946267-3946959 REVERSEweakly similar to ( 184) loc_os04g14810 12004.m06717 protein Werner syndrome ATP-dependent helicase, putative, expressed  no original description 	T
13.2.5.2	amino acid metabolism.degradation.serine-glycine-cysteine group.glycine	MDP0000282696	 moderately similar to ( 437) AT1G08630 | Symbols: THA1 | THA1 (Threonine Aldolase 1); aldehyde-lyase/ threonine aldolase | chr1:2743948-2745685 REVERSEmoderately similar to ( 431) loc_os04g43650 12004.m101586 protein L-allo-threonine aldolase, putative, expressed   no original description 	T
35.1.12	not assigned.no ontology.pumilio/Puf RNA-binding domain-containing protein	MDP0000264307	 moderately similar to ( 458) AT5G56510 | Symbols: APUM12 | APUM12 (ARABIDOPSIS PUMILIO 12); RNA binding / binding | chr5:22881721-22883842 FORWARDmoderately similar to ( 381) loc_os07g07540 12007.m28951 protein SHOOT1 protein, putative, expressed  no original description 	T
29.3.4.99	protein.targeting.secretory pathway.unspecified	MDP0000321041	 highly similar to ( 879) AT2G34250 | Symbols:  | protein transport protein sec61, putative | chr2:14462635-14464572 FORWARDhighly similar to ( 891) loc_os09g17840 12009.m05042 protein protein transport protein Sec61 alpha subunit isoform 2, putative, expressed  no original description 	T
34.12	transport.metal	MDP0000196606	 highly similar to ( 717) AT2G47600 | Symbols: ATMHX, MHX1, ATMHX1 | ATMHX (ARABIDOPSIS THALIANA MAGNESIUM/PROTON EXCHANGER); calcium:sodium antiporter/ cation:cation antiporter | chr2:19524518-19526828 REVERSEhighly similar to ( 690) loc_os11g43860 12011.m28865 protein ATMHX, putative, expressed no original description 	T
33.99	development.unspecified	MDP0000259632	 moderately similar to ( 393) AT3G12090 | Symbols: TET6 | TET6 (TETRASPANIN6) | chr3:3852326-3853714 REVERSEmoderately similar to ( 385) loc_os05g03530 12005.m27685 protein senescence-associated protein-like, putative, expressed   no original description 	T
35.2	not assigned.unknown	MDP0000135157	   no original description 	T
30.5	signalling.G-proteins	MDP0000198183	 moderately similar to ( 407) AT5G60860 | Symbols: AtRABA1f | AtRABA1f (Arabidopsis Rab GTPase homolog A1f); GTP binding | chr5:24484750-24485565 FORWARDmoderately similar to ( 392) RB11B_TOBAC Ras-related protein Rab11B - Nicotiana tabacum (Common tobacco)moderately similar to ( 397) loc_os09g15790 12009.m04840 protein ras-related protein Rab11B, putative, expressed no original description 	T
1.3.2	PS.calvin cycle.rubisco small subunit	MDP0000731480	 moderately similar to ( 297) AT5G38410 | Symbols:  | ribulose bisphosphate carboxylase small chain 3B / RuBisCO small subunit 3B (RBCS-3B) (ATS3B) | chr5:15377501-15378306 REVERSEmoderately similar to ( 315) RBS_BETVE Ribulose bisphosphate carboxylase small chain, chloroplast precursor (EC 4.1.1.39) (RuBisCO small subunit) - Betula verrucosa (White birch) (Betula pendula)moderately similar to ( 255) loc_os12g19394 12012.m73909 protein ribulose bisphosphate carboxylase small chain C, chloroplast precursor, putative, expressed  no original description 	T
35.1.41	not assigned.no ontology.hydroxyproline rich proteins	MDP0000219364	 weakly similar to ( 164) AT3G25690 | Symbols: CHUP1 | CHUP1 (CHLOROPLAST UNUSUAL POSITIONING 1) | chr3:9354061-9357757 FORWARDweakly similar to ( 140) loc_os12g01449 12012.m04148 protein expressed protein   no original description 	T
35.2	not assigned.unknown	MDP0000828036	 moderately similar to ( 271) AT3G55420 | Symbols:  | unknown protein | chr3:20547920-20548570 FORWARDmoderately similar to ( 283) loc_os05g38470 12005.m083744 protein expressed protein   no original description 	T
30.2.17	signalling.receptor kinases.DUF 26	MDP0000283397	 highly similar to ( 504) AT1G56130 | Symbols:  | leucine-rich repeat family protein / protein kinase family protein | chr1:20994931-21000887 REVERSEweakly similar to ( 139) NORK_PEA Nodulation receptor kinase precursor (EC 2.7.11.1) - Pisum sativum (Garden pea)highly similar to ( 511) loc_os04g52600 12004.m10153 protein protein kinase domain containing protein, expressed  no original description 	T
35.2	not assigned.unknown	MDP0000933141	 very weakly similar to (84.7) AT1G30845 | Symbols:  | unknown protein | chr1:10979856-10980427 FORWARDvery weakly similar to (95.1) loc_os03g03530 12003.m05886 protein cell growth defect factor 2, putative, expressed no original description 	T
27.3.80	RNA.regulation of transcription.zf-HD	MDP0000462091	 weakly similar to ( 164) AT4G24660 | Symbols: ATHB22, MEE68 | ATHB22 (HOMEOBOX PROTEIN 22); DNA binding / transcription factor | chr4:12724851-12725513 REVERSEweakly similar to ( 112) loc_os09g29130 12009.m06064 protein ZF-HD protein dimerisation region containing protein, expressed  TrbL  no original description 	T
30.5	signalling.G-proteins	MDP0000842413	 nearly identical (1156) AT5G13300 | Symbols: SFC, VAN3, AGD3 | SFC (SCARFACE); ARF GTPase activator/ phosphoinositide binding | chr5:4255923-4262018 REVERSEnearly identical (1049) loc_os09g33600 12009.m06409 protein VAN3, putative, expressed  no original description 	T
31.1	cell.organisation	MDP0000134730	 moderately similar to ( 349) AT1G30825 | Symbols: ARPC2A, DIS2 | DIS2 (DISTORTED TRICHOMES 2); structural molecule | chr1:10961106-10962999 FORWARDmoderately similar to ( 359) loc_os01g46580 12001.m10855 protein ARP2/3 complex 34 kDa subunit, putative, expressed    no original description 	T
31.1	cell.organisation	MDP0000303909	 nearly identical (1165) AT3G57410 | Symbols: VLN3, ATVLN3 | VLN3 (VILLIN 3); actin binding | chr3:21243615-21249809 REVERSEnearly identical (1062) loc_os03g24220 12003.m34866 protein villin-3, putative, expressed   no original description 	T
31.1	cell.organisation	MDP0000234621	 weakly similar to ( 142) AT5G04700 | Symbols:  | FUNCTIONS IN: molecular_function unknown; INVOLVED IN: biological_process unknown; LOCATED IN: cellular_component unknown; CONTAINS InterPro DOMAIN/s: Ankyrin (InterPro:IPR002110); BEST Arabidopsis thaliana protein match is: unknown protein (TAIR:AT5G04680.1); Has 868 Blast hits to 744 proteins in 75 species: Archae - 0; Bacteria - 22; Metazoa - 164; Fungi - 15; Plants - 471; Viruses - 0; Other Eukaryotes - 196 (source: NCBI BLink). | chr5:1354240-1356754 REVERSE  no original description 	T
13.1.2.2	amino acid metabolism.synthesis.glutamate family.proline	MDP0000909091	 highly similar to ( 976) AT3G55610 | Symbols: P5CS2 | P5CS2 (DELTA 1-PYRROLINE-5-CARBOXYLATE SYNTHASE 2); catalytic/ glutamate 5-kinase/ oxidoreductase | chr3:20625005-20628989 REVERSEhighly similar to ( 989) P5CS_ACTCH Delta 1-pyrroline-5-carboxylate synthetase (P5CS) [Includes: Glutamate 5-kinase (EC 2.7.2.11) (Gamma-glutamyl kinase) (GK); Gamma-glutamyl phosphate reductase (GPR) (EC 1.2.1.41) (Glutamate-5-semialdehyde dehydrogenase) (Glutamyl-gamma-highly similar to ( 944) loc_os05g38150 12005.m27606 protein delta 1-pyrroline-5-carboxylate synthetase, putative, expressed   no original description 	T
29.3.2	protein.targeting.mitochondria	MDP0000834339	 highly similar to ( 590) AT3G16480 | Symbols: MPPalpha | MPPalpha (mitochondrial processing peptidase alpha subunit); catalytic/ metal ion binding / metalloendopeptidase/ zinc ion binding | chr3:5599906-5602716 FORWARDhighly similar to ( 654) MPPA_SOLTU Mitochondrial-processing peptidase alpha subunit, mitochondrial precursor (EC 3.4.24.64) (Alpha-MPP) (Ubiquinol-cytochrome-c reductase subunit II) (EC 1.10.2.2) - Solanum tuberosum (Potato)highly similar to ( 684) loc_os01g53700 12001.m97538 protein mitochondrial-processing peptidase alpha subunit, mitochondrial precursor, putative, expressed  no original description 	T
31.3	cell.cycle	MDP0000155540	 weakly similar to ( 150) AT2G27960 | Symbols: CKS1, CKS1AT | CKS1 (CYCLIN-DEPENDENT KINASE-SUBUNIT 1); cyclin-dependent protein kinase/ protein binding | chr2:11910903-11911543 REVERSEweakly similar to ( 150) loc_os03g05300 12003.m06054 protein cyclin-dependent kinases regulatory subunit, putative, expressed no original description 	T
29.4	protein.postranslational modification	MDP0000184039	 highly similar to ( 896) AT2G44830 | Symbols:  | protein kinase, putative | chr2:18490398-18492779 FORWARDhighly similar to ( 616) G11A_ORYSA Protein kinase G11A (EC 2.7.11.1) - Oryza sativa (Rice)highly similar to ( 929) loc_os04g46180 12004.m101604 protein protein kinase G11A, putative, expressed   no original description 	T
29.1.11	protein.aa activation.serine-tRNA ligase	MDP0000215154	 highly similar to ( 714) AT5G27470 | Symbols:  | seryl-tRNA synthetase / serine--tRNA ligase | chr5:9695087-9697154 FORWARDhighly similar to ( 746) SYS_HELAN Seryl-tRNA synthetase (EC 6.1.1.11) (Serine--tRNA ligase) (SerRS) - Helianthus annuus (Common sunflower)highly similar to ( 645) loc_os03g10190 12003.m06472 protein seryl-tRNA synthetase, putative, expressed no original description 	T
27.3.25	RNA.regulation of transcription.MYB domain transcription factor family	MDP0000187872	 moderately similar to ( 244) AT5G67300 | Symbols: ATMYBR1, ATMYB44, MYBR1 | MYBR1 (MYB DOMAIN PROTEIN R1); DNA binding / transcription factor | chr5:26854109-26855026 FORWARDweakly similar to ( 109) GAM1_ORYSA Transcription factor GAMYB (OsGAMyb) - Oryza sativa (Rice)moderately similar to ( 231) loc_os09g01960 12009.m03570 protein sucrose responsive element binding protein, putative, expressed  no original description 	T
29.5.11.4.2	protein.degradation.ubiquitin.E3.RING	MDP0000204132	 moderately similar to ( 419) AT3G54360 | Symbols:  | protein binding / zinc ion binding | chr3:20128570-20131581 REVERSEmoderately similar to ( 414) loc_os01g01420 12001.m150459 protein expressed protein MSP1_C  no original description 	T
35.2	not assigned.unknown	MDP0000307862	 moderately similar to ( 364) AT4G21570 | Symbols:  | unknown protein | chr4:11471126-11472269 REVERSEmoderately similar to ( 367) loc_os07g32230 12007.m29186 protein MAPK activating protein, putative, expressed  no original description 	T
23.1.3	nucleotide metabolism.synthesis.PRS-PP	MDP0000531623	 highly similar to ( 550) AT2G42910 | Symbols:  | ribose-phosphate pyrophosphokinase 4 / phosphoribosyl diphosphate synthetase 4 (PRS4) | chr2:17856396-17858394 FORWARDhighly similar to ( 560) KPRS4_SPIOL Ribose-phosphate pyrophosphokinase 4 (EC 2.7.6.1) (Phosphoribosyl pyrophosphate synthetase 4) - Spinacia oleracea (Spinach)highly similar to ( 501) loc_os02g48390 12002.m33837 protein ribose-phosphate pyrophosphokinase 4, putative, expressed no original description 	T
26.13	misc.acid and other phosphatases	MDP0000758548	 moderately similar to ( 271) AT5G03080 | Symbols:  | phosphatidic acid phosphatase-related / PAP2-related | chr5:721976-722656 FORWARDmoderately similar to ( 238) loc_os03g17940 12003.m07219 protein dolichyldiphosphatase 1, putative, expressed   no original description 	T
10.6.2	cell wall.degradation.mannan-xylose-arabinose-fucose	MDP0000832632	 highly similar to ( 569) AT5G66460 | Symbols:  | (1-4)-beta-mannan endohydrolase, putative | chr5:26538911-26540837 REVERSEhighly similar to ( 526) loc_os01g47400 12001.m10935 protein hydrolase, hydrolyzing O-glycosyl compounds, putative, expressed  no original description 	T
35.1	not assigned.no ontology	MDP0000278669	 moderately similar to ( 346) AT5G46410 | Symbols:  | NLI interacting factor (NIF) family protein | chr5:18826007-18828156 FORWARDmoderately similar to ( 269) loc_os01g43870 12001.m97456 protein CTD-phosphatase-like protein, putative, expressed no original description 	T
35.1	not assigned.no ontology	MDP0000207264	 moderately similar to ( 324) AT3G17380 | Symbols:  | meprin and TRAF homology domain-containing protein / MATH domain-containing protein | chr3:5950240-5952124 FORWARDvery weakly similar to (83.6) loc_os12g30540 12012.m06866 protein ubiquitin carboxyl-terminal hydrolase 21, putative, expressed  no original description 	T
29.4	protein.postranslational modification	MDP0000319890	 highly similar to ( 698) AT1G13460 | Symbols:  | serine/threonine protein phosphatase 2A (PP2A) regulatory subunit B', putative | chr1:4616504-4618180 FORWARDhighly similar to ( 682) loc_os04g40860 12004.m09066 protein transposon protein, putative, unclassified, expressed   no original description 	T
17.8.1	hormone metabolism.salicylic acid.synthesis-degradation	MDP0000509245	 moderately similar to ( 370) AT1G19640 | Symbols: JMT | JMT (JASMONIC ACID CARBOXYL METHYLTRANSFERASE); jasmonate O-methyltransferase | chr1:6789166-6791708 REVERSEmoderately similar to ( 245) BAMT_ANTMA Benzoate carboxyl methyltransferase (EC 2.1.1.-) (S-adenosyl-L-methionine:benzoic acid carboxyl methyltransferase) - Antirrhinum majus (Garden snapdragon)moderately similar to ( 278) loc_os01g50610 12001.m150693 protein jasmonate O-methyltransferase, putative, expressed   no original description 	T
10.1.5	cell wall.precursor synthesis.UXS	MDP0000316092	 highly similar to ( 598) AT3G62830 | Symbols: UXS2, ATUXS2 | AUD1; UDP-glucuronate decarboxylase/ catalytic/ dTDP-glucose 4,6-dehydratase | chr3:23232539-23235353 FORWARDvery weakly similar to (93.2) GME1_ORYSA GDP-mannose 3,5-epimerase 1 (EC 5.1.3.18) (GDP-Man 3,5-epimerase 1) (OsGME-1) - Oryza sativa (Rice)highly similar to ( 615) loc_os05g29990 12005.m07235 protein UDP-glucuronic acid decarboxylase 1, putative, expressed   no original description 	T
35.2	not assigned.unknown	MDP0000167288	  no original description 	T
27.3.11	RNA.regulation of transcription.C2H2 zinc finger family	MDP0000807856	 moderately similar to ( 490) AT1G34370 | Symbols: STOP1 | STOP1 (sensitive to proton rhizotoxicity 1); nucleic acid binding / transcription factor/ zinc ion binding | chr1:12551449-12552501 FORWARDmoderately similar to ( 422) loc_os01g65080 12001.m150442 protein nucleic acid binding protein, putative, expressed  no original description 	T
33.99	development.unspecified	MDP0000226701	 moderately similar to ( 301) AT1G12260 | Symbols: VND4, EMB2749, ANAC007 | ANAC007 (ARABIDOPSIS NAC 007); transcription factor | chr1:4163058-4164486 REVERSEweakly similar to ( 189) NAC76_ORYSA NAC domain-containing protein 76 (ONAC076) - Oryza sativa (Rice)moderately similar to ( 275) loc_os02g42970 12002.m09332 protein NAC domain-containing protein 76, putative   no original description 	T
34.8	transport.metabolite transporters at the envelope membrane	MDP0000948690	 highly similar to ( 887) AT1G80300 | Symbols: NTT1, ATNTT1 | NTT1 (NUCLEOTIDE TRANSPORTER 1); ATP:ADP antiporter | chr1:30191954-30194280 FORWARDhighly similar to ( 907) TLC1_SOLTU Plastidic ATP/ADP-transporter - Solanum tuberosum (Potato)highly similar to ( 759) loc_os02g11740 12002.m100137 protein plastidic ATP/ADP-transporter, putative, expressed  no original description 	T
29.4	protein.postranslational modification	MDP0000277926	 weakly similar to ( 176) AT5G50200 | Symbols: WR3, ATNRT3.1, NRT3.1 | WR3 (WOUND-RESPONSIVE 3); nitrate transmembrane transporter | chr5:20436612-20437535 FORWARDweakly similar to ( 159) loc_os02g38230 12002.m08911 protein component of high affinity nitrate transporter, putative, expressed no original description 	T
34.4	transport.nitrate	MDP0000277926	 weakly similar to ( 176) AT5G50200 | Symbols: WR3, ATNRT3.1, NRT3.1 | WR3 (WOUND-RESPONSIVE 3); nitrate transmembrane transporter | chr5:20436612-20437535 FORWARDweakly similar to ( 159) loc_os02g38230 12002.m08911 protein component of high affinity nitrate transporter, putative, expressed no original description 	T
33.99	development.unspecified	MDP0000242052	 weakly similar to ( 182) AT4G35770 | Symbols: SEN1, ATSEN1, DIN1 | SEN1 (SENESCENCE 1) | chr4:16945073-16945964 FORWARDweakly similar to ( 180) DIN1_RAPSA Senescence-associated protein DIN1 - Raphanus sativus (Radish)weakly similar to ( 166) loc_os06g50930 12006.m09617 protein senescence-associated protein DIN1, putative, expressed no original description 	T
11.2.3	lipid metabolism.FA desaturation.omega 3 desaturase	MDP0000722255	 highly similar to ( 642) AT3G12120 | Symbols: FAD2 | FAD2 (FATTY ACID DESATURASE 2); delta12-fatty acid dehydrogenase/ omega-6 fatty acid desaturase | chr3:3860592-3861743 REVERSEhighly similar to ( 645) FD6E2_SOYBN Omega-6 fatty acid desaturase, endoplasmic reticulum isozyme 2 (EC 1.14.19.-) - Glycine max (Soybean)highly similar to ( 588) loc_os02g48560 12002.m100054 protein omega-6 fatty acid desaturase, endoplasmic reticulum isozyme 2, putative, expressed  no original description 	T
34.16	transport.ABC transporters and multidrug resistance systems	MDP0000320690	 nearly identical (1901) AT3G28860 | Symbols: ATMDR1, ATMDR11, PGP19, MDR11, MDR1, ATPGP19, ABCB19, ATABCB19 | ABCB19; ATPase, coupled to transmembrane movement of substances / auxin efflux transmembrane transporter | chr3:10870287-10877286 REVERSEhighly similar to ( 957) MDR_ORYSA Putative multidrug resistance protein (P-glycoprotein) - Oryza sativa (Rice)nearly identical (1825) loc_os04g38570 12004.m101543 protein multidrug resistance protein 11, putative, expressed   no original description 	T
30.3	signalling.calcium	MDP0000285559	 weakly similar to ( 134) AT2G15680 | Symbols:  | calmodulin-related protein, putative | chr2:6831024-6831587 FORWARDvery weakly similar to (78.6) CALM_CHLRE Calmodulin (CaM) - Chlamydomonas reinhardtiiweakly similar to ( 102) loc_os05g13580 12005.m05773 protein calmodulin-related protein 2, touch-induced, putative, expressed   no original description 	T
27.3.3	RNA.regulation of transcription.AP2/EREBP, APETALA2/Ethylene-responsive element binding protein family	MDP0000290880	 weakly similar to ( 197) AT1G15360 | Symbols: SHN1, WIN1 | SHN1 (SHINE 1); DNA binding / sequence-specific DNA binding / transcription factor | chr1:5283609-5284506 FORWARDvery weakly similar to (64.7) ERF4_TOBAC Ethylene-responsive transcription factor 4 (Ethylene-responsive element-binding factor 4 homolog) (EREBP-3) (NtERF3) - Nicotiana tabacum (Common tobacco)weakly similar to ( 193) loc_os02g10760 12002.m06374 protein ethylene responsive element, putative, expressed  no original description 	T
30.5	signalling.G-proteins	MDP0000404324	 moderately similar to ( 304) AT4G02080 | Symbols: ASAR1, ATSARA1C, ATSAR2 | ATSAR2 (ARABIDOPSIS THALIANA SECRETION-ASSOCIATED RAS SUPER FAMILY 2); GTP binding | chr4:921554-922547 FORWARDmoderately similar to ( 300) SAR1A_BRACM GTP-binding protein SAR1A - Brassica campestris (Field mustard)moderately similar to ( 303) loc_os01g15010 12001.m08102 protein GTP-binding protein SAR1A, putative, expressed no original description 	T
31.1	cell.organisation	MDP0000225059	 moderately similar to ( 218) AT2G31200 | Symbols: ADF6, ATADF6 | ADF6 (ACTIN DEPOLYMERIZING FACTOR 6); actin binding | chr2:13294171-13294948 FORWARDmoderately similar to ( 225) ADF_VITVI Actin-depolymerizing factor (ADF) - Vitis vinifera (Grape)moderately similar to ( 201) loc_os12g43340 12012.m08108 protein actin-depolymerizing factor, putative, expressed  no original description 	T
35.2	not assigned.unknown	MDP0000208401	  no original description 	T
30.2.11	signalling.receptor kinases.leucine rich repeat XI	MDP0000285813	 highly similar to ( 923) AT2G01210 | Symbols:  | leucine-rich repeat transmembrane protein kinase, putative | chr2:119509-121734 REVERSEmoderately similar to ( 251) RPK1_IPONI Receptor-like protein kinase precursor (EC 2.7.11.1) - Ipomoea nil (Japanese morning glory) (Pharbitis nil)highly similar to ( 830) loc_os01g33090 12001.m09626 protein ATP binding protein, putative, expressed   no original description 	T
30.2.3	signalling.receptor kinases.leucine rich repeat III	MDP0000285813	 highly similar to ( 923) AT2G01210 | Symbols:  | leucine-rich repeat transmembrane protein kinase, putative | chr2:119509-121734 REVERSEmoderately similar to ( 251) RPK1_IPONI Receptor-like protein kinase precursor (EC 2.7.11.1) - Ipomoea nil (Japanese morning glory) (Pharbitis nil)highly similar to ( 830) loc_os01g33090 12001.m09626 protein ATP binding protein, putative, expressed   no original description 	T
34.30	transport.H+ transporting pyrophosphatase	MDP0000154557	 moderately similar to ( 320) AT1G16780 | Symbols:  | vacuolar-type H+-translocating inorganic pyrophosphatase, putative | chr1:5739468-5743569 REVERSEvery weakly similar to (94.0) AVP_PHAAU Pyrophosphate-energized vacuolar membrane proton pump (EC 3.6.1.1) (Pyrophosphate-energized inorganic pyrophosphatase) (H(+)-PPase) (Vacuolar H(+)-pyrophosphatase) - Phaseolus aureus (Mung bean) (Vigna radiata)moderately similar to ( 213) loc_os02g33490 12002.m08442 protein pyrophosphate-energized membrane proton pump 3, putative, expressed    no original description 	T
13.2.4.4	amino acid metabolism.degradation.branched chain group.leucine	MDP0000143368	 highly similar to ( 967) AT1G03090 | Symbols: MCCA | MCCA; methylcrotonoyl-CoA carboxylase | chr1:739715-743819 FORWARDnearly identical (1033) MCCA_SOYBN Methylcrotonoyl-CoA carboxylase subunit alpha, mitochondrial precursor (EC 6.4.1.4) (3-methylcrotonyl-CoA carboxylase 1) (MCCase subunit alpha) (3-methylcrotonyl-CoA:carbon dioxide ligase subunit alpha) - Glycine max (Soybean)highly similar to ( 924) loc_os12g41250 12012.m26967 protein methylcrotonoyl-CoA carboxylase subunit alpha, mitochondrial precursor, putative, expressed  no original description 	T
35.2	not assigned.unknown	MDP0000314082	 moderately similar to ( 323) AT4G35760 | Symbols:  | LOCATED IN: chloroplast; EXPRESSED IN: 23 plant structures; EXPRESSED DURING: 13 growth stages; CONTAINS InterPro DOMAIN/s: Vitamin K epoxide reductase (InterPro:IPR012932), Thioredoxin-like fold (InterPro:IPR012336); Has 447 Blast hits to 447 proteins in 86 species: Archae - 0; Bacteria - 179; Metazoa - 0; Fungi - 0; Plants - 31; Viruses - 0; Other Eukaryotes - 237 (source: NCBI BLink). | chr4:16942733-16944622 REVERSEmoderately similar to ( 262) loc_os03g03949 12003.m35562 protein thioredoxin domain 2, putative, expressed  no original description 	T
26.26.1	misc.aminotransferases.aminotransferase class IV family protein	MDP0000936735	 moderately similar to ( 297) AT5G27410 | Symbols:  | aminotransferase class IV family protein | chr5:9678821-9682468 FORWARDmoderately similar to ( 273) loc_os03g24460 12003.m07792 protein aminotransferase, class IV family protein, expressed no original description 	T
27.3.29	RNA.regulation of transcription.TCP transcription factor family	MDP0000319941	 weakly similar to ( 182) AT3G47620 | Symbols: AtTCP14 | AtTCP14 (TEOSINTE BRANCHED1, CYCLOIDEA and PCF (TCP) 14); transcription factor | chr3:17559004-17560473 FORWARDweakly similar to ( 200) loc_os06g12230 12006.m05940 protein TCP-domain protein, putative, expressed  no original description 	T
30.2.11	signalling.receptor kinases.leucine rich repeat XI	MDP0000445041	 highly similar to ( 504) AT1G72300 | Symbols:  | leucine-rich repeat transmembrane protein kinase, putative | chr1:27217679-27220966 REVERSEmoderately similar to ( 340) PSKR_DAUCA Phytosulfokine receptor precursor (EC 2.7.11.1) (Phytosulfokine LRR receptor kinase) - Daucus carota (Carrot)moderately similar to ( 454) loc_os02g05930 12002.m05941 protein phytosulfokine receptor precursor, putative, expressed   no original description 	T
27.1.21	RNA.processing.siRNA methyltransferase	MDP0000154407	 highly similar to ( 803) AT4G20910 | Symbols: HEN1, CRM2 | HEN1 (HUA ENHANCER 1); RNA methyltransferase | chr4:11186264-11190575 REVERSEhighly similar to ( 769) loc_os07g06970 12007.m05157 protein HEN1, putative, expressed  no original description 	T
27.3.11	RNA.regulation of transcription.C2H2 zinc finger family	MDP0000122321	 moderately similar to ( 278) AT3G45260 | Symbols:  | zinc finger (C2H2 type) family protein | chr3:16596850-16598550 REVERSEmoderately similar to ( 267) loc_os01g09850 12001.m42906 protein zinc finger, C2H2-type, putative, expressed  MSP1_C  no original description 	T
29.4	protein.postranslational modification	MDP0000326020	 highly similar to ( 643) AT4G01370 | Symbols: ATMPK4, MPK4 | ATMPK4 (ARABIDOPSIS THALIANA MAP KINASE 4); MAP kinase/ kinase | chr4:567219-568889 FORWARDhighly similar to ( 648) MMK2_MEDSA Mitogen-activated protein kinase homolog MMK2 (EC 2.7.11.24) - Medicago sativa (Alfalfa)highly similar to ( 624) loc_os08g06060 12008.m04746 protein mitogen-activated protein kinase homolog 4, putative, expressed VAR1  no original description 	T
29.5.11.5	protein.degradation.ubiquitin.ubiquitin protease	MDP0000831007	 highly similar to ( 541) AT1G51710 | Symbols: UBP6 | UBP6 (UBIQUITIN-SPECIFIC PROTEASE 6); calmodulin binding / ubiquitin-specific protease | chr1:19175805-19179399 REVERSEmoderately similar to ( 496) loc_os01g36930 12001.m43049 protein ubiquitin carboxyl-terminal hydrolase 6, putative, expressed no original description 	T
16.2.1.10	secondary metabolism.phenylpropanoids.lignin biosynthesis.CAD	MDP0000873573	 highly similar to ( 595) AT5G51970 | Symbols:  | sorbitol dehydrogenase, putative / L-iditol 2-dehydrogenase, putative | chr5:21111820-21113284 FORWARDvery weakly similar to (97.4) ADHX_MAIZE Alcohol dehydrogenase class 3 (EC 1.1.1.1) (Alcohol dehydrogenase class III) (S-(hydroxymethyl)glutathione dehydrogenase) (EC 1.1.1.284) (Glutathione-dependent formaldehyde dehydrogenase) (FDH) (FALDH) (GSH-FDH) - Zea mays (Maize)highly similar to ( 563) loc_os08g43190 12008.m08293 protein sorbitol dehydrogenase, putative, expressed  no original description 	T
30.2.11	signalling.receptor kinases.leucine rich repeat XI	MDP0000230316	 moderately similar to ( 444) AT4G35470 | Symbols:  | leucine-rich repeat family protein | chr4:16846531-16848448 FORWARDvery weakly similar to (69.7) RPK1_IPONI Receptor-like protein kinase precursor (EC 2.7.11.1) - Ipomoea nil (Japanese morning glory) (Pharbitis nil)highly similar to ( 521) loc_os10g42190 12010.m21806 protein protein lap4, putative, expressed  no original description 	T
35.2	not assigned.unknown	MDP0000555486	   no original description 	T
35.1	not assigned.no ontology	MDP0000129786	 weakly similar to ( 159) AT4G02860 | Symbols:  | catalytic | chr4:1268952-1270508 REVERSEweakly similar to ( 146) loc_os01g16146 12001.m08212 protein phenazine biosynthesis protein, putative, expressed no original description 	T
34.3	transport.amino acids	MDP0000690396	 highly similar to ( 679) AT1G17120 | Symbols: CAT8 | CAT8 (CATIONIC AMINO ACID TRANSPORTER 8); basic amino acid transmembrane transporter/ cationic amino acid transmembrane transporter | chr1:5851964-5853736 FORWARDhighly similar to ( 508) loc_os01g11160 12001.m07736 protein cationic amino acid transporter 4, putative, expressed PRK08026  no original description 	T
35.2	not assigned.unknown	MDP0000140242	 moderately similar to ( 451) AT4G21210 | Symbols: ATRP1 | ATRP1 (PPDK REGULATORY PROTEIN); phosphoprotein phosphatase/ protein kinase | chr4:11307002-11308587 FORWARDmoderately similar to ( 476) loc_os07g34640 12007.m29204 protein ATP/GTP-binding protein, putative, expressed   no original description 	T
35.2	not assigned.unknown	MDP0000164794	 weakly similar to ( 103) AT1G21510 | Symbols:  | unknown protein | chr1:7531534-7532505 REVERSE no original description 	T
26.21	misc.protease inhibitor/seed storage/lipid transfer protein (LTP) family protein	MDP0000262811	 moderately similar to ( 295) AT1G67325 | Symbols:  | binding / zinc ion binding | chr1:25209825-25212412 REVERSEweakly similar to ( 121) HS22M_SOYBN Heat shock 22 kDa protein, mitochondrial precursor - Glycine max (Soybean)moderately similar to ( 222) loc_os08g41010 12008.m26752 protein Zn-finger, RanBP-type, containing protein, expressed    no original description 	T
27.1.19	RNA.processing.ribonucleases	MDP0000334586	 moderately similar to ( 219) AT3G20390 | Symbols:  | endoribonuclease L-PSP family protein | chr3:7110227-7111695 REVERSEmoderately similar to ( 215) loc_os07g33240 12007.m07585 protein expressed protein no original description 	T
31.1	cell.organisation	MDP0000329552	 moderately similar to ( 209) AT4G00680 | Symbols: ADF8 | ADF8 (ACTIN DEPOLYMERIZING FACTOR 8); actin binding | chr4:279780-280608 REVERSEmoderately similar to ( 221) ADF1_PETHY Actin-depolymerizing factor 1 (ADF 1) - Petunia hybrida (Petunia)moderately similar to ( 214) loc_os05g02250 12005.m64199 protein expressed protein  no original description 	T
11.1.9	lipid metabolism.FA synthesis and FA elongation.long chain fatty acid CoA ligase	MDP0000201853	 highly similar to ( 915) AT1G49430 | Symbols: LRD2, LACS2 | LACS2 (LONG-CHAIN ACYL-COA SYNTHETASE 2); long-chain-fatty-acid-CoA ligase | chr1:18291188-18295641 FORWARDvery weakly similar to (90.5) 4CL1_ORYSA 4-coumarate--CoA ligase 1 (EC 6.2.1.12) (4CL 1) (4-coumaroyl-CoA synthase 1) - Oryza sativa (Rice)highly similar to ( 922) loc_os11g35400 12011.m080023 protein acyl CoA synthetase, putative, expressed  no original description 	T
29.5.11.4.3.2	protein.degradation.ubiquitin.E3.SCF.FBOX	MDP0000142240	 moderately similar to ( 324) AT1G80630 | Symbols:  | leucine-rich repeat family protein | chr1:30308879-30310615 REVERSEvery weakly similar to (85.1) loc_os11g01780 12011.m04379 protein ubiquitin-protein ligase, putative, expressed   no original description 	T
10.1.5	cell wall.precursor synthesis.UXS	MDP0000364261	 weakly similar to ( 161) AT2G47650 | Symbols: UXS4 | UXS4 (UDP-XYLOSE SYNTHASE 4); UDP-glucuronate decarboxylase/ catalytic | chr2:19538751-19541364 REVERSEweakly similar to ( 155) loc_os05g29990 12005.m07235 protein UDP-glucuronic acid decarboxylase 1, putative, expressed no original description 	T
29.2.1.1.1.2.4	protein.synthesis.ribosomal protein.prokaryotic.chloroplast.50S subunit.L4	MDP0000260618	 moderately similar to ( 325) AT1G07320 | Symbols: RPL4 | RPL4; poly(U) binding / structural constituent of ribosome | chr1:2249190-2250173 FORWARDmoderately similar to ( 317) RK4_TOBAC 50S ribosomal protein L4, chloroplast precursor (CL4) (R-protein L4) - Nicotiana tabacum (Common tobacco)moderately similar to ( 315) loc_os03g15870 12003.m07024 protein 50S ribosomal protein L4, chloroplast precursor, putative, expressed   PRK08026  no original description 	T
29.5.11.20	protein.degradation.ubiquitin.proteasom	MDP0000272068	 weakly similar to ( 171) AT5G35590 | Symbols: PAA1 | PAA1 (PROTEASOME ALPHA SUBUNIT A 1); endopeptidase/ peptidase/ threonine-type endopeptidase | chr5:13765417-13767768 REVERSEweakly similar to ( 183) PSA6_SOYBN Proteasome subunit alpha type 6 (EC 3.4.25.1) (20S proteasome alpha subunit A) (20S proteasome subunit alpha-1) (Proteasome iota subunit) - Glycine max (Soybean)weakly similar to ( 165) loc_os03g08280 12003.m101213 protein proteasome subunit alpha type 6, putative, expressed no original description 	T
35.2	not assigned.unknown	MDP0000284647	 very weakly similar to (79.7) AT4G14380 | Symbols:  | unknown protein | chr4:8285815-8286417 FORWARD  no original description 	T
26.11	misc.alcohol dehydrogenases	MDP0000677354	 highly similar to ( 570) AT1G77120 | Symbols: ADH1, ADH, ATADH, ATADH1 | ADH1 (ALCOHOL DEHYDROGENASE 1); alcohol dehydrogenase | chr1:28975509-28977216 FORWARDhighly similar to ( 638) ADH_MALDO Alcohol dehydrogenase (EC 1.1.1.1) - Malus domestica (Apple) (Malus sylvestris)highly similar to ( 581) loc_os11g10480 12011.m28756 protein alcohol dehydrogenase 1, putative, expressed  no original description 	T
33.99	development.unspecified	MDP0000175733	 weakly similar to ( 151) AT5G14930 | Symbols: SAG101 | SAG101 (SENESCENCE-ASSOCIATED GENE 101); carboxylesterase/ triacylglycerol lipase | chr5:4828754-4830165 FORWARDvery weakly similar to (68.6) loc_os11g09010 12011.m079958 protein phytoalexin-deficient 4-1 protein, putative, expressed no original description 	T
29.5.11.4.2	protein.degradation.ubiquitin.E3.RING	MDP0000120956	 moderately similar to ( 288) AT5G08750 | Symbols:  | zinc finger (C3HC4-type RING finger) family protein | chr5:2852912-2854343 FORWARDmoderately similar to ( 218) loc_os04g59580 12004.m10833 protein protein binding protein, putative, expressed  no original description 	T
27.4	RNA.RNA binding	MDP0000310452	 moderately similar to ( 350) AT3G20890 | Symbols:  | RNA binding / nucleic acid binding / nucleotide binding | chr3:7320218-7321054 FORWARDmoderately similar to ( 237) loc_os11g03890 12011.m04584 protein RNA binding protein, putative, expressed no original description 	T
29.2.1.2.1.23	protein.synthesis.ribosomal protein.eukaryotic.40S subunit.S23	MDP0000327325	 moderately similar to ( 275) AT5G02960 | Symbols:  | 40S ribosomal protein S23 (RPS23B) | chr5:693280-694396 REVERSEmoderately similar to ( 283) RS23_FRAAN 40S ribosomal protein S23 (S12) - Fragaria ananassa (Strawberry)moderately similar to ( 283) loc_os03g60400 12003.m10930 protein 40S ribosomal protein S23, putative, expressed no original description 	T
35.1	not assigned.no ontology	MDP0000153039	 nearly identical (1075) AT5G35160 | Symbols:  | LOCATED IN: integral to membrane, Golgi apparatus; EXPRESSED IN: 24 plant structures; EXPRESSED DURING: 13 growth stages; CONTAINS InterPro DOMAIN/s: Nonaspanin (TM9SF) (InterPro:IPR004240); BEST Arabidopsis thaliana protein match is: unknown protein (TAIR:AT4G12650.1); Has 1053 Blast hits to 1004 proteins in 163 species: Archae - 0; Bacteria - 0; Metazoa - 478; Fungi - 143; Plants - 250; Viruses - 0; Other Eukaryotes - 182 (source: NCBI BLink). | chr5:13414945-13416921 FORWARDnearly identical (1079) loc_os02g55440 12002.m100394 protein transmembrane 9 superfamily protein member 2 precursor, putative, expressed  no original description 	T
35.2	not assigned.unknown	MDP0000377544	 very weakly similar to (86.7) AT1G20100 | Symbols:  | unknown protein | chr1:6969409-6970699 REVERSE   no original description 	T
29.3.4.4	protein.targeting.secretory pathway.plasma membrane	MDP0000838167	 highly similar to ( 553) AT2G30050 | Symbols:  | transducin family protein / WD-40 repeat family protein | chr2:12825540-12826448 FORWARDvery weakly similar to (57.8) GBB_MAIZE Guanine nucleotide-binding protein subunit beta - Zea mays (Maize)highly similar to ( 535) loc_os02g04320 12002.m33334 protein SEC13-related protein, putative, expressed   no original description 	T
34.9	transport.metabolite transporters at the mitochondrial membrane	MDP0000315710	 highly similar to ( 556) AT5G14040 | Symbols:  | mitochondrial phosphate transporter | chr5:4531059-4532965 REVERSEvery weakly similar to (55.1) BT1_MAIZE Protein brittle-1, chloroplast precursor - Zea mays (Maize)highly similar to ( 541) loc_os02g52860 12002.m10316 protein phosphate carrier protein, mitochondrial precursor, putative, expressed  no original description 	T
29.2.1.2.2.37	protein.synthesis.ribosomal protein.eukaryotic.60S subunit.L37	MDP0000922228	 weakly similar to ( 176) AT3G16080 | Symbols:  | 60S ribosomal protein L37 (RPL37C) | chr3:5454892-5455677 FORWARDweakly similar to ( 176) loc_os02g56990 12002.m10721 protein 60S ribosomal protein L37, putative, expressed no original description 	T
29.3.4.99	protein.targeting.secretory pathway.unspecified	MDP0000311915	 moderately similar to ( 310) AT1G14820 | Symbols:  | SEC14 cytosolic factor family protein / phosphoglyceride transfer family protein | chr1:5105237-5106454 REVERSEweakly similar to ( 144) loc_os03g63370 12003.m11204 protein polyphosphoinositide binding protein Ssh2p, putative, expressed no original description 	T
35.2	not assigned.unknown	MDP0000342721	  no original description 	T
35.1	not assigned.no ontology	MDP0000180195	 weakly similar to ( 185) AT1G04340 | Symbols:  | lesion inducing protein-related | chr1:1163345-1164737 REVERSEweakly similar to ( 197) loc_os03g05520 12003.m06075 protein nicotiana lesion-inducing like, putative, expressed   no original description 	T
29.4	protein.postranslational modification	MDP0000920333	 weakly similar to ( 164) AT1G34750 | Symbols:  | protein phosphatase 2C, putative / PP2C, putative | chr1:12736386-12737727 REVERSEweakly similar to ( 142) loc_os04g37904 12004.m35576 protein protein phosphatase 2C isoform epsilon, putative, expressed  no original description 	T
34.99	transport.misc	MDP0000756709	 moderately similar to ( 474) AT2G38330 | Symbols:  | MATE efflux family protein | chr2:16064571-16067318 FORWARDmoderately similar to ( 416) loc_os12g01580 12012.m26797 protein antiporter/ drug transporter, putative, expressed no original description 	T
29.2.1.2.1.19	protein.synthesis.ribosomal protein.eukaryotic.40S subunit.S19	MDP0000327126	 moderately similar to ( 262) AT3G02080 | Symbols:  | 40S ribosomal protein S19 (RPS19A) | chr3:364138-365161 REVERSEmoderately similar to ( 243) RS19_ORYSA 40S ribosomal protein S19 - Oryza sativa (Rice)moderately similar to ( 243) loc_os03g31090 12003.m08361 protein 40S ribosomal protein S19, putative, expressed  no original description 	T
31.2	cell.division	MDP0000188767	 moderately similar to ( 440) AT5G40770 | Symbols: ATPHB3 | ATPHB3 (PROHIBITIN 3) | chr5:16315589-16316621 REVERSEmoderately similar to ( 393) loc_os02g37000 12002.m08788 protein mitochondrial prohibitin complex protein 1, putative, expressed   no original description 	T
33.99	development.unspecified	MDP0000241871	 weakly similar to ( 151) AT3G02380 | Symbols: COL2 | COL2 (constans-like 2); transcription factor/ zinc ion binding | chr3:487438-488624 REVERSEweakly similar to ( 115) loc_os06g16370 12006.m06350 protein zinc finger protein CONSTANS, putative, expressed  no original description 	T
29.4	protein.postranslational modification	MDP0000593502	 highly similar to ( 614) AT1G07880 | Symbols: ATMPK13 | ATMPK13; MAP kinase/ kinase | chr1:2434193-2435712 REVERSEhighly similar to ( 634) NTF6_TOBAC Mitogen-activated protein kinase homolog NTF6 (EC 2.7.11.24) (P43) - Nicotiana tabacum (Common tobacco)highly similar to ( 584) loc_os10g38950 12010.m06663 protein OsMPK4 - putative MAPK based on amino acid sequence homology, expressed  no original description 	T
30.2.11	signalling.receptor kinases.leucine rich repeat XI	MDP0000266980	 highly similar to ( 890) AT2G35620 | Symbols: FEI2 | FEI2 (FEI 2); kinase | chr2:14961187-14964640 REVERSEmoderately similar to ( 310) PSKR_DAUCA Phytosulfokine receptor precursor (EC 2.7.11.1) (Phytosulfokine LRR receptor kinase) - Daucus carota (Carrot)highly similar to ( 843) loc_os03g16010 12003.m07038 protein BRASSINOSTEROID INSENSITIVE 1-associated receptor kinase 1 precursor, putative, expressed  no original description 	T
34.16	transport.ABC transporters and multidrug resistance systems	MDP0000231505	 moderately similar to ( 346) AT1G67940 | Symbols: ATNAP3 | ATNAP3; transporter | chr1:25477805-25478667 FORWARDweakly similar to ( 132) CYSA_CHLVU Probable sulfate/thiosulfate import ATP-binding protein cysA (EC 3.6.3.25) (Sulfate-transporting ATPase) - Chlorella vulgaris (Green alga)moderately similar to ( 334) loc_os06g48060 12006.m09332 protein phosphate import ATP-binding protein pstB 1, putative, expressed TrbL  no original description 	T
35.2	not assigned.unknown	MDP0000132296	  no original description 	T
35.2	not assigned.unknown	MDP0000369619	  no original description 	T
35.2	not assigned.unknown	MDP0000363622	  no original description 	T
35.2	not assigned.unknown	MDP0000500735	 moderately similar to ( 380) AT2G40480 | Symbols:  | unknown protein | chr2:16910425-16912444 FORWARDmoderately similar to ( 262) loc_os05g07632 12005.m27473 protein catalytic/ hydrolase, putative, expressed   no original description 	T
20.2.4	stress.abiotic.touch/wounding	MDP0000507273	 weakly similar to ( 151) AT5G42050 | Symbols:  | FUNCTIONS IN: molecular_function unknown; EXPRESSED IN: 22 plant structures; EXPRESSED DURING: 13 growth stages; CONTAINS InterPro DOMAIN/s: Development and cell death domain (InterPro:IPR013989), Kelch related (InterPro:IPR013089); BEST Arabidopsis thaliana protein match is: unknown protein (TAIR:AT3G27090.1); Has 5084 Blast hits to 2870 proteins in 85 species: Archae - 0; Bacteria - 24; Metazoa - 270; Fungi - 58; Plants - 157; Viruses - 8; Other Eukaryotes - 4567 (source: NCBI BLink). | chr5:16815630-16816932 FORWARDvery weakly similar to (86.7) B2_DAUCA B2 protein - Daucus carota (Carrot)weakly similar to ( 102) loc_os01g36950 12001.m150623 protein N-rich protein, putative, expressed no original description 	T
35.2	not assigned.unknown	MDP0000349798	 weakly similar to ( 135) AT5G20165 | Symbols:  | unknown protein | chr5:6805870-6806730 REVERSE no original description 	T
27.3.8	RNA.regulation of transcription.C2C2(Zn) DOF zinc finger family	MDP0000273251	 weakly similar to ( 157) AT3G21270 | Symbols: ADOF2 | ADOF2; DNA binding / transcription factor | chr3:7474934-7475548 FORWARDweakly similar to ( 105) PBF_MAIZE Dof zinc finger protein PBF (Prolamin box-binding factor) - Zea mays (Maize)weakly similar to ( 117) loc_os03g38870 12003.m08982 protein dof domain, zinc finger family protein, expressed  no original description 	T
26.17	misc.dynamin	MDP0000138874	 highly similar to ( 861) AT3G19720 | Symbols: ARC5 | ARC5 (ACCUMULATION AND REPLICATION OF CHLOROPLAST 5); GTP binding / GTPase | chr3:6850591-6855389 REVERSEhighly similar to ( 781) loc_os12g07880 12012.m04775 protein ARC5, putative, expressed  COG4907  no original description 	T
28.1.3	DNA.synthesis/chromatin structure.histone	MDP0000334368	 weakly similar to ( 159) AT1G51060 | Symbols: HTA10 | HTA10; DNA binding | chr1:18926948-18927443 FORWARDweakly similar to ( 159) H2A2_ORYSA Probable histone H2A.2 - Oryza sativa (Rice)weakly similar to ( 159) loc_os07g36140 12007.m079709 protein histone H2A.3, putative, expressed no original description 	T
21.2.1	redox.ascorbate and glutathione.ascorbate	MDP0000241173	 moderately similar to ( 325) AT1G07890 | Symbols: APX1, CS1 | APX1 (ascorbate peroxidase 1); L-ascorbate peroxidase | chr1:2438005-2439435 FORWARDmoderately similar to ( 327) APX1_PEA L-ascorbate peroxidase, cytosolic (EC 1.11.1.11) (AP) (PsAPx01) - Pisum sativum (Garden pea)moderately similar to ( 318) loc_os07g49400 12007.m29412 protein OsAPx2 - Cytosolic Ascorbate Peroxidase encoding gene, expressed  no original description 	T
29.5.11.4.3.2	protein.degradation.ubiquitin.E3.SCF.FBOX	MDP0000243435	 weakly similar to ( 164) AT4G24210 | Symbols: SLY1 | SLY1 (SLEEPY1) | chr4:12563658-12564113 FORWARDweakly similar to ( 106) GID2_ORYSA F-box protein GID2 (Gibberellin-insensitive dwarf protein 2) (Protein GIBBERELLIN INSENSITIVE DWARF2) - Oryza sativa (Rice)very weakly similar to (57.4) loc_os03g10040 12003.m06458 protein F-box protein GID2, putative, expressed  no original description 	T
20.1	stress.biotic	MDP0000204533	 weakly similar to ( 108) AT2G02100 | Symbols: LCR69, PDF2.2 | LCR69 (LOW-MOLECULAR-WEIGHT CYSTEINE-RICH 69); peptidase inhibitor | chr2:528397-528885 FORWARDweakly similar to ( 107) DEF2_CAPAN Defensin J1-2 precursor - Capsicum annuum (Bell pepper)weakly similar to ( 102) loc_os03g03810 12003.m05913 protein flower-specific gamma-thionin precursor, putative, expressed no original description 	T
21.4	redox.glutaredoxins	MDP0000277069	 highly similar to ( 641) AT3G11920 | Symbols:  | glutaredoxin-related | chr3:3772311-3774887 FORWARDhighly similar to ( 615) loc_os10g34170 12010.m06246 protein domain found in Dishevelled, Egl-10, and Pleckstrin family protein, expressed  no original description 	T
35.2	not assigned.unknown	MDP0000205893	 moderately similar to ( 431) AT1G11915 | Symbols:  | unknown protein | chr1:4021830-4023084 FORWARDmoderately similar to ( 326) loc_os06g25950 12006.m07242 protein expressed protein   no original description 	T
30.5	signalling.G-proteins	MDP0000248995	 highly similar to ( 765) AT2G44100 | Symbols: ATGDI1, AT-GDI1 | ATGDI1 (ARABIDOPSIS THALIANA GUANOSINE NUCLEOTIDE DIPHOSPHATE DISSOCIATION INHIBITOR 1); RAB GDP-dissociation inhibitor | chr2:18241804-18244731 FORWARDhighly similar to ( 796) loc_os05g23860 12005.m06680 protein rab GDP dissociation inhibitor alpha, putative, expressedMSP1_C  no original description 	T
35.2	not assigned.unknown	MDP0000285868	 weakly similar to ( 177) AT5G13140 | Symbols:  | FUNCTIONS IN: molecular_function unknown; INVOLVED IN: biological_process unknown; EXPRESSED IN: 22 plant structures; EXPRESSED DURING: 13 growth stages; CONTAINS InterPro DOMAIN/s: Pollen Ole e 1 allergen and extensin (InterPro:IPR006041); BEST Arabidopsis thaliana protein match is: unknown protein (TAIR:AT3G26960.1); Has 90 Blast hits to 85 proteins in 14 species: Archae - 0; Bacteria - 0; Metazoa - 5; Fungi - 0; Plants - 83; Viruses - 0; Other Eukaryotes - 2 (source: NCBI BLink). | chr5:4170688-4171744 REVERSEweakly similar to ( 137) loc_os09g32988 12009.m06392 protein pollen proteins Ole e I family protein, expressed no original description 	T
30.5	signalling.G-proteins	MDP0000177829	 moderately similar to ( 285) AT4G35750 | Symbols:  | Rho-GTPase-activating protein-related | chr4:16940865-16941674 REVERSEweakly similar to ( 188) loc_os04g47330 12004.m09679 protein cellular retinaldehyde-binding/triple function, C-terminal, putative, expressed  no original description 	T
23.1.1.1	nucleotide metabolism.synthesis.pyrimidine.carbamoyl phosphate synthetase	MDP0000280382	 highly similar to ( 528) AT3G27740 | Symbols: CARA | CARA (CARBAMOYL PHOSPHATE SYNTHETASE A); carbamoyl-phosphate synthase (glutamine-hydrolyzing)/ carbamoyl-phosphate synthase/ catalytic | chr3:10281470-10283792 REVERSEhighly similar to ( 511) loc_os02g47850 12002.m33834 protein carbamoyl-phosphate synthase small chain, putative, expressed  no original description 	T
27.3.29	RNA.regulation of transcription.TCP transcription factor family	MDP0000247249	 moderately similar to ( 444) AT3G55620 | Symbols: emb1624 | emb1624 (embryo defective 1624); ribosome binding / translation initiation factor | chr3:20634581-20636312 FORWARDmoderately similar to ( 337) IF6_BETVU Eukaryotic translation initiation factor 6 (eIF-6) (Fragment) - Beta vulgaris (Sugar beet)moderately similar to ( 447) loc_os07g44620 12007.m29287 protein eukaryotic translation initiation factor 6, putative, expressed   no original description 	T
35.2	not assigned.unknown	MDP0000263282	 moderately similar to ( 261) AT3G23910 | Symbols:  | unknown protein | chr3:8636594-8639344 REVERSEweakly similar to ( 142) loc_os04g31050 12004.m08209 protein expressed protein  no original description 	T
30.5	signalling.G-proteins	MDP0000253733	 moderately similar to ( 326) AT5G06120 | Symbols:  | Ran-binding protein, putative | chr5:1844797-1852601 FORWARDmoderately similar to ( 312) loc_os03g42050 12003.m09263 protein exportin-7, putative, expressed no original description 	T
30.1.2	signalling.in sugar and nutrient physiology.pyruvate dehydrogenase kinase	MDP0000703824	 moderately similar to ( 307) AT3G06483 | Symbols: PDK, ATPDHK | PDK (PYRUVATE DEHYDROGENASE KINASE); ATP binding / histidine phosphotransfer kinase/ pyruvate dehydrogenase (acetyl-transferring) kinase | chr3:1990727-1992581 REVERSEmoderately similar to ( 285) loc_os07g44330 12007.m29283 protein protein kinase, mitochondrial precursor, putative, expressed no original description 	T
30.5	signalling.G-proteins	MDP0000269288	 weakly similar to ( 187) AT3G15680 | Symbols:  | zinc finger (Ran-binding) family protein | chr3:5315437-5316048 FORWARDweakly similar to ( 164) loc_os02g10920 12002.m100134 protein RNA-binding protein cabeza, putative, expressed  no original description 	T
29.5.11.4.2	protein.degradation.ubiquitin.E3.RING	MDP0000183640	 very weakly similar to (65.1) AT1G24580 | Symbols:  | zinc finger (C3HC4-type RING finger) family protein | chr1:8710232-8710573 FORWARDvery weakly similar to (76.6) loc_os08g06090 12008.m04748 protein RING zinc finger protein-like, putative, expressed no original description 	T
29.4	protein.postranslational modification	MDP0000203314	 highly similar to ( 533) AT2G26700 | Symbols: PID2 | PID2 (PINOID2); ATP binding / kinase/ protein kinase | chr2:11368613-11370951 FORWARDmoderately similar to ( 389) G11A_ORYSA Protein kinase G11A (EC 2.7.11.1) - Oryza sativa (Rice)moderately similar to ( 498) loc_os01g07940 12001.m07419 protein protein kinase PVPK-1, putative, expressed   no original description 	T
35.1	not assigned.no ontology	MDP0000278323	 moderately similar to ( 470) AT4G11570 | Symbols:  | haloacid dehalogenase-like hydrolase family protein | chr4:7004743-7005864 FORWARDmoderately similar to ( 390) loc_os09g39560 12009.m06894 protein genetic modifier, putative, expressed  no original description 	T
35.2	not assigned.unknown	MDP0000218380	  COG4907  no original description 	T
27.3.67	RNA.regulation of transcription.putative transcription regulator	MDP0000010661	 moderately similar to ( 241) AT1G44770 | Symbols:  | unknown protein | chr1:16907834-16909409 REVERSEweakly similar to ( 146) loc_os03g49490 12003.m09948 protein expressed protein  no original description 	T
29.5.11.20	protein.degradation.ubiquitin.proteasom	MDP0000211280	 highly similar to ( 629) AT4G24820 | Symbols:  | 26S proteasome regulatory subunit, putative (RPN7) | chr4:12790471-12792599 REVERSEhighly similar to ( 617) PSMD6_ORYSA 26S proteasome non-ATPase regulatory subunit 6 (26S proteasome regulatory particle non-ATPase subunit 7) (OsRPN7) - Oryza sativa (Rice)highly similar to ( 617) loc_os04g40850 12004.m35346 protein 26S proteasome non-ATPase regulatory subunit 6, putative, expressed  MSP1_C  no original description 	T
35.2	not assigned.unknown	MDP0000255764	 moderately similar to ( 248) AT5G03560 | Symbols:  | nucleobase:cation symporter | chr5:901452-902719 REVERSEmoderately similar to ( 249) loc_os08g44650 12008.m08437 protein expressed protein no original description 	T
4.2.4	glycolysis.plastid branch.phosphofructokinase (PFK)	MDP0000895817	 highly similar to ( 617) AT1G20950 | Symbols:  | pyrophosphate--fructose-6-phosphate 1-phosphotransferase-related / pyrophosphate-dependent 6-phosphofructose-1-kinase-related | chr1:7297467-7301336 REVERSEhighly similar to ( 644) PFPA_RICCO Pyrophosphate--fructose 6-phosphate 1-phosphotransferase alpha subunit (EC 2.7.1.90) (PFP) (6-phosphofructokinase, pyrophosphate dependent) (Pyrophosphate-dependent 6-phosphofructose-1-kinase) (PPi-PFK) - Ricinus communis (Castor bean)highly similar to ( 613) loc_os02g48360 12002.m100340 protein pyrophosphate--fructose 6-phosphate 1-phosphotransferase alpha subunit, putative, expressed no original description 	T
10.8.1	cell wall.pectin*esterases.PME	MDP0000734600	 highly similar to ( 674) AT3G05620 | Symbols:  | pectinesterase family protein | chr3:1629658-1631766 REVERSEmoderately similar to ( 478) PME_PRUPE Pectinesterase PPE8B precursor (EC 3.1.1.11) (Pectin methylesterase) (PE) - Prunus persica (Peach)highly similar to ( 504) loc_os01g15039 12001.m08104 protein pectinesterase PPE8B precursor, putative, expressed   no original description 	T
3.6	minor CHO metabolism.callose	MDP0000229527	 nearly identical (3017) AT5G13000 | Symbols: ATGSL12, gsl12 | ATGSL12 (glucan synthase-like 12); 1,3-beta-glucan synthase/ transferase, transferring glycosyl groups | chr5:4110445-4121202 REVERSEnearly identical (2627) loc_os03g03610 12003.m34995 protein CALS1, putative, expressed   no original description 	T
10.6.2	cell wall.degradation.mannan-xylose-arabinose-fucose	MDP0000608429	 nearly identical (1064) AT3G19620 | Symbols:  | glycosyl hydrolase family 3 protein | chr3:6815613-6818308 REVERSEmoderately similar to ( 383) XYNB_PRUPE Putative beta-D-xylosidase (EC 3.2.1.-) (PpAz152) (Fragment) - Prunus persica (Peach)highly similar to ( 826) loc_os04g54810 12004.m10373 protein beta-D-xylosidase, putative, expressed  no original description 	T
34.10	transport.nucleotides	MDP0000127942	 moderately similar to ( 299) AT4G18210 | Symbols: ATPUP10 | ATPUP10; purine transmembrane transporter | chr4:10076175-10077495 FORWARDmoderately similar to ( 247) loc_os02g46380 12002.m33436 protein ATPUP11, putative, expressed   no original description 	T
31.3	cell.cycle	MDP0000890849	 moderately similar to ( 221) AT3G21870 | Symbols: CYCP2;1 | CYCP2;1 (cyclin p2;1); cyclin-dependent protein kinase | chr3:7703927-7704813 REVERSEweakly similar to ( 139) loc_os05g33040 12005.m083714 protein PREG-like protein, putative, expressed no original description 	T
26.21	misc.protease inhibitor/seed storage/lipid transfer protein (LTP) family protein	MDP0000835003	 very weakly similar to (90.1) AT3G18280 | Symbols:  | protease inhibitor/seed storage/lipid transfer protein (LTP) family protein | chr3:6267102-6267392 FORWARDvery weakly similar to (82.0) NLTP_VIGUN Probable nonspecific lipid-transfer protein AKCS9 precursor (LTP) - Vigna unguiculata (Cowpea)very weakly similar to (87.0) loc_os05g47700 12005.m08842 protein nonspecific lipid-transfer protein precursor, putative, expressed no original description 	T
35.2	not assigned.unknown	MDP0000243444	 moderately similar to ( 335) AT5G24840 | Symbols:  | tRNA (guanine-N7-)-methyltransferase | chr5:8533897-8534652 REVERSEmoderately similar to ( 321) loc_os06g12990 12006.m06015 protein tRNA (guanine-N(1)-)-methyltransferase, putative, expressed  no original description 	T
27.3.6	RNA.regulation of transcription.bHLH,Basic Helix-Loop-Helix family	MDP0000439540	 moderately similar to ( 288) AT4G00050 | Symbols: UNE10 | UNE10 (unfertilized embryo sac 10); DNA binding / transcription factor | chr4:17863-19848 FORWARDvery weakly similar to (56.2) ARRS_MAIZE Anthocyanin regulatory R-S protein - Zea mays (Maize)weakly similar to ( 102) loc_os10g40740 12010.m06842 protein long hypocotyl in far-red 1, putative, expressed   no original description 	T
20.2.99	stress.abiotic.unspecified	MDP0000894544	 weakly similar to ( 132) AT1G01170 | Symbols:  | ozone-responsive stress-related protein, putative | chr1:74105-74443 REVERSEweakly similar to ( 123) loc_os06g02470 12006.m04980 protein ATOZI1, putative, expressed no original description 	T
20.2	stress.abiotic	MDP0000776042	 moderately similar to ( 279) AT3G07700 | Symbols:  | ABC1 family protein | chr3:2459696-2463241 REVERSEmoderately similar to ( 255) loc_os09g07660 12009.m059938 protein ubiquinone biosynthesis protein ubiB, putative, expressed no original description 	T
34.16	transport.ABC transporters and multidrug resistance systems	MDP0000776042	 moderately similar to ( 279) AT3G07700 | Symbols:  | ABC1 family protein | chr3:2459696-2463241 REVERSEmoderately similar to ( 255) loc_os09g07660 12009.m059938 protein ubiquinone biosynthesis protein ubiB, putative, expressed no original description 	T
29.2.1.2.2.13	protein.synthesis.ribosomal protein.eukaryotic.60S subunit.L13	MDP0000630768	 moderately similar to ( 311) AT3G49010 | Symbols: ATBBC1, BBC1, RSU2 | ATBBC1 (ARABIDOPSIS THALIANA BREAST BASIC CONSERVED 1); structural constituent of ribosome | chr3:18166971-18168047 REVERSEmoderately similar to ( 306) RL131_BRANA 60S ribosomal protein L13-1 (Cold-induced protein C24A) - Brassica napus (Rape)moderately similar to ( 308) loc_os03g37970 12003.m34891 protein 60S ribosomal protein L13-2, putative, expressed no original description 	T
27.3.6	RNA.regulation of transcription.bHLH,Basic Helix-Loop-Helix family	MDP0000124019	 moderately similar to ( 231) AT5G48560 | Symbols:  | basic helix-loop-helix (bHLH) family protein | chr5:19684160-19686871 FORWARDmoderately similar to ( 233) loc_os01g68700 12001.m12972 protein BHLH transcription factor, putative, expressed  no original description 	T
26.22	misc.short chain dehydrogenase/reductase (SDR)	MDP0000305808	 moderately similar to ( 412) AT1G67730 | Symbols: YBR159, KCR1 | YBR159; ketoreductase/ oxidoreductase | chr1:25391676-25393365 FORWARDvery weakly similar to (60.5) SDR1_PICAB Short-chain type dehydrogenase/reductase (EC 1.-.-.-) - Picea abies (Norway spruce) (Picea excelsa)moderately similar to ( 382) loc_os02g38440 12002.m08932 protein steroid dehydrogenase KIK-I, putative, expressed   no original description 	T
11.5.2	lipid metabolism.glyceral metabolism.Glycerol-3-phosphate dehydrogenase (NAD+)	MDP0000391371	 highly similar to ( 813) AT2G41540 | Symbols: GPDHC1 | GPDHC1; NAD or NADH binding / glycerol-3-phosphate dehydrogenase (NAD+) | chr2:17326801-17328654 FORWARDhighly similar to ( 779) loc_os01g71280 12001.m13171 protein glycerol-3-phosphate dehydrogenase, putative, expressed    no original description 	T
35.2	not assigned.unknown	MDP0000325334	  no original description 	T
17.1.3	hormone metabolism.abscisic acid.induced-regulated-responsive-activated	MDP0000157647	 moderately similar to ( 237) AT1G74520 | Symbols: ATHVA22A | ATHVA22A | chr1:28008109-28009156 REVERSEvery weakly similar to ( 100) HVA22_HORVU Protein HVA22 - Hordeum vulgare (Barley)moderately similar to ( 205) loc_os06g12220 12006.m05939 protein HVA22-like protein a, putative, expressed no original description 	T
28.1.3	DNA.synthesis/chromatin structure.histone	MDP0000309757	 weakly similar to ( 189) AT2G38810 | Symbols: HTA8 | HTA8 (HISTONE H2A 8); DNA binding | chr2:16219444-16220679 REVERSEweakly similar to ( 186) H2AV1_ORYSA Probable histone H2A variant 1 - Oryza sativa (Rice)weakly similar to ( 186) loc_os03g06670 12003.m06185 protein histone H2A variant 1, putative, expressed   no original description 	T
13.1.4.1	amino acid metabolism.synthesis.branched chain group.common	MDP0000781460	 highly similar to ( 955) AT3G58610 | Symbols:  | ketol-acid reductoisomerase | chr3:21671561-21674639 FORWARDhighly similar to ( 947) ILV5_PEA Ketol-acid reductoisomerase, chloroplast precursor (EC 1.1.1.86) (Acetohydroxy-acid reductoisomerase) (Alpha-keto-beta-hydroxylacil reductoisomerase) - Pisum sativum (Garden pea)highly similar to ( 911) loc_os01g46380 12001.m10836 protein ketol-acid reductoisomerase, chloroplast precursor, putative, expressed  no original description 	T
27.4	RNA.RNA binding	MDP0000123012	 moderately similar to ( 388) AT5G19350 | Symbols:  | RNA-binding protein 45 (RBP45), putative | chr5:6518978-6521295 FORWARDvery weakly similar to (83.6) ROC1_SPIOL 28 kDa ribonucleoprotein, chloroplast (28RNP) - Spinacia oleracea (Spinach)moderately similar to ( 415) loc_os03g07800 12003.m06292 protein RNA-binding post-transcriptional regulator csx1, putative, expressed   Sporozoite_P67  no original description 	T
29.4	protein.postranslational modification	MDP0000151578	 nearly identical (1742) AT3G13530 | Symbols: MAPKKK7, MAP3KE1 | MAPKKK7; ATP binding / binding / kinase/ protein kinase/ protein serine/threonine kinase/ protein tyrosine kinase | chr3:4411934-4419320 REVERSEvery weakly similar to (88.2) CIPK1_ORYSA CIPK-like protein 1 (EC 2.7.11.1) (OsCK1) - Oryza sativa (Rice)nearly identical (1488) loc_os04g56530 12004.m10542 protein MAPKKK7, putative, expressed   no original description 	T
13.1.3.4.1	amino acid metabolism.synthesis.aspartate family.methionine.cystathionine gamma-synthase	MDP0000945657	 highly similar to ( 766) AT3G01120 | Symbols: MTO1, CGS, ATCYS1, CGS1 | MTO1 (METHIONINE OVERACCUMULATION 1); cystathionine gamma-synthase | chr3:39234-41865 REVERSEhighly similar to ( 621) loc_os03g25940 12003.m78868 protein cystathionine beta-lyase, putative, expressed  no original description 	T
27.3.12	RNA.regulation of transcription.C3H zinc finger family	MDP0000706016	 moderately similar to ( 386) AT1G32360 | Symbols:  | zinc finger (CCCH-type) family protein | chr1:11673325-11675162 FORWARDmoderately similar to ( 399) loc_os08g06330 12008.m04772 protein zinc finger C-x8-C-x5-C-x3-H type family protein, expressed  no original description 	T
29.5.11.4.2	protein.degradation.ubiquitin.E3.RING	MDP0000706016	 moderately similar to ( 386) AT1G32360 | Symbols:  | zinc finger (CCCH-type) family protein | chr1:11673325-11675162 FORWARDmoderately similar to ( 399) loc_os08g06330 12008.m04772 protein zinc finger C-x8-C-x5-C-x3-H type family protein, expressed  no original description 	T
26.13	misc.acid and other phosphatases	MDP0000226193	 moderately similar to ( 338) AT5G50400 | Symbols: ATPAP27, PAP27 | PAP27 (PURPLE ACID PHOSPHATASE 27); acid phosphatase/ protein serine/threonine phosphatase | chr5:20523575-20526231 REVERSEmoderately similar to ( 205) NPP_HORVU Nucleotide pyrophosphatase/phosphodiesterase (EC 3.-.-.-) (Fragments) - Hordeum vulgare (Barley)highly similar to ( 769) loc_os01g58640 12001.m12006 protein nucleotide pyrophosphatase/phosphodiesterase, putative, expressed no original description 	T
33.99	development.unspecified	MDP0000313704	 nearly identical (1327) AT4G34450 | Symbols:  | coatomer gamma-2 subunit, putative / gamma-2 coat protein, putative / gamma-2 COP, putative | chr4:16471956-16476795 FORWARDhighly similar to ( 677) GBB1_TOBAC Guanine nucleotide-binding protein subunit beta 1 - Nicotiana tabacum (Common tobacco)nearly identical (1322) loc_os07g10150 12007.m05470 protein coatomer subunit gamma, putative, expressed   no original description 	T
23.3.1.1	nucleotide metabolism.salvage.phosphoribosyltransferases.aprt	MDP0000159559	 moderately similar to ( 316) AT5G11160 | Symbols: APT5 | APT5 (Adenine phosphoribosyltransferase 5); adenine phosphoribosyltransferase | chr5:3550774-3551986 FORWARDmoderately similar to ( 253) APT1_WHEAT Adenine phosphoribosyltransferase 1 (EC 2.4.2.7) (APRT 1) - Triticum aestivum (Wheat)moderately similar to ( 282) loc_os04g42520 12004.m78967 protein adenine phosphoribosyltransferase 2, putative, expressed  no original description 	T
29.2.1.2.1.31	protein.synthesis.ribosomal protein.eukaryotic.40S subunit.SA	MDP0000708887	 moderately similar to ( 410) AT1G72370 | Symbols: P40, AP40, RP40, RPSAA | P40; structural constituent of ribosome | chr1:27243148-27244842 REVERSEmoderately similar to ( 437) RSSA_SOYBN 40S ribosomal protein SA (p40) - Glycine max (Soybean)moderately similar to ( 411) loc_os03g08440 12003.m35048 protein 40S ribosomal protein SA, putative, expressed  no original description 	T
29.3.4.99	protein.targeting.secretory pathway.unspecified	MDP0000291148	 highly similar to ( 787) AT5G46630 | Symbols:  | clathrin adaptor complexes medium subunit family protein | chr5:18920580-18923060 FORWARDhighly similar to ( 785) loc_os02g46510 12002.m09688 protein AP-2 complex subunit mu, putative, expressed  no original description 	T
29.5.9	protein.degradation.AAA type	MDP0000322270	 nearly identical (1018) AT3G19740 | Symbols:  | ATP binding / ATPase/ nucleoside-triphosphatase/ nucleotide binding | chr3:6855944-6862930 REVERSEweakly similar to ( 143) CDC48_SOYBN Cell division cycle protein 48 homolog (Valosin-containing protein homolog) (VCP) - Glycine max (Soybean)highly similar to ( 904) loc_os06g12160 12006.m05933 protein ATP binding protein, putative, expressed   no original description 	T
35.2	not assigned.unknown	MDP0000179040	 moderately similar to ( 448) AT1G64980 | Symbols:  | unknown protein | chr1:24137840-24138619 REVERSEmoderately similar to ( 345) loc_os07g23120 12007.m29140 protein expressed protein   no original description 	T
35.2	not assigned.unknown	MDP0000362009	 very weakly similar to (75.1) AT1G26355 | Symbols: SP1L1 | SP1L1 (SPIRAL1-LIKE1) | chr1:9117950-9118611 REVERSEvery weakly similar to (68.2) loc_os03g30430 12003.m34882 protein NAP16kDa protein, putative, expressed  no original description 	T
34.99	transport.misc	MDP0000141986	 highly similar to ( 620) AT1G58340 | Symbols: ZF14 | ZF14; antiporter/ drug transporter/ transporter | chr1:21653162-21655117 FORWARDmoderately similar to ( 461) loc_os03g12790 12003.m06727 protein antiporter/ drug transporter/ transporter, putative, expressed   no original description 	T
18.13	Co-factor and vitamine metabolism.isochorismatase	MDP0000135890	 moderately similar to ( 242) AT3G16190 | Symbols:  | isochorismatase hydrolase family protein | chr3:5489883-5491195 REVERSEmoderately similar to ( 239) loc_os02g17640 12002.m07007 protein isochorismatase family protein rutB, putative, expressed no original description 	T
20.1.7	stress.biotic.PR-proteins	MDP0000286425	 moderately similar to ( 430) AT5G66900 | Symbols:  | disease resistance protein (CC-NBS-LRR class), putative | chr5:26714931-26717757 REVERSEmoderately similar to ( 321) loc_os12g39620 12012.m073872 protein disease resistance protein, putative, expressed  no original description 	T
35.2	not assigned.unknown	MDP0000149922	     no original description 	T
35.2	not assigned.unknown	MDP0000297920	 highly similar to ( 892) AT2G42700 | Symbols:  | FUNCTIONS IN: molecular_function unknown; INVOLVED IN: vesicle-mediated transport, vesicle docking during exocytosis; LOCATED IN: cellular_component unknown; EXPRESSED IN: 22 plant structures; EXPRESSED DURING: 14 growth stages; CONTAINS InterPro DOMAIN/s: Sec1-like protein (InterPro:IPR001619); Has 95 Blast hits to 92 proteins in 33 species: Archae - 0; Bacteria - 0; Metazoa - 70; Fungi - 0; Plants - 20; Viruses - 0; Other Eukaryotes - 5 (source: NCBI BLink). | chr2:17778464-17782034 FORWARDhighly similar to ( 733) loc_os11g06810 12011.m04878 protein expressed protein  no original description 	T
20.2	stress.abiotic	MDP0000129126	 moderately similar to ( 346) AT5G52060 | Symbols: ATBAG1 | ATBAG1 (ARABIDOPSIS THALIANA BCL-2-ASSOCIATED ATHANOGENE 1); protein binding | chr5:21152449-21153741 REVERSEmoderately similar to ( 290) loc_os09g35630 12009.m06514 protein protein binding protein, putative, expressed  no original description 	T
17.2.3	hormone metabolism.auxin.induced-regulated-responsive-activated	MDP0000312758	 moderately similar to ( 319) AT1G78915 | Symbols:  | binding | chr1:29667729-29671081 REVERSEvery weakly similar to (62.0) ARG7_PHAAU Indole-3-acetic acid-induced protein ARG7 - Phaseolus aureus (Mung bean) (Vigna radiata)weakly similar to ( 192) loc_os07g18720 12007.m06311 protein TPR Domain containing protein, expressed  no original description 	T
2.2.1.3.1	major CHO metabolism.degradation.sucrose.invertases.neutral	MDP0000652278	 highly similar to ( 894) AT1G56560 | Symbols:  | beta-fructofuranosidase, putative / invertase, putative / saccharase, putative / beta-fructosidase, putative | chr1:21192593-21194948 FORWARDhighly similar to ( 816) loc_os03g20020 12003.m07415 protein beta-fructofuranosidase, putative, expressed  no original description 	T
35.2	not assigned.unknown	MDP0000485534	 moderately similar to ( 382) AT3G26750 | Symbols:  | unknown protein | chr3:9840139-9841719 REVERSEmoderately similar to ( 292) loc_os02g03820 12002.m05731 protein expressed protein  no original description 	T
34.99	transport.misc	MDP0000161125	 highly similar to ( 594) AT1G33110 | Symbols:  | MATE efflux family protein | chr1:12005084-12008040 FORWARDhighly similar to ( 572) loc_os11g03240 12011.m04520 protein transparent testa 12 protein, putative, expressed   no original description 	T
27.4	RNA.RNA binding	MDP0000200990	 weakly similar to ( 179) AT4G10110 | Symbols:  | RNA recognition motif (RRM)-containing protein | chr4:6309606-6310809 REVERSEweakly similar to ( 155) loc_os05g35274 12005.m083726 protein RNA recognition motif-containing protein, putative, expressed MSP1_C  no original description 	T
26.10	misc.cytochrome P450	MDP0000286933	 highly similar to ( 638) AT5G07990 | Symbols: TT7, CYP75B1, D501 | TT7 (TRANSPARENT TESTA 7); flavonoid 3'-monooxygenase/ oxygen binding | chr5:2560437-2562859 FORWARDhighly similar to ( 660) F3PH_PETHY Flavonoid 3'-monooxygenase (EC 1.14.13.21) (Flavonoid 3'-hydroxylase) (Cytochrome P450 75B2) - Petunia hybrida (Petunia)highly similar to ( 538) loc_os10g17260 12010.m04813 protein flavonoid 3-monooxygenase, putative, expressed   no original description 	T
20.1	stress.biotic	MDP0000216636	 weakly similar to ( 108) AT2G02100 | Symbols: LCR69, PDF2.2 | LCR69 (LOW-MOLECULAR-WEIGHT CYSTEINE-RICH 69); peptidase inhibitor | chr2:528397-528885 FORWARDweakly similar to ( 107) DEF2_CAPAN Defensin J1-2 precursor - Capsicum annuum (Bell pepper)weakly similar to ( 102) loc_os03g03810 12003.m05913 protein flower-specific gamma-thionin precursor, putative, expressed no original description 	T
27.3.6	RNA.regulation of transcription.bHLH,Basic Helix-Loop-Helix family	MDP0000176749	 moderately similar to ( 304) AT4G02590 | Symbols: UNE12 | UNE12 (unfertilized embryo sac 12); DNA binding / transcription factor | chr4:1137968-1140117 REVERSEweakly similar to ( 196) loc_os03g58330 12003.m10743 protein BHLH transcription factor, putative, expressed  no original description 	T
26.2	misc.UDP glucosyl and glucoronyl transferases	MDP0000813554	 weakly similar to ( 155) AT4G38040 | Symbols:  | exostosin family protein | chr4:17867501-17869131 FORWARDweakly similar to ( 164) loc_os02g09430 12002.m06240 protein exostosin-like, putative, expressed   no original description 	T
35.2	not assigned.unknown	MDP0000279658	 moderately similar to ( 375) AT3G01670 | Symbols:  | unknown protein | chr3:247288-250261 FORWARD   no original description 	T
20.2.1	stress.abiotic.heat	MDP0000630316	 weakly similar to ( 143) AT5G59720 | Symbols: HSP18.2 | HSP18.2 (heat shock protein 18.2) | chr5:24062632-24063117 FORWARDweakly similar to ( 162) HSP11_PEA 18.1 kDa class I heat shock protein (HSP 18.1) - Pisum sativum (Garden pea)weakly similar to ( 147) loc_os03g16030 12003.m07040 protein 17.4 kDa class I heat shock protein 3, putative, expressed  no original description 	T
30.3	signalling.calcium	MDP0000311132	 moderately similar to ( 222) AT5G64816 | Symbols:  | unknown protein | chr5:25913487-25913879 FORWARDmoderately similar to ( 223) loc_os01g11414 12001.m07759 protein calcium ion binding protein, putative, expressed  no original description 	T
35.2	not assigned.unknown	MDP0000368503	 weakly similar to ( 104) AT5G48385 | Symbols:  | FUNCTIONS IN: molecular_function unknown; INVOLVED IN: biological_process unknown; LOCATED IN: cellular_component unknown; CONTAINS InterPro DOMAIN/s: Frigida-like (InterPro:IPR012474); BEST Arabidopsis thaliana protein match is: hydroxyproline-rich glycoprotein family protein (TAIR:AT3G22440.1); Has 987 Blast hits to 921 proteins in 78 species: Archae - 0; Bacteria - 16; Metazoa - 58; Fungi - 7; Plants - 873; Viruses - 0; Other Eukaryotes - 33 (source: NCBI BLink). | chr5:19609471-19611712 FORWARDweakly similar to ( 124) loc_os09g07380 12009.m04007 protein ABI3-interacting protein 2, putative, expressed no original description 	T
20.2.99	stress.abiotic.unspecified	MDP0000903066	 weakly similar to ( 125) AT1G01170 | Symbols:  | ozone-responsive stress-related protein, putative | chr1:74105-74443 REVERSEweakly similar to ( 115) loc_os06g02470 12006.m04980 protein ATOZI1, putative, expressed no original description 	T
35.2	not assigned.unknown	MDP0000212810	 highly similar to ( 508) AT3G62370 | Symbols:  | unknown protein | chr3:23080789-23083234 FORWARDmoderately similar to ( 459) loc_os03g19580 12003.m35183 protein expressed protein   no original description 	T
12.4	N-metabolism.misc	MDP0000201435	 weakly similar to ( 196) AT5G67220 | Symbols:  | nitrogen regulation family protein | chr5:26820276-26821926 REVERSEweakly similar to ( 198) loc_os06g49870 12006.m09511 protein tRNA-dihydrouridine synthase B, putative, expressed no original description 	T
35.2	not assigned.unknown	MDP0000364685	  TrbL  no original description 	T
34.99	transport.misc	MDP0000344085	 very weakly similar to (95.9) AT1G76530 | Symbols:  | auxin efflux carrier family protein | chr1:28718407-28720274 FORWARDvery weakly similar to (95.1) loc_os09g31478 12009.m06269 protein auxin hydrogen symporter, putative, expressed no original description 	T
35.2	not assigned.unknown	MDP0000769596	 weakly similar to ( 103) AT1G27435 | Symbols:  | unknown protein | chr1:9527956-9528814 REVERSE no original description 	T
35.1	not assigned.no ontology	MDP0000239405	 moderately similar to ( 293) AT1G10950 | Symbols:  | endomembrane protein 70, putative | chr1:3659322-3663622 FORWARDmoderately similar to ( 274) loc_os11g07910 12011.m28746 protein transmembrane 9 superfamily protein member 3 precursor, putative, expressed no original description 	T
23.4.99	nucleotide metabolism.phosphotransfer and pyrophosphatases.misc	MDP0000130821	 moderately similar to ( 379) AT5G09650 | Symbols: AtPPa6 | AtPPa6 (Arabidopsis thaliana pyrophosphorylase 6); inorganic diphosphatase/ pyrophosphatase | chr5:2991331-2993117 REVERSEmoderately similar to ( 271) IPYR1_CHLRE Soluble inorganic pyrophosphatase 1, chloroplast precursor (EC 3.6.1.1) (Pyrophosphate phospho-hydrolase 1) (PPase 1) - Chlamydomonas reinhardtiimoderately similar to ( 349) loc_os02g52940 12002.m100369 protein inorganic pyrophosphatase, putative, expressed  no original description 	T
26.3.2	misc.gluco-, galacto- and mannosidases.beta-galactosidase	MDP0000127542	 highly similar to ( 672) AT3G52840 | Symbols: BGAL2 | BGAL2 (beta-galactosidase 2); beta-galactosidase/ catalytic/ cation binding | chr3:19581244-19586097 FORWARDhighly similar to ( 834) BGAL_MALDO Beta-galactosidase precursor (EC 3.2.1.23) (Lactase) (Acid beta-galactosidase) (Exo-(1-->4)-beta-D-galactanase) - Malus domestica (Apple) (Malus sylvestris)highly similar to ( 623) loc_os01g39830 12001.m10256 protein beta-galactosidase precursor, putative, expressed  no original description 	T
35.1	not assigned.no ontology	MDP0000147542	 highly similar to ( 729) AT1G72480 | Symbols:  | FUNCTIONS IN: molecular_function unknown; INVOLVED IN: biological_process unknown; LOCATED IN: Golgi apparatus; EXPRESSED IN: 22 plant structures; EXPRESSED DURING: 13 growth stages; CONTAINS InterPro DOMAIN/s: Transmembrane receptor, eukaryota (InterPro:IPR009637); BEST Arabidopsis thaliana protein match is: unknown protein (TAIR:AT2G01070.1); Has 422 Blast hits to 420 proteins in 121 species: Archae - 0; Bacteria - 0; Metazoa - 208; Fungi - 101; Plants - 83; Viruses - 0; Other Eukaryotes - 30 (source: NCBI BLink). | chr1:27286304-27288339 REVERSEvery weakly similar to (68.6) PPOC_TOBAC Protoporphyrinogen oxidase, chloroplast precursor (EC 1.3.3.4) (PPO I) (Protoporphyrinogen IX oxidase isozyme I) (PPX I) - Nicotiana tabacum (Common tobacco)highly similar to ( 657) loc_os09g26830 12009.m05835 protein TMEM87A protein, putative, expressed    no original description 	T
34.9	transport.metabolite transporters at the mitochondrial membrane	MDP0000216217	 highly similar to ( 620) AT3G08580 | Symbols: AAC1 | AAC1 (ADP/ATP CARRIER 1); ATP:ADP antiporter/ binding | chr3:2605706-2607030 REVERSEhighly similar to ( 638) ADT1_GOSHI ADP,ATP carrier protein 1, mitochondrial precursor (ADP/ATP translocase 1) (Adenine nucleotide translocator 1) (ANT 1) - Gossypium hirsutum (Upland cotton)highly similar to ( 570) loc_os02g48720 12002.m33839 protein ADP,ATP carrier protein, mitochondrial precursor, putative, expressed no original description 	T
16.4.2.1	secondary metabolism.N misc.betaine.betaine-aldehyde dehydrogenase	MDP0000283106	 highly similar to ( 989) AT3G66658 | Symbols: ALDH22a1 | ALDH22a1 (Aldehyde Dehydrogenase 22a1); 3-chloroallyl aldehyde dehydrogenase/ oxidoreductase | chr3:2095471-2099013 REVERSEmoderately similar to ( 214) BADH_SPIOL Betaine-aldehyde dehydrogenase, chloroplast precursor (EC 1.2.1.8) (BADH) - Spinacia oleracea (Spinach)highly similar to ( 978) loc_os07g48920 12007.m09101 protein betaine-aldehyde dehydrogenase, chloroplast precursor, putative, expressed no original description 	T
34.2	transport.sugars	MDP0000282973	 moderately similar to ( 388) AT1G61790 | Symbols:  | OST3/OST6 family protein | chr1:22814390-22815430 FORWARDvery weakly similar to (87.0) NTF2_ORYSA Nuclear transport factor 2 (NTF-2) - Oryza sativa (Rice)moderately similar to ( 363) loc_os03g36730 12003.m08792 protein oligosaccharide transporter, putative, expressed   PRK08026  no original description 	T
35.1.41	not assigned.no ontology.hydroxyproline rich proteins	MDP0000742911	 weakly similar to ( 101) AT2G18910 | Symbols:  | hydroxyproline-rich glycoprotein family protein | chr2:8193016-8194131 REVERSEvery weakly similar to (68.9) loc_os02g26370 12002.m33380 protein actin associated protein, putative, expressed  no original description 	T
26.28	misc.GDSL-motif lipase	MDP0000120560	 moderately similar to ( 409) AT1G75900 | Symbols:  | family II extracellular lipase 3 (EXL3) | chr1:28499179-28500943 FORWARDmoderately similar to ( 270) APG_BRANA Anter-specific proline-rich protein APG (Protein CEX) (Fragment) - Brassica napus (Rape)moderately similar to ( 331) loc_os10g30290 12010.m05891 protein anther-specific proline-rich protein APG precursor, putative, expressed  no original description 	T
20.1	stress.biotic	MDP0000191794	 highly similar to ( 625) AT3G14470 | Symbols:  | disease resistance protein (NBS-LRR class), putative | chr3:4857940-4861104 FORWARDhighly similar to ( 554) loc_os05g41290 12005.m08304 protein disease resistance RPP13-like protein 1, putative, expressed  no original description 	T
10.6.1	cell wall.degradation.cellulases and beta -1,4-glucanases	MDP0000062066	 highly similar to ( 938) AT5G20950 | Symbols:  | glycosyl hydrolase family 3 protein | chr5:7107609-7110775 REVERSEhighly similar to ( 921) loc_os03g53800 12003.m101132 protein periplasmic beta-glucosidase precursor, putative, expressed  no original description 	T
29.5.11.4.3.2	protein.degradation.ubiquitin.E3.SCF.FBOX	MDP0000281445	 highly similar to ( 597) AT5G49580 | Symbols:  | DNAJ heat shock N-terminal domain-containing protein | chr5:20123823-20126813 REVERSEhighly similar to ( 548) loc_os03g61730 12003.m11054 protein heat shock protein binding protein, putative, expressed  COG3889  no original description 	T
29.5.11.4.5.2	protein.degradation.ubiquitin.E3.BTB/POZ Cullin3.BTB/POZ	MDP0000187156	 moderately similar to ( 404) AT1G05690 | Symbols: BT3 | BT3 (BTB AND TAZ DOMAIN PROTEIN 3); protein binding / transcription regulator | chr1:1707809-1709132 FORWARDmoderately similar to ( 378) loc_os01g66890 12001.m43320 protein protein binding protein, putative, expressed  no original description 	T
27.3.24	RNA.regulation of transcription.MADS box transcription factor family	MDP0000904420	 weakly similar to ( 141) AT2G45660 | Symbols: AGL20, SOC1 | AGL20 (AGAMOUS-LIKE 20); transcription factor | chr2:18807799-18810193 REVERSEvery weakly similar to ( 100) MAD50_ORYSA MADS-box transcription factor 50 (OsMADS50) (Protein SUPPRESSOR OF CONSTANS OVEREXPRESSION 1-like) (OsSOC1) (Protein AGAMOUS-like 20) (RMADS208) - Oryza sativa (Rice)very weakly similar to (85.5) loc_os03g03070 12003.m05844 protein MADS-box transcription factor 50, putative, expressed no original description 	T
26.21	misc.protease inhibitor/seed storage/lipid transfer protein (LTP) family protein	MDP0000824440	 very weakly similar to (77.4) AT5G48485 | Symbols: DIR1 | DIR1 (DEFECTIVE IN INDUCED RESISTANCE 1); lipid binding / lipid transporter | chr5:19646317-19646625 REVERSEvery weakly similar to (89.7) loc_os07g18750 12007.m06314 protein PVR3-like protein, putative, expressed  no original description 	T
34.99	transport.misc	MDP0000243770	 moderately similar to ( 224) AT1G76520 | Symbols:  | auxin efflux carrier family protein | chr1:28715384-28717289 FORWARDmoderately similar to ( 203) loc_os09g31478 12009.m06269 protein auxin hydrogen symporter, putative, expressed  no original description 	T
1.2.2	PS.photorespiration.glycolate oxydase	MDP0000706975	 highly similar to ( 612) AT3G14420 | Symbols:  | (S)-2-hydroxy-acid oxidase, peroxisomal, putative / glycolate oxidase, putative / short chain alpha-hydroxy acid oxidase, putative | chr3:4821899-4823899 FORWARDhighly similar to ( 621) GOX_SPIOL Peroxisomal (S)-2-hydroxy-acid oxidase (EC 1.1.3.15) (Glycolate oxidase) (GOX) (Short chain alpha-hydroxy acid oxidase) - Spinacia oleracea (Spinach)highly similar to ( 582) loc_os07g05820 12007.m29070 protein hydroxyacid oxidase 1, putative, expressed  no original description 	T
29.5	protein.degradation	MDP0000536363	 moderately similar to ( 233) AT1G02170 | Symbols: LOL3, ATMCPB1, MCP1B, AMC1, ATMC1 | AMC1 (METACASPASE 1); cysteine-type endopeptidase | chr1:411883-413426 FORWARDmoderately similar to ( 225) loc_os03g27210 12003.m08050 protein LOL3, putative, expressed    no original description 	T
26.4.1	misc.beta 1,3 glucan hydrolases.glucan endo-1,3-beta-glucosidase	MDP0000302259	 moderately similar to ( 499) AT3G13560 | Symbols:  | glycosyl hydrolase family 17 protein | chr3:4425484-4427284 REVERSEweakly similar to ( 169) E13B_WHEAT Glucan endo-1,3-beta-glucosidase precursor (EC 3.2.1.39) ((1->3)-beta-glucan endohydrolase) ((1->3)-beta-glucanase) (Beta-1,3-endoglucanase) - Triticum aestivum (Wheat)moderately similar to ( 387) loc_os07g38930 12007.m08137 protein glucan endo-1,3-beta-glucosidase 4 precursor, putative, expressed   no original description 	T
29.2.1.2.2.537	protein.synthesis.ribosomal protein.eukaryotic.60S subunit.L37A	MDP0000286664	 weakly similar to ( 182) AT3G10950 | Symbols:  | 60S ribosomal protein L37a (RPL37aB) | chr3:3423893-3424566 FORWARDweakly similar to ( 186) RL37A_GOSHI 60S ribosomal protein L37a - Gossypium hirsutum (Upland cotton)weakly similar to ( 186) loc_os05g48320 12005.m27963 protein 60S ribosomal protein L37a, putative, expressed no original description 	T
33.99	development.unspecified	MDP0000253269	 moderately similar to ( 436) AT4G29590 | Symbols:  | methyltransferase | chr4:14512736-14514404 REVERSEmoderately similar to ( 435) loc_os01g51530 12001.m43163 protein methyltransferase, putative, expressed    no original description 	T
16.2	secondary metabolism.phenylpropanoids	MDP0000315078	 highly similar to ( 956) AT4G30210 | Symbols: ATR2, AR2 | ATR2 (ARABIDOPSIS P450 REDUCTASE 2); NADPH-hemoprotein reductase | chr4:14796900-14800578 FORWARDhighly similar to ( 963) NCPR_CATRO NADPH--cytochrome P450 reductase (EC 1.6.2.4) (CPR) (P450R) - Catharanthus roseus (Rosy periwinkle) (Madagascar periwinkle)highly similar to ( 914) loc_os09g38620 12009.m06803 protein NADPH--cytochrome P450 reductase, putative, expressed   no original description 	T
30.5	signalling.G-proteins	MDP0000434269	 moderately similar to ( 258) AT1G07140 | Symbols: SIRANBP | SIRANBP; Ran GTPase binding | chr1:2192360-2193688 REVERSEmoderately similar to ( 227) loc_os03g18180 12003.m07243 protein ran-binding protein 1 homolog c, putative, expressed  no original description 	T
29.4	protein.postranslational modification	MDP0000223943	 weakly similar to ( 166) AT1G31160 | Symbols:  | zinc-binding protein, putative / protein kinase C inhibitor, putative | chr1:11122880-11124106 REVERSEweakly similar to ( 139) ZB14_MAIZE 14 kDa zinc-binding protein (Protein kinase C inhibitor) (PKCI) - Zea mays (Maize)weakly similar to ( 135) loc_os03g20630 12003.m101330 protein zinc-binding protein, putative, expressed no original description 	T
35.2	not assigned.unknown	MDP0000300519	 highly similar to ( 713) AT4G27720 | Symbols:  | LOCATED IN: plasma membrane; EXPRESSED IN: 21 plant structures; EXPRESSED DURING: 13 growth stages; CONTAINS InterPro DOMAIN/s: Protein of unknown function DUF791 (InterPro:IPR008509), Major facilitator superfamily, general substrate transporter (InterPro:IPR016196); BEST Arabidopsis thaliana protein match is: unknown protein (TAIR:AT1G64650.1); Has 496 Blast hits to 491 proteins in 183 species: Archae - 5; Bacteria - 234; Metazoa - 75; Fungi - 33; Plants - 96; Viruses - 0; Other Eukaryotes - 53 (source: NCBI BLink). | chr4:13831203-13833521 FORWARDhighly similar to ( 665) loc_os10g37520 12010.m21796 protein major Facilitator Superfamily protein, expressed no original description 	T
26.27	misc.calcineurin-like phosphoesterase family protein	MDP0000219778	 moderately similar to ( 347) AT3G47810 | Symbols: MAG1 | MAG1 (MAIGO 1); hydrolase/ protein serine/threonine phosphatase | chr3:17637256-17639017 REVERSEmoderately similar to ( 366) loc_os02g16550 12002.m06898 protein vacuolar protein sorting 29, putative, expressed no original description 	T
33.99	development.unspecified	MDP0000281162	 very weakly similar to (92.0) AT1G17360 | Symbols:  | FUNCTIONS IN: molecular_function unknown; INVOLVED IN: biological_process unknown; LOCATED IN: cellular_component unknown; EXPRESSED IN: 22 plant structures; EXPRESSED DURING: 13 growth stages; BEST Arabidopsis thaliana protein match is: COP1-interacting protein-related (TAIR:AT1G72410.1); Has 8834 Blast hits to 6495 proteins in 406 species: Archae - 8; Bacteria - 626; Metazoa - 3389; Fungi - 793; Plants - 257; Viruses - 52; Other Eukaryotes - 3709 (source: NCBI BLink). | chr1:5947441-5951399 FORWARDmoderately similar to ( 374) loc_os03g21270 12003.m35204 protein expressed protein  no original description 	T
35.2	not assigned.unknown	MDP0000388228	 moderately similar to ( 482) AT1G74640 | Symbols:  | unknown protein | chr1:28032849-28034659 FORWARDmoderately similar to ( 445) loc_os09g24710 12009.m05625 protein hydrolases or acyltransferases, putative, expressed   no original description 	T
16.2	secondary metabolism.phenylpropanoids	MDP0000250804	 moderately similar to ( 317) AT1G65450 | Symbols:  | transferase family protein | chr1:24318035-24318895 FORWARDweakly similar to ( 154) HCBT1_DIACA Anthranilate N-benzoyltransferase protein 1 (EC 2.3.1.144) (Anthranilate N-hydroxycinnamoyl/benzoyltransferase 1) - Dianthus caryophyllus (Carnation) (Clove pink)moderately similar to ( 201) loc_os11g31090 12011.m07014 protein transferase, putative, expressed   no original description 	T
30.3	signalling.calcium	MDP0000311133	 highly similar to ( 649) AT1G53210 | Symbols:  | sodium/calcium exchanger family protein / calcium-binding EF hand family protein | chr1:19844790-19847533 FORWARDvery weakly similar to (55.5) CAX1C_ORYSA Vacuolar cation/proton exchanger 1c (Ca(2+)/H(+) exchanger 1c) (OsCAX1c) - Oryza sativa (Rice)highly similar to ( 608) loc_os01g11414 12001.m07759 protein calcium ion binding protein, putative, expressed  COG3889  no original description 	T
27.3.55	RNA.regulation of transcription.HDA	MDP0000916524	 very weakly similar to (99.0) AT5G03740 | Symbols: HD2C, HDT3 | HD2C (HISTONE DEACETYLASE 2C); histone deacetylase/ nucleic acid binding / transcription repressor/ zinc ion binding | chr5:981994-983961 FORWARDvery weakly similar to (95.9) HD2B_MAIZE Histone deacetylase 2b (HD2b) (Zm-HD2b) - Zea mays (Maize)very weakly similar to (89.4) loc_os05g51830 12005.m09245 protein histone deacetylase 2b, putative, expressed  no original description 	T
26.8	misc.nitrilases, *nitrile lyases, berberine bridge enzymes, reticuline oxidases, troponine reductases	MDP0000158474	 highly similar to ( 790) AT1G72970 | Symbols: HTH, EDA17 | HTH (HOTHEAD); FAD binding / aldehyde-lyase/ mandelonitrile lyase | chr1:27452872-27455706 FORWARDhighly similar to ( 707) loc_os09g19930 12009.m05200 protein protein HOTHEAD precursor, putative, expressed   no original description 	T
28.1.3	DNA.synthesis/chromatin structure.histone	MDP0000238589	 weakly similar to ( 159) AT5G59970 | Symbols:  | histone H4 | chr5:24146352-24146663 REVERSEweakly similar to ( 159) H4_PEA Histone H4 - Pisum sativum (Garden pea)weakly similar to ( 159) loc_os10g39410 12010.m06709 protein histone H4, putative, expressed no original description 	T
35.2	not assigned.unknown	MDP0000336096	 moderately similar to ( 303) AT1G53760 | Symbols:  | unknown protein | chr1:20069159-20070918 REVERSEmoderately similar to ( 235) loc_os02g05870 12002.m33560 protein expressed protein no original description 	T
29.4	protein.postranslational modification	MDP0000158387	 weakly similar to ( 142) AT4G25130 | Symbols:  | peptide methionine sulfoxide reductase, putative | chr4:12898802-12899998 REVERSEweakly similar to ( 195) MSRA_FRAAN Peptide methionine sulfoxide reductase (EC 1.8.4.11) (Protein-methionine-S-oxide reductase) (Peptide-methionine (S)-S-oxide reductase) (Peptide Met(O) reductase) (Fruit-ripening protein E4) - Fragaria ananassa (Strawberry)weakly similar to ( 145) loc_os04g40600 12004.m09041 protein peptide methionine sulfoxide reductase, putative, expressed no original description 	T
15.2	metal handling.binding, chelation and storage	MDP0000129164	 weakly similar to ( 146) AT3G24450 | Symbols:  | copper-binding family protein | chr3:8880286-8881611 REVERSEweakly similar to ( 137) loc_os01g52160 12001.m11390 protein metal ion binding protein, putative, expressed no original description 	T
26.8	misc.nitrilases, *nitrile lyases, berberine bridge enzymes, reticuline oxidases, troponine reductases	MDP0000220242	 highly similar to ( 918) AT5G13690 | Symbols:  | alpha-N-acetylglucosaminidase family / NAGLU family | chr5:4415808-4420159 FORWARDhighly similar to ( 953) loc_os04g55730 12004.m10464 protein alpha-N-acetylglucosaminidase precursor, putative, expressed  no original description 	T
31.3	cell.cycle	MDP0000256722	 weakly similar to ( 198) AT3G63120 | Symbols: CYCP1;1 | CYCP1;1 (cyclin p1;1); cyclin-dependent protein kinase | chr3:23323143-23323893 REVERSEmoderately similar to ( 216) loc_os05g33040 12005.m083714 protein PREG-like protein, putative, expressed  no original description 	T
29.4	protein.postranslational modification	MDP0000340624	 highly similar to ( 694) AT2G43790 | Symbols: ATMPK6, MPK6, MAPK6 | ATMPK6 (ARABIDOPSIS THALIANA MAP KINASE 6); MAP kinase/ kinase | chr2:18138477-18140693 FORWARDhighly similar to ( 719) MAPK_PEA Mitogen-activated protein kinase homolog D5 (EC 2.7.11.24) - Pisum sativum (Garden pea)highly similar to ( 688) loc_os06g06090 12006.m05335 protein OsMPK6 - putative MAPK based on amino acid sequence homology, expressed  no original description 	T
27.3.99	RNA.regulation of transcription.unclassified	MDP0000311556	 highly similar to ( 509) AT1G69420 | Symbols:  | zinc finger (DHHC type) family protein | chr1:26093690-26096736 FORWARDmoderately similar to ( 392) loc_os03g11110 12003.m06562 protein metal ion binding protein, putative, expressed no original description 	T
29.5.11.4.2	protein.degradation.ubiquitin.E3.RING	MDP0000881928	 highly similar to ( 984) AT1G24330 | Symbols:  | armadillo/beta-catenin repeat family protein / U-box domain-containing family protein | chr1:8631779-8634835 FORWARDweakly similar to ( 170) SPL11_ORYSA Spotted leaf protein 11 (Spotted leaf11) (Cell death-related protein SPL11) - Oryza sativa (Rice)highly similar to ( 813) loc_os01g67500 12001.m42788 protein ubiquitin-protein ligase, putative, expressed  no original description 	T
31.2	cell.division	MDP0000575967	 moderately similar to ( 431) AT5G11580 | Symbols:  | UVB-resistance protein-related / regulator of chromosome condensation (RCC1) family protein | chr5:3718913-3721125 FORWARDmoderately similar to ( 449) loc_os01g52630 12001.m11436 protein BNR repeat domain protein, putative, expressed   no original description 	T
30.3	signalling.calcium	MDP0000330029	 weakly similar to ( 170) AT1G18210 | Symbols:  | calcium-binding protein, putative | chr1:6268273-6268785 REVERSEvery weakly similar to ( 100) CALM2_ORYSA Calmodulin-2 (CaM-2) - Oryza sativa (Rice)weakly similar to ( 141) loc_os05g13580 12005.m05773 protein calmodulin-related protein 2, touch-induced, putative, expressed   no original description 	T
2.2.2.1.2	major CHO metabolism.degradation.starch.starch cleavage.beta amylase	MDP0000193684	 nearly identical (1377) AT5G53480 | Symbols:  | importin beta-2, putative | chr5:21714016-21716709 FORWARDmoderately similar to ( 293) AMYB_HORVU Beta-amylase (EC 3.2.1.2) (1,4-alpha-D-glucan maltohydrolase) - Hordeum vulgare (Barley)nearly identical (1323) loc_os12g38110 12012.m07597 protein importin beta-1 subunit, putative, expressed   no original description 	T
27.3.99	RNA.regulation of transcription.unclassified	MDP0000202548	 moderately similar to ( 397) AT3G26935 | Symbols:  | zinc finger (DHHC type) family protein | chr3:9933001-9935218 REVERSEmoderately similar to ( 483) loc_os08g42620 12008.m080224 protein palmitoyltransferase ZDHHC9, putative, expressed   tolA  no original description 	T
17.2.2	hormone metabolism.auxin.signal transduction	MDP0000138035	 highly similar to ( 883) AT1G73590 | Symbols: PIN1, ATPIN1 | PIN1 (PIN-FORMED 1); transporter | chr1:27659772-27662876 FORWARDhighly similar to ( 853) PIN1C_ORYSA Probable auxin efflux carrier component 1c (OsPIN1c) - Oryza sativa (Rice)highly similar to ( 853) loc_os06g12610 12006.m05977 protein auxin efflux carrier component 1c, putative, expressed  TrbL MSP1_C  no original description 	T
34.3	transport.amino acids	MDP0000383777	 highly similar to ( 734) AT1G77380 | Symbols: AAP3, ATAAP3 | AAP3; amino acid transmembrane transporter | chr1:29075201-29077252 REVERSEhighly similar to ( 681) loc_os05g34980 12005.m07728 protein amino acid carrier, putative, expressed no original description 	T
31.1	cell.organisation	MDP0000193559	 weakly similar to ( 135) AT4G03500 | Symbols:  | ankyrin repeat family protein | chr4:1553453-1556571 FORWARDvery weakly similar to (61.6) AKT1_ORYSA Potassium channel AKT1 (OsAKT1) - Oryza sativa (Rice)weakly similar to ( 118) loc_os09g16160 12009.m04876 protein conserved hypothetical protein  no original description 	T
20.2.1	stress.abiotic.heat	MDP0000697285	 nearly identical (1010) AT4G24280 | Symbols: cpHsc70-1 | cpHsc70-1 (chloroplast heat shock protein 70-1); ATP binding | chr4:12590094-12593437 FORWARDnearly identical (1011) HSP7S_PEA Stromal 70 kDa heat shock-related protein, chloroplast precursor - Pisum sativum (Garden pea)highly similar to ( 999) loc_os05g23740 12005.m06668 protein stromal 70 kDa heat shock-related protein, chloroplast precursor, putative, expressed  MSP1_C  no original description 	T
35.2	not assigned.unknown	MDP0000715622	  no original description 	T
11.1.15	lipid metabolism.FA synthesis and FA elongation.ACP desaturase	MDP0000253638	 highly similar to ( 634) AT2G43710 | Symbols: SSI2, FAB2 | SSI2; acyl-[acyl-carrier-protein] desaturase/ stearoyl-CoA 9-desaturase | chr2:18120107-18122495 FORWARDhighly similar to ( 679) STAD_RICCO Acyl-[acyl-carrier-protein] desaturase, chloroplast precursor (EC 1.14.19.2) (Stearoyl-ACP desaturase) (Delta(9) stearoyl-acyl carrier protein desaturase) - Ricinus communis (Castor bean)highly similar to ( 615) loc_os01g69080 12001.m13009 protein acyl-desaturase, chloroplast precursor, putative, expressed  no original description 	T
11.2.4	lipid metabolism.FA desaturation.omega 6 desaturase	MDP0000284275	 highly similar to ( 647) AT3G12120 | Symbols: FAD2 | FAD2 (FATTY ACID DESATURASE 2); delta12-fatty acid dehydrogenase/ omega-6 fatty acid desaturase | chr3:3860592-3861743 REVERSEhighly similar to ( 651) FD6E2_SOYBN Omega-6 fatty acid desaturase, endoplasmic reticulum isozyme 2 (EC 1.14.19.-) - Glycine max (Soybean)highly similar to ( 580) loc_os02g48560 12002.m100054 protein omega-6 fatty acid desaturase, endoplasmic reticulum isozyme 2, putative, expressed  no original description 	T
11.2.3	lipid metabolism.FA desaturation.omega 3 desaturase	MDP0000284275	 highly similar to ( 647) AT3G12120 | Symbols: FAD2 | FAD2 (FATTY ACID DESATURASE 2); delta12-fatty acid dehydrogenase/ omega-6 fatty acid desaturase | chr3:3860592-3861743 REVERSEhighly similar to ( 651) FD6E2_SOYBN Omega-6 fatty acid desaturase, endoplasmic reticulum isozyme 2 (EC 1.14.19.-) - Glycine max (Soybean)highly similar to ( 580) loc_os02g48560 12002.m100054 protein omega-6 fatty acid desaturase, endoplasmic reticulum isozyme 2, putative, expressed  no original description 	T
3.7	minor CHO metabolism.sugar kinases	MDP0000221409	 highly similar to ( 576) AT2G21370 | Symbols:  | xylulose kinase, putative | chr2:9137582-9139573 REVERSEmoderately similar to ( 357) loc_os07g22650 12007.m06549 protein xylulokinase, putative, expressed  no original description 	T
13.1.3.4.1	amino acid metabolism.synthesis.aspartate family.methionine.cystathionine gamma-synthase	MDP0000132750	 weakly similar to ( 150) AT3G01120 | Symbols: MTO1, CGS, ATCYS1, CGS1 | MTO1 (METHIONINE OVERACCUMULATION 1); cystathionine gamma-synthase | chr3:39234-41865 REVERSEvery weakly similar to (99.4) loc_os03g25940 12003.m78868 protein cystathionine beta-lyase, putative, expressed  no original description 	T
11.9.4.5	lipid metabolism.lipid degradation.beta-oxidation.acyl-CoA thioesterase	MDP0000674958	 weakly similar to ( 177) AT2G29590 | Symbols:  | thioesterase family protein | chr2:12654602-12655432 REVERSEweakly similar to ( 124) loc_os02g32200 12002.m08313 protein thioesterase family protein, putative, expressed no original description 	T
29.5.11.4.3.2	protein.degradation.ubiquitin.E3.SCF.FBOX	MDP0000313303	 very weakly similar to (80.1) AT3G06240 | Symbols:  | F-box family protein | chr3:1887336-1888619 FORWARDvery weakly similar to (62.8) loc_os06g07430 12006.m05465 protein F-box domain containing protein   no original description 	T
26.10	misc.cytochrome P450	MDP0000299766	 highly similar to ( 647) AT5G23880 | Symbols: EMB1265, CPSF100, ESP5, ATCPSF100 | CPSF100 (CLEAVAGE AND POLYADENYLATION SPECIFICITY FACTOR 100); DNA binding / protein binding | chr5:8052550-8058147 FORWARDhighly similar to ( 616) CPSF2_ORYSA Cleavage and polyadenylation specificity factor 100 kDa subunit (CPSF 100 kDa subunit) - Oryza sativa (Rice)highly similar to ( 616) loc_os09g39590 12009.m06897 protein cleavage and polyadenylation specificity factor, 100 kDa subunit, putative, expressed    no original description 	T
10.5.4	cell wall.cell wall proteins.HRGP	MDP0000453068	 moderately similar to ( 258) AT4G28300 | Symbols:  | hydroxyproline-rich glycoprotein family protein | chr4:14015414-14016823 FORWARDweakly similar to ( 154) loc_os01g55050 12001.m42723 protein calcium-binding protein, putative, expressed  no original description 	T
16.8.3.1	secondary metabolism.flavonoids.dihydroflavonols.dihydroflavonol 4-reductase	MDP0000138476	 weakly similar to ( 179) AT1G61720 | Symbols: BAN | BAN (BANYULS); oxidoreductase | chr1:22791326-22792757 REVERSEweakly similar to ( 168) DFRA_PETHY Dihydroflavonol-4-reductase (EC 1.1.1.219) (DFR) (Dihydrokaempferol 4-reductase) - Petunia hybrida (Petunia)moderately similar to ( 296) loc_os07g40986 12007.m79911 protein dihydroflavonol-4-reductase, putative, expressed no original description 	T
27.4	RNA.RNA binding	MDP0000262614	 moderately similar to ( 254) AT5G58130 | Symbols:  | RNA recognition motif (RRM)-containing protein | chr5:23521190-23523766 REVERSEmoderately similar to ( 211) loc_os02g04560 12002.m05805 protein expressed protein   no original description 	T
35.1	not assigned.no ontology	MDP0000179649	 highly similar to ( 518) AT5G12080 | Symbols: MSL10 | MSL10 (MECHANOSENSITIVE CHANNEL OF SMALL CONDUCTANCE-LIKE 10); mechanically-gated ion channel | chr5:3898182-3900923 REVERSEhighly similar to ( 518) loc_os06g10410 12006.m05759 protein mechanosensitive ion channel family protein, expressed  PRK08026  no original description 	T
13.1.3.5.5	amino acid metabolism.synthesis.aspartate family.lysine.diaminopimelate decarboxylase	MDP0000160102	 highly similar to ( 778) AT3G14390 | Symbols:  | diaminopimelate decarboxylase, putative / DAP carboxylase, putative | chr3:4806771-4808954 FORWARDvery weakly similar to (73.2) SPEA_BACTN Biosynthetic arginine decarboxylase (EC 4.1.1.19) (ADC) - Bacteroides thetaiotaomicronhighly similar to ( 682) loc_os02g24354 12002.m07582 protein diaminopimelate decarboxylase, putative, expressed  no original description 	T
26.2	misc.UDP glucosyl and glucoronyl transferases	MDP0000292949	 moderately similar to ( 449) AT5G59590 | Symbols: UGT76E2 | UGT76E2 (UDP-GLUCOSYL TRANSFERASE 76E2); UDP-glycosyltransferase/ quercetin 3-O-glucosyltransferase/ quercetin 7-O-glucosyltransferase | chr5:24009152-24010585 REVERSEweakly similar to ( 179) UFOG_SOLME Anthocyanidin 3-O-glucosyltransferase (EC 2.4.1.115) (Flavonol 3-O-glucosyltransferase) (UDP-glucose flavonoid 3-O-glucosyltransferase) - Solanum melongena (Eggplant) (Aubergine)moderately similar to ( 369) loc_os03g60960 12003.m10983 protein cytokinin-N-glucosyltransferase 1, putative, expressed Sporozoite_P67  no original description 	T
35.2	not assigned.unknown	MDP0000895343	  TrbL  no original description 	T
35.2	not assigned.unknown	MDP0000420309	   no original description 	T
29.5.11.5	protein.degradation.ubiquitin.ubiquitin protease	MDP0000388090	 moderately similar to ( 470) AT4G17890 | Symbols: AGD8 | AGD8 (ARF-GAP DOMAIN 8); ARF GTPase activator/ DNA binding / zinc ion binding | chr4:9937121-9938988 FORWARDmoderately similar to ( 454) loc_os03g63710 12003.m35539 protein DNA binding protein, putative, expressed   no original description 	T
29.5.5	protein.degradation.serine protease	MDP0000593481	 weakly similar to ( 105) AT4G01575 | Symbols:  | serine protease inhibitor, Kazal-type family protein | chr4:682040-682474 REVERSEvery weakly similar to ( 100) loc_os01g59610 12001.m12096 protein serine-type endopeptidase inhibitor, putative, expressed  no original description 	T
33.99	development.unspecified	MDP0000907489	 moderately similar to ( 310) AT4G35580 | Symbols: NTL9 | NTL9 (NAC transcription factor-like 9); transcription factor | chr4:16888534-16890744 REVERSEweakly similar to ( 181) NAC74_ORYSA NAC domain-containing protein 74 (ONAC074) - Oryza sativa (Rice)moderately similar to ( 220) loc_os08g06140 12008.m26586 protein ANAC014, putative, expressed  no original description 	T
15.1	metal handling.acquisition	MDP0000151434	 highly similar to ( 845) AT5G49740 | Symbols: ATFRO7, FRO7 | ATFRO7 (FERRIC REDUCTION OXIDASE 7); ferric-chelate reductase/ oxidoreductase | chr5:20205549-20208628 REVERSEhighly similar to ( 777) loc_os04g36720 12004.m08712 protein ferric reductase-like transmembrane component, putative, expressed  no original description 	T
31.4	cell.vesicle transport	MDP0000291524	 highly similar to ( 598) AT1G29150 | Symbols: ATS9, RPN6 | ATS9 (ARABIDOPSIS NON-ATPASE SUBUNIT 9) | chr1:10181240-10182499 FORWARDhighly similar to ( 622) loc_os04g36700 12004.m08710 protein 26S proteasome non-ATPase regulatory subunit 11, putative, expressed  no original description 	T
35.2	not assigned.unknown	MDP0000337580	 weakly similar to ( 196) AT5G20090 | Symbols:  | unknown protein | chr5:6787543-6788000 REVERSEweakly similar to ( 192) loc_os09g20660 12009.m059970 protein brain protein 44-like protein, putative, expressed no original description 	T
26.2	misc.UDP glucosyl and glucoronyl transferases	MDP0000308543	 weakly similar to ( 121) AT5G59590 | Symbols: UGT76E2 | UGT76E2 (UDP-GLUCOSYL TRANSFERASE 76E2); UDP-glycosyltransferase/ quercetin 3-O-glucosyltransferase/ quercetin 7-O-glucosyltransferase | chr5:24009152-24010585 REVERSEvery weakly similar to (55.5) IAAG_MAIZE Indole-3-acetate beta-glucosyltransferase (EC 2.4.1.121) (IAA-Glu synthetase) ((Uridine 5'-diphosphate-glucose:indol-3-ylacetyl)-beta-D-glucosyl transferase) - Zea mays (Maize)very weakly similar to (96.3) loc_os11g25454 12011.m06467 protein cytokinin-N-glucosyltransferase 1, putative, expressed TrbL  no original description 	T
17.5.1	hormone metabolism.ethylene.synthesis-degradation	MDP0000176227	 moderately similar to ( 306) AT4G16765 | Symbols:  | oxidoreductase, 2OG-Fe(II) oxygenase family protein | chr4:9430467-9431791 REVERSEvery weakly similar to (98.2) FLS_MALDO Flavonol synthase/flavanone 3-hydroxylase (EC 1.14.11.23) (EC 1.14.11.9) (FLS) - Malus domestica (Apple) (Malus sylvestris)moderately similar to ( 333) loc_os10g37899 12010.m06575 protein transposon protein, putative, unclassified, expressed no original description 	T
21.1	redox.thioredoxin	MDP0000795863	 weakly similar to ( 138) AT5G23440 | Symbols: FTRA1 | FTRA1 (ferredoxin/thioredoxin reductase subunit A (variable subunit) 1); catalytic/ ferredoxin reductase/ ferredoxin:thioredoxin reductase/ lipoate synthase | chr5:7903230-7903778 REVERSEweakly similar to ( 110) FTRV_MAIZE Ferredoxin-thioredoxin reductase, variable chain (FTR-V) (Ferredoxin-thioredoxin reductase subunit A) (FTR-A) - Zea mays (Maize)weakly similar to ( 116) loc_os04g44650 12004.m09424 protein ferredoxin-thioredoxin reductase, variable chain, putative, expressed no original description 	T
35.1.19	not assigned.no ontology.C2 domain-containing protein	MDP0000494908	 moderately similar to ( 434) AT3G57880 | Symbols:  | C2 domain-containing protein | chr3:21431198-21433519 REVERSEmoderately similar to ( 410) loc_os05g30750 12005.m083576 protein phosphoribosylanthranilate transferase, putative, expressed no original description 	T
10.5.4	cell wall.cell wall proteins.HRGP	MDP0000742981	 very weakly similar to (58.5) AT1G44191 | Symbols:  | Encodes a ECA1 gametogenesis related family protein | chr1:16813654-16814733 REVERSEvery weakly similar to (52.8) loc_os07g40510 12007.m08290 protein formin-2, putative, expressed no original description 	T
13.99	amino acid metabolism.misc	MDP0000278395	 highly similar to ( 596) AT1G49820 | Symbols: ATMTK | ATMTK (ARABIDOPSIS THALIANA S-METHYL-5-THIORIBOSE KINASE); S-methyl-5-thioribose kinase | chr1:18443005-18444907 FORWARDhighly similar to ( 561) loc_os04g57400 12004.m35509 protein methylthioribose kinase, putative, expressed  no original description 	T
29.6	protein.folding	MDP0000213300	 highly similar to ( 811) AT3G03960 | Symbols:  | chaperonin, putative | chr3:1024432-1027604 FORWARDweakly similar to ( 169) TCPE2_AVESA T-complex protein 1 subunit epsilon (TCP-1-epsilon) (CCT-epsilon) (TCP-K36) - Avena sativa (Oat)highly similar to ( 842) loc_os03g59020 12003.m10803 protein T-complex protein 1 subunit theta, putative, expressed  no original description 	T
26.28	misc.GDSL-motif lipase	MDP0000706828	 highly similar to ( 540) AT3G26430 | Symbols:  | GDSL-motif lipase/hydrolase family protein | chr3:9674419-9675889 FORWARDmoderately similar to ( 402) EST_HEVBR Esterase precursor (EC 3.1.1.-) (Early nodule-specific protein homolog) (Latex allergen Hev b 13) - Hevea brasiliensis (Para rubber tree)moderately similar to ( 437) loc_os07g39750 12007.m079815 protein alpha-L-fucosidase 2 precursor, putative, expressed  no original description 	T
35.2	not assigned.unknown	MDP0000252680	 moderately similar to ( 318) AT3G57400 | Symbols:  | unknown protein | chr3:21238547-21239971 FORWARD  MSP1_C  no original description 	T
30.5	signalling.G-proteins	MDP0000153185	 highly similar to ( 652) AT5G02010 | Symbols: ATROPGEF7, ROPGEF7 | ROPGEF7; Rho guanyl-nucleotide exchange factor | chr5:383493-385698 FORWARDhighly similar to ( 650) loc_os01g62990 12001.m12417 protein pollen-specific kinase partner protein, putative, expressed   no original description 	T
30.3	signalling.calcium	MDP0000421959	 highly similar to ( 817) AT1G27460 | Symbols: NPGR1 | NPGR1 (NO POLLEN GERMINATION RELATED 1); calmodulin binding | chr1:9534977-9537424 FORWARDhighly similar to ( 697) loc_os03g10350 12003.m101228 protein NPGR1, putative, expressed   no original description 	T
33.99	development.unspecified	MDP0000165295	 nearly identical (1085) AT3G09090 | Symbols: DEX1 | DEX1 (DEFECTIVE IN EXINE FORMATION 1); calcium ion binding | chr3:2783165-2787552 REVERSEnearly identical (1009) loc_os03g61050 12003.m10989 protein DEX1, putative, expressed  no original description 	T
30.2.11	signalling.receptor kinases.leucine rich repeat XI	MDP0000792856	 highly similar to ( 824) AT3G47570 | Symbols:  | leucine-rich repeat transmembrane protein kinase, putative | chr3:17527611-17530748 FORWARDmoderately similar to ( 421) RPK1_IPONI Receptor-like protein kinase precursor (EC 2.7.11.1) - Ipomoea nil (Japanese morning glory) (Pharbitis nil)highly similar to ( 799) loc_os11g36150 12011.m07467 protein receptor-like protein kinase precursor, putative, expressed   no original description 	T
35.2	not assigned.unknown	MDP0000572632	 very weakly similar to (78.2) AT1G26110 | Symbols:  | unknown protein | chr1:9024616-9027556 REVERSEvery weakly similar to (76.6) loc_os01g02090 12001.m42836 protein expressed protein TrbL  no original description 	T
31.1	cell.organisation	MDP0000264165	 weakly similar to ( 189) AT4G03500 | Symbols:  | ankyrin repeat family protein | chr4:1553453-1556571 FORWARDvery weakly similar to (61.6) AKT1_ORYSA Potassium channel AKT1 (OsAKT1) - Oryza sativa (Rice)weakly similar to ( 162) loc_os09g16550 12009.m04915 protein protein binding protein, putative, expressed  no original description 	T
29.4	protein.postranslational modification	MDP0000375492	 weakly similar to ( 182) AT1G49180 | Symbols:  | protein kinase family protein | chr1:18184840-18187444 REVERSEvery weakly similar to (69.7) CIPK1_ORYSA CIPK-like protein 1 (EC 2.7.11.1) (OsCK1) - Oryza sativa (Rice)weakly similar to ( 154) loc_os04g58990 12004.m10777 protein MAP/microtubule affinity-regulating kinase 4, putative, expressed  no original description 	T
35.2	not assigned.unknown	MDP0000209369	 moderately similar to ( 444) loc_os07g39980 12007.m08240 protein expressed protein  no original description 	T
20.2.1	stress.abiotic.heat	MDP0000231815	 highly similar to ( 501) AT3G62600 | Symbols: ATERDJ3B | ATERDJ3B; heat shock protein binding / unfolded protein binding | chr3:23151038-23153346 REVERSEweakly similar to ( 171) DNJH_CUCSA DnaJ protein homolog (DNAJ-1) - Cucumis sativus (Cucumber)highly similar to ( 502) loc_os05g06440 12005.m05171 protein dnaJ homolog subfamily B member 11 precursor, putative, expressed  no original description 	T
35.2	not assigned.unknown	MDP0000673070	 moderately similar to ( 387) AT1G74450 | Symbols:  | unknown protein | chr1:27982737-27983930 FORWARDmoderately similar to ( 295) loc_os04g59140 12004.m10791 protein expressed protein  TrbL  no original description 	T
11.3.7	lipid metabolism.Phospholipid synthesis.cyclopropane-fatty-acyl-phospholipid synthase	MDP0000276878	 nearly identical (1217) AT3G23530 | Symbols:  | cyclopropane fatty acid synthase, putative / CPA-FA synthase, putative | chr3:8437472-8442597 FORWARDvery weakly similar to (51.2) PPOCM_SPIOL Protoporphyrinogen oxidase, chloroplast/mitochondrial precursor (EC 1.3.3.4) (Protox II) (SO-POX2) - Spinacia oleracea (Spinach)highly similar to ( 937) loc_os12g16650 12012.m073808 protein cyclopropane fatty acid synthase, putative, expressed  no original description 	T
35.2	not assigned.unknown	MDP0000302366	 highly similar to ( 624) AT5G12960 | Symbols:  | catalytic | chr5:4097788-4101518 FORWARDhighly similar to ( 566) loc_os02g10190 12002.m06317 protein secreted protein, putative, expressed  no original description 	T
35.2	not assigned.unknown	MDP0000202279	 moderately similar to ( 254) AT2G30170 | Symbols:  | catalytic | chr2:12879802-12881046 REVERSEweakly similar to ( 144) loc_os01g07090 12001.m07336 protein protein phosphatase 2C homolog 7, putative, expressed  no original description 	T
27.3.44	RNA.regulation of transcription.Chromatin Remodeling Factors	MDP0000176677	 highly similar to ( 625) AT5G14170 | Symbols: CHC1 | CHC1 | chr5:4568696-4570444 REVERSEhighly similar to ( 576) loc_os04g31320 12004.m08234 protein SWI/SNF-related matrix-associated actin-dependent regulator of chromatin subfamily D member 3, putative, expressed  no original description 	T
16.2	secondary metabolism.phenylpropanoids	MDP0000428199	 moderately similar to ( 432) AT5G17540 | Symbols:  | transferase family protein | chr5:5782061-5783682 REVERSEweakly similar to ( 159) HCBT3_DIACA Anthranilate N-benzoyltransferase protein 3 (EC 2.3.1.144) (Anthranilate N-hydroxycinnamoyl/benzoyltransferase 3) - Dianthus caryophyllus (Carnation) (Clove pink)moderately similar to ( 441) loc_os10g35950 12010.m06411 protein 10-deacetylbaccatin III 10-O-acetyltransferase, putative, expressed  no original description 	T
30.2.17	signalling.receptor kinases.DUF 26	MDP0000496152	 moderately similar to ( 449) AT1G16670 | Symbols:  | protein kinase family protein | chr1:5697846-5699492 FORWARDmoderately similar to ( 228) NORK_PEA Nodulation receptor kinase precursor (EC 2.7.11.1) - Pisum sativum (Garden pea)moderately similar to ( 430) loc_os02g06930 12002.m100111 protein serine/threonine-protein kinase receptor precursor, putative, expressed no original description 	T
29.4.1.57	protein.postranslational modification.kinase.receptor like cytoplasmatic kinase VII	MDP0000496152	 moderately similar to ( 449) AT1G16670 | Symbols:  | protein kinase family protein | chr1:5697846-5699492 FORWARDmoderately similar to ( 228) NORK_PEA Nodulation receptor kinase precursor (EC 2.7.11.1) - Pisum sativum (Garden pea)moderately similar to ( 430) loc_os02g06930 12002.m100111 protein serine/threonine-protein kinase receptor precursor, putative, expressed no original description 	T
35.1	not assigned.no ontology	MDP0000162266	 weakly similar to ( 162) AT4G14420 | Symbols:  | lesion inducing protein-related | chr4:8302171-8303738 REVERSEweakly similar to ( 175) loc_os03g05520 12003.m06075 protein nicotiana lesion-inducing like, putative, expressed no original description 	T
13.1.6.3.1	amino acid metabolism.synthesis.aromatic aa.phenylalanine.arogenate dehydratase / prephenate dehydratase	MDP0000672088	 highly similar to ( 574) AT1G08250 | Symbols: ADT6 | ADT6 (arogenate dehydratase 6); arogenate dehydratase/ prephenate dehydratase | chr1:2588994-2590235 REVERSEhighly similar to ( 539) loc_os09g39260 12009.m06865 protein prephenate dehydratase, putative, expressed   no original description 	T
29.2.2	protein.synthesis.ribosome biogenesis	MDP0000186861	 weakly similar to ( 129) AT3G53470 | Symbols:  | unknown protein | chr3:19822813-19823298 FORWARDweakly similar to ( 127) loc_os03g17660 12003.m35167 protein expressed protein no original description 	T
28.1	DNA.synthesis/chromatin structure	MDP0000306035	 highly similar to ( 606) AT5G04130 | Symbols:  | DNA topoisomerase, ATP-hydrolyzing, putative / DNA topoisomerase II, putative / DNA gyrase, putative | chr5:1122084-1128031 REVERSEhighly similar to ( 553) GYRB_ORYSA DNA gyrase subunit B, chloroplast/mitochondrial precursor (EC 5.99.1.3) - Oryza sativa (Rice)highly similar to ( 553) loc_os01g16290 12001.m42965 protein DNA gyrase subunit B, putative, expressed    no original description 	T
20.1	stress.biotic	MDP0000121207	 weakly similar to ( 185) AT4G31470 | Symbols:  | pathogenesis-related protein, putative | chr4:15261746-15262303 REVERSEweakly similar to ( 171) PR1_MEDTR Pathogenesis-related protein PR-1 precursor - Medicago truncatula (Barrel medic)weakly similar to ( 155) loc_os01g28500 12001.m43020 protein pathogenesis-related protein PRMS precursor, putative, expressed  no original description 	T
33.99	development.unspecified	MDP0000243138	 weakly similar to ( 111) AT1G78020 | Symbols:  | senescence-associated protein-related | chr1:29338787-29339491 FORWARDvery weakly similar to (89.7) loc_os09g20240 12009.m059968 protein expressed protein no original description 	T
29.4.1.57	protein.postranslational modification.kinase.receptor like cytoplasmatic kinase VII	MDP0000225415	 highly similar to ( 598) AT3G59350 | Symbols:  | serine/threonine protein kinase, putative | chr3:21932930-21934883 FORWARDmoderately similar to ( 202) NORK_MEDTR Nodulation receptor kinase precursor (EC 2.7.11.1) (Does not make infections protein 2) (Symbiosis receptor-like kinase) (MtSYMRK) - Medicago truncatula (Barrel medic)highly similar to ( 521) loc_os09g33860 12009.m06435 protein expressed protein  no original description 	T
13.1.5.1.1	amino acid metabolism.synthesis.serine-glycine-cysteine group.serine.phosphoglycerate dehydrogenase	MDP0000166138	 highly similar to ( 742) AT1G17745 | Symbols: PGDH | PGDH (3-PHOSPHOGLYCERATE DEHYDROGENASE); phosphoglycerate dehydrogenase | chr1:6101157-6104979 FORWARDvery weakly similar to (99.4) DHGY_CUCSA Glycerate dehydrogenase (EC 1.1.1.29) (NADH-dependent hydroxypyruvate reductase) (HPR) (GDH) - Cucumis sativus (Cucumber)highly similar to ( 717) loc_os04g55720 12004.m35214 protein D-3-phosphoglycerate dehydrogenase, chloroplast precursor, putative, expressed   MSP1_C  no original description 	T
29.4	protein.postranslational modification	MDP0000308419	 highly similar to ( 533) AT3G51630 | Symbols: WNK5, ZIK1, ATWNK5 | WNK5 (WITH NO LYSINE (K) KINASE 5); protein kinase | chr3:19149487-19151924 FORWARDvery weakly similar to (68.9) MPK14_ORYSA Mitogen-activated protein kinase 14 (EC 2.7.11.24) (MAP kinase 14) - Oryza sativa (Rice)moderately similar to ( 480) loc_os11g02300 12011.m28642 protein mitogen-activated protein kinase, putative, expressed  no original description 	T
29.2.1.2.2.17	protein.synthesis.ribosomal protein.eukaryotic.60S subunit.L17	MDP0000541368	 moderately similar to ( 284) AT1G67430 | Symbols:  | 60S ribosomal protein L17 (RPL17B) | chr1:25262209-25263627 FORWARDmoderately similar to ( 277) RL172_HORVU 60S ribosomal protein L17-2 - Hordeum vulgare (Barley)moderately similar to ( 275) loc_os09g08430 12009.m04110 protein 60S ribosomal protein L17, putative, expressed no original description 	T
35.2	not assigned.unknown	MDP0000156548	 weakly similar to ( 120) AT1G69160 | Symbols:  | unknown protein | chr1:26000315-26001280 FORWARD   no original description 	T
20.2	stress.abiotic	MDP0000839889	 moderately similar to ( 336) AT5G52060 | Symbols: ATBAG1 | ATBAG1 (ARABIDOPSIS THALIANA BCL-2-ASSOCIATED ATHANOGENE 1); protein binding | chr5:21152449-21153741 REVERSEmoderately similar to ( 287) loc_os09g35630 12009.m06514 protein protein binding protein, putative, expressed no original description 	T
35.2	not assigned.unknown	MDP0000702081	 weakly similar to ( 121) AT2G17972 | Symbols:  | unknown protein | chr2:7821624-7822100 FORWARDweakly similar to ( 120) loc_os06g50130 12006.m09537 protein expressed protein  TrbL  no original description 	T
35.2	not assigned.unknown	MDP0000844351	 COG3889  no original description 	T
30.3	signalling.calcium	MDP0000167819	 highly similar to ( 644) AT5G61790 | Symbols: CNX1, ATCNX1 | calnexin 1 (CNX1) | chr5:24827394-24829642 REVERSEhighly similar to ( 664) CALX_SOYBN Calnexin homolog precursor - Glycine max (Soybean)highly similar to ( 630) loc_os04g32950 12004.m78935 protein calnexin homolog precursor, putative, expressed   no original description 	T
29.4	protein.postranslational modification	MDP0000155151	 nearly identical (1604) AT1G50240 | Symbols: FU | FU (FUSED); protein serine/threonine kinase | chr1:18607063-18614094 FORWARDweakly similar to ( 130) CIPK1_ORYSA CIPK-like protein 1 (EC 2.7.11.1) (OsCK1) - Oryza sativa (Rice)nearly identical (1389) loc_os12g24550 12012.m06286 protein expressed protein    no original description 	T
29.2.1.2.2.36	protein.synthesis.ribosomal protein.eukaryotic.60S subunit.L36	MDP0000292247	 weakly similar to ( 157) AT2G37600 | Symbols:  | 60S ribosomal protein L36 (RPL36A) | chr2:15774410-15775203 REVERSEweakly similar to ( 136) RL36_DAUCA 60S ribosomal protein L36 - Daucus carota (Carrot)weakly similar to ( 177) loc_os05g38520 12005.m27873 protein 60S ribosomal protein L36-2, putative, expressed  no original description 	T
35.1	not assigned.no ontology	MDP0000929417	 weakly similar to ( 150) AT1G04340 | Symbols:  | lesion inducing protein-related | chr1:1163345-1164737 REVERSEweakly similar to ( 167) loc_os03g05520 12003.m06075 protein nicotiana lesion-inducing like, putative, expressed  no original description 	T
29.4	protein.postranslational modification	MDP0000288637	 highly similar to ( 579) AT1G12680 | Symbols: PEPKR2 | PEPKR2 (Phosphoenolpyruvate carboxylase-related kinase 2); ATP binding / kinase/ protein kinase/ protein serine/threonine kinase | chr1:4320123-4322269 REVERSEmoderately similar to ( 245) CDPK3_ORYSA Calcium-dependent protein kinase, isoform 11 (EC 2.7.11.1) (CDPK 11) - Oryza sativa (Rice)moderately similar to ( 450) loc_os09g29170 12009.m21997 protein calcium-dependent protein kinase, putative, expressed  no original description 	T
29.4.1.57	protein.postranslational modification.kinase.receptor like cytoplasmatic kinase VII	MDP0000203534	 highly similar to ( 748) AT1G53730 | Symbols: SRF6 | SRF6 (STRUBBELIG-RECEPTOR FAMILY 6); ATP binding / protein binding / protein kinase/ protein serine/threonine kinase | chr1:20061771-20065475 FORWARDweakly similar to ( 197) NORK_PEA Nodulation receptor kinase precursor (EC 2.7.11.1) - Pisum sativum (Garden pea)highly similar to ( 720) loc_os10g25090 12010.m05459 protein leucine-rich repeat transmembrane protein kinase 1, putative, expressed  no original description 	T
35.2	not assigned.unknown	MDP0000168928	  no original description 	T
35.2	not assigned.unknown	MDP0000392057	 highly similar to ( 534) AT5G35430 | Symbols:  | binding | chr5:13662077-13665735 FORWARDmoderately similar to ( 464) loc_os09g03600 12009.m03730 protein expressed protein  no original description 	T
35.2	not assigned.unknown	MDP0000471083	 very weakly similar to (95.1) AT5G41761 | Symbols:  | unknown protein | chr5:16711437-16711742 FORWARDweakly similar to ( 102) loc_os06g33200 12006.m07857 protein expressed protein no original description 	T
34.99	transport.misc	MDP0000218729	 highly similar to ( 902) AT1G60030 | Symbols: ATNAT7, NAT7 | xanthine/uracil permease family protein | chr1:22113993-22116648 REVERSEhighly similar to ( 871) loc_os02g50820 12002.m10116 protein permease I, putative, expressed  no original description 	T
35.2	not assigned.unknown	MDP0000424231	 very weakly similar to (94.7) AT2G42110 | Symbols:  | unknown protein | chr2:17562234-17562732 REVERSEvery weakly similar to (62.4) loc_os03g03630 12003.m05896 protein expressed protein   no original description 	T
2.2.2.1	major CHO metabolism.degradation.starch.starch cleavage	MDP0000212372	 highly similar to ( 573) AT1G76130 | Symbols: ATAMY2, AMY2 | AMY2 (ALPHA-AMYLASE-LIKE 2); alpha-amylase/ calcium ion binding / catalytic/ cation binding | chr1:28561647-28563914 FORWARDmoderately similar to ( 303) AMY2_HORVU Alpha-amylase type B isozyme precursor (EC 3.2.1.1) (1,4-alpha-D-glucan glucanohydrolase) (AMY2-2) (High pI alpha-amylase) - Hordeum vulgare (Barley)moderately similar to ( 496) loc_os04g33040 12004.m08400 protein alpha-amylase type B isozyme precursor, putative, expressed no original description 	T
35.1	not assigned.no ontology	MDP0000469526	 highly similar to ( 539) AT4G36530 | Symbols:  | hydrolase, alpha/beta fold family protein | chr4:17240120-17241770 REVERSEmoderately similar to ( 469) loc_os02g47620 12002.m33833 protein catalytic/ hydrolase, putative, expressed   no original description 	T
26.6	misc.O-methyl transferases	MDP0000261123	 weakly similar to ( 196) AT3G12270 | Symbols: ATPRMT3, PRMT3 | PRMT3 (PROTEIN ARGININE METHYLTRANSFERASE 3); methyltransferase/ zinc ion binding | chr3:3910642-3913122 FORWARDweakly similar to ( 186) loc_os07g44640 12007.m08685 protein protein arginine N-methyltransferase 3-like protein, putative, expressed no original description 	T
1.3.6	PS.calvin cycle.aldolase	MDP0000151849	 highly similar to ( 676) AT4G38970 | Symbols:  | fructose-bisphosphate aldolase, putative | chr4:18163769-18165659 REVERSEhighly similar to ( 653) ALFC_ORYSA Fructose-bisphosphate aldolase, chloroplast precursor (EC 4.1.2.13) (ALDP) - Oryza sativa (Rice)highly similar to ( 653) loc_os11g07020 12011.m28744 protein fructose-bisphosphate aldolase, chloroplast precursor, putative, expressed  no original description 	T
29.2.1.2.1.16	protein.synthesis.ribosomal protein.eukaryotic.40S subunit.S16	MDP0000207901	 moderately similar to ( 261) AT2G09990 | Symbols:  | 40S ribosomal protein S16 (RPS16A) | chr2:3781442-3781882 FORWARDmoderately similar to ( 276) RS16_GOSHI 40S ribosomal protein S16 - Gossypium hirsutum (Upland cotton)moderately similar to ( 240) loc_os12g03090 12012.m04307 protein 40S ribosomal protein S16, putative, expressed  no original description 	T
34.14	transport.unspecified cations	MDP0000142739	 highly similar to ( 537) AT2G26900 | Symbols:  | bile acid:sodium symporter family protein | chr2:11475156-11477870 REVERSEhighly similar to ( 514) loc_os01g45750 12001.m43113 protein bile acid sodium symporter/ transporter, putative, expressed  no original description 	T
10.5.1.1	cell wall.cell wall proteins.AGPs.AGP	MDP0000221961	 very weakly similar to (68.9) AT2G46330 | Symbols: AGP16, ATAGP16 | AGP16 (ARABINOGALACTAN PROTEIN 16) | chr2:19018730-19019108 REVERSEvery weakly similar to (65.1) loc_os05g12580 12005.m05721 protein AGP20, putative no original description 	T
29.7	protein.glycosylation	MDP0000240682	 highly similar to ( 570) AT1G77810 | Symbols:  | galactosyltransferase family protein | chr1:29260899-29263001 REVERSEhighly similar to ( 592) loc_os02g35870 12002.m08675 protein avr9 elicitor response protein, putative, expressed  no original description 	T
26.11	misc.alcohol dehydrogenases	MDP0000680997	 highly similar to ( 602) AT1G77120 | Symbols: ADH1, ADH, ATADH, ATADH1 | ADH1 (ALCOHOL DEHYDROGENASE 1); alcohol dehydrogenase | chr1:28975509-28977216 FORWARDhighly similar to ( 605) ADH1_MAIZE Alcohol dehydrogenase 1 (EC 1.1.1.1) - Zea mays (Maize)highly similar to ( 600) loc_os11g10480 12011.m28756 protein alcohol dehydrogenase 1, putative, expressed  no original description 	T
34.12	transport.metal	MDP0000269871	 moderately similar to ( 354) AT1G55910 | Symbols: ZIP11 | ZIP11 (ZINC TRANSPORTER 11 PRECURSOR); cation transmembrane transporter/ metal ion transmembrane transporter | chr1:20906161-20907225 FORWARDmoderately similar to ( 358) loc_os03g29850 12003.m08248 protein zinc transporter 2 precursor, putative, expressed   no original description 	T
33.99	development.unspecified	MDP0000480581	 moderately similar to ( 334) AT2G17040 | Symbols: anac036 | anac036 (Arabidopsis NAC domain containing protein 36); transcription factor | chr2:7407123-7408120 FORWARDweakly similar to ( 164) NAC67_ORYSA NAC domain-containing protein 67 (ONAC067) - Oryza sativa (Rice)moderately similar to ( 288) loc_os03g04070 12003.m05939 protein NAC domain-containing protein 68, putative, expressed  no original description 	T
15.1	metal handling.acquisition	MDP0000613837	 moderately similar to ( 450) AT5G23990 | Symbols: ATFRO5, FRO5 | FRO5 (FERRIC REDUCTION OXIDASE 5); ferric-chelate reductase | chr5:8105565-8108590 REVERSEmoderately similar to ( 270) loc_os04g48930 12004.m101632 protein ferric-chelate reductase, putative, expressed   no original description 	T
35.2	not assigned.unknown	MDP0000305502	 moderately similar to ( 495) AT3G07660 | Symbols:  | unknown protein | chr3:2445275-2450372 REVERSEmoderately similar to ( 343) loc_os09g08680 12009.m04135 protein expressed protein   no original description 	T
26.4.1	misc.beta 1,3 glucan hydrolases.glucan endo-1,3-beta-glucosidase	MDP0000146863	 highly similar to ( 592) AT4G34480 | Symbols:  | catalytic/ cation binding / hydrolase, hydrolyzing O-glycosyl compounds | chr4:16481147-16483988 REVERSEmoderately similar to ( 354) E13B_WHEAT Glucan endo-1,3-beta-glucosidase precursor (EC 3.2.1.39) ((1->3)-beta-glucan endohydrolase) ((1->3)-beta-glucanase) (Beta-1,3-endoglucanase) - Triticum aestivum (Wheat)highly similar to ( 501) loc_os03g46660 12003.m09685 protein glucan endo-1,3-beta-glucosidase 7 precursor, putative, expressed  no original description 	T
29.3.3	protein.targeting.chloroplast	MDP0000258161	 moderately similar to ( 412) AT5G05000 | Symbols: TOC34, ATTOC34, OEP34 | TOC34 (TRANSLOCON AT THE OUTER ENVELOPE MEMBRANE OF CHLOROPLASTS 34); GTP binding | chr5:1474262-1475772 REVERSEmoderately similar to ( 446) TOC34_PEA Translocase of chloroplast 34 (EC 3.6.5.-) (34 kDa chloroplast outer envelope protein) (GTP-binding protein OEP34) (GTP-binding protein IAP34) - Pisum sativum (Garden pea)moderately similar to ( 377) loc_os03g13730 12003.m35119 protein translocase of chloroplast 34, putative, expressed no original description 	T
4.1.13	glycolysis.cytosolic branch.enolase	MDP0000155746	 highly similar to ( 722) AT1G74030 | Symbols:  | enolase, putative | chr1:27839465-27841901 REVERSEhighly similar to ( 532) ENO_RICCO Enolase (EC 4.2.1.11) (2-phosphoglycerate dehydratase) (2-phospho-D-glycerate hydro-lyase) - Ricinus communis (Castor bean)highly similar to ( 697) loc_os09g20820 12009.m05289 protein enolase, putative, expressed  no original description 	T
29.2.1.2.2.9	protein.synthesis.ribosomal protein.eukaryotic.60S subunit.L9	MDP0000249666	 moderately similar to ( 388) AT4G27585 | Symbols:  | band 7 family protein | chr4:13766984-13769832 REVERSEmoderately similar to ( 283) RL9_PEA 60S ribosomal protein L9 (Gibberellin-regulated protein GA) - Pisum sativum (Garden pea)moderately similar to ( 326) loc_os08g06200 12008.m04759 protein stomatin-like protein 2, putative, expressed   no original description 	T
11.1.7	lipid metabolism.FA synthesis and FA elongation.ACP thioesterase	MDP0000298759	 highly similar to ( 573) AT1G08510 | Symbols: FATB | FATB (fatty acyl-ACP thioesterases B); acyl carrier/ acyl-[acyl-carrier-protein] hydrolase | chr1:2691546-2693409 REVERSEhighly similar to ( 570) FATB_GOSHI Myristoyl-acyl carrier protein thioesterase, chloroplast precursor (EC 3.1.2.-) (16:0-acyl-carrier protein thioesterase) (16:0-ACP thioesterase) (Acyl-[acyl-carrier-protein] hydrolase) (PATE) - Gossypium hirsutum (Upland cotton)moderately similar to ( 489) loc_os06g05130 12006.m05242 protein myristoyl-acyl carrier protein thioesterase, chloroplast precursor, putative, expressed  no original description 	T
34.3	transport.amino acids	MDP0000312601	 highly similar to ( 940) AT1G47670 | Symbols:  | amino acid transporter family protein | chr1:17536834-17539486 REVERSEhighly similar to ( 760) loc_os12g30040 12012.m06817 protein amino acid transporter-like protein 1, putative, expressed   no original description 	T
13.2.3.2	amino acid metabolism.degradation.aspartate family.threonine	MDP0000825994	 moderately similar to ( 337) AT1G08110 | Symbols:  | lactoylglutathione lyase, putative / glyoxalase I, putative | chr1:2535463-2537630 FORWARDmoderately similar to ( 340) LGUL_CICAR Lactoylglutathione lyase (EC 4.4.1.5) (Methylglyoxalase) (Aldoketomutase) (Glyoxalase I) (Glx I) (Ketone-aldehyde mutase) (S-D-lactoylglutathione methylglyoxal lyase) - Cicer arietinum (Chickpea) (Garbanzo)moderately similar to ( 330) loc_os05g22970 12005.m06592 protein lactoylglutathione lyase, putative, expressed  no original description 	T
26.2	misc.UDP glucosyl and glucoronyl transferases	MDP0000310017	 highly similar to ( 687) AT1G18580 | Symbols: GAUT11 | GAUT11 (Galacturonosyltransferase 11); polygalacturonate 4-alpha-galacturonosyltransferase | chr1:6396144-6398005 FORWARDhighly similar to ( 583) loc_os03g30000 12003.m08261 protein transferase, transferring glycosyl groups, putative, expressed  no original description 	T
11.8	lipid metabolism.'exotics'(steroids, squalene etc)	MDP0000195910	 weakly similar to ( 110) AT1G10030 | Symbols: ERG28 | ERG28 (Arabidopsis homolog of yeast ergosterol28) | chr1:3273972-3275631 FORWARDweakly similar to ( 110) loc_os12g43670 12012.m08141 protein erg28 like protein, expressed no original description 	T
2.2.1.5	major CHO metabolism.degradation.sucrose.Susy	MDP0000126946	 nearly identical (1372) AT4G02280 | Symbols: SUS3, ATSUS3 | SUS3 (sucrose synthase 3); UDP-glycosyltransferase/ sucrose synthase/ transferase, transferring glycosyl groups | chr4:995166-998719 FORWARDnearly identical (1362) SUS2_PEA Sucrose synthase 2 (EC 2.4.1.13) (Sucrose-UDP glucosyltransferase 2) - Pisum sativum (Garden pea)nearly identical (1321) loc_os03g22120 12003.m34861 protein sucrose synthase 2, putative, expressed  no original description 	T
29.3.4.99	protein.targeting.secretory pathway.unspecified	MDP0000209144	 moderately similar to ( 360) AT5G14670 | Symbols: ATARFA1B | ATARFA1B (ADP-ribosylation factor A1B); GTP binding / phospholipase activator/ protein binding | chr5:4729319-4730495 FORWARDmoderately similar to ( 361) ARF2_ORYSA ADP-ribosylation factor 2 - Oryza sativa (Rice)moderately similar to ( 363) loc_os05g41060 12005.m083767 protein ADP-ribosylation factor, putative, expressed  no original description 	T
30.5	signalling.G-proteins	MDP0000866956	 moderately similar to ( 380) AT5G60860 | Symbols: AtRABA1f | AtRABA1f (Arabidopsis Rab GTPase homolog A1f); GTP binding | chr5:24484750-24485565 FORWARDmoderately similar to ( 372) RB11B_TOBAC Ras-related protein Rab11B - Nicotiana tabacum (Common tobacco)moderately similar to ( 378) loc_os05g48980 12005.m08969 protein ras-related protein Rab11B, putative, expressed no original description 	T
13.1.3.5.5	amino acid metabolism.synthesis.aspartate family.lysine.diaminopimelate decarboxylase	MDP0000242384	 highly similar to ( 778) AT2G16370 | Symbols: THY-1 | THY-1 (THYMIDYLATE SYNTHASE 1); dihydrofolate reductase/ thymidylate synthase | chr2:7082038-7084333 REVERSEhighly similar to ( 806) DRTS_SOYBN Bifunctional dihydrofolate reductase-thymidylate synthase (DHFR-TS) [Includes: Dihydrofolate reductase (EC 1.5.1.3); Thymidylate synthase (EC 2.1.1.45)] - Glycine max (Soybean)highly similar to ( 773) loc_os11g29390 12011.m06849 protein bifunctional dihydrofolate reductase-thymidylate synthase, putative, expressed   no original description 	T
33.99	development.unspecified	MDP0000126455	 moderately similar to ( 347) AT1G10200 | Symbols: WLIM1 | WLIM1; transcription factor/ zinc ion binding | chr1:3346677-3347763 REVERSEmoderately similar to ( 279) SF3_HELAN Pollen-specific protein SF3 - Helianthus annuus (Common sunflower)moderately similar to ( 325) loc_os12g32620 12012.m07062 protein pollen-specific protein SF3, putative, expressed no original description 	T
29.7	protein.glycosylation	MDP0000120172	 highly similar to ( 582) AT5G66680 | Symbols: DGL1 | DGL1; dolichyl-diphosphooligosaccharide-protein glycotransferase | chr5:26617840-26620581 REVERSEhighly similar to ( 566) loc_os07g10830 12007.m05538 protein dolichyl-diphosphooligosaccharide--protein glycosyltransferase 48 kDasubunit precursor, putative, expressed   no original description 	T
27.3.67	RNA.regulation of transcription.putative transcription regulator	MDP0000252504	 nearly identical (1536) AT1G02080 | Symbols:  | transcriptional regulator-related | chr1:373694-386682 FORWARDnearly identical (1285) loc_os10g40780 12010.m21965 protein transcription regulator, putative, expressed   no original description 	T
35.2	not assigned.unknown	MDP0000164046	 moderately similar to ( 246) AT3G16640 | Symbols: TCTP | TCTP (TRANSLATIONALLY CONTROLLED TUMOR PROTEIN) | chr3:5669709-5670729 REVERSEmoderately similar to ( 275) TCTP_ORYSA Translationally-controlled tumor protein homolog (TCTP) - Oryza sativa (Rice)moderately similar to ( 275) loc_os11g43900 12011.m08225 protein translationally-controlled tumor protein, putative, expressed  no original description 	T
27.3.80	RNA.regulation of transcription.zf-HD	MDP0000249735	 moderately similar to ( 201) AT1G14440 | Symbols: AtHB31 | AtHB31 (ARABIDOPSIS THALIANA HOMEOBOX PROTEIN 31); DNA binding / transcription factor | chr1:4939076-4940014 REVERSEweakly similar to ( 181) loc_os12g10630 12012.m05046 protein ZF-HD homeobox protein, putative, expressed   no original description 	T
29.5.11.4.3.2	protein.degradation.ubiquitin.E3.SCF.FBOX	MDP0000264934	 moderately similar to ( 332) AT5G67140 | Symbols:  | F-box family protein | chr5:26794009-26795213 REVERSEmoderately similar to ( 274) loc_os06g07000 12006.m31842 protein F-box domain containing protein, expressed  no original description 	T
26.6	misc.O-methyl transferases	MDP0000271124	 highly similar to ( 542) AT5G54160 | Symbols: ATOMT1, OMT1 | ATOMT1 (O-METHYLTRANSFERASE 1); caffeate O-methyltransferase/ myricetin 3'-O-methyltransferase/ quercetin 3-O-methyltransferase | chr5:21982075-21984167 FORWARDhighly similar to ( 560) COMT1_MEDSA Caffeic acid 3-O-methyltransferase (EC 2.1.1.68) (S-adenosysl-L-methionine:caffeic acid 3-O-methyltransferase) (COMT) (CAOMT) - Medicago sativa (Alfalfa)moderately similar to ( 395) loc_os08g06100 12008.m04749 protein quercetin 3-O-methyltransferase 1, putative, expressed  no original description 	T
16.2	secondary metabolism.phenylpropanoids	MDP0000271124	 highly similar to ( 542) AT5G54160 | Symbols: ATOMT1, OMT1 | ATOMT1 (O-METHYLTRANSFERASE 1); caffeate O-methyltransferase/ myricetin 3'-O-methyltransferase/ quercetin 3-O-methyltransferase | chr5:21982075-21984167 FORWARDhighly similar to ( 560) COMT1_MEDSA Caffeic acid 3-O-methyltransferase (EC 2.1.1.68) (S-adenosysl-L-methionine:caffeic acid 3-O-methyltransferase) (COMT) (CAOMT) - Medicago sativa (Alfalfa)moderately similar to ( 395) loc_os08g06100 12008.m04749 protein quercetin 3-O-methyltransferase 1, putative, expressed  no original description 	T
26.4.1	misc.beta 1,3 glucan hydrolases.glucan endo-1,3-beta-glucosidase	MDP0000321326	 highly similar to ( 640) AT2G05790 | Symbols:  | glycosyl hydrolase family 17 protein | chr2:2199450-2201293 FORWARDmoderately similar to ( 259) E13B_WHEAT Glucan endo-1,3-beta-glucosidase precursor (EC 3.2.1.39) ((1->3)-beta-glucan endohydrolase) ((1->3)-beta-glucanase) (Beta-1,3-endoglucanase) - Triticum aestivum (Wheat)moderately similar to ( 491) loc_os02g53200 12002.m10349 protein glucan endo-1,3-beta-glucosidase 7 precursor, putative, expressed  no original description 	T
35.2	not assigned.unknown	MDP0000174273	 weakly similar to ( 159) AT4G19100 | Symbols:  | unknown protein | chr4:10453453-10454359 FORWARDweakly similar to ( 135) loc_os06g21530 12006.m06810 protein ABC-type Co2+ transport system, permease component, putative, expressed  no original description 	T
30.2.11	signalling.receptor kinases.leucine rich repeat XI	MDP0000256952	 highly similar to ( 790) AT2G42810 | Symbols: PP5, PP5.2 | PP5.2 (PROTEIN PHOSPHATASE 5.2); phosphoprotein phosphatase/ protein binding / protein serine/threonine phosphatase | chr2:17812336-17815896 REVERSEmoderately similar to ( 223) PP1_ORYSA Serine/threonine-protein phosphatase PP1 (EC 3.1.3.16) - Oryza sativa (Rice)highly similar to ( 541) loc_os05g11550 12005.m083649 protein serine/threonine-protein phosphatase 5, putative, expressed   no original description 	T
29.5.5	protein.degradation.serine protease	MDP0000193126	 weakly similar to ( 129) AT1G49970 | Symbols: CLPR1, NCLPP5, SVR2 | CLPR1; serine-type endopeptidase | chr1:18501936-18504462 REVERSEvery weakly similar to (93.6) loc_os05g51450 12005.m09210 protein endopeptidase Clp, putative, expressed no original description 	T
27.2	RNA.transcription	MDP0000272676	 highly similar to ( 932) AT4G19210 | Symbols: ATRLI2, RLI2 | ATRLI2; transporter | chr4:10501906-10504776 FORWARDvery weakly similar to (68.6) CYSA_CHLVU Probable sulfate/thiosulfate import ATP-binding protein cysA (EC 3.6.3.25) (Sulfate-transporting ATPase) - Chlorella vulgaris (Green alga)highly similar to ( 932) loc_os11g34350 12011.m07293 protein ATP-binding cassette sub-family E member 1, putative, expressed  no original description 	T
34.16	transport.ABC transporters and multidrug resistance systems	MDP0000272676	 highly similar to ( 932) AT4G19210 | Symbols: ATRLI2, RLI2 | ATRLI2; transporter | chr4:10501906-10504776 FORWARDvery weakly similar to (68.6) CYSA_CHLVU Probable sulfate/thiosulfate import ATP-binding protein cysA (EC 3.6.3.25) (Sulfate-transporting ATPase) - Chlorella vulgaris (Green alga)highly similar to ( 932) loc_os11g34350 12011.m07293 protein ATP-binding cassette sub-family E member 1, putative, expressed  no original description 	T
26.10	misc.cytochrome P450	MDP0000679736	 highly similar to ( 808) AT1G11680 | Symbols: CYP51G1, EMB1738, CYP51A2, CYP51 | CYP51G1 (CYTOCHROME P450 51G1); oxygen binding / sterol 14-demethylase | chr1:3938925-3940585 FORWARDhighly similar to ( 754) CP51_SORBI Cytochrome P450 51 (EC 1.14.13.70) (CYPLI) (P450-LIA1) (Obtusifoliol 14-alpha demethylase) - Sorghum bicolor (Sorghum) (Sorghum vulgare)highly similar to ( 749) loc_os11g32240 12011.m07126 protein cytochrome P450 51, putative, expressed   no original description 	T
19.7	tetrapyrrole synthesis.uroporphyrinogen decarboxylase	MDP0000185869	 highly similar to ( 601) AT3G14930 | Symbols: HEME1 | HEME1; uroporphyrinogen decarboxylase | chr3:5021290-5022577 FORWARDmoderately similar to ( 380) DCUP_TOBAC Uroporphyrinogen decarboxylase, chloroplast precursor (EC 4.1.1.37) (URO-D) (UPD) - Nicotiana tabacum (Common tobacco)highly similar to ( 552) loc_os01g43390 12001.m150657 protein uroporphyrinogen decarboxylase, chloroplast precursor, putative, expressed  no original description 	T
33.99	development.unspecified	MDP0000244589	 moderately similar to ( 367) AT1G04770 | Symbols:  | male sterility MS5 family protein | chr1:1336564-1337767 REVERSEmoderately similar to ( 379) loc_os05g43040 12005.m08434 protein pollenless3, putative, expressed no original description 	T
29.5.11.4.3.2	protein.degradation.ubiquitin.E3.SCF.FBOX	MDP0000308224	 moderately similar to ( 246) AT5G57900 | Symbols: SKIP1 | SKIP1 (SKP1 INTERACTING PARTNER 1); protein binding | chr5:23449916-23450915 REVERSEmoderately similar to ( 250) loc_os08g16840 12008.m05805 protein ubiquitin-protein ligase, putative, expressed  no original description 	T
11.3	lipid metabolism.Phospholipid synthesis	MDP0000174766	 moderately similar to ( 498) AT1G02390 | Symbols: ATGPAT2, GPAT2 | GPAT2 (GLYCEROL-3-PHOSPHATE ACYLTRANSFERASE 2); acyltransferase | chr1:480921-483163 FORWARDmoderately similar to ( 464) loc_os11g45400 12011.m08372 protein glycerol-3-phosphate acyltransferase 1, putative, expressed    Sporozoite_P67 PRK08026  no original description 	T
29.5.3	protein.degradation.cysteine protease	MDP0000145032	 moderately similar to ( 233) AT3G12490 | Symbols: ATCYSB | cysteine protease inhibitor, putative / cystatin, putative | chr3:3960523-3961777 REVERSEweakly similar to ( 124) CYTI_VIGUN Cysteine proteinase inhibitor (Cystatin) - Vigna unguiculata (Cowpea)moderately similar to ( 240) loc_os01g16430 12001.m08239 protein multidomain cystatin, putative, expressed  no original description 	T
17.5.1	hormone metabolism.ethylene.synthesis-degradation	MDP0000523579	 moderately similar to ( 220) AT2G36690 | Symbols:  | oxidoreductase, 2OG-Fe(II) oxygenase family protein | chr2:15379930-15381987 FORWARDweakly similar to ( 142) FLS_PETHY Flavonol synthase/flavanone 3-hydroxylase (EC 1.14.11.23) (EC 1.14.11.9) (FLS) - Petunia hybrida (Petunia)moderately similar to ( 229) loc_os08g37456 12008.m080186 protein flavonol synthase/flavanone 3-hydroxylase, putative, expressed  no original description 	T
13.1.6.5.4	amino acid metabolism.synthesis.aromatic aa.tryptophan.indole-3-glycerol phosphate synthase	MDP0000289149	 highly similar to ( 521) AT5G48220 | Symbols:  | indole-3-glycerol phosphate synthase, putative | chr5:19550439-19552046 FORWARDmoderately similar to ( 466) loc_os04g39270 12004.m35153 protein indole-3-glycerol phosphate synthase, chloroplast precursor, putative, expressed  no original description 	T
27.3.10	RNA.regulation of transcription.C2C2(Zn) YABBY family	MDP0000261432	 weakly similar to ( 148) AT2G26580 | Symbols: YAB5 | YAB5 (YABBY5); transcription factor | chr2:11303923-11306739 REVERSEvery weakly similar to (94.0) YAB1_ORYSA Protein YABBY (OsYAB1) (Filamentous flower protein 1) - Oryza sativa (Rice)weakly similar to ( 108) loc_os03g44710 12003.m101420 protein axial regulator YABBY2, putative, expressed no original description 	T
21.4	redox.glutaredoxins	MDP0000341028	 weakly similar to ( 191) AT4G28730 | Symbols:  | glutaredoxin family protein | chr4:14199174-14200712 FORWARDvery weakly similar to (95.1) GLRX_ORYSA Glutaredoxin - Oryza sativa (Rice)weakly similar to ( 182) loc_os08g45140 12008.m08484 protein OsGrx_S12 - glutaredoxin subgroup I, expressed  no original description 	T
35.2	not assigned.unknown	MDP0000290184	 highly similar to ( 753) AT1G10180 | Symbols:  | unknown protein | chr1:3338381-3341494 REVERSEhighly similar to ( 657) loc_os07g38060 12007.m08055 protein expressed protein  no original description 	T
34.9	transport.metabolite transporters at the mitochondrial membrane	MDP0000230768	 highly similar to ( 736) AT5G64290 | Symbols: DCT, DIT2.1 | DIT2.1 (DICARBOXYLATE TRANSPORT 2.1); oxoglutarate:malate antiporter | chr5:25714495-25716642 REVERSEmoderately similar to ( 446) SOT1_SPIOL 2-oxoglutarate/malate translocator, chloroplast precursor - Spinacia oleracea (Spinach)highly similar to ( 736) loc_os08g37600 12008.m07746 protein 2-oxoglutarate/malate translocator, chloroplast precursor, putative, expressed   no original description 	T
29.3.1	protein.targeting.nucleus	MDP0000277629	 moderately similar to ( 275) AT5G43960 | Symbols:  | nuclear transport factor 2 (NTF2) family protein / RNA recognition motif (RRM)-containing protein | chr5:17689154-17691653 REVERSEmoderately similar to ( 253) loc_os02g03890 12002.m05738 protein RNA binding protein, putative, expressed  no original description 	T
29.5.11.1	protein.degradation.ubiquitin.ubiquitin	MDP0000149912	 highly similar to ( 518) AT2G30100 | Symbols:  | ubiquitin family protein | chr2:12847828-12851908 FORWARDmoderately similar to ( 465) loc_os05g28500 12005.m07135 protein expressed protein  no original description 	T
13.1.5.1.1	amino acid metabolism.synthesis.serine-glycine-cysteine group.serine.phosphoglycerate dehydrogenase	MDP0000911918	 highly similar to ( 701) AT3G19480 | Symbols:  | D-3-phosphoglycerate dehydrogenase, putative / 3-PGDH, putative | chr3:6752590-6754650 FORWARDvery weakly similar to (79.0) FDH1_ORYSA Formate dehydrogenase 1, mitochondrial precursor (EC 1.2.1.2) (NAD-dependent formate dehydrogenase 1) (FDH 1) - Oryza sativa (Rice)highly similar to ( 717) loc_os04g55720 12004.m35214 protein D-3-phosphoglycerate dehydrogenase, chloroplast precursor, putative, expressed   no original description 	T
29.2.2.3.5	protein.synthesis.ribosome biogenesis.Pre-rRNA processing and modifications.DExD-box helicases	MDP0000222814	 moderately similar to ( 489) AT1G17690 | Symbols:  | unknown protein | chr1:6082878-6088157 REVERSEmoderately similar to ( 368) loc_os05g22920 12005.m64182 protein expressed protein no original description 	T
29.2.3	protein.synthesis.initiation	MDP0000217678	 weakly similar to ( 172) AT2G24060 | Symbols:  | translation initiation factor 3 (IF-3) family protein | chr2:10229453-10231390 FORWARDweakly similar to ( 146) loc_os02g55120 12002.m10538 protein translation initiation factor IF-3, chloroplast precursor, putative, expressed   no original description 	T
13.2.5.2	amino acid metabolism.degradation.serine-glycine-cysteine group.glycine	MDP0000588069	 nearly identical (1789) AT4G33010 | Symbols: AtGLDP1 | AtGLDP1 (Arabidopsis thaliana glycine decarboxylase P-protein 1); catalytic/ glycine dehydrogenase (decarboxylating)/ pyridoxal phosphate binding | chr4:15927133-15931150 REVERSEnearly identical (1798) GCSP_SOLTU Glycine dehydrogenase [decarboxylating], mitochondrial precursor (EC 1.4.4.2) (Glycine decarboxylase) (Glycine cleavage system P-protein) - Solanum tuberosum (Potato)nearly identical (1670) loc_os01g51410 12001.m42703 protein glycine dehydrogenase, mitochondrial precursor, putative, expressed   no original description 	T
35.1	not assigned.no ontology	MDP0000141762	 moderately similar to ( 335) AT3G47590 | Symbols:  | esterase/lipase/thioesterase family protein | chr3:17536133-17537515 REVERSEmoderately similar to ( 305) loc_os01g39790 12001.m43070 protein esterase, putative, expressed no original description 	T
34.16	transport.ABC transporters and multidrug resistance systems	MDP0000842161	 weakly similar to ( 179) AT3G59140 | Symbols: ATMRP14 | ATMRP14; ATPase, coupled to transmembrane movement of substances | chr3:21863519-21868701 REVERSEweakly similar to ( 169) loc_os06g06440 12006.m05369 protein multidrug resistance-associated protein 14, putative, expressed no original description 	T
30.3	signalling.calcium	MDP0000408054	 moderately similar to ( 233) AT1G01110 | Symbols: IQD18 | IQD18 (IQ-domain 18) | chr1:52239-54494 FORWARDweakly similar to ( 187) loc_os08g02250 12008.m04369 protein calmodulin binding protein, putative, expressed  no original description 	T
34.3	transport.amino acids	MDP0000384437	 highly similar to ( 684) AT2G38120 | Symbols: AUX1, WAV5, PIR1, MAP1 | AUX1 (AUXIN RESISTANT 1); amino acid transmembrane transporter/ auxin binding / auxin influx transmembrane transporter/ transporter | chr2:15973493-15976792 FORWARDhighly similar to ( 688) LAX2_MEDTR Auxin transporter-like protein 2 (AUX1-like protein 2) (MtLAX2) - Medicago truncatula (Barrel medic)highly similar to ( 670) loc_os01g63770 12001.m43433 protein auxin transporter-like protein 1, putative, expressed  no original description 	T
31.1	cell.organisation	MDP0000951799	 highly similar to ( 830) AT5G23860 | Symbols: TUB8 | TUB8; structural constituent of cytoskeleton | chr5:8042962-8044528 FORWARDhighly similar to ( 828) TBB2_ORYSA Tubulin beta-2 chain (Beta-2 tubulin) - Oryza sativa (Rice)highly similar to ( 828) loc_os03g01530 12003.m05700 protein tubulin beta-2 chain, putative, expressed  no original description 	T
30.2.11	signalling.receptor kinases.leucine rich repeat XI	MDP0000199056	 highly similar to ( 972) AT5G06940 | Symbols:  | leucine-rich repeat family protein | chr5:2148078-2150771 REVERSEmoderately similar to ( 316) PSKR_DAUCA Phytosulfokine receptor precursor (EC 2.7.11.1) (Phytosulfokine LRR receptor kinase) - Daucus carota (Carrot)highly similar to ( 712) loc_os04g39650 12004.m08952 protein receptor protein kinase CLAVATA1 precursor, putative, expressed   no original description 	T
11.9.3.4	lipid metabolism.lipid degradation.lysophospholipases.phospholipase A2	MDP0000366609	 moderately similar to ( 221) AT2G06925 | Symbols: ATSPLA2-ALPHA, PLA2-ALPHA | PLA2-ALPHA; phospholipase A2 | chr2:2842475-2843212 REVERSEweakly similar to ( 176) loc_os03g50030 12003.m09999 protein phospholipase A2, putative, expressed  no original description 	T
34.16	transport.ABC transporters and multidrug resistance systems	MDP0000757954	 weakly similar to ( 171) AT4G31390 | Symbols:  | ABC1 family protein | chr4:15233126-15236764 FORWARDweakly similar to ( 161) loc_os11g11000 12011.m079963 protein ubiquinone biosynthesis protein ubiB, putative, expressed  no original description 	T
17.1.3	hormone metabolism.abscisic acid.induced-regulated-responsive-activated	MDP0000324254	 weakly similar to ( 166) AT4G24960 | Symbols: ATHVA22D | ATHVA22D | chr4:12828401-12828982 FORWARDweakly similar to ( 169) HVA22_HORVU Protein HVA22 - Hordeum vulgare (Barley)weakly similar to ( 171) loc_os11g30500 12011.m06958 protein HVA22-like protein e, putative, expressed no original description 	T
29.4.1.57	protein.postranslational modification.kinase.receptor like cytoplasmatic kinase VII	MDP0000231519	 moderately similar to ( 473) AT5G35960 | Symbols:  | protein kinase, putative | chr5:14108524-14110536 REVERSEweakly similar to ( 188) NORK_MEDTR Nodulation receptor kinase precursor (EC 2.7.11.1) (Does not make infections protein 2) (Symbiosis receptor-like kinase) (MtSYMRK) - Medicago truncatula (Barrel medic)moderately similar to ( 387) loc_os06g45350 12006.m31915 protein ATP binding protein, putative, expressed  no original description 	T
27.3.8	RNA.regulation of transcription.C2C2(Zn) DOF zinc finger family	MDP0000611302	 weakly similar to ( 183) AT2G28510 | Symbols:  | Dof-type zinc finger domain-containing protein | chr2:12199383-12200627 REVERSEweakly similar to ( 135) PBF_MAIZE Dof zinc finger protein PBF (Prolamin box-binding factor) - Zea mays (Maize)weakly similar to ( 132) loc_os02g49440 12002.m09978 protein expressed protein   no original description 	T
9.9	mitochondrial electron transport / ATP synthesis.F1-ATPase 	MDP0000566711	 moderately similar to ( 476) AT2G33040 | Symbols:  | ATP synthase gamma chain, mitochondrial (ATPC) | chr2:14018978-14021047 REVERSEmoderately similar to ( 498) ATPG3_IPOBA ATP synthase gamma chain, mitochondrial precursor (EC 3.6.3.14) - Ipomoea batatas (Sweet potato) (Batate)moderately similar to ( 456) loc_os10g17280 12010.m04815 protein ATP synthase gamma chain, mitochondrial precursor, putative, expressed   no original description 	T
17.7.1.2	hormone metabolism.jasmonate.synthesis-degradation.lipoxygenase	MDP0000172092	 nearly identical (1093) AT1G55020 | Symbols: LOX1 | LOX1; lipoxygenase | chr1:20525798-20530143 FORWARDnearly identical (1107) LOX1_SOLTU Lipoxygenase 1 (EC 1.13.11.12) - Solanum tuberosum (Potato)highly similar to ( 913) loc_os03g49380 12003.m35371 protein lipoxygenase 4, putative, expressed no original description 	T
27.3.70	RNA.regulation of transcription.Silencing Group	MDP0000310071	 moderately similar to ( 295) AT1G66740 | Symbols: AtSP7, SP7, SGA2, ASF1A | SGA2 | chr1:24892586-24893548 FORWARDmoderately similar to ( 304) loc_os05g48030 12005.m27955 protein anti-silencing protein 1, putative, expressed  no original description 	T
27.3.24	RNA.regulation of transcription.MADS box transcription factor family	MDP0000233948	 moderately similar to ( 234) AT2G22540 | Symbols: SVP, AGL22 | SVP (SHORT VEGETATIVE PHASE); transcription factor/ translation repressor, nucleic acid binding | chr2:9580417-9583603 FORWARDweakly similar to ( 174) MAD22_ORYSA MADS-box transcription factor 22 (OsMADS22) - Oryza sativa (Rice)weakly similar to ( 174) loc_os02g52340 12002.m10265 protein MADS-box transcription factor 22, putative, expressed  no original description 	T
11.9.4.13	lipid metabolism.lipid degradation.beta-oxidation.acyl CoA reductase	MDP0000185343	 moderately similar to ( 316) AT4G33790 | Symbols: CER4, G7, FAR3 | CER4 (ECERIFERUM 4); fatty acyl-CoA reductase (alcohol-forming)/ oxidoreductase, acting on the CH-CH group of donors | chr4:16204325-16207891 REVERSEmoderately similar to ( 249) loc_os08g20200 12008.m06087 protein male sterility protein 2, putative, expressed  no original description 	T
27.3.4	RNA.regulation of transcription.ARF, Auxin Response Factor family	MDP0000143749	 highly similar to ( 989) AT1G19850 | Symbols: MP, ARF5, IAA24 | MP (MONOPTEROS); transcription factor | chr1:6887353-6891182 FORWARDvery weakly similar to (57.8) IAA17_ORYSA Auxin-responsive protein IAA17 (Indoleacetic acid-induced protein 17) - Oryza sativa (Rice)highly similar to ( 576) loc_os02g06910 12002.m06038 protein auxin response factor 6, putative, expressed   no original description 	T
11.8	lipid metabolism.'exotics'(steroids, squalene etc)	MDP0000393227	 moderately similar to ( 317) AT5G54500 | Symbols: FQR1 | FQR1 (FLAVODOXIN-LIKE QUINONE REDUCTASE 1); FMN binding / oxidoreductase, acting on NADH or NADPH, quinone or similar compound as acceptor | chr5:22124674-22126256 FORWARDmoderately similar to ( 320) loc_os08g04460 12008.m04589 protein minor allergen Alt a 7, putative, expressed  no original description 	T
27.3.67	RNA.regulation of transcription.putative transcription regulator	MDP0000810896	 highly similar to ( 791) AT3G28720 | Symbols:  | unknown protein | chr3:10782276-10784339 FORWARDhighly similar to ( 684) loc_os06g38550 12006.m08390 protein expressed protein  TrbL Sporozoite_P67 PRK08026  no original description 	T
27.2	RNA.transcription	MDP0000611602	 moderately similar to ( 388) AT2G15430 | Symbols: RBP36A, RPB35.5A, NRPB3, NRPD3, NRPE3A | NRPB3; DNA binding / DNA-directed RNA polymerase/ protein dimerization | chr2:6733661-6735482 FORWARDmoderately similar to ( 370) loc_os09g02284 12009.m50207 protein DNA-directed RNA polymerase II 36 kDa polypeptide A, putative, expressed  no original description 	T
35.2	not assigned.unknown	MDP0000478232	 highly similar to ( 663) AT3G11760 | Symbols:  | unknown protein | chr3:3718529-3721123 FORWARDhighly similar to ( 637) loc_os03g44670 12003.m09506 protein expressed protein Sporozoite_P67  no original description 	T
27.3.3	RNA.regulation of transcription.AP2/EREBP, APETALA2/Ethylene-responsive element binding protein family	MDP0000542633	 weakly similar to ( 122) AT3G20310 | Symbols: ATERF-7, ATERF7, ERF7 | ERF7 (ETHYLINE RESPONSE FACTOR 7); DNA binding / protein binding / transcription factor/ transcription repressor | chr3:7085957-7086691 REVERSEweakly similar to ( 122) ERF3_TOBAC Ethylene-responsive transcription factor 3 (Ethylene-responsive element-binding factor 3 homolog) (EREBP-5) (NtERF5) - Nicotiana tabacum (Common tobacco)very weakly similar to (86.3) loc_os04g57340 12004.m10619 protein ethylene-responsive transcription factor 3, putative, expressed  no original description 	T
35.2	not assigned.unknown	MDP0000297541	 very weakly similar to (50.4) loc_os02g58010 12002.m10822 protein conserved hypothetical protein  no original description 	T
35.1	not assigned.no ontology	MDP0000582469	 moderately similar to ( 363) AT1G54780 | Symbols:  | thylakoid lumen 18.3 kDa protein | chr1:20439533-20440953 FORWARDmoderately similar to ( 345) loc_os05g33280 12005.m07560 protein beta-propeller domains of methanol dehydrogenase type, putative, expressed no original description 	T
35.2	not assigned.unknown	MDP0000180226	  no original description 	T
30.5	signalling.G-proteins	MDP0000176209	 moderately similar to ( 424) AT5G19320 | Symbols: RANGAP2 | RANGAP2 (RAN GTPASE ACTIVATING PROTEIN 2); RAN GTPase activator | chr5:6505310-6506947 REVERSEmoderately similar to ( 388) loc_os05g46560 12005.m27945 protein ran GTPase activating protein, putative, expressed no original description 	T
20.1.3	stress.biotic.signalling	MDP0000253215	 moderately similar to ( 354) AT3G48090 | Symbols: EDS1 | EDS1 (enhanced disease susceptibility 1); lipase/ signal transducer/ triacylglycerol lipase | chr3:17755553-17757292 REVERSEmoderately similar to ( 406) loc_os09g22450 12009.m05451 protein EDS1-like protein, putative, expressed  no original description 	T
31.1	cell.organisation	MDP0000525934	 highly similar to ( 843) AT5G19780 | Symbols: TUA5 | TUA5; structural constituent of cytoskeleton | chr5:6687212-6688926 FORWARDhighly similar to ( 842) TBA1_ORYSA Tubulin alpha-1 chain - Oryza sativa (Rice)highly similar to ( 842) loc_os07g38730 12007.m08118 protein tubulin alpha-1 chain, putative, expressed   no original description 	T
29.5.11.4.2	protein.degradation.ubiquitin.E3.RING	MDP0000319191	 moderately similar to ( 496) AT5G14420 | Symbols: RGLG2 | RGLG2 (RING domain Ligase2); ubiquitin-protein ligase | chr5:4648355-4650563 REVERSEmoderately similar to ( 465) loc_os01g68060 12001.m12910 protein copine-6, putative, expressed   no original description 	T
15.2	metal handling.binding, chelation and storage	MDP0000126820	 weakly similar to ( 120) AT1G23000 | Symbols:  | heavy-metal-associated domain-containing protein | chr1:8143614-8144865 FORWARDweakly similar to ( 121) loc_os01g03490 12001.m06987 protein heavy metal-associated domain containing protein, expressed   no original description 	T
29.5.11.4.2	protein.degradation.ubiquitin.E3.RING	MDP0000120367	 weakly similar to ( 102) AT2G35910 | Symbols:  | zinc finger (C3HC4-type RING finger) family protein | chr2:15073225-15073878 REVERSEvery weakly similar to (62.4) EL5_ORYSA E3 ubiquitin-protein ligase EL5 (EC 6.3.2.-) - Oryza sativa (Rice)weakly similar to ( 104) loc_os07g06560 12007.m05116 protein RING-H2 finger protein ATL2I, putative no original description 	T
35.1	not assigned.no ontology	MDP0000265111	 highly similar to ( 954) AT2G01970 | Symbols:  | endomembrane protein 70, putative | chr2:452197-454819 REVERSEhighly similar to ( 919) loc_os03g13380 12003.m06783 protein transmembrane 9 superfamily protein member 1 precursor, putative, expressed no original description 	T
35.2	not assigned.unknown	MDP0000852180	 weakly similar to ( 155) AT3G09085 | Symbols:  | unknown protein | chr3:2782238-2782576 FORWARDweakly similar to ( 129) loc_os03g15910 12003.m07028 protein transmembrane protein, putative, expressed no original description 	T
29.5.11.2	protein.degradation.ubiquitin.E1	MDP0000161175	 moderately similar to ( 391) AT5G50580 | Symbols: SAE1B, AT-SAE1-2 | SAE1B (SUMO-ACTIVATING ENZYME 1B); SUMO activating enzyme | chr5:20585500-20587401 FORWARDvery weakly similar to (81.6) UBE11_WHEAT Ubiquitin-activating enzyme E1 1 - Triticum aestivum (Wheat)moderately similar to ( 333) loc_os11g30410 12011.m080006 protein ubiquitin-like 1-activating enzyme E1A, putative, expressed  no original description 	T
29.2.1.2.1.23	protein.synthesis.ribosomal protein.eukaryotic.40S subunit.S23	MDP0000128876	 moderately similar to ( 270) AT5G02960 | Symbols:  | 40S ribosomal protein S23 (RPS23B) | chr5:693280-694396 REVERSEmoderately similar to ( 279) RS23_FRAAN 40S ribosomal protein S23 (S12) - Fragaria ananassa (Strawberry)moderately similar to ( 278) loc_os03g60400 12003.m10930 protein 40S ribosomal protein S23, putative, expressed  no original description 	T
34.16	transport.ABC transporters and multidrug resistance systems	MDP0000134135	 highly similar to ( 573) AT5G13750 | Symbols: ZIFL1 | ZIFL1 (ZINC INDUCED FACILITATOR-like 1); tetracycline:hydrogen antiporter | chr5:4438318-4440829 FORWARDhighly similar to ( 527) loc_os12g03899 12012.m04385 protein carbohydrate transporter/ sugar porter/ transporter, putative, expressed no original description 	T
35.1	not assigned.no ontology	MDP0000246856	 highly similar to ( 687) AT4G38650 | Symbols:  | glycosyl hydrolase family 10 protein | chr4:18063377-18065769 FORWARDhighly similar to ( 635) loc_os03g14010 12003.m34818 protein hydrolase, hydrolyzing O-glycosyl compounds, putative, expressed  no original description 	T
30.5	signalling.G-proteins	MDP0000436577	 moderately similar to ( 368) AT2G17800 | Symbols: ARAC1, ATGP2, ATRAC1, ROP3, ATROP3 | ARAC1; GTP binding | chr2:7740313-7741942 FORWARDmoderately similar to ( 367) RAC1_BETVU Rac-like GTP-binding protein RHO1 (RHO1Bv) - Beta vulgaris (Sugar beet)moderately similar to ( 358) loc_os02g02840 12002.m05634 protein rac-like GTP-binding protein 6, putative, expressed  no original description 	T
30.2.24	signalling.receptor kinases.S-locus glycoprotein like	MDP0000560467	 moderately similar to ( 440) AT1G78850 | Symbols:  | curculin-like (mannose-binding) lectin family protein | chr1:29642072-29643397 REVERSEmoderately similar to ( 424) EP1G_DAUCA Epidermis-specific secreted glycoprotein EP1 precursor (52/54 kDa medium protein) - Daucus carota (Carrot)weakly similar to ( 154) loc_os04g23760 12004.m07515 protein ATP binding protein, putative  no original description 	T
27.3.67	RNA.regulation of transcription.putative transcription regulator	MDP0000251454	 highly similar to ( 903) AT5G18230 | Symbols:  | transcription regulator NOT2/NOT3/NOT5 family protein | chr5:6021610-6027031 REVERSEhighly similar to ( 830) loc_os03g44900 12003.m35336 protein CCR4-NOT transcription complex subunit 3, putative, expressed   no original description 	T
29.5.11.20	protein.degradation.ubiquitin.proteasom	MDP0000294774	 moderately similar to ( 341) AT3G22630 | Symbols: PBD1, PRCGB | PBD1 (20S PROTEASOME BETA SUBUNIT D1); peptidase/ threonine-type endopeptidase | chr3:8009709-8010774 REVERSEmoderately similar to ( 291) PSB2_ORYSA Proteasome subunit beta type 2 (EC 3.4.25.1) (20S proteasome alpha subunit D) (20S proteasome subunit beta-4) - Oryza sativa (Rice)moderately similar to ( 291) loc_os03g48930 12003.m09894 protein proteasome subunit beta type 2, putative, expressed no original description 	T
26.10	misc.cytochrome P450	MDP0000389795	 moderately similar to ( 439) AT3G48270 | Symbols: CYP71A26 | CYP71A26; electron carrier/ heme binding / iron ion binding / monooxygenase/ oxygen binding | chr3:17876571-17878173 FORWARDmoderately similar to ( 441) C71A4_SOLME Cytochrome P450 71A4 (EC 1.14.-.-) (CYPLXXIA4) (P-450EG2) - Solanum melongena (Eggplant) (Aubergine)moderately similar to ( 341) loc_os01g12760 12001.m07889 protein cytochrome P450 71A6, putative, expressed  no original description 	T
8.1.9	TCA / org transformation.TCA.malate DH	MDP0000295823	 highly similar to ( 512) AT5G20040 | Symbols: ATIPT9 | ATIPT9; ATP binding / tRNA isopentenyltransferase | chr5:6767939-6770980 REVERSEmoderately similar to ( 465) MDHM_FRAAN Malate dehydrogenase, mitochondrial precursor (EC 1.1.1.37) - Fragaria ananassa (Strawberry)moderately similar to ( 445) loc_os05g49880 12005.m27985 protein malate dehydrogenase, mitochondrial precursor, putative, expressed   no original description 	T
16.8.5.1	secondary metabolism.flavonoids.isoflavones.isoflavone reductase	MDP0000239946	 moderately similar to ( 492) AT4G39230 | Symbols:  | isoflavone reductase, putative | chr4:18266024-18267604 REVERSEmoderately similar to ( 499) IFRH_SOLTU Isoflavone reductase homolog (EC 1.3.1.-) (CP100) - Solanum tuberosum (Potato)moderately similar to ( 436) loc_os06g27770 12006.m07322 protein isoflavone reductase, putative, expressed  no original description 	T
30.5	signalling.G-proteins	MDP0000288520	 moderately similar to ( 269) AT5G03530 | Symbols: ATRAB ALPHA, ATRAB, ATRAB18B, ATRABC2A, RABC2A | RABC2A (RAB GTPASE HOMOLOG C2A); GTP binding / GTP-dependent protein binding / myosin XI tail binding | chr5:885741-887061 REVERSEmoderately similar to ( 277) RS13_SOYBN 40S ribosomal protein S13 - Glycine max (Soybean)moderately similar to ( 265) loc_os01g01030 12001.m06750 protein monocopper oxidase precursor, putative, expressed   no original description 	T
29.7.5	protein.glycosylation.alpha-1,3-mannosyl-glycoprotein-beta-1,2-N-acetylglucosaminyltransferase(GnTI) 	MDP0000798095	 highly similar to ( 761) AT3G55260 | Symbols: HEXO1, ATHEX2 | HEXO1 (BETA-HEXOSAMINIDASE 1); beta-N-acetylhexosaminidase/ hexosaminidase/ hydrolase, hydrolyzing O-glycosyl compounds | chr3:20489317-20492858 FORWARDhighly similar to ( 721) loc_os05g02510 12005.m04787 protein beta-hexosaminidase beta chain precursor, putative, expressed  no original description 	T
29.7.8	protein.glycosylation.alpha-1,6-mannosyl-glycoprotein-beta-1,2-N-acetylglucosaminyltransferase(GnTII) 	MDP0000798095	 highly similar to ( 761) AT3G55260 | Symbols: HEXO1, ATHEX2 | HEXO1 (BETA-HEXOSAMINIDASE 1); beta-N-acetylhexosaminidase/ hexosaminidase/ hydrolase, hydrolyzing O-glycosyl compounds | chr3:20489317-20492858 FORWARDhighly similar to ( 721) loc_os05g02510 12005.m04787 protein beta-hexosaminidase beta chain precursor, putative, expressed  no original description 	T
29.2.1.2.2.19	protein.synthesis.ribosomal protein.eukaryotic.60S subunit.L19	MDP0000679282	 moderately similar to ( 311) AT1G02780 | Symbols: emb2386 | emb2386 (embryo defective 2386); structural constituent of ribosome | chr1:608120-609391 REVERSEweakly similar to ( 112) RL19_MAIZE 60S ribosomal protein L19 (Fragment) - Zea mays (Maize)moderately similar to ( 306) loc_os03g38260 12003.m08925 protein 60S ribosomal protein L19-3, putative, expressed   no original description 	T
35.2	not assigned.unknown	MDP0000668130	 weakly similar to ( 145) AT1G13360 | Symbols:  | unknown protein | chr1:4577229-4577813 REVERSEvery weakly similar to (77.8) loc_os01g04010 12001.m07037 protein expressed protein no original description 	T
8.2.99	TCA / org transformation.other organic acid transformatons.misc	MDP0000624696	 highly similar to ( 905) AT2G24270 | Symbols: ALDH11A3 | ALDH11A3; 3-chloroallyl aldehyde dehydrogenase/ glyceraldehyde-3-phosphate dehydrogenase (NADP+) | chr2:10327325-10329601 REVERSEhighly similar to ( 910) GAPN_NICPL NADP-dependent glyceraldehyde-3-phosphate dehydrogenase (EC 1.2.1.9) (Non-phosphorylating glyceraldehyde 3-phosphate dehydrogenase) (Glyceraldehyde-3-phosphate dehydrogenase [NADP+]) (Triosephosphate dehydrogenase) - Nicotiana plumbaginhighly similar to ( 892) loc_os08g34210 12008.m26702 protein NADP-dependent glyceraldehyde-3-phosphate dehydrogenase, putative, expressed   no original description 	T
1.3.4	PS.calvin cycle.GAP	MDP0000624696	 highly similar to ( 905) AT2G24270 | Symbols: ALDH11A3 | ALDH11A3; 3-chloroallyl aldehyde dehydrogenase/ glyceraldehyde-3-phosphate dehydrogenase (NADP+) | chr2:10327325-10329601 REVERSEhighly similar to ( 910) GAPN_NICPL NADP-dependent glyceraldehyde-3-phosphate dehydrogenase (EC 1.2.1.9) (Non-phosphorylating glyceraldehyde 3-phosphate dehydrogenase) (Glyceraldehyde-3-phosphate dehydrogenase [NADP+]) (Triosephosphate dehydrogenase) - Nicotiana plumbaginhighly similar to ( 892) loc_os08g34210 12008.m26702 protein NADP-dependent glyceraldehyde-3-phosphate dehydrogenase, putative, expressed   no original description 	T
5.10	fermentation.aldehyde dehydrogenase	MDP0000624696	 highly similar to ( 905) AT2G24270 | Symbols: ALDH11A3 | ALDH11A3; 3-chloroallyl aldehyde dehydrogenase/ glyceraldehyde-3-phosphate dehydrogenase (NADP+) | chr2:10327325-10329601 REVERSEhighly similar to ( 910) GAPN_NICPL NADP-dependent glyceraldehyde-3-phosphate dehydrogenase (EC 1.2.1.9) (Non-phosphorylating glyceraldehyde 3-phosphate dehydrogenase) (Glyceraldehyde-3-phosphate dehydrogenase [NADP+]) (Triosephosphate dehydrogenase) - Nicotiana plumbaginhighly similar to ( 892) loc_os08g34210 12008.m26702 protein NADP-dependent glyceraldehyde-3-phosphate dehydrogenase, putative, expressed   no original description 	T
27.2	RNA.transcription	MDP0000197246	 weakly similar to ( 174) AT5G51940 | Symbols: NRPB6A, NRPD6A, NRPE6A | NRPB6A; DNA binding / DNA-directed RNA polymerase | chr5:21104679-21105796 FORWARDweakly similar to ( 176) loc_os03g38960 12003.m08990 protein DNA-directed RNA polymerases I, II, and III 14.4 kDa polypeptide, putative, expressed  no original description 	T
21.1	redox.thioredoxin	MDP0000546099	 weakly similar to ( 142) AT5G39950 | Symbols: ATTRX2, ATH2, ATTRXH2, TRXH2 | ATTRX2 (THIOREDOXIN 2); oxidoreductase, acting on sulfur group of donors, disulfide as acceptor | chr5:15990885-15991881 REVERSEweakly similar to ( 129) TRXH_ORYSA Thioredoxin H-type (TRX-H) (Phloem sap 13 kDa protein 1) - Oryza sativa (Rice)weakly similar to ( 129) loc_os07g08840 12007.m079626 protein thioredoxin H-type, putative, expressed   no original description 	T
35.1.19	not assigned.no ontology.C2 domain-containing protein	MDP0000278649	 nearly identical (1437) AT3G57880 | Symbols:  | C2 domain-containing protein | chr3:21431198-21433519 REVERSEnearly identical (1338) loc_os05g30750 12005.m083576 protein phosphoribosylanthranilate transferase, putative, expressed   no original description 	T
35.2	not assigned.unknown	MDP0000365508	 very weakly similar to (72.4) AT5G08040 | Symbols: TOM5 | TOM5 (MITOCHONDRIAL IMPORT RECEPTOR SUBUNIT TOM5 HOMOLOG) | chr5:2577358-2578073 REVERSEvery weakly similar to (60.5) loc_os12g38650 12012.m07650 protein conserved hypothetical protein  no original description 	T
33.99	development.unspecified	MDP0000188194	 weakly similar to ( 162) AT3G63210 | Symbols: MARD1 | MARD1 | chr3:23354019-23354906 REVERSEweakly similar to ( 131) loc_os03g46260 12003.m101430 protein expressed protein  no original description 	T
27.3.99	RNA.regulation of transcription.unclassified	MDP0000315368	 weakly similar to ( 182) AT4G36970 | Symbols:  | remorin family protein | chr4:17429826-17431459 REVERSEweakly similar to ( 189) loc_os02g52810 12002.m33459 protein remorin, C-terminal region family protein, expressed  no original description 	T
27.3.7	RNA.regulation of transcription.C2C2(Zn) CO-like, Constans-like zinc finger family	MDP0000271388	 moderately similar to ( 237) AT1G68520 | Symbols:  | zinc finger (B-box type) family protein | chr1:25709331-25710749 REVERSEvery weakly similar to (55.5) PRR95_ORYSA Two-component response regulator-like PRR95 (Pseudo-response regulator 95) (OsPRR95) - Oryza sativa (Rice)moderately similar to ( 234) loc_os06g15330 12006.m06246 protein zinc finger protein CONSTANS-LIKE 16, putative, expressed   no original description 	T
29.4	protein.postranslational modification	MDP0000218678	 highly similar to ( 845) AT3G22790 | Symbols:  | kinase interacting family protein | chr3:8052446-8057888 REVERSEvery weakly similar to (88.2) MFP1_TOBAC MAR-binding filament-like protein 1-1 - Nicotiana tabacum (Common tobacco)moderately similar to ( 375) loc_os10g28610 12010.m05740 protein expressed protein   no original description 	T
35.1	not assigned.no ontology	MDP0000761754	 very weakly similar to (98.2) AT3G27160 | Symbols: GHS1 | GHS1 (GLUCOSE HYPERSENSITIVE 1); structural constituent of ribosome | chr3:10017531-10018854 FORWARDvery weakly similar to (60.1) RR21_SPIOL Chloroplast 30S ribosomal protein S21 (Fragment) - Spinacia oleracea (Spinach)very weakly similar to (67.4) loc_os01g11230 12001.m150508 protein heme-binding protein 2, putative, expressed  no original description 	T
26.10	misc.cytochrome P450	MDP0000260367	 highly similar to ( 796) AT1G31800 | Symbols: CYP97A3, LUT5 | CYP97A3 (CYTOCHROME P450-TYPE MONOOXYGENASE 97A3); carotene beta-ring hydroxylase/ oxygen binding | chr1:11396440-11399470 FORWARDmoderately similar to ( 316) C97B2_SOYBN Cytochrome P450 97B2 (EC 1.14.-.-) - Glycine max (Soybean)highly similar to ( 722) loc_os02g57290 12002.m33947 protein cytochrome P450 97B3, putative, expressed  MSP1_C  no original description 	T
33.3	development.squamosa promoter binding like (SPL)	MDP0000142582	 weakly similar to ( 153) AT5G50570 | Symbols:  | squamosa promoter-binding protein, putative | chr5:20582555-20583791 REVERSEweakly similar to ( 117) SBP2_ANTMA Squamosa promoter-binding protein 2 - Antirrhinum majus (Garden snapdragon)weakly similar to ( 152) loc_os09g32944 12009.m06381 protein teosinte glume architecture 1, putative, expressed   no original description 	T
35.2	not assigned.unknown	MDP0000207858	 moderately similar to ( 441) AT5G19050 | Symbols:  | unknown protein | chr5:6369307-6371726 FORWARDmoderately similar to ( 396) loc_os07g11060 12007.m28958 protein expressed protein  no original description 	T
10.1.5	cell wall.precursor synthesis.UXS	MDP0000194772	 highly similar to ( 689) AT5G28840 | Symbols: GME | GME (GDP-D-MANNOSE 3',5'-EPIMERASE); GDP-mannose 3,5-epimerase/ NAD or NADH binding / catalytic | chr5:10862472-10864024 REVERSEhighly similar to ( 686) GME1_ORYSA GDP-mannose 3,5-epimerase 1 (EC 5.1.3.18) (GDP-Man 3,5-epimerase 1) (OsGME-1) - Oryza sativa (Rice)highly similar to ( 686) loc_os10g28200 12010.m21888 protein GDP-mannose 3,5-epimerase 1, putative, expressed   no original description 	T
21.2.1.1	redox.ascorbate and glutathione.ascorbate.GME	MDP0000194772	 highly similar to ( 689) AT5G28840 | Symbols: GME | GME (GDP-D-MANNOSE 3',5'-EPIMERASE); GDP-mannose 3,5-epimerase/ NAD or NADH binding / catalytic | chr5:10862472-10864024 REVERSEhighly similar to ( 686) GME1_ORYSA GDP-mannose 3,5-epimerase 1 (EC 5.1.3.18) (GDP-Man 3,5-epimerase 1) (OsGME-1) - Oryza sativa (Rice)highly similar to ( 686) loc_os10g28200 12010.m21888 protein GDP-mannose 3,5-epimerase 1, putative, expressed   no original description 	T
35.1	not assigned.no ontology	MDP0000254162	 highly similar to ( 502) AT2G28315 | Symbols:  | LOCATED IN: membrane; CONTAINS InterPro DOMAIN/s: Protein of unknown function DUF250 (InterPro:IPR004853); BEST Arabidopsis thaliana protein match is: transporter-related (TAIR:AT1G06890.2); Has 1852 Blast hits to 1847 proteins in 207 species: Archae - 0; Bacteria - 58; Metazoa - 582; Fungi - 250; Plants - 685; Viruses - 0; Other Eukaryotes - 277 (source: NCBI BLink). | chr2:12088896-12090570 FORWARDvery weakly similar to (55.5) TPT_MAIZE Triose phosphate/phosphate translocator, chloroplast precursor (cTPT) - Zea mays (Maize)moderately similar to ( 477) loc_os02g41780 12002.m09215 protein integral membrane protein like, putative, expressed  no original description 	T
28.1	DNA.synthesis/chromatin structure	MDP0000302730	 nearly identical (1703) AT4G30100 | Symbols:  | tRNA-splicing endonuclease positive effector-related | chr4:14714191-14719335 FORWARDmoderately similar to ( 355) loc_os02g51610 12002.m33874 protein transporter-like protein, putative, expressed  no original description 	T
27.3.67	RNA.regulation of transcription.putative transcription regulator	MDP0000150371	 weakly similar to ( 143) AT3G52170 | Symbols:  | DNA binding | chr3:19347039-19349099 REVERSEvery weakly similar to (79.3) loc_os12g34350 12012.m07229 protein expressed protein  no original description 	T
26.2	misc.UDP glucosyl and glucoronyl transferases	MDP0000186170	 moderately similar to ( 479) AT2G22900 | Symbols:  | galactosyl transferase GMA12/MNN10 family protein | chr2:9744359-9746193 REVERSEmoderately similar to ( 401) loc_os02g49140 12002.m09948 protein glycosyltransferase 6, putative, expressed  MSP1_C  no original description 	T
28.1	DNA.synthesis/chromatin structure	MDP0000131617	 nearly identical (1482) AT5G67100 | Symbols: ICU2 | ICU2 (INCURVATA2); DNA-directed DNA polymerase | chr5:26776994-26785104 FORWARDnearly identical (1594) DPOLA_ORYSA DNA polymerase alpha catalytic subunit (EC 2.7.7.7) - Oryza sativa (Rice)nearly identical (1300) loc_os01g64820 12001.m12595 protein DNA polymerase alpha catalytic subunit, putative, expressed     MSP1_C  no original description 	T
17.2.3	hormone metabolism.auxin.induced-regulated-responsive-activated	MDP0000232281	 highly similar to ( 565) AT2G04850 | Symbols:  | auxin-responsive protein-related | chr2:1704298-1705608 FORWARDmoderately similar to ( 458) loc_os03g09850 12003.m06440 protein dopamine beta-monooxygenase, putative, expressed  PRK08026 TrbL Sporozoite_P67  no original description 	T
34.19.2	transport.Major Intrinsic Proteins.TIP	MDP0000460051	 moderately similar to ( 413) AT2G36830 | Symbols: GAMMA-TIP, TIP1;1, GAMMA-TIP1 | GAMMA-TIP (GAMMA TONOPLAST INTRINSIC PROTEIN); water channel | chr2:15445490-15446336 FORWARDmoderately similar to ( 404) TIP11_ORYSA Probable aquaporin TIP1.1 (Tonoplast intrinsic protein 1.1) (OsTIP1.1) (rTIP1) - Oryza sativa (Rice)moderately similar to ( 404) loc_os03g05290 12003.m06053 protein aquaporin TIP1.1, putative, expressed   no original description 	T
35.2	not assigned.unknown	MDP0000362412	 moderately similar to ( 416) AT4G12590 | Symbols:  | unknown protein | chr4:7451291-7452976 REVERSEmoderately similar to ( 372) loc_os09g32220 12009.m06311 protein protein pob, putative, expressed   no original description 	T
20.2.3	stress.abiotic.drought/salt	MDP0000286452	 highly similar to ( 996) AT1G26850 | Symbols:  | dehydration-responsive family protein | chr1:9301146-9303432 REVERSEhighly similar to ( 920) loc_os09g24900 12009.m22086 protein ankyrin protein kinase-like, putative, expressed   no original description 	T
26.7	misc.oxidases - copper, flavone etc	MDP0000266398	 highly similar to ( 929) AT4G12420 | Symbols: SKU5 | SKU5; copper ion binding / oxidoreductase | chr4:7349941-7352868 REVERSEhighly similar to ( 515) ASO_BRANA L-ascorbate oxidase homolog precursor (EC 1.10.3.3) (Ascorbase) - Brassica napus (Rape)highly similar to ( 824) loc_os06g01490 12006.m04887 protein monocopper oxidase precursor, putative, expressed no original description 	T
31.1	cell.organisation	MDP0000279407	 weakly similar to ( 140) AT3G18670 | Symbols:  | ankyrin repeat family protein | chr3:6424135-6426471 REVERSEvery weakly similar to (50.1) loc_os12g05520 12012.m04545 protein retrotransposon protein, putative, unclassified no original description 	T
27.3.3	RNA.regulation of transcription.AP2/EREBP, APETALA2/Ethylene-responsive element binding protein family	MDP0000939633	 moderately similar to ( 356) AT1G25560 | Symbols: TEM1 | TEM1 (TEMPRANILLO 1); transcription factor | chr1:8981891-8982976 REVERSEvery weakly similar to (62.4) ERF4_TOBAC Ethylene-responsive transcription factor 4 (Ethylene-responsive element-binding factor 4 homolog) (EREBP-3) (NtERF3) - Nicotiana tabacum (Common tobacco)moderately similar to ( 320) loc_os05g47650 12005.m08837 protein DNA-binding protein RAV1, putative, expressed  MSP1_C  no original description 	T
24.2	Biodegradation of Xenobiotics.lactoylglutathione lyase	MDP0000327504	 moderately similar to ( 250) AT5G57040 | Symbols:  | lactoylglutathione lyase family protein / glyoxalase I family protein | chr5:23084035-23085116 REVERSEmoderately similar to ( 224) loc_os07g06660 12007.m29075 protein glyoxalase/bleomycin resistance protein/dioxygenase, putative, expressed no original description 	T
26.8	misc.nitrilases, *nitrile lyases, berberine bridge enzymes, reticuline oxidases, troponine reductases	MDP0000188581	 moderately similar to ( 485) AT5G22300 | Symbols: NIT4 | NIT4 (NITRILASE 4); 3-cyanoalanine hydratase/ cyanoalanine nitrilase/ indole-3-acetonitrile nitrilase/ nitrilase/ nitrile hydratase | chr5:7379401-7381764 FORWARDhighly similar to ( 558) NRL4_TOBAC Nitrilase 4 (EC 3.5.5.1) - Nicotiana tabacum (Common tobacco)highly similar to ( 528) loc_os02g42350 12002.m100293 protein nitrilase 4, putative, expressed  no original description 	T
16.5.1.3.3	secondary metabolism.sulfur-containing.glucosinolates.degradation.nitrilase	MDP0000188581	 moderately similar to ( 485) AT5G22300 | Symbols: NIT4 | NIT4 (NITRILASE 4); 3-cyanoalanine hydratase/ cyanoalanine nitrilase/ indole-3-acetonitrile nitrilase/ nitrilase/ nitrile hydratase | chr5:7379401-7381764 FORWARDhighly similar to ( 558) NRL4_TOBAC Nitrilase 4 (EC 3.5.5.1) - Nicotiana tabacum (Common tobacco)highly similar to ( 528) loc_os02g42350 12002.m100293 protein nitrilase 4, putative, expressed  no original description 	T
27.3.21	RNA.regulation of transcription.GRAS transcription factor family	MDP0000289554	 highly similar to ( 507) AT5G41920 | Symbols:  | scarecrow transcription factor family protein | chr5:16779982-16781199 FORWARDmoderately similar to ( 213) RL27_PEA 60S ribosomal protein L27 - Pisum sativum (Garden pea)highly similar to ( 546) loc_os07g38030 12007.m08052 protein nodulation signaling pathway 2 protein, putative, expressed    no original description 	T
29.2.1.99.1.4	protein.synthesis.ribosomal protein.unknown.small subunit.S4	MDP0000300201	 moderately similar to ( 251) AT5G15750 | Symbols:  | RNA-binding S4 domain-containing protein | chr5:5141449-5142701 FORWARDmoderately similar to ( 242) loc_os04g56670 12004.m10554 protein 40S ribosomal protein S4-like, putative, expressed  no original description 	T
29.5.7	protein.degradation.metalloprotease	MDP0000306688	 moderately similar to ( 354) AT4G16640 | Symbols:  | matrix metalloproteinase, putative | chr4:9367396-9368490 REVERSEmoderately similar to ( 229) MEP1_SOYBN Metalloendoproteinase 1 precursor (EC 3.4.24.-) (SMEP1) - Glycine max (Soybean)moderately similar to ( 338) loc_os10g40830 12010.m06853 protein metalloendoproteinase 1 precursor, putative, expressed   no original description 	T
30.2.11	signalling.receptor kinases.leucine rich repeat XI	MDP0000876280	 nearly identical (1095) AT5G01890 | Symbols:  | leucine-rich repeat transmembrane protein kinase, putative | chr5:341661-344650 REVERSEmoderately similar to ( 348) RPK1_IPONI Receptor-like protein kinase precursor (EC 2.7.11.1) - Ipomoea nil (Japanese morning glory) (Pharbitis nil)nearly identical (1021) loc_os03g21730 12003.m07577 protein receptor-like protein kinase precursor, putative, expressed   no original description 	T
35.1.5	not assigned.no ontology.pentatricopeptide (PPR) repeat-containing protein	MDP0000731864	 nearly identical (1128) AT1G18900 | Symbols:  | pentatricopeptide (PPR) repeat-containing protein | chr1:6529778-6532360 FORWARDweakly similar to ( 145) RF1_ORYSA Rf1 protein, mitochondrial precursor (PPR protein) (Fertility restorer) (Restorer for CMS) - Oryza sativa (Rice)highly similar to ( 925) loc_os12g44170 12012.m08188 protein ATP binding protein, putative, expressed  no original description 	T
29.2.1.2.2.6	protein.synthesis.ribosomal protein.eukaryotic.60S subunit.L6	MDP0000417447	 weakly similar to ( 177) AT2G18400 | Symbols:  | ribosomal protein L6 family protein | chr2:7989665-7989973 REVERSEweakly similar to ( 139) RM06_MARPO Mitochondrial 60S ribosomal protein L6 - Marchantia polymorpha (Liverwort)weakly similar to ( 164) loc_os03g51510 12003.m101478 protein mitochondrial 60S ribosomal protein L6, putative, expressed no original description 	T
35.2	not assigned.unknown	MDP0000139654	 highly similar to ( 697) AT1G07970 | Symbols:  | unknown protein | chr1:2469528-2472518 REVERSEhighly similar to ( 615) loc_os03g17950 12003.m07220 protein expressed protein  no original description 	T
35.2	not assigned.unknown	MDP0000309260	  no original description 	T
35.1.5	not assigned.no ontology.pentatricopeptide (PPR) repeat-containing protein	MDP0000437580	 highly similar to ( 609) AT1G08610 | Symbols:  | pentatricopeptide (PPR) repeat-containing protein | chr1:2733788-2735467 REVERSEmoderately similar to ( 213) RF1_ORYSA Rf1 protein, mitochondrial precursor (PPR protein) (Fertility restorer) (Restorer for CMS) - Oryza sativa (Rice)moderately similar to ( 427) loc_os10g33700 12010.m21918 protein ATP binding protein, putative, expressed  no original description 	T
27.4	RNA.RNA binding	MDP0000320861	 moderately similar to ( 426) AT1G04020 | Symbols: ATBARD1, BARD1 | BARD1 (BREAST CANCER ASSOCIATED RING 1); DNA binding / transcription coactivator | chr1:1036610-1040045 FORWARDweakly similar to ( 104) ROC1_NICPL 30 kDa ribonucleoprotein, chloroplast precursor (CP-RBP30) - Nicotiana plumbaginifolia (Leadwort-leaved tobacco)moderately similar to ( 334) loc_os04g43300 12004.m09294 protein ATBRCA1, putative, expressed   no original description 	T
29.2.1.2.1.24	protein.synthesis.ribosomal protein.eukaryotic.40S subunit.S24	MDP0000199827	 weakly similar to ( 153) AT3G04920 | Symbols:  | 40S ribosomal protein S24 (RPS24A) | chr3:1360989-1362065 FORWARDweakly similar to ( 149) loc_os01g52490 12001.m11423 protein 40S ribosomal protein S24, putative, expressed no original description 	T
20.2.99	stress.abiotic.unspecified	MDP0000215167	 moderately similar to ( 217) AT2G47710 | Symbols:  | universal stress protein (USP) family protein | chr2:19555045-19555956 REVERSEmoderately similar to ( 208) loc_os12g36640 12012.m07450 protein fiber protein Fb19, putative, expressed  no original description 	T
30.2.11	signalling.receptor kinases.leucine rich repeat XI	MDP0000294206	 very weakly similar to (95.9) AT5G05180 | Symbols:  | unknown protein | chr5:1536924-1538318 FORWARDvery weakly similar to (63.5) RPK1_IPONI Receptor-like protein kinase precursor (EC 2.7.11.1) - Ipomoea nil (Japanese morning glory) (Pharbitis nil)very weakly similar to (74.7) loc_os06g38650 12006.m08404 protein receptor-like protein kinase precursor, putative, expressed  no original description 	T
26.28	misc.GDSL-motif lipase	MDP0000268854	 moderately similar to ( 492) AT3G04290 | Symbols: ATLTL1, LTL1 | LTL1 (LI-TOLERANT LIPASE 1); carboxylesterase/ hydrolase, acting on ester bonds | chr3:1133620-1136223 REVERSEweakly similar to ( 179) APG_BRANA Anter-specific proline-rich protein APG (Protein CEX) (Fragment) - Brassica napus (Rape)moderately similar to ( 469) loc_os02g40440 12002.m09130 protein anther-specific proline-rich protein APG precursor, putative, expressed  no original description 	T
28.1.3.1	DNA.synthesis/chromatin structure.histone.H1	MDP0000477621	 weakly similar to ( 104) AT2G18050 | Symbols: HIS1-3 | HIS1-3 (HISTONE H1-3); DNA binding / nucleosomal DNA binding | chr2:7846254-7846670 FORWARDvery weakly similar to (78.6) H1_MAIZE Histone H1 - Zea mays (Maize)very weakly similar to (99.0) loc_os06g04020 12006.m05133 protein histone H1, putative, expressed no original description 	T
29.4	protein.postranslational modification	MDP0000747045	 highly similar to ( 546) AT5G58380 | Symbols: CIPK10, PKS2, SIP1, SNRK3.8 | SIP1 (SOS3-INTERACTING PROTEIN 1); ATP binding / kinase/ protein kinase/ protein serine/threonine kinase | chr5:23597092-23598531 REVERSEmoderately similar to ( 395) CIPK1_ORYSA CIPK-like protein 1 (EC 2.7.11.1) (OsCK1) - Oryza sativa (Rice)highly similar to ( 580) loc_os05g26820 12005.m06974 protein CBL-interacting serine/threonine-protein kinase 15, putative, expressed  MSP1_C  no original description 	T
29.4	protein.postranslational modification	MDP0000229807	 moderately similar to ( 224) AT3G23610 | Symbols:  | dual specificity protein phosphatase (DsPTP1) | chr3:8478280-8479641 FORWARDvery weakly similar to (90.9) PTP3_CHLEU Dual specificity protein phosphatase (EC 3.1.3.48) (EC 3.1.3.16) - Chlamydomonas eugametosweakly similar to ( 191) loc_os01g29469 12001.m09382 protein dual specificity protein phosphatase 9, putative, expressed  no original description 	T
35.2	not assigned.unknown	MDP0000227338	 weakly similar to ( 152) AT2G28580 | Symbols:  | unknown protein | chr2:12244885-12248270 FORWARDvery weakly similar to (99.8) loc_os11g34090 12011.m07267 protein expressed protein   no original description 	T
10.1.20	cell wall.precursor synthesis.phosphomannose isomerase	MDP0000271872	 highly similar to ( 530) AT3G02570 | Symbols: MEE31, PMI1 | MEE31 (MATERNAL EFFECT EMBRYO ARREST 31); mannose-6-phosphate isomerase | chr3:543463-545478 REVERSEmoderately similar to ( 487) loc_os01g03710 12001.m42847 protein mannose-6-phosphate isomerase, putative, expressed no original description 	T
34.13	transport.peptides and oligopeptides	MDP0000431120	 moderately similar to ( 259) AT5G64410 | Symbols: ATOPT4, OPT4 | OPT4 (OLIGOPEPTIDE TRANSPORTER 4); oligopeptide transporter | chr5:25750921-25754974 REVERSEmoderately similar to ( 245) loc_os01g43940 12001.m10653 protein oligopeptide transporter 4, putative no original description 	T
35.2	not assigned.unknown	MDP0000893240	  no original description 	T
35.2	not assigned.unknown	MDP0000290650	 moderately similar to ( 454) AT3G08010 | Symbols: ATAB2 | ATAB2; RNA binding | chr3:2556046-2557426 FORWARDmoderately similar to ( 422) loc_os02g39740 12002.m09060 protein tab2 protein, putative, expressed  no original description 	T
35.2	not assigned.unknown	MDP0000809215	 highly similar to ( 759) AT5G49970 | Symbols: ATPPOX, PDX3 | ATPPOX (A. THALIANA PYRIDOXIN (PYRODOXAMINE) 5'-PHOSPHATE OXIDASE); pyridoxamine-phosphate oxidase | chr5:20329213-20332900 FORWARDhighly similar to ( 726) loc_os10g23120 12010.m05317 protein pyridoxamine-phosphate oxidase, putative, expressed no original description 	T
35.1	not assigned.no ontology	MDP0000307520	 moderately similar to ( 365) AT3G16310 | Symbols:  | mitotic phosphoprotein N' end (MPPN) family protein | chr3:5526593-5528106 REVERSEweakly similar to ( 187) loc_os01g10350 12001.m07657 protein MPPN domain containing protein, expressed   no original description 	T
35.1	not assigned.no ontology	MDP0000136545	 moderately similar to ( 204) AT3G20270 | Symbols:  | lipid-binding serum glycoprotein family protein | chr3:7068879-7070866 FORWARDmoderately similar to ( 231) loc_os09g32240 12009.m06313 protein lipid binding protein, putative, expressed  no original description 	T
29.2.1.1.3.1.15	protein.synthesis.ribosomal protein.prokaryotic.unknown organellar.30S subunit.S15	MDP0000142241	 moderately similar to ( 235) AT1G80620 | Symbols:  | ribosomal protein S15 family protein | chr1:30305404-30307182 FORWARDmoderately similar to ( 223) loc_os06g07996 12006.m05522 protein ribosomal protein S15 containing protein   TrbL  no original description 	T
26.1	misc.misc2	MDP0000231713	 moderately similar to ( 491) AT1G13560 | Symbols: AAPT1, ATAAPT1 | AAPT1 (AMINOALCOHOLPHOSPHOTRANSFERASE 1); phosphatidyltransferase/ phosphotransferase, for other substituted phosphate groups | chr1:4638834-4641691 REVERSEmoderately similar to ( 467) loc_os02g02750 12002.m33990 protein ethanolaminephosphotransferase, putative, expressed  no original description 	T
29.2.1.2.2.28	protein.synthesis.ribosomal protein.eukaryotic.60S subunit.L28	MDP0000219309	 weakly similar to ( 141) AT2G19730 | Symbols:  | 60S ribosomal protein L28 (RPL28A) | chr2:8511752-8512995 FORWARDweakly similar to ( 143) loc_os01g51020 12001.m11283 protein 60S ribosomal protein L28, putative, expressed  no original description 	T
31.2.5	cell.division.plastid	MDP0000638127	 moderately similar to ( 247) AT5G53280 | Symbols: PDV1 | PDV1 (PLASTID DIVISION1) | chr5:21607586-21609080 FORWARDmoderately similar to ( 203) loc_os02g16450 12002.m06888 protein expressed protein  no original description 	T
29.2.1.2.2.30	protein.synthesis.ribosomal protein.eukaryotic.60S subunit.L30	MDP0000329640	 weakly similar to ( 197) AT1G77940 | Symbols:  | 60S ribosomal protein L30 (RPL30B) | chr1:29304116-29305288 REVERSEmoderately similar to ( 205) RL30_LUPLU 60S ribosomal protein L30 - Lupinus luteus (European yellow lupin)weakly similar to ( 180) loc_os01g16890 12001.m42968 protein 60S ribosomal protein L30, putative, expressed no original description 	T
29.3.4.99	protein.targeting.secretory pathway.unspecified	MDP0000152199	 highly similar to ( 592) AT2G21520 | Symbols:  | transporter | chr2:9215956-9218953 FORWARDhighly similar to ( 568) loc_os02g04020 12002.m05751 protein phosphatidylinositol transfer-like protein III, putative, expressed  no original description 	T
35.2	not assigned.unknown	MDP0000921005	 moderately similar to ( 342) AT1G32520 | Symbols:  | unknown protein | chr1:11758422-11759272 REVERSEweakly similar to ( 187) loc_os11g11330 12011.m05326 protein retrotransposon protein, putative, unclassified, expressed  no original description 	T
4.1.8	glycolysis.cytosolic branch.glyceraldehyde 3-phosphate dehydrogenase (GAP-DH)	MDP0000481557	 moderately similar to ( 208) AT1G13440 | Symbols: GAPC-2, GAPC2 | GAPC2 (GLYCERALDEHYDE-3-PHOSPHATE DEHYDROGENASE C2); NAD or NADH binding / binding / catalytic/ glyceraldehyde-3-phosphate dehydrogenase (phosphorylating)/ glyceraldehyde-3-phosphate dehydrogenase | chr1:4608465-4610494 REVERSEmoderately similar to ( 214) G3PC_ANTMA Glyceraldehyde-3-phosphate dehydrogenase, cytosolic (EC 1.2.1.12) - Antirrhinum majus (Garden snapdragon)moderately similar to ( 203) loc_os04g40950 12004.m35161 protein glyceraldehyde-3-phosphate dehydrogenase, cytosolic 3, putative, expressed  no original description 	T
29.3.4.3	protein.targeting.secretory pathway.vacuole	MDP0000248773	 highly similar to ( 769) AT4G32940 | Symbols: GAMMA-VPE, GAMMAVPE | GAMMA-VPE (GAMMA VACUOLAR PROCESSING ENZYME); cysteine-type endopeptidase | chr4:15900557-15903161 REVERSEhighly similar to ( 821) VPE_CITSI Vacuolar-processing enzyme precursor (EC 3.4.22.-) (VPE) - Citrus sinensis (Sweet orange)highly similar to ( 710) loc_os01g37910 12001.m42664 protein vacuolar processing enzyme precursor, putative, expressed   no original description 	T
33.99	development.unspecified	MDP0000239973	 moderately similar to ( 248) AT2G18250 | Symbols: ATCOAD | ATCOAD (4-phosphopantetheine adenylyltransferase); nucleotidyltransferase/ pantetheine-phosphate adenylyltransferase | chr2:7940025-7941499 FORWARDmoderately similar to ( 207) loc_os07g08210 12007.m29083 protein bifunctional coenzyme A synthase, putative, expressed no original description 	T
20.2.3	stress.abiotic.drought/salt	MDP0000239973	 moderately similar to ( 248) AT2G18250 | Symbols: ATCOAD | ATCOAD (4-phosphopantetheine adenylyltransferase); nucleotidyltransferase/ pantetheine-phosphate adenylyltransferase | chr2:7940025-7941499 FORWARDmoderately similar to ( 207) loc_os07g08210 12007.m29083 protein bifunctional coenzyme A synthase, putative, expressed no original description 	T
18.4.8	Co-factor and vitamine metabolism.pantothenate.pantetheine-phosphate adenylyltransferase  (PPAT)	MDP0000239973	 moderately similar to ( 248) AT2G18250 | Symbols: ATCOAD | ATCOAD (4-phosphopantetheine adenylyltransferase); nucleotidyltransferase/ pantetheine-phosphate adenylyltransferase | chr2:7940025-7941499 FORWARDmoderately similar to ( 207) loc_os07g08210 12007.m29083 protein bifunctional coenzyme A synthase, putative, expressed no original description 	T
35.1.19	not assigned.no ontology.C2 domain-containing protein	MDP0000242522	 weakly similar to ( 157) AT1G63220 | Symbols:  | C2 domain-containing protein | chr1:23449017-23450244 FORWARDweakly similar to ( 157) ERG3_ORYSA Elicitor-responsive protein 3 (16 kDa phloem protein) (RPP16) - Oryza sativa (Rice)weakly similar to ( 172) loc_os04g58570 12004.m10739 protein elicitor-responsive protein 3, putative, expressed  no original description 	T
35.1	not assigned.no ontology	MDP0000133570	 highly similar to ( 504) AT5G57100 | Symbols:  | transporter-related | chr5:23106563-23108440 REVERSEvery weakly similar to (51.2) TPT_MAIZE Triose phosphate/phosphate translocator, chloroplast precursor (cTPT) - Zea mays (Maize)moderately similar to ( 275) loc_os02g49260 12002.m09960 protein expressed protein no original description 	T
35.1	not assigned.no ontology	MDP0000605825	 moderately similar to ( 318) AT2G02880 | Symbols:  | mucin-related | chr2:841303-843151 FORWARDmoderately similar to ( 294) loc_os03g26220 12003.m07962 protein expressed protein  no original description 	T
26.16	misc.myrosinases-lectin-jacalin	MDP0000296584	 moderately similar to ( 308) AT2G16050 | Symbols:  | FUNCTIONS IN: molecular_function unknown; INVOLVED IN: biological_process unknown; LOCATED IN: cellular_component unknown; CONTAINS InterPro DOMAIN/s: DC1 (InterPro:IPR004146); BEST Arabidopsis thaliana protein match is: DC1 domain-containing protein (TAIR:AT1G20990.1); Has 719 Blast hits to 448 proteins in 12 species: Archae - 0; Bacteria - 0; Metazoa - 0; Fungi - 2; Plants - 717; Viruses - 0; Other Eukaryotes - 0 (source: NCBI BLink). | chr2:6980450-6981939 FORWARDvery weakly similar to (65.9) PHAL_PHAVU Leucoagglutinating phytohemagglutinin precursor (PHA-L) - Phaseolus vulgaris (Kidney bean) (French bean)moderately similar to ( 223) loc_os08g40280 12008.m08008 protein lectin-like protein kinase, putative, expressed  no original description 	T
26.27	misc.calcineurin-like phosphoesterase family protein	MDP0000229894	 moderately similar to ( 347) AT3G47810 | Symbols: MAG1 | MAG1 (MAIGO 1); hydrolase/ protein serine/threonine phosphatase | chr3:17637256-17639017 REVERSEmoderately similar to ( 366) loc_os02g16550 12002.m06898 protein vacuolar protein sorting 29, putative, expressed no original description 	T
29.5.11.1	protein.degradation.ubiquitin.ubiquitin	MDP0000361173	 moderately similar to ( 255) AT3G52590 | Symbols: UBQ1, EMB2167, ERD16, HAP4 | UBQ1 (UBIQUITIN EXTENSION PROTEIN 1); protein binding / structural constituent of ribosome | chr3:19505668-19506681 FORWARDweakly similar to ( 150) UBIQ_WHEAT Ubiquitin - Triticum aestivum (Wheat)moderately similar to ( 255) loc_os09g39500 12009.m60242 protein ubiquitin fusion protein, putative, expressed no original description 	T
27.3.99	RNA.regulation of transcription.unclassified	MDP0000276670	 moderately similar to ( 252) AT3G51390 | Symbols:  | zinc finger (DHHC type) family protein | chr3:19075718-19077966 FORWARDweakly similar to ( 192) loc_os12g29560 12012.m06770 protein metal ion binding protein, putative, expressed no original description 	T
29.4	protein.postranslational modification	MDP0000139452	 highly similar to ( 610) AT4G38520 | Symbols:  | protein phosphatase 2C family protein / PP2C family protein | chr4:18015999-18017514 REVERSEhighly similar to ( 622) loc_os06g50380 12006.m31935 protein protein phosphatase 2C, putative, expressed  no original description 	T
31.4	cell.vesicle transport	MDP0000845007	 nearly identical (1602) AT1G52360 | Symbols:  | coatomer protein complex, subunit beta 2 (beta prime), putative | chr1:19499282-19505397 FORWARDvery weakly similar to (93.2) PF20_CHLRE Flagellar WD repeat protein PF20 - Chlamydomonas reinhardtiinearly identical (1568) loc_os06g05180 12006.m71402 protein coatomer subunit beta, putative, expressed   no original description 	T
33.99	development.unspecified	MDP0000845007	 nearly identical (1602) AT1G52360 | Symbols:  | coatomer protein complex, subunit beta 2 (beta prime), putative | chr1:19499282-19505397 FORWARDvery weakly similar to (93.2) PF20_CHLRE Flagellar WD repeat protein PF20 - Chlamydomonas reinhardtiinearly identical (1568) loc_os06g05180 12006.m71402 protein coatomer subunit beta, putative, expressed   no original description 	T
29.5.1	protein.degradation.subtilases	MDP0000684605	 nearly identical (1095) AT5G51750 | Symbols: ATSBT1.3, SBT1.3 | ATSBT1.3 (ARABIDOPSIS THALIANA SUBTILASE 1.3); identical protein binding / serine-type endopeptidase | chr5:21020266-21022608 FORWARDnearly identical (1010) loc_os04g48416 12004.m101731 protein subtilisin-like protease precursor, putative, expressed   no original description 	T
16.7	secondary metabolism.wax	MDP0000516853	 highly similar to ( 634) AT1G02205 | Symbols: CER1 | CER1 (ECERIFERUM 1); octadecanal decarbonylase | chr1:418818-422154 FORWARDhighly similar to ( 578) loc_os10g33250 12010.m21915 protein CER1, putative, expressed no original description 	T
24.2	Biodegradation of Xenobiotics.lactoylglutathione lyase	MDP0000238551	 weakly similar to ( 166) AT1G15380 | Symbols:  | lactoylglutathione lyase family protein / glyoxalase I family protein | chr1:5290955-5292287 FORWARDweakly similar to ( 162) loc_os01g07850 12001.m07412 protein lactoylglutathione lyase, putative  no original description 	T
31.1	cell.organisation	MDP0000293822	 moderately similar to ( 308) AT3G18670 | Symbols:  | ankyrin repeat family protein | chr3:6424135-6426471 REVERSE   no original description 	T
10.2	cell wall.cellulose synthesis	MDP0000263736	 highly similar to ( 899) AT5G22740 | Symbols: ATCSLA02, CSLA02, ATCSLA2 | ATCSLA02; mannan synthase/ transferase, transferring glycosyl groups | chr5:7555379-7559866 REVERSEhighly similar to ( 769) loc_os06g42020 12006.m08734 protein CSLA9 - cellulose synthase-like family A, expressed   no original description 	T
35.2	not assigned.unknown	MDP0000782890	 highly similar to ( 595) AT3G14920 | Symbols:  | unknown protein | chr3:5018275-5020104 FORWARDhighly similar to ( 527) loc_os01g10930 12001.m07713 protein peptide-N4-asparagine amidase A, putative, expressed MSP1_C  no original description 	T
15.2	metal handling.binding, chelation and storage	MDP0000888788	 weakly similar to ( 125) AT1G23000 | Symbols:  | heavy-metal-associated domain-containing protein | chr1:8143614-8144865 FORWARDweakly similar to ( 123) loc_os01g03490 12001.m06987 protein heavy metal-associated domain containing protein, expressed no original description 	T
35.1.5	not assigned.no ontology.pentatricopeptide (PPR) repeat-containing protein	MDP0000321498	 highly similar to ( 513) AT3G53360 | Symbols:  | pentatricopeptide (PPR) repeat-containing protein | chr3:19784502-19786808 FORWARDvery weakly similar to (77.0) RF1_ORYSA Rf1 protein, mitochondrial precursor (PPR protein) (Fertility restorer) (Restorer for CMS) - Oryza sativa (Rice)moderately similar to ( 432) loc_os06g30940 12006.m07636 protein pentatricopeptide repeat protein PPR986-12, putative, expressed   no original description 	T
29.3.1	protein.targeting.nucleus	MDP0000224825	 moderately similar to ( 353) AT5G60980 | Symbols:  | nuclear transport factor 2 (NTF2) family protein / RNA recognition motif (RRM)-containing protein | chr5:24543988-24546027 FORWARDvery weakly similar to (57.8) NTF2_ORYSA Nuclear transport factor 2 (NTF-2) - Oryza sativa (Rice)moderately similar to ( 300) loc_os04g30430 12004.m08148 protein RNA-binding protein-like, putative, expressed  no original description 	T
29.6	protein.folding	MDP0000246192	 moderately similar to ( 240) AT1G20810 | Symbols:  | immunophilin / FKBP-type peptidyl-prolyl cis-trans isomerase family protein | chr1:7232025-7233367 FORWARDmoderately similar to ( 235) loc_os02g02550 12002.m05604 protein peptidyl-prolyl cis-trans isomerase, FKBP-type family protein, expressedMSP1_C  no original description 	T
27.3.71	RNA.regulation of transcription.SNF7	MDP0000269136	 moderately similar to ( 243) AT5G09260 | Symbols: VPS20.2 | VPS20.2 (VACUOLAR PROTEIN SORTING-ASSOCIATED PROTEIN 20.2) | chr5:2876797-2878355 FORWARDmoderately similar to ( 214) loc_os02g38300 12002.m100264 protein charged multivesicular body protein 6, putative, expressed no original description 	T
30.3	signalling.calcium	MDP0000928028	 moderately similar to ( 384) AT4G05520 | Symbols: ATEHD2 | ATEHD2 (EPS15 HOMOLOGY DOMAIN 2); GTP binding / GTPase/ calcium ion binding | chr4:2804522-2807833 FORWARDmoderately similar to ( 391) loc_os06g47330 12006.m32161 protein EH-domain-containing protein 1, putative, expressed  no original description 	T
35.2	not assigned.unknown	MDP0000265633	 moderately similar to ( 327) AT3G13230 | Symbols:  | RNA binding | chr3:4270423-4271070 FORWARDmoderately similar to ( 326) loc_os03g02420 12003.m05784 protein partner of Nob1, putative, expressed no original description 	T
23.5.4	nucleotide metabolism.deoxynucleotide metabolism.ribonucleoside-diphosphate reductase	MDP0000014856	 nearly identical (1413) AT2G21790 | Symbols: R1, RNR1, CLS8, ATRNR1 | RNR1 (RIBONUCLEOTIDE REDUCTASE 1); ATP binding / protein binding / ribonucleoside-diphosphate reductase | chr2:9293529-9297580 FORWARDnearly identical (1405) loc_os06g07210 12006.m31786 protein ribonucleoside-diphosphate reductase large subunit, putative, expressed  no original description 	T
27.1.1	RNA.processing.splicing	MDP0000641578	 weakly similar to ( 165) AT5G06160 | Symbols: ATO | ATO (ATROPOS); nucleic acid binding / zinc ion binding | chr5:1862623-1866298 REVERSEweakly similar to ( 151) loc_os03g50850 12003.m35395 protein splicing factor 3A subunit 3, putative, expressed  no original description 	T
35.2	not assigned.unknown	MDP0000144167	  no original description 	T
35.2	not assigned.unknown	MDP0000234735	 moderately similar to ( 261) AT2G44870 | Symbols:  | unknown protein | chr2:18503250-18504422 FORWARDmoderately similar to ( 221) loc_os10g41940 12010.m06951 protein expressed protein  no original description 	T
27.3.22	RNA.regulation of transcription.HB,Homeobox transcription factor family	MDP0000899816	 weakly similar to ( 135) AT3G61890 | Symbols: ATHB-12, ATHB12 | ATHB-12 (ARABIDOPSIS THALIANA HOMEOBOX 12); transcription activator/ transcription factor | chr3:22914346-22915239 REVERSEweakly similar to ( 123) loc_os09g35910 12009.m22175 protein homeodomain-leucine zipper transcription factor TaHDZipI-1, putative, expressed no original description 	T
27.3.40	RNA.regulation of transcription.Aux/IAA family	MDP0000174664	 moderately similar to ( 280) AT4G29080 | Symbols: PAP2, IAA27 | PAP2 (PHYTOCHROME-ASSOCIATED PROTEIN 2); transcription factor | chr4:14323665-14325213 REVERSEmoderately similar to ( 232) IAA21_ORYSA Auxin-responsive protein IAA21 (Indoleacetic acid-induced protein 21) - Oryza sativa (Rice)moderately similar to ( 234) loc_os06g06730 12006.m05398 protein S-adenosylmethionine-dependent methyltransferase, putative, expressed    no original description 	T
29.4	protein.postranslational modification	MDP0000643284	 moderately similar to ( 265) AT4G25130 | Symbols:  | peptide methionine sulfoxide reductase, putative | chr4:12898802-12899998 REVERSEmoderately similar to ( 343) MSRA_FRAAN Peptide methionine sulfoxide reductase (EC 1.8.4.11) (Protein-methionine-S-oxide reductase) (Peptide-methionine (S)-S-oxide reductase) (Peptide Met(O) reductase) (Fruit-ripening protein E4) - Fragaria ananassa (Strawberry)moderately similar to ( 256) loc_os10g41400 12010.m22009 protein peptide methionine sulfoxide reductase, putative, expressed no original description 	T
27.3.40	RNA.regulation of transcription.Aux/IAA family	MDP0000213864	 moderately similar to ( 300) AT3G23050 | Symbols: IAA7, AXR2 | IAA7 (INDOLE-3-ACETIC ACID 7); transcription factor | chr3:8194768-8196716 FORWARDmoderately similar to ( 258) AUX28_SOYBN Auxin-induced protein AUX28 - Glycine max (Soybean)moderately similar to ( 251) loc_os12g40890 12012.m07872 protein OsIAA30 - Auxin-responsive Aux/IAA gene family member, expressed  no original description 	T
33.99	development.unspecified	MDP0000166306	 weakly similar to ( 138) AT3G47650 | Symbols:  | bundle-sheath defective protein 2 family / bsd2 family | chr3:17569576-17570262 FORWARDweakly similar to ( 144) loc_os06g22690 12006.m32073 protein bundle sheath defective protein 2, putative, expressed no original description 	T
30.7	signalling.14-3-3 proteins	MDP0000146698	 moderately similar to ( 456) AT5G38480 | Symbols: GRF3, RCI1 | GRF3 (GENERAL REGULATORY FACTOR 3); ATP binding / protein binding / protein phosphorylated amino acid binding | chr5:15410277-15411285 FORWARDmoderately similar to ( 468) 1433A_SOYBN 14-3-3-like protein A (SGF14A) - Glycine max (Soybean)moderately similar to ( 457) loc_os08g33370 12008.m26501 protein 14-3-3-like protein, putative, expressed   no original description 	T
26.7	misc.oxidases - copper, flavone etc	MDP0000299946	 highly similar to ( 512) AT5G16990 | Symbols:  | NADP-dependent oxidoreductase, putative | chr5:5581831-5583849 REVERSEmoderately similar to ( 450) loc_os12g12580 12012.m05231 protein NADP-dependent oxidoreductase P2, putative, expressed no original description 	T
31.1	cell.organisation	MDP0000859326	 nearly identical (1450) AT3G45850 | Symbols:  | kinesin motor protein-related | chr3:16855814-16860950 REVERSEhighly similar to ( 767) K125_TOBAC 125 kDa kinesin-related protein - Nicotiana tabacum (Common tobacco)nearly identical (1278) loc_os05g02670 12005.m083602 protein kinesin-related protein, putative, expressed  no original description 	T
29.5.11.4.2	protein.degradation.ubiquitin.E3.RING	MDP0000497565	 weakly similar to ( 127) AT4G14220 | Symbols: RHF1A | RHF1A (RING-H2 GROUP F1A); protein binding / ubiquitin-protein ligase/ zinc ion binding | chr4:8196012-8198240 FORWARDvery weakly similar to (87.0) loc_os11g36430 12011.m28683 protein zinc finger, RING-type, putative, expressed  no original description 	T
17.2.2	hormone metabolism.auxin.signal transduction	MDP0000379186	 moderately similar to ( 288) AT5G16530 | Symbols: PIN5 | PIN5 (PIN-FORMED 5); auxin:hydrogen symporter/ transporter | chr5:5400735-5402626 FORWARDmoderately similar to ( 242) PIN6_ORYSA Probable auxin efflux carrier component 6 (OsPIN6) - Oryza sativa (Rice)moderately similar to ( 242) loc_os01g69070 12001.m13008 protein auxin efflux carrier component 6, putative, expressed   no original description 	T
16.8.2	secondary metabolism.flavonoids.chalcones	MDP0000686666	 highly similar to ( 644) AT5G13930 | Symbols: CHS, TT4, ATCHS | TT4 (TRANSPARENT TESTA 4); naringenin-chalcone synthase | chr5:4488762-4490035 FORWARDhighly similar to ( 691) CHSA_PETHY Chalcone synthase A (EC 2.3.1.74) (Naringenin-chalcone synthase A) - Petunia hybrida (Petunia)highly similar to ( 645) loc_os11g32650 12011.m07166 protein chalcone synthase, putative, expressed   no original description 	T
35.1	not assigned.no ontology	MDP0000231048	 weakly similar to ( 120) AT5G58000 | Symbols:  | LOCATED IN: endoplasmic reticulum, chloroplast; CONTAINS InterPro DOMAIN/s: Reticulon (InterPro:IPR003388); BEST Arabidopsis thaliana protein match is: reticulon family protein (TAIR:AT4G28430.1); Has 1047 Blast hits to 1034 proteins in 125 species: Archae - 0; Bacteria - 2; Metazoa - 715; Fungi - 46; Plants - 58; Viruses - 4; Other Eukaryotes - 222 (source: NCBI BLink). | chr5:23477416-23479496 FORWARDvery weakly similar to (98.6) loc_os08g17870 12008.m05906 protein expressed protein no original description 	T
31.3	cell.cycle	MDP0000252048	 very weakly similar to (65.5) AT3G19150 | Symbols: KRP6, ICK4 | KRP6 (KIP-RELATED PROTEIN 6); cyclin binding / cyclin-dependent protein kinase inhibitor | chr3:6616930-6617655 REVERSEvery weakly similar to (54.7) loc_os03g04490 12003.m05977 protein cyclin dependent kinase inhibitor, putative, expressed   no original description 	T
17.5.1	hormone metabolism.ethylene.synthesis-degradation	MDP0000208098	 weakly similar to ( 171) AT3G21420 | Symbols:  | oxidoreductase, 2OG-Fe(II) oxygenase family protein | chr3:7541579-7543221 FORWARDweakly similar to ( 137) FLS_MALDO Flavonol synthase/flavanone 3-hydroxylase (EC 1.14.11.23) (EC 1.14.11.9) (FLS) - Malus domestica (Apple) (Malus sylvestris)weakly similar to ( 148) loc_os10g40934 12010.m06860 protein flavonol synthase/flavanone 3-hydroxylase, putative, expressed   no original description 	T
2.2.2.2	major CHO metabolism.degradation.starch.starch phosphorylase	MDP0000123354	 nearly identical (1363) AT3G46970 | Symbols: ATPHS2, PHS2 | PHS2 (ALPHA-GLUCAN PHOSPHORYLASE 2); phosphorylase/ transferase, transferring glycosyl groups | chr3:17301625-17306111 REVERSEnearly identical (1381) PHSH_SOLTU Alpha-glucan phosphorylase, H isozyme (EC 2.4.1.1) (Starch phosphorylase H) - Solanum tuberosum (Potato)nearly identical (1324) loc_os01g63270 12001.m43279 protein alpha-glucan phosphorylase, H isozyme, putative, expressed  no original description 	T
35.1	not assigned.no ontology	MDP0000380331	 moderately similar to ( 470) AT4G12230 | Symbols:  | esterase/lipase/thioesterase family protein | chr4:7284645-7287348 FORWARDmoderately similar to ( 438) loc_os10g08840 12010.m21836 protein maspardin, putative, expressed  no original description 	T
35.2	not assigned.unknown	MDP0000076511	 highly similar to ( 587) AT3G22290 | Symbols:  | unknown protein | chr3:7882082-7885260 FORWARDhighly similar to ( 531) loc_os03g57100 12003.m10630 protein serologically defined breast cancer antigen NY-BR-84, putative, expressed   no original description 	T
16.2	secondary metabolism.phenylpropanoids	MDP0000269632	 weakly similar to ( 188) AT4G35160 | Symbols:  | O-methyltransferase family 2 protein | chr4:16730989-16732808 REVERSEmoderately similar to ( 265) 7OMT9_MEDSA Isoflavone-7-O-methyltransferase 9 (EC 2.1.1.150) (Isoflavone-O-methyltransferase 9) (7 IOMT-9) - Medicago sativa (Alfalfa)moderately similar to ( 243) loc_os08g35310 12008.m07520 protein isoflavone-7-O-methytransferase 9, putative   no original description 	T
29.5.3	protein.degradation.cysteine protease	MDP0000232426	 highly similar to ( 511) AT1G02305 | Symbols:  | cathepsin B-like cysteine protease, putative | chr1:455816-457974 FORWARDweakly similar to ( 124) ALEU_HORVU Thiol protease aleurain precursor (EC 3.4.22.16) - Hordeum vulgare (Barley)highly similar to ( 508) loc_os05g24550 12005.m083685 protein cathepsin B-like cysteine proteinase 3 precursor, putative, expressed  no original description 	T
30.1	signalling.in sugar and nutrient physiology	MDP0000807958	 highly similar to ( 521) AT5G51550 | Symbols: EXL3 | EXL3 (EXORDIUM LIKE 3) | chr5:20939793-20940806 REVERSEmoderately similar to ( 414) loc_os06g04250 12006.m05154 protein phi-1-like phosphate-induced protein, putative, expressed   no original description 	T
21.2.2	redox.ascorbate and glutathione.glutathione	MDP0000291593	 moderately similar to ( 253) AT2G31570 | Symbols: ATGPX2 | ATGPX2 (GLUTATHIONE PEROXIDASE 2); glutathione peroxidase | chr2:13438211-13439775 REVERSEmoderately similar to ( 249) GPX4_CITSI Probable phospholipid hydroperoxide glutathione peroxidase (EC 1.11.1.12) (PHGPx) (Salt-associated protein) - Citrus sinensis (Sweet orange)moderately similar to ( 263) loc_os11g18170 12011.m05893 protein glutathione peroxidase 3, mitochondrial precursor, putative, expressed  no original description 	T
27.3.8	RNA.regulation of transcription.C2C2(Zn) DOF zinc finger family	MDP0000156241	 weakly similar to ( 134) AT1G28310 | Symbols:  | Dof-type zinc finger domain-containing protein | chr1:9912534-9913685 REVERSEweakly similar to ( 124) PBF_MAIZE Dof zinc finger protein PBF (Prolamin box-binding factor) - Zea mays (Maize)weakly similar to ( 129) loc_os03g60630 12003.m10953 protein expressed protein  no original description 	T
26.22	misc.short chain dehydrogenase/reductase (SDR)	MDP0000180845	 moderately similar to ( 363) AT3G55290 | Symbols:  | short-chain dehydrogenase/reductase (SDR) family protein | chr3:20502653-20503730 FORWARDweakly similar to ( 108) FABG3_BRANA 3-oxoacyl-[acyl-carrier-protein] reductase 3, chloroplast precursor (EC 1.1.1.100) (3-ketoacyl-acyl carrier protein reductase 3) - Brassica napus (Rape)moderately similar to ( 342) loc_os08g39960 12008.m26742 protein 3-oxoacyl-reductase, chloroplast precursor, putative, expressed  no original description 	T
27.3.54	RNA.regulation of transcription.Histone acetyltransferases	MDP0000211193	 highly similar to ( 534) AT5G56740 | Symbols: HAG2, HAC7, HAG02, HAC07 | HAG2 (HISTONE ACETYLTRANSFERASE OF THE GNAT FAMILY 2); H4 histone acetyltransferase/ histone acetyltransferase | chr5:22953009-22955577 REVERSEmoderately similar to ( 402) HAT1_ORYSA Probable acetyltransferase type B catalytic subunit (EC 2.3.1.48) (HAT B) - Oryza sativa (Rice)moderately similar to ( 402) loc_os09g17850 12009.m05043 protein acetyltransferase type B catalytic subunit, putative, expressed  no original description 	T
26.2	misc.UDP glucosyl and glucoronyl transferases	MDP0000292999	 moderately similar to ( 478) AT3G55830 | Symbols: EPC1 | EPC1 (ECTOPICALLY PARTING CELLS); UDP-glucosyltransferase/ transferase, transferring glycosyl groups | chr3:20715101-20717133 FORWARDmoderately similar to ( 353) loc_os06g49150 12006.m09439 protein exostosin-3, putative, expressed  no original description 	T
29.2.1.2.1.30	protein.synthesis.ribosomal protein.eukaryotic.40S subunit.S30	MDP0000319661	 weakly similar to ( 103) AT5G56670 | Symbols:  | 40S ribosomal protein S30 (RPS30C) | chr5:22935413-22935981 REVERSEweakly similar to ( 104) loc_os06g07580 12006.m05481 protein expressed protein no original description 	T
1.1.1.2	PS.lightreaction.photosystem II.PSII polypeptide subunits	MDP0000874065	 moderately similar to ( 263) AT2G39470 | Symbols: PPL2 | PPL2 (PsbP-like protein 2); calcium ion binding | chr2:16476335-16477402 FORWARDmoderately similar to ( 241) loc_os03g17174 12003.m101683 protein calcium ion binding protein, putative, expressed no original description 	T
35.2	not assigned.unknown	MDP0000308794	 nearly identical (1813) AT5G54440 | Symbols:  | unknown protein | chr5:22100056-22107695 FORWARDnearly identical (1658) loc_os12g36760 12012.m07461 protein expressed protein    no original description 	T
35.1	not assigned.no ontology	MDP0000317067	 moderately similar to ( 352) AT4G36850 | Symbols:  | INVOLVED IN: biological_process unknown; LOCATED IN: membrane; CONTAINS InterPro DOMAIN/s: Cystinosin/ERS1p repeat (InterPro:IPR006603); BEST Arabidopsis thaliana protein match is: PQ-loop repeat family protein / transmembrane family protein (TAIR:AT2G41050.1); Has 599 Blast hits to 441 proteins in 106 species: Archae - 0; Bacteria - 0; Metazoa - 197; Fungi - 311; Plants - 38; Viruses - 0; Other Eukaryotes - 53 (source: NCBI BLink). | chr4:17353415-17355822 REVERSEweakly similar to ( 167) loc_os01g16170 12001.m42960 protein PQ loop repeat family protein, expressed no original description 	T
20.1.7	stress.biotic.PR-proteins	MDP0000901201	 moderately similar to ( 208) AT4G12010 | Symbols:  | disease resistance protein (TIR-NBS-LRR class), putative | chr4:7197325-7201393 REVERSEvery weakly similar to (97.8) loc_os01g15580 12001.m08156 protein disease resistance protein RGA2, putative, expressed  no original description 	T
16.7	secondary metabolism.wax	MDP0000069348	 highly similar to ( 776) AT1G02205 | Symbols: CER1 | CER1 (ECERIFERUM 1); octadecanal decarbonylase | chr1:418818-422154 FORWARDhighly similar to ( 717) loc_os10g33250 12010.m21915 protein CER1, putative, expressed  no original description 	T
27.3.2	RNA.regulation of transcription.Alfin-like	MDP0000625291	 moderately similar to ( 294) AT5G26210 | Symbols: AL4 | AL4 (ALFIN-LIKE 4); DNA binding / methylated histone residue binding | chr5:9158566-9160221 REVERSEmoderately similar to ( 271) loc_os01g66420 12001.m42781 protein PHD finger protein, putative, expressed no original description 	T
13.1.6.2.1	amino acid metabolism.synthesis.aromatic aa.phenylalanine and tyrosine.chorismate mutase	MDP0000560179	 moderately similar to ( 404) AT1G69370 | Symbols: CM3, cm-3 | CM3 (chorismate mutase 3); chorismate mutase | chr1:26080098-26081559 FORWARDmoderately similar to ( 385) loc_os01g55870 12001.m11743 protein chorismate mutase, chloroplast precursor, putative, expressed MSP1_C  no original description 	T
17.3.1.2.6	hormone metabolism.brassinosteroid.synthesis-degradation.sterols.DWF7	MDP0000549234	 moderately similar to ( 295) AT3G02580 | Symbols: STE1, DWF7, BUL1 | STE1 (STEROL 1); C-5 sterol desaturase | chr3:547048-548615 FORWARDmoderately similar to ( 314) SC5D_TOBAC Delta(7)-sterol-C5(6)-desaturase (EC 1.3.3.-) (Delta-7-C-5 sterol desaturase) (Delta(7)-sterol-C5-desaturase) - Nicotiana tabacum (Common tobacco)moderately similar to ( 265) loc_os01g04260 12001.m07061 protein delta-7-sterol-C5, putative, expressed  no original description 	T
34.99	transport.misc	MDP0000304285	 highly similar to ( 714) AT2G26510 | Symbols: PDE135 | PDE135 (pigment defective embryo 135); transmembrane transporter | chr2:11274118-11277464 FORWARDhighly similar to ( 642) loc_os12g39420 12012.m073719 protein solute carrier family 23 member 2, putative, expressed  no original description 	T
20.2.1	stress.abiotic.heat	MDP0000235350	 weakly similar to ( 140) AT4G36040 | Symbols:  | DNAJ heat shock N-terminal domain-containing protein (J11) | chr4:17049708-17050193 REVERSEvery weakly similar to (51.6) DNJH_CUCSA DnaJ protein homolog (DNAJ-1) - Cucumis sativus (Cucumber)very weakly similar to (95.5) loc_os08g43490 12008.m08323 protein chaperone protein dnaJ 11, chloroplast precursor, putative, expressed  no original description 	T
33.99	development.unspecified	MDP0000301813	 weakly similar to ( 155) AT3G48140 | Symbols:  | senescence-associated protein, putative | chr3:17778471-17779299 FORWARDweakly similar to ( 137) loc_os06g13680 12006.m06083 protein B12D protein, expressed    no original description 	T
16.4.1	secondary metabolism.N misc.alkaloid-like	MDP0000230954	 moderately similar to ( 287) AT3G51430 | Symbols: YLS2 | YLS2; strictosidine synthase | chr3:19086548-19087909 FORWARDvery weakly similar to (80.9) STSY_CATRO Strictosidine synthase precursor (EC 4.3.3.2) - Catharanthus roseus (Rosy periwinkle) (Madagascar periwinkle)moderately similar to ( 291) loc_os10g39710 12010.m21960 protein strictosidine synthase, putative, expressed  no original description 	T
26.10	misc.cytochrome P450	MDP0000398616	 highly similar to ( 564) AT1G64950 | Symbols: CYP89A5 | CYP89A5; electron carrier/ heme binding / iron ion binding / monooxygenase/ oxygen binding | chr1:24127587-24129119 FORWARDmoderately similar to ( 321) C77A3_SOYBN Cytochrome P450 77A3 (EC 1.14.-.-) - Glycine max (Soybean)moderately similar to ( 450) loc_os10g37110 12010.m06511 protein transposon protein, putative, unclassified, expressed    Sporozoite_P67  no original description 	T
35.2	not assigned.unknown	MDP0000819899	 moderately similar to ( 368) AT1G61150 | Symbols:  | FUNCTIONS IN: molecular_function unknown; INVOLVED IN: biological_process unknown; LOCATED IN: cellular_component unknown; EXPRESSED IN: 23 plant structures; EXPRESSED DURING: 13 growth stages; CONTAINS InterPro DOMAIN/s: CTLH, C-terminal to LisH motif (InterPro:IPR006595), CT11-RanBPM (InterPro:IPR013144); BEST Arabidopsis thaliana protein match is: unknown protein (TAIR:AT4G09300.1); Has 486 Blast hits to 476 proteins in 122 species: Archae - 0; Bacteria - 4; Metazoa - 235; Fungi - 76; Plants - 132; Viruses - 0; Other Eukaryotes - 39 (source: NCBI BLink). | chr1:22543304-22544355 FORWARDmoderately similar to ( 363) loc_os03g13220 12003.m06768 protein pg4, putative, expressed no original description 	T
31.3.1	cell.cycle.peptidylprolyl isomerase	MDP0000251679	 highly similar to ( 777) AT4G38760 | Symbols:  | unknown protein | chr4:18091802-18096166 REVERSEmoderately similar to ( 242) CYPH_CATRO Peptidyl-prolyl cis-trans isomerase (EC 5.2.1.8) (PPIase) (Rotamase) (Cyclophilin) (Cyclosporin A-binding protein) - Catharanthus roseus (Rosy periwinkle) (Madagascar periwinkle)moderately similar to ( 346) loc_os10g06610 12010.m04056 protein expressed protein    no original description 	T
35.2	not assigned.unknown	MDP0000182482	 weakly similar to ( 125) AT2G17972 | Symbols:  | unknown protein | chr2:7821624-7822100 FORWARDweakly similar to ( 121) loc_os06g50130 12006.m09537 protein expressed protein    COG4907  no original description 	T
35.2	not assigned.unknown	MDP0000230253	 weakly similar to ( 131) AT3G17930 | Symbols:  | unknown protein | chr3:6142307-6143246 REVERSEweakly similar to ( 126) loc_os01g59080 12001.m12047 protein expressed protein no original description 	T
34.8	transport.metabolite transporters at the envelope membrane	MDP0000184567	 moderately similar to ( 462) AT3G01550 | Symbols: ATPPT2, PPT2 | PPT2 (PHOSPHOENOLPYRUVATE (PEP)/PHOSPHATE TRANSLOCATOR 2); antiporter/ triose-phosphate transmembrane transporter | chr3:216947-218856 REVERSEmoderately similar to ( 207) TPT_FLAPR Triose phosphate/phosphate translocator, chloroplast precursor (cTPT) - Flaveria pringleimoderately similar to ( 436) loc_os08g25624 12008.m26834 protein triose phosphate/phosphate translocator, non-green plastid,chloroplast precursor, putative, expressed no original description 	T
19.40	tetrapyrrole synthesis.regulation	MDP0000258572	 moderately similar to ( 304) AT3G14110 | Symbols: FLU | FLU (FLUORESCENT IN BLUE LIGHT); binding | chr3:4676222-4677602 REVERSEmoderately similar to ( 266) loc_os01g32730 12001.m43031 protein FLU, putative, expressed  no original description 	T
13.1.6.1.7	amino acid metabolism.synthesis.aromatic aa.chorismate.chorismate synthase	MDP0000244469	 highly similar to ( 532) AT1G48850 | Symbols: EMB1144 | EMB1144 (embryo defective 1144); chorismate synthase | chr1:18065399-18067956 REVERSEmoderately similar to ( 484) loc_os03g14990 12003.m06936 protein chorismate synthase 2, chloroplast precursor, putative, expressed   no original description 	T
1.3.13	PS.calvin cycle.rubisco interacting	MDP0000944409	 highly similar to ( 726) AT2G39730 | Symbols: RCA | RCA (RUBISCO ACTIVASE); ADP binding / ATP binding / enzyme regulator/ ribulose-1,5-bisphosphate carboxylase/oxygenase activator | chr2:16571174-16573345 REVERSEhighly similar to ( 734) RCA_ORYSA Ribulose bisphosphate carboxylase/oxygenase activase, chloroplast precursor (RuBisCO activase) (RA) - Oryza sativa (Rice)highly similar to ( 734) loc_os11g47970 12011.m28706 protein ribulose bisphosphate carboxylase/oxygenase activase, chloroplast precursor, putative, expressed  no original description 	T
29.2.3	protein.synthesis.initiation	MDP0000222178	 very weakly similar to (90.1) AT1G54270 | Symbols: EIF4A-2 | EIF4A-2; ATP-dependent helicase/ translation initiation factor | chr1:20260495-20262018 FORWARDweakly similar to ( 105) IF4A7_TOBAC Eukaryotic initiation factor 4A-7 (EC 3.6.1.-) (ATP-dependent RNA helicase eIF4A-7) (eIF-4A-7) - Nicotiana tabacum (Common tobacco)very weakly similar to (88.6) loc_os02g05330 12002.m05881 protein eukaryotic initiation factor 4A, putative, expressed  no original description 	T
35.2	not assigned.unknown	MDP0000704520	 moderately similar to ( 386) AT4G19950 | Symbols:  | unknown protein | chr4:10809977-10810942 FORWARDmoderately similar to ( 263) loc_os01g58620 12001.m12004 protein expressed protein      TrbL PRK08026 COG4625 DUF1750 Sporozoite_P67 COG2931  no original description 	T
13.1.3.5	amino acid metabolism.synthesis.aspartate family.lysine	MDP0000169985	 highly similar to ( 564) AT2G45440 | Symbols: DHDPS2 | DHDPS2 (DIHYDRODIPICOLINATE SYNTHASE); catalytic/ dihydrodipicolinate synthase/ lyase | chr2:18731294-18732867 FORWARDhighly similar to ( 555) DAPA_TOBAC Dihydrodipicolinate synthase, chloroplast precursor (EC 4.2.1.52) (DHDPS) - Nicotiana tabacum (Common tobacco)moderately similar to ( 487) loc_os04g18200 12004.m06985 protein dihydrodipicolinate synthase 2, chloroplast precursor, putative, expressed  no original description 	T
35.2	not assigned.unknown	MDP0000125925	  no original description 	T
29.4	protein.postranslational modification	MDP0000248817	 highly similar to ( 563) AT3G05580 | Symbols:  | serine/threonine protein phosphatase, putative | chr3:1618216-1619850 REVERSEhighly similar to ( 563) PP1_PHAVU Serine/threonine-protein phosphatase PP1 (EC 3.1.3.16) - Phaseolus vulgaris (Kidney bean) (French bean)moderately similar to ( 499) loc_os01g24750 12001.m08941 protein serine/threonine-protein phosphatase PP1, putative, expressed  no original description 	T
35.2	not assigned.unknown	MDP0000257598	 weakly similar to ( 163) AT1G68490 | Symbols:  | unknown protein | chr1:25693926-25694946 FORWARDvery weakly similar to (56.2) loc_os05g01210 12005.m28020 protein expressed protein   TrbL  no original description 	T
33.99	development.unspecified	MDP0000156516	 weakly similar to ( 150) AT5G45160 | Symbols:  | root hair defective 3 GTP-binding (RHD3) family protein | chr5:18264991-18270035 FORWARDweakly similar to ( 129) loc_os01g39310 12001.m43066 protein protein SEY1, putative, expressed no original description 	T
11.9.3.3	lipid metabolism.lipid degradation.lysophospholipases.glycerophosphodiester phosphodiesterase	MDP0000321216	 highly similar to ( 601) AT1G74210 | Symbols:  | glycerophosphoryl diester phosphodiesterase family protein | chr1:27910396-27912785 FORWARDhighly similar to ( 574) loc_os01g55780 12001.m11736 protein glycerophosphoryl diester phosphodiesterase precursor, putative, expressed no original description 	T
35.1	not assigned.no ontology	MDP0000164293	 moderately similar to ( 258) AT3G48690 | Symbols: ATCXE12, CXE12 | CXE12; carboxylesterase | chr3:18037186-18038160 REVERSEvery weakly similar to (96.7) GID1_ORYSA Gibberellin receptor GID1 (EC 3.-.-.-) (Gibberellin-insensitive dwarf protein 1) (Protein GIBBERELLIN INSENSITIVE DWARF1) - Oryza sativa (Rice)moderately similar to ( 229) loc_os09g28750 12009.m06026 protein gibberellin receptor GID1L2, putative, expressed  Sporozoite_P67 TrbL  no original description 	T
35.2	not assigned.unknown	MDP0000923094	     no original description 	T
29.2.1.2.1.2	protein.synthesis.ribosomal protein.eukaryotic.40S subunit.S2	MDP0000135672	 moderately similar to ( 403) AT3G57490 | Symbols:  | 40S ribosomal protein S2 (RPS2D) | chr3:21279824-21280887 REVERSEvery weakly similar to (75.9) RR5_SPIOL 30S ribosomal protein S5, chloroplast precursor - Spinacia oleracea (Spinach)moderately similar to ( 406) loc_os03g59310 12003.m10829 protein 40S ribosomal protein S2, putative, expressed  no original description 	T
29.5.11.4.3.3	protein.degradation.ubiquitin.E3.SCF.cullin	MDP0000822317	 moderately similar to ( 423) AT4G02570 | Symbols: ATCUL1, CUL1, AXR6 | ATCUL1 (ARABIDOPSIS THALIANA CULLIN 1); protein binding | chr4:1129315-1133435 FORWARDmoderately similar to ( 405) loc_os01g27150 12001.m42650 protein cullin-1, putative, expressed  no original description 	T
29.5.1	protein.degradation.subtilases	MDP0000201281	 highly similar to ( 910) AT5G59810 | Symbols: ATSBT5.4, SBT5.4 | SBT5.4; identical protein binding / serine-type endopeptidase | chr5:24096895-24100387 REVERSEhighly similar to ( 828) loc_os02g10520 12002.m06350 protein subtilisin-like protease precursor, putative, expressed  no original description 	T
35.1.5	not assigned.no ontology.pentatricopeptide (PPR) repeat-containing protein	MDP0000252130	 highly similar to ( 691) AT2G42580 | Symbols: TTL3, VIT | TTL3 (TETRATRICOPETIDE-REPEAT THIOREDOXIN-LIKE 3); binding / protein binding | chr2:17728855-17731461 FORWARDvery weakly similar to (71.6) STIP_SOYBN Heat shock protein STI (Stress-inducible protein) (GmSTI) - Glycine max (Soybean)highly similar to ( 573) loc_os01g11920 12001.m07808 protein retrotransposon protein, putative, unclassified, expressed   no original description 	T
30.2.11	signalling.receptor kinases.leucine rich repeat XI	MDP0000302881	 highly similar to ( 584) AT5G23400 | Symbols:  | disease resistance family protein / LRR family protein | chr5:7880603-7882372 FORWARDweakly similar to ( 167) RPK1_IPONI Receptor-like protein kinase precursor (EC 2.7.11.1) - Ipomoea nil (Japanese morning glory) (Pharbitis nil)moderately similar to ( 208) loc_os05g07820 12005.m05300 protein leucine-rich repeat receptor protein kinase EXS precursor, putative, expressed   no original description 	T
34.9	transport.metabolite transporters at the mitochondrial membrane	MDP0000230407	 highly similar to ( 528) AT4G01100 | Symbols: ADNT1 | ADNT1 (ADENINE NUCLEOTIDE TRANSPORTER 1); ADP transmembrane transporter/ AMP transmembrane transporter/ ATP transmembrane transporter/ binding | chr4:477411-479590 FORWARDweakly similar to ( 147) BT1_MAIZE Protein brittle-1, chloroplast precursor - Zea mays (Maize)highly similar to ( 519) loc_os05g50840 12005.m083826 protein Grave disease carrier protein, putative, expressed no original description 	T
4.1.8	glycolysis.cytosolic branch.glyceraldehyde 3-phosphate dehydrogenase (GAP-DH)	MDP0000165534	 highly similar to ( 560) AT1G16300 | Symbols: GAPCP-2 | GAPCP-2; NAD or NADH binding / binding / catalytic/ glyceraldehyde-3-phosphate dehydrogenase (phosphorylating)/ glyceraldehyde-3-phosphate dehydrogenase | chr1:5574433-5577406 FORWARDmoderately similar to ( 439) G3PC_GINBI Glyceraldehyde-3-phosphate dehydrogenase, cytosolic (EC 1.2.1.12) - Ginkgo biloba (Ginkgo)highly similar to ( 541) loc_os02g07490 12002.m06096 protein glyceraldehyde-3-phosphate dehydrogenase, cytosolic, putative, expressed  no original description 	T
35.2	not assigned.unknown	MDP0000273470	 very weakly similar to (80.1) AT1G69760 | Symbols:  | unknown protein | chr1:26240094-26240627 REVERSE   no original description 	T
30.2.99	signalling.receptor kinases.misc	MDP0000891518	 nearly identical (1170) AT1G67720 | Symbols:  | leucine-rich repeat family protein / protein kinase family protein | chr1:25386494-25390856 FORWARDmoderately similar to ( 402) NORK_MEDTR Nodulation receptor kinase precursor (EC 2.7.11.1) (Does not make infections protein 2) (Symbiosis receptor-like kinase) (MtSYMRK) - Medicago truncatula (Barrel medic)highly similar to ( 798) loc_os11g01200 12011.m079924 protein transposon protein, putative, unclassified, expressed   no original description 	T
31.3	cell.cycle	MDP0000775949	 moderately similar to ( 275) AT2G45080 | Symbols: cycp3;1 | cycp3;1 (cyclin p3;1); cyclin-dependent protein kinase | chr2:18591688-18592443 FORWARDweakly similar to ( 155) loc_os04g46660 12004.m09614 protein cyclin-dependent protein kinase, putative, expressed no original description 	T
35.1.5	not assigned.no ontology.pentatricopeptide (PPR) repeat-containing protein	MDP0000124997	 moderately similar to ( 270) AT1G04590 | Symbols:  | FUNCTIONS IN: molecular_function unknown; EXPRESSED IN: 23 plant structures; EXPRESSED DURING: 13 growth stages; BEST Arabidopsis thaliana protein match is: pentatricopeptide (PPR) repeat-containing protein (TAIR:AT4G18975.3); Has 54 Blast hits to 54 proteins in 8 species: Archae - 0; Bacteria - 0; Metazoa - 0; Fungi - 0; Plants - 52; Viruses - 0; Other Eukaryotes - 2 (source: NCBI BLink). | chr1:1258760-1261411 REVERSEmoderately similar to ( 213) loc_os11g03200 12011.m04516 protein EMB2748, putative, expressed  no original description 	T
35.1	not assigned.no ontology	MDP0000166320	 highly similar to ( 707) AT1G69040 | Symbols: ACR4 | ACR4 (ACT REPEAT 4); amino acid binding | chr1:25957843-25960079 FORWARDhighly similar to ( 616) loc_os03g29980 12003.m34884 protein ACR4, putative, expressed  no original description 	T
27.3.6	RNA.regulation of transcription.bHLH,Basic Helix-Loop-Helix family	MDP0000323291	 moderately similar to ( 266) AT5G54680 | Symbols: ILR3 | ILR3 (iaa-leucine resistant3); DNA binding / transcription factor | chr5:22217270-22218993 FORWARDmoderately similar to ( 223) loc_os07g35870 12007.m28990 protein bHLH transcription factor, putative, expressed   no original description 	T
33.99	development.unspecified	MDP0000294648	 moderately similar to ( 221) AT3G14770 | Symbols:  | nodulin MtN3 family protein | chr3:4957787-4959202 REVERSEmoderately similar to ( 216) loc_os01g36070 12001.m09904 protein mtN3 protein-like, putative, expressed  no original description 	T
30.2.11	signalling.receptor kinases.leucine rich repeat XI	MDP0000881654	 moderately similar to ( 379) AT1G53730 | Symbols: SRF6 | SRF6 (STRUBBELIG-RECEPTOR FAMILY 6); ATP binding / protein binding / protein kinase/ protein serine/threonine kinase | chr1:20061771-20065475 FORWARDvery weakly similar to (82.8) PGIP3_PHAVU Polygalacturonase inhibitor 3 precursor (Polygalacturonase-inhibiting protein) (PGIP-2) (PGIP-3) - Phaseolus vulgaris (Kidney bean) (French bean)moderately similar to ( 348) loc_os03g08550 12003.m101216 protein leucine-rich repeat transmembrane protein kinase 2, putative, expressed  no original description 	T
20.2.1	stress.abiotic.heat	MDP0000691087	 highly similar to ( 763) AT4G24190 | Symbols: SHD | SHD (SHEPHERD); ATP binding / unfolded protein binding | chr4:12551902-12555851 REVERSEnearly identical (1174) ENPL_CATRO Endoplasmin homolog precursor (GRP94 homolog) - Catharanthus roseus (Rosy periwinkle) (Madagascar periwinkle)highly similar to ( 757) loc_os06g50300 12006.m31934 protein endoplasmin homolog precursor, putative, expressed  no original description 	T
24.1	Biodegradation of Xenobiotics.hydroxyacylglutathione hydrolase	MDP0000132236	 moderately similar to ( 349) AT2G31350 | Symbols: GLX2-5 | GLX2-5 (GLYOXALASE 2-5); hydroxyacylglutathione hydrolase/ iron ion binding / zinc ion binding | chr2:13368451-13370802 FORWARDmoderately similar to ( 311) loc_os09g34100 12009.m06459 protein hydroxyacylglutathione hydrolase 2, mitochondrial precursor, putative, expressed   no original description 	T
34.3	transport.amino acids	MDP0000133621	 moderately similar to ( 488) AT1G08230 | Symbols:  | amino acid transporter family protein | chr1:2583715-2586700 REVERSEvery weakly similar to (75.5) LAX3_MEDTR Auxin transporter-like protein 3 (AUX1-like protein 3) (MtLAX3) - Medicago truncatula (Barrel medic)highly similar to ( 531) loc_os01g63854 12001.m12500 protein amino acid permease, putative, expressed   no original description 	T
20.2.99	stress.abiotic.unspecified	MDP0000271360	 moderately similar to ( 217) AT2G47710 | Symbols:  | universal stress protein (USP) family protein | chr2:19555045-19555956 REVERSEmoderately similar to ( 208) loc_os12g36640 12012.m07450 protein fiber protein Fb19, putative, expressed  no original description 	T
30.2.17	signalling.receptor kinases.DUF 26	MDP0000316178	 moderately similar to ( 440) AT1G53430 | Symbols:  | leucine-rich repeat family protein / protein kinase family protein | chr1:19936073-19940959 FORWARDmoderately similar to ( 223) NORK_MEDTR Nodulation receptor kinase precursor (EC 2.7.11.1) (Does not make infections protein 2) (Symbiosis receptor-like kinase) (MtSYMRK) - Medicago truncatula (Barrel medic)moderately similar to ( 426) loc_os09g17630 12009.m05021 protein receptor-like protein kinase 2, putative, expressed  no original description 	T
27.3.44	RNA.regulation of transcription.Chromatin Remodeling Factors	MDP0000153821	 highly similar to ( 646) AT5G14170 | Symbols: CHC1 | CHC1 | chr5:4568696-4570444 REVERSEhighly similar to ( 573) loc_os04g31320 12004.m08234 protein SWI/SNF-related matrix-associated actin-dependent regulator of chromatin subfamily D member 3, putative, expressed no original description 	T
35.2	not assigned.unknown	MDP0000810488	 weakly similar to ( 168) AT1G80130 | Symbols:  | binding | chr1:30141250-30142478 REVERSEweakly similar to ( 134) loc_os06g40440 12006.m08580 protein expressed protein     no original description 	T
29.5.11.1	protein.degradation.ubiquitin.ubiquitin	MDP0000464712	 moderately similar to ( 251) AT2G47110 | Symbols: UBQ6 | UBQ6; protein binding | chr2:19344701-19345174 FORWARDweakly similar to ( 150) UBIQ_WHEAT Ubiquitin - Triticum aestivum (Wheat)moderately similar to ( 246) loc_os01g22490 12001.m08729 protein 40S ribosomal protein S27a, putative, expressed no original description 	T
20.1.1	stress.biotic.respiratory burst	MDP0000920069	 highly similar to ( 826) AT1G64060 | Symbols: ATRBOH F, ATRBOHF, RBOHAP108, RBOHF | ATRBOH F (ARABIDOPSIS THALIANA RESPIRATORY BURST OXIDASE PROTEIN F); NAD(P)H oxidase | chr1:23770266-23776317 FORWARDhighly similar to ( 774) loc_os01g53294 12001.m11497 protein NADPH oxidase, putative, expressed no original description 	T
35.1	not assigned.no ontology	MDP0000243386	 weakly similar to ( 139) AT5G04820 | Symbols: ATOFP13, OFP13 | OFP13 (ARABIDOPSIS THALIANA OVATE FAMILY PROTEIN 13) | chr5:1399685-1400467 FORWARDweakly similar to ( 112) loc_os11g05770 12011.m04773 protein plant-specific domain TIGR01568 family protein, expressed  no original description 	T
29.5.11.4.2	protein.degradation.ubiquitin.E3.RING	MDP0000287017	 highly similar to ( 584) AT3G16730 | Symbols:  | unknown protein | chr3:5695633-5698863 REVERSEvery weakly similar to (89.7) EL5_ORYSA E3 ubiquitin-protein ligase EL5 (EC 6.3.2.-) - Oryza sativa (Rice)moderately similar to ( 444) loc_os07g33360 12007.m07596 protein expressed protein   no original description 	T
27.3.35	RNA.regulation of transcription.bZIP transcription factor family	MDP0000234166	 moderately similar to ( 330) AT1G06070 | Symbols:  | bZIP transcription factor, putative (bZIP69) | chr1:1835201-1837116 REVERSEmoderately similar to ( 216) RF2A_ORYSA Transcription factor RF2a - Oryza sativa (Rice)moderately similar to ( 216) loc_os09g34060 12009.m06455 protein transcription factor RF2a, putative, expressed  no original description 	T
11.8.1	lipid metabolism.'exotics' (steroids, squalene etc).sphingolipids	MDP0000760376	 highly similar to ( 602) AT2G46210 | Symbols:  | delta-8 sphingolipid desaturase, putative | chr2:18977542-18978891 FORWARDvery weakly similar to (63.9) CYB5_ORYSA Cytochrome b5 - Oryza sativa (Rice)highly similar to ( 556) loc_os09g16920 12009.m04952 protein desaturase/cytochrome b5 protein, putative, expressed  no original description 	T
1.1.5.2	PS.lightreaction.other electron carrier (ox/red).ferredoxin	MDP0000836119	 weakly similar to ( 185) AT2G27510 | Symbols: ATFD3 | ATFD3 (ferredoxin 3); 2 iron, 2 sulfur cluster binding / electron carrier/ iron-sulfur cluster binding | chr2:11758281-11758748 REVERSEweakly similar to ( 177) FER3_MAIZE Ferredoxin-3, chloroplast precursor (Ferredoxin III) (Fd III) - Zea mays (Maize)weakly similar to ( 172) loc_os05g37140 12005.m28058 protein ferredoxin-6, chloroplast precursor, putative, expressed  no original description 	T
30.2.11	signalling.receptor kinases.leucine rich repeat XI	MDP0000379191	 highly similar to ( 843) AT1G65380 | Symbols: CLV2, AtRLP10 | CLV2 (clavata 2); protein binding / receptor signaling protein | chr1:24286943-24289105 FORWARDmoderately similar to ( 241) RPK1_IPONI Receptor-like protein kinase precursor (EC 2.7.11.1) - Ipomoea nil (Japanese morning glory) (Pharbitis nil)moderately similar to ( 439) loc_os02g39100 12002.m08998 protein fasciated ear2, putative, expressed   no original description 	T
30.7	signalling.14-3-3 proteins	MDP0000327058	 moderately similar to ( 409) AT2G42590 | Symbols: GRF9, GF14 MU | GRF9 (GENERAL REGULATORY FACTOR 9); calcium ion binding / protein binding / protein phosphorylated amino acid binding | chr2:17732164-17733775 REVERSEmoderately similar to ( 436) 1433D_SOYBN 14-3-3-like protein D (SGF14D) - Glycine max (Soybean)moderately similar to ( 363) loc_os02g36974 12002.m100253 protein 14-3-3-like protein B, putative, expressed no original description 	T
20.1.7	stress.biotic.PR-proteins	MDP0000463166	 moderately similar to ( 211) AT4G27190 | Symbols:  | disease resistance protein (NBS-LRR class), putative | chr4:13620977-13623934 REVERSEmoderately similar to ( 225) loc_os11g29090 12011.m06821 protein NB-ARC domain containing protein PRK07003  no original description 	T
35.2	not assigned.unknown	MDP0000125950	 moderately similar to ( 245) AT1G52590 | Symbols:  | FUNCTIONS IN: molecular_function unknown; INVOLVED IN: biological_process unknown; LOCATED IN: plastoglobule; EXPRESSED IN: 22 plant structures; EXPRESSED DURING: 13 growth stages; CONTAINS InterPro DOMAIN/s: Putative thiol-disulphide oxidoreductase DCC (InterPro:IPR007263); BEST Arabidopsis thaliana protein match is: RNase H domain-containing protein (TAIR:AT1G24090.1); Has 622 Blast hits to 622 proteins in 201 species: Archae - 0; Bacteria - 363; Metazoa - 0; Fungi - 0; Plants - 36; Viruses - 0; Other Eukaryotes - 223 (source: NCBI BLink). | chr1:19589224-19590231 REVERSEweakly similar to ( 197) loc_os01g07800 12001.m07407 protein thiol-disulphide oxidoreductase DCC, putative, expressed no original description 	T
16.2.1.10	secondary metabolism.phenylpropanoids.lignin biosynthesis.CAD	MDP0000188052	 highly similar to ( 605) AT5G51970 | Symbols:  | sorbitol dehydrogenase, putative / L-iditol 2-dehydrogenase, putative | chr5:21111820-21113284 FORWARDvery weakly similar to ( 100) ADHX_ORYSA Alcohol dehydrogenase class 3 (EC 1.1.1.1) (Alcohol dehydrogenase class III) (S-(hydroxymethyl)glutathione dehydrogenase) (EC 1.1.1.284) (Glutathione-dependent formaldehyde dehydrogenase) (FDH) (FALDH) (GSH-FDH) - Oryza sativa (Rice)highly similar to ( 561) loc_os08g43190 12008.m08293 protein sorbitol dehydrogenase, putative, expressed  no original description 	T
8.1.7	TCA / org transformation.TCA.succinate dehydrogenase	MDP0000813172	 nearly identical (1097) AT5G66760 | Symbols: SDH1-1 | SDH1-1; ATP binding / succinate dehydrogenase | chr5:26653776-26657224 FORWARDnearly identical (1089) DHSA_ORYSA Succinate dehydrogenase [ubiquinone] flavoprotein subunit, mitochondrial precursor (EC 1.3.5.1) (FP) (Flavoprotein subunit of complex II) - Oryza sativa (Rice)nearly identical (1089) loc_os07g04240 12007.m04893 protein succinate dehydrogenase flavoprotein subunit,mitochondrial precursor, putative, expressed   no original description 	T
29.3.4.99	protein.targeting.secretory pathway.unspecified	MDP0000842619	 moderately similar to ( 379) AT1G11890 | Symbols: SEC22, ATSEC22 | SEC22; transporter | chr1:4011509-4012835 FORWARDmoderately similar to ( 368) loc_os06g09850 12006.m32005 protein 25.3 kDa vesicle transport protein, putative, expressed no original description 	T
27.3.3	RNA.regulation of transcription.AP2/EREBP, APETALA2/Ethylene-responsive element binding protein family	MDP0000517262	 weakly similar to ( 150) AT5G07580 | Symbols:  | DNA binding / transcription factor | chr5:2399525-2400349 FORWARDweakly similar to ( 130) ERF5_TOBAC Ethylene-responsive transcription factor 5 (Ethylene-responsive element-binding factor 5 homolog) (EREBP-4) (NtERF4) - Nicotiana tabacum (Common tobacco)weakly similar to ( 111) loc_os10g41330 12010.m06897 protein ethylene-responsive transcription factor 5, putative, expressed  no original description 	T
11.9.2.1	lipid metabolism.lipid degradation.lipases.triacylglycerol lipase	MDP0000252076	 moderately similar to ( 362) AT3G14075 | Symbols:  | lipase class 3 family protein | chr3:4663819-4666338 REVERSEweakly similar to ( 191) PSA6_SOYBN Proteasome subunit alpha type 6 (EC 3.4.25.1) (20S proteasome alpha subunit A) (20S proteasome subunit alpha-1) (Proteasome iota subunit) - Glycine max (Soybean)moderately similar to ( 337) loc_os02g54010 12002.m10430 protein triacylglycerol lipase, putative, expressed no original description 	T
35.2	not assigned.unknown	MDP0000185239	 weakly similar to ( 156) AT2G16900 | Symbols:  | FUNCTIONS IN: molecular_function unknown; INVOLVED IN: biological_process unknown; LOCATED IN: plasma membrane; EXPRESSED IN: 24 plant structures; EXPRESSED DURING: 13 growth stages; CONTAINS InterPro DOMAIN/s: Phospholipase-like, arabidopsis (InterPro:IPR007942); BEST Arabidopsis thaliana protein match is: unknown protein (TAIR:AT4G35110.4); Has 280 Blast hits to 196 proteins in 46 species: Archae - 2; Bacteria - 4; Metazoa - 40; Fungi - 6; Plants - 96; Viruses - 48; Other Eukaryotes - 84 (source: NCBI BLink). | chr2:7323857-7324886 REVERSEvery weakly similar to (51.6) loc_os06g49740 12006.m32188 protein GCIP-interacting protein, putative, expressed  no original description 	T
16.8.2	secondary metabolism.flavonoids.chalcones	MDP0000575740	 highly similar to ( 667) AT5G13930 | Symbols: CHS, TT4, ATCHS | TT4 (TRANSPARENT TESTA 4); naringenin-chalcone synthase | chr5:4488762-4490035 FORWARDhighly similar to ( 718) CHS1_CITSI Chalcone synthase 1 (EC 2.3.1.74) (Naringenin-chalcone synthase 1) - Citrus sinensis (Sweet orange)highly similar to ( 662) loc_os11g32650 12011.m07166 protein chalcone synthase, putative, expressed   no original description 	T
27.1	RNA.processing	MDP0000403091	 highly similar to ( 513) AT1G61010 | Symbols: CPSF73-I | CPSF73-I (CLEAVAGE AND POLYADENYLATION SPECIFICITY FACTOR 73-I); protein binding | chr1:22474954-22477660 REVERSEweakly similar to ( 101) CPSF2_ORYSA Cleavage and polyadenylation specificity factor 100 kDa subunit (CPSF 100 kDa subunit) - Oryza sativa (Rice)highly similar to ( 513) loc_os03g63590 12003.m11227 protein cleavage and polyadenylation specificity factor, 73 kDa subunit, putative, expressed  no original description 	T
1.3.3	PS.calvin cycle.phosphoglycerate kinase	MDP0000325406	 highly similar to ( 744) AT1G56190 | Symbols:  | phosphoglycerate kinase, putative | chr1:21028622-21030454 FORWARDhighly similar to ( 760) PGKH_TOBAC Phosphoglycerate kinase, chloroplast precursor (EC 2.7.2.3) - Nicotiana tabacum (Common tobacco)highly similar to ( 730) loc_os05g41640 12005.m08338 protein phosphoglycerate kinase, chloroplast precursor, putative, expressed  no original description 	T
28.1.3	DNA.synthesis/chromatin structure.histone	MDP0000361591	 weakly similar to ( 176) AT1G07790 | Symbols: HTB1 | HTB1; DNA binding | chr1:2413049-2413495 FORWARDweakly similar to ( 177) H2B1_MEDTR Probable histone H2B.1 - Medicago truncatula (Barrel medic)weakly similar to ( 171) loc_os01g62230 12001.m12346 protein histone H2B.4, putative, expressed no original description 	T
35.2	not assigned.unknown	MDP0000145486	 weakly similar to ( 155) AT5G20190 | Symbols:  | binding | chr5:6814093-6815171 FORWARDweakly similar to ( 134) loc_os06g40440 12006.m08580 protein expressed protein    no original description 	T
29.4.1.57	protein.postranslational modification.kinase.receptor like cytoplasmatic kinase VII	MDP0000322059	 highly similar to ( 691) AT3G03770 | Symbols:  | leucine-rich repeat transmembrane protein kinase, putative | chr3:945303-948436 REVERSEweakly similar to ( 101) CRI4_MAIZE Putative receptor protein kinase CRINKLY4 precursor (EC 2.7.11.1) - Zea mays (Maize)highly similar to ( 573) loc_os07g07230 12007.m079614 protein ATP binding protein, putative, expressed   no original description 	T
35.2	not assigned.unknown	MDP0000850393	 moderately similar to ( 228) AT1G50900 | Symbols:  | FUNCTIONS IN: molecular_function unknown; INVOLVED IN: biological_process unknown; LOCATED IN: chloroplast, chloroplast stroma; EXPRESSED IN: 22 plant structures; EXPRESSED DURING: 13 growth stages; CONTAINS InterPro DOMAIN/s: Ankyrin (InterPro:IPR002110); Has 30 Blast hits to 30 proteins in 12 species: Archae - 0; Bacteria - 0; Metazoa - 0; Fungi - 0; Plants - 27; Viruses - 0; Other Eukaryotes - 3 (source: NCBI BLink). | chr1:18866272-18867014 FORWARDweakly similar to ( 176) loc_os07g33660 12007.m07625 protein expressed protein   no original description 	T
35.2	not assigned.unknown	MDP0000349483	 weakly similar to ( 175) AT2G30230 | Symbols:  | unknown protein | chr2:12897156-12897689 FORWARDweakly similar to ( 157) loc_os03g18660 12003.m07287 protein conserved hypothetical protein  no original description 	T
20.2.99	stress.abiotic.unspecified	MDP0000397306	 moderately similar to ( 284) AT5G20630 | Symbols: GLP3, GLP3A, GLP3B, ATGER3, GER3 | GER3 (GERMIN 3); oxalate oxidase | chr5:6975315-6975950 REVERSEmoderately similar to ( 357) AB19A_PRUPE Auxin-binding protein ABP19a precursor - Prunus persica (Peach)moderately similar to ( 257) loc_os08g35760 12008.m07565 protein auxin-binding protein ABP20 precursor, putative, expressed no original description 	T
8.3	TCA / org transformation.carbonic anhydrases	MDP0000807738	 moderately similar to ( 359) AT5G14740 | Symbols: CA2, CA18, BETA CA2 | CA2 (CARBONIC ANHYDRASE 2); carbonate dehydratase/ zinc ion binding | chr5:4758257-4762315 FORWARDmoderately similar to ( 359) CAHC_SPIOL Carbonic anhydrase, chloroplast precursor (EC 4.2.1.1) (Carbonate dehydratase) - Spinacia oleracea (Spinach)moderately similar to ( 229) loc_os01g45274 12001.m150428 protein carbonic anhydrase, chloroplast precursor, putative, expressed  no original description 	T
35.2	not assigned.unknown	MDP0000802300	  no original description 	T
29.4	protein.postranslational modification	MDP0000925799	 moderately similar to ( 354) AT1G54510 | Symbols: ATNEK1 | ATNEK1 (ARABIDOPSIS THALIANA NIMA-RELATED SERINE/THREONINE KINASE 1); ATP binding / kinase/ protein kinase/ protein serine/threonine kinase | chr1:20358603-20362006 REVERSEmoderately similar to ( 340) loc_os12g41180 12012.m07900 protein LSTK-1-like kinase, putative, expressed  no original description 	T
35.2	not assigned.unknown	MDP0000197166	 weakly similar to ( 103) AT1G69760 | Symbols:  | unknown protein | chr1:26240094-26240627 REVERSE   no original description 	T
29.2.1.2.1.31	protein.synthesis.ribosomal protein.eukaryotic.40S subunit.SA	MDP0000260722	 moderately similar to ( 380) AT1G72370 | Symbols: P40, AP40, RP40, RPSAA | P40; structural constituent of ribosome | chr1:27243148-27244842 REVERSEmoderately similar to ( 396) RSSA_SOYBN 40S ribosomal protein SA (p40) - Glycine max (Soybean)moderately similar to ( 388) loc_os03g08440 12003.m35048 protein 40S ribosomal protein SA, putative, expressed no original description 	T
35.1	not assigned.no ontology	MDP0000323611	 moderately similar to ( 211) AT5G06320 | Symbols: NHL3 | NHL3 | chr5:1931016-1931711 REVERSEweakly similar to ( 119) loc_os04g58850 12004.m10765 protein harpin inducing protein, putative, expressed  no original description 	T
29.4	protein.postranslational modification	MDP0000292157	 moderately similar to ( 406) AT3G05640 | Symbols:  | protein phosphatase 2C, putative / PP2C, putative | chr3:1640610-1642227 REVERSEmoderately similar to ( 350) loc_os05g29030 12005.m07189 protein catalytic/ protein phosphatase type 2C, putative, expressed MSP1_C  no original description 	T
20.2.99	stress.abiotic.unspecified	MDP0000690287	 moderately similar to ( 293) AT3G10080 | Symbols:  | germin-like protein, putative | chr3:3107476-3108159 REVERSEweakly similar to ( 158) OXO2_HORVU Oxalate oxidase 2 precursor (EC 1.2.3.4) (Germin) - Hordeum vulgare (Barley)moderately similar to ( 239) loc_os02g32980 12002.m08391 protein germin-like protein subfamily 3 member 2 precursor, putative, expressed  no original description 	T
35.2	not assigned.unknown	MDP0000203907	   no original description 	T
13.1.3.2.1	amino acid metabolism.synthesis.aspartate family.threonine.threonine synthase	MDP0000873427	 highly similar to ( 802) AT4G29840 | Symbols: MTO2, TS | MTO2 (METHIONINE OVER-ACCUMULATOR 2); threonine synthase | chr4:14599434-14601014 REVERSEhighly similar to ( 806) THRC_SOLTU Threonine synthase, chloroplast precursor (EC 4.2.3.1) (TS) - Solanum tuberosum (Potato)highly similar to ( 721) loc_os05g47640 12005.m08836 protein threonine synthase, chloroplast precursor, putative, expressed  no original description 	T
30.11	signalling.light	MDP0000198439	 moderately similar to ( 223) AT5G48800 | Symbols:  | phototropic-responsive NPH3 family protein | chr5:19786881-19789003 FORWARDmoderately similar to ( 202) NPH3_ORYSA Coleoptile phototropism protein 1 (Non-phototropic hypocotyl 3-like protein) (NPH3-like protein) - Oryza sativa (Rice)moderately similar to ( 203) loc_os03g10800 12003.m34805 protein transposon protein, putative, Mutator sub-class, expressed no original description 	T
30.3	signalling.calcium	MDP0000319861	 nearly identical (1251) AT2G41560 | Symbols: ACA4 | ACA4 (AUTO-INHIBITED CA(2+)-ATPASE, ISOFORM 4); calcium-transporting ATPase/ calmodulin binding | chr2:17332256-17337179 REVERSEnearly identical (1196) ACA4_ORYSA Probable calcium-transporting ATPase 4, plasma membrane-type (EC 3.6.3.8) (Ca(2+)-ATPase isoform 4) - Oryza sativa (Rice)nearly identical (1196) loc_os12g04220 12012.m04416 protein calcium-transporting ATPase 4, plasma membrane-type, putative, expressed  no original description 	T
33.99	development.unspecified	MDP0000232313	 very weakly similar to (71.2) AT2G22540 | Symbols: SVP, AGL22 | SVP (SHORT VEGETATIVE PHASE); transcription factor/ translation repressor, nucleic acid binding | chr2:9580417-9583603 FORWARDvery weakly similar to (65.9) MAD47_ORYSA MADS-box transcription factor 47 (OsMADS47) - Oryza sativa (Rice)very weakly similar to (67.8) loc_os03g08754 12003.m35564 protein MADS-box transcription factor 47, putative, expressed no original description 	T
26.7	misc.oxidases - copper, flavone etc	MDP0000522743	 highly similar to ( 808) AT5G66920 | Symbols: sks17 | sks17 (SKU5 Similar 17); copper ion binding / oxidoreductase | chr5:26722963-26725370 FORWARDhighly similar to ( 563) ASO_BRANA L-ascorbate oxidase homolog precursor (EC 1.10.3.3) (Ascorbase) - Brassica napus (Rape)highly similar to ( 697) loc_os07g02810 12007.m04753 protein L-ascorbate oxidase homolog precursor, putative, expressed  no original description 	T
29.5.11.5	protein.degradation.ubiquitin.ubiquitin protease	MDP0000311570	 highly similar to ( 707) AT1G17110 | Symbols: UBP15 | UBP15 (UBIQUITIN-SPECIFIC PROTEASE 15); ubiquitin-specific protease | chr1:5845816-5849889 REVERSEhighly similar to ( 651) loc_os02g14730 12002.m06717 protein UBP15, putative, expressed  no original description 	T
28.99	DNA.unspecified	MDP0000322282	 highly similar to ( 970) AT2G32415 | Symbols:  | 3'-5' exonuclease/ nucleic acid binding | chr2:13761307-13765421 REVERSEhighly similar to ( 653) loc_os07g32890 12007.m07551 protein 3-5 exonuclease family protein, expressed  no original description 	T
30.2.24	signalling.receptor kinases.S-locus glycoprotein like	MDP0000264429	 moderately similar to ( 258) AT4G03230 | Symbols:  | ATP binding / kinase/ protein kinase/ protein serine/threonine kinase/ protein tyrosine kinase/ sugar binding | chr4:1419278-1422804 REVERSEweakly similar to ( 123) SLSG3_BRAOL S-locus-specific glycoprotein S13 precursor (SLSG-13) (Fragment) - Brassica oleracea (Wild cabbage)weakly similar to ( 137) loc_os09g37840 12009.m60217 protein ATP binding protein, putative  no original description 	T
29.4	protein.postranslational modification	MDP0000274290	 highly similar to ( 597) AT3G51630 | Symbols: WNK5, ZIK1, ATWNK5 | WNK5 (WITH NO LYSINE (K) KINASE 5); protein kinase | chr3:19149487-19151924 FORWARDvery weakly similar to (82.4) MPK14_ORYSA Mitogen-activated protein kinase 14 (EC 2.7.11.24) (MAP kinase 14) - Oryza sativa (Rice)highly similar to ( 525) loc_os11g02300 12011.m28642 protein mitogen-activated protein kinase, putative, expressed   no original description 	T
27.3.6	RNA.regulation of transcription.bHLH,Basic Helix-Loop-Helix family	MDP0000321049	 moderately similar to ( 219) AT2G46810 | Symbols:  | basic helix-loop-helix (bHLH) family protein | chr2:19239694-19242373 FORWARDweakly similar to ( 182) loc_os09g29360 12009.m06087 protein DNA binding protein, putative, expressed   no original description 	T
20.2.99	stress.abiotic.unspecified	MDP0000216647	 weakly similar to ( 168) AT5G15780 | Symbols:  | pollen Ole e 1 allergen and extensin family protein | chr5:5144898-5146297 REVERSEvery weakly similar to (61.2) loc_os10g13850 12010.m04638 protein expressed protein   no original description 	T
35.2	not assigned.unknown	MDP0000137570	 nearly identical (1406) AT5G13680 | Symbols: ELO2, ABO1 | ABO1 (ABA-OVERLY SENSITIVE 1); transcription elongation regulator | chr5:4410522-4415471 REVERSEnearly identical (1288) loc_os07g37640 12007.m08014 protein IKI3 family protein, expressed  no original description 	T
34.14	transport.unspecified cations	MDP0000513157	 highly similar to ( 577) AT1G16390 | Symbols: ATOCT3 | ATOCT3 (ARABIDOPSIS THALIANA ORGANIC CATION/CARNITINE TRANSPORTER 3); carbohydrate transmembrane transporter/ sugar:hydrogen symporter | chr1:5602921-5604477 FORWARDmoderately similar to ( 452) loc_os07g37510 12007.m08001 protein carbohydrate transporter/ sugar porter, putative   TrbL  no original description 	T
34.2	transport.sugars	MDP0000513157	 highly similar to ( 577) AT1G16390 | Symbols: ATOCT3 | ATOCT3 (ARABIDOPSIS THALIANA ORGANIC CATION/CARNITINE TRANSPORTER 3); carbohydrate transmembrane transporter/ sugar:hydrogen symporter | chr1:5602921-5604477 FORWARDmoderately similar to ( 452) loc_os07g37510 12007.m08001 protein carbohydrate transporter/ sugar porter, putative   TrbL  no original description 	T
11.1.3	lipid metabolism.FA synthesis and FA elongation.ketoacyl ACP synthase	MDP0000250127	 highly similar to ( 609) AT5G46290 | Symbols: KAS I | KAS I (3-KETOACYL-ACYL CARRIER PROTEIN SYNTHASE I); catalytic/ fatty-acid synthase | chr5:18774439-18776629 REVERSEhighly similar to ( 576) KASC1_HORVU 3-oxoacyl-[acyl-carrier-protein] synthase I, chloroplast precursor (EC 2.3.1.41) (Beta-ketoacyl-ACP synthase I) (KAS I) - Hordeum vulgare (Barley)highly similar to ( 589) loc_os06g09630 12006.m05681 protein 3-oxoacyl-synthase I, chloroplast precursor, putative, expressed   no original description 	T
35.1	not assigned.no ontology	MDP0000164828	 moderately similar to ( 338) AT4G03410 | Symbols:  | peroxisomal membrane protein-related | chr4:1501907-1503503 FORWARDmoderately similar to ( 327) loc_os03g38730 12003.m35298 protein mpv17 / PMP22 family protein, expressed  no original description 	T
26.4.1	misc.beta 1,3 glucan hydrolases.glucan endo-1,3-beta-glucosidase	MDP0000404948	 moderately similar to ( 485) AT1G32860 | Symbols:  | glycosyl hydrolase family 17 protein | chr1:11907308-11908803 REVERSEmoderately similar to ( 270) E13B_WHEAT Glucan endo-1,3-beta-glucosidase precursor (EC 3.2.1.39) ((1->3)-beta-glucan endohydrolase) ((1->3)-beta-glucanase) (Beta-1,3-endoglucanase) - Triticum aestivum (Wheat)moderately similar to ( 416) loc_os04g33640 12004.m35305 protein glucan endo-1,3-beta-glucosidase 7 precursor, putative, expressed  TrbL  no original description 	T
15.2	metal handling.binding, chelation and storage	MDP0000906645	 weakly similar to ( 169) AT3G06130 | Symbols:  | heavy-metal-associated domain-containing protein | chr3:1853048-1854673 REVERSEweakly similar to ( 124) loc_os12g05040 12012.m073762 protein metal ion binding protein, putative, expressed   no original description 	T
1.1.1.2	PS.lightreaction.photosystem II.PSII polypeptide subunits	MDP0000242738	 weakly similar to ( 129) AT5G02120 | Symbols: OHP | OHP (ONE HELIX PROTEIN) | chr5:419144-419633 FORWARDweakly similar to ( 127) loc_os05g22730 12005.m06569 protein expressed protein  no original description 	T
29.5	protein.degradation	MDP0000132788	 highly similar to ( 558) AT3G51800 | Symbols: ATG2 | ATG2; aminopeptidase/ metalloexopeptidase | chr3:19211261-19213568 REVERSEhighly similar to ( 573) loc_os05g28280 12005.m07114 protein proliferation-associated protein 2G4, putative, expressed  no original description 	T
35.2	not assigned.unknown	MDP0000250504	 moderately similar to ( 405) AT4G27010 | Symbols:  | unknown protein | chr4:13558466-13568296 FORWARDmoderately similar to ( 276) loc_os04g53380 12004.m10229 protein expressed protein  no original description 	T
31.1	cell.organisation	MDP0000651961	 moderately similar to ( 230) AT5G59890 | Symbols: ADF4, ATADF4 | ADF4 (ACTIN DEPOLYMERIZING FACTOR 4); actin binding | chr5:24123107-24123596 FORWARDmoderately similar to ( 244) ADF1_PETHY Actin-depolymerizing factor 1 (ADF 1) - Petunia hybrida (Petunia)moderately similar to ( 237) loc_os05g02250 12005.m64199 protein expressed protein  no original description 	T
10.7	cell wall.modification	MDP0000233177	 moderately similar to ( 388) AT1G10550 | Symbols: XTH33, XET | XTH33; hydrolase, acting on glycosyl bonds / hydrolase, hydrolyzing O-glycosyl compounds / xyloglucan:xyloglucosyl transferase | chr1:3479257-3480957 REVERSEweakly similar to ( 191) XTH_TOBAC Probable xyloglucan endotransglucosylase/hydrolase protein precursor (EC 2.4.1.207) - Nicotiana tabacum (Common tobacco)moderately similar to ( 246) loc_os03g13570 12003.m06802 protein xyloglucan endotransglucosylase/hydrolase protein 27 precursor, putative, expressed no original description 	T
27.3.3	RNA.regulation of transcription.AP2/EREBP, APETALA2/Ethylene-responsive element binding protein family	MDP0000525933	 weakly similar to ( 183) AT5G19790 | Symbols: RAP2.11 | RAP2.11 (related to AP2 11); DNA binding / transcription factor | chr5:6689271-6690032 REVERSEvery weakly similar to (57.0) ERF1_ORYSA Ethylene-responsive transcription factor 1 (Ethylene-responsive element-binding factor 1) (EREBP-1) (OsEREBP1) - Oryza sativa (Rice)weakly similar to ( 119) loc_os07g10410 12007.m05496 protein conserved hypothetical protein no original description 	T
9.7	mitochondrial electron transport / ATP synthesis.cytochrome c oxidase	MDP0000174827	 weakly similar to ( 157) AT1G80230 | Symbols:  | cytochrome c oxidase family protein | chr1:30169784-30170999 REVERSEweakly similar to ( 150) loc_os06g05080 12006.m05237 protein cytochrome c oxidase polypeptide Vb, mitochondrial precursor, putative, expressed  no original description 	T
35.2	not assigned.unknown	MDP0000148485	 moderately similar to ( 265) AT5G38900 | Symbols:  | DSBA oxidoreductase family protein | chr5:15573745-15574945 REVERSEmoderately similar to ( 271) loc_os03g40194 12003.m09103 protein disease resistance RPP13-like protein 1, putative, expressed  no original description 	T
33.99	development.unspecified	MDP0000168479	 highly similar to ( 796) AT1G73720 | Symbols:  | transducin family protein / WD-40 repeat family protein | chr1:27725059-27729722 FORWARDvery weakly similar to (88.2) PF20_CHLRE Flagellar WD repeat protein PF20 - Chlamydomonas reinhardtiihighly similar to ( 815) loc_os01g21940 12001.m08678 protein WD-40 repeat protein, putative, expressed   no original description 	T
29.5.11.5	protein.degradation.ubiquitin.ubiquitin protease	MDP0000278373	 highly similar to ( 646) AT4G31670 | Symbols: UBP18 | UBP18 (UBIQUITIN-SPECIFIC PROTEASE 18); cysteine-type endopeptidase/ ubiquitin thiolesterase | chr4:15336007-15339506 REVERSEhighly similar to ( 577) loc_os06g44380 12006.m08966 protein ubiquitin carboxyl-terminal hydrolase family protein, expressed  no original description 	T
20.2.1	stress.abiotic.heat	MDP0000187867	 moderately similar to ( 402) AT2G26890 | Symbols: GRV2, KAM2 | GRV2 (GRAVITROPISM DEFECTIVE 2); binding / heat shock protein binding | chr2:11462327-11472259 REVERSEmoderately similar to ( 421) loc_os10g42439 12010.m06997 protein GRV2, putative, expressed   no original description 	T
35.2	not assigned.unknown	MDP0000834121	 highly similar to ( 580) AT1G29980 | Symbols:  | INVOLVED IN: biological_process unknown; LOCATED IN: plasma membrane, anchored to membrane; EXPRESSED IN: 24 plant structures; EXPRESSED DURING: 13 growth stages; CONTAINS InterPro DOMAIN/s: Protein of unknown function DUF642 (InterPro:IPR006946), Galactose-binding like (InterPro:IPR008979); BEST Arabidopsis thaliana protein match is: unknown protein (TAIR:AT2G34510.1); Has 180 Blast hits to 163 proteins in 15 species: Archae - 0; Bacteria - 10; Metazoa - 0; Fungi - 0; Plants - 170; Viruses - 0; Other Eukaryotes - 0 (source: NCBI BLink). | chr1:10503411-10505994 REVERSEhighly similar to ( 518) loc_os01g55190 12001.m11679 protein expressed protein   no original description 	T
35.1	not assigned.no ontology	MDP0000258781	 nearly identical (1312) AT1G23870 | Symbols: ATTPS9, TPS9 | ATTPS9; transferase, transferring glycosyl groups / trehalose-phosphatase | chr1:8432695-8435506 FORWARDnearly identical (1125) loc_os08g31980 12008.m07192 protein trehalose 6-phosphate synthase, putative, expressed   no original description 	T
35.2	not assigned.unknown	MDP0000376446	 weakly similar to ( 147) AT3G45050 | Symbols:  | unknown protein | chr3:16476023-16477107 FORWARDweakly similar to ( 114) loc_os06g51470 12006.m09670 protein expressed protein no original description 	T
31.2	cell.division	MDP0000509426	 highly similar to ( 578) AT2G36250 | Symbols: FTSZ2-1, ATFTSZ2-1 | FTSZ2-1; protein binding / structural molecule | chr2:15197661-15199932 REVERSEhighly similar to ( 504) loc_os03g44420 12003.m09481 protein cell division protein ftsZ, putative, expressed   no original description 	T
27.3.99	RNA.regulation of transcription.unclassified	MDP0000135639	 highly similar to ( 540) AT4G24630 | Symbols:  | receptor/ zinc ion binding | chr4:12714919-12717111 FORWARDmoderately similar to ( 489) loc_os05g36090 12005.m07839 protein palmitoyltransferase ZDHHC9, putative, expressed no original description 	T
26.3.2	misc.gluco-, galacto- and mannosidases.beta-galactosidase	MDP0000195063	 nearly identical (1441) AT4G36360 | Symbols: BGAL3 | BGAL3 (beta-galactosidase 3); beta-galactosidase/ catalytic/ cation binding / sugar binding | chr4:17176840-17181143 REVERSEnearly identical (1117) BGAL_ASPOF Beta-galactosidase precursor (EC 3.2.1.23) (Lactase) - Asparagus officinalis (Garden asparagus)nearly identical (1298) loc_os03g06940 12003.m35030 protein beta-galactosidase precursor, putative, expressed  no original description 	T
31.1	cell.organisation	MDP0000832105	 highly similar to ( 841) AT1G50010 | Symbols: TUA2 | TUA2; structural constituent of cytoskeleton | chr1:18517737-18519729 FORWARDhighly similar to ( 843) TBA2_GOSHI Tubulin alpha-2 chain (Alpha-2 tubulin) - Gossypium hirsutum (Upland cotton)highly similar to ( 830) loc_os11g14220 12011.m28666 protein tubulin alpha-3 chain, putative, expressed   no original description 	T
29.4	protein.postranslational modification	MDP0000746382	 highly similar to ( 780) AT5G57020 | Symbols: NMT1, ATNMT1 | NMT1 (MYRISTOYL-COA:PROTEIN N-MYRISTOYLTRANSFERASE); glycylpeptide N-tetradecanoyltransferase/ myristoyltransferase | chr5:23075451-23076755 FORWARDhighly similar to ( 675) loc_os01g51170 12001.m11298 protein glycylpeptide N-tetradecanoyltransferase 1, putative, expressed  no original description 	T
35.2	not assigned.unknown	MDP0000666176	 nearly identical (1297) AT5G11560 | Symbols:  | catalytic | chr5:3709734-3713994 REVERSEnearly identical (1195) loc_os05g14170 12005.m05829 protein expressed protein   no original description 	T
27.3.4	RNA.regulation of transcription.ARF, Auxin Response Factor family	MDP0000412781	 highly similar to ( 820) AT5G60450 | Symbols: ARF4 | ARF4 (AUXIN RESPONSE FACTOR 4); transcription factor | chr5:24308558-24312187 REVERSEvery weakly similar to (53.9) AX22C_PHAAU Auxin-induced protein 22C (Indole-3-acetic acid-induced protein ARG12) - Phaseolus aureus (Mung bean) (Vigna radiata)moderately similar to ( 479) loc_os01g70270 12001.m43358 protein auxin response factor 2, putative, expressed  no original description 	T
1.1.4.4	PS.lightreaction.ATP synthase.gamma chain	MDP0000289861	 highly similar to ( 561) AT4G04640 | Symbols: ATPC1 | ATPC1; enzyme regulator | chr4:2350761-2351882 REVERSEhighly similar to ( 587) ATPG_TOBAC ATP synthase gamma chain, chloroplast precursor (EC 3.6.3.14) - Nicotiana tabacum (Common tobacco)moderately similar to ( 479) loc_os07g32880 12007.m07550 protein ATP synthase gamma chain, chloroplast precursor, putative, expressed  no original description 	T
26.10	misc.cytochrome P450	MDP0000221435	 highly similar to ( 519) AT4G31940 | Symbols: CYP82C4 | CYP82C4; electron carrier/ heme binding / iron ion binding / monooxygenase/ oxygen binding | chr4:15452040-15453966 FORWARDhighly similar to ( 587) C82A3_SOYBN Cytochrome P450 82A3 (EC 1.14.-.-) (P450 CP6) - Glycine max (Soybean)moderately similar to ( 346) loc_os09g26940 12009.m05846 protein flavonoid 3-monooxygenase, putative, expressed  no original description 	T
29.4	protein.postranslational modification	MDP0000141005	 nearly identical (1068) AT3G25800 | Symbols: PDF1, PR 65, PP2AA2 | PP2AA2 (PROTEIN PHOSPHATASE 2A SUBUNIT A2); protein phosphatase type 2A regulator | chr3:9423036-9425783 REVERSEhighly similar to ( 672) 2AAA_PEA Protein phosphatase PP2A regulatory subunit A (PR65) (Fragment) - Pisum sativum (Garden pea)nearly identical (1048) loc_os09g07510 12009.m22048 protein serine/threonine-protein phosphatase 2A 65 kDa regulatory subunit Abeta isoform, putative, expressed   no original description 	T
35.2	not assigned.unknown	MDP0000238960	 moderately similar to ( 337) AT4G31530 | Symbols:  | binding / catalytic | chr4:15282281-15284064 FORWARDmoderately similar to ( 323) loc_os03g23980 12003.m07744 protein 3-beta hydroxysteroid dehydrogenase/isomerase family protein, putative, expressed no original description 	T
35.2	not assigned.unknown	MDP0000300728	 moderately similar to ( 317) AT3G26580 | Symbols:  | INVOLVED IN: biological_process unknown; LOCATED IN: chloroplast thylakoid membrane; EXPRESSED IN: 23 plant structures; EXPRESSED DURING: 13 growth stages; CONTAINS InterPro DOMAIN/s: Tetratricopeptide region (InterPro:IPR013026); Has 2147 Blast hits to 1154 proteins in 168 species: Archae - 2; Bacteria - 99; Metazoa - 1301; Fungi - 180; Plants - 105; Viruses - 57; Other Eukaryotes - 403 (source: NCBI BLink). | chr3:9758933-9760349 FORWARDmoderately similar to ( 236) loc_os03g31490 12003.m08396 protein ATP-dependent protease Clp, ATPase subunit, putative, expressed  TrbL  no original description 	T
29.4	protein.postranslational modification	MDP0000185491	 highly similar to ( 516) AT3G48750 | Symbols: CDKA;1, CDC2AAT, CDK2, CDC2, CDC2A, CDKA1 | CDC2 (CELL DIVISION CONTROL 2); cyclin-dependent protein kinase/ kinase/ protein binding / protein kinase | chr3:18072238-18074296 FORWARDhighly similar to ( 552) CDC2A_ANTMA Cell division control protein 2 homolog A (EC 2.7.11.22) (EC 2.7.11.23) - Antirrhinum majus (Garden snapdragon)highly similar to ( 512) loc_os03g02680 12003.m101097 protein cell division control protein 2 homolog 1, putative, expressed  no original description 	T
35.2	not assigned.unknown	MDP0000331021	 moderately similar to ( 303) AT4G38260 | Symbols:  | unknown protein | chr4:17937077-17938210 REVERSEmoderately similar to ( 312) loc_os09g15480 12009.m04809 protein Ser/Thr-rich protein T10 in DGCR region, putative, expressed no original description 	T
35.2	not assigned.unknown	MDP0000821000	 highly similar to ( 606) AT1G15980 | Symbols: NDF1 | NDF1 (NDH-DEPENDENT CYCLIC ELECTRON FLOW 1) | chr1:5489314-5491199 FORWARDhighly similar to ( 515) loc_os08g17390 12008.m05858 protein expressed protein   no original description 	T
27.3.40	RNA.regulation of transcription.Aux/IAA family	MDP0000208345	 moderately similar to ( 212) AT1G04240 | Symbols: SHY2, IAA3 | SHY2 (SHORT HYPOCOTYL 2); transcription factor | chr1:1128564-1129319 REVERSEmoderately similar to ( 219) AX22B_PHAAU Auxin-induced protein 22B (Indole-3-acetic acid-induced protein ARG4) - Phaseolus aureus (Mung bean) (Vigna radiata)weakly similar to ( 161) loc_os05g14180 12005.m27564 protein OsIAA17 - Auxin-responsive Aux/IAA gene family member, expressed  no original description 	T
34.8	transport.metabolite transporters at the envelope membrane	MDP0000192816	 highly similar to ( 587) AT3G17430 | Symbols:  | phosphate translocator-related | chr3:5966597-5968962 FORWARDvery weakly similar to (54.7) TPT_PEA Triose phosphate/phosphate translocator, chloroplast precursor (cTPT) (p36) (E30) - Pisum sativum (Garden pea)highly similar to ( 535) loc_os03g49570 12003.m09957 protein organic anion transporter, putative, expressed no original description 	T
30.2.17	signalling.receptor kinases.DUF 26	MDP0000131814	 highly similar to ( 694) AT5G63710 | Symbols:  | leucine-rich repeat transmembrane protein kinase, putative | chr5:25499475-25502598 FORWARDmoderately similar to ( 233) PSKR_DAUCA Phytosulfokine receptor precursor (EC 2.7.11.1) (Phytosulfokine LRR receptor kinase) - Daucus carota (Carrot)highly similar to ( 583) loc_os02g18320 12002.m07075 protein BRASSINOSTEROID INSENSITIVE 1-associated receptor kinase 1 precursor, putative, expressed no original description 	T
30.2.99	signalling.receptor kinases.misc	MDP0000131814	 highly similar to ( 694) AT5G63710 | Symbols:  | leucine-rich repeat transmembrane protein kinase, putative | chr5:25499475-25502598 FORWARDmoderately similar to ( 233) PSKR_DAUCA Phytosulfokine receptor precursor (EC 2.7.11.1) (Phytosulfokine LRR receptor kinase) - Daucus carota (Carrot)highly similar to ( 583) loc_os02g18320 12002.m07075 protein BRASSINOSTEROID INSENSITIVE 1-associated receptor kinase 1 precursor, putative, expressed no original description 	T
29.4.1.57	protein.postranslational modification.kinase.receptor like cytoplasmatic kinase VII	MDP0000131814	 highly similar to ( 694) AT5G63710 | Symbols:  | leucine-rich repeat transmembrane protein kinase, putative | chr5:25499475-25502598 FORWARDmoderately similar to ( 233) PSKR_DAUCA Phytosulfokine receptor precursor (EC 2.7.11.1) (Phytosulfokine LRR receptor kinase) - Daucus carota (Carrot)highly similar to ( 583) loc_os02g18320 12002.m07075 protein BRASSINOSTEROID INSENSITIVE 1-associated receptor kinase 1 precursor, putative, expressed no original description 	T
11.3	lipid metabolism.Phospholipid synthesis	MDP0000310886	 highly similar to ( 852) AT3G18000 | Symbols: NMT1, XPL1, PEAMT | XPL1 (XIPOTL 1); methyltransferase/ phosphoethanolamine N-methyltransferase | chr3:6154578-6157331 FORWARDhighly similar to ( 852) PEAM1_ARATH Phosphoethanolamine N-methyltransferase 1 (EC 2.1.1.103) (PEAMT 1) (AtNMT1) - Arabidopsis thaliana (Mouse-ear cress)highly similar to ( 825) loc_os01g50030 12001.m11187 protein phosphoethanolamine N-methyltransferase, putative, expressed  no original description 	T
29.5.11.3	protein.degradation.ubiquitin.E2	MDP0000897736	 moderately similar to ( 379) AT3G17000 | Symbols: UBC32 | UBC32 (ubiquitin-conjugating enzyme 32); ubiquitin-protein ligase | chr3:5797296-5799467 FORWARDvery weakly similar to (63.2) UBC2_WHEAT Ubiquitin-conjugating enzyme E2-17 kDa (EC 6.3.2.19) (Ubiquitin-protein ligase) (Ubiquitin carrier protein) - Triticum aestivum (Wheat)moderately similar to ( 337) loc_os03g19500 12003.m07367 protein ubiquitinating enzyme, putative, expressed  no original description 	T
31.4	cell.vesicle transport	MDP0000148979	 nearly identical (1316) AT4G31480 | Symbols:  | coatomer beta subunit, putative / beta-coat protein, putative / beta-COP, putative | chr4:15264076-15267384 FORWARDnearly identical (1253) loc_os11g07280 12011.m28658 protein coatomer subunit beta, putative, expressed  no original description 	T
26.12	misc.peroxidases	MDP0000233961	 moderately similar to ( 327) AT5G06720 | Symbols:  | peroxidase, putative | chr5:2077567-2078857 REVERSEmoderately similar to ( 282) PERX_TOBAC Lignin-forming anionic peroxidase precursor (EC 1.11.1.7) (TOPA) - Nicotiana tabacum (Common tobacco)moderately similar to ( 291) loc_os03g13200 12003.m06766 protein peroxidase 53 precursor, putative, expressed no original description 	T
35.1	not assigned.no ontology	MDP0000422717	 weakly similar to ( 182) AT1G11760 | Symbols:  | unknown protein | chr1:3972356-3972811 REVERSEweakly similar to ( 157) loc_os10g40070 12010.m06779 protein expressed protein no original description 	T
27.3.36	RNA.regulation of transcription.Argonaute	MDP0000774227	 nearly identical (1117) AT2G27880 | Symbols: AGO5 | AGO5 (ARGONAUTE 5); nucleic acid binding | chr2:11871488-11876712 FORWARDnearly identical (1072) loc_os03g58600 12003.m10765 protein argonaute-like protein, putative, expressed   no original description 	T
34.19.1	transport.Major Intrinsic Proteins.PIP	MDP0000736385	 weakly similar to ( 152) AT1G01620 | Symbols: PIP1C, TMP-B, PIP1;3 | PIP1C (PLASMA MEMBRANE INTRINSIC PROTEIN 1C); water channel | chr1:225986-226960 REVERSEweakly similar to ( 149) PIP11_ORYSA Aquaporin PIP1.1 (Plasma membrane intrinsic protein 1a) (PIP1a) (OsPIP1.1) (Water channel protein RWC1) (RWC-1) - Oryza sativa (Rice)weakly similar to ( 149) loc_os02g44630 12002.m33812 protein aquaporin PIP1.1, putative, expressed no original description 	T
20.2.2	stress.abiotic.cold	MDP0000155077	 weakly similar to ( 110) AT2G21060 | Symbols: ATGRP2B | ATGRP2B (GLYCINE-RICH PROTEIN 2B); DNA binding / nucleic acid binding / zinc ion binding | chr2:9036983-9037588 REVERSEweakly similar to ( 120) GRP2_NICSY Glycine-rich protein 2 - Nicotiana sylvestris (Wood tobacco)weakly similar to ( 108) loc_os02g02870 12002.m05637 protein glycine-rich protein 2, putative, expressed   no original description 	T
29.4	protein.postranslational modification	MDP0000267986	 nearly identical (1541) AT5G27030 | Symbols: TPR3 | TPR3 (TOPLESS-RELATED 3) | chr5:9508913-9515263 REVERSEnearly identical (1483) loc_os01g15020 12001.m42634 protein lissencephaly type-1-like homology motif, putative, expressed   no original description 	T
20.2.1	stress.abiotic.heat	MDP0000183178	 moderately similar to ( 213) AT5G54660 | Symbols:  | heat shock protein-related | chr5:22203950-22204986 FORWARDvery weakly similar to (56.6) HSP11_DAUCA 17.8 kDa class I heat shock protein (Clone DCHSP17.7) - Daucus carota (Carrot)weakly similar to ( 155) loc_os05g42120 12005.m08385 protein expressed protein no original description 	T
25.6	C1-metabolism.methylenetetrahydrofolate reductase	MDP0000280298	 nearly identical (1037) AT2G44160 | Symbols: MTHFR2 | MTHFR2 (METHYLENETETRAHYDROFOLATE REDUCTASE 2); methylenetetrahydrofolate reductase (NADPH) | chr2:18262301-18265185 FORWARDnearly identical (1028) MTHR_ORYSA Probable methylenetetrahydrofolate reductase (EC 1.5.1.20) - Oryza sativa (Rice)nearly identical (1028) loc_os03g60090 12003.m35475 protein methylenetetrahydrofolate reductase, putative, expressed   no original description 	T
26.12	misc.peroxidases	MDP0000282167	 moderately similar to ( 414) AT2G34060 | Symbols:  | peroxidase, putative | chr2:14384914-14386530 FORWARDweakly similar to ( 200) PER2_ARAHY Cationic peroxidase 2 precursor (EC 1.11.1.7) (PNPC2) - Arachis hypogaea (Peanut)moderately similar to ( 246) loc_os03g55410 12003.m10456 protein peroxidase 51 precursor, putative, expressed  no original description 	T
29.5.3	protein.degradation.cysteine protease	MDP0000287242	 moderately similar to ( 460) AT3G19390 | Symbols:  | cysteine proteinase, putative / thiol protease, putative | chr3:6723024-6724768 FORWARDmoderately similar to ( 447) ORYA_ORYSA Oryzain alpha chain precursor (EC 3.4.22.-) - Oryza sativa (Rice)moderately similar to ( 405) loc_os04g57440 12004.m101443 protein cysteine protease 1 precursor, putative, expressed   no original description 	T
34.18	transport.unspecified anions	MDP0000135067	 moderately similar to ( 267) AT5G62290 | Symbols:  | nucleotide-sensitive chloride conductance regulator (ICln) family protein | chr5:25019568-25020972 FORWARDmoderately similar to ( 203) loc_os10g08879 12010.m065232 protein methylosome subunit pICln, putative, expressed no original description 	T
29.5.11.4.3.2	protein.degradation.ubiquitin.E3.SCF.FBOX	MDP0000208090	 highly similar to ( 746) AT5G01720 | Symbols:  | F-box family protein (FBL3) | chr5:267118-270391 REVERSEhighly similar to ( 604) loc_os11g01780 12011.m04379 protein ubiquitin-protein ligase, putative, expressed   no original description 	T
27.4	RNA.RNA binding	MDP0000304758	 moderately similar to ( 273) AT1G14340 | Symbols:  | RNA recognition motif (RRM)-containing protein | chr1:4897709-4898776 FORWARDmoderately similar to ( 236) loc_os04g54870 12004.m10379 protein RNA recognition motif containing protein, putative, expressed no original description 	T
17.2.3	hormone metabolism.auxin.induced-regulated-responsive-activated	MDP0000786165	 weakly similar to ( 123) AT2G46690 | Symbols:  | auxin-responsive family protein | chr2:19180904-19181269 FORWARDvery weakly similar to (70.5) ARG7_PHAAU Indole-3-acetic acid-induced protein ARG7 - Phaseolus aureus (Mung bean) (Vigna radiata)very weakly similar to (85.1) loc_os06g50040 12006.m09528 protein OsSAUR29 - Auxin-responsive SAUR gene family member, expressed  no original description 	T
29.3.4.3	protein.targeting.secretory pathway.vacuole	MDP0000172014	 highly similar to ( 769) AT4G32940 | Symbols: GAMMA-VPE, GAMMAVPE | GAMMA-VPE (GAMMA VACUOLAR PROCESSING ENZYME); cysteine-type endopeptidase | chr4:15900557-15903161 REVERSEhighly similar to ( 821) VPE_CITSI Vacuolar-processing enzyme precursor (EC 3.4.22.-) (VPE) - Citrus sinensis (Sweet orange)highly similar to ( 710) loc_os01g37910 12001.m42664 protein vacuolar processing enzyme precursor, putative, expressed   no original description 	T
29.6	protein.folding	MDP0000330297	 highly similar to ( 940) AT3G02530 | Symbols:  | chaperonin, putative | chr3:528806-532457 REVERSEmoderately similar to ( 214) TCPE2_AVESA T-complex protein 1 subunit epsilon (TCP-1-epsilon) (CCT-epsilon) (TCP-K36) - Avena sativa (Oat)highly similar to ( 935) loc_os05g05470 12005.m05074 protein T-complex protein 1 subunit zeta, putative, expressed  no original description 	T
29.2.1.2.1.4	protein.synthesis.ribosomal protein.eukaryotic.40S subunit.S4	MDP0000303283	 moderately similar to ( 449) AT5G58420 | Symbols:  | 40S ribosomal protein S4 (RPS4D) | chr5:23619599-23620896 FORWARDmoderately similar to ( 446) RS4_SOLTU 40S ribosomal protein S4 - Solanum tuberosum (Potato)moderately similar to ( 442) loc_os02g01560 12002.m05506 protein 40S ribosomal protein S4, putative, expressed  no original description 	T
35.2	not assigned.unknown	MDP0000770211	 moderately similar to ( 272) AT1G44960 | Symbols:  | INVOLVED IN: biological_process unknown; LOCATED IN: plasma membrane; EXPRESSED IN: 25 plant structures; EXPRESSED DURING: 15 growth stages; CONTAINS InterPro DOMAIN/s: SNARE associated Golgi protein (InterPro:IPR015414); BEST Arabidopsis thaliana protein match is: unknown protein (TAIR:AT1G03260.1); Has 947 Blast hits to 946 proteins in 318 species: Archae - 2; Bacteria - 660; Metazoa - 2; Fungi - 0; Plants - 77; Viruses - 0; Other Eukaryotes - 206 (source: NCBI BLink). | chr1:16999576-17001447 FORWARDmoderately similar to ( 235) loc_os11g33100 12011.m07211 protein dedA, putative, expressed no original description 	T
35.2	not assigned.unknown	MDP0000344010	  no original description 	T
11.2.1	lipid metabolism.FA desaturation.desaturase	MDP0000225067	 moderately similar to ( 287) AT2G31360 | Symbols: ADS2 | ADS2 (16:0Delta9 Arabidopsis desaturase 2); oxidoreductase | chr2:13372121-13373546 REVERSE  no original description 	T
27.1	RNA.processing	MDP0000143473	 moderately similar to ( 343) AT1G08370 | Symbols: DCP1, ATDCP1 | DCP1 (decapping 1); m7G(5')pppN diphosphatase/ protein homodimerization | chr1:2638355-2640367 FORWARDmoderately similar to ( 325) loc_os11g05650 12011.m04762 protein dcp1-like decapping family protein, expressed   no original description 	T
30.2.17	signalling.receptor kinases.DUF 26	MDP0000291837	 very weakly similar to (78.2) AT1G70530 | Symbols:  | protein kinase family protein | chr1:26588750-26591379 REVERSEvery weakly similar to (65.5) loc_os01g36790 12001.m09975 protein protein kinase, putative, expressed  TrbL  no original description 	T
33.99	development.unspecified	MDP0000151144	 moderately similar to ( 370) AT4G00150 | Symbols:  | scarecrow-like transcription factor 6 (SCL6) | chr4:57429-59105 REVERSEweakly similar to ( 120) RGA1_BRACM DELLA protein RGA1 (RGA-like protein 1) (BrRGA1) - Brassica campestris (Field mustard)moderately similar to ( 287) loc_os04g46860 12004.m09634 protein scarecrow-like 6, putative, expressed  no original description 	T
29.5.1	protein.degradation.subtilases	MDP0000245037	 moderately similar to ( 243) AT5G67090 | Symbols:  | subtilase family protein | chr5:26774111-26776321 REVERSEmoderately similar to ( 363) loc_os01g64850 12001.m12598 protein subtilisin-like protease precursor, putative   no original description 	T
29.4.1.57	protein.postranslational modification.kinase.receptor like cytoplasmatic kinase VII	MDP0000181884	 highly similar to ( 555) AT1G07870 | Symbols:  | protein kinase family protein | chr1:2429933-2431843 REVERSEmoderately similar to ( 226) NORK_MEDTR Nodulation receptor kinase precursor (EC 2.7.11.1) (Does not make infections protein 2) (Symbiosis receptor-like kinase) (MtSYMRK) - Medicago truncatula (Barrel medic)highly similar to ( 503) loc_os07g49470 12007.m09155 protein protein kinase APK1B, chloroplast precursor, putative, expressed  no original description 	T
15.2	metal handling.binding, chelation and storage	MDP0000886675	 weakly similar to ( 191) AT5G60800 | Symbols:  | heavy-metal-associated domain-containing protein | chr5:24461116-24462464 REVERSEweakly similar to ( 120) loc_os10g30450 12010.m05907 protein metal ion binding protein, putative, expressed  COG4907  no original description 	T
35.2	not assigned.unknown	MDP0000479961	 moderately similar to ( 343) AT3G07310 | Symbols:  | unknown protein | chr3:2328057-2329677 REVERSEmoderately similar to ( 253) loc_os11g26890 12011.m28798 protein expressed protein   no original description 	T
35.1	not assigned.no ontology	MDP0000942678	 highly similar to ( 897) AT1G21680 | Symbols:  | FUNCTIONS IN: molecular_function unknown; INVOLVED IN: biological_process unknown; LOCATED IN: plasma membrane, vacuole, plant-type cell wall; EXPRESSED IN: 25 plant structures; EXPRESSED DURING: 13 growth stages; CONTAINS InterPro DOMAIN/s: WD40-like Beta Propeller (InterPro:IPR011659), Six-bladed beta-propeller, TolB-like (InterPro:IPR011042); BEST Arabidopsis thaliana protein match is: unknown protein (TAIR:AT1G21670.1); Has 6869 Blast hits to 3873 proteins in 790 species: Archae - 41; Bacteria - 3697; Metazoa - 41; Fungi - 39; Plants - 64; Viruses - 0; Other Eukaryotes - 2987 (source: NCBI BLink). | chr1:7613028-7615148 FORWARDhighly similar to ( 736) loc_os03g62370 12003.m11113 protein WD40-like Beta Propeller Repeat family protein, expressed   no original description 	T
35.2	not assigned.unknown	MDP0000284624	 moderately similar to ( 297) AT1G01360 | Symbols:  | unknown protein | chr1:142138-142914 FORWARDmoderately similar to ( 301) loc_os02g15640 12002.m06807 protein AT-rich element binding factor 3, putative, expressed no original description 	T
35.2	not assigned.unknown	MDP0000332474	  no original description 	T
3.6	minor CHO metabolism.callose	MDP0000212172	 nearly identical (1832) AT2G36850 | Symbols: ATGSL08, GSL8, GSL08, ATGSL8 | GSL8 (GLUCAN SYNTHASE-LIKE 8); 1,3-beta-glucan synthase/ transferase, transferring glycosyl groups | chr2:15454935-15469666 REVERSEnearly identical (1201) loc_os06g08380 12006.m05559 protein beta-1,3-glucan synthase, putative, expressed  no original description 	T
35.2	not assigned.unknown	MDP0000250869	 very weakly similar to (56.6) AT1G23530 | Symbols:  | unknown protein | chr1:8345861-8346430 FORWARD no original description 	T
35.2	not assigned.unknown	MDP0000914155	 weakly similar to ( 133) AT5G06790 | Symbols:  | unknown protein | chr5:2098088-2098717 REVERSEvery weakly similar to (89.0) loc_os09g10730 12009.m04337 protein expressed protein  no original description 	T
17.2.3	hormone metabolism.auxin.induced-regulated-responsive-activated	MDP0000737171	 weakly similar to ( 178) AT4G12980 | Symbols:  | auxin-responsive protein, putative | chr4:7589670-7591074 REVERSEweakly similar to ( 137) loc_os08g41290 12008.m08107 protein AIR12, putative, expressed   no original description 	T
33.3	development.squamosa promoter binding like (SPL)	MDP0000155354	 moderately similar to ( 239) AT5G43270 | Symbols: SPL2 | SPL2 (SQUAMOSA PROMOTER BINDING PROTEIN-LIKE 2); DNA binding / transcription factor | chr5:17360527-17362143 REVERSEweakly similar to ( 112) LG1_MAIZE LIGULELESS1 protein - Zea mays (Maize)moderately similar to ( 241) loc_os06g49010 12006.m091569 protein squamosa promoter-binding-like protein 11, putative, expressed   no original description 	T
27.4	RNA.RNA binding	MDP0000260868	 moderately similar to ( 240) AT1G51580 | Symbols:  | KH domain-containing protein | chr1:19125612-19128102 FORWARDmoderately similar to ( 405) loc_os01g13470 12001.m07956 protein nucleic acid binding protein, putative, expressed   no original description 	T
10.1.3	cell wall.precursor synthesis.AXS	MDP0000723073	 highly similar to ( 711) AT1G08200 | Symbols: AXS2 | AXS2 (UDP-D-APIOSE/UDP-D-XYLOSE SYNTHASE 2); UDP-glucuronate decarboxylase | chr1:2574259-2576609 REVERSEvery weakly similar to (57.0) DFRA_DIACA Dihydroflavonol-4-reductase (EC 1.1.1.219) (DFR) (Dihydrokaempferol 4-reductase) - Dianthus caryophyllus (Carnation) (Clove pink)highly similar to ( 673) loc_os01g73790 12001.m13406 protein bifunctional polymyxin resistance arnA protein, putative, expressed   no original description 	T
1.1.40	PS.lightreaction.cyclic electron flow-chlororespiration	MDP0000131537	 moderately similar to ( 442) AT1G64770 | Symbols: NDF2 | NDF2 (NDH-DEPENDENT CYCLIC ELECTRON FLOW 1) | chr1:24057549-24058565 FORWARDmoderately similar to ( 390) loc_os03g06230 12003.m06143 protein expressed protein  no original description 	T
34.99	transport.misc	MDP0000259550	 moderately similar to ( 363) AT3G23560 | Symbols: ALF5 | ALF5 (ABERRANT LATERAL ROOT FORMATION 5); antiporter/ drug transporter/ transporter | chr3:8454361-8456588 REVERSEmoderately similar to ( 332) loc_os07g31884 12007.m07454 protein transparent testa 12 protein, putative, expressed no original description 	T
35.2	not assigned.unknown	MDP0000508254	 very weakly similar to (91.7) AT4G34560 | Symbols:  | unknown protein | chr4:16507923-16508588 FORWARD no original description 	T
35.2	not assigned.unknown	MDP0000168886	 weakly similar to ( 186) AT2G45980 | Symbols:  | unknown protein | chr2:18917680-18918569 REVERSEweakly similar to ( 124) loc_os04g08310 12004.m06120 protein expressed protein  no original description 	T
16.1.1.5	secondary metabolism.isoprenoids.non-mevalonate pathway.MCS	MDP0000255398	 weakly similar to ( 190) AT1G63970 | Symbols: ISPF, MECPS | ISPF; 2-C-methyl-D-erythritol 2,4-cyclodiphosphate synthase | chr1:23738923-23740336 REVERSEweakly similar to ( 186) ISPF_CATRO 2-C-methyl-D-erythritol 2,4-cyclodiphosphate synthase, chloroplast precursor (EC 4.6.1.12) (MECPS) (MECDP-synthase) - Catharanthus roseus (Rosy periwinkle) (Madagascar periwinkle)weakly similar to ( 174) loc_os02g45660 12002.m09603 protein 2-C-methyl-D-erythritol 2,4-cyclodiphosphate synthase, chloroplast precursor, putative, expressed  no original description 	T
21.1	redox.thioredoxin	MDP0000668704	 weakly similar to ( 181) AT1G07700 | Symbols:  | thioredoxin family protein | chr1:2380297-2381127 FORWARDvery weakly similar to (54.3) TRXH_ORYSA Thioredoxin H-type (TRX-H) (Phloem sap 13 kDa protein 1) - Oryza sativa (Rice)weakly similar to ( 186) loc_os02g35900 12002.m100241 protein thioredoxin-like 2, chloroplast precursor, putative, expressed   no original description 	T
29.2.1.2.2.31	protein.synthesis.ribosomal protein.eukaryotic.60S subunit.L31	MDP0000134646	 weakly similar to ( 179) AT2G19740 | Symbols:  | 60S ribosomal protein L31 (RPL31A) | chr2:8513577-8514346 FORWARDweakly similar to ( 180) RL31_PANGI 60S ribosomal protein L31 - Panax ginseng (Korean ginseng)weakly similar to ( 171) loc_os02g48660 12002.m09900 protein 60S ribosomal protein L31, putative, expressed no original description 	T
29.7	protein.glycosylation	MDP0000864705	 highly similar to ( 756) AT1G76400 | Symbols:  | ribophorin I family protein | chr1:28658713-28661672 REVERSEhighly similar to ( 744) loc_os05g23600 12005.m27570 protein dolichyl-diphosphooligosaccharide--protein glycosyltransferase 67 kDasubunit precursor, putative, expressed no original description 	T
26.22	misc.short chain dehydrogenase/reductase (SDR)	MDP0000304669	 highly similar to ( 585) AT5G14060 | Symbols: CARAB-AK-LYS | CARAB-AK-LYS; amino acid binding / aspartate kinase | chr5:4536143-4538993 FORWARDmoderately similar to ( 224) TS2_MAIZE Sex determination protein tasselseed-2 - Zea mays (Maize)highly similar to ( 599) loc_os07g20544 12007.m29379 protein lysine-sensitive aspartokinase 3, putative, expressed   TrbL Sporozoite_P67  no original description 	T
35.2	not assigned.unknown	MDP0000145229	 weakly similar to ( 162) AT3G25400 | Symbols:  | FUNCTIONS IN: molecular_function unknown; INVOLVED IN: biological_process unknown; LOCATED IN: cellular_component unknown; EXPRESSED IN: 11 plant structures; EXPRESSED DURING: 6 growth stages; CONTAINS InterPro DOMAIN/s: NTP Pyrophosphohydrolase MazG-related, RS21-C6 (InterPro:IPR011394), EAR (InterPro:IPR009039), NTP pyrophosphohydrolase MazG, putative catalytic core (InterPro:IPR004518); Has 572 Blast hits to 572 proteins in 181 species: Archae - 11; Bacteria - 286; Metazoa - 71; Fungi - 1; Plants - 31; Viruses - 0; Other Eukaryotes - 172 (source: NCBI BLink). | chr3:9213236-9214144 FORWARDweakly similar to ( 157) loc_os02g27810 12002.m07925 protein RS21-C6 protein, putative, expressed no original description 	T
27.3.99	RNA.regulation of transcription.unclassified	MDP0000902760	 highly similar to ( 511) AT3G52500 | Symbols:  | aspartyl protease family protein | chr3:19465644-19467053 REVERSEvery weakly similar to (55.5) 7SBG2_SOYBN Basic 7S globulin 2 precursor (Bg) (SBg7S) [Contains: Basic 7S globulin 2 high kDa subunit; Basic 7S globulin 2 low kDa subunit] - Glycine max (Soybean)moderately similar to ( 325) loc_os04g58840 12004.m10764 protein aspartic proteinase nepenthesin-2 precursor, putative, expressed  PRK08026  no original description 	T
22.2.1	polyamine metabolism.degradation.polyamin oxidase	MDP0000694227	 highly similar to ( 890) AT3G57420 | Symbols:  | unknown protein | chr3:21252605-21255115 REVERSEweakly similar to ( 119) PAO_MAIZE Polyamine oxidase precursor (EC 1.5.3.11) - Zea mays (Maize)highly similar to ( 808) loc_os05g32500 12005.m27810 protein expressed protein   no original description 	T
1.2.6	PS.photorespiration.hydroxypyruvate reductase	MDP0000321479	 moderately similar to ( 492) AT1G79870 | Symbols:  | oxidoreductase family protein | chr1:30044794-30045851 FORWARDweakly similar to ( 133) DHGY_CUCSA Glycerate dehydrogenase (EC 1.1.1.29) (NADH-dependent hydroxypyruvate reductase) (HPR) (GDH) - Cucumis sativus (Cucumber)moderately similar to ( 422) loc_os01g12830 12001.m43412 protein glyoxylate reductase, putative, expressed   no original description 	T
29.6	protein.folding	MDP0000685137	 moderately similar to ( 365) AT5G20720 | Symbols: CPN20, CPN10, CHCPN10, ATCPN21, CPN21 | CPN20 (CHAPERONIN 20); calmodulin binding | chr5:7015015-7016354 FORWARDmoderately similar to ( 317) CH10C_SPIOL 20 kDa chaperonin, chloroplast precursor (Protein Cpn21) (Chloroplast protein Cpn10) (Chloroplast chaperonin 10) (Ch-CPN10) - Spinacia oleracea (Spinach)moderately similar to ( 330) loc_os06g09679 12006.m31856 protein chaperonin, chloroplast precursor, putative, expressed  no original description 	T
35.2	not assigned.unknown	MDP0000290971	 moderately similar to ( 369) AT5G39590 | Symbols:  | FUNCTIONS IN: molecular_function unknown; INVOLVED IN: N-terminal protein myristoylation; LOCATED IN: cellular_component unknown; EXPRESSED IN: 24 plant structures; EXPRESSED DURING: 15 growth stages; CONTAINS InterPro DOMAIN/s: TLDc (InterPro:IPR006571); Has 144 Blast hits to 144 proteins in 62 species: Archae - 0; Bacteria - 0; Metazoa - 76; Fungi - 9; Plants - 26; Viruses - 0; Other Eukaryotes - 33 (source: NCBI BLink). | chr5:15851483-15853768 FORWARDmoderately similar to ( 234) loc_os04g56790 12004.m10566 protein TLD family protein, expressed  no original description 	T
35.2	not assigned.unknown	MDP0000135149	 very weakly similar to (97.8) AT5G02020 | Symbols:  | unknown protein | chr5:386999-387921 REVERSE no original description 	T
27.3.38	RNA.regulation of transcription.AT-rich interaction domain containing transcription factor family	MDP0000186031	 very weakly similar to (94.0) AT1G76510 | Symbols:  | ARID/BRIGHT DNA-binding domain-containing protein | chr1:28708927-28712498 REVERSEvery weakly similar to (89.4) loc_os06g41730 12006.m31901 protein ARID/BRIGHT DNA binding domain containing protein, expressed  no original description 	T
35.2	not assigned.unknown	MDP0000166591	 very weakly similar to (77.4) AT2G21180 | Symbols:  | unknown protein | chr2:9074956-9075351 REVERSEvery weakly similar to (80.5) loc_os03g14570 12003.m101273 protein expressed protein no original description 	T
20.1	stress.biotic	MDP0000295390	 moderately similar to ( 236) AT4G25780 | Symbols:  | pathogenesis-related protein, putative | chr4:13121107-13121679 FORWARDweakly similar to ( 177) PR1_MEDTR Pathogenesis-related protein PR-1 precursor - Medicago truncatula (Barrel medic)moderately similar to ( 225) loc_os02g54560 12002.m10485 protein pathogenesis-related protein PR-1 precursor, putative, expressed  no original description 	T
35.2	not assigned.unknown	MDP0000234690	 weakly similar to ( 137) AT5G52900 | Symbols:  | unknown protein | chr5:21452893-21453692 REVERSE  no original description 	T
29.5.11.4.3.2	protein.degradation.ubiquitin.E3.SCF.FBOX	MDP0000283767	 highly similar to ( 755) AT3G23790 | Symbols:  | AMP-binding protein, putative | chr3:8575268-8581001 FORWARDweakly similar to ( 109) 4CL1_PETCR 4-coumarate--CoA ligase 1 (EC 6.2.1.12) (4CL 1) (4-coumaroyl-CoA synthase 1) - Petroselinum crispum (Parsley) (Petroselinum hortense)highly similar to ( 702) loc_os03g62850 12003.m35526 protein acyl-CoA synthetase-like protein, putative, expressed  no original description 	T
1.1.1.1	PS.lightreaction.photosystem II.LHC-II	MDP0000193128	 moderately similar to ( 422) AT3G47470 | Symbols: LHCA4, CAB4 | LHCA4 (LIGHT-HARVESTING CHLOROPHYLL-PROTEIN COMPLEX I SUBUNIT A4); chlorophyll binding | chr3:17493622-17494773 REVERSEmoderately similar to ( 221) CB12_PETHY Chlorophyll a-b binding protein, chloroplast precursor (LHCI type II CAB) - Petunia hybrida (Petunia)moderately similar to ( 382) loc_os08g33820 12008.m080044 protein chlorophyll a-b binding protein 4, chloroplast precursor, putative, expressed  no original description 	T
13.1.3.4.12	amino acid metabolism.synthesis.aspartate family.methionine.homocysteine S-methyltransferase 	MDP0000129728	 moderately similar to ( 270) AT1G69523 | Symbols:  | UbiE/COQ5 methyltransferase family protein | chr1:26129827-26131157 FORWARDmoderately similar to ( 275) loc_os02g51030 12002.m10137 protein S-adenosylmethionine-dependent methyltransferase/ methyltransferase, putative, expressed  no original description 	T
9.7	mitochondrial electron transport / ATP synthesis.cytochrome c oxidase	MDP0000174368	 weakly similar to ( 149) AT3G15640 | Symbols:  | cytochrome c oxidase family protein | chr3:5299273-5301371 FORWARDweakly similar to ( 142) loc_os06g05080 12006.m05237 protein cytochrome c oxidase polypeptide Vb, mitochondrial precursor, putative, expressed  no original description 	T
26.21	misc.protease inhibitor/seed storage/lipid transfer protein (LTP) family protein	MDP0000880401	 very weakly similar to (85.5) AT5G48485 | Symbols: DIR1 | DIR1 (DEFECTIVE IN INDUCED RESISTANCE 1); lipid binding / lipid transporter | chr5:19646317-19646625 REVERSEvery weakly similar to (94.4) loc_os07g18750 12007.m06314 protein PVR3-like protein, putative, expressed  no original description 	T
26.10	misc.cytochrome P450	MDP0000156544	 highly similar to ( 606) AT4G12300 | Symbols: CYP706A4 | CYP706A4; electron carrier/ heme binding / iron ion binding / monooxygenase/ oxygen binding | chr4:7308016-7309692 REVERSEmoderately similar to ( 334) F3PH_PETHY Flavonoid 3'-monooxygenase (EC 1.14.13.21) (Flavonoid 3'-hydroxylase) (Cytochrome P450 75B2) - Petunia hybrida (Petunia)highly similar to ( 543) loc_os08g43440 12008.m08318 protein flavonoid 3-monooxygenase, putative, expressed    TrbL  no original description 	T
27.3.8	RNA.regulation of transcription.C2C2(Zn) DOF zinc finger family	MDP0000255023	 moderately similar to ( 225) AT5G62940 | Symbols:  | Dof-type zinc finger domain-containing protein | chr5:25257183-25258504 REVERSEweakly similar to ( 133) PBF_MAIZE Dof zinc finger protein PBF (Prolamin box-binding factor) - Zea mays (Maize)weakly similar to ( 172) loc_os02g47810 12002.m09815 protein dof domain, zinc finger family protein, expressed  PRK08026  no original description 	T
29.5.11.1	protein.degradation.ubiquitin.ubiquitin	MDP0000291439	 moderately similar to ( 403) AT5G38470 | Symbols:  | DNA repair protein RAD23, putative | chr5:15404720-15407251 FORWARDmoderately similar to ( 319) RAD23_ORYSA Probable DNA repair protein RAD23 (OsRAD23) - Oryza sativa (Rice)moderately similar to ( 349) loc_os06g15360 12006.m091670 protein DNA repair protein RAD23, putative, expressed   no original description 	T
16.1.3.3	secondary metabolism.isoprenoids.tocopherol biosynthesis.MPBQ/MSBQ methyltransferase	MDP0000516003	 moderately similar to ( 473) AT3G63410 | Symbols: APG1, VTE3, IEP37, E37 | APG1 (ALBINO OR PALE GREEN MUTANT 1); 2-methyl-6-phytyl-1,4-benzoquinone methyltransferase/ S-adenosylmethionine-dependent methyltransferase/ methyltransferase | chr3:23415816-23417002 REVERSEmoderately similar to ( 480) IN37_SPIOL 37 kDa inner envelope membrane protein, chloroplast precursor (E37) - Spinacia oleracea (Spinach)moderately similar to ( 461) loc_os07g08200 12007.m05277 protein inner envelope membrane protein, chloroplast precursor, putative  no original description 	T
35.2	not assigned.unknown	MDP0000191282	 moderately similar to ( 237) AT4G01995 | Symbols:  | unknown protein | chr4:873075-874619 FORWARDmoderately similar to ( 231) loc_os05g04070 12005.m27459 protein expressed protein   no original description 	T
26.18	misc.invertase/pectin methylesterase inhibitor family protein	MDP0000316607	 weakly similar to ( 125) AT5G38610 | Symbols:  | invertase/pectin methylesterase inhibitor family protein | chr5:15460845-15461444 REVERSEvery weakly similar to (68.2) PMEI_ACTCH Pectinesterase inhibitor (Pectin methylesterase inhibitor) (PMEI) - Actinidia chinensis (Kiwi) (Yangtao)very weakly similar to (65.5) loc_os02g01300 12002.m05480 protein invertase inhibitor precursor, putative, expressed  no original description 	T
35.2	not assigned.unknown	MDP0000555589	 very weakly similar to (88.6) PPO_MALDO Polyphenol oxidase, chloroplast precursor (EC 1.10.3.1) (PPO) (Catechol oxidase) - Malus domestica (Apple) (Malus sylvestris)very weakly similar to (57.0) loc_os01g58100 12001.m11955 protein polyphenol oxidase, chloroplast precursor, putative, expressed  no original description 	T
35.2	not assigned.unknown	MDP0000217331	 moderately similar to ( 226) AT5G47310 | Symbols:  | unknown protein | chr5:19201325-19202674 FORWARDmoderately similar to ( 236) loc_os06g36490 12006.m08185 protein EREBP-4 like protein, putative, expressed no original description 	T
35.2	not assigned.unknown	MDP0000203679	 highly similar to ( 741) AT3G26410 | Symbols:  | methyltransferase/ nucleic acid binding | chr3:9669508-9671273 REVERSEhighly similar to ( 727) loc_os02g35060 12002.m08594 protein RNA methylase, putative, expressed  no original description 	T
33.99	development.unspecified	MDP0000147080	 moderately similar to ( 237) AT5G13180 | Symbols: ANAC083 | ANAC083 (ARABIDOPSIS NAC DOMAIN CONTAINING PROTEIN 83); transcription factor | chr5:4196643-4197577 FORWARDweakly similar to ( 155) NAC68_ORYSA NAC domain-containing protein 68 (ONAC068) - Oryza sativa (Rice)weakly similar to ( 164) loc_os11g03300 12011.m04526 protein NAC domain transcription factor, putative, expressed no original description 	T
35.2	not assigned.unknown	MDP0000218971	 very weakly similar to (83.2) AT3G09110 | Symbols:  | unknown protein | chr3:2794850-2795963 REVERSEweakly similar to ( 174) loc_os01g06030 12001.m07234 protein conserved hypothetical protein   no original description 	T
20.1.7	stress.biotic.PR-proteins	MDP0000280403	 moderately similar to ( 415) AT1G61100 | Symbols:  | disease resistance protein (TIR class), putative | chr1:22508831-22512122 REVERSEmoderately similar to ( 223) loc_os07g26440 12007.m079669 protein CIP7, putative, expressed MSP1_C  no original description 	T
31.1	cell.organisation	MDP0000280403	 moderately similar to ( 415) AT1G61100 | Symbols:  | disease resistance protein (TIR class), putative | chr1:22508831-22512122 REVERSEmoderately similar to ( 223) loc_os07g26440 12007.m079669 protein CIP7, putative, expressed MSP1_C  no original description 	T
20.2.99	stress.abiotic.unspecified	MDP0000870405	 weakly similar to ( 129) AT5G15780 | Symbols:  | pollen Ole e 1 allergen and extensin family protein | chr5:5144898-5146297 REVERSEvery weakly similar to (54.3) loc_os10g13850 12010.m04638 protein expressed protein  no original description 	T
35.2	not assigned.unknown	MDP0000290153	 weakly similar to ( 200) AT5G01120 | Symbols:  | unknown protein | chr5:45281-46986 FORWARDweakly similar to ( 170) loc_os04g50800 12004.m09977 protein expressed protein  no original description 	T
10.8.2	cell wall.pectin*esterases.acetyl esterase	MDP0000258999	 weakly similar to ( 198) AT5G26670 | Symbols:  | pectinacetylesterase, putative | chr5:9319096-9320816 FORWARDweakly similar to ( 190) loc_os07g44070 12007.m29010 protein carboxylic ester hydrolase, putative, expressed  no original description 	T
35.1	not assigned.no ontology	MDP0000174318	 weakly similar to ( 169) AT3G29760 | Symbols:  | NLI interacting factor (NIF) family protein | chr3:11590064-11591833 REVERSEmoderately similar to ( 207) loc_os07g01850 12007.m04663 protein NLI interacting factor-like phosphatase family protein, expressed no original description 	T
35.2	not assigned.unknown	MDP0000806558	  no original description 	T
25.8	C1-metabolism.tetrahydrofolate synthase	MDP0000241549	 moderately similar to ( 499) AT5G05980 | Symbols: ATDFB | ATDFB (A. THALIANA DHFS-FPGS HOMOLOG B); tetrahydrofolylpolyglutamate synthase | chr5:1799738-1804177 REVERSEmoderately similar to ( 462) loc_os03g02030 12003.m101150 protein folylpolyglutamate synthase, mitochondrial precursor, putative, expressed   no original description 	T
15.2	metal handling.binding, chelation and storage	MDP0000741407	 weakly similar to ( 125) AT3G06130 | Symbols:  | heavy-metal-associated domain-containing protein | chr3:1853048-1854673 REVERSEweakly similar to ( 109) loc_os12g05040 12012.m073762 protein metal ion binding protein, putative, expressed  COG4907  no original description 	T
35.1	not assigned.no ontology	MDP0000304679	 moderately similar to ( 472) AT1G16180 | Symbols:  | TMS membrane family protein / tumour differentially expressed (TDE) family protein | chr1:5540905-5542670 FORWARDmoderately similar to ( 438) loc_os02g54990 12002.m10526 protein serine incorporator 3, putative, expressed   no original description 	T
29.2.1.2.2.30	protein.synthesis.ribosomal protein.eukaryotic.60S subunit.L30	MDP0000329639	 weakly similar to ( 193) AT1G77940 | Symbols:  | 60S ribosomal protein L30 (RPL30B) | chr1:29304116-29305288 REVERSEmoderately similar to ( 202) RL30_LUPLU 60S ribosomal protein L30 - Lupinus luteus (European yellow lupin)weakly similar to ( 178) loc_os01g16890 12001.m42968 protein 60S ribosomal protein L30, putative, expressed no original description 	T
16.2.1.3	secondary metabolism.phenylpropanoids.lignin biosynthesis.4CL	MDP0000277093	 highly similar to ( 754) AT4G05160 | Symbols:  | 4-coumarate--CoA ligase, putative / 4-coumaroyl-CoA synthase, putative | chr4:2664451-2666547 FORWARDmoderately similar to ( 391) 4CL2_SOYBN 4-coumarate--CoA ligase 2 (EC 6.2.1.12) (4CL 2) (4-coumaroyl-CoA synthase 2) (Clone 4CL16) - Glycine max (Soybean)highly similar to ( 572) loc_os03g05780 12003.m06100 protein 4-coumarate--CoA ligase 2, putative, expressed   no original description 	T
35.2	not assigned.unknown	MDP0000795821	 highly similar to ( 664) AT3G14920 | Symbols:  | unknown protein | chr3:5018275-5020104 FORWARDhighly similar to ( 570) loc_os01g10930 12001.m07713 protein peptide-N4-asparagine amidase A, putative, expressed  no original description 	T
29.3.4.4	protein.targeting.secretory pathway.plasma membrane	MDP0000314394	 highly similar to ( 944) AT2G17980 | Symbols: ATSLY1 | ATSLY1; protein transporter | chr2:7824352-7826404 FORWARDhighly similar to ( 701) SLY1_ORYSA SEC1-family transport protein SLY1 - Oryza sativa (Rice)highly similar to ( 701) loc_os03g42320 12003.m09290 protein SEC1-family transport protein SLY1, putative, expressed   no original description 	T
35.2	not assigned.unknown	MDP0000157736	 very weakly similar to (88.2) AT2G35470 | Symbols:  | unknown protein | chr2:14909014-14909720 REVERSE  no original description 	T
30.2.24	signalling.receptor kinases.S-locus glycoprotein like	MDP0000817733	 moderately similar to ( 452) AT1G78850 | Symbols:  | curculin-like (mannose-binding) lectin family protein | chr1:29642072-29643397 REVERSEmoderately similar to ( 425) EP1G_DAUCA Epidermis-specific secreted glycoprotein EP1 precursor (52/54 kDa medium protein) - Daucus carota (Carrot)weakly similar to ( 144) loc_os04g23760 12004.m07515 protein ATP binding protein, putative  no original description 	T
34.9	transport.metabolite transporters at the mitochondrial membrane	MDP0000286751	 moderately similar to ( 407) AT1G07030 | Symbols:  | mitochondrial substrate carrier family protein | chr1:2158631-2160524 REVERSEvery weakly similar to (88.2) BT1_MAIZE Protein brittle-1, chloroplast precursor - Zea mays (Maize)moderately similar to ( 353) loc_os03g18550 12003.m34847 protein mitochondrial RNA-splicing protein MRS3, putative, expressed  no original description 	T
29.5.11.4.3.2	protein.degradation.ubiquitin.E3.SCF.FBOX	MDP0000436064	 weakly similar to ( 119) AT3G16210 | Symbols:  | F-box family protein | chr3:5494533-5495615 REVERSEvery weakly similar to (92.0) loc_os12g06740 12012.m26845 protein F-box protein interaction domain containing protein, expressed  no original description 	T
29.2.1.2.2.82	protein.synthesis.ribosomal protein.eukaryotic.60S subunit.P2	MDP0000361422	 very weakly similar to (52.0) AT3G44590 | Symbols:  | 60S acidic ribosomal protein P2 (RPP2D) | chr3:16163983-16164658 FORWARD no original description 	T
20.1.7	stress.biotic.PR-proteins	MDP0000173893	 moderately similar to ( 480) AT5G36930 | Symbols:  | disease resistance protein (TIR-NBS-LRR class), putative | chr5:14567771-14571916 REVERSEvery weakly similar to (81.3) loc_os12g29280 12012.m06742 protein disease resistance protein RGA3, putative, expressed  no original description 	T
27.3.99	RNA.regulation of transcription.unclassified	MDP0000292844	 moderately similar to ( 201) AT1G51200 | Symbols:  | zinc finger (AN1-like) family protein | chr1:18985690-18986211 FORWARDweakly similar to ( 120) ISAP1_ORYSA Multiple stress-responsive zinc-finger protein ISAP1 (Stress-associated protein 1) (OsISAP1) - Oryza sativa (Rice)moderately similar to ( 221) loc_os06g41010 12006.m31898 protein zinc finger A20 and AN1 domains-containing protein, putative, expressed   no original description 	T
35.2	not assigned.unknown	MDP0000290858	 very weakly similar to (94.7) AT5G56550 | Symbols: OXS3, ATOXS3 | OXS3 (OXIDATIVE STRESS 3) | chr5:22895847-22896502 REVERSEvery weakly similar to (85.5) loc_os02g45930 12002.m09630 protein expressed protein no original description 	T
13.1.4.1	amino acid metabolism.synthesis.branched chain group.common	MDP0000293019	 moderately similar to ( 234) AT3G58610 | Symbols:  | ketol-acid reductoisomerase | chr3:21671561-21674639 FORWARDmoderately similar to ( 229) ILV5_PEA Ketol-acid reductoisomerase, chloroplast precursor (EC 1.1.1.86) (Acetohydroxy-acid reductoisomerase) (Alpha-keto-beta-hydroxylacil reductoisomerase) - Pisum sativum (Garden pea)moderately similar to ( 211) loc_os05g49800 12005.m09050 protein ketol-acid reductoisomerase, chloroplast precursor, putative, expressed  no original description 	T
30.3	signalling.calcium	MDP0000211258	 weakly similar to ( 127) AT2G38800 | Symbols:  | calmodulin-binding protein-related | chr2:16216999-16218837 FORWARDweakly similar to ( 119) loc_os05g31690 12005.m07403 protein expressed protein  no original description 	T
30.2.17	signalling.receptor kinases.DUF 26	MDP0000263659	 moderately similar to ( 251) AT5G03140 | Symbols:  | lectin protein kinase family protein | chr5:737750-739885 REVERSEweakly similar to ( 103) PSKR_DAUCA Phytosulfokine receptor precursor (EC 2.7.11.1) (Phytosulfokine LRR receptor kinase) - Daucus carota (Carrot)moderately similar to ( 253) loc_os06g17490 12006.m06461 protein protein kinase, putative, expressed   Sporozoite_P67  no original description 	T
26.16	misc.myrosinases-lectin-jacalin	MDP0000263659	 moderately similar to ( 251) AT5G03140 | Symbols:  | lectin protein kinase family protein | chr5:737750-739885 REVERSEweakly similar to ( 103) PSKR_DAUCA Phytosulfokine receptor precursor (EC 2.7.11.1) (Phytosulfokine LRR receptor kinase) - Daucus carota (Carrot)moderately similar to ( 253) loc_os06g17490 12006.m06461 protein protein kinase, putative, expressed   Sporozoite_P67  no original description 	T
27.3.71	RNA.regulation of transcription.SNF7	MDP0000428283	 moderately similar to ( 253) AT4G29160 | Symbols: SNF7.1 | SNF7.1 | chr4:14381019-14382342 FORWARDmoderately similar to ( 251) loc_os09g09480 12009.m059945 protein charged multivesicular body protein 4b, putative, expressed  no original description 	T
27.4	RNA.RNA binding	MDP0000138865	 highly similar to ( 526) AT4G03110 | Symbols:  | RNA-binding protein, putative | chr4:1376710-1379275 REVERSEvery weakly similar to (58.5) RRP2_SPIOL Plastid-specific 30S ribosomal protein 2, chloroplast precursor (PSRP-2) - Spinacia oleracea (Spinach)highly similar to ( 539) loc_os01g71200 12001.m13163 protein RNA binding protein, putative, expressed  no original description 	T
2.2.1.3.1	major CHO metabolism.degradation.sucrose.invertases.neutral	MDP0000297851	 highly similar to ( 879) AT5G22510 | Symbols:  | beta-fructofuranosidase, putative / invertase, putative / saccharase, putative / beta-fructosidase, putative | chr5:7474974-7477479 REVERSEhighly similar to ( 867) loc_os02g32730 12002.m08366 protein alkaline/neutral invertase, putative, expressed  no original description 	T
31.3.1	cell.cycle.peptidylprolyl isomerase	MDP0000238529	 moderately similar to ( 223) AT2G29960 | Symbols: CYP5, ATCYP5, CYP19-4 | CYP5 (CYCLOPHILIN 5); peptidyl-prolyl cis-trans isomerase | chr2:12769183-12770528 REVERSEweakly similar to ( 168) CYPH_CATRO Peptidyl-prolyl cis-trans isomerase (EC 5.2.1.8) (PPIase) (Rotamase) (Cyclophilin) (Cyclosporin A-binding protein) - Catharanthus roseus (Rosy periwinkle) (Madagascar periwinkle)moderately similar to ( 215) loc_os06g49480 12006.m32182 protein peptidyl-prolyl cis-trans isomerase CYP19-4 precursor, putative, expressed  no original description 	T
29.5.5	protein.degradation.serine protease	MDP0000834920	 very weakly similar to ( 100) AT4G01575 | Symbols:  | serine protease inhibitor, Kazal-type family protein | chr4:682040-682474 REVERSEvery weakly similar to (98.2) loc_os01g59610 12001.m12096 protein serine-type endopeptidase inhibitor, putative, expressed  no original description 	T
28.1.3	DNA.synthesis/chromatin structure.histone	MDP0000189688	 moderately similar to ( 210) AT1G08880 | Symbols: HTA5, H2AXA, G-H2AX, GAMMA-H2AX | H2AXA; DNA binding | chr1:2847144-2847676 REVERSEmoderately similar to ( 214) H2AX_CICAR Histone H2AX - Cicer arietinum (Chickpea) (Garbanzo)weakly similar to ( 196) loc_os12g34510 12012.m07245 protein histone H2A, putative, expressed  no original description 	T
29.3.1	protein.targeting.nucleus	MDP0000250685	 highly similar to ( 991) AT2G16950 | Symbols: TRN1, ATTRN1 | TRN1 (TRANSPORTIN 1); protein transporter | chr2:7353939-7360637 FORWARDhighly similar to ( 942) loc_os04g59494 12004.m35538 protein transportin-2, putative, expressed  no original description 	T
9.9	mitochondrial electron transport / ATP synthesis.F1-ATPase 	MDP0000360515	 highly similar to ( 747) AT5G08690 | Symbols:  | ATP synthase beta chain 2, mitochondrial | chr5:2825739-2828352 FORWARDhighly similar to ( 802) ATPBM_ORYSA ATP synthase subunit beta, mitochondrial precursor (EC 3.6.3.14) - Oryza sativa (Rice)highly similar to ( 802) loc_os05g47980 12005.m08870 protein ATP synthase beta chain, mitochondrial precursor, putative, expressed   no original description 	T
31.1	cell.organisation	MDP0000329966	 moderately similar to ( 225) AT2G31200 | Symbols: ADF6, ATADF6 | ADF6 (ACTIN DEPOLYMERIZING FACTOR 6); actin binding | chr2:13294171-13294948 FORWARDmoderately similar to ( 231) ADF_VITVI Actin-depolymerizing factor (ADF) - Vitis vinifera (Grape)moderately similar to ( 204) loc_os12g43340 12012.m08108 protein actin-depolymerizing factor, putative, expressed  no original description 	T
30.2.11	signalling.receptor kinases.leucine rich repeat XI	MDP0000630971	 moderately similar to ( 305) AT1G17250 | Symbols: AtRLP3 | AtRLP3 (Receptor Like Protein 3); protein binding / protein kinase | chr1:5901169-5903439 REVERSEweakly similar to ( 190) PSKR_DAUCA Phytosulfokine receptor precursor (EC 2.7.11.1) (Phytosulfokine LRR receptor kinase) - Daucus carota (Carrot)moderately similar to ( 297) loc_os02g05930 12002.m05941 protein phytosulfokine receptor precursor, putative, expressed no original description 	T
10.6.3	cell wall.degradation.pectate lyases and polygalacturonases	MDP0000322627	 highly similar to ( 535) AT3G55140 | Symbols:  | pectate lyase family protein | chr3:20439060-20440225 FORWARDweakly similar to ( 151) PEL_LILLO Pectate lyase precursor (EC 4.2.2.2) - Lilium longiflorum (Trumpet lily)moderately similar to ( 453) loc_os01g36620 12001.m09957 protein pectate lyase 4 precursor, putative, expressed  no original description 	T
15.2	metal handling.binding, chelation and storage	MDP0000237908	 very weakly similar to (55.5) AT3G15353 | Symbols: MT3 | MT3 (METALLOTHIONEIN 3); copper ion binding | chr3:5180825-5181325 REVERSEweakly similar to ( 105) MT3_MALDO Metallothionein-like protein type 3 (MT-3) - Malus domestica (Apple) (Malus sylvestris)very weakly similar to (53.1) loc_os05g11320 12005.m05601 protein metallothionein-like protein type 3, putative, expressed no original description 	T
27.3.6	RNA.regulation of transcription.bHLH,Basic Helix-Loop-Helix family	MDP0000573178	 moderately similar to ( 251) AT1G27450 | Symbols: APT1, ATAPT1 | APT1 (ADENINE PHOSPHORIBOSYL TRANSFERASE 1); adenine phosphoribosyltransferase | chr1:9532421-9533807 FORWARDmoderately similar to ( 231) APT1_WHEAT Adenine phosphoribosyltransferase 1 (EC 2.4.2.7) (APRT 1) - Triticum aestivum (Wheat)moderately similar to ( 229) loc_os12g39860 12012.m07770 protein adenine phosphoribosyltransferase 1, putative, expressed  no original description 	T
23.3.1.1	nucleotide metabolism.salvage.phosphoribosyltransferases.aprt	MDP0000573178	 moderately similar to ( 251) AT1G27450 | Symbols: APT1, ATAPT1 | APT1 (ADENINE PHOSPHORIBOSYL TRANSFERASE 1); adenine phosphoribosyltransferase | chr1:9532421-9533807 FORWARDmoderately similar to ( 231) APT1_WHEAT Adenine phosphoribosyltransferase 1 (EC 2.4.2.7) (APRT 1) - Triticum aestivum (Wheat)moderately similar to ( 229) loc_os12g39860 12012.m07770 protein adenine phosphoribosyltransferase 1, putative, expressed  no original description 	T
29.2.1.1.3.1.6	protein.synthesis.ribosomal protein.prokaryotic.unknown organellar.30S subunit.S6	MDP0000584292	 weakly similar to ( 172) AT3G18760 | Symbols:  | ribosomal protein S6 family protein | chr3:6457930-6459558 REVERSEweakly similar to ( 157) loc_os01g12090 12001.m07824 protein expressed protein no original description 	T
35.2	not assigned.unknown	MDP0000222403	 moderately similar to ( 442) AT3G08030 | Symbols:  | unknown protein | chr3:2564517-2565819 FORWARDmoderately similar to ( 398) loc_os03g59300 12003.m34946 protein expressed protein  no original description 	T
21.3	redox.heme	MDP0000152733	 moderately similar to ( 310) AT3G10130 | Symbols:  | SOUL heme-binding family protein | chr3:3131122-3133158 REVERSEmoderately similar to ( 292) loc_os02g33020 12002.m08395 protein SOUL heme-binding protein, putative, expressed  no original description 	T
5.10	fermentation.aldehyde dehydrogenase	MDP0000239328	 highly similar to ( 760) AT2G14170 | Symbols: ALDH6B2 | ALDH6B2; 3-chloroallyl aldehyde dehydrogenase/ methylmalonate-semialdehyde dehydrogenase (acylating)/ oxidoreductase | chr2:5977851-5981899 REVERSEweakly similar to ( 176) BADH_SPIOL Betaine-aldehyde dehydrogenase, chloroplast precursor (EC 1.2.1.8) (BADH) - Spinacia oleracea (Spinach)highly similar to ( 716) loc_os07g09060 12007.m29088 protein methylmalonate-semialdehyde dehydrogenase, putative, expressed   no original description 	T
29.5.11.2	protein.degradation.ubiquitin.E1	MDP0000204446	 moderately similar to ( 265) AT4G24940 | Symbols: SAE1A, AT-SAE1-1, ATSAE1A | SAE1A (SUMO-ACTIVATING ENZYME 1A); SUMO activating enzyme | chr4:12823651-12825971 FORWARDvery weakly similar to (72.0) UBE11_WHEAT Ubiquitin-activating enzyme E1 1 - Triticum aestivum (Wheat)moderately similar to ( 229) loc_os11g30410 12011.m080006 protein ubiquitin-like 1-activating enzyme E1A, putative, expressed  no original description 	T
35.2	not assigned.unknown	MDP0000357718	   no original description 	T
29.5.11.4.3.2	protein.degradation.ubiquitin.E3.SCF.FBOX	MDP0000206144	 very weakly similar to (57.4) AT2G36090 | Symbols:  | F-box family protein | chr2:15158631-15159584 FORWARD  no original description 	T
29.2.3	protein.synthesis.initiation	MDP0000608985	 highly similar to ( 608) AT5G36230 | Symbols:  | eIF4-gamma/eIF5/eIF2-epsilon domain-containing protein | chr5:14273519-14276773 FORWARDhighly similar to ( 602) loc_os11g21990 12011.m28792 protein eukaryotic initiation factor 5C CG2922-PF, isoform F, putative, expressed   no original description 	T
29.2.1.2.2.527	protein.synthesis.ribosomal protein.eukaryotic.60S subunit.L27A	MDP0000200882	 moderately similar to ( 260) AT1G70600 | Symbols:  | structural constituent of ribosome | chr1:26621168-26621608 REVERSEmoderately similar to ( 258) loc_os07g42170 12007.m08453 protein 60S ribosomal protein L27a-2, putative, expressed  no original description 	T
35.2	not assigned.unknown	MDP0000160390	 weakly similar to ( 112) AT5G64400 | Symbols:  | unknown protein | chr5:25748922-25749947 FORWARDweakly similar to ( 122) loc_os03g48080 12003.m09819 protein p8MTCP1, putative, expressed   no original description 	T
26.28	misc.GDSL-motif lipase	MDP0000569759	 moderately similar to ( 332) AT2G04570 | Symbols:  | GDSL-motif lipase/hydrolase family protein | chr2:1594747-1596129 FORWARDweakly similar to ( 152) APG_BRANA Anter-specific proline-rich protein APG (Protein CEX) (Fragment) - Brassica napus (Rape)moderately similar to ( 251) loc_os06g43044 12006.m08834 protein anther-specific proline-rich protein APG, putative, expressed  no original description 	T
30.2.11	signalling.receptor kinases.leucine rich repeat XI	MDP0000375826	 highly similar to ( 828) AT1G65380 | Symbols: CLV2, AtRLP10 | CLV2 (clavata 2); protein binding / receptor signaling protein | chr1:24286943-24289105 FORWARDmoderately similar to ( 254) RPK1_IPONI Receptor-like protein kinase precursor (EC 2.7.11.1) - Ipomoea nil (Japanese morning glory) (Pharbitis nil)moderately similar to ( 441) loc_os02g39100 12002.m08998 protein fasciated ear2, putative, expressed  no original description 	T
20.1	stress.biotic	MDP0000629887	 weakly similar to ( 191) AT5G66590 | Symbols:  | allergen V5/Tpx-1-related family protein | chr5:26574255-26574812 FORWARDweakly similar to ( 194) ST14_SOLTU STS14 protein precursor - Solanum tuberosum (Potato)weakly similar to ( 112) loc_os07g03730 12007.m04843 protein pathogenesis-related protein 1 precursor, putative, expressed no original description 	T
35.2	not assigned.unknown	MDP0000177377	 highly similar to ( 533) AT1G29050 | Symbols:  | unknown protein | chr1:10136376-10139082 REVERSEmoderately similar to ( 460) loc_os01g46350 12001.m10833 protein expressed protein  no original description 	T
35.2	not assigned.unknown	MDP0000693594	 very weakly similar to (91.7) AT4G37300 | Symbols: MEE59 | MEE59 (maternal effect embryo arrest 59) | chr4:17554805-17555498 FORWARDweakly similar to ( 167) loc_os03g15920 12003.m35599 protein expressed protein  no original description 	T
23.1.2.20	nucleotide metabolism.synthesis.purine.adenylosuccinate synthase	MDP0000305478	 highly similar to ( 762) AT3G57610 | Symbols: ADSS | ADSS (ADENYLOSUCCINATE SYNTHASE); adenylosuccinate synthase | chr3:21334519-21336603 REVERSEhighly similar to ( 710) PURA_WHEAT Adenylosuccinate synthetase, chloroplast precursor (EC 6.3.4.4) (IMP--aspartate ligase) (AdSS) (AMPSase) (Fragment) - Triticum aestivum (Wheat)highly similar to ( 708) loc_os03g18810 12003.m35176 protein transaminase/ transferase, transferring nitrogenous groups, putative, expressed  no original description 	T
3.1.2.2	minor CHO metabolism.raffinose family.raffinose synthases.putative	MDP0000129144	 highly similar to ( 733) AT3G57520 | Symbols: AtSIP2 | AtSIP2 (Arabidopsis thaliana seed imbibition 2); hydrolase, hydrolyzing O-glycosyl compounds | chr3:21289299-21292694 REVERSEweakly similar to ( 154) STSYN_PEA Stachyose synthase precursor (EC 2.4.1.67) (Galactinol--raffinose galactosyltransferase) - Pisum sativum (Garden pea)highly similar to ( 659) loc_os03g59430 12003.m10841 protein stachyose synthase precursor, putative, expressed   no original description 	T
11.3.1	lipid metabolism.Phospholipid synthesis.1-acylglycerol-3-phosphate O-acyltransferase	MDP0000784365	 moderately similar to ( 449) AT3G18850 | Symbols: LPAT5 | LPAT5; acyltransferase | chr3:6499529-6500840 REVERSEweakly similar to ( 158) LPAT2_BRAOL 1-acyl-sn-glycerol-3-phosphate acyltransferase 2 (EC 2.3.1.51) (Lysophosphatidyl acyltransferase 2) - Brassica oleracea (Wild cabbage)moderately similar to ( 441) loc_os05g42270 12005.m08400 protein 1-acyl-sn-glycerol-3-phosphate acyltransferase 4, putative, expressed  no original description 	T
20.2.3	stress.abiotic.drought/salt	MDP0000232706	 highly similar to ( 746) AT3G01100 | Symbols: HYP1, ATHYP1 | HYP1 (HYPOTHETICAL PROTEIN 1) | chr3:35810-38176 REVERSEhighly similar to ( 602) loc_os12g39320 12012.m07717 protein HYP1, putative, expressed  no original description 	T
33.99	development.unspecified	MDP0000124634	 very weakly similar to (77.0) AT2G03440 | Symbols:  | nodulin-related | chr2:1039409-1039972 REVERSEvery weakly similar to (64.3) loc_os03g11220 12003.m06573 protein RPGR, putative, expressed  no original description 	T
11.8	lipid metabolism.'exotics'(steroids, squalene etc)	MDP0000509613	 moderately similar to ( 333) AT5G54500 | Symbols: FQR1 | FQR1 (FLAVODOXIN-LIKE QUINONE REDUCTASE 1); FMN binding / oxidoreductase, acting on NADH or NADPH, quinone or similar compound as acceptor | chr5:22124674-22126256 FORWARDmoderately similar to ( 337) loc_os08g04460 12008.m04589 protein minor allergen Alt a 7, putative, expressed no original description 	T
31.1	cell.organisation	MDP0000242313	 moderately similar to ( 357) AT3G54070 | Symbols:  | ankyrin repeat family protein | chr3:20021330-20023603 REVERSEvery weakly similar to (85.5) loc_os11g24780 12011.m06403 protein expressed protein no original description 	T
8.1.2	TCA / org transformation.TCA.CS	MDP0000300560	 highly similar to ( 508) AT2G44350 | Symbols: ATCS, CSY4 | ATCS; ATP binding / ATP citrate synthase/ citrate (SI)-synthase | chr2:18316673-18320524 FORWARDhighly similar to ( 513) CISY_DAUCA Citrate synthase, mitochondrial precursor (EC 2.3.3.1) - Daucus carota (Carrot)moderately similar to ( 499) loc_os02g10070 12002.m33351 protein citrate synthase 4, mitochondrial precursor, putative, expressed  no original description 	T
26.28	misc.GDSL-motif lipase	MDP0000317158	 moderately similar to ( 449) AT2G42990 | Symbols:  | GDSL-motif lipase/hydrolase family protein | chr2:17879056-17880200 FORWARDmoderately similar to ( 220) APG_BRANA Anter-specific proline-rich protein APG (Protein CEX) (Fragment) - Brassica napus (Rape)moderately similar to ( 383) loc_os06g43044 12006.m08834 protein anther-specific proline-rich protein APG, putative, expressed    no original description 	T
29.3.4.2	protein.targeting.secretory pathway.golgi	MDP0000213649	 nearly identical (1326) AT3G07100 | Symbols:  | protein transport protein Sec24, putative | chr3:2245689-2250077 REVERSEnearly identical (1169) loc_os04g04020 12004.m05709 protein protein transport protein Sec24-like, putative, expressed   no original description 	T
35.2	not assigned.unknown	MDP0000362058	 COG3889  no original description 	T
11.1.12	lipid metabolism.FA synthesis and FA elongation.ACP protein	MDP0000159492	 weakly similar to ( 104) AT5G47630 | Symbols: mtACP3 | mtACP3 (mitochondrial acyl carrier protein 3); acyl carrier/ cofactor binding | chr5:19306397-19306885 FORWARDweakly similar to ( 102) loc_os03g39860 12003.m09069 protein acyl carrier protein, mitochondrial precursor, putative, expressed no original description 	T
27.3.3	RNA.regulation of transcription.AP2/EREBP, APETALA2/Ethylene-responsive element binding protein family	MDP0000783818	 moderately similar to ( 218) AT4G23750 | Symbols: CRF2 | CRF2 (CYTOKININ RESPONSE FACTOR 2); DNA binding / transcription factor | chr4:12376751-12377782 FORWARDvery weakly similar to (91.7) ERF3_TOBAC Ethylene-responsive transcription factor 3 (Ethylene-responsive element-binding factor 3 homolog) (EREBP-5) (NtERF5) - Nicotiana tabacum (Common tobacco)weakly similar to ( 139) loc_os01g12440 12001.m07859 protein ap2 domain protein, putative, expressed   no original description 	T
10.5.1.1	cell wall.cell wall proteins.AGPs.AGP	MDP0000200218	 very weakly similar to (68.9) AT2G46330 | Symbols: AGP16, ATAGP16 | AGP16 (ARABINOGALACTAN PROTEIN 16) | chr2:19018730-19019108 REVERSEvery weakly similar to (65.1) loc_os05g12580 12005.m05721 protein AGP20, putative no original description 	T
16.1.2.2	secondary metabolism.isoprenoids.mevalonate pathway.HMG-CoA synthase	MDP0000661951	 highly similar to ( 728) AT4G11820 | Symbols: MVA1 | MVA1; acetyl-CoA C-acetyltransferase/ hydroxymethylglutaryl-CoA synthase | chr4:7109124-7111901 REVERSEhighly similar to ( 710) loc_os09g34960 12009.m06496 protein hydroxymethylglutaryl-CoA synthase, putative, expressed   no original description 	T
29.5.11.5	protein.degradation.ubiquitin.ubiquitin protease	MDP0000439958	 highly similar to ( 966) AT3G11910 | Symbols: UBP13 | UBP13 (UBIQUITIN-SPECIFIC PROTEASE 13); ubiquitin thiolesterase/ ubiquitin-specific protease | chr3:3761758-3770290 REVERSEhighly similar to ( 972) loc_os11g36470 12011.m07499 protein cysteine-type endopeptidase/ double-stranded DNA binding protein, putative, expressed   no original description 	T
34.2	transport.sugars	MDP0000485591	 moderately similar to ( 468) AT1G34580 | Symbols:  | monosaccharide transporter, putative | chr1:12660631-12663553 FORWARDmoderately similar to ( 390) HEX6_RICCO Hexose carrier protein HEX6 - Ricinus communis (Castor bean)moderately similar to ( 411) loc_os04g38220 12004.m08811 protein sugar transport protein 5, putative, expressed   no original description 	T
27.4	RNA.RNA binding	MDP0000238655	 weakly similar to ( 141) AT1G07350 | Symbols:  | transformer serine/arginine-rich ribonucleoprotein, putative | chr1:2258816-2259586 REVERSEvery weakly similar to (61.6) ROC5_NICSY 33 kDa ribonucleoprotein, chloroplast precursor - Nicotiana sylvestris (Wood tobacco)weakly similar to ( 139) loc_os03g15890 12003.m35139 protein arginine/serine-rich splicing factor RSP41, putative, expressed    no original description 	T
35.2	not assigned.unknown	MDP0000932963	 weakly similar to ( 122) AT5G18130 | Symbols:  | unknown protein | chr5:5995634-5997165 FORWARDvery weakly similar to (78.2) loc_os03g59180 12003.m10818 protein expressed protein   no original description 	T
27.4	RNA.RNA binding	MDP0000377452	 weakly similar to ( 178) AT4G16830 | Symbols:  | nuclear RNA-binding protein (RGGA) | chr4:9470652-9472308 FORWARDweakly similar to ( 160) loc_os05g51180 12005.m09183 protein plasminogen activator inhibitor 1 RNA-binding protein, putative, expressed MSP1_C  no original description 	T
35.2	not assigned.unknown	MDP0000404961	 weakly similar to ( 114) AT5G09270 | Symbols:  | unknown protein | chr5:2879680-2880015 FORWARDvery weakly similar to (99.4) loc_os01g64190 12001.m12534 protein expressed protein no original description 	T
27.1.1	RNA.processing.splicing	MDP0000268577	 moderately similar to ( 274) AT3G49430 | Symbols: SRp34a | SRp34a (Ser/Arg-rich protein 34a); RNA binding / nucleic acid binding / nucleotide binding | chr3:18332668-18334617 FORWARDvery weakly similar to (93.6) RLA1_MAIZE 60S acidic ribosomal protein P1 (L12) - Zea mays (Maize)moderately similar to ( 245) loc_os07g47630 12007.m079789 protein pre-mRNA-splicing factor SF2, putative, expressed  no original description 	T
35.2	not assigned.unknown	MDP0000368249	  no original description 	T
35.1	not assigned.no ontology	MDP0000304911	 weakly similar to ( 154) AT3G26510 | Symbols:  | octicosapeptide/Phox/Bem1p (PB1) domain-containing protein | chr3:9711886-9712542 REVERSEweakly similar to ( 126) loc_os11g25800 12011.m06501 protein octicosapeptide/Phox/Bem1p, putative, expressed   no original description 	T
29.3.4.99	protein.targeting.secretory pathway.unspecified	MDP0000311212	 highly similar to ( 955) AT3G63460 | Symbols:  | WD-40 repeat family protein | chr3:23431009-23437241 REVERSEhighly similar to ( 812) loc_os07g46370 12007.m08854 protein EMB2221, putative, expressed  no original description 	T
30.4	signalling.phosphinositides	MDP0000186752	 moderately similar to ( 251) AT5G05710 | Symbols:  | pleckstrin homology (PH) domain-containing protein | chr5:1715943-1716377 FORWARDmoderately similar to ( 237) loc_os01g63800 12001.m12495 protein pleckstrin homology domain-containing protein 1, putative, expressed   no original description 	T
29.6	protein.folding	MDP0000286595	 highly similar to ( 640) AT5G55220 | Symbols:  | trigger factor type chaperone family protein | chr5:22397677-22400678 FORWARDhighly similar to ( 559) loc_os06g20320 12006.m06689 protein trigger factor-like protein, putative, expressed  no original description 	T
35.2	not assigned.unknown	MDP0000847090	    no original description 	T
35.2	not assigned.unknown	MDP0000347896	   no original description 	T
4.1.10	glycolysis.cytosolic branch.aldolase	MDP0000276844	 highly similar to ( 717) AT4G26510 | Symbols:  | ATP binding / kinase/ phosphotransferase, alcohol group as acceptor / uracil phosphoribosyltransferase | chr4:13384503-13387920 FORWARDhighly similar to ( 575) ALF1_PEA Fructose-bisphosphate aldolase, cytoplasmic isozyme 1 (EC 4.1.2.13) - Pisum sativum (Garden pea)highly similar to ( 651) loc_os02g17320 12002.m33637 protein uridine/cytidine kinase-like 1, putative, expressed   no original description 	T
31.4	cell.vesicle transport	MDP0000176111	 nearly identical (1353) AT1G62020 | Symbols:  | coatomer protein complex, subunit alpha, putative | chr1:22919814-22923728 FORWARDvery weakly similar to (71.2) PF20_CHLRE Flagellar WD repeat protein PF20 - Chlamydomonas reinhardtiinearly identical (1251) loc_os09g04110 12009.m22040 protein coatomer subunit alpha, putative, expressed  no original description 	T
33.99	development.unspecified	MDP0000299181	 moderately similar to ( 271) AT1G10200 | Symbols: WLIM1 | WLIM1; transcription factor/ zinc ion binding | chr1:3346677-3347763 REVERSEmoderately similar to ( 209) SF3_HELAN Pollen-specific protein SF3 - Helianthus annuus (Common sunflower)moderately similar to ( 248) loc_os12g32620 12012.m07062 protein pollen-specific protein SF3, putative, expressed no original description 	T
35.1	not assigned.no ontology	MDP0000297342	 moderately similar to ( 287) AT1G43130 | Symbols: LCV2 | LCV2 (LIKE COV 2) | chr1:16228661-16231158 REVERSEmoderately similar to ( 315) loc_os01g64780 12001.m43301 protein COV1-like protein, putative, expressed  no original description 	T
30.2.11	signalling.receptor kinases.leucine rich repeat XI	MDP0000298511	 nearly identical (1044) AT1G73080 | Symbols: PEPR1 | PEPR1 (PEP1 receptor 1); ATP binding / kinase/ protein binding / protein serine/threonine kinase | chr1:27484513-27488021 FORWARDnearly identical (1096) RPK1_IPONI Receptor-like protein kinase precursor (EC 2.7.11.1) - Ipomoea nil (Japanese morning glory) (Pharbitis nil)highly similar to ( 978) loc_os08g34640 12008.m07452 protein receptor-like protein kinase precursor, putative, expressed  no original description 	T
35.2	not assigned.unknown	MDP0000364675	  no original description 	T
10.7	cell wall.modification	MDP0000574554	 moderately similar to ( 332) AT2G03090 | Symbols: ATEXPA15, EXP15, ATEXP15, ATHEXP ALPHA 1.3 | ATEXPA15 (ARABIDOPSIS THALIANA EXPANSIN A15) | chr2:917361-918554 REVERSEmoderately similar to ( 300) EXPA4_ORYSA Expansin-A4 precursor (OsEXPA4) (Alpha-expansin-4) (OsEXP4) (OsaEXPa1.22) - Oryza sativa (Rice)moderately similar to ( 300) loc_os05g39990 12005.m08176 protein alpha-expansin 1 precursor, putative, expressed  no original description 	T
29.2.1.2.2.523	protein.synthesis.ribosomal protein.eukaryotic.60S subunit.L23A	MDP0000243760	 moderately similar to ( 221) AT2G39460 | Symbols: ATRPL23A, RPL23A, RPL23AA | RPL23AA (RIBOSOMAL PROTEIN L23AA); RNA binding / nucleotide binding / structural constituent of ribosome | chr2:16475049-16475904 FORWARDmoderately similar to ( 223) RL23A_DAUCA 60S ribosomal protein L23a - Daucus carota (Carrot)moderately similar to ( 201) loc_os04g42270 12004.m35367 protein 60S ribosomal protein L23a, putative, expressed   no original description 	T
1.3.13	PS.calvin cycle.rubisco interacting	MDP0000223905	 highly similar to ( 728) AT2G39730 | Symbols: RCA | RCA (RUBISCO ACTIVASE); ADP binding / ATP binding / enzyme regulator/ ribulose-1,5-bisphosphate carboxylase/oxygenase activator | chr2:16571174-16573345 REVERSEhighly similar to ( 738) RCA_MALDO Ribulose bisphosphate carboxylase/oxygenase activase, chloroplast precursor (RuBisCO activase) (RA) - Malus domestica (Apple) (Malus sylvestris)highly similar to ( 721) loc_os11g47970 12011.m28706 protein ribulose bisphosphate carboxylase/oxygenase activase, chloroplast precursor, putative, expressed   no original description 	T
30.2.11	signalling.receptor kinases.leucine rich repeat XI	MDP0000930936	 weakly similar to ( 184) AT4G20270 | Symbols: BAM3 | BAM3 (BARELY ANY MERISTEM 3); ATP binding / protein binding / protein kinase/ protein serine/threonine kinase | chr4:10949822-10952924 FORWARDvery weakly similar to (63.5) RPK1_IPONI Receptor-like protein kinase precursor (EC 2.7.11.1) - Ipomoea nil (Japanese morning glory) (Pharbitis nil)weakly similar to ( 155) loc_os07g04190 12007.m04888 protein receptor protein kinase CLAVATA1 precursor, putative, expressed  no original description 	T
29.4.1.57	protein.postranslational modification.kinase.receptor like cytoplasmatic kinase VII	MDP0000930936	 weakly similar to ( 184) AT4G20270 | Symbols: BAM3 | BAM3 (BARELY ANY MERISTEM 3); ATP binding / protein binding / protein kinase/ protein serine/threonine kinase | chr4:10949822-10952924 FORWARDvery weakly similar to (63.5) RPK1_IPONI Receptor-like protein kinase precursor (EC 2.7.11.1) - Ipomoea nil (Japanese morning glory) (Pharbitis nil)weakly similar to ( 155) loc_os07g04190 12007.m04888 protein receptor protein kinase CLAVATA1 precursor, putative, expressed  no original description 	T
10.5.1.1	cell wall.cell wall proteins.AGPs.AGP	MDP0000658332	 moderately similar to ( 481) AT2G45470 | Symbols: FLA8, AGP8 | FLA8 (FASCICLIN-LIKE ARABINOGALACTAN PROTEIN 8) | chr2:18742797-18744059 REVERSEmoderately similar to ( 403) loc_os04g48490 12004.m09785 protein fasciclin-like arabinogalactan protein 10 precursor, putative, expressed   no original description 	T
35.2	not assigned.unknown	MDP0000253162	 highly similar to ( 539) AT5G47860 | Symbols:  | unknown protein | chr5:19381656-19384310 FORWARDhighly similar to ( 534) loc_os04g33280 12004.m101523 protein expressed protein  no original description 	T
35.2	not assigned.unknown	MDP0000282006	 very weakly similar to (74.7) loc_os04g54370 12004.m10328 protein expressed protein  no original description 	T
28.2	DNA.repair	MDP0000210875	 moderately similar to ( 441) AT1G79650 | Symbols: RAD23 | RAD23; damaged DNA binding | chr1:29972406-29975132 REVERSEmoderately similar to ( 300) RAD23_ORYSA Probable DNA repair protein RAD23 (OsRAD23) - Oryza sativa (Rice)moderately similar to ( 375) loc_os02g08300 12002.m06176 protein DNA repair protein RAD23-1, putative, expressed   no original description 	T
2.2.2.10	major CHO metabolism.degradation.starch.laforin like phosphoglucan phosphatase (SEX4)	MDP0000283141	 moderately similar to ( 308) AT3G52180 | Symbols: ATPTPKIS1, DSP4, SEX4 | SEX4 (STARCH-EXCESS 4); polysaccharide binding / protein tyrosine/serine/threonine phosphatase | chr3:19350574-19353459 REVERSEmoderately similar to ( 288) loc_os03g01750 12003.m101148 protein protein tyrosine phosphatase, putative, expressed no original description 	T
30.8	signalling.misc	MDP0000768504	 weakly similar to ( 115) AT3G16570 | Symbols: RALFL23 | RALFL23 (ralf-like 23); signal transducer | chr3:5644748-5645164 FORWARDvery weakly similar to (93.6) loc_os12g35670 12012.m07360 protein RALF precursor, putative, expressed no original description 	T
1.1.6	PS.lightreaction.NADH DH	MDP0000335786	 moderately similar to ( 289) AT5G58260 | Symbols:  | Encodes subunit NDH-N of NAD(P)H:plastoquinone dehydrogenase complex (Ndh complex) present in the thylakoid membrane of chloroplasts. This subunit is thought to be required for Ndh complex assembly. | chr5:23561356-23561929 REVERSEmoderately similar to ( 293) loc_os01g66000 12001.m12708 protein NADH dehydrogenase I subunit N, putative, expressed   no original description 	T
35.1	not assigned.no ontology	MDP0000535448	 weakly similar to ( 169) AT4G27870 | Symbols:  | integral membrane family protein | chr4:13878983-13882679 FORWARDweakly similar to ( 130) loc_os06g01440 12006.m04883 protein integral membrane protein, expressed   no original description 	T
23.3.2.3	nucleotide metabolism.salvage.nucleoside kinases.thymidine kinase	MDP0000147702	 moderately similar to ( 290) AT3G07800 | Symbols:  | thymidine kinase, putative | chr3:2489944-2490935 REVERSEmoderately similar to ( 291) KITH_ORYSA Thymidine kinase (EC 2.7.1.21) - Oryza sativa (Rice)moderately similar to ( 296) loc_os03g02200 12003.m05763 protein thymidine kinase, putative, expressed   no original description 	T
27.4	RNA.RNA binding	MDP0000340057	 moderately similar to ( 251) AT5G04600 | Symbols:  | RNA recognition motif (RRM)-containing protein | chr5:1323700-1325486 FORWARDmoderately similar to ( 246) loc_os08g31810 12008.m080161 protein MKI67 FHA domain-interacting nucleolar phosphoprotein-like, putative, expressed  no original description 	T
29.2.1.2.2.3	protein.synthesis.ribosomal protein.eukaryotic.60S subunit.L3	MDP0000388232	 highly similar to ( 673) AT1G61580 | Symbols: RPL3B, ARP2 | RPL3B (R-PROTEIN L3 B); structural constituent of ribosome | chr1:22720833-22722402 REVERSEhighly similar to ( 664) RL3_ORYSJ 60S ribosomal protein L3 OS=Oryza sativa subsp. japonica GN=RPL3 PE=2 SV=2highly similar to ( 664) loc_os12g07010 12012.m04691 protein 60S ribosomal protein L3, putative, expressed  no original description 	T
20.1.7	stress.biotic.PR-proteins	MDP0000775293	 moderately similar to ( 387) AT5G17680 | Symbols:  | disease resistance protein (TIR-NBS-LRR class), putative | chr5:5822999-5827153 FORWARDweakly similar to ( 107) loc_os01g15580 12001.m08156 protein disease resistance protein RGA2, putative, expressed   no original description 	T
30.2.11	signalling.receptor kinases.leucine rich repeat XI	MDP0000798156	 nearly identical (1124) AT3G28040 | Symbols:  | leucine-rich repeat transmembrane protein kinase, putative | chr3:10435139-10438268 FORWARDmoderately similar to ( 331) PSKR_DAUCA Phytosulfokine receptor precursor (EC 2.7.11.1) (Phytosulfokine LRR receptor kinase) - Daucus carota (Carrot)highly similar to ( 979) loc_os01g72700 12001.m13304 protein ATP binding protein, putative, expressed   no original description 	T
35.1	not assigned.no ontology	MDP0000324348	 weakly similar to ( 148) AT1G12390 | Symbols:  | cornichon family protein | chr1:4220347-4221481 FORWARDweakly similar to ( 125) loc_os06g04500 12006.m091591 protein ER-derived vesicles protein ERV14, putative, expressed no original description 	T
17.3.1.2.6	hormone metabolism.brassinosteroid.synthesis-degradation.sterols.DWF7	MDP0000365472	 very weakly similar to (91.3) AT3G02580 | Symbols: STE1, DWF7, BUL1 | STE1 (STEROL 1); C-5 sterol desaturase | chr3:547048-548615 FORWARDvery weakly similar to (91.7) SC5D_TOBAC Delta(7)-sterol-C5(6)-desaturase (EC 1.3.3.-) (Delta-7-C-5 sterol desaturase) (Delta(7)-sterol-C5-desaturase) - Nicotiana tabacum (Common tobacco)very weakly similar to (92.0) loc_os01g04260 12001.m07061 protein delta-7-sterol-C5, putative, expressed no original description 	T
30.5	signalling.G-proteins	MDP0000281188	 nearly identical (1689) AT5G65750 | Symbols:  | 2-oxoglutarate dehydrogenase E1 component, putative / oxoglutarate decarboxylase, putative / alpha-ketoglutaric dehydrogenase, putative | chr5:26304212-26307947 FORWARDmoderately similar to ( 272) RB1BV_BETVU Ras-related protein RAB1BV - Beta vulgaris (Sugar beet)nearly identical (1600) loc_os07g49520 12007.m09160 protein 2-oxoglutarate dehydrogenase E1 component, mitochondrial precursor, putative, expressed   no original description 	T
35.2	not assigned.unknown	MDP0000937443	 very weakly similar to ( 100) AT1G31240 | Symbols:  | DNA binding | chr1:11164065-11164898 REVERSEvery weakly similar to (83.6) loc_os02g47160 12002.m09751 protein OsWAK17 - OsWAK short gene, expressed     TrbL SPT5 COG4907  no original description 	T
26.19	misc.plastocyanin-like	MDP0000326004	 weakly similar to ( 176) AT4G31840 | Symbols:  | plastocyanin-like domain-containing protein | chr4:15401798-15402426 FORWARDvery weakly similar to (95.9) NO552_SOYBN Early nodulin 55-2 precursor (N-55-2) (Nodulin-315) - Glycine max (Soybean)weakly similar to ( 110) loc_os01g57880 12001.m11936 protein early nodulin-like protein 3 precursor, putative, expressed no original description 	T
35.2	not assigned.unknown	MDP0000205529	 weakly similar to ( 150) AT5G62550 | Symbols:  | unknown protein | chr5:25105839-25107875 REVERSEweakly similar to ( 157) loc_os06g38960 12006.m32105 protein expressed protein no original description 	T
29.5.11.20	protein.degradation.ubiquitin.proteasom	MDP0000127609	 moderately similar to ( 341) AT1G21720 | Symbols: PBC1 | PBC1 (PROTEASOME BETA SUBUNIT C1); peptidase/ threonine-type endopeptidase | chr1:7626394-7628070 FORWARDmoderately similar to ( 325) PSB3_ORYSA Proteasome subunit beta type 3 (EC 3.4.25.1) (20S proteasome alpha subunit C) (20S proteasome subunit beta-3) - Oryza sativa (Rice)moderately similar to ( 325) loc_os02g08520 12002.m06198 protein proteasome subunit beta type 3, putative, expressed  no original description 	T
26.2	misc.UDP glucosyl and glucoronyl transferases	MDP0000715898	 moderately similar to ( 446) AT3G21760 | Symbols:  | UDP-glucoronosyl/UDP-glucosyl transferase family protein | chr3:7667099-7668556 FORWARDweakly similar to ( 144) ZOX_PHAVU Zeatin O-xylosyltransferase (EC 2.4.2.40) (Zeatin O-beta-D-xylosyltransferase) - Phaseolus vulgaris (Kidney bean) (French bean)moderately similar to ( 308) loc_os07g32010 12007.m07467 protein anthocyanidin 5,3-O-glucosyltransferase, putative, expressed   PRK08026  no original description 	T
35.2	not assigned.unknown	MDP0000134543	 moderately similar to ( 272) AT2G28370 | Symbols:  | FUNCTIONS IN: molecular_function unknown; INVOLVED IN: biological_process unknown; LOCATED IN: plasma membrane; EXPRESSED IN: 23 plant structures; EXPRESSED DURING: 15 growth stages; CONTAINS InterPro DOMAIN/s: Uncharacterised protein family UPF0497, trans-membrane plant (InterPro:IPR006702); BEST Arabidopsis thaliana protein match is: integral membrane protein, putative (TAIR:AT2G37200.1); Has 100 Blast hits to 100 proteins in 9 species: Archae - 0; Bacteria - 0; Metazoa - 0; Fungi - 0; Plants - 100; Viruses - 0; Other Eukaryotes - 0 (source: NCBI BLink). | chr2:12132092-12133447 FORWARDmoderately similar to ( 249) loc_os03g10870 12003.m06539 protein expressed protein no original description 	T
35.2	not assigned.unknown	MDP0000202766	  no original description 	T
27.3.29	RNA.regulation of transcription.TCP transcription factor family	MDP0000916623	 weakly similar to ( 198) AT3G15030 | Symbols: TCP4 | TCP4 (TCP family transcription factor 4); transcription factor | chr3:5062308-5063570 FORWARDweakly similar to ( 177) loc_os01g11550 12001.m07772 protein mutant cincinnata, putative, expressed no original description 	T
27.4	RNA.RNA binding	MDP0000334305	 highly similar to ( 502) AT5G53060 | Symbols:  | KH domain-containing protein | chr5:21515355-21518197 FORWARDmoderately similar to ( 449) loc_os06g23460 12006.m06998 protein nucleic acid binding protein, putative, expressed  no original description 	T
26.6	misc.O-methyl transferases	MDP0000854767	 moderately similar to ( 380) AT3G12270 | Symbols: ATPRMT3, PRMT3 | PRMT3 (PROTEIN ARGININE METHYLTRANSFERASE 3); methyltransferase/ zinc ion binding | chr3:3910642-3913122 FORWARDmoderately similar to ( 343) loc_os07g44640 12007.m08685 protein protein arginine N-methyltransferase 3-like protein, putative, expressed no original description 	T
29.6	protein.folding	MDP0000364484	 weakly similar to ( 187) AT3G60210 | Symbols:  | chloroplast chaperonin 10, putative | chr3:22251443-22252709 REVERSEweakly similar to ( 164) loc_os10g41710 12010.m06930 protein CHL-CPN10, putative, expressed  no original description 	T
17.5.2	hormone metabolism.ethylene.signal transduction	MDP0000219737	 highly similar to ( 775) AT3G23150 | Symbols: ETR2 | ETR2 (ethylene response 2); ethylene binding / glycogen synthase kinase 3/ protein histidine kinase/ receptor | chr3:8255156-8257560 FORWARDmoderately similar to ( 406) ETR1_MALDO Ethylene receptor (EC 2.7.13.3) - Malus domestica (Apple) (Malus sylvestris)highly similar to ( 630) loc_os04g08740 12004.m35115 protein ethylene receptor, putative, expressed  no original description 	T
31.1	cell.organisation	MDP0000733884	 moderately similar to ( 218) AT2G31200 | Symbols: ADF6, ATADF6 | ADF6 (ACTIN DEPOLYMERIZING FACTOR 6); actin binding | chr2:13294171-13294948 FORWARDmoderately similar to ( 225) ADF_VITVI Actin-depolymerizing factor (ADF) - Vitis vinifera (Grape)moderately similar to ( 201) loc_os12g43340 12012.m08108 protein actin-depolymerizing factor, putative, expressed  no original description 	T
30.5	signalling.G-proteins	MDP0000245693	 moderately similar to ( 447) AT5G11420 | Symbols:  | FUNCTIONS IN: molecular_function unknown; INVOLVED IN: biological_process unknown; LOCATED IN: cell wall, plant-type cell wall; EXPRESSED IN: 23 plant structures; EXPRESSED DURING: 13 growth stages; CONTAINS InterPro DOMAIN/s: Protein of unknown function DUF642 (InterPro:IPR006946), Galactose-binding like (InterPro:IPR008979); BEST Arabidopsis thaliana protein match is: unknown protein (TAIR:AT5G25460.1); Has 185 Blast hits to 157 proteins in 11 species: Archae - 0; Bacteria - 0; Metazoa - 0; Fungi - 0; Plants - 185; Viruses - 0; Other Eukaryotes - 0 (source: NCBI BLink). | chr5:3644655-3646991 FORWARDmoderately similar to ( 422) RAN_VICFA GTP-binding nuclear protein Ran/TC4 - Vicia faba (Broad bean)moderately similar to ( 413) loc_os01g42530 12001.m10517 protein GTP-binding nuclear protein Ran-2, putative, expressed  no original description 	T
27.3.23	RNA.regulation of transcription.HSF,Heat-shock transcription factor family	MDP0000260377	 moderately similar to ( 269) AT5G62020 | Symbols: AT-HSFB2A, HSFB2A | AT-HSFB2A; DNA binding / transcription factor | chr5:24916212-24917194 FORWARDmoderately similar to ( 235) loc_os08g43334 12008.m61974 protein heat shock factor protein 7, putative, expressed   no original description 	T
26.10	misc.cytochrome P450	MDP0000161853	 moderately similar to ( 459) AT3G48280 | Symbols: CYP71A25 | CYP71A25; electron carrier/ heme binding / iron ion binding / monooxygenase/ oxygen binding | chr3:17879594-17881164 FORWARDmoderately similar to ( 454) C71A4_SOLME Cytochrome P450 71A4 (EC 1.14.-.-) (CYPLXXIA4) (P-450EG2) - Solanum melongena (Eggplant) (Aubergine)moderately similar to ( 362) loc_os01g12760 12001.m07889 protein cytochrome P450 71A6, putative, expressed  no original description 	T
34.16	transport.ABC transporters and multidrug resistance systems	MDP0000881966	 highly similar to ( 539) AT2G34660 | Symbols: ATMRP2, EST4, MRP2 | ATMRP2 (ARABIDOPSIS THALIANA MULTIDRUG RESISTANCE-ASSOCIATED PROTEIN 2); ATPase, coupled to transmembrane movement of substances | chr2:14603267-14612387 FORWARDweakly similar to ( 107) MDR_ORYSA Putative multidrug resistance protein (P-glycoprotein) - Oryza sativa (Rice)highly similar to ( 540) loc_os04g52900 12004.m35462 protein multidrug resistance-associated protein 2, putative, expressed  no original description 	T
16.1.5	secondary metabolism.isoprenoids.terpenoids	MDP0000084546	 nearly identical (1317) AT2G07050 | Symbols: CAS1 | CAS1 (cycloartenol synthase 1); cycloartenol synthase | chr2:2924629-2930295 FORWARDnearly identical (1220) loc_os02g04710 12002.m33547 protein cycloartenol synthase, putative, expressed  PRK08026  no original description 	T
27.1	RNA.processing	MDP0000303387	 moderately similar to ( 270) AT1G43190 | Symbols:  | polypyrimidine tract-binding protein, putative / heterogeneous nuclear ribonucleoprotein, putative | chr1:16275432-16278185 REVERSEmoderately similar to ( 239) loc_os01g64770 12001.m43519 protein retrotransposon protein, putative, unclassified, expressed   no original description 	T
29.5.11.4.2	protein.degradation.ubiquitin.E3.RING	MDP0000199837	 moderately similar to ( 304) AT5G14420 | Symbols: RGLG2 | RGLG2 (RING domain Ligase2); ubiquitin-protein ligase | chr5:4648355-4650563 REVERSEmoderately similar to ( 344) loc_os08g04130 12008.m080076 protein copine-6, putative, expressed  no original description 	T
1.1.1.2	PS.lightreaction.photosystem II.PSII polypeptide subunits	MDP0000210295	 weakly similar to ( 193) AT4G28660 | Symbols: PSB28 | PSB28 (PHOTOSYSTEM II REACTION CENTER PSB28 PROTEIN) | chr4:14150008-14150933 FORWARDmoderately similar to ( 222) loc_os01g71190 12001.m13162 protein photosystem II reaction center W protein, putative, expressed  no original description 	T
34.12	transport.metal	MDP0000273257	 moderately similar to ( 340) AT3G12750 | Symbols: ZIP1 | ZIP1 (ZINC TRANSPORTER 1 PRECURSOR); zinc ion transmembrane transporter | chr3:4051950-4053156 REVERSEmoderately similar to ( 348) loc_os07g12890 12007.m28960 protein ZIP zinc/iron transport family protein, expressed no original description 	T
29.1.7	protein.aa activation.alanine-tRNA ligase	MDP0000175783	 nearly identical (1423) AT1G50200 | Symbols: ALATS, ACD | ALATS (ALANYL-TRNA SYNTHETASE); ATP binding / alanine-tRNA ligase/ ligase, forming aminoacyl-tRNA and related compounds / nucleic acid binding / nucleotide binding | chr1:18591429-18598311 REVERSEnearly identical (1254) loc_os10g10244 12010.m04361 protein alanyl-tRNA synthetase, mitochondrial precursor, putative, expressed  no original description 	T
27.3.32	RNA.regulation of transcription.WRKY domain transcription factor family	MDP0000169621	 moderately similar to ( 339) AT2G37260 | Symbols: TTG2, ATWRKY44, WRKY44 | TTG2 (TRANSPARENT TESTA GLABRA 2); transcription factor | chr2:15645603-15646792 FORWARDmoderately similar to ( 246) loc_os07g39480 12007.m08190 protein OsWRKY78 - Superfamily of rice TFs having WRKY and zinc finger domains, expressed   no original description 	T
15.2	metal handling.binding, chelation and storage	MDP0000247193	 weakly similar to ( 122) AT3G29130 | Symbols:  | unknown protein | chr3:11102546-11103294 REVERSEweakly similar to ( 106) loc_os03g43910 12003.m09432 protein expressed protein  no original description 	T
35.2	not assigned.unknown	MDP0000159103	 weakly similar to ( 112) AT5G64400 | Symbols:  | unknown protein | chr5:25748922-25749947 FORWARDweakly similar to ( 122) loc_os03g48080 12003.m09819 protein p8MTCP1, putative, expressed   no original description 	T
30.5	signalling.G-proteins	MDP0000272050	 moderately similar to ( 406) AT5G55190 | Symbols: RAN3, ATRAN3 | RAN3 (RAN GTPASE 3); GTP binding / GTPase/ protein binding | chr5:22392285-22393957 FORWARDmoderately similar to ( 406) RANB1_TOBAC GTP-binding nuclear protein Ran-B1 - Nicotiana tabacum (Common tobacco)moderately similar to ( 398) loc_os01g42530 12001.m10517 protein GTP-binding nuclear protein Ran-2, putative, expressed  no original description 	T
27.3.25	RNA.regulation of transcription.MYB domain transcription factor family	MDP0000259898	 moderately similar to ( 313) AT1G48830 | Symbols:  | 40S ribosomal protein S7 (RPS7A) | chr1:18059854-18060935 REVERSEmoderately similar to ( 312) RS7_ORYSA 40S ribosomal protein S7 - Oryza sativa (Rice)moderately similar to ( 318) loc_os03g18580 12003.m07280 protein 40S ribosomal protein S7, putative, expressed   no original description 	T
13.1.3.2.1	amino acid metabolism.synthesis.aspartate family.threonine.threonine synthase	MDP0000605118	 highly similar to ( 797) AT4G29840 | Symbols: MTO2, TS | MTO2 (METHIONINE OVER-ACCUMULATOR 2); threonine synthase | chr4:14599434-14601014 REVERSEhighly similar to ( 806) THRC_SOLTU Threonine synthase, chloroplast precursor (EC 4.2.3.1) (TS) - Solanum tuberosum (Potato)highly similar to ( 722) loc_os05g47640 12005.m08836 protein threonine synthase, chloroplast precursor, putative, expressed  no original description 	T
27.3.24	RNA.regulation of transcription.MADS box transcription factor family	MDP0000249592	 weakly similar to ( 166) AT5G62165 | Symbols: AGL42 | AGL42 (AGAMOUS LIKE 42); transcription factor | chr5:24965075-24968437 FORWARDweakly similar to ( 147) MAD50_ORYSA MADS-box transcription factor 50 (OsMADS50) (Protein SUPPRESSOR OF CONSTANS OVEREXPRESSION 1-like) (OsSOC1) (Protein AGAMOUS-like 20) (RMADS208) - Oryza sativa (Rice)weakly similar to ( 142) loc_os10g39130 12010.m21952 protein MADS-box transcription factor 56, putative, expressed no original description 	T
27.3.57	RNA.regulation of transcription.JUMONJI family	MDP0000244814	 highly similar to ( 791) AT5G46910 | Symbols:  | transcription factor jumonji (jmj) family protein | chr5:19047780-19050880 FORWARDhighly similar to ( 688) loc_os10g42690 12010.m07021 protein jmjC domain containing protein, expressed   no original description 	T
21.1	redox.thioredoxin	MDP0000142188	 moderately similar to ( 333) AT1G34780 | Symbols: ATAPRL4 | ATAPRL4 (APR-like 4) | chr1:12748835-12750124 REVERSEmoderately similar to ( 290) loc_os02g51850 12002.m33878 protein OsAPRL3 - Oryza sativa adenosine 5'-phosphosulfate reductase-like, expressed  no original description 	T
30.7	signalling.14-3-3 proteins	MDP0000147337	 moderately similar to ( 456) AT5G38480 | Symbols: GRF3, RCI1 | GRF3 (GENERAL REGULATORY FACTOR 3); ATP binding / protein binding / protein phosphorylated amino acid binding | chr5:15410277-15411285 FORWARDmoderately similar to ( 468) 1433A_SOYBN 14-3-3-like protein A (SGF14A) - Glycine max (Soybean)moderately similar to ( 457) loc_os08g33370 12008.m26501 protein 14-3-3-like protein, putative, expressed   no original description 	T
29.3.1	protein.targeting.nucleus	MDP0000347057	 very weakly similar to (72.8) AT5G43960 | Symbols:  | nuclear transport factor 2 (NTF2) family protein / RNA recognition motif (RRM)-containing protein | chr5:17689154-17691653 REVERSEvery weakly similar to (60.8) loc_os02g03890 12002.m05738 protein RNA binding protein, putative, expressed  no original description 	T
35.2	not assigned.unknown	MDP0000255781	  no original description 	T
15.2	metal handling.binding, chelation and storage	MDP0000153123	 very weakly similar to (55.5) AT3G51790 | Symbols: ATG1 | ATG1 (ARABIDOPSIS TRANSMEMBRANE PROTEIN G1P-RELATED 1) | chr3:19209893-19210663 REVERSEweakly similar to ( 110) MT2_MALDO Metallothionein-like protein type 2 - Malus domestica (Apple) (Malus sylvestris)very weakly similar to (71.2) loc_os01g05650 12001.m07196 protein metallothionein-like protein type 2, putative, expressed  no original description 	T
29.2.3	protein.synthesis.initiation	MDP0000336815	 moderately similar to ( 238) AT3G55620 | Symbols: emb1624 | emb1624 (embryo defective 1624); ribosome binding / translation initiation factor | chr3:20634581-20636312 FORWARDmoderately similar to ( 219) IF6_BETVU Eukaryotic translation initiation factor 6 (eIF-6) (Fragment) - Beta vulgaris (Sugar beet)moderately similar to ( 234) loc_os07g44620 12007.m29287 protein eukaryotic translation initiation factor 6, putative, expressed  no original description 	T
35.2	not assigned.unknown	MDP0000371891	 weakly similar to ( 163) AT5G01130 | Symbols:  | unknown protein | chr5:47642-49174 FORWARDweakly similar to ( 128) loc_os04g50800 12004.m09977 protein expressed protein  no original description 	T
11.3	lipid metabolism.Phospholipid synthesis	MDP0000447222	 moderately similar to ( 342) AT1G06520 | Symbols: ATGPAT1, GPAT1 | GPAT1 (GLYCEROL-3-PHOSPHATE ACYLTRANSFERASE 1); 1-acylglycerol-3-phosphate O-acyltransferase/ acyltransferase | chr1:1994170-1996067 REVERSEmoderately similar to ( 316) loc_os01g44069 12001.m10665 protein glycerol-3-phosphate acyltransferase 1, putative, expressed  no original description 	T
35.1	not assigned.no ontology	MDP0000231493	 highly similar to ( 590) AT3G50780 | Symbols:  | INVOLVED IN: biological_process unknown; LOCATED IN: cellular_component unknown; EXPRESSED IN: 22 plant structures; EXPRESSED DURING: 13 growth stages; BEST Arabidopsis thaliana protein match is: PRLI-interacting factor-related (TAIR:AT1G63850.1); Has 173 Blast hits to 173 proteins in 14 species: Archae - 0; Bacteria - 0; Metazoa - 8; Fungi - 0; Plants - 164; Viruses - 0; Other Eukaryotes - 1 (source: NCBI BLink). | chr3:18875662-18877341 REVERSEhighly similar to ( 525) loc_os03g55950 12003.m101512 protein protein binding protein, putative, expressed   no original description 	T
29.2.1.2.2.24	protein.synthesis.ribosomal protein.eukaryotic.60S subunit.L24	MDP0000948784	 moderately similar to ( 234) AT2G44860 | Symbols:  | 60S ribosomal protein L24, putative | chr2:18501034-18502128 REVERSEvery weakly similar to (77.4) RL24_CICAR 60S ribosomal protein L24 - Cicer arietinum (Chickpea) (Garbanzo)moderately similar to ( 225) loc_os07g19190 12007.m06356 protein ribosome biogenesis protein RLP24, putative, expressed no original description 	T
28.1.3	DNA.synthesis/chromatin structure.histone	MDP0000831145	 moderately similar to ( 204) AT3G54560 | Symbols: HTA11 | HTA11; DNA binding | chr3:20196532-20197466 FORWARDmoderately similar to ( 202) H2AV1_ORYSA Probable histone H2A variant 1 - Oryza sativa (Rice)moderately similar to ( 202) loc_os03g06670 12003.m06185 protein histone H2A variant 1, putative, expressed  no original description 	T
10.2	cell wall.cellulose synthesis	MDP0000297462	 highly similar to ( 625) AT4G31590 | Symbols: ATCSLC05, CSLC05, ATCSLC5 | ATCSLC5 (CELLULOSE-SYNTHASE LIKE C5); cellulose synthase/ transferase, transferring glycosyl groups | chr4:15309889-15312336 REVERSEmoderately similar to ( 479) loc_os08g15420 12008.m05665 protein CSLC3 - cellulose synthase-like family C, expressed   no original description 	T
35.2	not assigned.unknown	MDP0000334502	  no original description 	T
35.1	not assigned.no ontology	MDP0000329524	 moderately similar to ( 286) AT1G10950 | Symbols:  | endomembrane protein 70, putative | chr1:3659322-3663622 FORWARDmoderately similar to ( 256) loc_os11g07910 12011.m28746 protein transmembrane 9 superfamily protein member 3 precursor, putative, expressed  no original description 	T
34.15	transport.potassium	MDP0000176949	 moderately similar to ( 233) AT5G55000 | Symbols: FIP2 | FIP2; protein binding / voltage-gated potassium channel | chr5:22318644-22321435 FORWARDweakly similar to ( 200) loc_os03g06160 12003.m06136 protein potassium channel tetramerisation domain-containing protein 9, putative, expressed  no original description 	T
27.1	RNA.processing	MDP0000364275	 weakly similar to ( 148) AT4G30220 | Symbols: RUXF | RUXF (SMALL NUCLEAR RIBONUCLEOPROTEIN F) | chr4:14803100-14804259 REVERSEweakly similar to ( 147) loc_os11g43620 12011.m28864 protein small nuclear ribonucleoprotein F, putative, expressed no original description 	T
29.5.11.4.3.2	protein.degradation.ubiquitin.E3.SCF.FBOX	MDP0000854534	 moderately similar to ( 332) AT5G67140 | Symbols:  | F-box family protein | chr5:26794009-26795213 REVERSEmoderately similar to ( 274) loc_os06g07000 12006.m31842 protein F-box domain containing protein, expressed  no original description 	T
27.3.11	RNA.regulation of transcription.C2H2 zinc finger family	MDP0000179049	 moderately similar to ( 470) AT1G75710 | Symbols:  | zinc finger (C2H2 type) family protein | chr1:28428806-28431128 FORWARDmoderately similar to ( 301) loc_os04g02510 12004.m05563 protein nucleic acid binding protein, putative, expressed  no original description 	T
3.5	minor CHO metabolism.others	MDP0000408705	 moderately similar to ( 470) AT2G21250 | Symbols:  | mannose 6-phosphate reductase (NADPH-dependent), putative | chr2:9103408-9105116 REVERSEhighly similar to ( 624) S6PD_MALDO NADP-dependent D-sorbitol-6-phosphate dehydrogenase (EC 1.1.1.200) (Aldose-6-phosphate reductase [NADPH]) (NADP-S6PDH) - Malus domestica (Apple) (Malus sylvestris)moderately similar to ( 452) loc_os02g03100 12002.m05660 protein NADP-dependent D-sorbitol-6-phosphate dehydrogenase, putative, expressed  no original description 	T
26.21	misc.protease inhibitor/seed storage/lipid transfer protein (LTP) family protein	MDP0000361511	 weakly similar to ( 129) AT3G22142 | Symbols:  | structural constituent of cell wall | chr3:7803604-7808046 REVERSEvery weakly similar to (85.5) 14KD_DAUCA 14 kDa proline-rich protein DC2.15 precursor - Daucus carota (Carrot)weakly similar to ( 115) loc_os06g07220 12006.m05445 protein 36.4 kDa proline-rich protein, putative, expressed  no original description 	T
29.2.2.3.1	protein.synthesis.ribosome biogenesis.Pre-rRNA processing and modifications.snoRNPs	MDP0000210372	 weakly similar to ( 184) AT3G03920 | Symbols:  | Gar1 RNA-binding region family protein | chr3:1009123-1010379 REVERSEweakly similar to ( 189) loc_os11g37080 12011.m07556 protein h/ACA ribonucleoprotein complex subunit 1-like protein 1, putative, expressed no original description 	T
11.8.10	lipid metabolism.'exotics' (steroids, squalene etc).phosphatidylcholinesterol O-acyltransferase	MDP0000188724	 highly similar to ( 578) AT3G03310 | Symbols:  | lecithin:cholesterol acyltransferase family protein / LACT family protein | chr3:778767-781488 REVERSEmoderately similar to ( 449) loc_os09g33820 12009.m22221 protein phospholipase A1, putative, expressed  no original description 	T
34.10	transport.nucleotides	MDP0000320796	 highly similar to ( 756) AT2G34410 | Symbols:  | O-acetyltransferase family protein | chr2:14518719-14522053 FORWARDhighly similar to ( 706) loc_os01g44040 12001.m150664 protein O-acetyltransferase, putative, expressed   no original description 	T
29.5.11.3	protein.degradation.ubiquitin.E2	MDP0000192099	 moderately similar to ( 296) AT5G41700 | Symbols: UBC8, ATUBC8 | UBC8 (UBIQUITIN CONJUGATING ENZYME 8); protein binding / ubiquitin-protein ligase | chr5:16676072-16677223 FORWARDweakly similar to ( 141) UBC2_WHEAT Ubiquitin-conjugating enzyme E2-17 kDa (EC 6.3.2.19) (Ubiquitin-protein ligase) (Ubiquitin carrier protein) - Triticum aestivum (Wheat)moderately similar to ( 293) loc_os04g57220 12004.m101686 protein ubiquitin-conjugating enzyme E2-17 kDa, putative, expressed  no original description 	T
35.2	not assigned.unknown	MDP0000197338	 very weakly similar to (62.0) AT5G03120 | Symbols:  | unknown protein | chr5:734062-734683 FORWARD no original description 	T
1.3.2	PS.calvin cycle.rubisco small subunit	MDP0000252890	 moderately similar to ( 279) AT5G38410 | Symbols:  | ribulose bisphosphate carboxylase small chain 3B / RuBisCO small subunit 3B (RBCS-3B) (ATS3B) | chr5:15377501-15378306 REVERSEmoderately similar to ( 291) RBS_BETVE Ribulose bisphosphate carboxylase small chain, chloroplast precursor (EC 4.1.1.39) (RuBisCO small subunit) - Betula verrucosa (White birch) (Betula pendula)moderately similar to ( 231) loc_os12g19394 12012.m73909 protein ribulose bisphosphate carboxylase small chain C, chloroplast precursor, putative, expressed  no original description 	T
27.1.1	RNA.processing.splicing	MDP0000265130	 highly similar to ( 767) AT5G64270 | Symbols:  | splicing factor, putative | chr5:25706909-25710718 FORWARDhighly similar to ( 765) loc_os02g05410 12002.m05889 protein splicing factor 3B subunit 1, putative, expressed  no original description 	T
29.8	protein.assembly and cofactor ligation	MDP0000952041	 moderately similar to ( 288) AT4G25910 | Symbols: NFU3, ATCNFU3 | NFU3; structural molecule | chr4:13164128-13165094 FORWARDmoderately similar to ( 207) NIFU1_ORYSA NifU-like protein 1, chloroplast precursor (OsNifu1) - Oryza sativa (Rice)moderately similar to ( 259) loc_os06g47940 12006.m09320 protein NFU3, putative, expressed no original description 	T
10.1.4	cell wall.precursor synthesis.UGD	MDP0000146452	 highly similar to ( 876) AT3G29360 | Symbols:  | UDP-glucose 6-dehydrogenase, putative | chr3:11267375-11268817 REVERSEhighly similar to ( 885) UGDH_SOYBN UDP-glucose 6-dehydrogenase (EC 1.1.1.22) (UDP-Glc dehydrogenase) (UDP-GlcDH) (UDPGDH) - Glycine max (Soybean)highly similar to ( 883) loc_os12g25700 12012.m26903 protein UDP-glucose 6-dehydrogenase, putative, expressed  no original description 	T
35.1	not assigned.no ontology	MDP0000189628	 highly similar to ( 649) AT5G07830 | Symbols: AtGUS2 | AtGUS2 (Arabidopsis thaliana glucuronidase 2); beta-glucuronidase | chr5:2504168-2506567 FORWARDhighly similar to ( 623) loc_os12g38920 12012.m073868 protein heparanase-like protein 2 precursor, putative, expressed  no original description 	T
35.1	not assigned.no ontology	MDP0000235688	 moderately similar to ( 213) AT2G39795 | Symbols:  | mitochondrial glycoprotein family protein / MAM33 family protein | chr2:16597026-16598028 FORWARDmoderately similar to ( 223) loc_os01g05010 12001.m07135 protein mitochondrial glycoprotein, expressed no original description 	T
35.2	not assigned.unknown	MDP0000290504	 moderately similar to ( 495) AT1G43580 | Symbols:  | unknown protein | chr1:16410673-16412612 REVERSEmoderately similar to ( 497) loc_os04g34620 12004.m08556 protein expressed protein   no original description 	T
29.5.1	protein.degradation.subtilases	MDP0000327253	 weakly similar to ( 129) AT1G71950 | Symbols:  | identical protein binding / serine-type endopeptidase | chr1:27080453-27081573 REVERSEweakly similar to ( 119) loc_os05g35740 12005.m27840 protein Pi starvation-induced protein, putative, expressed  no original description 	T
27.1.1	RNA.processing.splicing	MDP0000122095	 weakly similar to ( 145) AT4G14342 | Symbols:  | pre-mRNA splicing factor 10 kDa subunit, putative | chr4:8253789-8255405 REVERSEweakly similar to ( 147) loc_os08g34480 12008.m07437 protein splicing factor 3B subunit 5, putative, expressed no original description 	T
26.9	misc.glutathione S transferases	MDP0000286249	 moderately similar to ( 263) AT3G62760 | Symbols: ATGSTF13 | ATGSTF13; glutathione transferase | chr3:23217425-23218246 REVERSEmoderately similar to ( 215) GSTH1_ORYSA Probable glutathione S-transferase GSTF1 (EC 2.5.1.18) (GST-I) - Oryza sativa (Rice)moderately similar to ( 228) loc_os01g27260 12001.m09177 protein glutathione S-transferase GSTF1, putative, expressed no original description 	T
29.2.1.2.1.18	protein.synthesis.ribosomal protein.eukaryotic.40S subunit.S18	MDP0000127145	 moderately similar to ( 256) AT4G09800 | Symbols: RPS18C | RPS18C (S18 RIBOSOMAL PROTEIN); RNA binding / nucleic acid binding / structural constituent of ribosome | chr4:6173818-6174963 FORWARDweakly similar to ( 200) RS18_CHLRE 40S ribosomal protein S18 - Chlamydomonas reinhardtiimoderately similar to ( 244) loc_os07g07709 12007.m29370 protein 40S ribosomal protein S18, putative, expressed  no original description 	T
35.2	not assigned.unknown	MDP0000259212	 highly similar to ( 690) AT3G15160 | Symbols:  | unknown protein | chr3:5104582-5108279 FORWARDhighly similar to ( 653) loc_os04g53530 12004.m10245 protein expressed protein no original description 	T
35.1	not assigned.no ontology	MDP0000263417	 moderately similar to ( 334) AT4G36090 | Symbols:  | oxidoreductase, 2OG-Fe(II) oxygenase family protein | chr4:17078376-17080670 REVERSEmoderately similar to ( 338) loc_os06g04660 12006.m091594 protein oxidoreductase, 2OG-Fe oxygenase family protein, expressed  no original description 	T
1.1.1.1	PS.lightreaction.photosystem II.LHC-II	MDP0000708928	 moderately similar to ( 468) AT2G05070 | Symbols: LHCB2.2, LHCB2 | LHCB2.2; chlorophyll binding | chr2:1799436-1800329 REVERSEmoderately similar to ( 474) CB21_GOSHI Chlorophyll a-b binding protein 151, chloroplast precursor (LHCII type II CAB-151) (LHCP) - Gossypium hirsutum (Upland cotton)moderately similar to ( 443) loc_os03g39610 12003.m35304 protein chlorophyll a-b binding protein, chloroplast precursor, putative, expressed  no original description 	T
35.2	not assigned.unknown	MDP0000260682	 moderately similar to ( 431) AT5G42810 | Symbols: ATIPK1 | ATIPK1 (Inositol-pentakisphosphate 2-kinase 1); inositol pentakisphosphate 2-kinase/ inositol tetrakisphosphate 2-kinase | chr5:17167073-17169337 FORWARDmoderately similar to ( 383) loc_os04g56580 12004.m10547 protein expressed protein  no original description 	T
30.5	signalling.G-proteins	MDP0000876872	 weakly similar to ( 188) AT3G15680 | Symbols:  | zinc finger (Ran-binding) family protein | chr3:5315437-5316048 FORWARDweakly similar to ( 167) loc_os02g10920 12002.m100134 protein RNA-binding protein cabeza, putative, expressed   no original description 	T
31.1	cell.organisation	MDP0000921834	 highly similar to ( 719) AT3G53750 | Symbols: ACT3 | ACT3 (actin 3); structural constituent of cytoskeleton | chr3:19915924-19917371 FORWARDhighly similar to ( 726) ACT_GOSHI Actin - Gossypium hirsutum (Upland cotton)highly similar to ( 721) loc_os03g50890 12003.m10074 protein actin-1, putative, expressed no original description 	T
26.4.1	misc.beta 1,3 glucan hydrolases.glucan endo-1,3-beta-glucosidase	MDP0000716700	 highly similar to ( 637) AT5G58480 | Symbols:  | glycosyl hydrolase family 17 protein | chr5:23641227-23642826 REVERSEweakly similar to ( 190) E13H_TOBAC Glucan endo-1,3-beta-glucosidase, acidic isoform PR-Q' precursor (EC 3.2.1.39) ((1->3)-beta-glucan endohydrolase) ((1->3)-beta-glucanase) (Beta-1,3-endoglucanase) (PR-35) - Nicotiana tabacum (Common tobacco)highly similar to ( 521) loc_os03g18520 12003.m07274 protein glucan endo-1,3-beta-glucosidase 5 precursor, putative, expressed    no original description 	T
20.1.7	stress.biotic.PR-proteins	MDP0000266801	 highly similar to ( 524) AT5G17680 | Symbols:  | disease resistance protein (TIR-NBS-LRR class), putative | chr5:5822999-5827153 FORWARDweakly similar to ( 108) loc_os01g15580 12001.m08156 protein disease resistance protein RGA2, putative, expressed  no original description 	T
26.28	misc.GDSL-motif lipase	MDP0000285848	 weakly similar to ( 125) AT1G28580 | Symbols:  | GDSL-motif lipase, putative | chr1:10044603-10045874 REVERSEvery weakly similar to (79.0) EST_HEVBR Esterase precursor (EC 3.1.1.-) (Early nodule-specific protein homolog) (Latex allergen Hev b 13) - Hevea brasiliensis (Para rubber tree)weakly similar to ( 115) loc_os01g11710 12001.m07788 protein esterase precursor, putative, expressed  no original description 	T
30.8	signalling.misc	MDP0000132257	 weakly similar to ( 117) AT5G67070 | Symbols: RALFL34 | RALFL34 (ralf-like 34); signal transducer | chr5:26768120-26768509 FORWARDvery weakly similar to (50.4) loc_os01g10470 12001.m07668 protein RALF precursor, putative, expressed  no original description 	T
34.9	transport.metabolite transporters at the mitochondrial membrane	MDP0000175314	 highly similar to ( 618) AT3G08580 | Symbols: AAC1 | AAC1 (ADP/ATP CARRIER 1); ATP:ADP antiporter/ binding | chr3:2605706-2607030 REVERSEhighly similar to ( 636) ADT1_GOSHI ADP,ATP carrier protein 1, mitochondrial precursor (ADP/ATP translocase 1) (Adenine nucleotide translocator 1) (ANT 1) - Gossypium hirsutum (Upland cotton)highly similar to ( 568) loc_os02g48720 12002.m33839 protein ADP,ATP carrier protein, mitochondrial precursor, putative, expressed no original description 	T
20.2.3	stress.abiotic.drought/salt	MDP0000315740	 highly similar to ( 576) AT5G64030 | Symbols:  | dehydration-responsive protein-related | chr5:25624965-25628257 FORWARDhighly similar to ( 573) loc_os01g66110 12001.m42777 protein ankyrin-like protein, putative, expressed  no original description 	T
30.5	signalling.G-proteins	MDP0000230637	 moderately similar to ( 232) AT1G16920 | Symbols: RAB11, ATRABA1B | ATRABA1B (ARABIDOPSIS RAB GTPASE HOMOLOG A1B); GTP binding | chr1:5787489-5789147 REVERSEmoderately similar to ( 231) RIC2_ORYSA Ras-related protein RIC2 - Oryza sativa (Rice)moderately similar to ( 235) loc_os01g54590 12001.m11622 protein ras-related protein RIC2, putative, expressed  no original description 	T
31.3	cell.cycle	MDP0000818047	 weakly similar to ( 150) AT2G27960 | Symbols: CKS1, CKS1AT | CKS1 (CYCLIN-DEPENDENT KINASE-SUBUNIT 1); cyclin-dependent protein kinase/ protein binding | chr2:11910903-11911543 REVERSEweakly similar to ( 150) loc_os03g05300 12003.m06054 protein cyclin-dependent kinases regulatory subunit, putative, expressed  no original description 	T
35.2	not assigned.unknown	MDP0000757026	 weakly similar to ( 161) AT4G02360 | Symbols:  | unknown protein | chr4:1041179-1041643 FORWARDweakly similar to ( 145) loc_os05g29920 12005.m07228 protein expressed protein   no original description 	T
16.8.3.1	secondary metabolism.flavonoids.dihydroflavonols.dihydroflavonol 4-reductase	MDP0000706020	 moderately similar to ( 248) AT2G23910 | Symbols:  | cinnamoyl-CoA reductase-related | chr2:10177902-10179789 FORWARDweakly similar to ( 106) DFRA_VITVI Dihydroflavonol-4-reductase (EC 1.1.1.219) (DFR) (Dihydrokaempferol 4-reductase) - Vitis vinifera (Grape)moderately similar to ( 277) loc_os10g42620 12010.m07015 protein dihydroflavonol-4-reductase, putative, expressed   no original description 	T
29.5.11	protein.degradation.ubiquitin	MDP0000039464	 moderately similar to ( 447) AT3G56740 | Symbols:  | ubiquitin-associated (UBA)/TS-N domain-containing protein | chr3:21015631-21017883 FORWARDmoderately similar to ( 345) loc_os01g16330 12001.m08229 protein UBA/TS-N domain, putative, expressed  no original description 	T
35.2	not assigned.unknown	MDP0000154430	  no original description 	T
35.2	not assigned.unknown	MDP0000319966	 moderately similar to ( 397) AT4G28100 | Symbols:  | unknown protein | chr4:13965300-13966697 REVERSEmoderately similar to ( 300) loc_os07g37156 12007.m79903 protein expressed protein   no original description 	T
26.28	misc.GDSL-motif lipase	MDP0000927927	 moderately similar to ( 405) AT5G45910 | Symbols:  | GDSL-motif lipase/hydrolase family protein | chr5:18620420-18622264 REVERSEweakly similar to ( 176) EST_HEVBR Esterase precursor (EC 3.1.1.-) (Early nodule-specific protein homolog) (Latex allergen Hev b 13) - Hevea brasiliensis (Para rubber tree)moderately similar to ( 407) loc_os01g11710 12001.m07788 protein esterase precursor, putative, expressed  no original description 	T
4.1.10	glycolysis.cytosolic branch.aldolase	MDP0000266003	 highly similar to ( 603) AT2G36460 | Symbols:  | fructose-bisphosphate aldolase, putative | chr2:15296929-15297732 REVERSEhighly similar to ( 606) ALF_ORYSA Fructose-bisphosphate aldolase cytoplasmic isozyme (EC 4.1.2.13) (Gravity-specific protein GSC 233) - Oryza sativa (Rice)highly similar to ( 606) loc_os05g33380 12005.m07570 protein fructose-bisphosphate aldolase cytoplasmic isozyme, putative, expressed   no original description 	T
29.5.5	protein.degradation.serine protease	MDP0000319039	 moderately similar to ( 280) AT5G38510 | Symbols:  | rhomboid family protein | chr5:15417839-15420002 REVERSEweakly similar to ( 174) loc_os02g22100 12002.m100193 protein rhomboid family protein, expressed  no original description 	T
29.3.2	protein.targeting.mitochondria	MDP0000234728	 weakly similar to ( 196) AT5G24650 | Symbols:  | mitochondrial import inner membrane translocase subunit Tim17/Tim22/Tim23 family protein | chr5:8437123-8438859 FORWARDmoderately similar to ( 214) loc_os04g33220 12004.m08417 protein SAM domain family protein, expressed  no original description 	T
26.28	misc.GDSL-motif lipase	MDP0000194060	 highly similar to ( 566) AT3G04290 | Symbols: ATLTL1, LTL1 | LTL1 (LI-TOLERANT LIPASE 1); carboxylesterase/ hydrolase, acting on ester bonds | chr3:1133620-1136223 REVERSEweakly similar to ( 167) APG_BRANA Anter-specific proline-rich protein APG (Protein CEX) (Fragment) - Brassica napus (Rape)highly similar to ( 512) loc_os02g40440 12002.m09130 protein anther-specific proline-rich protein APG precursor, putative, expressed  no original description 	T
33.3	development.squamosa promoter binding like (SPL)	MDP0000263766	 moderately similar to ( 222) AT5G50570 | Symbols:  | squamosa promoter-binding protein, putative | chr5:20582555-20583791 REVERSEweakly similar to ( 117) SBP1_ANTMA Squamosa promoter-binding protein 1 - Antirrhinum majus (Garden snapdragon)moderately similar to ( 214) loc_os08g41940 12008.m08172 protein teosinte glume architecture 1, putative, expressed   no original description 	T
26.7	misc.oxidases - copper, flavone etc	MDP0000288411	 moderately similar to ( 496) AT3G03080 | Symbols:  | NADP-dependent oxidoreductase, putative | chr3:698530-700278 REVERSEmoderately similar to ( 440) loc_os12g12580 12012.m05231 protein NADP-dependent oxidoreductase P2, putative, expressed  no original description 	T
26.10	misc.cytochrome P450	MDP0000269215	 moderately similar to ( 234) AT4G36220 | Symbols: FAH1, CYP84A1 | FAH1 (FERULIC ACID 5-HYDROXYLASE 1); ferulate 5-hydroxylase/ monooxygenase | chr4:17137584-17139619 REVERSEmoderately similar to ( 215) C75A3_PETHY Flavonoid 3',5'-hydroxylase 2 (EC 1.14.13.88) (F3'5'H) (Cytochrome P450 75A3) (CYPLXXVA3) - Petunia hybrida (Petunia)moderately similar to ( 226) loc_os09g26960 12009.m05848 protein flavonoid 3-monooxygenase, putative, expressed   no original description 	T
33.99	development.unspecified	MDP0000323212	 weakly similar to ( 129) AT1G28330 | Symbols:  | DYL1 (DORMANCY-ASSOCIATED PROTEIN-LIKE 1) | chr1:9934134-9935216 REVERSEweakly similar to ( 162) 12KD_FRAAN Auxin-repressed 12.5 kDa protein - Fragaria ananassa (Strawberry)weakly similar to ( 124) loc_os11g44810 12011.m28867 protein auxin-repressed 12.5 kDa protein, putative, expressed  no original description 	T
29.4	protein.postranslational modification	MDP0000507097	 highly similar to ( 828) AT4G32250 | Symbols:  | protein kinase family protein | chr4:15570285-15572528 REVERSEvery weakly similar to (57.8) PSKR_DAUCA Phytosulfokine receptor precursor (EC 2.7.11.1) (Phytosulfokine LRR receptor kinase) - Daucus carota (Carrot)highly similar to ( 758) loc_os06g43270 12006.m08857 protein protein kinase domain containing protein, expressed no original description 	T
33.99	development.unspecified	MDP0000196004	 moderately similar to ( 214) AT3G22550 | Symbols:  | senescence-associated protein-related | chr3:7991827-7992805 REVERSEweakly similar to ( 152) loc_os03g46260 12003.m101430 protein expressed protein   no original description 	T
1.1.1.1	PS.lightreaction.photosystem II.LHC-II	MDP0000269859	 moderately similar to ( 430) AT1G61520 | Symbols: LHCA3 | LHCA3; chlorophyll binding | chr1:22700493-22701149 FORWARDweakly similar to ( 152) CB12_PETHY Chlorophyll a-b binding protein, chloroplast precursor (LHCI type II CAB) - Petunia hybrida (Petunia)moderately similar to ( 383) loc_os02g10390 12002.m33354 protein chlorophyll a-b binding protein 8, chloroplast precursor, putative, expressed   no original description 	T
31.1	cell.organisation	MDP0000408630	 weakly similar to ( 198) AT2G19770 | Symbols: PRF5 | PRF5 (PROFILIN5); actin binding / actin monomer binding | chr2:8519885-8521119 REVERSEmoderately similar to ( 235) PROF1_MALDO Profilin-1 (GD4-1) (Pollen allergen Mal d 4.0301) - Malus domestica (Apple) (Malus sylvestris)moderately similar to ( 205) loc_os06g05880 12006.m05314 protein profilin-2, putative, expressed no original description 	T
35.2	not assigned.unknown	MDP0000866408	 very weakly similar to (52.8) AT1G65720 | Symbols:  | unknown protein | chr1:24440471-24441013 REVERSE  no original description 	T
28.1.3.1	DNA.synthesis/chromatin structure.histone.H1	MDP0000202751	 weakly similar to ( 102) AT1G14900 | Symbols: HMGA | HMGA (HIGH MOBILITY GROUP A); DNA binding | chr1:5138665-5139353 REVERSEweakly similar to ( 136) HMGYA_SOYBN HMG-Y-related protein A (SB16A protein) - Glycine max (Soybean)weakly similar to ( 112) loc_os08g33190 12008.m07310 protein linker histone H1 and H5 family protein, expressed  no original description 	T
35.1	not assigned.no ontology	MDP0000256961	 moderately similar to ( 266) AT5G62720 | Symbols:  | integral membrane HPP family protein | chr5:25191969-25194845 FORWARDmoderately similar to ( 219) loc_os03g48030 12003.m09814 protein HPP, putative, expressed no original description 	T
11.8.2	lipid metabolism.'exotics' (steroids, squalene etc).methylsterol monooxygenase	MDP0000308230	 moderately similar to ( 480) AT1G07420 | Symbols: ATSMO2, SMO2-1 | SMO2-1 (STEROL 4-ALPHA-METHYL-OXIDASE 2-1); 4-alpha-methyl-delta7-sterol-4alpha-methyl oxidase/ C-4 methylsterol oxidase | chr1:2278918-2280033 FORWARDvery weakly similar to (57.8) SC5D_TOBAC Delta(7)-sterol-C5(6)-desaturase (EC 1.3.3.-) (Delta-7-C-5 sterol desaturase) (Delta(7)-sterol-C5-desaturase) - Nicotiana tabacum (Common tobacco)moderately similar to ( 422) loc_os07g01150 12007.m079590 protein C-4 methylsterol oxidase, putative, expressed no original description 	T
16.7	secondary metabolism.wax	MDP0000172788	 moderately similar to ( 500) AT1G02205 | Symbols: CER1 | CER1 (ECERIFERUM 1); octadecanal decarbonylase | chr1:418818-422154 FORWARDmoderately similar to ( 468) loc_os02g40784 12002.m34021 protein CER1, putative, expressed  no original description 	T
30.2.11	signalling.receptor kinases.leucine rich repeat XI	MDP0000207066	 highly similar to ( 754) AT5G48380 | Symbols:  | leucine-rich repeat family protein / protein kinase family protein | chr5:19604584-19606532 REVERSEmoderately similar to ( 306) PSKR_DAUCA Phytosulfokine receptor precursor (EC 2.7.11.1) (Phytosulfokine LRR receptor kinase) - Daucus carota (Carrot)highly similar to ( 746) loc_os05g34270 12005.m07658 protein BRASSINOSTEROID INSENSITIVE 1-associated receptor kinase 1 precursor, putative, expressed  no original description 	T
35.2	not assigned.unknown	MDP0000436748	 moderately similar to ( 496) AT2G46890 | Symbols:  | oxidoreductase, acting on the CH-CH group of donors | chr2:19266879-19268134 REVERSEmoderately similar to ( 420) loc_os04g01510 12004.m05468 protein membrane protein, putative, expressed  no original description 	T
29.5.5	protein.degradation.serine protease	MDP0000792008	 moderately similar to ( 358) AT3G27925 | Symbols: DEGP1, Deg1 | DEGP1 (DegP protease 1); serine-type endopeptidase/ serine-type peptidase | chr3:10366659-10368864 REVERSEmoderately similar to ( 360) loc_os05g49380 12005.m083815 protein protease Do-like 1, chloroplast precursor, putative, expressed   no original description 	T
10.2	cell wall.cellulose synthesis	MDP0000174127	 highly similar to ( 951) AT4G31590 | Symbols: ATCSLC05, CSLC05, ATCSLC5 | ATCSLC5 (CELLULOSE-SYNTHASE LIKE C5); cellulose synthase/ transferase, transferring glycosyl groups | chr4:15309889-15312336 REVERSEhighly similar to ( 847) loc_os08g15420 12008.m05665 protein CSLC3 - cellulose synthase-like family C, expressed  no original description 	T
29.2.1.1.1.1.9	protein.synthesis.ribosomal protein.prokaryotic.chloroplast.30S subunit.S9	MDP0000182439	 moderately similar to ( 350) AT3G49080 | Symbols:  | ribosomal protein S9 family protein | chr3:18194500-18196657 REVERSEvery weakly similar to (85.9) RR9_SPIOL 30S ribosomal protein S9, chloroplast precursor (Fragment) - Spinacia oleracea (Spinach)moderately similar to ( 324) loc_os07g46440 12007.m08861 protein ribosomal protein S9 containing protein, expressed   no original description 	T
34.11	transport.NDP-sugars at the ER	MDP0000134427	 highly similar to ( 588) AT1G14360 | Symbols: ATUTR3, UTR3 | UTR3 (UDP-GALACTOSE TRANSPORTER 3); pyrimidine nucleotide sugar transmembrane transporter | chr1:4911362-4913029 REVERSEhighly similar to ( 501) loc_os06g39260 12006.m08464 protein solute carrier family 35 member B1, putative, expressed  no original description 	T
29.5.11.1	protein.degradation.ubiquitin.ubiquitin	MDP0000289628	 weakly similar to ( 174) AT4G26840 | Symbols: SUM1, SUMO 1, SUMO1, ATSUMO1 | SUMO1 (SMALL UBIQUITIN-LIKE MODIFIER 1); protein binding / protein tag | chr4:13497466-13498458 FORWARDweakly similar to ( 160) SMT3_ORYSA Ubiquitin-like protein SMT3 - Oryza sativa (Rice)weakly similar to ( 160) loc_os01g68950 12001.m42793 protein ubiquitin-like protein SMT3, putative, expressed no original description 	T
29.2.4	protein.synthesis.elongation	MDP0000361047	 moderately similar to ( 385) AT5G60390 | Symbols:  | elongation factor 1-alpha / EF-1-alpha | chr5:24289226-24290675 FORWARDmoderately similar to ( 393) EF1A_TOBAC Elongation factor 1-alpha (EF-1-alpha) (Vitronectin-like adhesion protein 1) (PVN1) - Nicotiana tabacum (Common tobacco)moderately similar to ( 393) loc_os03g08060 12003.m101211 protein elongation factor 1-alpha, putative, expressed  no original description 	T
35.2	not assigned.unknown	MDP0000613304	 moderately similar to ( 201) AT3G07480 | Symbols:  | electron carrier/ iron-sulfur cluster binding | chr3:2389026-2389505 FORWARDweakly similar to ( 194) loc_os09g33950 12009.m06444 protein ferredoxin, putative, expressed  no original description 	T
10.7	cell wall.modification	MDP0000147368	 weakly similar to ( 111) AT2G20750 | Symbols: ATEXPB1, EXPB1, ATHEXP BETA 1.5 | ATEXPB1 (ARABIDOPSIS THALIANA EXPANSIN B1) | chr2:8941185-8942430 FORWARDvery weakly similar to ( 100) EXB17_ORYSA Expansin-B17 precursor (OsEXPB17) (Beta-expansin-17) (OsaEXPb1.13) - Oryza sativa (Rice)very weakly similar to (99.0) loc_os02g42650 12002.m09300 protein beta-expansin 3 precursor, putative, expressed  no original description 	T
27.3.26	RNA.regulation of transcription.MYB-related transcription factor family	MDP0000221993	 moderately similar to ( 337) AT2G38090 | Symbols:  | myb family transcription factor | chr2:15945278-15946775 FORWARDmoderately similar to ( 238) loc_os05g37730 12005.m07952 protein MYB-like transcription factor DIVARICATA, putative, expressed  no original description 	T
35.1	not assigned.no ontology	MDP0000159376	 moderately similar to ( 455) AT1G70280 | Symbols:  | NHL repeat-containing protein | chr1:26466086-26468471 REVERSEmoderately similar to ( 465) loc_os02g49770 12002.m10011 protein expressed protein  no original description 	T
27.3.99	RNA.regulation of transcription.unclassified	MDP0000605874	 highly similar to ( 566) AT5G07030 | Symbols:  | aspartic-type endopeptidase | chr5:2183600-2185717 REVERSEmoderately similar to ( 452) loc_os07g46480 12007.m08865 protein aspartic proteinase nepenthesin-1 precursor, putative, expressed  no original description 	T
33.99	development.unspecified	MDP0000141458	 weakly similar to ( 121) AT1G53903 | Symbols:  | CONTAINS InterPro DOMAIN/s: Protein of unknown function DUF581 (InterPro:IPR007650); BEST Arabidopsis thaliana protein match is: senescence-associated protein-related (TAIR:AT1G53885.1); Has 277 Blast hits to 277 proteins in 14 species: Archae - 0; Bacteria - 0; Metazoa - 0; Fungi - 0; Plants - 277; Viruses - 0; Other Eukaryotes - 0 (source: NCBI BLink). | chr1:20132363-20132842 FORWARDvery weakly similar to (64.3) loc_os08g34984 12008.m80291 protein expressed protein no original description 	T
35.2	not assigned.unknown	MDP0000937221	 very weakly similar to (72.8) AT3G27520 | Symbols:  | unknown protein | chr3:10192484-10193159 FORWARD  no original description 	T
29.4.1.57	protein.postranslational modification.kinase.receptor like cytoplasmatic kinase VII	MDP0000270938	 highly similar to ( 934) AT5G48740 | Symbols:  | leucine-rich repeat family protein / protein kinase family protein | chr5:19765324-19769314 REVERSEmoderately similar to ( 247) NORK_MEDTR Nodulation receptor kinase precursor (EC 2.7.11.1) (Does not make infections protein 2) (Symbiosis receptor-like kinase) (MtSYMRK) - Medicago truncatula (Barrel medic)moderately similar to ( 349) loc_os12g37980 12012.m07584 protein senescence-induced receptor-like serine/threonine-protein kinase precursor, putative, expressed   no original description 	T
27.3.67	RNA.regulation of transcription.putative transcription regulator	MDP0000125070	 moderately similar to ( 254) AT2G29570 | Symbols: PCNA2, ATPCNA2 | PCNA2 (PROLIFERATING CELL NUCLEAR ANTIGEN 2); DNA binding / DNA polymerase processivity factor | chr2:12650139-12651598 REVERSEmoderately similar to ( 273) PCNA_SOYBN Proliferating cell nuclear antigen (PCNA) (Cyclin) (Fragment) - Glycine max (Soybean)moderately similar to ( 253) loc_os02g56130 12002.m10637 protein proliferating cell nuclear antigen, putative, expressed no original description 	T
33.99	development.unspecified	MDP0000246024	 moderately similar to ( 393) AT3G12090 | Symbols: TET6 | TET6 (TETRASPANIN6) | chr3:3852326-3853714 REVERSEmoderately similar to ( 385) loc_os05g03530 12005.m27685 protein senescence-associated protein-like, putative, expressed   no original description 	T
34.3	transport.amino acids	MDP0000296116	 highly similar to ( 575) AT2G39890 | Symbols: PROT1, ATPROT1 | PROT1 (PROLINE TRANSPORTER 1); L-proline transmembrane transporter/ amino acid transmembrane transporter | chr2:16656022-16658202 FORWARDvery weakly similar to (68.2) LECG_ARAHY Galactose-binding lectin precursor (Agglutinin) (PNA) - Arachis hypogaea (Peanut)highly similar to ( 545) loc_os07g01090 12007.m29032 protein proline transporter, putative, expressed  TrbL  no original description 	T
33.99	development.unspecified	MDP0000130123	 moderately similar to ( 283) AT5G13180 | Symbols: ANAC083 | ANAC083 (ARABIDOPSIS NAC DOMAIN CONTAINING PROTEIN 83); transcription factor | chr5:4196643-4197577 FORWARDweakly similar to ( 184) NAC68_ORYSA NAC domain-containing protein 68 (ONAC068) - Oryza sativa (Rice)weakly similar to ( 186) loc_os07g37920 12007.m08041 protein NAM-like protein, putative, expressed no original description 	T
27.3.36	RNA.regulation of transcription.Argonaute	MDP0000272708	 highly similar to ( 717) AT2G27040 | Symbols: AGO4, OCP11 | AGO4 (ARGONAUTE 4); nucleic acid binding | chr2:11536795-11541503 REVERSEvery weakly similar to (53.9) BABL_CUCSA Basic blue protein (Cusacyanin) (Plantacyanin) (CBP) - Cucumis sativus (Cucumber)highly similar to ( 728) loc_os01g16870 12001.m150417 protein argonaute-like protein, putative, expressed    no original description 	T
35.1	not assigned.no ontology	MDP0000255807	 weakly similar to ( 158) AT3G11780 | Symbols:  | MD-2-related lipid recognition domain-containing protein / ML domain-containing protein | chr3:3724326-3725476 REVERSEweakly similar to ( 155) loc_os03g57420 12003.m10660 protein ML domain protein, putative, expressed  no original description 	T
35.2	not assigned.unknown	MDP0000363287	  no original description 	T
35.1.41	not assigned.no ontology.hydroxyproline rich proteins	MDP0000130067	 weakly similar to ( 176) AT1G21090 | Symbols:  | hydroxyproline-rich glycoprotein family protein | chr1:7384854-7386199 FORWARD  no original description 	T
26.12	misc.peroxidases	MDP0000289293	 moderately similar to ( 415) AT2G24800 | Symbols:  | peroxidase, putative | chr2:10571255-10572570 REVERSEmoderately similar to ( 228) PER2_ARAHY Cationic peroxidase 2 precursor (EC 1.11.1.7) (PNPC2) - Arachis hypogaea (Peanut)moderately similar to ( 332) loc_os04g42100 12004.m09183 protein peroxidase 18 precursor, putative, expressed  no original description 	T
35.2	not assigned.unknown	MDP0000320406	 weakly similar to ( 152) AT1G11700 | Symbols:  | unknown protein | chr1:3945852-3946457 FORWARDvery weakly similar to (84.0) loc_os07g33270 12007.m07588 protein expressed protein   no original description 	T
11.6	lipid metabolism.lipid transfer proteins etc	MDP0000940078	 very weakly similar to (87.8) AT2G38540 | Symbols: LP1, LTP1, ATLTP1 | LP1; calmodulin binding | chr2:16130418-16130893 FORWARDvery weakly similar to (94.7) NLTP1_PRUPE Nonspecific lipid-transfer protein 1 (LTP 1) (Major allergen Pru p 3) (Pru p 1) - Prunus persica (Peach)very weakly similar to (79.7) loc_os11g02369 12011.m80079 protein nonspecific lipid-transfer protein 2 precursor, putative, expressed no original description 	T
35.2	not assigned.unknown	MDP0000120164	 moderately similar to ( 303) AT5G53160 | Symbols:  | unknown protein | chr5:21561026-21561953 FORWARDmoderately similar to ( 298) loc_os02g15640 12002.m06807 protein AT-rich element binding factor 3, putative, expressed  no original description 	T
35.2	not assigned.unknown	MDP0000298512	 nearly identical (1021) AT5G49830 | Symbols:  | EXPRESSED IN: 22 plant structures; EXPRESSED DURING: 13 growth stages; CONTAINS InterPro DOMAIN/s: Vps51/Vps67 (InterPro:IPR014812); BEST Arabidopsis thaliana protein match is: unknown protein (TAIR:AT1G10385.1). | chr5:20250803-20254766 REVERSEhighly similar to ( 918) loc_os07g10110 12007.m05466 protein expressed protein  no original description 	T
10.1.2	cell wall.precursor synthesis.UGE	MDP0000842286	 highly similar to ( 570) AT4G10960 | Symbols: UGE5 | UGE5 (UDP-D-glucose/UDP-D-galactose 4-epimerase 5); UDP-glucose 4-epimerase/ protein dimerization | chr4:6716083-6718472 REVERSEmoderately similar to ( 463) GALE1_PEA UDP-glucose 4-epimerase (EC 5.1.3.2) (Galactowaldenase) (UDP-galactose 4-epimerase) - Pisum sativum (Garden pea)highly similar to ( 559) loc_os05g51670 12005.m27995 protein UDP-glucose 4-epimerase GEPI48, putative, expressed  no original description 	T
29.5.1	protein.degradation.subtilases	MDP0000229839	 highly similar to ( 785) AT2G19170 | Symbols: SLP3 | SLP3; serine-type peptidase | chr2:8314154-8317620 REVERSEnearly identical (1083) loc_os06g48650 12006.m091776 protein SLP3, putative, expressed  no original description 	T
29.2.4	protein.synthesis.elongation	MDP0000679786	 weakly similar to ( 115) AT5G12110 | Symbols:  | elongation factor 1B alpha-subunit 1 (eEF1Balpha1) | chr5:3914483-3915732 FORWARDvery weakly similar to (95.1) EF1B_ORYSA Elongation factor 1-beta (EF-1-beta) (Elongation factor 1B-alpha 2) (eEF-1B alpha) (Elongation factor 1-beta') (EF-1-beta') - Oryza sativa (Rice)very weakly similar to (97.1) loc_os07g46750 12007.m08892 protein elongation factor 1-beta, putative, expressed  no original description 	T
29.1.22	protein.aa activation.asparagine-tRNA ligase	MDP0000746671	 highly similar to ( 868) AT5G56680 | Symbols: SYNC1, EMB2755, SYNC1 ARATH | SYNC1; ATP binding / aminoacyl-tRNA ligase/ asparagine-tRNA ligase/ aspartate-tRNA ligase/ nucleic acid binding / nucleotide binding | chr5:22936645-22938841 FORWARDhighly similar to ( 801) loc_os01g27520 12001.m43017 protein asparaginyl-tRNA synthetase, cytoplasmic 3, putative, expressed  no original description 	T
27.3.99	RNA.regulation of transcription.unclassified	MDP0000683912	 weakly similar to ( 194) AT1G51200 | Symbols:  | zinc finger (AN1-like) family protein | chr1:18985690-18986211 FORWARDweakly similar to ( 120) ISAP1_ORYSA Multiple stress-responsive zinc-finger protein ISAP1 (Stress-associated protein 1) (OsISAP1) - Oryza sativa (Rice)moderately similar to ( 220) loc_os06g41010 12006.m31898 protein zinc finger A20 and AN1 domains-containing protein, putative, expressed no original description 	T
34.6	transport.sulphate	MDP0000085223	 highly similar to ( 892) AT3G51895 | Symbols: SULTR3;1, AST12 | SULTR3;1 (SULFATE TRANSPORTER 3;1); secondary active sulfate transmembrane transporter/ sulfate transmembrane transporter/ transporter | chr3:19251503-19255677 REVERSEmoderately similar to ( 339) NO70_SOYBN Early nodulin 70 - Glycine max (Soybean)highly similar to ( 865) loc_os03g06520 12003.m06170 protein sulfate transporter 3.1, putative, expressed  no original description 	T
35.2	not assigned.unknown	MDP0000871895	 very weakly similar to (60.8) AT1G61260 | Symbols:  | unknown protein | chr1:22593756-22594986 REVERSEvery weakly similar to (63.5) loc_os07g37620 12007.m08012 protein fiber expressed protein, putative, expressed  no original description 	T
16.8.3.1	secondary metabolism.flavonoids.dihydroflavonols.dihydroflavonol 4-reductase	MDP0000077460	 moderately similar to ( 432) AT5G19440 | Symbols:  | cinnamyl-alcohol dehydrogenase, putative (CAD) | chr5:6556493-6558123 FORWARDmoderately similar to ( 232) DFRA_VITVI Dihydroflavonol-4-reductase (EC 1.1.1.219) (DFR) (Dihydrokaempferol 4-reductase) - Vitis vinifera (Grape)moderately similar to ( 401) loc_os01g34480 12001.m150871 protein dihydroflavonol-4-reductase, putative, expressed no original description 	T
13.1.4.1	amino acid metabolism.synthesis.branched chain group.common	MDP0000301987	 highly similar to ( 932) AT3G58610 | Symbols:  | ketol-acid reductoisomerase | chr3:21671561-21674639 FORWARDhighly similar to ( 933) ILV5_PEA Ketol-acid reductoisomerase, chloroplast precursor (EC 1.1.1.86) (Acetohydroxy-acid reductoisomerase) (Alpha-keto-beta-hydroxylacil reductoisomerase) - Pisum sativum (Garden pea)highly similar to ( 904) loc_os05g49800 12005.m09050 protein ketol-acid reductoisomerase, chloroplast precursor, putative, expressed  no original description 	T
30.2.11	signalling.receptor kinases.leucine rich repeat XI	MDP0000318241	 moderately similar to ( 476) AT2G34930 | Symbols:  | disease resistance family protein | chr2:14737169-14739886 REVERSEmoderately similar to ( 209) RPK1_IPONI Receptor-like protein kinase precursor (EC 2.7.11.1) - Ipomoea nil (Japanese morning glory) (Pharbitis nil)moderately similar to ( 479) loc_os01g41750 12001.m10443 protein leucine-rich repeat receptor protein kinase EXS precursor, putative, expressed no original description 	T
35.2	not assigned.unknown	MDP0000126996	    no original description 	T
29.3.4.4	protein.targeting.secretory pathway.plasma membrane	MDP0000261377	 highly similar to ( 513) AT2G30050 | Symbols:  | transducin family protein / WD-40 repeat family protein | chr2:12825540-12826448 FORWARDvery weakly similar to (56.6) GBB_ORYSA Guanine nucleotide-binding protein subunit beta - Oryza sativa (Rice)moderately similar to ( 495) loc_os02g04320 12002.m33334 protein SEC13-related protein, putative, expressed  no original description 	T
20.1.7	stress.biotic.PR-proteins	MDP0000291677	 moderately similar to ( 466) AT5G17680 | Symbols:  | disease resistance protein (TIR-NBS-LRR class), putative | chr5:5822999-5827153 FORWARDweakly similar to ( 129) loc_os01g15580 12001.m08156 protein disease resistance protein RGA2, putative, expressed  no original description 	T
27.3.7	RNA.regulation of transcription.C2C2(Zn) CO-like, Constans-like zinc finger family	MDP0000131980	 moderately similar to ( 293) AT1G25440 | Symbols:  | zinc finger (B-box type) family protein | chr1:8933939-8935284 REVERSEvery weakly similar to (55.8) PRR95_ORYSA Two-component response regulator-like PRR95 (Pseudo-response regulator 95) (OsPRR95) - Oryza sativa (Rice)moderately similar to ( 274) loc_os06g15330 12006.m06246 protein zinc finger protein CONSTANS-LIKE 16, putative, expressed  no original description 	T
35.2	not assigned.unknown	MDP0000611897	 highly similar to ( 553) AT5G12900 | Symbols:  | unknown protein | chr5:4072151-4074445 REVERSEhighly similar to ( 564) loc_os08g39740 12008.m07954 protein expressed protein   no original description 	T
35.2	not assigned.unknown	MDP0000230879	 moderately similar to ( 409) AT5G15900 | Symbols:  | unknown protein | chr5:5189524-5192070 REVERSEmoderately similar to ( 375) loc_os01g46400 12001.m10838 protein expressed protein  no original description 	T
28.2	DNA.repair	MDP0000253234	 nearly identical (1159) AT5G64420 | Symbols:  | DNA polymerase V family | chr5:25756416-25761122 FORWARDhighly similar to ( 613) loc_os02g04270 12002.m05776 protein DNA binding protein, putative, expressed   no original description 	T
35.2	not assigned.unknown	MDP0000384344	 moderately similar to ( 349) AT3G62580 | Symbols:  | FUNCTIONS IN: molecular_function unknown; INVOLVED IN: biological_process unknown; LOCATED IN: endomembrane system; EXPRESSED IN: 23 plant structures; EXPRESSED DURING: 13 growth stages; BEST Arabidopsis thaliana protein match is: late embryogenesis abundant domain-containing protein / LEA domain-containing protein (TAIR:AT1G72100.1); Has 237 Blast hits to 236 proteins in 85 species: Archae - 0; Bacteria - 4; Metazoa - 103; Fungi - 69; Plants - 61; Viruses - 0; Other Eukaryotes - 0 (source: NCBI BLink). | chr3:23146931-23147735 FORWARDmoderately similar to ( 308) loc_os12g37060 12012.m07491 protein expressed protein TrbL  no original description 	T
31.1	cell.organisation	MDP0000554878	 moderately similar to ( 409) AT5G23860 | Symbols: TUB8 | TUB8; structural constituent of cytoskeleton | chr5:8042962-8044528 FORWARDmoderately similar to ( 410) TBB9_GOSHI Tubulin beta-9 chain (Beta-9 tubulin) - Gossypium hirsutum (Upland cotton)moderately similar to ( 409) loc_os06g46000 12006.m31916 protein tubulin beta-3 chain, putative, expressed   no original description 	T
27.3.99	RNA.regulation of transcription.unclassified	MDP0000186353	 highly similar to ( 894) AT3G02320 | Symbols:  | RNA binding / tRNA (guanine-N2-)-methyltransferase | chr3:470097-473492 FORWARDhighly similar to ( 781) loc_os03g57280 12003.m10646 protein N-dimethylguanosine tRNA methyltransferase, putative, expressed   no original description 	T
31.1	cell.organisation	MDP0000222713	 moderately similar to ( 216) AT5G62500 | Symbols: ATEB1B, ATEB1, EB1B | ATEB1B (END BINDING PROTEIN 1B); microtubule binding | chr5:25092929-25095006 FORWARDmoderately similar to ( 273) loc_os04g54940 12004.m10386 protein ATEB1A, putative, expressed no original description 	T
31.1	cell.organisation	MDP0000295817	 highly similar to ( 783) AT3G61650 | Symbols: TUBG1 | TUBG1 (GAMMA-TUBULIN); GTP binding / GTPase/ structural molecule | chr3:22812601-22815011 REVERSEhighly similar to ( 746) TBG_PHYPA Tubulin gamma chain (Gamma tubulin) - Physcomitrella patens (Moss)highly similar to ( 739) loc_os05g06450 12005.m27549 protein tubulin gamma-2 chain, putative, expressed  no original description 	T
29.2.1.2.1.10	protein.synthesis.ribosomal protein.eukaryotic.40S subunit.S10	MDP0000552335	 moderately similar to ( 211) AT5G52650 | Symbols:  | 40S ribosomal protein S10 (RPS10C) | chr5:21355781-21357003 REVERSEmoderately similar to ( 207) RS10_ORYSA 40S ribosomal protein S10 - Oryza sativa (Rice)moderately similar to ( 207) loc_os04g35090 12004.m08600 protein 40S ribosomal protein S10, putative, expressed   no original description 	T
20.2.2	stress.abiotic.cold	MDP0000642609	 moderately similar to ( 202) AT3G53990 | Symbols:  | universal stress protein (USP) family protein | chr3:19989658-19991019 REVERSEweakly similar to ( 166) loc_os05g37970 12005.m083738 protein USP family protein, putative, expressed  no original description 	T
29.2.1.1.3.2.18	protein.synthesis.ribosomal protein.prokaryotic.unknown organellar.50S subunit.L18	MDP0000319679	 weakly similar to ( 147) AT5G27820 | Symbols:  | ribosomal protein L18 family protein | chr5:9860584-9860928 FORWARDweakly similar to ( 138) loc_os01g15290 12001.m42950 protein 50S ribosomal protein L18, putative, expressed  no original description 	T
3.6	minor CHO metabolism.callose	MDP0000310065	 highly similar to ( 797) AT1G06490 | Symbols: ATGSL07, gsl07, atgsl7 | ATGSL07 (glucan synthase-like 7); 1,3-beta-glucan synthase/ transferase, transferring glycosyl groups | chr1:1978762-1989046 FORWARDhighly similar to ( 746) loc_os01g34880 12001.m09792 protein ATGSL07, putative, expressed   no original description 	T
35.2	not assigned.unknown	MDP0000206093	 moderately similar to ( 216) AT4G04330 | Symbols:  | unknown protein | chr4:2116700-2118563 REVERSEweakly similar to ( 199) loc_os08g32930 12008.m07285 protein expressed protein  no original description 	T
30.2.11	signalling.receptor kinases.leucine rich repeat XI	MDP0000223968	 nearly identical (1414) AT2G26330 | Symbols: ER, QRP1 | ER (ERECTA); transmembrane receptor protein kinase | chr2:11208367-11213895 REVERSEmoderately similar to ( 444) RPK1_IPONI Receptor-like protein kinase precursor (EC 2.7.11.1) - Ipomoea nil (Japanese morning glory) (Pharbitis nil)nearly identical (1295) loc_os06g10230 12006.m32010 protein receptor-like protein kinase 5 precursor, putative, expressed  no original description 	T
29.2.1.1.1.2.22	protein.synthesis.ribosomal protein.prokaryotic.chloroplast.50S subunit.L22	MDP0000489801	 weakly similar to ( 191) AT4G28360 | Symbols:  | ribosomal protein L22 family  protein | chr4:14029294-14030926 REVERSEweakly similar to ( 161) loc_os01g42980 12001.m150656 protein structural constituent of ribosome, putative, expressed no original description 	T
35.2	not assigned.unknown	MDP0000341454	  no original description 	T
4.2.14	glycolysis.plastid branch.pyruvate kinase (PK)	MDP0000029865	 highly similar to ( 904) AT3G52990 | Symbols:  | pyruvate kinase, putative | chr3:19649336-19652237 FORWARDmoderately similar to ( 376) KPYC_SOYBN Pyruvate kinase, cytosolic isozyme (EC 2.7.1.40) (PK) - Glycine max (Soybean)highly similar to ( 905) loc_os11g05110 12011.m079911 protein pyruvate kinase, cytosolic isozyme, putative, expressed   no original description 	T
10.6.1	cell wall.degradation.cellulases and beta -1,4-glucanases	MDP0000252536	 highly similar to ( 779) AT2G32990 | Symbols: AtGH9B8 | AtGH9B8 (Arabidopsis thaliana glycosyl hydrolase 9B8); catalytic/ hydrolase, hydrolyzing O-glycosyl compounds | chr2:14003361-14005844 FORWARDhighly similar to ( 696) GUN7_ORYSA Endoglucanase 7 precursor (EC 3.2.1.4) (Endo-1,4-beta glucanase 7) (OsGLU10) - Oryza sativa (Rice)highly similar to ( 696) loc_os02g50490 12002.m10083 protein endoglucanase 1 precursor, putative, expressed  no original description 	T
16.1.1.10	secondary metabolism.isoprenoids.non-mevalonate pathway.geranylgeranyl pyrophosphate synthase	MDP0000272025	 highly similar to ( 868) AT1G17210 | Symbols:  | zinc ion binding | chr1:5880502-5884657 REVERSEvery weakly similar to (92.0) GGPPS_HEVBR Geranylgeranyl pyrophosphate synthetase, chloroplast precursor (GGPP synthetase) [Includes: Dimethylallyltranstransferase (EC 2.5.1.1); Geranyltranstransferase (EC 2.5.1.10); Farnesyltranstransferase (EC 2.5.1.29)] - Hevea brasiliensishighly similar to ( 863) loc_os03g57160 12003.m10635 protein expressed protein   no original description 	T
35.2	not assigned.unknown	MDP0000233123	 highly similar to ( 527) AT5G53860 | Symbols: emb2737 | emb2737 (embryo defective 2737) | chr5:21865353-21868576 FORWARD   no original description 	T
34.13	transport.peptides and oligopeptides	MDP0000301119	 highly similar to ( 968) AT4G24120 | Symbols: YSL1, ATYSL1 | YSL1 (YELLOW STRIPE LIKE 1); oligopeptide transporter | chr4:12524581-12527023 FORWARDhighly similar to ( 871) loc_os04g45860 12004.m101436 protein transposon protein, putative, unclassified, expressed    no original description 	T
29.4	protein.postranslational modification	MDP0000119707	 nearly identical (1316) AT2G27210 | Symbols: BSL3 | kelch repeat-containing serine/threonine phosphoesterase family protein | chr2:11630188-11636182 FORWARDnearly identical (1264) BSL2_ORYSA Serine/threonine-protein phosphatase BSL2 homolog (EC 3.1.3.16) (BSU1-like protein 2 homolog) - Oryza sativa (Rice)nearly identical (1267) loc_os03g44500 12003.m09488 protein serine/threonine-protein phosphatase BSL2, putative, expressed   no original description 	T
11.1.7	lipid metabolism.FA synthesis and FA elongation.ACP thioesterase	MDP0000294705	 highly similar to ( 573) AT1G08510 | Symbols: FATB | FATB (fatty acyl-ACP thioesterases B); acyl carrier/ acyl-[acyl-carrier-protein] hydrolase | chr1:2691546-2693409 REVERSEhighly similar to ( 570) FATB_GOSHI Myristoyl-acyl carrier protein thioesterase, chloroplast precursor (EC 3.1.2.-) (16:0-acyl-carrier protein thioesterase) (16:0-ACP thioesterase) (Acyl-[acyl-carrier-protein] hydrolase) (PATE) - Gossypium hirsutum (Upland cotton)moderately similar to ( 489) loc_os06g05130 12006.m05242 protein myristoyl-acyl carrier protein thioesterase, chloroplast precursor, putative, expressed   no original description 	T
31.1	cell.organisation	MDP0000203289	 moderately similar to ( 374) AT2G45740 | Symbols: PEX11D | PEX11D | chr2:18839865-18841102 FORWARDmoderately similar to ( 349) loc_os03g02590 12003.m101159 protein peroxisomal membrane protein PEX11-1, putative, expressed no original description 	T
29.3.5	protein.targeting.peroxisomes	MDP0000203289	 moderately similar to ( 374) AT2G45740 | Symbols: PEX11D | PEX11D | chr2:18839865-18841102 FORWARDmoderately similar to ( 349) loc_os03g02590 12003.m101159 protein peroxisomal membrane protein PEX11-1, putative, expressed no original description 	T
10.5.1.1	cell wall.cell wall proteins.AGPs.AGP	MDP0000428284	 very weakly similar to (74.7) AT3G61640 | Symbols: AGP20, AtAGP20 | AGP20 | chr3:22810283-22810629 REVERSEvery weakly similar to (64.3) loc_os05g12580 12005.m05721 protein AGP20, putative no original description 	T
29.2.1.2.2.3	protein.synthesis.ribosomal protein.eukaryotic.60S subunit.L3	MDP0000303386	 highly similar to ( 656) AT1G43170 | Symbols: ARP1, RPL3A | ARP1 (ARABIDOPSIS RIBOSOMAL PROTEIN 1); structural constituent of ribosome | chr1:16267519-16268631 FORWARDhighly similar to ( 659) RL3_ORYSJ 60S ribosomal protein L3 OS=Oryza sativa subsp. japonica GN=RPL3 PE=2 SV=2highly similar to ( 659) loc_os12g07010 12012.m04691 protein 60S ribosomal protein L3, putative, expressed   no original description 	T
33.99	development.unspecified	MDP0000852271	 moderately similar to ( 423) AT1G34190 | Symbols: anac017 | anac017 (Arabidopsis NAC domain containing protein 17); transcription factor | chr1:12451729-12453914 FORWARDweakly similar to ( 144) NAC74_ORYSA NAC domain-containing protein 74 (ONAC074) - Oryza sativa (Rice)weakly similar to ( 192) loc_os09g32040 12009.m06294 protein no apical meristem protein, expressed no original description 	T
29.2.1.2.1.29	protein.synthesis.ribosomal protein.eukaryotic.40S subunit.S29	MDP0000797824	 weakly similar to ( 120) AT4G33865 | Symbols:  | 40S ribosomal protein S29 (RPS29C) | chr4:16233395-16234114 REVERSEweakly similar to ( 128) loc_os11g41610 12011.m08002 protein 40S ribosomal protein S29, putative, expressed no original description 	T
20.1.7	stress.biotic.PR-proteins	MDP0000310092	 highly similar to ( 537) AT5G63020 | Symbols:  | disease resistance protein (CC-NBS-LRR class), putative | chr5:25283252-25286002 REVERSEmoderately similar to ( 401) loc_os01g57870 12001.m150747 protein disease resistance protein RPS2, putative, expressed  no original description 	T
30.2.17	signalling.receptor kinases.DUF 26	MDP0000680604	 weakly similar to ( 161) AT4G27300 | Symbols:  | S-locus protein kinase, putative | chr4:13669308-13672348 REVERSEvery weakly similar to (58.5) PSKR_DAUCA Phytosulfokine receptor precursor (EC 2.7.11.1) (Phytosulfokine LRR receptor kinase) - Daucus carota (Carrot)weakly similar to ( 149) loc_os01g57560 12001.m11907 protein serine/threonine-protein kinase receptor precursor, putative, expressed no original description 	T
26.2	misc.UDP glucosyl and glucoronyl transferases	MDP0000478252	 moderately similar to ( 436) AT5G17050 | Symbols: UGT78D2 | UGT78D2 (UDP-GLUCOSYL TRANSFERASE 78D2); UDP-glycosyltransferase/ anthocyanidin 3-O-glucosyltransferase/ quercetin 3-O-glucosyltransferase/ transferase, transferring glycosyl groups | chr5:5607828-5609392 REVERSEmoderately similar to ( 273) UFOG_SOLME Anthocyanidin 3-O-glucosyltransferase (EC 2.4.1.115) (Flavonol 3-O-glucosyltransferase) (UDP-glucose flavonoid 3-O-glucosyltransferase) - Solanum melongena (Eggplant) (Aubergine)moderately similar to ( 276) loc_os06g09240 12006.m05643 protein anthocyanidin 3-O-glucosyltransferase, putative, expressed   no original description 	T
27.3.99	RNA.regulation of transcription.unclassified	MDP0000201190	 moderately similar to ( 315) AT1G08680 | Symbols: ZIGA4 | ZIGA4 (ARF GAP-like zinc finger-containing protein ZiGA4); ARF GTPase activator/ DNA binding / zinc ion binding | chr1:2762820-2768387 FORWARDweakly similar to ( 194) loc_os01g59850 12001.m12119 protein ZIGA4, putative, expressed   no original description 	T
17.5.1	hormone metabolism.ethylene.synthesis-degradation	MDP0000240641	 highly similar to ( 501) AT4G22880 | Symbols: LDOX, TDS4, TT18, ANS | LDOX (LEUCOANTHOCYANIDIN DIOXYGENASE); leucocyanidin oxygenase | chr4:12004905-12006059 REVERSEhighly similar to ( 628) LDOX_MALDO Leucoanthocyanidin dioxygenase (EC 1.14.11.19) (LDOX) (Leucocyanidin oxygenase) (Leucoanthocyanidin hydroxylase) (Anthocyanidin synthase) - Malus domestica (Apple) (Malus sylvestris)moderately similar to ( 318) loc_os01g27490 12001.m09199 protein leucoanthocyanidin dioxygenase, putative, expressed no original description 	T
29.5.11.20	protein.degradation.ubiquitin.proteasom	MDP0000185254	 moderately similar to ( 258) AT4G31300 | Symbols: PBA1 | PBA1; endopeptidase/ peptidase/ threonine-type endopeptidase | chr4:15188927-15190935 FORWARDmoderately similar to ( 281) PSB6_TOBAC Proteasome subunit beta type 6 precursor (EC 3.4.25.1) (Proteasome delta chain) (Tobacco cryptogein-induced protein 7) (tcI 7) - Nicotiana tabacum (Common tobacco)moderately similar to ( 239) loc_os02g53060 12002.m10336 protein proteasome subunit beta type 6 precursor, putative, expressed   no original description 	T
35.2	not assigned.unknown	MDP0000514153	 very weakly similar to (72.8) AT5G65400 | Symbols:  | unknown protein | chr5:26131448-26133062 FORWARDweakly similar to ( 148) loc_os07g48770 12007.m09087 protein expressed protein no original description 	T
29.3.4.3	protein.targeting.secretory pathway.vacuole	MDP0000122571	 highly similar to ( 632) AT1G62710 | Symbols: BETA-VPE, BETAVPE | BETA-VPE (BETA VACUOLAR PROCESSING ENZYME); cysteine-type endopeptidase | chr1:23224070-23226857 REVERSEhighly similar to ( 706) VPE_SOYBN Vacuolar-processing enzyme precursor (EC 3.4.22.-) (VPE) - Glycine max (Soybean)highly similar to ( 590) loc_os02g43010 12002.m33425 protein vacuolar processing enzyme, beta-isozyme precursor, putative, expressed  no original description 	T
27.3.99	RNA.regulation of transcription.unclassified	MDP0000154584	 highly similar to ( 680) AT4G36860 | Symbols:  | zinc ion binding | chr4:17358580-17361189 REVERSEhighly similar to ( 620) loc_os06g08400 12006.m32223 protein zinc ion binding protein, putative, expressed   no original description 	T
20.2.99	stress.abiotic.unspecified	MDP0000688187	 weakly similar to ( 147) AT3G62550 | Symbols:  | universal stress protein (USP) family protein | chr3:23136194-23137074 FORWARDweakly similar to ( 185) loc_os01g19820 12001.m150564 protein universal stress protein, putative, expressed  no original description 	T
27.3.67	RNA.regulation of transcription.putative transcription regulator	MDP0000207889	 highly similar to ( 586) AT1G51540 | Symbols:  | INVOLVED IN: biological_process unknown; LOCATED IN: Golgi apparatus; EXPRESSED IN: 22 plant structures; EXPRESSED DURING: 13 growth stages; CONTAINS InterPro DOMAIN/s: Galactose oxidase/kelch, beta-propeller (InterPro:IPR011043), Kelch repeat type 1 (InterPro:IPR006652), Kelch repeat type 2 (InterPro:IPR011498), Kelch related (InterPro:IPR013089), Kelch-type beta propeller (InterPro:IPR015915); BEST Arabidopsis thaliana protein match is: kelch repeat-containing protein (TAIR:AT3G27220.1); Has 1571 Blast hits to 1365 proteins in 151 species: Archae - 11; Bacteria - 200; Metazoa - 1128; Fungi - 24; Plants - 53; Viruses - 41; Other Eukaryotes - 114 (source: NCBI BLink). | chr1:19115702-19117381 FORWARDhighly similar to ( 589) loc_os04g41020 12004.m09082 protein kelch motif family protein, expressed  no original description 	T
35.2	not assigned.unknown	MDP0000052687	 moderately similar to ( 445) AT4G09580 | Symbols:  | INVOLVED IN: biological_process unknown; LOCATED IN: endoplasmic reticulum; EXPRESSED IN: 24 plant structures; EXPRESSED DURING: 13 growth stages; CONTAINS InterPro DOMAIN/s: SNARE associated Golgi protein (InterPro:IPR015414); BEST Arabidopsis thaliana protein match is: unknown protein (TAIR:AT1G71940.1); Has 2023 Blast hits to 2023 proteins in 496 species: Archae - 2; Bacteria - 1014; Metazoa - 198; Fungi - 27; Plants - 131; Viruses - 0; Other Eukaryotes - 651 (source: NCBI BLink). | chr4:6052721-6054313 REVERSEmoderately similar to ( 360) loc_os03g49700 12003.m09969 protein gtk16 protein, putative, expressed no original description 	T
27.1.19	RNA.processing.ribonucleases	MDP0000826052	 moderately similar to ( 335) AT1G14220 | Symbols:  | ribonuclease T2 family protein | chr1:4858642-4859601 REVERSEweakly similar to ( 115) RN28_PANGI Ribonuclease-like storage protein precursor (Root 28 kDa major protein) - Panax ginseng (Korean ginseng)moderately similar to ( 277) loc_os08g33710 12008.m07361 protein ribonuclease 3 precursor, putative, expressed no original description 	T
34.10	transport.nucleotides	MDP0000546892	 moderately similar to ( 312) AT4G18210 | Symbols: ATPUP10 | ATPUP10; purine transmembrane transporter | chr4:10076175-10077495 FORWARDmoderately similar to ( 264) loc_os02g46380 12002.m33436 protein ATPUP11, putative, expressed   no original description 	T
20.2.1	stress.abiotic.heat	MDP0000230007	 highly similar to ( 905) AT1G79920 | Symbols:  | ATP binding | chr1:30058935-30062224 REVERSEmoderately similar to ( 277) HSP7C_PETHY Heat shock cognate 70 kDa protein - Petunia hybrida (Petunia)highly similar to ( 839) loc_os01g08560 12001.m150497 protein heat shock 70 kDa protein 4, putative, expressed   no original description 	T
35.2	not assigned.unknown	MDP0000195431	 moderately similar to ( 265) AT1G28520 | Symbols: VOZ1 | VOZ1 (VASCULAR PLANT ONE ZINC FINGER PROTEIN); transcription activator | chr1:10029713-10031479 FORWARDweakly similar to ( 168) loc_os01g54930 12001.m11654 protein vascular plant one zinc finger protein, putative, expressed no original description 	T
35.1	not assigned.no ontology	MDP0000199066	 highly similar to ( 573) AT5G34940 | Symbols: AtGUS3 | AtGUS3 (Arabidopsis thaliana glucuronidase 3); beta-glucuronidase | chr5:13236566-13238718 REVERSEhighly similar to ( 584) loc_os02g55870 12002.m10611 protein heparanase-like protein 3 precursor, putative, expressed   no original description 	T
10.8.1	cell wall.pectin*esterases.PME	MDP0000420628	 weakly similar to ( 151) AT4G33220 | Symbols: PME44, ATPME44 | enzyme inhibitor/ pectinesterase | chr4:16022506-16026130 FORWARDweakly similar to ( 161) PME_PRUPE Pectinesterase PPE8B precursor (EC 3.1.1.11) (Pectin methylesterase) (PE) - Prunus persica (Peach)weakly similar to ( 135) loc_os08g34900 12008.m07479 protein pectinesterase PPE8B precursor, putative, expressed  no original description 	T
31.1	cell.organisation	MDP0000170091	 moderately similar to ( 324) AT5G56180 | Symbols: ATARP8, ARP8 | ATARP8 (ACTIN-RELATED PROTEIN 8); structural constituent of cytoskeleton | chr5:22738330-22740986 REVERSEvery weakly similar to ( 100) ACT_MESVI Actin - Mesostigma viridemoderately similar to ( 293) loc_os04g57210 12004.m10606 protein actin-6, putative, expressed no original description 	T
29.4.1.57	protein.postranslational modification.kinase.receptor like cytoplasmatic kinase VII	MDP0000316714	 highly similar to ( 900) AT5G43745 | Symbols:  | phosphotransferase-related | chr5:17569435-17574954 REVERSEweakly similar to ( 164) DMI1L_ORYSA Probable ion channel DMI1-like, chloroplast precursor - Oryza sativa (Rice)highly similar to ( 817) loc_os03g06720 12003.m06190 protein expressed protein   no original description 	T
29.5.11	protein.degradation.ubiquitin	MDP0000232968	 moderately similar to ( 434) AT4G22290 | Symbols:  | ubiquitin thiolesterase | chr4:11783199-11785730 REVERSEmoderately similar to ( 283) loc_os08g13350 12008.m26625 protein cysteine-type endopeptidase/ ubiquitin thiolesterase, putative, expressed     no original description 	T
34.13	transport.peptides and oligopeptides	MDP0000173611	 highly similar to ( 872) AT1G48370 | Symbols: YSL8 | YSL8 (YELLOW STRIPE LIKE 8); oligopeptide transporter | chr1:17874560-17877256 FORWARDhighly similar to ( 848) loc_os02g42220 12002.m09259 protein transposon protein, putative, unclassified, expressed   no original description 	T
30.2.11	signalling.receptor kinases.leucine rich repeat XI	MDP0000839235	 highly similar to ( 555) AT1G03440 | Symbols:  | leucine-rich repeat family protein | chr1:852681-853874 FORWARDweakly similar to ( 117) RPK1_IPONI Receptor-like protein kinase precursor (EC 2.7.11.1) - Ipomoea nil (Japanese morning glory) (Pharbitis nil)moderately similar to ( 457) loc_os05g33690 12005.m27820 protein receptor-like protein kinase precursor, putative, expressed   no original description 	T
30.5	signalling.G-proteins	MDP0000143585	 highly similar to ( 619) AT3G07050 | Symbols:  | GTP-binding family protein | chr3:2229602-2232279 REVERSEhighly similar to ( 559) loc_os01g27730 12001.m09221 protein guanine nucleotide-binding protein-like 3, putative, expressed  no original description 	T
8.1.2	TCA / org transformation.TCA.CS	MDP0000321473	 highly similar to ( 766) AT2G44350 | Symbols: ATCS, CSY4 | ATCS; ATP binding / ATP citrate synthase/ citrate (SI)-synthase | chr2:18316673-18320524 FORWARDhighly similar to ( 762) CISY_DAUCA Citrate synthase, mitochondrial precursor (EC 2.3.3.1) - Daucus carota (Carrot)highly similar to ( 764) loc_os11g33240 12011.m080018 protein citrate synthase 4, mitochondrial precursor, putative, expressed  no original description 	T
30.11	signalling.light	MDP0000617714	 highly similar to ( 923) AT1G30440 | Symbols:  | phototropic-responsive NPH3 family protein | chr1:10759475-10762199 FORWARDmoderately similar to ( 429) NPH3_ORYSA Coleoptile phototropism protein 1 (Non-phototropic hypocotyl 3-like protein) (NPH3-like protein) - Oryza sativa (Rice)highly similar to ( 906) loc_os03g43990 12003.m35330 protein transposon protein, putative, Mutator sub-class, expressed   no original description 	T
27.3.26	RNA.regulation of transcription.MYB-related transcription factor family	MDP0000142580	 moderately similar to ( 327) AT2G38090 | Symbols:  | myb family transcription factor | chr2:15945278-15946775 FORWARDmoderately similar to ( 234) loc_os05g37730 12005.m07952 protein MYB-like transcription factor DIVARICATA, putative, expressed  no original description 	T
35.2	not assigned.unknown	MDP0000227882	 moderately similar to ( 338) AT5G11680 | Symbols:  | unknown protein | chr5:3759463-3760634 FORWARDmoderately similar to ( 321) loc_os09g33800 12009.m22015 protein arabinogalactan protein, putative, expressed no original description 	T
29.5.11.20	protein.degradation.ubiquitin.proteasom	MDP0000122708	 highly similar to ( 631) AT4G24820 | Symbols:  | 26S proteasome regulatory subunit, putative (RPN7) | chr4:12790471-12792599 REVERSEhighly similar to ( 619) PSMD6_ORYSA 26S proteasome non-ATPase regulatory subunit 6 (26S proteasome regulatory particle non-ATPase subunit 7) (OsRPN7) - Oryza sativa (Rice)highly similar to ( 619) loc_os04g40850 12004.m35346 protein 26S proteasome non-ATPase regulatory subunit 6, putative, expressed  MSP1_C  no original description 	T
35.2	not assigned.unknown	MDP0000138428	 moderately similar to ( 466) AT4G21150 | Symbols: HAP6 | HAP6 (HAPLESS 6); dolichyl-diphosphooligosaccharide-protein glycotransferase | chr4:11278646-11283599 FORWARDmoderately similar to ( 452) loc_os01g68324 12001.m12935 protein dolichyl-diphosphooligosaccharide--protein glycosyltransferase 63 kDasubunit precursor, putative, expressed  MSP1_C  no original description 	T
35.2	not assigned.unknown	MDP0000161217	 weakly similar to ( 119) AT4G25670 | Symbols:  | unknown protein | chr4:13085431-13085997 REVERSEweakly similar to ( 108) loc_os02g01360 12002.m05486 protein expressed protein no original description 	T
31.1	cell.organisation	MDP0000308456	 moderately similar to ( 268) AT3G18670 | Symbols:  | ankyrin repeat family protein | chr3:6424135-6426471 REVERSEvery weakly similar to (64.3) loc_os11g16910 12011.m05774 protein protein binding protein, putative no original description 	T
35.2	not assigned.unknown	MDP0000226838	 nearly identical (1043) AT1G64960 | Symbols:  | binding | chr1:24129918-24134049 FORWARDhighly similar to ( 714) loc_os03g42040 12003.m09262 protein HEAT repeat family protein, expressed   no original description 	T
30.3	signalling.calcium	MDP0000240032	 very weakly similar to (95.5) AT1G24620 | Symbols:  | polcalcin, putative / calcium-binding pollen allergen, putative | chr1:8723893-8724453 REVERSEvery weakly similar to (80.5) CALM_CHLRE Calmodulin (CaM) - Chlamydomonas reinhardtiivery weakly similar to (94.4) loc_os06g47640 12006.m09290 protein polcalcin Jun o 2, putative, expressed  no original description 	T
35.2	not assigned.unknown	MDP0000156049	 moderately similar to ( 464) AT3G17800 | Symbols:  | mRNA level of the MEB5.2 gene (At3g17800) remains unchanged after cutting the inflorescence stem | chr3:6091248-6092873 REVERSEmoderately similar to ( 386) loc_os03g50870 12003.m34915 protein expressed protein  no original description 	T
30.5	signalling.G-proteins	MDP0000167843	 nearly identical (1836) AT5G27970 | Symbols:  | binding | chr5:10004720-10015429 FORWARDhighly similar to ( 895) loc_os01g56590 12001.m11816 protein HEAT repeat family protein, expressed  no original description 	T
29.6	protein.folding	MDP0000299701	 highly similar to ( 934) AT5G56500 | Symbols:  | ATP binding / protein binding | chr5:22874058-22876966 FORWARDhighly similar to ( 946) RUBB_PEA RuBisCO large subunit-binding protein subunit beta, chloroplast precursor (60 kDa chaperonin subunit beta) (CPN-60 beta) - Pisum sativum (Garden pea)highly similar to ( 894) loc_os06g02380 12006.m31823 protein ruBisCO large subunit-binding protein subunit beta, chloroplast, putative, expressed  no original description 	T
13.1.2.2	amino acid metabolism.synthesis.glutamate family.proline	MDP0000845591	 nearly identical (1048) AT3G55610 | Symbols: P5CS2 | P5CS2 (DELTA 1-PYRROLINE-5-CARBOXYLATE SYNTHASE 2); catalytic/ glutamate 5-kinase/ oxidoreductase | chr3:20625005-20628989 REVERSEnearly identical (1069) P5CS_ACTCH Delta 1-pyrroline-5-carboxylate synthetase (P5CS) [Includes: Glutamate 5-kinase (EC 2.7.2.11) (Gamma-glutamyl kinase) (GK); Gamma-glutamyl phosphate reductase (GPR) (EC 1.2.1.41) (Glutamate-5-semialdehyde dehydrogenase) (Glutamyl-gamma-nearly identical (1032) loc_os05g38150 12005.m27606 protein delta 1-pyrroline-5-carboxylate synthetase, putative, expressed  no original description 	T
17.7.1.2	hormone metabolism.jasmonate.synthesis-degradation.lipoxygenase	MDP0000169311	 highly similar to ( 520) AT3G45140 | Symbols: LOX2, ATLOX2 | LOX2 (LIPOXYGENASE 2); lipoxygenase | chr3:16525437-16529233 FORWARDhighly similar to ( 538) LOX23_HORVU Lipoxygenase 2.3, chloroplast precursor (EC 1.13.11.12) (LOX2:Hv:3) - Hordeum vulgare (Barley)highly similar to ( 540) loc_os02g10120 12002.m06309 protein lipoxygenase 2.3, chloroplast precursor, putative, expressed  no original description 	T
26.1	misc.misc2	MDP0000543088	 moderately similar to ( 414) AT4G02340 | Symbols:  | epoxide hydrolase, putative | chr4:1035722-1037403 FORWARDmoderately similar to ( 429) loc_os05g19150 12005.m06318 protein epoxide hydrolase 2, putative, expressed   no original description 	T
11.8.1	lipid metabolism.'exotics' (steroids, squalene etc).sphingolipids	MDP0000286450	 moderately similar to ( 411) AT1G69640 | Symbols: SBH1 | SBH1 (SPHINGOID BASE HYDROXYLASE 1); catalytic/ sphingosine hydroxylase | chr1:26193933-26195466 REVERSEmoderately similar to ( 376) loc_os06g12250 12006.m05942 protein protein SUR2, putative, expressed no original description 	T
11.1.10	lipid metabolism.FA synthesis and FA elongation.beta ketoacyl CoA synthase	MDP0000392495	 highly similar to ( 864) AT1G68530 | Symbols: CUT1, POP1, CER6, G2, KCS6 | KCS6 (3-KETOACYL-COA SYNTHASE 6); catalytic/ transferase, transferring acyl groups other than amino-acyl groups | chr1:25713600-25714733 REVERSEvery weakly similar to (62.0) CHSE_IPONI Chalcone synthase E (EC 2.3.1.74) (Naringenin-chalcone synthase E) (CHS-E) - Ipomoea nil (Japanese morning glory) (Pharbitis nil)highly similar to ( 811) loc_os03g12030 12003.m06655 protein 3-ketoacyl-CoA synthase, putative, expressed   no original description 	T
34.22	transport.cyclic nucleotide or calcium regulated channels	MDP0000124820	 moderately similar to ( 284) AT5G53130 | Symbols: CNGC1, ATCNGC1 | CNGC1 (CYCLIC NUCLEOTIDE GATED CHANNEL 1); calmodulin binding / cation channel/ cyclic nucleotide binding / intracellular cyclic nucleotide activated cation channel/ inward rectifier potassium channel | chr5:21537830-21540490 REVERSEmoderately similar to ( 236) loc_os06g33570 12006.m07894 protein cyclic nucleotide-gated ion channel 1, putative, expressed  no original description 	T
35.2	not assigned.unknown	MDP0000370213	 moderately similar to ( 297) AT5G26910 | Symbols:  | unknown protein | chr5:9466804-9469523 REVERSEmoderately similar to ( 436) loc_os03g19080 12003.m101318 protein expressed protein  no original description 	T
34.21	transport.calcium	MDP0000193502	 moderately similar to ( 490) AT3G51860 | Symbols: CAX3, ATHCX1, CAX1-LIKE, ATCAX3 | CAX3 (CATION EXCHANGER 3); calcium:cation antiporter/ calcium:hydrogen antiporter/ cation:cation antiporter | chr3:19239458-19242519 FORWARDmoderately similar to ( 441) CAX1A_ORYSA Vacuolar cation/proton exchanger 1a (Ca(2+)/H(+) exchanger 1a) (OsCAX1a) - Oryza sativa (Rice)moderately similar to ( 441) loc_os01g37690 12001.m42663 protein vacuolar cation/proton exchanger 1a, putative, expressed no original description 	T
26.10	misc.cytochrome P450	MDP0000449345	 moderately similar to ( 483) AT1G74110 | Symbols: CYP78A10 | CYP78A10; electron carrier/ heme binding / iron ion binding / monooxygenase/ oxygen binding | chr1:27866667-27868368 REVERSEmoderately similar to ( 459) C78A3_SOYBN Cytochrome P450 78A3 (EC 1.14.-.-) - Glycine max (Soybean)moderately similar to ( 498) loc_os11g29720 12011.m06881 protein cytochrome P450 78A4, putative, expressed    PRK08026 MSP1_C  no original description 	T
27.1	RNA.processing	MDP0000152771	 highly similar to ( 735) AT1G49760 | Symbols: PAB8, PABP8 | PAB8 (POLY(A) BINDING PROTEIN 8); RNA binding / translation initiation factor | chr1:18416740-18419753 FORWARDvery weakly similar to (85.1) ROC1_NICSY 29 kDa ribonucleoprotein A, chloroplast precursor (CP29A) - Nicotiana sylvestris (Wood tobacco)highly similar to ( 769) loc_os09g02700 12009.m03642 protein polyadenylate-binding protein 2, putative, expressed TrbL  no original description 	T
27.4	RNA.RNA binding	MDP0000152771	 highly similar to ( 735) AT1G49760 | Symbols: PAB8, PABP8 | PAB8 (POLY(A) BINDING PROTEIN 8); RNA binding / translation initiation factor | chr1:18416740-18419753 FORWARDvery weakly similar to (85.1) ROC1_NICSY 29 kDa ribonucleoprotein A, chloroplast precursor (CP29A) - Nicotiana sylvestris (Wood tobacco)highly similar to ( 769) loc_os09g02700 12009.m03642 protein polyadenylate-binding protein 2, putative, expressed TrbL  no original description 	T
29.4	protein.postranslational modification	MDP0000589319	 highly similar to ( 662) AT3G04910 | Symbols: WNK1, ZIK4 | WNK1 (WITH NO LYSINE (K) 1); kinase/ protein kinase/ protein serine/threonine kinase | chr3:1356061-1358057 FORWARDweakly similar to ( 102) M2K1_ORYSA Mitogen-activated protein kinase kinase 1 (EC 2.7.12.2) (MAP kinase kinase 1) (MAPKK1) (OsMEK1) - Oryza sativa (Rice)highly similar to ( 626) loc_os07g38530 12007.m29232 protein protein kinase domain containing protein, expressed   no original description 	T
29.5.11.4.2	protein.degradation.ubiquitin.E3.RING	MDP0000201988	 weakly similar to ( 141) AT5G20885 | Symbols:  | zinc finger (C3HC4-type RING finger) family protein | chr5:7084133-7084663 REVERSEvery weakly similar to (55.8) EL5_ORYSA E3 ubiquitin-protein ligase EL5 (EC 6.3.2.-) - Oryza sativa (Rice)very weakly similar to (73.2) loc_os04g49000 12004.m09835 protein RING zinc finger protein-like, putative, expressed no original description 	T
20.2.99	stress.abiotic.unspecified	MDP0000452572	 weakly similar to ( 196) AT3G53990 | Symbols:  | universal stress protein (USP) family protein | chr3:19989658-19991019 REVERSEweakly similar to ( 179) loc_os05g37970 12005.m083738 protein USP family protein, putative, expressed   no original description 	T
34.3	transport.amino acids	MDP0000778359	 moderately similar to ( 324) AT2G39130 | Symbols:  | amino acid transporter family protein | chr2:16323171-16326744 REVERSEmoderately similar to ( 286) loc_os02g01100 12002.m100064 protein amino acid permease, putative, expressed no original description 	T
26.10	misc.cytochrome P450	MDP0000163110	 moderately similar to ( 368) AT3G48310 | Symbols: CYP71A22 | CYP71A22; electron carrier/ heme binding / iron ion binding / monooxygenase/ oxygen binding | chr3:17888192-17889749 FORWARDmoderately similar to ( 382) C71A4_SOLME Cytochrome P450 71A4 (EC 1.14.-.-) (CYPLXXIA4) (P-450EG2) - Solanum melongena (Eggplant) (Aubergine)moderately similar to ( 376) loc_os09g26960 12009.m05848 protein flavonoid 3-monooxygenase, putative, expressed   no original description 	T
28.1.3	DNA.synthesis/chromatin structure.histone	MDP0000446777	 moderately similar to ( 228) AT5G65360 | Symbols:  | histone H3 | chr5:26120099-26120509 REVERSEmoderately similar to ( 226) H32_WHEAT Histone H3.2 - Triticum aestivum (Wheat)moderately similar to ( 228) loc_os11g05730 12011.m04769 protein histone H3, putative, expressed no original description 	T
35.2	not assigned.unknown	MDP0000158114	 moderately similar to ( 449) AT1G27210 | Symbols:  | binding | chr1:9455669-9457917 REVERSEmoderately similar to ( 291) loc_os02g50640 12002.m10098 protein microtubule-associated protein TORTIFOLIA1, putative, expressed   no original description 	T
26.22	misc.short chain dehydrogenase/reductase (SDR)	MDP0000128721	 moderately similar to ( 360) AT3G61220 | Symbols:  | short-chain dehydrogenase/reductase (SDR) family protein | chr3:22663025-22664316 FORWARDvery weakly similar to (62.8) SDR1_PICAB Short-chain type dehydrogenase/reductase (EC 1.-.-.-) - Picea abies (Norway spruce) (Picea excelsa)moderately similar to ( 291) loc_os04g44950 12004.m09449 protein carbonyl reductase 1, putative, expressed no original description 	T
34.9	transport.metabolite transporters at the mitochondrial membrane	MDP0000214285	 highly similar to ( 556) AT5G14040 | Symbols:  | mitochondrial phosphate transporter | chr5:4531059-4532965 REVERSEvery weakly similar to (55.1) BT1_MAIZE Protein brittle-1, chloroplast precursor - Zea mays (Maize)highly similar to ( 541) loc_os02g52860 12002.m10316 protein phosphate carrier protein, mitochondrial precursor, putative, expressed  no original description 	T
29.5.11.4.3.2	protein.degradation.ubiquitin.E3.SCF.FBOX	MDP0000190407	 weakly similar to ( 150) AT4G14096 | Symbols:  | FUNCTIONS IN: molecular_function unknown; INVOLVED IN: biological_process unknown; LOCATED IN: cellular_component unknown; CONTAINS InterPro DOMAIN/s: Cyclin-like F-box (InterPro:IPR001810), FBD-like (InterPro:IPR006566), Leucine-rich repeat 2 (InterPro:IPR013101); BEST Arabidopsis thaliana protein match is: F-box family protein (TAIR:AT4G14103.1); Has 1300 Blast hits to 1271 proteins in 9 species: Archae - 0; Bacteria - 0; Metazoa - 0; Fungi - 0; Plants - 1300; Viruses - 0; Other Eukaryotes - 0 (source: NCBI BLink). | chr4:8124606-8126202 FORWARDvery weakly similar to (86.7) loc_os11g09970 12011.m05194 protein F-box domain containing protein, expressed  no original description 	T
35.2	not assigned.unknown	MDP0000291489	 moderately similar to ( 265) AT5G27390 | Symbols:  | LOCATED IN: chloroplast; CONTAINS InterPro DOMAIN/s: Mog1/PsbP/DUF1795, alpha/beta/alpha sandwich (InterPro:IPR016124); Has 11 Blast hits to 11 proteins in 5 species: Archae - 0; Bacteria - 0; Metazoa - 0; Fungi - 0; Plants - 11; Viruses - 0; Other Eukaryotes - 0 (source: NCBI BLink). | chr5:9674634-9676118 REVERSEmoderately similar to ( 259) loc_os05g33300 12005.m07562 protein tat pathway signal sequence family protein, expressed  no original description 	T
27.3.40	RNA.regulation of transcription.Aux/IAA family	MDP0000262602	 moderately similar to ( 276) AT4G29080 | Symbols: PAP2, IAA27 | PAP2 (PHYTOCHROME-ASSOCIATED PROTEIN 2); transcription factor | chr4:14323665-14325213 REVERSEmoderately similar to ( 262) IAA21_ORYSA Auxin-responsive protein IAA21 (Indoleacetic acid-induced protein 21) - Oryza sativa (Rice)moderately similar to ( 262) loc_os06g22870 12006.m091686 protein OsIAA21 - Auxin-responsive Aux/IAA gene family member, expressed   no original description 	T
34.9	transport.metabolite transporters at the mitochondrial membrane	MDP0000491142	 moderately similar to ( 432) AT4G32400 | Symbols: SHS1, ATBT1 | SHS1 (SODIUM HYPERSENSITIVE 1); binding / nucleotide transmembrane transporter/ transporter | chr4:15638686-15640238 FORWARDmoderately similar to ( 353) BT1_MAIZE Protein brittle-1, chloroplast precursor - Zea mays (Maize)moderately similar to ( 417) loc_os05g07900 12005.m083634 protein protein brittle-1, chloroplast precursor, putative, expressed   no original description 	T
1.3.13	PS.calvin cycle.rubisco interacting	MDP0000207359	 highly similar to ( 513) AT1G73110 | Symbols:  | ribulose bisphosphate carboxylase/oxygenase activase, putative / RuBisCO activase, putative | chr1:27494344-27496844 REVERSEmoderately similar to ( 262) RCA_MAIZE Ribulose bisphosphate carboxylase/oxygenase activase, chloroplast precursor (RuBisCO activase) (RA) - Zea mays (Maize)highly similar to ( 518) loc_os04g56320 12004.m10522 protein ribulose bisphosphate carboxylase/oxygenase activase, chloroplast precursor, putative, expressed  no original description 	T
30.2.11	signalling.receptor kinases.leucine rich repeat XI	MDP0000674377	 nearly identical (1247) AT2G41820 | Symbols:  | leucine-rich repeat transmembrane protein kinase, putative | chr2:17447170-17449914 FORWARDmoderately similar to ( 405) RPK1_IPONI Receptor-like protein kinase precursor (EC 2.7.11.1) - Ipomoea nil (Japanese morning glory) (Pharbitis nil)highly similar to ( 953) loc_os07g10630 12007.m05518 protein ATP binding protein, putative, expressed   no original description 	T
9.6	mitochondrial electron transport / ATP synthesis.cytochrome c	MDP0000504439	 moderately similar to ( 214) AT1G22840 | Symbols: CYTC-1, ATCYTC-A | CYTC-1 (CYTOCHROME C-1); electron carrier/ heme binding / iron ion binding | chr1:8079384-8080246 FORWARDmoderately similar to ( 219) CYC_PHAAU Cytochrome c - Phaseolus aureus (Mung bean) (Vigna radiata)moderately similar to ( 212) loc_os05g34770 12005.m27830 protein cytochrome c, putative, expressed no original description 	T
29.5.3	protein.degradation.cysteine protease	MDP0000279713	 weakly similar to ( 154) AT3G45310 | Symbols:  | cysteine proteinase, putative | chr3:16628704-16630473 REVERSEweakly similar to ( 147) CYSP2_MAIZE Cysteine proteinase 2 precursor (EC 3.4.22.-) - Zea mays (Maize)weakly similar to ( 145) loc_os09g27030 12009.m059986 protein thiol protease aleurain precursor, putative, expressed  no original description 	T
3.5	minor CHO metabolism.others	MDP0000211180	 moderately similar to ( 356) AT1G59960 | Symbols:  | aldo/keto reductase, putative | chr1:22071410-22073067 REVERSEmoderately similar to ( 348) 6DCS_SOYBN NAD(P)H-dependent 6'-deoxychalcone synthase (EC 2.3.1.170) - Glycine max (Soybean)moderately similar to ( 337) loc_os03g13390 12003.m06784 protein NAD(P)H-dependent oxidoreductase, putative, expressed  no original description 	T
31.3.1	cell.cycle.peptidylprolyl isomerase	MDP0000119443	 weakly similar to ( 152) AT5G48580 | Symbols: FKBP15-2 | FKBP15-2; FK506 binding / peptidyl-prolyl cis-trans isomerase | chr5:19696156-19697304 REVERSEweakly similar to ( 150) FKB15_VICFA FK506-binding protein 2 precursor (EC 5.2.1.8) (Peptidyl-prolyl cis-trans isomerase) (PPIase) (Rotamase) (15 kDa FKBP) (FKBP-15) - Vicia faba (Broad bean)weakly similar to ( 157) loc_os09g32526 12009.m22153 protein FK506-binding protein 2-1 precursor, putative, expressed  no original description 	T
29.6	protein.folding	MDP0000119443	 weakly similar to ( 152) AT5G48580 | Symbols: FKBP15-2 | FKBP15-2; FK506 binding / peptidyl-prolyl cis-trans isomerase | chr5:19696156-19697304 REVERSEweakly similar to ( 150) FKB15_VICFA FK506-binding protein 2 precursor (EC 5.2.1.8) (Peptidyl-prolyl cis-trans isomerase) (PPIase) (Rotamase) (15 kDa FKBP) (FKBP-15) - Vicia faba (Broad bean)weakly similar to ( 157) loc_os09g32526 12009.m22153 protein FK506-binding protein 2-1 precursor, putative, expressed  no original description 	T
27.3.10	RNA.regulation of transcription.C2C2(Zn) YABBY family	MDP0000252640	 weakly similar to ( 186) AT1G08465 | Symbols: YAB2 | YAB2 (YABBY2); transcription factor | chr1:2676033-2679382 FORWARDweakly similar to ( 135) YAB1_ORYSA Protein YABBY (OsYAB1) (Filamentous flower protein 1) - Oryza sativa (Rice)weakly similar to ( 155) loc_os03g44710 12003.m101420 protein axial regulator YABBY2, putative, expressed   no original description 	T
29.4	protein.postranslational modification	MDP0000284163	 highly similar to ( 570) AT5G06750 | Symbols:  | protein phosphatase 2C family protein / PP2C family protein | chr5:2086403-2088245 REVERSEhighly similar to ( 553) loc_os02g46080 12002.m77836 protein catalytic/ protein phosphatase type 2C, putative, expressed  no original description 	T
34.21	transport.calcium	MDP0000318887	 moderately similar to ( 327) AT1G08960 | Symbols: CAX11, ATCAX11 | CAX11; calcium:sodium antiporter/ cation:cation antiporter | chr1:2879698-2881877 FORWARDvery weakly similar to (99.4) loc_os10g30070 12010.m05869 protein sodium/potassium/calcium exchanger 6 precursor, putative, expressed   PRK07003 TrbL PRK08026 tolA  no original description 	T
29.4.1.57	protein.postranslational modification.kinase.receptor like cytoplasmatic kinase VII	MDP0000150425	 highly similar to ( 790) AT4G02010 | Symbols:  | protein kinase family protein | chr4:881457-885222 FORWARDmoderately similar to ( 233) NORK_MEDTR Nodulation receptor kinase precursor (EC 2.7.11.1) (Does not make infections protein 2) (Symbiosis receptor-like kinase) (MtSYMRK) - Medicago truncatula (Barrel medic)highly similar to ( 696) loc_os01g14932 12001.m42949 protein NAK-like ser/thr protein kinase, putative, expressed  no original description 	T
35.2	not assigned.unknown	MDP0000155548	 highly similar to ( 520) AT3G13990 | Symbols:  | unknown protein | chr3:4625708-4630383 REVERSEmoderately similar to ( 326) loc_os06g10430 12006.m05761 protein gb protein, putative, expressed   no original description 	T
35.2	not assigned.unknown	MDP0000906654	 weakly similar to ( 161) AT3G06145 | Symbols:  | unknown protein | chr3:1860118-1860618 REVERSE  no original description 	T
27.3.99	RNA.regulation of transcription.unclassified	MDP0000761511	 highly similar to ( 536) AT4G01730 | Symbols:  | zinc ion binding | chr4:749574-752034 FORWARDmoderately similar to ( 416) loc_os12g16210 12012.m05579 protein metal ion binding protein, putative, expressed  no original description 	T
35.2	not assigned.unknown	MDP0000452899	 very weakly similar to (57.8) AT3G17120 | Symbols:  | unknown protein | chr3:5842410-5843246 FORWARDvery weakly similar to (61.6) loc_os01g19410 12001.m08478 protein expressed protein  no original description 	T
27.2	RNA.transcription	MDP0000939377	 highly similar to ( 599) AT1G64860 | Symbols: SIGA, SIG1, SIG2, SIGB, RPOD1 | SIGA (SIGMA FACTOR A); DNA binding / DNA-directed RNA polymerase/ sigma factor/ transcription factor | chr1:24098497-24100746 FORWARDmoderately similar to ( 441) loc_os08g06630 12008.m26470 protein RNA polymerase sigma factor rpoD, putative, expressed  no original description 	T
30.2.17	signalling.receptor kinases.DUF 26	MDP0000163412	 highly similar to ( 964) AT1G29750 | Symbols: RKF1 | RKF1 (RECEPTOR-LIKE KINASE IN FLOWERS 1); ATP binding / kinase/ protein serine/threonine kinase/ receptor signaling protein serine/threonine kinase | chr1:10414071-10420469 REVERSEmoderately similar to ( 210) NORK_PEA Nodulation receptor kinase precursor (EC 2.7.11.1) - Pisum sativum (Garden pea)highly similar to ( 744) loc_os09g17630 12009.m05021 protein receptor-like protein kinase 2, putative, expressed  no original description 	T
35.2	not assigned.unknown	MDP0000308573	 moderately similar to ( 222) AT3G59090 | Symbols:  | LOCATED IN: endomembrane system; EXPRESSED IN: 24 plant structures; EXPRESSED DURING: 15 growth stages; BEST Arabidopsis thaliana protein match is: TOM1 (TOBAMOVIRUS MULTIPLICATION 1); protein binding (TAIR:AT4G21790.1); Has 115 Blast hits to 115 proteins in 19 species: Archae - 0; Bacteria - 0; Metazoa - 0; Fungi - 2; Plants - 108; Viruses - 0; Other Eukaryotes - 5 (source: NCBI BLink). | chr3:21839380-21842348 FORWARDweakly similar to ( 165) loc_os01g54784 12001.m11640 protein expressed protein  no original description 	T
35.2	not assigned.unknown	MDP0000151595	 weakly similar to ( 164) AT2G47360 | Symbols:  | unknown protein | chr2:19437820-19438731 FORWARD    no original description 	T
35.2	not assigned.unknown	MDP0000189787	 weakly similar to ( 148) AT5G03345 | Symbols:  | FUNCTIONS IN: molecular_function unknown; INVOLVED IN: biological_process unknown; LOCATED IN: endomembrane system; EXPRESSED IN: 24 plant structures; EXPRESSED DURING: 15 growth stages; CONTAINS InterPro DOMAIN/s: Magnesium transporter (InterPro:IPR018937); Has 120 Blast hits to 120 proteins in 49 species: Archae - 0; Bacteria - 0; Metazoa - 94; Fungi - 0; Plants - 25; Viruses - 0; Other Eukaryotes - 1 (source: NCBI BLink). | chr5:814196-815478 FORWARDweakly similar to ( 130) loc_os12g37880 12012.m07574 protein expressed protein no original description 	T
19.15	tetrapyrrole synthesis.chlorophyll synthase	MDP0000269497	 highly similar to ( 543) AT3G51820 | Symbols: ATG4, G4, CHLG | G4; chlorophyll synthetase | chr3:19216301-19218934 REVERSEhighly similar to ( 508) loc_os05g28200 12005.m083694 protein bacteriochlorophyll synthase 34 kDa chain, putative, expressed no original description 	T
35.2	not assigned.unknown	MDP0000572846	 very weakly similar to (93.6) AT5G03460 | Symbols:  | unknown protein | chr5:864391-865509 FORWARDweakly similar to ( 107) loc_os02g57766 12002.m100592 protein expressed protein no original description 	T
29.3.4.1	protein.targeting.secretory pathway.ER	MDP0000760092	 moderately similar to ( 469) AT4G38790 | Symbols:  | ER lumen protein retaining receptor family protein | chr4:18111426-18112977 FORWARDweakly similar to ( 104) ERD2_PETHY ER lumen protein retaining receptor (HDEL receptor) (PGP169-12) - Petunia hybrida (Petunia)moderately similar to ( 450) loc_os08g43470 12008.m080230 protein ER lumen protein retaining receptor C28H8.4, putative, expressed no original description 	T
27.3.82	RNA.regulation of transcription.plant TF (pbf2)	MDP0000268451	 moderately similar to ( 347) AT1G14410 | Symbols: ATWHY1, PTAC1, WHY1 | WHY1 (WHIRLY 1); DNA binding / telomeric DNA binding | chr1:4929352-4930810 REVERSEmoderately similar to ( 254) loc_os06g05350 12006.m05264 protein DNA-binding protein p24, putative, expressed  no original description 	T
27.3.67	RNA.regulation of transcription.putative transcription regulator	MDP0000268451	 moderately similar to ( 347) AT1G14410 | Symbols: ATWHY1, PTAC1, WHY1 | WHY1 (WHIRLY 1); DNA binding / telomeric DNA binding | chr1:4929352-4930810 REVERSEmoderately similar to ( 254) loc_os06g05350 12006.m05264 protein DNA-binding protein p24, putative, expressed  no original description 	T
29.5.5	protein.degradation.serine protease	MDP0000383043	 moderately similar to ( 459) AT2G27920 | Symbols: SCPL51 | SCPL51 (SERINE CARBOXYPEPTIDASE-LIKE 51); serine-type carboxypeptidase | chr2:11885777-11888732 REVERSEvery weakly similar to (83.2) CBPX_ORYSA Serine carboxypeptidase-like precursor (EC 3.4.16.-) - Oryza sativa (Rice)moderately similar to ( 397) loc_os03g27590 12003.m35571 protein retinoid-inducible serine carboxypeptidase precursor, putative, expressed no original description 	T
29.6	protein.folding	MDP0000656233	 moderately similar to ( 484) AT3G03960 | Symbols:  | chaperonin, putative | chr3:1024432-1027604 FORWARDvery weakly similar to (95.9) TCPE2_AVESA T-complex protein 1 subunit epsilon (TCP-1-epsilon) (CCT-epsilon) (TCP-K36) - Avena sativa (Oat)highly similar to ( 526) loc_os03g59020 12003.m10803 protein T-complex protein 1 subunit theta, putative, expressed  no original description 	T
27.3.38	RNA.regulation of transcription.AT-rich interaction domain containing transcription factor family	MDP0000306004	 moderately similar to ( 226) AT1G76510 | Symbols:  | ARID/BRIGHT DNA-binding domain-containing protein | chr1:28708927-28712498 REVERSEweakly similar to ( 198) loc_os06g41730 12006.m31901 protein ARID/BRIGHT DNA binding domain containing protein, expressed  no original description 	T
30.3	signalling.calcium	MDP0000162025	 weakly similar to ( 150) AT3G50770 | Symbols:  | calmodulin-related protein, putative | chr3:18873987-18874604 FORWARDvery weakly similar to (70.9) CALM1_SOLTU Calmodulin-1 (CaM-1) - Solanum tuberosum (Potato)weakly similar to ( 108) loc_os01g56030 12001.m11759 protein calmodulin-like protein 41, putative, expressed  no original description 	T
35.1.5	not assigned.no ontology.pentatricopeptide (PPR) repeat-containing protein	MDP0000249255	 moderately similar to ( 298) AT1G74400 | Symbols:  | pentatricopeptide (PPR) repeat-containing protein | chr1:27963953-27965341 FORWARDvery weakly similar to (70.5) EL5_ORYSA E3 ubiquitin-protein ligase EL5 (EC 6.3.2.-) - Oryza sativa (Rice)moderately similar to ( 227) loc_os08g42610 12008.m08239 protein tetratricopeptide-like helical, putative, expressed  no original description 	T
20.2.2	stress.abiotic.cold	MDP0000348471	 moderately similar to ( 426) AT5G55530 | Symbols:  | C2 domain-containing protein | chr5:22494439-22495656 FORWARDmoderately similar to ( 320) loc_os03g09840 12003.m35067 protein C2 domain containing protein, expressed   no original description 	T
35.2	not assigned.unknown	MDP0000343018	  no original description 	T
11.1.12	lipid metabolism.FA synthesis and FA elongation.ACP protein	MDP0000468488	 weakly similar to ( 124) AT4G25050 | Symbols: ACP4 | ACP4 (acyl carrier protein 4); acyl carrier | chr4:12870178-12871024 FORWARDweakly similar to ( 129) ACP2_HORVU Acyl carrier protein 2, chloroplast precursor (Acyl carrier protein II) (ACP II) - Hordeum vulgare (Barley)weakly similar to ( 125) loc_os12g34890 12012.m07282 protein acyl carrier protein 1, chloroplast precursor, putative, expressed no original description 	T
16.8.3.1	secondary metabolism.flavonoids.dihydroflavonols.dihydroflavonol 4-reductase	MDP0000494976	 highly similar to ( 518) AT5G42800 | Symbols: DFR, TT3, M318 | DFR (DIHYDROFLAVONOL 4-REDUCTASE); dihydrokaempferol 4-reductase | chr5:17164296-17165864 REVERSEhighly similar to ( 565) DFRA_VITVI Dihydroflavonol-4-reductase (EC 1.1.1.219) (DFR) (Dihydrokaempferol 4-reductase) - Vitis vinifera (Grape)moderately similar to ( 333) loc_os01g44260 12001.m10683 protein dihydroflavonol-4-reductase, putative, expressed   no original description 	T
29.5.5	protein.degradation.serine protease	MDP0000751972	 moderately similar to ( 430) AT1G47710 | Symbols:  | serpin, putative / serine protease inhibitor, putative | chr1:17558271-17560061 FORWARDmoderately similar to ( 333) PRTZ_HORVU Protein Z (Z4) (Major endosperm albumin) - Hordeum vulgare (Barley)moderately similar to ( 371) loc_os03g41438 12003.m09209 protein protein Z, putative, expressed   no original description 	T
27.3.12	RNA.regulation of transcription.C3H zinc finger family	MDP0000673185	 highly similar to ( 813) AT2G41900 | Symbols:  | zinc finger (CCCH-type) family protein | chr2:17491352-17493502 FORWARDhighly similar to ( 687) loc_os03g49170 12003.m101454 protein nucleic acid binding protein, putative, expressed   no original description 	T
33.99	development.unspecified	MDP0000261277	 moderately similar to ( 385) AT2G33340 | Symbols:  | nucleotide binding / ubiquitin-protein ligase | chr2:14126703-14131000 REVERSEvery weakly similar to (77.4) PF20_CHLRE Flagellar WD repeat protein PF20 - Chlamydomonas reinhardtiimoderately similar to ( 410) loc_os10g32880 12010.m21914 protein pre-mRNA-splicing factor 19, putative, expressed  no original description 	T
29.3.4.99	protein.targeting.secretory pathway.unspecified	MDP0000789115	 moderately similar to ( 233) AT3G07680 | Symbols:  | emp24/gp25L/p24 family protein | chr3:2455627-2456652 FORWARDmoderately similar to ( 234) loc_os08g42050 12008.m08183 protein transmembrane emp24 domain-containing protein 3 precursor, putative, expressed no original description 	T
27.4	RNA.RNA binding	MDP0000672129	 weakly similar to ( 179) AT3G26420 | Symbols: ATRZ-1A | ATRZ-1A; RNA binding / nucleotide binding | chr3:9671953-9673055 FORWARDweakly similar to ( 119) GRP2_SINAL Glycine-rich RNA-binding protein GRP2A - Sinapis alba (White mustard) (Brassica hirta)weakly similar to ( 157) loc_os03g61990 12003.m101556 protein glycine-rich RNA-binding protein 8, putative, expressed  no original description 	T
35.2	not assigned.unknown	MDP0000174754	 highly similar to ( 520) AT5G04550 | Symbols:  | unknown protein | chr5:1303757-1305556 REVERSEweakly similar to ( 193) loc_os12g05180 12012.m04512 protein expressed protein TrbL  no original description 	T
35.1	not assigned.no ontology	MDP0000155154	 weakly similar to ( 154) AT1G54130 | Symbols: RSH3, AT-RSH3, ATRSH3 | RSH3 (RELA/SPOT HOMOLOG 3); GTP diphosphokinase | chr1:20211177-20213761 FORWARDhighly similar to ( 786) loc_os02g47120 12002.m09747 protein region found in RelA / SpoT proteins containing protein, expressed   no original description 	T
17.6.2	hormone metabolism.gibberelin.signal transduction	MDP0000212192	 highly similar to ( 801) AT3G11540 | Symbols: SPY | SPY (SPINDLY); protein N-acetylglucosaminyltransferase/ protein binding / transferase, transferring glycosyl groups | chr3:3632842-3637547 FORWARDhighly similar to ( 782) SPY_PETHY Probable UDP-N-acetylglucosamine--peptide N-acetylglucosaminyltransferase SPINDLY (EC 2.4.1.-) (PhSPY) - Petunia hybrida (Petunia)highly similar to ( 751) loc_os08g44510 12008.m08424 protein UDP-N-acetylglucosamine--peptide N-acetylglucosaminyltransferase SPINDLY, putative, expressed   no original description 	T
35.2	not assigned.unknown	MDP0000339955	 very weakly similar to (68.6) AT4G29735 | Symbols:  | unknown protein | chr4:14562962-14564277 REVERSEvery weakly similar to (90.1) loc_os06g37070 12006.m08242 protein expressed protein  no original description 	T
29.5.9	protein.degradation.AAA type	MDP0000193605	 highly similar to ( 846) AT1G16900 | Symbols:  | sugar binding / transferase, transferring glycosyl groups | chr1:5779262-5782303 REVERSEhighly similar to ( 791) loc_os01g11070 12001.m07727 protein alpha-1,2-mannosyltransferase ALG9, putative, expressed  TrbL  no original description 	T
34.1.1	transport.p- and v-ATPases.H+-transporting two-sector ATPase	MDP0000844729	 nearly identical (1149) AT1G78900 | Symbols: VHA-A | VHA-A (VACUOLAR ATP SYNTHASE SUBUNIT A); ATP binding / hydrogen ion transporting ATP synthase, rotational mechanism / hydrolase, acting on acid anhydrides, catalyzing transmembrane movement of substances / proton-transporting ATPase, rotational mechanism | chr1:29660463-29664575 FORWARDnearly identical (1169) VATA_DAUCA Vacuolar ATP synthase catalytic subunit A (EC 3.6.3.14) (V-ATPase subunit A) (Vacuolar proton pump subunit alpha) (V-ATPase 69 kDa subunit) - Daucus carota (Carrot)nearly identical (1108) loc_os06g45120 12006.m09039 protein vacuolar ATP synthase catalytic subunit A, putative, expressed  no original description 	T
31.1	cell.organisation	MDP0000245712	 moderately similar to ( 204) AT5G59880 | Symbols: ADF3 | ADF3 (ACTIN DEPOLYMERIZING FACTOR 3); actin binding | chr5:24120382-24121628 FORWARDmoderately similar to ( 214) ADF2_PETHY Actin-depolymerizing factor 2 (ADF 2) - Petunia hybrida (Petunia)moderately similar to ( 204) loc_os05g02250 12005.m64199 protein expressed protein no original description 	T
26.10	misc.cytochrome P450	MDP0000199846	 moderately similar to ( 387) AT3G26310 | Symbols: CYP71B35 | CYP71B35; electron carrier/ heme binding / iron ion binding / monooxygenase/ oxygen binding | chr3:9641089-9642779 REVERSEmoderately similar to ( 420) C71A9_SOYBN Cytochrome P450 71A9 (EC 1.14.-.-) (P450 CP1) - Glycine max (Soybean)moderately similar to ( 382) loc_os12g16720 12012.m05630 protein cytochrome P450 71A1, putative, expressed  no original description 	T
20.1.3.1	stress.biotic.signalling.MLO-like	MDP0000141595	 moderately similar to ( 498) AT2G17480 | Symbols: MLO8, ATMLO8 | MLO8 (MILDEW RESISTANCE LOCUS O 8); calmodulin binding | chr2:7590559-7593768 REVERSEmoderately similar to ( 305) MLOH1_ORYSA MLO protein homolog 1 (OsMLO1) - Oryza sativa (Rice)moderately similar to ( 335) loc_os10g39520 12010.m06723 protein MLO-like protein 10, putative, expressed  no original description 	T
29.2.1.2.2.57	protein.synthesis.ribosomal protein.eukaryotic.60S subunit.L7A	MDP0000427753	 moderately similar to ( 223) AT5G08180 | Symbols:  | ribosomal protein L7Ae/L30e/S12e/Gadd45 family protein | chr5:2631843-2633374 REVERSEvery weakly similar to (52.4) RL7A_ORYSA 60S ribosomal protein L7a - Oryza sativa (Rice)moderately similar to ( 230) loc_os06g16290 12006.m06342 protein h/ACA ribonucleoprotein complex subunit 2, putative, expressed  no original description 	T
29.2.1.99.99	protein.synthesis.ribosomal protein.unknown.unknown	MDP0000947001	 weakly similar to ( 144) AT3G56010 | Symbols:  | unknown protein | chr3:20788758-20789656 FORWARD no original description 	T
16.1.1.10	secondary metabolism.isoprenoids.non-mevalonate pathway.geranylgeranyl pyrophosphate synthase	MDP0000576390	 moderately similar to ( 428) AT4G36810 | Symbols: GGPS1 | GGPS1 (GERANYLGERANYL PYROPHOSPHATE SYNTHASE 1); farnesyltranstransferase | chr4:17343513-17344628 FORWARDmoderately similar to ( 471) GGPPS_HEVBR Geranylgeranyl pyrophosphate synthetase, chloroplast precursor (GGPP synthetase) [Includes: Dimethylallyltranstransferase (EC 2.5.1.1); Geranyltranstransferase (EC 2.5.1.10); Farnesyltranstransferase (EC 2.5.1.29)] - Hevea brasiliensismoderately similar to ( 355) loc_os07g39270 12007.m28997 protein geranylgeranyl pyrophosphate synthetase 1, chloroplast precursor, putative, expressed   no original description 	T
29.5.11.20	protein.degradation.ubiquitin.proteasom	MDP0000137279	 moderately similar to ( 347) AT3G22630 | Symbols: PBD1, PRCGB | PBD1 (20S PROTEASOME BETA SUBUNIT D1); peptidase/ threonine-type endopeptidase | chr3:8009709-8010774 REVERSEmoderately similar to ( 295) PSB2_ORYSA Proteasome subunit beta type 2 (EC 3.4.25.1) (20S proteasome alpha subunit D) (20S proteasome subunit beta-4) - Oryza sativa (Rice)moderately similar to ( 295) loc_os03g48930 12003.m09894 protein proteasome subunit beta type 2, putative, expressedMSP1_C  no original description 	T
24.1	Biodegradation of Xenobiotics.hydroxyacylglutathione hydrolase	MDP0000123102	 moderately similar to ( 356) AT1G53580 | Symbols: GLX2-3, ETHE1, GLY3 | GLY3 (GLYOXALASE II 3); hydrolase/ hydroxyacylglutathione hydrolase | chr1:19991542-19993250 REVERSEmoderately similar to ( 340) loc_os01g47690 12001.m43132 protein hydroxyacylglutathione hydrolase 3, mitochondrial precursor, putative, expressed no original description 	T
1.1.1.1	PS.lightreaction.photosystem II.LHC-II	MDP0000262192	 moderately similar to ( 486) AT5G54270 | Symbols: LHCB3, LHCB3*1 | LHCB3 (LIGHT-HARVESTING CHLOROPHYLL B-BINDING PROTEIN 3); structural molecule | chr5:22038424-22039383 FORWARDmoderately similar to ( 467) CB23_HORVU Chlorophyll a-b binding protein of LHCII type III, chloroplast precursor (CAB) - Hordeum vulgare (Barley)moderately similar to ( 464) loc_os07g37550 12007.m079717 protein chlorophyll a-b binding protein of LHCII type III, chloroplast precursor, putative, expressed  no original description 	T
29.1.3	protein.aa activation.threonine-tRNA ligase	MDP0000136090	 highly similar to ( 950) AT5G26830 | Symbols:  | threonyl-tRNA synthetase / threonine--tRNA ligase (THRRS) | chr5:9437351-9441568 FORWARDhighly similar to ( 952) loc_os08g19850 12008.m06052 protein threonyl-tRNA synthetase, mitochondrial precursor, putative, expressed  no original description 	T
27.3.99	RNA.regulation of transcription.unclassified	MDP0000232150	 moderately similar to ( 271) AT5G07900 | Symbols:  | mitochondrial transcription termination factor family protein / mTERF family protein | chr5:2520188-2521405 FORWARDweakly similar to ( 146) loc_os07g24090 12007.m06687 protein mTERF family protein, expressed  no original description 	T
35.2	not assigned.unknown	MDP0000293856	 highly similar to ( 888) AT1G19835 | Symbols:  | unknown protein | chr1:6856212-6859513 REVERSEvery weakly similar to (55.5) MFP1_TOBAC MAR-binding filament-like protein 1-1 - Nicotiana tabacum (Common tobacco)highly similar to ( 800) loc_os03g14250 12003.m06867 protein expressed protein  no original description 	T
29.2.1.1.3.2.34	protein.synthesis.ribosomal protein.prokaryotic.unknown organellar.50S subunit.L34	MDP0000338473	 weakly similar to ( 145) AT3G13882 | Symbols:  | structural constituent of ribosome | chr3:4574521-4575852 FORWARDvery weakly similar to (87.8) loc_os01g39010 12001.m10181 protein LIN1 protein, putative, expressed   no original description 	T
29.2.1.2.1.8	protein.synthesis.ribosomal protein.eukaryotic.40S subunit.S8	MDP0000165455	 moderately similar to ( 300) AT5G20290 | Symbols:  | 40S ribosomal protein S8 (RPS8A) | chr5:6851695-6853012 REVERSEmoderately similar to ( 325) RS8_ORYSA 40S ribosomal protein S8 - Oryza sativa (Rice)moderately similar to ( 325) loc_os02g28810 12002.m33684 protein 40S ribosomal protein S8, putative, expressed   no original description 	T
35.2	not assigned.unknown	MDP0000166235	 moderately similar to ( 238) AT3G07760 | Symbols:  | FUNCTIONS IN: molecular_function unknown; INVOLVED IN: biological_process unknown; LOCATED IN: cellular_component unknown; EXPRESSED IN: 24 plant structures; EXPRESSED DURING: 15 growth stages; CONTAINS InterPro DOMAIN/s: Sterile alpha motif SAM (InterPro:IPR001660), Sterile alpha motif homology (InterPro:IPR010993), Sterile alpha motif homology 2 (InterPro:IPR011510); Has 31 Blast hits to 31 proteins in 12 species: Archae - 0; Bacteria - 0; Metazoa - 4; Fungi - 6; Plants - 19; Viruses - 0; Other Eukaryotes - 2 (source: NCBI BLink). | chr3:2476723-2477947 FORWARDmoderately similar to ( 241) loc_os12g32992 12012.m07097 protein expressed protein no original description 	T
35.2	not assigned.unknown	MDP0000191806	 weakly similar to ( 200) AT2G24020 | Symbols:  | unknown protein | chr2:10217869-10219269 REVERSEweakly similar to ( 197) loc_os02g08380 12002.m06184 protein complex interacting protein 9, putative, expressed  no original description 	T
26.1	misc.misc2	MDP0000224701	 moderately similar to ( 334) AT3G23600 | Symbols:  | dienelactone hydrolase family protein | chr3:8473833-8475655 FORWARDweakly similar to ( 163) E134_MAIZE Endo-1,3;1,4-beta-D-glucanase precursor (EC 3.2.1.-) - Zea mays (Maize)moderately similar to ( 312) loc_os05g33100 12005.m07542 protein endo-1,3;1,4-beta-D-glucanase precursor, putative, expressed no original description 	T
35.2	not assigned.unknown	MDP0000298194	 highly similar to ( 816) AT5G48160 | Symbols: OBE2 | OBE2 (OBERON2); protein binding / zinc ion binding | chr5:19528019-19529820 REVERSEhighly similar to ( 710) loc_os12g32980 12012.m07096 protein potyvirus VPg interacting protein, putative, expressed  no original description 	T
21.1	redox.thioredoxin	MDP0000252195	 moderately similar to ( 215) AT2G04700 | Symbols:  | ferredoxin thioredoxin reductase catalytic beta chain family protein | chr2:1646961-1648345 FORWARDmoderately similar to ( 222) FTRC_SOYBN Ferredoxin-thioredoxin reductase catalytic chain, chloroplast precursor (EC 1.18.-.-) (FTR-C) - Glycine max (Soybean)weakly similar to ( 191) loc_os09g07570 12009.m059937 protein ferredoxin-thioredoxin reductase catalytic chain, chloroplast precursor, putative, expressed   no original description 	T
35.2	not assigned.unknown	MDP0000380467	 very weakly similar to (96.7) AT2G46150 | Symbols:  | FUNCTIONS IN: molecular_function unknown; INVOLVED IN: biological_process unknown; LOCATED IN: plasma membrane; EXPRESSED IN: 15 plant structures; EXPRESSED DURING: 7 growth stages; CONTAINS InterPro DOMAIN/s: Harpin-induced 1 (InterPro:IPR010847); BEST Arabidopsis thaliana protein match is: unknown protein (TAIR:AT3G54200.1); Has 137 Blast hits to 136 proteins in 12 species: Archae - 0; Bacteria - 0; Metazoa - 0; Fungi - 4; Plants - 133; Viruses - 0; Other Eukaryotes - 0 (source: NCBI BLink). | chr2:18961424-18962089 FORWARDvery weakly similar to (75.1) loc_os01g53470 12001.m11515 protein harpin-induced protein, putative, expressed   no original description 	T
29.5.11.20	protein.degradation.ubiquitin.proteasom	MDP0000265659	 highly similar to ( 677) AT1G20200 | Symbols: EMB2719, HAP15 | EMB2719 (EMBRYO DEFECTIVE 2719); enzyme regulator | chr1:7001409-7004154 REVERSEhighly similar to ( 717) PSMD3_TOBAC Probable 26S proteasome non-ATPase regulatory subunit 3 (26S proteasome subunit S3) (Nuclear antigen 21D7) - Nicotiana tabacum (Common tobacco)highly similar to ( 650) loc_os08g43640 12008.m080046 protein 26S proteasome non-ATPase regulatory subunit 3, putative, expressed   no original description 	T
35.1	not assigned.no ontology	MDP0000124371	 moderately similar to ( 422) AT1G21070 | Symbols:  | transporter-related | chr1:7376148-7377810 REVERSEmoderately similar to ( 412) loc_os07g39280 12007.m08170 protein integral membrane protein like, putative, expressed  no original description 	T
35.2	not assigned.unknown	MDP0000275880	 moderately similar to ( 288) AT1G21350 | Symbols:  | antioxidant/ oxidoreductase | chr1:7477376-7479012 REVERSEmoderately similar to ( 253) loc_os07g15670 12007.m29122 protein thiol-disulfide isomerase and thioredoxins, putative, expressed  no original description 	T
27.3.99	RNA.regulation of transcription.unclassified	MDP0000218998	 highly similar to ( 739) AT5G58760 | Symbols: DDB2 | DDB2 (damaged DNA-binding 2); nucleic acid binding / nucleotide binding / zinc ion binding | chr5:23730741-23733606 REVERSEhighly similar to ( 656) loc_os01g04870 12001.m07121 protein nucleic acid binding protein, putative, expressed  no original description 	T
35.1	not assigned.no ontology	MDP0000214122	 moderately similar to ( 312) AT5G10860 | Symbols:  | CBS domain-containing protein | chr5:3429173-3430142 REVERSEmoderately similar to ( 300) loc_os03g52690 12003.m101601 protein CBS domain protein, putative, expressed no original description 	T
34.9	transport.metabolite transporters at the mitochondrial membrane	MDP0000146924	 highly similar to ( 619) AT3G08580 | Symbols: AAC1 | AAC1 (ADP/ATP CARRIER 1); ATP:ADP antiporter/ binding | chr3:2605706-2607030 REVERSEhighly similar to ( 634) ADT1_GOSHI ADP,ATP carrier protein 1, mitochondrial precursor (ADP/ATP translocase 1) (Adenine nucleotide translocator 1) (ANT 1) - Gossypium hirsutum (Upland cotton)highly similar to ( 568) loc_os02g48720 12002.m33839 protein ADP,ATP carrier protein, mitochondrial precursor, putative, expressed no original description 	T
26.2	misc.UDP glucosyl and glucoronyl transferases	MDP0000157869	 highly similar to ( 823) AT5G15470 | Symbols: GAUT14 | GAUT14 (Galacturonosyltransferase 14); polygalacturonate 4-alpha-galacturonosyltransferase/ transferase, transferring glycosyl groups / transferase, transferring hexosyl groups | chr5:5021433-5024168 REVERSEhighly similar to ( 687) loc_os12g38930 12012.m07678 protein transferase, transferring glycosyl groups, putative, expressed  no original description 	T
30.5	signalling.G-proteins	MDP0000274576	 moderately similar to ( 344) AT5G62880 | Symbols: ARAC10, ATRAC10, ATROP11 | ARAC10; GTP binding | chr5:25237236-25238939 FORWARDmoderately similar to ( 349) RAC3_ORYSA Rac-like GTP-binding protein 3 (OsRac3) - Oryza sativa (Rice)moderately similar to ( 349) loc_os02g50860 12002.m10120 protein rac-like GTP-binding protein 3, putative, expressed no original description 	T
27.3.22	RNA.regulation of transcription.HB,Homeobox transcription factor family	MDP0000126553	 nearly identical (1352) AT4G32880 | Symbols: ATHB-8, ATHB8 | ATHB-8 (HOMEOBOX GENE 8); DNA binding / transcription factor | chr4:15863587-15867822 REVERSEnearly identical (1167) loc_os03g43930 12003.m09434 protein class III HD-Zip protein 4, putative, expressed  no original description 	T
30.2.11	signalling.receptor kinases.leucine rich repeat XI	MDP0000612779	 highly similar to ( 823) AT3G47110 | Symbols:  | leucine-rich repeat transmembrane protein kinase, putative | chr3:17347103-17350296 REVERSEmoderately similar to ( 431) RPK1_IPONI Receptor-like protein kinase precursor (EC 2.7.11.1) - Ipomoea nil (Japanese morning glory) (Pharbitis nil)highly similar to ( 771) loc_os01g05870 12001.m07218 protein receptor-like protein kinase precursor, putative, expressed  no original description 	T
31.4	cell.vesicle transport	MDP0000144263	 weakly similar to ( 195) AT3G15980 | Symbols:  | coatomer protein complex, subunit beta 2 (beta prime), putative | chr3:5412015-5418313 REVERSEweakly similar to ( 181) loc_os06g05180 12006.m71402 protein coatomer subunit beta, putative, expressed  no original description 	T
33.99	development.unspecified	MDP0000622177	 nearly identical (1575) AT3G16830 | Symbols: TPR2 | TPR2 (TOPLESS-RELATED 2) | chr3:5731709-5737531 FORWARDnearly identical (1466) loc_os01g15020 12001.m42634 protein lissencephaly type-1-like homology motif, putative, expressed    no original description 	T
20.2.3	stress.abiotic.drought/salt	MDP0000141807	 highly similar to ( 513) AT4G18030 | Symbols:  | dehydration-responsive family protein | chr4:10012850-10015267 REVERSEmoderately similar to ( 462) loc_os03g26200 12003.m07960 protein ankyrin protein kinase-like, putative, expressed  no original description 	T
26.9	misc.glutathione S transferases	MDP0000321664	 moderately similar to ( 298) AT1G06660 | Symbols:  | unknown protein | chr1:2037461-2040148 REVERSEweakly similar to ( 115) GSTF1_TOBAC Glutathione S-transferase PARB (EC 2.5.1.18) (GST class-phi) - Nicotiana tabacum (Common tobacco)weakly similar to ( 149) loc_os03g56590 12003.m10569 protein expressed protein  no original description 	T
7.2.3	OPP.non-reductive PP.ribulose-phosphate 3-epimerase	MDP0000276466	 moderately similar to ( 453) AT5G61410 | Symbols: RPE | RPE; catalytic/ ribulose-phosphate 3-epimerase | chr5:24684085-24685836 REVERSEmoderately similar to ( 471) RPE_SPIOL Ribulose-phosphate 3-epimerase, chloroplast precursor (EC 5.1.3.1) (Pentose-5-phosphate 3-epimerase) (PPE) (RPE) (R5P3E) - Spinacia oleracea (Spinach)moderately similar to ( 422) loc_os03g07300 12003.m101203 protein ribulose-phosphate 3-epimerase, chloroplast precursor, putative, expressed  no original description 	T
31.3	cell.cycle	MDP0000333807	 weakly similar to ( 180) AT4G03270 | Symbols: CYCD6;1 | CYCD6;1 (Cyclin D6;1); cyclin-dependent protein kinase | chr4:1432375-1433691 REVERSEvery weakly similar to (54.3) CCNB1_SOYBN G2/mitotic-specific cyclin S13-6 (B-like cyclin) - Glycine max (Soybean)weakly similar to ( 147) loc_os07g37010 12007.m29223 protein CYCD6, putative, expressed   no original description 	T
35.2	not assigned.unknown	MDP0000293637	 weakly similar to ( 114) AT1G28150 | Symbols:  | unknown protein | chr1:9835122-9835842 REVERSEweakly similar to ( 101) loc_os04g44590 12004.m101593 protein expressed protein no original description 	T
29.5.1	protein.degradation.subtilases	MDP0000304236	 nearly identical (1037) AT1G04110 | Symbols: SDD1 | SDD1 (STOMATAL DENSITY AND DISTRIBUTION); serine-type endopeptidase | chr1:1061457-1063784 REVERSEhighly similar to ( 916) loc_os03g04950 12003.m06021 protein subtilisin-like protease precursor, putative   no original description 	T
30.2.17	signalling.receptor kinases.DUF 26	MDP0000566547	 moderately similar to ( 309) AT5G38280 | Symbols: PR5K | PR5K; kinase/ transmembrane receptor protein serine/threonine kinase | chr5:15293325-15295838 REVERSEweakly similar to ( 175) KPRO_MAIZE Putative receptor protein kinase ZmPK1 precursor (EC 2.7.11.1) - Zea mays (Maize)moderately similar to ( 356) loc_os01g02790 12001.m06919 protein receptor kinase LRK14, putative, expressed  no original description 	T
29.4	protein.postranslational modification	MDP0000566547	 moderately similar to ( 309) AT5G38280 | Symbols: PR5K | PR5K; kinase/ transmembrane receptor protein serine/threonine kinase | chr5:15293325-15295838 REVERSEweakly similar to ( 175) KPRO_MAIZE Putative receptor protein kinase ZmPK1 precursor (EC 2.7.11.1) - Zea mays (Maize)moderately similar to ( 356) loc_os01g02790 12001.m06919 protein receptor kinase LRK14, putative, expressed  no original description 	T
10.3	cell wall.hemicellulose synthesis	MDP0000230681	 highly similar to ( 645) AT2G03220 | Symbols: FT1, ATFUT1, ATFT1, MUR2 | FT1 (FUCOSYLTRANSFERASE 1); fucosyltransferase/ transferase, transferring glycosyl groups | chr2:970401-972353 REVERSEhighly similar to ( 734) FUT1_PEA Galactoside 2-alpha-L-fucosyltransferase (EC 2.4.1.69) (Xyloglucan alpha-(1,2)-fucosyltransferase) (PsFT1) - Pisum sativum (Garden pea)highly similar to ( 531) loc_os02g52640 12002.m10294 protein galactoside 2-alpha-L-fucosyltransferase, putative, expressed  no original description 	T
3.1.1.2	minor CHO metabolism.raffinose family.galactinol synthases.putative	MDP0000209143	 highly similar to ( 503) AT1G60470 | Symbols: AtGolS4 | AtGolS4 (Arabidopsis thaliana galactinol synthase 4); transferase, transferring glycosyl groups / transferase, transferring hexosyl groups | chr1:22279181-22280454 REVERSEmoderately similar to ( 467) loc_os03g20120 12003.m07424 protein galactinol synthase 3, putative, expressed no original description 	T
33.99	development.unspecified	MDP0000217662	 nearly identical (1274) AT4G04940 | Symbols:  | transducin family protein / WD-40 repeat family protein | chr4:2511212-2517052 REVERSEvery weakly similar to (73.2) GBLP_NICPL Guanine nucleotide-binding protein subunit beta-like protein - Nicotiana plumbaginifolia (Leadwort-leaved tobacco)nearly identical (1012) loc_os06g43690 12006.m08899 protein WD repeat-containing protein 36, putative, expressed  no original description 	T
35.2	not assigned.unknown	MDP0000859563	 very weakly similar to (75.9) AT3G27880 | Symbols:  | unknown protein | chr3:10338429-10339157 FORWARD no original description 	T
35.2	not assigned.unknown	MDP0000330306	 very weakly similar to (77.8) AT3G07230 | Symbols:  | wound-responsive protein-related | chr3:2299920-2300183 FORWARDvery weakly similar to (91.3) PR4_PHAVU Wound-induced basic protein - Phaseolus vulgaris (Kidney bean) (French bean) no original description 	T
35.2	not assigned.unknown	MDP0000269413	 moderately similar to ( 254) AT2G46100 | Symbols:  | unknown protein | chr2:18953326-18954082 FORWARDvery weakly similar to (79.0) loc_os04g48860 12004.m78984 protein expressed protein   no original description 	T
26.28	misc.GDSL-motif lipase	MDP0000244607	 moderately similar to ( 423) AT1G53920 | Symbols: GLIP5 | GLIP5; carboxylesterase/ lipase | chr1:20137725-20139637 FORWARDweakly similar to ( 123) APG_BRANA Anter-specific proline-rich protein APG (Protein CEX) (Fragment) - Brassica napus (Rape)weakly similar to ( 183) loc_os09g04710 12009.m21966 protein anther-specific proline-rich protein APG precursor, putative, expressed TrbL  no original description 	T
29.5.11.20	protein.degradation.ubiquitin.proteasom	MDP0000948037	 highly similar to ( 793) AT3G05530 | Symbols: RPT5A, ATS6A.2 | RPT5A (REGULATORY PARTICLE TRIPLE-A ATPASE 5A); ATPase/ calmodulin binding | chr3:1603540-1605993 FORWARDhighly similar to ( 793) PRS6A_BRACM 26S protease regulatory subunit 6A homolog (TAT-binding protein homolog 1) (TBP-1) - Brassica campestris (Field mustard)highly similar to ( 777) loc_os06g07630 12006.m05485 protein 26S protease regulatory subunit 6A, putative, expressed  no original description 	T
29.5.9	protein.degradation.AAA type	MDP0000948037	 highly similar to ( 793) AT3G05530 | Symbols: RPT5A, ATS6A.2 | RPT5A (REGULATORY PARTICLE TRIPLE-A ATPASE 5A); ATPase/ calmodulin binding | chr3:1603540-1605993 FORWARDhighly similar to ( 793) PRS6A_BRACM 26S protease regulatory subunit 6A homolog (TAT-binding protein homolog 1) (TBP-1) - Brassica campestris (Field mustard)highly similar to ( 777) loc_os06g07630 12006.m05485 protein 26S protease regulatory subunit 6A, putative, expressed  no original description 	T
35.2	not assigned.unknown	MDP0000258929	 moderately similar to ( 357) loc_os06g02430 12006.m04976 protein expressed protein  no original description 	T
34	transport	MDP0000304733	 highly similar to ( 528) AT3G08960 | Symbols:  | binding / protein transporter | chr3:2730405-2736762 REVERSEmoderately similar to ( 416) loc_os07g49260 12007.m09135 protein protein transporter, putative, expressed  no original description 	T
35.2	not assigned.unknown	MDP0000156219	 weakly similar to ( 114) AT3G44940 | Symbols:  | unknown protein | chr3:16413861-16415134 REVERSEvery weakly similar to (56.2) loc_os05g02450 12005.m04781 protein expressed protein  no original description 	T
29.5.3	protein.degradation.cysteine protease	MDP0000291220	 moderately similar to ( 459) AT3G19390 | Symbols:  | cysteine proteinase, putative / thiol protease, putative | chr3:6723024-6724768 FORWARDmoderately similar to ( 444) ORYA_ORYSA Oryzain alpha chain precursor (EC 3.4.22.-) - Oryza sativa (Rice)moderately similar to ( 402) loc_os04g57440 12004.m101443 protein cysteine protease 1 precursor, putative, expressed no original description 	T
35.2	not assigned.unknown	MDP0000184081	 moderately similar to ( 463) AT5G27290 | Symbols:  | unknown protein | chr5:9618787-9620473 REVERSEmoderately similar to ( 415) loc_os01g28540 12001.m150592 protein stress regulated protein, putative, expressed  no original description 	T
35.2	not assigned.unknown	MDP0000625155	 weakly similar to ( 188) AT5G22090 | Symbols:  | unknown protein | chr5:7315003-7316394 REVERSEvery weakly similar to (87.4) loc_os03g49830 12003.m09981 protein expressed protein   TrbL PRK07003 MSP1_C  no original description 	T
17.7.1.2	hormone metabolism.jasmonate.synthesis-degradation.lipoxygenase	MDP0000755511	 nearly identical (1287) AT1G17420 | Symbols: LOX3 | LOX3; electron carrier/ iron ion binding / lipoxygenase/ metal ion binding / oxidoreductase, acting on single donors with incorporation of molecular oxygen, incorporation of two atoms of oxygen | chr1:5977512-5981384 FORWARDnearly identical (1135) LOX6_ORYSA Probable lipoxygenase 6 (EC 1.13.11.12) - Oryza sativa (Rice)nearly identical (1135) loc_os03g08220 12003.m06331 protein lipoxygenase 6, putative, expressed   no original description 	T
26.13	misc.acid and other phosphatases	MDP0000303326	 weakly similar to ( 182) AT5G34850 | Symbols: ATPAP26, PAP26 | PAP26 (PURPLE ACID PHOSPHATASE 26); acid phosphatase/ protein serine/threonine phosphatase | chr5:13108475-13111217 REVERSEweakly similar to ( 130) PPAF_SOYBN Purple acid phosphatase precursor (EC 3.1.3.2) (Manganese(II) purple acid phosphatase) - Glycine max (Soybean)weakly similar to ( 165) loc_os06g43640 12006.m31909 protein purple acid phosphatase precursor, putative, expressed  no original description 	T
33.99	development.unspecified	MDP0000253632	 nearly identical (1765) AT1G80490 | Symbols: TPR1 | TPR1 (TOPLESS-RELATED 1) | chr1:30261094-30266446 REVERSEvery weakly similar to (57.0) GBLP_ORYSA Guanine nucleotide-binding protein subunit beta-like protein (GPB-LR) (RWD) - Oryza sativa (Rice)nearly identical (1716) loc_os03g14980 12003.m101111 protein lissencephaly type-1-like homology motif, putative, expressed  no original description 	T
21.1	redox.thioredoxin	MDP0000417792	 weakly similar to ( 181) AT1G07700 | Symbols:  | thioredoxin family protein | chr1:2380297-2381127 FORWARDvery weakly similar to (54.3) TRXH_ORYSA Thioredoxin H-type (TRX-H) (Phloem sap 13 kDa protein 1) - Oryza sativa (Rice)weakly similar to ( 186) loc_os02g35900 12002.m100241 protein thioredoxin-like 2, chloroplast precursor, putative, expressed   no original description 	T
35.2	not assigned.unknown	MDP0000355003	  no original description 	T
29.6	protein.folding	MDP0000201315	 weakly similar to ( 189) AT5G64350 | Symbols: FKBP12, ATFKBP12 | FKBP12 (FK506-BINDING PROTEIN); FK506 binding / peptidyl-prolyl cis-trans isomerase | chr5:25734810-25735990 REVERSEweakly similar to ( 200) FKB12_VICFA Peptidyl-prolyl isomerase FKBP12 (EC 5.2.1.8) (12 kDa FK506-binding protein) (12 kDa FKBP) (FKBP-12) (Immunophilin FKBP12) (Peptidyl-prolyl cis-trans isomerase) (PPIase) (Rotamase) - Vicia faba (Broad bean)weakly similar to ( 184) loc_os02g52290 12002.m10260 protein peptidyl-prolyl isomerase FKBP12, putative, expressed no original description 	T
27.1.2	RNA.processing.RNA helicase	MDP0000321215	 highly similar to ( 822) AT5G61000 | Symbols:  | replication protein, putative | chr5:24549682-24552641 REVERSEhighly similar to ( 789) loc_os03g11540 12003.m06606 protein replication protein A 70 kDa DNA-binding subunit, putative, expressed  no original description 	T
30.4	signalling.phosphinositides	MDP0000233214	 nearly identical (2161) AT3G59770 | Symbols: SAC9 | SAC9; inositol or phosphatidylinositol phosphatase | chr3:22079281-22084785 REVERSEhighly similar to ( 916) loc_os01g25330 12001.m150579 protein SAC9, putative, expressed  no original description 	T
20.2.99	stress.abiotic.unspecified	MDP0000164131	 very weakly similar to (68.9) AT1G70850 | Symbols: MLP34 | MLP34 (MLP-LIKE PROTEIN 34) | chr1:26715429-26716744 REVERSEvery weakly similar to (66.2) loc_os04g39150 12004.m35333 protein major latex protein 22, putative, expressed no original description 	T
13.1.6.5.2	amino acid metabolism.synthesis.aromatic aa.tryptophan.anthranilate phosphoribosyltransferase	MDP0000256567	 moderately similar to ( 405) AT5G17990 | Symbols: TRP1, pat1 | TRP1 (tryptophan biosynthesis 1); anthranilate phosphoribosyltransferase | chr5:5957330-5959681 FORWARDmoderately similar to ( 365) loc_os03g03450 12003.m34993 protein anthranilate phosphoribosyltransferase, chloroplast precursor, putative, expressed  no original description 	T
26.9	misc.glutathione S transferases	MDP0000726332	 weakly similar to ( 148) AT2G30860 | Symbols: ATGSTF9, GLUTTR, ATGSTF7 | ATGSTF9 (GLUTATHIONE S-TRANSFERASE PHI 9); glutathione peroxidase/ glutathione transferase | chr2:13139132-13140079 FORWARDvery weakly similar to (89.4) GSTH1_ORYSA Probable glutathione S-transferase GSTF1 (EC 2.5.1.18) (GST-I) - Oryza sativa (Rice)very weakly similar to (96.3) loc_os03g04260 12003.m05957 protein glutathione S-transferase, putative, expressed  no original description 	T
35.2	not assigned.unknown	MDP0000365990	   no original description 	T
30.2.11	signalling.receptor kinases.leucine rich repeat XI	MDP0000647479	 moderately similar to ( 318) AT1G45616 | Symbols: AtRLP6 | AtRLP6 (Receptor Like Protein 6); protein binding | chr1:17183550-17186534 REVERSEweakly similar to ( 166) PSKR_DAUCA Phytosulfokine receptor precursor (EC 2.7.11.1) (Phytosulfokine LRR receptor kinase) - Daucus carota (Carrot)moderately similar to ( 363) loc_os01g04070 12001.m07042 protein verticillium wilt disease resistance protein, putative, expressed   no original description 	T
26.10	misc.cytochrome P450	MDP0000248869	 moderately similar to ( 409) AT3G48270 | Symbols: CYP71A26 | CYP71A26; electron carrier/ heme binding / iron ion binding / monooxygenase/ oxygen binding | chr3:17876571-17878173 FORWARDmoderately similar to ( 372) C71A4_SOLME Cytochrome P450 71A4 (EC 1.14.-.-) (CYPLXXIA4) (P-450EG2) - Solanum melongena (Eggplant) (Aubergine)moderately similar to ( 306) loc_os01g12770 12001.m07890 protein cytochrome P450 71A26, putative, expressed  no original description 	T
10.7	cell wall.modification	MDP0000398765	 moderately similar to ( 491) AT5G13870 | Symbols: EXGT-A4 | EXGT-A4 (ENDOXYLOGLUCAN TRANSFERASE A4); hydrolase, acting on glycosyl bonds / hydrolase, hydrolyzing O-glycosyl compounds / xyloglucan:xyloglucosyl transferase | chr5:4475089-4476217 REVERSEhighly similar to ( 504) XTH_SOYBN Probable xyloglucan endotransglucosylase/hydrolase precursor (EC 2.4.1.207) (Fragment) - Glycine max (Soybean)moderately similar to ( 466) loc_os11g33270 12011.m07226 protein xyloglucan endotransglucosylase/hydrolase precursor, putative, expressed  no original description 	T
29.3.3	protein.targeting.chloroplast	MDP0000232493	 moderately similar to ( 350) AT3G24590 | Symbols: PLSP1 | PLSP1 (plastidic type I signal peptidase 1); peptidase | chr3:8970694-8972020 FORWARDmoderately similar to ( 321) loc_os02g16709 12002.m06913 protein signal peptidase I-1, putative, expressed    no original description 	T
16.5.99.1	secondary metabolism.sulfur-containing.misc.alliinase	MDP0000257862	 moderately similar to ( 493) AT1G34060 | Symbols:  | alliinase family protein | chr1:12396561-12398299 REVERSEmoderately similar to ( 444) loc_os01g52010 12001.m11376 protein alliin lyase precursor, putative, expressed  no original description 	T
26.2	misc.UDP glucosyl and glucoronyl transferases	MDP0000686894	 very weakly similar to (90.1) AT5G05900 | Symbols:  | UDP-glucoronosyl/UDP-glucosyl transferase family protein | chr5:1774513-1776381 FORWARDvery weakly similar to (60.5) loc_os03g60960 12003.m10983 protein cytokinin-N-glucosyltransferase 1, putative, expressed  no original description 	T
35.2	not assigned.unknown	MDP0000123200	 highly similar to ( 659) loc_os10g22520 12010.m05260 protein glucan 1,3-beta-glucosidase precursor, putative, expressed  no original description 	T
29.3.1	protein.targeting.nucleus	MDP0000336843	 moderately similar to ( 221) AT1G27970 | Symbols: NTF2B | NTF2B (NUCLEAR TRANSPORT FACTOR 2B); Ran GTPase binding / protein transporter | chr1:9746921-9748306 FORWARDweakly similar to ( 168) NTF2_ORYSA Nuclear transport factor 2 (NTF-2) - Oryza sativa (Rice)weakly similar to ( 168) loc_os08g42000 12008.m08178 protein nuclear transport factor 2, putative, expressed  no original description 	T
29.5.11.3	protein.degradation.ubiquitin.E2	MDP0000364057	 weakly similar to ( 174) AT3G52560 | Symbols: UEV1D-4 | UEV1D-4 (UBIQUITIN E2 VARIANT 1D-4); protein binding / ubiquitin-protein ligase | chr3:19494678-19495331 REVERSEweakly similar to ( 167) loc_os09g12570 12009.m04519 protein ubiquitin-conjugating enzyme spm2, putative, expressed  no original description 	T
27.3.69	RNA.regulation of transcription.SET-domain transcriptional regulator family	MDP0000854536	 highly similar to ( 728) AT2G23380 | Symbols: CLF, ICU1, SDG1, SET1 | CLF (CURLY LEAF); transcription factor | chr2:9955570-9960117 FORWARDhighly similar to ( 660) EZ1_MAIZE Polycomb protein EZ1 (Enhancer of zeste protein 1) - Zea mays (Maize)highly similar to ( 718) loc_os06g16390 12006.m06352 protein polycomb protein EZ1, putative, expressed  no original description 	T
16.2.1.1	secondary metabolism.phenylpropanoids.lignin biosynthesis.PAL	MDP0000191304	 nearly identical (1113) AT2G37040 | Symbols: pal1, ATPAL1 | pal1 (Phe ammonia lyase 1); phenylalanine ammonia-lyase | chr2:15557602-15560237 REVERSEnearly identical (1144) PAL3_PETCR Phenylalanine ammonia-lyase 3 (EC 4.3.1.5) - Petroselinum crispum (Parsley) (Petroselinum hortense)nearly identical (1023) loc_os04g43800 12004.m09339 protein phenylalanine ammonia-lyase, putative, expressed  no original description 	T
35.1.2	not assigned.no ontology.agenet domain-containing protein	MDP0000126098	 moderately similar to ( 241) AT4G32440 | Symbols:  | agenet domain-containing protein | chr4:15657295-15659354 FORWARDmoderately similar to ( 215) loc_os02g11000 12002.m33358 protein RNA binding protein, putative, expressed  no original description 	T
35.2	not assigned.unknown	MDP0000433647	 weakly similar to ( 102) AT4G09550 | Symbols:  | unknown protein | chr4:6039706-6039921 FORWARDvery weakly similar to (86.3) loc_os03g60900 12003.m10977 protein expressed protein no original description 	T
29.2.1.2.1.5	protein.synthesis.ribosomal protein.eukaryotic.40S subunit.S5	MDP0000184486	 weakly similar to ( 197) AT2G37270 | Symbols: ATRPS5B | ATRPS5B (RIBOSOMAL PROTEIN 5B); structural constituent of ribosome | chr2:15647883-15649042 REVERSEweakly similar to ( 200) RS5_CICAR 40S ribosomal protein S5 (Fragment) - Cicer arietinum (Chickpea) (Garbanzo)weakly similar to ( 192) loc_os11g29190 12011.m080000 protein 40S ribosomal protein S5, putative, expressed no original description 	T
35.2	not assigned.unknown	MDP0000172739	 highly similar to ( 512) AT2G46420 | Symbols:  | unknown protein | chr2:19054891-19056645 FORWARDmoderately similar to ( 417) loc_os01g12080 12001.m42921 protein plant-specific domain TIGR01589 family protein, expressed  no original description 	T
29.5.5	protein.degradation.serine protease	MDP0000719271	 highly similar to ( 531) AT5G53350 | Symbols: CLPX | CLPX; ATP binding / ATPase/ nucleoside-triphosphatase/ nucleotide binding / protein binding | chr5:21644060-21647503 FORWARDmoderately similar to ( 450) loc_os02g35630 12002.m08651 protein CLPX, putative, expressed   no original description 	T
11.1.13	lipid metabolism.FA synthesis and FA elongation.acyl-CoA binding protein	MDP0000266330	 highly similar to ( 891) AT3G05420 | Symbols: ACBP4 | ACBP4 (ACYL-COA BINDING PROTEIN 4); acyl-CoA binding | chr3:1561880-1567047 FORWARDvery weakly similar to (68.9) BSL2_ORYSA Serine/threonine-protein phosphatase BSL2 homolog (EC 3.1.3.16) (BSU1-like protein 2 homolog) - Oryza sativa (Rice)highly similar to ( 749) loc_os03g61930 12003.m35507 protein acyl-CoA binding protein, putative, expressed  no original description 	T
35.2	not assigned.unknown	MDP0000560140	 weakly similar to ( 164) AT2G47360 | Symbols:  | unknown protein | chr2:19437820-19438731 FORWARD    no original description 	T
29.4	protein.postranslational modification	MDP0000222170	 moderately similar to ( 493) AT2G25620 | Symbols:  | protein phosphatase 2C, putative / PP2C, putative | chr2:10903154-10904978 REVERSEmoderately similar to ( 446) loc_os06g39600 12006.m31889 protein protein phosphatase 2C homolog 2, putative, expressed  no original description 	T
35.2	not assigned.unknown	MDP0000312638	   no original description 	T
35.2	not assigned.unknown	MDP0000149039	 moderately similar to ( 266) AT2G46980 | Symbols:  | unknown protein | chr2:19299868-19303429 REVERSEweakly similar to ( 120) loc_os10g26560 12010.m05596 protein expressed protein  no original description 	T
34.8	transport.metabolite transporters at the envelope membrane	MDP0000308027	 moderately similar to ( 491) AT5G33320 | Symbols: CUE1, PPT, ARAPPT | CUE1 (CAB UNDEREXPRESSED 1); antiporter/ triose-phosphate transmembrane transporter | chr5:12588950-12591408 FORWARDmoderately similar to ( 209) TPT_SOLTU Triose phosphate/phosphate translocator, chloroplast precursor (cTPT) (E29) - Solanum tuberosum (Potato)moderately similar to ( 495) loc_os09g12600 12009.m22060 protein triose phosphate/phosphate translocator, non-green plastid,chloroplast precursor, putative, expressed   no original description 	T
26.22	misc.short chain dehydrogenase/reductase (SDR)	MDP0000869980	 moderately similar to ( 394) AT4G03140 | Symbols:  | binding / catalytic/ oxidoreductase | chr4:1392191-1393662 FORWARDmoderately similar to ( 229) TS2_MAIZE Sex determination protein tasselseed-2 - Zea mays (Maize)moderately similar to ( 298) loc_os11g32030 12011.m07105 protein sex determination protein tasselseed-2, putative, expressed no original description 	T
35.1.5	not assigned.no ontology.pentatricopeptide (PPR) repeat-containing protein	MDP0000200406	 moderately similar to ( 285) AT3G16760 | Symbols:  | tetratricopeptide repeat (TPR)-containing protein | chr3:5703213-5705080 FORWARDmoderately similar to ( 219) loc_os03g22140 12003.m07617 protein TPR Domain containing protein, expressed  no original description 	T
33.99	development.unspecified	MDP0000244504	 moderately similar to ( 273) AT5G13170 | Symbols: SAG29 | SAG29 (SENESCENCE-ASSOCIATED PROTEIN 29) | chr5:4181331-4183171 REVERSEmoderately similar to ( 255) loc_os02g30910 12002.m08234 protein MTN3, putative, expressed no original description 	T
35.2	not assigned.unknown	MDP0000633192	 moderately similar to ( 500) AT4G39790 | Symbols:  | unknown protein | chr4:18462316-18464584 REVERSEmoderately similar to ( 474) loc_os05g07980 12005.m05316 protein expressed protein  Sporozoite_P67 MSP1_C  no original description 	T
29.6	protein.folding	MDP0000704217	 moderately similar to ( 338) AT5G20720 | Symbols: CPN20, CPN10, CHCPN10, ATCPN21, CPN21 | CPN20 (CHAPERONIN 20); calmodulin binding | chr5:7015015-7016354 FORWARDmoderately similar to ( 297) CH10C_SPIOL 20 kDa chaperonin, chloroplast precursor (Protein Cpn21) (Chloroplast protein Cpn10) (Chloroplast chaperonin 10) (Ch-CPN10) - Spinacia oleracea (Spinach)moderately similar to ( 308) loc_os06g09679 12006.m31856 protein chaperonin, chloroplast precursor, putative, expressed  no original description 	T
29.3.4.99	protein.targeting.secretory pathway.unspecified	MDP0000670186	 moderately similar to ( 286) AT2G18180 | Symbols:  | SEC14 cytosolic factor, putative / phosphoglyceride transfer protein, putative | chr2:7911054-7913695 REVERSEmoderately similar to ( 277) loc_os10g03400 12010.m03757 protein CRAL/TRIO, N-terminus family protein, expressed no original description 	T
18.3.1	Co-factor and vitamine metabolism.riboflavin.GTP cyclohydrolase II	MDP0000275142	 highly similar to ( 750) AT5G64300 | Symbols: ATGCH, GCH, ATRIBA1, RFD1 | ATGCH; 3,4-dihydroxy-2-butanone-4-phosphate synthase/ GTP cyclohydrolase II | chr5:25718459-25720790 FORWARDhighly similar to ( 686) loc_os02g36340 12002.m33397 protein riboflavin biosynthesis protein ribAB, chloroplast precursor, putative, expressed  no original description 	T
35.2	not assigned.unknown	MDP0000817494	 weakly similar to ( 184) AT1G15760 | Symbols:  | FUNCTIONS IN: molecular_function unknown; INVOLVED IN: biological_process unknown; LOCATED IN: cellular_component unknown; CONTAINS InterPro DOMAIN/s: Sterile alpha motif homology (InterPro:IPR010993); BEST Arabidopsis thaliana protein match is: unknown protein (TAIR:AT1G80520.1); Has 30 Blast hits to 30 proteins in 7 species: Archae - 0; Bacteria - 0; Metazoa - 0; Fungi - 0; Plants - 30; Viruses - 0; Other Eukaryotes - 0 (source: NCBI BLink). | chr1:5425714-5426322 FORWARDweakly similar to ( 110) loc_os04g55120 12004.m10404 protein jp18, putative, expressed   no original description 	T
26.14	misc.oxygenases	MDP0000182483	 moderately similar to ( 459) AT2G17970 | Symbols:  | oxidoreductase, 2OG-Fe(II) oxygenase family protein | chr2:7819116-7820787 REVERSEmoderately similar to ( 388) loc_os06g04660 12006.m091594 protein oxidoreductase, 2OG-Fe oxygenase family protein, expressed   no original description 	T
26.7	misc.oxidases - copper, flavone etc	MDP0000182483	 moderately similar to ( 459) AT2G17970 | Symbols:  | oxidoreductase, 2OG-Fe(II) oxygenase family protein | chr2:7819116-7820787 REVERSEmoderately similar to ( 388) loc_os06g04660 12006.m091594 protein oxidoreductase, 2OG-Fe oxygenase family protein, expressed   no original description 	T
1.1.3.1	PS.lightreaction.cytochrome b6/f.iron sulfur subunit	MDP0000194460	 moderately similar to ( 273) AT4G03280 | Symbols: PETC, PGR1 | PETC (PHOTOSYNTHETIC ELECTRON TRANSFER C); electron transporter, transferring electrons from cytochrome b6/f complex of photosystem II | chr4:1440462-1441717 FORWARDmoderately similar to ( 281) UCRIA_PEA Cytochrome b6-f complex iron-sulfur subunit, chloroplast precursor (EC 1.10.99.1) (Rieske iron-sulfur protein) (Plastohydroquinone:plastocyanin oxidoreductase iron-sulfur protein) (ISP) (RISP) - Pisum sativum (Garden pea)moderately similar to ( 244) loc_os07g37030 12007.m07953 protein cytochrome b6-f complex iron-sulfur subunit, chloroplast precursor, putative, expressed  no original description 	T
35.2	not assigned.unknown	MDP0000125576	 moderately similar to ( 255) AT2G46220 | Symbols:  | unknown protein | chr2:18979655-18980557 FORWARDmoderately similar to ( 236) loc_os01g12200 12001.m07835 protein expressed protein   no original description 	T
35.1.40	not assigned.no ontology.glycine rich proteins	MDP0000302533	 very weakly similar to (99.4) AT3G03773 | Symbols:  | FUNCTIONS IN: molecular_function unknown; INVOLVED IN: biological_process unknown; LOCATED IN: endomembrane system; CONTAINS InterPro DOMAIN/s: CS domain (InterPro:IPR007052), HSP20-like chaperone (InterPro:IPR008978), CS (InterPro:IPR017447); BEST Arabidopsis thaliana protein match is: glycine-rich protein (TAIR:AT4G02450.2). | chr3:951628-953490 FORWARDweakly similar to ( 145) loc_os03g26460 12003.m35251 protein prostaglandin E synthase 3, putative, expressed  no original description 	T
30.5	signalling.G-proteins	MDP0000309310	 highly similar to ( 604) AT3G49350 | Symbols:  | RAB GTPase activator | chr3:18297663-18299846 REVERSEhighly similar to ( 555) loc_os09g35960 12009.m06547 protein GTPase activator protein of Rab-like small GTPases-like protein, putative, expressed   no original description 	T
35.2	not assigned.unknown	MDP0000764531	 highly similar to ( 553) AT3G48770 | Symbols:  | ATP binding / DNA binding | chr3:18079261-18086817 REVERSEhighly similar to ( 516) loc_os11g31480 12011.m07051 protein DNA binding protein, putative, expressed  no original description 	T
29.1.17	protein.aa activation.glutamate-tRNA ligase	MDP0000230305	 highly similar to ( 811) AT5G64050 | Symbols: ATERS, OVA3, ERS | ERS (GLUTAMATE TRNA SYNTHETASE); glutamate-tRNA ligase | chr5:25630196-25633099 REVERSEhighly similar to ( 788) SYE_TOBAC Glutamyl-tRNA synthetase (EC 6.1.1.17) (Glutamate--tRNA ligase) (GluRS) - Nicotiana tabacum (Common tobacco)highly similar to ( 754) loc_os02g02860 12002.m05636 protein glutamyl-tRNA synthetase, putative, expressed no original description 	T
17.3.1.2.6	hormone metabolism.brassinosteroid.synthesis-degradation.sterols.DWF7	MDP0000315887	 weakly similar to ( 118) AT3G02580 | Symbols: STE1, DWF7, BUL1 | STE1 (STEROL 1); C-5 sterol desaturase | chr3:547048-548615 FORWARDweakly similar to ( 117) SC5D_TOBAC Delta(7)-sterol-C5(6)-desaturase (EC 1.3.3.-) (Delta-7-C-5 sterol desaturase) (Delta(7)-sterol-C5-desaturase) - Nicotiana tabacum (Common tobacco)very weakly similar to (89.4) loc_os01g04260 12001.m07061 protein delta-7-sterol-C5, putative, expressed  no original description 	T
28.1.3	DNA.synthesis/chromatin structure.histone	MDP0000823582	 weakly similar to ( 176) AT1G07790 | Symbols: HTB1 | HTB1; DNA binding | chr1:2413049-2413495 FORWARDweakly similar to ( 177) H2B1_MEDTR Probable histone H2B.1 - Medicago truncatula (Barrel medic)weakly similar to ( 171) loc_os01g62230 12001.m12346 protein histone H2B.4, putative, expressed no original description 	T
29.5.1	protein.degradation.subtilases	MDP0000272177	 moderately similar to ( 300) AT4G00230 | Symbols: XSP1 | XSP1 (xylem serine peptidase 1); identical protein binding / serine-type endopeptidase | chr4:93935-97289 FORWARDmoderately similar to ( 238) loc_os09g36110 12009.m06561 protein subtilisin-like protease precursor, putative, expressed  no original description 	T
34.3	transport.amino acids	MDP0000885425	 highly similar to ( 852) AT1G77690 | Symbols: LAX3 | LAX3 (LIKE AUX1 3); amino acid transmembrane transporter/ auxin influx transmembrane transporter/ transporter | chr1:29201232-29203317 REVERSEhighly similar to ( 868) LAX3_MEDTR Auxin transporter-like protein 3 (AUX1-like protein 3) (MtLAX3) - Medicago truncatula (Barrel medic)highly similar to ( 833) loc_os03g14080 12003.m06851 protein auxin transporter-like protein 3, putative, expressed   no original description 	T
34.8	transport.metabolite transporters at the envelope membrane	MDP0000679625	 highly similar to ( 750) AT5G12860 | Symbols: DiT1 | DiT1 (dicarboxylate transporter 1); oxoglutarate:malate antiporter | chr5:4059850-4061919 REVERSEhighly similar to ( 709) SOT1_SPIOL 2-oxoglutarate/malate translocator, chloroplast precursor - Spinacia oleracea (Spinach)highly similar to ( 700) loc_os12g33080 12012.m07106 protein 2-oxoglutarate/malate translocator, chloroplast precursor, putative, expressed  PRK08026  no original description 	T
23.2.1.2	nucleotide metabolism.degradation.pyrimidine.uridine nucleosidase	MDP0000319340	 weakly similar to ( 172) AT1G05620 | Symbols: URH2 | URH2 (URIDINE-RIBOHYDROLASE 2); hydrolase | chr1:1679609-1681527 FORWARDweakly similar to ( 160) loc_os03g31170 12003.m35282 protein pyrimidine-specific ribonucleoside hydrolase rihA, putative, expressed no original description 	T
9.5	mitochondrial electron transport / ATP synthesis.cytochrome c reductase	MDP0000362465	 weakly similar to ( 116) AT3G52730 | Symbols:  | ubiquinol-cytochrome C reductase UQCRX/QCR9-like family protein | chr3:19543146-19544167 REVERSEweakly similar to ( 113) UCR10_SOLTU Ubiquinol-cytochrome c reductase complex 8.0 kDa protein (EC 1.10.2.2) - Solanum tuberosum (Potato)weakly similar to ( 107) loc_os05g33210 12005.m83959 protein ubiquinol-cytochrome c reductase complex 8.0 kDa protein, putative, expressed  no original description 	T
29.5.11.4.6	protein.degradation.ubiquitin.E3.DCX	MDP0000281481	 moderately similar to ( 327) AT4G10180 | Symbols: DET1, FUS2, ATDET1 | DET1 (DE-ETIOLATED 1); catalytic | chr4:6346543-6349146 FORWARDhighly similar to ( 508) loc_os01g01484 12001.m42824 protein light-mediated development protein DET1, putative, expressed   no original description 	T
33.99	development.unspecified	MDP0000281481	 moderately similar to ( 327) AT4G10180 | Symbols: DET1, FUS2, ATDET1 | DET1 (DE-ETIOLATED 1); catalytic | chr4:6346543-6349146 FORWARDhighly similar to ( 508) loc_os01g01484 12001.m42824 protein light-mediated development protein DET1, putative, expressed   no original description 	T
30.11	signalling.light	MDP0000281481	 moderately similar to ( 327) AT4G10180 | Symbols: DET1, FUS2, ATDET1 | DET1 (DE-ETIOLATED 1); catalytic | chr4:6346543-6349146 FORWARDhighly similar to ( 508) loc_os01g01484 12001.m42824 protein light-mediated development protein DET1, putative, expressed   no original description 	T
29.5.3	protein.degradation.cysteine protease	MDP0000218404	 highly similar to ( 559) AT5G60360 | Symbols: AALP | AALP (Arabidopsis aleurain-like protease); cysteine-type peptidase | chr5:24280044-24282157 FORWARDhighly similar to ( 517) ORYC_ORYSA Oryzain gamma chain precursor (EC 3.4.22.-) - Oryza sativa (Rice)highly similar to ( 517) loc_os09g27030 12009.m059986 protein thiol protease aleurain precursor, putative, expressed  no original description 	T
35.1	not assigned.no ontology	MDP0000639101	 weakly similar to ( 134) AT3G06610 | Symbols:  | DNA-binding enhancer protein-related | chr3:2060837-2061845 FORWARDweakly similar to ( 149) loc_os03g61680 12003.m11049 protein huntingtin interacting protein K, putative, expressed no original description 	T
29.4	protein.postranslational modification	MDP0000241912	 highly similar to ( 632) AT4G18950 | Symbols:  | ankyrin protein kinase, putative | chr4:10375685-10378129 FORWARDweakly similar to ( 110) CIPK1_ORYSA CIPK-like protein 1 (EC 2.7.11.1) (OsCK1) - Oryza sativa (Rice)highly similar to ( 560) loc_os01g54480 12001.m150710 protein serine/threonine-protein kinase TNNI3K, putative, expressed  no original description 	T
17.5.1	hormone metabolism.ethylene.synthesis-degradation	MDP0000265117	 highly similar to ( 533) AT1G48420 | Symbols: D-CDES | D-CDES (D-CYSTEINE DESULFHYDRASE); 1-aminocyclopropane-1-carboxylate deaminase/ D-cysteine desulfhydrase/ catalytic | chr1:17896767-17898803 REVERSEhighly similar to ( 536) loc_os02g53330 12002.m100439 protein 1-aminocyclopropane-1-carboxylate deaminase, putative, expressed  no original description 	T
27.3.22	RNA.regulation of transcription.HB,Homeobox transcription factor family	MDP0000311522	 weakly similar to ( 189) AT3G61890 | Symbols: ATHB-12, ATHB12 | ATHB-12 (ARABIDOPSIS THALIANA HOMEOBOX 12); transcription activator/ transcription factor | chr3:22914346-22915239 REVERSEweakly similar to ( 115) loc_os08g13350 12008.m26625 protein cysteine-type endopeptidase/ ubiquitin thiolesterase, putative, expressed  COG3889  no original description 	T
30.2.11	signalling.receptor kinases.leucine rich repeat XI	MDP0000150878	 highly similar to ( 882) AT1G34420 | Symbols:  | leucine-rich repeat family protein / protein kinase family protein | chr1:12584587-12587570 FORWARDmoderately similar to ( 298) PSKR_DAUCA Phytosulfokine receptor precursor (EC 2.7.11.1) (Phytosulfokine LRR receptor kinase) - Daucus carota (Carrot)highly similar to ( 831) loc_os12g08180 12012.m04805 protein receptor-like protein kinase precursor, putative, expressed   no original description 	T
34.9	transport.metabolite transporters at the mitochondrial membrane	MDP0000129248	 highly similar to ( 961) AT2G35800 | Symbols:  | mitochondrial substrate carrier family protein | chr2:15044437-15048352 FORWARDvery weakly similar to (69.3) BT1_MAIZE Protein brittle-1, chloroplast precursor - Zea mays (Maize)highly similar to ( 717) loc_os11g01270 12011.m04332 protein expressed protein   no original description 	T
29.4	protein.postranslational modification	MDP0000294529	 moderately similar to ( 398) AT4G11260 | Symbols: ATSGT1B, ETA3, RPR1, EDM1, SGT1B | SGT1B; protein binding | chr4:6851515-6853719 REVERSEvery weakly similar to (77.0) STIP_SOYBN Heat shock protein STI (Stress-inducible protein) (GmSTI) - Glycine max (Soybean)moderately similar to ( 404) loc_os01g43540 12001.m10616 protein suppressor of G2 allele of SKP1, putative, expressed  no original description 	T
35.1	not assigned.no ontology	MDP0000300206	 very weakly similar to (93.2) AT3G19508 | Symbols:  | unknown protein | chr3:6762378-6762696 REVERSEvery weakly similar to (82.8) loc_os01g24020 12001.m43002 protein expressed protein   no original description 	T
29.4	protein.postranslational modification	MDP0000265328	 highly similar to ( 531) AT1G12580 | Symbols: PEPKR1 | PEPKR1 (Phosphoenolpyruvate carboxylase-related kinase 1); ATP binding / kinase/ protein kinase/ protein serine/threonine kinase | chr1:4283635-4285675 FORWARDmoderately similar to ( 301) CDPK3_ORYSA Calcium-dependent protein kinase, isoform 11 (EC 2.7.11.1) (CDPK 11) - Oryza sativa (Rice)highly similar to ( 502) loc_os06g03682 12006.m05100 protein calcium-dependent protein kinase, isoform AK1, putative, expressed  no original description 	T
10.7	cell wall.modification	MDP0000269483	 moderately similar to ( 475) AT5G65730 | Symbols:  | xyloglucan:xyloglucosyl transferase, putative / xyloglucan endotransglycosylase, putative / endo-xyloglucan transferase, putative | chr5:26299080-26300290 FORWARDmoderately similar to ( 323) XTH_TOBAC Probable xyloglucan endotransglucosylase/hydrolase protein precursor (EC 2.4.1.207) - Nicotiana tabacum (Common tobacco)moderately similar to ( 317) loc_os11g33270 12011.m07226 protein xyloglucan endotransglucosylase/hydrolase precursor, putative, expressedCOG3889  no original description 	T
35.2	not assigned.unknown	MDP0000179622	 moderately similar to ( 305) AT4G32350 | Symbols:  | unknown protein | chr4:15617634-15620642 FORWARDmoderately similar to ( 230) loc_os02g06430 12002.m05991 protein expressed protein  no original description 	T
27.1.1	RNA.processing.splicing	MDP0000271362	 moderately similar to ( 428) AT4G02440 | Symbols: EID1 | EID1 (EMPFINDLICHER IM DUNKELROTEN LICHT 1); ubiquitin-protein ligase | chr4:1072555-1073565 FORWARDmoderately similar to ( 323) loc_os05g30190 12005.m07255 protein phytochrome A-associated F-box protein, putative, expressed   no original description 	T
9.5	mitochondrial electron transport / ATP synthesis.cytochrome c reductase	MDP0000454229	 moderately similar to ( 399) AT5G13430 | Symbols:  | ubiquinol-cytochrome C reductase iron-sulfur subunit, mitochondrial, putative / Rieske iron-sulfur protein, putative | chr5:4305414-4307399 REVERSEmoderately similar to ( 409) UCRI3_TOBAC Ubiquinol-cytochrome c reductase iron-sulfur subunit 3, mitochondrial precursor (EC 1.10.2.2) (Rieske iron-sulfur protein 3) (RISP3) - Nicotiana tabacum (Common tobacco)moderately similar to ( 396) loc_os02g32120 12002.m08305 protein ubiquinol-cytochrome c reductase iron-sulfur subunit, mitochondrial precursor, putative, expressed  no original description 	T
33.99	development.unspecified	MDP0000242528	 highly similar to ( 574) AT1G18080 | Symbols: ATARCA, RACK1A_AT | ATARCA; nucleotide binding | chr1:6222325-6223901 FORWARDhighly similar to ( 592) GBLP_SOYBN Guanine nucleotide-binding protein subunit beta-like protein - Glycine max (Soybean)moderately similar to ( 488) loc_os01g49290 12001.m11115 protein guanine nucleotide-binding protein beta subunit-like protein, putative, expressed   no original description 	T
1.3.9	PS.calvin cycle.seduheptulose bisphosphatase	MDP0000267169	 highly similar to ( 554) AT3G55800 | Symbols: SBPASE | SBPASE (sedoheptulose-bisphosphatase); phosphoric ester hydrolase/ sedoheptulose-bisphosphatase | chr3:20709640-20711421 FORWARDhighly similar to ( 531) S17P_SPIOL Sedoheptulose-1,7-bisphosphatase, chloroplast precursor (EC 3.1.3.37) (Sedoheptulose-bisphosphatase) (SBPase) (SED(1,7)P2ase) - Spinacia oleracea (Spinach)highly similar to ( 527) loc_os04g16680 12004.m06845 protein sedoheptulose-1,7-bisphosphatase, chloroplast precursor, putative, expressed  no original description 	T
29.5.11.4.2	protein.degradation.ubiquitin.E3.RING	MDP0000120368	 weakly similar to ( 102) AT2G35910 | Symbols:  | zinc finger (C3HC4-type RING finger) family protein | chr2:15073225-15073878 REVERSEvery weakly similar to (62.4) EL5_ORYSA E3 ubiquitin-protein ligase EL5 (EC 6.3.2.-) - Oryza sativa (Rice)weakly similar to ( 104) loc_os07g06560 12007.m05116 protein RING-H2 finger protein ATL2I, putative no original description 	T
11.9.3.4	lipid metabolism.lipid degradation.lysophospholipases.phospholipase A2	MDP0000130878	 moderately similar to ( 209) AT2G06925 | Symbols: ATSPLA2-ALPHA, PLA2-ALPHA | PLA2-ALPHA; phospholipase A2 | chr2:2842475-2843212 REVERSEweakly similar to ( 168) loc_os03g50030 12003.m09999 protein phospholipase A2, putative, expressed  no original description 	T
20.1	stress.biotic	MDP0000876794	 moderately similar to ( 410) AT4G21790 | Symbols: TOM1, ATTOM1 | TOM1 (TOBAMOVIRUS MULTIPLICATION 1); protein binding | chr4:11569924-11572163 FORWARDmoderately similar to ( 391) loc_os03g36790 12003.m35615 protein tobamovirus multiplication 1, putative, expressed  no original description 	T
35.2	not assigned.unknown	MDP0000254265	 very weakly similar to (76.6) AT2G03440 | Symbols:  | nodulin-related | chr2:1039409-1039972 REVERSEvery weakly similar to (50.4) CS120_WHEAT Cold-shock protein CS120 - Triticum aestivum (Wheat)very weakly similar to (77.0) loc_os06g12580 12006.m32024 protein pro-resilin precursor, putative, expressed   no original description 	T
17.2.3	hormone metabolism.auxin.induced-regulated-responsive-activated	MDP0000207761	 highly similar to ( 674) AT1G38131 | Symbols:  | unknown protein | chr1:14293392-14296020 REVERSEhighly similar to ( 504) loc_os12g07540 12012.m04743 protein growth regulator protein, putative, expressed  MSP1_C  no original description 	T
27.3.66	RNA.regulation of transcription.Psudo ARR transcription factor family	MDP0000248765	 moderately similar to ( 330) AT5G24470 | Symbols: APRR5, PRR5 | APRR5 (ARABIDOPSIS PSEUDO-RESPONSE REGULATOR 5); transcription regulator/ two-component response regulator | chr5:8356204-8358873 REVERSEmoderately similar to ( 268) PRR95_ORYSA Two-component response regulator-like PRR95 (Pseudo-response regulator 95) (OsPRR95) - Oryza sativa (Rice)moderately similar to ( 268) loc_os09g36220 12009.m06571 protein two-component response regulator-like PRR95, putative, expressed    no original description 	T
27.3.5	RNA.regulation of transcription.ARR	MDP0000248765	 moderately similar to ( 330) AT5G24470 | Symbols: APRR5, PRR5 | APRR5 (ARABIDOPSIS PSEUDO-RESPONSE REGULATOR 5); transcription regulator/ two-component response regulator | chr5:8356204-8358873 REVERSEmoderately similar to ( 268) PRR95_ORYSA Two-component response regulator-like PRR95 (Pseudo-response regulator 95) (OsPRR95) - Oryza sativa (Rice)moderately similar to ( 268) loc_os09g36220 12009.m06571 protein two-component response regulator-like PRR95, putative, expressed    no original description 	T
4.3.12	glycolysis.unclear/dually targeted.phosphoglycerate mutase	MDP0000191423	 moderately similar to ( 457) AT1G22170 | Symbols:  | phosphoglycerate/bisphosphoglycerate mutase family protein | chr1:7826603-7828055 FORWARDmoderately similar to ( 451) loc_os02g51590 12002.m33276 protein 2,3-bisphosphoglycerate-dependent phosphoglycerate mutase, putative, expressed no original description 	T
4.2.14	glycolysis.plastid branch.pyruvate kinase (PK)	MDP0000177897	 weakly similar to ( 179) AT3G22960 | Symbols: PKP1, PKP-ALPHA | PKP-ALPHA; pyruvate kinase | chr3:8139369-8141771 FORWARDweakly similar to ( 174) KPYA_RICCO Pyruvate kinase isozyme A, chloroplast precursor (EC 2.7.1.40) - Ricinus communis (Castor bean)moderately similar to ( 213) loc_os03g46910 12003.m09706 protein pyruvate kinase isozyme A, chloroplast precursor, putative, expressed  no original description 	T
16.99	secondary metabolism.unspecified	MDP0000062070	 highly similar to ( 612) AT5G04620 | Symbols: ATBIOF | ATBIOF (BIOTIN F); 8-amino-7-oxononanoate synthase/ transaminase | chr5:1328004-1329934 FORWARDhighly similar to ( 515) loc_os10g41150 12010.m06879 protein 8-amino-7-oxononanoate synthase, putative, expressed  no original description 	T
30.5	signalling.G-proteins	MDP0000302467	 highly similar to ( 667) AT5G19320 | Symbols: RANGAP2 | RANGAP2 (RAN GTPASE ACTIVATING PROTEIN 2); RAN GTPase activator | chr5:6505310-6506947 REVERSEhighly similar to ( 608) loc_os05g46560 12005.m27945 protein ran GTPase activating protein, putative, expressed no original description 	T
28.1.3	DNA.synthesis/chromatin structure.histone	MDP0000180827	 weakly similar to ( 177) AT5G02560 | Symbols: HTA12 | HTA12; DNA binding | chr5:575437-576456 FORWARDweakly similar to ( 185) H2A2_MEDTR Probable histone H2A.2 - Medicago truncatula (Barrel medic)weakly similar to ( 181) loc_os05g38640 12005.m08043 protein histone H2A, putative, expressed  no original description 	T
30.2.11	signalling.receptor kinases.leucine rich repeat XI	MDP0000158780	 highly similar to ( 991) AT4G20270 | Symbols: BAM3 | BAM3 (BARELY ANY MERISTEM 3); ATP binding / protein binding / protein kinase/ protein serine/threonine kinase | chr4:10949822-10952924 FORWARDmoderately similar to ( 439) RPK1_IPONI Receptor-like protein kinase precursor (EC 2.7.11.1) - Ipomoea nil (Japanese morning glory) (Pharbitis nil)nearly identical (1054) loc_os11g12620 12011.m05454 protein receptor protein kinase CLAVATA1 precursor, putative, expressed  no original description 	T
20.1	stress.biotic	MDP0000259527	 moderately similar to ( 286) AT3G14470 | Symbols:  | disease resistance protein (NBS-LRR class), putative | chr3:4857940-4861104 FORWARDmoderately similar to ( 385) loc_os05g41290 12005.m08304 protein disease resistance RPP13-like protein 1, putative, expressed  no original description 	T
35.1	not assigned.no ontology	MDP0000095488	 moderately similar to ( 472) AT1G06890 | Symbols:  | transporter-related | chr1:2111728-2114038 REVERSEmoderately similar to ( 453) loc_os05g07670 12005.m05285 protein integral membrane protein like, putative, expressed no original description 	T
35.2	not assigned.unknown	MDP0000781314	 highly similar to ( 657) AT2G40550 | Symbols: ETG1 | ETG1 (E2F TARGET GENE 1) | chr2:16934927-16938033 FORWARDhighly similar to ( 589) loc_os01g07260 12001.m07352 protein expressed protein  no original description 	T
30.3	signalling.calcium	MDP0000169320	 highly similar to ( 660) AT1G53210 | Symbols:  | sodium/calcium exchanger family protein / calcium-binding EF hand family protein | chr1:19844790-19847533 FORWARDvery weakly similar to (53.9) CAX2_ORYSA Vacuolar cation/proton exchanger 2 (Ca(2+)/H(+) exchanger 2) (OsCAX2) - Oryza sativa (Rice)highly similar to ( 640) loc_os01g11414 12001.m07759 protein calcium ion binding protein, putative, expressed   no original description 	T
29.5.11.20	protein.degradation.ubiquitin.proteasom	MDP0000665545	 highly similar to ( 748) AT3G05530 | Symbols: RPT5A, ATS6A.2 | RPT5A (REGULATORY PARTICLE TRIPLE-A ATPASE 5A); ATPase/ calmodulin binding | chr3:1603540-1605993 FORWARDhighly similar to ( 748) PRS6A_BRACM 26S protease regulatory subunit 6A homolog (TAT-binding protein homolog 1) (TBP-1) - Brassica campestris (Field mustard)highly similar to ( 739) loc_os06g07630 12006.m05485 protein 26S protease regulatory subunit 6A, putative, expressed no original description 	T
29.5.9	protein.degradation.AAA type	MDP0000665545	 highly similar to ( 748) AT3G05530 | Symbols: RPT5A, ATS6A.2 | RPT5A (REGULATORY PARTICLE TRIPLE-A ATPASE 5A); ATPase/ calmodulin binding | chr3:1603540-1605993 FORWARDhighly similar to ( 748) PRS6A_BRACM 26S protease regulatory subunit 6A homolog (TAT-binding protein homolog 1) (TBP-1) - Brassica campestris (Field mustard)highly similar to ( 739) loc_os06g07630 12006.m05485 protein 26S protease regulatory subunit 6A, putative, expressed no original description 	T
29.6	protein.folding	MDP0000136536	 moderately similar to ( 375) AT5G61670 | Symbols:  | Encodes a close homolog of the Cauliflower OR (Orange) protein.  The function of OR is to induce the differentiation of proplastids or other noncolored plastids into chromoplasts for carotenoid accumulation.  Both proteins contain a Cysteine-rich zinc finger domain that is highly specific to DnaJ-like molecular chaperons. | chr5:24783629-24785201 FORWARDmoderately similar to ( 358) loc_os02g43500 12002.m33801 protein expressed protein no original description 	T
29.4	protein.postranslational modification	MDP0000809424	 moderately similar to ( 439) AT3G59380 | Symbols: FTA, PLP, ATFTA, PFT/PGGT-IALPHA | FTA (FARNESYLTRANSFERASE A); farnesyltranstransferase/ protein heterodimerization/ protein prenyltransferase | chr3:21944209-21945781 FORWARDmoderately similar to ( 452) PFTA_PEA Protein farnesyltransferase/geranylgeranyltransferase type I alpha subunit (EC 2.5.1.58) (EC 2.5.1.59) (CAAX farnesyltransferase alpha subunit) (Ras proteins prenyltransferase alpha) (FTase-alpha) (Type I protein geranyl-geranyltransferasmoderately similar to ( 421) loc_os09g33930 12009.m060040 protein protein farnesyltransferase/geranylgeranyltransferase type I alphasubunit, putative, expressed   no original description 	T
30.5	signalling.G-proteins	MDP0000306963	 moderately similar to ( 363) AT1G09630 | Symbols: ATRAB11C, ATRABA2A, ATRAB-A2A, RAB-A2A | ATRAB11C (ARABIDOPSIS RAB GTPASE 11C); GTP binding | chr1:3118350-3119571 REVERSEmoderately similar to ( 314) RB2BV_BETVU Ras-related protein Rab2BV - Beta vulgaris (Sugar beet)moderately similar to ( 317) loc_os05g01480 12005.m04686 protein ras-related protein Rab11C, putative, expressed no original description 	T
29.5.11.1	protein.degradation.ubiquitin.ubiquitin	MDP0000220163	 moderately similar to ( 240) AT3G52590 | Symbols: UBQ1, EMB2167, ERD16, HAP4 | UBQ1 (UBIQUITIN EXTENSION PROTEIN 1); protein binding / structural constituent of ribosome | chr3:19505668-19506681 FORWARDweakly similar to ( 150) UBIQ_WHEAT Ubiquitin - Triticum aestivum (Wheat)moderately similar to ( 240) loc_os09g39500 12009.m60242 protein ubiquitin fusion protein, putative, expressed no original description 	T
35.1	not assigned.no ontology	MDP0000320189	 moderately similar to ( 252) AT5G51110 | Symbols:  | 4-alpha-hydroxytetrahydrobiopterin dehydratase | chr5:20778316-20779380 REVERSEweakly similar to ( 187) loc_os03g01030 12003.m05654 protein expressed protein  no original description 	T
11.1.6	lipid metabolism.FA synthesis and FA elongation.enoyl ACP reductase	MDP0000207724	 highly similar to ( 513) AT2G05990 | Symbols: MOD1, ENR1 | MOD1 (MOSAIC DEATH 1); enoyl-[acyl-carrier-protein] reductase (NADH)/ enoyl-[acyl-carrier-protein] reductase/ oxidoreductase | chr2:2322876-2324867 FORWARDhighly similar to ( 501) FABI_BRANA Enoyl-[acyl-carrier-protein] reductase [NADH], chloroplast precursor (EC 1.3.1.9) (NADH-dependent enoyl-ACP reductase) - Brassica napus (Rape)moderately similar to ( 466) loc_os08g23810 12008.m26662 protein enoyl-[acyl-carrier-protein] reductase [NADH], chloroplast precursor, expressed  no original description 	T
31.1	cell.organisation	MDP0000227298	 weakly similar to ( 147) AT3G18670 | Symbols:  | ankyrin repeat family protein | chr3:6424135-6426471 REVERSE   no original description 	T
35.2	not assigned.unknown	MDP0000271854	 nearly identical (1040) AT3G57940 | Symbols:  | unknown protein | chr3:21449560-21455834 FORWARDhighly similar to ( 537) loc_os12g07300 12012.m073778 protein expressed protein  no original description 	T
27.3.99	RNA.regulation of transcription.unclassified	MDP0000296585	 moderately similar to ( 476) AT4G38160 | Symbols: pde191 | pde191 (pigment defective 191) | chr4:17902412-17903784 FORWARDmoderately similar to ( 414) loc_os08g40430 12008.m08022 protein PDE191, putative, expressed  no original description 	T
13.2.4.4	amino acid metabolism.degradation.branched chain group.leucine	MDP0000414288	 highly similar to ( 602) AT2G26800 | Symbols:  | hydroxymethylglutaryl-CoA lyase, putative / 3-hydroxy-3-methylglutarate-CoA lyase, putative / HMG-CoA lyase, putative | chr2:11429192-11432290 REVERSEmoderately similar to ( 475) loc_os11g04210 12011.m04617 protein hydroxymethylglutaryl-CoA lyase, mitochondrial precursor, putative, expressed no original description 	T
29.3.2	protein.targeting.mitochondria	MDP0000523206	 weakly similar to ( 108) AT1G61570 | Symbols: TIM13 | TIM13 (TRANSLOCASE OF THE INNER MITOCHONDRIAL MEMBRANE 13); P-P-bond-hydrolysis-driven protein transmembrane transporter | chr1:22718897-22719473 REVERSEweakly similar to ( 113) TIM13_ORYSA Mitochondrial import inner membrane translocase subunit Tim13 - Oryza sativa (Rice)weakly similar to ( 113) loc_os02g45820 12002.m100802 protein mitochondrial import inner membrane translocase subunit Tim13, putative, expressed  no original description 	T
35.1.5	not assigned.no ontology.pentatricopeptide (PPR) repeat-containing protein	MDP0000292200	 highly similar to ( 739) AT3G01610 | Symbols: CDC48C, emb1354 | CDC48C; ATP binding / ATPase/ nucleoside-triphosphatase/ nucleotide binding | chr3:231787-235057 FORWARDmoderately similar to ( 441) CDC48_SOYBN Cell division cycle protein 48 homolog (Valosin-containing protein homolog) (VCP) - Glycine max (Soybean)highly similar to ( 793) loc_os06g01980 12006.m04934 protein cell division control protein 48 homolog C, putative, expressed    no original description 	T
27.4	RNA.RNA binding	MDP0000605318	 moderately similar to ( 292) AT5G15270 | Symbols:  | KH domain-containing protein | chr5:4958738-4960947 FORWARDmoderately similar to ( 272) loc_os03g25970 12003.m34871 protein nucleic acid binding protein, putative, expressed no original description 	T
1.3.5	PS.calvin cycle.TPI	MDP0000152242	 moderately similar to ( 396) AT3G55440 | Symbols: ATCTIMC, TPI | TPI (TRIOSEPHOSPHATE ISOMERASE); triose-phosphate isomerase | chr3:20553794-20556078 FORWARDmoderately similar to ( 411) TPIS_PETHY Triosephosphate isomerase, cytosolic (EC 5.3.1.1) (TIM) (Triose-phosphate isomerase) - Petunia hybrida (Petunia)moderately similar to ( 399) loc_os01g05490 12001.m42862 protein triosephosphate isomerase, cytosolic, putative, expressed no original description 	T
4.1.7	glycolysis.cytosolic branch.triosephosphate isomerase (TPI)	MDP0000152242	 moderately similar to ( 396) AT3G55440 | Symbols: ATCTIMC, TPI | TPI (TRIOSEPHOSPHATE ISOMERASE); triose-phosphate isomerase | chr3:20553794-20556078 FORWARDmoderately similar to ( 411) TPIS_PETHY Triosephosphate isomerase, cytosolic (EC 5.3.1.1) (TIM) (Triose-phosphate isomerase) - Petunia hybrida (Petunia)moderately similar to ( 399) loc_os01g05490 12001.m42862 protein triosephosphate isomerase, cytosolic, putative, expressed no original description 	T
29.5.1	protein.degradation.subtilases	MDP0000270440	 weakly similar to ( 181) AT4G15040 | Symbols:  | identical protein binding / serine-type endopeptidase | chr4:8581373-8584122 REVERSEweakly similar to ( 125) loc_os02g53860 12002.m10415 protein subtilisin-like protease precursor, putative, expressed no original description 	T
35.2	not assigned.unknown	MDP0000292934	 nearly identical (1019) AT3G56040 | Symbols: UGP3 | UGP3 (UDP-GLUCOSE PYROPHOSPHORYLASE 3) | chr3:20792689-20797482 REVERSEhighly similar to ( 855) loc_os05g39230 12005.m08102 protein low photochemical bleaching 1 protein, putative, expressed  MSP1_C  no original description 	T
26.3.2	misc.gluco-, galacto- and mannosidases.beta-galactosidase	MDP0000899966	 nearly identical (1277) AT4G36360 | Symbols: BGAL3 | BGAL3 (beta-galactosidase 3); beta-galactosidase/ catalytic/ cation binding / sugar binding | chr4:17176840-17181143 REVERSEnearly identical (1102) BGAL_ASPOF Beta-galactosidase precursor (EC 3.2.1.23) (Lactase) - Asparagus officinalis (Garden asparagus)nearly identical (1256) loc_os03g06940 12003.m35030 protein beta-galactosidase precursor, putative, expressed  no original description 	T
16.7	secondary metabolism.wax	MDP0000477496	 moderately similar to ( 323) AT2G37700 | Symbols:  | catalytic/ iron ion binding / oxidoreductase | chr2:15811641-15814176 REVERSEmoderately similar to ( 390) loc_os10g33250 12010.m21915 protein CER1, putative, expressed   no original description 	T
15.2	metal handling.binding, chelation and storage	MDP0000308193	 weakly similar to ( 115) AT3G56891 | Symbols:  | metal ion binding | chr3:21064199-21064922 REVERSEweakly similar to ( 108) loc_os03g02860 12003.m05825 protein metal ion binding protein, putative, expressed   no original description 	T
20.2.1	stress.abiotic.heat	MDP0000481314	 highly similar to ( 625) AT2G47440 | Symbols:  | DNAJ heat shock N-terminal domain-containing protein | chr2:19469912-19471660 FORWARDmoderately similar to ( 458) loc_os03g19200 12003.m07337 protein heat shock protein binding protein, putative, expressed   no original description 	T
29.2.1.2.2.57	protein.synthesis.ribosomal protein.eukaryotic.60S subunit.L7A	MDP0000300095	 moderately similar to ( 381) AT3G62870 | Symbols:  | 60S ribosomal protein L7A (RPL7aB) | chr3:23242862-23244273 REVERSEmoderately similar to ( 390) RL7A_ORYSA 60S ribosomal protein L7a - Oryza sativa (Rice)moderately similar to ( 390) loc_os09g32976 12009.m22160 protein 60S ribosomal protein L7a, putative, expressed  no original description 	T
16.1.1.10	secondary metabolism.isoprenoids.non-mevalonate pathway.geranylgeranyl pyrophosphate synthase	MDP0000279433	 highly similar to ( 552) AT1G17050 | Symbols: SPS2 | SPS2 (Solanesyl diphosphate synthase 2); trans-octaprenyltranstransferase | chr1:5829289-5831215 FORWARDweakly similar to ( 142) GGPPS_HEVBR Geranylgeranyl pyrophosphate synthetase, chloroplast precursor (GGPP synthetase) [Includes: Dimethylallyltranstransferase (EC 2.5.1.1); Geranyltranstransferase (EC 2.5.1.10); Farnesyltranstransferase (EC 2.5.1.29)] - Hevea brasiliensishighly similar to ( 509) loc_os05g50550 12005.m09124 protein prenyl transferase, putative, expressed  no original description 	T
26.9	misc.glutathione S transferases	MDP0000197722	 moderately similar to ( 353) AT1G09640 | Symbols:  | elongation factor 1B-gamma, putative / eEF-1B gamma, putative | chr1:3120162-3122152 FORWARDmoderately similar to ( 401) EF1G2_ORYSA Elongation factor 1-gamma 2 (EF-1-gamma 2) (eEF-1B gamma 2) - Oryza sativa (Rice)moderately similar to ( 401) loc_os02g12794 12002.m100462 protein elongation factor 1-gamma 2, putative, expressed no original description 	T
31.4	cell.vesicle transport	MDP0000258298	 nearly identical (2014) AT1G62020 | Symbols:  | coatomer protein complex, subunit alpha, putative | chr1:22919814-22923728 FORWARDweakly similar to ( 102) PF20_CHLRE Flagellar WD repeat protein PF20 - Chlamydomonas reinhardtiinearly identical (1933) loc_os09g04110 12009.m22040 protein coatomer subunit alpha, putative, expressed  no original description 	T
16.5.99.1	secondary metabolism.sulfur-containing.misc.alliinase	MDP0000290615	 very weakly similar to (76.6) AT1G34060 | Symbols:  | alliinase family protein | chr1:12396561-12398299 REVERSEvery weakly similar to (64.7) loc_os01g51980 12001.m11373 protein alliin lyase precursor, putative, expressed no original description 	T
16.1.4.5	secondary metabolism.isoprenoids.carotenoids.lycopene beta cyclase	MDP0000258205	 weakly similar to ( 196) AT3G10230 | Symbols: LYC | LYC (LYCOPENE CYCLASE); lycopene beta cyclase | chr3:3164340-3165449 REVERSEmoderately similar to ( 285) CCS_CITSI Capsanthin/capsorubin synthase, chloroplast precursor (EC 5.3.99.8) - Citrus sinensis (Sweet orange)weakly similar to ( 182) loc_os02g09750 12002.m06272 protein lycopene beta cyclase, chloroplast precursor, putative, expressed   no original description 	T
30.2.99	signalling.receptor kinases.misc	MDP0000294423	 weakly similar to ( 152) AT4G21410 | Symbols:  | protein kinase family protein | chr4:11402463-11405025 REVERSEweakly similar to ( 148) loc_os05g41370 12005.m08312 protein receptor-like protein kinase homolog RK20-1, putative, expressed no original description 	T
9.1.2	mitochondrial electron transport / ATP synthesis.NADH-DH.localisation not clear	MDP0000330039	 moderately similar to ( 218) AT5G67590 | Symbols: FRO1 | FRO1 (FROSTBITE1); NADH dehydrogenase (ubiquinone) | chr5:26958073-26959356 FORWARDmoderately similar to ( 216) loc_os07g39710 12007.m08213 protein NADH-ubiquinone oxidoreductase 18 kDa subunit, mitochondrial precursor, putative, expressed  no original description 	T
29.5.1	protein.degradation.subtilases	MDP0000231217	 moderately similar to ( 434) AT1G62340 | Symbols: ALE1, ALE | ALE1 (ABNORMAL LEAF-SHAPE 1); serine-type endopeptidase | chr1:23051123-23055656 REVERSEmoderately similar to ( 421) loc_os06g48650 12006.m091776 protein SLP3, putative, expressed   no original description 	T
10.8.1	cell wall.pectin*esterases.PME	MDP0000267128	 highly similar to ( 665) AT2G26440 | Symbols:  | pectinesterase family protein | chr2:11247407-11249407 FORWARDmoderately similar to ( 392) PME_PRUPE Pectinesterase PPE8B precursor (EC 3.1.1.11) (Pectin methylesterase) (PE) - Prunus persica (Peach)moderately similar to ( 408) loc_os06g09340 12006.m05653 protein pectinesterase U1 precursor, putative, expressed  no original description 	T
26.8	misc.nitrilases, *nitrile lyases, berberine bridge enzymes, reticuline oxidases, troponine reductases	MDP0000210966	 moderately similar to ( 328) AT1G12570 | Symbols:  | glucose-methanol-choline (GMC) oxidoreductase family protein | chr1:4278192-4280753 REVERSEmoderately similar to ( 277) loc_os02g45530 12002.m09590 protein protein HOTHEAD precursor, putative, expressed no original description 	T
26.10	misc.cytochrome P450	MDP0000313816	 highly similar to ( 680) AT5G05690 | Symbols: CPD, CYP90A, CYP90, CBB3, DWF3, CYP90A1 | CPD (CONSTITUTIVE PHOTOMORPHOGENIC DWARF); electron carrier/ heme binding / iron ion binding / monooxygenase/ oxygen binding | chr5:1702907-1706348 REVERSEmoderately similar to ( 328) C90D2_ORYSA Cytochrome P450 90D2 (EC 1.14.-.-) (C6-oxidase) - Oryza sativa (Rice)highly similar to ( 514) loc_os11g04710 12011.m04669 protein cytochrome P450 724B1, putative, expressed   no original description 	T
35.2	not assigned.unknown	MDP0000878878	   no original description 	T
34.14	transport.unspecified cations	MDP0000231700	 highly similar to ( 672) AT3G05030 | Symbols: NHX2, ATNHX2 | NHX2 (SODIUM HYDROGEN EXCHANGER 2); sodium ion transmembrane transporter/ sodium:hydrogen antiporter | chr3:1393456-1395753 REVERSEhighly similar to ( 669) loc_os07g47100 12007.m29310 protein sodium/hydrogen exchanger 2, putative, expressed no original description 	T
35.1	not assigned.no ontology	MDP0000319426	 moderately similar to ( 271) AT3G10260 | Symbols:  | reticulon family protein | chr3:3171413-3172568 REVERSEmoderately similar to ( 259) loc_os03g56900 12003.m101524 protein seed maturation protein, putative, expressed   no original description 	T
17.5.1.1002	hormone metabolism.ethylene.synthesis-degradation	(s)-trans-2-amino-4-(2-aminoethoxy)-3-butenoate	ethylene biosynthesis inhibitor	M
17.5.1.1001	hormone metabolism.ethylene.synthesis-degradation	1-aminocyclopropanecarboxylate	ethylene biosynthesis precursor	M
16.99.1011	secondary metabolism.unspecified	1-caffeoylquinate	secondary metabolism	M
11.5.1001	lipid metabolism.glyceral metabolism	1-monohexadecanoglycerol		M
11.5.1002	lipid metabolism.glyceral metabolism	1-monooctodecanoglycerol		M
16.99.1008	secondary metabolism.unspecified	1-o-benzyl-beta-glucoside	secondary metabolism	M
3.99.1001	minor CHO metabolism.misc	1-o-methyl-alpha-galactoside	minor CHO metabolism.misc	M
3.99.1002	minor CHO metabolism.misc	1-o-methyl-beta-galactoside	minor CHO metabolism.misc	M
10.6.1001	cell wall.degradation	1,6-anhydroglucose	very tentative	M
11.1040	lipid metabolism	13-hydroperoxylinolenic acid		M
11.1041	lipid metabolism	13(s)-hydroperoxy-9-(z),11(e)-octadecadienoic acid		M
13.2.3.5.1002	amino acid metabolism.degradation.aspartate family.lysine	2-aminoadipic acid	amino acid degradation.lysine	M
16.99.1007	secondary metabolism.unspecified	2-aminobenzoate	secondary metabolism	M
35.1.1001	not assigned.no ontology	2-aminobutyrate	GABA analog	M
16.2.99.1021	secondary metabolism.phenylpropanoids.unspecified	2-coumarate	secondary metabolism, phenylpropanoids (ligning biosynthesis)	M
3.99.1003	minor CHO metabolism.misc	2-deoxyglucose	minor CHO metabolism.misc	M
11.1039	lipid metabolism	2-ethylhexanoic acid		M
16.2.99.1019	secondary metabolism.phenylpropanoids.unspecified	2-hydroxycinnamate	secondary metabolism, phenylpropanoids	M
16.2.99.1020	secondary metabolism.phenylpropanoids.unspecified	2-methoxycinnamate	secondary metabolism, phenylpropanoids	M
8.2.1001	TCA / org transformation. Various	2-methylcitrate	Propanoate metabolism in bacteria	M
11.10.1001	lipid metabolism.galactolipid synthesis	2-o-glycerol-beta-d-galactose		M
8.2.1002	TCA / org transformation. Various	2-oxogluconate		M
8.1.1004	TCA / org transformation.TCA	2-oxoglutarate	gluconeogenese/ glyoxylate cycle; TCA / org transformation; N-metabolism.ammonia metabolism; amino acid metabolism	M
16.2.99.1002	secondary metabolism.phenylpropanoids.unspecified	2,5-dimethoxycinnamate	secondary metabolism, phenylpropanoids	M
11.1036	lipid metabolism	2(e)-hexenal	volatile	M
17.3.1.1001	hormone metabolism.brassinosteroid.synthesis-degradation	22(s),23(s)-homobrassinolide	hormone metabolism.brassinosteroid	M
17.3.1.1002	hormone metabolism.brassinosteroid.synthesis-degradation	24-epibrassinolide	hormone metabolism.brassinosteroid	M
17.3.1.1003	hormone metabolism.brassinosteroid.synthesis-degradation	24-epicastasterone	hormone metabolism.brassinosteroid	M
17.3.1.1004	hormone metabolism.brassinosteroid.synthesis-degradation	28-homocastasterone√ç¬†	hormone metabolism.brassinosteroid	M
16.99.1010	secondary metabolism.unspecified	3-(2,4-dihydoxyphenyl)propionate	secondary metabolism	M
35.1.1002	not assigned.no ontology	3-aminobutyrate	GABA analog	M
16.2.99.1051	secondary metabolism.phenylpropanoids.unspecified	3-caffeoylquinate		M
16.2.99.1001	secondary metabolism.phenylpropanoids.unspecified	3-dehydroquinate	secondary metabolism, phenylpropanoids	M
3.99.1004	minor CHO metabolism.misc	3-deoxyglucose	minor CHO metabolism.misc	M
16.2.99.1013	secondary metabolism.phenylpropanoids.unspecified	3-hydroxycinnamate	secondary metabolism, phenylpropanoids	M
16.2.99.1014	secondary metabolism.phenylpropanoids.unspecified	3-methoxycinnamate	secondary metabolism, phenylpropanoids	M
3.99.1005	minor CHO metabolism.misc	3-o-methylglucose	minor CHO metabolism.misc	M
16.99.1006	secondary metabolism.unspecified	3-phenylpropionate	secondary metabolism	M
35.1.1005	not assigned.no ontology	3-pyrroline	KnownCompoundUnknownFunction	M
16.20.1006	secondary metabolism.amino acid derivatives	3,4-dihydroxybenzylamine	Dopamine derivative	M
16.2.99.1015	secondary metabolism.phenylpropanoids.unspecified	3,4-dihydroxyhydrocinnamate	secondary metabolism, phenylpropanoids	M
16.2.99.1016	secondary metabolism.phenylpropanoids.unspecified	3,4-dimethoxycinnamate	secondary metabolism, phenylpropanoids	M
16.2.99.1018	secondary metabolism.phenylpropanoids.unspecified	3,4,5-trimethoxycinnamate	secondary metabolism, phenylpropanoids	M
16.2.99.1017	secondary metabolism.phenylpropanoids.unspecified	3,5-dimethoxycinnamate	secondary metabolism, phenylpropanoids	M
11.1030	lipid metabolism	3(e)-hexenal		M
11.1038	lipid metabolism	3(e)-hexenol	volatile	M
11.1037	lipid metabolism	3(z)-hexen-1-ol	volatile	M
11.1031	lipid metabolism	3(z)-hexenal		M
16.99.1004	secondary metabolism.unspecified	4-(methylamino)benzoate	secondary metabolism	M
16.99.1005	secondary metabolism.unspecified	4-aminobenzoate	secondary metabolism	M
16.2.99.1012	secondary metabolism.phenylpropanoids.unspecified	4-aminocinnamate	secondary metabolism, phenylpropanoids	M
16.2.99.1011	secondary metabolism.phenylpropanoids.unspecified	4-caffeoylquinate	secondary metabolism, phenylpropanoids	M
16.2.1.1002	secondary metabolism.phenylpropanoids.lignin biosynthesis	4-coumarate	secondary metabolism, phenylpropanoids	M
16.2.1.1004	secondary metabolism.phenylpropanoids.lignin biosynthesis	4-coumaryl alcohol	secondary metabolism, phenylpropanoids	M
16.2.1.1003	secondary metabolism.phenylpropanoids.lignin biosynthesis	4-coumaryl aldehyde	secondary metabolism, phenylpropanoids	M
16.2.99.1009	secondary metabolism.phenylpropanoids.unspecified	4-hydroxy-3-methoxycinnamaldehyde	secondary metabolism, phenylpropanoids	M
16.2.99.1008	secondary metabolism.phenylpropanoids.unspecified	4-methoxycinnamate	secondary metabolism, phenylpropanoids	M
11.8.1012	lipid metabolism.'exotics' (steroids, squalene etc)	5-alpha-cholestane	secondary metabolism, steroids	M
16.2.99.1007	secondary metabolism.phenylpropanoids.unspecified	5-caffeoylquinate	secondary metabolism, phenylpropanoids	M
17.4.1.1001	hormone metabolism.cytokinin.synthesis-degradation	6-benzylaminopurine	hormone metabolism.cytokinin	M
17.4.1.1002	hormone metabolism.cytokinin.synthesis-degradation	6-benzylaminopurine-9-(beta-glucoside)	hormone metabolism.cytokinin	M
11.2.1001	lipid metabolism.FA desaturation	9,12-octadecadieneoic acid		M
17.1.1.1002	hormone metabolism.abscisic acid.synthesis-degradation	aba	hormone metabolism.abscisic acid	M
16.2.99.1039	secondary metabolism.phenylpropanoids.unspecified	acetophenone		M
11.1025	lipid metabolism	acetyl-coa	lipid metabolism, organic acids	M
11.1001	lipid metabolism	acetyloleanolic acid	lipid metabolism	M
16.99.1003	secondary metabolism.unspecified	acetylsalicylate	secondary metabolism	M
8.1.1002	TCA / org transformation.TCA	aconitate	gluconeogenese/ glyoxylate cycle; TCA / org transformation	M
16.99.1016	secondary metabolism.unspecified	acrylamide	Styrene degradation (to confirm in plants)	M
23.1.1.1001	nucleotide metabolism.synthesis.pyrimidines	adenine		M
23.1.1.1002	nucleotide metabolism.synthesis.pyrimidines	adenosine		M
13.1.5.3.1002	amino acid metabolism.synthesis.serine-glycine-cysteine group.cysteine	adenosine 5'-phosphosulfate	amino acid synthesis.cysteine; S-assimilation	M
23.1.1.1004	nucleotide metabolism.synthesis.pyrimidines	adp		M
2.1.2.1004	major CHO metabolism.synthesis.starch	adp-glucose	major CHO metabolism.synthesis.starch	M
22.1002	polyamine metabolism	agmatine	polyamine metabolism	M
13.1.1.3.10001	amino acid synthesis.alanine	alanine	amino acid synthesis.alanine; amino acid degradation.alanine; amino acid degradation.tryptophan; Co-factor metabolism; fermentation	M
5.1002	fermentation	alanine	amino acid synthesis.alanine; amino acid degradation.alanine; amino acid degradation.tryptophan; Co-factor metabolism; fermentation	M
23.2.2.1007	nucleotide metabolism.degradation.purine	allantoin	Co-factor metabolism; nucleotide metabolism.degradation; purine metabolism	M
16.1.4.1004	secondary metabolism.isoprenoids.carotenoids.alpha carotene	alpha carotene		M
16.1.4.1005	secondary metabolism.isoprenoids.carotenoids.beta carotene	alpha carotene		M
16.2.99.1038	secondary metabolism.phenylpropanoids.unspecified	alpha-hydroxyphenylpropionate		M
16.1.2.1012	secondary metabolism.isoprenoids.mevalonate pathway	alpha-pinene		M
16.1.3.1003	secondary metabolism.isoprenoids.tocopherol biosynthesis	alpha-tocopherol	secondary metabolism, tocopherols	M
12.2.1001	N-metabolism.ammonia metabolism	ammonium	N-metabolism.ammonia metabolism	M
23.1.1.1003	nucleotide metabolism.synthesis.pyrimidines	amp		M
10.2.1006	cell wall degradation	arabinose	cell wall degradation; minor CHO metabolism	M
13.1.2.3.1002	amino acid metabolism.synthesis.glutamate family.arginine	arginine	amino acid synthesis.arginine; amino acid degradation.arginine; amino acid degradation.glutamate; polyamine metabolism	M
20.1001	stress	ascorbate	hormone metabolism.ethylene; stress; redox regulation	M
21.1008	redox	ascorbate	hormone metabolism.ethylene; stress; redox regulation	M
13.1.3.1.1001	amino acid metabolism.synthesis.aspartate family.asparagine	asparagine	amino acid synthesis.asparagines; amino acid degradation.asparagines; amino acid synthesis.aspartate; amino acid degradation.aspartate	M
13.1.1.2.1001	amino acid metabolism.synthesis.central amino acid metabolism.aspartate	aspartate	amino acid synthesis.homoserine; amino acid synthesis.lysine; amino acid synthesis.arginine; amino acid degradation.arginine; amino acid synthesis.asparagines; amino acid degradation.asparagines; amino acid synthesis.aspartate; amino acid degradation.aspartate; amino acid synthesis.glutamate; amino acid degradation.glutamate; Co-factor metabolism; nucleotide metabolism.synthesis	M
23.1.1.1005	nucleotide metabolism.synthesis.pyrimidines	atp		M
16.2.99.1040	secondary metabolism.phenylpropanoids.unspecified	benzaldehyde		M
16.2.99.1022	secondary metabolism.phenylpropanoids.unspecified	benzoate	secondary metabolism, phenylpropanoids (ligning biosynthesis)	M
16.2.99.1045	secondary metabolism.phenylpropanoids.unspecified	benzoylformic acid		M
16.2.99.1041	secondary metabolism.phenylpropanoids.unspecified	benzylalcohol		M
23.2.2.1002	nucleotide metabolism.degradation.purine	beta-alanine	Co-factor metabolism; nucleotide metabolism.degradation; aspartate degradation	M
16.1.1.1012	secondary metabolism.isoprenoids.non-mevalonate pathway	beta-damascenone		M
3.99.1006	minor CHO metabolism.misc	beta-gentiobiose	minor CHO metabolism.misc	M
16.2.99.1046	secondary metabolism.phenylpropanoids.unspecified	beta-hydroxyphenylpropionate		M
17.2.1.1001	hormone metabolism.auxin.synthesis-degradation	beta-indole-3-acetonitrile	hormone metabolism.auxin	M
16.1.1.1011	secondary metabolism.isoprenoids.non-mevalonate pathway	beta-ionone		M
16.2.99.1047	secondary metabolism.phenylpropanoids.unspecified	beta-oxophenylpropionate		M
16.1.2.1014	secondary metabolism.isoprenoids.mevalonate pathway	beta-pinene		M
16.1.5.1002	secondary metabolism.isoprenoids.terpenoids	beta-sitosterol	secondary metabolism, Phytosterol, HEALTH: reduces blood cholesterol, ameliorates prostate problems	M
16.1.3.1004	secondary metabolism.isoprenoids.tocopherol biosynthesis	beta-tocopherol	secondary metabolism, tocopherols	M
20.2.1001	stress.abiotic	betaine		M
17.3.1.1005	hormone metabolism.brassinosteroid.synthesis-degradation	brassinolide	hormone metabolism.brassinosteroid	M
16.99.1012	secondary metabolism.unspecified	buthylhydroxytoluene	secondary metabolism	M
16.2.99.1023	secondary metabolism.phenylpropanoids.unspecified	caffeate	secondary metabolism, phenylpropanoids (lignin biosynthesis)	M
16.4.1.1001	secondary metabolism.N misc.alkaloid-like	calystegine a3	secondary metabolism, alkaloids	M
16.4.1.1002	secondary metabolism.N misc.alkaloid-like	calystegine b1	secondary metabolism, alkaloids	M
16.4.1.1003	secondary metabolism.N misc.alkaloid-like	calystegine b2	secondary metabolism, alkaloids	M
16.4.1.1004	secondary metabolism.N misc.alkaloid-like	calystegine b3	secondary metabolism, alkaloids	M
16.4.1.1005	secondary metabolism.N misc.alkaloid-like	calystegine b4	secondary metabolism, alkaloids	M
16.4.1.1006	secondary metabolism.N misc.alkaloid-like	calystegine c1	secondary metabolism, alkaloids	M
30.1001	signaling	camp	Second messenger signaling pathway; MAPK signaling pathway; purine metabolism; cell cycle	M
16.1.5.1003	secondary metabolism.isoprenoids.terpenoids	campesterol	secondary metabolism, Phytosterol (brassinosteroids synthesis)	M
17.3.1.1006	hormone metabolism.brassinosteroid.synthesis-degradation	castasterone	hormone metabolism.brassinosteroid	M
23.1.1.1008	nucleotide metabolism.synthesis.pyrimidines	cdp		M
16.2.99.1032	secondary metabolism.phenylpropanoids.unspecified	chavicol	p-allylphenol	M
16.2.99.1024	secondary metabolism.phenylpropanoids.unspecified	chlorogenate	secondary metabolism, phenylpropanoids	M
1.1.1002	PS.lightreaction.binding proteins.Chlorophyll a	chlorophyll a	PS.lightreaction, chlorophyll	M
1.1.1003	PS.lightreaction.binding proteins.Chlorophyll b	chlorophyll b	PS.lightreaction, chlorophyll	M
11.8.1010	lipid metabolism.'exotics' (steroids, squalene etc)	cholesterol	secondary metabolism, steroids	M
11.8.1007	lipid metabolism.'exotics' (steroids, squalene etc)	cholesteryl oleate	secondary metabolism, steroids	M
11.1013	lipid metabolism	choline	lipid metabolism, glycerolipids	M
13.1.6.1.1002	amino acid metabolism.synthesis.aromatic aa.chorismate	chorismate	amino acid synthesis.phenylalanine; amino acid synthesis.tryptophan; amino acid synthesis.tyrosine	M
16.2.99.1042	secondary metabolism.phenylpropanoids.unspecified	cinnamaldehyde		M
16.2.99.1025	secondary metabolism.phenylpropanoids.unspecified	cinnamate	secondary metabolism, phenylpropanoids (lignin biosynthesis)	M
35.1.1010	not assigned.no ontology	citramalate	KnownCompoundUnknownFunction	M
8.1.1001	TCA / org transformation.TCA	citrate	gluconeogenese/ glyoxylate cycle; TCA / org transformation	M
13.1.2.3.1003	amino acid metabolism.synthesis.glutamate family.arginine	citrulline	amino acid synthesis.arginine; amino acid degradation.arginine	M
16.99.1017	secondary metabolism.unspecified	clotrimazole	secondary metabolism (?); Inhibitor of P450	M
23.1.1.1007	nucleotide metabolism.synthesis.pyrimidines	cmp		M
11.1027	lipid metabolism	coa-s-s-coa	lipid metabolism, organic acids	M
11.1026	lipid metabolism	coa-sh	lipid metabolism, organic acids	M
16.2.1.1007	secondary metabolism.phenylpropanoids.lignin biosynthesis	coniferyl alcohol	secondary metabolism	M
16.2.1.1008	secondary metabolism.phenylpropanoids.lignin biosynthesis	coniferyl aldehyde	secondary metabolism	M
16.2.99.1006	secondary metabolism.phenylpropanoids.unspecified	coumarine	secondary metabolism, phenylpropanoids	M
23.1.1.1009	nucleotide metabolism.synthesis.pyrimidines	ctp		M
13.1.3.4.1002	amino acid metabolism.synthesis.aspartate family.methionine	cystathionine	amino acid synthesis.methionine	M
13.1.5.3.1004	amino acid metabolism.synthesis.serine-glycine-cysteine group.cysteine	cysteine	amino acid synthesis.cysteine; S-assimilation	M
23.1.1.1006	nucleotide metabolism.synthesis.pyrimidines	cytidine		M
23.1.1.1010	nucleotide metabolism.synthesis.pyrimidines	dctp		M
1.1.1004	PS.lightreaction.PS II.PQ	decyl-plastoquinone	PS.lightreaction	M
20.1002	stress	dehydroascorbate	stress; redox regulation	M
21.1009	redox	dehydroascorbate	stress; redox regulation	M
16.1.4.1006	secondary metabolism.isoprenoids.carotenoids.delta carotene	delta carotene		M
16.1.3.1001	secondary metabolism.isoprenoids.tocopherol biosynthesis	delta-tocopherol	secondary metabolism, tocopherols	M
11.10.1002	lipid metabolism.galactolipid synthesis	digalactosylglycerol		M
16.99.1001	secondary metabolism.unspecified	digitoxin	secondary metabolism	M
35.1.1003	not assigned.no ontology	digoxigenin	Used to label nucleic acids (analyte?)	M
11.8.1013	lipid metabolism.'exotics' (steroids, squalene etc)	digoxin	secondary metabolism; from Digitalis (foxglove plant)	M
16.2.99.1036	secondary metabolism.phenylpropanoids.unspecified	dihydro 4-coumaryl alcohol		M
16.2.99.1037	secondary metabolism.phenylpropanoids.unspecified	dihydro coniferyl alcohol		M
23.1.2.1001	nucleotide metabolism.synthesis.purines	dihydroorotate	Co-factor metabolism; nucleotide metabolism	M
1.3.1005	PS.calvin cycle	dihydroxyacetone-p	major CHO metabolism; PS.calvin cycle; glycolysis; OPP.oxidative PP	M
4.1001	glycolysis	dihydroxyacetone-p	PS.calvin cycle; photorespiration; glycolysis	M
16.1.2.1005	secondary metabolism.isoprenoids.mevalonate pathway	dimethylallyl-pyrophosphate		M
11.1014	lipid metabolism	dioleoylglycerol	lipid metabolism, glycerolipids	M
11.1015	lipid metabolism	diphosphatidylglycerol	lipid metabolism, glycerolipids	M
35.1.1023	not assigned.no ontology	docosanol	KnownCompoundUnknownFunction	M
16.20.1001	secondary metabolism.amino acid derivatives	dopa	secondary metabolism, amino acid derivatives	M
16.20.1002	secondary metabolism.amino acid derivatives	dopamine	secondary metabolism, amino acid derivatives	M
11.1.1001	lipid metabolism.FA synthesis and FA elongation	eicosanoate		M
11.8.1006	lipid metabolism.'exotics' (steroids, squalene etc)	ergosterol	secondary metabolism, steroids	M
3.3.1003	minor CHO metabolism.sugar alcohols	erythritol	minor CHO metabolism.polyols	M
3.99.1024	minor CHO metabolism.misc	erythronic acid		M
3.99.1007	minor CHO metabolism.misc	erythrose	minor CHO metabolism.misc	M
11.1016	lipid metabolism	ethanolamine	lipid metabolism, glycerolipids	M
16.2.99.1034	secondary metabolism.phenylpropanoids.unspecified	eugenol	2-methoxy-4-(2-propenyl)phenol	M
11.1002	lipid metabolism	fa 16:0	lipid metabolism	M
11.1003	lipid metabolism	fa 18:0	lipid metabolism	M
16.1.2.1008	secondary metabolism.isoprenoids.mevalonate pathway	farnesyl pyrophosphate		M
16.2.99.1026	secondary metabolism.phenylpropanoids.unspecified	ferulate	secondary metabolism, phenylpropanoids (lignin biosyntehesis)	M
2.2.1.1001	major CHO metabolism.degradation.sucrose	fructose	major CHO metabolism.synthesis.sucrose; major CHO metabolism.degradation.sucrose	M
1.3.1006	PS.calvin cycle	fructose-1,6-bisp	major CHO metabolism; PS.calvin cycle; glycolysis; OPP.oxidative PP	M
2.1.1.1005	major CHO metabolism.synthesis.sucrose	fructose-2,6-bisp	major CHO metabolism.synthesis.sucrose	M
1.3.1007	PS.calvin cycle	fructose-6-p	cell wall synthesis.cellulose synthesis; major CHO metabolism.synthesis.sucrose; major CHO metabolism.synthesis.starch; calvin cycle; glycolysis; OPP.oxidative PP	M
2.1.1.1001	major CHO metabolism.synthesis.sucrose	fructose-6-p	cell wall synthesis.cellulose synthesis; major CHO metabolism.synthesis.sucrose; major CHO metabolism.synthesis.starch; glycolysis; OPP.oxidative PP	M
2.1.2.1001	major CHO metabolism.synthesis.starch	fructose-6-p	cell wall synthesis.cellulose synthesis; major CHO metabolism.synthesis.sucrose; major CHO metabolism.synthesis.starch; glycolysis; OPP.oxidative PP	M
10.2.1003	cell wall degradation	fucose	cell wall degradation	M
11.8.1011	lipid metabolism.'exotics' (steroids, squalene etc)	fucosterol	secondary metabolism, steroids	M
8.1.1006	TCA / org transformation.TCA	fumarate	amino acid synthesis.arginine; amino acid degradation.tyrosine; amino acid synthesis.asparagines; amino acid synthesis.aspartate; nucleotide metabolism.synthesis; gluconeogenese/ glyoxylate cycle; TCA / org transformation	M
22.1001	polyamine metabolism	gaba	amino acid degradation.glutamate; polyamine metabolism	M
3.1.1002	minor CHO metabolism.raffinose family	galactinol		M
3.3.1005	minor CHO metabolism.sugar alcohols	galactitol	minor CHO metabolism.polyols	M
3.99.1020	minor CHO metabolism.misc	galactonate	minor CHO metabolism.misc; galactose metabolism	M
21.1007	redox	galactonate-gamma-lactone	redox regulation; ascorbate metabolism; precursor	M
10.2.1005	cell wall degradation	galactose	cell wall degradation; minor CHO metabolism	M
3.99.1021	minor CHO metabolism.misc	galactose	minor CHO metabolism.misc; galactose metabolism; cell wall degradation	M
3.99.1022	minor CHO metabolism.misc	galactose-6-p	minor CHO metabolism.misc; galactose metabolism	M
10.2.1007	cell wall degradation	galacturonate	cell wall degradation	M
16.1.3.1005	secondary metabolism.isoprenoids.tocopherol biosynthesis	gamma-tocopherol	secondary metabolism, tocopherols	M
23.1.2.1010	nucleotide metabolism.synthesis.purines	gdp	Co-factor metabolism; nucleotide metabolism	M
16.1.2.1006	secondary metabolism.isoprenoids.mevalonate pathway	geranyl pyrophosphate		M
16.1.1.1014	secondary metabolism.isoprenoids.non-mevalonate pathway	geranylacetone		M
16.1.1.1010	secondary metabolism.isoprenoids.mevalonate pathway	geranylgeranyl pyrophosphate		M
17.6.1.1001	hormone metabolism.gibberelin.synthesis-degradation	gibberellate (ga3)	hormone metabolism.gibberelin	M
21.1002	redox	glucarate	redox regulation; ascorbate metabolism; precursor	M
35.1.1006	not assigned.no ontology	gluconate	KnownCompoundUnknownFunction (analyte?)	M
7.1.1001	OPP.oxidative PP	gluconate-6-p	OPP.oxidative PP	M
10.2.1009	cell wall degradation	glucosaminate	cell wall degradation	M
10.2.1008	cell wall degradation	glucosamine	cell wall degradation	M
2.2.1.1002	major CHO metabolism.degradation.sucrose	glucose	cell wall degradation; major CHO metabolism.synthesis.sucrose; major CHO metabolism.degradation.starch	M
2.1.1.1003	major CHO metabolism.synthesis.sucrose	glucose-1-p	cell wall synthesis.cellulose synthesis; major CHO metabolism.synthesis.sucrose; major CHO metabolism.synthesis.starch	M
2.1.2.1003	major CHO metabolism.synthesis.starch	glucose-1-p	cell wall synthesis.cellulose synthesis; major CHO metabolism.synthesis.sucrose; major CHO metabolism.synthesis.starch	M
2.1.1.1004	major CHO metabolism.synthesis.sucrose	glucose-1,6-bisp	cell wall synthesis.cellulose synthesis; major CHO metabolism.synthesis.sucrose; major CHO metabolism.synthesis.starch	M
35.1.1004	not assigned.no ontology	glucose-3-sulfate	KnownCompoundUnknownFunction	M
2.1.1.1002	major CHO metabolism.synthesis.sucrose	glucose-6-p	cell wall synthesis.cellulose synthesis; major CHO metabolism.synthesis.sucrose; major CHO metabolism.synthesis.starch; glycolysis; OPP.oxidative PP	M
2.1.2.1002	major CHO metabolism.synthesis.starch	glucose-6-p	cell wall synthesis.cellulose synthesis; major CHO metabolism.synthesis.sucrose; major CHO metabolism.synthesis.starch; glycolysis; OPP.oxidative PP	M
21.1004	redox	glucuronate	redox regulation; ascorbate metabolism; precursor	M
12.2.1002	N-metabolism.ammonia metabolism	glutamate	N-metabolism.ammonia metabolism; amino acid synthesis.alanine; amino acid degradation.alanine; amino acid synthesis.isoleucine; amino acid degradation.isoleucine; amino acid synthesis.leucineamino acid degradation.leucine; amino acid synthesis.lysine; amino acid degradation.lysine; amino acid synthesis.phenylalanine; amino acid synthesis.proline; amino acid degradation.proline; amino acid synthesis.arginine; amino acid degradation.arginine; amino acid synthesis.tryptophan; amino acid synthesis.tyrosine; amino acid synthesis.valine; amino acid degradation.valine; amino acid degradation.hydroxyproline; amino acid synthesis.asparagine; amino acid degradation.asparagines; amino acid degradation.cysteine; amino acid synthesis.glutamate; amino acid degradation.glutamate; amino acid synthesis.glutamine; amino acid degradation.glutamine; amino acid synthesis.histidine; Co-factor metabolism; tetrapyrrole synthesis; redox regulation; nucleotide metabolism.synthesis; C1-metabolism	M
12.2.1003	N-metabolism.ammonia metabolism	glutamine	N-metabolism.ammonia metabolism; amino acid synthesis.arginine; amino acid synthesis.tryptophan; amino acid synthesis.asparagines; amino acid degradation.asparagines; amino acid synthesis.glutamine; amino acid degradation.glutamine; amino acid synthesis.histidine; nucleotide metabolism.synthesis; nucleotide metabolism.degradation; C1-metabolism	M
13.2.3.5.1001	amino acid metabolism.degradation.aspartate family.lysine	glutarate	amino acid degradation.lysine, lipids	M
1.3.1004	PS.calvin cycle	glyceraldehyde-3-p	major CHO metabolism; PS.calvin cycle; glycolysis; OPP.oxidative PP	M
4.1002	glycolysis	glyceraldehyde-3-p	PS.calvin cycle; photorespiration; glycolysis	M
1.2.1001	PS.photorespiration.glycerate	glycerate	PS.photorepiration; lipid metabolism; amino acid degradation.serine; amino acid degradation.glycine; gluconeogenese/ glyoxylate cycle; TCA / org transformation	M
13.2.5.1.1001	amino acid metabolism.degradation.serine-glycine-cysteine group.serine	glycerate	PS.photorepiration; lipid metabolism; amino acid degradation.serine; amino acid degradation.glycine; gluconeogenese/ glyoxylate cycle; TCA / org transformation	M
1.3.1003	PS.calvin cycle	glycerate-1,3-bisp	major CHO metabolism; PS.calvin cycle; glycolysis; OPP.oxidative PP	M
4.1003	glycolysis	glycerate-1,3-bisp	PS.calvin cycle; photorespiration; glycolysis	M
4.1007	glycolysis	glycerate-2-p	glyclolysis	M
1.3.1002	PS.calvin cycle	glycerate-3-p	major CHO metabolism; PS.calvin cycle; glycolysis; OPP.oxidative PP	M
4.1004	glycolysis	glycerate-3-p	PS.calvin cycle; photorespiration; glycolysis	M
11.1017	lipid metabolism	glycerol	lipid metabolism, glycerolipids	M
11.1018	lipid metabolism	glycerol-2-p	lipid metabolism, glycerolipids	M
11.1028	lipid metabolism	glycerol-3-p	lipid metabolism; hormone metabolism.auxin (is polar fraction)	M
1.2.1003	PS.photorepiration.glycine	glycine	PS.photorepiration; amino acid synthesis.serine; amino acid synthesis.glycine; amino acid degradation.glycine; redox regulation; nucleotide metabolism.synthesis; C1-metabolism	M
13.1.5.2.1001	amino acid metabolism.synthesis.serine-glycine-cysteine group.glycine	glycine	PS.photorepiration; amino acid synthesis.serine; amino acid synthesis.glycine; amino acid degradation.glycine; redox regulation; nucleotide metabolism.synthesis; C1-metabolism	M
1.2.1002	PS.photorepiration.glycolate	glycolate	PS.photorepiration	M
1.2.1005	PS.photorepiration.glyoxylate	glyoxylate	PS.photorepiration	M
23.1.2.1009	nucleotide metabolism.synthesis.purines	gmp	Co-factor metabolism; nucleotide metabolism	M
23.1.2.1011	nucleotide metabolism.synthesis.purines	gtp	Co-factor metabolism; nucleotide metabolism	M
23.1.2.1007	nucleotide metabolism.synthesis.purines	guanine	Co-factor metabolism; nucleotide metabolism	M
23.1.2.1008	nucleotide metabolism.synthesis.purines	guanosine	Co-factor metabolism; nucleotide metabolism	M
21.1011	redox	gulonate		M
21.1003	redox	gulonate-gamma-lactone	redox regulation; ascorbate metabolism; precursor	M
3.99.1025	minor CHO metabolism.misc	gulose		M
16.99.1014	secondary metabolism.unspecified	helicin	secondary metabolism, glucoside	M
11.1012	lipid metabolism	heptanoic acid	lipid metabolism, also called oenanthic acid	M
35.1.1009	not assigned.no ontology	hexachlorobenzene	pesticide, insecticide, also called lindane	M
35.1.1021	not assigned.no ontology	hexacosanol	KnownCompoundUnknownFunction, (C26-OH)	M
11.1032	lipid metabolism	hexanal		M
11.1033	lipid metabolism	hexanoic acid		M
11.1035	lipid metabolism	hexanol		M
13.1.7.1002	amino acid metabolism.synthesis.histidine	histidine	amino acid synthesis.histidine	M
13.1.7.1001	amino acid metabolism.synthesis.histidine	histidinol-p	amino acid synthesis.histidine	M
13.1.3.6.2.1001	amino acid metabolism.synthesis.aspartate family.misc.homocysteine	homocysteine	amino acid synthesis.homocysteine	M
35.1.1014	not assigned.no ontology	homoglutamine	KnownCompoundUnknownFunction	M
13.1.3.6.1.1001	amino acid metabolism.synthesis.aspartate family.misc.homoserine	homoserine	amino acid synthesis.homoserine; amino acid degradation.homoserine; amino acid synthesis.methionine; amino acid degradation.methionine; amino acid metabolism.threonine; amino acid synthesis.threonine	M
35.1.1012	not assigned.no ontology	hydroxylamine	KnownCompoundUnknownFunction	M
10.2.1013	cell wall degradation	hydroxyproline	cell wall degradation	M
1.2.1006	PS.photorepiration.hydroxypyruvate	hydroxypyruvate	PS.photorepiration	M
35.1.1025	not assigned.no ontology.Hydroxyurea	hydroxyurea	KnownCompoundUnknownFunction	M
23.2.2.1005	nucleotide metabolism.degradation.purine	hypoxanthine	Co-factor metabolism; nucleotide metabolism.degradation; purine metabolism	M
35.1.1015	not assigned.no ontology	imidazole	KnownCompoundUnknownFunction (analyte?)	M
17.2.1.1002	hormone metabolism.auxin	indole-3-acetaldehyde	hormone metabolism.auxin	M
17.2.1.1003	hormone metabolism.auxin	indole-3-acetate	hormone metabolism.auxin	M
17.2.1.1004	hormone metabolism.auxin	indole-3-acetyl-aspartate	hormone metabolism.auxin	M
17.2.1.1005	hormone metabolism.auxin	indole-3-butyrate	hormone metabolism.auxin	M
17.2.1.1006	hormone metabolism.auxin	indoxyl-beta-d-glucoside	hormone metabolism.auxin	M
23.1.2.1012	nucleotide metabolism.synthesis.purines	inosine	Co-factor metabolism; nucleotide metabolism	M
3.4.1001	minor CHO metabolism.myo-inositol	inositol	minor CHO metabolism.inositol	M
3.4.1002	minor CHO metabolism.myo-inositol	inositol-1-p	minor CHO metabolism.inositol	M
3.4.1004	minor CHO metabolism.myo-inositol	inositol-2-p	minor CHO metabolism.inositol	M
35.1.1007	not assigned.no ontology	isoascorbate	KnownCompoundUnknownFunction (analyte?)	M
8.1.1003	TCA / org transformation.TCA	isocitrate	gluconeogenese/ glyoxylate cycle; TCA / org transformation	M
13.1.4.5.1001	amino acid metabolism.synthesis.branched chain group.isoleucine specific	isoleucine	amino acid synthesis.isoleucine; amino acid degradation.isoleucine	M
2.2.2.1003	major CHO metabolism.degradation.starch	isomaltose	cell wall synthesis; major CHO metabolism.degradation.starch	M
16.1.2.1004	secondary metabolism.isoprenoids.mevalonate pathway	isopentenyl-pyrophoshate		M
8.2.1005	TCA / org transformation. Various	itaconate		M
17.7.1.1002	hormone metabolism.jasmonate.synthesis-degradation	jasmonic acid	hormone metabolism.jasmonate	M
16.2.99.1027	secondary metabolism.phenylpropanoids.unspecified	kaempferol-3-o-rutinoside	secondary metabolism, phenylpropanoids	M
35.1.1024	not assigned.no ontology.Kojic acid	kojic acid	KnownCompoundUnknownFunction	M
5.1001	fermentation	lactate	fermentation	M
3.99.1008	minor CHO metabolism.misc	lactose	minor CHO metabolism.misc	M
3.99.1027	minor CHO metabolism.misc.Laminaribiose	laminaribiose	laminarabiose	M
11.8.1005	lipid metabolism.'exotics' (steroids, squalene etc)	lanosterol	secondary metabolism, steroids	M
13.1.4.4.1001	amino acid metabolism.synthesis.branched chain group.leucine specific	leucine	amino acid synthesis.leucine; amino acid degradation.leucine	M
10.2.1012	cell wall degradation	levoglucosan	cell wall degradation, degradation of cellulose	M
16.1.2.1013	secondary metabolism.isoprenoids.mevalonate pathway	limonene		M
16.1.2.1011	secondary metabolism.isoprenoids.mevalonate pathway	linaloyl pyrophosphate		M
11.1024	lipid metabolism	linoleic acid	lipid metabolism, is FA 9,12(Z,Z)-18:2	M
11.1029	lipid metabolism	linolenic acid	lipid metabolism, is FA 9,12,15(Z,Z,Z)-18:3	M
18.1009	Co-factor and vitamine metabolism.lumichrome	lumichrome	7,8-Dimethylalloxazine; Riboflavin metabolism	M
16.1.4.1003	secondary metabolism.isoprenoids.carotenoids.lycopene	lycopene		M
16.8.1.1001	secondary metabolism.flavonoids.anthocyanins	lycopine	secondary metabolism, anthocyanin	M
13.1.3.5.1001	amino acid metabolism.synthesis.aspartate family.lysine	lysine	amino acid synthesis.lysine; amino acid degradation.lysine; polyamine metabolism	M
3.99.1009	minor CHO metabolism.misc	lyxose	minor CHO metabolism.misc	M
8.1.1007	TCA / org transformation.TCA	malate	gluconeogenese/ glyoxylate cycle; TCA / org transformation	M
35.1.1011	not assigned.no ontology	maleate	KnownCompoundUnknownFunction	M
3.3.1004	minor CHO metabolism.sugar alcohols	maltitol	minor CHO metabolism.polyols	M
2.2.2.1002	major CHO metabolism.degradation.starch	maltose	cell wall synthesis; major CHO metabolism.degradation.starch	M
2.2.2.1004	major CHO metabolism.degradation.starch	maltotriose	cell wall synthesis; major CHO metabolism.degradation.starch	M
16.99.1013	secondary metabolism.unspecified	mandelate	secondary metabolism	M
16.2.99.1044	secondary metabolism.phenylpropanoids.unspecified	mandelic acid		M
3.3.1002	minor CHO metabolism.sugar alcohols	mannitol	minor CHO metabolism.polyols	M
10.2.1004	cell wall degradation	mannose	cell wall degradation; minor CHO metabolism	M
21.1006	redox	mannose-1-p	redox regulation; ascorbate metabolism; precursor	M
21.1005	redox	mannose-6-p	redox regulation; ascorbate metabolism; precursor	M
20.1.1001	stress.biotic	melezitose	commonly found in honeys and in honey dew produced by apids but lacking from plant nectar	M
3.99.1010	minor CHO metabolism.misc	melezitose	minor CHO metabolism.misc	M
10.2.1011	cell wall degradation	melibiose	cell wall degradation; minor CHO metabolism	M
13.1.3.4.1003	amino acid metabolism.synthesis.aspartate family.methionine	methionine	amino acid synthesis.methionine; amino acid degradation.methionine; hormone metabolism.ethylene; C1-metabolism	M
13.1001	amino acid metabolism.methionine S-oxide	methionine s-oxide	oxydized product is recycled via reeductase; Methionine sulfoxide	M
3.99.1011	minor CHO metabolism.misc	methyl-alpha-mannopyranoside	minor CHO metabolism.misc	M
16.2.99.1033	secondary metabolism.phenylpropanoids.unspecified	methyl-chavicol	Estragole, or p-allylanisole	M
3.99.1012	minor CHO metabolism.misc	methyl-d-glucoside	minor CHO metabolism.misc	M
16.2.99.1035	secondary metabolism.phenylpropanoids.unspecified	methyl-eugenol		M
13.2.3.2.1001	amino acid metabolism.degradation.aspartate family.threonine	methylglyoxal	amino acid metabolism.threonine; amino acid degradation.threonine	M
17.8.1002	hormone metabolism.salicylic acid.synthesis-degradation.Methylsalicylate	methylsalicylate		M
16.1.2.1001	secondary metabolism.isoprenoids.mevalonate pathway	mevalonate		M
16.1.2.1002	secondary metabolism.isoprenoids.mevalonate pathway	mevalonate-5-phosphate		M
16.1.2.1003	secondary metabolism.isoprenoids.mevalonate pathway	mevalonate-5-pyrophosphate		M
17.1.1.1001	hormone metabolism.abscisic acid.synthesis-degradation	mevalonolactone	hormone metabolism.abscisic acid precursor	M
27.1001	RNA	monomethylphosphate	RNA (actually small nuclear RNA)	M
17.2.1.1007	hormone metabolism.auxin	n-(3-indolylacetyl)-alanine		M
30.1007	signaling	n-(beta-oxoocaproyl)-homoserine lactone	signaling, HOMOSERINE LACTONE-MEDIATED GENE REGULATION IN PLANT-ASSOCIATED BACTERIA	M
35.1.1013	not assigned.no ontology	n-acetyl-serine	KnownCompoundUnknownFunction	M
13.1.2.3.1004	amino acid metabolism.synthesis.glutamate family.arginine	n-acetylglutamic acid		M
30.1002	signaling	n-butyryl-homoserine lactone	signaling, HOMOSERINE LACTONE-MEDIATED GENE REGULATION IN PLANT-ASSOCIATED BACTERIA	M
16.2.99.1005	secondary metabolism.phenylpropanoids.unspecified	n-cinnamoylglycine	secondary metabolism, phenylpropanoids	M
30.1003	signaling	n-decanoyl-homoserine lactone	signaling, HOMOSERINE LACTONE-MEDIATED GENE REGULATION IN PLANT-ASSOCIATED BACTERIA	M
30.1004	signaling	n-dodecanoyl-homoserine lactone	signaling, HOMOSERINE LACTONE-MEDIATED GENE REGULATION IN PLANT-ASSOCIATED BACTERIA	M
30.1005	signaling	n-heptanoyl-homoserine lactone	signaling, HOMOSERINE LACTONE-MEDIATED GENE REGULATION IN PLANT-ASSOCIATED BACTERIA	M
30.1006	signaling	n-hexanoyl-homoserine lactone	signaling, HOMOSERINE LACTONE-MEDIATED GENE REGULATION IN PLANT-ASSOCIATED BACTERIA	M
16.4.1.1007	secondary metabolism.N misc.alkaloid-like	n-methyl-calystegine b2	secondary metabolism, alkaloids	M
30.1008	signaling	n-octanoyl-homoserine lactone	signaling, HOMOSERINE LACTONE-MEDIATED GENE REGULATION IN PLANT-ASSOCIATED BACTERIA	M
30.1009	signaling	n-tetradecanoyl-homoserine lactone	signaling, HOMOSERINE LACTONE-MEDIATED GENE REGULATION IN PLANT-ASSOCIATED BACTERIA	M
18.1003	Co-factor and vitamine metabolism	nad	Co-factor metabolism; energy misc	M
18.1004	Co-factor and vitamine metabolism	nadh	Co-factor metabolism; energy misc	M
18.1005	Co-factor and vitamine metabolism	nadp	Co-factor metabolism; energy misc	M
18.1006	Co-factor and vitamine metabolism	nadph	Co-factor metabolism; energy misc	M
16.2.99.1028	secondary metabolism.phenylpropanoids.unspecified	naringenin	secondary metabolism, phenylpropanoids	M
16.1.2.1007	secondary metabolism.isoprenoids.mevalonate pathway	neryl pyrophosphate		M
18.1002	Co-factor and vitamine metabolism	nicotinamide	Co-factor metabolism; energy misc	M
18.1001	Co-factor and vitamine metabolism	nicotinate	Co-factor metabolism; energy misc	M
16.4.1.1001	secondary metabolism.N misc.alkaloid-like	nicotine	secondary metabolism, alkaloids	M
11.1.1002	lipid metabolism.FA synthesis and FA elongation	nonanoate		M
16.20.1003	secondary metabolism.amino acid derivatives	noradrenaline	secondary metabolism, amino acid derivatives	M
35.1.1016	not assigned.no ontology	norleucine	KnownCompoundUnknownFunction	M
35.1.1017	not assigned.no ontology	norvaline	KnownCompoundUnknownFunction	M
13.1.5.3.1001	amino acid metabolism.synthesis.serine-glycine-cysteine group.cysteine	o-acetyl-serine	amino acid synthesis.cysteine; S-assimilation	M
13.1.3.4.1001	amino acid metabolism.synthesis.aspartate family.methionine	o-succinylhomoserine	amino acid synthesis.methionine	M
35.1.1020	not assigned.no ontology	octadecanol	KnownCompoundUnknownFunction, C18OH	M
11.8.1008	lipid metabolism.'exotics' (steroids, squalene etc)	oleanolate	secondary metabolism, steroids	M
11.1004	lipid metabolism	oleic acid	lipid metabolism	M
11.1005	lipid metabolism	oleyl alcohol	lipid metabolism	M
11.1006	lipid metabolism	oleyl oleate	lipid metabolism	M
3.99.1013	minor CHO metabolism.misc	oligofructan (total)	minor CHO metabolism.misc	M
3.3.1007	minor CHO metabolism.sugar alcohols	ononitol	minor CHO metabolism.polyols	M
13.1.2.3.1001	amino acid metabolism.synthesis.glutamate family.arginine	ornithine	amino acid synthesis.arginine; amino acid degradation.arginine; amino acid degradation.glutamate; polyamine metabolism	M
23.1.2.1002	nucleotide metabolism.synthesis.purines	orotate	Co-factor metabolism; nucleotide metabolism	M
8.2.1003	TCA / org transformation. Various	oxalate		M
8.1.1008	TCA / org transformation.TCA	oxaloacetate	TCA cycle	M
17.7.1.1001	hormone metabolism.jasmonate.synthesis-degradation	oxophytodienoic acid	hormone metabolism.jasmonate. Precursor of jasmonic acid	M
4.1005	glycolysis	p-enolpyruvate	glyclolysis	M
11.1007	lipid metabolism	palmitic acid	lipid metabolism	M
18.1008	Co-factor and vitamine metabolism	pantothenate		M
35.1.1019	not assigned.no ontology	parabanate	KnownCompoundUnknownFunction	M
16.7.1001	secondary metabolism.wax	pentadecane	secondary metabolism, waxes	M
11.1008	lipid metabolism	pentadecanoic acid	lipid metabolism	M
11.1034	lipid metabolism	pentanoic acid		M
16.2.99.1043	secondary metabolism.phenylpropanoids.unspecified	phenylacetate		M
13.1.6.3.1002	amino acid metabolism.synthesis.aromatic aa.phenylalanine	phenylalanine	amino acid synthesis.phenylalanine; amino acid degradation.phenylalanine; secondary metabolism	M
16.2.99.1048	secondary metabolism.phenylpropanoids.unspecified	phenylethanol		M
13.1.6.3.1003	amino acid metabolism.synthesis.aromatic aa.phenylalanine	phenylpyruvate		M
991.1.1001	Mineral Nutrition.phosphate	phosphate	Mineral Nutrition	M
11.1019	lipid metabolism	phosphatidylcholine	lipid metabolism, glycerolipids	M
11.1020	lipid metabolism	phosphatidylethanolamine glycine	lipid metabolism, glycerolipids	M
11.1021	lipid metabolism	phosphatidylserine	lipid metabolism, glycerolipids	M
16.1.4.1001	secondary metabolism.isoprenoids.carotenoids.phytoene	phytoene	Carotene precursor	M
1.1.1001	PS.lightreaction	phytol	PS.lightreaction, chlorophyll	M
3.3.1006	minor CHO metabolism.sugar alcohols	pinitol	minor CHO metabolism.polyols	M
18.1007	Co-factor and vitamine metabolism	ppi	Co-factor metabolism; energy misc	M
13.1.6.3.1001	amino acid metabolism.synthesis.aromatic aa.phenylalanine	prephenate	amino acid synthesis.phenylalanine; amino acid synthesis.tryptophan; amino acid synthesis.tyrosine	M
13.1.2.2.1001	amino acid metabolism.synthesis.glutamate family.proline	proline	amino acid synthesis.proline; amino acid degradation.proline; stress	M
20.1003	stress	proline	amino acid synthesis.proline; amino acid degradation.proline; stress	M
16.99.1018	secondary metabolism.unspecified	propyl gallate	secondary metabolism, antioxydant	M
16.1.1.1013	secondary metabolism.isoprenoids.non-mevalonate pathway	pseudoionone		M
3.99.1014	minor CHO metabolism.misc	psicose	minor CHO metabolism.misc	M
22.1003	polyamine metabolism	putrescine	amino acid degradation.arginine; polyamine metabolism	M
21.1010	redox	pyroglutamate	redox regulation, glutathion metabolism, degradation product. Also analyte	M
4.1006	glycolysis	pyruvate	glyclolysis	M
3.3.1012	minor CHO metabolism.sugar alcohols	quebranchitol		M
16.99.1014	secondary metabolism.unspecified	quinate	secondary metabolism	M
3.1.1001	minor CHO metabolism.raffinose family	raffinose	minor CHO metabolism.raffinose	M
10.2.1010	cell wall degradation	rhamnose	cell wall degradation; minor CHO metabolism	M
3.3.1008	minor CHO metabolism.sugar alcohols	ribitol	minor CHO metabolism.polyols	M
10.2.1002	cell wall degradation	ribonate	cell wall degradation; minor CHO metabolism; auxin metabolism	M
10.2.1001	cell wall degradation	ribose	cell wall degradation; minor CHO metabolism; auxin metabolism	M
1.3.1001	PS.calvin cycle.ribulose-1,5-bisP	ribulose-1,5-bisp	major CHO metabolism; PS.calvin cycle	M
16.2.99.1029	secondary metabolism.phenylpropanoids.unspecified	rutin	secondary metabolism, phenylpropanoids	M
13.1.5.3.1003	amino acid metabolism.synthesis.serine-glycine-cysteine group.cysteine	s-methylcysteine	amino acid synthesis.cysteine; S-assimilation, storage form of sulfur in the seeds of several legume species	M
35.1.1008	not assigned.no ontology	saccharate	KnownCompoundUnknownFunction	M
16.99.1009	secondary metabolism.unspecified	salicin	secondary metabolism	M
16.2.99.1030	secondary metabolism.phenylpropanoids.unspecified	salicylate	secondary metabolism, phenylpropanoids	M
17.8.1001	hormone metabolism.salicylic acid.synthesis-degradation	salicylic acid-glucose		M
3.3.1010	minor CHO metabolism.sugar alcohols	sequoyitol		M
1.2.1004	PS.photorepiration.serine	serine	PS.photorepiration; lipid metabolism; amino acid degradation.homoserine; amino acid synthesis.serine; amino acid degradation.serine; amino acid degradation.methionine; S-assimilation	M
13.1.5.1.1001	amino acid metabolism.synthesis.serine-glycine-cysteine group.serine	serine	PS.photorepiration; lipid metabolism; amino acid degradation.homoserine; amino acid synthesis.serine; amino acid degradation.serine; amino acid degradation.methionine; S-assimilation	M
16.1.2.1009	secondary metabolism.isoprenoids.mevalonate pathway	sesquiterpene		M
13.1.6.1.1001	amino acid metabolism.synthesis.serine-glycine-cysteine group.cysteine	shikimate	amino acid synthesis.phenylalanine; amino acid synthesis.tryptophan; amino acid synthesis.tyrosine	M
16.2.1.1011	secondary metabolism.phenylpropanoids.lignin biosynthesis	sinapinaldehyde	secondary metabolism, phenylpropanoids (lignin biosynthesis)	M
16.2.99.1031	secondary metabolism.phenylpropanoids.unspecified	sinapinate	secondary metabolism, phenylpropanoids (lignin biosynthesis)	M
16.2.1.1010	secondary metabolism.phenylpropanoids.lignin biosynthesis	sinapyl alcohol	secondary metabolism, phenylpropanoids (lignin biosynthesis)	M
3.99.1015	minor CHO metabolism.misc	sophorose	minor CHO metabolism.misc	M
3.3.1001	minor CHO metabolism.sugar alcohols	sorbitol	minor CHO metabolism.polyols	M
3.99.1016	minor CHO metabolism.misc	sorbitol-6-p	minor CHO metabolism.misc	M
3.99.1026	minor CHO metabolism.misc	sorbose		M
22.1004	polyamine metabolism	spermidine	polyamine metabolism	M
22.1005	polyamine metabolism	spermine	polyamine metabolism	M
16.1.5.1001	secondary metabolism.isoprenoids.terpenoids	squalen	secondary metabolism, steroids, terpenoids	M
16.1.2.1010	secondary metabolism.isoprenoids.mevalonate pathway	squalene		M
3.99.1023	minor CHO metabolism.misc	stachyose	minor CHO metabolism.misc; galactose metabolism	M
2.2.2.1001	major CHO metabolism.degradation.starch	starch (total)	cell wall synthesis; major CHO metabolism.degradation.starch	M
11.1009	lipid metabolism	stearic acid	lipid metabolism	M
11.8.1004	lipid metabolism.'exotics' (steroids, squalene etc)	stigmastadienone	secondary metabolism, steroids	M
11.8.1002	lipid metabolism.'exotics' (steroids, squalene etc)	stigmastanol	secondary metabolism, steroids	M
11.8.1001	lipid metabolism.'exotics' (steroids, squalene etc)	stigmasterol	secondary metabolism, steroids	M
11.8.1003	lipid metabolism.'exotics' (steroids, squalene etc)	stigmasterol acetate	secondary metabolism, steroids	M
8.2.1004	TCA / org transformation. Various	suberate	Also called cork acid, octanedioic acid	M
35.1.1018	not assigned.no ontology	suberyl glycine	KnownCompoundUnknownFunction	M
8.1.1005	TCA / org transformation.TCA	succinate	amino acid synthesis.lysine; secondary metabolism; gluconeogenese/ glyoxylate cycle; TCA / org transformation	M
2.1.1.1008	major CHO metabolism.synthesis.sucrose	sucrose	major CHO metabolism.synthesis.sucrose; major CHO metabolism.degradation.sucrose	M
2.1.1.1007	major CHO metabolism.synthesis.sucrose	sucrose-6-p	major CHO metabolism.synthesis.sucrose	M
14.1001	Mineral Nutrition	sulfate	Mineral Nutrition	M
3.99.1017	minor CHO metabolism.misc	tagatose	minor CHO metabolism.misc	M
11.1.1003	lipid metabolism.FA synthesis and FA elongation	tetradecanoic acid		M
3.3.1009	minor CHO metabolism.sugar alcohols	threitol		M
21.1001	redox	threonate	redox regulation; ascorbate metabolism; precursor	M
35.1.1022	not assigned.no ontology	threonic acid-1,4-lactone	KnownCompoundUnknownFunction	M
13.1.3.2.1001	amino acid metabolism.synthesis.aspartate family.threonine	threonine	amino acid synthesis.isoleucine; amino acid metabolism.threonine; amino acid synthesis.threonine; amino acid degradation.threonine	M
23.1.1.1011	nucleotide metabolism.synthesis.pyrimidines	thymidine		M
16.1.3.1002	secondary metabolism.isoprenoids.tocopherol biosynthesis	tocopherol acetate	secondary metabolism, tocopherols	M
16.2.99.1003	secondary metabolism.phenylpropanoids.unspecified	trans-3,5-dimethoxy-4-hydroxycinnamaldehyde	secondary metabolism, phenylpropanoids	M
16.2.1.1001	secondary metabolism.phenylpropanoids.lignin biosynthesis	trans-cinnamate	secondary metabolism, phenylpropanoids	M
3.2.1001	minor CHO metabolism.trehalose	trehalose	minor CHO metabolism.trehalose	M
11.1010	lipid metabolism	tridecanoic acid	lipid metabolism	M
11.1022	lipid metabolism	trilinolein	lipid metabolism, glycerolipids	M
11.1023	lipid metabolism	triolein	lipid metabolism, glycerolipids	M
3.4.1003	minor CHO metabolism.myo-inositol	triphosphoinositide	minor CHO metabolism.inositol	M
16.20.1004	secondary metabolism.amino acid derivatives	tryptamine	secondary metabolism, shikimic acid pathway	M
13.1.6.5.1001	amino acid metabolism.synthesis.aromatic aa.tryptophan	tryptophan	amino acid synthesis.tryptophan; amino acid degradation.tryptophan; hormone metabolism.auxin	M
16.20.1005	secondary metabolism.amino acid derivatives	tyramine	secondary metabolism, shikimic acid pathway	M
13.1.6.4.1001	amino acid metabolism.synthesis.aromatic aa.tyrosine	tyrosine	amino acid synthesis.tyrosine; amino acid degradation.tyrosine; secondary metabolism; Co-factor metabolism	M
9.1001	mitochondrial electron transport / ATP synthesis	ubiquinone-20	Respiration; Redox electron carrier	M
23.1.2.1005	nucleotide metabolism.synthesis.purines	udp	Co-factor metabolism; nucleotide metabolism	M
2.1.1.1006	major CHO metabolism.synthesis.sucrose	udp-glucose	cell wall synthesis.cellulose synthesis; major CHO metabolism.synthesis.sucrose	M
23.1.2.1004	nucleotide metabolism.synthesis.purines	ump	Co-factor metabolism; nucleotide metabolism	M
11.1011	lipid metabolism	undecanoic acid	lipid metabolism	M
35.2.1001	not assigned.unknown	unknown001	UnknownCompound	M
23.2.2.1001	nucleotide metabolism.degradation.purine	uracil	Co-factor metabolism; nucleotide metabolism.degradation	M
23.2.2.1006	nucleotide metabolism.degradation.purine	urate	Co-factor metabolism; nucleotide metabolism.degradation; purine metabolism	M
13.2.2.3.1001	amino acid metabolism.degradation.glutamate family.arginine	urea	amino acid synthesis.arginine; amino acid degradation.arginine	M
23.1.2.1003	nucleotide metabolism.synthesis.purines	uridine	Co-factor metabolism; nucleotide metabolism	M
11.8.1009	lipid metabolism.'exotics' (steroids, squalene etc)	ursolate	secondary metabolism, steroids	M
23.1.2.1006	nucleotide metabolism.synthesis.purines	utp	Co-factor metabolism; nucleotide metabolism	M
13.1.4.3.1001	amino acid metabolism.synthesis.branched chain group.valine specific	valine	amino acid synthesis.valine; amino acid degradation.valine	M
3.3.1011	minor CHO metabolism.sugar alcohols	viburnitol		M
9.1001	mitochondrial electron transport / ATP synthesis	vitamin k1	Respiration; Redox electron carrier	M
23.2.2.1004	nucleotide metabolism.degradation.purine	xanthine	Co-factor metabolism; nucleotide metabolism.degradation; purine metabolism	M
23.2.2.1003	nucleotide metabolism.degradation.purine	xanthosine	Co-factor metabolism; nucleotide metabolism.degradation; purine metabolism	M
3.99.1018	minor CHO metabolism.misc	xylitol	minor CHO metabolism.misc	M
3.99.1019	minor CHO metabolism.misc	xylose	minor CHO metabolism.misc	M
17.4.1.1003	hormone metabolism.cytokinin.synthesis-degradation	zeatin	hormone metabolism.cytokinin	M
16.1.4.1002	secondary metabolism.isoprenoids.carotenoids.zeta carotene	zeta carotene		M
```
